# Fifteen-MiRNA-Based Signature Is a Reliable Prognosis-Predicting Tool for Prostate Cancer Patients

**DOI:** 10.7150/ijms.49412

**Published:** 2021-01-01

**Authors:** Zichen Bian, Xinbo Huang, Yiding Chen, Jialin Meng, Xingliang Feng, Meng Zhang, Li Zhang, Jun Zhou, Chaozhao Liang

**Affiliations:** 1 Department of Urology, The First Affiliated Hospital of Anhui Medical University and Institute of Urology and Anhui Province Key Laboratory of Genitourinary Diseases, Anhui Medical University, Jixi Road 218th, Shushan District, Hefei, Anhui, 230022, People's Republic of China.; 2 Guangdong and Shenzhen Key Laboratory of Male Reproductive Medicine and Genetics, Institute of Urology, Peking University Shenzhen Hospital, Shenzhen-Peking University-the Hong Kong University of Science and Technology Medical Center, Shenzhen 518000, China.; 3 Institute of Urology of Shenzhen University, The Third Affiliated Hospital of Shenzhen University, Shenzhen Luohu Hospital Group, Shenzhen 518000, People's Republic of China.

**Keywords:** microRNA, prostate cancer, recurrence-free survival

## Abstract

Recurrence is a major problem for prostate cancer patients, thus, identifying prognosis-related markers to evaluate clinical outcomes is essential. Here, we established a fifteen-miRNA-based recurrence-free survival (RFS) predicting signature based on the miRNA expression profile extracted from The Cancer Genome Atlas (TCGA) database by the LASSO Cox regression analysis. The median risk score generated by the signature in both the TCGA training and the external Memorial Sloan-Kettering Cancer Center (MSKCC) validation cohorts was employed and the patients were subclassified into low- and high-risk subgroups. The Kaplan-Meier plot and log-rank analyses showed significant survival differences between low- and high-risk subgroups of patients (TCGA, log-rank *P* < 0.001 & MSKCC, log-rank *P* = 0.045). In addition, the receiver operating characteristic curves of both the training and external validation cohorts indicated the good performance of our model. After predicting the downstream genes of these miRNAs, the miRNA-mRNA network was visualized by Cytoscape software. In addition, pathway analyses found that the differences between two groups were mainly enriched on tumor progression and drug resistance-related pathways. Multivariate analyses revealed that the miRNA signature is an independent indicator of RFS prognosis for prostate cancer patients with or without clinicopathological features. In summary, our novel fifteen-miRNA-based prediction signature is a reliable method to evaluate the prognosis of prostate cancer patients.

## Introduction

Prostate cancer (PCa) causes a heavy health burden for men around the world, accounting for more than 350,000 cancer-specific deaths, and it was the fifth leading cause of cancer in 2018 1. Approximately 5% of PCa patients have advanced type PCa or metastases at the time of diagnosis 2. For advanced PCa, initial hormonal therapy can inhibit the progression of PCa in most patients by decreasing the function of the testosterone and androgen receptor (AR) signaling pathway; however, these patients tend to develop castration-resistant prostate cancer (CRPC), and approximately 10-20% of these patients arrive at this stage in the first five years after diagnosis 3, 4. For patients over 75-years-old, the incidence rate of metastasis increases to 48% at the time of diagnosis, and PCa-specific death is also as high as 53% 5.

A class of non-coding RNAs that are 17-25 bp in length that could impact protein-encoded genes through binding to the 3' untranslated region of mRNA are named as microRNAs (miRNAs). Approximately half of the mRNAs' expression could be modified with miRNAs through this mechanism 6-11. Numerous articles have reported that miRNAs could participate in the process of normal cell physiology, including cell differentiation, apoptosis, proliferation, and cell cycle arrest 12-14. In addition, miRNAs are also involved in the extensive process of tumorigenesis in various cancers, such as pancreatic cancer, brain glioma, cervical cancer and prostate cancer 15-20. With the application of gene sequencing in tumors, miRNAs have been considered novel biomarkers in the prediction of prognosis and drug resistance 21-25, and integrating multiple miRNAs could be more effective and lead to a better prediction than single miRNAs 16, 23, 26-29.

In the current study, our purpose was to establish a multiple-miRNA-based signature to predict the recurrence risk of PCa patients, to help the clinician develop treatment plan.

## Materials and Methods

### Datasets Downloaded and Identification of Candidate miRNAs

The miRNA profile and clinical information of PCa patients from TCGA database was download through UCSC Xena HUGO probeMap (https://xenabrowser.net/datapages/). The expression level of miRNA is described as log_2_ (RPM+1). A total of 491 patients with recurrence-free survival (RFS) information were selected. The MSKCC cohort, also known as GSE21032, was downloaded from the Cbioportal (http://cbio.mskcc.org/), which included miRNA profile (normalized log_2_ miRNA expression data), and matched clinicopathological features of 105 PCa patients. MSKCC cohort was prepared as the external validation dataset.

### Establishment of the Prognosticate Signature

In order to find out the most suitable miRNA candidates that could be used in different cohorts. Univariate Cox regression analysis was performed to present the RFS-related miRNA candidates in the TCGA cohort, with a cut-off of *P* value less than 0.01. LASSO Cox regression test was performed to select the RFS-related miRNA candidates for the prognostic signature 30. Then, with a linear combination of the coefficients (β) derived from the LASSO Cox regression model combined with the expression of miRNA candidates, a miRNA-related RFS predicting signature was established. The risk score = (β_miRNA#1_ * expression level of miRNA^#1^) + (β_miRNA#2_ * expression level of miRNA^#2^) + (β_miRNA#3_ * expression level of miRNA^#3^) + ⋯ + (β_miRNA#n_ * expression level of miRNA^#n^). Then, with the median risk score calculated above, the patients were assigned into two subgroups which represented the risk level.

### Kaplan-Meier and Receiver Operating Characteristic (ROC) Curve

Kaplan-Meier curves were performed to compare the RFS outcome between the high- and low-risk patients determined by the miRNA-based signature. The ROC curve was plotted with the R package “pROC”. The predictive value was assessed by the area under the ROC curve (AUC). In addition, nomogram ROC analysis was used to synthesize the miRNA-based signature with the clinicopathological features, as well as the laboratory test results. In addition, the Kaplan-Meier plot and log-rank analyses were used to determine the survival difference between high- and low-risk subgroups, and the univariate Cox analysis was used to generate the hazed ratio (HR) and 95% confidential interval (95% CI) among high- and low-risk subgroups.

### Target Gene Pathway Enrichment Analyses and Network Construction

miRNA target genes were predicted based on the R package “multiMiR”, which combines the predict results from miRecords, miRtarbase, and Tarbase databases 31-33. Pathway enrichment analysis was performed using the R package “clusterProfiler”. The downstream genes, which were targeted by at least eight miRNAs were enrolled to establish the miRNA-mRNA interaction network, which was subsequently displayed by the Cytoscape software (San Diego, CA, USA) 34.

## Results

### Characteristics of enrolled patients

The miRNA expression profile derived from the TCGA dataset was set as the training cohort, while the external validation cohort was extracted from the MSKCC database. The detailed clinicopathological features of two cohorts were presented in **Table 1**. In addition, we also performed hierarchical grouping analyses for the TCGA cohort. According to a five-tiered risk features provided by the Cambridge Prognostic Group (CPG) classification for non-metastatic PCa patients, the patients were assigned to TCGA-group-1 and TCGA-group-2 subgroups 35 (**Table 1**).

### Establishment of the Fifteen-MiRNA-Based Prognosticated Classifier by LASSO Cox Regression Analysis

**Figure 1:**
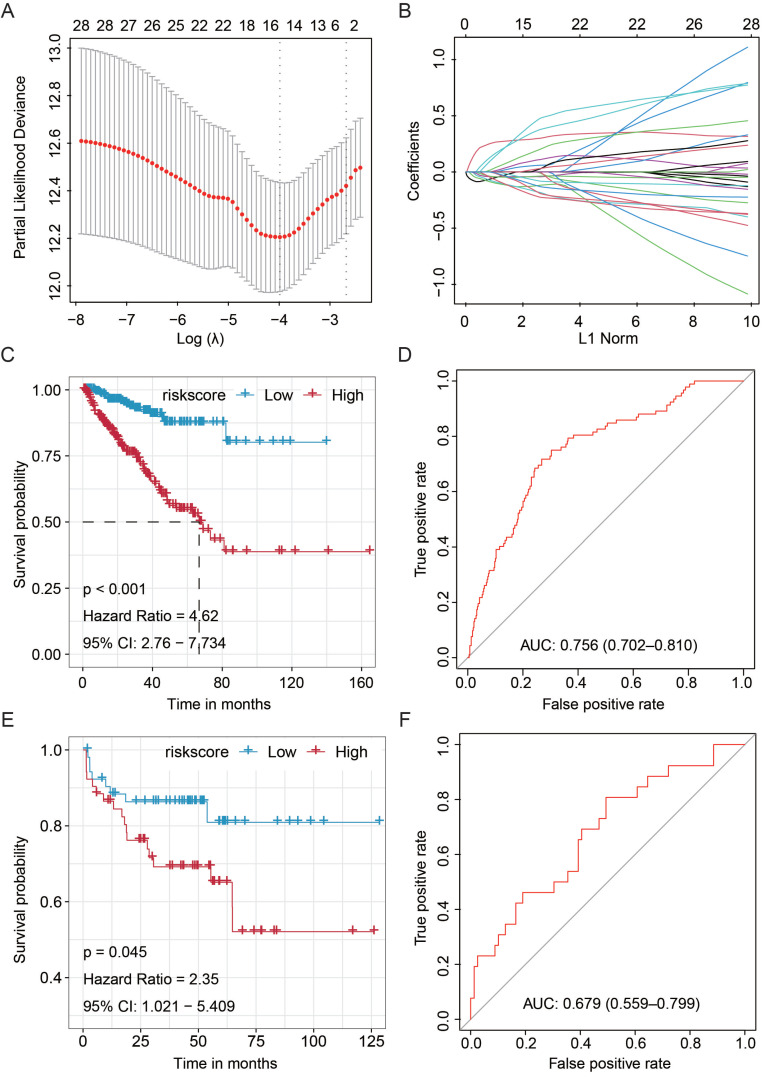
** Construction and validation of the Fifteen-miRNA-based RFS predicting signature. (A)** Optimal parameter (lambda) selection in the LASSO model used ten-fold cross-validation *via* minimum criteria; **(B)** LASSO coefficient profiles of the selected features;** (C)** The Kaplan-Meier analysis on the TCGA training cohort and **(E)** MSKCC validation cohort; ROC curve displayed the predictive value of the fifteen-miRNA-based signature in the training cohort** (D)** and external MSKCC validation cohort **(F)**. TCGA, The Cancer Genome Atlas; MSKCC, Memorial Sloan-Kettering Cancer Center; RFS, recurrence-free survival; LASSO, Least absolute shrinkage and selection operator; HR, hazard ratio.

First, using the TCGA miRNA data matrix (491 patients with available RFS information), univariate Cox regression analyses were performed for each miRNA individually to screen out the RFS-related miRNAs. A total of 28 miRNAs that were proved associated with the RFS of PCa patients were obtained (*P* < 0.05, **Table S1**). Then, the fifteen-miRNA-based RFS predicting signature was established according to the co-ef of each miRNA candidate by LASSO Cox regression analysis (**Fig. 1A-B**, and**Fig. S1**) 36. The recurrence associated-risk score formula = hsa-miR-21-5p * 0.289491318957396 - hsa-miR-222-3p * 0.127897328008817 - hsa-miR-582-5p * 0.157549264897543 - hsa-miR-582-3p * 0.0427286815391999 - hsa-miR-505-3p * 0.135717999372277 - hsa-miR-326 * 0.0417944938919528 + hsa-miR-192-5p * 0.0526251671113488 + hsa-miR-15b-5p * 0.33426964970166 + hsa-miR-106b-5p * 0.0917968239648465 - hsa-miR-212-3p * 0.192801776889974 + hsa-miR-181a-5p * 0.402851982794915 - hsa-miR-296-5p * 0.0612398683637298 + hsa-miR-18a-5p * 0.0146713545780353 + hsa-miR-301a-3p * 0.162223416448074 - hsa-miR-144-3p * 0.100417278760513, according to which, all patients were separated into low- or high-risk groups referring to the median risk score in both the TCGA training and MSKCC validation cohorts. Further analyses would be performed based on these results.

### Testing the Prognosticate Value and Accuracy of the Fifteen-miRNA-Based RFS Predicting Signature

To assess whether the signature could effectively predict the RFS of PCa patients, we performed the Kaplan-Meier and log-rank tests on the TCGA training cohort and MSKCC validation cohort. The patients in the TCGA cohort with higher scores showed unfavorable RFS than those with lower scores (HR = 4.62, 95%CI: 2.76-7.734, *P* < 0.001, **Fig. 1C**). Besides, the Kaplan-Meier analysis was performed on the external MSKCC validation cohort, and similar results were obtained (HR = 2.35, 95%CI: 1.021-5.409, *P* = 0.045, **Fig. 1E**). In addition, the predictive value of the fifteen-miRNA-based signature was determined by ROC analysis, and the AUC values of the training and internal validation cohorts confirmed the high prognosticate value of this signature in predicting RFS of PCa (TCGA training set, AUC = 0.756, 95%CI = 0.702-0.810, **Fig. 1D**; and MSKCC validation set, AUC = 0.679, 95%CI: 0.559-0.799, **Fig. 1F**).

### Validation in Subgroup of Patients Stratified by Hierarchical Grouping

**Figure 2:**
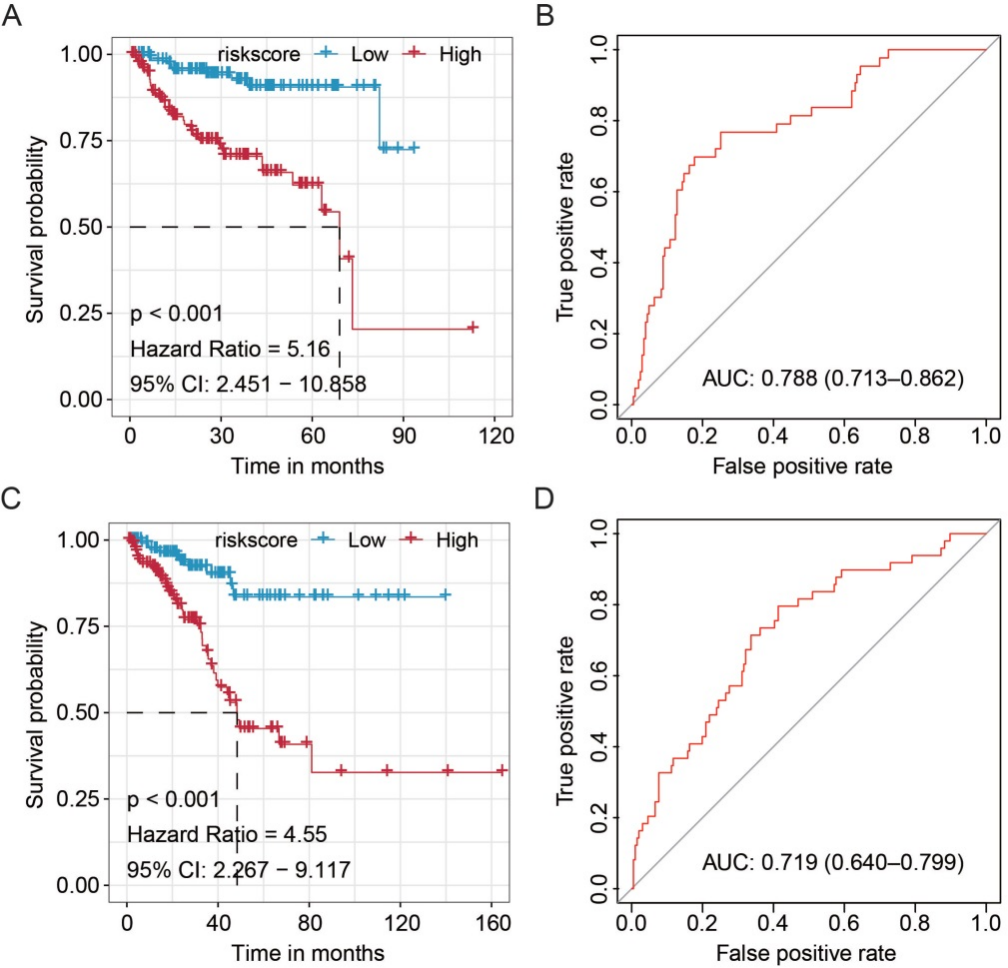
** Validation of the clinical significance and stability of the miRNA-based signature in different clinical subgroup stratified by hierarchical grouping analyses.** The Kaplan-Meier analysis on the TCGA-Group-1 **(A)** and TCGA-Group-2 **(C)**; ROC curve for the prostate cancer patients in the TCGA-Group-1 **(B)** and TCGA-Group-2 **(D)**. TCGA, The Cancer Genome Atlas; ROC, receiver operating characteristic.

To further validate the prognostic value of our newly established miRNA-based signature, we stratified the patients from the TCGA cohort by hierarchical grouping analyses. The patients were subclassified into two similar groups based on the clinicopathological features, as well as laboratory data (**Table 1**). Subsequently, the Kaplan-Meier and ROC analyses were performed to validate the significance and stability of this signature (**Fig. 2**). We found that the signature significantly discriminated high- and low-risk PCa patients in the TCGA-Group-1 (HR = 5.16, 95%CI: 2.451-10.858, *P* < 0.001) and TCGA-Group-2 (HR = 4.55, 95%CI: 2.267-9.117, *P* < 0.001), and also obtained moderate predicting efficacy in both groups (TCGA-Group-1: AUC = 0.788, 95%CI: 0.713-0.862; TCGA-Group-2: AUC = 0.719, 95%CI: 0.640-0.799). All these results proved the stability of the current signature.

### Target Gene Prediction, Network Construction, and Pathway Enrichment

**Figure 3:**
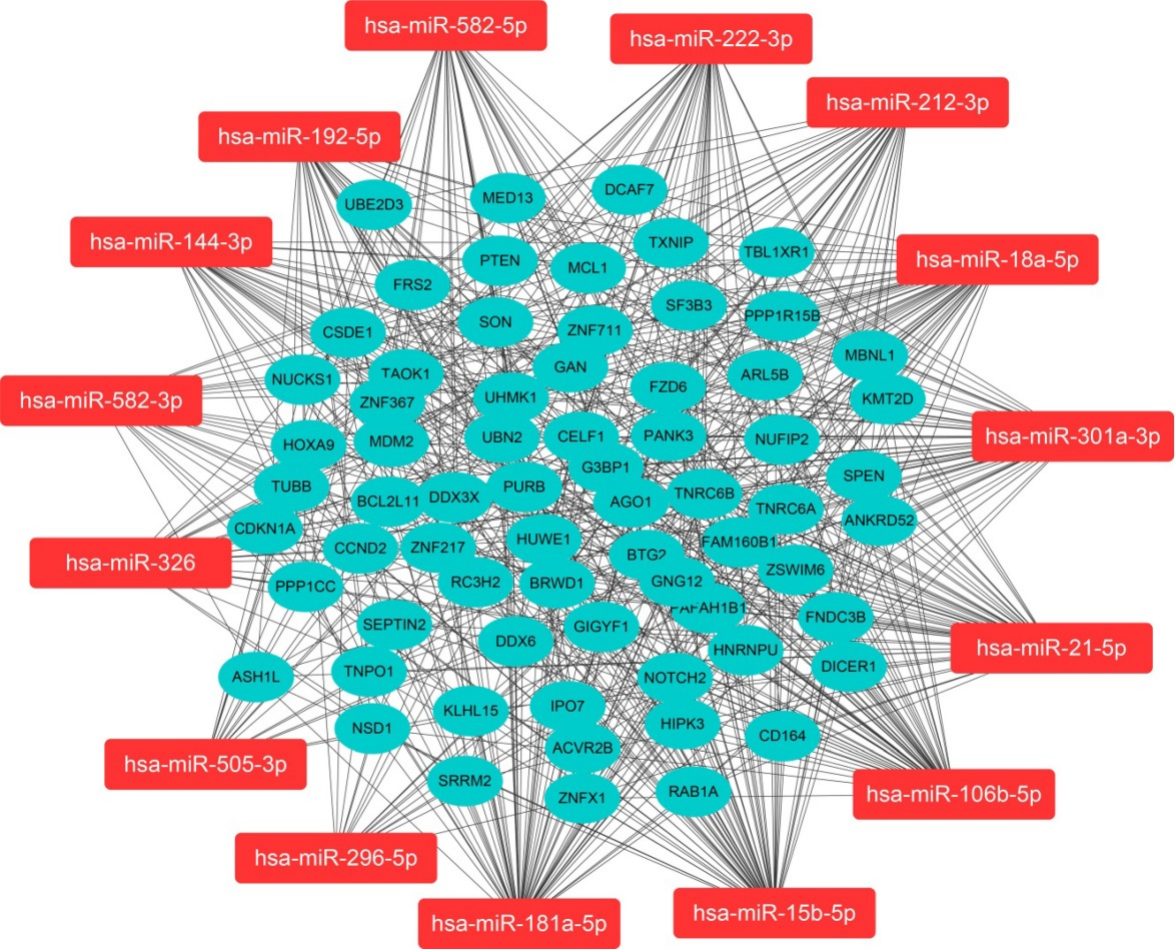
** Regulatory network of the fifteen miRNA markers and their trustable target genes.** MiRNAs were highlighted in red. We filtered out genes that were targeted by at least eight miRNAs to construct the miRNA-mRNA network.

**Figure 4:**
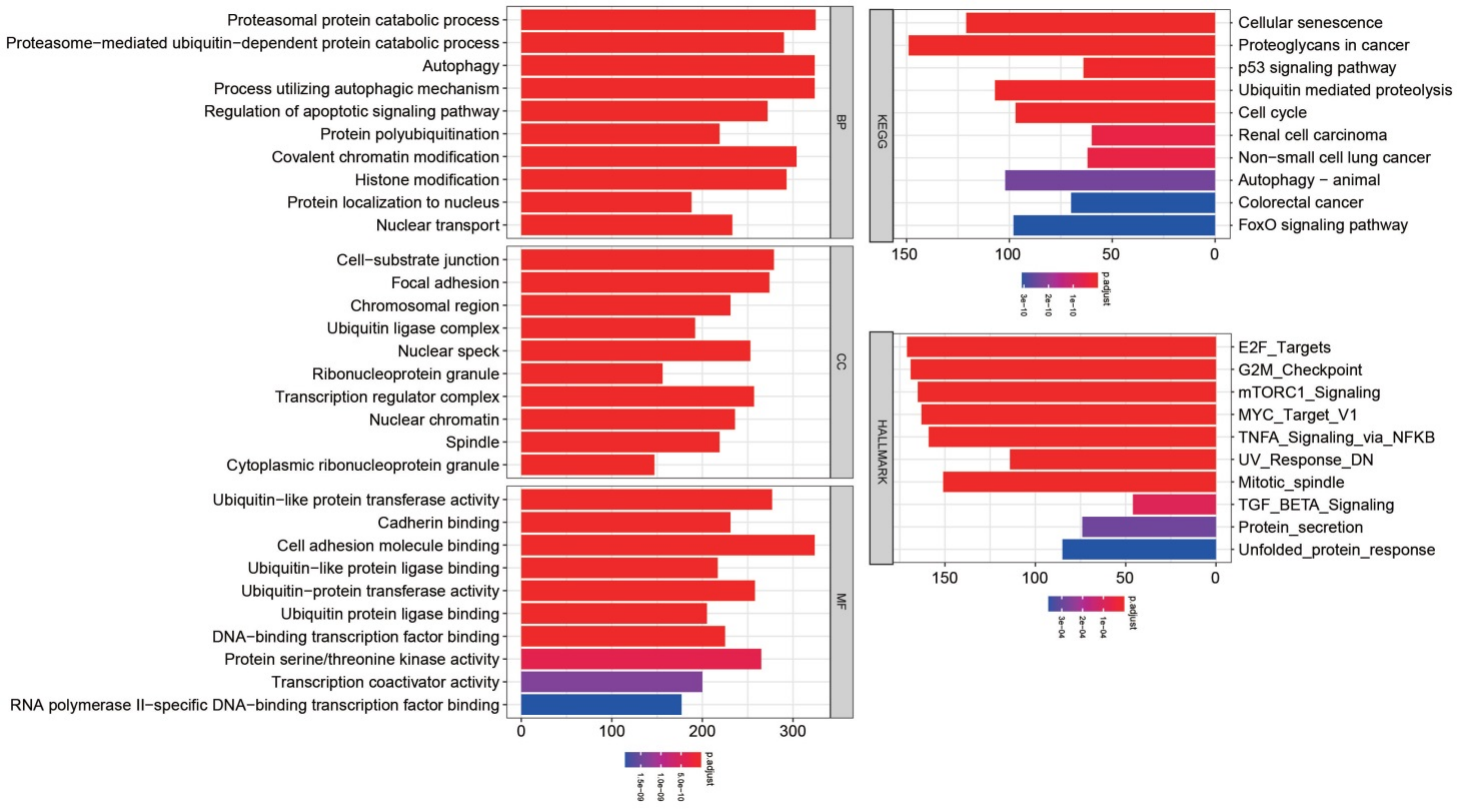
Functional enrichment for the targeted genes of the enrolled fifteen miRNAs.

**Figure 5:**
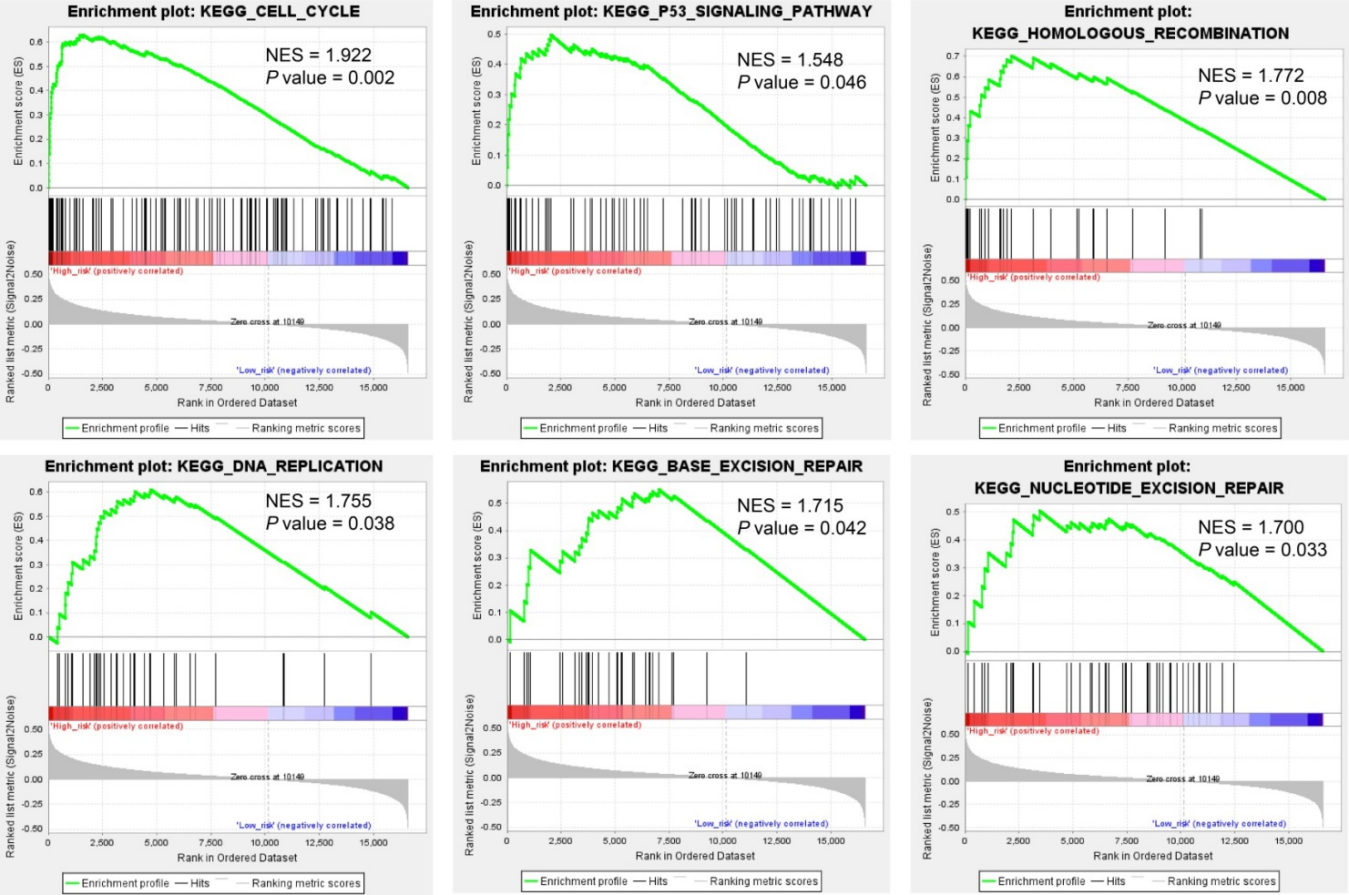
Gene Set Enrichment Analysis (GSEA) analysis compared the difference between high- and low-risk subgroups at the pathway level.

We predicted miRNA target genes using miRecords, miRtarbase, and Tarbase databases, and consequently, filtered out genes that were targeted by at least eight miRNAs and enrolled to construct the miRNA-mRNA network (**Fig. 3**, and**Table S2)**. These miRNAs potentially interact with their downstream genes to influence the tumorigenesis and progression of PCa. In addition, functional enrichment analyses were performed for these downstream genes. The GO-biological process (BP, CC, and MF) revealed that the targeted genes were enriched in Proteasomal protein catabolic process, Autophagy, Regulation of apoptotic signaling pathway, Histone modification, Cell-substrate junction, Focal adhesion, Chromosomal region, Ubiquitin-like protein transferase activity, Cadherin binding, etc. (**Fig. 4**, and**Table S3**). KEGG analysis found that these targeted genes were enriched in Cellular senescence, Proteoglycans in cancer, p53 signaling pathway, etc., while significant enrichment of E2F_Targets, G2M_Checkpoint, mTORC1_Signaling, MYC_Target_V1 pathway was revealed by Hallmark pathway enrichment analysis (**Fig. 4**, and**Table S4**). Highlighted, GSEA analysis was employed to compare the differences between high- and low-risk groups, and the differences were majorly enriched in Cell Cycle, Oocyte Meiosis, Homologous Recombination, DNA Replication and P53 Signaling Pathways (**Fig. 5**, and**Table S5**), which were proved by plenty of works that significantly associated with tumor proliferation and drug resistance.

### Multivariate Analyses Revealed the Prognosis Predicting Value

**Figure 6:**
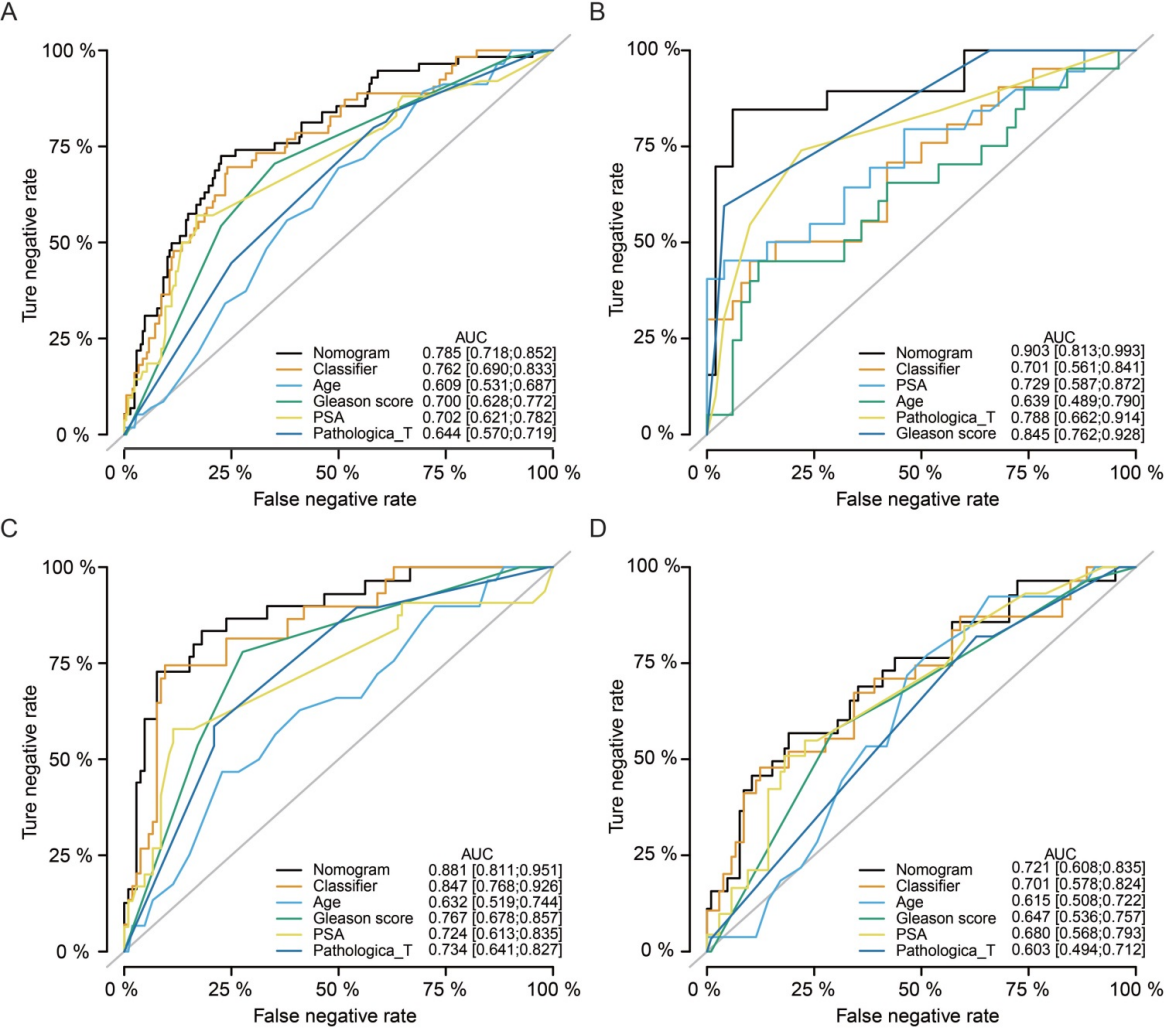
** Nomogram analyses by synthesizing the miRNA-based signature and clinicopathological features.** Nomogram ROC analysis for the TCGA cohort **(A)**, MSKCC cohort **(B)**, TCGA-subgroup-1 **(C)**, and TCGA-subgroup-2 **(D)**. TCGA, The Cancer Genome Atlas; MSKCC, Memorial Sloan-Kettering Cancer Center; ROC, receiver operating characteristic.

Multivariate Cox regression analyses indicated that our classifier was an independent risk factor of recurrence for PCa patients (HR = 2.9, 95%CI: 1.63-5.2, *P* < 0.001), better than Gleason score (HR = 2.3, 95%CI: 1.40-3.9, *P* = 0.001) and pathological T stage (HR = 1.9, 95%CI: 1.04-3.6, *P* = 0.038, **Table 2**). Meanwhile, nomogram ROC analysis was executed to test the synthesis effects after combining the miRNA-based signature with clinicopathological features (**Fig. 6A**). The AUC value of the nomogram (AUC = 0.785, 95%CI: 0.718-0.852) was slightly higher than our signature (AUC = 0.762, 95%CI: 0.690-0.833), and higher than Age (AUC = 0.609, 95%CI: 0.531-0.687), Gleason score (AUC = 0.700, 95%CI: 0.628-0.772), and PSA level (AUC = 0.702, 95%CI: 0.621-0.782). In addition, we also analyzed the synthesized effects in the MSKCC cohort, TCGA-subgroup-1, and TCGA-subgroup-2 cohorts, and similar results obtained as indicated in the TCGA cohort (**Fig. 6B-D**).

**Figure 7:**
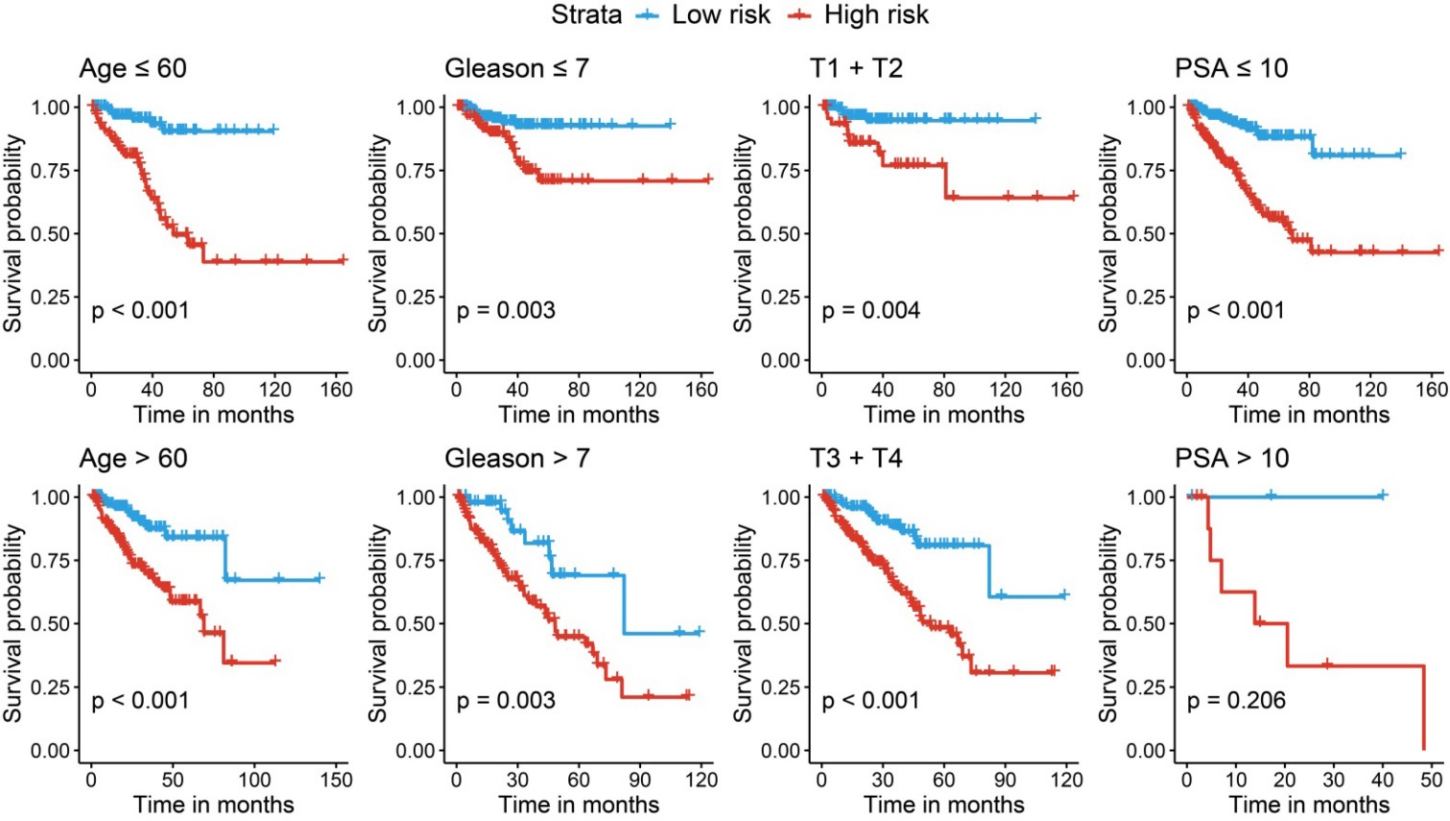
** Stratified analyses for different clinicopathological subgroups in the TCGA cohort.** TCGA, The Cancer Genome Atlas.

We further performed stratified survival analyses to evaluate the prognosticate values of our risk model in different subgroups. According to **Fig. 7**, in general, the miRNA signature could distinguish the recurrence risk for different age populations (≤ 60 or > 60) and Gleason score populations (≤ 7 or > 7). Although the novel miRNA signature could only indicate the recurrence risk in patients whose PSA was lower than 10 ng/ml (*P* < 0.001) subgroup, the tendency was satisfied in PSA > 10 ng/ml subgroup, and the failed statistical analyses were potentially due to limited sample size.

## Discussion

In the United States, PCa accounts for the majority in newly diagnosed cases and ranks as the third-leading cause of cancer-specific death 37. At an early stage, most PCa patients could benefit from curative treatment and acquire a relatively good prognosis; however, advanced PCa patients tend to have poor prognoses due to recurrence or distant metastases 38. Thus, the identification of biomarkers to predict the prognosis of PCa patients is warranted, which could clinically benefit the decision-making process. Nevertheless, few effective biomarkers that could be used for prognosis prediction are currently available.

It is clear that miRNAs play pivotal roles among various cellular processes and are also involved in the initiation and progression of various cancers 39. Here, we established and validated a stable fifteen-miRNA-based signature that could be used to predict the prognosis of PCa patients. The results showed that this classifier effectively assigns PCa patients into high- or low-risk groups, and found that the patients categorized in the high-risk group were more likely to have unfavorable RFS rate. These results were confirmed in the external MSKCC validation cohort. The multivariate analysis found the classifier was an independent risk factor for recurrence of PCa, and even better than Gleason score, PSA level, and pathological T grade. Nomogram analyses found that the classifier adds value to the currently available staging system. In addition, the targeted genes were predicted by three online databases, and the functions of these downstream genes were annotated by GSEA, KEGG, and GO analyses. All these results prove the clinical significance of the miRNA-based signature and predict the underlying mechanisms of how these miRNAs influence the tumor progression.

He *et al.* 40 found that the increased expression of hsa-miR-21-5p suppresses the proliferation and migration of colon cancer cells* in vivo* and* in vitro*, which means the overexpression of hsa-miR-21-5p may promote the prognosis of cancer patients. Elevated expression of hsa-miR-222-3p has been reported in diverse cancers, and it promotes the proliferation, invasion, metastasis, and immune escape of cancer cells 41. Cheng *et al.* 42 found that hsa-miR-222-3p serves as an independent risk factor in the recurrence of prostate cancer. Fang *et al.* 43 found that high expression of hsa-miR-582-3p is associated with unfavorable prognosis of lung cancer patients, and it maintains the stem cell-like traits of lung cancer by activating Wnt/β-catenin signaling. Chen *et al.* 44 established a miRNA-based prognostic signature and the higher expression of hsa-miR-326 is linked to unfavorable overall survival among non-small cell lung cancer (NSCLC) patients. Zou *et al.* 45 demonstrated that suppressing miR-192-5p expression regulates lung cancer cell proliferation, migration, and invasion by negatively regulating TRIM44 expression. Similar results are obtained by Zheng *et al.* 46 that miRNA‑192 plays an oncogenic role in colon cancer, and simvastatin inhibits cancer cell growth by activating miR-192. Li *et al.* 47 found that downregulation of miR-181a-5p is observed in aggressive breast and colon cancers, and it inhibits the migration and angiogenesis *via* downregulation of MMP14; furthermore, the role of miR-181a-5p is consistent in prostate cancer study 48. For miR-144-3p, researchers found that it promotes the growth and metastasis of papillary thyroid carcinoma by targeting the PAX8 gene 49. While for other candidates, few studies report their expression and function in cancers, or their prognosis effects are not completely consistent with previous publications.

Highlighted, it is interesting to explore the differences between high- and low-risk PCa populations. GSEA analysis suggested that the differential expressed genes were mostly enriched in Cell Cycle, Oocyte Meiosis, Base Excision Repair, Homologous Recombination, DNA Replication, Spliceosome, Nucleotide Excision Repair, and P53 Signaling Pathways. Cancer is demonstrated as uncontrolled cell proliferation attributing to aberrant activities of various cell cycle-related proteins; thus, regulators of the cell cycle process are considered as therapeutic targets in cancers 50. For example, PARP inhibitors are highly successful used in treating BRCA1/BRCA2-mutant tumors. DNA replication has been proved to play a critical role in tumor cell proliferation. Mutations in DNA replication genes could cause hereditary forms of colorectal, breast, ovary, and skin cancers 51-54. P53 is regarding as an extraordinary multifunctional protein, which participates in the regulation of cell cycle, differentiation, immune response, DNA repair, *etc*. 55-59 In addition, the localization of p53 at active replication forks and the p53-dependent effects on DNA elongation indicates that the p53 protein involves in the DNA replication process 60, 61. Combined these results, dysregulation of DNA replication and cell cycle processes were potential causes of tumor progression. For other significantly enriched pathways, many reports also indicated their significance in cancer progression. Our results present a novel genetic aspect of prostate cancer recurrence, which will benefit to the personalized treatment for these patients.

## Conclusion

Overall, we establish a novel fifteen-miRNA-based RFS prediction signature for PCa patients, which can successfully classify PCa patients into low- and high-risk groups and predict their prognosis. The novel miRNA signature is a reliable tool to assess the prognosis of PCa patients. Currently, our center is ongoing to collect PCa tissues and serum samples and would validate these findings in the near future.

## Highlights

- The fifteen-miRNA-based signature is able to predict recurrence-free survival of prostate cancer patients;
- The signature serves as an independent indicator of prognosis and adds predictive value to the currently available staging system;
- The potential miRNA-mRNA interaction and the underlying mechanisms of how these miRNA influence tumor progressions have been investigated, which provide clues to basic research in the future.

## Supplementary Material

Supplementary figures and tables.

## Figures and Tables

**Table 1:** Clinicopathological features of prostate cancer patients enrolled in each cohort

Parameters	TCGA	TCGA-subgroup-1	TCGA-subgroup-2	*P*-value^$^	MSKCC
Patients, number	491	246	245		105
**Age, years old**					
≤60	220	113	107	0.614	65
>60	271	133	138		40
Gleason*				0.488	
6	45	22	23		32
7	244	129	115		55
8	63	34	29		8
9	136	60	76		8
10	3	1	2		-
**PSA^#^, ng/dl**				0.151	
≤10	420	212	208		84
>10	16	11	5		20
**T Stage^†^**				0.262	
≤T2	186	87	99		69
>T2	298	155	143		36

^$^Different distribution of features between TCGA-PRAD based Group 1 and Group 2 was conducted by Chi-square test; *Lack of Gleason score: 2 in MSKCC; ^#^Lack of PSA value: 55 in TCGA, 23 in Group 1, 32 in Group 2, 1 in MSKCC; ^†^Lack of T Stage value: 7 in TCGA, 4 in Group 1, 3 in Group 2; TCGA: The Cancer Genome Atlas; MSKCC: Memorial Sloan-Kettering Cancer Center.

**Table 2:** Multivariate Cox analysis among the risk score and clinical features

Parameters	Number	OR	95%CI	*P-*value
**Age**				
≤ 60	193	reference		
> 60	237	1.144	0.737-1.777	0.549
**Gleason score**				
≤ 7	253	reference		
> 7	177	2.348	1.399-3.942	0.001*
**Pathological T stage**				
T1 + T2	164	reference		
T3 + T4	266	1.946	1.039-3.647	0.038*
**PSA**				
≤ 10	415	reference		
> 10	15	2.252	0.898-5.645	0.083
**Risk**				
Low-risk	216	reference		
High-risk	214	2.918	1.632-5.216	< 0.001*

* *P* < 0.05; OR: odds ratio; 95%CI: 95% confidential interval; PSA: prostate-specific antigen.
