# Supplementary material for: Fifteen-MiRNA-Based Signature Is a Reliable Prognosis-Predicting Tool for Prostate Cancer Patients
Source: Int J Med Sci. 2021 Jan 1;18(1):284–94. doi: 10.7150/ijms.49412 (PMC7738977; doi:10.7150/ijms.49412)
Supplement: Supplementary file 1 — Supplementary figures and tables. [file ijmsv18p0284s1.pdf]

Fig. S1

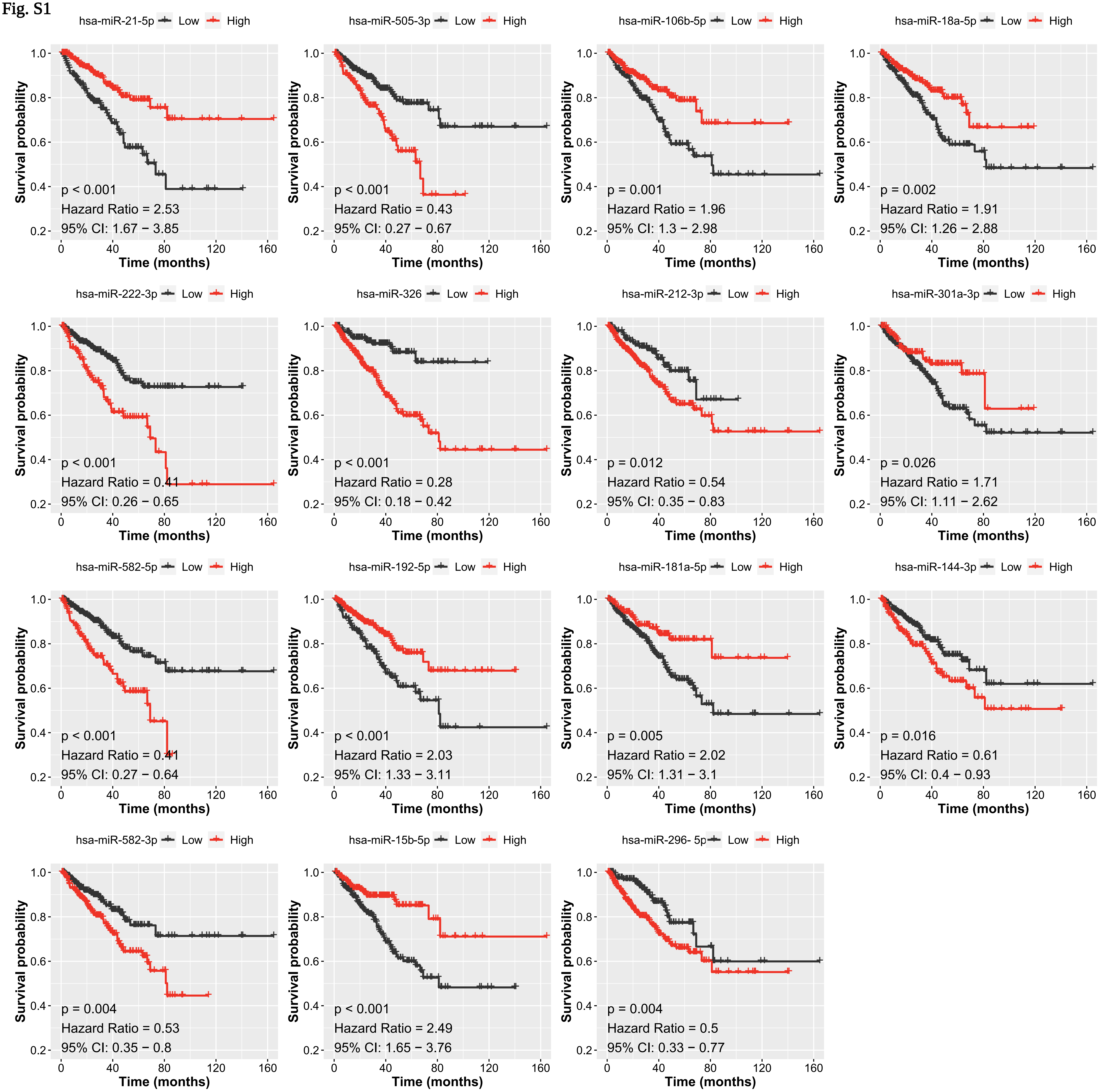
hsa-miR-21-5p Low High
p < 0.001
Hazard Ratio = 2.53
95% CI: 1.67 − 3.85
hsa-miR-505-3p Low High
p < 0.001
Hazard Ratio = 0.43
95% CI: 0.27 − 0.67
hsa-miR-106b-5p Low High
p = 0.001
Hazard Ratio = 1.96
95% CI: 1.3 − 2.98
hsa-miR-18a-5p Low High
p = 0.002
Hazard Ratio = 1.91
95% CI: 1.26 − 2.88
hsa-miR-222-3p Low High
p < 0.001
Hazard Ratio = 0.41
95% CI: 0.26 − 0.65
hsa-miR-326 Low High
p < 0.001
Hazard Ratio = 0.28
95% CI: 0.18 − 0.42
hsa-miR-212-3p Low High
p = 0.012
Hazard Ratio = 0.54
95% CI: 0.35 − 0.83
hsa-miR-301a-3p Low High
p = 0.026
Hazard Ratio = 1.71
95% CI: 1.11 − 2.62
hsa-miR-582-5p Low High
p < 0.001
Hazard Ratio = 0.41
95% CI: 0.27 − 0.64
hsa-miR-192-5p Low High
p < 0.001
Hazard Ratio = 2.03
95% CI: 1.33 − 3.11
hsa-miR-181a-5p Low High
p = 0.005
Hazard Ratio = 2.02
95% CI: 1.31 − 3.1
hsa-miR-144-3p Low High
p = 0.016
Hazard Ratio = 0.61
95% CI: 0.4 − 0.93
hsa-miR-582-3p Low High
p = 0.004
Hazard Ratio = 0.53
95% CI: 0.35 − 0.8
hsa-miR-15b-5p Low High
p < 0.001
Hazard Ratio = 2.49
95% CI: 1.65 − 3.76
hsa-miR-296- 5p Low High
p = 0.004
Hazard Ratio = 0.5
95% CI: 0.33 − 0.77
Survival probability
Time (months)

Table S1. Univariate Cox regression analysis

ID	HR	HR.95L	HR.95H	P-value
hsa-miR-133b	0.720738915	0.62978066	0.824834131	1.96E-06
hsa-miR-21-5p	2.293514183	1.592331035	3.303463408	8.24E-06
hsa-miR-222-3p	0.585724692	0.462117875	0.742393734	9.73E-06
hsa-miR-221-3p	0.628602924	0.507618311	0.778422739	2.08E-05
hsa-miR-582-5p	0.645496672	0.51020291	0.816667143	2.65E-04
hsa-miR-582-3p	0.675577214	0.546044109	0.835838286	3.05E-04
hsa-miR-93-5p	1.695080001	1.268729222	2.264704052	3.57E-04
hsa-miR-505-3p	0.460602165	0.300346276	0.706365855	3.80E-04
hsa-miR-326	0.708320169	0.585514537	0.856883014	3.85E-04
hsa-miR-192-5p	1.641562675	1.240710109	2.171923962	5.21E-04
hsa-miR-15b-5p	1.733520666	1.269259777	2.367595627	5.42E-04
hsa-miR-106b-5p	1.84326764	1.298327947	2.616931724	6.26E-04
hsa-miR-15a-5p	1.802478532	1.268596612	2.561041729	1.01E-03
hsa-miR-212-3p	0.668049262	0.523381727	0.852704239	1.20E-03
hsa-miR-125b-5p	0.571318312	0.403289376	0.809355843	1.63E-03
hsa-miR-145-5p	0.682341218	0.537284107	0.866561159	1.72E-03
hsa-miR-181a-5p	1.920562213	1.276906054	2.888669221	1.73E-03
hsa-miR-139-3p	0.740017771	0.612110877	0.894652134	1.87E-03
hsa-miR-96-5p	1.443203983	1.138950253	1.8287346	2.39E-03
hsa-miR-425-5p	1.463095931	1.137365826	1.882111855	3.06E-03
hsa-miR-103a-3p	1.722867468	1.185288851	2.504260721	4.36E-03
hsa-miR-194-5p	1.644620377	1.160384484	2.330931017	5.18E-03
hsa-miR-296-5p	0.737652508	0.595443167	0.913825621	5.36E-03
hsa-miR-574-3p	0.56563673	0.378516348	0.845260481	5.43E-03
hsa-miR-197-3p	1.791313075	1.158605756	2.769537882	8.74E-03
hsa-miR-18a-5p	1.390670331	1.086602533	1.779826488	8.80E-03
hsa-miR-301a-3p	1.513363103	1.109956508	2.063385245	8.81E-03
hsa-miR-144-3p	0.775094694	0.63935999	0.939645573	9.49E-03
hsa-miR-499a-5p	0.742178868	0.58962091	0.934209528	1.11E-02
hsa-miR-374b-5p	0.640525137	0.445678711	0.920556537	1.61E-02
hsa-miR-139-5p	0.716812304	0.544524247	0.943612488	1.76E-02
hsa-miR-501-3p	1.352284183	1.053356516	1.736043289	1.79E-02
hsa-miR-20a-5p	1.326580997	1.048824453	1.677894845	1.84E-02
hsa-miR-204-5p	0.789253804	0.64822533	0.960964557	1.85E-02
hsa-miR-378a-3p	0.745947036	0.578709935	0.96151275	2.36E-02
hsa-miR-127-3p	0.743513836	0.574403696	0.962411677	2.44E-02
hsa-miR-379-5p	0.739702657	0.568561563	0.962358445	2.47E-02
hsa-miR-100-5p	0.759486357	0.597232857	0.965820148	2.49E-02
hsa-miR-32-5p	1.332825315	1.033925138	1.71813534	2.66E-02
hsa-miR-206	0.912011794	0.840357077	0.989776294	2.74E-02
hsa-miR-143-3p	0.795058475	0.648442202	0.974825476	2.74E-02
hsa-miR-340-5p	1.343138747	1.031143153	1.749535638	2.87E-02
hsa-miR-29c-3p	0.665938892	0.458238739	0.9677807	3.30E-02
hsa-miR-484	1.507598077	1.031995985	2.202384501	3.38E-02
hsa-miR-30e-5p	0.582486266	0.351635617	0.964891591	3.58E-02
hsa-miR-660-5p	1.40741484	1.01594792	1.949722511	3.99E-02
hsa-let-7g-5p	1.619858131	1.014009828	2.58768731	4.36E-02
hsa-miR-454-3p	1.366727103	1.007873433	1.853350742	4.44E-02
hsa-miR-199a-5p	1.45987879	1.006115388	2.118291904	4.64E-02
hsa-miR-126-3p	1.345779132	0.997946619	1.814848046	5.16E-02
hsa-miR-324-3p	0.738321749	0.543606386	1.002782562	5.21E-02
hsa-miR-182-5p	1.236027152	0.994619182	1.53602821	5.60E-02
hsa-miR-151a-5p	1.448412544	0.986911713	2.125720943	5.84E-02
hsa-miR-183-5p	1.232995826	0.992002945	1.53253447	5.91E-02
hsa-miR-34b-3p	1.175080236	0.993489335	1.38986249	5.96E-02
hsa-miR-26a-5p	0.670675561	0.442232416	1.017125139	6.01E-02

hsa-miR-23b-3p	0.702749013	0.485577441	1.017049258	6.14E-02
hsa-miR-1224-5p	1.223336401	0.990381936	1.51108567	6.14E-02
hsa-miR-29a-3p	0.679711928	0.451801141	1.02259216	6.39E-02
hsa-miR-16-5p	1.51963657	0.961601099	2.401510674	7.31E-02
hsa-miR-148b-3p	1.547150171	0.956818207	2.501701611	7.51E-02
hsa-miR-106a-5p	1.187020401	0.979118959	1.439066642	8.10E-02
hsa-miR-483-5p	0.769336951	0.567955906	1.042121999	9.04E-02
hsa-miR-654-3p	0.799309909	0.614945596	1.038947729	9.41E-02
hsa-miR-491-5p	0.813512099	0.638558774	1.036399407	9.48E-02
hsa-miR-615-3p	1.147970034	0.97624354	1.349904144	9.51E-02
hsa-miR-141-3p	1.214963924	0.966070211	1.527981424	9.59E-02
hsa-miR-19a-3p	1.198640527	0.968085402	1.484103686	9.64E-02
hsa-miR-135a-5p	0.832008261	0.668023047	1.036248299	1.01E-01
hsa-miR-146b-5p	1.214297277	0.957408575	1.540113506	1.09E-01
hsa-miR-532-3p	0.774686923	0.565927325	1.060453883	1.11E-01
hsa-miR-542-5p	0.843926868	0.683558525	1.041918917	1.15E-01
hsa-miR-940	1.198356677	0.954609855	1.504340981	1.19E-01
hsa-miR-432-5p	0.814352932	0.627571038	1.056726104	1.22E-01
hsa-miR-766-3p	0.788053926	0.58196306	1.067127854	1.24E-01
hsa-miR-132-3p	0.740690573	0.504979743	1.086424818	1.25E-01
hsa-miR-218-5p	0.741863221	0.506200756	1.087238672	1.26E-01
hsa-miR-18b-5p	1.167162809	0.955674913	1.425452321	1.30E-01
hsa-miR-185-5p	1.374403296	0.910891462	2.073775526	1.30E-01
hsa-miR-423-5p	1.230151314	0.935085134	1.618325647	1.39E-01
hsa-miR-338-5p	0.867577029	0.717935846	1.048408301	1.41E-01
hsa-miR-129-5p	1.116902451	0.96215199	1.296542644	1.46E-01
hsa-miR-199b-5p	1.249846034	0.924793392	1.68915038	1.47E-01
hsa-miR-671-5p	1.18907604	0.936825068	1.509248502	1.55E-01
hsa-miR-155-5p	0.839074216	0.658207554	1.069640625	1.57E-01
hsa-miR-30a-5p	0.814997222	0.613160219	1.083273916	1.59E-01
hsa-miR-526b-5p	1.17109755	0.938903308	1.460714282	1.61E-01
hsa-miR-423-3p	1.362943863	0.876679413	2.118922774	1.69E-01
hsa-miR-550a-5p	0.848621793	0.671097345	1.073106535	1.70E-01
hsa-miR-99a-5p	0.79324401	0.569333575	1.105215091	1.71E-01
hsa-miR-374a-5p	0.780045458	0.543637833	1.119257859	1.78E-01
hsa-miR-25-3p	1.294833122	0.888659517	1.886653755	1.79E-01
hsa-let-7f-5p	1.171846281	0.924858067	1.484793998	1.89E-01
hsa-miR-451a	0.876072695	0.717011773	1.070419477	1.96E-01
hsa-miR-150-5p	0.872782481	0.709846909	1.073117667	1.97E-01
hsa-miR-338-3p	1.130174845	0.937886137	1.361887259	1.98E-01
hsa-miR-181b-5p	1.319641328	0.861626143	2.021123951	2.02E-01
hsa-miR-455-3p	0.857632258	0.677094684	1.086307584	2.03E-01
hsa-miR-877-5p	1.172677713	0.917556098	1.498734543	2.03E-01
hsa-miR-19b-3p	1.171601331	0.914066044	1.501696389	2.11E-01
hsa-miR-299-5p	0.869402283	0.695787517	1.086337871	2.18E-01
hsa-miR-125a-5p	0.787787092	0.538059591	1.153419643	2.20E-01
hsa-miR-339-3p	1.251212024	0.873831669	1.791571062	2.21E-01
hsa-miR-361-5p	1.466671496	0.793258395	2.711758602	2.22E-01
hsa-let-7e-5p	1.220179092	0.886355318	1.67972932	2.22E-01
hsa-miR-376a-3p	0.8530169	0.65772077	1.106302043	2.31E-01
hsa-miR-502-5p	0.871984791	0.695765692	1.092835539	2.34E-01
hsa-miR-214-3p	0.866843117	0.684522673	1.097724034	2.36E-01
hsa-miR-149-5p	1.126725201	0.922390963	1.376324931	2.43E-01
hsa-miR-342-3p	1.230451799	0.867642285	1.744972156	2.45E-01
hsa-miR-377-3p	1.151753291	0.903941117	1.467502273	2.53E-01
hsa-miR-491-3p	1.148838425	0.902744215	1.462019589	2.59E-01
hsa-miR-652-3p	1.253991827	0.841122433	1.869520347	2.67E-01
hsa-miR-28-3p	0.803914294	0.542647768	1.190971805	2.76E-01

hsa-miR-130b-3p	1.16112052	0.883207325	1.52648288	2.85E-01
hsa-miR-92b-3p	0.866776837	0.666468238	1.127288657	2.86E-01
hsa-miR-455-5p	0.867574441	0.668004035	1.126767762	2.87E-01
hsa-miR-363-3p	1.121566938	0.904505002	1.390719117	2.96E-01
hsa-miR-148a-3p	0.846719458	0.614618347	1.166469963	3.09E-01
hsa-miR-7-5p	1.102093073	0.906389504	1.34005208	3.30E-01
hsa-miR-330-3p	0.888680677	0.69403738	1.137911832	3.49E-01
hsa-miR-769-5p	1.203988887	0.81191378	1.785398	3.56E-01
hsa-miR-151a-3p	0.83125501	0.560043618	1.233805492	3.59E-01
hsa-miR-424-5p	0.897883761	0.710485777	1.134709903	3.67E-01
hsa-miR-539-5p	0.907740951	0.733685515	1.123088321	3.73E-01
hsa-miR-497-5p	1.166412729	0.819822983	1.659527341	3.92E-01
hsa-miR-502-3p	0.852844127	0.591040427	1.230614814	3.95E-01
hsa-miR-140-3p	1.193016744	0.792145997	1.796750796	3.98E-01
hsa-miR-331-3p	0.912454177	0.735028205	1.132708404	4.06E-01
hsa-miR-382-5p	1.149007598	0.824499815	1.601235604	4.12E-01
hsa-miR-590-5p	1.137816827	0.83530407	1.549887256	4.13E-01
hsa-miR-205-5p	0.964730784	0.884410983	1.052345011	4.18E-01
hsa-miR-33b-5p	0.933235812	0.787437833	1.106029002	4.25E-01
hsa-miR-337-3p	1.133826281	0.831572536	1.545940951	4.27E-01
hsa-miR-10b-5p	1.094313352	0.872067637	1.373198203	4.36E-01
hsa-miR-92a-3p	1.151777051	0.801775733	1.654565385	4.45E-01
hsa-miR-193b-3p	1.110669515	0.845115697	1.459666144	4.52E-01
hsa-miR-27a-3p	1.164404463	0.772248556	1.755701249	4.68E-01
hsa-miR-483-3p	1.075403764	0.883693508	1.308704032	4.68E-01
hsa-miR-30b-5p	1.107744917	0.838716893	1.463066752	4.71E-01
hsa-miR-421	0.915788143	0.718676656	1.166961409	4.77E-01
hsa-miR-23a-3p	0.859316095	0.565096673	1.306721817	4.78E-01
hsa-miR-200b-3p	0.958315387	0.851561848	1.07845177	4.80E-01
hsa-miR-409-3p	0.889091851	0.63962343	1.235858916	4.84E-01
hsa-miR-625-5p	1.092319852	0.849326555	1.404833808	4.92E-01
hsa-miR-769-3p	0.917218071	0.709726312	1.185371002	5.09E-01
hsa-miR-181c-5p	1.119858013	0.80006749	1.567470226	5.09E-01
hsa-miR-34a-5p	0.883694685	0.606326167	1.287947541	5.20E-01
hsa-miR-154-5p	0.925754363	0.726496594	1.179662984	5.33E-01
hsa-miR-196b-5p	0.914591579	0.681557055	1.227304083	5.52E-01
hsa-miR-33a-5p	1.051569618	0.887694789	1.245696916	5.61E-01
hsa-miR-107	1.122202785	0.750585675	1.677808588	5.74E-01
hsa-miR-24-3p	0.910922734	0.645805549	1.284876274	5.95E-01
hsa-miR-744-5p	1.086194962	0.80023689	1.474337799	5.96E-01
hsa-miR-193a-3p	1.075195312	0.814150319	1.419940435	6.09E-01
hsa-miR-532-5p	1.102511158	0.739666691	1.643349455	6.32E-01
hsa-miR-193a-5p	0.923350483	0.655900338	1.299856191	6.48E-01
hsa-miR-500a-5p	1.063943354	0.808296752	1.400445391	6.58E-01
hsa-miR-760	0.942724207	0.720236017	1.233941248	6.68E-01
hsa-miR-629-5p	0.944130104	0.724610182	1.230153366	6.70E-01
hsa-miR-29b-3p	1.090286843	0.72627228	1.636748962	6.77E-01
hsa-miR-361-3p	1.092792864	0.71760733	1.664136073	6.79E-01
hsa-miR-365a-3p	1.064068603	0.776971319	1.457250692	6.99E-01
hsa-miR-429	1.022407278	0.912369731	1.145716047	7.03E-01
hsa-miR-224-5p	0.962582136	0.784946538	1.180417167	7.14E-01
hsa-miR-186-5p	0.88517508	0.460959353	1.699791787	7.14E-01
hsa-miR-584-5p	1.05128133	0.791407665	1.396489426	7.30E-01
hsa-miR-335-5p	0.960792533	0.761850274	1.211684661	7.35E-01
hsa-miR-485-3p	0.958777537	0.750411769	1.224999932	7.36E-01
hsa-miR-142-3p	1.033794178	0.851828933	1.254630315	7.37E-01
hsa-miR-142-5p	1.041221898	0.817006467	1.326969964	7.44E-01
hsa-miR-146a-5p	0.971911405	0.793286739	1.190757052	7.83E-01

hsa-miR-101-3p	0.949808226	0.652743482	1.382067675	7.88E-01
hsa-miR-195-5p	0.962688643	0.721272764	1.284908385	7.96E-01
hsa-miR-188-5p	1.033620825	0.794056696	1.345460616	8.06E-01
hsa-miR-362-5p	1.028453015	0.821563428	1.287442417	8.07E-01
hsa-miR-27b-3p	0.956407022	0.648867105	1.409709916	8.22E-01
hsa-miR-342-5p	1.036263044	0.751217915	1.429466837	8.28E-01
hsa-miR-22-3p	0.948123959	0.585442226	1.535487196	8.29E-01
hsa-miR-26b-5p	0.958198792	0.633641099	1.448998378	8.40E-01
hsa-miR-28-5p	1.063412772	0.572161323	1.976447337	8.46E-01
hsa-miR-99b-5p	0.960747446	0.635198357	1.453145531	8.50E-01
hsa-miR-551b-3p	1.021979211	0.812324187	1.285744688	8.53E-01
hsa-miR-223-3p	1.024409664	0.758320157	1.383868212	8.75E-01
hsa-miR-125a-3p	1.01963257	0.798229718	1.30244534	8.76E-01
hsa-miR-200a-3p	0.990900176	0.872611804	1.125223328	8.88E-01
hsa-miR-452-5p	0.984876301	0.778383954	1.24614764	8.99E-01
hsa-miR-362-3p	0.984398394	0.766671892	1.263956862	9.02E-01
hsa-miR-345-5p	1.014315402	0.773980969	1.32927782	9.18E-01
hsa-miR-196a-5p	1.006284657	0.859150526	1.178616296	9.38E-01
hsa-miR-31-5p	0.994162961	0.853197835	1.158418308	9.40E-01
hsa-miR-200c-3p	0.989627207	0.735980375	1.330690386	9.45E-01
hsa-miR-130a-3p	0.992250657	0.718030844	1.371196482	9.62E-01
hsa-miR-30c-5p	0.992353813	0.700942214	1.404917652	9.65E-01
hsa-miR-136-5p	1.003921218	0.804566098	1.252672483	9.72E-01
hsa-miR-324-5p	0.996988716	0.708626824	1.402693868	9.86E-01
hsa-miR-140-5p	0.997770932	0.648123489	1.536044981	9.92E-01
hsa-miR-30d-5p	0.999375054	0.699780311	1.427234353	9.97E-01
hsa-miR-10a-5p	1.000074704	0.773632939	1.292795799	1.00E+00

HR: hazard ratio; HR.95L, hazard ratio low; HR.95.H, hazard ratio high

Table S2. The predicted targeted genes of the eight miRNAs

Database	Mature_miRNA_ID	Target_symbol	Target_entrez	Target_ensembl	Pubmed_ID	Support_type
mirecords	hsa-miR-222-3p	KIT	3815	ENSG00000157404	16330772	validated
mirecords	hsa-miR-21-5p	TPM1	7168	ENSG00000140416	17363372	validated
mirecords	hsa-miR-181a-5p	HOXA11	3207	ENSG00000005073	16489342	validated
mirecords	hsa-miR-21-5p	NFIB	4781	ENSG00000147862	18384814	validated
mirecords	hsa-miR-21-5p	PDCD4	27250	ENSG00000150593	18372920	validated
mirecords	hsa-miR-106b-5p	E2F1	1869	ENSG00000101412	18328430	validated
mirecords	hsa-miR-106b-5p	CDKN1A	1026	ENSG00000124762	18328430	validated
mirecords	hsa-miR-106b-5p	VEGFA	7422	ENSG00000112715	18320040	validated
mirecords	hsa-miR-21-5p	SERPINB5	5268	ENSG00000206075	18270520	validated
mirecords	hsa-miR-212-3p	TJP1	7082	ENSG00000104067	18162065	validated
mirecords	hsa-miR-21-5p	CDKN1A	1026	ENSG00000124762	17991735	validated
mirecords	hsa-miR-21-5p	FAS	355	ENSG00000026103	17991735	validated
mirecords	hsa-miR-21-5p	FAM3C	10447	ENSG00000196937	17991735	validated
mirecords	hsa-miR-21-5p	HIPK3	10114	ENSG00000110422	17991735	validated
mirecords	hsa-miR-21-5p	PRRG4	79056	ENSG00000135378	17991735	validated
mirecords	hsa-miR-21-5p	ACTA2	59	ENSG00000107796	17991735	validated
mirecords	hsa-miR-21-5p	BTG2	7832	ENSG00000159388	17991735	validated
mirecords	hsa-miR-21-5p	BMPR2	659	ENSG00000204217	17991735	validated
mirecords	hsa-miR-21-5p	SESN1	27244	ENSG00000080546	17991735	validated
mirecords	hsa-miR-21-5p	IL6R	3570	ENSG00000160712	17991735	validated
mirecords	hsa-miR-21-5p	SOCS5	9655	ENSG00000171150	17991735	validated
mirecords	hsa-miR-21-5p	GLCCI1	113263	ENSG00000106415	17991735	validated
mirecords	hsa-miR-21-5p	APAF1	317	ENSG00000120868	17991735	validated
mirecords	hsa-miR-21-5p	SLC16A10	117247	ENSG00000112394	17991735	validated
mirecords	hsa-miR-21-5p	SGK3	23678	ENSG00000104205	17991735	validated
mirecords	hsa-miR-21-5p	RP2	6102	ENSG00000102218	17991735	validated
mirecords	hsa-miR-21-5p	CDK6	1021	ENSG00000105810	17991735	validated
mirecords	hsa-miR-21-5p	CFL2	1073	ENSG00000165410	17991735	validated
mirecords	hsa-miR-222-3p	CDKN1B	1027	ENSG00000111276	17721077	validated
mirecords	hsa-miR-222-3p	CDKN1C	1028	ENSG00000129757	18413744	validated
mirecords	hsa-miR-15b-5p	BCL2	596	ENSG00000171791	18449891	validated
mirecords	hsa-miR-21-5p	RECK	8434	ENSG00000122707	18794849	validated
mirecords	hsa-miR-222-3p	ESR1	2099	ENSG00000091831	18790736	validated
mirecords	hsa-miR-326	SMO	6608	ENSG00000128602	18756266	validated
mirecords	hsa-miR-181a-5p	TAF6L	10629	ENSG00000162227	18728182	validated
mirecords	hsa-miR-106b-5p	TAF6L	10629	ENSG00000162227	18728182	validated
mirecords	hsa-miR-21-5p	TIMP3	7078	ENSG00000100234	18591254	validated
mirecords	hsa-miR-15b-5p	CCNE1	898	ENSG00000105173	19135980	validated
mirecords	hsa-miR-106b-5p	ITCH	83737	ENSG00000078747	19096009	validated
mirecords	hsa-miR-192-5p	DHFR	1719	ENSG00000228716	19088023	validated
mirecords	hsa-miR-21-5p	MTAP	4507	ENSG00000099810	19013014	validated
mirecords	hsa-miR-21-5p	SOX5	6660	ENSG00000134532	19013014	validated
mirecords	hsa-miR-296-5p	HGS	9146	ENSG00000185359	18977327	validated
mirecords	hsa-miR-21-5p	JMY	133746	ENSG00000152409	18829576	validated
mirecords	hsa-miR-21-5p	TGFBR2	7048	ENSG00000163513	18829576	validated
mirecords	hsa-miR-21-5p	TGFBR3	7049	ENSG00000069702	11829576	validated
mirecords	hsa-miR-21-5p	HNRNPK	3190	ENSG00000165119	11829576	validated
mirecords	hsa-miR-21-5p	TP63	8626	ENSG00000073282	11829576	validated
mirecords	hsa-miR-21-5p	TOPORS	10210	ENSG00000197579	11829576	validated
mirecords	hsa-miR-21-5p	DAXX	1616	ENSG00000206279	18829576	validated
mirecords	hsa-miR-21-5p	TP53BP2	7159	ENSG00000143514	18829576	validated
mirecords	hsa-miR-21-5p	PPIF	10105	ENSG00000108179	11829576	validated
mirecords	hsa-miR-21-5p	PTEN	5728	ENSG00000171862	18850008	validated
mirecords	hsa-miR-106b-5p	RB1	5925	ENSG00000139687	19175831	validated
mirecords	hsa-miR-21-5p	TGFB1	7040	ENSG00000105329	19264808	validated
mirecords	hsa-miR-21-5p	RASA1	5921	ENSG00000145715	19264808	validated
mirecords	hsa-miR-21-5p	RASGRP1	10125	ENSG00000172575	19264808	validated
mirecords	hsa-miR-181a-5p	CDKN1B	1027	ENSG00000111276	19273599	validated
mirecords	hsa-miR-296-5p	CCND1	595	ENSG00000110092	20485139	validated
mirecords	hsa-miR-296-5p	CDKN1B	1027	ENSG00000111276	20485139	validated
mirecords	hsa-miR-296-5p	ABCB1	5243	ENSG00000085563	20485139	validated
mirecords	hsa-miR-296-5p	BCL2	596	ENSG00000171791	20485139	validated
mirecords	hsa-miR-296-5p	BAX	581	ENSG00000087088	20485139	validated
mirecords	hsa-miR-18a-5p	CTGF	1490	ENSG00000118523	20305691	validated
mirecords	hsa-miR-18a-5p	THBS1	7057	ENSG00000137801	20299512	validated
mirecords	hsa-miR-18a-5p	BIM			20299512	validated
mirecords	hsa-miR-18a-5p	ESR1	2099	ENSG00000091831	20080637	validated
mirecords	hsa-miR-222-3p	FOS	2353	ENSG00000170345	20299489	validated
mirecords	hsa-miR-181a-5p	BCL2	596	ENSG00000171791	20204284	validated

mirecords	hsa-miR-21-5p	E2F1	1869	ENSG00000101412	19906824	validated
mirecords	hsa-miR-15b-5p	RECK	8434	ENSG00000122707	20154725	validated
mirecords	hsa-miR-222-3p	PPP2R2A	5520	ENSG00000221914	20103675	validated
mirecords	hsa-miR-15b-5p	MKK4			19861690	validated
mirecords	hsa-miR-21-5p	CDC25A	993	ENSG00000164045	19826040	validated
mirecords	hsa-miR-181a-5p	PLAG1	5324	ENSG00000181690	19692702	validated
mirecords	hsa-miR-181a-5p	ESR1	2099	ENSG00000091831	19684618	validated
mirecords	hsa-miR-181a-5p	CDX2	1045	ENSG00000165556	19585654	validated
mirecords	hsa-miR-181a-5p	GATA6	2627	ENSG00000141448	19585654	validated
mirecords	hsa-miR-181a-5p	NLK	51701	ENSG00000087095	19585654	validated
mirecords	hsa-miR-21-5p	LRRFIP1	9208	ENSG00000124831	19559015	validated
mirecords	hsa-miR-222-3p	MMP1	4312	ENSG00000196611	19487542	validated
mirecords	hsa-miR-222-3p	SOD2	6648	ENSG00000112096	19487542	validated
mirecords	hsa-miR-222-3p	BIM			19438724	validated
mirecords	hsa-miR-21-5p	JAG1	182	ENSG00000101384	19398721	validated
mirecords	hsa-miR-21-5p	MARCKS	4082	ENSG00000277443	19302977	validated
mirecords	hsa-miR-21-5p	PELI1	57162	ENSG00000197329	20167875	validated
mirecords	hsa-miR-192-5p	WNK1	65125	ENSG00000060237	20813867	validated
mirecords	hsa-miR-222-3p	BBC3	27113	ENSG00000105327	20813046	validated
mirecords	hsa-miR-18a-5p	HSF2	3298	ENSG00000025156	20724452	validated
mirecords	hsa-miR-222-3p	STAT5A	6776	ENSG00000126561	20489169	validated
mirecords	hsa-miR-222-3p	PTEN	5728	ENSG00000171862	20618998	validated
mirecords	hsa-miR-21-5p	PPARA	5465	ENSG00000186951	20693317	validated
mirecords	hsa-miR-192-5p	RB1	5925	ENSG00000139687	21511813	validated
mirecords	hsa-miR-222-3p	p27			21226887	validated
mirecords	hsa-miR-21-5p	STAT3	6774	ENSG00000168610	21381024	validated
mirecords	hsa-miR-296-5p	PUMA			21633093	validated
mirecords	hsa-miR-15b-5p	BMI1	648	ENSG00000168283	21725369	validated
mirecords	hsa-miR-296-5p	IKBKE	9641	ENSG00000263528	22114321	validated
mirecords	hsa-miR-222-3p	TIMP3	7078	ENSG00000100234	22009755	validated
mirecords	hsa-miR-21-5p	NT-3			22019057	validated
mirtarbase	hsa-miR-106b-5p	ABCA1	19	ENSG00000165029	22473208	validated
mirtarbase	hsa-miR-15b-5p	ABL2	27	ENSG00000143322	23592263	validated
mirtarbase	hsa-miR-18a-5p	ABR	29	ENSG00000278741	23622248	validated
mirtarbase	hsa-miR-222-3p	ACACA	31	ENSG00000275176	23622248	validated
mirtarbase	hsa-miR-192-5p	ACADSB	36	ENSG00000196177	19074876	validated
mirtarbase	hsa-miR-106b-5p	ACADSB	36	ENSG00000196177	22473208	validated
mirtarbase	hsa-miR-21-5p	ACAT1	38	ENSG00000075239	18591254	validated
mirtarbase	hsa-miR-212-3p	ACHE	43	ENSG00000087085	23974008	validated
mirtarbase	hsa-miR-106b-5p	ACOX1	51	ENSG00000161533	19536157	validated
mirtarbase	hsa-miR-15b-5p	ACOX1	51	ENSG00000161533	22473208	validated
mirtarbase	hsa-miR-326	ACTB	60	ENSG00000075624	23622248	validated
mirtarbase	hsa-miR-222-3p	ACTB	60	ENSG00000075624	23622248	validated
mirtarbase	hsa-miR-326	ACTG1	71	ENSG00000184009	23622248	validated
mirtarbase	hsa-miR-222-3p	ACTG1	71	ENSG00000184009	23622248	validated
mirtarbase	hsa-miR-18a-5p	ACTL6A	86	ENSG00000136518	20371350	validated
mirtarbase	hsa-miR-301a-3p	ACVR1	90	ENSG00000115170	21572407	validated
mirtarbase	hsa-miR-106b-5p	ACVR1B	91	ENSG00000135503	23446348	validated
mirtarbase	hsa-miR-192-5p	ACVR2A	92	ENSG00000121989	19074876	validated
mirtarbase	hsa-miR-15b-5p	ACVR2A	92	ENSG00000121989	20371350	validated
mirtarbase	hsa-miR-192-5p	ACVR2B	93	ENSG00000114739	22431721	validated
mirtarbase	hsa-miR-296-5p	ACVRL1	94	ENSG00000139567	19536157	validated
mirtarbase	hsa-miR-181a-5p	ACYP1	97	ENSG00000119640	22473208	validated
mirtarbase	hsa-miR-106b-5p	ADAR	103	ENSG00000160710	23592263	validated
mirtarbase	hsa-miR-301a-3p	ADARB2	105	ENSG00000185736	22012620	validated
mirtarbase	hsa-miR-212-3p	ADCY1	107	ENSG00000164742	25766675	validated
mirtarbase	hsa-miR-181a-5p	ADCY9	115	ENSG00000162104	26701625	validated
mirtarbase	hsa-miR-106b-5p	ADD1	118	ENSG00000087274	22473208	validated
mirtarbase	hsa-miR-106b-5p	ADH5	128	ENSG00000197894	23622248	validated
mirtarbase	hsa-miR-15b-5p	ADORA3	140	ENSG00000282608	19536157	validated
mirtarbase	hsa-miR-21-5p	PARP1	142	ENSG00000143799	18591254	validated
mirtarbase	hsa-miR-301a-3p	PARP1	142	ENSG00000143799	23446348	validated
mirtarbase	hsa-miR-15b-5p	ADRA2B	151	ENSG00000274286	23824327	validated
mirtarbase	hsa-miR-181a-5p	GRK2	156	ENSG00000173020	22473208	validated
mirtarbase	hsa-miR-192-5p	GRK3	157	ENSG00000100077	19074876	validated
mirtarbase	hsa-miR-106b-5p	GRK3	157	ENSG00000100077	23824327	validated
mirtarbase	hsa-miR-505-3p	GRK3	157	ENSG00000100077	23824327	validated
mirtarbase	hsa-miR-222-3p	AP1B1	162	ENSG00000100280	23622248	validated
mirtarbase	hsa-miR-106b-5p	AP2B1	163	ENSG00000006125	23622248	validated
mirtarbase	hsa-miR-15b-5p	AP2B1	163	ENSG00000006125	26701625	validated
mirtarbase	hsa-miR-106b-5p	AP1G1	164	ENSG00000166747	23622248	validated

mirtarbase	hsa-miR-582-5p	AP1G1	164	ENSG00000166747	23592263	validated
mirtarbase	hsa-miR-144-3p	AP1G1	164	ENSG00000166747	23592263	validated
mirtarbase	hsa-miR-106b-5p	AFP	174	ENSG00000081051	17242205	validated
mirtarbase	hsa-miR-181a-5p	ACAN	176	ENSG00000157766	22942087	validated
mirtarbase	hsa-miR-192-5p	AGL	178	ENSG00000162688	19074876	validated
mirtarbase	hsa-miR-181a-5p	AHR	196	ENSG00000106546	24329418	validated
mirtarbase	hsa-miR-21-5p	CRYBG1	202	ENSG00000112297	18591254	validated
mirtarbase	hsa-miR-326	AKT1	207	ENSG00000142208	21572407	validated
mirtarbase	hsa-miR-192-5p	AKT1	207	ENSG00000142208	26351877	validated
mirtarbase	hsa-miR-21-5p	AKT2	208	ENSG00000105221	20048743	validated
mirtarbase	hsa-miR-192-5p	ALCAM	214	ENSG00000170017	16822819	validated
mirtarbase	hsa-miR-181a-5p	ALDH1A1	216	ENSG00000165092	26693182	validated
mirtarbase	hsa-miR-15b-5p	ALDH3B1	221	ENSG00000006534	23592263	validated
mirtarbase	hsa-miR-192-5p	ALDH9A1	223	ENSG00000143149	19074876	validated
mirtarbase	hsa-miR-106b-5p	ALDH9A1	223	ENSG00000143149	22473208	validated
mirtarbase	hsa-miR-582-3p	ALDH9A1	223	ENSG00000143149	23592263	validated
mirtarbase	hsa-miR-181a-5p	ALDH9A1	223	ENSG00000143149	22473208	validated
mirtarbase	hsa-miR-301a-3p	ABCD2	225	ENSG00000173208	20371350	validated
mirtarbase	hsa-miR-212-3p	AMD1	262	ENSG00000123505	23446348	validated
mirtarbase	hsa-miR-582-5p	AMD1	262	ENSG00000123505	23313552	validated
mirtarbase	hsa-miR-582-3p	AMD1	262	ENSG00000123505	27292025	validated
mirtarbase	hsa-miR-505-3p	AMFR	267	ENSG00000159461	26032086	validated
mirtarbase	hsa-miR-192-5p	ANG	283	ENSG00000214274	19074876	validated
mirtarbase	hsa-miR-106b-5p	SLC25A5	292	ENSG00000005022	23622248	validated
mirtarbase	hsa-miR-326	ANXA6	309	ENSG00000197043	28735896	validated
mirtarbase	hsa-miR-212-3p	APBA1	320	ENSG00000107282	19536157	validated
mirtarbase	hsa-miR-106b-5p	APC	324	ENSG00000134982	23087084	validated
mirtarbase	hsa-miR-192-5p	APC	324	ENSG00000134982	19074876	validated
mirtarbase	hsa-miR-21-5p	APC	324	ENSG00000134982	18591254	validated
mirtarbase	hsa-miR-181a-5p	XIAP	331	ENSG00000101966	22610076	validated
mirtarbase	hsa-miR-192-5p	XIAP	331	ENSG00000101966	19074876	validated
mirtarbase	hsa-miR-106b-5p	XIAP	331	ENSG00000101966	21572407	validated
mirtarbase	hsa-miR-505-3p	XIAP	331	ENSG00000101966	22012620	validated
mirtarbase	hsa-miR-301a-3p	XIAP	331	ENSG00000101966	23824327	validated
mirtarbase	hsa-miR-106b-5p	APLP2	334	ENSG00000084234	23622248	validated
mirtarbase	hsa-miR-106b-5p	APOH	350	ENSG00000091583	23824327	validated
mirtarbase	hsa-miR-106b-5p	APP	351	ENSG00000142192	19110058	validated
mirtarbase	hsa-miR-222-3p	APP	351	ENSG00000142192	23622248	validated
mirtarbase	hsa-miR-15b-5p	APP	351	ENSG00000142192	21572407	validated
mirtarbase	hsa-miR-144-3p	APP	351	ENSG00000142192	21572407	validated
mirtarbase	hsa-miR-15b-5p	APRT	353	ENSG00000198931	23622248	validated
mirtarbase	hsa-miR-18a-5p	FAS	355	ENSG00000026103	24955218	validated
mirtarbase	hsa-miR-106b-5p	FAS	355	ENSG00000026103	24955218	validated
mirtarbase	hsa-miR-21-5p	FASLG	356	ENSG00000117560	21820586	validated
mirtarbase	hsa-miR-326	AR	367	ENSG00000169083	22012620	validated
mirtarbase	hsa-miR-326	ABCC6	368	ENSG00000091262	23592263	validated
mirtarbase	hsa-miR-15b-5p	ABCC6	368	ENSG00000091262	23592263	validated
mirtarbase	hsa-miR-15b-5p	ARCN1	372	ENSG00000095139	22473208	validated
mirtarbase	hsa-miR-106b-5p	ARCN1	372	ENSG00000095139	22473208	validated
mirtarbase	hsa-miR-192-5p	TRIM23	373	ENSG00000113595	19074876	validated
mirtarbase	hsa-miR-582-3p	ARF3	377	ENSG00000134287	23592263	validated
mirtarbase	hsa-miR-181a-5p	ARF6	382	ENSG00000165527	17612493	validated
mirtarbase	hsa-miR-18a-5p	ARF6	382	ENSG00000165527	20371350	validated
mirtarbase	hsa-miR-21-5p	RHOB	388	ENSG00000143878	20460403	validated
mirtarbase	hsa-miR-106b-5p	RHOC	389	ENSG00000155366	21572407	validated
mirtarbase	hsa-miR-181a-5p	RHOG	391	ENSG00000177105	22473208	validated
mirtarbase	hsa-miR-106b-5p	ARHGAP1	392	ENSG00000175220	22473208	validated
mirtarbase	hsa-miR-301a-3p	ARHGAP1	392	ENSG00000175220	23592263	validated
mirtarbase	hsa-miR-15b-5p	ARHGDIA	396	ENSG00000141522	22473208	validated
mirtarbase	hsa-miR-505-3p	RHOH	399	ENSG00000168421	19536157	validated
mirtarbase	hsa-miR-106b-5p	ARL1	400	ENSG00000120805	22473208	validated
mirtarbase	hsa-miR-181a-5p	PHOX2A	401	ENSG00000165462	17612493	validated
mirtarbase	hsa-miR-181a-5p	ARRB2	409	ENSG00000141480	22473208	validated
mirtarbase	hsa-miR-301a-3p	ARSA	410	ENSG00000100299	27292025	validated
mirtarbase	hsa-miR-212-3p	ART4	420	ENSG00000111339	22012620	validated
mirtarbase	hsa-miR-18a-5p	ARVCF	421	ENSG00000099889	23622248	validated
mirtarbase	hsa-miR-15b-5p	ASGR2	433	ENSG00000161944	23446348	validated
mirtarbase	hsa-miR-192-5p	ASPH	444	ENSG00000198363	19074876	validated
mirtarbase	hsa-miR-192-5p	ATF1	466	ENSG00000123268	19074876	validated
mirtarbase	hsa-miR-181a-5p	ATM	472	ENSG00000149311	21274007	validated
mirtarbase	hsa-miR-18a-5p	ATM	472	ENSG00000149311	21980462	validated

mirtarbase	hsa-miR-301a-3p	RERE	473	ENSG00000142599	23622248	validated
mirtarbase	hsa-miR-18a-5p	ATP1A1	476	ENSG00000163399	23622248	validated
mirtarbase	hsa-miR-192-5p	ATP1B1	481	ENSG00000143153	23221637	validated
mirtarbase	hsa-miR-106b-5p	ATP1B3	483	ENSG00000069849	23313552	validated
mirtarbase	hsa-miR-15b-5p	ATP2A2	488	ENSG00000174437	23622248	validated
mirtarbase	hsa-miR-106b-5p	ATP2B1	490	ENSG00000070961	22473208	validated
mirtarbase	hsa-miR-181a-5p	ATP2B1	490	ENSG00000070961	22473208	validated
mirtarbase	hsa-miR-21-5p	ATP2B4	493	ENSG00000058668	18591254	validated
mirtarbase	hsa-miR-15b-5p	ATP5MC3	518	ENSG00000154518	21572407	validated
mirtarbase	hsa-miR-301a-3p	ATP6V1B2	526	ENSG00000147416	23592263	validated
mirtarbase	hsa-miR-301a-3p	ATP6V1C1	528	ENSG00000155097	22012620	validated
mirtarbase	hsa-miR-222-3p	ATP7B	540	ENSG00000123191	23622248	validated
mirtarbase	hsa-miR-21-5p	ATRX	546	ENSG00000085224	18591254	validated
mirtarbase	hsa-miR-106b-5p	B2M	567	ENSG00000166710	23592263	validated
mirtarbase	hsa-miR-181a-5p	NKX3-2	579	ENSG00000109705	17612493	validated
mirtarbase	hsa-miR-582-5p	NKX3-2	579	ENSG00000109705	23824327	validated
mirtarbase	hsa-miR-192-5p	BARD1	580	ENSG00000138376	16822819	validated
mirtarbase	hsa-miR-181a-5p	BAX	581	ENSG00000087088	26028030	validated
mirtarbase	hsa-miR-15b-5p	BAX	581	ENSG00000087088	27499071	validated
mirtarbase	hsa-miR-21-5p	BCAT1	586	ENSG00000060982	18591254	validated
mirtarbase	hsa-miR-15b-5p	CCND1	595	ENSG00000110092	19135980	validated
mirtarbase	hsa-miR-106b-5p	CCND1	595	ENSG00000110092	21283765	validated
mirtarbase	hsa-miR-326	CCND1	595	ENSG00000110092	23592263	validated
mirtarbase	hsa-miR-18a-5p	CCND1	595	ENSG00000110092	21572407	validated
mirtarbase	hsa-miR-21-5p	BCL2	596	ENSG00000171791	17072344	validated
mirtarbase	hsa-miR-192-5p	BCL2	596	ENSG00000171791	19074876	validated
mirtarbase	hsa-miR-18a-5p	BCL2	596	ENSG00000171791	24280866	validated
mirtarbase	hsa-miR-326	BCL2L1	598	ENSG00000171552	25875481	validated
mirtarbase	hsa-miR-106b-5p	BCL2L2	599	ENSG00000129473	23446348	validated
mirtarbase	hsa-miR-21-5p	BCL6	604	ENSG00000113916	23416424	validated
mirtarbase	hsa-miR-21-5p	BCL7A	605	ENSG00000110987	22473208	validated
mirtarbase	hsa-miR-15b-5p	BCL7A	605	ENSG00000110987	22473208	validated
mirtarbase	hsa-miR-18a-5p	BDH1	622	ENSG00000275544	23824327	validated
mirtarbase	hsa-miR-192-5p	BGLAP	632	ENSG00000242252	19074876	validated
mirtarbase	hsa-miR-192-5p	BICD1	636	ENSG00000151746	19074876	validated
mirtarbase	hsa-miR-222-3p	PRDM1	639	ENSG00000057657	26655851	validated
mirtarbase	hsa-miR-192-5p	BLM	641	ENSG00000197299	19074876	validated
mirtarbase	hsa-miR-192-5p	BMI1	648	ENSG00000168283	26717043	validated
mirtarbase	hsa-miR-21-5p	BMI1	648	ENSG00000168283	27644439	validated
mirtarbase	hsa-miR-106b-5p	BMP2	650	ENSG00000125845	23592263	validated
mirtarbase	hsa-miR-505-3p	BMP2	650	ENSG00000125845	23592263	validated
mirtarbase	hsa-miR-181a-5p	BMP3	651	ENSG00000152785	22942087	validated
mirtarbase	hsa-miR-301a-3p	BMP3	651	ENSG00000152785	21572407	validated
mirtarbase	hsa-miR-326	BMP7	655	ENSG00000101144	23824327	validated
mirtarbase	hsa-miR-106b-5p	BMP8B	656	ENSG00000116985	23446348	validated
mirtarbase	hsa-miR-192-5p	BMPR2	659	ENSG00000204217	19074876	validated
mirtarbase	hsa-miR-181a-5p	BMPR2	659	ENSG00000204217	22942087	validated
mirtarbase	hsa-miR-106b-5p	BMPR2	659	ENSG00000204217	22473208	validated
mirtarbase	hsa-miR-21-5p	BNIP2	663	ENSG00000140299	22473208	validated
mirtarbase	hsa-miR-106b-5p	BNIP2	663	ENSG00000140299	22473208	validated
mirtarbase	hsa-miR-192-5p	DST	667	ENSG00000151914	19074876	validated
mirtarbase	hsa-miR-181a-5p	BPGM	669	ENSG00000172331	17612493	validated
mirtarbase	hsa-miR-212-3p	BRCA1	672	ENSG00000012048	17875710	validated
mirtarbase	hsa-miR-192-5p	BRCA1	672	ENSG00000012048	19074876	validated
mirtarbase	hsa-miR-21-5p	BRCA1	672	ENSG00000012048	18591254	validated
mirtarbase	hsa-miR-181a-5p	BRCA1	672	ENSG00000012048	23622248	validated
mirtarbase	hsa-miR-192-5p	BRCA2	675	ENSG00000139618	19074876	validated
mirtarbase	hsa-miR-181a-5p	ZFP36L1	677	ENSG00000185650	22473208	validated
mirtarbase	hsa-miR-181a-5p	ZFP36L2	678	ENSG00000152518	23622248	validated
mirtarbase	hsa-miR-18a-5p	ZFP36L2	678	ENSG00000152518	23622248	validated
mirtarbase	hsa-miR-582-5p	ZFP36L2	678	ENSG00000152518	21572407	validated
mirtarbase	hsa-miR-144-3p	ZFP36L2	678	ENSG00000152518	21572407	validated
mirtarbase	hsa-miR-15b-5p	BSG	682	ENSG00000172270	22473208	validated
mirtarbase	hsa-miR-192-5p	BTC	685	ENSG00000174808	19074876	validated
mirtarbase	hsa-miR-21-5p	KLF9	687	ENSG00000119138	20371350	validated
mirtarbase	hsa-miR-21-5p	KLF5	688	ENSG00000102554	18591254	validated
mirtarbase	hsa-miR-301a-3p	BTG1	694	ENSG00000133639	23313552	validated
mirtarbase	hsa-miR-192-5p	BUB1B	701	ENSG00000156970	19074876	validated
mirtarbase	hsa-miR-192-5p	BYSL	705	ENSG00000112578	20371350	validated
mirtarbase	hsa-miR-301a-3p	C1QBP	708	ENSG00000108561	23622248	validated
mirtarbase	hsa-miR-326	C1R	715	ENSG00000159403	23824327	validated

mirtarbase	hsa-miR-181a-5p	C8A	731	ENSG00000157131	17612493	validated
mirtarbase	hsa-miR-296-5p	VPS51	738	ENSG00000149823	23708386	validated
mirtarbase	hsa-miR-192-5p	CA8	767	ENSG00000178538	19074876	validated
mirtarbase	hsa-miR-15b-5p	CA8	767	ENSG00000178538	21572407	validated
mirtarbase	hsa-miR-18a-5p	CA12	771	ENSG00000074410	20371350	validated
mirtarbase	hsa-miR-301a-3p	CAD	790	ENSG00000084774	23622248	validated
mirtarbase	hsa-miR-18a-5p	CAD	790	ENSG00000084774	23592263	validated
mirtarbase	hsa-miR-21-5p	CALD1	800	ENSG00000122786	18591254	validated
mirtarbase	hsa-miR-301a-3p	CALM2	805	ENSG00000143933	23446348	validated
mirtarbase	hsa-miR-15b-5p	CALU	813	ENSG00000128595	22473208	validated
mirtarbase	hsa-miR-192-5p	CAMK4	814	ENSG00000152495	19074876	validated
mirtarbase	hsa-miR-222-3p	CANX	821	ENSG00000127022	23622248	validated
mirtarbase	hsa-miR-18a-5p	CANX	821	ENSG00000127022	23622248	validated
mirtarbase	hsa-miR-15b-5p	CANX	821	ENSG00000127022	21572407	validated
mirtarbase	hsa-miR-505-3p	CANX	821	ENSG00000127022	23592263	validated
mirtarbase	hsa-miR-15b-5p	CAPZA2	830	ENSG00000198898	20371350	validated
mirtarbase	hsa-miR-106b-5p	CAPZA2	830	ENSG00000198898	23313552	validated
mirtarbase	hsa-miR-222-3p	CAST	831	ENSG00000153113	23622248	validated
mirtarbase	hsa-miR-582-5p	CAPZB	832	ENSG00000077549	27292025	validated
mirtarbase	hsa-miR-106b-5p	CASP2	835	ENSG00000106144	22473208	validated
mirtarbase	hsa-miR-582-5p	CASP3	836	ENSG00000164305	24805821	validated
mirtarbase	hsa-miR-106b-5p	CASP7	840	ENSG00000165806	17242205	validated
mirtarbase	hsa-miR-192-5p	CASP7	840	ENSG00000165806	19074876	validated
mirtarbase	hsa-miR-106b-5p	CASP8	841	ENSG00000064012	24166509	validated
mirtarbase	hsa-miR-296-5p	CASP8	841	ENSG00000064012	27927008	validated
mirtarbase	hsa-miR-21-5p	CASP8	841	ENSG00000064012	26080425	validated
mirtarbase	hsa-miR-582-5p	CASP9	842	ENSG00000132906	24805821	validated
mirtarbase	hsa-miR-192-5p	CAV1	857	ENSG00000105974	24623846	validated
mirtarbase	hsa-miR-106b-5p	CAV1	857	ENSG00000105974	20371350	validated
mirtarbase	hsa-miR-222-3p	RUNX2	860	ENSG00000124813	26809090	validated
mirtarbase	hsa-miR-18a-5p	RUNX1	861	ENSG00000159216	25452107	validated
mirtarbase	hsa-miR-181a-5p	RUNX1	861	ENSG00000159216	26580398	validated
mirtarbase	hsa-miR-106b-5p	RUNX1T1	862	ENSG00000079102	17242205	validated
mirtarbase	hsa-miR-15b-5p	RUNX1T1	862	ENSG00000079102	21572407	validated
mirtarbase	hsa-miR-15b-5p	CBFA2T3	863	ENSG00000129993	22473208	validated
mirtarbase	hsa-miR-301a-3p	RUNX3	864	ENSG00000020633	23338485	validated
mirtarbase	hsa-miR-106b-5p	RUNX3	864	ENSG00000020633	20371350	validated
mirtarbase	hsa-miR-222-3p	SERPINH1	871	ENSG00000149257	23824327	validated
mirtarbase	hsa-miR-21-5p	KRIT1	889	ENSG00000001631	22473208	validated
mirtarbase	hsa-miR-212-3p	CCNA2	890	ENSG00000145386	21329664	validated
mirtarbase	hsa-miR-301a-3p	CCNA2	890	ENSG00000145386	23592263	validated
mirtarbase	hsa-miR-212-3p	CCNB1	891	ENSG00000134057	21329664	validated
mirtarbase	hsa-miR-106b-5p	CCND2	894	ENSG00000118971	21283765	validated
mirtarbase	hsa-miR-301a-3p	CCND2	894	ENSG00000118971	23622248	validated
mirtarbase	hsa-miR-15b-5p	CCND2	894	ENSG00000118971	21572407	validated
mirtarbase	hsa-miR-505-3p	CCND2	894	ENSG00000118971	21572407	validated
mirtarbase	hsa-miR-15b-5p	CCND3	896	ENSG00000112576	26144250	validated
mirtarbase	hsa-miR-192-5p	CCNE1	898	ENSG00000105173	19074876	validated
mirtarbase	hsa-miR-582-5p	CCNF	899	ENSG00000162063	23592263	validated
mirtarbase	hsa-miR-181a-5p	CCNG1	900	ENSG00000113328	17612493	validated
mirtarbase	hsa-miR-21-5p	CCNG1	900	ENSG00000113328	18591254	validated
mirtarbase	hsa-miR-15b-5p	CCNT1	904	ENSG00000129315	20371350	validated
mirtarbase	hsa-miR-15b-5p	CCNT2	905	ENSG00000082258	22473208	validated
mirtarbase	hsa-miR-18a-5p	CCT6A	908	ENSG00000146731	23622248	validated
mirtarbase	hsa-miR-301a-3p	CCT6A	908	ENSG00000146731	21572407	validated
mirtarbase	hsa-miR-181a-5p	CD4	920	ENSG00000010610	23393335	validated
mirtarbase	hsa-miR-326	CD9	928	ENSG00000010278	19883630	validated
mirtarbase	hsa-miR-106b-5p	CD28	940	ENSG00000178562	23313552	validated
mirtarbase	hsa-miR-106b-5p	CD34	947	ENSG00000174059	17242205	validated
mirtarbase	hsa-miR-222-3p	SCARB2	950	ENSG00000138760	23622248	validated
mirtarbase	hsa-miR-15b-5p	ENTPD1	953	ENSG00000138185	22473208	validated
mirtarbase	hsa-miR-15b-5p	ENTPD6	955	ENSG00000197586	22473208	validated
mirtarbase	hsa-miR-192-5p	ENTPD3	956	ENSG00000168032	16822819	validated
mirtarbase	hsa-miR-15b-5p	CD44	960	ENSG00000026508	23622248	validated
mirtarbase	hsa-miR-21-5p	CD47	961	ENSG00000196776	18591254	validated
mirtarbase	hsa-miR-505-3p	CD47	961	ENSG00000196776	23622248	validated
mirtarbase	hsa-miR-106b-5p	CD47	961	ENSG00000196776	22473208	validated
mirtarbase	hsa-miR-192-5p	CD47	961	ENSG00000196776	26506238	validated
mirtarbase	hsa-miR-582-5p	CD81	975	ENSG00000110651	23592263	validated
mirtarbase	hsa-miR-15b-5p	CDK1	983	ENSG00000170312	23446348	validated
mirtarbase	hsa-miR-505-3p	CDC5L	988	ENSG00000096401	23622248	validated

mirtarbase	hsa-miR-192-5p	CDC20	991	ENSG00000117399	19074876	validated
mirtarbase	hsa-miR-18a-5p	CDC20	991	ENSG00000117399	23622248	validated
mirtarbase	hsa-miR-192-5p	CDC25A	993	ENSG00000164045	19074876	validated
mirtarbase	hsa-miR-15b-5p	CDC25A	993	ENSG00000164045	22473208	validated
mirtarbase	hsa-miR-222-3p	CDC27	996	ENSG00000004897	20371350	validated
mirtarbase	hsa-miR-15b-5p	CDC27	996	ENSG00000004897	22473208	validated
mirtarbase	hsa-miR-106b-5p	CDC42	998	ENSG00000070831	23622248	validated
mirtarbase	hsa-miR-181a-5p	CDH13	1012	ENSG00000140945	22942087	validated
mirtarbase	hsa-miR-15b-5p	CDK4	1019	ENSG00000135446	23622248	validated
mirtarbase	hsa-miR-301a-3p	CDK4	1019	ENSG00000135446	23824327	validated
mirtarbase	hsa-miR-15b-5p	CDK6	1021	ENSG00000105810	23592263	validated
mirtarbase	hsa-miR-222-3p	CDK6	1021	ENSG00000105810	20371350	validated
mirtarbase	hsa-miR-15b-5p	CDKN1A	1026	ENSG00000124762	22473208	validated
mirtarbase	hsa-miR-582-5p	CDKN1A	1026	ENSG00000124762	23592263	validated
mirtarbase	hsa-miR-181a-5p	CDKN1A	1026	ENSG00000124762	27534652	validated
mirtarbase	hsa-miR-212-3p	CDKN1A	1026	ENSG00000124762	26701625	validated
mirtarbase	hsa-miR-192-5p	CDKN1B	1027	ENSG00000111276	17627278	validated
mirtarbase	hsa-miR-192-5p	CDKN2A	1029	ENSG00000147889	19074876	validated
mirtarbase	hsa-miR-192-5p	CDKN2D	1032	ENSG00000129355	19074876	validated
mirtarbase	hsa-miR-192-5p	CDKN3	1033	ENSG00000100526	19074876	validated
mirtarbase	hsa-miR-296-5p	CDX1	1044	ENSG00000113722	23353818	validated
mirtarbase	hsa-miR-582-3p	CEACAM5	1048	ENSG00000105388	23824327	validated
mirtarbase	hsa-miR-181a-5p	CEBPA	1050	ENSG00000245848	27673564	validated
mirtarbase	hsa-miR-21-5p	CEBPB	1051	ENSG00000172216	25327529	validated
mirtarbase	hsa-miR-192-5p	CENPA	1058	ENSG00000115163	19074876	validated
mirtarbase	hsa-miR-192-5p	CENPE	1062	ENSG00000138778	19074876	validated
mirtarbase	hsa-miR-192-5p	CENPF	1063	ENSG00000117724	19074876	validated
mirtarbase	hsa-miR-192-5p	CFL2	1073	ENSG00000165410	19074876	validated
mirtarbase	hsa-miR-106b-5p	CFL2	1073	ENSG00000165410	22473208	validated
mirtarbase	hsa-miR-301a-3p	CFL2	1073	ENSG00000165410	21572407	validated
mirtarbase	hsa-miR-144-3p	CFTR	1080	ENSG00000001626	23226399	validated
mirtarbase	hsa-miR-192-5p	RCBTB2	1102	ENSG00000136161	19074876	validated
mirtarbase	hsa-miR-15b-5p	CHEK1	1111	ENSG00000149554	23622248	validated
mirtarbase	hsa-miR-505-3p	CHEK1	1111	ENSG00000149554	23313552	validated
mirtarbase	hsa-miR-21-5p	FOXN3	1112	ENSG00000053254	18591254	validated
mirtarbase	hsa-miR-192-5p	CHM	1121	ENSG00000188419	19074876	validated
mirtarbase	hsa-miR-192-5p	CHML	1122	ENSG00000203668	19074876	validated
mirtarbase	hsa-miR-144-3p	ERCC8	1161	ENSG00000049167	20371350	validated
mirtarbase	hsa-miR-15b-5p	AP2M1	1173	ENSG00000161203	23622248	validated
mirtarbase	hsa-miR-301a-3p	CLCN3	1182	ENSG00000109572	22012620	validated
mirtarbase	hsa-miR-21-5p	CLCN5	1184	ENSG00000171365	18591254	validated
mirtarbase	hsa-miR-15b-5p	CLCN6	1185	ENSG00000011021	23622248	validated
mirtarbase	hsa-miR-21-5p	CLU	1191	ENSG00000120885	24327270	validated
mirtarbase	hsa-miR-15b-5p	CLU	1191	ENSG00000120885	26701625	validated
mirtarbase	hsa-miR-326	CLU	1191	ENSG00000120885	26701625	validated
mirtarbase	hsa-miR-192-5p	CLIC1	1192	ENSG00000223639	16822819	validated
mirtarbase	hsa-miR-192-5p	CLK1	1195	ENSG00000013441	19074876	validated
mirtarbase	hsa-miR-301a-3p	TPP1	1200	ENSG00000166340	23592263	validated
mirtarbase	hsa-miR-106b-5p	CLTC	1213	ENSG00000141367	23622248	validated
mirtarbase	hsa-miR-21-5p	CCR1	1230	ENSG00000163823	21131358	validated
mirtarbase	hsa-miR-301a-3p	CCR6	1235	ENSG00000112486	23824327	validated
mirtarbase	hsa-miR-21-5p	CCR7	1236	ENSG00000126353	22473208	validated
mirtarbase	hsa-miR-21-5p	COL4A1	1282	ENSG00000187498	22573493	validated
mirtarbase	hsa-miR-505-3p	COL4A1	1282	ENSG00000187498	23313552	validated
mirtarbase	hsa-miR-21-5p	COL5A2	1290	ENSG00000204262	18591254	validated
mirtarbase	hsa-miR-222-3p	COL5A2	1290	ENSG00000204262	23622248	validated
mirtarbase	hsa-miR-181a-5p	COL16A1	1307	ENSG00000084636	23238588	validated
mirtarbase	hsa-miR-181a-5p	KLF6	1316	ENSG00000067082	22581522	validated
mirtarbase	hsa-miR-106b-5p	KLF6	1316	ENSG00000067082	23592263	validated
mirtarbase	hsa-miR-301a-3p	KLF6	1316	ENSG00000067082	23592263	validated
mirtarbase	hsa-miR-21-5p	SLC31A1	1317	ENSG00000136868	18591254	validated
mirtarbase	hsa-miR-301a-3p	SLC31A1	1317	ENSG00000136868	23824327	validated
mirtarbase	hsa-miR-144-3p	MAP3K8	1326	ENSG00000107968	27717821	validated
mirtarbase	hsa-miR-106b-5p	COX6B1	1340	ENSG00000126267	20371350	validated
mirtarbase	hsa-miR-18a-5p	COX7B	1349	ENSG00000131174	23622248	validated
mirtarbase	hsa-miR-301a-3p	COX10	1352	ENSG00000006695	23592263	validated
mirtarbase	hsa-miR-144-3p	COX10	1352	ENSG00000006695	23592263	validated
mirtarbase	hsa-miR-15b-5p	CPOX	1371	ENSG00000080819	23622248	validated
mirtarbase	hsa-miR-106b-5p	CPOX	1371	ENSG00000080819	22473208	validated
mirtarbase	hsa-miR-181a-5p	CPOX	1371	ENSG00000080819	22473208	validated
mirtarbase	hsa-miR-144-3p	CPS1	1373	ENSG00000021826	23592263	validated

mirtarbase	hsa-miR-106b-5p	CPS1	1373	ENSG00000021826	23313552	validated
mirtarbase	hsa-miR-106b-5p	CPT1A	1374	ENSG00000110090	23313552	validated
mirtarbase	hsa-miR-106b-5p	CREB1	1385	ENSG00000118260	23446348	validated
mirtarbase	hsa-miR-582-5p	CREB1	1385	ENSG00000118260	26978739	validated
mirtarbase	hsa-miR-21-5p	ATF2	1386	ENSG00000115966	18591254	validated
mirtarbase	hsa-miR-192-5p	CREBL2	1389	ENSG00000111269	19074876	validated
mirtarbase	hsa-miR-18a-5p	CREBL2	1389	ENSG00000111269	20371350	validated
mirtarbase	hsa-miR-15b-5p	CREBL2	1389	ENSG00000111269	21572407	validated
mirtarbase	hsa-miR-192-5p	CRK	1398	ENSG00000167193	19074876	validated
mirtarbase	hsa-miR-15b-5p	CRK	1398	ENSG00000167193	23622248	validated
mirtarbase	hsa-miR-106b-5p	CRK	1398	ENSG00000167193	22473208	validated
mirtarbase	hsa-miR-15b-5p	CRKL	1399	ENSG00000099942	21572407	validated
mirtarbase	hsa-miR-21-5p	HAPLN1	1404	ENSG00000145681	18591254	validated
mirtarbase	hsa-miR-106b-5p	CRY2	1408	ENSG00000121671	22473208	validated
mirtarbase	hsa-miR-505-3p	CSE1L	1434	ENSG00000124207	23824327	validated
mirtarbase	hsa-miR-505-3p	CSF1	1435	ENSG00000184371	23622248	validated
mirtarbase	hsa-miR-21-5p	CSNK1A1	1452	ENSG00000113712	18591254	validated
mirtarbase	hsa-miR-106b-5p	CSNK1A1	1452	ENSG00000113712	23592263	validated
mirtarbase	hsa-miR-181a-5p	CSNK1A1	1452	ENSG00000113712	21572407	validated
mirtarbase	hsa-miR-505-3p	CSNK1A1	1452	ENSG00000113712	21572407	validated
mirtarbase	hsa-miR-15b-5p	CSNK1E	1454	ENSG00000213923	23592263	validated
mirtarbase	hsa-miR-301a-3p	CSNK2A1	1457	ENSG00000101266	26701625	validated
mirtarbase	hsa-miR-222-3p	SLC25A10	1468	ENSG00000183048	23622248	validated
mirtarbase	hsa-miR-181a-5p	CST5	1473	ENSG00000170367	17612493	validated
mirtarbase	hsa-miR-18a-5p	CCN2	1490	ENSG00000118523	16331254	validated
mirtarbase	hsa-miR-192-5p	CTH	1491	ENSG00000116761	19074876	validated
mirtarbase	hsa-miR-181a-5p	CTNNB1	1499	ENSG00000168036	28086904	validated
mirtarbase	hsa-miR-106b-5p	CTSS	1520	ENSG00000163131	22473208	validated
mirtarbase	hsa-miR-505-3p	CTSZ	1522	ENSG00000101160	23622248	validated
mirtarbase	hsa-miR-106b-5p	CYB5A	1528	ENSG00000166347	23824327	validated
mirtarbase	hsa-miR-15b-5p	CYBA	1535	ENSG00000051523	23622248	validated
mirtarbase	hsa-miR-106b-5p	CYBB	1536	ENSG00000165168	28235791	validated
mirtarbase	hsa-miR-106b-5p	CYLD	1540	ENSG00000083799	22473208	validated
mirtarbase	hsa-miR-15b-5p	CYLD	1540	ENSG00000083799	22473208	validated
mirtarbase	hsa-miR-192-5p	CYP24A1	1591	ENSG00000019186	19074876	validated
mirtarbase	hsa-miR-15b-5p	CYP51A1	1595	ENSG00000001630	23622248	validated
mirtarbase	hsa-miR-144-3p	DAB2	1601	ENSG00000153071	23592263	validated
mirtarbase	hsa-miR-106b-5p	DAB2	1601	ENSG00000153071	26769181	validated
mirtarbase	hsa-miR-301a-3p	DAD1	1603	ENSG00000129562	21572407	validated
mirtarbase	hsa-miR-222-3p	DAG1	1605	ENSG00000173402	23622248	validated
mirtarbase	hsa-miR-301a-3p	DAPK1	1612	ENSG00000196730	21572407	validated
mirtarbase	hsa-miR-222-3p	DAXX	1616	ENSG00000204209	23622248	validated
mirtarbase	hsa-miR-222-3p	DBN1	1627	ENSG00000113758	23622248	validated
mirtarbase	hsa-miR-192-5p	DBT	1629	ENSG00000137992	19074876	validated
mirtarbase	hsa-miR-192-5p	DDOST	1650	ENSG00000244038	16822819	validated
mirtarbase	hsa-miR-192-5p	DDX3X	1654	ENSG00000215301	16822819	validated
mirtarbase	hsa-miR-181a-5p	DDX3X	1654	ENSG00000215301	20371350	validated
mirtarbase	hsa-miR-21-5p	DDX3X	1654	ENSG00000215301	18591254	validated
mirtarbase	hsa-miR-15b-5p	DDX3X	1654	ENSG00000215301	24398324	validated
mirtarbase	hsa-miR-505-3p	DDX3X	1654	ENSG00000215301	21572407	validated
mirtarbase	hsa-miR-15b-5p	DDX5	1655	ENSG00000108654	23622248	validated
mirtarbase	hsa-miR-18a-5p	DDX5	1655	ENSG00000108654	23622248	validated
mirtarbase	hsa-miR-106b-5p	DDX5	1655	ENSG00000108654	22473208	validated
mirtarbase	hsa-miR-505-3p	DDX6	1656	ENSG00000110367	23622248	validated
mirtarbase	hsa-miR-582-5p	DDX6	1656	ENSG00000110367	23592263	validated
mirtarbase	hsa-miR-222-3p	DDX6	1656	ENSG00000110367	23592263	validated
mirtarbase	hsa-miR-192-5p	DDX6	1656	ENSG00000110367	22012620	validated
mirtarbase	hsa-miR-301a-3p	DDX6	1656	ENSG00000110367	26701625	validated
mirtarbase	hsa-miR-505-3p	DHX9	1660	ENSG00000135829	23622248	validated
mirtarbase	hsa-miR-15b-5p	DECR1	1666	ENSG00000104325	21572407	validated
mirtarbase	hsa-miR-192-5p	COCH	1690	ENSG00000100473	19074876	validated
mirtarbase	hsa-miR-192-5p	DHCR24	1718	ENSG00000116133	19074876	validated
mirtarbase	hsa-miR-15b-5p	DHFR	1719	ENSG00000228716	23622248	validated
mirtarbase	hsa-miR-106b-5p	DHODH	1723	ENSG00000102967	23824327	validated
mirtarbase	hsa-miR-18a-5p	DIAPH1	1729	ENSG00000131504	23622248	validated
mirtarbase	hsa-miR-15b-5p	DIAPH1	1729	ENSG00000131504	26701625	validated
mirtarbase	hsa-miR-21-5p	DLG1	1739	ENSG00000075711	18591254	validated
mirtarbase	hsa-miR-21-5p	DMD	1756	ENSG00000198947	18591254	validated
mirtarbase	hsa-miR-15b-5p	DMPK	1760	ENSG00000104936	23446348	validated
mirtarbase	hsa-miR-222-3p	DMWD	1762	ENSG00000185800	23622248	validated
mirtarbase	hsa-miR-21-5p	DYNC1LI2	1783	ENSG00000135720	18591254	validated

mirtarbase	hsa-miR-106b-5p	DYNC1LI2	1783	ENSG00000135720	22473208	validated
mirtarbase	hsa-miR-301a-3p	DYNC1LI2	1783	ENSG00000135720	21572407	validated
mirtarbase	hsa-miR-181a-5p	DYNC1LI2	1783	ENSG00000135720	22473208	validated
mirtarbase	hsa-miR-582-5p	DYNC1LI2	1783	ENSG00000135720	27292025	validated
mirtarbase	hsa-miR-18a-5p	DNMT1	1786	ENSG00000130816	23306545	validated
mirtarbase	hsa-miR-106b-5p	DPYSL2	1808	ENSG00000092964	22012620	validated
mirtarbase	hsa-miR-301a-3p	DPYSL2	1808	ENSG00000092964	22012620	validated
mirtarbase	hsa-miR-582-5p	DSC1	1823	ENSG00000134765	27292025	validated
mirtarbase	hsa-miR-15b-5p	DSG2	1829	ENSG00000046604	23622248	validated
mirtarbase	hsa-miR-18a-5p	TSC22D3	1831	ENSG00000157514	19131573	validated
mirtarbase	hsa-miR-505-3p	TSC22D3	1831	ENSG00000157514	23446348	validated
mirtarbase	hsa-miR-106b-5p	DSPP	1834	ENSG00000152591	20371350	validated
mirtarbase	hsa-miR-192-5p	SLC26A2	1836	ENSG00000155850	19074876	validated
mirtarbase	hsa-miR-21-5p	SLC26A2	1836	ENSG00000155850	18591254	validated
mirtarbase	hsa-miR-192-5p	HBEGF	1839	ENSG00000113070	19074876	validated
mirtarbase	hsa-miR-106b-5p	DUSP2	1844	ENSG00000158050	23592263	validated
mirtarbase	hsa-miR-181a-5p	DUSP5	1847	ENSG00000138166	17382377	validated
mirtarbase	hsa-miR-181a-5p	DUSP6	1848	ENSG00000139318	17382377	validated
mirtarbase	hsa-miR-21-5p	DUSP8	1850	ENSG00000273793	18591254	validated
mirtarbase	hsa-miR-192-5p	DYRK1A	1859	ENSG00000157540	19074876	validated
mirtarbase	hsa-miR-21-5p	E2F2	1870	ENSG00000007968	19528081	validated
mirtarbase	hsa-miR-106b-5p	E2F2	1870	ENSG00000007968	22473208	validated
mirtarbase	hsa-miR-106b-5p	E2F3	1871	ENSG00000112242	21283765	validated
mirtarbase	hsa-miR-15b-5p	E2F3	1871	ENSG00000112242	22473208	validated
mirtarbase	hsa-miR-21-5p	E2F3	1871	ENSG00000112242	21572407	validated
mirtarbase	hsa-miR-192-5p	E2F5	1875	ENSG00000133740	16822819	validated
mirtarbase	hsa-miR-106b-5p	E2F5	1875	ENSG00000133740	23803041	validated
mirtarbase	hsa-miR-181a-5p	E2F5	1875	ENSG00000133740	24529171	validated
mirtarbase	hsa-miR-106b-5p	GPR183	1880	ENSG00000169508	26701625	validated
mirtarbase	hsa-miR-15b-5p	ECHS1	1892	ENSG00000127884	23622248	validated
mirtarbase	hsa-miR-192-5p	ECT2	1894	ENSG00000114346	19074876	validated
mirtarbase	hsa-miR-301a-3p	EDN1	1906	ENSG00000078401	23592263	validated
mirtarbase	hsa-miR-192-5p	PHC2	1912	ENSG00000134686	19074876	validated
mirtarbase	hsa-miR-505-3p	EEF1A1	1915	ENSG00000156508	23622248	validated
mirtarbase	hsa-miR-106b-5p	EEF1A1	1915	ENSG00000156508	23622248	validated
mirtarbase	hsa-miR-15b-5p	EEF1A1	1915	ENSG00000156508	23622248	validated
mirtarbase	hsa-miR-18a-5p	EEF1A1	1915	ENSG00000156508	23622248	validated
mirtarbase	hsa-miR-192-5p	EEF1A2	1917	ENSG00000101210	19074876	validated
mirtarbase	hsa-miR-222-3p	EEF2	1938	ENSG00000167658	23622248	validated
mirtarbase	hsa-miR-18a-5p	EEF2	1938	ENSG00000167658	23622248	validated
mirtarbase	hsa-miR-192-5p	EFNB2	1948	ENSG00000125266	19074876	validated
mirtarbase	hsa-miR-582-5p	EFNB2	1948	ENSG00000125266	25176332	validated
mirtarbase	hsa-miR-15b-5p	EFNB2	1948	ENSG00000125266	22012620	validated
mirtarbase	hsa-miR-21-5p	MEGF9	1955	ENSG00000106780	18591254	validated
mirtarbase	hsa-miR-222-3p	MEGF9	1955	ENSG00000106780	22815788	validated
mirtarbase	hsa-miR-21-5p	EGFR	1956	ENSG00000146648	20864407	validated
mirtarbase	hsa-miR-192-5p	EGR1	1958	ENSG00000120738	16822819	validated
mirtarbase	hsa-miR-181a-5p	EGR1	1958	ENSG00000120738	28077323	validated
mirtarbase	hsa-miR-21-5p	EIF1AX	1964	ENSG00000173674	23446348	validated
mirtarbase	hsa-miR-505-3p	EIF1AX	1964	ENSG00000173674	20371350	validated
mirtarbase	hsa-miR-15b-5p	EIF1AX	1964	ENSG00000173674	23592263	validated
mirtarbase	hsa-miR-21-5p	EIF2S1	1965	ENSG00000134001	19253296	validated
mirtarbase	hsa-miR-192-5p	EIF2S1	1965	ENSG00000134001	20371350	validated
mirtarbase	hsa-miR-222-3p	EIF2S1	1965	ENSG00000134001	23622248	validated
mirtarbase	hsa-miR-106b-5p	EIF2S1	1965	ENSG00000134001	21572407	validated
mirtarbase	hsa-miR-212-3p	EIF2S3	1968	ENSG00000130741	20371350	validated
mirtarbase	hsa-miR-144-3p	EIF2S3	1968	ENSG00000130741	20371350	validated
mirtarbase	hsa-miR-222-3p	EIF2S3	1968	ENSG00000130741	26701625	validated
mirtarbase	hsa-miR-15b-5p	EIF4A1	1973	ENSG00000161960	16609010	validated
mirtarbase	hsa-miR-21-5p	EIF4A2	1974	ENSG00000156976	21219636	validated
mirtarbase	hsa-miR-106b-5p	EIF4A2	1974	ENSG00000156976	20371350	validated
mirtarbase	hsa-miR-21-5p	EIF4EBP2	1979	ENSG00000148730	18591254	validated
mirtarbase	hsa-miR-18a-5p	EIF4EBP2	1979	ENSG00000148730	23622248	validated
mirtarbase	hsa-miR-582-3p	EIF4EBP2	1979	ENSG00000148730	27292025	validated
mirtarbase	hsa-miR-106b-5p	EIF4G2	1982	ENSG00000110321	22473208	validated
mirtarbase	hsa-miR-582-5p	EIF4G2	1982	ENSG00000110321	21572407	validated
mirtarbase	hsa-miR-21-5p	EIF5	1983	ENSG00000100664	18591254	validated
mirtarbase	hsa-miR-106b-5p	ELAVL2	1993	ENSG00000107105	22012620	validated
mirtarbase	hsa-miR-296-5p	ELAVL1	1994	ENSG00000066044	26898797	validated
mirtarbase	hsa-miR-296-5p	ELAVL3	1995	ENSG00000196361	23824327	validated
mirtarbase	hsa-miR-582-5p	ELAVL3	1995	ENSG00000196361	27418678	validated

mirtarbase	hsa-miR-21-5p	ELAVL4	1996	ENSG00000162374	20584986	validated
mirtarbase	hsa-miR-505-3p	ELF4	2000	ENSG00000102034	23622248	validated
mirtarbase	hsa-miR-106b-5p	ELK3	2004	ENSG00000111145	17242205	validated
mirtarbase	hsa-miR-505-3p	ELK3	2004	ENSG00000111145	22012620	validated
mirtarbase	hsa-miR-181a-5p	ELK4	2005	ENSG00000158711	23824327	validated
mirtarbase	hsa-miR-15b-5p	ELK4	2005	ENSG00000158711	23446348	validated
mirtarbase	hsa-miR-106b-5p	ELK4	2005	ENSG00000158711	23446348	validated
mirtarbase	hsa-miR-192-5p	EML1	2009	ENSG00000066629	19074876	validated
mirtarbase	hsa-miR-15b-5p	EN2	2020	ENSG00000164778	23446348	validated
mirtarbase	hsa-miR-181a-5p	EN2	2020	ENSG00000164778	19536157	validated
mirtarbase	hsa-miR-222-3p	ENO1	2023	ENSG00000074800	23622248	validated
mirtarbase	hsa-miR-15b-5p	SLC29A1	2030	ENSG00000112759	21572407	validated
mirtarbase	hsa-miR-222-3p	EPB41L2	2037	ENSG00000079819	23622248	validated
mirtarbase	hsa-miR-21-5p	EPHA4	2043	ENSG00000116106	18591254	validated
mirtarbase	hsa-miR-505-3p	EPHA4	2043	ENSG00000116106	23622248	validated
mirtarbase	hsa-miR-106b-5p	EPHA4	2043	ENSG00000116106	21572407	validated
mirtarbase	hsa-miR-181a-5p	EPHA5	2044	ENSG00000145242	17612493	validated
mirtarbase	hsa-miR-326	EPHB3	2049	ENSG00000182580	23824327	validated
mirtarbase	hsa-miR-181a-5p	STX2	2054	ENSG00000111450	22473208	validated
mirtarbase	hsa-miR-18a-5p	CLN8	2055	ENSG00000182372	21572407	validated
mirtarbase	hsa-miR-192-5p	EPS8	2059	ENSG00000151491	19074876	validated
mirtarbase	hsa-miR-181a-5p	EPS8	2059	ENSG00000151491	23446348	validated
mirtarbase	hsa-miR-181a-5p	EPS15	2060	ENSG00000085832	22473208	validated
mirtarbase	hsa-miR-106b-5p	NR2F6	2063	ENSG00000160113	23446348	validated
mirtarbase	hsa-miR-582-5p	NR2F6	2063	ENSG00000160113	27292025	validated
mirtarbase	hsa-miR-21-5p	ERBB2	2064	ENSG00000141736	19419954	validated
mirtarbase	hsa-miR-326	ERBB2	2064	ENSG00000141736	23622248	validated
mirtarbase	hsa-miR-181a-5p	EREG	2069	ENSG00000124882	22815788	validated
mirtarbase	hsa-miR-106b-5p	EREG	2069	ENSG00000124882	26701625	validated
mirtarbase	hsa-miR-301a-3p	EREG	2069	ENSG00000124882	26701625	validated
mirtarbase	hsa-miR-181a-5p	EYA4	2070	ENSG00000112319	17612493	validated
mirtarbase	hsa-miR-192-5p	ERCC3	2071	ENSG00000163161	19074876	validated
mirtarbase	hsa-miR-192-5p	ERCC4	2072	ENSG00000175595	21672525	validated
mirtarbase	hsa-miR-301a-3p	ESR1	2099	ENSG00000091831	24398324	validated
mirtarbase	hsa-miR-192-5p	ESR1	2099	ENSG00000091831	27304060	validated
mirtarbase	hsa-miR-106b-5p	ESR2	2100	ENSG00000140009	23313552	validated
mirtarbase	hsa-miR-106b-5p	ETF1	2107	ENSG00000120705	22473208	validated
mirtarbase	hsa-miR-18a-5p	ETFA	2108	ENSG00000140374	23622248	validated
mirtarbase	hsa-miR-222-3p	ETS1	2113	ENSG00000134954	21310411	validated
mirtarbase	hsa-miR-181a-5p	ETS1	2113	ENSG00000134954	23824327	validated
mirtarbase	hsa-miR-144-3p	ETS1	2113	ENSG00000134954	26826553	validated
mirtarbase	hsa-miR-18a-5p	ETS2	2114	ENSG00000157557	23622248	validated
mirtarbase	hsa-miR-582-5p	ETS2	2114	ENSG00000157557	20371350	validated
mirtarbase	hsa-miR-15b-5p	EXT1	2131	ENSG00000182197	21572407	validated
mirtarbase	hsa-miR-192-5p	EXTL2	2135	ENSG00000162694	19074876	validated
mirtarbase	hsa-miR-15b-5p	EZH1	2145	ENSG00000108799	22473208	validated
mirtarbase	hsa-miR-106b-5p	EZH1	2145	ENSG00000108799	22473208	validated
mirtarbase	hsa-miR-144-3p	EZH2	2146	ENSG00000106462	23815091	validated
mirtarbase	hsa-miR-106b-5p	F2R	2149	ENSG00000181104	20371350	validated
mirtarbase	hsa-miR-301a-3p	F2RL1	2150	ENSG00000164251	23824327	validated
mirtarbase	hsa-miR-106b-5p	F2RL1	2150	ENSG00000164251	23313552	validated
mirtarbase	hsa-miR-18a-5p	F3	2152	ENSG00000117525	23592263	validated
mirtarbase	hsa-miR-106b-5p	F3	2152	ENSG00000117525	23592263	validated
mirtarbase	hsa-miR-326	F9	2158	ENSG00000101981	19536157	validated
mirtarbase	hsa-miR-15b-5p	FANCC	2176	ENSG00000158169	23622248	validated
mirtarbase	hsa-miR-505-3p	FANCD2	2177	ENSG00000144554	23622248	validated
mirtarbase	hsa-miR-192-5p	ACSL1	2180	ENSG00000151726	19074876	validated
mirtarbase	hsa-miR-106b-5p	ACSL4	2182	ENSG00000068366	21572407	validated
mirtarbase	hsa-miR-212-3p	ACSL4	2182	ENSG00000068366	21572407	validated
mirtarbase	hsa-miR-301a-3p	ACSL4	2182	ENSG00000068366	21572407	validated
mirtarbase	hsa-miR-144-3p	ACSL4	2182	ENSG00000068366	21572407	validated
mirtarbase	hsa-miR-222-3p	BPTF	2186	ENSG00000171634	23622248	validated
mirtarbase	hsa-miR-326	FASN	2194	ENSG00000169710	23622248	validated
mirtarbase	hsa-miR-222-3p	FASN	2194	ENSG00000169710	23622248	validated
mirtarbase	hsa-miR-15b-5p	FASN	2194	ENSG00000169710	21572407	validated
mirtarbase	hsa-miR-222-3p	FAT1	2195	ENSG00000083857	23622248	validated
mirtarbase	hsa-miR-181a-5p	FAT1	2195	ENSG00000083857	23622248	validated
mirtarbase	hsa-miR-192-5p	FBN1	2200	ENSG00000166147	19074876	validated
mirtarbase	hsa-miR-18a-5p	FCGR2B	2213	ENSG00000072694	24169826	validated
mirtarbase	hsa-miR-301a-3p	FKTN	2218	ENSG00000106692	19536157	validated
mirtarbase	hsa-miR-192-5p	FEN1	2237	ENSG00000168496	19074876	validated

mirtarbase	hsa-miR-192-5p	GPC4	2239	ENSG00000076716	19074876	validated
mirtarbase	hsa-miR-582-3p	FER	2241	ENSG00000151422	23824327	validated
mirtarbase	hsa-miR-144-3p	FGA	2243	ENSG00000171560	20570858	validated
mirtarbase	hsa-miR-144-3p	FGB	2244	ENSG00000171564	20570858	validated
mirtarbase	hsa-miR-326	FGF1	2246	ENSG00000113578	26183397	validated
mirtarbase	hsa-miR-192-5p	FGF2	2247	ENSG00000138685	19074876	validated
mirtarbase	hsa-miR-15b-5p	FGF2	2247	ENSG00000138685	24560946	validated
mirtarbase	hsa-miR-144-3p	FGF2	2247	ENSG00000138685	22012620	validated
mirtarbase	hsa-miR-582-5p	FGF2	2247	ENSG00000138685	22012620	validated
mirtarbase	hsa-miR-15b-5p	FGF5	2250	ENSG00000138675	23622248	validated
mirtarbase	hsa-miR-21-5p	FGF12	2257	ENSG00000114279	23824327	validated
mirtarbase	hsa-miR-296-5p	FGFR1	2260	ENSG00000077782	27714806	validated
mirtarbase	hsa-miR-15b-5p	FGFR4	2264	ENSG00000160867	23592263	validated
mirtarbase	hsa-miR-144-3p	FGG	2266	ENSG00000171557	20570858	validated
mirtarbase	hsa-miR-181a-5p	FKBP1A	2280	ENSG00000088832	21572407	validated
mirtarbase	hsa-miR-15b-5p	FKBP1A	2280	ENSG00000088832	21572407	validated
mirtarbase	hsa-miR-21-5p	FKBP5	2289	ENSG00000096060	18591254	validated
mirtarbase	hsa-miR-106b-5p	FOXC1	2296	ENSG00000054598	21572407	validated
mirtarbase	hsa-miR-181a-5p	FOXL1	2300	ENSG00000176678	21572407	validated
mirtarbase	hsa-miR-15b-5p	FOXO1	2308	ENSG00000150907	24862853	validated
mirtarbase	hsa-miR-222-3p	FOXO1	2308	ENSG00000150907	27746366	validated
mirtarbase	hsa-miR-21-5p	FOXO1	2308	ENSG00000150907	25909227	validated
mirtarbase	hsa-miR-222-3p	FOXO3	2309	ENSG00000118689	20388878	validated
mirtarbase	hsa-miR-21-5p	FOXO3	2309	ENSG00000118689	20371350	validated
mirtarbase	hsa-miR-144-3p	MLANA	2315	ENSG00000120215	22100165	validated
mirtarbase	hsa-miR-222-3p	FLNA	2316	ENSG00000196924	23622248	validated
mirtarbase	hsa-miR-15b-5p	FLOT2	2319	ENSG00000132589	22473208	validated
mirtarbase	hsa-miR-582-5p	FLT1	2321	ENSG00000102755	24906430	validated
mirtarbase	hsa-miR-21-5p	FMOD	2331	ENSG00000122176	19906824	validated
mirtarbase	hsa-miR-21-5p	FMR1	2332	ENSG00000102081	18591254	validated
mirtarbase	hsa-miR-181a-5p	FOS	2353	ENSG00000170345	22956783	validated
mirtarbase	hsa-miR-192-5p	FOS	2353	ENSG00000170345	19074876	validated
mirtarbase	hsa-miR-222-3p	FPGS	2356	ENSG00000136877	23622248	validated
mirtarbase	hsa-miR-326	FPR1	2357	ENSG00000171051	27418678	validated
mirtarbase	hsa-miR-144-3p	MTOR	2475	ENSG00000198793	22983984	validated
mirtarbase	hsa-miR-192-5p	CENPI	2491	ENSG00000102384	19074876	validated
mirtarbase	hsa-miR-15b-5p	FUT2	2524	ENSG00000176920	24122375	validated
mirtarbase	hsa-miR-106b-5p	FYN	2534	ENSG00000010810	27520374	validated
mirtarbase	hsa-miR-301a-3p	G6PC	2538	ENSG00000131482	23592263	validated
mirtarbase	hsa-miR-222-3p	SLC37A4	2542	ENSG00000137700	23622248	validated
mirtarbase	hsa-miR-505-3p	XRCC6	2547	ENSG00000196419	23622248	validated
mirtarbase	hsa-miR-326	XRCC6	2547	ENSG00000196419	23622248	validated
mirtarbase	hsa-miR-106b-5p	GAB1	2549	ENSG00000109458	22473208	validated
mirtarbase	hsa-miR-106b-5p	GABBR1	2550	ENSG00000206511	22473208	validated
mirtarbase	hsa-miR-18a-5p	GABPA	2551	ENSG00000154727	20371350	validated
mirtarbase	hsa-miR-15b-5p	GABPA	2551	ENSG00000154727	21572407	validated
mirtarbase	hsa-miR-106b-5p	GABPB1	2553	ENSG00000104064	22473208	validated
mirtarbase	hsa-miR-192-5p	GABPB1	2553	ENSG00000104064	22012620	validated
mirtarbase	hsa-miR-301a-3p	GAK	2580	ENSG00000178950	23622248	validated
mirtarbase	hsa-miR-106b-5p	GAK	2580	ENSG00000178950	22473208	validated
mirtarbase	hsa-miR-15b-5p	GALNT1	2589	ENSG00000141429	21572407	validated
mirtarbase	hsa-miR-222-3p	GALNT3	2591	ENSG00000115339	22012620	validated
mirtarbase	hsa-miR-505-3p	GAS1	2619	ENSG00000180447	21572407	validated
mirtarbase	hsa-miR-106b-5p	GATA6	2627	ENSG00000141448	24398324	validated
mirtarbase	hsa-miR-192-5p	GATM	2628	ENSG00000171766	19074876	validated
mirtarbase	hsa-miR-106b-5p	GBP3	2635	ENSG00000117226	22473208	validated
mirtarbase	hsa-miR-192-5p	GCH1	2643	ENSG00000131979	19074876	validated
mirtarbase	hsa-miR-18a-5p	GCH1	2643	ENSG00000131979	23622248	validated
mirtarbase	hsa-miR-181a-5p	NR6A1	2649	ENSG00000148200	20371350	validated
mirtarbase	hsa-miR-15b-5p	NR6A1	2649	ENSG00000148200	23592263	validated
mirtarbase	hsa-miR-181a-5p	GCNT1	2650	ENSG00000187210	17612493	validated
mirtarbase	hsa-miR-222-3p	GDI1	2664	ENSG00000203879	23622248	validated
mirtarbase	hsa-miR-222-3p	GDI2	2665	ENSG00000057608	23622248	validated
mirtarbase	hsa-miR-505-3p	GGCX	2677	ENSG00000115486	23824327	validated
mirtarbase	hsa-miR-15b-5p	B4GALT1	2683	ENSG00000086062	23622248	validated
mirtarbase	hsa-miR-18a-5p	B4GALT1	2683	ENSG00000086062	23622248	validated
mirtarbase	hsa-miR-582-3p	B4GALT1	2683	ENSG00000086062	23592263	validated
mirtarbase	hsa-miR-296-5p	B4GALT1	2683	ENSG00000086062	23313552	validated
mirtarbase	hsa-miR-222-3p	GJA1	2697	ENSG00000152661	22294051	validated
mirtarbase	hsa-miR-21-5p	GLG1	2734	ENSG00000090863	18591254	validated
mirtarbase	hsa-miR-326	GLI1	2735	ENSG00000111087	18756266	validated

mirtarbase	hsa-miR-222-3p	GLO1	2739	ENSG00000124767	23622248	validated
mirtarbase	hsa-miR-106b-5p	GLO1	2739	ENSG00000124767	22473208	validated
mirtarbase	hsa-miR-301a-3p	GMFB	2764	ENSG00000197045	23592263	validated
mirtarbase	hsa-miR-222-3p	GNAI2	2771	ENSG00000114353	27811362	validated
mirtarbase	hsa-miR-181a-5p	GNAI3	2773	ENSG00000065135	20371350	validated
mirtarbase	hsa-miR-222-3p	GNAI3	2773	ENSG00000065135	25444921	validated
mirtarbase	hsa-miR-15b-5p	GNAL	2774	ENSG00000141404	23592263	validated
mirtarbase	hsa-miR-21-5p	GNAQ	2776	ENSG00000156052	18591254	validated
mirtarbase	hsa-miR-106b-5p	GNAS	2778	ENSG00000087460	22473208	validated
mirtarbase	hsa-miR-18a-5p	GNAS	2778	ENSG00000087460	23592263	validated
mirtarbase	hsa-miR-15b-5p	GNAT1	2779	ENSG00000114349	21572407	validated
mirtarbase	hsa-miR-15b-5p	GNB1	2782	ENSG00000078369	22473208	validated
mirtarbase	hsa-miR-582-5p	GNB1	2782	ENSG00000078369	21572407	validated
mirtarbase	hsa-miR-212-3p	GNB1	2782	ENSG00000078369	21572407	validated
mirtarbase	hsa-miR-296-5p	GNB2	2783	ENSG00000172354	23592263	validated
mirtarbase	hsa-miR-18a-5p	GNL1	2794	ENSG00000206492	23592263	validated
mirtarbase	hsa-miR-192-5p	GNRH1	2796	ENSG00000147437	19074876	validated
mirtarbase	hsa-miR-106b-5p	GNS	2799	ENSG00000135677	22473208	validated
mirtarbase	hsa-miR-181a-5p	GNS	2799	ENSG00000135677	22473208	validated
mirtarbase	hsa-miR-106b-5p	GOLGA1	2800	ENSG00000136935	22473208	validated
mirtarbase	hsa-miR-181a-5p	GOLGA1	2800	ENSG00000136935	22473208	validated
mirtarbase	hsa-miR-106b-5p	GOLGA2	2801	ENSG00000167110	19536157	validated
mirtarbase	hsa-miR-21-5p	GOLGA4	2803	ENSG00000144674	18591254	validated
mirtarbase	hsa-miR-181a-5p	GOT1	2805	ENSG00000120053	22473208	validated
mirtarbase	hsa-miR-301a-3p	GP2	2813	ENSG00000169347	23824327	validated
mirtarbase	hsa-miR-21-5p	GP5	2814	ENSG00000178732	22012620	validated
mirtarbase	hsa-miR-21-5p	GPD2	2820	ENSG00000115159	18591254	validated
mirtarbase	hsa-miR-505-3p	GPLD1	2822	ENSG00000112293	23622248	validated
mirtarbase	hsa-miR-106b-5p	GPM6A	2823	ENSG00000150625	17242205	validated
mirtarbase	hsa-miR-192-5p	GPR19	2842	ENSG00000183150	19074876	validated
mirtarbase	hsa-miR-15b-5p	GPR27	2850	ENSG00000170837	21572407	validated
mirtarbase	hsa-miR-192-5p	GPR37	2861	ENSG00000170775	19074876	validated
mirtarbase	hsa-miR-326	GRK6	2870	ENSG00000198055	23622248	validated
mirtarbase	hsa-miR-21-5p	MKNK2	2872	ENSG00000099875	18591254	validated
mirtarbase	hsa-miR-106b-5p	MKNK2	2872	ENSG00000099875	22473208	validated
mirtarbase	hsa-miR-15b-5p	GRB2	2885	ENSG00000177885	21572407	validated
mirtarbase	hsa-miR-222-3p	GRB10	2887	ENSG00000106070	23622248	validated
mirtarbase	hsa-miR-301a-3p	GRB10	2887	ENSG00000106070	23446348	validated
mirtarbase	hsa-miR-192-5p	GRIA1	2890	ENSG00000155511	16822819	validated
mirtarbase	hsa-miR-144-3p	GRIK3	2899	ENSG00000163873	23446348	validated
mirtarbase	hsa-miR-18a-5p	NR3C1	2908	ENSG00000113580	19131573	validated
mirtarbase	hsa-miR-144-3p	NR3C1	2908	ENSG00000113580	20371350	validated
mirtarbase	hsa-miR-106b-5p	NR3C1	2908	ENSG00000113580	27292025	validated
mirtarbase	hsa-miR-106b-5p	ARHGAP35	2909	ENSG00000160007	22473208	validated
mirtarbase	hsa-miR-582-3p	GRM5	2915	ENSG00000168959	23446348	validated
mirtarbase	hsa-miR-582-3p	GRM6	2916	ENSG00000113262	23824327	validated
mirtarbase	hsa-miR-301a-3p	GRSF1	2926	ENSG00000132463	27292025	validated
mirtarbase	hsa-miR-15b-5p	GSK3B	2932	ENSG00000082701	22473208	validated
mirtarbase	hsa-miR-181a-5p	GSTM2	2946	ENSG00000213366	17612493	validated
mirtarbase	hsa-miR-21-5p	MSH6	2956	ENSG00000116062	21078976	validated
mirtarbase	hsa-miR-192-5p	MSH6	2956	ENSG00000116062	19074876	validated
mirtarbase	hsa-miR-106b-5p	MSH6	2956	ENSG00000116062	23622248	validated
mirtarbase	hsa-miR-21-5p	GTF2A1	2957	ENSG00000165417	18591254	validated
mirtarbase	hsa-miR-106b-5p	GTF2H3	2967	ENSG00000111358	23313552	validated
mirtarbase	hsa-miR-21-5p	GTF2I	2969	ENSG00000263001	18591254	validated
mirtarbase	hsa-miR-326	GYS1	2997	ENSG00000104812	19536157	validated
mirtarbase	hsa-miR-181a-5p	H1-0	3005	ENSG00000189060	20371350	validated
mirtarbase	hsa-miR-106b-5p	H2BC5	3017	ENSG00000158373	21572407	validated
mirtarbase	hsa-miR-192-5p	H3-3A	3020	ENSG00000163041	28217257	validated
mirtarbase	hsa-miR-181a-5p	H3-3B	3021	ENSG00000132475	20371350	validated
mirtarbase	hsa-miR-505-3p	H3-3B	3021	ENSG00000132475	23446348	validated
mirtarbase	hsa-miR-301a-3p	HADHB	3032	ENSG00000138029	22012620	validated
mirtarbase	hsa-miR-192-5p	HADH	3033	ENSG00000138796	19074876	validated
mirtarbase	hsa-miR-106b-5p	HAS2	3037	ENSG00000170961	22927820	validated
mirtarbase	hsa-miR-192-5p	HAS3	3038	ENSG00000103044	19074876	validated
mirtarbase	hsa-miR-222-3p	HCFC1	3054	ENSG00000172534	23622248	validated
mirtarbase	hsa-miR-106b-5p	HTT	3064	ENSG00000197386	23622248	validated
mirtarbase	hsa-miR-222-3p	HDGF	3068	ENSG00000143321	23622248	validated
mirtarbase	hsa-miR-15b-5p	HDGF	3068	ENSG00000143321	23592263	validated
mirtarbase	hsa-miR-222-3p	HDLBP	3069	ENSG00000115677	23622248	validated
mirtarbase	hsa-miR-505-3p	HDLBP	3069	ENSG00000115677	23592263	validated

mirtarbase	hsa-miR-582-5p	HFE	3077	ENSG00000010704	27292025	validated
mirtarbase	hsa-miR-18a-5p	HIF1A	3091	ENSG00000100644	20371350	validated
mirtarbase	hsa-miR-106b-5p	HIF1A	3091	ENSG00000100644	22473208	validated
mirtarbase	hsa-miR-21-5p	HIF1A	3091	ENSG00000100644	23726431	validated
mirtarbase	hsa-miR-106b-5p	HIP1	3092	ENSG00000127946	23824327	validated
mirtarbase	hsa-miR-192-5p	UBE2K	3093	ENSG00000078140	22100165	validated
mirtarbase	hsa-miR-326	HLA-C	3107	ENSG00000206435	26701625	validated
mirtarbase	hsa-miR-582-5p	MNX1	3110	ENSG00000130675	23446348	validated
mirtarbase	hsa-miR-106b-5p	HMGB1	3146	ENSG00000189403	23446348	validated
mirtarbase	hsa-miR-21-5p	HMGB1	3146	ENSG00000189403	25327529	validated
mirtarbase	hsa-miR-181a-5p	HMGB2	3148	ENSG00000164104	17612493	validated
mirtarbase	hsa-miR-582-5p	HMGB2	3148	ENSG00000164104	23592263	validated
mirtarbase	hsa-miR-106b-5p	HMGB2	3148	ENSG00000164104	20371350	validated
mirtarbase	hsa-miR-106b-5p	HMGB3	3149	ENSG00000029993	23592263	validated
mirtarbase	hsa-miR-21-5p	HMGB3	3149	ENSG00000029993	18591254	validated
mirtarbase	hsa-miR-106b-5p	HMGCL	3155	ENSG00000117305	23622248	validated
mirtarbase	hsa-miR-192-5p	HMGCS1	3157	ENSG00000112972	19074876	validated
mirtarbase	hsa-miR-18a-5p	HMGCS1	3157	ENSG00000112972	20371350	validated
mirtarbase	hsa-miR-222-3p	HMGA1	3159	ENSG00000137309	23622248	validated
mirtarbase	hsa-miR-15b-5p	HMGA1	3159	ENSG00000137309	22473208	validated
mirtarbase	hsa-miR-296-5p	HMGA1	3159	ENSG00000137309	26898758	validated
mirtarbase	hsa-miR-192-5p	HMMR	3161	ENSG00000072571	19074876	validated
mirtarbase	hsa-miR-106b-5p	HMMR	3161	ENSG00000072571	23622248	validated
mirtarbase	hsa-miR-192-5p	FOXA1	3169	ENSG00000129514	19074876	validated
mirtarbase	hsa-miR-15b-5p	HNRNPA1	3178	ENSG00000135486	23313552	validated
mirtarbase	hsa-miR-326	HNRNPA1	3178	ENSG00000135486	23313552	validated
mirtarbase	hsa-miR-15b-5p	HNRNPA2B1	3181	ENSG00000122566	20371350	validated
mirtarbase	hsa-miR-181a-5p	HNRNPAB	3182	ENSG00000197451	23622248	validated
mirtarbase	hsa-miR-144-3p	HNRNPAB	3182	ENSG00000197451	20371350	validated
mirtarbase	hsa-miR-222-3p	HNRNPD	3184	ENSG00000138668	23622248	validated
mirtarbase	hsa-miR-505-3p	HNRNPF	3185	ENSG00000169813	23622248	validated
mirtarbase	hsa-miR-144-3p	HNRNPF	3185	ENSG00000169813	23592263	validated
mirtarbase	hsa-miR-21-5p	HNRNPH1	3187	ENSG00000169045	18591254	validated
mirtarbase	hsa-miR-222-3p	HNRNPH1	3187	ENSG00000169045	23622248	validated
mirtarbase	hsa-miR-181a-5p	HNRNPH1	3187	ENSG00000169045	27418678	validated
mirtarbase	hsa-miR-15b-5p	HNRNPH3	3189	ENSG00000096746	23622248	validated
mirtarbase	hsa-miR-15b-5p	HNRNPK	3190	ENSG00000165119	23622248	validated
mirtarbase	hsa-miR-106b-5p	HNRNPL	3191	ENSG00000104824	23622248	validated
mirtarbase	hsa-miR-144-3p	HNRNPU	3192	ENSG00000153187	26701625	validated
mirtarbase	hsa-miR-15b-5p	HOXA3	3200	ENSG00000105997	23592263	validated
mirtarbase	hsa-miR-301a-3p	HOXA5	3202	ENSG00000106004	21572407	validated
mirtarbase	hsa-miR-21-5p	HOXA9	3205	ENSG00000078399	18591254	validated
mirtarbase	hsa-miR-18a-5p	HOXA9	3205	ENSG00000078399	20371350	validated
mirtarbase	hsa-miR-505-3p	HOXA9	3205	ENSG00000078399	21572407	validated
mirtarbase	hsa-miR-192-5p	HOXA10	3206	ENSG00000253293	19074876	validated
mirtarbase	hsa-miR-144-3p	HOXA10	3206	ENSG00000253293	23446348	validated
mirtarbase	hsa-miR-15b-5p	HOXA10	3206	ENSG00000253293	21572407	validated
mirtarbase	hsa-miR-192-5p	HOXA13	3209	ENSG00000106031	19074876	validated
mirtarbase	hsa-miR-222-3p	HOXA13	3209	ENSG00000106031	23622248	validated
mirtarbase	hsa-miR-301a-3p	HOXB3	3213	ENSG00000120093	21572407	validated
mirtarbase	hsa-miR-192-5p	HOXB9	3219	ENSG00000170689	19074876	validated
mirtarbase	hsa-miR-212-3p	HOXC4	3221	ENSG00000198353	23592263	validated
mirtarbase	hsa-miR-15b-5p	HOXC8	3224	ENSG00000037965	21572407	validated
mirtarbase	hsa-miR-301a-3p	HOXD11	3237	ENSG00000128713	21572407	validated
mirtarbase	hsa-miR-106b-5p	HOXD11	3237	ENSG00000128713	21572407	validated
mirtarbase	hsa-miR-21-5p	HPGD	3248	ENSG00000164120	24699315	validated
mirtarbase	hsa-miR-301a-3p	HPRT1	3251	ENSG00000165704	20371350	validated
mirtarbase	hsa-miR-181a-5p	HRAS	3265	ENSG00000276536	21167132	validated
mirtarbase	hsa-miR-18a-5p	AGFG1	3267	ENSG00000173744	23446348	validated
mirtarbase	hsa-miR-106b-5p	AGFG2	3268	ENSG00000106351	22473208	validated
mirtarbase	hsa-miR-192-5p	HRH1	3269	ENSG00000196639	19074876	validated
mirtarbase	hsa-miR-222-3p	HES1	3280	ENSG00000114315	23622248	validated
mirtarbase	hsa-miR-181a-5p	HSD17B3	3293	ENSG00000130948	17612493	validated
mirtarbase	hsa-miR-15b-5p	DNAJA1	3301	ENSG00000086061	24398324	validated
mirtarbase	hsa-miR-582-5p	DNAJA1	3301	ENSG00000086061	23446348	validated
mirtarbase	hsa-miR-505-3p	HSPA1B	3304	ENSG00000231555	23622248	validated
mirtarbase	hsa-miR-326	HSPA1B	3304	ENSG00000231555	23622248	validated
mirtarbase	hsa-miR-106b-5p	HSPA1B	3304	ENSG00000231555	23622248	validated
mirtarbase	hsa-miR-212-3p	HSPA1B	3304	ENSG00000231555	21572407	validated
mirtarbase	hsa-miR-15b-5p	HSPA1B	3304	ENSG00000231555	22473208	validated
mirtarbase	hsa-miR-181a-5p	HSPA1B	3304	ENSG00000231555	24398324	validated

mirtarbase	hsa-miR-192-5p	HSPA2	3306	ENSG00000126803	19074876	validated
mirtarbase	hsa-miR-15b-5p	HSPA8	3312	ENSG00000109971	23622248	validated
mirtarbase	hsa-miR-106b-5p	HSPA8	3312	ENSG00000109971	23592263	validated
mirtarbase	hsa-miR-301a-3p	HSPA8	3312	ENSG00000109971	23592263	validated
mirtarbase	hsa-miR-222-3p	HSP90AA1	3320	ENSG00000080824	23622248	validated
mirtarbase	hsa-miR-582-5p	HSP90AA1	3320	ENSG00000080824	21572407	validated
mirtarbase	hsa-miR-15b-5p	HSP90AB1	3326	ENSG00000096384	23622248	validated
mirtarbase	hsa-miR-21-5p	FOXN2	3344	ENSG00000170802	23592263	validated
mirtarbase	hsa-miR-222-3p	IARS1	3376	ENSG00000196305	23622248	validated
mirtarbase	hsa-miR-222-3p	ICAM1	3383	ENSG00000090339	19949084	validated
mirtarbase	hsa-miR-21-5p	ICAM1	3383	ENSG00000090339	21131358	validated
mirtarbase	hsa-miR-192-5p	ICAM3	3385	ENSG00000076662	19074876	validated
mirtarbase	hsa-miR-192-5p	ID1	3397	ENSG00000125968	19074876	validated
mirtarbase	hsa-miR-192-5p	ID2	3398	ENSG00000115738	19074876	validated
mirtarbase	hsa-miR-192-5p	ID4	3400	ENSG00000172201	20371350	validated
mirtarbase	hsa-miR-18a-5p	ID4	3400	ENSG00000172201	20371350	validated
mirtarbase	hsa-miR-181a-5p	ID4	3400	ENSG00000172201	20371350	validated
mirtarbase	hsa-miR-505-3p	ID4	3400	ENSG00000172201	20371350	validated
mirtarbase	hsa-miR-192-5p	IDI1	3422	ENSG00000067064	19074876	validated
mirtarbase	hsa-miR-181a-5p	IDS	3423	ENSG00000010404	17612493	validated
mirtarbase	hsa-miR-181a-5p	CFI	3426	ENSG00000205403	17612493	validated
mirtarbase	hsa-miR-106b-5p	IFNAR1	3454	ENSG00000142166	22473208	validated
mirtarbase	hsa-miR-106b-5p	IFNAR2	3455	ENSG00000159110	17242205	validated
mirtarbase	hsa-miR-18a-5p	IFNAR2	3455	ENSG00000159110	23446348	validated
mirtarbase	hsa-miR-15b-5p	IFNG	3458	ENSG00000111537	22379033	validated
mirtarbase	hsa-miR-181a-5p	IFNG	3458	ENSG00000111537	24704866	validated
mirtarbase	hsa-miR-15b-5p	IFNGR2	3460	ENSG00000262795	23622248	validated
mirtarbase	hsa-miR-192-5p	IFRD1	3475	ENSG00000006652	19074876	validated
mirtarbase	hsa-miR-505-3p	IGF1	3479	ENSG00000017427	22012620	validated
mirtarbase	hsa-miR-192-5p	IGF1R	3480	ENSG00000140443	19074876	validated
mirtarbase	hsa-miR-21-5p	IGF1R	3480	ENSG00000140443	24161401	validated
mirtarbase	hsa-miR-301a-3p	IGF2R	3482	ENSG00000197081	19536157	validated
mirtarbase	hsa-miR-301a-3p	IGFBP5	3488	ENSG00000115461	20371350	validated
mirtarbase	hsa-miR-18a-5p	IGHMBP2	3508	ENSG00000132740	23622248	validated
mirtarbase	hsa-miR-18a-5p	RBPJ	3516	ENSG00000168214	23622248	validated
mirtarbase	hsa-miR-15b-5p	RBPJ	3516	ENSG00000168214	23592263	validated
mirtarbase	hsa-miR-326	IHH	3549	ENSG00000163501	22100165	validated
mirtarbase	hsa-miR-181a-5p	IL1A	3552	ENSG00000115008	22473208	validated
mirtarbase	hsa-miR-21-5p	IL1B	3553	ENSG00000125538	21131358	validated
mirtarbase	hsa-miR-192-5p	IL1R1	3554	ENSG00000115594	19074876	validated
mirtarbase	hsa-miR-192-5p	IL1RAP	3556	ENSG00000196083	19074876	validated
mirtarbase	hsa-miR-192-5p	IL6R	3570	ENSG00000160712	23313552	validated
mirtarbase	hsa-miR-192-5p	IL6ST	3572	ENSG00000134352	19074876	validated
mirtarbase	hsa-miR-192-5p	IL7	3574	ENSG00000104432	19074876	validated
mirtarbase	hsa-miR-21-5p	IL12A	3592	ENSG00000168811	22815788	validated
mirtarbase	hsa-miR-192-5p	IL15	3600	ENSG00000164136	19074876	validated
mirtarbase	hsa-miR-106b-5p	FOXK2	3607	ENSG00000141568	22473208	validated
mirtarbase	hsa-miR-222-3p	ILK	3611	ENSG00000166333	23622248	validated
mirtarbase	hsa-miR-192-5p	IMPA1	3612	ENSG00000133731	19074876	validated
mirtarbase	hsa-miR-181a-5p	INCENP	3619	ENSG00000149503	17612493	validated
mirtarbase	hsa-miR-21-5p	CXCL10	3627	ENSG00000169245	24889251	validated
mirtarbase	hsa-miR-326	INPPL1	3636	ENSG00000165458	23622248	validated
mirtarbase	hsa-miR-192-5p	INSIG1	3638	ENSG00000186480	19074876	validated
mirtarbase	hsa-miR-106b-5p	INSIG1	3638	ENSG00000186480	23622248	validated
mirtarbase	hsa-miR-15b-5p	INSR	3643	ENSG00000171105	26179126	validated
mirtarbase	hsa-miR-21-5p	IPP	3652	ENSG00000197429	18591254	validated
mirtarbase	hsa-miR-222-3p	IPP	3652	ENSG00000197429	23622248	validated
mirtarbase	hsa-miR-106b-5p	IPP	3652	ENSG00000197429	23313552	validated
mirtarbase	hsa-miR-192-5p	IRAK1	3654	ENSG00000184216	19074876	validated
mirtarbase	hsa-miR-222-3p	IRAK1	3654	ENSG00000184216	23622248	validated
mirtarbase	hsa-miR-21-5p	IRAK1	3654	ENSG00000184216	23633945	validated
mirtarbase	hsa-miR-15b-5p	ITGA6	3655	ENSG00000091409	23622248	validated
mirtarbase	hsa-miR-21-5p	IREB2	3658	ENSG00000136381	18591254	validated
mirtarbase	hsa-miR-301a-3p	IRF1	3659	ENSG00000125347	23824327	validated
mirtarbase	hsa-miR-18a-5p	IRF2	3660	ENSG00000168310	22012620	validated
mirtarbase	hsa-miR-15b-5p	IRF4	3662	ENSG00000137265	22473208	validated
mirtarbase	hsa-miR-144-3p	IRS1	3667	ENSG00000169047	27069535	validated
mirtarbase	hsa-miR-15b-5p	ITGA2	3673	ENSG00000164171	21572407	validated
mirtarbase	hsa-miR-18a-5p	ITGA2	3673	ENSG00000164171	21572407	validated
mirtarbase	hsa-miR-106b-5p	ITGA2	3673	ENSG00000164171	22100165	validated
mirtarbase	hsa-miR-192-5p	ITGA3	3675	ENSG00000005884	27292025	validated

mirtarbase	hsa-miR-192-5p	ITGAV	3685	ENSG00000138448	19074876	validated
mirtarbase	hsa-miR-192-5p	ITGB1	3688	ENSG00000150093	19074876	validated
mirtarbase	hsa-miR-106b-5p	ITGB1	3688	ENSG00000150093	20371350	validated
mirtarbase	hsa-miR-192-5p	ITGB3	3690	ENSG00000259207	26506238	validated
mirtarbase	hsa-miR-505-3p	EIF6	3692	ENSG00000242372	22012620	validated
mirtarbase	hsa-miR-21-5p	ITGB8	3696	ENSG00000105855	22473208	validated
mirtarbase	hsa-miR-106b-5p	ITPKB	3707	ENSG00000143772	22473208	validated
mirtarbase	hsa-miR-15b-5p	ITPR1	3708	ENSG00000150995	22473208	validated
mirtarbase	hsa-miR-106b-5p	JAK1	3716	ENSG00000162434	17242205	validated
mirtarbase	hsa-miR-15b-5p	JAK1	3716	ENSG00000162434	23622248	validated
mirtarbase	hsa-miR-15b-5p	JARID2	3720	ENSG00000008083	23446348	validated
mirtarbase	hsa-miR-505-3p	JARID2	3720	ENSG00000008083	22012620	validated
mirtarbase	hsa-miR-301a-3p	JARID2	3720	ENSG00000008083	23313552	validated
mirtarbase	hsa-miR-106b-5p	KCNA7	3743	ENSG00000104848	23446348	validated
mirtarbase	hsa-miR-106b-5p	KCNB1	3745	ENSG00000158445	20371350	validated
mirtarbase	hsa-miR-301a-3p	KCNB1	3745	ENSG00000158445	20371350	validated
mirtarbase	hsa-miR-582-5p	KCNB1	3745	ENSG00000158445	23824327	validated
mirtarbase	hsa-miR-582-3p	KCNC1	3746	ENSG00000129159	23295946	validated
mirtarbase	hsa-miR-582-5p	KCNC1	3746	ENSG00000129159	23295946	validated
mirtarbase	hsa-miR-192-5p	KCNC3	3748	ENSG00000131398	23824327	validated
mirtarbase	hsa-miR-106b-5p	KCND3	3752	ENSG00000171385	23313552	validated
mirtarbase	hsa-miR-505-3p	KCNH1	3756	ENSG00000143473	19536157	validated
mirtarbase	hsa-miR-212-3p	KCNJ2	3759	ENSG00000123700	22880819	validated
mirtarbase	hsa-miR-106b-5p	KCNJ8	3764	ENSG00000121361	22012620	validated
mirtarbase	hsa-miR-106b-5p	KCNMB1	3779	ENSG00000145936	23446348	validated
mirtarbase	hsa-miR-192-5p	KCNS3	3790	ENSG00000170745	19074876	validated
mirtarbase	hsa-miR-15b-5p	KDR	3791	ENSG00000128052	23688497	validated
mirtarbase	hsa-miR-296-5p	KDR	3791	ENSG00000128052	26500043	validated
mirtarbase	hsa-miR-192-5p	KIF5B	3799	ENSG00000170759	19074876	validated
mirtarbase	hsa-miR-15b-5p	KIF5B	3799	ENSG00000170759	22473208	validated
mirtarbase	hsa-miR-192-5p	KIF5C	3800	ENSG00000276734	19074876	validated
mirtarbase	hsa-miR-106b-5p	KIF5C	3800	ENSG00000276734	23622248	validated
mirtarbase	hsa-miR-222-3p	KIF5C	3800	ENSG00000276734	23622248	validated
mirtarbase	hsa-miR-326	KLK2	3817	ENSG00000167751	23824327	validated
mirtarbase	hsa-miR-21-5p	KLK2	3817	ENSG00000167751	19536157	validated
mirtarbase	hsa-miR-301a-3p	KLRD1	3824	ENSG00000134539	19536157	validated
mirtarbase	hsa-miR-106b-5p	KLRD1	3824	ENSG00000134539	19536157	validated
mirtarbase	hsa-miR-15b-5p	KPNA1	3836	ENSG00000114030	21572407	validated
mirtarbase	hsa-miR-212-3p	KPNA1	3836	ENSG00000114030	21572407	validated
mirtarbase	hsa-miR-181a-5p	KPNA1	3836	ENSG00000114030	22473208	validated
mirtarbase	hsa-miR-222-3p	KPNB1	3837	ENSG00000108424	23622248	validated
mirtarbase	hsa-miR-326	KPNA2	3838	ENSG00000182481	23622248	validated
mirtarbase	hsa-miR-144-3p	KPNA2	3838	ENSG00000182481	23592263	validated
mirtarbase	hsa-miR-106b-5p	KPNA2	3838	ENSG00000182481	22473208	validated
mirtarbase	hsa-miR-582-5p	KPNA2	3838	ENSG00000182481	23592263	validated
mirtarbase	hsa-miR-222-3p	KPNA2	3838	ENSG00000182481	23592263	validated
mirtarbase	hsa-miR-15b-5p	KPNA3	3839	ENSG00000102753	21572407	validated
mirtarbase	hsa-miR-192-5p	KPNA5	3841	ENSG00000196911	19074876	validated
mirtarbase	hsa-miR-21-5p	TNPO1	3842	ENSG00000083312	18591254	validated
mirtarbase	hsa-miR-18a-5p	TNPO1	3842	ENSG00000083312	23622248	validated
mirtarbase	hsa-miR-181a-5p	TNPO1	3842	ENSG00000083312	23592263	validated
mirtarbase	hsa-miR-144-3p	TNPO1	3842	ENSG00000083312	23592263	validated
mirtarbase	hsa-miR-505-3p	TNPO1	3842	ENSG00000083312	23313552	validated
mirtarbase	hsa-miR-222-3p	IPO5	3843	ENSG00000065150	23622248	validated
mirtarbase	hsa-miR-181a-5p	IPO5	3843	ENSG00000065150	22473208	validated
mirtarbase	hsa-miR-181a-5p	KRAS	3845	ENSG00000133703	20371350	validated
mirtarbase	hsa-miR-326	KRAS	3845	ENSG00000133703	24398324	validated
mirtarbase	hsa-miR-106b-5p	KRT10	3858	ENSG00000186395	23592263	validated
mirtarbase	hsa-miR-15b-5p	KRT33B	3884	ENSG00000263012	20371350	validated
mirtarbase	hsa-miR-181a-5p	LAMA3	3909	ENSG00000053747	23622248	validated
mirtarbase	hsa-miR-106b-5p	LAMC1	3915	ENSG00000135862	22473208	validated
mirtarbase	hsa-miR-15b-5p	LAMC1	3915	ENSG00000135862	23592263	validated
mirtarbase	hsa-miR-21-5p	LAMP2	3920	ENSG00000005893	18591254	validated
mirtarbase	hsa-miR-15b-5p	LAMP2	3920	ENSG00000005893	23446348	validated
mirtarbase	hsa-miR-222-3p	STMN1	3925	ENSG00000117632	23446348	validated
mirtarbase	hsa-miR-106b-5p	LASP1	3927	ENSG00000002834	22473208	validated
mirtarbase	hsa-miR-181a-5p	LBR	3930	ENSG00000143815	20371350	validated
mirtarbase	hsa-miR-106b-5p	LDLR	3949	ENSG00000130164	22473208	validated
mirtarbase	hsa-miR-301a-3p	LDLR	3949	ENSG00000130164	22012620	validated
mirtarbase	hsa-miR-181a-5p	LDLR	3949	ENSG00000130164	23313552	validated
mirtarbase	hsa-miR-212-3p	LDLR	3949	ENSG00000130164	21572407	validated

mirtarbase	hsa-miR-181a-5p	LFNG	3955	ENSG00000106003	17612493	validated
mirtarbase	hsa-miR-222-3p	LGALS3BP	3959	ENSG00000108679	23622248	validated
mirtarbase	hsa-miR-505-3p	LGALS8	3964	ENSG00000116977	28735896	validated
mirtarbase	hsa-miR-21-5p	LIFR	3977	ENSG00000113594	18591254	validated
mirtarbase	hsa-miR-212-3p	LIFR	3977	ENSG00000113594	21572407	validated
mirtarbase	hsa-miR-144-3p	LIFR	3977	ENSG00000113594	21572407	validated
mirtarbase	hsa-miR-222-3p	ABLIM1	3983	ENSG00000099204	22100165	validated
mirtarbase	hsa-miR-106b-5p	LIMK1	3984	ENSG00000106683	17242205	validated
mirtarbase	hsa-miR-192-5p	LIMS1	3987	ENSG00000169756	19074876	validated
mirtarbase	hsa-miR-505-3p	LIMS1	3987	ENSG00000169756	23622248	validated
mirtarbase	hsa-miR-192-5p	LIPA	3988	ENSG00000107798	19074876	validated
mirtarbase	hsa-miR-301a-3p	LIPA	3988	ENSG00000107798	21572407	validated
mirtarbase	hsa-miR-192-5p	FADS1	3992	ENSG00000149485	19074876	validated
mirtarbase	hsa-miR-192-5p	LMAN1	3998	ENSG00000074695	19074876	validated
mirtarbase	hsa-miR-181a-5p	LMAN1	3998	ENSG00000074695	22473208	validated
mirtarbase	hsa-miR-192-5p	LMNB1	4001	ENSG00000113368	19074876	validated
mirtarbase	hsa-miR-192-5p	LOXL2	4017	ENSG00000134013	16822819	validated
mirtarbase	hsa-miR-21-5p	LRP6	4040	ENSG00000070018	28337300	validated
mirtarbase	hsa-miR-106b-5p	LRPAP1	4043	ENSG00000163956	20371350	validated
mirtarbase	hsa-miR-15b-5p	CD180	4064	ENSG00000134061	22291592	validated
mirtarbase	hsa-miR-582-5p	CD180	4064	ENSG00000134061	26701625	validated
mirtarbase	hsa-miR-222-3p	LYN	4067	ENSG00000254087	23622248	validated
mirtarbase	hsa-miR-15b-5p	TM4SF1	4071	ENSG00000169908	23592263	validated
mirtarbase	hsa-miR-106b-5p	M6PR	4074	ENSG00000003056	22473208	validated
mirtarbase	hsa-miR-21-5p	CAPRIN1	4076	ENSG00000135387	18591254	validated
mirtarbase	hsa-miR-222-3p	CAPRIN1	4076	ENSG00000135387	23622248	validated
mirtarbase	hsa-miR-15b-5p	MARCKS	4082	ENSG00000277443	23622248	validated
mirtarbase	hsa-miR-192-5p	MAD2L1	4085	ENSG00000164109	19074876	validated
mirtarbase	hsa-miR-181a-5p	SMAD2	4087	ENSG00000175387	22942087	validated
mirtarbase	hsa-miR-18a-5p	SMAD2	4087	ENSG00000175387	24664294	validated
mirtarbase	hsa-miR-505-3p	SMAD2	4087	ENSG00000175387	23313552	validated
mirtarbase	hsa-miR-15b-5p	SMAD2	4087	ENSG00000175387	27499071	validated
mirtarbase	hsa-miR-212-3p	SMAD2	4087	ENSG00000175387	25988335	validated
mirtarbase	hsa-miR-18a-5p	SMAD3	4088	ENSG00000166949	23249750	validated
mirtarbase	hsa-miR-15b-5p	SMAD3	4088	ENSG00000166949	22473208	validated
mirtarbase	hsa-miR-18a-5p	SMAD4	4089	ENSG00000141646	20940405	validated
mirtarbase	hsa-miR-301a-3p	SMAD4	4089	ENSG00000141646	23393589	validated
mirtarbase	hsa-miR-106b-5p	SMAD4	4089	ENSG00000141646	23446348	validated
mirtarbase	hsa-miR-144-3p	SMAD4	4089	ENSG00000141646	26918315	validated
mirtarbase	hsa-miR-106b-5p	SMAD5	4090	ENSG00000113658	22473208	validated
mirtarbase	hsa-miR-222-3p	SMAD5	4090	ENSG00000113658	26809090	validated
mirtarbase	hsa-miR-106b-5p	SMAD6	4091	ENSG00000137834	22473208	validated
mirtarbase	hsa-miR-21-5p	SMAD7	4092	ENSG00000101665	23292313	validated
mirtarbase	hsa-miR-15b-5p	SMAD7	4092	ENSG00000101665	23622248	validated
mirtarbase	hsa-miR-106b-5p	SMAD7	4092	ENSG00000101665	22286770	validated
mirtarbase	hsa-miR-106b-5p	SMAD9	4093	ENSG00000120693	17242205	validated
mirtarbase	hsa-miR-326	MAGEB4	4115	ENSG00000120289	23824327	validated
mirtarbase	hsa-miR-192-5p	MAN2A2	4122	ENSG00000196547	19074876	validated
mirtarbase	hsa-miR-222-3p	MAP1B	4131	ENSG00000131711	23622248	validated
mirtarbase	hsa-miR-222-3p	MARS1	4141	ENSG00000166986	23622248	validated
mirtarbase	hsa-miR-326	MAT2A	4144	ENSG00000168906	23622248	validated
mirtarbase	hsa-miR-106b-5p	MAT2A	4144	ENSG00000168906	23622248	validated
mirtarbase	hsa-miR-222-3p	MAT2A	4144	ENSG00000168906	23592263	validated
mirtarbase	hsa-miR-326	MAZ	4150	ENSG00000103495	23622248	validated
mirtarbase	hsa-miR-222-3p	MAZ	4150	ENSG00000103495	23622248	validated
mirtarbase	hsa-miR-181a-5p	MAZ	4150	ENSG00000103495	23622248	validated
mirtarbase	hsa-miR-505-3p	MAZ	4150	ENSG00000103495	19536157	validated
mirtarbase	hsa-miR-18a-5p	MBD1	4152	ENSG00000141644	23313552	validated
mirtarbase	hsa-miR-21-5p	MBNL1	4154	ENSG00000152601	20371350	validated
mirtarbase	hsa-miR-301a-3p	MBNL1	4154	ENSG00000152601	22012620	validated
mirtarbase	hsa-miR-144-3p	MBNL1	4154	ENSG00000152601	20371350	validated
mirtarbase	hsa-miR-192-5p	MCAM	4162	ENSG00000076706	19074876	validated
mirtarbase	hsa-miR-106b-5p	MCC	4163	ENSG00000171444	21572407	validated
mirtarbase	hsa-miR-301a-3p	MCC	4163	ENSG00000171444	21572407	validated
mirtarbase	hsa-miR-582-5p	MCL1	4170	ENSG00000143384	20829195	validated
mirtarbase	hsa-miR-181a-5p	MCL1	4170	ENSG00000143384	22610076	validated
mirtarbase	hsa-miR-106b-5p	MCL1	4170	ENSG00000143384	22473208	validated
mirtarbase	hsa-miR-144-3p	MCL1	4170	ENSG00000143384	23592263	validated
mirtarbase	hsa-miR-192-5p	MCM3	4172	ENSG00000112118	19074876	validated
mirtarbase	hsa-miR-222-3p	MCM3	4172	ENSG00000112118	23622248	validated
mirtarbase	hsa-miR-18a-5p	MCM5	4174	ENSG00000100297	23622248	validated

mirtarbase	hsa-miR-192-5p	MCM6	4175	ENSG00000076003	19074876	validated
mirtarbase	hsa-miR-222-3p	MCM7	4176	ENSG00000166508	23622248	validated
mirtarbase	hsa-miR-181a-5p	CD46	4179	ENSG00000117335	17612493	validated
mirtarbase	hsa-miR-192-5p	DNAJB9	4189	ENSG00000128590	19074876	validated
mirtarbase	hsa-miR-505-3p	DNAJB9	4189	ENSG00000128590	23622248	validated
mirtarbase	hsa-miR-106b-5p	DNAJB9	4189	ENSG00000128590	24398324	validated
mirtarbase	hsa-miR-15b-5p	MDH1	4190	ENSG00000014641	23622248	validated
mirtarbase	hsa-miR-222-3p	MDH2	4191	ENSG00000146701	23622248	validated
mirtarbase	hsa-miR-222-3p	MDM2	4193	ENSG00000135679	23592263	validated
mirtarbase	hsa-miR-106b-5p	MDM2	4193	ENSG00000135679	23446348	validated
mirtarbase	hsa-miR-192-5p	MDM4	4194	ENSG00000198625	19074876	validated
mirtarbase	hsa-miR-21-5p	MDM4	4194	ENSG00000198625	18591254	validated
mirtarbase	hsa-miR-222-3p	MEA1	4201	ENSG00000124733	23622248	validated
mirtarbase	hsa-miR-212-3p	MECP2	4204	ENSG00000169057	20020497	validated
mirtarbase	hsa-miR-106b-5p	MECP2	4204	ENSG00000169057	22473208	validated
mirtarbase	hsa-miR-21-5p	MEF2A	4205	ENSG00000068305	18591254	validated
mirtarbase	hsa-miR-21-5p	MEF2C	4208	ENSG00000081189	21170291	validated
mirtarbase	hsa-miR-18a-5p	MEF2D	4209	ENSG00000116604	26356851	validated
mirtarbase	hsa-miR-21-5p	MEIS1	4211	ENSG00000143995	18591254	validated
mirtarbase	hsa-miR-192-5p	MEIS2	4212	ENSG00000134138	19074876	validated
mirtarbase	hsa-miR-192-5p	MAP3K1	4214	ENSG00000095015	16822819	validated
mirtarbase	hsa-miR-21-5p	MAP3K1	4214	ENSG00000095015	18591254	validated
mirtarbase	hsa-miR-181a-5p	MAP3K3	4215	ENSG00000198909	22942087	validated
mirtarbase	hsa-miR-106b-5p	MAP3K3	4215	ENSG00000198909	22473208	validated
mirtarbase	hsa-miR-144-3p	MAP3K4	4216	ENSG00000085511	23592263	validated
mirtarbase	hsa-miR-301a-3p	MAP3K5	4217	ENSG00000197442	27775615	validated
mirtarbase	hsa-miR-301a-3p	MEOX2	4223	ENSG00000106511	20470754	validated
mirtarbase	hsa-miR-144-3p	MET	4233	ENSG00000105976	25927670	validated
mirtarbase	hsa-miR-192-5p	MELTF	4241	ENSG00000163975	19074876	validated
mirtarbase	hsa-miR-181a-5p	MGAT5	4249	ENSG00000152127	23622248	validated
mirtarbase	hsa-miR-222-3p	KITLG	4254	ENSG00000049130	23622248	validated
mirtarbase	hsa-miR-222-3p	MGMT	4255	ENSG00000170430	24147153	validated
mirtarbase	hsa-miR-106b-5p	MICB	4277	ENSG00000234218	22473208	validated
mirtarbase	hsa-miR-18a-5p	MID1	4281	ENSG00000101871	20371350	validated
mirtarbase	hsa-miR-192-5p	MKI67	4288	ENSG00000148773	19074876	validated
mirtarbase	hsa-miR-505-3p	MKI67	4288	ENSG00000148773	23622248	validated
mirtarbase	hsa-miR-144-3p	MKLN1	4289	ENSG00000128585	23592263	validated
mirtarbase	hsa-miR-301a-3p	MAP3K9	4293	ENSG00000006432	23592263	validated
mirtarbase	hsa-miR-181a-5p	MAP3K10	4294	ENSG00000130758	22942087	validated
mirtarbase	hsa-miR-192-5p	KMT2A	4297	ENSG00000118058	23313552	validated
mirtarbase	hsa-miR-106b-5p	MLLT1	4298	ENSG00000130382	23592263	validated
mirtarbase	hsa-miR-106b-5p	AFF1	4299	ENSG00000172493	22473208	validated
mirtarbase	hsa-miR-15b-5p	AFDN	4301	ENSG00000130396	23622248	validated
mirtarbase	hsa-miR-15b-5p	MLLT6	4302	ENSG00000275023	22473208	validated
mirtarbase	hsa-miR-21-5p	MMP2	4313	ENSG00000087245	19435867	validated
mirtarbase	hsa-miR-106b-5p	MMP2	4313	ENSG00000087245	24164962	validated
mirtarbase	hsa-miR-21-5p	MMP9	4318	ENSG00000100985	19435867	validated
mirtarbase	hsa-miR-15b-5p	MMP9	4318	ENSG00000100985	25901555	validated
mirtarbase	hsa-miR-181a-5p	MMP14	4323	ENSG00000157227	22942087	validated
mirtarbase	hsa-miR-301a-3p	MOCS2	4338	ENSG00000164172	19536157	validated
mirtarbase	hsa-miR-15b-5p	MPP1	4354	ENSG00000130830	23622248	validated
mirtarbase	hsa-miR-582-3p	MPP2	4355	ENSG00000108852	26701625	validated
mirtarbase	hsa-miR-21-5p	MSH2	4436	ENSG00000095002	21078976	validated
mirtarbase	hsa-miR-326	MSH3	4437	ENSG00000113318	19883630	validated
mirtarbase	hsa-miR-106b-5p	MSH3	4437	ENSG00000113318	23313552	validated
mirtarbase	hsa-miR-192-5p	MSN	4478	ENSG00000147065	16822819	validated
mirtarbase	hsa-miR-144-3p	MSN	4478	ENSG00000147065	26701625	validated
mirtarbase	hsa-miR-192-5p	MT1F	4494	ENSG00000198417	19074876	validated
mirtarbase	hsa-miR-192-5p	MT1X	4501	ENSG00000187193	19074876	validated
mirtarbase	hsa-miR-21-5p	MT-CO2	4513	ENSG00000198712	26604791	validated
mirtarbase	hsa-miR-106b-5p	MTF1	4520	ENSG00000188786	22473208	validated
mirtarbase	hsa-miR-106b-5p	MTHFD1	4522	ENSG00000100714	23622248	validated
mirtarbase	hsa-miR-15b-5p	MTHFR	4524	ENSG00000177000	21572407	validated
mirtarbase	hsa-miR-181a-5p	MT-ND2	4536	ENSG00000198763	23622248	validated
mirtarbase	hsa-miR-106b-5p	MYO1F	4542	ENSG00000142347	23313552	validated
mirtarbase	hsa-miR-106b-5p	MTR	4548	ENSG00000116984	23622248	validated
mirtarbase	hsa-miR-21-5p	MUC1	4582	ENSG00000185499	18591254	validated
mirtarbase	hsa-miR-106b-5p	TRIM37	4591	ENSG00000108395	24398324	validated
mirtarbase	hsa-miR-301a-3p	TRIM37	4591	ENSG00000108395	24398324	validated
mirtarbase	hsa-miR-106b-5p	MXI1	4601	ENSG00000119950	17242205	validated
mirtarbase	hsa-miR-192-5p	MXI1	4601	ENSG00000119950	19074876	validated

mirtarbase	hsa-miR-192-5p	MYB	4602	ENSG00000118513	19074876	validated
mirtarbase	hsa-miR-505-3p	MYBPC1	4604	ENSG00000196091	23824327	validated
mirtarbase	hsa-miR-505-3p	MYBL2	4605	ENSG00000101057	23622248	validated
mirtarbase	hsa-miR-21-5p	MYC	4609	ENSG00000136997	19528081	validated
mirtarbase	hsa-miR-212-3p	MYC	4609	ENSG00000136997	21053104	validated
mirtarbase	hsa-miR-106b-5p	MYC	4609	ENSG00000136997	24955218	validated
mirtarbase	hsa-miR-222-3p	MYC	4609	ENSG00000136997	24510096	validated
mirtarbase	hsa-miR-582-5p	MYCN	4613	ENSG00000134323	22012620	validated
mirtarbase	hsa-miR-144-3p	MYCN	4613	ENSG00000134323	22012620	validated
mirtarbase	hsa-miR-21-5p	MYD88	4615	ENSG00000172936	23633945	validated
mirtarbase	hsa-miR-106b-5p	MYH9	4627	ENSG00000100345	24906430	validated
mirtarbase	hsa-miR-505-3p	MYH10	4628	ENSG00000133026	23622248	validated
mirtarbase	hsa-miR-301a-3p	MYH11	4629	ENSG00000276480	23824327	validated
mirtarbase	hsa-miR-106b-5p	MYL6	4637	ENSG00000092841	23622248	validated
mirtarbase	hsa-miR-18a-5p	MYLK	4638	ENSG00000065534	26233958	validated
mirtarbase	hsa-miR-582-3p	MYO1C	4641	ENSG00000197879	23592263	validated
mirtarbase	hsa-miR-106b-5p	MYO1D	4642	ENSG00000176658	23592263	validated
mirtarbase	hsa-miR-144-3p	MYO5A	4644	ENSG00000197535	23446348	validated
mirtarbase	hsa-miR-15b-5p	MYO5A	4644	ENSG00000197535	22473208	validated
mirtarbase	hsa-miR-21-5p	MYO9A	4649	ENSG00000066933	18591254	validated
mirtarbase	hsa-miR-192-5p	PPP1R12A	4659	ENSG00000058272	19074876	validated
mirtarbase	hsa-miR-106b-5p	PPP1R12B	4660	ENSG00000077157	22473208	validated
mirtarbase	hsa-miR-192-5p	NAB1	4664	ENSG00000138386	19074876	validated
mirtarbase	hsa-miR-15b-5p	NAB1	4664	ENSG00000138386	23622248	validated
mirtarbase	hsa-miR-15b-5p	HNRNPM	4670	ENSG00000099783	23622248	validated
mirtarbase	hsa-miR-582-5p	NAP1L1	4673	ENSG00000187109	20371350	validated
mirtarbase	hsa-miR-222-3p	NARS1	4677	ENSG00000134440	23622248	validated
mirtarbase	hsa-miR-106b-5p	NARS1	4677	ENSG00000134440	23446348	validated
mirtarbase	hsa-miR-301a-3p	NARS1	4677	ENSG00000134440	23824327	validated
mirtarbase	hsa-miR-106b-5p	NBL1	4681	ENSG00000158747	26701625	validated
mirtarbase	hsa-miR-106b-5p	NUBP1	4682	ENSG00000103274	17242205	validated
mirtarbase	hsa-miR-192-5p	NBN	4683	ENSG00000104320	19074876	validated
mirtarbase	hsa-miR-192-5p	NCBP1	4686	ENSG00000136937	19074876	validated
mirtarbase	hsa-miR-15b-5p	SEPTIN2	4735	ENSG00000168385	23622248	validated
mirtarbase	hsa-miR-181a-5p	SEPTIN2	4735	ENSG00000168385	23446348	validated
mirtarbase	hsa-miR-106b-5p	SEPTIN2	4735	ENSG00000168385	22473208	validated
mirtarbase	hsa-miR-21-5p	SEPTIN2	4735	ENSG00000168385	22473208	validated
mirtarbase	hsa-miR-18a-5p	NEDD9	4739	ENSG00000111859	22308110	validated
mirtarbase	hsa-miR-106b-5p	NEFH	4744	ENSG00000100285	17242205	validated
mirtarbase	hsa-miR-192-5p	NEK1	4750	ENSG00000137601	19074876	validated
mirtarbase	hsa-miR-21-5p	NEK1	4750	ENSG00000137601	18591254	validated
mirtarbase	hsa-miR-18a-5p	NEO1	4756	ENSG00000067141	24657544	validated
mirtarbase	hsa-miR-301a-3p	NF2	4771	ENSG00000186575	23824327	validated
mirtarbase	hsa-miR-326	NF2	4771	ENSG00000186575	26701625	validated
mirtarbase	hsa-miR-21-5p	NFIA	4774	ENSG00000162599	24980967	validated
mirtarbase	hsa-miR-301a-3p	NFE2L1	4779	ENSG00000082641	23824327	validated
mirtarbase	hsa-miR-144-3p	NFE2L2	4780	ENSG00000116044	23236440	validated
mirtarbase	hsa-miR-192-5p	NFIB	4781	ENSG00000147862	19074876	validated
mirtarbase	hsa-miR-301a-3p	NFIB	4781	ENSG00000147862	23592263	validated
mirtarbase	hsa-miR-106b-5p	NFIB	4781	ENSG00000147862	23592263	validated
mirtarbase	hsa-miR-15b-5p	NFIC	4782	ENSG00000141905	23592263	validated
mirtarbase	hsa-miR-296-5p	NFIC	4782	ENSG00000141905	20371350	validated
mirtarbase	hsa-miR-296-5p	NFIX	4784	ENSG00000008441	27292025	validated
mirtarbase	hsa-miR-21-5p	NFKB1	4790	ENSG00000109320	21081469	validated
mirtarbase	hsa-miR-192-5p	NFYA	4800	ENSG00000001167	19074876	validated
mirtarbase	hsa-miR-181a-5p	NFYB	4801	ENSG00000120837	20371350	validated
mirtarbase	hsa-miR-296-5p	NGFR	4804	ENSG00000064300	27927008	validated
mirtarbase	hsa-miR-106b-5p	NID1	4811	ENSG00000116962	23622248	validated
mirtarbase	hsa-miR-192-5p	NID1	4811	ENSG00000116962	25857602	validated
mirtarbase	hsa-miR-21-5p	NKTR	4820	ENSG00000114857	20371350	validated
mirtarbase	hsa-miR-192-5p	NKX3-1	4824	ENSG00000167034	19074876	validated
mirtarbase	hsa-miR-301a-3p	CNOT4	4850	ENSG00000080802	23592263	validated
mirtarbase	hsa-miR-106b-5p	CNOT4	4850	ENSG00000080802	23592263	validated
mirtarbase	hsa-miR-326	NOTCH1	4851	ENSG00000148400	19955368	validated
mirtarbase	hsa-miR-144-3p	NOTCH1	4851	ENSG00000148400	21285251	validated
mirtarbase	hsa-miR-181a-5p	NOTCH1	4851	ENSG00000148400	22916024	validated
mirtarbase	hsa-miR-296-5p	NOTCH1	4851	ENSG00000148400	26500043	validated
mirtarbase	hsa-miR-326	NOTCH2	4853	ENSG00000134250	19955368	validated
mirtarbase	hsa-miR-181a-5p	NOTCH2	4853	ENSG00000134250	20371350	validated
mirtarbase	hsa-miR-15b-5p	NOTCH2	4853	ENSG00000134250	22473208	validated
mirtarbase	hsa-miR-106b-5p	NPAT	4863	ENSG00000149308	22473208	validated

mirtarbase	hsa-miR-15b-5p	NPM1	4869	ENSG00000181163	23622248	validated
mirtarbase	hsa-miR-144-3p	NPM1	4869	ENSG00000181163	26701625	validated
mirtarbase	hsa-miR-301a-3p	NPTX1	4884	ENSG00000171246	20371350	validated
mirtarbase	hsa-miR-505-3p	NPTX1	4884	ENSG00000171246	23824327	validated
mirtarbase	hsa-miR-582-5p	SLC11A2	4891	ENSG00000110911	23592263	validated
mirtarbase	hsa-miR-181a-5p	NRAS	4893	ENSG00000213281	27517749	validated
mirtarbase	hsa-miR-106b-5p	YBX1	4904	ENSG00000065978	23622248	validated
mirtarbase	hsa-miR-192-5p	NSF	4905	ENSG00000276262	19074876	validated
mirtarbase	hsa-miR-21-5p	NTF3	4908	ENSG00000185652	22019057	validated
mirtarbase	hsa-miR-21-5p	DDR2	4921	ENSG00000162733	18591254	validated
mirtarbase	hsa-miR-192-5p	NUCB2	4925	ENSG00000070081	19074876	validated
mirtarbase	hsa-miR-106b-5p	NUP98	4928	ENSG00000110713	22473208	validated
mirtarbase	hsa-miR-326	NUP98	4928	ENSG00000110713	19536157	validated
mirtarbase	hsa-miR-222-3p	OAZ2	4947	ENSG00000180304	23622248	validated
mirtarbase	hsa-miR-181a-5p	OCA2	4948	ENSG00000104044	23622248	validated
mirtarbase	hsa-miR-106b-5p	OCRL	4952	ENSG00000122126	22473208	validated
mirtarbase	hsa-miR-15b-5p	OCRL	4952	ENSG00000122126	23313552	validated
mirtarbase	hsa-miR-192-5p	ODC1	4953	ENSG00000115758	16822819	validated
mirtarbase	hsa-miR-301a-3p	OMD	4958	ENSG00000127083	23824327	validated
mirtarbase	hsa-miR-21-5p	OLR1	4973	ENSG00000173391	18591254	validated
mirtarbase	hsa-miR-192-5p	OPA1	4976	ENSG00000198836	19074876	validated
mirtarbase	hsa-miR-21-5p	TNFRSF11B	4982	ENSG00000164761	20371350	validated
mirtarbase	hsa-miR-181a-5p	TNFRSF11B	4982	ENSG00000164761	22942087	validated
mirtarbase	hsa-miR-106b-5p	OPRK1	4986	ENSG00000082556	17242205	validated
mirtarbase	hsa-miR-181a-5p	SIX6	4990	ENSG00000184302	17612493	validated
mirtarbase	hsa-miR-192-5p	ORC1	4998	ENSG00000085840	19074876	validated
mirtarbase	hsa-miR-301a-3p	ORC1	4998	ENSG00000085840	27292025	validated
mirtarbase	hsa-miR-505-3p	ORC2	4999	ENSG00000115942	23622248	validated
mirtarbase	hsa-miR-15b-5p	ORC4	5000	ENSG00000115947	24398324	validated
mirtarbase	hsa-miR-21-5p	ORC4	5000	ENSG00000115947	22012620	validated
mirtarbase	hsa-miR-192-5p	ORC4	5000	ENSG00000115947	23824327	validated
mirtarbase	hsa-miR-505-3p	OSBP	5007	ENSG00000110048	23622248	validated
mirtarbase	hsa-miR-301a-3p	OSBP	5007	ENSG00000110048	23592263	validated
mirtarbase	hsa-miR-181a-5p	OTX2	5015	ENSG00000165588	17612493	validated
mirtarbase	hsa-miR-192-5p	OVOL1	5017	ENSG00000172818	19074876	validated
mirtarbase	hsa-miR-21-5p	OXTR	5021	ENSG00000180914	26273428	validated
mirtarbase	hsa-miR-222-3p	PEBP1	5037	ENSG00000089220	23622248	validated
mirtarbase	hsa-miR-181a-5p	PEBP1	5037	ENSG00000089220	22473208	validated
mirtarbase	hsa-miR-15b-5p	FURIN	5045	ENSG00000140564	23592263	validated
mirtarbase	hsa-miR-192-5p	PAFAH1B1	5048	ENSG00000007168	19074876	validated
mirtarbase	hsa-miR-18a-5p	PAFAH1B1	5048	ENSG00000007168	20371350	validated
mirtarbase	hsa-miR-106b-5p	PAFAH1B1	5048	ENSG00000007168	22473208	validated
mirtarbase	hsa-miR-15b-5p	PAFAH1B1	5048	ENSG00000007168	22473208	validated
mirtarbase	hsa-miR-15b-5p	PAFAH1B2	5049	ENSG00000168092	20371350	validated
mirtarbase	hsa-miR-301a-3p	PAFAH1B2	5049	ENSG00000168092	22012620	validated
mirtarbase	hsa-miR-222-3p	PAFAH1B2	5049	ENSG00000168092	21572407	validated
mirtarbase	hsa-miR-301a-3p	SERPINE1	5054	ENSG00000106366	21175428	validated
mirtarbase	hsa-miR-192-5p	SERPINE1	5054	ENSG00000106366	27216198	validated
mirtarbase	hsa-miR-15b-5p	PAK2	5062	ENSG00000180370	21572407	validated
mirtarbase	hsa-miR-582-5p	PAK3	5063	ENSG00000077264	23824327	validated
mirtarbase	hsa-miR-181a-5p	PRKN	5071	ENSG00000185345	27281615	validated
mirtarbase	hsa-miR-192-5p	PARN	5073	ENSG00000274829	19074876	validated
mirtarbase	hsa-miR-192-5p	PAWR	5074	ENSG00000177425	19074876	validated
mirtarbase	hsa-miR-21-5p	PBX1	5087	ENSG00000185630	18591254	validated
mirtarbase	hsa-miR-326	PBX2	5089	ENSG00000224952	23622248	validated
mirtarbase	hsa-miR-222-3p	PBX2	5089	ENSG00000224952	23622248	validated
mirtarbase	hsa-miR-181a-5p	PBX3	5090	ENSG00000167081	22251480	validated
mirtarbase	hsa-miR-144-3p	PBX3	5090	ENSG00000167081	28111340	validated
mirtarbase	hsa-miR-21-5p	PCBP1	5093	ENSG00000169564	19253296	validated
mirtarbase	hsa-miR-222-3p	PCBP2	5094	ENSG00000197111	23622248	validated
mirtarbase	hsa-miR-106b-5p	PCBP2	5094	ENSG00000197111	23446348	validated
mirtarbase	hsa-miR-582-5p	PCCB	5096	ENSG00000114054	20371350	validated
mirtarbase	hsa-miR-144-3p	PCCB	5096	ENSG00000114054	20371350	validated
mirtarbase	hsa-miR-192-5p	PCDH7	5099	ENSG00000169851	19074876	validated
mirtarbase	hsa-miR-18a-5p	PCDH9	5101	ENSG00000184226	22100165	validated
mirtarbase	hsa-miR-15b-5p	PCMT1	5110	ENSG00000120265	22473208	validated
mirtarbase	hsa-miR-18a-5p	PCNA	5111	ENSG00000132646	23622248	validated
mirtarbase	hsa-miR-222-3p	PCNT	5116	ENSG00000160299	23622248	validated
mirtarbase	hsa-miR-15b-5p	CDK17	5128	ENSG00000059758	22473208	validated
mirtarbase	hsa-miR-222-3p	CDK18	5129	ENSG00000117266	23622248	validated
mirtarbase	hsa-miR-15b-5p	PDCD1	5133	ENSG00000276977	21572407	validated

mirtarbase	hsa-miR-192-5p	PDE2A	5138	ENSG00000186642	16822819	validated
mirtarbase	hsa-miR-326	PDE3A	5139	ENSG00000172572	23824327	validated
mirtarbase	hsa-miR-106b-5p	PDE4C	5143	ENSG00000105650	23313552	validated
mirtarbase	hsa-miR-18a-5p	PDE4D	5144	ENSG00000113448	20371350	validated
mirtarbase	hsa-miR-15b-5p	PDE4D	5144	ENSG00000113448	23592263	validated
mirtarbase	hsa-miR-326	PDE1B	5153	ENSG00000123360	19536157	validated
mirtarbase	hsa-miR-106b-5p	PDGFB	5155	ENSG00000100311	23592263	validated
mirtarbase	hsa-miR-181a-5p	PDGFRA	5156	ENSG00000134853	22942087	validated
mirtarbase	hsa-miR-326	PDHA1	5160	ENSG00000131828	23622248	validated
mirtarbase	hsa-miR-21-5p	PDHA2	5161	ENSG00000163114	19253296	validated
mirtarbase	hsa-miR-106b-5p	PDHB	5162	ENSG00000168291	23313552	validated
mirtarbase	hsa-miR-582-5p	PDK1	5163	ENSG00000152256	27418678	validated
mirtarbase	hsa-miR-181a-5p	PDK3	5165	ENSG00000067992	19536157	validated
mirtarbase	hsa-miR-106b-5p	PDPK1	5170	ENSG00000140992	23446348	validated
mirtarbase	hsa-miR-192-5p	GATB	5188	ENSG00000059691	19074876	validated
mirtarbase	hsa-miR-15b-5p	PEX12	5193	ENSG00000108733	22473208	validated
mirtarbase	hsa-miR-15b-5p	PEX13	5194	ENSG00000162928	22473208	validated
mirtarbase	hsa-miR-301a-3p	PEX13	5194	ENSG00000162928	22012620	validated
mirtarbase	hsa-miR-212-3p	PFAS	5198	ENSG00000178921	23592263	validated
mirtarbase	hsa-miR-181a-5p	ATP8B1	5205	ENSG00000081923	22012620	validated
mirtarbase	hsa-miR-21-5p	PFKFB2	5208	ENSG00000123836	18591254	validated
mirtarbase	hsa-miR-181a-5p	PFKFB2	5208	ENSG00000123836	23622248	validated
mirtarbase	hsa-miR-106b-5p	PFKFB2	5208	ENSG00000123836	22100165	validated
mirtarbase	hsa-miR-106b-5p	PFKP	5214	ENSG00000067057	22473208	validated
mirtarbase	hsa-miR-222-3p	PFN1	5216	ENSG00000108518	23622248	validated
mirtarbase	hsa-miR-192-5p	CDK14	5218	ENSG00000058091	19074876	validated
mirtarbase	hsa-miR-144-3p	PGAM1	5223	ENSG00000171314	26701625	validated
mirtarbase	hsa-miR-15b-5p	PGD	5226	ENSG00000142657	23622248	validated
mirtarbase	hsa-miR-181a-5p	PGD	5226	ENSG00000142657	23622248	validated
mirtarbase	hsa-miR-582-3p	PGGT1B	5229	ENSG00000164219	23295946	validated
mirtarbase	hsa-miR-582-5p	PGGT1B	5229	ENSG00000164219	23295946	validated
mirtarbase	hsa-miR-192-5p	PGM3	5238	ENSG00000013375	19074876	validated
mirtarbase	hsa-miR-181a-5p	PGR	5241	ENSG00000082175	22492871	validated
mirtarbase	hsa-miR-21-5p	ABCB1	5243	ENSG00000085563	27040946	validated
mirtarbase	hsa-miR-15b-5p	PHKA1	5255	ENSG00000067177	23446348	validated
mirtarbase	hsa-miR-21-5p	SERPINI1	5274	ENSG00000163536	18591254	validated
mirtarbase	hsa-miR-326	PIGA	5277	ENSG00000165195	23622248	validated
mirtarbase	hsa-miR-301a-3p	PIGA	5277	ENSG00000165195	22012620	validated
mirtarbase	hsa-miR-21-5p	PIK3C2A	5286	ENSG00000011405	18591254	validated
mirtarbase	hsa-miR-15b-5p	PIK3C2B	5287	ENSG00000133056	23622248	validated
mirtarbase	hsa-miR-192-5p	PIM1	5292	ENSG00000137193	19074876	validated
mirtarbase	hsa-miR-15b-5p	PIM1	5292	ENSG00000137193	23446348	validated
mirtarbase	hsa-miR-582-5p	PIK3CD	5293	ENSG00000171608	26701625	validated
mirtarbase	hsa-miR-21-5p	PIK3R1	5295	ENSG00000145675	18591254	validated
mirtarbase	hsa-miR-15b-5p	PIK3R1	5295	ENSG00000145675	23446348	validated
mirtarbase	hsa-miR-18a-5p	PI4KB	5298	ENSG00000143393	23622248	validated
mirtarbase	hsa-miR-296-5p	PIN1	5300	ENSG00000127445	24915000	validated
mirtarbase	hsa-miR-106b-5p	PIP4K2A	5305	ENSG00000150867	22473208	validated
mirtarbase	hsa-miR-106b-5p	PITPNA	5306	ENSG00000174238	23592263	validated
mirtarbase	hsa-miR-106b-5p	PKD2	5311	ENSG00000118762	20709030	validated
mirtarbase	hsa-miR-21-5p	PKD2	5311	ENSG00000118762	18591254	validated
mirtarbase	hsa-miR-326	PKM	5315	ENSG00000067225	20667897	validated
mirtarbase	hsa-miR-222-3p	PKM	5315	ENSG00000067225	23622248	validated
mirtarbase	hsa-miR-21-5p	PKNOX1	5316	ENSG00000160199	18591254	validated
mirtarbase	hsa-miR-106b-5p	PKNOX1	5316	ENSG00000160199	23446348	validated
mirtarbase	hsa-miR-222-3p	PKP2	5318	ENSG00000057294	23622248	validated
mirtarbase	hsa-miR-144-3p	PLAG1	5324	ENSG00000181690	19347935	validated
mirtarbase	hsa-miR-15b-5p	PLAG1	5324	ENSG00000181690	22473208	validated
mirtarbase	hsa-miR-192-5p	PLAGL2	5326	ENSG00000126003	19074876	validated
mirtarbase	hsa-miR-106b-5p	PLAGL2	5326	ENSG00000126003	22473208	validated
mirtarbase	hsa-miR-212-3p	PLAGL2	5326	ENSG00000126003	23592263	validated
mirtarbase	hsa-miR-21-5p	PLAT	5327	ENSG00000104368	21131358	validated
mirtarbase	hsa-miR-192-5p	PLAU	5328	ENSG00000122861	19074876	validated
mirtarbase	hsa-miR-21-5p	PLD1	5337	ENSG00000075651	18591254	validated
mirtarbase	hsa-miR-296-5p	PLK1	5347	ENSG00000166851	26549165	validated
mirtarbase	hsa-miR-192-5p	PLOD1	5351	ENSG00000083444	19074876	validated
mirtarbase	hsa-miR-15b-5p	PLRG1	5356	ENSG00000171566	23446348	validated
mirtarbase	hsa-miR-106b-5p	PLRG1	5356	ENSG00000171566	21572407	validated
mirtarbase	hsa-miR-106b-5p	PLS1	5357	ENSG00000120756	17242205	validated
mirtarbase	hsa-miR-144-3p	PLS1	5357	ENSG00000120756	21572407	validated
mirtarbase	hsa-miR-192-5p	PLS3	5358	ENSG00000102024	19074876	validated

mirtarbase	hsa-miR-106b-5p	PLXNA1	5361	ENSG00000114554	22473208	validated
mirtarbase	hsa-miR-181a-5p	PMAIP1	5366	ENSG00000141682	22473208	validated
mirtarbase	hsa-miR-106b-5p	PMAIP1	5366	ENSG00000141682	27292025	validated
mirtarbase	hsa-miR-222-3p	EXOSC10	5394	ENSG00000171824	23622248	validated
mirtarbase	hsa-miR-106b-5p	PRRX1	5396	ENSG00000116132	26617763	validated
mirtarbase	hsa-miR-192-5p	UBL3	5412	ENSG00000122042	19074876	validated
mirtarbase	hsa-miR-181a-5p	UBL3	5412	ENSG00000122042	22815788	validated
mirtarbase	hsa-miR-192-5p	PODXL	5420	ENSG00000128567	19074876	validated
mirtarbase	hsa-miR-222-3p	POLD2	5425	ENSG00000106628	23622248	validated
mirtarbase	hsa-miR-222-3p	POLE	5426	ENSG00000177084	23622248	validated
mirtarbase	hsa-miR-505-3p	POLG	5428	ENSG00000140521	23824327	validated
mirtarbase	hsa-miR-505-3p	POLR2D	5433	ENSG00000144231	23592263	validated
mirtarbase	hsa-miR-301a-3p	POLR2D	5433	ENSG00000144231	23824327	validated
mirtarbase	hsa-miR-296-5p	POLR2E	5434	ENSG00000099817	22012620	validated
mirtarbase	hsa-miR-15b-5p	POLR2E	5434	ENSG00000099817	26701625	validated
mirtarbase	hsa-miR-106b-5p	PON2	5445	ENSG00000105854	17242205	validated
mirtarbase	hsa-miR-18a-5p	PON2	5445	ENSG00000105854	23622248	validated
mirtarbase	hsa-miR-15b-5p	POU2AF1	5450	ENSG00000110777	22473208	validated
mirtarbase	hsa-miR-192-5p	POU2F1	5451	ENSG00000143190	19074876	validated
mirtarbase	hsa-miR-15b-5p	POU2F1	5451	ENSG00000143190	23622248	validated
mirtarbase	hsa-miR-144-3p	POU2F1	5451	ENSG00000143190	20371350	validated
mirtarbase	hsa-miR-192-5p	PPARG	5468	ENSG00000132170	19074876	validated
mirtarbase	hsa-miR-192-5p	PPAT	5471	ENSG00000128059	19074876	validated
mirtarbase	hsa-miR-326	CTSA	5476	ENSG00000064601	26701625	validated
mirtarbase	hsa-miR-106b-5p	PPIA	5478	ENSG00000196262	23622248	validated
mirtarbase	hsa-miR-18a-5p	PPIA	5478	ENSG00000196262	23622248	validated
mirtarbase	hsa-miR-301a-3p	PPIB	5479	ENSG00000166794	23622248	validated
mirtarbase	hsa-miR-144-3p	PPIC	5480	ENSG00000168938	23592263	validated
mirtarbase	hsa-miR-192-5p	PPM1A	5494	ENSG00000100614	19074876	validated
mirtarbase	hsa-miR-15b-5p	PPM1A	5494	ENSG00000100614	20371350	validated
mirtarbase	hsa-miR-106b-5p	PPM1B	5495	ENSG00000138032	23622248	validated
mirtarbase	hsa-miR-192-5p	PPP1CA	5499	ENSG00000172531	19074876	validated
mirtarbase	hsa-miR-192-5p	PPP1CB	5500	ENSG00000213639	19074876	validated
mirtarbase	hsa-miR-18a-5p	PPP1CB	5500	ENSG00000213639	20371350	validated
mirtarbase	hsa-miR-505-3p	PPP1CB	5500	ENSG00000213639	23622248	validated
mirtarbase	hsa-miR-15b-5p	PPP1CB	5500	ENSG00000213639	23622248	validated
mirtarbase	hsa-miR-15b-5p	PPP1CC	5501	ENSG00000186298	23622248	validated
mirtarbase	hsa-miR-582-5p	PPP1CC	5501	ENSG00000186298	23592263	validated
mirtarbase	hsa-miR-222-3p	PPP2R1A	5518	ENSG00000105568	23622248	validated
mirtarbase	hsa-miR-144-3p	PPP2R2A	5520	ENSG00000221914	20371350	validated
mirtarbase	hsa-miR-326	PTPA	5524	ENSG00000119383	23622248	validated
mirtarbase	hsa-miR-15b-5p	PPP2R5C	5527	ENSG00000078304	21572407	validated
mirtarbase	hsa-miR-582-5p	PPP2R5E	5529	ENSG00000154001	23592263	validated
mirtarbase	hsa-miR-181a-5p	PPP2R5E	5529	ENSG00000154001	23592263	validated
mirtarbase	hsa-miR-181a-5p	PPP3CA	5530	ENSG00000138814	23677691	validated
mirtarbase	hsa-miR-106b-5p	PPP4C	5531	ENSG00000149923	23622248	validated
mirtarbase	hsa-miR-106b-5p	PPP3R1	5534	ENSG00000221823	22473208	validated
mirtarbase	hsa-miR-222-3p	PPP6C	5537	ENSG00000119414	23622248	validated
mirtarbase	hsa-miR-106b-5p	PPP6C	5537	ENSG00000119414	22473208	validated
mirtarbase	hsa-miR-144-3p	PPP6C	5537	ENSG00000119414	23446348	validated
mirtarbase	hsa-miR-15b-5p	PPP6C	5537	ENSG00000119414	22473208	validated
mirtarbase	hsa-miR-15b-5p	PPT1	5538	ENSG00000131238	23622248	validated
mirtarbase	hsa-miR-181a-5p	SRGN	5552	ENSG00000122862	22473208	validated
mirtarbase	hsa-miR-192-5p	PRIM1	5557	ENSG00000198056	19074876	validated
mirtarbase	hsa-miR-106b-5p	PRIM1	5557	ENSG00000198056	23446348	validated
mirtarbase	hsa-miR-582-5p	PRKAA1	5562	ENSG00000132356	23592263	validated
mirtarbase	hsa-miR-301a-3p	PRKAA1	5562	ENSG00000132356	23446348	validated
mirtarbase	hsa-miR-15b-5p	PRKAA1	5562	ENSG00000132356	21572407	validated
mirtarbase	hsa-miR-192-5p	PRKAA2	5563	ENSG00000162409	19074876	validated
mirtarbase	hsa-miR-21-5p	PRKAB2	5565	ENSG00000131791	18591254	validated
mirtarbase	hsa-miR-106b-5p	PRKACB	5567	ENSG00000142875	21572407	validated
mirtarbase	hsa-miR-192-5p	PRKAR1A	5573	ENSG00000108946	19074876	validated
mirtarbase	hsa-miR-106b-5p	PRKAR1A	5573	ENSG00000108946	22012620	validated
mirtarbase	hsa-miR-15b-5p	PRKAR2A	5576	ENSG00000114302	23592263	validated
mirtarbase	hsa-miR-505-3p	PRKCA	5578	ENSG00000154229	23622248	validated
mirtarbase	hsa-miR-106b-5p	PRKCB	5579	ENSG00000166501	24906430	validated
mirtarbase	hsa-miR-15b-5p	PRKCD	5580	ENSG00000163932	22473208	validated
mirtarbase	hsa-miR-181a-5p	PRKCD	5580	ENSG00000163932	22473208	validated
mirtarbase	hsa-miR-21-5p	PRKCE	5581	ENSG00000171132	18591254	validated
mirtarbase	hsa-miR-15b-5p	MAPK1	5594	ENSG00000100030	23622248	validated
mirtarbase	hsa-miR-181a-5p	MAPK1	5594	ENSG00000100030	23911747	validated

mirtarbase	hsa-miR-106b-5p	MAPK1	5594	ENSG00000100030	22473208	validated
mirtarbase	hsa-miR-301a-3p	MAPK1	5594	ENSG00000100030	24398324	validated
mirtarbase	hsa-miR-106b-5p	MAPK9	5601	ENSG00000050748	21283765	validated
mirtarbase	hsa-miR-192-5p	MAPK9	5601	ENSG00000050748	19074876	validated
mirtarbase	hsa-miR-18a-5p	MAPK13	5603	ENSG00000156711	23446348	validated
mirtarbase	hsa-miR-181a-5p	MAP2K1	5604	ENSG00000169032	20371350	validated
mirtarbase	hsa-miR-21-5p	MAP2K3	5606	ENSG00000034152	24112539	validated
mirtarbase	hsa-miR-15b-5p	MAP2K3	5606	ENSG00000034152	26701625	validated
mirtarbase	hsa-miR-15b-5p	EIF2AK2	5610	ENSG00000055332	23622248	validated
mirtarbase	hsa-miR-192-5p	DNAJC3	5611	ENSG00000102580	19074876	validated
mirtarbase	hsa-miR-181a-5p	PRLR	5618	ENSG00000113494	17612493	validated
mirtarbase	hsa-miR-192-5p	PRNP	5621	ENSG00000171867	19074876	validated
mirtarbase	hsa-miR-106b-5p	PRNP	5621	ENSG00000171867	22473208	validated
mirtarbase	hsa-miR-301a-3p	PRNP	5621	ENSG00000171867	22100165	validated
mirtarbase	hsa-miR-181a-5p	PROX1	5629	ENSG00000117707	20558617	validated
mirtarbase	hsa-miR-15b-5p	PRPS1	5631	ENSG00000147224	23622248	validated
mirtarbase	hsa-miR-192-5p	PRPS2	5634	ENSG00000101911	19074876	validated
mirtarbase	hsa-miR-106b-5p	PRRG1	5638	ENSG00000130962	17242205	validated
mirtarbase	hsa-miR-192-5p	LGMN	5641	ENSG00000100600	19074876	validated
mirtarbase	hsa-miR-15b-5p	PSKH1	5681	ENSG00000159792	22473208	validated
mirtarbase	hsa-miR-212-3p	PSMA2	5683	ENSG00000106588	23446348	validated
mirtarbase	hsa-miR-106b-5p	PSMA3	5684	ENSG00000100567	23622248	validated
mirtarbase	hsa-miR-18a-5p	PSMB5	5693	ENSG00000100804	20371350	validated
mirtarbase	hsa-miR-301a-3p	PSMB5	5693	ENSG00000100804	23824327	validated
mirtarbase	hsa-miR-15b-5p	PSMB5	5693	ENSG00000100804	22473208	validated
mirtarbase	hsa-miR-192-5p	PSMB5	5693	ENSG00000100804	27418678	validated
mirtarbase	hsa-miR-222-3p	PSMC4	5704	ENSG00000013275	23622248	validated
mirtarbase	hsa-miR-15b-5p	PSMD7	5713	ENSG00000103035	23622248	validated
mirtarbase	hsa-miR-21-5p	PSMD9	5715	ENSG00000110801	26500453	validated
mirtarbase	hsa-miR-505-3p	PSMD10	5716	ENSG00000101843	26394032	validated
mirtarbase	hsa-miR-15b-5p	PTBP1	5725	ENSG00000011304	23622248	validated
mirtarbase	hsa-miR-212-3p	PTCH1	5727	ENSG00000185920	22357618	validated
mirtarbase	hsa-miR-18a-5p	PTEN	5728	ENSG00000171862	20008935	validated
mirtarbase	hsa-miR-106b-5p	PTEN	5728	ENSG00000171862	21283765	validated
mirtarbase	hsa-miR-144-3p	PTEN	5728	ENSG00000171862	23125220	validated
mirtarbase	hsa-miR-301a-3p	PTEN	5728	ENSG00000171862	24315818	validated
mirtarbase	hsa-miR-181a-5p	PTEN	5728	ENSG00000171862	26323677	validated
mirtarbase	hsa-miR-106b-5p	PTGER4	5734	ENSG00000171522	22473208	validated
mirtarbase	hsa-miR-21-5p	PTGFR	5737	ENSG00000122420	18591254	validated
mirtarbase	hsa-miR-106b-5p	PTGFRN	5738	ENSG00000134247	23592263	validated
mirtarbase	hsa-miR-106b-5p	PTGIS	5740	ENSG00000124212	23313552	validated
mirtarbase	hsa-miR-181a-5p	PTGS2	5743	ENSG00000073756	17612493	validated
mirtarbase	hsa-miR-144-3p	PTGS2	5743	ENSG00000073756	26959737	validated
mirtarbase	hsa-miR-21-5p	PTK2	5747	ENSG00000169398	18591254	validated
mirtarbase	hsa-miR-106b-5p	TWF1	5756	ENSG00000151239	22473208	validated
mirtarbase	hsa-miR-18a-5p	TWF1	5756	ENSG00000151239	21572407	validated
mirtarbase	hsa-miR-106b-5p	QSOX1	5768	ENSG00000116260	23446348	validated
mirtarbase	hsa-miR-301a-3p	QSOX1	5768	ENSG00000116260	27292025	validated
mirtarbase	hsa-miR-21-5p	PTPN3	5774	ENSG00000070159	18591254	validated
mirtarbase	hsa-miR-106b-5p	PTPN4	5775	ENSG00000088179	22473208	validated
mirtarbase	hsa-miR-301a-3p	PTPN4	5775	ENSG00000088179	24398324	validated
mirtarbase	hsa-miR-181a-5p	PTPN11	5781	ENSG00000179295	17382377	validated
mirtarbase	hsa-miR-21-5p	PTPN14	5784	ENSG00000152104	20048743	validated
mirtarbase	hsa-miR-15b-5p	PTPRD	5789	ENSG00000153707	21572407	validated
mirtarbase	hsa-miR-192-5p	PTPRE	5791	ENSG00000132334	19074876	validated
mirtarbase	hsa-miR-301a-3p	PTPRG	5793	ENSG00000144724	21572407	validated
mirtarbase	hsa-miR-15b-5p	PTPRJ	5795	ENSG00000149177	22473208	validated
mirtarbase	hsa-miR-505-3p	PTPRJ	5795	ENSG00000149177	23824327	validated
mirtarbase	hsa-miR-181a-5p	PTPRZ1	5803	ENSG00000106278	17612493	validated
mirtarbase	hsa-miR-192-5p	PTS	5805	ENSG00000150787	19074876	validated
mirtarbase	hsa-miR-21-5p	PTX3	5806	ENSG00000163661	21131358	validated
mirtarbase	hsa-miR-192-5p	RAD1	5810	ENSG00000113456	19074876	validated
mirtarbase	hsa-miR-15b-5p	PURA	5813	ENSG00000185129	22835829	validated
mirtarbase	hsa-miR-106b-5p	PURA	5813	ENSG00000185129	22835829	validated
mirtarbase	hsa-miR-192-5p	PURA	5813	ENSG00000185129	19074876	validated
mirtarbase	hsa-miR-21-5p	PURA	5813	ENSG00000185129	18591254	validated
mirtarbase	hsa-miR-21-5p	PURB	5814	ENSG00000146676	18591254	validated
mirtarbase	hsa-miR-106b-5p	PURB	5814	ENSG00000146676	22473208	validated
mirtarbase	hsa-miR-582-5p	PURB	5814	ENSG00000146676	21572407	validated
mirtarbase	hsa-miR-144-3p	PURB	5814	ENSG00000146676	21572407	validated
mirtarbase	hsa-miR-181a-5p	PURB	5814	ENSG00000146676	22473208	validated

mirtarbase	hsa-miR-106b-5p	PVR	5817	ENSG00000073008	21572407	validated
mirtarbase	hsa-miR-21-5p	ABCD3	5825	ENSG00000117528	18591254	validated
mirtarbase	hsa-miR-15b-5p	PXMP2	5827	ENSG00000176894	23622248	validated
mirtarbase	hsa-miR-212-3p	PXN	5829	ENSG00000089159	26693054	validated
mirtarbase	hsa-miR-15b-5p	QARS1	5859	ENSG00000172053	23622248	validated
mirtarbase	hsa-miR-582-5p	QDPR	5860	ENSG00000151552	23592263	validated
mirtarbase	hsa-miR-192-5p	RAB1A	5861	ENSG00000138069	19074876	validated
mirtarbase	hsa-miR-15b-5p	RAB1A	5861	ENSG00000138069	26023735	validated
mirtarbase	hsa-miR-192-5p	RAB2A	5862	ENSG00000104388	16822819	validated
mirtarbase	hsa-miR-192-5p	RAB3B	5865	ENSG00000169213	19074876	validated
mirtarbase	hsa-miR-18a-5p	RAB5A	5868	ENSG00000144566	20371350	validated
mirtarbase	hsa-miR-106b-5p	RAB5B	5869	ENSG00000111540	22473208	validated
mirtarbase	hsa-miR-301a-3p	RAB5B	5869	ENSG00000111540	23592263	validated
mirtarbase	hsa-miR-212-3p	RAB5B	5869	ENSG00000111540	21572407	validated
mirtarbase	hsa-miR-21-5p	RAB6A	5870	ENSG00000175582	18591254	validated
mirtarbase	hsa-miR-15b-5p	MAP4K2	5871	ENSG00000168067	21572407	validated
mirtarbase	hsa-miR-192-5p	RAB27A	5873	ENSG00000069974	19074876	validated
mirtarbase	hsa-miR-582-3p	RAB27A	5873	ENSG00000069974	23295946	validated
mirtarbase	hsa-miR-582-5p	RAB27A	5873	ENSG00000069974	23295946	validated
mirtarbase	hsa-miR-18a-5p	RAB5C	5878	ENSG00000108774	23592263	validated
mirtarbase	hsa-miR-582-5p	RAC1	5879	ENSG00000136238	23592263	validated
mirtarbase	hsa-miR-144-3p	RAC1	5879	ENSG00000136238	23592263	validated
mirtarbase	hsa-miR-192-5p	RAD21	5885	ENSG00000164754	19074876	validated
mirtarbase	hsa-miR-106b-5p	RAD23B	5887	ENSG00000119318	23622248	validated
mirtarbase	hsa-miR-15b-5p	RAD23B	5887	ENSG00000119318	22473208	validated
mirtarbase	hsa-miR-192-5p	RAD51	5888	ENSG00000051180	19074876	validated
mirtarbase	hsa-miR-301a-3p	RAG1	5896	ENSG00000166349	22012620	validated
mirtarbase	hsa-miR-181a-5p	RALA	5898	ENSG00000006451	22442671	validated
mirtarbase	hsa-miR-106b-5p	RAN	5901	ENSG00000132341	22473208	validated
mirtarbase	hsa-miR-301a-3p	RAN	5901	ENSG00000132341	23446348	validated
mirtarbase	hsa-miR-181a-5p	RAN	5901	ENSG00000132341	22473208	validated
mirtarbase	hsa-miR-106b-5p	RANGAP1	5905	ENSG00000100401	23824327	validated
mirtarbase	hsa-miR-18a-5p	RAP1A	5906	ENSG00000116473	23446348	validated
mirtarbase	hsa-miR-181a-5p	RAP1B	5908	ENSG00000127314	24573637	validated
mirtarbase	hsa-miR-192-5p	RAP1GAP	5909	ENSG00000076864	19074876	validated
mirtarbase	hsa-miR-15b-5p	RARB	5915	ENSG00000077092	23446348	validated
mirtarbase	hsa-miR-212-3p	RB1	5925	ENSG00000139687	21329664	validated
mirtarbase	hsa-miR-21-5p	RB1	5925	ENSG00000139687	18591254	validated
mirtarbase	hsa-miR-21-5p	ARID4A	5926	ENSG00000032219	18591254	validated
mirtarbase	hsa-miR-181a-5p	KDM5A	5927	ENSG00000073614	22473208	validated
mirtarbase	hsa-miR-15b-5p	RBBP6	5930	ENSG00000122257	24398324	validated
mirtarbase	hsa-miR-106b-5p	RBBP7	5931	ENSG00000102054	22473208	validated
mirtarbase	hsa-miR-106b-5p	RBL1	5933	ENSG00000080839	21283765	validated
mirtarbase	hsa-miR-192-5p	RBL2	5934	ENSG00000103479	16822819	validated
mirtarbase	hsa-miR-106b-5p	RBL2	5934	ENSG00000103479	21283765	validated
mirtarbase	hsa-miR-222-3p	RBMS2	5939	ENSG00000076067	23824327	validated
mirtarbase	hsa-miR-212-3p	RBP2	5948	ENSG00000114113	23922798	validated
mirtarbase	hsa-miR-192-5p	RECQL	5965	ENSG00000004700	19074876	validated
mirtarbase	hsa-miR-15b-5p	REL	5966	ENSG00000162924	23592263	validated
mirtarbase	hsa-miR-582-5p	REL	5966	ENSG00000162924	23592263	validated
mirtarbase	hsa-miR-21-5p	REST	5978	ENSG00000084093	19242418	validated
mirtarbase	hsa-miR-106b-5p	REST	5978	ENSG00000084093	23446348	validated
mirtarbase	hsa-miR-505-3p	REST	5978	ENSG00000084093	20371350	validated
mirtarbase	hsa-miR-192-5p	RET	5979	ENSG00000165731	19074876	validated
mirtarbase	hsa-miR-21-5p	REV3L	5980	ENSG00000009413	18591254	validated
mirtarbase	hsa-miR-222-3p	RFC1	5981	ENSG00000035928	23622248	validated
mirtarbase	hsa-miR-301a-3p	RFC2	5982	ENSG00000049541	23824327	validated
mirtarbase	hsa-miR-192-5p	RFC4	5984	ENSG00000163918	19074876	validated
mirtarbase	hsa-miR-301a-3p	RFXAP	5994	ENSG00000133111	19536157	validated
mirtarbase	hsa-miR-106b-5p	RFXAP	5994	ENSG00000133111	19536157	validated
mirtarbase	hsa-miR-212-3p	RFXAP	5994	ENSG00000133111	26337469	validated
mirtarbase	hsa-miR-505-3p	RGS2	5997	ENSG00000116741	28735896	validated
mirtarbase	hsa-miR-181a-5p	RGS16	6004	ENSG00000143333	22473208	validated
mirtarbase	hsa-miR-21-5p	RHO	6010	ENSG00000163914	23446999	validated
mirtarbase	hsa-miR-181a-5p	RLF	6018	ENSG00000117000	17612493	validated
mirtarbase	hsa-miR-192-5p	RNASE4	6038	ENSG00000258818	19074876	validated
mirtarbase	hsa-miR-192-5p	RNASEL	6041	ENSG00000135828	19074876	validated
mirtarbase	hsa-miR-181a-5p	RNF2	6045	ENSG00000121481	21840484	validated
mirtarbase	hsa-miR-222-3p	RNF4	6047	ENSG00000063978	23622248	validated
mirtarbase	hsa-miR-18a-5p	RNF4	6047	ENSG00000063978	23622248	validated
mirtarbase	hsa-miR-192-5p	RNF6	6049	ENSG00000127870	19074876	validated

mirtarbase	hsa-miR-21-5p	RNF6	6049	ENSG00000127870	21572407	validated
mirtarbase	hsa-miR-181a-5p	RNF6	6049	ENSG00000127870	21572407	validated
mirtarbase	hsa-miR-15b-5p	RNH1	6050	ENSG00000276230	22473208	validated
mirtarbase	hsa-miR-106b-5p	RORA	6095	ENSG00000069667	17242205	validated
mirtarbase	hsa-miR-144-3p	RORA	6095	ENSG00000069667	22012620	validated
mirtarbase	hsa-miR-18a-5p	RORA	6095	ENSG00000069667	21572407	validated
mirtarbase	hsa-miR-181a-5p	RP2	6102	ENSG00000102218	22473208	validated
mirtarbase	hsa-miR-106b-5p	RPA2	6118	ENSG00000117748	22473208	validated
mirtarbase	hsa-miR-106b-5p	RPL7	6129	ENSG00000147604	23622248	validated
mirtarbase	hsa-miR-18a-5p	RPL7A	6130	ENSG00000148303	23622248	validated
mirtarbase	hsa-miR-222-3p	RPL8	6132	ENSG00000161016	23622248	validated
mirtarbase	hsa-miR-505-3p	RPL9	6133	ENSG00000163682	23622248	validated
mirtarbase	hsa-miR-18a-5p	RPL9	6133	ENSG00000163682	23622248	validated
mirtarbase	hsa-miR-222-3p	RPL12	6136	ENSG00000197958	23622248	validated
mirtarbase	hsa-miR-106b-5p	RPL17	6139	ENSG00000265681	22473208	validated
mirtarbase	hsa-miR-106b-5p	RPL18	6141	ENSG00000063177	23622248	validated
mirtarbase	hsa-miR-301a-3p	RPL18A	6142	ENSG00000105640	23622248	validated
mirtarbase	hsa-miR-106b-5p	RPL18A	6142	ENSG00000105640	23622248	validated
mirtarbase	hsa-miR-106b-5p	RPL21	6144	ENSG00000122026	23622248	validated
mirtarbase	hsa-miR-15b-5p	RPL27A	6157	ENSG00000166441	23622248	validated
mirtarbase	hsa-miR-505-3p	RPLP0	6175	ENSG00000089157	23622248	validated
mirtarbase	hsa-miR-15b-5p	RPLP0	6175	ENSG00000089157	23622248	validated
mirtarbase	hsa-miR-222-3p	RPS2	6187	ENSG00000140988	23622248	validated
mirtarbase	hsa-miR-15b-5p	RPS3	6188	ENSG00000149273	23622248	validated
mirtarbase	hsa-miR-15b-5p	RPS3A	6189	ENSG00000145425	23622248	validated
mirtarbase	hsa-miR-15b-5p	RPS5	6193	ENSG00000083845	22473208	validated
mirtarbase	hsa-miR-15b-5p	RPS6	6194	ENSG00000137154	23622248	validated
mirtarbase	hsa-miR-21-5p	RPS6KA3	6197	ENSG00000177189	18591254	validated
mirtarbase	hsa-miR-181a-5p	RPS6KA3	6197	ENSG00000177189	23824327	validated
mirtarbase	hsa-miR-15b-5p	RPS6KA3	6197	ENSG00000177189	22473208	validated
mirtarbase	hsa-miR-192-5p	RPS6KB1	6198	ENSG00000108443	19074876	validated
mirtarbase	hsa-miR-15b-5p	RPS6KB1	6198	ENSG00000108443	24398324	validated
mirtarbase	hsa-miR-21-5p	RPS7	6201	ENSG00000171863	19253296	validated
mirtarbase	hsa-miR-181a-5p	RPS8	6202	ENSG00000142937	23622248	validated
mirtarbase	hsa-miR-106b-5p	RPS14	6208	ENSG00000164587	23622248	validated
mirtarbase	hsa-miR-505-3p	RPS15	6209	ENSG00000115268	23622248	validated
mirtarbase	hsa-miR-301a-3p	RPS15A	6210	ENSG00000134419	23824327	validated
mirtarbase	hsa-miR-222-3p	RPS17	6218	ENSG00000278229	23622248	validated
mirtarbase	hsa-miR-106b-5p	RPS27A	6233	ENSG00000143947	23592263	validated
mirtarbase	hsa-miR-301a-3p	RPS27A	6233	ENSG00000143947	23592263	validated
mirtarbase	hsa-miR-106b-5p	RREB1	6239	ENSG00000124782	23622248	validated
mirtarbase	hsa-miR-582-5p	RREB1	6239	ENSG00000124782	23592263	validated
mirtarbase	hsa-miR-582-3p	RREB1	6239	ENSG00000124782	23592263	validated
mirtarbase	hsa-miR-144-3p	RREB1	6239	ENSG00000124782	20371350	validated
mirtarbase	hsa-miR-192-5p	RRM1	6240	ENSG00000167325	16822819	validated
mirtarbase	hsa-miR-106b-5p	RRM2	6241	ENSG00000171848	22473208	validated
mirtarbase	hsa-miR-582-5p	RRM2	6241	ENSG00000171848	23592263	validated
mirtarbase	hsa-miR-15b-5p	RS1	6247	ENSG00000102104	23706177	validated
mirtarbase	hsa-miR-301a-3p	CLIP1	6249	ENSG00000130779	24398324	validated
mirtarbase	hsa-miR-106b-5p	RTN2	6253	ENSG00000125744	23313552	validated
mirtarbase	hsa-miR-181a-5p	S100A1	6271	ENSG00000160678	17612493	validated
mirtarbase	hsa-miR-222-3p	SORT1	6272	ENSG00000134243	23622248	validated
mirtarbase	hsa-miR-181a-5p	SORT1	6272	ENSG00000134243	22473208	validated
mirtarbase	hsa-miR-296-5p	S100A2	6273	ENSG00000196754	22012620	validated
mirtarbase	hsa-miR-296-5p	S100B	6285	ENSG00000160307	27186401	validated
mirtarbase	hsa-miR-505-3p	SALL1	6299	ENSG00000103449	22012620	validated
mirtarbase	hsa-miR-15b-5p	SALL1	6299	ENSG00000103449	21572407	validated
mirtarbase	hsa-miR-21-5p	SATB1	6304	ENSG00000182568	22227207	validated
mirtarbase	hsa-miR-582-5p	MSMO1	6307	ENSG00000052802	23592263	validated
mirtarbase	hsa-miR-106b-5p	MSMO1	6307	ENSG00000052802	22473208	validated
mirtarbase	hsa-miR-301a-3p	MSMO1	6307	ENSG00000052802	21572407	validated
mirtarbase	hsa-miR-106b-5p	ATXN1	6310	ENSG00000124788	22473208	validated
mirtarbase	hsa-miR-144-3p	ATXN1	6310	ENSG00000124788	21572407	validated
mirtarbase	hsa-miR-192-5p	ATXN7	6314	ENSG00000285258	16822819	validated
mirtarbase	hsa-miR-181a-5p	ATXN7	6314	ENSG00000285258	23824327	validated
mirtarbase	hsa-miR-181a-5p	SCD	6319	ENSG00000099194	20371350	validated
mirtarbase	hsa-miR-192-5p	SCD	6319	ENSG00000099194	19074876	validated
mirtarbase	hsa-miR-106b-5p	SCD	6319	ENSG00000099194	21572407	validated
mirtarbase	hsa-miR-505-3p	SCD	6319	ENSG00000099194	26701625	validated
mirtarbase	hsa-miR-582-3p	SCN4B	6330	ENSG00000177098	26701625	validated
mirtarbase	hsa-miR-192-5p	SCN5A	6331	ENSG00000183873	26209011	validated

mirtarbase	hsa-miR-181a-5p	SCN8A	6334	ENSG00000196876	23824327	validated
mirtarbase	hsa-miR-21-5p	CCL1	6346	ENSG00000108702	22473208	validated
mirtarbase	hsa-miR-106b-5p	CCL1	6346	ENSG00000108702	22473208	validated
mirtarbase	hsa-miR-192-5p	CCL3L1	6349	ENSG00000277336	19074876	validated
mirtarbase	hsa-miR-192-5p	CCL3L3	6349	ENSG00000277336	19074876	validated
mirtarbase	hsa-miR-106b-5p	CCL5	6352	ENSG00000274233	23313552	validated
mirtarbase	hsa-miR-21-5p	CCL20	6364	ENSG00000115009	22001440	validated
mirtarbase	hsa-miR-181a-5p	CCL22	6367	ENSG00000102962	22473208	validated
mirtarbase	hsa-miR-18a-5p	SDC4	6385	ENSG00000124145	25089138	validated
mirtarbase	hsa-miR-222-3p	SDHA	6389	ENSG00000073578	23622248	validated
mirtarbase	hsa-miR-505-3p	SEC13	6396	ENSG00000157020	23622248	validated
mirtarbase	hsa-miR-21-5p	TRAPPC2	6399	ENSG00000196459	18591254	validated
mirtarbase	hsa-miR-106b-5p	TRAPPC2	6399	ENSG00000196459	21572407	validated
mirtarbase	hsa-miR-222-3p	SELE	6401	ENSG00000007908	19949084	validated
mirtarbase	hsa-miR-15b-5p	MAP2K4	6416	ENSG00000065559	23622248	validated
mirtarbase	hsa-miR-582-5p	SET	6418	ENSG00000119335	23592263	validated
mirtarbase	hsa-miR-21-5p	SET	6418	ENSG00000119335	22473208	validated
mirtarbase	hsa-miR-582-3p	SFRP1	6422	ENSG00000104332	26468775	validated
mirtarbase	hsa-miR-505-3p	SRSF1	6426	ENSG00000136450	20923760	validated
mirtarbase	hsa-miR-15b-5p	SRSF1	6426	ENSG00000136450	23446348	validated
mirtarbase	hsa-miR-106b-5p	SRSF2	6427	ENSG00000161547	22473208	validated
mirtarbase	hsa-miR-222-3p	SRSF2	6427	ENSG00000161547	20371350	validated
mirtarbase	hsa-miR-301a-3p	SRSF2	6427	ENSG00000161547	20371350	validated
mirtarbase	hsa-miR-18a-5p	SRSF6	6431	ENSG00000124193	23622248	validated
mirtarbase	hsa-miR-181a-5p	SRSF7	6432	ENSG00000115875	20371350	validated
mirtarbase	hsa-miR-192-5p	SGCB	6443	ENSG00000163069	19074876	validated
mirtarbase	hsa-miR-21-5p	SGCB	6443	ENSG00000163069	18591254	validated
mirtarbase	hsa-miR-181a-5p	SH3BGRL	6451	ENSG00000131171	17612493	validated
mirtarbase	hsa-miR-582-5p	SHMT1	6470	ENSG00000176974	27292025	validated
mirtarbase	hsa-miR-21-5p	ST6GAL1	6480	ENSG00000073849	18591254	validated
mirtarbase	hsa-miR-192-5p	STIL	6491	ENSG00000123473	19074876	validated
mirtarbase	hsa-miR-106b-5p	SKI	6497	ENSG00000157933	22473208	validated
mirtarbase	hsa-miR-15b-5p	SKI	6497	ENSG00000157933	22473208	validated
mirtarbase	hsa-miR-106b-5p	SKIL	6498	ENSG00000136603	24398324	validated
mirtarbase	hsa-miR-222-3p	SKIV2L	6499	ENSG00000223493	23622248	validated
mirtarbase	hsa-miR-21-5p	SKP2	6502	ENSG00000145604	18591254	validated
mirtarbase	hsa-miR-192-5p	SLC1A4	6509	ENSG00000115902	19074876	validated
mirtarbase	hsa-miR-15b-5p	SLC1A5	6510	ENSG00000105281	23622248	validated
mirtarbase	hsa-miR-106b-5p	SLC1A5	6510	ENSG00000105281	23313552	validated
mirtarbase	hsa-miR-15b-5p	SLC2A3	6515	ENSG00000059804	23446348	validated
mirtarbase	hsa-miR-106b-5p	SLC2A4	6517	ENSG00000181856	27165190	validated
mirtarbase	hsa-miR-21-5p	SLC5A3	6526	ENSG00000198743	18591254	validated
mirtarbase	hsa-miR-106b-5p	SLC5A3	6526	ENSG00000198743	21572407	validated
mirtarbase	hsa-miR-106b-5p	SLC6A4	6532	ENSG00000108576	23313552	validated
mirtarbase	hsa-miR-181a-5p	SLC7A1	6541	ENSG00000139514	22473208	validated
mirtarbase	hsa-miR-192-5p	SLC7A2	6542	ENSG00000003989	19074876	validated
mirtarbase	hsa-miR-144-3p	SLC7A2	6542	ENSG00000003989	21572407	validated
mirtarbase	hsa-miR-582-5p	SLC7A2	6542	ENSG00000003989	21572407	validated
mirtarbase	hsa-miR-106b-5p	SLC9A1	6548	ENSG00000090020	23622248	validated
mirtarbase	hsa-miR-15b-5p	SLC9A1	6548	ENSG00000090020	22473208	validated
mirtarbase	hsa-miR-192-5p	SLC9A5	6553	ENSG00000135740	19074876	validated
mirtarbase	hsa-miR-181a-5p	SLCO2A1	6578	ENSG00000174640	17612493	validated
mirtarbase	hsa-miR-181a-5p	SNAI2	6591	ENSG00000019549	17612493	validated
mirtarbase	hsa-miR-192-5p	SMARCA2	6595	ENSG00000080503	19074876	validated
mirtarbase	hsa-miR-21-5p	SMARCA4	6597	ENSG00000127616	21317927	validated
mirtarbase	hsa-miR-222-3p	SMARCA4	6597	ENSG00000127616	23622248	validated
mirtarbase	hsa-miR-192-5p	SMARCB1	6598	ENSG00000099956	19074876	validated
mirtarbase	hsa-miR-21-5p	SMN2	6606	ENSG00000273772	23657402	validated
mirtarbase	hsa-miR-21-5p	SMN1	6606	ENSG00000275349	23657402	validated
mirtarbase	hsa-miR-15b-5p	SNCG	6623	ENSG00000173267	26701625	validated
mirtarbase	hsa-miR-326	FSCN1	6624	ENSG00000075618	26359764	validated
mirtarbase	hsa-miR-15b-5p	SNRPB2	6629	ENSG00000125870	24398324	validated
mirtarbase	hsa-miR-192-5p	SNRPD1	6632	ENSG00000167088	19074876	validated
mirtarbase	hsa-miR-301a-3p	SNTB1	6641	ENSG00000172164	23592263	validated
mirtarbase	hsa-miR-15b-5p	SNTB2	6645	ENSG00000260873	22012620	validated
mirtarbase	hsa-miR-106b-5p	SNTB2	6645	ENSG00000260873	22473208	validated
mirtarbase	hsa-miR-301a-3p	SNTB2	6645	ENSG00000260873	23824327	validated
mirtarbase	hsa-miR-192-5p	SOAT1	6646	ENSG00000057252	19074876	validated
mirtarbase	hsa-miR-212-3p	SOD2	6648	ENSG00000112096	23583431	validated
mirtarbase	hsa-miR-106b-5p	SOD2	6648	ENSG00000112096	22473208	validated
mirtarbase	hsa-miR-505-3p	SOD2	6648	ENSG00000112096	23313552	validated

mirtarbase	hsa-miR-21-5p	SOD3	6649	ENSG00000109610	22836756	validated
mirtarbase	hsa-miR-106b-5p	CAPN15	6650	ENSG00000103326	23592263	validated
mirtarbase	hsa-miR-18a-5p	SON	6651	ENSG00000159140	20371350	validated
mirtarbase	hsa-miR-222-3p	SON	6651	ENSG00000159140	23622248	validated
mirtarbase	hsa-miR-106b-5p	SON	6651	ENSG00000159140	27418678	validated
mirtarbase	hsa-miR-21-5p	SOX2	6657	ENSG00000181449	18591254	validated
mirtarbase	hsa-miR-106b-5p	SOX4	6659	ENSG00000124766	23592263	validated
mirtarbase	hsa-miR-301a-3p	SOX4	6659	ENSG00000124766	23592263	validated
mirtarbase	hsa-miR-212-3p	SOX4	6659	ENSG00000124766	26377202	validated
mirtarbase	hsa-miR-181a-5p	SOX5	6660	ENSG00000134532	22942087	validated
mirtarbase	hsa-miR-505-3p	SOX11	6664	ENSG00000176887	23446348	validated
mirtarbase	hsa-miR-212-3p	SOX11	6664	ENSG00000176887	25766675	validated
mirtarbase	hsa-miR-296-5p	SOX12	6666	ENSG00000177732	23592263	validated
mirtarbase	hsa-miR-18a-5p	SOX12	6666	ENSG00000177732	23313552	validated
mirtarbase	hsa-miR-21-5p	SP1	6667	ENSG00000185591	22034194	validated
mirtarbase	hsa-miR-296-5p	SP1	6667	ENSG00000185591	23592263	validated
mirtarbase	hsa-miR-326	SP1	6667	ENSG00000185591	27460077	validated
mirtarbase	hsa-miR-106b-5p	SP2	6668	ENSG00000167182	27418678	validated
mirtarbase	hsa-miR-192-5p	SP4	6671	ENSG00000105866	19074876	validated
mirtarbase	hsa-miR-106b-5p	SP4	6671	ENSG00000105866	23446348	validated
mirtarbase	hsa-miR-18a-5p	SP100	6672	ENSG00000067066	23592263	validated
mirtarbase	hsa-miR-106b-5p	SPAG1	6674	ENSG00000104450	17242205	validated
mirtarbase	hsa-miR-582-5p	SPAG1	6674	ENSG00000104450	20371350	validated
mirtarbase	hsa-miR-144-3p	SPAG1	6674	ENSG00000104450	20371350	validated
mirtarbase	hsa-miR-192-5p	SPARC	6678	ENSG00000113140	16822819	validated
mirtarbase	hsa-miR-106b-5p	SPIB	6689	ENSG00000269404	23824327	validated
mirtarbase	hsa-miR-192-5p	SPTBN1	6711	ENSG00000115306	19074876	validated
mirtarbase	hsa-miR-222-3p	SPTBN2	6712	ENSG00000173898	23622248	validated
mirtarbase	hsa-miR-192-5p	SRD5A1	6715	ENSG00000145545	19074876	validated
mirtarbase	hsa-miR-582-5p	SREBF2	6721	ENSG00000198911	23592263	validated
mirtarbase	hsa-miR-15b-5p	SRPK1	6732	ENSG00000096063	22473208	validated
mirtarbase	hsa-miR-181a-5p	SRPK2	6733	ENSG00000135250	17612493	validated
mirtarbase	hsa-miR-21-5p	SRPK2	6733	ENSG00000135250	18591254	validated
mirtarbase	hsa-miR-15b-5p	SRPRA	6734	ENSG00000182934	22473208	validated
mirtarbase	hsa-miR-301a-3p	RO60	6738	ENSG00000116747	20371350	validated
mirtarbase	hsa-miR-21-5p	ITPRID2	6744	ENSG00000138434	18591254	validated
mirtarbase	hsa-miR-15b-5p	SSRP1	6749	ENSG00000149136	22473208	validated
mirtarbase	hsa-miR-301a-3p	SSTR2	6752	ENSG00000180616	23824327	validated
mirtarbase	hsa-miR-192-5p	SSTR2	6752	ENSG00000180616	27418678	validated
mirtarbase	hsa-miR-582-3p	SSX5	6758	ENSG00000165583	23824327	validated
mirtarbase	hsa-miR-192-5p	SS18	6760	ENSG00000141380	19074876	validated
mirtarbase	hsa-miR-222-3p	SS18	6760	ENSG00000141380	23622248	validated
mirtarbase	hsa-miR-106b-5p	STAT3	6774	ENSG00000168610	25307786	validated
mirtarbase	hsa-miR-181a-5p	STAT3	6774	ENSG00000168610	24329418	validated
mirtarbase	hsa-miR-181a-5p	HSPA13	6782	ENSG00000155304	20371350	validated
mirtarbase	hsa-miR-144-3p	HSPA13	6782	ENSG00000155304	20371350	validated
mirtarbase	hsa-miR-21-5p	ELOVL4	6785	ENSG00000118402	18591254	validated
mirtarbase	hsa-miR-192-5p	STK3	6788	ENSG00000104375	19074876	validated
mirtarbase	hsa-miR-18a-5p	STK4	6789	ENSG00000101109	24752237	validated
mirtarbase	hsa-miR-222-3p	CDKL5	6792	ENSG00000008086	23622248	validated
mirtarbase	hsa-miR-106b-5p	STX4	6810	ENSG00000103496	23313552	validated
mirtarbase	hsa-miR-15b-5p	STXBP1	6812	ENSG00000136854	23622248	validated
mirtarbase	hsa-miR-15b-5p	STXBP3	6814	ENSG00000116266	22473208	validated
mirtarbase	hsa-miR-582-5p	STYX	6815	ENSG00000198252	23592263	validated
mirtarbase	hsa-miR-582-3p	SURF4	6836	ENSG00000148248	20371350	validated
mirtarbase	hsa-miR-192-5p	MED22	6837	ENSG00000148297	19074876	validated
mirtarbase	hsa-miR-296-5p	VAMP1	6843	ENSG00000139190	23592263	validated
mirtarbase	hsa-miR-18a-5p	SYPL1	6856	ENSG00000008282	20371350	validated
mirtarbase	hsa-miR-15b-5p	SYPL1	6856	ENSG00000008282	24398324	validated
mirtarbase	hsa-miR-106b-5p	SYT4	6860	ENSG00000132872	17242205	validated
mirtarbase	hsa-miR-505-3p	SYT4	6860	ENSG00000132872	22012620	validated
mirtarbase	hsa-miR-181a-5p	ADAM17	6868	ENSG00000151694	23313552	validated
mirtarbase	hsa-miR-21-5p	TAF1	6872	ENSG00000147133	18591254	validated
mirtarbase	hsa-miR-181a-5p	TAF2	6873	ENSG00000064313	17612493	validated
mirtarbase	hsa-miR-21-5p	TAF5	6877	ENSG00000148835	18591254	validated
mirtarbase	hsa-miR-505-3p	TAF9	6880	ENSG00000276463	23622248	validated
mirtarbase	hsa-miR-15b-5p	TAF13	6884	ENSG00000197780	21572407	validated
mirtarbase	hsa-miR-144-3p	TAF13	6884	ENSG00000197780	27292025	validated
mirtarbase	hsa-miR-15b-5p	MAP3K7	6885	ENSG00000135341	20371350	validated
mirtarbase	hsa-miR-21-5p	TAP1	6890	ENSG00000227816	25327529	validated
mirtarbase	hsa-miR-15b-5p	TARBP2	6895	ENSG00000139546	21572407	validated

mirtarbase	hsa-miR-192-5p	TBL1X	6907	ENSG00000101849	19074876	validated
mirtarbase	hsa-miR-18a-5p	TCEA2	6919	ENSG00000171703	23622248	validated
mirtarbase	hsa-miR-212-3p	ELOC	6921	ENSG00000154582	24398324	validated
mirtarbase	hsa-miR-106b-5p	ELOC	6921	ENSG00000154582	23824327	validated
mirtarbase	hsa-miR-222-3p	ELOB	6923	ENSG00000103363	23622248	validated
mirtarbase	hsa-miR-192-5p	ELOA	6924	ENSG00000011007	19074876	validated
mirtarbase	hsa-miR-106b-5p	TCF4	6925	ENSG00000196628	22473208	validated
mirtarbase	hsa-miR-181a-5p	TCF4	6925	ENSG00000196628	28086904	validated
mirtarbase	hsa-miR-15b-5p	HNF1A	6927	ENSG00000135100	24705650	validated
mirtarbase	hsa-miR-192-5p	HNF1B	6928	ENSG00000275410	19074876	validated
mirtarbase	hsa-miR-15b-5p	TCF3	6929	ENSG00000071564	22473208	validated
mirtarbase	hsa-miR-192-5p	TCF7	6932	ENSG00000081059	19074876	validated
mirtarbase	hsa-miR-106b-5p	TCF7L2	6934	ENSG00000148737	21572407	validated
mirtarbase	hsa-miR-301a-3p	TCF7L2	6934	ENSG00000148737	21572407	validated
mirtarbase	hsa-miR-505-3p	TCF7L2	6934	ENSG00000148737	23824327	validated
mirtarbase	hsa-miR-144-3p	ZEB1	6935	ENSG00000148516	27785072	validated
mirtarbase	hsa-miR-21-5p	TCF21	6943	ENSG00000118526	23206776	validated
mirtarbase	hsa-miR-181a-5p	TCF21	6943	ENSG00000118526	17612493	validated
mirtarbase	hsa-miR-222-3p	TCOF1	6949	ENSG00000070814	23622248	validated
mirtarbase	hsa-miR-15b-5p	TCP1	6950	ENSG00000120438	23622248	validated
mirtarbase	hsa-miR-18a-5p	TCP1	6950	ENSG00000120438	23622248	validated
mirtarbase	hsa-miR-15b-5p	PPP1R11	6992	ENSG00000204619	23313552	validated
mirtarbase	hsa-miR-326	DYNLT1	6993	ENSG00000146425	23622248	validated
mirtarbase	hsa-miR-192-5p	TDG	6996	ENSG00000139372	19074876	validated
mirtarbase	hsa-miR-181a-5p	TEAD4	7004	ENSG00000197905	23622248	validated
mirtarbase	hsa-miR-181a-5p	TEF	7008	ENSG00000167074	23313552	validated
mirtarbase	hsa-miR-106b-5p	TMBIM6	7009	ENSG00000139644	22473208	validated
mirtarbase	hsa-miR-144-3p	TMBIM6	7009	ENSG00000139644	23446348	validated
mirtarbase	hsa-miR-15b-5p	TERF2	7014	ENSG00000132604	23622248	validated
mirtarbase	hsa-miR-301a-3p	TERF2	7014	ENSG00000132604	23824327	validated
mirtarbase	hsa-miR-181a-5p	TERT	7015	ENSG00000164362	25444904	validated
mirtarbase	hsa-miR-106b-5p	TFAM	7019	ENSG00000108064	23592263	validated
mirtarbase	hsa-miR-15b-5p	TFAP2A	7020	ENSG00000137203	23592263	validated
mirtarbase	hsa-miR-222-3p	TFAP2A	7020	ENSG00000137203	20371350	validated
mirtarbase	hsa-miR-582-5p	TFAP4	7023	ENSG00000090447	21572407	validated
mirtarbase	hsa-miR-144-3p	TFAP4	7023	ENSG00000090447	21572407	validated
mirtarbase	hsa-miR-144-3p	NR2F2	7026	ENSG00000185551	23592263	validated
mirtarbase	hsa-miR-582-5p	NR2F2	7026	ENSG00000185551	23592263	validated
mirtarbase	hsa-miR-181a-5p	TFRC	7037	ENSG00000072274	22473208	validated
mirtarbase	hsa-miR-505-3p	TGFA	7039	ENSG00000163235	26832151	validated
mirtarbase	hsa-miR-144-3p	TGFB1	7040	ENSG00000105329	21991303	validated
mirtarbase	hsa-miR-15b-5p	TGFB1	7040	ENSG00000105329	27499071	validated
mirtarbase	hsa-miR-21-5p	TGFB2	7042	ENSG00000092969	18591254	validated
mirtarbase	hsa-miR-21-5p	TGFBI	7045	ENSG00000120708	19136465	validated
mirtarbase	hsa-miR-181a-5p	TGFBR1	7046	ENSG00000106799	22942087	validated
mirtarbase	hsa-miR-18a-5p	TGFBR2	7048	ENSG00000163513	20940405	validated
mirtarbase	hsa-miR-106b-5p	TGFBR2	7048	ENSG00000163513	22473208	validated
mirtarbase	hsa-miR-301a-3p	TGFBR2	7048	ENSG00000163513	22012620	validated
mirtarbase	hsa-miR-582-5p	TGFBR2	7048	ENSG00000163513	23592263	validated
mirtarbase	hsa-miR-505-3p	TGFBR2	7048	ENSG00000163513	19536157	validated
mirtarbase	hsa-miR-181a-5p	TGFBR3	7049	ENSG00000069702	20371350	validated
mirtarbase	hsa-miR-582-5p	TGFBR3	7049	ENSG00000069702	21572407	validated
mirtarbase	hsa-miR-144-3p	TGFBR3	7049	ENSG00000069702	21572407	validated
mirtarbase	hsa-miR-15b-5p	TGFBR3	7049	ENSG00000069702	20371350	validated
mirtarbase	hsa-miR-21-5p	TGIF1	7050	ENSG00000177426	19906824	validated
mirtarbase	hsa-miR-192-5p	THBD	7056	ENSG00000178726	19074876	validated
mirtarbase	hsa-miR-192-5p	THOP1	7064	ENSG00000172009	19074876	validated
mirtarbase	hsa-miR-505-3p	THOP1	7064	ENSG00000172009	23622248	validated
mirtarbase	hsa-miR-222-3p	THOP1	7064	ENSG00000172009	23622248	validated
mirtarbase	hsa-miR-18a-5p	THRA	7067	ENSG00000126351	16331254	validated
mirtarbase	hsa-miR-15b-5p	THRA	7067	ENSG00000126351	23622248	validated
mirtarbase	hsa-miR-301a-3p	THRA	7067	ENSG00000126351	23592263	validated
mirtarbase	hsa-miR-192-5p	KLF10	7071	ENSG00000155090	19074876	validated
mirtarbase	hsa-miR-106b-5p	KLF10	7071	ENSG00000155090	23313552	validated
mirtarbase	hsa-miR-181a-5p	TIAL1	7073	ENSG00000151923	20371350	validated
mirtarbase	hsa-miR-21-5p	TIAM1	7074	ENSG00000156299	20826792	validated
mirtarbase	hsa-miR-181a-5p	TIMP1	7076	ENSG00000102265	22492871	validated
mirtarbase	hsa-miR-301a-3p	TIMP2	7077	ENSG00000035862	26464662	validated
mirtarbase	hsa-miR-505-3p	TLE3	7090	ENSG00000140332	23622248	validated
mirtarbase	hsa-miR-222-3p	TLE3	7090	ENSG00000140332	23622248	validated
mirtarbase	hsa-miR-18a-5p	TLE3	7090	ENSG00000140332	23622248	validated

mirtarbase	hsa-miR-15b-5p	TLE4	7091	ENSG00000106829	22473208	validated
mirtarbase	hsa-miR-15b-5p	TLL1	7092	ENSG00000038295	22100165	validated
mirtarbase	hsa-miR-222-3p	TLN1	7094	ENSG00000137076	23622248	validated
mirtarbase	hsa-miR-106b-5p	TLR2	7097	ENSG00000137462	17242205	validated
mirtarbase	hsa-miR-21-5p	TLR3	7098	ENSG00000164342	25327529	validated
mirtarbase	hsa-miR-21-5p	TLR4	7099	ENSG00000136869	18591254	validated
mirtarbase	hsa-miR-106b-5p	TSPAN6	7105	ENSG00000000003	23313552	validated
mirtarbase	hsa-miR-144-3p	TSPAN6	7105	ENSG00000000003	21572407	validated
mirtarbase	hsa-miR-181a-5p	GPR137B	7107	ENSG00000077585	17612493	validated
mirtarbase	hsa-miR-106b-5p	GPR137B	7107	ENSG00000077585	20371350	validated
mirtarbase	hsa-miR-106b-5p	TRAPPC10	7109	ENSG00000160218	22473208	validated
mirtarbase	hsa-miR-181a-5p	TMF1	7110	ENSG00000144747	22473208	validated
mirtarbase	hsa-miR-192-5p	TMPO	7112	ENSG00000120802	19074876	validated
mirtarbase	hsa-miR-106b-5p	TMPO	7112	ENSG00000120802	23622248	validated
mirtarbase	hsa-miR-582-5p	TNFAIP1	7126	ENSG00000109079	23592263	validated
mirtarbase	hsa-miR-106b-5p	TNFAIP1	7126	ENSG00000109079	22473208	validated
mirtarbase	hsa-miR-21-5p	TNFAIP3	7128	ENSG00000118503	21131358	validated
mirtarbase	hsa-miR-505-3p	TNFAIP3	7128	ENSG00000118503	21572407	validated
mirtarbase	hsa-miR-18a-5p	TNFAIP3	7128	ENSG00000118503	26783726	validated
mirtarbase	hsa-miR-106b-5p	TNNI1	7135	ENSG00000159173	17242205	validated
mirtarbase	hsa-miR-192-5p	TOP1	7150	ENSG00000198900	19074876	validated
mirtarbase	hsa-miR-192-5p	TOP1P2	7152		19074876	validated
mirtarbase	hsa-miR-21-5p	TOP2A	7153	ENSG00000131747	18591254	validated
mirtarbase	hsa-miR-222-3p	TOP3A	7156	ENSG00000177302	23622248	validated
mirtarbase	hsa-miR-222-3p	TP53	7157	ENSG00000141510	21226887	validated
mirtarbase	hsa-miR-18a-5p	TP53	7157	ENSG00000141510	24955218	validated
mirtarbase	hsa-miR-106b-5p	TP53	7157	ENSG00000141510	24955218	validated
mirtarbase	hsa-miR-505-3p	TP53BP1	7158	ENSG00000067369	23622248	validated
mirtarbase	hsa-miR-222-3p	TP53BP2	7159	ENSG00000143514	22815788	validated
mirtarbase	hsa-miR-505-3p	TPBG	7162	ENSG00000146242	23622248	validated
mirtarbase	hsa-miR-505-3p	TPD52	7163	ENSG00000076554	23622248	validated
mirtarbase	hsa-miR-15b-5p	TPD52	7163	ENSG00000076554	23622248	validated
mirtarbase	hsa-miR-582-5p	TPI1	7167	ENSG00000111669	26701625	validated
mirtarbase	hsa-miR-15b-5p	TPM2	7169	ENSG00000198467	23592263	validated
mirtarbase	hsa-miR-15b-5p	TPM3	7170	ENSG00000143549	21572407	validated
mirtarbase	hsa-miR-192-5p	TPM4	7171	ENSG00000167460	19074876	validated
mirtarbase	hsa-miR-106b-5p	TPM4	7171	ENSG00000167460	23592263	validated
mirtarbase	hsa-miR-582-5p	TPM4	7171	ENSG00000167460	23592263	validated
mirtarbase	hsa-miR-21-5p	NR2C2	7182	ENSG00000177463	18591254	validated
mirtarbase	hsa-miR-106b-5p	NR2C2	7182	ENSG00000177463	22473208	validated
mirtarbase	hsa-miR-15b-5p	NR2C2	7182	ENSG00000177463	22473208	validated
mirtarbase	hsa-miR-192-5p	HSP90B1	7184	ENSG00000166598	16822819	validated
mirtarbase	hsa-miR-181a-5p	HSP90B1	7184	ENSG00000166598	23446348	validated
mirtarbase	hsa-miR-582-5p	HSP90B1	7184	ENSG00000166598	26701625	validated
mirtarbase	hsa-miR-192-5p	TRPC1	7220	ENSG00000144935	19074876	validated
mirtarbase	hsa-miR-301a-3p	TRPC3	7222	ENSG00000138741	21572407	validated
mirtarbase	hsa-miR-222-3p	TRPS1	7227	ENSG00000104447	21673316	validated
mirtarbase	hsa-miR-582-5p	TSN	7247	ENSG00000211460	23592263	validated
mirtarbase	hsa-miR-106b-5p	TSC1	7248	ENSG00000165699	23622248	validated
mirtarbase	hsa-miR-106b-5p	TSG101	7251	ENSG00000074319	22473208	validated
mirtarbase	hsa-miR-181a-5p	TSG101	7251	ENSG00000074319	22473208	validated
mirtarbase	hsa-miR-181a-5p	TSHR	7253	ENSG00000165409	17612493	validated
mirtarbase	hsa-miR-21-5p	TSNAX	7257	ENSG00000116918	18591254	validated
mirtarbase	hsa-miR-144-3p	TSPYL1	7259	ENSG00000189241	24398324	validated
mirtarbase	hsa-miR-15b-5p	TTC1	7265	ENSG00000113312	23622248	validated
mirtarbase	hsa-miR-181a-5p	DNAJC7	7266	ENSG00000168259	17612493	validated
mirtarbase	hsa-miR-192-5p	TTK	7272	ENSG00000112742	19074876	validated
mirtarbase	hsa-miR-144-3p	TTN	7273	ENSG00000155657	24453045	validated
mirtarbase	hsa-miR-192-5p	TTPA	7274	ENSG00000137561	19074876	validated
mirtarbase	hsa-miR-15b-5p	TUBB2A	7280	ENSG00000137267	20371350	validated
mirtarbase	hsa-miR-505-3p	TUBB2A	7280	ENSG00000137267	20371350	validated
mirtarbase	hsa-miR-181a-5p	TUBB2A	7280	ENSG00000137267	20371350	validated
mirtarbase	hsa-miR-106b-5p	TWIST1	7291	ENSG00000122691	24002805	validated
mirtarbase	hsa-miR-181a-5p	TWIST1	7291	ENSG00000122691	24148247	validated
mirtarbase	hsa-miR-106b-5p	TXK	7294	ENSG00000074966	22012620	validated
mirtarbase	hsa-miR-222-3p	TXN	7295	ENSG00000136810	23622248	validated
mirtarbase	hsa-miR-505-3p	TXNRD1	7296	ENSG00000198431	20371350	validated
mirtarbase	hsa-miR-301a-3p	UBB	7314	ENSG00000170315	23592263	validated
mirtarbase	hsa-miR-18a-5p	UBC	7316	ENSG00000150991	23622248	validated
mirtarbase	hsa-miR-106b-5p	UBC	7316	ENSG00000150991	22473208	validated
mirtarbase	hsa-miR-301a-3p	UBC	7316	ENSG00000150991	26701625	validated

mirtarbase	hsa-miR-222-3p	UBA1	7317	ENSG00000130985	23622248	validated
mirtarbase	hsa-miR-144-3p	UBE2A	7319	ENSG00000077721	23446348	validated
mirtarbase	hsa-miR-192-5p	UBE2D1	7321	ENSG00000072401	19074876	validated
mirtarbase	hsa-miR-192-5p	UBE2D2	7322	ENSG00000131508	19074876	validated
mirtarbase	hsa-miR-301a-3p	UBE2D2	7322	ENSG00000131508	21572407	validated
mirtarbase	hsa-miR-301a-3p	UBE2D3	7323	ENSG00000109332	23446348	validated
mirtarbase	hsa-miR-505-3p	UBE2D3	7323	ENSG00000109332	23446348	validated
mirtarbase	hsa-miR-18a-5p	UBE2G1	7326	ENSG00000132388	20371350	validated
mirtarbase	hsa-miR-15b-5p	UBE2H	7328	ENSG00000186591	20371350	validated
mirtarbase	hsa-miR-222-3p	UBE2N	7334	ENSG00000177889	20371350	validated
mirtarbase	hsa-miR-21-5p	UBE2N	7334	ENSG00000177889	25327529	validated
mirtarbase	hsa-miR-15b-5p	UBE2V1	7335	ENSG00000244687	23592263	validated
mirtarbase	hsa-miR-192-5p	UBE2V2	7336	ENSG00000169139	19074876	validated
mirtarbase	hsa-miR-106b-5p	UBE2V2	7336	ENSG00000169139	23313552	validated
mirtarbase	hsa-miR-181a-5p	UCHL1	7345	ENSG00000154277	23622248	validated
mirtarbase	hsa-miR-222-3p	SLC35A2	7355	ENSG00000102100	23622248	validated
mirtarbase	hsa-miR-106b-5p	UGCG	7357	ENSG00000148154	23592263	validated
mirtarbase	hsa-miR-106b-5p	UGP2	7360	ENSG00000169764	23622248	validated
mirtarbase	hsa-miR-15b-5p	UGT2B4	7363	ENSG00000156096	21572407	validated
mirtarbase	hsa-miR-192-5p	UGT8	7368	ENSG00000174607	19074876	validated
mirtarbase	hsa-miR-192-5p	UNG	7374	ENSG00000076248	19074876	validated
mirtarbase	hsa-miR-192-5p	UPP1	7378	ENSG00000183696	19074876	validated
mirtarbase	hsa-miR-301a-3p	UQCRB	7381	ENSG00000156467	22100165	validated
mirtarbase	hsa-miR-21-5p	UQCRB	7381	ENSG00000156467	23446348	validated
mirtarbase	hsa-miR-192-5p	UQCRC2	7385	ENSG00000140740	19074876	validated
mirtarbase	hsa-miR-106b-5p	UQCRFS1	7386	ENSG00000169021	23622248	validated
mirtarbase	hsa-miR-505-3p	UQCRFS1	7386	ENSG00000169021	20371350	validated
mirtarbase	hsa-miR-222-3p	UROD	7389	ENSG00000126088	23622248	validated
mirtarbase	hsa-miR-192-5p	USP1	7398	ENSG00000162607	19074876	validated
mirtarbase	hsa-miR-21-5p	UTRN	7402	ENSG00000152818	18591254	validated
mirtarbase	hsa-miR-505-3p	UTRN	7402	ENSG00000152818	23622248	validated
mirtarbase	hsa-miR-192-5p	UVRAG	7405	ENSG00000198382	19074876	validated
mirtarbase	hsa-miR-301a-3p	UVRAG	7405	ENSG00000198382	26990571	validated
mirtarbase	hsa-miR-15b-5p	VAV2	7410	ENSG00000160293	20371350	validated
mirtarbase	hsa-miR-505-3p	VBP1	7411	ENSG00000155959	23313552	validated
mirtarbase	hsa-miR-181a-5p	VCAM1	7412	ENSG00000162692	19536157	validated
mirtarbase	hsa-miR-222-3p	VCL	7414	ENSG00000035403	23622248	validated
mirtarbase	hsa-miR-15b-5p	VCL	7414	ENSG00000035403	22473208	validated
mirtarbase	hsa-miR-222-3p	VCP	7415	ENSG00000165280	23622248	validated
mirtarbase	hsa-miR-18a-5p	VCP	7415	ENSG00000165280	23592263	validated
mirtarbase	hsa-miR-106b-5p	VDAC1	7416	ENSG00000213585	22473208	validated
mirtarbase	hsa-miR-15b-5p	VDAC2	7417	ENSG00000165637	23622248	validated
mirtarbase	hsa-miR-15b-5p	VEGFA	7422	ENSG00000112715	18320040	validated
mirtarbase	hsa-miR-21-5p	VEGFA	7422	ENSG00000112715	19435867	validated
mirtarbase	hsa-miR-181a-5p	VEGFA	7422	ENSG00000112715	25106798	validated
mirtarbase	hsa-miR-296-5p	VEGFA	7422	ENSG00000112715	26500043	validated
mirtarbase	hsa-miR-21-5p	VHL	7428	ENSG00000134086	24012640	validated
mirtarbase	hsa-miR-18a-5p	EZR	7430	ENSG00000092820	20371350	validated
mirtarbase	hsa-miR-222-3p	EZR	7430	ENSG00000092820	23622248	validated
mirtarbase	hsa-miR-192-5p	TRPV1	7442	ENSG00000196689	19074876	validated
mirtarbase	hsa-miR-106b-5p	EIF4H	7458	ENSG00000106682	22012620	validated
mirtarbase	hsa-miR-21-5p	CORO2A	7464	ENSG00000106789	19342589	validated
mirtarbase	hsa-miR-106b-5p	WEE1	7465	ENSG00000166483	21283765	validated
mirtarbase	hsa-miR-15b-5p	WEE1	7465	ENSG00000166483	21572407	validated
mirtarbase	hsa-miR-21-5p	WFS1	7466	ENSG00000109501	19253296	validated
mirtarbase	hsa-miR-192-5p	NSD2	7468	ENSG00000109685	19074876	validated
mirtarbase	hsa-miR-21-5p	NSD2	7468	ENSG00000109685	18591254	validated
mirtarbase	hsa-miR-106b-5p	NSD2	7468	ENSG00000109685	22473208	validated
mirtarbase	hsa-miR-181a-5p	NSD2	7468	ENSG00000109685	23592263	validated
mirtarbase	hsa-miR-181a-5p	WNT2	7472	ENSG00000105989	17612493	validated
mirtarbase	hsa-miR-192-5p	WNT3	7473	ENSG00000277641	19074876	validated
mirtarbase	hsa-miR-21-5p	WNT5A	7474	ENSG00000114251	18591254	validated
mirtarbase	hsa-miR-144-3p	WNT7A	7476	ENSG00000154764	23824327	validated
mirtarbase	hsa-miR-144-3p	WRN	7486	ENSG00000165392	23592263	validated
mirtarbase	hsa-miR-212-3p	WT1	7490	ENSG00000184937	17875710	validated
mirtarbase	hsa-miR-192-5p	WT1	7490	ENSG00000184937	22012620	validated
mirtarbase	hsa-miR-144-3p	XIST	7503		28059474	validated
mirtarbase	hsa-miR-192-5p	XK	7504	ENSG00000047597	19074876	validated
mirtarbase	hsa-miR-192-5p	XPA	7507	ENSG00000136936	16822819	validated
mirtarbase	hsa-miR-192-5p	XPO1	7514	ENSG00000082898	19074876	validated
mirtarbase	hsa-miR-106b-5p	YES1	7525	ENSG00000176105	23622248	validated

mirtarbase	hsa-miR-301a-3p	YY1	7528	ENSG00000100811	23592263	validated
mirtarbase	hsa-miR-222-3p	YWHAG	7532	ENSG00000170027	23622248	validated
mirtarbase	hsa-miR-15b-5p	YWHAH	7533	ENSG00000128245	20371350	validated
mirtarbase	hsa-miR-106b-5p	YWHAZ	7534	ENSG00000164924	21572407	validated
mirtarbase	hsa-miR-192-5p	ZBTB14	7541	ENSG00000198081	19074876	validated
mirtarbase	hsa-miR-144-3p	ZFX	7543	ENSG00000005889	22955854	validated
mirtarbase	hsa-miR-18a-5p	ZNF711	7552	ENSG00000147180	20371350	validated
mirtarbase	hsa-miR-301a-3p	ZNF711	7552	ENSG00000147180	23446348	validated
mirtarbase	hsa-miR-212-3p	ZNF711	7552	ENSG00000147180	20371350	validated
mirtarbase	hsa-miR-106b-5p	ZNF7	7553	ENSG00000147789	23313552	validated
mirtarbase	hsa-miR-181a-5p	ZNF12	7559	ENSG00000164631	17612493	validated
mirtarbase	hsa-miR-106b-5p	ZNF12	7559	ENSG00000164631	22473208	validated
mirtarbase	hsa-miR-301a-3p	ZNF12	7559	ENSG00000164631	24398324	validated
mirtarbase	hsa-miR-18a-5p	ZNF708	7562	ENSG00000182141	20371350	validated
mirtarbase	hsa-miR-222-3p	ZNF708	7562	ENSG00000182141	23622248	validated
mirtarbase	hsa-miR-181a-5p	ZNF23	7571	ENSG00000167377	20371350	validated
mirtarbase	hsa-miR-301a-3p	ZNF24	7572	ENSG00000172466	23592263	validated
mirtarbase	hsa-miR-18a-5p	ZNF33A	7581	ENSG00000189180	20371350	validated
mirtarbase	hsa-miR-181a-5p	ZNF35	7584	ENSG00000169981	20371350	validated
mirtarbase	hsa-miR-21-5p	ZNF35	7584	ENSG00000169981	20371350	validated
mirtarbase	hsa-miR-106b-5p	ZNF35	7584	ENSG00000169981	20371350	validated
mirtarbase	hsa-miR-192-5p	ZNF45	7596	ENSG00000124459	19074876	validated
mirtarbase	hsa-miR-106b-5p	ZBTB25	7597	ENSG00000089775	23313552	validated
mirtarbase	hsa-miR-106b-5p	ZNF70	7621	ENSG00000187792	23592263	validated
mirtarbase	hsa-miR-192-5p	ZNF85	7639	ENSG00000278091	19074876	validated
mirtarbase	hsa-miR-15b-5p	ZNF91	7644	ENSG00000167232	22473208	validated
mirtarbase	hsa-miR-181a-5p	ZNF121	7675	ENSG00000197961	22473208	validated
mirtarbase	hsa-miR-582-5p	ZNF124	7678	ENSG00000196418	27292025	validated
mirtarbase	hsa-miR-222-3p	ZNF131	7690	ENSG00000172262	23622248	validated
mirtarbase	hsa-miR-18a-5p	ZNF132	7691	ENSG00000131849	23446348	validated
mirtarbase	hsa-miR-181a-5p	ZNF136	7695	ENSG00000196646	22473208	validated
mirtarbase	hsa-miR-21-5p	PCGF2	7703	ENSG00000277258	27542229	validated
mirtarbase	hsa-miR-15b-5p	ZBTB16	7704	ENSG00000109906	20371350	validated
mirtarbase	hsa-miR-181a-5p	ZNF148	7707	ENSG00000163848	20371350	validated
mirtarbase	hsa-miR-301a-3p	ZNF154	7710	ENSG00000179909	23446348	validated
mirtarbase	hsa-miR-326	ZNF154	7710	ENSG00000179909	19536157	validated
mirtarbase	hsa-miR-192-5p	ZNF165	7718	ENSG00000197279	19074876	validated
mirtarbase	hsa-miR-106b-5p	ZNF174	7727	ENSG00000103343	23313552	validated
mirtarbase	hsa-miR-106b-5p	ZNF180	7733	ENSG00000167384	23446348	validated
mirtarbase	hsa-miR-192-5p	ZNF184	7738	ENSG00000096654	19074876	validated
mirtarbase	hsa-miR-21-5p	ZMYM2	7750	ENSG00000121741	18591254	validated
mirtarbase	hsa-miR-192-5p	ZNF200	7752	ENSG00000010539	19074876	validated
mirtarbase	hsa-miR-106b-5p	ZNF202	7753	ENSG00000166261	23446348	validated
mirtarbase	hsa-miR-21-5p	ZNF207	7756	ENSG00000010244	18591254	validated
mirtarbase	hsa-miR-144-3p	ZNF207	7756	ENSG00000010244	20371350	validated
mirtarbase	hsa-miR-505-3p	ZNF208	7757	ENSG00000160321	23824327	validated
mirtarbase	hsa-miR-192-5p	ZNF215	7762	ENSG00000149054	19074876	validated
mirtarbase	hsa-miR-18a-5p	ZFAND5	7763	ENSG00000107372	20371350	validated
mirtarbase	hsa-miR-15b-5p	ZFAND5	7763	ENSG00000107372	23622248	validated
mirtarbase	hsa-miR-222-3p	ZFAND5	7763	ENSG00000107372	22815788	validated
mirtarbase	hsa-miR-21-5p	ZNF217	7764	ENSG00000171940	18591254	validated
mirtarbase	hsa-miR-15b-5p	ZNF217	7764	ENSG00000171940	23622248	validated
mirtarbase	hsa-miR-582-5p	ZNF223	7766	ENSG00000178386	22012620	validated
mirtarbase	hsa-miR-301a-3p	ZNF224	7767	ENSG00000267680	21572407	validated
mirtarbase	hsa-miR-192-5p	ZNF226	7769	ENSG00000167380	19074876	validated
mirtarbase	hsa-miR-192-5p	ZNF230	7773	ENSG00000159882	19074876	validated
mirtarbase	hsa-miR-222-3p	ZNF236	7776	ENSG00000130856	19536157	validated
mirtarbase	hsa-miR-106b-5p	SLC30A1	7779	ENSG00000170385	23592263	validated
mirtarbase	hsa-miR-15b-5p	LUZP1	7798	ENSG00000169641	23446348	validated
mirtarbase	hsa-miR-296-5p	LUZP1	7798	ENSG00000169641	23446348	validated
mirtarbase	hsa-miR-144-3p	PTP4A1	7803	ENSG00000112245	23592263	validated
mirtarbase	hsa-miR-106b-5p	PTP4A1	7803	ENSG00000112245	23592263	validated
mirtarbase	hsa-miR-192-5p	LRP8	7804	ENSG00000157193	19074876	validated
mirtarbase	hsa-miR-326	CSDE1	7812	ENSG00000009307	23622248	validated
mirtarbase	hsa-miR-106b-5p	CSDE1	7812	ENSG00000009307	21572407	validated
mirtarbase	hsa-miR-15b-5p	CSDE1	7812	ENSG00000009307	22473208	validated
mirtarbase	hsa-miR-106b-5p	DAP3	7818	ENSG00000132676	23622248	validated
mirtarbase	hsa-miR-15b-5p	BTG2	7832	ENSG00000159388	22473208	validated
mirtarbase	hsa-miR-144-3p	BTG2	7832	ENSG00000159388	24398324	validated
mirtarbase	hsa-miR-106b-5p	BTG2	7832	ENSG00000159388	23592263	validated
mirtarbase	hsa-miR-192-5p	PXDN	7837	ENSG00000130508	19074876	validated

mirtarbase	hsa-miR-21-5p	ALMS1	7840	ENSG00000116127	18591254	validated
mirtarbase	hsa-miR-21-5p	RNF103	7844	ENSG00000239305	22473208	validated
mirtarbase	hsa-miR-505-3p	FZD5	7855	ENSG00000163251	22100165	validated
mirtarbase	hsa-miR-21-5p	SLMAP	7871	ENSG00000163681	18591254	validated
mirtarbase	hsa-miR-192-5p	MANF	7873	ENSG00000145050	19074876	validated
mirtarbase	hsa-miR-21-5p	USP7	7874	ENSG00000187555	18591254	validated
mirtarbase	hsa-miR-106b-5p	REEP5	7905	ENSG00000129625	22473208	validated
mirtarbase	hsa-miR-192-5p	DEK	7913	ENSG00000124795	19074876	validated
mirtarbase	hsa-miR-192-5p	ALDH5A1	7915	ENSG00000112294	19074876	validated
mirtarbase	hsa-miR-222-3p	PRRC2A	7916	ENSG00000226618	23622248	validated
mirtarbase	hsa-miR-222-3p	BAG6	7917	ENSG00000233348	23622248	validated
mirtarbase	hsa-miR-192-5p	EPM2A	7957	ENSG00000112425	19074876	validated
mirtarbase	hsa-miR-21-5p	EPM2A	7957	ENSG00000112425	18591254	validated
mirtarbase	hsa-miR-15b-5p	MAFK	7975	ENSG00000198517	22473208	validated
mirtarbase	hsa-miR-326	MAFK	7975	ENSG00000198517	26701625	validated
mirtarbase	hsa-miR-181a-5p	TUSC3	7991	ENSG00000104723	28288641	validated
mirtarbase	hsa-miR-21-5p	KAT6A	7994	ENSG00000083168	20371350	validated
mirtarbase	hsa-miR-192-5p	BRD3	8019	ENSG00000169925	16822819	validated
mirtarbase	hsa-miR-15b-5p	NUP214	8021	ENSG00000126883	23622248	validated
mirtarbase	hsa-miR-181a-5p	DGS2	8026		24398324	validated
mirtarbase	hsa-miR-505-3p	DGS2	8026		23313552	validated
mirtarbase	hsa-miR-192-5p	STAM	8027	ENSG00000136738	19074876	validated
mirtarbase	hsa-miR-181a-5p	CCDC6	8030	ENSG00000108091	17612493	validated
mirtarbase	hsa-miR-106b-5p	CCDC6	8030	ENSG00000108091	20371350	validated
mirtarbase	hsa-miR-106b-5p	SHOC2	8036	ENSG00000108061	23592263	validated
mirtarbase	hsa-miR-15b-5p	SHOC2	8036	ENSG00000108061	22473208	validated
mirtarbase	hsa-miR-181a-5p	SHOC2	8036	ENSG00000108061	22473208	validated
mirtarbase	hsa-miR-192-5p	CUL5	8065	ENSG00000166266	19074876	validated
mirtarbase	hsa-miR-181a-5p	CUL5	8065	ENSG00000166266	22473208	validated
mirtarbase	hsa-miR-106b-5p	PTP4A2	8073	ENSG00000184007	23622248	validated
mirtarbase	hsa-miR-106b-5p	KMT2D	8085	ENSG00000167548	23622248	validated
mirtarbase	hsa-miR-15b-5p	KMT2D	8085	ENSG00000167548	23622248	validated
mirtarbase	hsa-miR-222-3p	KMT2D	8085	ENSG00000167548	23622248	validated
mirtarbase	hsa-miR-212-3p	FXR1	8087	ENSG00000114416	23446348	validated
mirtarbase	hsa-miR-301a-3p	FXR1	8087	ENSG00000114416	26701625	validated
mirtarbase	hsa-miR-181a-5p	HMGA2	8091	ENSG00000149948	21572407	validated
mirtarbase	hsa-miR-326	HMGA2	8091	ENSG00000149948	26701625	validated
mirtarbase	hsa-miR-21-5p	CDK2AP1	8099	ENSG00000111328	21328460	validated
mirtarbase	hsa-miR-144-3p	PABPN1	8106	ENSG00000100836	23592263	validated
mirtarbase	hsa-miR-21-5p	ANP32A	8125	ENSG00000140350	21317927	validated
mirtarbase	hsa-miR-582-3p	GAN	8139	ENSG00000261609	23824327	validated
mirtarbase	hsa-miR-582-5p	GAN	8139	ENSG00000261609	27292025	validated
mirtarbase	hsa-miR-15b-5p	SLC7A5	8140	ENSG00000103257	22473208	validated
mirtarbase	hsa-miR-296-5p	SLC7A5	8140	ENSG00000103257	26701625	validated
mirtarbase	hsa-miR-181a-5p	TAF15	8148	ENSG00000276833	17612493	validated
mirtarbase	hsa-miR-15b-5p	COIL	8161	ENSG00000121058	23622248	validated
mirtarbase	hsa-miR-222-3p	COIL	8161	ENSG00000121058	23622248	validated
mirtarbase	hsa-miR-106b-5p	COIL	8161	ENSG00000121058	23592263	validated
mirtarbase	hsa-miR-212-3p	DPF1	8193	ENSG00000011332	23622248	validated
mirtarbase	hsa-miR-222-3p	MKKS	8195	ENSG00000125863	22012620	validated
mirtarbase	hsa-miR-21-5p	GDF5	8200	ENSG00000125965	24577233	validated
mirtarbase	hsa-miR-21-5p	NCOA3	8202	ENSG00000124151	19528081	validated
mirtarbase	hsa-miR-18a-5p	NCOA3	8202	ENSG00000124151	19706389	validated
mirtarbase	hsa-miR-192-5p	NCOA3	8202	ENSG00000124151	19074876	validated
mirtarbase	hsa-miR-106b-5p	NCOA3	8202	ENSG00000124151	22473208	validated
mirtarbase	hsa-miR-181a-5p	NCOA3	8202	ENSG00000124151	22473208	validated
mirtarbase	hsa-miR-15b-5p	NRIP1	8204	ENSG00000180530	23622248	validated
mirtarbase	hsa-miR-15b-5p	LZTR1	8216	ENSG00000099949	23622248	validated
mirtarbase	hsa-miR-582-3p	ESS2	8220	ENSG00000100056	26701625	validated
mirtarbase	hsa-miR-106b-5p	PNPLA4	8228	ENSG00000006757	22473208	validated
mirtarbase	hsa-miR-222-3p	USP9X	8239	ENSG00000124486	23622248	validated
mirtarbase	hsa-miR-222-3p	RBM10	8241	ENSG00000182872	23622248	validated
mirtarbase	hsa-miR-212-3p	KDM5C	8242	ENSG00000126012	23592263	validated
mirtarbase	hsa-miR-21-5p	SMC1A	8243	ENSG00000072501	18591254	validated
mirtarbase	hsa-miR-301a-3p	SLC10A3	8273	ENSG00000126903	27292025	validated
mirtarbase	hsa-miR-15b-5p	TKTL1	8277	ENSG00000007350	22473208	validated
mirtarbase	hsa-miR-144-3p	ARID1A	8289	ENSG00000117713	20371350	validated
mirtarbase	hsa-miR-222-3p	ARID1A	8289	ENSG00000117713	26909602	validated
mirtarbase	hsa-miR-106b-5p	SERF1B	8293	ENSG00000277429	22012620	validated
mirtarbase	hsa-miR-106b-5p	SERF1A	8293	ENSG00000275581	22012620	validated
mirtarbase	hsa-miR-106b-5p	TRRAP	8295	ENSG00000196367	23622248	validated

mirtarbase	hsa-miR-181a-5p	KLRC4	8302	ENSG00000183542	17612493	validated
mirtarbase	hsa-miR-15b-5p	AXIN2	8313	ENSG00000168646	21501592	validated
mirtarbase	hsa-miR-582-3p	AXIN2	8313	ENSG00000168646	26468775	validated
mirtarbase	hsa-miR-192-5p	CDC7	8317	ENSG00000097046	19074876	validated
mirtarbase	hsa-miR-106b-5p	EOMES	8320	ENSG00000163508	20709030	validated
mirtarbase	hsa-miR-192-5p	FZD1	8321	ENSG00000157240	19074876	validated
mirtarbase	hsa-miR-192-5p	FZD4	8322	ENSG00000174804	19074876	validated
mirtarbase	hsa-miR-301a-3p	FZD6	8323	ENSG00000164930	23592263	validated
mirtarbase	hsa-miR-144-3p	FZD6	8323	ENSG00000164930	24398324	validated
mirtarbase	hsa-miR-15b-5p	FZD6	8323	ENSG00000164930	24398324	validated
mirtarbase	hsa-miR-212-3p	FZD6	8323	ENSG00000164930	23592263	validated
mirtarbase	hsa-miR-21-5p	FZD6	8323	ENSG00000164930	27347343	validated
mirtarbase	hsa-miR-192-5p	FZD7	8324	ENSG00000155760	19074876	validated
mirtarbase	hsa-miR-106b-5p	FZD9	8326	ENSG00000188763	23446348	validated
mirtarbase	hsa-miR-15b-5p	FZD9	8326	ENSG00000188763	20371350	validated
mirtarbase	hsa-miR-106b-5p	H2BC8	8339	ENSG00000273802	23824327	validated
mirtarbase	hsa-miR-15b-5p	H2BC21	8349	ENSG00000184678	21572407	validated
mirtarbase	hsa-miR-181a-5p	H3C4	8351	ENSG00000197409	22473208	validated
mirtarbase	hsa-miR-144-3p	H3C2	8358	ENSG00000286522	23313552	validated
mirtarbase	hsa-miR-505-3p	H4C4	8360	ENSG00000277157	23622248	validated
mirtarbase	hsa-miR-222-3p	PIP5K1A	8394	ENSG00000143398	23622248	validated
mirtarbase	hsa-miR-192-5p	PIP4K2B	8396	ENSG00000277292	19074876	validated
mirtarbase	hsa-miR-106b-5p	ULK1	8408	ENSG00000177169	21572407	validated
mirtarbase	hsa-miR-181a-5p	ULK1	8408	ENSG00000177169	22473208	validated
mirtarbase	hsa-miR-192-5p	EEA1	8411	ENSG00000102189	19074876	validated
mirtarbase	hsa-miR-106b-5p	EEA1	8411	ENSG00000102189	22473208	validated
mirtarbase	hsa-miR-192-5p	STX7	8417	ENSG00000079950	16822819	validated
mirtarbase	hsa-miR-505-3p	LTBP4	8425	ENSG00000090006	26701625	validated
mirtarbase	hsa-miR-222-3p	RECK	8434	ENSG00000122707	22321642	validated
mirtarbase	hsa-miR-505-3p	NCK2	8440	ENSG00000071051	23592263	validated
mirtarbase	hsa-miR-222-3p	DYRK3	8444	ENSG00000143479	23592263	validated
mirtarbase	hsa-miR-15b-5p	DYRK3	8444	ENSG00000143479	23592263	validated
mirtarbase	hsa-miR-106b-5p	DYRK2	8445	ENSG00000127334	21572407	validated
mirtarbase	hsa-miR-582-5p	DYRK2	8445	ENSG00000127334	21572407	validated
mirtarbase	hsa-miR-18a-5p	CUL4A	8451	ENSG00000139842	23622248	validated
mirtarbase	hsa-miR-192-5p	CUL3	8452	ENSG00000036257	16822819	validated
mirtarbase	hsa-miR-301a-3p	CUL3	8452	ENSG00000036257	22012620	validated
mirtarbase	hsa-miR-15b-5p	CUL3	8452	ENSG00000036257	23824327	validated
mirtarbase	hsa-miR-15b-5p	CUL2	8453	ENSG00000108094	22473208	validated
mirtarbase	hsa-miR-192-5p	TTF2	8458	ENSG00000116830	19074876	validated
mirtarbase	hsa-miR-15b-5p	SUPT3H	8464	ENSG00000196284	23622248	validated
mirtarbase	hsa-miR-144-3p	SMARCA5	8467	ENSG00000153147	20371350	validated
mirtarbase	hsa-miR-222-3p	IRS4	8471	ENSG00000133124	23622248	validated
mirtarbase	hsa-miR-18a-5p	IRS4	8471	ENSG00000133124	23622248	validated
mirtarbase	hsa-miR-505-3p	IRS4	8471	ENSG00000133124	23824327	validated
mirtarbase	hsa-miR-15b-5p	OGT	8473	ENSG00000147162	22473208	validated
mirtarbase	hsa-miR-15b-5p	RAE1	8480	ENSG00000101146	23622248	validated
mirtarbase	hsa-miR-106b-5p	SEMA7A	8482	ENSG00000138623	23592263	validated
mirtarbase	hsa-miR-181a-5p	RGS5	8490	ENSG00000143248	23308108	validated
mirtarbase	hsa-miR-15b-5p	PPM1D	8493	ENSG00000170836	25092292	validated
mirtarbase	hsa-miR-21-5p	PPFIA4	8497	ENSG00000143847	18591254	validated
mirtarbase	hsa-miR-192-5p	RANBP3	8498	ENSG00000031823	16822819	validated
mirtarbase	hsa-miR-192-5p	PKP4	8502	ENSG00000144283	19074876	validated
mirtarbase	hsa-miR-192-5p	ENC1	8507	ENSG00000171617	19074876	validated
mirtarbase	hsa-miR-301a-3p	IFITM1	8519	ENSG00000185885	27292025	validated
mirtarbase	hsa-miR-106b-5p	DGKD	8527	ENSG00000077044	23622248	validated
mirtarbase	hsa-miR-18a-5p	YBX3	8531	ENSG00000060138	23622248	validated
mirtarbase	hsa-miR-181a-5p	CBX4	8535	ENSG00000141582	23592263	validated
mirtarbase	hsa-miR-21-5p	CBX4	8535	ENSG00000141582	23592263	validated
mirtarbase	hsa-miR-15b-5p	CBX4	8535	ENSG00000141582	22473208	validated
mirtarbase	hsa-miR-106b-5p	MAPKAPK5	8550	ENSG00000089022	19536157	validated
mirtarbase	hsa-miR-301a-3p	MAPKAPK5	8550	ENSG00000089022	27292025	validated
mirtarbase	hsa-miR-15b-5p	BHLHE40	8553	ENSG00000134107	22473208	validated
mirtarbase	hsa-miR-192-5p	CDC14A	8556	ENSG00000079335	19074876	validated
mirtarbase	hsa-miR-301a-3p	CDC14A	8556	ENSG00000079335	27323075	validated
mirtarbase	hsa-miR-192-5p	DEGS1	8560	ENSG00000143753	19074876	validated
mirtarbase	hsa-miR-106b-5p	DEGS1	8560	ENSG00000143753	23824327	validated
mirtarbase	hsa-miR-15b-5p	DENR	8562	ENSG00000139726	23622248	validated
mirtarbase	hsa-miR-181a-5p	MADD	8567	ENSG00000110514	22942087	validated
mirtarbase	hsa-miR-15b-5p	CASK	8573	ENSG00000147044	23446348	validated
mirtarbase	hsa-miR-106b-5p	AKR7A2	8574	ENSG00000053371	23592263	validated

mirtarbase	hsa-miR-192-5p	TMEFF1	8577	ENSG00000241697	19074876	validated
mirtarbase	hsa-miR-18a-5p	TNFSF11	8600	ENSG00000120659	16331254	validated
mirtarbase	hsa-miR-106b-5p	TNFSF11	8600	ENSG00000120659	26053181	validated
mirtarbase	hsa-miR-326	FAM193A	8603	ENSG00000125386	19536157	validated
mirtarbase	hsa-miR-505-3p	SLC25A12	8604	ENSG00000115840	22291592	validated
mirtarbase	hsa-miR-15b-5p	SLC25A12	8604	ENSG00000115840	22473208	validated
mirtarbase	hsa-miR-181a-5p	PLA2G4C	8605	ENSG00000105499	17612493	validated
mirtarbase	hsa-miR-21-5p	PLPP1	8611	ENSG00000067113	18591254	validated
mirtarbase	hsa-miR-181a-5p	PLPP3	8613	ENSG00000162407	21572407	validated
mirtarbase	hsa-miR-15b-5p	PLPP3	8613	ENSG00000162407	21572407	validated
mirtarbase	hsa-miR-582-3p	STC2	8614	ENSG00000113739	22012620	validated
mirtarbase	hsa-miR-106b-5p	RFXANK	8625	ENSG00000064490	22473208	validated
mirtarbase	hsa-miR-21-5p	SOCS1	8651	ENSG00000185338	27811366	validated
mirtarbase	hsa-miR-15b-5p	DDX3Y	8653	ENSG00000067048	22473208	validated
mirtarbase	hsa-miR-222-3p	EIF3B	8662	ENSG00000106263	23622248	validated
mirtarbase	hsa-miR-106b-5p	EIF3F	8665	ENSG00000175390	23622248	validated
mirtarbase	hsa-miR-222-3p	EIF3I	8668	ENSG00000084623	23622248	validated
mirtarbase	hsa-miR-301a-3p	STX16	8675	ENSG00000124222	23592263	validated
mirtarbase	hsa-miR-144-3p	STX16	8675	ENSG00000124222	23592263	validated
mirtarbase	hsa-miR-212-3p	PEA15	8682	ENSG00000162734	20388802	validated
mirtarbase	hsa-miR-106b-5p	PEA15	8682	ENSG00000162734	19536157	validated
mirtarbase	hsa-miR-301a-3p	GALNT4	8693	ENSG00000257594	24398324	validated
mirtarbase	hsa-miR-192-5p	B3GALNT1	8706	ENSG00000169255	16822819	validated
mirtarbase	hsa-miR-21-5p	B3GALNT1	8706	ENSG00000169255	18591254	validated
mirtarbase	hsa-miR-192-5p	ABCC3	8714	ENSG00000108846	16822819	validated
mirtarbase	hsa-miR-181a-5p	NOL4	8715	ENSG00000101746	17612493	validated
mirtarbase	hsa-miR-222-3p	SNX4	8723	ENSG00000114520	21572407	validated
mirtarbase	hsa-miR-181a-5p	EED	8726	ENSG00000074266	22473208	validated
mirtarbase	hsa-miR-106b-5p	GBF1	8729	ENSG00000107862	22473208	validated
mirtarbase	hsa-miR-181a-5p	RNMT	8731	ENSG00000101654	23313552	validated
mirtarbase	hsa-miR-15b-5p	RNMT	8731	ENSG00000101654	22473208	validated
mirtarbase	hsa-miR-192-5p	TNFSF13	8741	ENSG00000161955	19074876	validated
mirtarbase	hsa-miR-222-3p	TNFSF10	8743	ENSG00000121858	18246122	validated
mirtarbase	hsa-miR-15b-5p	TNFSF9	8744	ENSG00000125657	22473208	validated
mirtarbase	hsa-miR-18a-5p	ADAM15	8751	ENSG00000143537	23622248	validated
mirtarbase	hsa-miR-222-3p	ADAM1A	8759		24736554	validated
mirtarbase	hsa-miR-15b-5p	CDS2	8760	ENSG00000101290	22473208	validated
mirtarbase	hsa-miR-192-5p	PABPC4	8761	ENSG00000090621	19074876	validated
mirtarbase	hsa-miR-192-5p	CD164	8763	ENSG00000135535	16822819	validated
mirtarbase	hsa-miR-15b-5p	FADD	8772	ENSG00000168040	23622248	validated
mirtarbase	hsa-miR-15b-5p	NAPG	8774	ENSG00000134265	23592263	validated
mirtarbase	hsa-miR-106b-5p	MTMR1	8776	ENSG00000063601	23622248	validated
mirtarbase	hsa-miR-21-5p	TNFRSF10B	8795	ENSG00000120889	22473208	validated
mirtarbase	hsa-miR-301a-3p	TNFRSF10B	8795	ENSG00000120889	23592263	validated
mirtarbase	hsa-miR-106b-5p	TNFRSF10B	8795	ENSG00000120889	22012620	validated
mirtarbase	hsa-miR-106b-5p	TNFRSF10A	8797	ENSG00000104689	28410209	validated
mirtarbase	hsa-miR-15b-5p	CREG1	8804	ENSG00000143162	22473208	validated
mirtarbase	hsa-miR-181a-5p	CCNK	8812	ENSG00000090061	22473208	validated
mirtarbase	hsa-miR-144-3p	CCNK	8812	ENSG00000090061	21572407	validated
mirtarbase	hsa-miR-192-5p	SAP30	8819	ENSG00000164105	19074876	validated
mirtarbase	hsa-miR-212-3p	SAP30	8819	ENSG00000164105	28380454	validated
mirtarbase	hsa-miR-192-5p	HESX1	8820	ENSG00000163666	19074876	validated
mirtarbase	hsa-miR-222-3p	INPP4B	8821	ENSG00000109452	22815788	validated
mirtarbase	hsa-miR-181a-5p	INPP4B	8821	ENSG00000109452	28224609	validated
mirtarbase	hsa-miR-106b-5p	CES2	8824	ENSG00000172831	23622248	validated
mirtarbase	hsa-miR-192-5p	IQGAP1	8826	ENSG00000140575	19074876	validated
mirtarbase	hsa-miR-181a-5p	NRP1	8829	ENSG00000099250	17612493	validated
mirtarbase	hsa-miR-181a-5p	KAT2B	8850	ENSG00000114166	18728182	validated
mirtarbase	hsa-miR-106b-5p	KAT2B	8850	ENSG00000114166	18728182	validated
mirtarbase	hsa-miR-192-5p	KAT2B	8850	ENSG00000114166	19074876	validated
mirtarbase	hsa-miR-18a-5p	NR1I2	8856	ENSG00000144852	28408657	validated
mirtarbase	hsa-miR-582-5p	LDB1	8861	ENSG00000198728	27418678	validated
mirtarbase	hsa-miR-21-5p	PER3	8863	ENSG00000049246	18591254	validated
mirtarbase	hsa-miR-21-5p	PER2	8864	ENSG00000132326	23824327	validated
mirtarbase	hsa-miR-181a-5p	PER2	8864	ENSG00000132326	22473208	validated
mirtarbase	hsa-miR-15b-5p	SYNJ1	8867	ENSG00000159082	23622248	validated
mirtarbase	hsa-miR-106b-5p	IER3	8870	ENSG00000237155	22473208	validated
mirtarbase	hsa-miR-106b-5p	ARHGEF7	8874	ENSG00000102606	22473208	validated
mirtarbase	hsa-miR-106b-5p	SQSTM1	8878	ENSG00000161011	22473208	validated
mirtarbase	hsa-miR-144-3p	SGPL1	8879	ENSG00000166224	23592263	validated
mirtarbase	hsa-miR-106b-5p	SGPL1	8879	ENSG00000166224	23592263	validated

mirtarbase	hsa-miR-192-5p	FUBP1	8880	ENSG00000162613	19074876	validated
mirtarbase	hsa-miR-21-5p	FUBP1	8880	ENSG00000162613	18591254	validated
mirtarbase	hsa-miR-222-3p	FUBP1	8880	ENSG00000162613	23622248	validated
mirtarbase	hsa-miR-106b-5p	TAX1BP1	8887	ENSG00000106052	22473208	validated
mirtarbase	hsa-miR-222-3p	MCM3AP	8888	ENSG00000160294	23622248	validated
mirtarbase	hsa-miR-106b-5p	EIF2B2	8892	ENSG00000119718	23313552	validated
mirtarbase	hsa-miR-15b-5p	EIF2B2	8892	ENSG00000119718	23824327	validated
mirtarbase	hsa-miR-582-3p	CPNE3	8895	ENSG00000085719	23592263	validated
mirtarbase	hsa-miR-181a-5p	MTMR3	8897	ENSG00000100330	22971574	validated
mirtarbase	hsa-miR-106b-5p	MTMR3	8897	ENSG00000100330	22473208	validated
mirtarbase	hsa-miR-15b-5p	MTMR3	8897	ENSG00000100330	20371350	validated
mirtarbase	hsa-miR-15b-5p	CPNE1	8904	ENSG00000214078	22473208	validated
mirtarbase	hsa-miR-192-5p	AP1S2	8905	ENSG00000182287	19074876	validated
mirtarbase	hsa-miR-181a-5p	AP1M1	8907	ENSG00000072958	23622248	validated
mirtarbase	hsa-miR-21-5p	BCL10	8915	ENSG00000142867	25327529	validated
mirtarbase	hsa-miR-181a-5p	HERC3	8916	ENSG00000138641	17612493	validated
mirtarbase	hsa-miR-192-5p	HERC2	8924	ENSG00000277278	19074876	validated
mirtarbase	hsa-miR-18a-5p	HERC1	8925	ENSG00000103657	23622248	validated
mirtarbase	hsa-miR-181a-5p	PHOX2B	8929	ENSG00000109132	22012620	validated
mirtarbase	hsa-miR-15b-5p	MBD4	8930	ENSG00000129071	21572407	validated
mirtarbase	hsa-miR-582-3p	RTL8C	8933	ENSG00000134590	27292025	validated
mirtarbase	hsa-miR-505-3p	RTL8C	8933	ENSG00000134590	28735896	validated
mirtarbase	hsa-miR-106b-5p	CDK5R2	8941	ENSG00000171450	17242205	validated
mirtarbase	hsa-miR-505-3p	BTRC	8945	ENSG00000166167	22012620	validated
mirtarbase	hsa-miR-15b-5p	BTRC	8945	ENSG00000166167	22473208	validated
mirtarbase	hsa-miR-106b-5p	H2BC11	8970	ENSG00000124635	23313552	validated
mirtarbase	hsa-miR-301a-3p	USP13	8975	ENSG00000058056	21572407	validated
mirtarbase	hsa-miR-301a-3p	WASL	8976	ENSG00000106299	21572407	validated
mirtarbase	hsa-miR-106b-5p	WASL	8976	ENSG00000106299	21572407	validated
mirtarbase	hsa-miR-21-5p	PLOD3	8985	ENSG00000106397	19253296	validated
mirtarbase	hsa-miR-181a-5p	ATP6V0E1	8992	ENSG00000113732	17612493	validated
mirtarbase	hsa-miR-15b-5p	ATP6V0E1	8992	ENSG00000113732	23622248	validated
mirtarbase	hsa-miR-106b-5p	ATP6V0E1	8992	ENSG00000113732	22473208	validated
mirtarbase	hsa-miR-301a-3p	ATP6V0E1	8992	ENSG00000113732	27292025	validated
mirtarbase	hsa-miR-106b-5p	F2RL3	9002	ENSG00000127533	21572407	validated
mirtarbase	hsa-miR-192-5p	MPZL1	9019	ENSG00000197965	19074876	validated
mirtarbase	hsa-miR-106b-5p	MAP3K14	9020	ENSG00000282637	22473208	validated
mirtarbase	hsa-miR-15b-5p	SOCS3	9021	ENSG00000184557	25888955	validated
mirtarbase	hsa-miR-21-5p	BAZ1B	9031	ENSG00000009954	18591254	validated
mirtarbase	hsa-miR-106b-5p	TM4SF5	9032	ENSG00000142484	23824327	validated
mirtarbase	hsa-miR-21-5p	SEMA5A	9037	ENSG00000112902	18591254	validated
mirtarbase	hsa-miR-144-3p	SEMA5A	9037	ENSG00000112902	22100165	validated
mirtarbase	hsa-miR-192-5p	SPAG9	9043	ENSG00000008294	19074876	validated
mirtarbase	hsa-miR-505-3p	BTAF1	9044	ENSG00000095564	23622248	validated
mirtarbase	hsa-miR-15b-5p	BTAF1	9044	ENSG00000095564	23622248	validated
mirtarbase	hsa-miR-181a-5p	RPL14	9045	ENSG00000188846	23622248	validated
mirtarbase	hsa-miR-15b-5p	RPL14	9045	ENSG00000188846	21572407	validated
mirtarbase	hsa-miR-106b-5p	RPL14	9045	ENSG00000188846	23313552	validated
mirtarbase	hsa-miR-301a-3p	GPRC5A	9052	ENSG00000013588	23592263	validated
mirtarbase	hsa-miR-15b-5p	GPRC5A	9052	ENSG00000013588	23824327	validated
mirtarbase	hsa-miR-106b-5p	MAP7	9053	ENSG00000135525	24398324	validated
mirtarbase	hsa-miR-301a-3p	MAP7	9053	ENSG00000135525	24398324	validated
mirtarbase	hsa-miR-222-3p	NFS1	9054	ENSG00000244005	23622248	validated
mirtarbase	hsa-miR-582-3p	NFS1	9054	ENSG00000244005	26701625	validated
mirtarbase	hsa-miR-505-3p	PRC1	9055	ENSG00000198901	23622248	validated
mirtarbase	hsa-miR-18a-5p	PAPSS2	9060	ENSG00000198682	23622248	validated
mirtarbase	hsa-miR-222-3p	DIRAS3	9077	ENSG00000162595	21071579	validated
mirtarbase	hsa-miR-18a-5p	PKMYT1	9088	ENSG00000127564	23622248	validated
mirtarbase	hsa-miR-106b-5p	PKMYT1	9088	ENSG00000127564	22473208	validated
mirtarbase	hsa-miR-222-3p	PIGQ	9091	ENSG00000007541	23622248	validated
mirtarbase	hsa-miR-222-3p	SART1	9092	ENSG00000175467	23622248	validated
mirtarbase	hsa-miR-144-3p	TBX18	9096	ENSG00000112837	23446348	validated
mirtarbase	hsa-miR-192-5p	USP14	9097	ENSG00000101557	19074876	validated
mirtarbase	hsa-miR-212-3p	USP8	9101	ENSG00000138592	23313552	validated
mirtarbase	hsa-miR-15b-5p	MTMR6	9107	ENSG00000139505	23622248	validated
mirtarbase	hsa-miR-15b-5p	MTMR4	9110	ENSG00000108389	22473208	validated
mirtarbase	hsa-miR-192-5p	NMI	9111	ENSG00000123609	19074876	validated
mirtarbase	hsa-miR-21-5p	LATS1	9113	ENSG00000131023	18591254	validated
mirtarbase	hsa-miR-301a-3p	ATP6V0D1	9114	ENSG00000159720	23446348	validated
mirtarbase	hsa-miR-192-5p	SLC16A6	9120	ENSG00000108932	19074876	validated
mirtarbase	hsa-miR-106b-5p	PRPF4	9128	ENSG00000136875	22473208	validated

mirtarbase	hsa-miR-301a-3p	PRPF4	9128	ENSG00000136875	23313552	validated
mirtarbase	hsa-miR-15b-5p	CCNE2	9134	ENSG00000175305	23446348	validated
mirtarbase	hsa-miR-106b-5p	RABEP1	9135	ENSG00000029725	22473208	validated
mirtarbase	hsa-miR-106b-5p	CBFA2T2	9139	ENSG00000078699	23622248	validated
mirtarbase	hsa-miR-582-5p	ATG12	9140	ENSG00000145782	26701625	validated
mirtarbase	hsa-miR-192-5p	SYNGR1	9145	ENSG00000100321	19074876	validated
mirtarbase	hsa-miR-106b-5p	SLC28A1	9154	ENSG00000156222	23446348	validated
mirtarbase	hsa-miR-21-5p	SCAF11	9169	ENSG00000139218	18591254	validated
mirtarbase	hsa-miR-106b-5p	LPAR2	9170	ENSG00000064547	23592263	validated
mirtarbase	hsa-miR-222-3p	ZW10	9183	ENSG00000086827	23622248	validated
mirtarbase	hsa-miR-222-3p	DDX21	9188	ENSG00000165732	23622248	validated
mirtarbase	hsa-miR-106b-5p	ZBED1	9189	ENSG00000214717	19536157	validated
mirtarbase	hsa-miR-192-5p	SLC16A7	9194	ENSG00000118596	19074876	validated
mirtarbase	hsa-miR-18a-5p	ZMYM3	9203	ENSG00000147130	23622248	validated
mirtarbase	hsa-miR-192-5p	LRRFIP1	9208	ENSG00000124831	19074876	validated
mirtarbase	hsa-miR-192-5p	LRRFIP2	9209	ENSG00000093167	19074876	validated
mirtarbase	hsa-miR-15b-5p	LRRFIP2	9209	ENSG00000093167	21572407	validated
mirtarbase	hsa-miR-18a-5p	LARGE1	9215	ENSG00000133424	23622248	validated
mirtarbase	hsa-miR-505-3p	MTA2	9219	ENSG00000149480	23622248	validated
mirtarbase	hsa-miR-222-3p	MTA2	9219	ENSG00000149480	23622248	validated
mirtarbase	hsa-miR-222-3p	NOLC1	9221	ENSG00000166197	23622248	validated
mirtarbase	hsa-miR-192-5p	DLG5	9231	ENSG00000274429	19074876	validated
mirtarbase	hsa-miR-301a-3p	DLG5	9231	ENSG00000274429	23592263	validated
mirtarbase	hsa-miR-15b-5p	TBRG4	9238	ENSG00000136270	23622248	validated
mirtarbase	hsa-miR-144-3p	GPR50	9248	ENSG00000102195	21572407	validated
mirtarbase	hsa-miR-106b-5p	RPS6KA5	9252	ENSG00000100784	22012620	validated
mirtarbase	hsa-miR-301a-3p	RPS6KA5	9252	ENSG00000100784	21572407	validated
mirtarbase	hsa-miR-18a-5p	RPS6KA5	9252	ENSG00000100784	21572407	validated
mirtarbase	hsa-miR-15b-5p	MAPKAPK2	9261	ENSG00000162889	22473208	validated
mirtarbase	hsa-miR-192-5p	STK17B	9262	ENSG00000081320	19074876	validated
mirtarbase	hsa-miR-106b-5p	STK17B	9262	ENSG00000081320	22473208	validated
mirtarbase	hsa-miR-222-3p	PIWIL1	9271	ENSG00000275051	23824327	validated
mirtarbase	hsa-miR-301a-3p	S1PR2	9294	ENSG00000267534	23824327	validated
mirtarbase	hsa-miR-21-5p	SRSF11	9295	ENSG00000116754	18591254	validated
mirtarbase	hsa-miR-21-5p	SOCS6	9306	ENSG00000170677	27570566	validated
mirtarbase	hsa-miR-192-5p	CD83	9308	ENSG00000112149	19074876	validated
mirtarbase	hsa-miR-192-5p	MMP20	9313	ENSG00000137674	19074876	validated
mirtarbase	hsa-miR-192-5p	NREP	9315	ENSG00000134986	19074876	validated
mirtarbase	hsa-miR-181a-5p	COPS2	9318	ENSG00000166200	17612493	validated
mirtarbase	hsa-miR-192-5p	TRIP13	9319	ENSG00000071539	19074876	validated
mirtarbase	hsa-miR-106b-5p	TRIP12	9320	ENSG00000153827	23622248	validated
mirtarbase	hsa-miR-106b-5p	TRIP10	9322	ENSG00000125733	23592263	validated
mirtarbase	hsa-miR-192-5p	ZNHIT3	9326	ENSG00000278574	19074876	validated
mirtarbase	hsa-miR-222-3p	GTF3C5	9328	ENSG00000148308	23622248	validated
mirtarbase	hsa-miR-192-5p	B4GALT6	9331	ENSG00000118276	19074876	validated
mirtarbase	hsa-miR-222-3p	TCEAL1	9338	ENSG00000172465	21226887	validated
mirtarbase	hsa-miR-106b-5p	TCEAL1	9338	ENSG00000172465	21283757	validated
mirtarbase	hsa-miR-15b-5p	GLP2R	9340	ENSG00000065325	23706177	validated
mirtarbase	hsa-miR-505-3p	SNAP29	9342	ENSG00000099940	23622248	validated
mirtarbase	hsa-miR-15b-5p	EFTUD2	9343	ENSG00000108883	23592263	validated
mirtarbase	hsa-miR-15b-5p	UBE4A	9354	ENSG00000110344	22473208	validated
mirtarbase	hsa-miR-326	UBE4A	9354	ENSG00000110344	23592263	validated
mirtarbase	hsa-miR-15b-5p	PPIG	9360	ENSG00000138398	23446348	validated
mirtarbase	hsa-miR-301a-3p	PPIG	9360	ENSG00000138398	23446348	validated
mirtarbase	hsa-miR-222-3p	KIF3B	9371	ENSG00000101350	23622248	validated
mirtarbase	hsa-miR-181a-5p	KIF3B	9371	ENSG00000101350	22473208	validated
mirtarbase	hsa-miR-15b-5p	KIF3B	9371	ENSG00000101350	22473208	validated
mirtarbase	hsa-miR-222-3p	ZFYVE9	9372	ENSG00000157077	23622248	validated
mirtarbase	hsa-miR-106b-5p	ZFYVE9	9372	ENSG00000157077	23592263	validated
mirtarbase	hsa-miR-301a-3p	ZFYVE9	9372	ENSG00000157077	23592263	validated
mirtarbase	hsa-miR-15b-5p	TM9SF2	9375	ENSG00000125304	22473208	validated
mirtarbase	hsa-miR-181a-5p	TGFBRAP1	9392	ENSG00000135966	22942087	validated
mirtarbase	hsa-miR-181a-5p	NMT2	9397	ENSG00000152465	22473208	validated
mirtarbase	hsa-miR-192-5p	ARHGAP29	9411	ENSG00000137962	19074876	validated
mirtarbase	hsa-miR-192-5p	MED21	9412	ENSG00000152944	22100165	validated
mirtarbase	hsa-miR-192-5p	FAM189A2	9413	ENSG00000135063	19074876	validated
mirtarbase	hsa-miR-192-5p	TJP2	9414	ENSG00000119139	19074876	validated
mirtarbase	hsa-miR-106b-5p	DDX23	9416	ENSG00000174243	23622248	validated
mirtarbase	hsa-miR-106b-5p	ZNF264	9422	ENSG00000083844	22012620	validated
mirtarbase	hsa-miR-106b-5p	KCNK6	9424	ENSG00000099337	23313552	validated
mirtarbase	hsa-miR-192-5p	ABCG2	9429	ENSG00000118777	16822819	validated

mirtarbase	hsa-miR-181a-5p	ABCG2	9429	ENSG00000118777	23780685	validated
mirtarbase	hsa-miR-222-3p	ABCG2	9429	ENSG00000118777	26517090	validated
mirtarbase	hsa-miR-212-3p	ABCG2	9429	ENSG00000118777	25766675	validated
mirtarbase	hsa-miR-106b-5p	MED17	9440	ENSG00000042429	22473208	validated
mirtarbase	hsa-miR-222-3p	QKI	9444	ENSG00000112531	23622248	validated
mirtarbase	hsa-miR-106b-5p	QKI	9444	ENSG00000112531	23446348	validated
mirtarbase	hsa-miR-301a-3p	QKI	9444	ENSG00000112531	23446348	validated
mirtarbase	hsa-miR-181a-5p	MAP4K4	9448	ENSG00000071054	22942087	validated
mirtarbase	hsa-miR-192-5p	EIF2AK3	9451	ENSG00000172071	19074876	validated
mirtarbase	hsa-miR-192-5p	AKAP7	9465	ENSG00000118507	19074876	validated
mirtarbase	hsa-miR-106b-5p	SH3BP5	9467	ENSG00000131370	23313552	validated
mirtarbase	hsa-miR-181a-5p	ATG5	9474	ENSG00000057663	23322078	validated
mirtarbase	hsa-miR-296-5p	PSMF1	9491	ENSG00000125818	19536157	validated
mirtarbase	hsa-miR-106b-5p	KIF23	9493	ENSG00000137807	17242205	validated
mirtarbase	hsa-miR-192-5p	KIF23	9493	ENSG00000137807	19074876	validated
mirtarbase	hsa-miR-15b-5p	KIF23	9493	ENSG00000137807	22473208	validated
mirtarbase	hsa-miR-181a-5p	TBX4	9496	ENSG00000121075	17612493	validated
mirtarbase	hsa-miR-106b-5p	SLC4A7	9497	ENSG00000033867	22473208	validated
mirtarbase	hsa-miR-15b-5p	LITAF	9516	ENSG00000189067	22473208	validated
mirtarbase	hsa-miR-106b-5p	SPTLC2	9517	ENSG00000100596	22012620	validated
mirtarbase	hsa-miR-18a-5p	TBPL1	9519	ENSG00000028839	23592263	validated
mirtarbase	hsa-miR-15b-5p	TBPL1	9519	ENSG00000028839	22473208	validated
mirtarbase	hsa-miR-15b-5p	GOSR1	9527	ENSG00000108587	21572407	validated
mirtarbase	hsa-miR-15b-5p	BAG4	9530	ENSG00000156735	20371350	validated
mirtarbase	hsa-miR-181a-5p	BAG2	9532	ENSG00000112208	17612493	validated
mirtarbase	hsa-miR-106b-5p	IGDCC3	9543	ENSG00000174498	23622248	validated
mirtarbase	hsa-miR-192-5p	ATP6V1G1	9550	ENSG00000136888	19074876	validated
mirtarbase	hsa-miR-181a-5p	MACROH2A1	9555	ENSG00000113648	23622248	validated
mirtarbase	hsa-miR-21-5p	VPS26A	9559	ENSG00000122958	18591254	validated
mirtarbase	hsa-miR-106b-5p	VPS26A	9559	ENSG00000122958	22473208	validated
mirtarbase	hsa-miR-296-5p	GOSR2	9570	ENSG00000108433	23824327	validated
mirtarbase	hsa-miR-326	GOSR2	9570	ENSG00000108433	23824327	validated
mirtarbase	hsa-miR-106b-5p	CLOCK	9575	ENSG00000134852	17242205	validated
mirtarbase	hsa-miR-21-5p	CLOCK	9575	ENSG00000134852	18591254	validated
mirtarbase	hsa-miR-21-5p	PREPL	9581	ENSG00000138078	18591254	validated
mirtarbase	hsa-miR-582-3p	ENTPD4	9583	ENSG00000197217	20371350	validated
mirtarbase	hsa-miR-106b-5p	ENTPD4	9583	ENSG00000197217	22927820	validated
mirtarbase	hsa-miR-192-5p	KIF20B	9585	ENSG00000138182	19074876	validated
mirtarbase	hsa-miR-181a-5p	AKAP12	9590	ENSG00000131016	23622248	validated
mirtarbase	hsa-miR-15b-5p	IER2	9592	ENSG00000160888	22473208	validated
mirtarbase	hsa-miR-15b-5p	NCOR2	9612	ENSG00000196498	22473208	validated
mirtarbase	hsa-miR-222-3p	SEC24C	9632	ENSG00000176986	23622248	validated
mirtarbase	hsa-miR-106b-5p	FEZ2	9637	ENSG00000171055	22473208	validated
mirtarbase	hsa-miR-192-5p	ARHGEF10	9639	ENSG00000104728	19074876	validated
mirtarbase	hsa-miR-505-3p	MORF4L2	9643	ENSG00000123562	23622248	validated
mirtarbase	hsa-miR-582-5p	TTC37	9652	ENSG00000198677	20371350	validated
mirtarbase	hsa-miR-144-3p	TTC37	9652	ENSG00000198677	20371350	validated
mirtarbase	hsa-miR-15b-5p	SOCS5	9655	ENSG00000171150	23592263	validated
mirtarbase	hsa-miR-106b-5p	SOCS5	9655	ENSG00000171150	23446348	validated
mirtarbase	hsa-miR-505-3p	SOCS5	9655	ENSG00000171150	23446348	validated
mirtarbase	hsa-miR-505-3p	MDC1	9656	ENSG00000224587	23622248	validated
mirtarbase	hsa-miR-18a-5p	MDC1	9656	ENSG00000224587	23622248	validated
mirtarbase	hsa-miR-505-3p	PDE4DIP	9659	ENSG00000178104	23622248	validated
mirtarbase	hsa-miR-582-5p	PDE4DIP	9659	ENSG00000178104	22012620	validated
mirtarbase	hsa-miR-15b-5p	EIF5B	9669	ENSG00000158417	23622248	validated
mirtarbase	hsa-miR-106b-5p	SLC25A44	9673	ENSG00000160785	21572407	validated
mirtarbase	hsa-miR-505-3p	KDM4A	9682	ENSG00000066135	23622248	validated
mirtarbase	hsa-miR-106b-5p	N4BP1	9683	ENSG00000102921	22473208	validated
mirtarbase	hsa-miR-15b-5p	N4BP1	9683	ENSG00000102921	22473208	validated
mirtarbase	hsa-miR-222-3p	VGLL4	9686	ENSG00000144560	26045994	validated
mirtarbase	hsa-miR-15b-5p	BZW1	9689	ENSG00000082153	23592263	validated
mirtarbase	hsa-miR-106b-5p	BZW1	9689	ENSG00000082153	23592263	validated
mirtarbase	hsa-miR-582-5p	BZW1	9689	ENSG00000082153	23592263	validated
mirtarbase	hsa-miR-144-3p	BZW1	9689	ENSG00000082153	23592263	validated
mirtarbase	hsa-miR-15b-5p	UBE3C	9690	ENSG00000009335	22473208	validated
mirtarbase	hsa-miR-181a-5p	PUM1	9698	ENSG00000134644	20371350	validated
mirtarbase	hsa-miR-106b-5p	CEP57	9702	ENSG00000166037	22473208	validated
mirtarbase	hsa-miR-181a-5p	KIAA0100	9703	ENSG00000007202	23622248	validated
mirtarbase	hsa-miR-18a-5p	KIAA0100	9703	ENSG00000007202	23622248	validated
mirtarbase	hsa-miR-15b-5p	RUBCN	9711	ENSG00000145016	23592263	validated
mirtarbase	hsa-miR-18a-5p	UTP14C	9724	ENSG00000253797	21572407	validated

mirtarbase	hsa-miR-21-5p	SECISBP2L	9728	ENSG00000138593	22815788	validated
mirtarbase	hsa-miR-301a-3p	SECISBP2L	9728	ENSG00000138593	23592263	validated
mirtarbase	hsa-miR-106b-5p	CEP104	9731	ENSG00000116198	23313552	validated
mirtarbase	hsa-miR-21-5p	DOCK4	9732	ENSG00000128512	22158624	validated
mirtarbase	hsa-miR-192-5p	USP34	9736	ENSG00000115464	19074876	validated
mirtarbase	hsa-miR-21-5p	USP34	9736	ENSG00000115464	18591254	validated
mirtarbase	hsa-miR-106b-5p	CCP110	9738	ENSG00000103540	22473208	validated
mirtarbase	hsa-miR-106b-5p	LAPTM4A	9741	ENSG00000068697	22473208	validated
mirtarbase	hsa-miR-15b-5p	ARHGAP32	9743	ENSG00000134909	19536157	validated
mirtarbase	hsa-miR-21-5p	SLK	9748	ENSG00000065613	20371350	validated
mirtarbase	hsa-miR-106b-5p	SLK	9748	ENSG00000065613	22473208	validated
mirtarbase	hsa-miR-21-5p	PHACTR2	9749	ENSG00000112419	18591254	validated
mirtarbase	hsa-miR-181a-5p	PHACTR2	9749	ENSG00000112419	22815788	validated
mirtarbase	hsa-miR-106b-5p	KMT2B	9757	ENSG00000272333	22012620	validated
mirtarbase	hsa-miR-301a-3p	MLEC	9761	ENSG00000110917	21572407	validated
mirtarbase	hsa-miR-144-3p	MLEC	9761	ENSG00000110917	23313552	validated
mirtarbase	hsa-miR-106b-5p	KIAA0513	9764	ENSG00000135709	23592263	validated
mirtarbase	hsa-miR-21-5p	ZFYVE16	9765	ENSG00000039319	18591254	validated
mirtarbase	hsa-miR-106b-5p	ZFYVE16	9765	ENSG00000039319	23622248	validated
mirtarbase	hsa-miR-222-3p	ZFYVE16	9765	ENSG00000039319	23622248	validated
mirtarbase	hsa-miR-106b-5p	SUSD6	9766	ENSG00000100647	22473208	validated
mirtarbase	hsa-miR-21-5p	JADE3	9767	ENSG00000102221	18591254	validated
mirtarbase	hsa-miR-181a-5p	PCLAF	9768	ENSG00000166803	17612493	validated
mirtarbase	hsa-miR-106b-5p	PCLAF	9768	ENSG00000166803	23446348	validated
mirtarbase	hsa-miR-15b-5p	RASSF2	9770	ENSG00000101265	23708386	validated
mirtarbase	hsa-miR-181a-5p	TMEM94	9772	ENSG00000177728	24398324	validated
mirtarbase	hsa-miR-106b-5p	KIAA0232	9778	ENSG00000170871	23824327	validated
mirtarbase	hsa-miR-18a-5p	MATR3	9782	ENSG00000015479	23622248	validated
mirtarbase	hsa-miR-15b-5p	RIMS3	9783	ENSG00000117016	23446348	validated
mirtarbase	hsa-miR-192-5p	DLGAP5	9787	ENSG00000126787	19074876	validated
mirtarbase	hsa-miR-192-5p	MTSS1	9788	ENSG00000170873	19074876	validated
mirtarbase	hsa-miR-15b-5p	MTSS1	9788	ENSG00000170873	25783158	validated
mirtarbase	hsa-miR-505-3p	BMS1	9790	ENSG00000165733	22012620	validated
mirtarbase	hsa-miR-21-5p	CKAP5	9793	ENSG00000175216	18591254	validated
mirtarbase	hsa-miR-18a-5p	CKAP5	9793	ENSG00000175216	20371350	validated
mirtarbase	hsa-miR-15b-5p	CKAP5	9793	ENSG00000175216	23622248	validated
mirtarbase	hsa-miR-15b-5p	PHYHIP	9796	ENSG00000168490	23592263	validated
mirtarbase	hsa-miR-222-3p	TATDN2	9797	ENSG00000157014	23622248	validated
mirtarbase	hsa-miR-181a-5p	DAZAP2	9802	ENSG00000183283	22473208	validated
mirtarbase	hsa-miR-582-5p	DAZAP2	9802	ENSG00000183283	23592263	validated
mirtarbase	hsa-miR-212-3p	DAZAP2	9802	ENSG00000183283	26701625	validated
mirtarbase	hsa-miR-18a-5p	TOMM20	9804	ENSG00000173726	23592263	validated
mirtarbase	hsa-miR-21-5p	SCRN1	9805	ENSG00000136193	18591254	validated
mirtarbase	hsa-miR-296-5p	RNF40	9810	ENSG00000103549	26701625	validated
mirtarbase	hsa-miR-106b-5p	EFCAB14	9813	ENSG00000159658	17242205	validated
mirtarbase	hsa-miR-18a-5p	EFCAB14	9813	ENSG00000159658	23313552	validated
mirtarbase	hsa-miR-181a-5p	EFCAB14	9813	ENSG00000159658	22473208	validated
mirtarbase	hsa-miR-15b-5p	URB2	9816	ENSG00000135763	23622248	validated
mirtarbase	hsa-miR-181a-5p	NUP58	9818	ENSG00000139496	20371350	validated
mirtarbase	hsa-miR-15b-5p	TSC22D2	9819	ENSG00000196428	23592263	validated
mirtarbase	hsa-miR-144-3p	TSC22D2	9819	ENSG00000196428	24906430	validated
mirtarbase	hsa-miR-192-5p	ARHGAP11A	9824	ENSG00000198826	19074876	validated
mirtarbase	hsa-miR-144-3p	SPATA2	9825	ENSG00000158480	24398324	validated
mirtarbase	hsa-miR-15b-5p	TRIM14	9830	ENSG00000106785	28350138	validated
mirtarbase	hsa-miR-106b-5p	MELK	9833	ENSG00000165304	23824327	validated
mirtarbase	hsa-miR-181a-5p	ZEB2	9839	ENSG00000169554	23622248	validated
mirtarbase	hsa-miR-222-3p	ZEB2	9839	ENSG00000169554	24510096	validated
mirtarbase	hsa-miR-144-3p	ZEB2	9839	ENSG00000169554	27785072	validated
mirtarbase	hsa-miR-106b-5p	PLEKHM1	9842	ENSG00000276358	23622248	validated
mirtarbase	hsa-miR-192-5p	GAB2	9846	ENSG00000033327	19074876	validated
mirtarbase	hsa-miR-192-5p	EPM2AIP1	9852	ENSG00000178567	19074876	validated
mirtarbase	hsa-miR-21-5p	EPM2AIP1	9852	ENSG00000178567	22473208	validated
mirtarbase	hsa-miR-15b-5p	EPM2AIP1	9852	ENSG00000178567	21572407	validated
mirtarbase	hsa-miR-192-5p	PPP1R26	9858	ENSG00000196422	19074876	validated
mirtarbase	hsa-miR-106b-5p	CEP170	9859	ENSG00000276725	22100165	validated
mirtarbase	hsa-miR-301a-3p	CEP170	9859	ENSG00000276725	23446348	validated
mirtarbase	hsa-miR-15b-5p	LRIG2	9860	ENSG00000198799	24398324	validated
mirtarbase	hsa-miR-192-5p	PSMD6	9861	ENSG00000163636	19074876	validated
mirtarbase	hsa-miR-505-3p	PJA2	9867	ENSG00000198961	23622248	validated
mirtarbase	hsa-miR-192-5p	SEC24D	9871	ENSG00000150961	19074876	validated
mirtarbase	hsa-miR-15b-5p	TLK1	9874	ENSG00000198586	22473208	validated

mirtarbase	hsa-miR-505-3p	TLK1	9874	ENSG00000198586	19536157	validated
mirtarbase	hsa-miR-582-5p	ZC3H11A	9877	ENSG00000058673	23592263	validated
mirtarbase	hsa-miR-144-3p	ZC3H11A	9877	ENSG00000058673	23592263	validated
mirtarbase	hsa-miR-21-5p	DDX46	9879	ENSG00000145833	18591254	validated
mirtarbase	hsa-miR-106b-5p	ZBTB39	9880	ENSG00000166860	23622248	validated
mirtarbase	hsa-miR-222-3p	POM121	9883	ENSG00000196313	23622248	validated
mirtarbase	hsa-miR-106b-5p	SMG7	9887	ENSG00000116698	23622248	validated
mirtarbase	hsa-miR-15b-5p	TECPR2	9895	ENSG00000196663	22473208	validated
mirtarbase	hsa-miR-301a-3p	FIG4	9896	ENSG00000112367	23824327	validated
mirtarbase	hsa-miR-181a-5p	WASHC5	9897	ENSG00000164961	22473208	validated
mirtarbase	hsa-miR-222-3p	SV2A	9900	ENSG00000159164	23622248	validated
mirtarbase	hsa-miR-301a-3p	KLHL21	9903	ENSG00000162413	20371350	validated
mirtarbase	hsa-miR-15b-5p	AP5Z1	9907	ENSG00000242802	21572407	validated
mirtarbase	hsa-miR-181a-5p	G3BP2	9908	ENSG00000138757	22473208	validated
mirtarbase	hsa-miR-106b-5p	RABGAP1L	9910	ENSG00000152061	23313552	validated
mirtarbase	hsa-miR-18a-5p	SUPT7L	9913	ENSG00000119760	20371350	validated
mirtarbase	hsa-miR-192-5p	FAM20B	9917	ENSG00000116199	19074876	validated
mirtarbase	hsa-miR-21-5p	FAM20B	9917	ENSG00000116199	18591254	validated
mirtarbase	hsa-miR-222-3p	NCAPD2	9918	ENSG00000010292	23622248	validated
mirtarbase	hsa-miR-106b-5p	NCAPD2	9918	ENSG00000010292	22473208	validated
mirtarbase	hsa-miR-301a-3p	NCAPD2	9918	ENSG00000010292	23446348	validated
mirtarbase	hsa-miR-106b-5p	SEC16A	9919	ENSG00000148396	22473208	validated
mirtarbase	hsa-miR-301a-3p	SEC16A	9919	ENSG00000148396	26701625	validated
mirtarbase	hsa-miR-192-5p	KBTBD11	9920	ENSG00000176595	19074876	validated
mirtarbase	hsa-miR-222-3p	RNF10	9921	ENSG00000022840	23622248	validated
mirtarbase	hsa-miR-106b-5p	IQSEC1	9922	ENSG00000144711	22473208	validated
mirtarbase	hsa-miR-222-3p	PAN2	9924	ENSG00000135473	23622248	validated
mirtarbase	hsa-miR-106b-5p	ZBTB5	9925	ENSG00000168795	23592263	validated
mirtarbase	hsa-miR-222-3p	ZBTB5	9925	ENSG00000168795	22012620	validated
mirtarbase	hsa-miR-15b-5p	ZBTB5	9925	ENSG00000168795	22473208	validated
mirtarbase	hsa-miR-181a-5p	LPGAT1	9926	ENSG00000123684	17612493	validated
mirtarbase	hsa-miR-21-5p	LPGAT1	9926	ENSG00000123684	18591254	validated
mirtarbase	hsa-miR-106b-5p	LPGAT1	9926	ENSG00000123684	22473208	validated
mirtarbase	hsa-miR-106b-5p	MFN2	9927	ENSG00000116688	23954742	validated
mirtarbase	hsa-miR-192-5p	KIF14	9928	ENSG00000118193	19074876	validated
mirtarbase	hsa-miR-144-3p	RBM8A	9939	ENSG00000265241	23592263	validated
mirtarbase	hsa-miR-106b-5p	WDR1	9948	ENSG00000071127	22473208	validated
mirtarbase	hsa-miR-181a-5p	AMMECR1	9949	ENSG00000101935	17612493	validated
mirtarbase	hsa-miR-192-5p	AMMECR1	9949	ENSG00000101935	19074876	validated
mirtarbase	hsa-miR-192-5p	HS3ST3B1	9953	ENSG00000125430	19074876	validated
mirtarbase	hsa-miR-21-5p	HS3ST3B1	9953	ENSG00000125430	20371350	validated
mirtarbase	hsa-miR-212-3p	HS3ST3B1	9953	ENSG00000125430	23446348	validated
mirtarbase	hsa-miR-106b-5p	HS3ST1	9957	ENSG00000002587	23313552	validated
mirtarbase	hsa-miR-222-3p	USP15	9958	ENSG00000135655	23622248	validated
mirtarbase	hsa-miR-15b-5p	USP15	9958	ENSG00000135655	20371350	validated
mirtarbase	hsa-miR-106b-5p	USP3	9960	ENSG00000140455	22473208	validated
mirtarbase	hsa-miR-15b-5p	USP3	9960	ENSG00000140455	22473208	validated
mirtarbase	hsa-miR-326	THRAP3	9967	ENSG00000054118	23622248	validated
mirtarbase	hsa-miR-15b-5p	THRAP3	9967	ENSG00000054118	20371350	validated
mirtarbase	hsa-miR-582-3p	CCS	9973	ENSG00000173992	23824327	validated
mirtarbase	hsa-miR-15b-5p	HNRNPDL	9987	ENSG00000152795	23446348	validated
mirtarbase	hsa-miR-21-5p	DMTF1	9988	ENSG00000135164	18591254	validated
mirtarbase	hsa-miR-15b-5p	DMTF1	9988	ENSG00000135164	22473208	validated
mirtarbase	hsa-miR-106b-5p	SLC12A6	9990	ENSG00000140199	27292025	validated
mirtarbase	hsa-miR-21-5p	PTBP3	9991	ENSG00000119314	18591254	validated
mirtarbase	hsa-miR-181a-5p	PTBP3	9991	ENSG00000119314	23824327	validated
mirtarbase	hsa-miR-222-3p	PTBP3	9991	ENSG00000119314	21572407	validated
mirtarbase	hsa-miR-15b-5p	AKT3	10000	ENSG00000275199	23592263	validated
mirtarbase	hsa-miR-222-3p	AKT3	10000	ENSG00000275199	20371350	validated
mirtarbase	hsa-miR-192-5p	ZBTB33	10009	ENSG00000177485	19074876	validated
mirtarbase	hsa-miR-106b-5p	ZBTB33	10009	ENSG00000177485	22473208	validated
mirtarbase	hsa-miR-181a-5p	ZBTB33	10009	ENSG00000177485	22473208	validated
mirtarbase	hsa-miR-15b-5p	ZBTB33	10009	ENSG00000177485	22473208	validated
mirtarbase	hsa-miR-181a-5p	HDAC6	10013	ENSG00000094631	23622248	validated
mirtarbase	hsa-miR-18a-5p	BCL2L10	10017	ENSG00000137875	24184144	validated
mirtarbase	hsa-miR-222-3p	BCL2L11	10018	ENSG00000153094	23446348	validated
mirtarbase	hsa-miR-106b-5p	BCL2L11	10018	ENSG00000153094	23028803	validated
mirtarbase	hsa-miR-181a-5p	BCL2L11	10018	ENSG00000153094	20841506	validated
mirtarbase	hsa-miR-301a-3p	BCL2L11	10018	ENSG00000153094	22628193	validated
mirtarbase	hsa-miR-192-5p	BCL2L11	10018	ENSG00000153094	23592263	validated
mirtarbase	hsa-miR-144-3p	BCL2L11	10018	ENSG00000153094	23592263	validated

mirtarbase	hsa-miR-192-5p	SH2B3	10019	ENSG00000111252	19074876	validated
mirtarbase	hsa-miR-21-5p	GNE	10020	ENSG00000159921	18591254	validated
mirtarbase	hsa-miR-192-5p	HCN4	10021	ENSG00000138622	19074876	validated
mirtarbase	hsa-miR-192-5p	TROAP	10024	ENSG00000135451	19074876	validated
mirtarbase	hsa-miR-106b-5p	MED16	10025	ENSG00000175221	23446348	validated
mirtarbase	hsa-miR-106b-5p	CHAF1A	10036	ENSG00000167670	22473208	validated
mirtarbase	hsa-miR-222-3p	TOM1	10043	ENSG00000100284	23622248	validated
mirtarbase	hsa-miR-296-5p	TOM1	10043	ENSG00000100284	19536157	validated
mirtarbase	hsa-miR-106b-5p	DNAJB6	10049	ENSG00000105993	17242205	validated
mirtarbase	hsa-miR-192-5p	SMC4	10051	ENSG00000113810	19074876	validated
mirtarbase	hsa-miR-181a-5p	UBA2	10054	ENSG00000126261	23622248	validated
mirtarbase	hsa-miR-106b-5p	DNM1L	10059	ENSG00000087470	22473208	validated
mirtarbase	hsa-miR-21-5p	DNM1L	10059	ENSG00000087470	28347692	validated
mirtarbase	hsa-miR-326	ABCF2	10061	ENSG00000033050	23622248	validated
mirtarbase	hsa-miR-106b-5p	SCAMP2	10066	ENSG00000140497	22473208	validated
mirtarbase	hsa-miR-181a-5p	SCAMP2	10066	ENSG00000140497	21572407	validated
mirtarbase	hsa-miR-106b-5p	HUWE1	10075	ENSG00000086758	23622248	validated
mirtarbase	hsa-miR-181a-5p	HUWE1	10075	ENSG00000086758	23622248	validated
mirtarbase	hsa-miR-21-5p	EDIL3	10085	ENSG00000164176	18591254	validated
mirtarbase	hsa-miR-192-5p	ACTR2	10097	ENSG00000138071	19074876	validated
mirtarbase	hsa-miR-21-5p	ACTR2	10097	ENSG00000138071	18591254	validated
mirtarbase	hsa-miR-15b-5p	ACTR2	10097	ENSG00000138071	22473208	validated
mirtarbase	hsa-miR-106b-5p	ACTR2	10097	ENSG00000138071	23446348	validated
mirtarbase	hsa-miR-15b-5p	TSPAN3	10099	ENSG00000140391	23622248	validated
mirtarbase	hsa-miR-301a-3p	TSPAN3	10099	ENSG00000140391	21572407	validated
mirtarbase	hsa-miR-106b-5p	CTDSP2	10106	ENSG00000175215	23622248	validated
mirtarbase	hsa-miR-192-5p	KIF20A	10112	ENSG00000112984	19074876	validated
mirtarbase	hsa-miR-106b-5p	FEM1B	10116	ENSG00000169018	21572407	validated
mirtarbase	hsa-miR-192-5p	ACTR1B	10120	ENSG00000115073	19074876	validated
mirtarbase	hsa-miR-192-5p	ARL4C	10123	ENSG00000188042	19074876	validated
mirtarbase	hsa-miR-296-5p	ARL4C	10123	ENSG00000188042	23824327	validated
mirtarbase	hsa-miR-192-5p	ZNF263	10127	ENSG00000006194	20371350	validated
mirtarbase	hsa-miR-15b-5p	LRPPRC	10128	ENSG00000138095	22473208	validated
mirtarbase	hsa-miR-181a-5p	PDIA6	10130	ENSG00000143870	23313552	validated
mirtarbase	hsa-miR-15b-5p	PDIA6	10130	ENSG00000143870	20371350	validated
mirtarbase	hsa-miR-222-3p	TRAP1	10131	ENSG00000126602	23622248	validated
mirtarbase	hsa-miR-192-5p	AKAP9	10142	ENSG00000127914	16822819	validated
mirtarbase	hsa-miR-21-5p	AKAP9	10142	ENSG00000127914	19242418	validated
mirtarbase	hsa-miR-181a-5p	FAM13A	10144	ENSG00000138640	19536157	validated
mirtarbase	hsa-miR-582-5p	G3BP1	10146	ENSG00000145907	23592263	validated
mirtarbase	hsa-miR-21-5p	ADGRG2	10149	ENSG00000173698	18591254	validated
mirtarbase	hsa-miR-106b-5p	ABI2	10152	ENSG00000138443	23706177	validated
mirtarbase	hsa-miR-222-3p	PLXNC1	10154	ENSG00000136040	26370254	validated
mirtarbase	hsa-miR-505-3p	TRIM28	10155	ENSG00000130726	23622248	validated
mirtarbase	hsa-miR-106b-5p	SERF2	10169	ENSG00000140264	23446348	validated
mirtarbase	hsa-miR-192-5p	LHFPL6	10186	ENSG00000183722	19074876	validated
mirtarbase	hsa-miR-505-3p	ALYREF	10189	ENSG00000183684	23622248	validated
mirtarbase	hsa-miR-222-3p	ALG3	10195	ENSG00000214160	23622248	validated
mirtarbase	hsa-miR-106b-5p	PRMT3	10196	ENSG00000185238	23622248	validated
mirtarbase	hsa-miR-15b-5p	MPHOSPH9	10198	ENSG00000051825	23622248	validated
mirtarbase	hsa-miR-106b-5p	NME6	10201	ENSG00000172113	22012620	validated
mirtarbase	hsa-miR-15b-5p	PATJ	10207	ENSG00000132849	23622248	validated
mirtarbase	hsa-miR-106b-5p	TOPORS	10210	ENSG00000197579	22473208	validated
mirtarbase	hsa-miR-18a-5p	CTDSPL	10217	ENSG00000144677	20371350	validated
mirtarbase	hsa-miR-15b-5p	CTDSPL	10217	ENSG00000144677	21572407	validated
mirtarbase	hsa-miR-181a-5p	CTDSPL	10217	ENSG00000144677	25174404	validated
mirtarbase	hsa-miR-106b-5p	GDF11	10220	ENSG00000135414	21572407	validated
mirtarbase	hsa-miR-144-3p	TRIB1	10221	ENSG00000173334	26701625	validated
mirtarbase	hsa-miR-192-5p	ZNF443	10224	ENSG00000180855	19074876	validated
mirtarbase	hsa-miR-192-5p	MFSD10	10227	ENSG00000109736	16822819	validated
mirtarbase	hsa-miR-106b-5p	STX6	10228	ENSG00000135823	22473208	validated
mirtarbase	hsa-miR-301a-3p	STX6	10228	ENSG00000135823	23592263	validated
mirtarbase	hsa-miR-21-5p	MSLN	10232	ENSG00000102854	28125734	validated
mirtarbase	hsa-miR-181a-5p	LRRC17	10234	ENSG00000128606	17612493	validated
mirtarbase	hsa-miR-18a-5p	HNRNPR	10236	ENSG00000125944	20371350	validated
mirtarbase	hsa-miR-106b-5p	HNRNPR	10236	ENSG00000125944	23446348	validated
mirtarbase	hsa-miR-326	DCAF7	10238	ENSG00000136485	23622248	validated
mirtarbase	hsa-miR-222-3p	DCAF7	10238	ENSG00000136485	23622248	validated
mirtarbase	hsa-miR-18a-5p	DCAF7	10238	ENSG00000136485	23622248	validated
mirtarbase	hsa-miR-18a-5p	AP3S2	10239	ENSG00000157823	20371350	validated
mirtarbase	hsa-miR-301a-3p	POP7	10248	ENSG00000172336	21572407	validated

mirtarbase	hsa-miR-326	SRRM1	10250	ENSG00000133226	23622248	validated
mirtarbase	hsa-miR-21-5p	SPRY2	10253	ENSG00000136158	18508928	validated
mirtarbase	hsa-miR-181a-5p	SPRY2	10253	ENSG00000136158	20371350	validated
mirtarbase	hsa-miR-301a-3p	CDK2AP2	10263	ENSG00000167797	20371350	validated
mirtarbase	hsa-miR-21-5p	STUB1	10273	ENSG00000103266	26841045	validated
mirtarbase	hsa-miR-212-3p	STAG1	10274	ENSG00000118007	22100165	validated
mirtarbase	hsa-miR-181a-5p	STAG1	10274	ENSG00000118007	22473208	validated
mirtarbase	hsa-miR-21-5p	SMNDC1	10285	ENSG00000119953	20371350	validated
mirtarbase	hsa-miR-301a-3p	SF3A1	10291	ENSG00000099995	23824327	validated
mirtarbase	hsa-miR-192-5p	TRAIP	10293	ENSG00000183763	19074876	validated
mirtarbase	hsa-miR-106b-5p	MARCHF6	10299	ENSG00000145495	23446348	validated
mirtarbase	hsa-miR-192-5p	ZNF267	10308	ENSG00000185947	19074876	validated
mirtarbase	hsa-miR-15b-5p	ZNF267	10308	ENSG00000185947	21572407	validated
mirtarbase	hsa-miR-192-5p	CCNO	10309	ENSG00000152669	19074876	validated
mirtarbase	hsa-miR-15b-5p	VPS26C	10311	ENSG00000157538	22473208	validated
mirtarbase	hsa-miR-15b-5p	LANCL1	10314	ENSG00000115365	20371350	validated
mirtarbase	hsa-miR-181a-5p	TNIP1	10318	ENSG00000145901	23622248	validated
mirtarbase	hsa-miR-582-3p	TNIP1	10318	ENSG00000145901	23446348	validated
mirtarbase	hsa-miR-326	PCGF3	10336	ENSG00000185619	26701625	validated
mirtarbase	hsa-miR-192-5p	TFG	10342	ENSG00000114354	16822819	validated
mirtarbase	hsa-miR-192-5p	ABCA8	10351	ENSG00000141338	16822819	validated
mirtarbase	hsa-miR-181a-5p	NPM3	10360	ENSG00000107833	23446348	validated
mirtarbase	hsa-miR-106b-5p	IRF9	10379	ENSG00000213928	17242205	validated
mirtarbase	hsa-miR-505-3p	TUBB3	10381	ENSG00000258947	23622248	validated
mirtarbase	hsa-miR-301a-3p	TUBB4B	10383	ENSG00000188229	23622248	validated
mirtarbase	hsa-miR-106b-5p	TUBB4B	10383	ENSG00000188229	23622248	validated
mirtarbase	hsa-miR-106b-5p	BTN3A3	10384	ENSG00000111801	17242205	validated
mirtarbase	hsa-miR-21-5p	BTN3A3	10384	ENSG00000111801	22473208	validated
mirtarbase	hsa-miR-15b-5p	BTN3A3	10384	ENSG00000111801	21572407	validated
mirtarbase	hsa-miR-192-5p	CORO2B	10391	ENSG00000103647	19074876	validated
mirtarbase	hsa-miR-192-5p	ANAPC10	10393	ENSG00000164162	19074876	validated
mirtarbase	hsa-miR-301a-3p	DLC1	10395	ENSG00000164741	20371350	validated
mirtarbase	hsa-miR-181a-5p	ATP8A1	10396	ENSG00000124406	20371350	validated
mirtarbase	hsa-miR-505-3p	NDRG1	10397	ENSG00000104419	23622248	validated
mirtarbase	hsa-miR-18a-5p	RACK1	10399	ENSG00000204628	23622248	validated
mirtarbase	hsa-miR-21-5p	PIAS3	10401	ENSG00000131788	22316494	validated
mirtarbase	hsa-miR-18a-5p	PIAS3	10401	ENSG00000131788	23322197	validated
mirtarbase	hsa-miR-192-5p	ST3GAL6	10402	ENSG00000064225	19074876	validated
mirtarbase	hsa-miR-21-5p	BASP1	10409	ENSG00000176788	19253296	validated
mirtarbase	hsa-miR-21-5p	TESK2	10420	ENSG00000070759	18591254	validated
mirtarbase	hsa-miR-106b-5p	CDIPT	10423	ENSG00000103502	19536157	validated
mirtarbase	hsa-miR-21-5p	PGRMC2	10424	ENSG00000164040	18591254	validated
mirtarbase	hsa-miR-192-5p	TUBGCP3	10426	ENSG00000126216	19074876	validated
mirtarbase	hsa-miR-18a-5p	SEC24B	10427	ENSG00000138802	23622248	validated
mirtarbase	hsa-miR-21-5p	TMEM147	10430	ENSG00000105677	23622248	validated
mirtarbase	hsa-miR-222-3p	LYPLA1	10434	ENSG00000120992	22815788	validated
mirtarbase	hsa-miR-192-5p	C1D	10438	ENSG00000197223	16822819	validated
mirtarbase	hsa-miR-106b-5p	TIMM17A	10440	ENSG00000134375	22012620	validated
mirtarbase	hsa-miR-106b-5p	N4BP2L2	10443	ENSG00000244754	23706177	validated
mirtarbase	hsa-miR-18a-5p	FAM3C	10447	ENSG00000196937	21572407	validated
mirtarbase	hsa-miR-181a-5p	FAM3C	10447	ENSG00000196937	20371350	validated
mirtarbase	hsa-miR-21-5p	ECI2	10455	ENSG00000198721	20371350	validated
mirtarbase	hsa-miR-222-3p	COG5	10466	ENSG00000164597	22012620	validated
mirtarbase	hsa-miR-106b-5p	PFDN6	10471	ENSG00000224782	23622248	validated
mirtarbase	hsa-miR-192-5p	ZBTB18	10472	ENSG00000179456	19074876	validated
mirtarbase	hsa-miR-106b-5p	ZBTB18	10472	ENSG00000179456	22473208	validated
mirtarbase	hsa-miR-301a-3p	ZBTB18	10472	ENSG00000179456	20371350	validated
mirtarbase	hsa-miR-505-3p	ZBTB18	10472	ENSG00000179456	20371350	validated
mirtarbase	hsa-miR-505-3p	HMGN4	10473	ENSG00000182952	23622248	validated
mirtarbase	hsa-miR-21-5p	TRIM38	10475	ENSG00000112343	18591254	validated
mirtarbase	hsa-miR-21-5p	SLC9A6	10479	ENSG00000198689	18591254	validated
mirtarbase	hsa-miR-15b-5p	SLC9A6	10479	ENSG00000198689	22473208	validated
mirtarbase	hsa-miR-192-5p	SEC23B	10483	ENSG00000101310	19074876	validated
mirtarbase	hsa-miR-301a-3p	SEC23B	10483	ENSG00000101310	21572407	validated
mirtarbase	hsa-miR-106b-5p	SEC23A	10484	ENSG00000100934	22473208	validated
mirtarbase	hsa-miR-15b-5p	LRRC41	10489	ENSG00000132128	23622248	validated
mirtarbase	hsa-miR-18a-5p	LRRC41	10489	ENSG00000132128	23622248	validated
mirtarbase	hsa-miR-192-5p	SYNCRIP	10492	ENSG00000135316	19074876	validated
mirtarbase	hsa-miR-181a-5p	CARM1	10498	ENSG00000142453	22473208	validated
mirtarbase	hsa-miR-15b-5p	CARM1	10498	ENSG00000142453	27292025	validated
mirtarbase	hsa-miR-192-5p	SEMA4D	10507	ENSG00000187764	16822819	validated

mirtarbase	hsa-miR-106b-5p	SEMA4B	10509	ENSG00000185033	22473208	validated
mirtarbase	hsa-miR-144-3p	APPBP2	10513	ENSG00000062725	23592263	validated
mirtarbase	hsa-miR-192-5p	CIB2	10518	ENSG00000136425	19074876	validated
mirtarbase	hsa-miR-106b-5p	DDX17	10521	ENSG00000100201	23622248	validated
mirtarbase	hsa-miR-192-5p	DEAF1	10522	ENSG00000177030	19074876	validated
mirtarbase	hsa-miR-301a-3p	CHERP	10523	ENSG00000085872	19536157	validated
mirtarbase	hsa-miR-15b-5p	HYOU1	10525	ENSG00000149428	22473208	validated
mirtarbase	hsa-miR-106b-5p	IPO7	10527	ENSG00000205339	23622248	validated
mirtarbase	hsa-miR-582-5p	IPO7	10527	ENSG00000205339	22012620	validated
mirtarbase	hsa-miR-222-3p	ZNRD2	10534	ENSG00000173465	26446789	validated
mirtarbase	hsa-miR-18a-5p	DCTN2	10540	ENSG00000175203	23622248	validated
mirtarbase	hsa-miR-192-5p	LAMTOR5	10542	ENSG00000134248	19074876	validated
mirtarbase	hsa-miR-505-3p	TM9SF1	10548	ENSG00000100926	23592263	validated
mirtarbase	hsa-miR-222-3p	PRDX4	10549	ENSG00000123131	23622248	validated
mirtarbase	hsa-miR-106b-5p	RPP30	10556	ENSG00000148688	23622248	validated
mirtarbase	hsa-miR-326	SPTLC1	10558	ENSG00000090054	21572407	validated
mirtarbase	hsa-miR-15b-5p	SPTLC1	10558	ENSG00000090054	22473208	validated
mirtarbase	hsa-miR-192-5p	SLC19A2	10560	ENSG00000117479	19074876	validated
mirtarbase	hsa-miR-181a-5p	SLC19A2	10560	ENSG00000117479	22473208	validated
mirtarbase	hsa-miR-192-5p	ARFGEF1	10565	ENSG00000066777	19074876	validated
mirtarbase	hsa-miR-18a-5p	RABAC1	10567	ENSG00000105404	23622248	validated
mirtarbase	hsa-miR-505-3p	CCT7	10574	ENSG00000135624	23622248	validated
mirtarbase	hsa-miR-144-3p	CCT2	10576	ENSG00000166226	23592263	validated
mirtarbase	hsa-miR-106b-5p	TXNRD2	10587	ENSG00000184470	23622248	validated
mirtarbase	hsa-miR-192-5p	SMC2	10592	ENSG00000136824	19074876	validated
mirtarbase	hsa-miR-222-3p	SMC2	10592	ENSG00000136824	19536157	validated
mirtarbase	hsa-miR-192-5p	TRAPPC2B	10597	ENSG00000256060	18835392	validated
mirtarbase	hsa-miR-222-3p	AHSA1	10598	ENSG00000100591	23622248	validated
mirtarbase	hsa-miR-106b-5p	USP16	10600	ENSG00000156256	22473208	validated
mirtarbase	hsa-miR-505-3p	PAICS	10606	ENSG00000128050	23622248	validated
mirtarbase	hsa-miR-222-3p	HEXIM1	10614	ENSG00000186834	23622248	validated
mirtarbase	hsa-miR-505-3p	STAMBP	10617	ENSG00000124356	23622248	validated
mirtarbase	hsa-miR-144-3p	STAMBP	10617	ENSG00000124356	21572407	validated
mirtarbase	hsa-miR-15b-5p	TGOLN2	10618	ENSG00000152291	23622248	validated
mirtarbase	hsa-miR-106b-5p	TGOLN2	10618	ENSG00000152291	22473208	validated
mirtarbase	hsa-miR-106b-5p	POLR3F	10621	ENSG00000132664	23824327	validated
mirtarbase	hsa-miR-106b-5p	POLR3G	10622	ENSG00000113356	22473208	validated
mirtarbase	hsa-miR-21-5p	IVNS1ABP	10625	ENSG00000116679	18591254	validated
mirtarbase	hsa-miR-15b-5p	IVNS1ABP	10625	ENSG00000116679	23592263	validated
mirtarbase	hsa-miR-106b-5p	TXNIP	10628	ENSG00000265972	22473208	validated
mirtarbase	hsa-miR-301a-3p	TXNIP	10628	ENSG00000265972	24398324	validated
mirtarbase	hsa-miR-18a-5p	TXNIP	10628	ENSG00000265972	23592263	validated
mirtarbase	hsa-miR-15b-5p	TXNIP	10628	ENSG00000265972	27292025	validated
mirtarbase	hsa-miR-192-5p	ATP5MG	10632	ENSG00000167283	19074876	validated
mirtarbase	hsa-miR-18a-5p	RAD51AP1	10635	ENSG00000111247	23592263	validated
mirtarbase	hsa-miR-582-5p	LEFTY1	10637	ENSG00000243709	22012620	validated
mirtarbase	hsa-miR-301a-3p	LEFTY1	10637	ENSG00000243709	22012620	validated
mirtarbase	hsa-miR-144-3p	LEFTY1	10637	ENSG00000243709	22012620	validated
mirtarbase	hsa-miR-21-5p	EXOC5	10640	ENSG00000070367	18591254	validated
mirtarbase	hsa-miR-18a-5p	IGF2BP2	10644	ENSG00000073792	16331254	validated
mirtarbase	hsa-miR-505-3p	SPINT2	10653	ENSG00000167642	27292025	validated
mirtarbase	hsa-miR-15b-5p	DMRT2	10655	ENSG00000173253	22100165	validated
mirtarbase	hsa-miR-505-3p	DMRT2	10655	ENSG00000173253	19536157	validated
mirtarbase	hsa-miR-192-5p	KHDRBS3	10656	ENSG00000131773	19074876	validated
mirtarbase	hsa-miR-192-5p	CTCF	10664	ENSG00000102974	19074876	validated
mirtarbase	hsa-miR-505-3p	CD226	10666	ENSG00000150637	23824327	validated
mirtarbase	hsa-miR-212-3p	CD226	10666	ENSG00000150637	23824327	validated
mirtarbase	hsa-miR-106b-5p	DCTN6	10671	ENSG00000104671	23446348	validated
mirtarbase	hsa-miR-192-5p	TNFSF13B	10673	ENSG00000102524	19074876	validated
mirtarbase	hsa-miR-15b-5p	B3GNT2	10678	ENSG00000170340	21572407	validated
mirtarbase	hsa-miR-106b-5p	GNB5	10681	ENSG00000069966	23313552	validated
mirtarbase	hsa-miR-505-3p	PNMA2	10687	ENSG00000240694	23446348	validated
mirtarbase	hsa-miR-301a-3p	CCT8	10694	ENSG00000156261	23622248	validated
mirtarbase	hsa-miR-15b-5p	TBR1	10716	ENSG00000136535	27499071	validated
mirtarbase	hsa-miR-192-5p	POLQ	10721	ENSG00000051341	19074876	validated
mirtarbase	hsa-miR-106b-5p	POLQ	10721	ENSG00000051341	22473208	validated
mirtarbase	hsa-miR-296-5p	SLC12A7	10723	ENSG00000113504	22012620	validated
mirtarbase	hsa-miR-301a-3p	SLC12A7	10723	ENSG00000113504	22012620	validated
mirtarbase	hsa-miR-21-5p	NFAT5	10725	ENSG00000102908	18591254	validated
mirtarbase	hsa-miR-106b-5p	NFAT5	10725	ENSG00000102908	22473208	validated
mirtarbase	hsa-miR-106b-5p	PTGES3	10728	ENSG00000110958	22473208	validated

mirtarbase	hsa-miR-21-5p	YME1L1	10730	ENSG00000136758	18591254	validated
mirtarbase	hsa-miR-192-5p	YME1L1	10730	ENSG00000136758	23824327	validated
mirtarbase	hsa-miR-192-5p	TCFL5	10732	ENSG00000101190	19074876	validated
mirtarbase	hsa-miR-192-5p	PLK4	10733	ENSG00000142731	19074876	validated
mirtarbase	hsa-miR-21-5p	STAG2	10735	ENSG00000101972	20371350	validated
mirtarbase	hsa-miR-181a-5p	STAG2	10735	ENSG00000101972	23622248	validated
mirtarbase	hsa-miR-192-5p	PHTF1	10745	ENSG00000116793	19074876	validated
mirtarbase	hsa-miR-21-5p	PHTF1	10745	ENSG00000116793	18591254	validated
mirtarbase	hsa-miR-21-5p	MAP3K2	10746	ENSG00000169967	18591254	validated
mirtarbase	hsa-miR-106b-5p	MAP3K2	10746	ENSG00000169967	22473208	validated
mirtarbase	hsa-miR-181a-5p	CHL1	10752	ENSG00000134121	17612493	validated
mirtarbase	hsa-miR-106b-5p	TRAF3IP2	10758	ENSG00000056972	23313552	validated
mirtarbase	hsa-miR-15b-5p	NUP50	10762	ENSG00000093000	22473208	validated
mirtarbase	hsa-miR-212-3p	NUP50	10762	ENSG00000093000	21572407	validated
mirtarbase	hsa-miR-15b-5p	TOB2	10766	ENSG00000183864	23592263	validated
mirtarbase	hsa-miR-192-5p	ZMYND11	10771	ENSG00000015171	19074876	validated
mirtarbase	hsa-miR-222-3p	ZMYND11	10771	ENSG00000015171	23622248	validated
mirtarbase	hsa-miR-296-5p	SRSF10	10772	ENSG00000188529	19536157	validated
mirtarbase	hsa-miR-192-5p	ZBTB6	10773	ENSG00000186130	19074876	validated
mirtarbase	hsa-miR-106b-5p	ZBTB6	10773	ENSG00000186130	22473208	validated
mirtarbase	hsa-miR-181a-5p	ZNF266	10781	ENSG00000174652	21572407	validated
mirtarbase	hsa-miR-582-5p	NCKAP1	10787	ENSG00000061676	23592263	validated
mirtarbase	hsa-miR-15b-5p	NCKAP1	10787	ENSG00000061676	20371350	validated
mirtarbase	hsa-miR-192-5p	ZNF273	10793	ENSG00000198039	19074876	validated
mirtarbase	hsa-miR-222-3p	ZNF460	10794	ENSG00000197714	23622248	validated
mirtarbase	hsa-miR-15b-5p	ZNF460	10794	ENSG00000197714	23592263	validated
mirtarbase	hsa-miR-21-5p	ZNF460	10794	ENSG00000197714	23446348	validated
mirtarbase	hsa-miR-18a-5p	ZNF460	10794	ENSG00000197714	21572407	validated
mirtarbase	hsa-miR-181a-5p	ZNF268	10795	ENSG00000090612	20371350	validated
mirtarbase	hsa-miR-326	MTHFD2	10797	ENSG00000065911	23824327	validated
mirtarbase	hsa-miR-15b-5p	SEC24A	10802	ENSG00000113615	23592263	validated
mirtarbase	hsa-miR-21-5p	FRS2	10818	ENSG00000166225	22012620	validated
mirtarbase	hsa-miR-106b-5p	FRS2	10818	ENSG00000166225	21572407	validated
mirtarbase	hsa-miR-21-5p	FAXDC2	10826	ENSG00000170271	18591254	validated
mirtarbase	hsa-miR-15b-5p	ZNF275	10838	ENSG00000063587	21572407	validated
mirtarbase	hsa-miR-222-3p	ZNF275	10838	ENSG00000063587	21572407	validated
mirtarbase	hsa-miR-106b-5p	SRCAP	10847	ENSG00000080603	23824327	validated
mirtarbase	hsa-miR-106b-5p	HCP5	10866		22473208	validated
mirtarbase	hsa-miR-192-5p	NMU	10874	ENSG00000109255	19074876	validated
mirtarbase	hsa-miR-181a-5p	GPR83	10888	ENSG00000123901	17612493	validated
mirtarbase	hsa-miR-106b-5p	RAB10	10890	ENSG00000084733	21572407	validated
mirtarbase	hsa-miR-21-5p	MALT1	10892	ENSG00000172175	18591254	validated
mirtarbase	hsa-miR-222-3p	JTB	10899	ENSG00000143543	23622248	validated
mirtarbase	hsa-miR-301a-3p	BLCAP	10904	ENSG00000166619	23592263	validated
mirtarbase	hsa-miR-181a-5p	MAN1A2	10905	ENSG00000198162	23446348	validated
mirtarbase	hsa-miR-15b-5p	PNPLA6	10908	ENSG00000032444	23592263	validated
mirtarbase	hsa-miR-181a-5p	GADD45G	10912	ENSG00000130222	17612493	validated
mirtarbase	hsa-miR-222-3p	TCERG1	10915	ENSG00000113649	23622248	validated
mirtarbase	hsa-miR-106b-5p	RNPS1	10921	ENSG00000205937	23622248	validated
mirtarbase	hsa-miR-222-3p	RNPS1	10921	ENSG00000205937	21572407	validated
mirtarbase	hsa-miR-15b-5p	RNPS1	10921	ENSG00000205937	22473208	validated
mirtarbase	hsa-miR-505-3p	SUB1	10923	ENSG00000113387	21572407	validated
mirtarbase	hsa-miR-21-5p	SPIN1	10927	ENSG00000106723	18591254	validated
mirtarbase	hsa-miR-222-3p	MORF4L1	10933	ENSG00000185787	23622248	validated
mirtarbase	hsa-miR-106b-5p	MORF4L1	10933	ENSG00000185787	23446348	validated
mirtarbase	hsa-miR-181a-5p	PRDX3	10935	ENSG00000165672	17612493	validated
mirtarbase	hsa-miR-15b-5p	PRDX3	10935	ENSG00000165672	23622248	validated
mirtarbase	hsa-miR-301a-3p	GPR75	10936	ENSG00000119737	27292025	validated
mirtarbase	hsa-miR-15b-5p	PRSS21	10942	ENSG00000007038	21572407	validated
mirtarbase	hsa-miR-18a-5p	MSL3	10943	ENSG00000005302	23622248	validated
mirtarbase	hsa-miR-301a-3p	KDELR1	10945	ENSG00000105438	23824327	validated
mirtarbase	hsa-miR-192-5p	AP3M2	10947	ENSG00000070718	19074876	validated
mirtarbase	hsa-miR-181a-5p	AP3M2	10947	ENSG00000070718	23824327	validated
mirtarbase	hsa-miR-106b-5p	BTG3	10950	ENSG00000281484	17242205	validated
mirtarbase	hsa-miR-106b-5p	CBX1	10951	ENSG00000108468	21572407	validated
mirtarbase	hsa-miR-301a-3p	SERINC3	10955	ENSG00000132824	23592263	validated
mirtarbase	hsa-miR-301a-3p	PNRC1	10957	ENSG00000146278	23592263	validated
mirtarbase	hsa-miR-106b-5p	PNRC1	10957	ENSG00000146278	23824327	validated
mirtarbase	hsa-miR-192-5p	RAB40B	10966	ENSG00000141542	19074876	validated
mirtarbase	hsa-miR-15b-5p	RAB40B	10966	ENSG00000141542	22473208	validated
mirtarbase	hsa-miR-505-3p	EBNA1BP2	10969	ENSG00000117395	23622248	validated

mirtarbase	hsa-miR-192-5p	CKAP4	10970	ENSG00000136026	19074876	validated
mirtarbase	hsa-miR-15b-5p	YWHAQ	10971	ENSG00000134308	21572407	validated
mirtarbase	hsa-miR-192-5p	TMED10	10972	ENSG00000170348	19074876	validated
mirtarbase	hsa-miR-21-5p	FERMT2	10979	ENSG00000073712	20371350	validated
mirtarbase	hsa-miR-222-3p	GCN1	10985	ENSG00000089154	23622248	validated
mirtarbase	hsa-miR-326	SLC27A4	10999	ENSG00000167114	23824327	validated
mirtarbase	hsa-miR-181a-5p	KIF2C	11004	ENSG00000142945	22473208	validated
mirtarbase	hsa-miR-301a-3p	KDELR2	11014	ENSG00000136240	23622248	validated
mirtarbase	hsa-miR-106b-5p	LIAS	11019	ENSG00000121897	23824327	validated
mirtarbase	hsa-miR-301a-3p	LILRA2	11027	ENSG00000275290	23824327	validated
mirtarbase	hsa-miR-192-5p	TENT4A	11044	ENSG00000112941	19074876	validated
mirtarbase	hsa-miR-582-5p	TENT4A	11044	ENSG00000112941	23592263	validated
mirtarbase	hsa-miR-192-5p	CPSF6	11052	ENSG00000111605	19074876	validated
mirtarbase	hsa-miR-222-3p	CPSF6	11052	ENSG00000111605	20371350	validated
mirtarbase	hsa-miR-505-3p	OGFR	11054	ENSG00000060491	23622248	validated
mirtarbase	hsa-miR-181a-5p	DDX52	11056	ENSG00000278053	22473208	validated
mirtarbase	hsa-miR-106b-5p	ABHD2	11057	ENSG00000140526	22473208	validated
mirtarbase	hsa-miR-15b-5p	ABHD2	11057	ENSG00000140526	23592263	validated
mirtarbase	hsa-miR-21-5p	WWP1	11059	ENSG00000123124	22227207	validated
mirtarbase	hsa-miR-21-5p	CNTRL	11064	ENSG00000119397	18591254	validated
mirtarbase	hsa-miR-582-5p	SNRNP35	11066	ENSG00000184209	27292025	validated
mirtarbase	hsa-miR-106b-5p	RAPGEF4	11069	ENSG00000091428	22473208	validated
mirtarbase	hsa-miR-192-5p	TOPBP1	11073	ENSG00000163781	19074876	validated
mirtarbase	hsa-miR-181a-5p	TOPBP1	11073	ENSG00000163781	23313552	validated
mirtarbase	hsa-miR-106b-5p	TRIOBP	11078	ENSG00000100106	23313552	validated
mirtarbase	hsa-miR-192-5p	DNAJB4	11080	ENSG00000162616	19074876	validated
mirtarbase	hsa-miR-106b-5p	DNAJB4	11080	ENSG00000162616	23313552	validated
mirtarbase	hsa-miR-582-5p	DIDO1	11083	ENSG00000101191	21572407	validated
mirtarbase	hsa-miR-144-3p	DIDO1	11083	ENSG00000101191	21572407	validated
mirtarbase	hsa-miR-296-5p	ADAMTS13	11093	ENSG00000160323	23824327	validated
mirtarbase	hsa-miR-192-5p	PRSS23	11098	ENSG00000150687	19074876	validated
mirtarbase	hsa-miR-326	RPP14	11102	ENSG00000163684	23622248	validated
mirtarbase	hsa-miR-582-3p	KRR1	11103	ENSG00000111615	23313552	validated
mirtarbase	hsa-miR-192-5p	PRDM4	11108	ENSG00000110851	19074876	validated
mirtarbase	hsa-miR-15b-5p	PRDM4	11108	ENSG00000110851	21572407	validated
mirtarbase	hsa-miR-192-5p	HIBADH	11112	ENSG00000106049	19074876	validated
mirtarbase	hsa-miR-192-5p	CIT	11113	ENSG00000122966	19074876	validated
mirtarbase	hsa-miR-106b-5p	CIT	11113	ENSG00000122966	22473208	validated
mirtarbase	hsa-miR-192-5p	FGFR1OP	11116	ENSG00000213066	19074876	validated
mirtarbase	hsa-miR-301a-3p	FGFR1OP	11116	ENSG00000213066	20371350	validated
mirtarbase	hsa-miR-106b-5p	FGFR1OP	11116	ENSG00000213066	20371350	validated
mirtarbase	hsa-miR-106b-5p	BTN3A2	11118	ENSG00000186470	22473208	validated
mirtarbase	hsa-miR-106b-5p	BTN3A1	11119	ENSG00000026950	22473208	validated
mirtarbase	hsa-miR-15b-5p	RCAN3	11123	ENSG00000117602	23446348	validated
mirtarbase	hsa-miR-106b-5p	POLR3A	11128	ENSG00000148606	21572407	validated
mirtarbase	hsa-miR-106b-5p	KAT7	11143	ENSG00000136504	23622248	validated
mirtarbase	hsa-miR-18a-5p	BVES	11149	ENSG00000112276	23622248	validated
mirtarbase	hsa-miR-326	BVES	11149	ENSG00000112276	23824327	validated
mirtarbase	hsa-miR-222-3p	CORO1A	11151	ENSG00000102879	21226887	validated
mirtarbase	hsa-miR-106b-5p	FICD	11153	ENSG00000198855	21572407	validated
mirtarbase	hsa-miR-222-3p	FICD	11153	ENSG00000198855	21572407	validated
mirtarbase	hsa-miR-15b-5p	AP4S1	11154	ENSG00000100478	23622248	validated
mirtarbase	hsa-miR-192-5p	PTP4A3	11156	ENSG00000275575	16822819	validated
mirtarbase	hsa-miR-192-5p	ERLIN2	11160	ENSG00000147475	16822819	validated
mirtarbase	hsa-miR-326	ERLIN2	11160	ENSG00000147475	23622248	validated
mirtarbase	hsa-miR-106b-5p	NUDT3	11165	ENSG00000272325	23313552	validated
mirtarbase	hsa-miR-192-5p	NUDT3	11165	ENSG00000272325	27292025	validated
mirtarbase	hsa-miR-15b-5p	BAZ2A	11176	ENSG00000076108	21572407	validated
mirtarbase	hsa-miR-181a-5p	BAZ2A	11176	ENSG00000076108	22473208	validated
mirtarbase	hsa-miR-222-3p	WDR6	11180	ENSG00000178252	23622248	validated
mirtarbase	hsa-miR-181a-5p	RASSF1	11186	ENSG00000068028	26041820	validated
mirtarbase	hsa-miR-222-3p	CEP250	11190	ENSG00000126001	23622248	validated
mirtarbase	hsa-miR-106b-5p	PTENP1	11191		28212532	validated
mirtarbase	hsa-miR-181a-5p	WIF1	11197	ENSG00000156076	24755295	validated
mirtarbase	hsa-miR-15b-5p	SUPT16H	11198	ENSG00000092201	22473208	validated
mirtarbase	hsa-miR-301a-3p	CHEK2	11200	ENSG00000183765	21572407	validated
mirtarbase	hsa-miR-181a-5p	PLPBP	11212	ENSG00000147471	17612493	validated
mirtarbase	hsa-miR-15b-5p	PLPBP	11212	ENSG00000147471	20371350	validated
mirtarbase	hsa-miR-106b-5p	AKAP11	11215	ENSG00000023516	17242205	validated
mirtarbase	hsa-miR-192-5p	AKAP11	11215	ENSG00000023516	19074876	validated
mirtarbase	hsa-miR-15b-5p	AKAP11	11215	ENSG00000023516	22473208	validated

mirtarbase	hsa-miR-21-5p	DUSP10	11221	ENSG00000143507	22387553	validated
mirtarbase	hsa-miR-192-5p	RASSF8	11228	ENSG00000123094	19074876	validated
mirtarbase	hsa-miR-192-5p	SEC63	11231	ENSG00000025796	19074876	validated
mirtarbase	hsa-miR-21-5p	SEC63	11231	ENSG00000025796	18591254	validated
mirtarbase	hsa-miR-21-5p	HPS5	11234	ENSG00000110756	18591254	validated
mirtarbase	hsa-miR-21-5p	FILIP1L	11259	ENSG00000168386	20371350	validated
mirtarbase	hsa-miR-144-3p	CHP1	11261	ENSG00000187446	26701625	validated
mirtarbase	hsa-miR-582-5p	DDX19B	11269	ENSG00000157349	27292025	validated
mirtarbase	hsa-miR-181a-5p	PRR4	11272	ENSG00000263247	17612493	validated
mirtarbase	hsa-miR-326	ATXN2L	11273	ENSG00000168488	23622248	validated
mirtarbase	hsa-miR-192-5p	USP18	11274	ENSG00000184979	19074876	validated
mirtarbase	hsa-miR-326	SYNRG	11276	ENSG00000275066	23824327	validated
mirtarbase	hsa-miR-15b-5p	SYNRG	11276	ENSG00000275066	19536157	validated
mirtarbase	hsa-miR-222-3p	MGAT4B	11282	ENSG00000161013	23622248	validated
mirtarbase	hsa-miR-222-3p	PARK7	11315	ENSG00000116288	23622248	validated
mirtarbase	hsa-miR-21-5p	MGAT4A	11320	ENSG00000071073	18591254	validated
mirtarbase	hsa-miR-15b-5p	STK38	11329	ENSG00000112079	23592263	validated
mirtarbase	hsa-miR-181a-5p	PDAP1	11333	ENSG00000106244	22942087	validated
mirtarbase	hsa-miR-15b-5p	GABARAP	11337	ENSG00000170296	22473208	validated
mirtarbase	hsa-miR-222-3p	OIP5	11339	ENSG00000104147	22100165	validated
mirtarbase	hsa-miR-15b-5p	OIP5	11339	ENSG00000104147	28184024	validated
mirtarbase	hsa-miR-144-3p	Adamts1	11504		25151965	validated
mirtarbase	hsa-miR-222-3p	CASC3	22794	ENSG00000108349	23622248	validated
mirtarbase	hsa-miR-106b-5p	RRAS2	22800	ENSG00000133818	20371350	validated
mirtarbase	hsa-miR-222-3p	ATF5	22809	ENSG00000169136	23622248	validated
mirtarbase	hsa-miR-181a-5p	PHLDA1	22822	ENSG00000139289	22815788	validated
mirtarbase	hsa-miR-15b-5p	HSPA4L	22824	ENSG00000164070	24398324	validated
mirtarbase	hsa-miR-106b-5p	HSPA4L	22824	ENSG00000164070	21572407	validated
mirtarbase	hsa-miR-181a-5p	ZNF652	22834	ENSG00000198740	17612493	validated
mirtarbase	hsa-miR-192-5p	ZNF652	22834	ENSG00000198740	19074876	validated
mirtarbase	hsa-miR-106b-5p	ZNF652	22834	ENSG00000198740	23592263	validated
mirtarbase	hsa-miR-222-3p	ZNF652	22834	ENSG00000198740	23446348	validated
mirtarbase	hsa-miR-505-3p	ZNF652	22834	ENSG00000198740	23824327	validated
mirtarbase	hsa-miR-192-5p	ZFP30	22835	ENSG00000120784	19074876	validated
mirtarbase	hsa-miR-222-3p	ZFP30	22835	ENSG00000120784	21572407	validated
mirtarbase	hsa-miR-192-5p	RHOBTB3	22836	ENSG00000164292	19074876	validated
mirtarbase	hsa-miR-192-5p	COBLL1	22837	ENSG00000082438	19074876	validated
mirtarbase	hsa-miR-21-5p	COBLL1	22837	ENSG00000082438	18591254	validated
mirtarbase	hsa-miR-296-5p	RNF44	22838	ENSG00000146083	20371350	validated
mirtarbase	hsa-miR-192-5p	RAB11FIP2	22841	ENSG00000107560	19074876	validated
mirtarbase	hsa-miR-21-5p	RAB11FIP2	22841	ENSG00000107560	18591254	validated
mirtarbase	hsa-miR-15b-5p	RAB11FIP2	22841	ENSG00000107560	22473208	validated
mirtarbase	hsa-miR-18a-5p	ZNF507	22847	ENSG00000168813	20371350	validated
mirtarbase	hsa-miR-106b-5p	AAK1	22848	ENSG00000115977	22473208	validated
mirtarbase	hsa-miR-21-5p	CPEB3	22849	ENSG00000107864	18591254	validated
mirtarbase	hsa-miR-15b-5p	CPEB3	22849	ENSG00000107864	22473208	validated
mirtarbase	hsa-miR-106b-5p	CHSY1	22856	ENSG00000131873	23592263	validated
mirtarbase	hsa-miR-192-5p	ICK	22858	ENSG00000112144	19074876	validated
mirtarbase	hsa-miR-144-3p	FNDC3A	22862	ENSG00000102531	23592263	validated
mirtarbase	hsa-miR-106b-5p	ATG14	22863	ENSG00000126775	22473208	validated
mirtarbase	hsa-miR-15b-5p	ATG14	22863	ENSG00000126775	23446348	validated
mirtarbase	hsa-miR-301a-3p	PPP6R1	22870	ENSG00000105063	23592263	validated
mirtarbase	hsa-miR-192-5p	ENPP4	22875	ENSG00000001561	19074876	validated
mirtarbase	hsa-miR-301a-3p	ENPP4	22875	ENSG00000001561	21572407	validated
mirtarbase	hsa-miR-192-5p	INPP5F	22876	ENSG00000198825	19074876	validated
mirtarbase	hsa-miR-106b-5p	INPP5F	22876	ENSG00000198825	22473208	validated
mirtarbase	hsa-miR-106b-5p	MLXIP	22877	ENSG00000175727	22473208	validated
mirtarbase	hsa-miR-15b-5p	MLXIP	22877	ENSG00000175727	22473208	validated
mirtarbase	hsa-miR-192-5p	ANKRD6	22881	ENSG00000135299	19074876	validated
mirtarbase	hsa-miR-192-5p	CLSTN1	22883	ENSG00000171603	19074876	validated
mirtarbase	hsa-miR-106b-5p	WDR37	22884	ENSG00000047056	22473208	validated
mirtarbase	hsa-miR-106b-5p	FOXJ3	22887	ENSG00000198815	22473208	validated
mirtarbase	hsa-miR-106b-5p	UBOX5	22888	ENSG00000185019	23313552	validated
mirtarbase	hsa-miR-18a-5p	KHDC4	22889	ENSG00000132680	20371350	validated
mirtarbase	hsa-miR-505-3p	BAHD1	22893	ENSG00000140320	23313552	validated
mirtarbase	hsa-miR-192-5p	RUFY3	22902	ENSG00000018189	19074876	validated
mirtarbase	hsa-miR-21-5p	RUFY3	22902	ENSG00000018189	18591254	validated
mirtarbase	hsa-miR-505-3p	RUFY3	22902	ENSG00000018189	23622248	validated
mirtarbase	hsa-miR-181a-5p	BTBD3	22903	ENSG00000132640	17612493	validated
mirtarbase	hsa-miR-21-5p	BTBD3	22903	ENSG00000132640	18591254	validated
mirtarbase	hsa-miR-301a-3p	BTBD3	22903	ENSG00000132640	23592263	validated

mirtarbase	hsa-miR-15b-5p	TRAK1	22906	ENSG00000182606	23824327	validated
mirtarbase	hsa-miR-15b-5p	DHX30	22907	ENSG00000132153	23622248	validated
mirtarbase	hsa-miR-21-5p	SACM1L	22908	ENSG00000211456	18591254	validated
mirtarbase	hsa-miR-192-5p	WDR47	22911	ENSG00000085433	19074876	validated
mirtarbase	hsa-miR-192-5p	NCBP2	22916	ENSG00000114503	20371350	validated
mirtarbase	hsa-miR-21-5p	KIFAP3	22920	ENSG00000075945	18591254	validated
mirtarbase	hsa-miR-106b-5p	MAPRE3	22924	ENSG00000084764	24398324	validated
mirtarbase	hsa-miR-301a-3p	MAPRE3	22924	ENSG00000084764	24398324	validated
mirtarbase	hsa-miR-301a-3p	HABP4	22927	ENSG00000130956	22012620	validated
mirtarbase	hsa-miR-212-3p	RAB18	22931	ENSG00000099246	22012620	validated
mirtarbase	hsa-miR-21-5p	SIRT2	22933	ENSG00000068903	24161395	validated
mirtarbase	hsa-miR-192-5p	ELL2	22936	ENSG00000118985	19074876	validated
mirtarbase	hsa-miR-301a-3p	ELL2	22936	ENSG00000118985	23592263	validated
mirtarbase	hsa-miR-144-3p	ELL2	22936	ENSG00000118985	23592263	validated
mirtarbase	hsa-miR-106b-5p	TRIM32	22954	ENSG00000119401	22473208	validated
mirtarbase	hsa-miR-192-5p	SCMH1	22955	ENSG00000010803	19074876	validated
mirtarbase	hsa-miR-21-5p	NT5C2	22978	ENSG00000076685	18591254	validated
mirtarbase	hsa-miR-505-3p	DIP2C	22982	ENSG00000151240	23622248	validated
mirtarbase	hsa-miR-18a-5p	ACIN1	22985	ENSG00000100813	20371350	validated
mirtarbase	hsa-miR-106b-5p	ACIN1	22985	ENSG00000100813	23622248	validated
mirtarbase	hsa-miR-326	MYH15	22989	ENSG00000144821	28735896	validated
mirtarbase	hsa-miR-21-5p	CEP152	22995	ENSG00000103995	18591254	validated
mirtarbase	hsa-miR-192-5p	LIMCH1	22998	ENSG00000064042	19074876	validated
mirtarbase	hsa-miR-21-5p	LIMCH1	22998	ENSG00000064042	18591254	validated
mirtarbase	hsa-miR-181a-5p	WDFY3	23001	ENSG00000163625	27418678	validated
mirtarbase	hsa-miR-21-5p	DAAM1	23002	ENSG00000100592	18591254	validated
mirtarbase	hsa-miR-15b-5p	KLHDC10	23008	ENSG00000128607	22473208	validated
mirtarbase	hsa-miR-222-3p	KLHDC10	23008	ENSG00000128607	26701625	validated
mirtarbase	hsa-miR-296-5p	KLHDC10	23008	ENSG00000128607	26701625	validated
mirtarbase	hsa-miR-106b-5p	SPEN	23013	ENSG00000065526	23622248	validated
mirtarbase	hsa-miR-18a-5p	SPEN	23013	ENSG00000065526	26701625	validated
mirtarbase	hsa-miR-222-3p	FBXO21	23014	ENSG00000135108	23622248	validated
mirtarbase	hsa-miR-106b-5p	FBXO21	23014	ENSG00000135108	22473208	validated
mirtarbase	hsa-miR-21-5p	PALLD	23022	ENSG00000129116	18591254	validated
mirtarbase	hsa-miR-181a-5p	TMCC1	23023	ENSG00000172765	21572407	validated
mirtarbase	hsa-miR-222-3p	TMCC1	23023	ENSG00000172765	20371350	validated
mirtarbase	hsa-miR-106b-5p	PHLPP2	23035	ENSG00000040199	22473208	validated
mirtarbase	hsa-miR-15b-5p	PHLPP2	23035	ENSG00000040199	21572407	validated
mirtarbase	hsa-miR-181a-5p	PHLPP2	23035	ENSG00000040199	27915346	validated
mirtarbase	hsa-miR-21-5p	ZNF292	23036	ENSG00000188994	18591254	validated
mirtarbase	hsa-miR-21-5p	MON2	23041	ENSG00000061987	18591254	validated
mirtarbase	hsa-miR-222-3p	PDXDC1	23042	ENSG00000275498	23622248	validated
mirtarbase	hsa-miR-192-5p	FNBP1	23048	ENSG00000187239	19074876	validated
mirtarbase	hsa-miR-21-5p	FNBP1	23048	ENSG00000187239	18591254	validated
mirtarbase	hsa-miR-106b-5p	FNBP1	23048	ENSG00000187239	23622248	validated
mirtarbase	hsa-miR-181a-5p	SMG1	23049	ENSG00000157106	23622248	validated
mirtarbase	hsa-miR-192-5p	ENDOD1	23052	ENSG00000149218	19074876	validated
mirtarbase	hsa-miR-18a-5p	NCOA6	23054	ENSG00000198646	20371350	validated
mirtarbase	hsa-miR-181a-5p	CLUAP1	23059	ENSG00000103351	17612493	validated
mirtarbase	hsa-miR-192-5p	EMC1	23065	ENSG00000127463	19074876	validated
mirtarbase	hsa-miR-106b-5p	EMC1	23065	ENSG00000127463	22100165	validated
mirtarbase	hsa-miR-222-3p	SETD1B	23067	ENSG00000139718	23622248	validated
mirtarbase	hsa-miR-15b-5p	SETD1B	23067	ENSG00000139718	20371350	validated
mirtarbase	hsa-miR-21-5p	SETD1B	23067	ENSG00000139718	23592263	validated
mirtarbase	hsa-miR-296-5p	SETD1B	23067	ENSG00000139718	23592263	validated
mirtarbase	hsa-miR-21-5p	ERP44	23071	ENSG00000023318	22100165	validated
mirtarbase	hsa-miR-505-3p	RRP1B	23076	ENSG00000160208	23622248	validated
mirtarbase	hsa-miR-21-5p	MYCBP2	23077	ENSG00000005810	18591254	validated
mirtarbase	hsa-miR-15b-5p	MYCBP2	23077	ENSG00000005810	23622248	validated
mirtarbase	hsa-miR-15b-5p	AVL9	23080	ENSG00000105778	23592263	validated
mirtarbase	hsa-miR-222-3p	PPRC1	23082	ENSG00000148840	23622248	validated
mirtarbase	hsa-miR-18a-5p	EXPH5	23086	ENSG00000110723	26233958	validated
mirtarbase	hsa-miR-15b-5p	TRIM35	23087	ENSG00000104228	23446348	validated
mirtarbase	hsa-miR-18a-5p	TRIM35	23087	ENSG00000104228	27292025	validated
mirtarbase	hsa-miR-15b-5p	TTLL5	23093	ENSG00000119685	20371350	validated
mirtarbase	hsa-miR-18a-5p	CDK19	23097	ENSG00000155111	22308110	validated
mirtarbase	hsa-miR-21-5p	CDK19	23097	ENSG00000155111	18591254	validated
mirtarbase	hsa-miR-301a-3p	CDK19	23097	ENSG00000155111	22100165	validated
mirtarbase	hsa-miR-222-3p	RAP1GAP2	23108	ENSG00000132359	23622248	validated
mirtarbase	hsa-miR-301a-3p	SPART	23111	ENSG00000133104	23446348	validated
mirtarbase	hsa-miR-21-5p	TNRC6B	23112	ENSG00000100354	18591254	validated

mirtarbase	hsa-miR-18a-5p	TNRC6B	23112	ENSG00000100354	20371350	validated
mirtarbase	hsa-miR-222-3p	TNRC6B	23112	ENSG00000100354	23622248	validated
mirtarbase	hsa-miR-15b-5p	TNRC6B	23112	ENSG00000100354	22473208	validated
mirtarbase	hsa-miR-106b-5p	TNRC6B	23112	ENSG00000100354	23446348	validated
mirtarbase	hsa-miR-301a-3p	TNRC6B	23112	ENSG00000100354	23592263	validated
mirtarbase	hsa-miR-181a-5p	TNRC6B	23112	ENSG00000100354	22473208	validated
mirtarbase	hsa-miR-181a-5p	TAB2	23118	ENSG00000055208	20371350	validated
mirtarbase	hsa-miR-15b-5p	TAB2	23118	ENSG00000055208	23622248	validated
mirtarbase	hsa-miR-21-5p	HIC2	23119	ENSG00000169635	22473208	validated
mirtarbase	hsa-miR-301a-3p	POGZ	23126	ENSG00000143442	21572407	validated
mirtarbase	hsa-miR-106b-5p	ATG2A	23130	ENSG00000110046	22473208	validated
mirtarbase	hsa-miR-192-5p	ATG2A	23130	ENSG00000110046	20371350	validated
mirtarbase	hsa-miR-15b-5p	GPATCH8	23131	ENSG00000186566	22473208	validated
mirtarbase	hsa-miR-326	PHF8	23133	ENSG00000172943	21572407	validated
mirtarbase	hsa-miR-106b-5p	KDM6B	23135	ENSG00000132510	20371350	validated
mirtarbase	hsa-miR-505-3p	GRAMD4	23151	ENSG00000075240	22012620	validated
mirtarbase	hsa-miR-181a-5p	CLCC1	23155	ENSG00000121940	22473208	validated
mirtarbase	hsa-miR-106b-5p	TBC1D9	23158	ENSG00000109436	17242205	validated
mirtarbase	hsa-miR-192-5p	SNX13	23161	ENSG00000071189	19074876	validated
mirtarbase	hsa-miR-15b-5p	GGA3	23163	ENSG00000125447	22473208	validated
mirtarbase	hsa-miR-21-5p	GPD1L	23171	ENSG00000152642	18591254	validated
mirtarbase	hsa-miR-181a-5p	GPD1L	23171	ENSG00000152642	28280258	validated
mirtarbase	hsa-miR-181a-5p	METAP1	23173	ENSG00000164024	17612493	validated
mirtarbase	hsa-miR-15b-5p	ZCCHC14	23174	ENSG00000140948	23622248	validated
mirtarbase	hsa-miR-192-5p	PASK	23178	ENSG00000115687	19074876	validated
mirtarbase	hsa-miR-15b-5p	MESD	23184	ENSG00000117899	23622248	validated
mirtarbase	hsa-miR-222-3p	MESD	23184	ENSG00000117899	23622248	validated
mirtarbase	hsa-miR-15b-5p	RCOR1	23186	ENSG00000089902	22473208	validated
mirtarbase	hsa-miR-181a-5p	RCOR1	23186	ENSG00000089902	22473208	validated
mirtarbase	hsa-miR-15b-5p	KANK1	23189	ENSG00000107104	21572407	validated
mirtarbase	hsa-miR-222-3p	GANAB	23193	ENSG00000089597	23622248	validated
mirtarbase	hsa-miR-181a-5p	GANAB	23193	ENSG00000089597	23622248	validated
mirtarbase	hsa-miR-15b-5p	GANAB	23193	ENSG00000089597	22473208	validated
mirtarbase	hsa-miR-106b-5p	FBXL7	23194	ENSG00000183580	22012620	validated
mirtarbase	hsa-miR-222-3p	MDN1	23195	ENSG00000112159	23622248	validated
mirtarbase	hsa-miR-106b-5p	FAF2	23197	ENSG00000113194	22473208	validated
mirtarbase	hsa-miR-21-5p	ATP11B	23200	ENSG00000058063	18591254	validated
mirtarbase	hsa-miR-222-3p	ATP11B	23200	ENSG00000058063	23622248	validated
mirtarbase	hsa-miR-222-3p	ARL6IP1	23204	ENSG00000170540	23592263	validated
mirtarbase	hsa-miR-301a-3p	ARL6IP1	23204	ENSG00000170540	27292025	validated
mirtarbase	hsa-miR-212-3p	RRS1	23212	ENSG00000179041	21572407	validated
mirtarbase	hsa-miR-222-3p	XPO6	23214	ENSG00000169180	23622248	validated
mirtarbase	hsa-miR-181a-5p	PRRC2C	23215	ENSG00000117523	22473208	validated
mirtarbase	hsa-miR-15b-5p	PRRC2C	23215	ENSG00000117523	22473208	validated
mirtarbase	hsa-miR-181a-5p	FBXO28	23219	ENSG00000143756	20371350	validated
mirtarbase	hsa-miR-301a-3p	FBXO28	23219	ENSG00000143756	21572407	validated
mirtarbase	hsa-miR-301a-3p	DTX4	23220	ENSG00000110042	19536157	validated
mirtarbase	hsa-miR-106b-5p	RHOBTB2	23221	ENSG00000008853	23622248	validated
mirtarbase	hsa-miR-21-5p	SYNE2	23224	ENSG00000054654	18591254	validated
mirtarbase	hsa-miR-15b-5p	NUP210	23225	ENSG00000132182	23622248	validated
mirtarbase	hsa-miR-181a-5p	PLCL2	23228	ENSG00000154822	17612493	validated
mirtarbase	hsa-miR-21-5p	VPS13A	23230	ENSG00000197969	18591254	validated
mirtarbase	hsa-miR-15b-5p	VPS13A	23230	ENSG00000197969	23622248	validated
mirtarbase	hsa-miR-15b-5p	DNAJC9	23234	ENSG00000213551	22473208	validated
mirtarbase	hsa-miR-181a-5p	SIK2	23235	ENSG00000170145	23622248	validated
mirtarbase	hsa-miR-18a-5p	PHLPP1	23239	ENSG00000081913	22116552	validated
mirtarbase	hsa-miR-106b-5p	TMEM131L	23240	ENSG00000121210	22012620	validated
mirtarbase	hsa-miR-222-3p	PACS2	23241	ENSG00000179364	23622248	validated
mirtarbase	hsa-miR-21-5p	ANKRD28	23243	ENSG00000206560	18591254	validated
mirtarbase	hsa-miR-301a-3p	RPRD2	23248	ENSG00000163125	22012620	validated
mirtarbase	hsa-miR-15b-5p	RPRD2	23248	ENSG00000163125	24398324	validated
mirtarbase	hsa-miR-21-5p	RPRD2	23248	ENSG00000163125	23446348	validated
mirtarbase	hsa-miR-106b-5p	RPRD2	23248	ENSG00000163125	20371350	validated
mirtarbase	hsa-miR-296-5p	RPRD2	23248	ENSG00000163125	27418678	validated
mirtarbase	hsa-miR-192-5p	OTUD3	23252	ENSG00000169914	19074876	validated
mirtarbase	hsa-miR-301a-3p	OTUD3	23252	ENSG00000169914	21572407	validated
mirtarbase	hsa-miR-212-3p	OTUD3	23252	ENSG00000169914	27292025	validated
mirtarbase	hsa-miR-106b-5p	ANKRD12	23253	ENSG00000101745	22473208	validated
mirtarbase	hsa-miR-144-3p	ANKRD12	23253	ENSG00000101745	22100165	validated
mirtarbase	hsa-miR-181a-5p	MTCL1	23255	ENSG00000168502	23622248	validated
mirtarbase	hsa-miR-21-5p	DDHD2	23259	ENSG00000085788	18591254	validated

mirtarbase	hsa-miR-106b-5p	CAMTA1	23261	ENSG00000171735	22473208	validated
mirtarbase	hsa-miR-15b-5p	PPIP5K2	23262	ENSG00000145725	19536157	validated
mirtarbase	hsa-miR-21-5p	MGA	23269	ENSG00000174197	17072344	validated
mirtarbase	hsa-miR-301a-3p	CAMSAP2	23271	ENSG00000118200	24398324	validated
mirtarbase	hsa-miR-15b-5p	CLUH	23277	ENSG00000132361	26701625	validated
mirtarbase	hsa-miR-505-3p	CLUH	23277	ENSG00000132361	26701625	validated
mirtarbase	hsa-miR-15b-5p	NUP160	23279	ENSG00000030066	23622248	validated
mirtarbase	hsa-miR-222-3p	CSTF2T	23283	ENSG00000177613	21572407	validated
mirtarbase	hsa-miR-106b-5p	WWC1	23286	ENSG00000113645	23313552	validated
mirtarbase	hsa-miR-505-3p	BICD2	23299	ENSG00000185963	23592263	validated
mirtarbase	hsa-miR-144-3p	BICD2	23299	ENSG00000185963	21572407	validated
mirtarbase	hsa-miR-106b-5p	BICD2	23299	ENSG00000185963	20371350	validated
mirtarbase	hsa-miR-21-5p	ATMIN	23300	ENSG00000166454	20371350	validated
mirtarbase	hsa-miR-301a-3p	ATMIN	23300	ENSG00000166454	22012620	validated
mirtarbase	hsa-miR-192-5p	NEMP1	23306	ENSG00000166881	19074876	validated
mirtarbase	hsa-miR-192-5p	FKBP15	23307	ENSG00000119321	19074876	validated
mirtarbase	hsa-miR-21-5p	ICOSLG	23308	ENSG00000160223	25327529	validated
mirtarbase	hsa-miR-192-5p	SATB2	23314	ENSG00000119042	19074876	validated
mirtarbase	hsa-miR-301a-3p	SATB2	23314	ENSG00000119042	21572407	validated
mirtarbase	hsa-miR-106b-5p	DNAJC13	23317	ENSG00000138246	23622248	validated
mirtarbase	hsa-miR-181a-5p	TRIM2	23321	ENSG00000109654	20371350	validated
mirtarbase	hsa-miR-21-5p	TRIM2	23321	ENSG00000109654	18591254	validated
mirtarbase	hsa-miR-192-5p	RPGRIP1L	23322	ENSG00000103494	19074876	validated
mirtarbase	hsa-miR-106b-5p	MAN2B2	23324	ENSG00000013288	23313552	validated
mirtarbase	hsa-miR-21-5p	SASH1	23328	ENSG00000111961	18591254	validated
mirtarbase	hsa-miR-181a-5p	SASH1	23328	ENSG00000111961	22815788	validated
mirtarbase	hsa-miR-582-5p	DPY19L1	23333	ENSG00000173852	23592263	validated
mirtarbase	hsa-miR-296-5p	DPY19L1	23333	ENSG00000173852	22291592	validated
mirtarbase	hsa-miR-21-5p	WDR7	23335	ENSG00000091157	18591254	validated
mirtarbase	hsa-miR-18a-5p	SYNM	23336	ENSG00000182253	21572407	validated
mirtarbase	hsa-miR-192-5p	SYNM	23336	ENSG00000182253	21572407	validated
mirtarbase	hsa-miR-21-5p	DNAJC16	23341	ENSG00000116138	22473208	validated
mirtarbase	hsa-miR-181a-5p	SMCHD1	23347	ENSG00000101596	17612493	validated
mirtarbase	hsa-miR-18a-5p	SMCHD1	23347	ENSG00000101596	23622248	validated
mirtarbase	hsa-miR-106b-5p	U2SURP	23350	ENSG00000163714	22473208	validated
mirtarbase	hsa-miR-15b-5p	U2SURP	23350	ENSG00000163714	22473208	validated
mirtarbase	hsa-miR-15b-5p	SUN1	23353	ENSG00000164828	22473208	validated
mirtarbase	hsa-miR-326	PSD3	23362	ENSG00000156011	23622248	validated
mirtarbase	hsa-miR-21-5p	ARHGEF12	23365	ENSG00000196914	18591254	validated
mirtarbase	hsa-miR-15b-5p	KIAA0895	23366	ENSG00000164542	22291592	validated
mirtarbase	hsa-miR-505-3p	PPP1R13B	23368	ENSG00000088808	23622248	validated
mirtarbase	hsa-miR-106b-5p	ARHGEF18	23370	ENSG00000104880	22473208	validated
mirtarbase	hsa-miR-212-3p	CRTC1	23373	ENSG00000105662	23446348	validated
mirtarbase	hsa-miR-21-5p	NCSTN	23385	ENSG00000162736	20371350	validated
mirtarbase	hsa-miR-582-3p	MED13L	23389	ENSG00000123066	23824327	validated
mirtarbase	hsa-miR-21-5p	ADNP	23394	ENSG00000101126	18591254	validated
mirtarbase	hsa-miR-192-5p	NCAPH	23397	ENSG00000121152	19074876	validated
mirtarbase	hsa-miR-222-3p	DICER1	23405	ENSG00000100697	21761362	validated
mirtarbase	hsa-miR-18a-5p	DICER1	23405	ENSG00000100697	23097559	validated
mirtarbase	hsa-miR-192-5p	DICER1	23405	ENSG00000100697	19074876	validated
mirtarbase	hsa-miR-21-5p	DICER1	23405	ENSG00000100697	22473208	validated
mirtarbase	hsa-miR-582-5p	DICER1	23405	ENSG00000100697	23592263	validated
mirtarbase	hsa-miR-301a-3p	DICER1	23405	ENSG00000100697	23592263	validated
mirtarbase	hsa-miR-15b-5p	DICER1	23405	ENSG00000100697	22473208	validated
mirtarbase	hsa-miR-15b-5p	SIRT4	23409	ENSG00000089163	22473208	validated
mirtarbase	hsa-miR-181a-5p	SIRT1	23411	ENSG00000096717	22476949	validated
mirtarbase	hsa-miR-192-5p	SIRT1	23411	ENSG00000096717	19074876	validated
mirtarbase	hsa-miR-18a-5p	CRB1	23418	ENSG00000134376	23592263	validated
mirtarbase	hsa-miR-301a-3p	GPR161	23432	ENSG00000143147	27292025	validated
mirtarbase	hsa-miR-21-5p	RHOQ	23433	ENSG00000119729	18591254	validated
mirtarbase	hsa-miR-192-5p	TARDBP	23435	ENSG00000120948	19074876	validated
mirtarbase	hsa-miR-15b-5p	TARDBP	23435	ENSG00000120948	23622248	validated
mirtarbase	hsa-miR-15b-5p	SF3B3	23450	ENSG00000189091	23622248	validated
mirtarbase	hsa-miR-222-3p	SF3B3	23450	ENSG00000189091	23622248	validated
mirtarbase	hsa-miR-326	SF3B3	23450	ENSG00000189091	23592263	validated
mirtarbase	hsa-miR-106b-5p	SF3B3	23450	ENSG00000189091	23313552	validated
mirtarbase	hsa-miR-192-5p	ABCB10	23456	ENSG00000135776	19074876	validated
mirtarbase	hsa-miR-106b-5p	ICMT	23463	ENSG00000116237	23592263	validated
mirtarbase	hsa-miR-15b-5p	CBX6	23466	ENSG00000183741	23446348	validated
mirtarbase	hsa-miR-192-5p	NPTXR	23467	ENSG00000221890	19074876	validated
mirtarbase	hsa-miR-106b-5p	CBX5	23468	ENSG00000094916	23824327	validated

mirtarbase	hsa-miR-582-5p	PHF3	23469	ENSG00000118482	20371350	validated
mirtarbase	hsa-miR-144-3p	PHF3	23469	ENSG00000118482	20371350	validated
mirtarbase	hsa-miR-15b-5p	TRAM1	23471	ENSG00000067167	22473208	validated
mirtarbase	hsa-miR-21-5p	ISCU	23479	ENSG00000136003	20480266	validated
mirtarbase	hsa-miR-18a-5p	ISCU	23479	ENSG00000136003	26701625	validated
mirtarbase	hsa-miR-192-5p	LEPROTL1	23484	ENSG00000104660	19074876	validated
mirtarbase	hsa-miR-181a-5p	HEY2	23493	ENSG00000135547	17612493	validated
mirtarbase	hsa-miR-192-5p	MACF1	23499	ENSG00000127603	19074876	validated
mirtarbase	hsa-miR-222-3p	MACF1	23499	ENSG00000127603	23622248	validated
mirtarbase	hsa-miR-18a-5p	DAAM2	23500	ENSG00000146122	20371350	validated
mirtarbase	hsa-miR-106b-5p	ZFYVE26	23503	ENSG00000072121	23592263	validated
mirtarbase	hsa-miR-301a-3p	ZFYVE26	23503	ENSG00000072121	23592263	validated
mirtarbase	hsa-miR-192-5p	BICRAL	23506	ENSG00000112624	19074876	validated
mirtarbase	hsa-miR-106b-5p	TTC9	23508	ENSG00000133985	23446348	validated
mirtarbase	hsa-miR-106b-5p	POFUT1	23509	ENSG00000101346	22473208	validated
mirtarbase	hsa-miR-326	POFUT1	23509	ENSG00000101346	22100165	validated
mirtarbase	hsa-miR-181a-5p	KCTD2	23510	ENSG00000180901	23622248	validated
mirtarbase	hsa-miR-21-5p	SUZ12	23512	ENSG00000178691	18591254	validated
mirtarbase	hsa-miR-296-5p	SCRIB	23513	ENSG00000274287	21643016	validated
mirtarbase	hsa-miR-21-5p	MORC3	23515	ENSG00000159256	20371350	validated
mirtarbase	hsa-miR-144-3p	MORC3	23515	ENSG00000159256	21572407	validated
mirtarbase	hsa-miR-15b-5p	SLC39A14	23516	ENSG00000104635	23622248	validated
mirtarbase	hsa-miR-181a-5p	RPL13A	23521	ENSG00000142541	26701625	validated
mirtarbase	hsa-miR-222-3p	SRRM2	23524	ENSG00000167978	23622248	validated
mirtarbase	hsa-miR-106b-5p	ACAP2	23527	ENSG00000114331	22473208	validated
mirtarbase	hsa-miR-505-3p	ACAP2	23527	ENSG00000114331	23824327	validated
mirtarbase	hsa-miR-326	ACAP2	23527	ENSG00000114331	23824327	validated
mirtarbase	hsa-miR-106b-5p	NNT	23530	ENSG00000112992	23622248	validated
mirtarbase	hsa-miR-15b-5p	NNT	23530	ENSG00000112992	23446348	validated
mirtarbase	hsa-miR-15b-5p	TNPO3	23534	ENSG00000064419	23622248	validated
mirtarbase	hsa-miR-21-5p	TTC33	23548	ENSG00000113638	19253296	validated
mirtarbase	hsa-miR-212-3p	TSPAN12	23554	ENSG00000106025	21572407	validated
mirtarbase	hsa-miR-21-5p	PIGN	23556	ENSG00000197563	18591254	validated
mirtarbase	hsa-miR-301a-3p	SNAPIN	23557	ENSG00000143553	23592263	validated
mirtarbase	hsa-miR-192-5p	ZNF346	23567	ENSG00000113761	19074876	validated
mirtarbase	hsa-miR-192-5p	ARL2BP	23568	ENSG00000102931	19074876	validated
mirtarbase	hsa-miR-21-5p	DDAH1	23576	ENSG00000153904	18591254	validated
mirtarbase	hsa-miR-18a-5p	DDAH1	23576	ENSG00000153904	23622248	validated
mirtarbase	hsa-miR-296-5p	CDC42EP4	23580	ENSG00000179604	19536157	validated
mirtarbase	hsa-miR-582-5p	CDC42EP4	23580	ENSG00000179604	27292025	validated
mirtarbase	hsa-miR-106b-5p	PDSS1	23590	ENSG00000148459	23622248	validated
mirtarbase	hsa-miR-106b-5p	ACOT9	23597	ENSG00000123130	23313552	validated
mirtarbase	hsa-miR-326	PATZ1	23598	ENSG00000100105	22012620	validated
mirtarbase	hsa-miR-15b-5p	CD2AP	23607	ENSG00000198087	21572407	validated
mirtarbase	hsa-miR-106b-5p	MKRN1	23608	ENSG00000133606	22473208	validated
mirtarbase	hsa-miR-18a-5p	ZMYND8	23613	ENSG00000101040	23622248	validated
mirtarbase	hsa-miR-505-3p	BACE1	23621	ENSG00000186318	19536157	validated
mirtarbase	hsa-miR-222-3p	KPNA6	23633	ENSG00000025800	23622248	validated
mirtarbase	hsa-miR-106b-5p	KPNA6	23633	ENSG00000025800	27292025	validated
mirtarbase	hsa-miR-326	SSBP2	23635	ENSG00000145687	23824327	validated
mirtarbase	hsa-miR-192-5p	RABGAP1	23637	ENSG00000011454	16822819	validated
mirtarbase	hsa-miR-21-5p	RABGAP1	23637	ENSG00000011454	18591254	validated
mirtarbase	hsa-miR-15b-5p	EDC4	23644	ENSG00000038358	23622248	validated
mirtarbase	hsa-miR-192-5p	POLA2	23649	ENSG00000014138	19074876	validated
mirtarbase	hsa-miR-15b-5p	TRIM29	23650	ENSG00000137699	26872369	validated
mirtarbase	hsa-miR-222-3p	PLXNB2	23654	ENSG00000196576	23622248	validated
mirtarbase	hsa-miR-181a-5p	SLC7A11	23657	ENSG00000151012	17612493	validated
mirtarbase	hsa-miR-192-5p	SLC7A11	23657	ENSG00000151012	19074876	validated
mirtarbase	hsa-miR-106b-5p	SLC7A11	23657	ENSG00000151012	23313552	validated
mirtarbase	hsa-miR-301a-3p	UBBP4	23666		23446348	validated
mirtarbase	hsa-miR-192-5p	CEMIP2	23670	ENSG00000135048	19074876	validated
mirtarbase	hsa-miR-21-5p	CEMIP2	23670	ENSG00000135048	18591254	validated
mirtarbase	hsa-miR-301a-3p	CEMIP2	23670	ENSG00000135048	23313552	validated
mirtarbase	hsa-miR-222-3p	CEMIP2	23670	ENSG00000135048	23313552	validated
mirtarbase	hsa-miR-18a-5p	CEMIP2	23670	ENSG00000135048	23313552	validated
mirtarbase	hsa-miR-106b-5p	STX12	23673	ENSG00000117758	23622248	validated
mirtarbase	hsa-miR-18a-5p	STX12	23673	ENSG00000117758	23622248	validated
mirtarbase	hsa-miR-192-5p	SH3BP4	23677	ENSG00000130147	19074876	validated
mirtarbase	hsa-miR-15b-5p	SH3BP4	23677	ENSG00000130147	23592263	validated
mirtarbase	hsa-miR-212-3p	SGK3	23678	ENSG00000104205	25720694	validated
mirtarbase	hsa-miR-192-5p	PRKD3	23683	ENSG00000115825	19074876	validated

mirtarbase	hsa-miR-192-5p	CADM1	23705	ENSG00000182985	16822819	validated
mirtarbase	hsa-miR-144-3p	CADM1	23705	ENSG00000182985	20371350	validated
mirtarbase	hsa-miR-21-5p	CADM1	23705	ENSG00000182985	27055844	validated
mirtarbase	hsa-miR-15b-5p	GABARAPL1	23710	ENSG00000139112	23446348	validated
mirtarbase	hsa-miR-106b-5p	TMEM245	23731	ENSG00000106771	17242205	validated
mirtarbase	hsa-miR-21-5p	TMEM245	23731	ENSG00000106771	20371350	validated
mirtarbase	hsa-miR-222-3p	TMEM245	23731	ENSG00000106771	23622248	validated
mirtarbase	hsa-miR-15b-5p	TMEM245	23731	ENSG00000106771	23446348	validated
mirtarbase	hsa-miR-106b-5p	BHMT2	23743	ENSG00000132840	23313552	validated
mirtarbase	hsa-miR-15b-5p	PPIL2	23759	ENSG00000100023	23622248	validated
mirtarbase	hsa-miR-181a-5p	PITPNB	23760	ENSG00000180957	20371350	validated
mirtarbase	hsa-miR-192-5p	PITPNB	23760	ENSG00000180957	19074876	validated
mirtarbase	hsa-miR-15b-5p	PISD	23761	ENSG00000241878	23592263	validated
mirtarbase	hsa-miR-505-3p	PISD	23761	ENSG00000241878	24398324	validated
mirtarbase	hsa-miR-192-5p	BCL2L13	23786	ENSG00000099968	19074876	validated
mirtarbase	hsa-miR-222-3p	KIF4A	24137	ENSG00000090889	23622248	validated
mirtarbase	hsa-miR-106b-5p	IFIT5	24138	ENSG00000152778	17242205	validated
mirtarbase	hsa-miR-192-5p	PANX1	24145	ENSG00000110218	16822819	validated
mirtarbase	hsa-miR-106b-5p	FJX1	24147	ENSG00000179431	21572407	validated
mirtarbase	hsa-miR-106b-5p	HYPK	25764	ENSG00000242028	23446348	validated
mirtarbase	hsa-miR-301a-3p	CBY1	25776	ENSG00000100211	21572407	validated
mirtarbase	hsa-miR-222-3p	SUN2	25777	ENSG00000100242	23622248	validated
mirtarbase	hsa-miR-301a-3p	SUN2	25777	ENSG00000100242	21572407	validated
mirtarbase	hsa-miR-192-5p	DSTYK	25778	ENSG00000133059	19074876	validated
mirtarbase	hsa-miR-301a-3p	DSTYK	25778	ENSG00000133059	22012620	validated
mirtarbase	hsa-miR-106b-5p	DSTYK	25778	ENSG00000133059	27292025	validated
mirtarbase	hsa-miR-21-5p	RASGRP3	25780	ENSG00000152689	18591254	validated
mirtarbase	hsa-miR-192-5p	DGCR11	25786		19074876	validated
mirtarbase	hsa-miR-192-5p	FSBP	25788	ENSG00000265817	19074876	validated
mirtarbase	hsa-miR-192-5p	RAD54B	25788	ENSG00000197275	19074876	validated
mirtarbase	hsa-miR-326	CIZ1	25792	ENSG00000148337	23622248	validated
mirtarbase	hsa-miR-18a-5p	CIZ1	25792	ENSG00000148337	23622248	validated
mirtarbase	hsa-miR-144-3p	SLC39A6	25800	ENSG00000141424	21572407	validated
mirtarbase	hsa-miR-505-3p	LSM4	25804	ENSG00000130520	27292025	validated
mirtarbase	hsa-miR-106b-5p	BAMBI	25805	ENSG00000095739	23622248	validated
mirtarbase	hsa-miR-15b-5p	BAMBI	25805	ENSG00000095739	23622248	validated
mirtarbase	hsa-miR-21-5p	ATXN10	25814	ENSG00000130638	22473208	validated
mirtarbase	hsa-miR-15b-5p	ARIH1	25820	ENSG00000166233	22473208	validated
mirtarbase	hsa-miR-212-3p	MTO1	25821	ENSG00000135297	27292025	validated
mirtarbase	hsa-miR-192-5p	DNAJB5	25822	ENSG00000137094	19074876	validated
mirtarbase	hsa-miR-21-5p	FBXL2	25827	ENSG00000153558	18591254	validated
mirtarbase	hsa-miR-21-5p	HECTD1	25831	ENSG00000092148	18591254	validated
mirtarbase	hsa-miR-21-5p	NIPBL	25836	ENSG00000164190	18591254	validated
mirtarbase	hsa-miR-106b-5p	ABTB2	25841	ENSG00000166016	23622248	validated
mirtarbase	hsa-miR-212-3p	ASF1A	25842	ENSG00000111875	21572407	validated
mirtarbase	hsa-miR-18a-5p	MOB4	25843	ENSG00000115540	20371350	validated
mirtarbase	hsa-miR-15b-5p	MOB4	25843	ENSG00000115540	24398324	validated
mirtarbase	hsa-miR-144-3p	MOB4	25843	ENSG00000115540	20371350	validated
mirtarbase	hsa-miR-15b-5p	ANAPC13	25847	ENSG00000129055	21572407	validated
mirtarbase	hsa-miR-192-5p	PARM1	25849	ENSG00000169116	19074876	validated
mirtarbase	hsa-miR-296-5p	SUMF2	25870	ENSG00000129103	23592263	validated
mirtarbase	hsa-miR-15b-5p	RPL36	25873	ENSG00000130255	22473208	validated
mirtarbase	hsa-miR-192-5p	ANAPC15	25906	ENSG00000110200	19074876	validated
mirtarbase	hsa-miR-106b-5p	C1orf43	25912	ENSG00000143612	23622248	validated
mirtarbase	hsa-miR-15b-5p	C1orf43	25912	ENSG00000143612	23622248	validated
mirtarbase	hsa-miR-106b-5p	ATL3	25923	ENSG00000184743	22473208	validated
mirtarbase	hsa-miR-301a-3p	CLIC4	25932	ENSG00000169504	23446348	validated
mirtarbase	hsa-miR-144-3p	CLIC4	25932	ENSG00000169504	23446348	validated
mirtarbase	hsa-miR-106b-5p	CLIC4	25932	ENSG00000169504	21572407	validated
mirtarbase	hsa-miR-18a-5p	WWTR1	25937	ENSG00000018408	23622248	validated
mirtarbase	hsa-miR-192-5p	SAMHD1	25939	ENSG00000101347	19074876	validated
mirtarbase	hsa-miR-181a-5p	SAMHD1	25939	ENSG00000101347	25890101	validated
mirtarbase	hsa-miR-192-5p	TPGS2	25941	ENSG00000134779	19074876	validated
mirtarbase	hsa-miR-21-5p	C20orf194	25943	ENSG00000088854	18591254	validated
mirtarbase	hsa-miR-106b-5p	ZNF385A	25946	ENSG00000161642	22012620	validated
mirtarbase	hsa-miR-192-5p	PNKD	25953	ENSG00000127838	22100165	validated
mirtarbase	hsa-miR-181a-5p	PNKD	25953	ENSG00000127838	19536157	validated
mirtarbase	hsa-miR-18a-5p	PNISR	25957	ENSG00000132424	23446348	validated
mirtarbase	hsa-miR-15b-5p	PNISR	25957	ENSG00000132424	22473208	validated
mirtarbase	hsa-miR-106b-5p	KANK2	25959	ENSG00000197256	23622248	validated
mirtarbase	hsa-miR-222-3p	TIPARP	25976	ENSG00000163659	22815788	validated

mirtarbase	hsa-miR-181a-5p	CHMP2B	25978	ENSG00000083937	21572407	validated
mirtarbase	hsa-miR-106b-5p	TSKU	25987	ENSG00000182704	20371350	validated
mirtarbase	hsa-miR-15b-5p	HIGD1A	25994	ENSG00000181061	21572407	validated
mirtarbase	hsa-miR-21-5p	MOXD1	26002	ENSG00000079931	18591254	validated
mirtarbase	hsa-miR-21-5p	SPATS2L	26010	ENSG00000196141	19253296	validated
mirtarbase	hsa-miR-222-3p	NSMF	26012	ENSG00000165802	23622248	validated
mirtarbase	hsa-miR-192-5p	LRIG1	26018	ENSG00000144749	19074876	validated
mirtarbase	hsa-miR-222-3p	LRP10	26020	ENSG00000197324	23622248	validated
mirtarbase	hsa-miR-505-3p	PTCD1	26024	ENSG00000106246	23622248	validated
mirtarbase	hsa-miR-505-3p	PLEKHG3	26030	ENSG00000126822	27292025	validated
mirtarbase	hsa-miR-21-5p	OSBPL3	26031	ENSG00000070882	18591254	validated
mirtarbase	hsa-miR-15b-5p	OSBPL3	26031	ENSG00000070882	20371350	validated
mirtarbase	hsa-miR-181a-5p	OSBPL3	26031	ENSG00000070882	22473208	validated
mirtarbase	hsa-miR-192-5p	ATRNL1	26033	ENSG00000107518	19074876	validated
mirtarbase	hsa-miR-181a-5p	SIPA1L1	26037	ENSG00000197555	22473208	validated
mirtarbase	hsa-miR-21-5p	AUTS2	26053	ENSG00000158321	19253296	validated
mirtarbase	hsa-miR-222-3p	AUTS2	26053	ENSG00000158321	23622248	validated
mirtarbase	hsa-miR-582-5p	ANKRD17	26057	ENSG00000132466	22100165	validated
mirtarbase	hsa-miR-505-3p	GIGYF2	26058	ENSG00000204120	23622248	validated
mirtarbase	hsa-miR-192-5p	APPL1	26060	ENSG00000157500	19074876	validated
mirtarbase	hsa-miR-21-5p	APPL1	26060	ENSG00000157500	18591254	validated
mirtarbase	hsa-miR-21-5p	RAI14	26064	ENSG00000039560	18591254	validated
mirtarbase	hsa-miR-15b-5p	LSM14A	26065	ENSG00000257103	23622248	validated
mirtarbase	hsa-miR-144-3p	LSM14A	26065	ENSG00000257103	23446348	validated
mirtarbase	hsa-miR-18a-5p	RTL8A	26071	ENSG00000203950	20371350	validated
mirtarbase	hsa-miR-326	RTL8A	26071	ENSG00000203950	23446348	validated
mirtarbase	hsa-miR-192-5p	ARHGEF26	26084	ENSG00000277101	19074876	validated
mirtarbase	hsa-miR-301a-3p	ARHGEF26	26084	ENSG00000277101	21572407	validated
mirtarbase	hsa-miR-222-3p	HERC4	26091	ENSG00000148634	23622248	validated
mirtarbase	hsa-miR-192-5p	TOR1AIP1	26092	ENSG00000143337	19074876	validated
mirtarbase	hsa-miR-181a-5p	DCAF4	26094	ENSG00000119599	22100165	validated
mirtarbase	hsa-miR-18a-5p	DCAF4	26094	ENSG00000119599	22100165	validated
mirtarbase	hsa-miR-192-5p	EDRF1	26098	ENSG00000107938	19074876	validated
mirtarbase	hsa-miR-21-5p	EDRF1	26098	ENSG00000107938	22473208	validated
mirtarbase	hsa-miR-15b-5p	SZRD1	26099	ENSG00000055070	21572407	validated
mirtarbase	hsa-miR-15b-5p	WIPI2	26100	ENSG00000157954	22473208	validated
mirtarbase	hsa-miR-21-5p	WSB1	26118	ENSG00000109046	22473208	validated
mirtarbase	hsa-miR-106b-5p	WSB1	26118	ENSG00000109046	23446348	validated
mirtarbase	hsa-miR-301a-3p	KIFBP	26128	ENSG00000198954	23622248	validated
mirtarbase	hsa-miR-21-5p	GAPVD1	26130	ENSG00000165219	18591254	validated
mirtarbase	hsa-miR-106b-5p	TRPC4AP	26133	ENSG00000100991	23622248	validated
mirtarbase	hsa-miR-326	TRPC4AP	26133	ENSG00000100991	23824327	validated
mirtarbase	hsa-miR-505-3p	SERBP1	26135	ENSG00000142864	23622248	validated
mirtarbase	hsa-miR-15b-5p	SERBP1	26135	ENSG00000142864	23622248	validated
mirtarbase	hsa-miR-18a-5p	SERBP1	26135	ENSG00000142864	23622248	validated
mirtarbase	hsa-miR-21-5p	ZBTB20	26137	ENSG00000181722	18591254	validated
mirtarbase	hsa-miR-144-3p	ZBTB20	26137	ENSG00000181722	22291592	validated
mirtarbase	hsa-miR-192-5p	PHF19	26147	ENSG00000119403	19074876	validated
mirtarbase	hsa-miR-15b-5p	PHF19	26147	ENSG00000119403	20371350	validated
mirtarbase	hsa-miR-192-5p	FBXW2	26190	ENSG00000119402	19074876	validated
mirtarbase	hsa-miR-106b-5p	FBXW2	26190	ENSG00000119402	23622248	validated
mirtarbase	hsa-miR-222-3p	FBXW2	26190	ENSG00000119402	23622248	validated
mirtarbase	hsa-miR-181a-5p	PTPN22	26191	ENSG00000134242	17382377	validated
mirtarbase	hsa-miR-192-5p	PITPNC1	26207	ENSG00000154217	19074876	validated
mirtarbase	hsa-miR-18a-5p	FBXL3	26224	ENSG00000005812	21572407	validated
mirtarbase	hsa-miR-106b-5p	FBXL5	26234	ENSG00000118564	22473208	validated
mirtarbase	hsa-miR-18a-5p	KLHL3	26249	ENSG00000146021	27418678	validated
mirtarbase	hsa-miR-222-3p	CABYR	26256	ENSG00000154040	23622248	validated
mirtarbase	hsa-miR-192-5p	BLOC1S6	26258	ENSG00000104164	23824327	validated
mirtarbase	hsa-miR-192-5p	FBXO25	26260	ENSG00000147364	19074876	validated
mirtarbase	hsa-miR-106b-5p	FBXO22	26263	ENSG00000167196	23622248	validated
mirtarbase	hsa-miR-106b-5p	FBXO10	26267	ENSG00000147912	22473208	validated
mirtarbase	hsa-miR-192-5p	FBXO5	26271	ENSG00000112029	19074876	validated
mirtarbase	hsa-miR-192-5p	FBXO4	26272	ENSG00000151876	19074876	validated
mirtarbase	hsa-miR-192-5p	FBXO3	26273	ENSG00000110429	19074876	validated
mirtarbase	hsa-miR-21-5p	FBXO3	26273	ENSG00000110429	18591254	validated
mirtarbase	hsa-miR-15b-5p	VPS33B	26276	ENSG00000184056	22473208	validated
mirtarbase	hsa-miR-106b-5p	SACS	26278	ENSG00000151835	22473208	validated
mirtarbase	hsa-miR-21-5p	SLC17A5	26503	ENSG00000119899	18591254	validated
mirtarbase	hsa-miR-326	CNNM4	26504	ENSG00000158158	23592263	validated
mirtarbase	hsa-miR-15b-5p	HEYL	26508	ENSG00000163909	23592263	validated

mirtarbase	hsa-miR-15b-5p	TIMM13	26517	ENSG00000099800	22473208	validated
mirtarbase	hsa-miR-106b-5p	AGO1	26523	ENSG00000092847	17242205	validated
mirtarbase	hsa-miR-326	AGO1	26523	ENSG00000092847	23622248	validated
mirtarbase	hsa-miR-212-3p	AGO1	26523	ENSG00000092847	23622248	validated
mirtarbase	hsa-miR-222-3p	DAZAP1	26528	ENSG00000071626	23622248	validated
mirtarbase	hsa-miR-181a-5p	OR11A1	26531	ENSG00000232289	17612493	validated
mirtarbase	hsa-miR-192-5p	PCOLCE2	26577	ENSG00000163710	19074876	validated
mirtarbase	hsa-miR-106b-5p	BSCL2	26580	ENSG00000168000	23592263	validated
mirtarbase	hsa-miR-222-3p	CKAP2	26586	ENSG00000136108	23622248	validated
mirtarbase	hsa-miR-106b-5p	CKAP2	26586	ENSG00000136108	20371350	validated
mirtarbase	hsa-miR-192-5p	NUFIP1	26747	ENSG00000083635	19074876	validated
mirtarbase	hsa-miR-106b-5p	HBP1	26959	ENSG00000105856	22473208	validated
mirtarbase	hsa-miR-301a-3p	HBP1	26959	ENSG00000105856	23592263	validated
mirtarbase	hsa-miR-21-5p	NBEA	26960	ENSG00000172915	18591254	validated
mirtarbase	hsa-miR-18a-5p	NBEA	26960	ENSG00000172915	23622248	validated
mirtarbase	hsa-miR-222-3p	CHORDC1	26973	ENSG00000110172	21572407	validated
mirtarbase	hsa-miR-144-3p	CHORDC1	26973	ENSG00000110172	20371350	validated
mirtarbase	hsa-miR-21-5p	AP3M1	26985	ENSG00000185009	18591254	validated
mirtarbase	hsa-miR-15b-5p	AP3M1	26985	ENSG00000185009	22473208	validated
mirtarbase	hsa-miR-181a-5p	PABPC1	26986	ENSG00000070756	23622248	validated
mirtarbase	hsa-miR-21-5p	RNF11	26994	ENSG00000123091	20048743	validated
mirtarbase	hsa-miR-301a-3p	RNF11	26994	ENSG00000123091	20371350	validated
mirtarbase	hsa-miR-106b-5p	TPK1	27010	ENSG00000196511	22012620	validated
mirtarbase	hsa-miR-144-3p	BEX3	27018	ENSG00000166681	23592263	validated
mirtarbase	hsa-miR-192-5p	NPHP3	27031	ENSG00000113971	19074876	validated
mirtarbase	hsa-miR-18a-5p	PELP1	27043	ENSG00000141456	23622248	validated
mirtarbase	hsa-miR-181a-5p	ANKRD1	27063	ENSG00000148677	17612493	validated
mirtarbase	hsa-miR-222-3p	TSPAN13	27075	ENSG00000106537	21572407	validated
mirtarbase	hsa-miR-106b-5p	UQCRQ	27089	ENSG00000164405	23622248	validated
mirtarbase	hsa-miR-296-5p	BBC3	27113	ENSG00000105327	21633093	validated
mirtarbase	hsa-miR-582-3p	DKK3	27122	ENSG00000050165	26468775	validated
mirtarbase	hsa-miR-222-3p	DKK2	27123	ENSG00000155011	23587485	validated
mirtarbase	hsa-miR-18a-5p	AFF4	27125	ENSG00000072364	23622248	validated
mirtarbase	hsa-miR-181a-5p	AFF4	27125	ENSG00000072364	22473208	validated
mirtarbase	hsa-miR-15b-5p	AFF4	27125	ENSG00000072364	22473208	validated
mirtarbase	hsa-miR-301a-3p	SNX5	27131	ENSG00000089006	22291592	validated
mirtarbase	hsa-miR-106b-5p	MORC1	27136	ENSG00000114487	22291592	validated
mirtarbase	hsa-miR-505-3p	INTU	27152	ENSG00000164066	23824327	validated
mirtarbase	hsa-miR-106b-5p	ZNF777	27153	ENSG00000196453	23622248	validated
mirtarbase	hsa-miR-192-5p	AGO2	27161	ENSG00000123908	19074876	validated
mirtarbase	hsa-miR-21-5p	AGO2	27161	ENSG00000123908	20371350	validated
mirtarbase	hsa-miR-15b-5p	AGO2	27161	ENSG00000123908	23592263	validated
mirtarbase	hsa-miR-222-3p	AGO2	27161	ENSG00000123908	21572407	validated
mirtarbase	hsa-miR-106b-5p	SALL3	27164	ENSG00000263310	22012620	validated
mirtarbase	hsa-miR-301a-3p	SALL3	27164	ENSG00000263310	22012620	validated
mirtarbase	hsa-miR-192-5p	SLC39A1	27173	ENSG00000143570	19074876	validated
mirtarbase	hsa-miR-301a-3p	SIGLEC9	27180	ENSG00000129450	27292025	validated
mirtarbase	hsa-miR-15b-5p	VPS4A	27183	ENSG00000132612	24398324	validated
mirtarbase	hsa-miR-301a-3p	GPR82	27197	ENSG00000171657	23824327	validated
mirtarbase	hsa-miR-181a-5p	GPR78	27201	ENSG00000155269	23085757	validated
mirtarbase	hsa-miR-181a-5p	NMRK2	27231	ENSG00000077009	17612493	validated
mirtarbase	hsa-miR-106b-5p	TNFRSF21	27242	ENSG00000146072	22473208	validated
mirtarbase	hsa-miR-106b-5p	SESN1	27244	ENSG00000080546	27292025	validated
mirtarbase	hsa-miR-106b-5p	RNF115	27246	ENSG00000265491	23313552	validated
mirtarbase	hsa-miR-106b-5p	MMADHC	27249	ENSG00000168288	23622248	validated
mirtarbase	hsa-miR-15b-5p	PDCD4	27250	ENSG00000150593	22473208	validated
mirtarbase	hsa-miR-106b-5p	KLHL20	27252	ENSG00000076321	22473208	validated
mirtarbase	hsa-miR-106b-5p	LSM3	27258	ENSG00000170860	23824327	validated
mirtarbase	hsa-miR-192-5p	DIMT1	27292	ENSG00000086189	19074876	validated
mirtarbase	hsa-miR-192-5p	PDLIM3	27295	ENSG00000154553	19074876	validated
mirtarbase	hsa-miR-106b-5p	CRCP	27297	ENSG00000241258	23446348	validated
mirtarbase	hsa-miR-326	MOCS3	27304	ENSG00000124217	23824327	validated
mirtarbase	hsa-miR-106b-5p	RAB30	27314	ENSG00000137502	22473208	validated
mirtarbase	hsa-miR-144-3p	TNRC18P2	27320		21572407	validated
mirtarbase	hsa-miR-106b-5p	TNRC6A	27327	ENSG00000090905	22473208	validated
mirtarbase	hsa-miR-301a-3p	TNRC6A	27327	ENSG00000090905	23592263	validated
mirtarbase	hsa-miR-582-5p	TNRC6A	27327	ENSG00000090905	23592263	validated
mirtarbase	hsa-miR-192-5p	KCNMB4	27345	ENSG00000135643	19074876	validated
mirtarbase	hsa-miR-505-3p	KCNMB4	27345	ENSG00000135643	23824327	validated
mirtarbase	hsa-miR-192-5p	TOR1B	27348	ENSG00000136816	19074876	validated
mirtarbase	hsa-miR-106b-5p	POLM	27434	ENSG00000122678	23824327	validated

mirtarbase	hsa-miR-212-3p	EML4	27436	ENSG00000143924	23592263	validated
mirtarbase	hsa-miR-192-5p	PPP2R3B	28227	ENSG00000276438	19074876	validated
mirtarbase	hsa-miR-192-5p	SLCO3A1	28232	ENSG00000176463	19074876	validated
mirtarbase	hsa-miR-15b-5p	SLCO3A1	28232	ENSG00000176463	23313552	validated
mirtarbase	hsa-miR-106b-5p	NKIRAS1	28512	ENSG00000197885	20371350	validated
mirtarbase	hsa-miR-106b-5p	TRIB2	28951	ENSG00000071575	23622248	validated
mirtarbase	hsa-miR-106b-5p	OSTM1	28962	ENSG00000081087	17242205	validated
mirtarbase	hsa-miR-106b-5p	C11orf54	28970	ENSG00000182919	23313552	validated
mirtarbase	hsa-miR-106b-5p	SPCS1	28972	ENSG00000114902	23824327	validated
mirtarbase	hsa-miR-181a-5p	TMEM14A	28978	ENSG00000096092	17612493	validated
mirtarbase	hsa-miR-192-5p	FLVCR1	28982	ENSG00000162769	19074876	validated
mirtarbase	hsa-miR-192-5p	NOB1	28987	ENSG00000141101	19074876	validated
mirtarbase	hsa-miR-326	NOB1	28987	ENSG00000141101	23869222	validated
mirtarbase	hsa-miR-222-3p	DBNL	28988	ENSG00000136279	23622248	validated
mirtarbase	hsa-miR-181a-5p	HIPK2	28996	ENSG00000064393	21274007	validated
mirtarbase	hsa-miR-222-3p	HIPK2	28996	ENSG00000064393	23622248	validated
mirtarbase	hsa-miR-192-5p	MRPL13	28998	ENSG00000172172	19074876	validated
mirtarbase	hsa-miR-106b-5p	ATAD2	29028	ENSG00000156802	17242205	validated
mirtarbase	hsa-miR-192-5p	ATAD2	29028	ENSG00000156802	19074876	validated
mirtarbase	hsa-miR-15b-5p	C16orf72	29035	ENSG00000182831	22473208	validated
mirtarbase	hsa-miR-106b-5p	SETD2	29072	ENSG00000181555	23622248	validated
mirtarbase	hsa-miR-21-5p	SETD2	29072	ENSG00000181555	24983504	validated
mirtarbase	hsa-miR-181a-5p	PHPT1	29085	ENSG00000054148	23622248	validated
mirtarbase	hsa-miR-181a-5p	LGALSL	29094	ENSG00000119862	20371350	validated
mirtarbase	hsa-miR-15b-5p	SSU72	29101	ENSG00000160075	21572407	validated
mirtarbase	hsa-miR-505-3p	DROSHA	29102	ENSG00000113360	23622248	validated
mirtarbase	hsa-miR-222-3p	MYLIP	29116	ENSG00000007944	23446348	validated
mirtarbase	hsa-miR-106b-5p	MYLIP	29116	ENSG00000007944	23592263	validated
mirtarbase	hsa-miR-301a-3p	MYLIP	29116	ENSG00000007944	20371350	validated
mirtarbase	hsa-miR-15b-5p	CLEC2D	29121	ENSG00000069493	20371350	validated
mirtarbase	hsa-miR-582-5p	ANKRD11	29123	ENSG00000167522	23592263	validated
mirtarbase	hsa-miR-144-3p	ANKRD11	29123	ENSG00000167522	23592263	validated
mirtarbase	hsa-miR-326	CD274	29126	ENSG00000120217	24577056	validated
mirtarbase	hsa-miR-15b-5p	CD274	29126	ENSG00000120217	27292025	validated
mirtarbase	hsa-miR-192-5p	RACGAP1	29127	ENSG00000161800	19074876	validated
mirtarbase	hsa-miR-15b-5p	RACGAP1	29127	ENSG00000161800	21572407	validated
mirtarbase	hsa-miR-106b-5p	RACGAP1	29127	ENSG00000161800	20371350	validated
mirtarbase	hsa-miR-301a-3p	RACGAP1	29127	ENSG00000161800	20371350	validated
mirtarbase	hsa-miR-144-3p	USP25	29761	ENSG00000155313	20371350	validated
mirtarbase	hsa-miR-505-3p	TMOD3	29766	ENSG00000138594	23622248	validated
mirtarbase	hsa-miR-301a-3p	TMOD3	29766	ENSG00000138594	23592263	validated
mirtarbase	hsa-miR-106b-5p	TMOD3	29766	ENSG00000138594	23313552	validated
mirtarbase	hsa-miR-15b-5p	CARD10	29775	ENSG00000100065	23592263	validated
mirtarbase	hsa-miR-222-3p	OLA1	29789	ENSG00000138430	23622248	validated
mirtarbase	hsa-miR-296-5p	ZDHHC8	29801	ENSG00000099904	19536157	validated
mirtarbase	hsa-miR-192-5p	GRHL1	29841	ENSG00000134317	19074876	validated
mirtarbase	hsa-miR-192-5p	TFCP2L1	29842	ENSG00000115112	19074876	validated
mirtarbase	hsa-miR-192-5p	SENP1	29843	ENSG00000079387	19074876	validated
mirtarbase	hsa-miR-106b-5p	SENP1	29843	ENSG00000079387	22473208	validated
mirtarbase	hsa-miR-106b-5p	CNOT7	29883	ENSG00000198791	23622248	validated
mirtarbase	hsa-miR-192-5p	SNX10	29887	ENSG00000086300	19074876	validated
mirtarbase	hsa-miR-222-3p	RBM15B	29890	ENSG00000259956	23622248	validated
mirtarbase	hsa-miR-192-5p	PSMC3IP	29893	ENSG00000131470	19074876	validated
mirtarbase	hsa-miR-192-5p	GPSM2	29899	ENSG00000121957	19074876	validated
mirtarbase	hsa-miR-192-5p	UBIAD1	29914	ENSG00000120942	19074876	validated
mirtarbase	hsa-miR-222-3p	UBIAD1	29914	ENSG00000120942	23622248	validated
mirtarbase	hsa-miR-15b-5p	HCFC2	29915	ENSG00000111727	21572407	validated
mirtarbase	hsa-miR-15b-5p	SNX11	29916	ENSG00000002919	22473208	validated
mirtarbase	hsa-miR-18a-5p	SEC61A1	29927	ENSG00000058262	23592263	validated
mirtarbase	hsa-miR-15b-5p	SEC61A1	29927	ENSG00000058262	26701625	validated
mirtarbase	hsa-miR-21-5p	DSE	29940	ENSG00000111817	18591254	validated
mirtarbase	hsa-miR-21-5p	PURG	29942	ENSG00000172733	23446348	validated
mirtarbase	hsa-miR-301a-3p	PURG	29942	ENSG00000172733	21572407	validated
mirtarbase	hsa-miR-18a-5p	SERTAD3	29946	ENSG00000167565	20371350	validated
mirtarbase	hsa-miR-222-3p	CERS2	29956	ENSG00000143418	22393241	validated
mirtarbase	hsa-miR-106b-5p	NRBP1	29959	ENSG00000115216	22473208	validated
mirtarbase	hsa-miR-222-3p	PRICKLE4	29964	ENSG00000278224	23622248	validated
mirtarbase	hsa-miR-106b-5p	PRICKLE4	29964	ENSG00000278224	23592263	validated
mirtarbase	hsa-miR-106b-5p	LRP12	29967	ENSG00000147650	20371350	validated
mirtarbase	hsa-miR-15b-5p	PSAT1	29968	ENSG00000135069	22473208	validated
mirtarbase	hsa-miR-18a-5p	PSAT1	29968	ENSG00000135069	23592263	validated

mirtarbase	hsa-miR-106b-5p	A1CF	29974	ENSG00000148584	22100165	validated
mirtarbase	hsa-miR-301a-3p	NRBF2	29982	ENSG00000148572	24398324	validated
mirtarbase	hsa-miR-582-5p	BICRA	29998	ENSG00000063169	20371350	validated
mirtarbase	hsa-miR-106b-5p	ERO1A	30001	ENSG00000197930	23622248	validated
mirtarbase	hsa-miR-582-5p	ERO1A	30001	ENSG00000197930	26701625	validated
mirtarbase	hsa-miR-106b-5p	DNTTIP2	30836	ENSG00000067334	23446348	validated
mirtarbase	hsa-miR-106b-5p	SOCS7	30837	ENSG00000274211	21572407	validated
mirtarbase	hsa-miR-144-3p	EHD2	30846	ENSG00000024422	26701625	validated
mirtarbase	hsa-miR-192-5p	PIK3R4	30849	ENSG00000196455	16822819	validated
mirtarbase	hsa-miR-222-3p	MINK1	50488	ENSG00000141503	23622248	validated
mirtarbase	hsa-miR-15b-5p	MINK1	50488	ENSG00000141503	22473208	validated
mirtarbase	hsa-miR-106b-5p	MINK1	50488	ENSG00000141503	22473208	validated
mirtarbase	hsa-miR-21-5p	ITSN2	50618	ENSG00000198399	18591254	validated
mirtarbase	hsa-miR-144-3p	ITSN2	50618	ENSG00000198399	23592263	validated
mirtarbase	hsa-miR-192-5p	CUZD1	50624	ENSG00000138161	19074876	validated
mirtarbase	hsa-miR-21-5p	DCAF8	50717	ENSG00000132716	20048743	validated
mirtarbase	hsa-miR-18a-5p	DCAF8	50717	ENSG00000132716	23592263	validated
mirtarbase	hsa-miR-106b-5p	DCAF8	50717	ENSG00000132716	20371350	validated
mirtarbase	hsa-miR-21-5p	MYEF2	50804	ENSG00000104177	18591254	validated
mirtarbase	hsa-miR-192-5p	IRX4	50805	ENSG00000113430	19074876	validated
mirtarbase	hsa-miR-106b-5p	ASAP1	50807	ENSG00000153317	23622248	validated
mirtarbase	hsa-miR-192-5p	COPS7A	50813	ENSG00000111652	19074876	validated
mirtarbase	hsa-miR-222-3p	TRAT1	50852	ENSG00000163519	22291592	validated
mirtarbase	hsa-miR-192-5p	RNF141	50862	ENSG00000110315	19074876	validated
mirtarbase	hsa-miR-192-5p	CDON	50937	ENSG00000064309	19074876	validated
mirtarbase	hsa-miR-21-5p	FOXP3	50943	ENSG00000049768	26383248	validated
mirtarbase	hsa-miR-301a-3p	TMED5	50999	ENSG00000117500	23622248	validated
mirtarbase	hsa-miR-144-3p	TMED5	50999	ENSG00000117500	23592263	validated
mirtarbase	hsa-miR-192-5p	MTERF3	51001	ENSG00000156469	19074876	validated
mirtarbase	hsa-miR-15b-5p	ASCC1	51008	ENSG00000138303	22100165	validated
mirtarbase	hsa-miR-222-3p	DERL2	51009	ENSG00000072849	23824327	validated
mirtarbase	hsa-miR-505-3p	FAHD2A	51011	ENSG00000115042	23824327	validated
mirtarbase	hsa-miR-222-3p	EXOSC1	51013	ENSG00000171311	23622248	validated
mirtarbase	hsa-miR-222-3p	TMED7	51014	ENSG00000134970	21226887	validated
mirtarbase	hsa-miR-582-5p	TMED7	51014	ENSG00000134970	23592263	validated
mirtarbase	hsa-miR-106b-5p	ISOC1	51015	ENSG00000066583	21572407	validated
mirtarbase	hsa-miR-505-3p	FIS1	51024	ENSG00000214253	23622248	validated
mirtarbase	hsa-miR-106b-5p	GOLT1B	51026	ENSG00000111711	23622248	validated
mirtarbase	hsa-miR-21-5p	VPS36	51028	ENSG00000136100	18591254	validated
mirtarbase	hsa-miR-106b-5p	ZBTB7B	51043	ENSG00000160685	23622248	validated
mirtarbase	hsa-miR-301a-3p	ZBTB7B	51043	ENSG00000160685	23592263	validated
mirtarbase	hsa-miR-212-3p	GMNN	51053	ENSG00000112312	27292025	validated
mirtarbase	hsa-miR-15b-5p	ZNF691	51058	ENSG00000164011	23446348	validated
mirtarbase	hsa-miR-222-3p	DERA	51071	ENSG00000023697	23622248	validated
mirtarbase	hsa-miR-15b-5p	FCF1	51077	ENSG00000119616	21572407	validated
mirtarbase	hsa-miR-15b-5p	SIDT2	51092	ENSG00000149577	22100165	validated
mirtarbase	hsa-miR-15b-5p	SCCPDH	51097	ENSG00000143653	23622248	validated
mirtarbase	hsa-miR-106b-5p	SH3GLB1	51100	ENSG00000097033	21572407	validated
mirtarbase	hsa-miR-21-5p	PHF20L1	51105	ENSG00000129292	18591254	validated
mirtarbase	hsa-miR-212-3p	PHF20L1	51105	ENSG00000129292	23824327	validated
mirtarbase	hsa-miR-15b-5p	TFB1M	51106	ENSG00000029639	22473208	validated
mirtarbase	hsa-miR-192-5p	METTL9	51108	ENSG00000197006	19074876	validated
mirtarbase	hsa-miR-21-5p	RDH11	51109	ENSG00000072042	17363372	validated
mirtarbase	hsa-miR-301a-3p	RDH11	51109	ENSG00000072042	21572407	validated
mirtarbase	hsa-miR-106b-5p	KMT5B	51111	ENSG00000110066	22012620	validated
mirtarbase	hsa-miR-106b-5p	ZNF706	51123	ENSG00000120963	23622248	validated
mirtarbase	hsa-miR-301a-3p	IER3IP1	51124	ENSG00000134049	21572407	validated
mirtarbase	hsa-miR-212-3p	IER3IP1	51124	ENSG00000134049	23313552	validated
mirtarbase	hsa-miR-144-3p	GOLGA7	51125	ENSG00000147533	21572407	validated
mirtarbase	hsa-miR-192-5p	NAA20	51126	ENSG00000173418	19074876	validated
mirtarbase	hsa-miR-15b-5p	RLIM	51132	ENSG00000131263	23622248	validated
mirtarbase	hsa-miR-106b-5p	RLIM	51132	ENSG00000131263	21572407	validated
mirtarbase	hsa-miR-18a-5p	RLIM	51132	ENSG00000131263	21572407	validated
mirtarbase	hsa-miR-301a-3p	RLIM	51132	ENSG00000131263	21572407	validated
mirtarbase	hsa-miR-181a-5p	RLIM	51132	ENSG00000131263	19536157	validated
mirtarbase	hsa-miR-181a-5p	KCTD3	51133	ENSG00000136636	17612493	validated
mirtarbase	hsa-miR-192-5p	IRAK4	51135	ENSG00000198001	19074876	validated
mirtarbase	hsa-miR-212-3p	IRAK4	51135	ENSG00000198001	23264652	validated
mirtarbase	hsa-miR-106b-5p	IRAK4	51135	ENSG00000198001	23313552	validated
mirtarbase	hsa-miR-192-5p	RNFT1	51136	ENSG00000189050	19074876	validated
mirtarbase	hsa-miR-301a-3p	CERCAM	51148	ENSG00000167123	21572407	validated

mirtarbase	hsa-miR-106b-5p	CERCAM	51148	ENSG00000167123	21572407	validated
mirtarbase	hsa-miR-106b-5p	JPT1	51155	ENSG00000189159	17242205	validated
mirtarbase	hsa-miR-301a-3p	C3orf18	51161	ENSG00000088543	19536157	validated
mirtarbase	hsa-miR-222-3p	HSPA14	51182	ENSG00000284024	23622248	validated
mirtarbase	hsa-miR-192-5p	CKLF	51192	ENSG00000217555	19074876	validated
mirtarbase	hsa-miR-192-5p	PLCE1	51196	ENSG00000138193	19074876	validated
mirtarbase	hsa-miR-192-5p	NIN	51199	ENSG00000100503	19074876	validated
mirtarbase	hsa-miR-21-5p	NIN	51199	ENSG00000100503	18591254	validated
mirtarbase	hsa-miR-106b-5p	NIN	51199	ENSG00000100503	23592263	validated
mirtarbase	hsa-miR-181a-5p	NIN	51199	ENSG00000100503	22473208	validated
mirtarbase	hsa-miR-192-5p	ZDHHC2	51201	ENSG00000104219	19074876	validated
mirtarbase	hsa-miR-301a-3p	ACP6	51205	ENSG00000162836	23824327	validated
mirtarbase	hsa-miR-222-3p	GLRX5	51218	ENSG00000182512	23622248	validated
mirtarbase	hsa-miR-18a-5p	GLRX5	51218	ENSG00000182512	23622248	validated
mirtarbase	hsa-miR-582-5p	GLRX5	51218	ENSG00000182512	21572407	validated
mirtarbase	hsa-miR-21-5p	PHF20	51230	ENSG00000025293	18591254	validated
mirtarbase	hsa-miR-15b-5p	CRIM1	51232	ENSG00000277354	24435757	validated
mirtarbase	hsa-miR-18a-5p	CRIM1	51232	ENSG00000277354	22012620	validated
mirtarbase	hsa-miR-192-5p	CCDC174	51244	ENSG00000154781	19074876	validated
mirtarbase	hsa-miR-222-3p	PAIP2	51247	ENSG00000120727	23622248	validated
mirtarbase	hsa-miR-106b-5p	PDZD11	51248	ENSG00000120509	22473208	validated
mirtarbase	hsa-miR-301a-3p	PDZD11	51248	ENSG00000120509	23592263	validated
mirtarbase	hsa-miR-15b-5p	TMEM69	51249	ENSG00000159596	22473208	validated
mirtarbase	hsa-miR-582-5p	NT5C3A	51251	ENSG00000122643	21572407	validated
mirtarbase	hsa-miR-144-3p	NT5C3A	51251	ENSG00000122643	21572407	validated
mirtarbase	hsa-miR-181a-5p	TBC1D7	51256	ENSG00000145979	22473208	validated
mirtarbase	hsa-miR-106b-5p	KLF3	51274	ENSG00000109787	22012620	validated
mirtarbase	hsa-miR-106b-5p	DNAJC27	51277	ENSG00000115137	22473208	validated
mirtarbase	hsa-miR-582-5p	IER5	51278	ENSG00000162783	27292025	validated
mirtarbase	hsa-miR-15b-5p	ANKMY1	51281	ENSG00000144504	23824327	validated
mirtarbase	hsa-miR-106b-5p	TLR7	51284	ENSG00000196664	23313552	validated
mirtarbase	hsa-miR-106b-5p	ERGIC2	51290	ENSG00000087502	23592263	validated
mirtarbase	hsa-miR-222-3p	FAM53C	51307	ENSG00000120709	23622248	validated
mirtarbase	hsa-miR-181a-5p	SLC25A37	51312	ENSG00000147454	22473208	validated
mirtarbase	hsa-miR-18a-5p	SLC25A37	51312	ENSG00000147454	23446348	validated
mirtarbase	hsa-miR-582-3p	SLC25A37	51312	ENSG00000147454	23824327	validated
mirtarbase	hsa-miR-18a-5p	MRPL35	51318	ENSG00000132313	21572407	validated
mirtarbase	hsa-miR-192-5p	RSRC1	51319	ENSG00000174891	19074876	validated
mirtarbase	hsa-miR-505-3p	RSRC1	51319	ENSG00000174891	21572407	validated
mirtarbase	hsa-miR-15b-5p	WAC	51322	ENSG00000095787	23622248	validated
mirtarbase	hsa-miR-18a-5p	WAC	51322	ENSG00000095787	23622248	validated
mirtarbase	hsa-miR-106b-5p	WAC	51322	ENSG00000095787	23592263	validated
mirtarbase	hsa-miR-222-3p	NGRN	51335	ENSG00000182768	23622248	validated
mirtarbase	hsa-miR-192-5p	THEM6	51337	ENSG00000130193	19074876	validated
mirtarbase	hsa-miR-222-3p	ZBTB7A	51341	ENSG00000178951	23622248	validated
mirtarbase	hsa-miR-106b-5p	ZBTB7A	51341	ENSG00000178951	22473208	validated
mirtarbase	hsa-miR-144-3p	ZBTB7A	51341	ENSG00000178951	23313552	validated
mirtarbase	hsa-miR-301a-3p	ZBTB7A	51341	ENSG00000178951	26701625	validated
mirtarbase	hsa-miR-21-5p	UBR5	51366	ENSG00000104517	18591254	validated
mirtarbase	hsa-miR-106b-5p	UBR5	51366	ENSG00000104517	22473208	validated
mirtarbase	hsa-miR-301a-3p	POMP	51371	ENSG00000132963	23622248	validated
mirtarbase	hsa-miR-192-5p	CRLF3	51379	ENSG00000176390	19074876	validated
mirtarbase	hsa-miR-181a-5p	WNT16	51384	ENSG00000002745	17612493	validated
mirtarbase	hsa-miR-301a-3p	NIP7	51388	ENSG00000132603	23824327	validated
mirtarbase	hsa-miR-15b-5p	AIG1	51390	ENSG00000146416	23622248	validated
mirtarbase	hsa-miR-181a-5p	ZNF107	51427	ENSG00000196247	21572407	validated
mirtarbase	hsa-miR-106b-5p	ZNF107	51427	ENSG00000196247	21572407	validated
mirtarbase	hsa-miR-301a-3p	ZNF107	51427	ENSG00000196247	20371350	validated
mirtarbase	hsa-miR-106b-5p	SUCO	51430	ENSG00000094975	22473208	validated
mirtarbase	hsa-miR-106b-5p	ANAPC7	51434	ENSG00000196510	23622248	validated
mirtarbase	hsa-miR-106b-5p	YTHDF2	51441	ENSG00000198492	23622248	validated
mirtarbase	hsa-miR-18a-5p	RNF138	51444	ENSG00000134758	21572407	validated
mirtarbase	hsa-miR-15b-5p	RNF138	51444	ENSG00000134758	22473208	validated
mirtarbase	hsa-miR-192-5p	GULP1	51454	ENSG00000144366	19074876	validated
mirtarbase	hsa-miR-21-5p	REV1	51455	ENSG00000135945	18591254	validated
mirtarbase	hsa-miR-106b-5p	REV1	51455	ENSG00000135945	22473208	validated
mirtarbase	hsa-miR-192-5p	RHCG	51458	ENSG00000140519	22100165	validated
mirtarbase	hsa-miR-106b-5p	LIMA1	51474	ENSG00000050405	22473208	validated
mirtarbase	hsa-miR-106b-5p	CABP2	51475	ENSG00000167791	17242205	validated
mirtarbase	hsa-miR-106b-5p	ANKFY1	51479	ENSG00000185722	23622248	validated
mirtarbase	hsa-miR-181a-5p	HACD3	51495	ENSG00000074696	17612493	validated

mirtarbase	hsa-miR-192-5p	HACD3	51495	ENSG00000074696	19074876	validated
mirtarbase	hsa-miR-192-5p	DTL	51514	ENSG00000143476	19074876	validated
mirtarbase	hsa-miR-192-5p	LARS1	51520	ENSG00000133706	19074876	validated
mirtarbase	hsa-miR-21-5p	LARS1	51520	ENSG00000133706	18591254	validated
mirtarbase	hsa-miR-106b-5p	LARS1	51520	ENSG00000133706	23622248	validated
mirtarbase	hsa-miR-106b-5p	TMEM138	51524	ENSG00000149483	22473208	validated
mirtarbase	hsa-miR-15b-5p	TMEM138	51524	ENSG00000149483	22473208	validated
mirtarbase	hsa-miR-181a-5p	GSKIP	51527	ENSG00000100744	22473208	validated
mirtarbase	hsa-miR-21-5p	VPS54	51542	ENSG00000143952	18591254	validated
mirtarbase	hsa-miR-301a-3p	CINP	51550	ENSG00000100865	23824327	validated
mirtarbase	hsa-miR-301a-3p	RAB14	51552	ENSG00000119396	23592263	validated
mirtarbase	hsa-miR-144-3p	PEX5L	51555	ENSG00000114757	23446348	validated
mirtarbase	hsa-miR-326	PEX5L	51555	ENSG00000114757	22012620	validated
mirtarbase	hsa-miR-106b-5p	LGSN	51557	ENSG00000146166	23313552	validated
mirtarbase	hsa-miR-21-5p	ARMCX3	51566	ENSG00000102401	18591254	validated
mirtarbase	hsa-miR-326	GDE1	51573	ENSG00000006007	23824327	validated
mirtarbase	hsa-miR-21-5p	TRIM33	51592	ENSG00000197323	18591254	validated
mirtarbase	hsa-miR-222-3p	SRRT	51593	ENSG00000087087	23622248	validated
mirtarbase	hsa-miR-192-5p	TAF9B	51616	ENSG00000187325	19074876	validated
mirtarbase	hsa-miR-192-5p	CCZ1	51622	ENSG00000122674	19074876	validated
mirtarbase	hsa-miR-144-3p	DYNC2LI1	51626	ENSG00000138036	21572407	validated
mirtarbase	hsa-miR-222-3p	LUC7L2	51631	ENSG00000146963	23622248	validated
mirtarbase	hsa-miR-15b-5p	PPIL1	51645	ENSG00000137168	23622248	validated
mirtarbase	hsa-miR-15b-5p	CHMP3	51652	ENSG00000115561	22473208	validated
mirtarbase	hsa-miR-181a-5p	FKBP7	51661	ENSG00000079150	17612493	validated
mirtarbase	hsa-miR-505-3p	ASB1	51665	ENSG00000065802	23622248	validated
mirtarbase	hsa-miR-106b-5p	ASB1	51665	ENSG00000065802	22473208	validated
mirtarbase	hsa-miR-181a-5p	ASB1	51665	ENSG00000065802	22473208	validated
mirtarbase	hsa-miR-192-5p	SARAF	51669	ENSG00000133872	19074876	validated
mirtarbase	hsa-miR-106b-5p	HECA	51696	ENSG00000112406	22473208	validated
mirtarbase	hsa-miR-192-5p	NLK	51701	ENSG00000087095	19074876	validated
mirtarbase	hsa-miR-192-5p	RAB23	51715	ENSG00000112210	19074876	validated
mirtarbase	hsa-miR-18a-5p	RAB23	51715	ENSG00000112210	20371350	validated
mirtarbase	hsa-miR-15b-5p	RAB23	51715	ENSG00000112210	21572407	validated
mirtarbase	hsa-miR-192-5p	CMPK1	51727	ENSG00000162368	19074876	validated
mirtarbase	hsa-miR-106b-5p	CMPK1	51727	ENSG00000162368	22473208	validated
mirtarbase	hsa-miR-21-5p	RAPGEF6	51735	ENSG00000158987	18591254	validated
mirtarbase	hsa-miR-106b-5p	ARID4B	51742	ENSG00000054267	22473208	validated
mirtarbase	hsa-miR-15b-5p	LUC7L3	51747	ENSG00000108848	22100165	validated
mirtarbase	hsa-miR-106b-5p	ERAP1	51752	ENSG00000164307	22473208	validated
mirtarbase	hsa-miR-192-5p	RAB8B	51762	ENSG00000166128	19074876	validated
mirtarbase	hsa-miR-15b-5p	TM7SF3	51768	ENSG00000064115	21572407	validated
mirtarbase	hsa-miR-21-5p	RSF1	51773	ENSG00000048649	18591254	validated
mirtarbase	hsa-miR-181a-5p	RSF1	51773	ENSG00000048649	22100165	validated
mirtarbase	hsa-miR-192-5p	SIX4	51804	ENSG00000100625	20371350	validated
mirtarbase	hsa-miR-582-5p	SIX4	51804	ENSG00000100625	20371350	validated
mirtarbase	hsa-miR-144-3p	SIX4	51804	ENSG00000100625	20371350	validated
mirtarbase	hsa-miR-192-5p	COQ3	51805	ENSG00000132423	19074876	validated
mirtarbase	hsa-miR-301a-3p	PHAX	51808	ENSG00000164902	23622248	validated
mirtarbase	hsa-miR-222-3p	PHAX	51808	ENSG00000164902	23622248	validated
mirtarbase	hsa-miR-106b-5p	CHIC1	53344	ENSG00000204116	22473208	validated
mirtarbase	hsa-miR-301a-3p	CHIC1	53344	ENSG00000204116	21572407	validated
mirtarbase	hsa-miR-15b-5p	CHIC1	53344	ENSG00000204116	23446348	validated
mirtarbase	hsa-miR-144-3p	PANK1	53354	ENSG00000152782	23313552	validated
mirtarbase	hsa-miR-15b-5p	PANK1	53354	ENSG00000152782	22473208	validated
mirtarbase	hsa-miR-181a-5p	FXYD6	53826	ENSG00000137726	17612493	validated
mirtarbase	hsa-miR-106b-5p	FXYD5	53827	ENSG00000089327	23446348	validated
mirtarbase	hsa-miR-505-3p	IL20RB	53833	ENSG00000174564	23446348	validated
mirtarbase	hsa-miR-582-5p	IL20RB	53833	ENSG00000174564	23446348	validated
mirtarbase	hsa-miR-144-3p	IL20RB	53833	ENSG00000174564	23446348	validated
mirtarbase	hsa-miR-21-5p	FGFRL1	53834	ENSG00000127418	22473208	validated
mirtarbase	hsa-miR-15b-5p	C11orf24	53838	ENSG00000171067	21572407	validated
mirtarbase	hsa-miR-222-3p	CSNK1G1	53944	ENSG00000169118	23622248	validated
mirtarbase	hsa-miR-326	A4GALT	53947	ENSG00000128274	23824327	validated
mirtarbase	hsa-miR-106b-5p	BRWD1	54014	ENSG00000185658	23622248	validated
mirtarbase	hsa-miR-192-5p	BRWD1	54014	ENSG00000185658	23622248	validated
mirtarbase	hsa-miR-301a-3p	BRWD1	54014	ENSG00000185658	21572407	validated
mirtarbase	hsa-miR-212-3p	BRWD1	54014	ENSG00000185658	21572407	validated
mirtarbase	hsa-miR-222-3p	BRWD1	54014	ENSG00000185658	23313552	validated
mirtarbase	hsa-miR-192-5p	SETD4	54093	ENSG00000185917	16822819	validated
mirtarbase	hsa-miR-192-5p	C21orf91	54149	ENSG00000154642	19074876	validated

mirtarbase	hsa-miR-106b-5p	DCUN1D1	54165	ENSG00000043093	23622248	validated
mirtarbase	hsa-miR-144-3p	DCUN1D1	54165	ENSG00000043093	21572407	validated
mirtarbase	hsa-miR-192-5p	CYCS	54205	ENSG00000172115	23446348	validated
mirtarbase	hsa-miR-106b-5p	CYCS	54205	ENSG00000172115	23446348	validated
mirtarbase	hsa-miR-18a-5p	ERRFI1	54206	ENSG00000116285	23622248	validated
mirtarbase	hsa-miR-505-3p	TREM1	54210	ENSG00000124731	23824327	validated
mirtarbase	hsa-miR-301a-3p	SLC38A2	54407	ENSG00000134294	23592263	validated
mirtarbase	hsa-miR-212-3p	SLC38A2	54407	ENSG00000134294	23592263	validated
mirtarbase	hsa-miR-181a-5p	SLC38A2	54407	ENSG00000134294	22473208	validated
mirtarbase	hsa-miR-106b-5p	DNAJC10	54431	ENSG00000077232	23446348	validated
mirtarbase	hsa-miR-15b-5p	DNAJC10	54431	ENSG00000077232	21572407	validated
mirtarbase	hsa-miR-15b-5p	RBM27	54439	ENSG00000091009	23622248	validated
mirtarbase	hsa-miR-582-5p	RBM27	54439	ENSG00000091009	23592263	validated
mirtarbase	hsa-miR-301a-3p	RBM27	54439	ENSG00000091009	23824327	validated
mirtarbase	hsa-miR-192-5p	ANLN	54443	ENSG00000011426	19074876	validated
mirtarbase	hsa-miR-21-5p	ATAD2B	54454	ENSG00000119778	18591254	validated
mirtarbase	hsa-miR-106b-5p	CCSER2	54462	ENSG00000107771	22473208	validated
mirtarbase	hsa-miR-106b-5p	ANKIB1	54467	ENSG00000001629	22473208	validated
mirtarbase	hsa-miR-181a-5p	ZFAND6	54469	ENSG00000086666	23446348	validated
mirtarbase	hsa-miR-222-3p	NLE1	54475	ENSG00000073536	23622248	validated
mirtarbase	hsa-miR-106b-5p	RNF216	54476	ENSG00000011275	22473208	validated
mirtarbase	hsa-miR-144-3p	NEURL1B	54492	ENSG00000214357	24398324	validated
mirtarbase	hsa-miR-106b-5p	TMX3	54495	ENSG00000166479	22473208	validated
mirtarbase	hsa-miR-301a-3p	TMCO1	54499	ENSG00000143183	23824327	validated
mirtarbase	hsa-miR-326	RBM47	54502	ENSG00000163694	22012620	validated
mirtarbase	hsa-miR-505-3p	PUS7	54517	ENSG00000091127	23622248	validated
mirtarbase	hsa-miR-192-5p	WDR44	54521	ENSG00000131725	16822819	validated
mirtarbase	hsa-miR-106b-5p	MIER2	54531	ENSG00000105556	23622248	validated
mirtarbase	hsa-miR-15b-5p	USP53	54532	ENSG00000145390	24398324	validated
mirtarbase	hsa-miR-192-5p	MRPL50	54534	ENSG00000136897	19074876	validated
mirtarbase	hsa-miR-222-3p	SHLD2	54537	ENSG00000122376	20371350	validated
mirtarbase	hsa-miR-181a-5p	DDIT4	54541	ENSG00000168209	21274007	validated
mirtarbase	hsa-miR-181a-5p	MTMR12	54545	ENSG00000150712	17612493	validated
mirtarbase	hsa-miR-192-5p	MTMR12	54545	ENSG00000150712	19074876	validated
mirtarbase	hsa-miR-21-5p	MTMR12	54545	ENSG00000150712	18591254	validated
mirtarbase	hsa-miR-15b-5p	GNL3L	54552	ENSG00000130119	23622248	validated
mirtarbase	hsa-miR-505-3p	GNL3L	54552	ENSG00000130119	23824327	validated
mirtarbase	hsa-miR-582-3p	GNL3L	54552	ENSG00000130119	23824327	validated
mirtarbase	hsa-miR-21-5p	SGTB	54557	ENSG00000197860	22473208	validated
mirtarbase	hsa-miR-106b-5p	SGTB	54557	ENSG00000197860	22473208	validated
mirtarbase	hsa-miR-296-5p	DLL4	54567	ENSG00000128917	26500043	validated
mirtarbase	hsa-miR-21-5p	EGLN1	54583	ENSG00000135766	27030384	validated
mirtarbase	hsa-miR-192-5p	NDFIP2	54602	ENSG00000102471	19074876	validated
mirtarbase	hsa-miR-192-5p	MINDY2	54629	ENSG00000128923	19074876	validated
mirtarbase	hsa-miR-181a-5p	TBC1D13	54662	ENSG00000107021	22473208	validated
mirtarbase	hsa-miR-192-5p	RSBN1	54665	ENSG00000081019	19074876	validated
mirtarbase	hsa-miR-181a-5p	LRRN3	54674	ENSG00000173114	17612493	validated
mirtarbase	hsa-miR-106b-5p	CROT	54677	ENSG00000005469	17242205	validated
mirtarbase	hsa-miR-106b-5p	RRN3	54700	ENSG00000278494	21572407	validated
mirtarbase	hsa-miR-106b-5p	OTUD4	54726	ENSG00000164164	20371350	validated
mirtarbase	hsa-miR-106b-5p	LEPROT	54741	ENSG00000213625	21572407	validated
mirtarbase	hsa-miR-192-5p	EPDR1	54749	ENSG00000086289	19074876	validated
mirtarbase	hsa-miR-505-3p	KLHDC4	54758	ENSG00000104731	19536157	validated
mirtarbase	hsa-miR-21-5p	ZRANB1	54764	ENSG00000019995	18591254	validated
mirtarbase	hsa-miR-106b-5p	ZRANB1	54764	ENSG00000019995	22473208	validated
mirtarbase	hsa-miR-222-3p	TRIM44	54765	ENSG00000166326	23622248	validated
mirtarbase	hsa-miR-21-5p	RNF111	54778	ENSG00000157450	22473208	validated
mirtarbase	hsa-miR-144-3p	RNF111	54778	ENSG00000157450	23446348	validated
mirtarbase	hsa-miR-212-3p	ALKBH4	54784	ENSG00000160993	23824327	validated
mirtarbase	hsa-miR-106b-5p	MED18	54797	ENSG00000130772	23446348	validated
mirtarbase	hsa-miR-326	MED18	54797	ENSG00000130772	23824327	validated
mirtarbase	hsa-miR-301a-3p	MED18	54797	ENSG00000130772	23824327	validated
mirtarbase	hsa-miR-21-5p	KLHL24	54800	ENSG00000114796	18591254	validated
mirtarbase	hsa-miR-181a-5p	KLHL24	54800	ENSG00000114796	19536157	validated
mirtarbase	hsa-miR-18a-5p	AHI1	54806	ENSG00000135541	23446348	validated
mirtarbase	hsa-miR-181a-5p	ZNF562	54811	ENSG00000171466	17612493	validated
mirtarbase	hsa-miR-106b-5p	ZNF562	54811	ENSG00000171466	23622248	validated
mirtarbase	hsa-miR-181a-5p	AFTPH	54812	ENSG00000119844	20371350	validated
mirtarbase	hsa-miR-21-5p	AFTPH	54812	ENSG00000119844	18591254	validated
mirtarbase	hsa-miR-106b-5p	KLHL28	54813	ENSG00000179454	22473208	validated
mirtarbase	hsa-miR-15b-5p	GATAD2A	54815	ENSG00000167491	26701625	validated

mirtarbase	hsa-miR-18a-5p	GATAD2A	54815	ENSG00000167491	26701625	validated
mirtarbase	hsa-miR-192-5p	NDE1	54820	ENSG00000275911	19074876	validated
mirtarbase	hsa-miR-192-5p	ERCC6L	54821	ENSG00000186871	19074876	validated
mirtarbase	hsa-miR-222-3p	ERCC6L	54821	ENSG00000186871	23622248	validated
mirtarbase	hsa-miR-21-5p	TRPM7	54822	ENSG00000092439	18591254	validated
mirtarbase	hsa-miR-326	BCAS3	54828	ENSG00000141376	28735896	validated
mirtarbase	hsa-miR-192-5p	VPS13C	54832	ENSG00000129003	19074876	validated
mirtarbase	hsa-miR-326	VPS13C	54832	ENSG00000129003	23622248	validated
mirtarbase	hsa-miR-106b-5p	VPS13C	54832	ENSG00000129003	22473208	validated
mirtarbase	hsa-miR-18a-5p	GDAP2	54834	ENSG00000196505	23622248	validated
mirtarbase	hsa-miR-15b-5p	BSPRY	54836	ENSG00000119411	23824327	validated
mirtarbase	hsa-miR-582-3p	WBP1L	54838	ENSG00000166272	22100165	validated
mirtarbase	hsa-miR-192-5p	BIVM	54841	ENSG00000134897	19074876	validated
mirtarbase	hsa-miR-144-3p	MFSD6	54842	ENSG00000151690	23446348	validated
mirtarbase	hsa-miR-106b-5p	TENT5C	54855	ENSG00000183508	22473208	validated
mirtarbase	hsa-miR-106b-5p	GON4L	54856	ENSG00000116580	23622248	validated
mirtarbase	hsa-miR-222-3p	PGPEP1	54858	ENSG00000130517	22012620	validated
mirtarbase	hsa-miR-21-5p	SNRK	54861	ENSG00000163788	18591254	validated
mirtarbase	hsa-miR-15b-5p	QRICH1	54870	ENSG00000198218	23622248	validated
mirtarbase	hsa-miR-301a-3p	PIGG	54872	ENSG00000174227	23824327	validated
mirtarbase	hsa-miR-505-3p	FNBP1L	54874	ENSG00000137942	23622248	validated
mirtarbase	hsa-miR-106b-5p	FNBP1L	54874	ENSG00000137942	23446348	validated
mirtarbase	hsa-miR-144-3p	ZCCHC2	54877	ENSG00000141664	23446348	validated
mirtarbase	hsa-miR-222-3p	DPP8	54878	ENSG00000074603	23622248	validated
mirtarbase	hsa-miR-15b-5p	DPP8	54878	ENSG00000074603	22473208	validated
mirtarbase	hsa-miR-192-5p	BCOR	54880	ENSG00000183337	19074876	validated
mirtarbase	hsa-miR-222-3p	TEX10	54881	ENSG00000136891	23622248	validated
mirtarbase	hsa-miR-505-3p	CWC25	54883	ENSG00000276761	23622248	validated
mirtarbase	hsa-miR-192-5p	UHRF1BP1	54887	ENSG00000065060	19074876	validated
mirtarbase	hsa-miR-21-5p	NSUN2	54888	ENSG00000037474	22473208	validated
mirtarbase	hsa-miR-582-3p	INO80D	54891	ENSG00000114933	22291592	validated
mirtarbase	hsa-miR-582-5p	INO80D	54891	ENSG00000114933	21572407	validated
mirtarbase	hsa-miR-144-3p	INO80D	54891	ENSG00000114933	21572407	validated
mirtarbase	hsa-miR-181a-5p	INO80D	54891	ENSG00000114933	22473208	validated
mirtarbase	hsa-miR-106b-5p	PXK	54899	ENSG00000168297	23592263	validated
mirtarbase	hsa-miR-301a-3p	PXK	54899	ENSG00000168297	23592263	validated
mirtarbase	hsa-miR-192-5p	NSD3	54904	ENSG00000147548	19074876	validated
mirtarbase	hsa-miR-21-5p	NSD3	54904	ENSG00000147548	19398721	validated
mirtarbase	hsa-miR-301a-3p	NSD3	54904	ENSG00000147548	23622248	validated
mirtarbase	hsa-miR-301a-3p	YTHDF1	54915	ENSG00000149658	23622248	validated
mirtarbase	hsa-miR-505-3p	YTHDF1	54915	ENSG00000149658	20371350	validated
mirtarbase	hsa-miR-192-5p	CMTM6	54918	ENSG00000091317	19074876	validated
mirtarbase	hsa-miR-144-3p	CMTM6	54918	ENSG00000091317	20371350	validated
mirtarbase	hsa-miR-106b-5p	OCIAD1	54940	ENSG00000109180	23313552	validated
mirtarbase	hsa-miR-301a-3p	RNF125	54941	ENSG00000101695	19536157	validated
mirtarbase	hsa-miR-144-3p	DNAJC28	54943	ENSG00000262911	22291592	validated
mirtarbase	hsa-miR-106b-5p	DNAJC28	54943	ENSG00000262911	21572407	validated
mirtarbase	hsa-miR-18a-5p	FAM120C	54954	ENSG00000184083	20371350	validated
mirtarbase	hsa-miR-181a-5p	C1orf109	54955	ENSG00000116922	17612493	validated
mirtarbase	hsa-miR-192-5p	PARP16	54956	ENSG00000138617	19074876	validated
mirtarbase	hsa-miR-106b-5p	GEMIN8	54960	ENSG00000046647	23313552	validated
mirtarbase	hsa-miR-192-5p	PIGX	54965	ENSG00000163964	19074876	validated
mirtarbase	hsa-miR-21-5p	PIGX	54965	ENSG00000163964	18591254	validated
mirtarbase	hsa-miR-106b-5p	SLC35F6	54978	ENSG00000213699	22927820	validated
mirtarbase	hsa-miR-15b-5p	C2orf42	54980	ENSG00000115998	22473208	validated
mirtarbase	hsa-miR-192-5p	CLN6	54982	ENSG00000128973	19074876	validated
mirtarbase	hsa-miR-222-3p	ZNF770	54989	ENSG00000198146	23622248	validated
mirtarbase	hsa-miR-18a-5p	ZNF770	54989	ENSG00000198146	23622248	validated
mirtarbase	hsa-miR-106b-5p	ZNF770	54989	ENSG00000198146	22473208	validated
mirtarbase	hsa-miR-192-5p	GID8	54994	ENSG00000101193	19074876	validated
mirtarbase	hsa-miR-15b-5p	AURKAIP1	54998	ENSG00000175756	26701625	validated
mirtarbase	hsa-miR-144-3p	TUG1	55000		27261864	validated
mirtarbase	hsa-miR-106b-5p	LAMTOR1	55004	ENSG00000149357	24398324	validated
mirtarbase	hsa-miR-106b-5p	RMND1	55005	ENSG00000155906	23706177	validated
mirtarbase	hsa-miR-15b-5p	STX17	55014	ENSG00000136874	22473208	validated
mirtarbase	hsa-miR-21-5p	PRPF39	55015	ENSG00000185246	20371350	validated
mirtarbase	hsa-miR-106b-5p	C14orf119	55017	ENSG00000179933	23313552	validated
mirtarbase	hsa-miR-106b-5p	ANKRD40CL	55018	ENSG00000167117	17242205	validated
mirtarbase	hsa-miR-21-5p	PHIP	55023	ENSG00000146247	18591254	validated
mirtarbase	hsa-miR-181a-5p	FBXO34	55030	ENSG00000178974	17612493	validated
mirtarbase	hsa-miR-21-5p	USP47	55031	ENSG00000170242	18591254	validated

mirtarbase	hsa-miR-181a-5p	FKBP14	55033	ENSG00000106080	23446348	validated
mirtarbase	hsa-miR-144-3p	FKBP14	55033	ENSG00000106080	23446348	validated
mirtarbase	hsa-miR-106b-5p	FKBP14	55033	ENSG00000106080	23313552	validated
mirtarbase	hsa-miR-582-3p	PTCD3	55037	ENSG00000132300	27418678	validated
mirtarbase	hsa-miR-192-5p	CDCA4	55038	ENSG00000170779	19074876	validated
mirtarbase	hsa-miR-15b-5p	CDCA4	55038	ENSG00000170779	22473208	validated
mirtarbase	hsa-miR-301a-3p	CDCA4	55038	ENSG00000170779	19536157	validated
mirtarbase	hsa-miR-15b-5p	PLEKHB2	55041	ENSG00000115762	26701625	validated
mirtarbase	hsa-miR-106b-5p	ATG16L1	55054	ENSG00000085978	24036151	validated
mirtarbase	hsa-miR-106b-5p	C9orf40	55071	ENSG00000135045	17242205	validated
mirtarbase	hsa-miR-106b-5p	OXR1	55074	ENSG00000164830	22473208	validated
mirtarbase	hsa-miR-144-3p	OXR1	55074	ENSG00000164830	23592263	validated
mirtarbase	hsa-miR-192-5p	UACA	55075	ENSG00000137831	19074876	validated
mirtarbase	hsa-miR-15b-5p	UACA	55075	ENSG00000137831	23622248	validated
mirtarbase	hsa-miR-181a-5p	TMEM45A	55076	ENSG00000181458	17612493	validated
mirtarbase	hsa-miR-192-5p	RADX	55086	ENSG00000147231	19074876	validated
mirtarbase	hsa-miR-21-5p	MED9	55090	ENSG00000141026	22473208	validated
mirtarbase	hsa-miR-106b-5p	ATG2B	55102	ENSG00000066739	22473208	validated
mirtarbase	hsa-miR-505-3p	ATG2B	55102	ENSG00000066739	21572407	validated
mirtarbase	hsa-miR-181a-5p	ATG2B	55102	ENSG00000066739	22473208	validated
mirtarbase	hsa-miR-21-5p	RALGPS2	55103	ENSG00000116191	18591254	validated
mirtarbase	hsa-miR-21-5p	AGGF1	55109	ENSG00000164252	18591254	validated
mirtarbase	hsa-miR-301a-3p	AKIRIN2	55122	ENSG00000135334	21572407	validated
mirtarbase	hsa-miR-106b-5p	PIWIL2	55124	ENSG00000197181	24906430	validated
mirtarbase	hsa-miR-301a-3p	CEP192	55125	ENSG00000101639	23622248	validated
mirtarbase	hsa-miR-192-5p	TRIM68	55128	ENSG00000167333	19074876	validated
mirtarbase	hsa-miR-582-3p	RBM28	55131	ENSG00000106344	23824327	validated
mirtarbase	hsa-miR-505-3p	RBM28	55131	ENSG00000106344	23313552	validated
mirtarbase	hsa-miR-192-5p	LARP1B	55132	ENSG00000138709	19074876	validated
mirtarbase	hsa-miR-21-5p	FIGN	55137	ENSG00000182263	18591254	validated
mirtarbase	hsa-miR-181a-5p	LRRC8D	55144	ENSG00000171492	22473208	validated
mirtarbase	hsa-miR-301a-3p	RBM23	55147	ENSG00000100461	23824327	validated
mirtarbase	hsa-miR-222-3p	UBR7	55148	ENSG00000278787	23622248	validated
mirtarbase	hsa-miR-106b-5p	MTPAP	55149	ENSG00000107951	22012620	validated
mirtarbase	hsa-miR-222-3p	DARS2	55157	ENSG00000117593	23622248	validated
mirtarbase	hsa-miR-192-5p	RFWD3	55159	ENSG00000168411	19074876	validated
mirtarbase	hsa-miR-18a-5p	PNPO	55163	ENSG00000108439	23622248	validated
mirtarbase	hsa-miR-15b-5p	PNPO	55163	ENSG00000108439	22473208	validated
mirtarbase	hsa-miR-192-5p	CEP55	55165	ENSG00000138180	19074876	validated
mirtarbase	hsa-miR-301a-3p	CEP55	55165	ENSG00000138180	21572407	validated
mirtarbase	hsa-miR-15b-5p	CEP55	55165	ENSG00000138180	24398324	validated
mirtarbase	hsa-miR-106b-5p	CENPQ	55166	ENSG00000031691	23446348	validated
mirtarbase	hsa-miR-21-5p	CENPQ	55166	ENSG00000031691	22473208	validated
mirtarbase	hsa-miR-15b-5p	TBCCD1	55171	ENSG00000113838	22473208	validated
mirtarbase	hsa-miR-21-5p	MRPS10	55173	ENSG00000048544	18591254	validated
mirtarbase	hsa-miR-106b-5p	MRPS10	55173	ENSG00000048544	27418678	validated
mirtarbase	hsa-miR-192-5p	FAIM	55179	ENSG00000158234	19074876	validated
mirtarbase	hsa-miR-15b-5p	RIF1	55183	ENSG00000080345	20371350	validated
mirtarbase	hsa-miR-222-3p	SLC25A36	55186	ENSG00000114120	23622248	validated
mirtarbase	hsa-miR-192-5p	RIC8B	55188	ENSG00000111785	19074876	validated
mirtarbase	hsa-miR-21-5p	PBRM1	55193	ENSG00000163939	18591254	validated
mirtarbase	hsa-miR-181a-5p	PBRM1	55193	ENSG00000163939	22473208	validated
mirtarbase	hsa-miR-106b-5p	CCDC198	55195	ENSG00000100557	23313552	validated
mirtarbase	hsa-miR-21-5p	RESF1	55196	ENSG00000174718	18591254	validated
mirtarbase	hsa-miR-181a-5p	RESF1	55196	ENSG00000174718	22473208	validated
mirtarbase	hsa-miR-106b-5p	RESF1	55196	ENSG00000174718	23446348	validated
mirtarbase	hsa-miR-505-3p	MAP1S	55201	ENSG00000130479	23622248	validated
mirtarbase	hsa-miR-21-5p	ZNF532	55205	ENSG00000074657	18591254	validated
mirtarbase	hsa-miR-106b-5p	ZNF532	55205	ENSG00000074657	22012620	validated
mirtarbase	hsa-miR-15b-5p	SBNO1	55206	ENSG00000139697	23446348	validated
mirtarbase	hsa-miR-212-3p	SETD5	55209	ENSG00000168137	26701625	validated
mirtarbase	hsa-miR-192-5p	BBS7	55212	ENSG00000138686	19074876	validated
mirtarbase	hsa-miR-192-5p	FANCI	55215	ENSG00000140525	19074876	validated
mirtarbase	hsa-miR-21-5p	FANCI	55215	ENSG00000140525	18591254	validated
mirtarbase	hsa-miR-505-3p	FANCI	55215	ENSG00000140525	23622248	validated
mirtarbase	hsa-miR-222-3p	NKAPD1	55216	ENSG00000150776	23622248	validated
mirtarbase	hsa-miR-301a-3p	NKAPD1	55216	ENSG00000150776	23592263	validated
mirtarbase	hsa-miR-144-3p	LRRC1	55227	ENSG00000137269	20371350	validated
mirtarbase	hsa-miR-181a-5p	MOB1A	55233	ENSG00000114978	20371350	validated
mirtarbase	hsa-miR-222-3p	OGFOD1	55239	ENSG00000087263	23622248	validated
mirtarbase	hsa-miR-326	SLC47A1	55244	ENSG00000142494	23592263	validated

mirtarbase	hsa-miR-192-5p	NEIL3	55247	ENSG00000109674	19074876	validated
mirtarbase	hsa-miR-222-3p	YY1AP1	55249	ENSG00000163374	23622248	validated
mirtarbase	hsa-miR-326	ASXL2	55252	ENSG00000143970	23622248	validated
mirtarbase	hsa-miR-106b-5p	MAP11	55262	ENSG00000146826	22473208	validated
mirtarbase	hsa-miR-192-5p	NUDT15	55270	ENSG00000136159	19074876	validated
mirtarbase	hsa-miR-15b-5p	TMEM100	55273	ENSG00000166292	23446348	validated
mirtarbase	hsa-miR-106b-5p	TMEM100	55273	ENSG00000166292	21572407	validated
mirtarbase	hsa-miR-192-5p	PHF10	55274	ENSG00000130024	19074876	validated
mirtarbase	hsa-miR-106b-5p	VPS53	55275	ENSG00000141252	23313552	validated
mirtarbase	hsa-miR-106b-5p	CWF19L1	55280	ENSG00000095485	23622248	validated
mirtarbase	hsa-miR-192-5p	UBE2W	55284	ENSG00000104343	19074876	validated
mirtarbase	hsa-miR-106b-5p	RBM41	55285	ENSG00000089682	23313552	validated
mirtarbase	hsa-miR-106b-5p	PPP6R3	55291	ENSG00000110075	21572407	validated
mirtarbase	hsa-miR-301a-3p	PPP6R3	55291	ENSG00000110075	21572407	validated
mirtarbase	hsa-miR-15b-5p	PPP6R3	55291	ENSG00000110075	26701625	validated
mirtarbase	hsa-miR-144-3p	FBXW7	55294	ENSG00000109670	26701625	validated
mirtarbase	hsa-miR-15b-5p	PI4K2B	55300	ENSG00000038210	23446348	validated
mirtarbase	hsa-miR-106b-5p	OLAH	55301	ENSG00000152463	27418678	validated
mirtarbase	hsa-miR-21-5p	SPTLC3	55304	ENSG00000172296	18591254	validated
mirtarbase	hsa-miR-181a-5p	SPTLC3	55304	ENSG00000172296	23824327	validated
mirtarbase	hsa-miR-106b-5p	RFK	55312	ENSG00000135002	23313552	validated
mirtarbase	hsa-miR-15b-5p	RFK	55312	ENSG00000135002	27292025	validated
mirtarbase	hsa-miR-15b-5p	AP5S1	55317	ENSG00000125843	23622248	validated
mirtarbase	hsa-miR-192-5p	MIS18BP1	55320	ENSG00000129534	19074876	validated
mirtarbase	hsa-miR-192-5p	AGPAT5	55326	ENSG00000155189	19074876	validated
mirtarbase	hsa-miR-15b-5p	AGPAT5	55326	ENSG00000155189	23622248	validated
mirtarbase	hsa-miR-21-5p	LIN7C	55327	ENSG00000148943	18591254	validated
mirtarbase	hsa-miR-18a-5p	RNLS	55328	ENSG00000184719	23592263	validated
mirtarbase	hsa-miR-192-5p	MNS1	55329	ENSG00000138587	19074876	validated
mirtarbase	hsa-miR-181a-5p	DRAM1	55332	ENSG00000136048	22473208	validated
mirtarbase	hsa-miR-106b-5p	SYNJ2BP	55333	ENSG00000213463	26701625	validated
mirtarbase	hsa-miR-192-5p	SLC39A9	55334	ENSG00000029364	19074876	validated
mirtarbase	hsa-miR-15b-5p	SLC39A9	55334	ENSG00000029364	23592263	validated
mirtarbase	hsa-miR-106b-5p	WDR33	55339	ENSG00000136709	23622248	validated
mirtarbase	hsa-miR-21-5p	STRBP	55342	ENSG00000165209	18591254	validated
mirtarbase	hsa-miR-192-5p	CHDH	55349	ENSG00000016391	19074876	validated
mirtarbase	hsa-miR-181a-5p	LAPTM4B	55353	ENSG00000104341	19536157	validated
mirtarbase	hsa-miR-192-5p	HJURP	55355	ENSG00000123485	19074876	validated
mirtarbase	hsa-miR-15b-5p	TMEM63B	55362	ENSG00000137216	23622248	validated
mirtarbase	hsa-miR-106b-5p	LRRC59	55379	ENSG00000108829	23622248	validated
mirtarbase	hsa-miR-181a-5p	MEG3	55384		26253106	validated
mirtarbase	hsa-miR-192-5p	MCM10	55388	ENSG00000065328	19074876	validated
mirtarbase	hsa-miR-181a-5p	YOD1	55432	ENSG00000180667	20371350	validated
mirtarbase	hsa-miR-21-5p	YOD1	55432	ENSG00000180667	20371350	validated
mirtarbase	hsa-miR-106b-5p	YOD1	55432	ENSG00000180667	22473208	validated
mirtarbase	hsa-miR-144-3p	YOD1	55432	ENSG00000180667	23446348	validated
mirtarbase	hsa-miR-21-5p	AP1AR	55435	ENSG00000138660	22473208	validated
mirtarbase	hsa-miR-15b-5p	STRADB	55437	ENSG00000082146	20371350	validated
mirtarbase	hsa-miR-192-5p	CSGALNACT2	55454	ENSG00000169826	19074876	validated
mirtarbase	hsa-miR-21-5p	ETNK1	55500	ENSG00000139163	18591254	validated
mirtarbase	hsa-miR-15b-5p	ETNK1	55500	ENSG00000139163	23622248	validated
mirtarbase	hsa-miR-192-5p	CHST12	55501	ENSG00000136213	19074876	validated
mirtarbase	hsa-miR-192-5p	HES6	55502	ENSG00000144485	19074876	validated
mirtarbase	hsa-miR-301a-3p	SLC35E3	55508	ENSG00000175782	23824327	validated
mirtarbase	hsa-miR-106b-5p	FEM1A	55527	ENSG00000141965	23824327	validated
mirtarbase	hsa-miR-144-3p	MAML3	55534	ENSG00000196782	21572407	validated
mirtarbase	hsa-miR-505-3p	MAML3	55534	ENSG00000196782	28735896	validated
mirtarbase	hsa-miR-192-5p	IL17RB	55540	ENSG00000056736	19074876	validated
mirtarbase	hsa-miR-18a-5p	RBM38	55544	ENSG00000132819	21572407	validated
mirtarbase	hsa-miR-192-5p	ZNF823	55552	ENSG00000197933	19074876	validated
mirtarbase	hsa-miR-192-5p	ENOSF1	55556	ENSG00000132199	16822819	validated
mirtarbase	hsa-miR-15b-5p	CDV3	55573	ENSG00000091527	22473208	validated
mirtarbase	hsa-miR-106b-5p	NAGK	55577	ENSG00000124357	23446348	validated
mirtarbase	hsa-miR-15b-5p	UBE2Q1	55585	ENSG00000160714	22473208	validated
mirtarbase	hsa-miR-192-5p	BMP2K	55589	ENSG00000138756	19074876	validated
mirtarbase	hsa-miR-505-3p	OTUD5	55593	ENSG00000068308	23622248	validated
mirtarbase	hsa-miR-192-5p	RNPC3	55599	ENSG00000185946	19074876	validated
mirtarbase	hsa-miR-21-5p	TENT5A	55603	ENSG00000112773	18591254	validated
mirtarbase	hsa-miR-181a-5p	PPP1R9A	55607	ENSG00000158528	23622248	validated
mirtarbase	hsa-miR-106b-5p	ZNF280C	55609	ENSG00000056277	22473208	validated
mirtarbase	hsa-miR-106b-5p	VPS50	55610	ENSG00000004766	22100165	validated

mirtarbase	hsa-miR-15b-5p	OTUB1	55611	ENSG00000167770	22473208	validated
mirtarbase	hsa-miR-192-5p	FERMT1	55612	ENSG00000101311	19074876	validated
mirtarbase	hsa-miR-15b-5p	TASP1	55617	ENSG00000089123	22473208	validated
mirtarbase	hsa-miR-21-5p	DOCK10	55619	ENSG00000135905	18591254	validated
mirtarbase	hsa-miR-222-3p	POMGNT1	55624	ENSG00000085998	23622248	validated
mirtarbase	hsa-miR-181a-5p	PNRC2	55629	ENSG00000189266	22473208	validated
mirtarbase	hsa-miR-15b-5p	PNRC2	55629	ENSG00000189266	21572407	validated
mirtarbase	hsa-miR-222-3p	PNRC2	55629	ENSG00000189266	20371350	validated
mirtarbase	hsa-miR-192-5p	G2E3	55632	ENSG00000092140	19074876	validated
mirtarbase	hsa-miR-582-5p	G2E3	55632	ENSG00000092140	23592263	validated
mirtarbase	hsa-miR-192-5p	TBC1D22B	55633	ENSG00000065491	19074876	validated
mirtarbase	hsa-miR-181a-5p	KRBOX4	55634	ENSG00000147121	21572407	validated
mirtarbase	hsa-miR-192-5p	DEPDC1	55635	ENSG00000024526	19074876	validated
mirtarbase	hsa-miR-301a-3p	DEPDC1	55635	ENSG00000024526	21572407	validated
mirtarbase	hsa-miR-192-5p	CHD7	55636	ENSG00000171316	19074876	validated
mirtarbase	hsa-miR-192-5p	SLC48A1	55652	ENSG00000211584	19074876	validated
mirtarbase	hsa-miR-106b-5p	BCAS4	55653	ENSG00000124243	23313552	validated
mirtarbase	hsa-miR-106b-5p	TMEM127	55654	ENSG00000135956	22473208	validated
mirtarbase	hsa-miR-181a-5p	DDX27	55661	ENSG00000124228	23622248	validated
mirtarbase	hsa-miR-106b-5p	HIF1AN	55662	ENSG00000166135	22473208	validated
mirtarbase	hsa-miR-106b-5p	ZNF446	55663	ENSG00000083838	23313552	validated
mirtarbase	hsa-miR-106b-5p	CDC37L1	55664	ENSG00000106993	17242205	validated
mirtarbase	hsa-miR-15b-5p	CDC37L1	55664	ENSG00000106993	22473208	validated
mirtarbase	hsa-miR-106b-5p	NPLOC4	55666	ENSG00000182446	23622248	validated
mirtarbase	hsa-miR-106b-5p	MFN1	55669	ENSG00000171109	23446348	validated
mirtarbase	hsa-miR-106b-5p	RUFY2	55680	ENSG00000204130	21572407	validated
mirtarbase	hsa-miR-21-5p	KANSL3	55683	ENSG00000114982	27292025	validated
mirtarbase	hsa-miR-222-3p	RABL6	55684	ENSG00000196642	23622248	validated
mirtarbase	hsa-miR-301a-3p	MREG	55686	ENSG00000118242	27292025	validated
mirtarbase	hsa-miR-296-5p	TRMU	55687	ENSG00000100416	26701625	validated
mirtarbase	hsa-miR-192-5p	KDM4D	55693	ENSG00000186280	19074876	validated
mirtarbase	hsa-miR-222-3p	NSUN5	55695	ENSG00000130305	23622248	validated
mirtarbase	hsa-miR-192-5p	RADIL	55698	ENSG00000157927	19074876	validated
mirtarbase	hsa-miR-18a-5p	MAP7D1	55700	ENSG00000116871	20371350	validated
mirtarbase	hsa-miR-192-5p	ARHGEF40	55701	ENSG00000165801	19074876	validated
mirtarbase	hsa-miR-21-5p	POLR3B	55703	ENSG00000013503	18591254	validated
mirtarbase	hsa-miR-222-3p	NDC1	55706	ENSG00000058804	23622248	validated
mirtarbase	hsa-miR-192-5p	SLF2	55719	ENSG00000119906	19074876	validated
mirtarbase	hsa-miR-106b-5p	TSR1	55720	ENSG00000167721	22100165	validated
mirtarbase	hsa-miR-18a-5p	TSR1	55720	ENSG00000167721	23592263	validated
mirtarbase	hsa-miR-192-5p	IQCC	55721	ENSG00000160051	19074876	validated
mirtarbase	hsa-miR-106b-5p	CEP72	55722	ENSG00000112877	23446348	validated
mirtarbase	hsa-miR-21-5p	BTBD7	55727	ENSG00000277222	18591254	validated
mirtarbase	hsa-miR-106b-5p	BTBD7	55727	ENSG00000277222	22473208	validated
mirtarbase	hsa-miR-192-5p	N4BP2	55728	ENSG00000078177	19074876	validated
mirtarbase	hsa-miR-144-3p	ATF7IP	55729	ENSG00000171681	20371350	validated
mirtarbase	hsa-miR-301a-3p	ATF7IP	55729	ENSG00000171681	23824327	validated
mirtarbase	hsa-miR-21-5p	ATF7IP	55729	ENSG00000171681	19536157	validated
mirtarbase	hsa-miR-106b-5p	FAM222B	55731	ENSG00000173065	23622248	validated
mirtarbase	hsa-miR-181a-5p	FAM222B	55731	ENSG00000173065	23622248	validated
mirtarbase	hsa-miR-192-5p	C1orf112	55732	ENSG00000000460	19074876	validated
mirtarbase	hsa-miR-505-3p	ZFP64	55734	ENSG00000020256	23313552	validated
mirtarbase	hsa-miR-15b-5p	VPS35	55737	ENSG00000069329	23622248	validated
mirtarbase	hsa-miR-181a-5p	ENAH	55740	ENSG00000154380	17612493	validated
mirtarbase	hsa-miR-21-5p	ENAH	55740	ENSG00000154380	18591254	validated
mirtarbase	hsa-miR-181a-5p	AP5M1	55745	ENSG00000053770	23824327	validated
mirtarbase	hsa-miR-582-5p	AGK	55750	ENSG00000262327	22012620	validated
mirtarbase	hsa-miR-222-3p	SEPTIN11	55752	ENSG00000138758	23622248	validated
mirtarbase	hsa-miR-192-5p	TMEM30A	55754	ENSG00000112697	19074876	validated
mirtarbase	hsa-miR-301a-3p	TMEM30A	55754	ENSG00000112697	21572407	validated
mirtarbase	hsa-miR-181a-5p	TMEM30A	55754	ENSG00000112697	22473208	validated
mirtarbase	hsa-miR-192-5p	ZNF701	55762	ENSG00000167562	19074876	validated
mirtarbase	hsa-miR-181a-5p	ZNF83	55769	ENSG00000167766	24398324	validated
mirtarbase	hsa-miR-192-5p	TDP1	55775	ENSG00000042088	19074876	validated
mirtarbase	hsa-miR-192-5p	SAYSD1	55776	ENSG00000112167	19074876	validated
mirtarbase	hsa-miR-106b-5p	CMTR2	55783	ENSG00000180917	22473208	validated
mirtarbase	hsa-miR-181a-5p	ZNF415	55786	ENSG00000170954	20371350	validated
mirtarbase	hsa-miR-106b-5p	LRIF1	55791	ENSG00000121931	23313552	validated
mirtarbase	hsa-miR-15b-5p	LRIF1	55791	ENSG00000121931	22473208	validated
mirtarbase	hsa-miR-192-5p	MBNL3	55796	ENSG00000076770	19074876	validated
mirtarbase	hsa-miR-301a-3p	MBNL3	55796	ENSG00000076770	21572407	validated

mirtarbase	hsa-miR-192-5p	DCP1A	55802	ENSG00000272886	19074876	validated
mirtarbase	hsa-miR-21-5p	DCP1A	55802	ENSG00000272886	18372920	validated
mirtarbase	hsa-miR-106b-5p	FOXJ2	55810	ENSG00000065970	21572407	validated
mirtarbase	hsa-miR-21-5p	PAG1	55824	ENSG00000076641	18591254	validated
mirtarbase	hsa-miR-15b-5p	PAG1	55824	ENSG00000076641	21572407	validated
mirtarbase	hsa-miR-192-5p	CAND1	55832	ENSG00000111530	22100165	validated
mirtarbase	hsa-miR-192-5p	EAF2	55840	ENSG00000145088	19074876	validated
mirtarbase	hsa-miR-192-5p	WWC3	55841	ENSG00000047644	19074876	validated
mirtarbase	hsa-miR-192-5p	PPP2R2D	55844	ENSG00000175470	19074876	validated
mirtarbase	hsa-miR-15b-5p	BEX1	55859	ENSG00000133169	23622248	validated
mirtarbase	hsa-miR-15b-5p	ASH1L	55870	ENSG00000116539	22473208	validated
mirtarbase	hsa-miR-505-3p	CBWD1	55871	ENSG00000172785	24398324	validated
mirtarbase	hsa-miR-106b-5p	UNC45A	55898	ENSG00000140553	23622248	validated
mirtarbase	hsa-miR-181a-5p	KMT2E	55904	ENSG00000005483	22473208	validated
mirtarbase	hsa-miR-301a-3p	ERBIN	55914	ENSG00000112851	22012620	validated
mirtarbase	hsa-miR-192-5p	RCC2	55920	ENSG00000179051	23622248	validated
mirtarbase	hsa-miR-301a-3p	NKRF	55922	ENSG00000186416	21113131	validated
mirtarbase	hsa-miR-192-5p	NKRF	55922	ENSG00000186416	19074876	validated
mirtarbase	hsa-miR-326	NKRF	55922	ENSG00000186416	23622248	validated
mirtarbase	hsa-miR-222-3p	NKRF	55922	ENSG00000186416	23622248	validated
mirtarbase	hsa-miR-144-3p	GNG12	55970	ENSG00000172380	24398324	validated
mirtarbase	hsa-miR-15b-5p	GNG12	55970	ENSG00000172380	20371350	validated
mirtarbase	hsa-miR-192-5p	SLC25A40	55972	ENSG00000075303	19074876	validated
mirtarbase	hsa-miR-106b-5p	UBFD1	56061	ENSG00000103353	22473208	validated
mirtarbase	hsa-miR-181a-5p	PCDHB8	56128	ENSG00000120322	17612493	validated
mirtarbase	hsa-miR-181a-5p	PCDHB6	56130	ENSG00000113211	22942087	validated
mirtarbase	hsa-miR-181a-5p	PCDHAC2	56134	ENSG00000243232	22942087	validated
mirtarbase	hsa-miR-181a-5p	PCDHAC1	56135	ENSG00000248383	22942087	validated
mirtarbase	hsa-miR-181a-5p	PCDHA13	56136	ENSG00000239389	22942087	validated
mirtarbase	hsa-miR-181a-5p	PCDHA12	56137	ENSG00000251664	22942087	validated
mirtarbase	hsa-miR-181a-5p	PCDHA11	56138	ENSG00000249158	22942087	validated
mirtarbase	hsa-miR-181a-5p	PCDHA10	56139	ENSG00000250120	22942087	validated
mirtarbase	hsa-miR-181a-5p	PCDHA8	56140	ENSG00000204962	22942087	validated
mirtarbase	hsa-miR-181a-5p	PCDHA7	56141	ENSG00000204963	22942087	validated
mirtarbase	hsa-miR-181a-5p	PCDHA6	56142	ENSG00000081842	22942087	validated
mirtarbase	hsa-miR-181a-5p	PCDHA5	56143	ENSG00000204965	22942087	validated
mirtarbase	hsa-miR-181a-5p	PCDHA4	56144	ENSG00000204967	22942087	validated
mirtarbase	hsa-miR-181a-5p	PCDHA3	56145	ENSG00000255408	22942087	validated
mirtarbase	hsa-miR-181a-5p	PCDHA2	56146	ENSG00000204969	22942087	validated
mirtarbase	hsa-miR-181a-5p	PCDHA1	56147	ENSG00000204970	22942087	validated
mirtarbase	hsa-miR-106b-5p	ANKH	56172	ENSG00000154122	23592263	validated
mirtarbase	hsa-miR-181a-5p	MOSPD1	56180	ENSG00000101928	22815788	validated
mirtarbase	hsa-miR-15b-5p	MTFR1L	56181	ENSG00000117640	20371350	validated
mirtarbase	hsa-miR-15b-5p	FAM214A	56204	ENSG00000047346	23622248	validated
mirtarbase	hsa-miR-222-3p	FAM214A	56204	ENSG00000047346	22815788	validated
mirtarbase	hsa-miR-296-5p	SUSD2	56241	ENSG00000099994	19536157	validated
mirtarbase	hsa-miR-181a-5p	ZNF253	56242	ENSG00000256771	23313552	validated
mirtarbase	hsa-miR-15b-5p	C21orf62	56245	ENSG00000262938	27292025	validated
mirtarbase	hsa-miR-222-3p	YLPM1	56252	ENSG00000119596	23622248	validated
mirtarbase	hsa-miR-21-5p	TMX4	56255	ENSG00000125827	18591254	validated
mirtarbase	hsa-miR-192-5p	SERTAD4	56256	ENSG00000082497	19074876	validated
mirtarbase	hsa-miR-222-3p	SERTAD4	56256	ENSG00000082497	23622248	validated
mirtarbase	hsa-miR-106b-5p	PARD3	56288	ENSG00000148498	23313552	validated
mirtarbase	hsa-miR-192-5p	CTPS2	56474	ENSG00000047230	19074876	validated
mirtarbase	hsa-miR-144-3p	KCNQ5	56479	ENSG00000185760	19536157	validated
mirtarbase	hsa-miR-15b-5p	CYP26B1	56603	ENSG00000003137	21572407	validated
mirtarbase	hsa-miR-106b-5p	EIF5A2	56648	ENSG00000163577	17242205	validated
mirtarbase	hsa-miR-144-3p	EIF5A2	56648	ENSG00000163577	21572407	validated
mirtarbase	hsa-miR-15b-5p	POLE4	56655	ENSG00000115350	22473208	validated
mirtarbase	hsa-miR-106b-5p	TMEM9B	56674	ENSG00000175348	22100165	validated
mirtarbase	hsa-miR-106b-5p	NRIP3	56675	ENSG00000175352	22100165	validated
mirtarbase	hsa-miR-21-5p	SAR1A	56681	ENSG00000079332	18591254	validated
mirtarbase	hsa-miR-18a-5p	SAR1A	56681	ENSG00000079332	21572407	validated
mirtarbase	hsa-miR-582-5p	CFAP298	56683	ENSG00000159079	27292025	validated
mirtarbase	hsa-miR-21-5p	JPH1	56704	ENSG00000104369	18591254	validated
mirtarbase	hsa-miR-192-5p	EMC7	56851	ENSG00000134153	19074876	validated
mirtarbase	hsa-miR-21-5p	UGGT1	56886	ENSG00000136731	18591254	validated
mirtarbase	hsa-miR-21-5p	TM9SF3	56889	ENSG00000077147	19253296	validated
mirtarbase	hsa-miR-181a-5p	TM9SF3	56889	ENSG00000077147	20371350	validated
mirtarbase	hsa-miR-15b-5p	TM9SF3	56889	ENSG00000077147	23622248	validated
mirtarbase	hsa-miR-18a-5p	UBQLN4	56893	ENSG00000160803	23622248	validated

mirtarbase	hsa-miR-21-5p	BDH2	56898	ENSG00000164039	20048743	validated
mirtarbase	hsa-miR-505-3p	C15orf39	56905	ENSG00000167173	23622248	validated
mirtarbase	hsa-miR-15b-5p	C15orf39	56905	ENSG00000167173	22473208	validated
mirtarbase	hsa-miR-181a-5p	SPIRE1	56907	ENSG00000134278	21572407	validated
mirtarbase	hsa-miR-192-5p	PAK6	56924	ENSG00000137843	19074876	validated
mirtarbase	hsa-miR-106b-5p	PAK6	56924	ENSG00000137843	23824327	validated
mirtarbase	hsa-miR-582-3p	NCLN	56926	ENSG00000125912	26701625	validated
mirtarbase	hsa-miR-18a-5p	FEM1C	56929	ENSG00000145780	20371350	validated
mirtarbase	hsa-miR-106b-5p	FEM1C	56929	ENSG00000145780	22473208	validated
mirtarbase	hsa-miR-192-5p	ARNTL2	56938	ENSG00000029153	19074876	validated
mirtarbase	hsa-miR-106b-5p	EMSY	56946	ENSG00000158636	22473208	validated
mirtarbase	hsa-miR-181a-5p	EMSY	56946	ENSG00000158636	22473208	validated
mirtarbase	hsa-miR-301a-3p	MFF	56947	ENSG00000168958	21572407	validated
mirtarbase	hsa-miR-15b-5p	C5orf15	56951	ENSG00000113583	23622248	validated
mirtarbase	hsa-miR-192-5p	NT5M	56953	ENSG00000205309	19074876	validated
mirtarbase	hsa-miR-222-3p	ATXN7L3	56970	ENSG00000087152	23622248	validated
mirtarbase	hsa-miR-192-5p	STOX2	56977	ENSG00000173320	19074876	validated
mirtarbase	hsa-miR-222-3p	STOX2	56977	ENSG00000173320	23622248	validated
mirtarbase	hsa-miR-505-3p	PRDM10	56980	ENSG00000170325	23313552	validated
mirtarbase	hsa-miR-106b-5p	BBX	56987	ENSG00000114439	23592263	validated
mirtarbase	hsa-miR-15b-5p	CDC42SE2	56990	ENSG00000158985	21572407	validated
mirtarbase	hsa-miR-192-5p	KIF15	56992	ENSG00000163808	19074876	validated
mirtarbase	hsa-miR-192-5p	CHPT1	56994	ENSG00000111666	19074876	validated
mirtarbase	hsa-miR-192-5p	CTNNBIP1	56998	ENSG00000178585	19074876	validated
mirtarbase	hsa-miR-192-5p	CCDC47	57003	ENSG00000108588	19074876	validated
mirtarbase	hsa-miR-106b-5p	CCDC47	57003	ENSG00000108588	23622248	validated
mirtarbase	hsa-miR-222-3p	CCDC47	57003	ENSG00000108588	23622248	validated
mirtarbase	hsa-miR-15b-5p	AKR1B10	57016	ENSG00000198074	23446348	validated
mirtarbase	hsa-miR-18a-5p	CCNL1	57018	ENSG00000163660	16331254	validated
mirtarbase	hsa-miR-222-3p	CIAPIN1	57019	ENSG00000005194	23592263	validated
mirtarbase	hsa-miR-106b-5p	RSRP1	57035	ENSG00000117616	22473208	validated
mirtarbase	hsa-miR-582-5p	TBX20	57057	ENSG00000164532	27292025	validated
mirtarbase	hsa-miR-192-5p	KNL1	57082	ENSG00000137812	19074876	validated
mirtarbase	hsa-miR-21-5p	KNL1	57082	ENSG00000137812	18591254	validated
mirtarbase	hsa-miR-15b-5p	PLSCR4	57088	ENSG00000114698	23622248	validated
mirtarbase	hsa-miR-192-5p	ENTPD7	57089	ENSG00000198018	19074876	validated
mirtarbase	hsa-miR-15b-5p	ENTPD7	57089	ENSG00000198018	22473208	validated
mirtarbase	hsa-miR-106b-5p	ENTPD7	57089	ENSG00000198018	22473208	validated
mirtarbase	hsa-miR-18a-5p	PCNP	57092	ENSG00000081154	20371350	validated
mirtarbase	hsa-miR-296-5p	PCNP	57092	ENSG00000081154	27418678	validated
mirtarbase	hsa-miR-21-5p	PITHD1	57095	ENSG00000057757	23592263	validated
mirtarbase	hsa-miR-212-3p	PARP11	57097	ENSG00000111224	22012620	validated
mirtarbase	hsa-miR-21-5p	RTN4	57142	ENSG00000115310	19253296	validated
mirtarbase	hsa-miR-15b-5p	RTN4	57142	ENSG00000115310	21572407	validated
mirtarbase	hsa-miR-15b-5p	RALGAPB	57148	ENSG00000170471	21572407	validated
mirtarbase	hsa-miR-15b-5p	SMURF1	57154	ENSG00000198742	24435757	validated
mirtarbase	hsa-miR-192-5p	PHTF2	57157	ENSG00000006576	19074876	validated
mirtarbase	hsa-miR-106b-5p	PHTF2	57157	ENSG00000006576	23446348	validated
mirtarbase	hsa-miR-222-3p	PELI2	57161	ENSG00000139946	23622248	validated
mirtarbase	hsa-miR-106b-5p	ZNFX1	57169	ENSG00000124201	22473208	validated
mirtarbase	hsa-miR-106b-5p	KIAA1191	57179	ENSG00000122203	22473208	validated
mirtarbase	hsa-miR-301a-3p	KIAA1191	57179	ENSG00000122203	24398324	validated
mirtarbase	hsa-miR-15b-5p	ACTR3B	57180	ENSG00000133627	23592263	validated
mirtarbase	hsa-miR-582-5p	SLC39A10	57181	ENSG00000196950	23446348	validated
mirtarbase	hsa-miR-301a-3p	ANKRD50	57182	ENSG00000151458	23592263	validated
mirtarbase	hsa-miR-106b-5p	ANKRD50	57182	ENSG00000151458	23592263	validated
mirtarbase	hsa-miR-21-5p	THOC2	57187	ENSG00000125676	18591254	validated
mirtarbase	hsa-miR-106b-5p	DENND11	57189	ENSG00000262599	23446348	validated
mirtarbase	hsa-miR-192-5p	ATP10D	57205	ENSG00000145246	16822819	validated
mirtarbase	hsa-miR-192-5p	ZNF248	57209	ENSG00000198105	19074876	validated
mirtarbase	hsa-miR-192-5p	ADGRG6	57211	ENSG00000112414	19074876	validated
mirtarbase	hsa-miR-106b-5p	PPP4R3B	57223	ENSG00000275052	23622248	validated
mirtarbase	hsa-miR-192-5p	NHSL1	57224	ENSG00000135540	19074876	validated
mirtarbase	hsa-miR-106b-5p	CBX8	57332	ENSG00000141570	23824327	validated
mirtarbase	hsa-miR-192-5p	CLK4	57396	ENSG00000113240	19074876	validated
mirtarbase	hsa-miR-21-5p	RAB22A	57403	ENSG00000124209	18591254	validated
mirtarbase	hsa-miR-106b-5p	RAB22A	57403	ENSG00000124209	22473208	validated
mirtarbase	hsa-miR-301a-3p	CYP20A1	57404	ENSG00000119004	23824327	validated
mirtarbase	hsa-miR-212-3p	CYP20A1	57404	ENSG00000119004	23824327	validated
mirtarbase	hsa-miR-192-5p	AS3MT	57412	ENSG00000214435	23824327	validated
mirtarbase	hsa-miR-18a-5p	AS3MT	57412	ENSG00000214435	23824327	validated

mirtarbase	hsa-miR-15b-5p	REXO1	57455	ENSG00000079313	22473208	validated
mirtarbase	hsa-miR-192-5p	KIAA1143	57456	ENSG00000163807	19074876	validated
mirtarbase	hsa-miR-181a-5p	GATAD2B	57459	ENSG00000261992	20371350	validated
mirtarbase	hsa-miR-222-3p	PPM1H	57460	ENSG00000111110	23622248	validated
mirtarbase	hsa-miR-106b-5p	ISY1	57461	ENSG00000240682	27292025	validated
mirtarbase	hsa-miR-106b-5p	CNOT6	57472	ENSG00000113300	23622248	validated
mirtarbase	hsa-miR-582-5p	ZNF490	57474	ENSG00000188033	21572407	validated
mirtarbase	hsa-miR-15b-5p	USP31	57478	ENSG00000103404	22473208	validated
mirtarbase	hsa-miR-144-3p	NLN	57486	ENSG00000123213	23592263	validated
mirtarbase	hsa-miR-21-5p	ESYT2	57488	ENSG00000117868	18591254	validated
mirtarbase	hsa-miR-15b-5p	ODF2L	57489	ENSG00000122417	24398324	validated
mirtarbase	hsa-miR-18a-5p	AHRR	57491	ENSG00000286169	23622248	validated
mirtarbase	hsa-miR-144-3p	ARID1B	57492	ENSG00000049618	23446348	validated
mirtarbase	hsa-miR-192-5p	MRTFB	57496	ENSG00000186260	19074876	validated
mirtarbase	hsa-miR-192-5p	KIDINS220	57498	ENSG00000134313	16822819	validated
mirtarbase	hsa-miR-106b-5p	MAVS	57506	ENSG00000088888	21572407	validated
mirtarbase	hsa-miR-301a-3p	MAVS	57506	ENSG00000088888	21572407	validated
mirtarbase	hsa-miR-181a-5p	MTUS1	57509	ENSG00000129422	22473208	validated
mirtarbase	hsa-miR-222-3p	CASKIN2	57513	ENSG00000177303	23622248	validated
mirtarbase	hsa-miR-106b-5p	SERINC1	57515	ENSG00000111897	23313552	validated
mirtarbase	hsa-miR-181a-5p	HECW2	57520	ENSG00000138411	22473208	validated
mirtarbase	hsa-miR-181a-5p	SRGAP1	57522	ENSG00000196935	22815788	validated
mirtarbase	hsa-miR-15b-5p	CASKIN1	57524	ENSG00000167971	23592263	validated
mirtarbase	hsa-miR-21-5p	NUFIP2	57532	ENSG00000108256	20371350	validated
mirtarbase	hsa-miR-106b-5p	NUFIP2	57532	ENSG00000108256	23446348	validated
mirtarbase	hsa-miR-15b-5p	NUFIP2	57532	ENSG00000108256	22473208	validated
mirtarbase	hsa-miR-582-5p	NUFIP2	57532	ENSG00000108256	20371350	validated
mirtarbase	hsa-miR-15b-5p	TBC1D14	57533	ENSG00000132405	22473208	validated
mirtarbase	hsa-miR-192-5p	MIB1	57534	ENSG00000101752	19074876	validated
mirtarbase	hsa-miR-21-5p	MIB1	57534	ENSG00000101752	18591254	validated
mirtarbase	hsa-miR-15b-5p	MIB1	57534	ENSG00000101752	22473208	validated
mirtarbase	hsa-miR-106b-5p	SORCS2	57537	ENSG00000184985	22100165	validated
mirtarbase	hsa-miR-326	SORCS2	57537	ENSG00000184985	22012620	validated
mirtarbase	hsa-miR-181a-5p	KLHL42	57542	ENSG00000087448	20371350	validated
mirtarbase	hsa-miR-192-5p	KLHL42	57542	ENSG00000087448	19074876	validated
mirtarbase	hsa-miR-21-5p	KLHL42	57542	ENSG00000087448	18591254	validated
mirtarbase	hsa-miR-301a-3p	PDP2	57546	ENSG00000172840	23824327	validated
mirtarbase	hsa-miR-144-3p	TAOK1	57551	ENSG00000160551	23592263	validated
mirtarbase	hsa-miR-15b-5p	TAOK1	57551	ENSG00000160551	21572407	validated
mirtarbase	hsa-miR-18a-5p	TAOK1	57551	ENSG00000160551	23592263	validated
mirtarbase	hsa-miR-301a-3p	TAOK1	57551	ENSG00000160551	23592263	validated
mirtarbase	hsa-miR-192-5p	NCEH1	57552	ENSG00000144959	19074876	validated
mirtarbase	hsa-miR-192-5p	STAMBPL1	57559	ENSG00000138134	19074876	validated
mirtarbase	hsa-miR-181a-5p	ARRDC3	57561	ENSG00000113369	22473208	validated
mirtarbase	hsa-miR-192-5p	KLHL8	57563	ENSG00000145332	19074876	validated
mirtarbase	hsa-miR-222-3p	KLHL8	57563	ENSG00000145332	26701625	validated
mirtarbase	hsa-miR-222-3p	TRMT5	57570	ENSG00000126814	23622248	validated
mirtarbase	hsa-miR-106b-5p	MARCHF4	57574	ENSG00000144583	22100165	validated
mirtarbase	hsa-miR-21-5p	ARHGAP21	57584	ENSG00000107863	18591254	validated
mirtarbase	hsa-miR-582-5p	USP36	57602	ENSG00000055483	23592263	validated
mirtarbase	hsa-miR-15b-5p	TRMT9B	57604	ENSG00000250305	20371350	validated
mirtarbase	hsa-miR-15b-5p	KIAA1456	57604	ENSG00000284872	20371350	validated
mirtarbase	hsa-miR-582-5p	TRMT9B	57604	ENSG00000250305	26701625	validated
mirtarbase	hsa-miR-582-5p	KIAA1456	57604	ENSG00000284872	26701625	validated
mirtarbase	hsa-miR-21-5p	SLAIN2	57606	ENSG00000109171	18591254	validated
mirtarbase	hsa-miR-106b-5p	SLAIN2	57606	ENSG00000109171	22473208	validated
mirtarbase	hsa-miR-181a-5p	JCAD	57608	ENSG00000165757	20371350	validated
mirtarbase	hsa-miR-582-5p	JCAD	57608	ENSG00000165757	21572407	validated
mirtarbase	hsa-miR-144-3p	JCAD	57608	ENSG00000165757	21572407	validated
mirtarbase	hsa-miR-15b-5p	RANBP10	57610	ENSG00000141084	23622248	validated
mirtarbase	hsa-miR-222-3p	RANBP10	57610	ENSG00000141084	23622248	validated
mirtarbase	hsa-miR-21-5p	TSHZ3	57616	ENSG00000121297	18591254	validated
mirtarbase	hsa-miR-18a-5p	VPS18	57617	ENSG00000104142	23824327	validated
mirtarbase	hsa-miR-18a-5p	POGK	57645	ENSG00000143157	20371350	validated
mirtarbase	hsa-miR-181a-5p	USP28	57646	ENSG00000048028	17612493	validated
mirtarbase	hsa-miR-106b-5p	USP28	57646	ENSG00000048028	22473208	validated
mirtarbase	hsa-miR-15b-5p	DHX37	57647	ENSG00000150990	23622248	validated
mirtarbase	hsa-miR-301a-3p	PHF12	57649	ENSG00000109118	23446348	validated
mirtarbase	hsa-miR-192-5p	PHF12	57649	ENSG00000109118	19536157	validated
mirtarbase	hsa-miR-15b-5p	UVSSA	57654	ENSG00000163945	23622248	validated
mirtarbase	hsa-miR-106b-5p	GRAMD1A	57655	ENSG00000089351	22473208	validated

mirtarbase	hsa-miR-192-5p	HCN3	57657	ENSG00000263324	19074876	validated
mirtarbase	hsa-miR-106b-5p	ZBTB4	57659	ENSG00000174282	22752225	validated
mirtarbase	hsa-miR-301a-3p	ZBTB4	57659	ENSG00000174282	24398324	validated
mirtarbase	hsa-miR-181a-5p	ZBTB4	57659	ENSG00000174282	22473208	validated
mirtarbase	hsa-miR-222-3p	PHRF1	57661	ENSG00000274780	23622248	validated
mirtarbase	hsa-miR-582-5p	RNF213	57674	ENSG00000173821	23824327	validated
mirtarbase	hsa-miR-21-5p	GPAM	57678	ENSG00000119927	18591254	validated
mirtarbase	hsa-miR-106b-5p	GPAM	57678	ENSG00000119927	22473208	validated
mirtarbase	hsa-miR-18a-5p	ZSWIM6	57688	ENSG00000130449	23622248	validated
mirtarbase	hsa-miR-212-3p	TNRC6C	57690	ENSG00000078687	19536157	validated
mirtarbase	hsa-miR-505-3p	TNRC6C	57690	ENSG00000078687	23824327	validated
mirtarbase	hsa-miR-301a-3p	ZNF317	57693	ENSG00000130803	23446348	validated
mirtarbase	hsa-miR-21-5p	DDX55	57696	ENSG00000111364	23446348	validated
mirtarbase	hsa-miR-192-5p	FANCM	57697	ENSG00000187790	19074876	validated
mirtarbase	hsa-miR-106b-5p	SHTN1	57698	ENSG00000187164	17242205	validated
mirtarbase	hsa-miR-505-3p	FAM160B1	57700	ENSG00000151553	21572407	validated
mirtarbase	hsa-miR-106b-5p	FAM160B1	57700	ENSG00000151553	23592263	validated
mirtarbase	hsa-miR-582-3p	FAM160B1	57700	ENSG00000151553	21572407	validated
mirtarbase	hsa-miR-222-3p	NCKAP5L	57701	ENSG00000167566	23622248	validated
mirtarbase	hsa-miR-192-5p	DENND1A	57706	ENSG00000119522	19074876	validated
mirtarbase	hsa-miR-181a-5p	MEAK7	57707	ENSG00000140950	23824327	validated
mirtarbase	hsa-miR-301a-3p	ZNF529	57711	ENSG00000186020	23313552	validated
mirtarbase	hsa-miR-222-3p	GPR107	57720	ENSG00000148358	26701625	validated
mirtarbase	hsa-miR-192-5p	IGDCC4	57722	ENSG00000103742	19074876	validated
mirtarbase	hsa-miR-326	EPG5	57724	ENSG00000152223	23313552	validated
mirtarbase	hsa-miR-192-5p	ZFYVE28	57732	ENSG00000159733	19074876	validated
mirtarbase	hsa-miR-192-5p	TRIB3	57761	ENSG00000101255	19074876	validated
mirtarbase	hsa-miR-106b-5p	SUGP1	57794	ENSG00000105705	23313552	validated
mirtarbase	hsa-miR-192-5p	GATAD1	57798	ENSG00000157259	19074876	validated
mirtarbase	hsa-miR-106b-5p	GATAD1	57798	ENSG00000157259	23824327	validated
mirtarbase	hsa-miR-106b-5p	RAP2C	57826	ENSG00000123728	21572407	validated
mirtarbase	hsa-miR-582-5p	RAP2C	57826	ENSG00000123728	21572407	validated
mirtarbase	hsa-miR-144-3p	RAP2C	57826	ENSG00000123728	21572407	validated
mirtarbase	hsa-miR-15b-5p	RAP2C	57826	ENSG00000123728	22473208	validated
mirtarbase	hsa-miR-192-5p	PTBP2	58155	ENSG00000117569	19074876	validated
mirtarbase	hsa-miR-15b-5p	SRPRB	58477	ENSG00000144867	22473208	validated
mirtarbase	hsa-miR-15b-5p	TRAPPC1	58485	ENSG00000170043	23622248	validated
mirtarbase	hsa-miR-18a-5p	TRAPPC1	58485	ENSG00000170043	23622248	validated
mirtarbase	hsa-miR-192-5p	CREBZF	58487	ENSG00000137504	19074876	validated
mirtarbase	hsa-miR-106b-5p	ABHD17C	58489	ENSG00000136379	23622248	validated
mirtarbase	hsa-miR-15b-5p	RPRD1B	58490	ENSG00000101413	26701625	validated
mirtarbase	hsa-miR-192-5p	INIP	58493	ENSG00000148153	19074876	validated
mirtarbase	hsa-miR-106b-5p	LY6G5B	58496	ENSG00000239497	23313552	validated
mirtarbase	hsa-miR-15b-5p	KMT2C	58508	ENSG00000055609	23622248	validated
mirtarbase	hsa-miR-15b-5p	DLGAP3	58512	ENSG00000116544	20371350	validated
mirtarbase	hsa-miR-106b-5p	EPS15L1	58513	ENSG00000127527	23446348	validated
mirtarbase	hsa-miR-181a-5p	RBM25	58517	ENSG00000119707	23824327	validated
mirtarbase	hsa-miR-301a-3p	MID1IP1	58526	ENSG00000165175	24398324	validated
mirtarbase	hsa-miR-106b-5p	RRAGD	58528	ENSG00000025039	23446348	validated
mirtarbase	hsa-miR-301a-3p	RRAGD	58528	ENSG00000025039	23446348	validated
mirtarbase	hsa-miR-106b-5p	ENPP5	59084	ENSG00000112796	21572407	validated
mirtarbase	hsa-miR-301a-3p	ENPP5	59084	ENSG00000112796	21572407	validated
mirtarbase	hsa-miR-18a-5p	UBL5	59286	ENSG00000198258	20371350	validated
mirtarbase	hsa-miR-21-5p	PLEKHA1	59338	ENSG00000107679	18591254	validated
mirtarbase	hsa-miR-15b-5p	PLEKHA1	59338	ENSG00000107679	21572407	validated
mirtarbase	hsa-miR-21-5p	PLEKHA2	59339	ENSG00000169499	22100165	validated
mirtarbase	hsa-miR-296-5p	PLEKHA2	59339	ENSG00000169499	23824327	validated
mirtarbase	hsa-miR-21-5p	GNB4	59345	ENSG00000114450	20371350	validated
mirtarbase	hsa-miR-181a-5p	ZNF350	59348	ENSG00000256683	20371350	validated
mirtarbase	hsa-miR-582-5p	ZNF350	59348	ENSG00000256683	20371350	validated
mirtarbase	hsa-miR-144-3p	ZNF350	59348	ENSG00000256683	20371350	validated
mirtarbase	hsa-miR-181a-5p	TGIF2	60436	ENSG00000118707	20371350	validated
mirtarbase	hsa-miR-192-5p	ELOVL5	60481	ENSG00000012660	19074876	validated
mirtarbase	hsa-miR-192-5p	AASDHPPT	60496	ENSG00000149313	19074876	validated
mirtarbase	hsa-miR-21-5p	LDAH	60526	ENSG00000118961	18591254	validated
mirtarbase	hsa-miR-192-5p	SPCS3	60559	ENSG00000129128	19074876	validated
mirtarbase	hsa-miR-21-5p	GAS5	60674	ENSG00000234741	23933812	validated
mirtarbase	hsa-miR-222-3p	GAS5	60674	ENSG00000234741	26446789	validated
mirtarbase	hsa-miR-181a-5p	FKBP10	60681	ENSG00000141756	17612493	validated
mirtarbase	hsa-miR-106b-5p	SLC22A23	63027	ENSG00000137266	23592263	validated
mirtarbase	hsa-miR-192-5p	BCORL1	63035	ENSG00000085185	19074876	validated

mirtarbase	hsa-miR-505-3p	THADA	63892	ENSG00000115970	23622248	validated
mirtarbase	hsa-miR-106b-5p	THADA	63892	ENSG00000115970	23622248	validated
mirtarbase	hsa-miR-505-3p	UBE2O	63893	ENSG00000175931	23622248	validated
mirtarbase	hsa-miR-181a-5p	XPNPEP3	63929	ENSG00000196236	22012620	validated
mirtarbase	hsa-miR-181a-5p	MRPS14	63931	ENSG00000120333	17612493	validated
mirtarbase	hsa-miR-21-5p	ZNF667	63934	ENSG00000198046	18591254	validated
mirtarbase	hsa-miR-181a-5p	ZNF667	63934	ENSG00000198046	24398324	validated
mirtarbase	hsa-miR-21-5p	FAM217B	63939	ENSG00000196227	18591254	validated
mirtarbase	hsa-miR-144-3p	FAM217B	63939	ENSG00000196227	23592263	validated
mirtarbase	hsa-miR-301a-3p	FAM217B	63939	ENSG00000196227	23824327	validated
mirtarbase	hsa-miR-192-5p	CLSPN	63967	ENSG00000092853	19074876	validated
mirtarbase	hsa-miR-15b-5p	CLSPN	63967	ENSG00000092853	21572407	validated
mirtarbase	hsa-miR-301a-3p	KIF13A	63971	ENSG00000137177	23592263	validated
mirtarbase	hsa-miR-212-3p	PRDM15	63977	ENSG00000141956	22291592	validated
mirtarbase	hsa-miR-192-5p	PERP	64065	ENSG00000112378	16822819	validated
mirtarbase	hsa-miR-15b-5p	SNX16	64089	ENSG00000104497	23446348	validated
mirtarbase	hsa-miR-106b-5p	SMOC1	64093	ENSG00000198732	21572407	validated
mirtarbase	hsa-miR-301a-3p	SMOC1	64093	ENSG00000198732	21572407	validated
mirtarbase	hsa-miR-192-5p	CENPK	64105	ENSG00000123219	19074876	validated
mirtarbase	hsa-miR-192-5p	MOAP1	64112	ENSG00000278268	19074876	validated
mirtarbase	hsa-miR-21-5p	MOAP1	64112	ENSG00000278268	18591254	validated
mirtarbase	hsa-miR-15b-5p	VSIR	64115	ENSG00000107738	23592263	validated
mirtarbase	hsa-miR-192-5p	SLC39A8	64116	ENSG00000138821	19074876	validated
mirtarbase	hsa-miR-21-5p	RRAGC	64121	ENSG00000116954	18591254	validated
mirtarbase	hsa-miR-192-5p	NOD2	64127	ENSG00000167207	19074876	validated
mirtarbase	hsa-miR-106b-5p	LIN7B	64130	ENSG00000104863	17242205	validated
mirtarbase	hsa-miR-18a-5p	XYLT2	64132	ENSG00000015532	20371350	validated
mirtarbase	hsa-miR-15b-5p	IFIH1	64135	ENSG00000115267	23622248	validated
mirtarbase	hsa-miR-222-3p	RBSN	64145	ENSG00000131381	23622248	validated
mirtarbase	hsa-miR-106b-5p	C17orf75	64149	ENSG00000108666	27418678	validated
mirtarbase	hsa-miR-21-5p	NCAPG	64151	ENSG00000109805	19253296	validated
mirtarbase	hsa-miR-181a-5p	NCAPG	64151	ENSG00000109805	22473208	validated
mirtarbase	hsa-miR-192-5p	TOR3A	64222	ENSG00000186283	19074876	validated
mirtarbase	hsa-miR-21-5p	HERPUD2	64224	ENSG00000122557	18591254	validated
mirtarbase	hsa-miR-106b-5p	ABCG8	64241	ENSG00000143921	23313552	validated
mirtarbase	hsa-miR-301a-3p	ABCG8	64241	ENSG00000143921	27292025	validated
mirtarbase	hsa-miR-192-5p	TENT4B	64282	ENSG00000121274	19074876	validated
mirtarbase	hsa-miR-181a-5p	TENT4B	64282	ENSG00000121274	22473208	validated
mirtarbase	hsa-miR-106b-5p	TENT4B	64282	ENSG00000121274	23313552	validated
mirtarbase	hsa-miR-15b-5p	COP1	64326	ENSG00000143207	22473208	validated
mirtarbase	hsa-miR-21-5p	LMBR1	64327	ENSG00000105983	18591254	validated
mirtarbase	hsa-miR-192-5p	NFKBIZ	64332	ENSG00000144802	19074876	validated
mirtarbase	hsa-miR-296-5p	NXN	64359	ENSG00000280563	23592263	validated
mirtarbase	hsa-miR-192-5p	ZMAT3	64393	ENSG00000172667	19074876	validated
mirtarbase	hsa-miR-301a-3p	ZMAT3	64393	ENSG00000172667	24398324	validated
mirtarbase	hsa-miR-15b-5p	ZMAT3	64393	ENSG00000172667	24398324	validated
mirtarbase	hsa-miR-106b-5p	ZMAT3	64393	ENSG00000172667	23313552	validated
mirtarbase	hsa-miR-192-5p	MPP5	64398	ENSG00000072415	19074876	validated
mirtarbase	hsa-miR-21-5p	MPP5	64398	ENSG00000072415	18591254	validated
mirtarbase	hsa-miR-181a-5p	MPP5	64398	ENSG00000072415	22473208	validated
mirtarbase	hsa-miR-106b-5p	AKTIP	64400	ENSG00000166971	22473208	validated
mirtarbase	hsa-miR-106b-5p	TMEM267	64417	ENSG00000151881	22473208	validated
mirtarbase	hsa-miR-301a-3p	NOM1	64434	ENSG00000146909	23824327	validated
mirtarbase	hsa-miR-181a-5p	GIGYF1	64599	ENSG00000146830	20371350	validated
mirtarbase	hsa-miR-18a-5p	GIGYF1	64599	ENSG00000146830	20371350	validated
mirtarbase	hsa-miR-106b-5p	GIGYF1	64599	ENSG00000146830	22473208	validated
mirtarbase	hsa-miR-296-5p	GIGYF1	64599	ENSG00000146830	19536157	validated
mirtarbase	hsa-miR-18a-5p	PLA2G2F	64600	ENSG00000158786	23592263	validated
mirtarbase	hsa-miR-296-5p	COPS7B	64708	ENSG00000144524	23592263	validated
mirtarbase	hsa-miR-192-5p	NUCKS1	64710	ENSG00000069275	19074876	validated
mirtarbase	hsa-miR-505-3p	NUCKS1	64710	ENSG00000069275	23622248	validated
mirtarbase	hsa-miR-301a-3p	NUCKS1	64710	ENSG00000069275	23622248	validated
mirtarbase	hsa-miR-15b-5p	NUCKS1	64710	ENSG00000069275	22473208	validated
mirtarbase	hsa-miR-15b-5p	WDR13	64743	ENSG00000101940	26701625	validated
mirtarbase	hsa-miR-15b-5p	C16orf58	64755	ENSG00000140688	26701625	validated
mirtarbase	hsa-miR-21-5p	TNS3	64759	ENSG00000136205	18591254	validated
mirtarbase	hsa-miR-15b-5p	IPPK	64768	ENSG00000127080	23592263	validated
mirtarbase	hsa-miR-192-5p	CCDC14	64770	ENSG00000175455	19074876	validated
mirtarbase	hsa-miR-21-5p	CCDC14	64770	ENSG00000175455	18591254	validated
mirtarbase	hsa-miR-212-3p	ILRUN	64771	ENSG00000196821	23592263	validated
mirtarbase	hsa-miR-15b-5p	ILRUN	64771	ENSG00000196821	22473208	validated

mirtarbase	hsa-miR-192-5p	FNDC3B	64778	ENSG00000075420	19074876	validated
mirtarbase	hsa-miR-181a-5p	FNDC3B	64778	ENSG00000075420	22473208	validated
mirtarbase	hsa-miR-144-3p	CERK	64781	ENSG00000100422	23592263	validated
mirtarbase	hsa-miR-106b-5p	CRTC3	64784	ENSG00000140577	22473208	validated
mirtarbase	hsa-miR-222-3p	EXO5	64789	ENSG00000164002	22012620	validated
mirtarbase	hsa-miR-106b-5p	EXO5	64789	ENSG00000164002	22012620	validated
mirtarbase	hsa-miR-21-5p	RMND5A	64795	ENSG00000153561	18591254	validated
mirtarbase	hsa-miR-192-5p	ELOVL1	64834	ENSG00000066322	19074876	validated
mirtarbase	hsa-miR-15b-5p	KLC2	64837	ENSG00000174996	22473208	validated
mirtarbase	hsa-miR-21-5p	FBXL17	64839	ENSG00000145743	18591254	validated
mirtarbase	hsa-miR-505-3p	YTHDC2	64848	ENSG00000047188	23622248	validated
mirtarbase	hsa-miR-18a-5p	TUT1	64852	ENSG00000149016	23622248	validated
mirtarbase	hsa-miR-106b-5p	USP46	64854	ENSG00000109189	17242205	validated
mirtarbase	hsa-miR-192-5p	PLEKHG2	64857	ENSG00000090924	19074876	validated
mirtarbase	hsa-miR-106b-5p	NABP1	64859	ENSG00000173559	22473208	validated
mirtarbase	hsa-miR-301a-3p	NABP1	64859	ENSG00000173559	23446348	validated
mirtarbase	hsa-miR-301a-3p	RFX7	64864	ENSG00000181827	22012620	validated
mirtarbase	hsa-miR-326	BCL11B	64919	ENSG00000127152	22291592	validated
mirtarbase	hsa-miR-582-5p	SLC30A5	64924	ENSG00000145740	23446348	validated
mirtarbase	hsa-miR-15b-5p	MRPS11	64963	ENSG00000181991	23622248	validated
mirtarbase	hsa-miR-15b-5p	MRPL40	64976	ENSG00000185608	22473208	validated
mirtarbase	hsa-miR-181a-5p	MRPL34	64981	ENSG00000130312	21572407	validated
mirtarbase	hsa-miR-192-5p	REEP1	65055	ENSG00000068615	19074876	validated
mirtarbase	hsa-miR-21-5p	RAPH1	65059	ENSG00000173166	18591254	validated
mirtarbase	hsa-miR-15b-5p	RAPH1	65059	ENSG00000173166	20371350	validated
mirtarbase	hsa-miR-15b-5p	TMEM135	65084	ENSG00000166575	22473208	validated
mirtarbase	hsa-miR-212-3p	PRAMEF1	65121	ENSG00000116721	20371350	validated
mirtarbase	hsa-miR-192-5p	SOWAHC	65124	ENSG00000198142	19074876	validated
mirtarbase	hsa-miR-21-5p	SOWAHC	65124	ENSG00000198142	18591254	validated
mirtarbase	hsa-miR-15b-5p	SOWAHC	65124	ENSG00000198142	21572407	validated
mirtarbase	hsa-miR-21-5p	WNK1	65125	ENSG00000060237	18591254	validated
mirtarbase	hsa-miR-144-3p	WNK1	65125	ENSG00000060237	20371350	validated
mirtarbase	hsa-miR-181a-5p	ZFP69B	65243	ENSG00000187801	23446348	validated
mirtarbase	hsa-miR-106b-5p	MPPE1	65258	ENSG00000154889	27418678	validated
mirtarbase	hsa-miR-222-3p	C8orf33	65265	ENSG00000182307	23622248	validated
mirtarbase	hsa-miR-296-5p	WNK4	65266	ENSG00000126562	20561597	validated
mirtarbase	hsa-miR-21-5p	WNK3	65267	ENSG00000196632	21544242	validated
mirtarbase	hsa-miR-106b-5p	WNK3	65267	ENSG00000196632	22100165	validated
mirtarbase	hsa-miR-301a-3p	WNK3	65267	ENSG00000196632	22100165	validated
mirtarbase	hsa-miR-15b-5p	WNK3	65267	ENSG00000196632	20371350	validated
mirtarbase	hsa-miR-144-3p	PLEKHA3	65977	ENSG00000116095	21572407	validated
mirtarbase	hsa-miR-181a-5p	PHACTR4	65979	ENSG00000204138	22815788	validated
mirtarbase	hsa-miR-222-3p	PHACTR4	65979	ENSG00000204138	22815788	validated
mirtarbase	hsa-miR-106b-5p	CAPRIN2	65981	ENSG00000110888	22473208	validated
mirtarbase	hsa-miR-582-5p	CAPRIN2	65981	ENSG00000110888	23592263	validated
mirtarbase	hsa-miR-301a-3p	CAPRIN2	65981	ENSG00000110888	23592263	validated
mirtarbase	hsa-miR-181a-5p	CAPRIN2	65981	ENSG00000110888	23592263	validated
mirtarbase	hsa-miR-15b-5p	GRAMD2B	65983	ENSG00000155324	22473208	validated
mirtarbase	hsa-miR-192-5p	ZBTB10	65986	ENSG00000205189	19074876	validated
mirtarbase	hsa-miR-15b-5p	ZBTB10	65986	ENSG00000205189	21572407	validated
mirtarbase	hsa-miR-21-5p	MTMR9	66036	ENSG00000104643	18591254	validated
mirtarbase	hsa-miR-106b-5p	MTMR9	66036	ENSG00000104643	22927820	validated
mirtarbase	hsa-miR-192-5p	MTMR9	66036	ENSG00000104643	27292025	validated
mirtarbase	hsa-miR-192-5p	OTUB2	78990	ENSG00000277276	19074876	validated
mirtarbase	hsa-miR-326	VKORC1	79001	ENSG00000167397	23622248	validated
mirtarbase	hsa-miR-192-5p	MIS12	79003	ENSG00000167842	19074876	validated
mirtarbase	hsa-miR-192-5p	DDX50	79009	ENSG00000107625	19074876	validated
mirtarbase	hsa-miR-21-5p	GID4	79018	ENSG00000141034	18591254	validated
mirtarbase	hsa-miR-106b-5p	GID4	79018	ENSG00000141034	22473208	validated
mirtarbase	hsa-miR-505-3p	GID4	79018	ENSG00000141034	20371350	validated
mirtarbase	hsa-miR-212-3p	GID4	79018	ENSG00000141034	23313552	validated
mirtarbase	hsa-miR-106b-5p	CENPM	79019	ENSG00000100162	23622248	validated
mirtarbase	hsa-miR-505-3p	NABP2	79035	ENSG00000139579	23622248	validated
mirtarbase	hsa-miR-106b-5p	ZFYVE21	79038	ENSG00000100711	23592263	validated
mirtarbase	hsa-miR-192-5p	DDX54	79039	ENSG00000123064	20371350	validated
mirtarbase	hsa-miR-106b-5p	TMEM38A	79041	ENSG00000072954	23446348	validated
mirtarbase	hsa-miR-192-5p	KCTD15	79047	ENSG00000153885	19074876	validated
mirtarbase	hsa-miR-192-5p	PRRG4	79056	ENSG00000135378	19074876	validated
mirtarbase	hsa-miR-106b-5p	PRRG4	79056	ENSG00000135378	20371350	validated
mirtarbase	hsa-miR-301a-3p	PRRG4	79056	ENSG00000135378	20371350	validated
mirtarbase	hsa-miR-15b-5p	ATG9A	79065	ENSG00000198925	22473208	validated

mirtarbase	hsa-miR-106b-5p	ELOVL6	79071	ENSG00000170522	23622248	validated
mirtarbase	hsa-miR-15b-5p	TMEM109	79073	ENSG00000110108	22473208	validated
mirtarbase	hsa-miR-192-5p	C2orf49	79074	ENSG00000135974	19074876	validated
mirtarbase	hsa-miR-106b-5p	DCTPP1	79077	ENSG00000179958	23622248	validated
mirtarbase	hsa-miR-106b-5p	C1orf50	79078	ENSG00000164008	23313552	validated
mirtarbase	hsa-miR-181a-5p	ZNF426	79088	ENSG00000130818	17612493	validated
mirtarbase	hsa-miR-106b-5p	ZNF426	79088	ENSG00000130818	23313552	validated
mirtarbase	hsa-miR-18a-5p	TMUB2	79089	ENSG00000168591	20371350	validated
mirtarbase	hsa-miR-15b-5p	CHAC1	79094	ENSG00000128965	23592263	validated
mirtarbase	hsa-miR-144-3p	CHAC1	79094	ENSG00000128965	23592263	validated
mirtarbase	hsa-miR-212-3p	CHAC1	79094	ENSG00000128965	23592263	validated
mirtarbase	hsa-miR-192-5p	RNF26	79102	ENSG00000173456	19074876	validated
mirtarbase	hsa-miR-21-5p	DERL1	79139	ENSG00000136986	19253296	validated
mirtarbase	hsa-miR-181a-5p	CHCHD7	79145	ENSG00000170791	22473208	validated
mirtarbase	hsa-miR-301a-3p	GNPTAB	79158	ENSG00000111670	21572407	validated
mirtarbase	hsa-miR-106b-5p	GNPTAB	79158	ENSG00000111670	21572407	validated
mirtarbase	hsa-miR-181a-5p	CENPO	79172	ENSG00000138092	19536157	validated
mirtarbase	hsa-miR-106b-5p	TTPAL	79183	ENSG00000124120	23622248	validated
mirtarbase	hsa-miR-181a-5p	TTPAL	79183	ENSG00000124120	22473208	validated
mirtarbase	hsa-miR-15b-5p	TMEM43	79188	ENSG00000170876	23622248	validated
mirtarbase	hsa-miR-21-5p	DCAF10	79269	ENSG00000122741	18591254	validated
mirtarbase	hsa-miR-192-5p	ZXDC	79364	ENSG00000070476	19074876	validated
mirtarbase	hsa-miR-192-5p	HAUS3	79441	ENSG00000214367	19074876	validated
mirtarbase	hsa-miR-15b-5p	HAUS3	79441	ENSG00000214367	23446348	validated
mirtarbase	hsa-miR-106b-5p	FYCO1	79443	ENSG00000163820	22473208	validated
mirtarbase	hsa-miR-15b-5p	PAGR1	79447	ENSG00000280789	23446348	validated
mirtarbase	hsa-miR-326	ULBP3	79465	ENSG00000131019	23824327	validated
mirtarbase	hsa-miR-15b-5p	ATP13A3	79572	ENSG00000133657	22473208	validated
mirtarbase	hsa-miR-301a-3p	NKAP	79576	ENSG00000101882	23824327	validated
mirtarbase	hsa-miR-582-5p	NKAP	79576	ENSG00000101882	27292025	validated
mirtarbase	hsa-miR-15b-5p	CHPF	79586	ENSG00000123989	23622248	validated
mirtarbase	hsa-miR-192-5p	OBI1	79596	ENSG00000152193	19074876	validated
mirtarbase	hsa-miR-21-5p	CEP97	79598	ENSG00000182504	18591254	validated
mirtarbase	hsa-miR-106b-5p	CEP97	79598	ENSG00000182504	22473208	validated
mirtarbase	hsa-miR-106b-5p	VCPKMT	79609	ENSG00000100483	22473208	validated
mirtarbase	hsa-miR-15b-5p	TANGO6	79613	ENSG00000103047	23622248	validated
mirtarbase	hsa-miR-222-3p	TANGO6	79613	ENSG00000103047	23622248	validated
mirtarbase	hsa-miR-15b-5p	HMBOX1	79618	ENSG00000147421	22473208	validated
mirtarbase	hsa-miR-222-3p	HMBOX1	79618	ENSG00000147421	22012620	validated
mirtarbase	hsa-miR-106b-5p	HMBOX1	79618	ENSG00000147421	22012620	validated
mirtarbase	hsa-miR-106b-5p	ARMT1	79624	ENSG00000146476	27418678	validated
mirtarbase	hsa-miR-192-5p	OGFRL1	79627	ENSG00000119900	19074876	validated
mirtarbase	hsa-miR-212-3p	SH3TC2	79628	ENSG00000169247	27292025	validated
mirtarbase	hsa-miR-192-5p	SCRN3	79634	ENSG00000144306	19074876	validated
mirtarbase	hsa-miR-192-5p	CCDC121	79635	ENSG00000176714	19074876	validated
mirtarbase	hsa-miR-106b-5p	ARSJ	79642	ENSG00000180801	21572407	validated
mirtarbase	hsa-miR-181a-5p	ARSJ	79642	ENSG00000180801	21572407	validated
mirtarbase	hsa-miR-106b-5p	PANK3	79646	ENSG00000120137	23622248	validated
mirtarbase	hsa-miR-222-3p	PANK3	79646	ENSG00000120137	23622248	validated
mirtarbase	hsa-miR-144-3p	AKIRIN1	79647	ENSG00000174574	23592263	validated
mirtarbase	hsa-miR-15b-5p	MCPH1	79648	ENSG00000147316	23622248	validated
mirtarbase	hsa-miR-192-5p	RPAP3	79657	ENSG00000005175	19074876	validated
mirtarbase	hsa-miR-106b-5p	PPP1R3B	79660	ENSG00000173281	22473208	validated
mirtarbase	hsa-miR-192-5p	HSPBAP1	79663	ENSG00000169087	19074876	validated
mirtarbase	hsa-miR-326	DHX40	79665	ENSG00000108406	23592263	validated
mirtarbase	hsa-miR-192-5p	PLEKHF2	79666	ENSG00000175895	19074876	validated
mirtarbase	hsa-miR-301a-3p	PLEKHF2	79666	ENSG00000175895	24398324	validated
mirtarbase	hsa-miR-222-3p	SMC6	79677	ENSG00000163029	23622248	validated
mirtarbase	hsa-miR-296-5p	RTL10	79680	ENSG00000215012	26701625	validated
mirtarbase	hsa-miR-222-3p	SAP30L	79685	ENSG00000164576	23622248	validated
mirtarbase	hsa-miR-15b-5p	YRDC	79693	ENSG00000196449	21572407	validated
mirtarbase	hsa-miR-192-5p	GALNT12	79695	ENSG00000119514	19074876	validated
mirtarbase	hsa-miR-21-5p	ZYG11B	79699	ENSG00000162378	18591254	validated
mirtarbase	hsa-miR-192-5p	NOL9	79707	ENSG00000162408	23824327	validated
mirtarbase	hsa-miR-21-5p	TBL1XR1	79718	ENSG00000177565	18591254	validated
mirtarbase	hsa-miR-326	TBL1XR1	79718	ENSG00000177565	23592263	validated
mirtarbase	hsa-miR-106b-5p	TBL1XR1	79718	ENSG00000177565	22012620	validated
mirtarbase	hsa-miR-181a-5p	TBL1XR1	79718	ENSG00000177565	22473208	validated
mirtarbase	hsa-miR-15b-5p	TBL1XR1	79718	ENSG00000177565	22473208	validated
mirtarbase	hsa-miR-296-5p	VPS37B	79720	ENSG00000139722	23446348	validated
mirtarbase	hsa-miR-301a-3p	VPS37B	79720	ENSG00000139722	23446348	validated

mirtarbase	hsa-miR-181a-5p	SUV39H2	79723	ENSG00000152455	20371350	validated
mirtarbase	hsa-miR-192-5p	E2F8	79733	ENSG00000129173	19074876	validated
mirtarbase	hsa-miR-106b-5p	TBC1D17	79735	ENSG00000104946	22473208	validated
mirtarbase	hsa-miR-192-5p	CLIP4	79745	ENSG00000115295	19074876	validated
mirtarbase	hsa-miR-21-5p	CLIP4	79745	ENSG00000115295	18591254	validated
mirtarbase	hsa-miR-106b-5p	CLIP4	79745	ENSG00000115295	20371350	validated
mirtarbase	hsa-miR-15b-5p	CLIP4	79745	ENSG00000115295	21572407	validated
mirtarbase	hsa-miR-15b-5p	SLC25A22	79751	ENSG00000177542	22473208	validated
mirtarbase	hsa-miR-301a-3p	SNIP1	79753	ENSG00000163877	26338824	validated
mirtarbase	hsa-miR-18a-5p	TXNDC15	79770	ENSG00000113621	23622248	validated
mirtarbase	hsa-miR-15b-5p	ZFHX4	79776	ENSG00000091656	23446348	validated
mirtarbase	hsa-miR-301a-3p	KLHL36	79786	ENSG00000135686	23446348	validated
mirtarbase	hsa-miR-106b-5p	KLHL36	79786	ENSG00000135686	23446348	validated
mirtarbase	hsa-miR-106b-5p	ZNF665	79788	ENSG00000197497	23313552	validated
mirtarbase	hsa-miR-192-5p	FBXO31	79791	ENSG00000103264	19074876	validated
mirtarbase	hsa-miR-106b-5p	FBXO31	79791	ENSG00000103264	22473208	validated
mirtarbase	hsa-miR-192-5p	VASH2	79805	ENSG00000143494	19074876	validated
mirtarbase	hsa-miR-21-5p	VASH2	79805	ENSG00000143494	19253296	validated
mirtarbase	hsa-miR-106b-5p	AGMAT	79814	ENSG00000116771	20371350	validated
mirtarbase	hsa-miR-181a-5p	MOB3B	79817	ENSG00000120162	17612493	validated
mirtarbase	hsa-miR-106b-5p	METTL8	79828	ENSG00000123600	23313552	validated
mirtarbase	hsa-miR-192-5p	ZMYM1	79830	ENSG00000197056	19074876	validated
mirtarbase	hsa-miR-106b-5p	ZMYM1	79830	ENSG00000197056	22012620	validated
mirtarbase	hsa-miR-505-3p	QSER1	79832	ENSG00000060749	20371350	validated
mirtarbase	hsa-miR-106b-5p	PEAK1	79834	ENSG00000173517	22473208	validated
mirtarbase	hsa-miR-106b-5p	PIP4K2C	79837	ENSG00000166908	22473208	validated
mirtarbase	hsa-miR-192-5p	MFSD13A	79847	ENSG00000138111	19074876	validated
mirtarbase	hsa-miR-106b-5p	TLCD3A	79850	ENSG00000167695	22473208	validated
mirtarbase	hsa-miR-181a-5p	ZNF669	79862	ENSG00000188295	24398324	validated
mirtarbase	hsa-miR-192-5p	BORA	79866	ENSG00000136122	19074876	validated
mirtarbase	hsa-miR-15b-5p	CPSF7	79869	ENSG00000149532	23592263	validated
mirtarbase	hsa-miR-192-5p	RPAP2	79871	ENSG00000122484	19074876	validated
mirtarbase	hsa-miR-192-5p	UBA5	79876	ENSG00000081307	22291592	validated
mirtarbase	hsa-miR-181a-5p	LPCAT1	79888	ENSG00000153395	22473208	validated
mirtarbase	hsa-miR-21-5p	CYBRD1	79901	ENSG00000071967	18591254	validated
mirtarbase	hsa-miR-301a-3p	CYBRD1	79901	ENSG00000071967	23622248	validated
mirtarbase	hsa-miR-106b-5p	CYBRD1	79901	ENSG00000071967	22473208	validated
mirtarbase	hsa-miR-15b-5p	TMC7	79905	ENSG00000170537	23622248	validated
mirtarbase	hsa-miR-192-5p	ATAD5	79915	ENSG00000176208	19074876	validated
mirtarbase	hsa-miR-15b-5p	ATAD5	79915	ENSG00000176208	23446348	validated
mirtarbase	hsa-miR-301a-3p	ADM2	79924	ENSG00000128165	23824327	validated
mirtarbase	hsa-miR-192-5p	MAP6D1	79929	ENSG00000180834	19074876	validated
mirtarbase	hsa-miR-106b-5p	TNIP3	79931	ENSG00000050730	23313552	validated
mirtarbase	hsa-miR-106b-5p	SYNPO2L	79933	ENSG00000166317	23313552	validated
mirtarbase	hsa-miR-192-5p	CNTNAP3	79937	ENSG00000106714	19074876	validated
mirtarbase	hsa-miR-192-5p	L2HGDH	79944	ENSG00000087299	19074876	validated
mirtarbase	hsa-miR-582-5p	L2HGDH	79944	ENSG00000087299	23592263	validated
mirtarbase	hsa-miR-15b-5p	L2HGDH	79944	ENSG00000087299	24398324	validated
mirtarbase	hsa-miR-301a-3p	PLEKHS1	79949	ENSG00000148735	23824327	validated
mirtarbase	hsa-miR-192-5p	ERMP1	79956	ENSG00000099219	19074876	validated
mirtarbase	hsa-miR-192-5p	JADE1	79960	ENSG00000077684	19074876	validated
mirtarbase	hsa-miR-21-5p	JADE1	79960	ENSG00000077684	18591254	validated
mirtarbase	hsa-miR-192-5p	WDR76	79968	ENSG00000092470	19074876	validated
mirtarbase	hsa-miR-106b-5p	ATAT1	79969	ENSG00000223752	23824327	validated
mirtarbase	hsa-miR-192-5p	DSN1	79980	ENSG00000149636	19074876	validated
mirtarbase	hsa-miR-222-3p	DNAJB14	79982	ENSG00000164031	26701625	validated
mirtarbase	hsa-miR-222-3p	STN1	79991	ENSG00000107960	23446348	validated
mirtarbase	hsa-miR-21-5p	ELOVL7	79993	ENSG00000164181	18591254	validated
mirtarbase	hsa-miR-106b-5p	PCNX2	80003	ENSG00000135749	21572407	validated
mirtarbase	hsa-miR-21-5p	DOCK5	80005	ENSG00000147459	22158624	validated
mirtarbase	hsa-miR-222-3p	DOCK5	80005	ENSG00000147459	23622248	validated
mirtarbase	hsa-miR-192-5p	RMI1	80010	ENSG00000178966	19074876	validated
mirtarbase	hsa-miR-181a-5p	PSME3IP1	80011	ENSG00000172775	22473208	validated
mirtarbase	hsa-miR-181a-5p	PHC3	80012	ENSG00000173889	22473208	validated
mirtarbase	hsa-miR-15b-5p	PHC3	80012	ENSG00000173889	22473208	validated
mirtarbase	hsa-miR-192-5p	WWC2	80014	ENSG00000151718	19074876	validated
mirtarbase	hsa-miR-21-5p	WWC2	80014	ENSG00000151718	19253296	validated
mirtarbase	hsa-miR-15b-5p	NAA25	80018	ENSG00000111300	23592263	validated
mirtarbase	hsa-miR-106b-5p	FOXRED2	80020	ENSG00000100350	23313552	validated
mirtarbase	hsa-miR-222-3p	FBXL18	80028	ENSG00000155034	23313552	validated
mirtarbase	hsa-miR-15b-5p	FBXL18	80028	ENSG00000155034	26701625	validated

mirtarbase	hsa-miR-181a-5p	ZNF556	80032	ENSG00000172000	23824327	validated
mirtarbase	hsa-miR-18a-5p	CSRNP3	80034	ENSG00000178662	16331254	validated
mirtarbase	hsa-miR-106b-5p	GPR157	80045	ENSG00000180758	23592263	validated
mirtarbase	hsa-miR-181a-5p	PGAP1	80055	ENSG00000197121	22473208	validated
mirtarbase	hsa-miR-181a-5p	ATF7IP2	80063	ENSG00000166669	17612493	validated
mirtarbase	hsa-miR-15b-5p	DCAF17	80067	ENSG00000115827	23706177	validated
mirtarbase	hsa-miR-212-3p	DCAF17	80067	ENSG00000115827	23706177	validated
mirtarbase	hsa-miR-192-5p	CCDC15	80071	ENSG00000149548	19074876	validated
mirtarbase	hsa-miR-15b-5p	C3orf36	80111		23824327	validated
mirtarbase	hsa-miR-192-5p	PIF1	80119	ENSG00000140451	19074876	validated
mirtarbase	hsa-miR-192-5p	BBOF1	80127	ENSG00000119636	19074876	validated
mirtarbase	hsa-miR-18a-5p	ZNF703	80139	ENSG00000183779	23622248	validated
mirtarbase	hsa-miR-106b-5p	SIKE1	80143	ENSG00000052723	22473208	validated
mirtarbase	hsa-miR-106b-5p	UXS1	80146	ENSG00000115652	22473208	validated
mirtarbase	hsa-miR-192-5p	ASRGL1	80150	ENSG00000162174	19074876	validated
mirtarbase	hsa-miR-21-5p	ASRGL1	80150	ENSG00000162174	18591254	validated
mirtarbase	hsa-miR-15b-5p	EDC3	80153	ENSG00000179151	22473208	validated
mirtarbase	hsa-miR-296-5p	NAA15	80155	ENSG00000164134	23824327	validated
mirtarbase	hsa-miR-106b-5p	ABHD18	80167	ENSG00000164074	23313552	validated
mirtarbase	hsa-miR-15b-5p	IFT74	80173	ENSG00000096872	22473208	validated
mirtarbase	hsa-miR-192-5p	DBF4B	80174	ENSG00000161692	19074876	validated
mirtarbase	hsa-miR-505-3p	MYO19	80179	ENSG00000278372	23622248	validated
mirtarbase	hsa-miR-106b-5p	TMEM134	80194	ENSG00000172663	23446348	validated
mirtarbase	hsa-miR-181a-5p	RNF34	80196	ENSG00000170633	20371350	validated
mirtarbase	hsa-miR-106b-5p	RNF34	80196	ENSG00000170633	23313552	validated
mirtarbase	hsa-miR-181a-5p	FBXO11	80204	ENSG00000138081	20371350	validated
mirtarbase	hsa-miR-21-5p	FBXO11	80204	ENSG00000138081	18591254	validated
mirtarbase	hsa-miR-106b-5p	CHD9	80205	ENSG00000177200	22473208	validated
mirtarbase	hsa-miR-181a-5p	CHD9	80205	ENSG00000177200	22473208	validated
mirtarbase	hsa-miR-21-5p	SPG11	80208	ENSG00000104133	18591254	validated
mirtarbase	hsa-miR-21-5p	PROSER1	80209	ENSG00000120685	18591254	validated
mirtarbase	hsa-miR-192-5p	NAA50	80218	ENSG00000121579	19074876	validated
mirtarbase	hsa-miR-106b-5p	NAA50	80218	ENSG00000121579	22473208	validated
mirtarbase	hsa-miR-21-5p	NAA50	80218	ENSG00000121579	22473208	validated
mirtarbase	hsa-miR-301a-3p	NAA50	80218	ENSG00000121579	23824327	validated
mirtarbase	hsa-miR-181a-5p	NAA50	80218	ENSG00000121579	22473208	validated
mirtarbase	hsa-miR-301a-3p	RAB11FIP1	80223	ENSG00000156675	23824327	validated
mirtarbase	hsa-miR-106b-5p	RAB11FIP1	80223	ENSG00000156675	22473208	validated
mirtarbase	hsa-miR-21-5p	NUBPL	80224	ENSG00000151413	18591254	validated
mirtarbase	hsa-miR-106b-5p	ORAI2	80228	ENSG00000160991	23313552	validated
mirtarbase	hsa-miR-582-5p	ORAI2	80228	ENSG00000160991	26701625	validated
mirtarbase	hsa-miR-301a-3p	CXorf21	80231	ENSG00000120280	23824327	validated
mirtarbase	hsa-miR-106b-5p	SLC35F5	80255	ENSG00000115084	22473208	validated
mirtarbase	hsa-miR-144-3p	SLC35F5	80255	ENSG00000115084	23592263	validated
mirtarbase	hsa-miR-301a-3p	C16orf70	80262	ENSG00000125149	21572407	validated
mirtarbase	hsa-miR-106b-5p	C16orf70	80262	ENSG00000125149	21572407	validated
mirtarbase	hsa-miR-505-3p	EDEM3	80267	ENSG00000116406	23313552	validated
mirtarbase	hsa-miR-106b-5p	PLEKHO2	80301	ENSG00000241839	22473208	validated
mirtarbase	hsa-miR-192-5p	WDCP	80304	ENSG00000163026	19074876	validated
mirtarbase	hsa-miR-144-3p	WDCP	80304	ENSG00000163026	23592263	validated
mirtarbase	hsa-miR-21-5p	PDGFD	80310	ENSG00000170962	18591254	validated
mirtarbase	hsa-miR-192-5p	KLHL15	80311	ENSG00000174010	19074876	validated
mirtarbase	hsa-miR-21-5p	KLHL15	80311	ENSG00000174010	18591254	validated
mirtarbase	hsa-miR-181a-5p	KLHL15	80311	ENSG00000174010	23622248	validated
mirtarbase	hsa-miR-106b-5p	KLHL15	80311	ENSG00000174010	22473208	validated
mirtarbase	hsa-miR-15b-5p	KLHL15	80311	ENSG00000174010	20371350	validated
mirtarbase	hsa-miR-21-5p	TET1	80312	ENSG00000138336	18591254	validated
mirtarbase	hsa-miR-505-3p	TET1	80312	ENSG00000138336	23824327	validated
mirtarbase	hsa-miR-181a-5p	CPEB4	80315	ENSG00000113742	22473208	validated
mirtarbase	hsa-miR-15b-5p	DNAJC5	80331	ENSG00000101152	23622248	validated
mirtarbase	hsa-miR-192-5p	TRAF3IP3	80342	ENSG00000009790	19074876	validated
mirtarbase	hsa-miR-21-5p	DCAF11	80344	ENSG00000100897	26701625	validated
mirtarbase	hsa-miR-296-5p	REEP4	80346	ENSG00000168476	27292025	validated
mirtarbase	hsa-miR-144-3p	COASY	80347	ENSG00000068120	23592263	validated
mirtarbase	hsa-miR-106b-5p	TNKS2	80351	ENSG00000107854	22473208	validated
mirtarbase	hsa-miR-222-3p	SLC19A3	80704	ENSG00000135917	23592263	validated
mirtarbase	hsa-miR-181a-5p	SLC35G2	80723	ENSG00000168917	21572407	validated
mirtarbase	hsa-miR-15b-5p	NDFIP1	80762	ENSG00000131507	23622248	validated
mirtarbase	hsa-miR-582-5p	ZNF436	80818	ENSG00000125945	23592263	validated
mirtarbase	hsa-miR-192-5p	DDHD1	80821	ENSG00000100523	19074876	validated
mirtarbase	hsa-miR-106b-5p	DDHD1	80821	ENSG00000100523	22473208	validated

mirtarbase	hsa-miR-192-5p	DUSP16	80824	ENSG00000111266	19074876	validated
mirtarbase	hsa-miR-181a-5p	APOL6	80830	ENSG00000221963	22473208	validated
mirtarbase	hsa-miR-21-5p	LNPK	80856	ENSG00000144320	18591254	validated
mirtarbase	hsa-miR-212-3p	SLC25A32	81034	ENSG00000164933	23824327	validated
mirtarbase	hsa-miR-222-3p	PTDSS2	81490	ENSG00000282367	23622248	validated
mirtarbase	hsa-miR-192-5p	SGPP1	81537	ENSG00000126821	19074876	validated
mirtarbase	hsa-miR-15b-5p	VOPP1	81552	ENSG00000154978	23622248	validated
mirtarbase	hsa-miR-15b-5p	C1orf21	81563	ENSG00000116667	23446348	validated
mirtarbase	hsa-miR-301a-3p	PDRG1	81572	ENSG00000088356	23592263	validated
mirtarbase	hsa-miR-106b-5p	PDRG1	81572	ENSG00000088356	23592263	validated
mirtarbase	hsa-miR-181a-5p	ANKRD13C	81573	ENSG00000118454	17612493	validated
mirtarbase	hsa-miR-106b-5p	ANKRD13C	81573	ENSG00000118454	22473208	validated
mirtarbase	hsa-miR-192-5p	APOLD1	81575	ENSG00000178878	19074876	validated
mirtarbase	hsa-miR-21-5p	APOLD1	81575	ENSG00000178878	18591254	validated
mirtarbase	hsa-miR-301a-3p	PLA2G12A	81579	ENSG00000123739	23824327	validated
mirtarbase	hsa-miR-15b-5p	CDADC1	81602	ENSG00000102543	20371350	validated
mirtarbase	hsa-miR-301a-3p	CDADC1	81602	ENSG00000102543	22100165	validated
mirtarbase	hsa-miR-106b-5p	TRIM8	81603	ENSG00000171206	22473208	validated
mirtarbase	hsa-miR-192-5p	FAM83D	81610	ENSG00000101447	19074876	validated
mirtarbase	hsa-miR-106b-5p	FAM83D	81610	ENSG00000101447	22473208	validated
mirtarbase	hsa-miR-192-5p	CAB39L	81617	ENSG00000102547	19074876	validated
mirtarbase	hsa-miR-18a-5p	ITM2C	81618	ENSG00000135916	23446348	validated
mirtarbase	hsa-miR-192-5p	MAP1LC3B	81631	ENSG00000140941	19074876	validated
mirtarbase	hsa-miR-505-3p	VMP1	81671	ENSG00000062716	23592263	validated
mirtarbase	hsa-miR-212-3p	VMP1	81671	ENSG00000062716	23592263	validated
mirtarbase	hsa-miR-192-5p	C6orf62	81688	ENSG00000112308	19074876	validated
mirtarbase	hsa-miR-192-5p	REXO5	81691	ENSG00000005189	19074876	validated
mirtarbase	hsa-miR-301a-3p	PPP1R14C	81706	ENSG00000198729	21572407	validated
mirtarbase	hsa-miR-222-3p	PPP1R14C	81706	ENSG00000198729	23446348	validated
mirtarbase	hsa-miR-106b-5p	SLCO5A1	81796	ENSG00000137571	22100165	validated
mirtarbase	hsa-miR-21-5p	NETO2	81831	ENSG00000171208	18591254	validated
mirtarbase	hsa-miR-106b-5p	NETO2	81831	ENSG00000171208	23592263	validated
mirtarbase	hsa-miR-192-5p	VANGL1	81839	ENSG00000173218	19074876	validated
mirtarbase	hsa-miR-222-3p	VANGL1	81839	ENSG00000173218	22012620	validated
mirtarbase	hsa-miR-18a-5p	RNF146	81847	ENSG00000118518	21572407	validated
mirtarbase	hsa-miR-192-5p	SPRY4	81848	ENSG00000187678	19074876	validated
mirtarbase	hsa-miR-21-5p	SPRY4	81848	ENSG00000187678	23824327	validated
mirtarbase	hsa-miR-192-5p	ZNF611	81856	ENSG00000213020	19074876	validated
mirtarbase	hsa-miR-222-3p	ISG20L2	81875	ENSG00000143319	23622248	validated
mirtarbase	hsa-miR-15b-5p	RAB1B	81876	ENSG00000174903	23622248	validated
mirtarbase	hsa-miR-106b-5p	FAHD1	81889	ENSG00000180185	19536157	validated
mirtarbase	hsa-miR-18a-5p	SLC25A28	81894	ENSG00000155287	23592263	validated
mirtarbase	hsa-miR-192-5p	KIF18A	81930	ENSG00000121621	19074876	validated
mirtarbase	hsa-miR-106b-5p	ZNF93	81931	ENSG00000184635	22012620	validated
mirtarbase	hsa-miR-15b-5p	GSG1	83445	ENSG00000111305	21572407	validated
mirtarbase	hsa-miR-582-5p	RAB33B	83452	ENSG00000172007	21572407	validated
mirtarbase	hsa-miR-18a-5p	MXD3	83463	ENSG00000213347	23622248	validated
mirtarbase	hsa-miR-192-5p	NUF2	83540	ENSG00000143228	19074876	validated
mirtarbase	hsa-miR-192-5p	DNAL1	83544	ENSG00000119661	19074876	validated
mirtarbase	hsa-miR-106b-5p	DNAL1	83544	ENSG00000119661	23313552	validated
mirtarbase	hsa-miR-192-5p	UCK1	83549	ENSG00000130717	19074876	validated
mirtarbase	hsa-miR-326	UCK1	83549	ENSG00000130717	23622248	validated
mirtarbase	hsa-miR-296-5p	MFRP	83552	ENSG00000235718	19536157	validated
mirtarbase	hsa-miR-222-3p	TMUB1	83590	ENSG00000164897	23622248	validated
mirtarbase	hsa-miR-15b-5p	RASSF5	83593	ENSG00000266094	22473208	validated
mirtarbase	hsa-miR-181a-5p	NUDT12	83594	ENSG00000112874	17612493	validated
mirtarbase	hsa-miR-15b-5p	BCL2L12	83596	ENSG00000126453	26701625	validated
mirtarbase	hsa-miR-15b-5p	AMMECR1L	83607	ENSG00000144233	23622248	validated
mirtarbase	hsa-miR-222-3p	C19orf12	83636	ENSG00000131943	21572407	validated
mirtarbase	hsa-miR-15b-5p	RAMAC	83640	ENSG00000169612	23446348	validated
mirtarbase	hsa-miR-21-5p	PARP9	83666	ENSG00000138496	18591254	validated
mirtarbase	hsa-miR-106b-5p	SESN2	83667	ENSG00000130766	22473208	validated
mirtarbase	hsa-miR-212-3p	CALN1	83698	ENSG00000183166	19536157	validated
mirtarbase	hsa-miR-106b-5p	CRISPLD2	83716	ENSG00000103196	23446348	validated
mirtarbase	hsa-miR-181a-5p	ATG10	83734	ENSG00000152348	17612493	validated
mirtarbase	hsa-miR-192-5p	ATG10	83734	ENSG00000152348	19074876	validated
mirtarbase	hsa-miR-326	GRWD1	83743	ENSG00000105447	23824327	validated
mirtarbase	hsa-miR-181a-5p	ROPN1L	83853	ENSG00000145491	17612493	validated
mirtarbase	hsa-miR-192-5p	ROPN1L	83853	ENSG00000145491	19074876	validated
mirtarbase	hsa-miR-181a-5p	FSD1L	83856	ENSG00000106701	22473208	validated
mirtarbase	hsa-miR-301a-3p	TMTC1	83857	ENSG00000133687	19536157	validated

mirtarbase	hsa-miR-301a-3p	RAB34	83871	ENSG00000109113	21572407	validated
mirtarbase	hsa-miR-106b-5p	MIXL1	83881	ENSG00000185155	23446348	validated
mirtarbase	hsa-miR-301a-3p	KCTD10	83892	ENSG00000110906	23592263	validated
mirtarbase	hsa-miR-192-5p	HASPIN	83903	ENSG00000177602	19074876	validated
mirtarbase	hsa-miR-106b-5p	STK40	83931	ENSG00000196182	23622248	validated
mirtarbase	hsa-miR-296-5p	TMEM133	83935		22012620	validated
mirtarbase	hsa-miR-106b-5p	TMEM133	83935		21572407	validated
mirtarbase	hsa-miR-192-5p	BRIP1	83990	ENSG00000136492	19074876	validated
mirtarbase	hsa-miR-301a-3p	KREMEN1	83999	ENSG00000183762	22012620	validated
mirtarbase	hsa-miR-192-5p	B3GNT5	84002	ENSG00000176597	19074876	validated
mirtarbase	hsa-miR-21-5p	B3GNT5	84002	ENSG00000176597	18591254	validated
mirtarbase	hsa-miR-106b-5p	KATNAL1	84056	ENSG00000102781	17242205	validated
mirtarbase	hsa-miR-192-5p	KATNAL1	84056	ENSG00000102781	20371350	validated
mirtarbase	hsa-miR-15b-5p	KATNAL1	84056	ENSG00000102781	22473208	validated
mirtarbase	hsa-miR-181a-5p	FAM160A2	84067	ENSG00000051009	20371350	validated
mirtarbase	hsa-miR-106b-5p	SLC10A7	84068	ENSG00000120519	23622248	validated
mirtarbase	hsa-miR-222-3p	SLC10A7	84068	ENSG00000120519	23824327	validated
mirtarbase	hsa-miR-181a-5p	SLC10A7	84068	ENSG00000120519	22473208	validated
mirtarbase	hsa-miR-212-3p	SLC10A7	84068	ENSG00000120519	27292025	validated
mirtarbase	hsa-miR-21-5p	KBTBD7	84078	ENSG00000120696	18591254	validated
mirtarbase	hsa-miR-192-5p	RAB6C	84084	ENSG00000222014	19074876	validated
mirtarbase	hsa-miR-21-5p	RAB6C	84084	ENSG00000222014	18591254	validated
mirtarbase	hsa-miR-15b-5p	FBXO30	84085	ENSG00000118496	23622248	validated
mirtarbase	hsa-miR-106b-5p	QRFPR	84109	ENSG00000186867	23313552	validated
mirtarbase	hsa-miR-326	ZNF394	84124	ENSG00000160908	23446348	validated
mirtarbase	hsa-miR-192-5p	CEP78	84131	ENSG00000148019	19074876	validated
mirtarbase	hsa-miR-15b-5p	USP42	84132	ENSG00000106346	23446348	validated
mirtarbase	hsa-miR-15b-5p	ZNRF3	84133	ENSG00000183579	22100165	validated
mirtarbase	hsa-miR-192-5p	ZNRF3	84133	ENSG00000183579	22100165	validated
mirtarbase	hsa-miR-301a-3p	RPF2	84154	ENSG00000197498	21572407	validated
mirtarbase	hsa-miR-106b-5p	RPF2	84154	ENSG00000197498	21572407	validated
mirtarbase	hsa-miR-106b-5p	GTF2IRD2	84163	ENSG00000196275	23313552	validated
mirtarbase	hsa-miR-106b-5p	POLR1B	84172	ENSG00000125630	23446348	validated
mirtarbase	hsa-miR-301a-3p	POLR1B	84172	ENSG00000125630	23446348	validated
mirtarbase	hsa-miR-192-5p	TMEM164	84187	ENSG00000157600	19074876	validated
mirtarbase	hsa-miR-181a-5p	CIAO2A	84191	ENSG00000166797	23592263	validated
mirtarbase	hsa-miR-15b-5p	USP48	84196	ENSG00000090686	21572407	validated
mirtarbase	hsa-miR-106b-5p	USP48	84196	ENSG00000090686	21572407	validated
mirtarbase	hsa-miR-192-5p	MEX3B	84206	ENSG00000183496	19074876	validated
mirtarbase	hsa-miR-181a-5p	IQCG	84223	ENSG00000114473	17612493	validated
mirtarbase	hsa-miR-192-5p	LRRC8C	84230	ENSG00000171488	19074876	validated
mirtarbase	hsa-miR-21-5p	TRAF7	84231	ENSG00000131653	26080425	validated
mirtarbase	hsa-miR-222-3p	FYTTD1	84248	ENSG00000122068	23622248	validated
mirtarbase	hsa-miR-192-5p	SLF1	84250	ENSG00000133302	19074876	validated
mirtarbase	hsa-miR-181a-5p	SLC37A3	84255	ENSG00000157800	20371350	validated
mirtarbase	hsa-miR-192-5p	DCUN1D5	84259	ENSG00000137692	22100165	validated
mirtarbase	hsa-miR-192-5p	TCHP	84260	ENSG00000139437	19074876	validated
mirtarbase	hsa-miR-192-5p	CHCHD5	84269	ENSG00000125611	23824327	validated
mirtarbase	hsa-miR-15b-5p	POLDIP3	84271	ENSG00000100227	21572407	validated
mirtarbase	hsa-miR-106b-5p	YIPF4	84272	ENSG00000119820	23446348	validated
mirtarbase	hsa-miR-582-5p	SLC25A33	84275	ENSG00000171612	23592263	validated
mirtarbase	hsa-miR-106b-5p	SLC25A33	84275	ENSG00000171612	23313552	validated
mirtarbase	hsa-miR-192-5p	PRADC1	84279	ENSG00000135617	19074876	validated
mirtarbase	hsa-miR-15b-5p	ZDHHC16	84287	ENSG00000171307	21572407	validated
mirtarbase	hsa-miR-106b-5p	PRXL2A	84293	ENSG00000122378	23313552	validated
mirtarbase	hsa-miR-192-5p	PHF6	84295	ENSG00000156531	19074876	validated
mirtarbase	hsa-miR-106b-5p	PHF6	84295	ENSG00000156531	22473208	validated
mirtarbase	hsa-miR-106b-5p	GINS4	84296	ENSG00000147536	23446348	validated
mirtarbase	hsa-miR-106b-5p	LLPH	84298	ENSG00000139233	22100165	validated
mirtarbase	hsa-miR-106b-5p	DDI2	84301	ENSG00000197312	23313552	validated
mirtarbase	hsa-miR-192-5p	PGAP4	84302	ENSG00000165152	19074876	validated
mirtarbase	hsa-miR-21-5p	PYM1	84305	ENSG00000170473	19253296	validated
mirtarbase	hsa-miR-106b-5p	BRMS1L	84312	ENSG00000100916	17242205	validated
mirtarbase	hsa-miR-181a-5p	BRMS1L	84312	ENSG00000100916	17612493	validated
mirtarbase	hsa-miR-106b-5p	LZIC	84328	ENSG00000162441	23446348	validated
mirtarbase	hsa-miR-301a-3p	LZIC	84328	ENSG00000162441	23446348	validated
mirtarbase	hsa-miR-582-5p	LZIC	84328	ENSG00000162441	20371350	validated
mirtarbase	hsa-miR-144-3p	LZIC	84328	ENSG00000162441	20371350	validated
mirtarbase	hsa-miR-18a-5p	MCRIP2	84331	ENSG00000172366	23446348	validated
mirtarbase	hsa-miR-192-5p	PCGF5	84333	ENSG00000180628	19074876	validated
mirtarbase	hsa-miR-192-5p	HOOK3	84376	ENSG00000168172	19074876	validated

mirtarbase	hsa-miR-181a-5p	HOOK3	84376	ENSG00000168172	23622248	validated
mirtarbase	hsa-miR-106b-5p	HOOK3	84376	ENSG00000168172	23313552	validated
mirtarbase	hsa-miR-212-3p	HOOK3	84376	ENSG00000168172	22100165	validated
mirtarbase	hsa-miR-326	HOOK3	84376	ENSG00000168172	23824327	validated
mirtarbase	hsa-miR-301a-3p	MSANTD4	84437	ENSG00000170903	23446348	validated
mirtarbase	hsa-miR-15b-5p	MSANTD4	84437	ENSG00000170903	20371350	validated
mirtarbase	hsa-miR-144-3p	LCOR	84458	ENSG00000196233	23446348	validated
mirtarbase	hsa-miR-144-3p	MCM8	84515	ENSG00000125885	27418678	validated
mirtarbase	hsa-miR-15b-5p	DCTN5	84516	ENSG00000166847	22473208	validated
mirtarbase	hsa-miR-18a-5p	DCTN5	84516	ENSG00000166847	24398324	validated
mirtarbase	hsa-miR-106b-5p	DCTN5	84516	ENSG00000166847	23313552	validated
mirtarbase	hsa-miR-106b-5p	C15orf41	84529	ENSG00000186073	23824327	validated
mirtarbase	hsa-miR-192-5p	KIAA1841	84542	ENSG00000162929	19074876	validated
mirtarbase	hsa-miR-106b-5p	KIAA1841	84542	ENSG00000162929	23313552	validated
mirtarbase	hsa-miR-192-5p	MRPL43	84545	ENSG00000055950	19074876	validated
mirtarbase	hsa-miR-505-3p	TMEM185A	84548	ENSG00000269556	23622248	validated
mirtarbase	hsa-miR-222-3p	MAK16	84549	ENSG00000198042	23622248	validated
mirtarbase	hsa-miR-106b-5p	MAK16	84549	ENSG00000198042	22473208	validated
mirtarbase	hsa-miR-192-5p	FAXC	84553	ENSG00000146267	19074876	validated
mirtarbase	hsa-miR-15b-5p	FAXC	84553	ENSG00000146267	23622248	validated
mirtarbase	hsa-miR-106b-5p	FAXC	84553	ENSG00000146267	23824327	validated
mirtarbase	hsa-miR-18a-5p	PARD6B	84612	ENSG00000124171	20371350	validated
mirtarbase	hsa-miR-106b-5p	PARD6B	84612	ENSG00000124171	22473208	validated
mirtarbase	hsa-miR-106b-5p	ZBTB37	84614	ENSG00000185278	20371350	validated
mirtarbase	hsa-miR-222-3p	ZBTB37	84614	ENSG00000185278	22012620	validated
mirtarbase	hsa-miR-181a-5p	ZNF594	84622	ENSG00000180626	17612493	validated
mirtarbase	hsa-miR-192-5p	MFSD14B	84641	ENSG00000148110	19074876	validated
mirtarbase	hsa-miR-106b-5p	MYPN	84665	ENSG00000138347	23313552	validated
mirtarbase	hsa-miR-192-5p	FAM126A	84668	ENSG00000122591	19074876	validated
mirtarbase	hsa-miR-301a-3p	FAM126A	84668	ENSG00000122591	23622248	validated
mirtarbase	hsa-miR-106b-5p	USP32	84669	ENSG00000170832	22473208	validated
mirtarbase	hsa-miR-301a-3p	USP32	84669	ENSG00000170832	23824327	validated
mirtarbase	hsa-miR-106b-5p	ZNF347	84671	ENSG00000197937	23313552	validated
mirtarbase	hsa-miR-181a-5p	DSCR8	84677		17612493	validated
mirtarbase	hsa-miR-181a-5p	GTPBP3	84705	ENSG00000130299	20371350	validated
mirtarbase	hsa-miR-106b-5p	PIGO	84720	ENSG00000165282	22473208	validated
mirtarbase	hsa-miR-192-5p	PSRC1	84722	ENSG00000134222	19074876	validated
mirtarbase	hsa-miR-21-5p	PLEKHA8	84725	ENSG00000106086	23313552	validated
mirtarbase	hsa-miR-505-3p	PRRC2B	84726	ENSG00000130723	23622248	validated
mirtarbase	hsa-miR-181a-5p	PRRC2B	84726	ENSG00000130723	23622248	validated
mirtarbase	hsa-miR-222-3p	CBX2	84733	ENSG00000173894	23622248	validated
mirtarbase	hsa-miR-15b-5p	CBX2	84733	ENSG00000173894	20371350	validated
mirtarbase	hsa-miR-326	FAM167B	84734	ENSG00000183615	23824327	validated
mirtarbase	hsa-miR-505-3p	FUT10	84750	ENSG00000172728	23592263	validated
mirtarbase	hsa-miR-106b-5p	FUT10	84750	ENSG00000172728	23592263	validated
mirtarbase	hsa-miR-192-5p	LMNB2	84823	ENSG00000176619	19074876	validated
mirtarbase	hsa-miR-18a-5p	LMNB2	84823	ENSG00000176619	20371350	validated
mirtarbase	hsa-miR-144-3p	LMNB2	84823	ENSG00000176619	23592263	validated
mirtarbase	hsa-miR-106b-5p	ZNF514	84874	ENSG00000144026	23446348	validated
mirtarbase	hsa-miR-106b-5p	ORAI1	84876	ENSG00000276045	22473208	validated
mirtarbase	hsa-miR-106b-5p	MFSD2A	84879	ENSG00000168389	23824327	validated
mirtarbase	hsa-miR-15b-5p	AIFM2	84883	ENSG00000042286	23622248	validated
mirtarbase	hsa-miR-192-5p	C1orf198	84886	ENSG00000119280	19074876	validated
mirtarbase	hsa-miR-301a-3p	MIGA2	84895	ENSG00000148343	23592263	validated
mirtarbase	hsa-miR-181a-5p	MIGA2	84895	ENSG00000148343	23592263	validated
mirtarbase	hsa-miR-192-5p	ATAD1	84896	ENSG00000138138	19074876	validated
mirtarbase	hsa-miR-15b-5p	TBRG1	84897	ENSG00000154144	21572407	validated
mirtarbase	hsa-miR-181a-5p	PLXDC2	84898	ENSG00000120594	17612493	validated
mirtarbase	hsa-miR-192-5p	TMTC4	84899	ENSG00000125247	19074876	validated
mirtarbase	hsa-miR-106b-5p	NFATC2IP	84901	ENSG00000176953	22473208	validated
mirtarbase	hsa-miR-582-3p	ARHGEF39	84904	ENSG00000137135	23824327	validated
mirtarbase	hsa-miR-192-5p	FAM136A	84908	ENSG00000035141	20371350	validated
mirtarbase	hsa-miR-21-5p	FAM136A	84908	ENSG00000035141	23592263	validated
mirtarbase	hsa-miR-181a-5p	SLC35B4	84912	ENSG00000205060	20371350	validated
mirtarbase	hsa-miR-192-5p	SLC35B4	84912	ENSG00000205060	19074876	validated
mirtarbase	hsa-miR-21-5p	ZNF587	84914	ENSG00000198466	18591254	validated
mirtarbase	hsa-miR-106b-5p	ZNF587	84914	ENSG00000198466	23622248	validated
mirtarbase	hsa-miR-192-5p	FAM222A	84915	ENSG00000139438	19074876	validated
mirtarbase	hsa-miR-106b-5p	PPP1R15B	84919	ENSG00000158615	24398324	validated
mirtarbase	hsa-miR-301a-3p	PPP1R15B	84919	ENSG00000158615	24398324	validated
mirtarbase	hsa-miR-582-5p	PPP1R15B	84919	ENSG00000158615	23592263	validated

mirtarbase	hsa-miR-192-5p	ALG10	84920	ENSG00000139133	19074876	validated
mirtarbase	hsa-miR-18a-5p	FAM104A	84923	ENSG00000133193	23622248	validated
mirtarbase	hsa-miR-106b-5p	MASTL	84930	ENSG00000120539	22473208	validated
mirtarbase	hsa-miR-301a-3p	MASTL	84930	ENSG00000120539	23824327	validated
mirtarbase	hsa-miR-181a-5p	RAB2B	84932	ENSG00000129472	22473208	validated
mirtarbase	hsa-miR-15b-5p	ZNRF1	84937	ENSG00000186187	23622248	validated
mirtarbase	hsa-miR-106b-5p	WDR73	84942	ENSG00000177082	23706177	validated
mirtarbase	hsa-miR-192-5p	SERAC1	84947	ENSG00000122335	19074876	validated
mirtarbase	hsa-miR-21-5p	SERAC1	84947	ENSG00000122335	18591254	validated
mirtarbase	hsa-miR-192-5p	PRPF38A	84950	ENSG00000134748	19074876	validated
mirtarbase	hsa-miR-582-3p	PRPF38A	84950	ENSG00000134748	23824327	validated
mirtarbase	hsa-miR-301a-3p	PRPF38A	84950	ENSG00000134748	27292025	validated
mirtarbase	hsa-miR-192-5p	CGNL1	84952	ENSG00000128849	19074876	validated
mirtarbase	hsa-miR-505-3p	MICAL2	84953	ENSG00000133816	23824327	validated
mirtarbase	hsa-miR-15b-5p	RELT	84957	ENSG00000054967	22473208	validated
mirtarbase	hsa-miR-192-5p	UBASH3B	84959	ENSG00000154127	19074876	validated
mirtarbase	hsa-miR-15b-5p	FBXL20	84961	ENSG00000108306	21572407	validated
mirtarbase	hsa-miR-326	LSM10	84967	ENSG00000181817	19536157	validated
mirtarbase	hsa-miR-192-5p	CEP19	84984	ENSG00000174007	19074876	validated
mirtarbase	hsa-miR-192-5p	ARHGAP19	84986	ENSG00000213390	19074876	validated
mirtarbase	hsa-miR-192-5p	USP45	85015	ENSG00000123552	19074876	validated
mirtarbase	hsa-miR-181a-5p	COL27A1	85301	ENSG00000196739	17612493	validated
mirtarbase	hsa-miR-326	PAQR8	85315	ENSG00000170915	23622248	validated
mirtarbase	hsa-miR-106b-5p	BAGE5	85316		22473208	validated
mirtarbase	hsa-miR-15b-5p	ZCCHC3	85364	ENSG00000247315	24398324	validated
mirtarbase	hsa-miR-21-5p	ZCCHC3	85364	ENSG00000247315	22473208	validated
mirtarbase	hsa-miR-296-5p	ZCCHC3	85364	ENSG00000247315	26701625	validated
mirtarbase	hsa-miR-326	KIAA1671	85379	ENSG00000197077	23622248	validated
mirtarbase	hsa-miR-192-5p	KIAA1671	85379	ENSG00000197077	23622248	validated
mirtarbase	hsa-miR-192-5p	EAF1	85403	ENSG00000144597	19074876	validated
mirtarbase	hsa-miR-192-5p	ZIC5	85416	ENSG00000139800	19074876	validated
mirtarbase	hsa-miR-301a-3p	ZIC5	85416	ENSG00000139800	21572407	validated
mirtarbase	hsa-miR-21-5p	DOCK7	85440	ENSG00000116641	22158624	validated
mirtarbase	hsa-miR-192-5p	LRRCC1	85444	ENSG00000133739	19074876	validated
mirtarbase	hsa-miR-106b-5p	UNK	85451	ENSG00000132478	22012620	validated
mirtarbase	hsa-miR-582-3p	DIXDC1	85458	ENSG00000150764	23295946	validated
mirtarbase	hsa-miR-582-5p	DIXDC1	85458	ENSG00000150764	23295946	validated
mirtarbase	hsa-miR-192-5p	CEP295	85459	ENSG00000166004	19074876	validated
mirtarbase	hsa-miR-106b-5p	TANC1	85461	ENSG00000115183	22473208	validated
mirtarbase	hsa-miR-192-5p	FHDC1	85462	ENSG00000137460	19074876	validated
mirtarbase	hsa-miR-106b-5p	FHDC1	85462	ENSG00000137460	22012620	validated
mirtarbase	hsa-miR-192-5p	ZC3H12C	85463	ENSG00000149289	19074876	validated
mirtarbase	hsa-miR-106b-5p	ZC3H12C	85463	ENSG00000149289	23446348	validated
mirtarbase	hsa-miR-222-3p	SSH2	85464	ENSG00000141298	23622248	validated
mirtarbase	hsa-miR-106b-5p	SSH2	85464	ENSG00000141298	22473208	validated
mirtarbase	hsa-miR-15b-5p	SELENOI	85465	ENSG00000138018	22473208	validated
mirtarbase	hsa-miR-582-3p	SELENOI	85465	ENSG00000138018	23313552	validated
mirtarbase	hsa-miR-192-5p	ANKRD13A	88455	ENSG00000076513	19074876	validated
mirtarbase	hsa-miR-505-3p	TRIM4	89122	ENSG00000146833	22012620	validated
mirtarbase	hsa-miR-301a-3p	TRIM4	89122	ENSG00000146833	21572407	validated
mirtarbase	hsa-miR-181a-5p	WNT3A	89780	ENSG00000154342	17612493	validated
mirtarbase	hsa-miR-192-5p	LMLN	89782	ENSG00000185621	19074876	validated
mirtarbase	hsa-miR-301a-3p	LMLN	89782	ENSG00000185621	23446348	validated
mirtarbase	hsa-miR-21-5p	NAV3	89795	ENSG00000067798	25579119	validated
mirtarbase	hsa-miR-181a-5p	CHRFAM7A	89832	ENSG00000166664	17612493	validated
mirtarbase	hsa-miR-21-5p	KBTBD6	89890	ENSG00000165572	18591254	validated
mirtarbase	hsa-miR-505-3p	KBTBD6	89890	ENSG00000165572	23446348	validated
mirtarbase	hsa-miR-301a-3p	KBTBD6	89890	ENSG00000165572	19536157	validated
mirtarbase	hsa-miR-15b-5p	UBE3B	89910	ENSG00000151148	23622248	validated
mirtarbase	hsa-miR-301a-3p	BMERB1	89927	ENSG00000278823	23824327	validated
mirtarbase	hsa-miR-222-3p	SAPCD2	89958	ENSG00000186193	23622248	validated
mirtarbase	hsa-miR-21-5p	RSPRY1	89970	ENSG00000159579	18591254	validated
mirtarbase	hsa-miR-222-3p	MIDN	90007	ENSG00000167470	20371350	validated
mirtarbase	hsa-miR-106b-5p	MIDN	90007	ENSG00000167470	22473208	validated
mirtarbase	hsa-miR-192-5p	EFCAB11	90141	ENSG00000140025	19074876	validated
mirtarbase	hsa-miR-106b-5p	EFCAB11	90141	ENSG00000140025	23446348	validated
mirtarbase	hsa-miR-181a-5p	ZNF616	90317	ENSG00000204611	20371350	validated
mirtarbase	hsa-miR-192-5p	C5orf30	90355	ENSG00000181751	19074876	validated
mirtarbase	hsa-miR-192-5p	TICRR	90381	ENSG00000140534	19074876	validated
mirtarbase	hsa-miR-505-3p	MCFD2	90411	ENSG00000180398	23313552	validated
mirtarbase	hsa-miR-15b-5p	MCFD2	90411	ENSG00000180398	23313552	validated

mirtarbase	hsa-miR-222-3p	KNSTRN	90417	ENSG00000128944	23622248	validated
mirtarbase	hsa-miR-222-3p	BMF	90427	ENSG00000104081	26370254	validated
mirtarbase	hsa-miR-15b-5p	ZNF622	90441	ENSG00000173545	22473208	validated
mirtarbase	hsa-miR-181a-5p	ZNF439	90594	ENSG00000171291	21572407	validated
mirtarbase	hsa-miR-192-5p	LYRM7	90624	ENSG00000186687	19074876	validated
mirtarbase	hsa-miR-21-5p	LYRM7	90624	ENSG00000186687	18591254	validated
mirtarbase	hsa-miR-301a-3p	STARD13	90627	ENSG00000133121	24398324	validated
mirtarbase	hsa-miR-106b-5p	COX19	90639	ENSG00000240230	23446348	validated
mirtarbase	hsa-miR-15b-5p	PIP4P1	90809	ENSG00000165782	22473208	validated
mirtarbase	hsa-miR-106b-5p	ZNF598	90850	ENSG00000167962	23622248	validated
mirtarbase	hsa-miR-15b-5p	JPT2	90861	ENSG00000206053	27292025	validated
mirtarbase	hsa-miR-106b-5p	TRIM41	90933	ENSG00000146063	26701625	validated
mirtarbase	hsa-miR-296-5p	TRIM41	90933	ENSG00000146063	26701625	validated
mirtarbase	hsa-miR-106b-5p	FMNL3	91010	ENSG00000161791	22473208	validated
mirtarbase	hsa-miR-106b-5p	DPP9	91039	ENSG00000142002	17242205	validated
mirtarbase	hsa-miR-21-5p	CCDC34	91057	ENSG00000109881	18591254	validated
mirtarbase	hsa-miR-106b-5p	ZNF682	91120	ENSG00000197124	23446348	validated
mirtarbase	hsa-miR-106b-5p	SLC25A46	91137	ENSG00000164209	21572407	validated
mirtarbase	hsa-miR-106b-5p	TMEM67	91147	ENSG00000164953	22473208	validated
mirtarbase	hsa-miR-181a-5p	C12orf29	91298	ENSG00000133641	22815788	validated
mirtarbase	hsa-miR-106b-5p	CDKN2AIPNL	91368	ENSG00000237190	23313552	validated
mirtarbase	hsa-miR-15b-5p	CDKN2AIPNL	91368	ENSG00000237190	22473208	validated
mirtarbase	hsa-miR-21-5p	SESTD1	91404	ENSG00000187231	18591254	validated
mirtarbase	hsa-miR-15b-5p	SESTD1	91404	ENSG00000187231	23446348	validated
mirtarbase	hsa-miR-192-5p	BTF3L4	91408	ENSG00000134717	19074876	validated
mirtarbase	hsa-miR-301a-3p	BTF3L4	91408	ENSG00000134717	23592263	validated
mirtarbase	hsa-miR-106b-5p	BTF3L4	91408	ENSG00000134717	23446348	validated
mirtarbase	hsa-miR-106b-5p	RCCD1	91433	ENSG00000166965	23622248	validated
mirtarbase	hsa-miR-106b-5p	FAAP24	91442	ENSG00000131944	23313552	validated
mirtarbase	hsa-miR-21-5p	RNF185	91445	ENSG00000138942	23592263	validated
mirtarbase	hsa-miR-21-5p	ACBD5	91452	ENSG00000107897	20371350	validated
mirtarbase	hsa-miR-106b-5p	ACBD5	91452	ENSG00000107897	22473208	validated
mirtarbase	hsa-miR-301a-3p	ACBD5	91452	ENSG00000107897	21572407	validated
mirtarbase	hsa-miR-192-5p	C12orf65	91574	ENSG00000130921	19074876	validated
mirtarbase	hsa-miR-222-3p	C12orf65	91574	ENSG00000130921	23622248	validated
mirtarbase	hsa-miR-106b-5p	C12orf65	91574	ENSG00000130921	23313552	validated
mirtarbase	hsa-miR-222-3p	SLFN11	91607	ENSG00000172716	23622248	validated
mirtarbase	hsa-miR-106b-5p	CHURC1	91612	ENSG00000258289	22473208	validated
mirtarbase	hsa-miR-21-5p	BOC	91653	ENSG00000144857	18591254	validated
mirtarbase	hsa-miR-505-3p	ZNF845	91664	ENSG00000213799	23313552	validated
mirtarbase	hsa-miR-15b-5p	SMDT1	91689	ENSG00000272835	20371350	validated
mirtarbase	hsa-miR-301a-3p	LONRF1	91694	ENSG00000154359	23622248	validated
mirtarbase	hsa-miR-181a-5p	LONRF1	91694	ENSG00000154359	22473208	validated
mirtarbase	hsa-miR-15b-5p	YTHDC1	91746	ENSG00000275272	23446348	validated
mirtarbase	hsa-miR-106b-5p	YTHDC1	91746	ENSG00000275272	19536157	validated
mirtarbase	hsa-miR-106b-5p	ELMSAN1	91748	ENSG00000156030	22473208	validated
mirtarbase	hsa-miR-181a-5p	ELMSAN1	91748	ENSG00000156030	22473208	validated
mirtarbase	hsa-miR-192-5p	NEK9	91754	ENSG00000119638	23824327	validated
mirtarbase	hsa-miR-106b-5p	CABLES1	91768	ENSG00000134508	23592263	validated
mirtarbase	hsa-miR-15b-5p	CHMP7	91782	ENSG00000147457	23622248	validated
mirtarbase	hsa-miR-106b-5p	MYLK3	91807	ENSG00000140795	23313552	validated
mirtarbase	hsa-miR-301a-3p	RFT1	91869	ENSG00000163933	23446348	validated
mirtarbase	hsa-miR-21-5p	FDXACB1	91893	ENSG00000255561	27292025	validated
mirtarbase	hsa-miR-181a-5p	CCNQ	92002	ENSG00000262919	22473208	validated
mirtarbase	hsa-miR-192-5p	SLC25A51	92014	ENSG00000122696	19074876	validated
mirtarbase	hsa-miR-222-3p	SLC25A51	92014	ENSG00000122696	23622248	validated
mirtarbase	hsa-miR-181a-5p	ZC3HAV1L	92092	ENSG00000146858	23824327	validated
mirtarbase	hsa-miR-192-5p	MTDH	92140	ENSG00000147649	19074876	validated
mirtarbase	hsa-miR-296-5p	MTSS2	92154	ENSG00000132613	23592263	validated
mirtarbase	hsa-miR-18a-5p	UBTD2	92181	ENSG00000168246	20371350	validated
mirtarbase	hsa-miR-18a-5p	ZNF585B	92285	ENSG00000245680	22012620	validated
mirtarbase	hsa-miR-15b-5p	ZNF585B	92285	ENSG00000245680	21572407	validated
mirtarbase	hsa-miR-301a-3p	ASB16	92591	ENSG00000161664	21572407	validated
mirtarbase	hsa-miR-106b-5p	ASB16	92591	ENSG00000161664	23313552	validated
mirtarbase	hsa-miR-181a-5p	MOB1B	92597	ENSG00000173542	20371350	validated
mirtarbase	hsa-miR-222-3p	TIMM50	92609	ENSG00000105197	23622248	validated
mirtarbase	hsa-miR-18a-5p	TIMM50	92609	ENSG00000105197	26701625	validated
mirtarbase	hsa-miR-301a-3p	TIMM50	92609	ENSG00000105197	27292025	validated
mirtarbase	hsa-miR-192-5p	TIFA	92610	ENSG00000145365	19074876	validated
mirtarbase	hsa-miR-301a-3p	FAM114A1	92689	ENSG00000197712	19536157	validated
mirtarbase	hsa-miR-192-5p	TMEM169	92691	ENSG00000163449	19074876	validated

mirtarbase	hsa-miR-326	ARRDC1	92714	ENSG00000197070	23622248	validated
mirtarbase	hsa-miR-106b-5p	UBE2Q2	92912	ENSG00000140367	22473208	validated
mirtarbase	hsa-miR-15b-5p	UBE2Q2	92912	ENSG00000140367	22473208	validated
mirtarbase	hsa-miR-21-5p	ZBTB47	92999	ENSG00000114853	18591254	validated
mirtarbase	hsa-miR-18a-5p	ZBTB47	92999	ENSG00000114853	23446348	validated
mirtarbase	hsa-miR-192-5p	TEX30	93081	ENSG00000151287	19074876	validated
mirtarbase	hsa-miR-18a-5p	ORAI3	93129	ENSG00000175938	23622248	validated
mirtarbase	hsa-miR-192-5p	PIGM	93183	ENSG00000143315	20371350	validated
mirtarbase	hsa-miR-222-3p	HAUS8	93323	ENSG00000131351	23622248	validated
mirtarbase	hsa-miR-106b-5p	HAUS8	93323	ENSG00000131351	22473208	validated
mirtarbase	hsa-miR-301a-3p	MMGT1	93380	ENSG00000169446	23592263	validated
mirtarbase	hsa-miR-18a-5p	ZNF670	93474	ENSG00000277462	20371350	validated
mirtarbase	hsa-miR-181a-5p	MAPK1IP1L	93487	ENSG00000168175	22942087	validated
mirtarbase	hsa-miR-192-5p	TBC1D31	93594	ENSG00000156787	19074876	validated
mirtarbase	hsa-miR-15b-5p	TADA2B	93624	ENSG00000173011	22473208	validated
mirtarbase	hsa-miR-106b-5p	TADA2B	93624	ENSG00000173011	22473208	validated
mirtarbase	hsa-miR-212-3p	TJAP1	93643	ENSG00000137221	21572407	validated
mirtarbase	hsa-miR-192-5p	CADPS2	93664	ENSG00000081803	19074876	validated
mirtarbase	hsa-miR-181a-5p	CADPS2	93664	ENSG00000081803	27292025	validated
mirtarbase	hsa-miR-192-5p	TTYH2	94015	ENSG00000141540	27292025	validated
mirtarbase	hsa-miR-106b-5p	CAMK2N2	94032	ENSG00000163888	22473208	validated
mirtarbase	hsa-miR-582-3p	LENG9	94059	ENSG00000273574	23824327	validated
mirtarbase	hsa-miR-21-5p	SFXN1	94081	ENSG00000164466	18591254	validated
mirtarbase	hsa-miR-106b-5p	ORMDL3	94103	ENSG00000172057	22473208	validated
mirtarbase	hsa-miR-181a-5p	ARHGAP12	94134	ENSG00000165322	20371350	validated
mirtarbase	hsa-miR-106b-5p	ARHGAP12	94134	ENSG00000165322	22473208	validated
mirtarbase	hsa-miR-301a-3p	ARHGAP12	94134	ENSG00000165322	21572407	validated
mirtarbase	hsa-miR-15b-5p	ARHGAP12	94134	ENSG00000165322	22473208	validated
mirtarbase	hsa-miR-301a-3p	FOXQ1	94234	ENSG00000164379	23592263	validated
mirtarbase	hsa-miR-106b-5p	FOXQ1	94234	ENSG00000164379	23592263	validated
mirtarbase	hsa-miR-106b-5p	TP53INP1	94241	ENSG00000164938	22100165	validated
mirtarbase	hsa-miR-582-3p	MYL12B	103910	ENSG00000118680	20371350	validated
mirtarbase	hsa-miR-106b-5p	EGLN3	112399	ENSG00000129521	21572407	validated
mirtarbase	hsa-miR-301a-3p	EGLN3	112399	ENSG00000129521	21572407	validated
mirtarbase	hsa-miR-21-5p	MRAP2	112609	ENSG00000135324	18591254	validated
mirtarbase	hsa-miR-192-5p	FAM71E1	112703	ENSG00000142530	19074876	validated
mirtarbase	hsa-miR-106b-5p	WDR89	112840	ENSG00000140006	22473208	validated
mirtarbase	hsa-miR-582-5p	NACC1	112939	ENSG00000160877	23592263	validated
mirtarbase	hsa-miR-144-3p	NACC1	112939	ENSG00000160877	23592263	validated
mirtarbase	hsa-miR-18a-5p	NACC1	112939	ENSG00000160877	23592263	validated
mirtarbase	hsa-miR-301a-3p	MED8	112950	ENSG00000159479	19536157	validated
mirtarbase	hsa-miR-18a-5p	CDCA5	113130	ENSG00000146670	23622248	validated
mirtarbase	hsa-miR-15b-5p	AHNAK2	113146	ENSG00000185567	20371350	validated
mirtarbase	hsa-miR-15b-5p	SCAMP4	113178	ENSG00000227500	20371350	validated
mirtarbase	hsa-miR-301a-3p	SLC46A1	113235	ENSG00000076351	23446348	validated
mirtarbase	hsa-miR-192-5p	GLCCI1	113263	ENSG00000106415	19074876	validated
mirtarbase	hsa-miR-192-5p	TMEM106A	113277	ENSG00000184988	19074876	validated
mirtarbase	hsa-miR-106b-5p	SLC52A3	113278	ENSG00000101276	23622248	validated
mirtarbase	hsa-miR-15b-5p	SFT2D1	113402	ENSG00000198818	23622248	validated
mirtarbase	hsa-miR-15b-5p	MCM3AP-AS1	114044	ENSG00000215424	22473208	validated
mirtarbase	hsa-miR-181a-5p	SLC25A25	114789	ENSG00000148339	23592263	validated
mirtarbase	hsa-miR-106b-5p	STK11IP	114790	ENSG00000144589	21572407	validated
mirtarbase	hsa-miR-21-5p	TUBGCP5	114791	ENSG00000275835	18591254	validated
mirtarbase	hsa-miR-106b-5p	FMNL2	114793	ENSG00000157827	21572407	validated
mirtarbase	hsa-miR-181a-5p	TMEM132B	114795	ENSG00000139364	20371350	validated
mirtarbase	hsa-miR-505-3p	TMEM132B	114795	ENSG00000139364	23824327	validated
mirtarbase	hsa-miR-192-5p	TMEM200A	114801	ENSG00000164484	19074876	validated
mirtarbase	hsa-miR-18a-5p	RNF157	114804	ENSG00000141576	23622248	validated
mirtarbase	hsa-miR-21-5p	OSBPL1A	114876	ENSG00000141447	18591254	validated
mirtarbase	hsa-miR-192-5p	OSBPL6	114880	ENSG00000079156	19074876	validated
mirtarbase	hsa-miR-192-5p	OSBPL8	114882	ENSG00000091039	19074876	validated
mirtarbase	hsa-miR-192-5p	OSBPL10	114884	ENSG00000144645	19074876	validated
mirtarbase	hsa-miR-222-3p	OSBPL10	114884	ENSG00000144645	23622248	validated
mirtarbase	hsa-miR-192-5p	C1QTNF3	114899	ENSG00000082196	19074876	validated
mirtarbase	hsa-miR-106b-5p	TMEM123	114908	ENSG00000152558	22473208	validated
mirtarbase	hsa-miR-301a-3p	FLYWCH2	114984	ENSG00000162076	23824327	validated
mirtarbase	hsa-miR-301a-3p	WDR31	114987	ENSG00000148225	23824327	validated
mirtarbase	hsa-miR-192-5p	ATG4A	115201	ENSG00000101844	19074876	validated
mirtarbase	hsa-miR-192-5p	KCTD12	115207	ENSG00000178695	19074876	validated
mirtarbase	hsa-miR-296-5p	KCTD12	115207	ENSG00000178695	23446348	validated
mirtarbase	hsa-miR-106b-5p	RAB42	115273	ENSG00000188060	23706177	validated

mirtarbase	hsa-miR-106b-5p	PCMTD1	115294	ENSG00000168300	22473208	validated
mirtarbase	hsa-miR-222-3p	UHRF2	115426	ENSG00000147854	23622248	validated
mirtarbase	hsa-miR-326	ZNF689	115509	ENSG00000156853	23622248	validated
mirtarbase	hsa-miR-106b-5p	FCHO2	115548	ENSG00000157107	22473208	validated
mirtarbase	hsa-miR-192-5p	DIS3L	115752	ENSG00000166938	19074876	validated
mirtarbase	hsa-miR-106b-5p	DIS3L	115752	ENSG00000166938	21572407	validated
mirtarbase	hsa-miR-192-5p	LRRC58	116064	ENSG00000163428	19074876	validated
mirtarbase	hsa-miR-106b-5p	LRRC58	116064	ENSG00000163428	27418678	validated
mirtarbase	hsa-miR-181a-5p	LYSMD3	116068	ENSG00000176018	17612493	validated
mirtarbase	hsa-miR-192-5p	LYSMD3	116068	ENSG00000176018	19074876	validated
mirtarbase	hsa-miR-106b-5p	LYSMD3	116068	ENSG00000176018	22473208	validated
mirtarbase	hsa-miR-144-3p	LYSMD3	116068	ENSG00000176018	23592263	validated
mirtarbase	hsa-miR-106b-5p	WDR92	116143	ENSG00000243667	27418678	validated
mirtarbase	hsa-miR-301a-3p	NUS1	116150	ENSG00000153989	22291592	validated
mirtarbase	hsa-miR-144-3p	NUS1	116150	ENSG00000153989	23592263	validated
mirtarbase	hsa-miR-21-5p	COX20	116228	ENSG00000203667	23446348	validated
mirtarbase	hsa-miR-301a-3p	COX20	116228	ENSG00000203667	22012620	validated
mirtarbase	hsa-miR-106b-5p	ABHD15	116236	ENSG00000168792	22012620	validated
mirtarbase	hsa-miR-192-5p	GINM1	116254	ENSG00000055211	19074876	validated
mirtarbase	hsa-miR-192-5p	RBP7	116362	ENSG00000162444	19074876	validated
mirtarbase	hsa-miR-144-3p	RAB39B	116442	ENSG00000155961	21572407	validated
mirtarbase	hsa-miR-106b-5p	NIBAN1	116496	ENSG00000135842	22473208	validated
mirtarbase	hsa-miR-106b-5p	SNAP47	116841	ENSG00000143740	20371350	validated
mirtarbase	hsa-miR-192-5p	LEAP2	116842	ENSG00000164406	19074876	validated
mirtarbase	hsa-miR-18a-5p	ACAP3	116983	ENSG00000131584	23622248	validated
mirtarbase	hsa-miR-106b-5p	ARAP2	116984	ENSG00000047365	22473208	validated
mirtarbase	hsa-miR-582-5p	ARAP2	116984	ENSG00000047365	23592263	validated
mirtarbase	hsa-miR-144-3p	ARAP2	116984	ENSG00000047365	23592263	validated
mirtarbase	hsa-miR-106b-5p	THEM4	117145	ENSG00000159445	23313552	validated
mirtarbase	hsa-miR-15b-5p	RAB3IP	117177	ENSG00000127328	20371350	validated
mirtarbase	hsa-miR-106b-5p	RAB3IP	117177	ENSG00000127328	26701625	validated
mirtarbase	hsa-miR-222-3p	SSX2IP	117178	ENSG00000117155	23776679	validated
mirtarbase	hsa-miR-106b-5p	SSX2IP	117178	ENSG00000117155	22473208	validated
mirtarbase	hsa-miR-181a-5p	SSX2IP	117178	ENSG00000117155	22473208	validated
mirtarbase	hsa-miR-21-5p	RFFL	117584	ENSG00000092871	22815788	validated
mirtarbase	hsa-miR-301a-3p	ZNF354B	117608	ENSG00000178338	23446348	validated
mirtarbase	hsa-miR-106b-5p	ZNF354B	117608	ENSG00000178338	20371350	validated
mirtarbase	hsa-miR-181a-5p	PRAP1	118471	ENSG00000165828	21779487	validated
mirtarbase	hsa-miR-505-3p	ZNF511	118472	ENSG00000198546	23622248	validated
mirtarbase	hsa-miR-106b-5p	PIK3AP1	118788	ENSG00000155629	23622248	validated
mirtarbase	hsa-miR-181a-5p	FRA10AC1	118924	ENSG00000148690	17612493	validated
mirtarbase	hsa-miR-192-5p	SFR1	119392	ENSG00000156384	19074876	validated
mirtarbase	hsa-miR-144-3p	SFXN4	119559	ENSG00000183605	23592263	validated
mirtarbase	hsa-miR-582-3p	LRRK2	120892	ENSG00000188906	23295946	validated
mirtarbase	hsa-miR-582-5p	LRRK2	120892	ENSG00000188906	23295946	validated
mirtarbase	hsa-miR-18a-5p	LRIG3	121227	ENSG00000139263	23622248	validated
mirtarbase	hsa-miR-192-5p	ZNF641	121274	ENSG00000167528	19074876	validated
mirtarbase	hsa-miR-192-5p	NEDD1	121441	ENSG00000139350	19074876	validated
mirtarbase	hsa-miR-21-5p	SPPL3	121665	ENSG00000157837	22473208	validated
mirtarbase	hsa-miR-301a-3p	ANKRD9	122416	ENSG00000156381	23824327	validated
mirtarbase	hsa-miR-106b-5p	C14orf28	122525	ENSG00000179476	22473208	validated
mirtarbase	hsa-miR-301a-3p	MRPL52	122704	ENSG00000172590	23592263	validated
mirtarbase	hsa-miR-106b-5p	FRMD6	122786	ENSG00000139926	22473208	validated
mirtarbase	hsa-miR-21-5p	SOCS4	122809	ENSG00000180008	18591254	validated
mirtarbase	hsa-miR-21-5p	NAA30	122830	ENSG00000139977	18591254	validated
mirtarbase	hsa-miR-15b-5p	NAA30	122830	ENSG00000139977	23622248	validated
mirtarbase	hsa-miR-144-3p	NAA30	122830	ENSG00000139977	21572407	validated
mirtarbase	hsa-miR-326	NAA30	122830	ENSG00000139977	23313552	validated
mirtarbase	hsa-miR-106b-5p	ISCA2	122961	ENSG00000165898	23313552	validated
mirtarbase	hsa-miR-15b-5p	SLC25A29	123096	ENSG00000197119	22473208	validated
mirtarbase	hsa-miR-106b-5p	C15orf40	123207	ENSG00000169609	23824327	validated
mirtarbase	hsa-miR-296-5p	MTFMT	123263	ENSG00000103707	23592263	validated
mirtarbase	hsa-miR-192-5p	NIPA1	123606	ENSG00000170113	19074876	validated
mirtarbase	hsa-miR-106b-5p	NIPA1	123606	ENSG00000170113	22473208	validated
mirtarbase	hsa-miR-301a-3p	NIPA1	123606	ENSG00000170113	23446348	validated
mirtarbase	hsa-miR-582-3p	FSD2	123722	ENSG00000186628	27418678	validated
mirtarbase	hsa-miR-192-5p	C16orf46	123775	ENSG00000166455	19074876	validated
mirtarbase	hsa-miR-18a-5p	DCUN1D3	123879	ENSG00000188215	20371350	validated
mirtarbase	hsa-miR-301a-3p	DCUN1D3	123879	ENSG00000188215	20371350	validated
mirtarbase	hsa-miR-192-5p	NOXO1	124056	ENSG00000196408	19074876	validated
mirtarbase	hsa-miR-106b-5p	ZC3H18	124245	ENSG00000158545	23622248	validated

mirtarbase	hsa-miR-18a-5p	ZC3H18	124245	ENSG00000158545	23622248	validated
mirtarbase	hsa-miR-21-5p	TMEM170A	124491	ENSG00000166822	21572407	validated
mirtarbase	hsa-miR-326	USH1G	124590	ENSG00000182040	24398324	validated
mirtarbase	hsa-miR-301a-3p	CYB5D1	124637	ENSG00000182224	23824327	validated
mirtarbase	hsa-miR-326	SEZ6	124925	ENSG00000063015	27418678	validated
mirtarbase	hsa-miR-505-3p	ANKRD13B	124930	ENSG00000198720	23622248	validated
mirtarbase	hsa-miR-15b-5p	ANKRD13B	124930	ENSG00000198720	22473208	validated
mirtarbase	hsa-miR-505-3p	GJD3	125111	ENSG00000183153	21572407	validated
mirtarbase	hsa-miR-106b-5p	FAM210A	125228	ENSG00000177150	22473208	validated
mirtarbase	hsa-miR-192-5p	KRT18P8	125242		19074876	validated
mirtarbase	hsa-miR-15b-5p	OSCAR	126014	ENSG00000275736	21572407	validated
mirtarbase	hsa-miR-296-5p	OSCAR	126014	ENSG00000275736	23313552	validated
mirtarbase	hsa-miR-181a-5p	ZNF440	126070	ENSG00000171295	23446348	validated
mirtarbase	hsa-miR-106b-5p	TNFAIP8L1	126282	ENSG00000185361	22012620	validated
mirtarbase	hsa-miR-18a-5p	IRGQ	126298	ENSG00000167378	23622248	validated
mirtarbase	hsa-miR-192-5p	GABPB2	126626	ENSG00000143458	19074876	validated
mirtarbase	hsa-miR-326	RPTN	126638	ENSG00000215853	19536157	validated
mirtarbase	hsa-miR-192-5p	CCSAP	126731	ENSG00000154429	19074876	validated
mirtarbase	hsa-miR-21-5p	TPRG1L	127262	ENSG00000158109	18591254	validated
mirtarbase	hsa-miR-106b-5p	TPRG1L	127262	ENSG00000158109	22473208	validated
mirtarbase	hsa-miR-505-3p	TPRG1L	127262	ENSG00000158109	21572407	validated
mirtarbase	hsa-miR-21-5p	TCEANC2	127428	ENSG00000116205	23824327	validated
mirtarbase	hsa-miR-106b-5p	RNF19B	127544	ENSG00000116514	23313552	validated
mirtarbase	hsa-miR-296-5p	ARL8A	127829	ENSG00000143862	23592263	validated
mirtarbase	hsa-miR-296-5p	SYT2	127833	ENSG00000143858	23592263	validated
mirtarbase	hsa-miR-15b-5p	TBC1D20	128637	ENSG00000125875	22473208	validated
mirtarbase	hsa-miR-15b-5p	CHMP4B	128866	ENSG00000101421	20371350	validated
mirtarbase	hsa-miR-106b-5p	NUP35	129401	ENSG00000163002	22473208	validated
mirtarbase	hsa-miR-106b-5p	XIRP2	129446	ENSG00000163092	22012620	validated
mirtarbase	hsa-miR-505-3p	TYW5	129450	ENSG00000162971	23824327	validated
mirtarbase	hsa-miR-192-5p	CMPK2	129607	ENSG00000134326	19074876	validated
mirtarbase	hsa-miR-106b-5p	ICA1L	130026	ENSG00000163596	23824327	validated
mirtarbase	hsa-miR-21-5p	OSR1	130497	ENSG00000143867	18591254	validated
mirtarbase	hsa-miR-21-5p	UBR3	130507	ENSG00000144357	18591254	validated
mirtarbase	hsa-miR-15b-5p	UBR3	130507	ENSG00000144357	21572407	validated
mirtarbase	hsa-miR-192-5p	LYPD6	130574	ENSG00000187123	19074876	validated
mirtarbase	hsa-miR-192-5p	AHSA2	130872		19074876	validated
mirtarbase	hsa-miR-21-5p	AHSA2	130872		18591254	validated
mirtarbase	hsa-miR-192-5p	FBXO36	130888	ENSG00000153832	19074876	validated
mirtarbase	hsa-miR-192-5p	DNAJC19	131118	ENSG00000205981	19074876	validated
mirtarbase	hsa-miR-15b-5p	KLHL40	131377	ENSG00000157119	23824327	validated
mirtarbase	hsa-miR-301a-3p	TRIM71	131405	ENSG00000206557	22012620	validated
mirtarbase	hsa-miR-296-5p	CHCHD4	131474	ENSG00000163528	19536157	validated
mirtarbase	hsa-miR-192-5p	CRYBG3	131544	ENSG00000080200	22100165	validated
mirtarbase	hsa-miR-192-5p	DCBLD2	131566	ENSG00000057019	19074876	validated
mirtarbase	hsa-miR-106b-5p	DCBLD2	131566	ENSG00000057019	26701625	validated
mirtarbase	hsa-miR-144-3p	DCBLD2	131566	ENSG00000057019	26701625	validated
mirtarbase	hsa-miR-181a-5p	DCBLD2	131566	ENSG00000057019	26701625	validated
mirtarbase	hsa-miR-301a-3p	DCBLD2	131566	ENSG00000057019	26701625	validated
mirtarbase	hsa-miR-505-3p	NUDT16	131870	ENSG00000198585	23824327	validated
mirtarbase	hsa-miR-106b-5p	GRK7	131890	ENSG00000114124	23446348	validated
mirtarbase	hsa-miR-18a-5p	LIN54	132660	ENSG00000189308	23446348	validated
mirtarbase	hsa-miR-192-5p	FAM241A	132720	ENSG00000174749	19074876	validated
mirtarbase	hsa-miR-106b-5p	FAM241A	132720	ENSG00000174749	27418678	validated
mirtarbase	hsa-miR-106b-5p	GNPDA2	132789	ENSG00000163281	17242205	validated
mirtarbase	hsa-miR-15b-5p	CPEB2	132864	ENSG00000137449	22473208	validated
mirtarbase	hsa-miR-301a-3p	C4orf36	132989	ENSG00000163633	23824327	validated
mirtarbase	hsa-miR-192-5p	EMB	133418	ENSG00000170571	19074876	validated
mirtarbase	hsa-miR-192-5p	PRRC1	133619	ENSG00000164244	19074876	validated
mirtarbase	hsa-miR-21-5p	PRRC1	133619	ENSG00000164244	18591254	validated
mirtarbase	hsa-miR-181a-5p	UGT3A1	133688	ENSG00000145626	17612493	validated
mirtarbase	hsa-miR-301a-3p	JMY	133746	ENSG00000152409	21572407	validated
mirtarbase	hsa-miR-144-3p	JMY	133746	ENSG00000152409	23592263	validated
mirtarbase	hsa-miR-18a-5p	JMY	133746	ENSG00000152409	23824327	validated
mirtarbase	hsa-miR-18a-5p	ATPSCKMT	134145	ENSG00000150756	23622248	validated
mirtarbase	hsa-miR-192-5p	AFAP1L1	134265	ENSG00000157510	19074876	validated
mirtarbase	hsa-miR-21-5p	GRPEL2	134266	ENSG00000164284	20371350	validated
mirtarbase	hsa-miR-106b-5p	GRPEL2	134266	ENSG00000164284	22473208	validated
mirtarbase	hsa-miR-582-3p	GRPEL2	134266	ENSG00000164284	20371350	validated
mirtarbase	hsa-miR-15b-5p	LSM11	134353	ENSG00000155858	22473208	validated
mirtarbase	hsa-miR-181a-5p	ACOT12	134526	ENSG00000172497	17612493	validated

mirtarbase	hsa-miR-106b-5p	ADAT2	134637	ENSG00000189007	22012620	validated
mirtarbase	hsa-miR-181a-5p	IRAK1BP1	134728	ENSG00000146243	20371350	validated
mirtarbase	hsa-miR-15b-5p	IRAK1BP1	134728	ENSG00000146243	22473208	validated
mirtarbase	hsa-miR-21-5p	STXBP5	134957	ENSG00000164506	20048743	validated
mirtarbase	hsa-miR-181a-5p	NCOA7	135112	ENSG00000111912	21572407	validated
mirtarbase	hsa-miR-192-5p	HINT3	135114	ENSG00000111911	19074876	validated
mirtarbase	hsa-miR-21-5p	PM20D2	135293	ENSG00000146281	18591254	validated
mirtarbase	hsa-miR-106b-5p	ZNF786	136051	ENSG00000197362	27418678	validated
mirtarbase	hsa-miR-21-5p	MTPN	136319	ENSG00000105887	18591254	validated
mirtarbase	hsa-miR-301a-3p	MTPN	136319	ENSG00000105887	23446348	validated
mirtarbase	hsa-miR-222-3p	CLDN23	137075	ENSG00000253958	23622248	validated
mirtarbase	hsa-miR-18a-5p	VPS37A	137492	ENSG00000155975	23622248	validated
mirtarbase	hsa-miR-301a-3p	VPS37A	137492	ENSG00000155975	21572407	validated
mirtarbase	hsa-miR-106b-5p	NACC2	138151	ENSG00000148411	23446348	validated
mirtarbase	hsa-miR-301a-3p	NACC2	138151	ENSG00000148411	23446348	validated
mirtarbase	hsa-miR-582-5p	CARNMT1	138199	ENSG00000156017	23592263	validated
mirtarbase	hsa-miR-21-5p	PTPDC1	138639	ENSG00000158079	18591254	validated
mirtarbase	hsa-miR-106b-5p	PTPDC1	138639	ENSG00000158079	21572407	validated
mirtarbase	hsa-miR-181a-5p	PTPDC1	138639	ENSG00000158079	21572407	validated
mirtarbase	hsa-miR-192-5p	FAM199X	139231	ENSG00000123575	19074876	validated
mirtarbase	hsa-miR-106b-5p	FAM199X	139231	ENSG00000123575	23622248	validated
mirtarbase	hsa-miR-15b-5p	AMER1	139285	ENSG00000184675	22473208	validated
mirtarbase	hsa-miR-18a-5p	PTCHD1	139411	ENSG00000165186	22100165	validated
mirtarbase	hsa-miR-15b-5p	DOCK11	139818	ENSG00000147251	23592263	validated
mirtarbase	hsa-miR-192-5p	SPIN4	139886	ENSG00000186767	19074876	validated
mirtarbase	hsa-miR-15b-5p	ZFP28	140612	ENSG00000196867	20371350	validated
mirtarbase	hsa-miR-15b-5p	NOL4L	140688	ENSG00000197183	22473208	validated
mirtarbase	hsa-miR-222-3p	MROH8	140699	ENSG00000101353	23622248	validated
mirtarbase	hsa-miR-106b-5p	BRI3BP	140707	ENSG00000184992	23824327	validated
mirtarbase	hsa-miR-505-3p	RIMS4	140730	ENSG00000101098	27292025	validated
mirtarbase	hsa-miR-15b-5p	DYNLL2	140735	ENSG00000264364	23446348	validated
mirtarbase	hsa-miR-505-3p	TMEM37	140738	ENSG00000171227	23592263	validated
mirtarbase	hsa-miR-181a-5p	SMCR8	140775	ENSG00000176994	22473208	validated
mirtarbase	hsa-miR-505-3p	SRXN1	140809	ENSG00000271303	23313552	validated
mirtarbase	hsa-miR-301a-3p	ROMO1	140823	ENSG00000125995	23824327	validated
mirtarbase	hsa-miR-192-5p	ZSWIM3	140831	ENSG00000132801	19074876	validated
mirtarbase	hsa-miR-106b-5p	ZNF280B	140883	ENSG00000275004	22473208	validated
mirtarbase	hsa-miR-212-3p	ZNF280B	140883	ENSG00000275004	21572407	validated
mirtarbase	hsa-miR-21-5p	SREK1	140890	ENSG00000153914	18591254	validated
mirtarbase	hsa-miR-15b-5p	SREK1	140890	ENSG00000153914	24398324	validated
mirtarbase	hsa-miR-106b-5p	SAMD8	142891	ENSG00000156671	21572407	validated
mirtarbase	hsa-miR-301a-3p	SAMD8	142891	ENSG00000156671	21572407	validated
mirtarbase	hsa-miR-212-3p	TRUB1	142940	ENSG00000165832	21572407	validated
mirtarbase	hsa-miR-106b-5p	VTI1A	143187	ENSG00000151532	22473208	validated
mirtarbase	hsa-miR-15b-5p	CACUL1	143384	ENSG00000151893	21572407	validated
mirtarbase	hsa-miR-106b-5p	SESN3	143686	ENSG00000149212	20371350	validated
mirtarbase	hsa-miR-222-3p	ARHGAP42	143872	ENSG00000165895	23446348	validated
mirtarbase	hsa-miR-181a-5p	KBTBD3	143879	ENSG00000182359	17612493	validated
mirtarbase	hsa-miR-192-5p	POGLUT3	143888	ENSG00000178202	19074876	validated
mirtarbase	hsa-miR-296-5p	TMEM86A	144110	ENSG00000151117	22012620	validated
mirtarbase	hsa-miR-192-5p	PRICKLE1	144165	ENSG00000139174	19074876	validated
mirtarbase	hsa-miR-192-5p	ALG10B	144245	ENSG00000175548	19074876	validated
mirtarbase	hsa-miR-181a-5p	ZNF664	144348	ENSG00000179195	22473208	validated
mirtarbase	hsa-miR-15b-5p	ETFRF1	144363	ENSG00000205707	22473208	validated
mirtarbase	hsa-miR-15b-5p	E2F7	144455	ENSG00000165891	22473208	validated
mirtarbase	hsa-miR-326	FAM216B	144809	ENSG00000179813	23824327	validated
mirtarbase	hsa-miR-15b-5p	HNRNPA1L2	144983	ENSG00000139675	23313552	validated
mirtarbase	hsa-miR-326	HNRNPA1L2	144983	ENSG00000139675	23313552	validated
mirtarbase	hsa-miR-192-5p	MIPOL1	145282	ENSG00000151338	19074876	validated
mirtarbase	hsa-miR-301a-3p	PTGR2	145482	ENSG00000140043	23824327	validated
mirtarbase	hsa-miR-192-5p	CEP128	145508	ENSG00000100629	19074876	validated
mirtarbase	hsa-miR-192-5p	NRG4	145957	ENSG00000169752	19074876	validated
mirtarbase	hsa-miR-15b-5p	CMTM4	146223	ENSG00000183723	20371350	validated
mirtarbase	hsa-miR-505-3p	FBXL16	146330	ENSG00000127585	23824327	validated
mirtarbase	hsa-miR-326	FBXL16	146330	ENSG00000127585	23824327	validated
mirtarbase	hsa-miR-106b-5p	ZNF597	146434	ENSG00000167981	22473208	validated
mirtarbase	hsa-miR-181a-5p	ZNF597	146434	ENSG00000167981	19536157	validated
mirtarbase	hsa-miR-106b-5p	ZNF785	146540	ENSG00000197162	23706177	validated
mirtarbase	hsa-miR-192-5p	TCAM1P	146771		19074876	validated
mirtarbase	hsa-miR-301a-3p	ODF4	146852	ENSG00000184650	23824327	validated
mirtarbase	hsa-miR-181a-5p	SLC35G3	146861	ENSG00000164729	23824327	validated

mirtarbase	hsa-miR-106b-5p	RUNDC1	146923	ENSG00000198863	22473208	validated
mirtarbase	hsa-miR-301a-3p	RUNDC1	146923	ENSG00000198863	23313552	validated
mirtarbase	hsa-miR-192-5p	EME1	146956	ENSG00000154920	19074876	validated
mirtarbase	hsa-miR-301a-3p	WIPF2	147179	ENSG00000171475	21572407	validated
mirtarbase	hsa-miR-106b-5p	WIPF2	147179	ENSG00000171475	21572407	validated
mirtarbase	hsa-miR-222-3p	C18orf25	147339	ENSG00000152242	22815788	validated
mirtarbase	hsa-miR-144-3p	ZNF480	147657	ENSG00000198464	22100165	validated
mirtarbase	hsa-miR-106b-5p	ZNF578	147660	ENSG00000258405	23313552	validated
mirtarbase	hsa-miR-301a-3p	ZNF417	147687	ENSG00000173480	23446348	validated
mirtarbase	hsa-miR-106b-5p	ZNF417	147687	ENSG00000173480	23446348	validated
mirtarbase	hsa-miR-181a-5p	ZNF558	148156	ENSG00000167785	17612493	validated
mirtarbase	hsa-miR-222-3p	ZNF714	148206	ENSG00000160352	23622248	validated
mirtarbase	hsa-miR-106b-5p	ZNF681	148213	ENSG00000196172	21572407	validated
mirtarbase	hsa-miR-192-5p	SAMD13	148418	ENSG00000203943	19074876	validated
mirtarbase	hsa-miR-21-5p	TLCD4	148534	ENSG00000152078	18591254	validated
mirtarbase	hsa-miR-192-5p	SLC35F3	148641	ENSG00000183780	19074876	validated
mirtarbase	hsa-miR-144-3p	B3GALNT2	148789	ENSG00000162885	27292025	validated
mirtarbase	hsa-miR-222-3p	B3GALNT2	148789	ENSG00000162885	27292025	validated
mirtarbase	hsa-miR-106b-5p	SLC30A7	148867	ENSG00000162695	23592263	validated
mirtarbase	hsa-miR-181a-5p	DCST1	149095	ENSG00000163357	17612493	validated
mirtarbase	hsa-miR-106b-5p	MANEAL	149175	ENSG00000185090	23313552	validated
mirtarbase	hsa-miR-301a-3p	IL23R	149233	ENSG00000162594	23824327	validated
mirtarbase	hsa-miR-21-5p	EXOC8	149371	ENSG00000116903	18591254	validated
mirtarbase	hsa-miR-222-3p	EXOC8	149371	ENSG00000116903	20371350	validated
mirtarbase	hsa-miR-18a-5p	PDIK1L	149420	ENSG00000175087	23622248	validated
mirtarbase	hsa-miR-15b-5p	PDIK1L	149420	ENSG00000175087	22473208	validated
mirtarbase	hsa-miR-222-3p	PDIK1L	149420	ENSG00000175087	21572407	validated
mirtarbase	hsa-miR-181a-5p	RNF187	149603	ENSG00000168159	19536157	validated
mirtarbase	hsa-miR-192-5p	LSM14B	149986	ENSG00000149657	19074876	validated
mirtarbase	hsa-miR-106b-5p	SIK1	150094	ENSG00000142178	21572407	validated
mirtarbase	hsa-miR-15b-5p	SIK1	150094	ENSG00000142178	22473208	validated
mirtarbase	hsa-miR-301a-3p	SIK1	150094	ENSG00000142178	23592263	validated
mirtarbase	hsa-miR-106b-5p	DUSP18	150290	ENSG00000167065	22473208	validated
mirtarbase	hsa-miR-192-5p	DUSP18	150290	ENSG00000167065	22291592	validated
mirtarbase	hsa-miR-301a-3p	DUSP18	150290	ENSG00000167065	23824327	validated
mirtarbase	hsa-miR-582-3p	DUSP18	150290	ENSG00000167065	27292025	validated
mirtarbase	hsa-miR-192-5p	CKAP2L	150468	ENSG00000169607	19074876	validated
mirtarbase	hsa-miR-106b-5p	FAM117B	150864	ENSG00000138439	22473208	validated
mirtarbase	hsa-miR-192-5p	GAREM2	150946	ENSG00000157833	19074876	validated
mirtarbase	hsa-miR-192-5p	SEPTIN10	151011	ENSG00000186522	19074876	validated
mirtarbase	hsa-miR-192-5p	ZNF385B	151126	ENSG00000144331	19074876	validated
mirtarbase	hsa-miR-192-5p	ERFE	151176	ENSG00000178752	19074876	validated
mirtarbase	hsa-miR-181a-5p	ARL6IP6	151188	ENSG00000177917	17612493	validated
mirtarbase	hsa-miR-192-5p	KLHL23	151230	ENSG00000213160	19074876	validated
mirtarbase	hsa-miR-192-5p	SGO2	151246	ENSG00000163535	19074876	validated
mirtarbase	hsa-miR-222-3p	LRATD1	151354	ENSG00000162981	22291592	validated
mirtarbase	hsa-miR-192-5p	SLC16A14	151473	ENSG00000163053	19074876	validated
mirtarbase	hsa-miR-106b-5p	GPR155	151556	ENSG00000163328	23446348	validated
mirtarbase	hsa-miR-21-5p	DTX3L	151636	ENSG00000163840	18591254	validated
mirtarbase	hsa-miR-21-5p	PPM1L	151742	ENSG00000163590	20371350	validated
mirtarbase	hsa-miR-15b-5p	CCDC80	151887	ENSG00000091986	20371350	validated
mirtarbase	hsa-miR-326	CCDC12	151903	ENSG00000160799	23622248	validated
mirtarbase	hsa-miR-301a-3p	MB21D2	151963	ENSG00000180611	21572407	validated
mirtarbase	hsa-miR-15b-5p	RNF38	152006	ENSG00000137075	22473208	validated
mirtarbase	hsa-miR-144-3p	ZNF827	152485	ENSG00000151612	22012620	validated
mirtarbase	hsa-miR-192-5p	NIPAL1	152519	ENSG00000163293	19074876	validated
mirtarbase	hsa-miR-106b-5p	THAP6	152815	ENSG00000174796	17242205	validated
mirtarbase	hsa-miR-301a-3p	THAP6	152815	ENSG00000174796	21572407	validated
mirtarbase	hsa-miR-326	DAB2IP	153090	ENSG00000136848	26701625	validated
mirtarbase	hsa-miR-301a-3p	SLC38A9	153129	ENSG00000177058	23824327	validated
mirtarbase	hsa-miR-15b-5p	CREBRF	153222	ENSG00000164463	22473208	validated
mirtarbase	hsa-miR-106b-5p	TMEM167A	153339	ENSG00000174695	22473208	validated
mirtarbase	hsa-miR-15b-5p	TMEM161B	153396	ENSG00000164180	21572407	validated
mirtarbase	hsa-miR-192-5p	BTNL9	153579	ENSG00000165810	19074876	validated
mirtarbase	hsa-miR-21-5p	SNRNP48	154007	ENSG00000168566	18591254	validated
mirtarbase	hsa-miR-106b-5p	CNKSR3	154043	ENSG00000153721	23446348	validated
mirtarbase	hsa-miR-15b-5p	CNKSR3	154043	ENSG00000153721	21572407	validated
mirtarbase	hsa-miR-192-5p	SAMD3	154075	ENSG00000164483	19074876	validated
mirtarbase	hsa-miR-106b-5p	BMT2	154743	ENSG00000164603	22473208	validated
mirtarbase	hsa-miR-15b-5p	AMOT	154796	ENSG00000126016	21572407	validated
mirtarbase	hsa-miR-15b-5p	AMOTL1	154810	ENSG00000166025	23592263	validated

mirtarbase	hsa-miR-326	ZNF746	155061	ENSG00000181220	23622248	validated
mirtarbase	hsa-miR-326	METTL27	155368	ENSG00000165171	23824327	validated
mirtarbase	hsa-miR-192-5p	SBDSP1	155370		19074876	validated
mirtarbase	hsa-miR-15b-5p	PEBP4	157310	ENSG00000134020	25721211	validated
mirtarbase	hsa-miR-18a-5p	RDH10	157506	ENSG00000121039	23592263	validated
mirtarbase	hsa-miR-21-5p	ANKRD46	157567	ENSG00000186106	21219636	validated
mirtarbase	hsa-miR-192-5p	ESCO2	157570	ENSG00000171320	19074876	validated
mirtarbase	hsa-miR-106b-5p	FAM91A1	157769	ENSG00000176853	23622248	validated
mirtarbase	hsa-miR-192-5p	CLVS1	157807	ENSG00000177182	19074876	validated
mirtarbase	hsa-miR-15b-5p	CAMSAP1	157922	ENSG00000130559	22473208	validated
mirtarbase	hsa-miR-192-5p	C9orf163	158055	ENSG00000196366	19074876	validated
mirtarbase	hsa-miR-21-5p	RASEF	158158	ENSG00000165105	18591254	validated
mirtarbase	hsa-miR-15b-5p	RASEF	158158	ENSG00000165105	20371350	validated
mirtarbase	hsa-miR-192-5p	FAM120AOS	158293	ENSG00000188938	19074876	validated
mirtarbase	hsa-miR-301a-3p	FAM120AOS	158293	ENSG00000188938	23824327	validated
mirtarbase	hsa-miR-192-5p	KIAA1958	158405	ENSG00000165185	19074876	validated
mirtarbase	hsa-miR-301a-3p	PRUNE2	158471	ENSG00000106772	21572407	validated
mirtarbase	hsa-miR-144-3p	CBLL2	158506	ENSG00000175809	22291592	validated
mirtarbase	hsa-miR-222-3p	CSAG1	158511	ENSG00000198930	23622248	validated
mirtarbase	hsa-miR-296-5p	ZDHHC15	158866	ENSG00000102383	22012620	validated
mirtarbase	hsa-miR-181a-5p	ZDHHC15	158866	ENSG00000102383	22012620	validated
mirtarbase	hsa-miR-582-5p	ZDHHC15	158866	ENSG00000102383	23824327	validated
mirtarbase	hsa-miR-106b-5p	CXorf38	159013	ENSG00000185753	23313552	validated
mirtarbase	hsa-miR-144-3p	FAM122B	159090	ENSG00000156504	24398324	validated
mirtarbase	hsa-miR-15b-5p	FAM122B	159090	ENSG00000156504	24398324	validated
mirtarbase	hsa-miR-192-5p	USP54	159195	ENSG00000166348	19074876	validated
mirtarbase	hsa-miR-192-5p	SLC35G1	159371	ENSG00000176273	19074876	validated
mirtarbase	hsa-miR-505-3p	SLC5A12	159963	ENSG00000148942	19536157	validated
mirtarbase	hsa-miR-192-5p	TMTC2	160335	ENSG00000179104	19074876	validated
mirtarbase	hsa-miR-192-5p	TMTC3	160418	ENSG00000139324	19074876	validated
mirtarbase	hsa-miR-144-3p	TMTC3	160418	ENSG00000139324	21572407	validated
mirtarbase	hsa-miR-106b-5p	DENND5B	160518	ENSG00000170456	22473208	validated
mirtarbase	hsa-miR-582-5p	DENND5B	160518	ENSG00000170456	23313552	validated
mirtarbase	hsa-miR-144-3p	DENND5B	160518	ENSG00000170456	23313552	validated
mirtarbase	hsa-miR-15b-5p	PPTC7	160760	ENSG00000196850	23622248	validated
mirtarbase	hsa-miR-505-3p	DGKH	160851	ENSG00000102780	27292025	validated
mirtarbase	hsa-miR-192-5p	GPR180	160897	ENSG00000152749	19074876	validated
mirtarbase	hsa-miR-15b-5p	GPR180	160897	ENSG00000152749	23446348	validated
mirtarbase	hsa-miR-15b-5p	SPRED1	161742	ENSG00000166068	22473208	validated
mirtarbase	hsa-miR-106b-5p	SPRED1	161742	ENSG00000166068	23592263	validated
mirtarbase	hsa-miR-582-5p	PGBD4	161779	ENSG00000182405	26701625	validated
mirtarbase	hsa-miR-181a-5p	FSIP1	161835	ENSG00000150667	17612493	validated
mirtarbase	hsa-miR-192-5p	ZFP1	162239	ENSG00000184517	19074876	validated
mirtarbase	hsa-miR-222-3p	ZFP1	162239	ENSG00000184517	23824327	validated
mirtarbase	hsa-miR-192-5p	ZNF519	162655	ENSG00000175322	19074876	validated
mirtarbase	hsa-miR-192-5p	C18orf54	162681	ENSG00000166845	19074876	validated
mirtarbase	hsa-miR-192-5p	ZNF320	162967	ENSG00000182986	19074876	validated
mirtarbase	hsa-miR-181a-5p	ZNF846	162993	ENSG00000196605	20371350	validated
mirtarbase	hsa-miR-181a-5p	ZNF791	163049	ENSG00000173875	23446348	validated
mirtarbase	hsa-miR-192-5p	ZNF114	163071	ENSG00000178150	19074876	validated
mirtarbase	hsa-miR-181a-5p	ZNF781	163115	ENSG00000196381	24398324	validated
mirtarbase	hsa-miR-181a-5p	ZNF780B	163131	ENSG00000128000	21572407	validated
mirtarbase	hsa-miR-582-5p	ZNF100	163227	ENSG00000197020	23446348	validated
mirtarbase	hsa-miR-21-5p	TOR1AIP2	163590	ENSG00000169905	22815788	validated
mirtarbase	hsa-miR-582-5p	TOR1AIP2	163590	ENSG00000169905	23592263	validated
mirtarbase	hsa-miR-144-3p	TOR1AIP2	163590	ENSG00000169905	23592263	validated
mirtarbase	hsa-miR-192-5p	IFNLR1	163702	ENSG00000185436	19074876	validated
mirtarbase	hsa-miR-301a-3p	IFNLR1	163702	ENSG00000185436	19536157	validated
mirtarbase	hsa-miR-192-5p	SASS6	163786	ENSG00000156876	19074876	validated
mirtarbase	hsa-miR-18a-5p	SASS6	163786	ENSG00000156876	23622248	validated
mirtarbase	hsa-miR-181a-5p	HFM1	164045	ENSG00000162669	23824327	validated
mirtarbase	hsa-miR-21-5p	LONRF2	164832	ENSG00000170500	18591254	validated
mirtarbase	hsa-miR-296-5p	TOGARAM2	165186	ENSG00000189350	23824327	validated
mirtarbase	hsa-miR-192-5p	FAM171B	165215	ENSG00000144369	19074876	validated
mirtarbase	hsa-miR-106b-5p	UBXN2A	165324	ENSG00000173960	21572407	validated
mirtarbase	hsa-miR-212-3p	UBXN2A	165324	ENSG00000173960	23706177	validated
mirtarbase	hsa-miR-15b-5p	RNF168	165918	ENSG00000163961	23592263	validated
mirtarbase	hsa-miR-21-5p	PRICKLE2	166336	ENSG00000163637	18591254	validated
mirtarbase	hsa-miR-15b-5p	PRICKLE2	166336	ENSG00000163637	24398324	validated
mirtarbase	hsa-miR-505-3p	PRICKLE2	166336	ENSG00000163637	21572407	validated
mirtarbase	hsa-miR-181a-5p	RASSF6	166824	ENSG00000169435	17612493	validated

mirtarbase	hsa-miR-192-5p	SGMS2	166929	ENSG00000164023	19074876	validated
mirtarbase	hsa-miR-301a-3p	TENT2	167153	ENSG00000164329	21572407	validated
mirtarbase	hsa-miR-106b-5p	CCDC71L	168455	ENSG00000253276	20371350	validated
mirtarbase	hsa-miR-212-3p	BMPER	168667	ENSG00000164619	23708386	validated
mirtarbase	hsa-miR-106b-5p	ZNF800	168850	ENSG00000048405	22473208	validated
mirtarbase	hsa-miR-144-3p	ZNF800	168850	ENSG00000048405	23592263	validated
mirtarbase	hsa-miR-301a-3p	ZNF800	168850	ENSG00000048405	23592263	validated
mirtarbase	hsa-miR-181a-5p	TMEM64	169200	ENSG00000180694	20371350	validated
mirtarbase	hsa-miR-106b-5p	TMEM64	169200	ENSG00000180694	22473208	validated
mirtarbase	hsa-miR-212-3p	OLFML2A	169611	ENSG00000185585	23824327	validated
mirtarbase	hsa-miR-181a-5p	FAM47B	170062	ENSG00000189132	17612493	validated
mirtarbase	hsa-miR-192-5p	FUT11	170384	ENSG00000196968	19074876	validated
mirtarbase	hsa-miR-505-3p	FUT11	170384	ENSG00000196968	23824327	validated
mirtarbase	hsa-miR-301a-3p	FUT11	170384	ENSG00000196968	27418678	validated
mirtarbase	hsa-miR-106b-5p	COMMD6	170622	ENSG00000188243	17242205	validated
mirtarbase	hsa-miR-192-5p	ZNF525	170958	ENSG00000203326	19074876	validated
mirtarbase	hsa-miR-582-5p	ZNF431	170959	ENSG00000196705	22012620	validated
mirtarbase	hsa-miR-301a-3p	ZNF431	170959	ENSG00000196705	23824327	validated
mirtarbase	hsa-miR-15b-5p	ASXL1	171023	ENSG00000171456	22473208	validated
mirtarbase	hsa-miR-15b-5p	RHOV	171177	ENSG00000104140	22473208	validated
mirtarbase	hsa-miR-181a-5p	HIGD2A	192286	ENSG00000146066	22473208	validated
mirtarbase	hsa-miR-106b-5p	AGO3	192669	ENSG00000126070	23446348	validated
mirtarbase	hsa-miR-301a-3p	AGO3	192669	ENSG00000126070	23824327	validated
mirtarbase	hsa-miR-21-5p	AGO4	192670	ENSG00000134698	18591254	validated
mirtarbase	hsa-miR-15b-5p	AGO4	192670	ENSG00000134698	20371350	validated
mirtarbase	hsa-miR-582-5p	AGO4	192670	ENSG00000134698	20371350	validated
mirtarbase	hsa-miR-144-3p	AGO4	192670	ENSG00000134698	20371350	validated
mirtarbase	hsa-miR-106b-5p	SCAMP5	192683	ENSG00000198794	22473208	validated
mirtarbase	hsa-miR-15b-5p	SCAMP5	192683	ENSG00000198794	22473208	validated
mirtarbase	hsa-miR-21-5p	ZNF367	195828	ENSG00000165244	20371350	validated
mirtarbase	hsa-miR-15b-5p	ZNF367	195828	ENSG00000165244	23592263	validated
mirtarbase	hsa-miR-18a-5p	ZNF367	195828	ENSG00000165244	20371350	validated
mirtarbase	hsa-miR-192-5p	ANO6	196527	ENSG00000177119	19074876	validated
mirtarbase	hsa-miR-15b-5p	ARID2	196528	ENSG00000189079	23622248	validated
mirtarbase	hsa-miR-212-3p	ARID2	196528	ENSG00000189079	23592263	validated
mirtarbase	hsa-miR-106b-5p	LDHD	197257	ENSG00000166816	23313552	validated
mirtarbase	hsa-miR-192-5p	ACSF3	197322	ENSG00000176715	19074876	validated
mirtarbase	hsa-miR-582-5p	ALG14	199857	ENSG00000172339	20371350	validated
mirtarbase	hsa-miR-18a-5p	ALG14	199857	ENSG00000172339	20371350	validated
mirtarbase	hsa-miR-192-5p	FAAP20	199990	ENSG00000162585	19074876	validated
mirtarbase	hsa-miR-15b-5p	TXLNA	200081	ENSG00000084652	23622248	validated
mirtarbase	hsa-miR-106b-5p	TXLNA	200081	ENSG00000084652	22473208	validated
mirtarbase	hsa-miR-222-3p	RNF215	200312	ENSG00000099999	23622248	validated
mirtarbase	hsa-miR-106b-5p	TET3	200424	ENSG00000187605	22473208	validated
mirtarbase	hsa-miR-15b-5p	TET3	200424	ENSG00000187605	22473208	validated
mirtarbase	hsa-miR-192-5p	TMEM17	200728	ENSG00000186889	19074876	validated
mirtarbase	hsa-miR-192-5p	SPRED2	200734	ENSG00000198369	20371350	validated
mirtarbase	hsa-miR-106b-5p	ARL13B	200894	ENSG00000169379	17242205	validated
mirtarbase	hsa-miR-192-5p	DHFR2	200895	ENSG00000178700	19074876	validated
mirtarbase	hsa-miR-582-5p	TVP23C	201158	ENSG00000175106	21572407	validated
mirtarbase	hsa-miR-15b-5p	FLCN	201163	ENSG00000154803	23446348	validated
mirtarbase	hsa-miR-106b-5p	TRIM65	201292	ENSG00000141569	27292025	validated
mirtarbase	hsa-miR-192-5p	RDM1	201299	ENSG00000276432	19074876	validated
mirtarbase	hsa-miR-106b-5p	RAB12	201475	ENSG00000206418	22473208	validated
mirtarbase	hsa-miR-15b-5p	STT3B	201595	ENSG00000163527	23622248	validated
mirtarbase	hsa-miR-15b-5p	DENND6A	201627	ENSG00000174839	26701625	validated
mirtarbase	hsa-miR-181a-5p	TMEM192	201931	ENSG00000170088	23622248	validated
mirtarbase	hsa-miR-582-5p	TMEM192	201931	ENSG00000170088	21572407	validated
mirtarbase	hsa-miR-144-3p	TMEM192	201931	ENSG00000170088	21572407	validated
mirtarbase	hsa-miR-192-5p	PRIMPOL	201973	ENSG00000164306	19074876	validated
mirtarbase	hsa-miR-106b-5p	CCDC125	202243	ENSG00000277868	23313552	validated
mirtarbase	hsa-miR-505-3p	TUBB	203068	ENSG00000227739	23622248	validated
mirtarbase	hsa-miR-181a-5p	TUBB	203068	ENSG00000227739	22473208	validated
mirtarbase	hsa-miR-15b-5p	TUBB	203068	ENSG00000227739	22473208	validated
mirtarbase	hsa-miR-192-5p	ERICH5	203111	ENSG00000177459	19074876	validated
mirtarbase	hsa-miR-192-5p	ZNF449	203523	ENSG00000173275	19074876	validated
mirtarbase	hsa-miR-15b-5p	ZNF449	203523	ENSG00000173275	21572407	validated
mirtarbase	hsa-miR-181a-5p	ZNF449	203523	ENSG00000173275	19536157	validated
mirtarbase	hsa-miR-18a-5p	VMA21	203547	ENSG00000160131	23592263	validated
mirtarbase	hsa-miR-181a-5p	C2orf69	205327	ENSG00000178074	22473208	validated
mirtarbase	hsa-miR-106b-5p	C2orf69	205327	ENSG00000178074	22473208	validated

mirtarbase	hsa-miR-18a-5p	SLC36A1	206358	ENSG00000123643	20371350	validated
mirtarbase	hsa-miR-106b-5p	SAMD9L	219285	ENSG00000177409	22473208	validated
mirtarbase	hsa-miR-192-5p	USP12	219333	ENSG00000152484	19074876	validated
mirtarbase	hsa-miR-181a-5p	UNC5B	219699	ENSG00000107731	23824327	validated
mirtarbase	hsa-miR-192-5p	FAM241B	219738	ENSG00000171224	19074876	validated
mirtarbase	hsa-miR-181a-5p	ZNF25	219749	ENSG00000175395	17612493	validated
mirtarbase	hsa-miR-192-5p	CCNY	219771	ENSG00000108100	19074876	validated
mirtarbase	hsa-miR-192-5p	RTKN2	219790	ENSG00000182010	19074876	validated
mirtarbase	hsa-miR-192-5p	HYLS1	219844	ENSG00000198331	19074876	validated
mirtarbase	hsa-miR-192-5p	TLCD5	219902	ENSG00000181264	19074876	validated
mirtarbase	hsa-miR-15b-5p	CYB561A3	220002	ENSG00000162144	22473208	validated
mirtarbase	hsa-miR-15b-5p	CCDC83	220047	ENSG00000150676	21572407	validated
mirtarbase	hsa-miR-192-5p	SKA1	220134	ENSG00000262634	19074876	validated
mirtarbase	hsa-miR-181a-5p	OTUD1	220213	ENSG00000165312	17612493	validated
mirtarbase	hsa-miR-192-5p	OTUD1	220213	ENSG00000165312	19074876	validated
mirtarbase	hsa-miR-21-5p	OTUD1	220213	ENSG00000165312	23622248	validated
mirtarbase	hsa-miR-505-3p	CBWD5	220869	ENSG00000147996	24398324	validated
mirtarbase	hsa-miR-106b-5p	SLC16A9	220963	ENSG00000165449	22473208	validated
mirtarbase	hsa-miR-582-3p	SLC16A9	220963	ENSG00000165449	21572407	validated
mirtarbase	hsa-miR-505-3p	HNRNPA3	220988	ENSG00000170144	23622248	validated
mirtarbase	hsa-miR-106b-5p	REEP3	221035	ENSG00000165476	22473208	validated
mirtarbase	hsa-miR-326	JMJD1C	221037	ENSG00000171988	23622248	validated
mirtarbase	hsa-miR-144-3p	ARL5B	221079	ENSG00000165997	23592263	validated
mirtarbase	hsa-miR-326	ARL5B	221079	ENSG00000165997	23446348	validated
mirtarbase	hsa-miR-326	HNRNPUL2	221092	ENSG00000214753	23622248	validated
mirtarbase	hsa-miR-192-5p	SPATA13	221178	ENSG00000182957	19074876	validated
mirtarbase	hsa-miR-505-3p	OPN5	221391	ENSG00000124818	23446348	validated
mirtarbase	hsa-miR-106b-5p	KIF6	221458	ENSG00000164627	23446348	validated
mirtarbase	hsa-miR-15b-5p	ARMC12	221481	ENSG00000157343	20371350	validated
mirtarbase	hsa-miR-505-3p	ZBTB9	221504	ENSG00000236515	23622248	validated
mirtarbase	hsa-miR-106b-5p	ZBTB9	221504	ENSG00000236515	22473208	validated
mirtarbase	hsa-miR-192-5p	KDM1B	221656	ENSG00000165097	19074876	validated
mirtarbase	hsa-miR-106b-5p	SMIM13	221710	ENSG00000224531	23592263	validated
mirtarbase	hsa-miR-192-5p	PXDC1	221749	ENSG00000168994	19074876	validated
mirtarbase	hsa-miR-222-3p	PRPS1L1	221823	ENSG00000229937	20371350	validated
mirtarbase	hsa-miR-212-3p	TWISTNB	221830	ENSG00000105849	22012620	validated
mirtarbase	hsa-miR-192-5p	SP8	221833	ENSG00000164651	22100165	validated
mirtarbase	hsa-miR-15b-5p	BRAT1	221927	ENSG00000106009	23622248	validated
mirtarbase	hsa-miR-15b-5p	FOXK1	221937	ENSG00000164916	23622248	validated
mirtarbase	hsa-miR-106b-5p	FOXK1	221937	ENSG00000164916	23446348	validated
mirtarbase	hsa-miR-192-5p	CCZ1B	221960	ENSG00000146574	19074876	validated
mirtarbase	hsa-miR-181a-5p	TMED4	222068	ENSG00000158604	20371350	validated
mirtarbase	hsa-miR-192-5p	RSBN1L	222194	ENSG00000187257	19074876	validated
mirtarbase	hsa-miR-181a-5p	RSBN1L	222194	ENSG00000187257	21572407	validated
mirtarbase	hsa-miR-21-5p	FBXL13	222235	ENSG00000161040	23446348	validated
mirtarbase	hsa-miR-18a-5p	ADGRF2	222611	ENSG00000164393	27418678	validated
mirtarbase	hsa-miR-15b-5p	ZNRF2	223082	ENSG00000180233	23446348	validated
mirtarbase	hsa-miR-326	ZNRF2	223082	ENSG00000180233	22100165	validated
mirtarbase	hsa-miR-21-5p	TXLNGY	246126		18591254	validated
mirtarbase	hsa-miR-106b-5p	CNOT6L	246175	ENSG00000138767	23622248	validated
mirtarbase	hsa-miR-106b-5p	RNASEH1	246243	ENSG00000171865	22012620	validated
mirtarbase	hsa-miR-192-5p	STXBP4	252983	ENSG00000166263	19074876	validated
mirtarbase	hsa-miR-106b-5p	PRR14L	253143	ENSG00000183530	22473208	validated
mirtarbase	hsa-miR-21-5p	PRR14L	253143	ENSG00000183530	26701625	validated
mirtarbase	hsa-miR-18a-5p	RICTOR	253260	ENSG00000164327	23622248	validated
mirtarbase	hsa-miR-301a-3p	IPMK	253430	ENSG00000151151	21572407	validated
mirtarbase	hsa-miR-21-5p	ZBTB38	253461	ENSG00000177311	18591254	validated
mirtarbase	hsa-miR-192-5p	SLC25A30	253512	ENSG00000174032	19074876	validated
mirtarbase	hsa-miR-181a-5p	LCLAT1	253558	ENSG00000172954	20371350	validated
mirtarbase	hsa-miR-301a-3p	LCLAT1	253558	ENSG00000172954	21572407	validated
mirtarbase	hsa-miR-106b-5p	CADM2	253559	ENSG00000175161	22100165	validated
mirtarbase	hsa-miR-15b-5p	ZNF620	253639	ENSG00000177842	23446348	validated
mirtarbase	hsa-miR-301a-3p	ZNF620	253639	ENSG00000177842	21572407	validated
mirtarbase	hsa-miR-144-3p	MMS22L	253714	ENSG00000146263	23592263	validated
mirtarbase	hsa-miR-192-5p	CERS6	253782	ENSG00000172292	19074876	validated
mirtarbase	hsa-miR-21-5p	CERS6	253782	ENSG00000172292	18591254	validated
mirtarbase	hsa-miR-192-5p	MSRB3	253827	ENSG00000174099	19074876	validated
mirtarbase	hsa-miR-106b-5p	ZDHHC20	253832	ENSG00000180776	23592263	validated
mirtarbase	hsa-miR-144-3p	UBN2	254048	ENSG00000157741	20371350	validated
mirtarbase	hsa-miR-301a-3p	UBN2	254048	ENSG00000157741	23446348	validated
mirtarbase	hsa-miR-15b-5p	UBN2	254048	ENSG00000157741	22473208	validated

mirtarbase	hsa-miR-222-3p	UBN2	254048	ENSG00000157741	23592263	validated
mirtarbase	hsa-miR-192-5p	EHBP1L1	254102	ENSG00000173442	19074876	validated
mirtarbase	hsa-miR-181a-5p	FBXO33	254170	ENSG00000165355	20371350	validated
mirtarbase	hsa-miR-505-3p	FBXO33	254170	ENSG00000165355	23622248	validated
mirtarbase	hsa-miR-326	CALHM5	254228	ENSG00000178033	22012620	validated
mirtarbase	hsa-miR-21-5p	LCORL	254251	ENSG00000178177	18591254	validated
mirtarbase	hsa-miR-582-5p	ZDHHC24	254359	ENSG00000174165	27292025	validated
mirtarbase	hsa-miR-192-5p	MCM9	254394	ENSG00000111877	19074876	validated
mirtarbase	hsa-miR-192-5p	VXN	254778	ENSG00000169085	19074876	validated
mirtarbase	hsa-miR-192-5p	ZDHHC23	254887	ENSG00000184307	19074876	validated
mirtarbase	hsa-miR-21-5p	CASC2	255082		25446261	validated
mirtarbase	hsa-miR-326	PLA2G4F	255189	ENSG00000168907	23622248	validated
mirtarbase	hsa-miR-21-5p	LRRC57	255252	ENSG00000180979	18591254	validated
mirtarbase	hsa-miR-15b-5p	LRRC57	255252	ENSG00000180979	22473208	validated
mirtarbase	hsa-miR-192-5p	PCSK9	255738	ENSG00000169174	19074876	validated
mirtarbase	hsa-miR-192-5p	NPNT	255743	ENSG00000168743	19074876	validated
mirtarbase	hsa-miR-106b-5p	NPNT	255743	ENSG00000168743	22100165	validated
mirtarbase	hsa-miR-192-5p	CNEP1R1	255919	ENSG00000205423	19074876	validated
mirtarbase	hsa-miR-582-5p	CNEP1R1	255919	ENSG00000205423	23313552	validated
mirtarbase	hsa-miR-144-3p	CNEP1R1	255919	ENSG00000205423	23313552	validated
mirtarbase	hsa-miR-21-5p	PAN3	255967	ENSG00000152520	18591254	validated
mirtarbase	hsa-miR-106b-5p	TMEM196	256130	ENSG00000173452	22012620	validated
mirtarbase	hsa-miR-21-5p	GK5	256356	ENSG00000175066	22473208	validated
mirtarbase	hsa-miR-181a-5p	GK5	256356	ENSG00000175066	22473208	validated
mirtarbase	hsa-miR-106b-5p	MFSD8	256471	ENSG00000164073	23446348	validated
mirtarbase	hsa-miR-181a-5p	WDR72	256764	ENSG00000166415	23824327	validated
mirtarbase	hsa-miR-582-3p	C10orf67	256815	ENSG00000179133	21572407	validated
mirtarbase	hsa-miR-505-3p	NEGR1	257194	ENSG00000172260	23313552	validated
mirtarbase	hsa-miR-15b-5p	NEGR1	257194	ENSG00000172260	23313552	validated
mirtarbase	hsa-miR-181a-5p	TAB3	257397	ENSG00000157625	22942087	validated
mirtarbase	hsa-miR-106b-5p	ANKS4B	257629	ENSG00000175311	23313552	validated
mirtarbase	hsa-miR-106b-5p	SGMS1	259230	ENSG00000198964	23592263	validated
mirtarbase	hsa-miR-192-5p	ASPM	259266	ENSG00000066279	19074876	validated
mirtarbase	hsa-miR-181a-5p	OFCC1	266553		17612493	validated
mirtarbase	hsa-miR-106b-5p	SSX7	280658	ENSG00000187754	17242205	validated
mirtarbase	hsa-miR-181a-5p	BLOC1S2	282991	ENSG00000196072	22473208	validated
mirtarbase	hsa-miR-106b-5p	RBM20	282996	ENSG00000203867	23446348	validated
mirtarbase	hsa-miR-301a-3p	RBM20	282996	ENSG00000203867	23446348	validated
mirtarbase	hsa-miR-326	RBM20	282996	ENSG00000203867	26701625	validated
mirtarbase	hsa-miR-15b-5p	MKX	283078	ENSG00000150051	21572407	validated
mirtarbase	hsa-miR-106b-5p	PGM2L1	283209	ENSG00000165434	22473208	validated
mirtarbase	hsa-miR-18a-5p	RASSF3	283349	ENSG00000153179	20371350	validated
mirtarbase	hsa-miR-106b-5p	ANKRD52	283373	ENSG00000139645	22473208	validated
mirtarbase	hsa-miR-192-5p	GAS2L3	283431	ENSG00000139354	19074876	validated
mirtarbase	hsa-miR-192-5p	SLC46A3	283537	ENSG00000139508	19074876	validated
mirtarbase	hsa-miR-144-3p	SLC46A3	283537	ENSG00000139508	24398324	validated
mirtarbase	hsa-miR-192-5p	GPR137C	283554	ENSG00000180998	19074876	validated
mirtarbase	hsa-miR-192-5p	C17orf58	284018	ENSG00000186665	19074876	validated
mirtarbase	hsa-miR-106b-5p	KANSL1	284058	ENSG00000278458	23622248	validated
mirtarbase	hsa-miR-106b-5p	FAM171A2	284069	ENSG00000161682	23622248	validated
mirtarbase	hsa-miR-106b-5p	NEK8	284086	ENSG00000160602	23446348	validated
mirtarbase	hsa-miR-106b-5p	CAVIN1	284119	ENSG00000177469	23446348	validated
mirtarbase	hsa-miR-192-5p	ZADH2	284273	ENSG00000180011	19074876	validated
mirtarbase	hsa-miR-21-5p	ZADH2	284273	ENSG00000180011	18591254	validated
mirtarbase	hsa-miR-181a-5p	ZADH2	284273	ENSG00000180011	23824327	validated
mirtarbase	hsa-miR-192-5p	ZNF776	284309	ENSG00000152443	23592263	validated
mirtarbase	hsa-miR-192-5p	ZNF780A	284323	ENSG00000197782	19074876	validated
mirtarbase	hsa-miR-106b-5p	ZNF780A	284323	ENSG00000197782	23446348	validated
mirtarbase	hsa-miR-181a-5p	ZNF763	284390	ENSG00000197054	20591824	validated
mirtarbase	hsa-miR-181a-5p	ZNF844	284391	ENSG00000223547	21572407	validated
mirtarbase	hsa-miR-106b-5p	SPATA46	284680	ENSG00000171722	17242205	validated
mirtarbase	hsa-miR-21-5p	ZNF326	284695	ENSG00000162664	18591254	validated
mirtarbase	hsa-miR-192-5p	RIMKLA	284716	ENSG00000177181	19074876	validated
mirtarbase	hsa-miR-192-5p	CCDC150	284992	ENSG00000144395	19074876	validated
mirtarbase	hsa-miR-15b-5p	RNF149	284996	ENSG00000163162	23622248	validated
mirtarbase	hsa-miR-301a-3p	RNF149	284996	ENSG00000163162	27418678	validated
mirtarbase	hsa-miR-192-5p	FAM126B	285172	ENSG00000155744	19074876	validated
mirtarbase	hsa-miR-21-5p	FAM126B	285172	ENSG00000155744	18591254	validated
mirtarbase	hsa-miR-222-3p	FAM126B	285172	ENSG00000155744	23622248	validated
mirtarbase	hsa-miR-106b-5p	FAM126B	285172	ENSG00000155744	22473208	validated
mirtarbase	hsa-miR-192-5p	EOGT	285203	ENSG00000163378	19074876	validated

mirtarbase	hsa-miR-301a-3p	EOGT	285203	ENSG00000163378	23592263	validated
mirtarbase	hsa-miR-106b-5p	C3orf38	285237	ENSG00000179021	24398324	validated
mirtarbase	hsa-miR-106b-5p	SUMF1	285362	ENSG00000144455	17242205	validated
mirtarbase	hsa-miR-192-5p	CYP4V2	285440	ENSG00000145476	19074876	validated
mirtarbase	hsa-miR-21-5p	CYP4V2	285440	ENSG00000145476	18591254	validated
mirtarbase	hsa-miR-181a-5p	GPRIN3	285513	ENSG00000185477	20371350	validated
mirtarbase	hsa-miR-106b-5p	GPRIN3	285513	ENSG00000185477	23313552	validated
mirtarbase	hsa-miR-15b-5p	FRYL	285527	ENSG00000075539	22473208	validated
mirtarbase	hsa-miR-582-3p	ARL10	285598	ENSG00000175414	23592263	validated
mirtarbase	hsa-miR-222-3p	C5orf51	285636	ENSG00000205765	23592263	validated
mirtarbase	hsa-miR-505-3p	C5orf64	285668		22012620	validated
mirtarbase	hsa-miR-106b-5p	SREK1IP1	285672	ENSG00000153006	22100165	validated
mirtarbase	hsa-miR-106b-5p	ZNF454	285676	ENSG00000178187	19536157	validated
mirtarbase	hsa-miR-106b-5p	RGMB	285704	ENSG00000174136	21572407	validated
mirtarbase	hsa-miR-582-5p	RPL7L1	285855	ENSG00000146223	21572407	validated
mirtarbase	hsa-miR-144-3p	RPL7L1	285855	ENSG00000146223	21572407	validated
mirtarbase	hsa-miR-15b-5p	TCAF2	285966	ENSG00000170379	23622248	validated
mirtarbase	hsa-miR-181a-5p	TUSC1	286319	ENSG00000198680	17612493	validated
mirtarbase	hsa-miR-15b-5p	LURAP1L	286343	ENSG00000153714	21572407	validated
mirtarbase	hsa-miR-15b-5p	YIPF6	286451	ENSG00000181704	21572407	validated
mirtarbase	hsa-miR-192-5p	LIN9	286826	ENSG00000183814	19074876	validated
mirtarbase	hsa-miR-192-5p	TRIM59	286827	ENSG00000213186	19074876	validated
mirtarbase	hsa-miR-21-5p	TRIM59	286827	ENSG00000213186	18591254	validated
mirtarbase	hsa-miR-181a-5p	TAAR6	319100	ENSG00000146383	17612493	validated
mirtarbase	hsa-miR-192-5p	MMAB	326625	ENSG00000139428	19074876	validated
mirtarbase	hsa-miR-15b-5p	H3C15	333932	ENSG00000203852	23622248	validated
mirtarbase	hsa-miR-192-5p	CHSY3	337876	ENSG00000198108	19074876	validated
mirtarbase	hsa-miR-106b-5p	LUZP2	338645	ENSG00000187398	21572407	validated
mirtarbase	hsa-miR-505-3p	ANKRD13D	338692	ENSG00000172932	23622248	validated
mirtarbase	hsa-miR-181a-5p	C1QTNF9	338872	ENSG00000240654	17612493	validated
mirtarbase	hsa-miR-106b-5p	CCDC137	339230	ENSG00000185298	22473208	validated
mirtarbase	hsa-miR-301a-3p	CCDC137	339230	ENSG00000185298	20371350	validated
mirtarbase	hsa-miR-15b-5p	MSL1	339287	ENSG00000188895	23446348	validated
mirtarbase	hsa-miR-582-5p	ZNF678	339500	ENSG00000181450	20371350	validated
mirtarbase	hsa-miR-18a-5p	ZNF678	339500	ENSG00000181450	21572407	validated
mirtarbase	hsa-miR-301a-3p	ZNF678	339500	ENSG00000181450	20371350	validated
mirtarbase	hsa-miR-106b-5p	SPOPL	339745	ENSG00000144228	22473208	validated
mirtarbase	hsa-miR-181a-5p	TMPRSS11A	339967	ENSG00000187054	17612493	validated
mirtarbase	hsa-miR-505-3p	ACER2	340485	ENSG00000177076	24398324	validated
mirtarbase	hsa-miR-106b-5p	ACER2	340485	ENSG00000177076	24398324	validated
mirtarbase	hsa-miR-582-5p	PABPC1L2A	340529	ENSG00000186288	23824327	validated
mirtarbase	hsa-miR-144-3p	DCAF12L2	340578	ENSG00000198354	22012620	validated
mirtarbase	hsa-miR-181a-5p	HEPHL1	341208	ENSG00000181333	22012620	validated
mirtarbase	hsa-miR-192-5p	GOLGA6A	342096	ENSG00000159289	16822819	validated
mirtarbase	hsa-miR-192-5p	TMC3	342125	ENSG00000188869	19074876	validated
mirtarbase	hsa-miR-144-3p	ATXN1L	342371	ENSG00000224470	21572407	validated
mirtarbase	hsa-miR-301a-3p	SMTNL2	342527	ENSG00000188176	23824327	validated
mirtarbase	hsa-miR-106b-5p	STAC2	342667	ENSG00000141750	22927820	validated
mirtarbase	hsa-miR-144-3p	ZNF284	342909	ENSG00000186026	20371350	validated
mirtarbase	hsa-miR-15b-5p	ZNF284	342909	ENSG00000186026	27418678	validated
mirtarbase	hsa-miR-181a-5p	PRAMEF9	343070	ENSG00000281573	27292025	validated
mirtarbase	hsa-miR-192-5p	CCDC18	343099	ENSG00000122483	19074876	validated
mirtarbase	hsa-miR-106b-5p	HSP90B3P	343477		17242205	validated
mirtarbase	hsa-miR-15b-5p	XKR7	343702	ENSG00000260903	20371350	validated
mirtarbase	hsa-miR-181a-5p	MTX3	345778	ENSG00000177034	22473208	validated
mirtarbase	hsa-miR-15b-5p	ZNF391	346157	ENSG00000124613	20371350	validated
mirtarbase	hsa-miR-192-5p	KIF24	347240	ENSG00000186638	19074876	validated
mirtarbase	hsa-miR-144-3p	LANCL3	347404	ENSG00000147036	27418678	validated
mirtarbase	hsa-miR-582-5p	ANKDD1A	348094	ENSG00000166839	27292025	validated
mirtarbase	hsa-miR-192-5p	GEN1	348654	ENSG00000178295	19074876	validated
mirtarbase	hsa-miR-106b-5p	WDR53	348793	ENSG00000185798	23824327	validated
mirtarbase	hsa-miR-181a-5p	ZNF445	353274	ENSG00000185219	20371350	validated
mirtarbase	hsa-miR-505-3p	ZNF445	353274	ENSG00000185219	23824327	validated
mirtarbase	hsa-miR-192-5p	TICAM2	353376	ENSG00000243414	19074876	validated
mirtarbase	hsa-miR-21-5p	TICAM2	353376	ENSG00000243414	25327529	validated
mirtarbase	hsa-miR-192-5p	RFLNB	359845	ENSG00000183688	19074876	validated
mirtarbase	hsa-miR-222-3p	IRF2BP2	359948	ENSG00000168264	23622248	validated
mirtarbase	hsa-miR-144-3p	NANOGNB	360030	ENSG00000205857	22012620	validated
mirtarbase	hsa-miR-192-5p	FAM111B	374393	ENSG00000189057	19074876	validated
mirtarbase	hsa-miR-106b-5p	DNAJB13	374407	ENSG00000187726	23313552	validated
mirtarbase	hsa-miR-144-3p	TEX9	374618	ENSG00000151575	19536157	validated

mirtarbase	hsa-miR-181a-5p	ZNF699	374879	ENSG00000196110	22012620	validated
mirtarbase	hsa-miR-106b-5p	SBSN	374897	ENSG00000189001	17242205	validated
mirtarbase	hsa-miR-181a-5p	ZNF829	374899	ENSG00000185869	23824327	validated
mirtarbase	hsa-miR-106b-5p	DRAXIN	374946	ENSG00000162490	23313552	validated
mirtarbase	hsa-miR-15b-5p	MIGA1	374986	ENSG00000180488	22473208	validated
mirtarbase	hsa-miR-326	STUM	375057	ENSG00000203685	19536157	validated
mirtarbase	hsa-miR-106b-5p	FAM89A	375061	ENSG00000182118	21572407	validated
mirtarbase	hsa-miR-505-3p	FAM89A	375061	ENSG00000182118	23824327	validated
mirtarbase	hsa-miR-15b-5p	ANKRD36	375248	ENSG00000135976	23824327	validated
mirtarbase	hsa-miR-301a-3p	RBM43	375287	ENSG00000184898	21572407	validated
mirtarbase	hsa-miR-192-5p	LHFPL4	375323	ENSG00000156959	20371350	validated
mirtarbase	hsa-miR-192-5p	C5orf34	375444	ENSG00000172244	19074876	validated
mirtarbase	hsa-miR-505-3p	GJB7	375519	ENSG00000164411	22012620	validated
mirtarbase	hsa-miR-181a-5p	GJB7	375519	ENSG00000164411	22012620	validated
mirtarbase	hsa-miR-21-5p	PTAR1	375743	ENSG00000188647	18591254	validated
mirtarbase	hsa-miR-15b-5p	PTAR1	375743	ENSG00000188647	23622248	validated
mirtarbase	hsa-miR-15b-5p	RAB15	376267	ENSG00000139998	19536157	validated
mirtarbase	hsa-miR-192-5p	CA13	377677	ENSG00000185015	19074876	validated
mirtarbase	hsa-miR-18a-5p	CA13	377677	ENSG00000185015	20371350	validated
mirtarbase	hsa-miR-505-3p	SOGA3	387104	ENSG00000214338	27418678	validated
mirtarbase	hsa-miR-192-5p	CEP85L	387119	ENSG00000111860	22100165	validated
mirtarbase	hsa-miR-106b-5p	C6orf120	387263	ENSG00000185127	23622248	validated
mirtarbase	hsa-miR-15b-5p	TMEM189	387521	ENSG00000240849	23592263	validated
mirtarbase	hsa-miR-15b-5p	TMEM189-UBE2V1	387522	ENSG00000124208	23592263	validated
mirtarbase	hsa-miR-301a-3p	CLEC12B	387837	ENSG00000256660	22012620	validated
mirtarbase	hsa-miR-106b-5p	CLEC12B	387837	ENSG00000256660	22012620	validated
mirtarbase	hsa-miR-106b-5p	SHISA2	387914	ENSG00000180730	23622248	validated
mirtarbase	hsa-miR-222-3p	SHISA2	387914	ENSG00000180730	23622248	validated
mirtarbase	hsa-miR-192-5p	NHLRC3	387921	ENSG00000188811	19074876	validated
mirtarbase	hsa-miR-106b-5p	NHLRC3	387921	ENSG00000188811	22473208	validated
mirtarbase	hsa-miR-181a-5p	NHLRC3	387921	ENSG00000188811	22473208	validated
mirtarbase	hsa-miR-21-5p	CCDC9B	388115	ENSG00000188549	23592263	validated
mirtarbase	hsa-miR-181a-5p	ZNF788	388507		24398324	validated
mirtarbase	hsa-miR-106b-5p	BLOC1S3	388552	ENSG00000189114	22473208	validated
mirtarbase	hsa-miR-144-3p	ZNF749	388567	ENSG00000186230	20371350	validated
mirtarbase	hsa-miR-144-3p	DIPK1A	388650	ENSG00000154511	20371350	validated
mirtarbase	hsa-miR-192-5p	C1orf53	388722	ENSG00000203724	19074876	validated
mirtarbase	hsa-miR-192-5p	VGLL3	389136	ENSG00000206538	19074876	validated
mirtarbase	hsa-miR-505-3p	BEND4	389206	ENSG00000188848	21572407	validated
mirtarbase	hsa-miR-15b-5p	LIN28B	389421	ENSG00000187772	23622248	validated
mirtarbase	hsa-miR-21-5p	SAMD5	389432	ENSG00000203727	18591254	validated
mirtarbase	hsa-miR-106b-5p	GTF2IRD2B	389524	ENSG00000174428	23313552	validated
mirtarbase	hsa-miR-106b-5p	NUGGC	389643	ENSG00000189233	23313552	validated
mirtarbase	hsa-miR-15b-5p	XKR9	389668	ENSG00000221947	23622248	validated
mirtarbase	hsa-miR-106b-5p	RBM12B	389677	ENSG00000183808	23622248	validated
mirtarbase	hsa-miR-582-5p	RBM12B	389677	ENSG00000183808	20371350	validated
mirtarbase	hsa-miR-106b-5p	OR52K1	390036	ENSG00000196778	17242205	validated
mirtarbase	hsa-miR-106b-5p	ZNF805	390980	ENSG00000204524	21572407	validated
mirtarbase	hsa-miR-222-3p	ZNF805	390980	ENSG00000204524	20371350	validated
mirtarbase	hsa-miR-106b-5p	FAM102A	399665	ENSG00000167106	22473208	validated
mirtarbase	hsa-miR-106b-5p	PATE2	399967	ENSG00000196844	23622248	validated
mirtarbase	hsa-miR-21-5p	PATE2	399967	ENSG00000196844	22012620	validated
mirtarbase	hsa-miR-15b-5p	MED11	400569	ENSG00000161920	22473208	validated
mirtarbase	hsa-miR-222-3p	ZNF772	400720	ENSG00000197128	23622248	validated
mirtarbase	hsa-miR-326	ZNF772	400720	ENSG00000197128	23446348	validated
mirtarbase	hsa-miR-181a-5p	PRAMEF4	400735	ENSG00000243073	27292025	validated
mirtarbase	hsa-miR-15b-5p	C1orf226	400793	ENSG00000239887	23592263	validated
mirtarbase	hsa-miR-192-5p	EML6	400954	ENSG00000214595	22100165	validated
mirtarbase	hsa-miR-106b-5p	LRRD1	401387	ENSG00000240720	23313552	validated
mirtarbase	hsa-miR-144-3p	SAMD12	401474	ENSG00000177570	21572407	validated
mirtarbase	hsa-miR-106b-5p	SAMD12	401474	ENSG00000177570	21572407	validated
mirtarbase	hsa-miR-326	TOMM5	401505	ENSG00000175768	23446348	validated
mirtarbase	hsa-miR-21-5p	SNX30	401548	ENSG00000148158	18591254	validated
mirtarbase	hsa-miR-192-5p	ZBTB34	403341	ENSG00000177125	19074876	validated
mirtarbase	hsa-miR-15b-5p	ZBTB34	403341	ENSG00000177125	23446348	validated
mirtarbase	hsa-miR-192-5p	LCN10	414332	ENSG00000187922	19536157	validated
mirtarbase	hsa-miR-15b-5p	CCDC88C	440193	ENSG00000015133	20371350	validated
mirtarbase	hsa-miR-181a-5p	CCDC88C	440193	ENSG00000015133	22473208	validated
mirtarbase	hsa-miR-181a-5p	GOLGA8B	440270	ENSG00000215252	22473208	validated
mirtarbase	hsa-miR-212-3p	ZNF724	440519	ENSG00000196081	20371350	validated
mirtarbase	hsa-miR-181a-5p	PRAMEF11	440560	ENSG00000239810	27292025	validated

mirtarbase	hsa-miR-106b-5p	ZYG11A	440590	ENSG00000203995	23313552	validated
mirtarbase	hsa-miR-192-5p	RP9P	441212		19074876	validated
mirtarbase	hsa-miR-582-3p	ZNF716	441234	ENSG00000182111	23824327	validated
mirtarbase	hsa-miR-582-5p	PGAM4	441531	ENSG00000226784	21572407	validated
mirtarbase	hsa-miR-106b-5p	C18orf32	497661	ENSG00000177576	20371350	validated
mirtarbase	hsa-miR-18a-5p	CXorf40B	541578	ENSG00000197021	23622248	validated
mirtarbase	hsa-miR-15b-5p	ATXN7L3B	552889	ENSG00000253719	23592263	validated
mirtarbase	hsa-miR-582-5p	ATXN7L3B	552889	ENSG00000253719	21572407	validated
mirtarbase	hsa-miR-106b-5p	ATXN7L3B	552889	ENSG00000253719	23592263	validated
mirtarbase	hsa-miR-106b-5p	GDF5OS	554250		23313552	validated
mirtarbase	hsa-miR-106b-5p	FBXO48	554251	ENSG00000204923	22473208	validated
mirtarbase	hsa-miR-505-3p	H4C15	554313	ENSG00000270276	23622248	validated
mirtarbase	hsa-miR-222-3p	H4C15	554313	ENSG00000270276	23622248	validated
mirtarbase	hsa-miR-144-3p	C1orf147	574431		23446348	validated
mirtarbase	hsa-miR-21-5p	C1orf147	574431		22012620	validated
mirtarbase	hsa-miR-15b-5p	FAM229B	619208	ENSG00000203778	21572407	validated
mirtarbase	hsa-miR-192-5p	ZNF704	619279	ENSG00000164684	19074876	validated
mirtarbase	hsa-miR-15b-5p	ZNF704	619279	ENSG00000164684	21572407	validated
mirtarbase	hsa-miR-192-5p	FAM110C	642273	ENSG00000184731	19074876	validated
mirtarbase	hsa-miR-181a-5p	FKBP1C	642489		22291592	validated
mirtarbase	hsa-miR-181a-5p	ZNF487	642819	ENSG00000243660	24398324	validated
mirtarbase	hsa-miR-582-3p	SMIM15	643155	ENSG00000188725	23824327	validated
mirtarbase	hsa-miR-21-5p	CCT6P1	643253		18591254	validated
mirtarbase	hsa-miR-326	MACF1	643314	ENSG00000127603	23824327	validated
mirtarbase	hsa-miR-222-3p	FAM83G	644815	ENSG00000188522	23622248	validated
mirtarbase	hsa-miR-181a-5p	PRAMEF25	645359	ENSG00000276299	27292025	validated
mirtarbase	hsa-miR-181a-5p	PRAMEF26	645359	ENSG00000280267	27292025	validated
mirtarbase	hsa-miR-106b-5p	TMEM200C	645369	ENSG00000206432	21572407	validated
mirtarbase	hsa-miR-106b-5p	LINC00598	646982		23313552	validated
mirtarbase	hsa-miR-192-5p	C6orf132	647024	ENSG00000188112	22012620	validated
mirtarbase	hsa-miR-106b-5p	ANKRD33B	651746	ENSG00000164236	23592263	validated
mirtarbase	hsa-miR-21-5p	ZBTB8A	653121	ENSG00000160062	18591254	validated
mirtarbase	hsa-miR-301a-3p	ZBTB8A	653121	ENSG00000160062	23824327	validated
mirtarbase	hsa-miR-301a-3p	SFTPA1	653509	ENSG00000122852	21572407	validated
mirtarbase	hsa-miR-15b-5p	PHLDB3	653583	ENSG00000176531	23622248	validated
mirtarbase	hsa-miR-181a-5p	PRAMEF15	653619	ENSG00000204501	27292025	validated
mirtarbase	hsa-miR-21-5p	GXYLT2	727936	ENSG00000172986	20371350	validated
mirtarbase	hsa-miR-222-3p	MROH1	727957	ENSG00000179832	23622248	validated
mirtarbase	hsa-miR-192-5p	ZBTB8B	728116	ENSG00000273274	23824327	validated
mirtarbase	hsa-miR-106b-5p	GTF2H2	728340	ENSG00000276910	23824327	validated
mirtarbase	hsa-miR-106b-5p	GTF2H2C	728340	ENSG00000183474	23824327	validated
mirtarbase	hsa-miR-296-5p	DNLZ	728489	ENSG00000213221	23592263	validated
mirtarbase	hsa-miR-18a-5p	WDR82P1	728505		23592263	validated
mirtarbase	hsa-miR-212-3p	CCDC169	728591	ENSG00000242715	22012620	validated
mirtarbase	hsa-miR-106b-5p	CCDC30	728621	ENSG00000186409	22927820	validated
mirtarbase	hsa-miR-301a-3p	SLC35E2B	728661	ENSG00000189339	23824327	validated
mirtarbase	hsa-miR-15b-5p	SLC35E2B	728661	ENSG00000189339	19536157	validated
mirtarbase	hsa-miR-505-3p	NBPF8	728841	ENSG00000270231	22100165	validated
mirtarbase	hsa-miR-192-5p	FAM182B	728882		23446348	validated
mirtarbase	hsa-miR-505-3p	CASTOR2	729438	ENSG00000274070	22100165	validated
mirtarbase	hsa-miR-326	CASTOR2	729438	ENSG00000274070	26701625	validated
mirtarbase	hsa-miR-106b-5p	TMEM242	729515	ENSG00000215712	23446348	validated
mirtarbase	hsa-miR-301a-3p	PRR23A	729627	ENSG00000206260	23824327	validated
mirtarbase	hsa-miR-192-5p	MOSMO	730094	ENSG00000185716	19074876	validated
mirtarbase	hsa-miR-106b-5p	MOSMO	730094	ENSG00000185716	21572407	validated
mirtarbase	hsa-miR-15b-5p	POM121C	100101267	ENSG00000272391	23446348	validated
mirtarbase	hsa-miR-326	HOTAIR	100124700	ENSG00000228630	26183397	validated
mirtarbase	hsa-miR-505-3p	PET117	100303755	ENSG00000232838	19536157	validated
mirtarbase	hsa-miR-301a-3p	ARL17B	100506084	ENSG00000276276	23824327	validated
mirtarbase	hsa-miR-212-3p	OCLN	100506658	ENSG00000273814	21572407	validated
mirtarbase	hsa-miR-326	C8orf17	100507249		23824327	validated
mirtarbase	hsa-miR-21-5p	C8orf17	100507249		23313552	validated
mirtarbase	hsa-miR-18a-5p	ARPIN-AP3S2	100526783	ENSG00000250021	20371350	validated
mirtarbase	hsa-miR-106b-5p	RPL17-C18orf32	100526842	ENSG00000215472	20371350	validated
mirtarbase	hsa-miR-301a-3p	POC1B-GALNT4	100528030	ENSG00000259075	24398324	validated
mirtarbase	hsa-miR-144-3p	BCL2L2-PABPN1	100529063	ENSG00000258643	23592263	validated
mirtarbase	hsa-miR-15b-5p	HSPE1-MOB4	100529241	ENSG00000270757	24398324	validated
mirtarbase	hsa-miR-144-3p	HSPE1-MOB4	100529241	ENSG00000270757	20371350	validated
mirtarbase	hsa-miR-144-3p	NDUFC2-KCTD14	100532726	ENSG00000259112	23592263	validated
mirtarbase	hsa-miR-181a-5p	RTEL1-TNFRSF6B	100533107		17612493	validated
tarbase	hsa-miR-21-5p	39701				validated

tarbase	hsa-miR-192-5p	AB002442			validated
tarbase	hsa-miR-192-5p	AB002443			validated
tarbase	hsa-miR-192-5p	AB033076			validated
tarbase	hsa-miR-192-5p	AB040883			validated
tarbase	hsa-miR-192-5p	AF075045			validated
tarbase	hsa-miR-192-5p	AF075069			validated
tarbase	hsa-miR-192-5p	AF078844			validated
tarbase	hsa-miR-192-5p	AF085923			validated
tarbase	hsa-miR-192-5p	AF086032			validated
tarbase	hsa-miR-192-5p	AF086173			validated
tarbase	hsa-miR-192-5p	AF086431			validated
tarbase	hsa-miR-192-5p	AF086561			validated
tarbase	hsa-miR-192-5p	AF125104			validated
tarbase	hsa-miR-192-5p	AF131784			validated
tarbase	hsa-miR-192-5p	AF131846			validated
tarbase	hsa-miR-192-5p	AJ227908			validated
tarbase	hsa-miR-192-5p	AK021796			validated
tarbase	hsa-miR-192-5p	AK022645			validated
tarbase	hsa-miR-192-5p	AK023367			validated
tarbase	hsa-miR-192-5p	AK024121			validated
tarbase	hsa-miR-192-5p	AK024556			validated
tarbase	hsa-miR-192-5p	AK024927			validated
tarbase	hsa-miR-192-5p	AK054764			validated
tarbase	hsa-miR-192-5p	AK055620			validated
tarbase	hsa-miR-192-5p	AK055807			validated
tarbase	hsa-miR-192-5p	AK057062			validated
tarbase	hsa-miR-192-5p	AK057167			validated
tarbase	hsa-miR-192-5p	AK057710			validated
tarbase	hsa-miR-192-5p	AK057835			validated
tarbase	hsa-miR-192-5p	AK075186			validated
tarbase	hsa-miR-192-5p	AK090904			validated
tarbase	hsa-miR-192-5p	AK092985			validated
tarbase	hsa-miR-192-5p	AK093202			validated
tarbase	hsa-miR-192-5p	AK095096			validated
tarbase	hsa-miR-192-5p	AK095149			validated
tarbase	hsa-miR-192-5p	AK098664			validated
tarbase	hsa-miR-21-5p	AKT-2			validated
tarbase	hsa-miR-192-5p	AL049354			validated
tarbase	hsa-miR-192-5p	AL049452			validated
tarbase	hsa-miR-192-5p	AL078636			validated
tarbase	hsa-miR-192-5p	AL110163			validated
tarbase	hsa-miR-192-5p	AL133090			validated
tarbase	hsa-miR-192-5p	AL133577			validated
tarbase	hsa-miR-192-5p	AL137310			validated
tarbase	hsa-miR-192-5p	AL137333			validated
tarbase	hsa-miR-192-5p	AL137347			validated
tarbase	hsa-miR-192-5p	AL137535			validated
tarbase	hsa-miR-192-5p	BC002811			validated
tarbase	hsa-miR-192-5p	BC004287			validated
tarbase	hsa-miR-192-5p	BC031660			validated
tarbase	hsa-miR-192-5p	BC031864			validated
tarbase	hsa-miR-192-5p	BC033326			validated
tarbase	hsa-miR-192-5p	BC039371			validated
tarbase	hsa-miR-192-5p	BC040287			validated
tarbase	hsa-miR-192-5p	BC041380			validated
tarbase	hsa-miR-192-5p	BX487921			validated
tarbase	hsa-miR-21-5p	MMP16	4325	ENSG00000156103	validated
tarbase	hsa-miR-21-5p	ACAP2	23527	ENSG00000114331	validated
tarbase	hsa-miR-18a-5p	SCYL3	57147	ENSG00000000457	validated
tarbase	hsa-miR-212-3p	SCYL3	57147	ENSG00000000457	validated
tarbase	hsa-miR-21-5p	SCYL3	57147	ENSG00000000457	validated
tarbase	hsa-miR-21-5p	C1orf112	55732	ENSG00000000460	validated
tarbase	hsa-miR-15b-5p	FUCA2	2519	ENSG00000001036	validated
tarbase	hsa-miR-18a-5p	GCLC	2729	ENSG00000001084	validated
tarbase	hsa-miR-21-5p	GCLC	2729	ENSG00000001084	validated
tarbase	hsa-miR-212-3p	NFYA	4800	ENSG00000001167	validated
tarbase	hsa-miR-301a-3p	NFYA	4800	ENSG00000001167	validated
tarbase	hsa-miR-18a-5p	NIPAL3	57185	ENSG00000001461	validated
tarbase	hsa-miR-106b-5p	ENPP4	22875	ENSG00000001561	validated
tarbase	hsa-miR-582-5p	ENPP4	22875	ENSG00000001561	validated
tarbase	hsa-miR-15b-5p	ENPP4	22875	ENSG00000001561	validated

tarbase	hsa-miR-15b-5p	ANKIB1	54467	ENSG00000001629	validated
tarbase	hsa-miR-181a-5p	ANKIB1	54467	ENSG00000001629	validated
tarbase	hsa-miR-212-3p	ANKIB1	54467	ENSG00000001629	validated
tarbase	hsa-miR-301a-3p	ANKIB1	54467	ENSG00000001629	validated
tarbase	hsa-miR-582-5p	CYP51A1	1595	ENSG00000001630	validated
tarbase	hsa-miR-181a-5p	KRIT1	889	ENSG00000001631	validated
tarbase	hsa-miR-192-5p	KRIT1	889	ENSG00000001631	validated
tarbase	hsa-miR-301a-3p	CD99	4267	ENSG00000002586	validated
tarbase	hsa-miR-326	HS3ST1	9957	ENSG00000002587	validated
tarbase	hsa-miR-18a-5p	M6PR	4074	ENSG00000003056	validated
tarbase	hsa-miR-301a-3p	DBNDD1	79007	ENSG00000003249	validated
tarbase	hsa-miR-15b-5p	ALS2	57679	ENSG00000003393	validated
tarbase	hsa-miR-21-5p	ALS2	57679	ENSG00000003393	validated
tarbase	hsa-miR-15b-5p	CFLAR	8837	ENSG00000003402	validated
tarbase	hsa-miR-212-3p	CFLAR	8837	ENSG00000003402	validated
tarbase	hsa-miR-326	CFLAR	8837	ENSG00000003402	validated
tarbase	hsa-miR-15b-5p	TFPI	7035	ENSG00000003436	validated
tarbase	hsa-miR-181a-5p	TFPI	7035	ENSG00000003436	validated
tarbase	hsa-miR-181a-5p	SLC7A2	6542	ENSG00000003989	validated
tarbase	hsa-miR-15b-5p	SARM1	23098	ENSG00000004139	validated
tarbase	hsa-miR-106b-5p	POLDIP2	26073	ENSG00000004142	validated
tarbase	hsa-miR-212-3p	AK2	204	ENSG00000004455	validated
tarbase	hsa-miR-301a-3p	AK2	204	ENSG00000004455	validated
tarbase	hsa-miR-106b-5p	CD38	952	ENSG00000004468	validated
tarbase	hsa-miR-21-5p	CD38	952	ENSG00000004468	validated
tarbase	hsa-miR-181a-5p	FKBP4	2288	ENSG00000004478	validated
tarbase	hsa-miR-212-3p	FKBP4	2288	ENSG00000004478	validated
tarbase	hsa-miR-106b-5p	KDM1A	23028	ENSG00000004487	validated
tarbase	hsa-miR-18a-5p	KDM1A	23028	ENSG00000004487	validated
tarbase	hsa-miR-15b-5p	RBM6	10180	ENSG00000004534	validated
tarbase	hsa-miR-181a-5p	RBM6	10180	ENSG00000004534	validated
tarbase	hsa-miR-181a-5p	NDUFAB1	4706	ENSG00000004779	validated
tarbase	hsa-miR-106b-5p	PDK4	5166	ENSG00000004799	validated
tarbase	hsa-miR-326	SLC25A13	10165	ENSG00000004864	validated
tarbase	hsa-miR-582-5p	ST7	7982	ENSG00000004866	validated
tarbase	hsa-miR-181a-5p	CDC27	996	ENSG00000004897	validated
tarbase	hsa-miR-582-5p	CDC27	996	ENSG00000004897	validated
tarbase	hsa-miR-212-3p	CDC27	996	ENSG00000004897	validated
tarbase	hsa-miR-18a-5p	HCCS	3052	ENSG00000004961	validated
tarbase	hsa-miR-301a-3p	HCCS	3052	ENSG00000004961	validated
tarbase	hsa-miR-181a-5p	DVL2	1856	ENSG00000004975	validated
tarbase	hsa-miR-582-3p	SKAP2	8935	ENSG00000005020	validated
tarbase	hsa-miR-106b-5p	DHX33	56919	ENSG00000005100	validated
tarbase	hsa-miR-15b-5p	DHX33	56919	ENSG00000005100	validated
tarbase	hsa-miR-181a-5p	DHX33	56919	ENSG00000005100	validated
tarbase	hsa-miR-582-3p	DHX33	56919	ENSG00000005100	validated
tarbase	hsa-miR-296-5p	RPAP3	79657	ENSG00000005175	validated
tarbase	hsa-miR-181a-5p	RPAP3	79657	ENSG00000005175	validated
tarbase	hsa-miR-212-3p	REXO5	81691	ENSG00000005189	validated
tarbase	hsa-miR-15b-5p	SPPL2B	56928	ENSG00000005206	validated
tarbase	hsa-miR-326	SPPL2B	56928	ENSG00000005206	validated
tarbase	hsa-miR-212-3p	COPZ2	51226	ENSG00000005243	validated
tarbase	hsa-miR-106b-5p	PRKAR2B	5577	ENSG00000005249	validated
tarbase	hsa-miR-181a-5p	PRKAR2B	5577	ENSG00000005249	validated
tarbase	hsa-miR-15b-5p	CREBBP	1387	ENSG00000005339	validated
tarbase	hsa-miR-106b-5p	KMT2E	55904	ENSG00000005483	validated
tarbase	hsa-miR-144-3p	KMT2E	55904	ENSG00000005483	validated
tarbase	hsa-miR-582-5p	KMT2E	55904	ENSG00000005483	validated
tarbase	hsa-miR-181a-5p	IBTK	25998	ENSG00000005700	validated
tarbase	hsa-miR-18a-5p	IBTK	25998	ENSG00000005700	validated
tarbase	hsa-miR-181a-5p	MYCBP2	23077	ENSG00000005810	validated
tarbase	hsa-miR-301a-3p	MYCBP2	23077	ENSG00000005810	validated
tarbase	hsa-miR-106b-5p	FBXL3	26224	ENSG00000005812	validated
tarbase	hsa-miR-181a-5p	FBXL3	26224	ENSG00000005812	validated
tarbase	hsa-miR-212-3p	FBXL3	26224	ENSG00000005812	validated
tarbase	hsa-miR-301a-3p	FBXL3	26224	ENSG00000005812	validated
tarbase	hsa-miR-21-5p	FBXL3	26224	ENSG00000005812	validated
tarbase	hsa-miR-192-5p	ZFX	7543	ENSG00000005889	validated
tarbase	hsa-miR-582-3p	ZFX	7543	ENSG00000005889	validated
tarbase	hsa-miR-301a-3p	LAMP2	3920	ENSG00000005893	validated
tarbase	hsa-miR-15b-5p	GDE1	51573	ENSG00000006007	validated

tarbase	hsa-miR-15b-5p	OSBPL7	114881	ENSG00000006025	validated
tarbase	hsa-miR-301a-3p	MAP3K14	9020	ENSG00000006062	validated
tarbase	hsa-miR-212-3p	CACNG3	10368	ENSG00000006116	validated
tarbase	hsa-miR-181a-5p	AP2B1	163	ENSG00000006125	validated
tarbase	hsa-miR-212-3p	TAC1	6863	ENSG00000006128	validated
tarbase	hsa-miR-15b-5p	TNFRSF12A	51330	ENSG00000006327	validated
tarbase	hsa-miR-106b-5p	MAP3K9	4293	ENSG00000006432	validated
tarbase	hsa-miR-15b-5p	MAP3K9	4293	ENSG00000006432	validated
tarbase	hsa-miR-18a-5p	MAP3K9	4293	ENSG00000006432	validated
tarbase	hsa-miR-21-5p	RALA	5898	ENSG00000006451	validated
tarbase	hsa-miR-106b-5p	ETV1	2115	ENSG00000006468	validated
tarbase	hsa-miR-582-5p	ETV1	2115	ENSG00000006468	validated
tarbase	hsa-miR-222-3p	ETV1	2115	ENSG00000006468	validated
tarbase	hsa-miR-15b-5p	AGK	55750	ENSG00000006530	validated
tarbase	hsa-miR-21-5p	TTC22	55001	ENSG00000006555	validated
tarbase	hsa-miR-181a-5p	PHTF2	57157	ENSG00000006576	validated
tarbase	hsa-miR-212-3p	PHTF2	57157	ENSG00000006576	validated
tarbase	hsa-miR-106b-5p	FARP2	9855	ENSG00000006607	validated
tarbase	hsa-miR-181a-5p	FARP2	9855	ENSG00000006607	validated
tarbase	hsa-miR-181a-5p	GGCT	79017	ENSG00000006625	validated
tarbase	hsa-miR-222-3p	GGCT	79017	ENSG00000006625	validated
tarbase	hsa-miR-301a-3p	GGCT	79017	ENSG00000006625	validated
tarbase	hsa-miR-106b-5p	IFRD1	3475	ENSG00000006652	validated
tarbase	hsa-miR-21-5p	LGALS14	56891	ENSG00000006659	validated
tarbase	hsa-miR-106b-5p	COX10	1352	ENSG00000006695	validated
tarbase	hsa-miR-106b-5p	PAF1	54623	ENSG00000006712	validated
tarbase	hsa-miR-582-3p	PAF1	54623	ENSG00000006712	validated
tarbase	hsa-miR-21-5p	VPS41	27072	ENSG00000006715	validated
tarbase	hsa-miR-505-3p	VPS41	27072	ENSG00000006715	validated
tarbase	hsa-miR-15b-5p	PNPLA4	8228	ENSG00000006757	validated
tarbase	hsa-miR-144-3p	ADIPOR2	79602	ENSG00000006831	validated
tarbase	hsa-miR-181a-5p	ADIPOR2	79602	ENSG00000006831	validated
tarbase	hsa-miR-582-5p	ADIPOR2	79602	ENSG00000006831	validated
tarbase	hsa-miR-21-5p	PAFAH1B1	5048	ENSG00000007168	validated
tarbase	hsa-miR-301a-3p	PAFAH1B1	5048	ENSG00000007168	validated
tarbase	hsa-miR-326	PAFAH1B1	5048	ENSG00000007168	validated
tarbase	hsa-miR-181a-5p	PAFAH1B1	5048	ENSG00000007168	validated
tarbase	hsa-miR-15b-5p	KIAA0100	9703	ENSG00000007202	validated
tarbase	hsa-miR-192-5p	KIAA0100	9703	ENSG00000007202	validated
tarbase	hsa-miR-212-3p	ST7L	54879	ENSG00000007341	validated
tarbase	hsa-miR-15b-5p	CRAMP1	57585	ENSG00000007545	validated
tarbase	hsa-miR-296-5p	CRAMP1	57585	ENSG00000007545	validated
tarbase	hsa-miR-106b-5p	TEAD3	7005	ENSG00000007866	validated
tarbase	hsa-miR-505-3p	DNAJC11	55735	ENSG00000007923	validated
tarbase	hsa-miR-15b-5p	MYLIP	29116	ENSG00000007944	validated
tarbase	hsa-miR-192-5p	MYLIP	29116	ENSG00000007944	validated
tarbase	hsa-miR-301a-3p	E2F2	1870	ENSG00000007968	validated
tarbase	hsa-miR-326	E2F2	1870	ENSG00000007968	validated
tarbase	hsa-miR-18a-5p	PSMB1	5689	ENSG00000008018	validated
tarbase	hsa-miR-181a-5p	JARID2	3720	ENSG00000008083	validated
tarbase	hsa-miR-582-5p	JARID2	3720	ENSG00000008083	validated
tarbase	hsa-miR-21-5p	CDK11A	728642	ENSG00000008128	validated
tarbase	hsa-miR-296-5p	CYTH3	9265	ENSG00000008256	validated
tarbase	hsa-miR-21-5p	ADAM22	53616	ENSG00000008277	validated
tarbase	hsa-miR-181a-5p	SYPL1	6856	ENSG00000008282	validated
tarbase	hsa-miR-21-5p	SYPL1	6856	ENSG00000008282	validated
tarbase	hsa-miR-301a-3p	CYB561	1534	ENSG00000008283	validated
tarbase	hsa-miR-106b-5p	SPAG9	9043	ENSG00000008294	validated
tarbase	hsa-miR-222-3p	SPAG9	9043	ENSG00000008294	validated
tarbase	hsa-miR-15b-5p	SPAG9	9043	ENSG00000008294	validated
tarbase	hsa-miR-181a-5p	SPAG9	9043	ENSG00000008294	validated
tarbase	hsa-miR-212-3p	AASS	10157	ENSG00000008311	validated
tarbase	hsa-miR-15b-5p	AASS	10157	ENSG00000008311	validated
tarbase	hsa-miR-181a-5p	SS18L2	51188	ENSG00000008324	validated
tarbase	hsa-miR-301a-3p	MGST1	4257	ENSG00000008394	validated
tarbase	hsa-miR-212-3p	MMP25	64386	ENSG00000008516	validated
tarbase	hsa-miR-212-3p	IL32	9235	ENSG00000008517	validated
tarbase	hsa-miR-106b-5p	PKD1	5310	ENSG00000008710	validated
tarbase	hsa-miR-18a-5p	PKD1	5310	ENSG00000008710	validated
tarbase	hsa-miR-326	PKD1	5310	ENSG00000008710	validated
tarbase	hsa-miR-582-3p	PKD1	5310	ENSG00000008710	validated

tarbase	hsa-miR-18a-5p	MAPK8IP2	23542	ENSG00000008735	validated
tarbase	hsa-miR-192-5p	MAPK8IP2	23542	ENSG00000008735	validated
tarbase	hsa-miR-505-3p	SEC62	7095	ENSG00000008952	validated
tarbase	hsa-miR-212-3p	RPS20	6224	ENSG00000008988	validated
tarbase	hsa-miR-582-3p	RPS20	6224	ENSG00000008988	validated
tarbase	hsa-miR-18a-5p	CSDE1	7812	ENSG00000009307	validated
tarbase	hsa-miR-212-3p	CSDE1	7812	ENSG00000009307	validated
tarbase	hsa-miR-21-5p	CSDE1	7812	ENSG00000009307	validated
tarbase	hsa-miR-296-5p	CSDE1	7812	ENSG00000009307	validated
tarbase	hsa-miR-301a-3p	CSDE1	7812	ENSG00000009307	validated
tarbase	hsa-miR-106b-5p	UBE3C	9690	ENSG00000009335	validated
tarbase	hsa-miR-181a-5p	UBE3C	9690	ENSG00000009335	validated
tarbase	hsa-miR-18a-5p	UBE3C	9690	ENSG00000009335	validated
tarbase	hsa-miR-222-3p	UBE3C	9690	ENSG00000009335	validated
tarbase	hsa-miR-301a-3p	UBE3C	9690	ENSG00000009335	validated
tarbase	hsa-miR-582-3p	UBE3C	9690	ENSG00000009335	validated
tarbase	hsa-miR-106b-5p	REV3L	5980	ENSG00000009413	validated
tarbase	hsa-miR-15b-5p	REV3L	5980	ENSG00000009413	validated
tarbase	hsa-miR-18a-5p	REV3L	5980	ENSG00000009413	validated
tarbase	hsa-miR-212-3p	REV3L	5980	ENSG00000009413	validated
tarbase	hsa-miR-222-3p	REV3L	5980	ENSG00000009413	validated
tarbase	hsa-miR-106b-5p	VTA1	51534	ENSG00000009844	validated
tarbase	hsa-miR-15b-5p	VTA1	51534	ENSG00000009844	validated
tarbase	hsa-miR-181a-5p	VTA1	51534	ENSG00000009844	validated
tarbase	hsa-miR-296-5p	MLXIPL	51085	ENSG00000009950	validated
tarbase	hsa-miR-18a-5p	BAZ1B	9031	ENSG00000009954	validated
tarbase	hsa-miR-296-5p	BAZ1B	9031	ENSG00000009954	validated
tarbase	hsa-miR-144-3p	RANBP9	10048	ENSG00000010017	validated
tarbase	hsa-miR-15b-5p	RANBP9	10048	ENSG00000010017	validated
tarbase	hsa-miR-582-5p	RANBP9	10048	ENSG00000010017	validated
tarbase	hsa-miR-15b-5p	SPRTN	83932	ENSG00000010072	validated
tarbase	hsa-miR-106b-5p	ZNF207	7756	ENSG00000010244	validated
tarbase	hsa-miR-15b-5p	ZNF207	7756	ENSG00000010244	validated
tarbase	hsa-miR-212-3p	ZNF207	7756	ENSG00000010244	validated
tarbase	hsa-miR-582-3p	ZNF207	7756	ENSG00000010244	validated
tarbase	hsa-miR-181a-5p	ZNF207	7756	ENSG00000010244	validated
tarbase	hsa-miR-192-5p	STARD3NL	83930	ENSG00000010270	validated
tarbase	hsa-miR-15b-5p	NCAPD2	9918	ENSG00000010292	validated
tarbase	hsa-miR-582-5p	NISCH	11188	ENSG00000010322	validated
tarbase	hsa-miR-18a-5p	IDS	3423	ENSG00000010404	validated
tarbase	hsa-miR-582-3p	IDS	3423	ENSG00000010404	validated
tarbase	hsa-miR-181a-5p	ZNF200	7752	ENSG00000010539	validated
tarbase	hsa-miR-326	ZNF200	7752	ENSG00000010539	validated
tarbase	hsa-miR-222-3p	CD4	920	ENSG00000010610	validated
tarbase	hsa-miR-21-5p	HFE	3077	ENSG00000010704	validated
tarbase	hsa-miR-21-5p	FYN	2534	ENSG00000010810	validated
tarbase	hsa-miR-15b-5p	HIVEP2	3097	ENSG00000010818	validated
tarbase	hsa-miR-106b-5p	ELOA	6924	ENSG00000011007	validated
tarbase	hsa-miR-181a-5p	ELOA	6924	ENSG00000011007	validated
tarbase	hsa-miR-301a-3p	ELOA	6924	ENSG00000011007	validated
tarbase	hsa-miR-582-3p	ELOA	6924	ENSG00000011007	validated
tarbase	hsa-miR-15b-5p	LYPLA2	11313	ENSG00000011009	validated
tarbase	hsa-miR-181a-5p	BTBD7	55727	ENSG00000011114	validated
tarbase	hsa-miR-212-3p	BTBD7	55727	ENSG00000011114	validated
tarbase	hsa-miR-222-3p	BTBD7	55727	ENSG00000011114	validated
tarbase	hsa-miR-301a-3p	BTBD7	55727	ENSG00000011114	validated
tarbase	hsa-miR-582-3p	BTBD7	55727	ENSG00000011114	validated
tarbase	hsa-miR-326	MKS1	54903	ENSG00000011143	validated
tarbase	hsa-miR-106b-5p	ABHD5	51099	ENSG00000011198	validated
tarbase	hsa-miR-106b-5p	TTC19	54902	ENSG00000011295	validated
tarbase	hsa-miR-106b-5p	PTBP1	5725	ENSG00000011304	validated
tarbase	hsa-miR-192-5p	PTBP1	5725	ENSG00000011304	validated
tarbase	hsa-miR-21-5p	PTBP1	5725	ENSG00000011304	validated
tarbase	hsa-miR-505-3p	PTBP1	5725	ENSG00000011304	validated
tarbase	hsa-miR-582-3p	PTBP1	5725	ENSG00000011304	validated
tarbase	hsa-miR-582-3p	PIK3C2A	5286	ENSG00000011405	validated
tarbase	hsa-miR-582-5p	PIK3C2A	5286	ENSG00000011405	validated
tarbase	hsa-miR-181a-5p	PIK3C2A	5286	ENSG00000011405	validated
tarbase	hsa-miR-144-3p	ANLN	54443	ENSG00000011426	validated
tarbase	hsa-miR-15b-5p	ANLN	54443	ENSG00000011426	validated
tarbase	hsa-miR-21-5p	ANLN	54443	ENSG00000011426	validated

tarbase	hsa-miR-582-5p	ANLN	54443	ENSG00000011426	validated
tarbase	hsa-miR-212-3p	ANLN	54443	ENSG00000011426	validated
tarbase	hsa-miR-181a-5p	WIZ	58525	ENSG00000011451	validated
tarbase	hsa-miR-181a-5p			ENSG00000011454	validated
tarbase	hsa-miR-21-5p			ENSG00000011454	validated
tarbase	hsa-miR-301a-3p			ENSG00000011454	validated
tarbase	hsa-miR-222-3p	DCN	1634	ENSG00000011465	validated
tarbase	hsa-miR-301a-3p	CEP68	23177	ENSG00000011523	validated
tarbase	hsa-miR-181a-5p	MAP4K3	8491	ENSG00000011566	validated
tarbase	hsa-miR-582-5p	MAP4K3	8491	ENSG00000011566	validated
tarbase	hsa-miR-301a-3p	MAP4K3	8491	ENSG00000011566	validated
tarbase	hsa-miR-15b-5p	TMEM159	57146	ENSG00000011638	validated
tarbase	hsa-miR-212-3p	TMEM159	57146	ENSG00000011638	validated
tarbase	hsa-miR-15b-5p	MBTPS2	51360	ENSG00000012174	validated
tarbase	hsa-miR-21-5p	MBTPS2	51360	ENSG00000012174	validated
tarbase	hsa-miR-15b-5p	ELOVL5	60481	ENSG00000012660	validated
tarbase	hsa-miR-18a-5p	ELOVL5	60481	ENSG00000012660	validated
tarbase	hsa-miR-301a-3p	UBR7	55148	ENSG00000012963	validated
tarbase	hsa-miR-582-3p	UBR7	55148	ENSG00000012963	validated
tarbase	hsa-miR-212-3p	MAP4K5	11183	ENSG00000012983	validated
tarbase	hsa-miR-106b-5p	EHD3	30845	ENSG00000013016	validated
tarbase	hsa-miR-222-3p	CLDN11	5010	ENSG00000013297	validated
tarbase	hsa-miR-15b-5p	SLC25A39	51629	ENSG00000013306	validated
tarbase	hsa-miR-212-3p	MVP	9961	ENSG00000013364	validated
tarbase	hsa-miR-181a-5p	NUB1	51667	ENSG00000013374	validated
tarbase	hsa-miR-582-3p	NUB1	51667	ENSG00000013374	validated
tarbase	hsa-miR-21-5p	PGM3	5238	ENSG00000013375	validated
tarbase	hsa-miR-212-3p	DNASE1L1	1774	ENSG00000013563	validated
tarbase	hsa-miR-18a-5p	GPRC5A	9052	ENSG00000013588	validated
tarbase	hsa-miR-21-5p	GPRC5A	9052	ENSG00000013588	validated
tarbase	hsa-miR-582-5p	GPRC5A	9052	ENSG00000013588	validated
tarbase	hsa-miR-18a-5p	MAMLD1	10046	ENSG00000013619	validated
tarbase	hsa-miR-212-3p	MAMLD1	10046	ENSG00000013619	validated
tarbase	hsa-miR-181a-5p	POLA2	23649	ENSG00000014138	validated
tarbase	hsa-miR-222-3p	COX15	1355	ENSG00000014919	validated
tarbase	hsa-miR-15b-5p	ZMYND11	10771	ENSG00000015171	validated
tarbase	hsa-miR-181a-5p	ZMYND11	10771	ENSG00000015171	validated
tarbase	hsa-miR-21-5p	ZMYND11	10771	ENSG00000015171	validated
tarbase	hsa-miR-505-3p	ZMYND11	10771	ENSG00000015171	validated
tarbase	hsa-miR-21-5p	BID	637	ENSG00000015475	validated
tarbase	hsa-miR-106b-5p	MATR3	9782	ENSG00000015479	validated
tarbase	hsa-miR-15b-5p	MATR3	9782	ENSG00000015479	validated
tarbase	hsa-miR-296-5p	MATR3	9782	ENSG00000015479	validated
tarbase	hsa-miR-144-3p	NPC1L1	29881	ENSG00000015520	validated
tarbase	hsa-miR-301a-3p	XYLT2	64132	ENSG00000015532	validated
tarbase	hsa-miR-326	XYLT2	64132	ENSG00000015532	validated
tarbase	hsa-miR-15b-5p	CLCA1	1179	ENSG00000016490	validated
tarbase	hsa-miR-144-3p	ATP2C1	27032	ENSG00000017260	validated
tarbase	hsa-miR-301a-3p	ATP2C1	27032	ENSG00000017260	validated
tarbase	hsa-miR-15b-5p	ATP2C1	27032	ENSG00000017260	validated
tarbase	hsa-miR-181a-5p	RALBP1	10928	ENSG00000017797	validated
tarbase	hsa-miR-181a-5p	CNTN1	1272	ENSG00000018236	validated
tarbase	hsa-miR-582-3p	WWTR1	25937	ENSG00000018408	validated
tarbase	hsa-miR-582-5p	WWTR1	25937	ENSG00000018408	validated
tarbase	hsa-miR-106b-5p	CXorf56	63932	ENSG00000018610	validated
tarbase	hsa-miR-181a-5p	TTC27	55622	ENSG00000018699	validated
tarbase	hsa-miR-582-3p	TTC27	55622	ENSG00000018699	validated
tarbase	hsa-miR-18a-5p	PRDM11	56981	ENSG00000019485	validated
tarbase	hsa-miR-21-5p	PRDM11	56981	ENSG00000019485	validated
tarbase	hsa-miR-15b-5p	HGF	3082	ENSG00000019991	validated
tarbase	hsa-miR-222-3p	ZRANB1	54764	ENSG00000019995	validated
tarbase	hsa-miR-21-5p	SAMD4A	23034	ENSG00000020577	validated
tarbase	hsa-miR-21-5p	MRE11	4361	ENSG00000020922	validated
tarbase	hsa-miR-15b-5p	SPAST	6683	ENSG00000021574	validated
tarbase	hsa-miR-212-3p	SPAST	6683	ENSG00000021574	validated
tarbase	hsa-miR-212-3p	NRXN3	9369	ENSG00000021645	validated
tarbase	hsa-miR-181a-5p	AQR	9716	ENSG00000021776	validated
tarbase	hsa-miR-21-5p	CPS1	1373	ENSG00000021826	validated
tarbase	hsa-miR-582-5p	CPS1	1373	ENSG00000021826	validated
tarbase	hsa-miR-582-5p	FHL1	2273	ENSG00000022267	validated
tarbase	hsa-miR-212-3p	FHL1	2273	ENSG00000022267	validated

tarbase	hsa-miR-106b-5p	RTF2	51507	ENSG00000022277	validated
tarbase	hsa-miR-15b-5p	GABRA1	2554	ENSG00000022355	validated
tarbase	hsa-miR-181a-5p	GABRA1	2554	ENSG00000022355	validated
tarbase	hsa-miR-106b-5p	RNH1	6050	ENSG00000023191	validated
tarbase	hsa-miR-21-5p	NDUFS1	4719	ENSG00000023228	validated
tarbase	hsa-miR-15b-5p	NDUFS1	4719	ENSG00000023228	validated
tarbase	hsa-miR-192-5p	RB1CC1	9821	ENSG00000023287	validated
tarbase	hsa-miR-212-3p	RB1CC1	9821	ENSG00000023287	validated
tarbase	hsa-miR-301a-3p	ERP44	23071	ENSG00000023318	validated
tarbase	hsa-miR-582-5p	ERP44	23071	ENSG00000023318	validated
tarbase	hsa-miR-222-3p	BIRC3	330	ENSG00000023445	validated
tarbase	hsa-miR-582-5p	BIRC3	330	ENSG00000023445	validated
tarbase	hsa-miR-21-5p	BIRC3	330	ENSG00000023445	validated
tarbase	hsa-miR-296-5p	AKAP11	11215	ENSG00000023516	validated
tarbase	hsa-miR-582-5p	AKAP11	11215	ENSG00000023516	validated
tarbase	hsa-miR-181a-5p	AKAP11	11215	ENSG00000023516	validated
tarbase	hsa-miR-212-3p	DERA	51071	ENSG00000023697	validated
tarbase	hsa-miR-582-5p	GCLM	2730	ENSG00000023909	validated
tarbase	hsa-miR-106b-5p	DEPDC1	55635	ENSG00000024526	validated
tarbase	hsa-miR-212-3p	DEPDC1	55635	ENSG00000024526	validated
tarbase	hsa-miR-15b-5p	RRAGD	58528	ENSG00000025039	validated
tarbase	hsa-miR-181a-5p	RRAGD	58528	ENSG00000025039	validated
tarbase	hsa-miR-21-5p	HSF2	3298	ENSG00000025156	validated
tarbase	hsa-miR-212-3p	HSF2	3298	ENSG00000025156	validated
tarbase	hsa-miR-212-3p	PHF20	51230	ENSG00000025293	validated
tarbase	hsa-miR-301a-3p	PHF20	51230	ENSG00000025293	validated
tarbase	hsa-miR-212-3p	HSD17B6	8630	ENSG00000025423	validated
tarbase	hsa-miR-212-3p	NR1H3	10062	ENSG00000025434	validated
tarbase	hsa-miR-106b-5p	SEC63	11231	ENSG00000025796	validated
tarbase	hsa-miR-181a-5p	KPNA6	23633	ENSG00000025800	validated
tarbase	hsa-miR-18a-5p	KPNA6	23633	ENSG00000025800	validated
tarbase	hsa-miR-326	KPNA6	23633	ENSG00000025800	validated
tarbase	hsa-miR-301a-3p	KPNA6	23633	ENSG00000025800	validated
tarbase	hsa-miR-15b-5p	VIM	7431	ENSG00000026025	validated
tarbase	hsa-miR-21-5p	VIM	7431	ENSG00000026025	validated
tarbase	hsa-miR-296-5p	RNASET2	8635	ENSG00000026297	validated
tarbase	hsa-miR-21-5p	CD44	960	ENSG00000026508	validated
tarbase	hsa-miR-212-3p	CD44	960	ENSG00000026508	validated
tarbase	hsa-miR-192-5p	SLAMF7	57823	ENSG00000026751	validated
tarbase	hsa-miR-212-3p	MIPEP	4285	ENSG00000027001	validated
tarbase	hsa-miR-18a-5p	INSRR	3645	ENSG00000027644	validated
tarbase	hsa-miR-212-3p	SH2D2A	9047	ENSG00000027869	validated
tarbase	hsa-miR-106b-5p	VEZT	55591	ENSG00000028203	validated
tarbase	hsa-miR-15b-5p	VEZT	55591	ENSG00000028203	validated
tarbase	hsa-miR-18a-5p	VEZT	55591	ENSG00000028203	validated
tarbase	hsa-miR-582-3p	VEZT	55591	ENSG00000028203	validated
tarbase	hsa-miR-21-5p	VEZT	55591	ENSG00000028203	validated
tarbase	hsa-miR-326	BRD9	65980	ENSG00000028310	validated
tarbase	hsa-miR-18a-5p	SNX1	6642	ENSG00000028528	validated
tarbase	hsa-miR-181a-5p	TBPL1	9519	ENSG00000028839	validated
tarbase	hsa-miR-582-5p	TBPL1	9519	ENSG00000028839	validated
tarbase	hsa-miR-106b-5p	ARNTL2	56938	ENSG00000029153	validated
tarbase	hsa-miR-181a-5p	BCLAF1	9774	ENSG00000029363	validated
tarbase	hsa-miR-106b-5p	SLC39A9	55334	ENSG00000029364	validated
tarbase	hsa-miR-222-3p	SLC39A9	55334	ENSG00000029364	validated
tarbase	hsa-miR-582-3p	SLC39A9	55334	ENSG00000029364	validated
tarbase	hsa-miR-181a-5p	TFB1M	51106	ENSG00000029639	validated
tarbase	hsa-miR-296-5p	TFB1M	51106	ENSG00000029639	validated
tarbase	hsa-miR-18a-5p	RABEP1	9135	ENSG00000029725	validated
tarbase	hsa-miR-296-5p	RABEP1	9135	ENSG00000029725	validated
tarbase	hsa-miR-181a-5p	HMGB3	3149	ENSG00000029993	validated
tarbase	hsa-miR-18a-5p	NUP160	23279	ENSG00000030066	validated
tarbase	hsa-miR-301a-3p	NUP160	23279	ENSG00000030066	validated
tarbase	hsa-miR-212-3p	NUP160	23279	ENSG00000030066	validated
tarbase	hsa-miR-296-5p	BAK1	578	ENSG00000030110	validated
tarbase	hsa-miR-301a-3p	IKZF2	22807	ENSG00000030419	validated
tarbase	hsa-miR-505-3p	IKZF2	22807	ENSG00000030419	validated
tarbase	hsa-miR-212-3p	FAM13B	51306	ENSG00000031003	validated
tarbase	hsa-miR-212-3p	CENPQ	55166	ENSG00000031691	validated
tarbase	hsa-miR-296-5p	RANBP3	8498	ENSG00000031823	validated
tarbase	hsa-miR-212-3p	ARID4A	5926	ENSG00000032219	validated

tarbase	hsa-miR-212-3p	IFT88	8100	ENSG00000032742	validated
tarbase	hsa-miR-15b-5p	ABCF2	10061	ENSG00000033050	validated
tarbase	hsa-miR-301a-3p	ABCF2	10061	ENSG00000033050	validated
tarbase	hsa-miR-15b-5p	CHPF2	54480	ENSG00000033100	validated
tarbase	hsa-miR-181a-5p	CHPF2	54480	ENSG00000033100	validated
tarbase	hsa-miR-15b-5p	LRRC7	57554	ENSG00000033122	validated
tarbase	hsa-miR-296-5p	FUT8	2530	ENSG00000033170	validated
tarbase	hsa-miR-15b-5p	UBA6	55236	ENSG00000033178	validated
tarbase	hsa-miR-21-5p	UBA6	55236	ENSG00000033178	validated
tarbase	hsa-miR-192-5p	ATP6V0A1	535	ENSG00000033627	validated
tarbase	hsa-miR-15b-5p	PIAS1	8554	ENSG00000033800	validated
tarbase	hsa-miR-181a-5p	PIAS1	8554	ENSG00000033800	validated
tarbase	hsa-miR-181a-5p	SLC4A7	9497	ENSG00000033867	validated
tarbase	hsa-miR-222-3p	SLC4A7	9497	ENSG00000033867	validated
tarbase	hsa-miR-18a-5p	MAP2K3	5606	ENSG00000034152	validated
tarbase	hsa-miR-212-3p	RNF19A	25897	ENSG00000034677	validated
tarbase	hsa-miR-222-3p	RNF19A	25897	ENSG00000034677	validated
tarbase	hsa-miR-582-3p	RNF19A	25897	ENSG00000034677	validated
tarbase	hsa-miR-106b-5p	FAM136A	84908	ENSG00000035141	validated
tarbase	hsa-miR-106b-5p	VCL	7414	ENSG00000035403	validated
tarbase	hsa-miR-181a-5p	VCL	7414	ENSG00000035403	validated
tarbase	hsa-miR-21-5p	VCL	7414	ENSG00000035403	validated
tarbase	hsa-miR-326	VCL	7414	ENSG00000035403	validated
tarbase	hsa-miR-15b-5p	DEPDC1B	55789	ENSG00000035499	validated
tarbase	hsa-miR-582-3p	DEPDC1B	55789	ENSG00000035499	validated
tarbase	hsa-miR-212-3p	DEPDC1B	55789	ENSG00000035499	validated
tarbase	hsa-miR-15b-5p	ADSS2	159	ENSG00000035687	validated
tarbase	hsa-miR-181a-5p	ADSS2	159	ENSG00000035687	validated
tarbase	hsa-miR-181a-5p	TIMP2	7077	ENSG00000035862	validated
tarbase	hsa-miR-18a-5p	TIMP2	7077	ENSG00000035862	validated
tarbase	hsa-miR-222-3p	TIMP2	7077	ENSG00000035862	validated
tarbase	hsa-miR-144-3p	RFC1	5981	ENSG00000035928	validated
tarbase	hsa-miR-21-5p	RFC1	5981	ENSG00000035928	validated
tarbase	hsa-miR-582-5p	RFC1	5981	ENSG00000035928	validated
tarbase	hsa-miR-582-5p	TBC1D23	55773	ENSG00000036054	validated
tarbase	hsa-miR-106b-5p	CUL3	8452	ENSG00000036257	validated
tarbase	hsa-miR-21-5p	CUL3	8452	ENSG00000036257	validated
tarbase	hsa-miR-222-3p	CUL3	8452	ENSG00000036257	validated
tarbase	hsa-miR-181a-5p	AC118549.1	26009	ENSG00000036549	validated
tarbase	hsa-miR-212-3p	TUBG2	27175	ENSG00000037042	validated
tarbase	hsa-miR-296-5p	FLT4	2324	ENSG00000037280	validated
tarbase	hsa-miR-106b-5p	NSUN2	54888	ENSG00000037474	validated
tarbase	hsa-miR-15b-5p	NSUN2	54888	ENSG00000037474	validated
tarbase	hsa-miR-106b-5p	MFAP3	4238	ENSG00000037749	validated
tarbase	hsa-miR-181a-5p	MFAP3	4238	ENSG00000037749	validated
tarbase	hsa-miR-222-3p	MFAP3	4238	ENSG00000037749	validated
tarbase	hsa-miR-301a-3p	MFAP3	4238	ENSG00000037749	validated
tarbase	hsa-miR-326	MRI1	84245	ENSG00000037757	validated
tarbase	hsa-miR-21-5p	PI4K2B	55300	ENSG00000038210	validated
tarbase	hsa-miR-106b-5p	BOD1L1	259282	ENSG00000038219	validated
tarbase	hsa-miR-144-3p	BOD1L1	259282	ENSG00000038219	validated
tarbase	hsa-miR-18a-5p	BOD1L1	259282	ENSG00000038219	validated
tarbase	hsa-miR-21-5p	BOD1L1	259282	ENSG00000038219	validated
tarbase	hsa-miR-106b-5p	MAT2B	27430	ENSG00000038274	validated
tarbase	hsa-miR-21-5p	EDC4	23644	ENSG00000038358	validated
tarbase	hsa-miR-296-5p	TRIO	7204	ENSG00000038382	validated
tarbase	hsa-miR-582-3p	TRIO	7204	ENSG00000038382	validated
tarbase	hsa-miR-582-5p	TRIO	7204	ENSG00000038382	validated
tarbase	hsa-miR-106b-5p	VCAN	1462	ENSG00000038427	validated
tarbase	hsa-miR-144-3p	VCAN	1462	ENSG00000038427	validated
tarbase	hsa-miR-18a-5p	VCAN	1462	ENSG00000038427	validated
tarbase	hsa-miR-192-5p	VCAN	1462	ENSG00000038427	validated
tarbase	hsa-miR-222-3p	VCAN	1462	ENSG00000038427	validated
tarbase	hsa-miR-181a-5p	CLEC16A	23274	ENSG00000038532	validated
tarbase	hsa-miR-15b-5p	CDH1	999	ENSG00000039068	validated
tarbase	hsa-miR-106b-5p	RAI14	26064	ENSG00000039560	validated
tarbase	hsa-miR-15b-5p	RAI14	26064	ENSG00000039560	validated
tarbase	hsa-miR-181a-5p	RAI14	26064	ENSG00000039560	validated
tarbase	hsa-miR-222-3p	RAI14	26064	ENSG00000039560	validated
tarbase	hsa-miR-301a-3p	RAI14	26064	ENSG00000039560	validated
tarbase	hsa-miR-301a-3p	PHLPP2	23035	ENSG00000040199	validated

tarbase	hsa-miR-18a-5p	PHLPP2	23035	ENSG00000040199	validated
tarbase	hsa-miR-181a-5p	SPDL1	54908	ENSG00000040275	validated
tarbase	hsa-miR-15b-5p	PHF23	79142	ENSG00000040633	validated
tarbase	hsa-miR-296-5p	PHF23	79142	ENSG00000040633	validated
tarbase	hsa-miR-15b-5p	INPP4A	3631	ENSG00000040933	validated
tarbase	hsa-miR-181a-5p	INPP4A	3631	ENSG00000040933	validated
tarbase	hsa-miR-192-5p	RAB27B	5874	ENSG00000041353	validated
tarbase	hsa-miR-21-5p	RAB27B	5874	ENSG00000041353	validated
tarbase	hsa-miR-181a-5p	PSMA4	5685	ENSG00000041357	validated
tarbase	hsa-miR-15b-5p	LSG1	55341	ENSG00000041802	validated
tarbase	hsa-miR-181a-5p	LSG1	55341	ENSG00000041802	validated
tarbase	hsa-miR-21-5p	LSG1	55341	ENSG00000041802	validated
tarbase	hsa-miR-296-5p	TNC	3371	ENSG00000041982	validated
tarbase	hsa-miR-505-3p	TNC	3371	ENSG00000041982	validated
tarbase	hsa-miR-212-3p	TNC	3371	ENSG00000041982	validated
tarbase	hsa-miR-212-3p	C2orf83	56918	ENSG00000042304	validated
tarbase	hsa-miR-106b-5p	AL049834.1	55812	ENSG00000042317	validated
tarbase	hsa-miR-212-3p	AL049834.1	55812	ENSG00000042317	validated
tarbase	hsa-miR-301a-3p	MED17	9440	ENSG00000042429	validated
tarbase	hsa-miR-505-3p	MED17	9440	ENSG00000042429	validated
tarbase	hsa-miR-15b-5p	MED17	9440	ENSG00000042429	validated
tarbase	hsa-miR-222-3p	DCUN1D1	54165	ENSG00000043093	validated
tarbase	hsa-miR-181a-5p	ZIC2	7546	ENSG00000043355	validated
tarbase	hsa-miR-212-3p	ZIC2	7546	ENSG00000043355	validated
tarbase	hsa-miR-326	ZIC2	7546	ENSG00000043355	validated
tarbase	hsa-miR-582-5p	ADRB1	153	ENSG00000043591	validated
tarbase	hsa-miR-301a-3p	CUL7	9820	ENSG00000044090	validated
tarbase	hsa-miR-18a-5p	CTNNA1	1495	ENSG00000044115	validated
tarbase	hsa-miR-15b-5p	HSPA5	3309	ENSG00000044574	validated
tarbase	hsa-miR-181a-5p	HSPA5	3309	ENSG00000044574	validated
tarbase	hsa-miR-21-5p	HSPA5	3309	ENSG00000044574	validated
tarbase	hsa-miR-222-3p	HSPA5	3309	ENSG00000044574	validated
tarbase	hsa-miR-181a-5p	DSG2	1829	ENSG00000046604	validated
tarbase	hsa-miR-296-5p	DSG2	1829	ENSG00000046604	validated
tarbase	hsa-miR-15b-5p	GPM6B	2824	ENSG00000046653	validated
tarbase	hsa-miR-181a-5p	PREX2	80243	ENSG00000046889	validated
tarbase	hsa-miR-181a-5p	WDR37	22884	ENSG00000047056	validated
tarbase	hsa-miR-21-5p	YTHDC2	64848	ENSG00000047188	validated
tarbase	hsa-miR-181a-5p	YTHDC2	64848	ENSG00000047188	validated
tarbase	hsa-miR-15b-5p	CTPS2	56474	ENSG00000047230	validated
tarbase	hsa-miR-212-3p	ATP6V1H	51606	ENSG00000047249	validated
tarbase	hsa-miR-106b-5p	POLR2B	5431	ENSG00000047315	validated
tarbase	hsa-miR-15b-5p	POLR2B	5431	ENSG00000047315	validated
tarbase	hsa-miR-181a-5p	POLR2B	5431	ENSG00000047315	validated
tarbase	hsa-miR-212-3p	POLR2B	5431	ENSG00000047315	validated
tarbase	hsa-miR-301a-3p	POLR2B	5431	ENSG00000047315	validated
tarbase	hsa-miR-181a-5p	FAM214A	56204	ENSG00000047346	validated
tarbase	hsa-miR-301a-3p	FAM214A	56204	ENSG00000047346	validated
tarbase	hsa-miR-18a-5p	ARAP2	116984	ENSG00000047365	validated
tarbase	hsa-miR-301a-3p	ARAP2	116984	ENSG00000047365	validated
tarbase	hsa-miR-15b-5p	ARAP2	116984	ENSG00000047365	validated
tarbase	hsa-miR-181a-5p	ARAP2	116984	ENSG00000047365	validated
tarbase	hsa-miR-106b-5p	TPR	7175	ENSG00000047410	validated
tarbase	hsa-miR-181a-5p	TPR	7175	ENSG00000047410	validated
tarbase	hsa-miR-212-3p	TPR	7175	ENSG00000047410	validated
tarbase	hsa-miR-106b-5p	SCML1	6322	ENSG00000047634	validated
tarbase	hsa-miR-15b-5p	SCML1	6322	ENSG00000047634	validated
tarbase	hsa-miR-181a-5p	SCML1	6322	ENSG00000047634	validated
tarbase	hsa-miR-18a-5p	SCML1	6322	ENSG00000047634	validated
tarbase	hsa-miR-505-3p	SCML1	6322	ENSG00000047634	validated
tarbase	hsa-miR-582-5p	SCML1	6322	ENSG00000047634	validated
tarbase	hsa-miR-15b-5p	MAP4	4134	ENSG00000047849	validated
tarbase	hsa-miR-582-3p	MAP4	4134	ENSG00000047849	validated
tarbase	hsa-miR-582-5p	MAP4	4134	ENSG00000047849	validated
tarbase	hsa-miR-301a-3p	USP28	57646	ENSG00000048028	validated
tarbase	hsa-miR-301a-3p	TSPAN17	26262	ENSG00000048140	validated
tarbase	hsa-miR-15b-5p	RRM2B	50484	ENSG00000048392	validated
tarbase	hsa-miR-181a-5p	RRM2B	50484	ENSG00000048392	validated
tarbase	hsa-miR-181a-5p	ZNF800	168850	ENSG00000048405	validated
tarbase	hsa-miR-582-5p	ZNF800	168850	ENSG00000048405	validated
tarbase	hsa-miR-106b-5p	TNFRSF17	608	ENSG00000048462	validated

tarbase	hsa-miR-21-5p	SNX29	92017	ENSG00000048471	validated
tarbase	hsa-miR-181a-5p	LMO3	55885	ENSG00000048540	validated
tarbase	hsa-miR-106b-5p	RSF1	51773	ENSG00000048649	validated
tarbase	hsa-miR-106b-5p	VPS13D	55187	ENSG00000048707	validated
tarbase	hsa-miR-181a-5p	VPS13D	55187	ENSG00000048707	validated
tarbase	hsa-miR-296-5p	VPS13D	55187	ENSG00000048707	validated
tarbase	hsa-miR-301a-3p	VPS13D	55187	ENSG00000048707	validated
tarbase	hsa-miR-106b-5p	CELF2	10659	ENSG00000048740	validated
tarbase	hsa-miR-144-3p	CELF2	10659	ENSG00000048740	validated
tarbase	hsa-miR-21-5p	CELF2	10659	ENSG00000048740	validated
tarbase	hsa-miR-582-5p	CELF2	10659	ENSG00000048740	validated
tarbase	hsa-miR-106b-5p	FAM120A	23196	ENSG00000048828	validated
tarbase	hsa-miR-15b-5p	FAM120A	23196	ENSG00000048828	validated
tarbase	hsa-miR-301a-3p	FAM120A	23196	ENSG00000048828	validated
tarbase	hsa-miR-212-3p	COL9A2	1298	ENSG00000049089	validated
tarbase	hsa-miR-212-3p	ADAMTS6	11174	ENSG00000049192	validated
tarbase	hsa-miR-301a-3p	VAMP3	9341	ENSG00000049245	validated
tarbase	hsa-miR-326	VAMP3	9341	ENSG00000049245	validated
tarbase	hsa-miR-582-5p	VAMP3	9341	ENSG00000049245	validated
tarbase	hsa-miR-212-3p	TNFRSF9	3604	ENSG00000049249	validated
tarbase	hsa-miR-106b-5p	LTBP1	4052	ENSG00000049323	validated
tarbase	hsa-miR-15b-5p	LTBP1	4052	ENSG00000049323	validated
tarbase	hsa-miR-296-5p	LTBP1	4052	ENSG00000049323	validated
tarbase	hsa-miR-301a-3p	LTBP1	4052	ENSG00000049323	validated
tarbase	hsa-miR-21-5p	RCN1	5954	ENSG00000049449	validated
tarbase	hsa-miR-181a-5p	RCN1	5954	ENSG00000049449	validated
tarbase	hsa-miR-212-3p	RCN1	5954	ENSG00000049449	validated
tarbase	hsa-miR-181a-5p	RFC2	5982	ENSG00000049541	validated
tarbase	hsa-miR-181a-5p	ARID1B	57492	ENSG00000049618	validated
tarbase	hsa-miR-18a-5p	CLPTM1L	81037	ENSG00000049656	validated
tarbase	hsa-miR-106b-5p	NEDD4L	23327	ENSG00000049759	validated
tarbase	hsa-miR-192-5p	NEDD4L	23327	ENSG00000049759	validated
tarbase	hsa-miR-15b-5p	JKAMP	51528	ENSG00000050130	validated
tarbase	hsa-miR-181a-5p	MCUR1	63933	ENSG00000050393	validated
tarbase	hsa-miR-21-5p	LIMA1	51474	ENSG00000050405	validated
tarbase	hsa-miR-301a-3p	LIMA1	51474	ENSG00000050405	validated
tarbase	hsa-miR-15b-5p	MAPK9	5601	ENSG00000050748	validated
tarbase	hsa-miR-582-3p	MAPK9	5601	ENSG00000050748	validated
tarbase	hsa-miR-144-3p	HERPUD1	9709	ENSG00000051108	validated
tarbase	hsa-miR-181a-5p	HERPUD1	9709	ENSG00000051108	validated
tarbase	hsa-miR-301a-3p	HERPUD1	9709	ENSG00000051108	validated
tarbase	hsa-miR-181a-5p	PIK3CB	5291	ENSG00000051382	validated
tarbase	hsa-miR-301a-3p	PIK3CB	5291	ENSG00000051382	validated
tarbase	hsa-miR-301a-3p	MPHOSPH9	10198	ENSG00000051825	validated
tarbase	hsa-miR-15b-5p	PLEKHA5	54477	ENSG00000052126	validated
tarbase	hsa-miR-21-5p	SIKE1	80143	ENSG00000052723	validated
tarbase	hsa-miR-222-3p	RRP12	23223	ENSG00000052749	validated
tarbase	hsa-miR-18a-5p	RRP12	23223	ENSG00000052749	validated
tarbase	hsa-miR-18a-5p	FNIP2	57600	ENSG00000052795	validated
tarbase	hsa-miR-21-5p	FNIP2	57600	ENSG00000052795	validated
tarbase	hsa-miR-222-3p	FNIP2	57600	ENSG00000052795	validated
tarbase	hsa-miR-505-3p	FNIP2	57600	ENSG00000052795	validated
tarbase	hsa-miR-181a-5p	FNIP2	57600	ENSG00000052795	validated
tarbase	hsa-miR-18a-5p	MSMO1	6307	ENSG00000052802	validated
tarbase	hsa-miR-15b-5p	TTC17	55761	ENSG00000052841	validated
tarbase	hsa-miR-181a-5p	TTC17	55761	ENSG00000052841	validated
tarbase	hsa-miR-212-3p	ALX4	60529	ENSG00000052850	validated
tarbase	hsa-miR-18a-5p	AKR7A2	8574	ENSG00000053371	validated
tarbase	hsa-miR-326	MRTO4	51154	ENSG00000053372	validated
tarbase	hsa-miR-582-5p	MRTO4	51154	ENSG00000053372	validated
tarbase	hsa-miR-212-3p	USE1	55850	ENSG00000053501	validated
tarbase	hsa-miR-212-3p	NRIP2	83714	ENSG00000053702	validated
tarbase	hsa-miR-106b-5p	LAMA3	3909	ENSG00000053747	validated
tarbase	hsa-miR-296-5p	THRAP3	9967	ENSG00000054118	validated
tarbase	hsa-miR-106b-5p	LY75	4065	ENSG00000054219	validated
tarbase	hsa-miR-18a-5p	LY75	4065	ENSG00000054219	validated
tarbase	hsa-miR-181a-5p	ARID4B	51742	ENSG00000054267	validated
tarbase	hsa-miR-18a-5p	ARID4B	51742	ENSG00000054267	validated
tarbase	hsa-miR-212-3p	ARID4B	51742	ENSG00000054267	validated
tarbase	hsa-miR-21-5p	ARID4B	51742	ENSG00000054267	validated
tarbase	hsa-miR-301a-3p	ARID4B	51742	ENSG00000054267	validated

tarbase	hsa-miR-212-3p	SDCCAG8	10806	ENSG00000054282	validated
tarbase	hsa-miR-326	PTPRN	5798	ENSG00000054356	validated
tarbase	hsa-miR-106b-5p	KIF1B	23095	ENSG00000054523	validated
tarbase	hsa-miR-15b-5p	KIF1B	23095	ENSG00000054523	validated
tarbase	hsa-miR-181a-5p	KIF1B	23095	ENSG00000054523	validated
tarbase	hsa-miR-222-3p	FOXC1	2296	ENSG00000054598	validated
tarbase	hsa-miR-301a-3p	SYNE2	23224	ENSG00000054654	validated
tarbase	hsa-miR-326	SYNE2	23224	ENSG00000054654	validated
tarbase	hsa-miR-582-3p	SYNE2	23224	ENSG00000054654	validated
tarbase	hsa-miR-106b-5p	ATP9A	10079	ENSG00000054793	validated
tarbase	hsa-miR-181a-5p	ATP9A	10079	ENSG00000054793	validated
tarbase	hsa-miR-212-3p	ATP9A	10079	ENSG00000054793	validated
tarbase	hsa-miR-106b-5p	FAM168A	23201	ENSG00000054965	validated
tarbase	hsa-miR-222-3p	RELT	84957	ENSG00000054967	validated
tarbase	hsa-miR-301a-3p	SZRD1	26099	ENSG00000055070	validated
tarbase	hsa-miR-106b-5p	CUL1	8454	ENSG00000055130	validated
tarbase	hsa-miR-505-3p	FAM114A2	10827	ENSG00000055147	validated
tarbase	hsa-miR-106b-5p	GINM1	116254	ENSG00000055211	validated
tarbase	hsa-miR-18a-5p	GINM1	116254	ENSG00000055211	validated
tarbase	hsa-miR-18a-5p	EIF2AK2	5610	ENSG00000055332	validated
tarbase	hsa-miR-301a-3p	EIF2AK2	5610	ENSG00000055332	validated
tarbase	hsa-miR-212-3p	EIF2AK2	5610	ENSG00000055332	validated
tarbase	hsa-miR-106b-5p	AC022966.1	57602	ENSG00000055483	validated
tarbase	hsa-miR-106b-5p	KMT2C	58508	ENSG00000055609	validated
tarbase	hsa-miR-181a-5p	KMT2C	58508	ENSG00000055609	validated
tarbase	hsa-miR-21-5p	KMT2C	58508	ENSG00000055609	validated
tarbase	hsa-miR-222-3p	KMT2C	58508	ENSG00000055609	validated
tarbase	hsa-miR-301a-3p	KMT2C	58508	ENSG00000055609	validated
tarbase	hsa-miR-181a-5p	CCDC85A	114800	ENSG00000055813	validated
tarbase	hsa-miR-106b-5p	PUM2	23369	ENSG00000055917	validated
tarbase	hsa-miR-15b-5p	PUM2	23369	ENSG00000055917	validated
tarbase	hsa-miR-181a-5p	PUM2	23369	ENSG00000055917	validated
tarbase	hsa-miR-192-5p	PUM2	23369	ENSG00000055917	validated
tarbase	hsa-miR-212-3p	PUM2	23369	ENSG00000055917	validated
tarbase	hsa-miR-301a-3p	PUM2	23369	ENSG00000055917	validated
tarbase	hsa-miR-582-5p	PUM2	23369	ENSG00000055917	validated
tarbase	hsa-miR-106b-5p	MRPL43	84545	ENSG00000055950	validated
tarbase	hsa-miR-15b-5p	ZFR	51663	ENSG00000056097	validated
tarbase	hsa-miR-181a-5p	ZFR	51663	ENSG00000056097	validated
tarbase	hsa-miR-212-3p	ZFR	51663	ENSG00000056097	validated
tarbase	hsa-miR-222-3p	ZFR	51663	ENSG00000056097	validated
tarbase	hsa-miR-18a-5p	ZNF280C	55609	ENSG00000056277	validated
tarbase	hsa-miR-212-3p	ZNF280C	55609	ENSG00000056277	validated
tarbase	hsa-miR-106b-5p	RC3H2	54542	ENSG00000056586	validated
tarbase	hsa-miR-15b-5p	RC3H2	54542	ENSG00000056586	validated
tarbase	hsa-miR-181a-5p	RC3H2	54542	ENSG00000056586	validated
tarbase	hsa-miR-212-3p	RC3H2	54542	ENSG00000056586	validated
tarbase	hsa-miR-21-5p	RC3H2	54542	ENSG00000056586	validated
tarbase	hsa-miR-222-3p	RC3H2	54542	ENSG00000056586	validated
tarbase	hsa-miR-296-5p	RC3H2	54542	ENSG00000056586	validated
tarbase	hsa-miR-301a-3p	RC3H2	54542	ENSG00000056586	validated
tarbase	hsa-miR-582-3p	RC3H2	54542	ENSG00000056586	validated
tarbase	hsa-miR-582-5p	RC3H2	54542	ENSG00000056586	validated
tarbase	hsa-miR-192-5p	KIFC1	3833	ENSG00000056678	validated
tarbase	hsa-miR-181a-5p	SOAT1	6646	ENSG00000057252	validated
tarbase	hsa-miR-106b-5p	GDI2	2665	ENSG00000057608	validated
tarbase	hsa-miR-15b-5p	GDI2	2665	ENSG00000057608	validated
tarbase	hsa-miR-181a-5p	GDI2	2665	ENSG00000057608	validated
tarbase	hsa-miR-15b-5p	PRDM1	639	ENSG00000057657	validated
tarbase	hsa-miR-181a-5p	PRDM1	639	ENSG00000057657	validated
tarbase	hsa-miR-192-5p	PITHD1	57095	ENSG00000057757	validated
tarbase	hsa-miR-212-3p	PITHD1	57095	ENSG00000057757	validated
tarbase	hsa-miR-21-5p	USP13	8975	ENSG00000058056	validated
tarbase	hsa-miR-301a-3p	ATP11B	23200	ENSG00000058063	validated
tarbase	hsa-miR-582-5p	ATP11B	23200	ENSG00000058063	validated
tarbase	hsa-miR-15b-5p	LAMC2	3918	ENSG00000058085	validated
tarbase	hsa-miR-106b-5p	CDK14	5218	ENSG00000058091	validated
tarbase	hsa-miR-582-3p	SEC61A1	29927	ENSG00000058262	validated
tarbase	hsa-miR-21-5p	SEC61A1	29927	ENSG00000058262	validated
tarbase	hsa-miR-106b-5p	PPP1R12A	4659	ENSG00000058272	validated
tarbase	hsa-miR-144-3p	PPP1R12A	4659	ENSG00000058272	validated

tarbase	hsa-miR-181a-5p	PPP1R12A	4659	ENSG00000058272	validated
tarbase	hsa-miR-212-3p	CAMK2B	816	ENSG00000058404	validated
tarbase	hsa-miR-326	CROCC	9696	ENSG00000058453	validated
tarbase	hsa-miR-15b-5p	ZC3H11A	9877	ENSG00000058673	validated
tarbase	hsa-miR-181a-5p	ZC3H11A	9877	ENSG00000058673	validated
tarbase	hsa-miR-222-3p	ZC3H11A	9877	ENSG00000058673	validated
tarbase	hsa-miR-326	ZC3H11A	9877	ENSG00000058673	validated
tarbase	hsa-miR-15b-5p	NDC1	55706	ENSG00000058804	validated
tarbase	hsa-miR-181a-5p	NDC1	55706	ENSG00000058804	validated
tarbase	hsa-miR-106b-5p	UNKL	64718	ENSG00000059145	validated
tarbase	hsa-miR-181a-5p	UNKL	64718	ENSG00000059145	validated
tarbase	hsa-miR-296-5p	UNKL	64718	ENSG00000059145	validated
tarbase	hsa-miR-15b-5p	UNKL	64718	ENSG00000059145	validated
tarbase	hsa-miR-212-3p	PARP12	64761	ENSG00000059378	validated
tarbase	hsa-miR-15b-5p	ALDH18A1	5832	ENSG00000059573	validated
tarbase	hsa-miR-21-5p	ALDH18A1	5832	ENSG00000059573	validated
tarbase	hsa-miR-21-5p	TARBP1	6894	ENSG00000059588	validated
tarbase	hsa-miR-21-5p	MXD1	4084	ENSG00000059728	validated
tarbase	hsa-miR-106b-5p	CDK17	5128	ENSG00000059758	validated
tarbase	hsa-miR-181a-5p	CDK17	5128	ENSG00000059758	validated
tarbase	hsa-miR-212-3p	CDK17	5128	ENSG00000059758	validated
tarbase	hsa-miR-106b-5p	SLC2A3	6515	ENSG00000059804	validated
tarbase	hsa-miR-181a-5p	SLC2A3	6515	ENSG00000059804	validated
tarbase	hsa-miR-21-5p	SLC2A3	6515	ENSG00000059804	validated
tarbase	hsa-miR-15b-5p	PSD	5662	ENSG00000059915	validated
tarbase	hsa-miR-106b-5p	WNK1	65125	ENSG00000060237	validated
tarbase	hsa-miR-181a-5p	WNK1	65125	ENSG00000060237	validated
tarbase	hsa-miR-301a-3p	WNK1	65125	ENSG00000060237	validated
tarbase	hsa-miR-21-5p	CCAR1	55749	ENSG00000060339	validated
tarbase	hsa-miR-181a-5p	CCAR1	55749	ENSG00000060339	validated
tarbase	hsa-miR-106b-5p	CCAR1	55749	ENSG00000060339	validated
tarbase	hsa-miR-15b-5p	OGFR	11054	ENSG00000060491	validated
tarbase	hsa-miR-15b-5p	PIGV	55650	ENSG00000060642	validated
tarbase	hsa-miR-181a-5p	SNRNP40	9410	ENSG00000060688	validated
tarbase	hsa-miR-18a-5p	SNRNP40	9410	ENSG00000060688	validated
tarbase	hsa-miR-21-5p	SNRNP40	9410	ENSG00000060688	validated
tarbase	hsa-miR-15b-5p	QSER1	79832	ENSG00000060749	validated
tarbase	hsa-miR-21-5p	QSER1	79832	ENSG00000060749	validated
tarbase	hsa-miR-222-3p	QSER1	79832	ENSG00000060749	validated
tarbase	hsa-miR-301a-3p	QSER1	79832	ENSG00000060749	validated
tarbase	hsa-miR-582-3p	QSER1	79832	ENSG00000060749	validated
tarbase	hsa-miR-181a-5p	BCAT1	586	ENSG00000060982	validated
tarbase	hsa-miR-212-3p	BCAT1	586	ENSG00000060982	validated
tarbase	hsa-miR-301a-3p	BCAT1	586	ENSG00000060982	validated
tarbase	hsa-miR-296-5p	LZTS1	11178	ENSG00000061337	validated
tarbase	hsa-miR-106b-5p	NCKAP1	10787	ENSG00000061676	validated
tarbase	hsa-miR-144-3p	NCKAP1	10787	ENSG00000061676	validated
tarbase	hsa-miR-181a-5p	NCKAP1	10787	ENSG00000061676	validated
tarbase	hsa-miR-18a-5p	NCKAP1	10787	ENSG00000061676	validated
tarbase	hsa-miR-301a-3p	NCKAP1	10787	ENSG00000061676	validated
tarbase	hsa-miR-212-3p	MRPS35	60488	ENSG00000061794	validated
tarbase	hsa-miR-15b-5p	MON2	23041	ENSG00000061987	validated
tarbase	hsa-miR-181a-5p	MON2	23041	ENSG00000061987	validated
tarbase	hsa-miR-18a-5p	GPBP1	65056	ENSG00000062194	validated
tarbase	hsa-miR-181a-5p	GPBP1	65056	ENSG00000062194	validated
tarbase	hsa-miR-582-5p	CS	1431	ENSG00000062485	validated
tarbase	hsa-miR-106b-5p	ELMO2	63916	ENSG00000062598	validated
tarbase	hsa-miR-15b-5p	ELMO2	63916	ENSG00000062598	validated
tarbase	hsa-miR-15b-5p	WAPL	23063	ENSG00000062650	validated
tarbase	hsa-miR-301a-3p	WAPL	23063	ENSG00000062650	validated
tarbase	hsa-miR-15b-5p	VMP1	81671	ENSG00000062716	validated
tarbase	hsa-miR-106b-5p	APPBP2	10513	ENSG00000062725	validated
tarbase	hsa-miR-21-5p	APPBP2	10513	ENSG00000062725	validated
tarbase	hsa-miR-301a-3p	APPBP2	10513	ENSG00000062725	validated
tarbase	hsa-miR-192-5p	POLD1	5424	ENSG00000062822	validated
tarbase	hsa-miR-301a-3p	SPHK2	56848	ENSG00000063176	validated
tarbase	hsa-miR-212-3p	RPL18	6141	ENSG00000063177	validated
tarbase	hsa-miR-296-5p	RPL18	6141	ENSG00000063177	validated
tarbase	hsa-miR-15b-5p	U2AF2	11338	ENSG00000063244	validated
tarbase	hsa-miR-326	U2AF2	11338	ENSG00000063244	validated
tarbase	hsa-miR-15b-5p	EPN1	29924	ENSG00000063245	validated

tarbase	hsa-miR-296-5p	EPN1	29924	ENSG00000063245	validated
tarbase	hsa-miR-106b-5p	AHRR		ENSG00000063438	validated
tarbase	hsa-miR-21-5p	ZNF275	10838	ENSG00000063587	validated
tarbase	hsa-miR-15b-5p	MTMR1	8776	ENSG00000063601	validated
tarbase	hsa-miR-212-3p	MTMR1	8776	ENSG00000063601	validated
tarbase	hsa-miR-106b-5p	RNF4	6047	ENSG00000063978	validated
tarbase	hsa-miR-212-3p	RNF4	6047	ENSG00000063978	validated
tarbase	hsa-miR-21-5p	RNF4	6047	ENSG00000063978	validated
tarbase	hsa-miR-582-3p	RNF4	6047	ENSG00000063978	validated
tarbase	hsa-miR-301a-3p	CASP8	841	ENSG00000064012	validated
tarbase	hsa-miR-212-3p	CCN5	8839	ENSG00000064205	validated
tarbase	hsa-miR-301a-3p	DMRT3	58524	ENSG00000064218	validated
tarbase	hsa-miR-181a-5p	CDON	50937	ENSG00000064309	validated
tarbase	hsa-miR-301a-3p	TAF2	6873	ENSG00000064313	validated
tarbase	hsa-miR-15b-5p	HIPK2	28996	ENSG00000064393	validated
tarbase	hsa-miR-18a-5p	HIPK2	28996	ENSG00000064393	validated
tarbase	hsa-miR-192-5p	HIPK2	28996	ENSG00000064393	validated
tarbase	hsa-miR-21-5p	HIPK2	28996	ENSG00000064393	validated
tarbase	hsa-miR-106b-5p	TNPO3	23534	ENSG00000064419	validated
tarbase	hsa-miR-181a-5p	TNPO3	23534	ENSG00000064419	validated
tarbase	hsa-miR-301a-3p	TNPO3	23534	ENSG00000064419	validated
tarbase	hsa-miR-296-5p	RFXANK	8625	ENSG00000064490	validated
tarbase	hsa-miR-15b-5p	TMEM161A	54929	ENSG00000064545	validated
tarbase	hsa-miR-106b-5p	CTSA	5476	ENSG00000064601	validated
tarbase	hsa-miR-15b-5p	CTSA	5476	ENSG00000064601	validated
tarbase	hsa-miR-212-3p	CTSA	5476	ENSG00000064601	validated
tarbase	hsa-miR-181a-5p	SUGP2	10147	ENSG00000064607	validated
tarbase	hsa-miR-582-3p	SLC12A2	6558	ENSG00000064651	validated
tarbase	hsa-miR-15b-5p	SLC12A2	6558	ENSG00000064651	validated
tarbase	hsa-miR-21-5p	CNN2	1265	ENSG00000064666	validated
tarbase	hsa-miR-222-3p	DDX20	11218	ENSG00000064703	validated
tarbase	hsa-miR-18a-5p	BTBD1	53339	ENSG00000064726	validated
tarbase	hsa-miR-212-3p	PMS1	5378	ENSG00000064933	validated
tarbase	hsa-miR-181a-5p	CALCRL	10203	ENSG00000064989	validated
tarbase	hsa-miR-106b-5p	AP3D1	8943	ENSG00000065000	validated
tarbase	hsa-miR-181a-5p	AP3D1	8943	ENSG00000065000	validated
tarbase	hsa-miR-222-3p	ZNF76	7629	ENSG00000065029	validated
tarbase	hsa-miR-15b-5p	UHRF1BP1	54887	ENSG00000065060	validated
tarbase	hsa-miR-106b-5p	GNAI3	2773	ENSG00000065135	validated
tarbase	hsa-miR-15b-5p	GNAI3	2773	ENSG00000065135	validated
tarbase	hsa-miR-106b-5p	IPO5	3843	ENSG00000065150	validated
tarbase	hsa-miR-222-3p	OAT	4942	ENSG00000065154	validated
tarbase	hsa-miR-181a-5p	OAT	4942	ENSG00000065154	validated
tarbase	hsa-miR-21-5p	WDR3	10885	ENSG00000065183	validated
tarbase	hsa-miR-106b-5p	PKN2	5586	ENSG00000065243	validated
tarbase	hsa-miR-181a-5p	PKN2	5586	ENSG00000065243	validated
tarbase	hsa-miR-18a-5p	TRAM2	9697	ENSG00000065308	validated
tarbase	hsa-miR-212-3p	TRAM2	9697	ENSG00000065308	validated
tarbase	hsa-miR-15b-5p	NTN1	9423	ENSG00000065320	validated
tarbase	hsa-miR-106b-5p	MCM10	55388	ENSG00000065328	validated
tarbase	hsa-miR-181a-5p	ANKRD44	91526	ENSG00000065413	validated
tarbase	hsa-miR-15b-5p	KARS1	3735	ENSG00000065427	validated
tarbase	hsa-miR-222-3p	KARS1	3735	ENSG00000065427	validated
tarbase	hsa-miR-15b-5p	SPEN	23013	ENSG00000065526	validated
tarbase	hsa-miR-181a-5p	SPEN	23013	ENSG00000065526	validated
tarbase	hsa-miR-21-5p	SPEN	23013	ENSG00000065526	validated
tarbase	hsa-miR-296-5p	SPEN	23013	ENSG00000065526	validated
tarbase	hsa-miR-301a-3p	SPEN	23013	ENSG00000065526	validated
tarbase	hsa-miR-326	SPEN	23013	ENSG00000065526	validated
tarbase	hsa-miR-222-3p	ZC3H15	55854	ENSG00000065548	validated
tarbase	hsa-miR-21-5p	MAP2K4	6416	ENSG00000065559	validated
tarbase	hsa-miR-15b-5p	PACC1	55248	ENSG00000065600	validated
tarbase	hsa-miR-181a-5p	SLK	9748	ENSG00000065613	validated
tarbase	hsa-miR-15b-5p	SLK	9748	ENSG00000065613	validated
tarbase	hsa-miR-21-5p	CYB5R4	51167	ENSG00000065615	validated
tarbase	hsa-miR-212-3p	COL17A1	1308	ENSG00000065618	validated
tarbase	hsa-miR-21-5p	GSTO2	119391	ENSG00000065621	validated
tarbase	hsa-miR-15b-5p	SEC61A2	55176	ENSG00000065665	validated
tarbase	hsa-miR-296-5p	SEC61A2	55176	ENSG00000065665	validated
tarbase	hsa-miR-15b-5p	ASB1	51665	ENSG00000065802	validated
tarbase	hsa-miR-18a-5p	ASB1	51665	ENSG00000065802	validated

tarbase	hsa-miR-212-3p	ASB1	51665	ENSG00000065802	validated
tarbase	hsa-miR-144-3p	ME1	4199	ENSG00000065833	validated
tarbase	hsa-miR-212-3p	ME1	4199	ENSG00000065833	validated
tarbase	hsa-miR-21-5p	ME1	4199	ENSG00000065833	validated
tarbase	hsa-miR-582-5p	ME1	4199	ENSG00000065833	validated
tarbase	hsa-miR-18a-5p	TBC1D1	23216	ENSG00000065882	validated
tarbase	hsa-miR-582-3p	CDK13	8621	ENSG00000065883	validated
tarbase	hsa-miR-582-5p	CDK13	8621	ENSG00000065883	validated
tarbase	hsa-miR-301a-3p	MTHFD2	10797	ENSG00000065911	validated
tarbase	hsa-miR-582-3p	MTHFD2	10797	ENSG00000065911	validated
tarbase	hsa-miR-15b-5p	MTHFD2	10797	ENSG00000065911	validated
tarbase	hsa-miR-106b-5p	PDE4A	5141	ENSG00000065989	validated
tarbase	hsa-miR-181a-5p	ELAVL1	1994	ENSG00000066044	validated
tarbase	hsa-miR-18a-5p	ELAVL1	1994	ENSG00000066044	validated
tarbase	hsa-miR-326	ELAVL1	1994	ENSG00000066044	validated
tarbase	hsa-miR-181a-5p	DIP2B	57609	ENSG00000066084	validated
tarbase	hsa-miR-144-3p	SMARCD1	6602	ENSG00000066117	validated
tarbase	hsa-miR-181a-5p	SMARCD1	6602	ENSG00000066117	validated
tarbase	hsa-miR-18a-5p	SMARCD1	6602	ENSG00000066117	validated
tarbase	hsa-miR-21-5p	SMARCD1	6602	ENSG00000066117	validated
tarbase	hsa-miR-582-3p	SMARCD1	6602	ENSG00000066117	validated
tarbase	hsa-miR-106b-5p	KDM4A	9682	ENSG00000066135	validated
tarbase	hsa-miR-181a-5p	NFYC	4802	ENSG00000066136	validated
tarbase	hsa-miR-15b-5p	ASPM	259266	ENSG00000066279	validated
tarbase	hsa-miR-18a-5p	ASPM	259266	ENSG00000066279	validated
tarbase	hsa-miR-301a-3p	ASPM	259266	ENSG00000066279	validated
tarbase	hsa-miR-212-3p	ASPM	259266	ENSG00000066279	validated
tarbase	hsa-miR-21-5p	ASPM	259266	ENSG00000066279	validated
tarbase	hsa-miR-212-3p	ELOVL1	64834	ENSG00000066322	validated
tarbase	hsa-miR-192-5p	MPPED2	744	ENSG00000066382	validated
tarbase	hsa-miR-15b-5p	ZBTB11	27107	ENSG00000066422	validated
tarbase	hsa-miR-181a-5p	ZBTB11	27107	ENSG00000066422	validated
tarbase	hsa-miR-106b-5p	ATXN3	4287	ENSG00000066427	validated
tarbase	hsa-miR-181a-5p	ATXN3	4287	ENSG00000066427	validated
tarbase	hsa-miR-301a-3p	ATXN3	4287	ENSG00000066427	validated
tarbase	hsa-miR-15b-5p	ISOC1	51015	ENSG00000066583	validated
tarbase	hsa-miR-15b-5p	THUMPD1	55623	ENSG00000066654	validated
tarbase	hsa-miR-181a-5p	THUMPD1	55623	ENSG00000066654	validated
tarbase	hsa-miR-212-3p	MSANTD3	91283	ENSG00000066697	validated
tarbase	hsa-miR-212-3p	ARFGEF1	10565	ENSG00000066777	validated
tarbase	hsa-miR-21-5p	ARFGEF1	10565	ENSG00000066777	validated
tarbase	hsa-miR-212-3p	MTFR1	9650	ENSG00000066855	validated
tarbase	hsa-miR-15b-5p	FECH	2235	ENSG00000066926	validated
tarbase	hsa-miR-301a-3p	FECH	2235	ENSG00000066926	validated
tarbase	hsa-miR-106b-5p	FECH	2235	ENSG00000066926	validated
tarbase	hsa-miR-181a-5p	MYO9A	4649	ENSG00000066933	validated
tarbase	hsa-miR-212-3p	MYO9A	4649	ENSG00000066933	validated
tarbase	hsa-miR-181a-5p	DDX3Y	8653	ENSG00000067048	validated
tarbase	hsa-miR-222-3p	DDX3Y	8653	ENSG00000067048	validated
tarbase	hsa-miR-301a-3p	DDX3Y	8653	ENSG00000067048	validated
tarbase	hsa-miR-106b-5p	DDX3Y	8653	ENSG00000067048	validated
tarbase	hsa-miR-21-5p	DDX3Y	8653	ENSG00000067048	validated
tarbase	hsa-miR-18a-5p	PFKP	5214	ENSG00000067057	validated
tarbase	hsa-miR-582-5p	PFKP	5214	ENSG00000067057	validated
tarbase	hsa-miR-212-3p	PFKP	5214	ENSG00000067057	validated
tarbase	hsa-miR-181a-5p	IDI1	3422	ENSG00000067064	validated
tarbase	hsa-miR-21-5p	IDI1	3422	ENSG00000067064	validated
tarbase	hsa-miR-212-3p	SP100	6672	ENSG00000067066	validated
tarbase	hsa-miR-505-3p	KLF6	1316	ENSG00000067082	validated
tarbase	hsa-miR-18a-5p	KLF6	1316	ENSG00000067082	validated
tarbase	hsa-miR-181a-5p	NEO1	4756	ENSG00000067141	validated
tarbase	hsa-miR-301a-3p	NEO1	4756	ENSG00000067141	validated
tarbase	hsa-miR-222-3p	TRAM1	23471	ENSG00000067167	validated
tarbase	hsa-miR-326	PHKA1	5255	ENSG00000067177	validated
tarbase	hsa-miR-15b-5p	PKM	5315	ENSG00000067225	validated
tarbase	hsa-miR-296-5p	PKM	5315	ENSG00000067225	validated
tarbase	hsa-miR-582-3p	PKM	5315	ENSG00000067225	validated
tarbase	hsa-miR-301a-3p	DNTTIP2	30836	ENSG00000067334	validated
tarbase	hsa-miR-181a-5p	DNTTIP2	30836	ENSG00000067334	validated
tarbase	hsa-miR-21-5p	TP53BP1	7158	ENSG00000067369	validated
tarbase	hsa-miR-301a-3p	RHOA	387	ENSG00000067560	validated

tarbase	hsa-miR-18a-5p	DHX8	1659	ENSG00000067596	validated
tarbase	hsa-miR-222-3p	DHX8	1659	ENSG00000067596	validated
tarbase	hsa-miR-582-5p	IARS2	55699	ENSG00000067704	validated
tarbase	hsa-miR-15b-5p	ROGDI	79641	ENSG00000067836	validated
tarbase	hsa-miR-212-3p	ROGDI	79641	ENSG00000067836	validated
tarbase	hsa-miR-582-5p	ROCK1	6093	ENSG00000067900	validated
tarbase	hsa-miR-106b-5p	CBFB	865	ENSG00000067955	validated
tarbase	hsa-miR-15b-5p	CBFB	865	ENSG00000067955	validated
tarbase	hsa-miR-21-5p	CBFB	865	ENSG00000067955	validated
tarbase	hsa-miR-222-3p	CBFB	865	ENSG00000067955	validated
tarbase	hsa-miR-301a-3p	CBFB	865	ENSG00000067955	validated
tarbase	hsa-miR-582-5p	CBFB	865	ENSG00000067955	validated
tarbase	hsa-miR-192-5p	CBFB	865	ENSG00000067955	validated
tarbase	hsa-miR-212-3p	HDAC4	9759	ENSG00000068024	validated
tarbase	hsa-miR-212-3p	IFI35	3430	ENSG00000068079	validated
tarbase	hsa-miR-18a-5p	HEATR6	63897	ENSG00000068097	validated
tarbase	hsa-miR-15b-5p	COASY	80347	ENSG00000068120	validated
tarbase	hsa-miR-181a-5p	COASY	80347	ENSG00000068120	validated
tarbase	hsa-miR-505-3p	COASY	80347	ENSG00000068120	validated
tarbase	hsa-miR-106b-5p	MEF2A	4205	ENSG00000068305	validated
tarbase	hsa-miR-15b-5p	TBC1D25	4943	ENSG00000068354	validated
tarbase	hsa-miR-15b-5p	ACSL4	2182	ENSG00000068366	validated
tarbase	hsa-miR-181a-5p	ACSL4	2182	ENSG00000068366	validated
tarbase	hsa-miR-21-5p	ACSL4	2182	ENSG00000068366	validated
tarbase	hsa-miR-582-5p	INPP5A	3632	ENSG00000068383	validated
tarbase	hsa-miR-144-3p	PRR11	55771	ENSG00000068489	validated
tarbase	hsa-miR-18a-5p	REEP1	65055	ENSG00000068615	validated
tarbase	hsa-miR-301a-3p	ATP11A	23250	ENSG00000068650	validated
tarbase	hsa-miR-15b-5p	POLR1A	25885	ENSG00000068654	validated
tarbase	hsa-miR-15b-5p	LAPTM4A	9741	ENSG00000068697	validated
tarbase	hsa-miR-296-5p	LAPTM4A	9741	ENSG00000068697	validated
tarbase	hsa-miR-18a-5p	LAPTM4A	9741	ENSG00000068697	validated
tarbase	hsa-miR-21-5p	LAPTM4A	9741	ENSG00000068697	validated
tarbase	hsa-miR-212-3p	TTC7A	57217	ENSG00000068724	validated
tarbase	hsa-miR-326	IP6K2	51447	ENSG00000068745	validated
tarbase	hsa-miR-15b-5p	KIF2A	3796	ENSG00000068796	validated
tarbase	hsa-miR-301a-3p	PSME4	23198	ENSG00000068878	validated
tarbase	hsa-miR-181a-5p	IFT80	57560	ENSG00000068885	validated
tarbase	hsa-miR-106b-5p	MAST4	375449	ENSG00000069020	validated
tarbase	hsa-miR-15b-5p	MAST4	375449	ENSG00000069020	validated
tarbase	hsa-miR-296-5p	MAST4	375449	ENSG00000069020	validated
tarbase	hsa-miR-15b-5p	ADGRF5	221395	ENSG00000069122	validated
tarbase	hsa-miR-106b-5p	NUP133	55746	ENSG00000069248	validated
tarbase	hsa-miR-301a-3p	NUP133	55746	ENSG00000069248	validated
tarbase	hsa-miR-106b-5p	NUCKS1	64710	ENSG00000069275	validated
tarbase	hsa-miR-181a-5p	NUCKS1	64710	ENSG00000069275	validated
tarbase	hsa-miR-18a-5p	NUCKS1	64710	ENSG00000069275	validated
tarbase	hsa-miR-212-3p	NUCKS1	64710	ENSG00000069275	validated
tarbase	hsa-miR-222-3p	NUCKS1	64710	ENSG00000069275	validated
tarbase	hsa-miR-296-5p	NUCKS1	64710	ENSG00000069275	validated
tarbase	hsa-miR-582-3p	NUCKS1	64710	ENSG00000069275	validated
tarbase	hsa-miR-582-5p	NUCKS1	64710	ENSG00000069275	validated
tarbase	hsa-miR-15b-5p	DNAJA2	10294	ENSG00000069345	validated
tarbase	hsa-miR-181a-5p	DNAJA2	10294	ENSG00000069345	validated
tarbase	hsa-miR-582-3p	ABCC9	10060	ENSG00000069431	validated
tarbase	hsa-miR-18a-5p	GAL	51083	ENSG00000069482	validated
tarbase	hsa-miR-181a-5p	RORA	6095	ENSG00000069667	validated
tarbase	hsa-miR-192-5p	RORA	6095	ENSG00000069667	validated
tarbase	hsa-miR-582-5p	RORA	6095	ENSG00000069667	validated
tarbase	hsa-miR-15b-5p	RORA	6095	ENSG00000069667	validated
tarbase	hsa-miR-582-3p	TGFBR3	7049	ENSG00000069702	validated
tarbase	hsa-miR-212-3p			ENSG00000069712	validated
tarbase	hsa-miR-18a-5p	NEDD4	4734	ENSG00000069869	validated
tarbase	hsa-miR-15b-5p	PIGB	9488	ENSG00000069943	validated
tarbase	hsa-miR-181a-5p	MAPK6	5597	ENSG00000069956	validated
tarbase	hsa-miR-181a-5p	RAB27A	5873	ENSG00000069974	validated
tarbase	hsa-miR-181a-5p	LRP6	4040	ENSG00000070018	validated
tarbase	hsa-miR-21-5p	PHRF1	57661	ENSG00000070047	validated
tarbase	hsa-miR-106b-5p	PFN2	5217	ENSG00000070087	validated
tarbase	hsa-miR-15b-5p	PFN2	5217	ENSG00000070087	validated
tarbase	hsa-miR-181a-5p	PFN2	5217	ENSG00000070087	validated

tarbase	hsa-miR-15b-5p	PTPN3	5774	ENSG00000070159	validated
tarbase	hsa-miR-15b-5p	SLC44A1	23446	ENSG00000070214	validated
tarbase	hsa-miR-21-5p	SLC44A1	23446	ENSG00000070214	validated
tarbase	hsa-miR-222-3p	SLC44A1	23446	ENSG00000070214	validated
tarbase	hsa-miR-301a-3p	SLC44A1	23446	ENSG00000070214	validated
tarbase	hsa-miR-582-5p	SLC44A1	23446	ENSG00000070214	validated
tarbase	hsa-miR-181a-5p	SLC44A1	23446	ENSG00000070214	validated
tarbase	hsa-miR-301a-3p	EXOC5	10640	ENSG00000070367	validated
tarbase	hsa-miR-181a-5p	EXOC5	10640	ENSG00000070367	validated
tarbase	hsa-miR-181a-5p	DGCR2	9993	ENSG00000070413	validated
tarbase	hsa-miR-15b-5p	MNT	4335	ENSG00000070444	validated
tarbase	hsa-miR-301a-3p	MNT	4335	ENSG00000070444	validated
tarbase	hsa-miR-582-5p	MNT	4335	ENSG00000070444	validated
tarbase	hsa-miR-106b-5p	WIPI1	55062	ENSG00000070540	validated
tarbase	hsa-miR-181a-5p	GBA2	57704	ENSG00000070610	validated
tarbase	hsa-miR-15b-5p	NDST1	3340	ENSG00000070614	validated
tarbase	hsa-miR-18a-5p	NDST1	3340	ENSG00000070614	validated
tarbase	hsa-miR-21-5p	ASNS	440	ENSG00000070669	validated
tarbase	hsa-miR-15b-5p	ST6GALNAC2	10610	ENSG00000070731	validated
tarbase	hsa-miR-212-3p	ST6GALNAC2	10610	ENSG00000070731	validated
tarbase	hsa-miR-18a-5p	PABPC1	26986	ENSG00000070756	validated
tarbase	hsa-miR-212-3p	PABPC1	26986	ENSG00000070756	validated
tarbase	hsa-miR-222-3p	PABPC1	26986	ENSG00000070756	validated
tarbase	hsa-miR-212-3p	CSNK2A2	1459	ENSG00000070770	validated
tarbase	hsa-miR-21-5p	PTPN21	11099	ENSG00000070778	validated
tarbase	hsa-miR-15b-5p	CDC42	998	ENSG00000070831	validated
tarbase	hsa-miR-582-3p	CDC42	998	ENSG00000070831	validated
tarbase	hsa-miR-106b-5p	RAD18	56852	ENSG00000070950	validated
tarbase	hsa-miR-181a-5p	RAD18	56852	ENSG00000070950	validated
tarbase	hsa-miR-15b-5p	ATP2B1	490	ENSG00000070961	validated
tarbase	hsa-miR-18a-5p	ATP2B1	490	ENSG00000070961	validated
tarbase	hsa-miR-212-3p	ATP2B1	490	ENSG00000070961	validated
tarbase	hsa-miR-21-5p	ATP2B1	490	ENSG00000070961	validated
tarbase	hsa-miR-18a-5p	MAP4K4	9448	ENSG00000071054	validated
tarbase	hsa-miR-144-3p	MGAT4A	11320	ENSG00000071073	validated
tarbase	hsa-miR-15b-5p	MGAT4A	11320	ENSG00000071073	validated
tarbase	hsa-miR-301a-3p	MGAT4A	11320	ENSG00000071073	validated
tarbase	hsa-miR-212-3p	RPL31	6160	ENSG00000071082	validated
tarbase	hsa-miR-301a-3p	WDR1	9948	ENSG00000071127	validated
tarbase	hsa-miR-18a-5p	SNX13	23161	ENSG00000071189	validated
tarbase	hsa-miR-21-5p	SNX13	23161	ENSG00000071189	validated
tarbase	hsa-miR-106b-5p	ING3	54556	ENSG00000071243	validated
tarbase	hsa-miR-181a-5p	VASH1	22846	ENSG00000071246	validated
tarbase	hsa-miR-106b-5p	LMCD1	29995	ENSG00000071282	validated
tarbase	hsa-miR-106b-5p	BUD23	114049	ENSG00000071462	validated
tarbase	hsa-miR-18a-5p	BUD23	114049	ENSG00000071462	validated
tarbase	hsa-miR-301a-3p	BUD23	114049	ENSG00000071462	validated
tarbase	hsa-miR-181a-5p	SEL1L	6400	ENSG00000071537	validated
tarbase	hsa-miR-18a-5p	SEL1L	6400	ENSG00000071537	validated
tarbase	hsa-miR-18a-5p	TRIB2	28951	ENSG00000071575	validated
tarbase	hsa-miR-15b-5p	MBD3	53615	ENSG00000071655	validated
tarbase	hsa-miR-181a-5p	MBD3	53615	ENSG00000071655	validated
tarbase	hsa-miR-18a-5p	MBD3	53615	ENSG00000071655	validated
tarbase	hsa-miR-296-5p	MBD3	53615	ENSG00000071655	validated
tarbase	hsa-miR-15b-5p	RDH11	51109	ENSG00000072042	validated
tarbase	hsa-miR-192-5p	RDH11	51109	ENSG00000072042	validated
tarbase	hsa-miR-15b-5p	PRKACA	5566	ENSG00000072062	validated
tarbase	hsa-miR-15b-5p	ADGRL1	22859	ENSG00000072071	validated
tarbase	hsa-miR-181a-5p	ZFYVE26	23503	ENSG00000072121	validated
tarbase	hsa-miR-18a-5p	ZFYVE26	23503	ENSG00000072121	validated
tarbase	hsa-miR-192-5p	ZFYVE26	23503	ENSG00000072121	validated
tarbase	hsa-miR-212-3p	ZFYVE26	23503	ENSG00000072121	validated
tarbase	hsa-miR-181a-5p	EPN2	22905	ENSG00000072134	validated
tarbase	hsa-miR-296-5p	ALDH3A2	224	ENSG00000072210	validated
tarbase	hsa-miR-106b-5p	ALDH3A2	224	ENSG00000072210	validated
tarbase	hsa-miR-21-5p	TFRC	7037	ENSG00000072274	validated
tarbase	hsa-miR-582-3p	TFRC	7037	ENSG00000072274	validated
tarbase	hsa-miR-18a-5p	SREBF1	6720	ENSG00000072310	validated
tarbase	hsa-miR-192-5p	SREBF1	6720	ENSG00000072310	validated
tarbase	hsa-miR-106b-5p	AFF4	27125	ENSG00000072364	validated
tarbase	hsa-miR-21-5p	AFF4	27125	ENSG00000072364	validated

tarbase	hsa-miR-505-3p	AFF4	27125	ENSG00000072364	validated
tarbase	hsa-miR-15b-5p	UBE2D1	7321	ENSG00000072401	validated
tarbase	hsa-miR-222-3p	UBE2D1	7321	ENSG00000072401	validated
tarbase	hsa-miR-326	UBE2D1	7321	ENSG00000072401	validated
tarbase	hsa-miR-21-5p	UBE2D1	7321	ENSG00000072401	validated
tarbase	hsa-miR-301a-3p	MPP5	64398	ENSG00000072415	validated
tarbase	hsa-miR-106b-5p	SMC1A	8243	ENSG00000072501	validated
tarbase	hsa-miR-181a-5p	SMC1A	8243	ENSG00000072501	validated
tarbase	hsa-miR-181a-5p	MARK2	2011	ENSG00000072518	validated
tarbase	hsa-miR-18a-5p	MARK2	2011	ENSG00000072518	validated
tarbase	hsa-miR-212-3p	HMMR	3161	ENSG00000072571	validated
tarbase	hsa-miR-15b-5p	NFATC3	4775	ENSG00000072736	validated
tarbase	hsa-miR-18a-5p	NFATC3	4775	ENSG00000072736	validated
tarbase	hsa-miR-192-5p	NFATC3	4775	ENSG00000072736	validated
tarbase	hsa-miR-222-3p	NFATC3	4775	ENSG00000072736	validated
tarbase	hsa-miR-212-3p	FBXW11	23291	ENSG00000072803	validated
tarbase	hsa-miR-106b-5p	DERL2	51009	ENSG00000072849	validated
tarbase	hsa-miR-181a-5p	DERL2	51009	ENSG00000072849	validated
tarbase	hsa-miR-106b-5p	MCM2	4171	ENSG00000073111	validated
tarbase	hsa-miR-15b-5p	CLCN4	1183	ENSG00000073464	validated
tarbase	hsa-miR-505-3p	KDM5A	5927	ENSG00000073614	validated
tarbase	hsa-miR-582-5p	KDM5A	5927	ENSG00000073614	validated
tarbase	hsa-miR-106b-5p	PPP2R3A	5523	ENSG00000073711	validated
tarbase	hsa-miR-181a-5p	PPP2R3A	5523	ENSG00000073711	validated
tarbase	hsa-miR-18a-5p	PPP2R3A	5523	ENSG00000073711	validated
tarbase	hsa-miR-106b-5p	FERMT2	10979	ENSG00000073712	validated
tarbase	hsa-miR-222-3p	FERMT2	10979	ENSG00000073712	validated
tarbase	hsa-miR-212-3p	PTGS2	5743	ENSG00000073756	validated
tarbase	hsa-miR-582-5p	PTGS2	5743	ENSG00000073756	validated
tarbase	hsa-miR-181a-5p	MAP3K13	9175	ENSG00000073803	validated
tarbase	hsa-miR-212-3p	PICALM	8301	ENSG00000073921	validated
tarbase	hsa-miR-15b-5p	NSF	4905	ENSG00000073969	validated
tarbase	hsa-miR-15b-5p	GLI2	2736	ENSG00000074047	validated
tarbase	hsa-miR-15b-5p	CLASP1	23332	ENSG00000074054	validated
tarbase	hsa-miR-181a-5p	CLASP1	23332	ENSG00000074054	validated
tarbase	hsa-miR-15b-5p	NOTCH3	4854	ENSG00000074181	validated
tarbase	hsa-miR-15b-5p	CLNS1A	1207	ENSG00000074201	validated
tarbase	hsa-miR-21-5p	CLNS1A	1207	ENSG00000074201	validated
tarbase	hsa-miR-326	TEAD2	8463	ENSG00000074219	validated
tarbase	hsa-miR-582-5p	TSG101	7251	ENSG00000074319	validated
tarbase	hsa-miR-212-3p	NCBP3	55421	ENSG00000074356	validated
tarbase	hsa-miR-18a-5p	ATP2A3	489	ENSG00000074370	validated
tarbase	hsa-miR-326	ATP2A3	489	ENSG00000074370	validated
tarbase	hsa-miR-181a-5p	CA12	771	ENSG00000074410	validated
tarbase	hsa-miR-301a-3p	CA12	771	ENSG00000074410	validated
tarbase	hsa-miR-106b-5p	NTN4	59277	ENSG00000074527	validated
tarbase	hsa-miR-212-3p	NTN4	59277	ENSG00000074527	validated
tarbase	hsa-miR-18a-5p	DPP8	54878	ENSG00000074603	validated
tarbase	hsa-miR-21-5p	DPP8	54878	ENSG00000074603	validated
tarbase	hsa-miR-301a-3p	DPP8	54878	ENSG00000074603	validated
tarbase	hsa-miR-15b-5p	ZNF532	55205	ENSG00000074657	validated
tarbase	hsa-miR-15b-5p	LMAN1	3998	ENSG00000074695	validated
tarbase	hsa-miR-15b-5p	IPCEF1	26034	ENSG00000074706	validated
tarbase	hsa-miR-106b-5p	ZZEF1	23140	ENSG00000074755	validated
tarbase	hsa-miR-326	ZZEF1	23140	ENSG00000074755	validated
tarbase	hsa-miR-106b-5p	ENO1	2023	ENSG00000074800	validated
tarbase	hsa-miR-181a-5p	ENO1	2023	ENSG00000074800	validated
tarbase	hsa-miR-296-5p	ENO1	2023	ENSG00000074800	validated
tarbase	hsa-miR-301a-3p	ENO1	2023	ENSG00000074800	validated
tarbase	hsa-miR-18a-5p	MYDGF	56005	ENSG00000074842	validated
tarbase	hsa-miR-212-3p	TUBE1	51175	ENSG00000074935	validated
tarbase	hsa-miR-106b-5p	ACTR6	64431	ENSG00000075089	validated
tarbase	hsa-miR-106b-5p	TIPIN	54962	ENSG00000075131	validated
tarbase	hsa-miR-144-3p	TIPIN	54962	ENSG00000075131	validated
tarbase	hsa-miR-21-5p	TIPIN	54962	ENSG00000075131	validated
tarbase	hsa-miR-106b-5p	EIF4G3	8672	ENSG00000075151	validated
tarbase	hsa-miR-15b-5p	EIF4G3	8672	ENSG00000075151	validated
tarbase	hsa-miR-21-5p	EIF4G3	8672	ENSG00000075151	validated
tarbase	hsa-miR-222-3p	EIF4G3	8672	ENSG00000075151	validated
tarbase	hsa-miR-106b-5p	SEMA3A	10371	ENSG00000075213	validated
tarbase	hsa-miR-15b-5p	SEMA3A	10371	ENSG00000075213	validated

tarbase	hsa-miR-212-3p	SEMA3A	10371	ENSG00000075213	validated
tarbase	hsa-miR-181a-5p	GTSE1	51512	ENSG00000075218	validated
tarbase	hsa-miR-15b-5p	SEMA3C	10512	ENSG00000075223	validated
tarbase	hsa-miR-181a-5p	SEMA3C	10512	ENSG00000075223	validated
tarbase	hsa-miR-106b-5p	SEMA3C	10512	ENSG00000075223	validated
tarbase	hsa-miR-301a-3p	CELSR1	9620	ENSG00000075275	validated
tarbase	hsa-miR-144-3p	ZNF638	27332	ENSG00000075292	validated
tarbase	hsa-miR-181a-5p	ZNF638	27332	ENSG00000075292	validated
tarbase	hsa-miR-222-3p	ZNF638	27332	ENSG00000075292	validated
tarbase	hsa-miR-326	ZNF638	27332	ENSG00000075292	validated
tarbase	hsa-miR-212-3p	SLC25A40	55972	ENSG00000075303	validated
tarbase	hsa-miR-505-3p	SLC25A40	55972	ENSG00000075303	validated
tarbase	hsa-miR-582-3p	TIMM21	29090	ENSG00000075336	validated
tarbase	hsa-miR-212-3p	VPS9D1	9605	ENSG00000075399	validated
tarbase	hsa-miR-326	ZNF37A	7587	ENSG00000075407	validated
tarbase	hsa-miR-181a-5p	ZNF37A	7587	ENSG00000075407	validated
tarbase	hsa-miR-21-5p	MARK3	4140	ENSG00000075413	validated
tarbase	hsa-miR-15b-5p	SLC25A3	5250	ENSG00000075415	validated
tarbase	hsa-miR-212-3p	SLC25A3	5250	ENSG00000075415	validated
tarbase	hsa-miR-301a-3p	SLC25A3	5250	ENSG00000075415	validated
tarbase	hsa-miR-106b-5p	FNDC3B	64778	ENSG00000075420	validated
tarbase	hsa-miR-15b-5p	FNDC3B	64778	ENSG00000075420	validated
tarbase	hsa-miR-212-3p	FNDC3B	64778	ENSG00000075420	validated
tarbase	hsa-miR-21-5p	FNDC3B	64778	ENSG00000075420	validated
tarbase	hsa-miR-222-3p	FNDC3B	64778	ENSG00000075420	validated
tarbase	hsa-miR-582-3p	FNDC3B	64778	ENSG00000075420	validated
tarbase	hsa-miR-15b-5p	FOSL2	2355	ENSG00000075426	validated
tarbase	hsa-miR-301a-3p	FOSL2	2355	ENSG00000075426	validated
tarbase	hsa-miR-192-5p	FSCN1	6624	ENSG00000075618	validated
tarbase	hsa-miR-15b-5p	ACTB	60	ENSG00000075624	validated
tarbase	hsa-miR-181a-5p	ACTB	60	ENSG00000075624	validated
tarbase	hsa-miR-18a-5p	ACTB	60	ENSG00000075624	validated
tarbase	hsa-miR-192-5p	ACTB	60	ENSG00000075624	validated
tarbase	hsa-miR-21-5p	ACTB	60	ENSG00000075624	validated
tarbase	hsa-miR-144-3p	RAB7A	7879	ENSG00000075785	validated
tarbase	hsa-miR-212-3p	RAB7A	7879	ENSG00000075785	validated
tarbase	hsa-miR-15b-5p	BCAP29	55973	ENSG00000075790	validated
tarbase	hsa-miR-181a-5p	BCAP29	55973	ENSG00000075790	validated
tarbase	hsa-miR-15b-5p	KIFAP3	22920	ENSG00000075945	validated
tarbase	hsa-miR-192-5p	KIFAP3	22920	ENSG00000075945	validated
tarbase	hsa-miR-18a-5p	MKRN2	23609	ENSG00000075975	validated
tarbase	hsa-miR-21-5p	MCM6	4175	ENSG00000076003	validated
tarbase	hsa-miR-212-3p	MCM6	4175	ENSG00000076003	validated
tarbase	hsa-miR-106b-5p	RBM7	10179	ENSG00000076053	validated
tarbase	hsa-miR-15b-5p	RBM7	10179	ENSG00000076053	validated
tarbase	hsa-miR-21-5p	RBM7	10179	ENSG00000076053	validated
tarbase	hsa-miR-144-3p	BAZ2A	11176	ENSG00000076108	validated
tarbase	hsa-miR-18a-5p	BAZ2A	11176	ENSG00000076108	validated
tarbase	hsa-miR-301a-3p	BAZ2A	11176	ENSG00000076108	validated
tarbase	hsa-miR-582-3p	BAZ2A	11176	ENSG00000076108	validated
tarbase	hsa-miR-15b-5p	UNG	7374	ENSG00000076248	validated
tarbase	hsa-miR-18a-5p	UNG	7374	ENSG00000076248	validated
tarbase	hsa-miR-106b-5p	UNG	7374	ENSG00000076248	validated
tarbase	hsa-miR-18a-5p	KLHL20	27252	ENSG00000076321	validated
tarbase	hsa-miR-301a-3p	KLHL20	27252	ENSG00000076321	validated
tarbase	hsa-miR-15b-5p	PLXNA2	5362	ENSG00000076356	validated
tarbase	hsa-miR-21-5p	PLXNA2	5362	ENSG00000076356	validated
tarbase	hsa-miR-144-3p	TPD52	7163	ENSG00000076554	validated
tarbase	hsa-miR-222-3p	TPD52	7163	ENSG00000076554	validated
tarbase	hsa-miR-301a-3p	TPD52	7163	ENSG00000076554	validated
tarbase	hsa-miR-15b-5p	TRAF4	9618	ENSG00000076604	validated
tarbase	hsa-miR-301a-3p	TRAF4	9618	ENSG00000076604	validated
tarbase	hsa-miR-582-3p	TRAF4	9618	ENSG00000076604	validated
tarbase	hsa-miR-21-5p	MCAM	4162	ENSG00000076706	validated
tarbase	hsa-miR-15b-5p	GPC4	2239	ENSG00000076716	validated
tarbase	hsa-miR-326	GPC4	2239	ENSG00000076716	validated
tarbase	hsa-miR-21-5p	MAP2K7	5609	ENSG00000076984	validated
tarbase	hsa-miR-212-3p	NMRK2	27231	ENSG00000077009	validated
tarbase	hsa-miR-15b-5p	CTTNBP2	83992	ENSG00000077063	validated
tarbase	hsa-miR-582-5p	RARB	5915	ENSG00000077092	validated
tarbase	hsa-miR-106b-5p	TM9SF3	56889	ENSG00000077147	validated

tarbase	hsa-miR-192-5p	TM9SF3	56889	ENSG00000077147	validated
tarbase	hsa-miR-212-3p	TM9SF3	56889	ENSG00000077147	validated
tarbase	hsa-miR-582-3p	TM9SF3	56889	ENSG00000077147	validated
tarbase	hsa-miR-15b-5p	UBE2T	29089	ENSG00000077152	validated
tarbase	hsa-miR-21-5p	UBE2T	29089	ENSG00000077152	validated
tarbase	hsa-miR-505-3p	UBE2T	29089	ENSG00000077152	validated
tarbase	hsa-miR-15b-5p	PPP1R12B	4660	ENSG00000077157	validated
tarbase	hsa-miR-181a-5p	DNAJC10	54431	ENSG00000077232	validated
tarbase	hsa-miR-181a-5p	USP33	23032	ENSG00000077254	validated
tarbase	hsa-miR-301a-3p	USP33	23032	ENSG00000077254	validated
tarbase	hsa-miR-18a-5p	SNRPA	6626	ENSG00000077312	validated
tarbase	hsa-miR-15b-5p	POLD3	10714	ENSG00000077514	validated
tarbase	hsa-miR-15b-5p	GPR137B	7107	ENSG00000077585	validated
tarbase	hsa-miR-192-5p	GPR137B	7107	ENSG00000077585	validated
tarbase	hsa-miR-212-3p	GPR137B	7107	ENSG00000077585	validated
tarbase	hsa-miR-21-5p	GPR137B	7107	ENSG00000077585	validated
tarbase	hsa-miR-301a-3p	JADE1	79960	ENSG00000077684	validated
tarbase	hsa-miR-21-5p	SLC25A43	203427	ENSG00000077713	validated
tarbase	hsa-miR-15b-5p	FGFR1	2260	ENSG00000077782	validated
tarbase	hsa-miR-144-3p	ITGA8	8516	ENSG00000077943	validated
tarbase	hsa-miR-222-3p	ITGA8	8516	ENSG00000077943	validated
tarbase	hsa-miR-15b-5p	PIAS2	9063	ENSG00000078043	validated
tarbase	hsa-miR-181a-5p	PIAS2	9063	ENSG00000078043	validated
tarbase	hsa-miR-106b-5p	LAMP3	27074	ENSG00000078081	validated
tarbase	hsa-miR-212-3p	FAP	2191	ENSG00000078098	validated
tarbase	hsa-miR-106b-5p	NEBL	10529	ENSG00000078114	validated
tarbase	hsa-miR-15b-5p	NEBL	10529	ENSG00000078114	validated
tarbase	hsa-miR-181a-5p	NEBL	10529	ENSG00000078114	validated
tarbase	hsa-miR-106b-5p	PIK3C3	5289	ENSG00000078142	validated
tarbase	hsa-miR-106b-5p	N4BP2	55728	ENSG00000078177	validated
tarbase	hsa-miR-181a-5p	N4BP2	55728	ENSG00000078177	validated
tarbase	hsa-miR-15b-5p	TIGAR	57103	ENSG00000078237	validated
tarbase	hsa-miR-181a-5p	PPP2R5C	5527	ENSG00000078304	validated
tarbase	hsa-miR-212-3p	PPP2R5C	5527	ENSG00000078304	validated
tarbase	hsa-miR-301a-3p	PPP2R5C	5527	ENSG00000078304	validated
tarbase	hsa-miR-181a-5p	RBFOX1	54715	ENSG00000078328	validated
tarbase	hsa-miR-181a-5p	GNB1	2782	ENSG00000078369	validated
tarbase	hsa-miR-301a-3p	GNB1	2782	ENSG00000078369	validated
tarbase	hsa-miR-212-3p	HOXA9	3205	ENSG00000078399	validated
tarbase	hsa-miR-222-3p	HOXA9	3205	ENSG00000078399	validated
tarbase	hsa-miR-301a-3p	HOXA9	3205	ENSG00000078399	validated
tarbase	hsa-miR-582-5p	HOXA9	3205	ENSG00000078399	validated
tarbase	hsa-miR-181a-5p	HOXA9	3205	ENSG00000078399	validated
tarbase	hsa-miR-212-3p	EDN1	1906	ENSG00000078401	validated
tarbase	hsa-miR-144-3p	EDN1	1906	ENSG00000078401	validated
tarbase	hsa-miR-106b-5p	MLLT10	8028	ENSG00000078403	validated
tarbase	hsa-miR-301a-3p	MLLT10	8028	ENSG00000078403	validated
tarbase	hsa-miR-181a-5p	MLLT10	8028	ENSG00000078403	validated
tarbase	hsa-miR-15b-5p	VDAC3	7419	ENSG00000078668	validated
tarbase	hsa-miR-15b-5p	PCM1	5108	ENSG00000078674	validated
tarbase	hsa-miR-181a-5p	PCM1	5108	ENSG00000078674	validated
tarbase	hsa-miR-106b-5p	TNRC6C	57690	ENSG00000078687	validated
tarbase	hsa-miR-15b-5p	TNRC6C	57690	ENSG00000078687	validated
tarbase	hsa-miR-181a-5p	TNRC6C	57690	ENSG00000078687	validated
tarbase	hsa-miR-301a-3p	TNRC6C	57690	ENSG00000078687	validated
tarbase	hsa-miR-18a-5p	CBFA2T2	9139	ENSG00000078699	validated
tarbase	hsa-miR-181a-5p	ITCH	83737	ENSG00000078747	validated
tarbase	hsa-miR-212-3p	ITCH	83737	ENSG00000078747	validated
tarbase	hsa-miR-582-3p	ITCH	83737	ENSG00000078747	validated
tarbase	hsa-miR-106b-5p	TP53INP2	58476	ENSG00000078804	validated
tarbase	hsa-miR-212-3p	TP53INP2	58476	ENSG00000078804	validated
tarbase	hsa-miR-106b-5p	TOLLIP	54472	ENSG00000078902	validated
tarbase	hsa-miR-212-3p	RUNX1T1	862	ENSG00000079102	validated
tarbase	hsa-miR-106b-5p	FKBP7	51661	ENSG00000079150	validated
tarbase	hsa-miR-212-3p	FKBP7	51661	ENSG00000079150	validated
tarbase	hsa-miR-21-5p	OSBPL6	114880	ENSG00000079156	validated
tarbase	hsa-miR-144-3p	XRCC5	7520	ENSG00000079246	validated
tarbase	hsa-miR-15b-5p	XRCC5	7520	ENSG00000079246	validated
tarbase	hsa-miR-21-5p	XRCC5	7520	ENSG00000079246	validated
tarbase	hsa-miR-21-5p	TNS1	7145	ENSG00000079308	validated
tarbase	hsa-miR-15b-5p	CDC14A	8556	ENSG00000079335	validated

tarbase	hsa-miR-21-5p	SENP1	29843	ENSG00000079387	validated
tarbase	hsa-miR-106b-5p	CIC	23152	ENSG00000079432	validated
tarbase	hsa-miR-296-5p	CIC	23152	ENSG00000079432	validated
tarbase	hsa-miR-301a-3p	CIC	23152	ENSG00000079432	validated
tarbase	hsa-miR-181a-5p	FDFT1	2222	ENSG00000079459	validated
tarbase	hsa-miR-15b-5p	KIF22	3835	ENSG00000079616	validated
tarbase	hsa-miR-15b-5p	DDX1	1653	ENSG00000079785	validated
tarbase	hsa-miR-21-5p	DDX1	1653	ENSG00000079785	validated
tarbase	hsa-miR-21-5p	DNM2	1785	ENSG00000079805	validated
tarbase	hsa-miR-301a-3p	DNM2	1785	ENSG00000079805	validated
tarbase	hsa-miR-15b-5p	EPB41L2	2037	ENSG00000079819	validated
tarbase	hsa-miR-106b-5p	MOXD1	26002	ENSG00000079931	validated
tarbase	hsa-miR-181a-5p	STX7	8417	ENSG00000079950	validated
tarbase	hsa-miR-181a-5p	SLC35C2	51006	ENSG00000080189	validated
tarbase	hsa-miR-106b-5p	CRYBG3	131544	ENSG00000080200	validated
tarbase	hsa-miR-181a-5p	CRYBG3	131544	ENSG00000080200	validated
tarbase	hsa-miR-212-3p	CRYBG3	131544	ENSG00000080200	validated
tarbase	hsa-miR-212-3p	EPHA6	285220	ENSG00000080224	validated
tarbase	hsa-miR-15b-5p	RFX3	5991	ENSG00000080298	validated
tarbase	hsa-miR-106b-5p	RIF1	55183	ENSG00000080345	validated
tarbase	hsa-miR-181a-5p	RIF1	55183	ENSG00000080345	validated
tarbase	hsa-miR-505-3p	RIF1	55183	ENSG00000080345	validated
tarbase	hsa-miR-192-5p	RAB21	23011	ENSG00000080371	validated
tarbase	hsa-miR-582-3p	RAB21	23011	ENSG00000080371	validated
tarbase	hsa-miR-106b-5p	SLC4A4	8671	ENSG00000080493	validated
tarbase	hsa-miR-21-5p	SMARCA2	6595	ENSG00000080503	validated
tarbase	hsa-miR-15b-5p	SESN1	27244	ENSG00000080546	validated
tarbase	hsa-miR-181a-5p	SESN1	27244	ENSG00000080546	validated
tarbase	hsa-miR-212-3p	SESN1	27244	ENSG00000080546	validated
tarbase	hsa-miR-212-3p	PIH1D3	139212	ENSG00000080572	validated
tarbase	hsa-miR-301a-3p	COL5A3	50509	ENSG00000080573	validated
tarbase	hsa-miR-15b-5p	SRCAP	10847	ENSG00000080603	validated
tarbase	hsa-miR-18a-5p	SRCAP	10847	ENSG00000080603	validated
tarbase	hsa-miR-296-5p	SRCAP	10847	ENSG00000080603	validated
tarbase	hsa-miR-582-3p	SRCAP	10847	ENSG00000080603	validated
tarbase	hsa-miR-21-5p	PUM3	9933	ENSG00000080608	validated
tarbase	hsa-miR-106b-5p	KCNN2	3781	ENSG00000080709	validated
tarbase	hsa-miR-212-3p	KCNN2	3781	ENSG00000080709	validated
tarbase	hsa-miR-15b-5p	CNOT4	4850	ENSG00000080802	validated
tarbase	hsa-miR-181a-5p	CNOT4	4850	ENSG00000080802	validated
tarbase	hsa-miR-18a-5p	CNOT4	4850	ENSG00000080802	validated
tarbase	hsa-miR-582-5p	CNOT4	4850	ENSG00000080802	validated
tarbase	hsa-miR-582-5p	CPOX	1371	ENSG00000080819	validated
tarbase	hsa-miR-222-3p	CLDND1	56650	ENSG00000080822	validated
tarbase	hsa-miR-15b-5p	CLDND1	56650	ENSG00000080822	validated
tarbase	hsa-miR-106b-5p	HSP90AA1	3320	ENSG00000080824	validated
tarbase	hsa-miR-15b-5p	HSP90AA1	3320	ENSG00000080824	validated
tarbase	hsa-miR-181a-5p	HSP90AA1	3320	ENSG00000080824	validated
tarbase	hsa-miR-301a-3p	HSP90AA1	3320	ENSG00000080824	validated
tarbase	hsa-miR-15b-5p	RBL1	5933	ENSG00000080839	validated
tarbase	hsa-miR-212-3p	IGSF9B	22997	ENSG00000080854	validated
tarbase	hsa-miR-212-3p	NDC80	10403	ENSG00000080986	validated
tarbase	hsa-miR-106b-5p	RSBN1	54665	ENSG00000081019	validated
tarbase	hsa-miR-15b-5p	RSBN1	54665	ENSG00000081019	validated
tarbase	hsa-miR-192-5p	CXCL2	2920	ENSG00000081041	validated
tarbase	hsa-miR-144-3p	OSTM1	28962	ENSG00000081087	validated
tarbase	hsa-miR-222-3p	OSTM1	28962	ENSG00000081087	validated
tarbase	hsa-miR-106b-5p	PCNP	57092	ENSG00000081154	validated
tarbase	hsa-miR-181a-5p	PCNP	57092	ENSG00000081154	validated
tarbase	hsa-miR-15b-5p	PKP1	5317	ENSG00000081277	validated
tarbase	hsa-miR-582-5p	UBA5	79876	ENSG00000081307	validated
tarbase	hsa-miR-15b-5p	ZNF510	22869	ENSG00000081386	validated
tarbase	hsa-miR-181a-5p	ZNF510	22869	ENSG00000081386	validated
tarbase	hsa-miR-222-3p	ZNF510	22869	ENSG00000081386	validated
tarbase	hsa-miR-181a-5p	ZNF506	440515	ENSG00000081665	validated
tarbase	hsa-miR-582-3p	PHLPP1	23239	ENSG00000081913	validated
tarbase	hsa-miR-106b-5p	ATP8B1	5205	ENSG00000081923	validated
tarbase	hsa-miR-212-3p	PGR	5241	ENSG00000082175	validated
tarbase	hsa-miR-18a-5p	C5orf22	55322	ENSG00000082213	validated
tarbase	hsa-miR-326	C5orf22	55322	ENSG00000082213	validated
tarbase	hsa-miR-106b-5p	CCNT2	905	ENSG00000082258	validated

tarbase	hsa-miR-144-3p	CCNT2	905	ENSG00000082258	validated
tarbase	hsa-miR-181a-5p	CCNT2	905	ENSG00000082258	validated
tarbase	hsa-miR-212-3p	CCNT2	905	ENSG00000082258	validated
tarbase	hsa-miR-106b-5p	FAM135A	57579	ENSG00000082269	validated
tarbase	hsa-miR-15b-5p	FAM135A	57579	ENSG00000082269	validated
tarbase	hsa-miR-181a-5p	FAM135A	57579	ENSG00000082269	validated
tarbase	hsa-miR-301a-3p	FAM135A	57579	ENSG00000082269	validated
tarbase	hsa-miR-181a-5p	EPB41L3	23136	ENSG00000082397	validated
tarbase	hsa-miR-106b-5p	KCNK2	3776	ENSG00000082482	validated
tarbase	hsa-miR-212-3p	SERTAD4	56256	ENSG00000082497	validated
tarbase	hsa-miR-15b-5p	TRAF5	7188	ENSG00000082512	validated
tarbase	hsa-miR-18a-5p	TRAF5	7188	ENSG00000082512	validated
tarbase	hsa-miR-212-3p	GEMIN5	25929	ENSG00000082516	validated
tarbase	hsa-miR-505-3p	GEMIN5	25929	ENSG00000082516	validated
tarbase	hsa-miR-106b-5p	NFE2L1	4779	ENSG00000082641	validated
tarbase	hsa-miR-15b-5p	NFE2L1	4779	ENSG00000082641	validated
tarbase	hsa-miR-181a-5p	NFE2L1	4779	ENSG00000082641	validated
tarbase	hsa-miR-21-5p	NFE2L1	4779	ENSG00000082641	validated
tarbase	hsa-miR-18a-5p	NFE2L1	4779	ENSG00000082641	validated
tarbase	hsa-miR-212-3p	GSK3B	2932	ENSG00000082701	validated
tarbase	hsa-miR-582-3p	GSK3B	2932	ENSG00000082701	validated
tarbase	hsa-miR-106b-5p	ERC1	23085	ENSG00000082805	validated
tarbase	hsa-miR-15b-5p	XPO1	7514	ENSG00000082898	validated
tarbase	hsa-miR-181a-5p	XPO1	7514	ENSG00000082898	validated
tarbase	hsa-miR-18a-5p	XPO1	7514	ENSG00000082898	validated
tarbase	hsa-miR-326	XPO1	7514	ENSG00000082898	validated
tarbase	hsa-miR-212-3p	XPO1	7514	ENSG00000082898	validated
tarbase	hsa-miR-301a-3p	XPO1	7514	ENSG00000082898	validated
tarbase	hsa-miR-21-5p	PALB2	79728	ENSG00000083093	validated
tarbase	hsa-miR-212-3p	DOP1A	23033	ENSG00000083097	validated
tarbase	hsa-miR-21-5p	LYRM2	57226	ENSG00000083099	validated
tarbase	hsa-miR-106b-5p	KAT6A	7994	ENSG00000083168	validated
tarbase	hsa-miR-144-3p	KAT6A	7994	ENSG00000083168	validated
tarbase	hsa-miR-181a-5p	KAT6A	7994	ENSG00000083168	validated
tarbase	hsa-miR-18a-5p	KAT6A	7994	ENSG00000083168	validated
tarbase	hsa-miR-301a-3p	ULK2	9706	ENSG00000083290	validated
tarbase	hsa-miR-326	ULK2	9706	ENSG00000083290	validated
tarbase	hsa-miR-15b-5p	TNPO1	3842	ENSG00000083312	validated
tarbase	hsa-miR-192-5p	TNPO1	3842	ENSG00000083312	validated
tarbase	hsa-miR-301a-3p	TNPO1	3842	ENSG00000083312	validated
tarbase	hsa-miR-582-3p	TNPO1	3842	ENSG00000083312	validated
tarbase	hsa-miR-181a-5p	DIS3	22894	ENSG00000083520	validated
tarbase	hsa-miR-582-5p	DIS3	22894	ENSG00000083520	validated
tarbase	hsa-miR-181a-5p	PIBF1	10464	ENSG00000083535	validated
tarbase	hsa-miR-212-3p	PIBF1	10464	ENSG00000083535	validated
tarbase	hsa-miR-106b-5p	TDRD3	81550	ENSG00000083544	validated
tarbase	hsa-miR-15b-5p	PDS5B	23047	ENSG00000083642	validated
tarbase	hsa-miR-181a-5p	PDS5B	23047	ENSG00000083642	validated
tarbase	hsa-miR-18a-5p	PDS5B	23047	ENSG00000083642	validated
tarbase	hsa-miR-212-3p	PDS5B	23047	ENSG00000083642	validated
tarbase	hsa-miR-301a-3p	CYLD	1540	ENSG00000083799	validated
tarbase	hsa-miR-181a-5p	CYLD	1540	ENSG00000083799	validated
tarbase	hsa-miR-15b-5p	ZNF264	9422	ENSG00000083844	validated
tarbase	hsa-miR-181a-5p	ZNF264	9422	ENSG00000083844	validated
tarbase	hsa-miR-18a-5p	ZNF264	9422	ENSG00000083844	validated
tarbase	hsa-miR-212-3p	ZNF264	9422	ENSG00000083844	validated
tarbase	hsa-miR-301a-3p	ZNF264	9422	ENSG00000083844	validated
tarbase	hsa-miR-15b-5p	FAT1	2195	ENSG00000083857	validated
tarbase	hsa-miR-582-3p	FAT1	2195	ENSG00000083857	validated
tarbase	hsa-miR-582-5p	FAT1	2195	ENSG00000083857	validated
tarbase	hsa-miR-181a-5p	YTHDC1	91746	ENSG00000083896	validated
tarbase	hsa-miR-106b-5p	ZMPSTE24	10269	ENSG00000084073	validated
tarbase	hsa-miR-15b-5p	ZMPSTE24	10269	ENSG00000084073	validated
tarbase	hsa-miR-106b-5p	STARD7	56910	ENSG00000084090	validated
tarbase	hsa-miR-18a-5p	STARD7	56910	ENSG00000084090	validated
tarbase	hsa-miR-181a-5p	STARD7	56910	ENSG00000084090	validated
tarbase	hsa-miR-144-3p	REST	5978	ENSG00000084093	validated
tarbase	hsa-miR-15b-5p	REST	5978	ENSG00000084093	validated
tarbase	hsa-miR-181a-5p	REST	5978	ENSG00000084093	validated
tarbase	hsa-miR-301a-3p	REST	5978	ENSG00000084093	validated
tarbase	hsa-miR-106b-5p	SSH1	54434	ENSG00000084112	validated

tarbase	hsa-miR-18a-5p	SSH1	54434	ENSG00000084112	validated
tarbase	hsa-miR-301a-3p	SSH1	54434	ENSG00000084112	validated
tarbase	hsa-miR-582-5p	SSH1	54434	ENSG00000084112	validated
tarbase	hsa-miR-15b-5p	GSTP1	2950	ENSG00000084207	validated
tarbase	hsa-miR-15b-5p	APLP2	334	ENSG00000084234	validated
tarbase	hsa-miR-181a-5p	APLP2	334	ENSG00000084234	validated
tarbase	hsa-miR-18a-5p	APLP2	334	ENSG00000084234	validated
tarbase	hsa-miR-222-3p	APLP2	334	ENSG00000084234	validated
tarbase	hsa-miR-505-3p	APLP2	334	ENSG00000084234	validated
tarbase	hsa-miR-582-5p	APLP2	334	ENSG00000084234	validated
tarbase	hsa-miR-15b-5p	FAM234B	57613	ENSG00000084444	validated
tarbase	hsa-miR-181a-5p	FAM234B	57613	ENSG00000084444	validated
tarbase	hsa-miR-181a-5p	EIF3I	8668	ENSG00000084623	validated
tarbase	hsa-miR-21-5p	EIF3I	8668	ENSG00000084623	validated
tarbase	hsa-miR-301a-3p	EIF3I	8668	ENSG00000084623	validated
tarbase	hsa-miR-212-3p	COL16A1	1307	ENSG00000084636	validated
tarbase	hsa-miR-18a-5p	TXLNA	200081	ENSG00000084652	validated
tarbase	hsa-miR-212-3p	TXLNA	200081	ENSG00000084652	validated
tarbase	hsa-miR-222-3p	TXLNA	200081	ENSG00000084652	validated
tarbase	hsa-miR-15b-5p	RAB10	10890	ENSG00000084733	validated
tarbase	hsa-miR-15b-5p	HADHA	3030	ENSG00000084754	validated
tarbase	hsa-miR-21-5p	HADHA	3030	ENSG00000084754	validated
tarbase	hsa-miR-15b-5p	MAPRE3	22924	ENSG00000084764	validated
tarbase	hsa-miR-15b-5p	CAD	790	ENSG00000084774	validated
tarbase	hsa-miR-181a-5p	CD59	966	ENSG00000085063	validated
tarbase	hsa-miR-106b-5p	BCORL1	63035	ENSG00000085185	validated
tarbase	hsa-miR-582-3p	ATRX	546	ENSG00000085224	validated
tarbase	hsa-miR-505-3p	AK6	102157402	ENSG00000085231	validated
tarbase	hsa-miR-181a-5p	MYNN	55892	ENSG00000085274	validated
tarbase	hsa-miR-582-3p	SCAMP1	9522	ENSG00000085365	validated
tarbase	hsa-miR-181a-5p	SCAMP1	9522	ENSG00000085365	validated
tarbase	hsa-miR-15b-5p	PREP	5550	ENSG00000085377	validated
tarbase	hsa-miR-181a-5p	HACE1	57531	ENSG00000085382	validated
tarbase	hsa-miR-222-3p	SEH1L	81929	ENSG00000085415	validated
tarbase	hsa-miR-582-5p	SEH1L	81929	ENSG00000085415	validated
tarbase	hsa-miR-21-5p	WDR47	22911	ENSG00000085433	validated
tarbase	hsa-miR-301a-3p	WDR47	22911	ENSG00000085433	validated
tarbase	hsa-miR-212-3p	WDR47	22911	ENSG00000085433	validated
tarbase	hsa-miR-301a-3p	WDFY1	57590	ENSG00000085449	validated
tarbase	hsa-miR-212-3p	OVGP1	5016	ENSG00000085465	validated
tarbase	hsa-miR-212-3p	SLC25A24	29957	ENSG00000085491	validated
tarbase	hsa-miR-21-5p	MAP3K4	4216	ENSG00000085511	validated
tarbase	hsa-miR-222-3p	MAP3K4	4216	ENSG00000085511	validated
tarbase	hsa-miR-326	ABCB1	5243	ENSG00000085563	validated
tarbase	hsa-miR-15b-5p	ABCB1	5243	ENSG00000085563	validated
tarbase	hsa-miR-181a-5p	CPNE3	8895	ENSG00000085719	validated
tarbase	hsa-miR-21-5p	CPNE3	8895	ENSG00000085719	validated
tarbase	hsa-miR-15b-5p	RRN3	54700	ENSG00000085721	validated
tarbase	hsa-miR-21-5p	RRN3	54700	ENSG00000085721	validated
tarbase	hsa-miR-18a-5p	CTTN	2017	ENSG00000085733	validated
tarbase	hsa-miR-21-5p	CTTN	2017	ENSG00000085733	validated
tarbase	hsa-miR-222-3p	CTTN	2017	ENSG00000085733	validated
tarbase	hsa-miR-15b-5p	EPS15	2060	ENSG00000085832	validated
tarbase	hsa-miR-301a-3p	EPS15	2060	ENSG00000085832	validated
tarbase	hsa-miR-296-5p	CHERP	10523	ENSG00000085872	validated
tarbase	hsa-miR-301a-3p	ATG16L1	55054	ENSG00000085978	validated
tarbase	hsa-miR-106b-5p	MAST2	23139	ENSG00000086015	validated
tarbase	hsa-miR-301a-3p	DNAJA1	3301	ENSG00000086061	validated
tarbase	hsa-miR-326	DNAJA1	3301	ENSG00000086061	validated
tarbase	hsa-miR-181a-5p	DNAJA1	3301	ENSG00000086061	validated
tarbase	hsa-miR-181a-5p	B4GALT1	2683	ENSG00000086062	validated
tarbase	hsa-miR-192-5p	B4GALT1	2683	ENSG00000086062	validated
tarbase	hsa-miR-301a-3p	NFX1	4799	ENSG00000086102	validated
tarbase	hsa-miR-212-3p	DIMT1	27292	ENSG00000086189	validated
tarbase	hsa-miR-21-5p	IPO11	51194	ENSG00000086200	validated
tarbase	hsa-miR-181a-5p	EPDR1	54749	ENSG00000086289	validated
tarbase	hsa-miR-18a-5p	EPDR1	54749	ENSG00000086289	validated
tarbase	hsa-miR-106b-5p	SEPHS1	22929	ENSG00000086475	validated
tarbase	hsa-miR-301a-3p	MRPL28	10573	ENSG00000086504	validated
tarbase	hsa-miR-15b-5p	RBM22	55696	ENSG00000086589	validated
tarbase	hsa-miR-21-5p	RBM22	55696	ENSG00000086589	validated

tarbase	hsa-miR-181a-5p	RBM22	55696	ENSG00000086589	validated
tarbase	hsa-miR-15b-5p	TMED2	10959	ENSG00000086598	validated
tarbase	hsa-miR-18a-5p	TMED2	10959	ENSG00000086598	validated
tarbase	hsa-miR-181a-5p	ERO1B	56605	ENSG00000086619	validated
tarbase	hsa-miR-15b-5p	ZFAND6	54469	ENSG00000086666	validated
tarbase	hsa-miR-222-3p	ZFAND6	54469	ENSG00000086666	validated
tarbase	hsa-miR-222-3p	TXLNG	55787	ENSG00000086712	validated
tarbase	hsa-miR-15b-5p	HUWE1	10075	ENSG00000086758	validated
tarbase	hsa-miR-18a-5p	HUWE1	10075	ENSG00000086758	validated
tarbase	hsa-miR-192-5p	HUWE1	10075	ENSG00000086758	validated
tarbase	hsa-miR-212-3p	HUWE1	10075	ENSG00000086758	validated
tarbase	hsa-miR-21-5p	HUWE1	10075	ENSG00000086758	validated
tarbase	hsa-miR-222-3p	HUWE1	10075	ENSG00000086758	validated
tarbase	hsa-miR-301a-3p	HUWE1	10075	ENSG00000086758	validated
tarbase	hsa-miR-15b-5p	ZW10	9183	ENSG00000086827	validated
tarbase	hsa-miR-106b-5p	ALG9	79796	ENSG00000086848	validated
tarbase	hsa-miR-15b-5p	ACOX3	8310	ENSG00000087008	validated
tarbase	hsa-miR-181a-5p	ACOX3	8310	ENSG00000087008	validated
tarbase	hsa-miR-15b-5p	MTMR2	8898	ENSG00000087053	validated
tarbase	hsa-miR-222-3p	PPP1R15A	23645	ENSG00000087074	validated
tarbase	hsa-miR-212-3p	PPP1R15A	23645	ENSG00000087074	validated
tarbase	hsa-miR-15b-5p	SRRT	51593	ENSG00000087087	validated
tarbase	hsa-miR-192-5p	SRRT	51593	ENSG00000087087	validated
tarbase	hsa-miR-106b-5p	NLK	51701	ENSG00000087095	validated
tarbase	hsa-miR-15b-5p	PIGS	94005	ENSG00000087111	validated
tarbase	hsa-miR-301a-3p	PIGS	94005	ENSG00000087111	validated
tarbase	hsa-miR-181a-5p	ADAMTS2	9509	ENSG00000087116	validated
tarbase	hsa-miR-106b-5p	ATXN7L3	56970	ENSG00000087152	validated
tarbase	hsa-miR-15b-5p	ATXN7L3	56970	ENSG00000087152	validated
tarbase	hsa-miR-18a-5p	ATXN7L3	56970	ENSG00000087152	validated
tarbase	hsa-miR-212-3p	UIMC1	51720	ENSG00000087206	validated
tarbase	hsa-miR-296-5p	MMP2	4313	ENSG00000087245	validated
tarbase	hsa-miR-326	MMP2	4313	ENSG00000087245	validated
tarbase	hsa-miR-582-5p	GNAO1	2775	ENSG00000087258	validated
tarbase	hsa-miR-21-5p	OGFOD1	55239	ENSG00000087263	validated
tarbase	hsa-miR-15b-5p	SH3BP2	6452	ENSG00000087266	validated
tarbase	hsa-miR-21-5p	NOP14	8602	ENSG00000087269	validated
tarbase	hsa-miR-18a-5p	ADD1	118	ENSG00000087274	validated
tarbase	hsa-miR-144-3p	TXNDC16	57544	ENSG00000087301	validated
tarbase	hsa-miR-18a-5p	RTRAF	51637	ENSG00000087302	validated
tarbase	hsa-miR-21-5p	NID2	22795	ENSG00000087303	validated
tarbase	hsa-miR-21-5p	SF3B2	10992	ENSG00000087365	validated
tarbase	hsa-miR-505-3p	KLHL42	57542	ENSG00000087448	validated
tarbase	hsa-miR-18a-5p	KLHL42	57542	ENSG00000087448	validated
tarbase	hsa-miR-192-5p	GNAS	2778	ENSG00000087460	validated
tarbase	hsa-miR-15b-5p	DNM1L	10059	ENSG00000087470	validated
tarbase	hsa-miR-301a-3p	DNM1L	10059	ENSG00000087470	validated
tarbase	hsa-miR-181a-5p	DNM1L	10059	ENSG00000087470	validated
tarbase	hsa-miR-181a-5p	ERGIC2	51290	ENSG00000087502	validated
tarbase	hsa-miR-582-3p	AURKA	6790	ENSG00000087586	validated
tarbase	hsa-miR-212-3p	AURKA	6790	ENSG00000087586	validated
tarbase	hsa-miR-181a-5p	METTL2A	339175	ENSG00000087995	validated
tarbase	hsa-miR-301a-3p	METTL2A	339175	ENSG00000087995	validated
tarbase	hsa-miR-582-5p	METTL2A	339175	ENSG00000087995	validated
tarbase	hsa-miR-505-3p	METTL2A	339175	ENSG00000087995	validated
tarbase	hsa-miR-296-5p	CNOT3	4849	ENSG00000088038	validated
tarbase	hsa-miR-15b-5p	PTPN4	5775	ENSG00000088179	validated
tarbase	hsa-miR-181a-5p	PTPN4	5775	ENSG00000088179	validated
tarbase	hsa-miR-21-5p	PTPN4	5775	ENSG00000088179	validated
tarbase	hsa-miR-326	DDX18	8886	ENSG00000088205	validated
tarbase	hsa-miR-106b-5p	KHSRP	8570	ENSG00000088247	validated
tarbase	hsa-miR-181a-5p	KHSRP	8570	ENSG00000088247	validated
tarbase	hsa-miR-296-5p	KHSRP	8570	ENSG00000088247	validated
tarbase	hsa-miR-21-5p	TPX2	22974	ENSG00000088325	validated
tarbase	hsa-miR-301a-3p	TPX2	22974	ENSG00000088325	validated
tarbase	hsa-miR-106b-5p	TPX2	22974	ENSG00000088325	validated
tarbase	hsa-miR-21-5p	DOCK9	23348	ENSG00000088387	validated
tarbase	hsa-miR-326	DOCK9	23348	ENSG00000088387	validated
tarbase	hsa-miR-181a-5p	ANKRD10	55608	ENSG00000088448	validated
tarbase	hsa-miR-21-5p	DOCK3	1795	ENSG00000088538	validated
tarbase	hsa-miR-296-5p	CRLS1	54675	ENSG00000088766	validated

tarbase	hsa-miR-301a-3p	CRLS1	54675	ENSG00000088766	validated
tarbase	hsa-miR-181a-5p	ATRN	8455	ENSG00000088812	validated
tarbase	hsa-miR-15b-5p	MAVS	57506	ENSG00000088888	validated
tarbase	hsa-miR-21-5p	MAVS	57506	ENSG00000088888	validated
tarbase	hsa-miR-326	RPL6	6128	ENSG00000089009	validated
tarbase	hsa-miR-222-3p	ESF1	51575	ENSG00000089048	validated
tarbase	hsa-miR-301a-3p	ESF1	51575	ENSG00000089048	validated
tarbase	hsa-miR-21-5p	ANAPC5	51433	ENSG00000089053	validated
tarbase	hsa-miR-326	SLC23A2	9962	ENSG00000089057	validated
tarbase	hsa-miR-505-3p	KDM2B	84678	ENSG00000089094	validated
tarbase	hsa-miR-212-3p	OAS1	4938	ENSG00000089127	validated
tarbase	hsa-miR-144-3p	GCN1	10985	ENSG00000089154	validated
tarbase	hsa-miR-21-5p	GCN1	10985	ENSG00000089154	validated
tarbase	hsa-miR-326	GCN1	10985	ENSG00000089154	validated
tarbase	hsa-miR-181a-5p	RPLP0	6175	ENSG00000089157	validated
tarbase	hsa-miR-15b-5p	PXN	5829	ENSG00000089159	validated
tarbase	hsa-miR-212-3p	SIRT4	23409	ENSG00000089163	validated
tarbase	hsa-miR-15b-5p	KIF16B	55614	ENSG00000089177	validated
tarbase	hsa-miR-301a-3p	KIF16B	55614	ENSG00000089177	validated
tarbase	hsa-miR-212-3p	CHGB	1114	ENSG00000089199	validated
tarbase	hsa-miR-21-5p	PEBP1	5037	ENSG00000089220	validated
tarbase	hsa-miR-181a-5p	FUS	2521	ENSG00000089280	validated
tarbase	hsa-miR-21-5p	FUS	2521	ENSG00000089280	validated
tarbase	hsa-miR-326	FUS	2521	ENSG00000089280	validated
tarbase	hsa-miR-582-5p	FUS	2521	ENSG00000089280	validated
tarbase	hsa-miR-212-3p	FUS	2521	ENSG00000089280	validated
tarbase	hsa-miR-212-3p	FXYD5	53827	ENSG00000089327	validated
tarbase	hsa-miR-181a-5p	ZNF302	55900	ENSG00000089335	validated
tarbase	hsa-miR-296-5p	GRAMD1A	57655	ENSG00000089351	validated
tarbase	hsa-miR-18a-5p	KCNH4	23415	ENSG00000089558	validated
tarbase	hsa-miR-582-5p	GANAB	23193	ENSG00000089597	validated
tarbase	hsa-miR-15b-5p	BIRC5	332	ENSG00000089685	validated
tarbase	hsa-miR-181a-5p	BIRC5	332	ENSG00000089685	validated
tarbase	hsa-miR-21-5p	BIRC5	332	ENSG00000089685	validated
tarbase	hsa-miR-326	BIRC5	332	ENSG00000089685	validated
tarbase	hsa-miR-192-5p	DDX24	57062	ENSG00000089737	validated
tarbase	hsa-miR-181a-5p	NECAP1	25977	ENSG00000089818	validated
tarbase	hsa-miR-15b-5p	NECAP1	25977	ENSG00000089818	validated
tarbase	hsa-miR-18a-5p	RCOR1	23186	ENSG00000089902	validated
tarbase	hsa-miR-21-5p	RCOR1	23186	ENSG00000089902	validated
tarbase	hsa-miR-505-3p	RCOR1	23186	ENSG00000089902	validated
tarbase	hsa-miR-106b-5p	GPATCH2L	55668	ENSG00000089916	validated
tarbase	hsa-miR-212-3p	GPATCH2L	55668	ENSG00000089916	validated
tarbase	hsa-miR-326	GPATCH2L	55668	ENSG00000089916	validated
tarbase	hsa-miR-15b-5p	BLVRB	645	ENSG00000090013	validated
tarbase	hsa-miR-21-5p	BLVRB	645	ENSG00000090013	validated
tarbase	hsa-miR-106b-5p	PAPOLA	10914	ENSG00000090060	validated
tarbase	hsa-miR-21-5p	PAPOLA	10914	ENSG00000090060	validated
tarbase	hsa-miR-21-5p	RGS1	5996	ENSG00000090104	validated
tarbase	hsa-miR-106b-5p	STRN4	29888	ENSG00000090372	validated
tarbase	hsa-miR-296-5p	STRN4	29888	ENSG00000090372	validated
tarbase	hsa-miR-181a-5p	SPG21	51324	ENSG00000090487	validated
tarbase	hsa-miR-106b-5p	DNAJB11	51726	ENSG00000090520	validated
tarbase	hsa-miR-15b-5p	P3H2	55214	ENSG00000090530	validated
tarbase	hsa-miR-106b-5p	GOLGA3	2802	ENSG00000090615	validated
tarbase	hsa-miR-15b-5p	GOLGA3	2802	ENSG00000090615	validated
tarbase	hsa-miR-21-5p	GOLGA3	2802	ENSG00000090615	validated
tarbase	hsa-miR-106b-5p	PABPC4	8761	ENSG00000090621	validated
tarbase	hsa-miR-15b-5p	PABPC4	8761	ENSG00000090621	validated
tarbase	hsa-miR-582-3p	PABPC4	8761	ENSG00000090621	validated
tarbase	hsa-miR-106b-5p	PDPR	55066	ENSG00000090857	validated
tarbase	hsa-miR-18a-5p	PDPR	55066	ENSG00000090857	validated
tarbase	hsa-miR-582-5p	GLG1	2734	ENSG00000090863	validated
tarbase	hsa-miR-15b-5p	GLG1	2734	ENSG00000090863	validated
tarbase	hsa-miR-144-3p	TNRC6A	27327	ENSG00000090905	validated
tarbase	hsa-miR-15b-5p	TNRC6A	27327	ENSG00000090905	validated
tarbase	hsa-miR-181a-5p	TNRC6A	27327	ENSG00000090905	validated
tarbase	hsa-miR-18a-5p	TNRC6A	27327	ENSG00000090905	validated
tarbase	hsa-miR-212-3p	TNRC6A	27327	ENSG00000090905	validated
tarbase	hsa-miR-21-5p	TNRC6A	27327	ENSG00000090905	validated
tarbase	hsa-miR-582-3p	TNRC6A	27327	ENSG00000090905	validated

tarbase	hsa-miR-18a-5p	PLEKHG2	64857	ENSG00000090924	validated
tarbase	hsa-miR-15b-5p	PITPNM2	57605	ENSG00000090975	validated
tarbase	hsa-miR-106b-5p	EXOC1	55763	ENSG00000090989	validated
tarbase	hsa-miR-181a-5p	RBM27	54439	ENSG00000091009	validated
tarbase	hsa-miR-192-5p	RBM27	54439	ENSG00000091009	validated
tarbase	hsa-miR-21-5p	RBM27	54439	ENSG00000091009	validated
tarbase	hsa-miR-106b-5p	OSBPL8	114882	ENSG00000091039	validated
tarbase	hsa-miR-181a-5p	OSBPL8	114882	ENSG00000091039	validated
tarbase	hsa-miR-222-3p	OSBPL8	114882	ENSG00000091039	validated
tarbase	hsa-miR-21-5p	PUS7	54517	ENSG00000091127	validated
tarbase	hsa-miR-212-3p	NRCAM	4897	ENSG00000091129	validated
tarbase	hsa-miR-181a-5p	NRCAM	4897	ENSG00000091129	validated
tarbase	hsa-miR-21-5p	LAMB1	3912	ENSG00000091136	validated
tarbase	hsa-miR-15b-5p	DLD	1738	ENSG00000091140	validated
tarbase	hsa-miR-181a-5p	WDR7	23335	ENSG00000091157	validated
tarbase	hsa-miR-212-3p	TXNL1	9352	ENSG00000091164	validated
tarbase	hsa-miR-21-5p	TXNL1	9352	ENSG00000091164	validated
tarbase	hsa-miR-181a-5p	TXNL1	9352	ENSG00000091164	validated
tarbase	hsa-miR-21-5p	CMTM6	54918	ENSG00000091317	validated
tarbase	hsa-miR-106b-5p	CMTM6	54918	ENSG00000091317	validated
tarbase	hsa-miR-144-3p	ITGA6	3655	ENSG00000091409	validated
tarbase	hsa-miR-106b-5p	ITGA6	3655	ENSG00000091409	validated
tarbase	hsa-miR-181a-5p	RAPGEF4	11069	ENSG00000091428	validated
tarbase	hsa-miR-181a-5p	MAP3K20	51776	ENSG00000091436	validated
tarbase	hsa-miR-301a-3p	MAP3K20	51776	ENSG00000091436	validated
tarbase	hsa-miR-181a-5p	FH	2271	ENSG00000091483	validated
tarbase	hsa-miR-301a-3p	SEL1L3	23231	ENSG00000091490	validated
tarbase	hsa-miR-106b-5p	CDV3	55573	ENSG00000091527	validated
tarbase	hsa-miR-181a-5p	CDV3	55573	ENSG00000091527	validated
tarbase	hsa-miR-212-3p	CDV3	55573	ENSG00000091527	validated
tarbase	hsa-miR-222-3p	CDV3	55573	ENSG00000091527	validated
tarbase	hsa-miR-301a-3p	CDV3	55573	ENSG00000091527	validated
tarbase	hsa-miR-18a-5p	CDV3	55573	ENSG00000091527	validated
tarbase	hsa-miR-15b-5p	NLRP1	22861	ENSG00000091592	validated
tarbase	hsa-miR-181a-5p	ORC6	23594	ENSG00000091651	validated
tarbase	hsa-miR-301a-3p	ORC6	23594	ENSG00000091651	validated
tarbase	hsa-miR-106b-5p	ZFHX4	79776	ENSG00000091656	validated
tarbase	hsa-miR-181a-5p	ZFHX4	79776	ENSG00000091656	validated
tarbase	hsa-miR-15b-5p	ZC3HC1	51530	ENSG00000091732	validated
tarbase	hsa-miR-21-5p	ESR1	2099	ENSG00000091831	validated
tarbase	hsa-miR-181a-5p	ANGPT2	285	ENSG00000091879	validated
tarbase	hsa-miR-212-3p	PSME1	5720	ENSG00000092010	validated
tarbase	hsa-miR-582-5p	PPP2R3C	55012	ENSG00000092020	validated
tarbase	hsa-miR-582-5p	SLC7A8	23428	ENSG00000092068	validated
tarbase	hsa-miR-15b-5p	SCFD1	23256	ENSG00000092108	validated
tarbase	hsa-miR-18a-5p	SCFD1	23256	ENSG00000092108	validated
tarbase	hsa-miR-212-3p	G2E3	55632	ENSG00000092140	validated
tarbase	hsa-miR-222-3p	G2E3	55632	ENSG00000092140	validated
tarbase	hsa-miR-15b-5p	HECTD1	25831	ENSG00000092148	validated
tarbase	hsa-miR-181a-5p	HECTD1	25831	ENSG00000092148	validated
tarbase	hsa-miR-212-3p	HECTD1	25831	ENSG00000092148	validated
tarbase	hsa-miR-222-3p	HECTD1	25831	ENSG00000092148	validated
tarbase	hsa-miR-301a-3p	HECTD1	25831	ENSG00000092148	validated
tarbase	hsa-miR-106b-5p	SUPT16H	11198	ENSG00000092201	validated
tarbase	hsa-miR-18a-5p	SUPT16H	11198	ENSG00000092201	validated
tarbase	hsa-miR-222-3p	SUPT16H	11198	ENSG00000092201	validated
tarbase	hsa-miR-301a-3p	SUPT16H	11198	ENSG00000092201	validated
tarbase	hsa-miR-15b-5p	TOX4	9878	ENSG00000092203	validated
tarbase	hsa-miR-181a-5p	TRPM7	54822	ENSG00000092439	validated
tarbase	hsa-miR-192-5p	TRPM7	54822	ENSG00000092439	validated
tarbase	hsa-miR-106b-5p	SNAP23	8773	ENSG00000092531	validated
tarbase	hsa-miR-222-3p	SNAP23	8773	ENSG00000092531	validated
tarbase	hsa-miR-301a-3p	PHGDH	26227	ENSG00000092621	validated
tarbase	hsa-miR-21-5p	EZR	7430	ENSG00000092820	validated
tarbase	hsa-miR-582-3p	EZR	7430	ENSG00000092820	validated
tarbase	hsa-miR-296-5p	MYL6	4637	ENSG00000092841	validated
tarbase	hsa-miR-15b-5p	AGO1	26523	ENSG00000092847	validated
tarbase	hsa-miR-181a-5p	AGO1	26523	ENSG00000092847	validated
tarbase	hsa-miR-21-5p	AGO1	26523	ENSG00000092847	validated
tarbase	hsa-miR-301a-3p	AGO1	26523	ENSG00000092847	validated
tarbase	hsa-miR-582-3p	AGO1	26523	ENSG00000092847	validated

tarbase	hsa-miR-21-5p	CLSPN	63967	ENSG00000092853	validated
tarbase	hsa-miR-181a-5p	RFFL	117584	ENSG00000092871	validated
tarbase	hsa-miR-301a-3p	MFSD11	79157	ENSG00000092931	validated
tarbase	hsa-miR-21-5p	DPYSL2	1808	ENSG00000092964	validated
tarbase	hsa-miR-222-3p	DPYSL2	1808	ENSG00000092964	validated
tarbase	hsa-miR-181a-5p	DPYSL2	1808	ENSG00000092964	validated
tarbase	hsa-miR-106b-5p	GPATCH2	55105	ENSG00000092978	validated
tarbase	hsa-miR-15b-5p	GPATCH2	55105	ENSG00000092978	validated
tarbase	hsa-miR-21-5p	GPATCH2	55105	ENSG00000092978	validated
tarbase	hsa-miR-582-5p	GPATCH2	55105	ENSG00000092978	validated
tarbase	hsa-miR-181a-5p	NUP50	10762	ENSG00000093000	validated
tarbase	hsa-miR-18a-5p	NUP50	10762	ENSG00000093000	validated
tarbase	hsa-miR-106b-5p	CDC45	8318	ENSG00000093009	validated
tarbase	hsa-miR-15b-5p	COMT	1312	ENSG00000093010	validated
tarbase	hsa-miR-106b-5p	ECHDC1	55862	ENSG00000093144	validated
tarbase	hsa-miR-15b-5p	ECHDC1	55862	ENSG00000093144	validated
tarbase	hsa-miR-15b-5p	SEC22C	9117	ENSG00000093183	validated
tarbase	hsa-miR-18a-5p	SEC22C	9117	ENSG00000093183	validated
tarbase	hsa-miR-582-3p	SEC22C	9117	ENSG00000093183	validated
tarbase	hsa-miR-106b-5p	CDC6	990	ENSG00000094804	validated
tarbase	hsa-miR-144-3p	CBX5	23468	ENSG00000094916	validated
tarbase	hsa-miR-15b-5p	CBX5	23468	ENSG00000094916	validated
tarbase	hsa-miR-181a-5p	CBX5	23468	ENSG00000094916	validated
tarbase	hsa-miR-21-5p	CBX5	23468	ENSG00000094916	validated
tarbase	hsa-miR-582-5p	CBX5	23468	ENSG00000094916	validated
tarbase	hsa-miR-15b-5p	SUCO	51430	ENSG00000094975	validated
tarbase	hsa-miR-181a-5p	SUCO	51430	ENSG00000094975	validated
tarbase	hsa-miR-582-5p	SUCO	51430	ENSG00000094975	validated
tarbase	hsa-miR-181a-5p	MSH2	4436	ENSG00000095002	validated
tarbase	hsa-miR-212-3p	MSH2	4436	ENSG00000095002	validated
tarbase	hsa-miR-18a-5p	MAP3K1	4214	ENSG00000095015	validated
tarbase	hsa-miR-582-3p	MAP3K1	4214	ENSG00000095015	validated
tarbase	hsa-miR-181a-5p	ARCN1	372	ENSG00000095139	validated
tarbase	hsa-miR-21-5p	ARCN1	372	ENSG00000095139	validated
tarbase	hsa-miR-582-5p	ARCN1	372	ENSG00000095139	validated
tarbase	hsa-miR-15b-5p	PSMD5	5711	ENSG00000095261	validated
tarbase	hsa-miR-181a-5p	NUP188	23511	ENSG00000095319	validated
tarbase	hsa-miR-326	CRAT	1384	ENSG00000095321	validated
tarbase	hsa-miR-181a-5p	NANS	54187	ENSG00000095380	validated
tarbase	hsa-miR-301a-3p	TBC1D2	55357	ENSG00000095383	validated
tarbase	hsa-miR-106b-5p	SEMA4G	57715	ENSG00000095539	validated
tarbase	hsa-miR-144-3p	BTAF1	9044	ENSG00000095564	validated
tarbase	hsa-miR-582-3p	BTAF1	9044	ENSG00000095564	validated
tarbase	hsa-miR-106b-5p	IKZF5	64376	ENSG00000095574	validated
tarbase	hsa-miR-18a-5p	IKZF5	64376	ENSG00000095574	validated
tarbase	hsa-miR-106b-5p	BLNK	29760	ENSG00000095585	validated
tarbase	hsa-miR-21-5p	TDRD1	56165	ENSG00000095627	validated
tarbase	hsa-miR-21-5p	BAMBI	25805	ENSG00000095739	validated
tarbase	hsa-miR-301a-3p	BAMBI	25805	ENSG00000095739	validated
tarbase	hsa-miR-181a-5p	WAC	51322	ENSG00000095787	validated
tarbase	hsa-miR-301a-3p	WAC	51322	ENSG00000095787	validated
tarbase	hsa-miR-18a-5p	HIVEP1	3096	ENSG00000095951	validated
tarbase	hsa-miR-192-5p	HIVEP1	3096	ENSG00000095951	validated
tarbase	hsa-miR-106b-5p	FKBP5	2289	ENSG00000096060	validated
tarbase	hsa-miR-106b-5p	SRPK1	6732	ENSG00000096063	validated
tarbase	hsa-miR-21-5p	SRPK1	6732	ENSG00000096063	validated
tarbase	hsa-miR-301a-3p	SRPK1	6732	ENSG00000096063	validated
tarbase	hsa-miR-18a-5p	BRPF3	27154	ENSG00000096070	validated
tarbase	hsa-miR-212-3p	BRPF3	27154	ENSG00000096070	validated
tarbase	hsa-miR-192-5p			ENSG00000096153	validated
tarbase	hsa-miR-21-5p	HSP90AB1	3326	ENSG00000096384	validated
tarbase	hsa-miR-106b-5p	CDC5L	988	ENSG00000096401	validated
tarbase	hsa-miR-181a-5p	CDC5L	988	ENSG00000096401	validated
tarbase	hsa-miR-301a-3p	CDC5L	988	ENSG00000096401	validated
tarbase	hsa-miR-212-3p	ZNF184	7738	ENSG00000096654	validated
tarbase	hsa-miR-106b-5p	DSP	1832	ENSG00000096696	validated
tarbase	hsa-miR-192-5p	DSP	1832	ENSG00000096696	validated
tarbase	hsa-miR-21-5p	DSP	1832	ENSG00000096696	validated
tarbase	hsa-miR-181a-5p	HNRNPH3	3189	ENSG00000096746	validated
tarbase	hsa-miR-301a-3p	ABL1	25	ENSG00000097007	validated
tarbase	hsa-miR-21-5p	ACOT7	11332	ENSG00000097021	validated

tarbase	hsa-miR-181a-5p	SH3GLB1	51100	ENSG00000097033	validated
tarbase	hsa-miR-15b-5p	CDC7	8317	ENSG00000097046	validated
tarbase	hsa-miR-212-3p	PCSK5	5125	ENSG00000099139	validated
tarbase	hsa-miR-21-5p	SCD	6319	ENSG00000099194	validated
tarbase	hsa-miR-222-3p	SCD	6319	ENSG00000099194	validated
tarbase	hsa-miR-582-5p	SCD	6319	ENSG00000099194	validated
tarbase	hsa-miR-15b-5p	ERMP1	79956	ENSG00000099219	validated
tarbase	hsa-miR-301a-3p	ERMP1	79956	ENSG00000099219	validated
tarbase	hsa-miR-106b-5p	RAB18	22931	ENSG00000099246	validated
tarbase	hsa-miR-15b-5p	RAB18	22931	ENSG00000099246	validated
tarbase	hsa-miR-18a-5p	RAB18	22931	ENSG00000099246	validated
tarbase	hsa-miR-181a-5p	RAB18	22931	ENSG00000099246	validated
tarbase	hsa-miR-222-3p	NRP1	8829	ENSG00000099250	validated
tarbase	hsa-miR-301a-3p	NRP1	8829	ENSG00000099250	validated
tarbase	hsa-miR-326	NRP1	8829	ENSG00000099250	validated
tarbase	hsa-miR-181a-5p	PSMD8	5714	ENSG00000099341	validated
tarbase	hsa-miR-15b-5p	FBXL19	54620	ENSG00000099364	validated
tarbase	hsa-miR-296-5p	FBXL19	54620	ENSG00000099364	validated
tarbase	hsa-miR-326	FBXL19	54620	ENSG00000099364	validated
tarbase	hsa-miR-296-5p	SETD1A	9739	ENSG00000099381	validated
tarbase	hsa-miR-582-3p	SETD1A	9739	ENSG00000099381	validated
tarbase	hsa-miR-18a-5p	MAGEB2	4113	ENSG00000099399	validated
tarbase	hsa-miR-15b-5p	CIRBP	1153	ENSG00000099622	validated
tarbase	hsa-miR-212-3p	HNRNPM	4670	ENSG00000099783	validated
tarbase	hsa-miR-296-5p	HNRNPM	4670	ENSG00000099783	validated
tarbase	hsa-miR-301a-3p	HNRNPM	4670	ENSG00000099783	validated
tarbase	hsa-miR-505-3p	HNRNPM	4670	ENSG00000099783	validated
tarbase	hsa-miR-296-5p	TIMM13	26517	ENSG00000099800	validated
tarbase	hsa-miR-192-5p	MTAP	4507	ENSG00000099810	validated
tarbase	hsa-miR-192-5p	CEP170B	283638	ENSG00000099814	validated
tarbase	hsa-miR-212-3p	CEP170B	283638	ENSG00000099814	validated
tarbase	hsa-miR-326	POLR2E	5434	ENSG00000099817	validated
tarbase	hsa-miR-301a-3p	GADD45B	4616	ENSG00000099860	validated
tarbase	hsa-miR-212-3p	MADCAM1	8174	ENSG00000099866	validated
tarbase	hsa-miR-144-3p	MKNK2	2872	ENSG00000099875	validated
tarbase	hsa-miR-181a-5p	MKNK2	2872	ENSG00000099875	validated
tarbase	hsa-miR-192-5p	MKNK2	2872	ENSG00000099875	validated
tarbase	hsa-miR-301a-3p	MED15	51586	ENSG00000099917	validated
tarbase	hsa-miR-21-5p	SNAP29	9342	ENSG00000099940	validated
tarbase	hsa-miR-181a-5p	CRKL	1399	ENSG00000099942	validated
tarbase	hsa-miR-212-3p	CRKL	1399	ENSG00000099942	validated
tarbase	hsa-miR-21-5p	CRKL	1399	ENSG00000099942	validated
tarbase	hsa-miR-505-3p	CABIN1	23523	ENSG00000099991	validated
tarbase	hsa-miR-212-3p	SEC14L2	23541	ENSG00000100003	validated
tarbase	hsa-miR-106b-5p	SPECC1L	23384	ENSG00000100014	validated
tarbase	hsa-miR-181a-5p	SPECC1L	23384	ENSG00000100014	validated
tarbase	hsa-miR-106b-5p	PPIL2	23759	ENSG00000100023	validated
tarbase	hsa-miR-326	PPIL2	23759	ENSG00000100023	validated
tarbase	hsa-miR-106b-5p	YPEL1	29799	ENSG00000100027	validated
tarbase	hsa-miR-106b-5p	SNRPD3	6634	ENSG00000100028	validated
tarbase	hsa-miR-15b-5p	SNRPD3	6634	ENSG00000100028	validated
tarbase	hsa-miR-18a-5p	PES1	23481	ENSG00000100029	validated
tarbase	hsa-miR-582-3p	PES1	23481	ENSG00000100029	validated
tarbase	hsa-miR-212-3p	MAPK1	5594	ENSG00000100030	validated
tarbase	hsa-miR-21-5p	MAPK1	5594	ENSG00000100030	validated
tarbase	hsa-miR-582-5p	MAPK1	5594	ENSG00000100030	validated
tarbase	hsa-miR-21-5p	PPM1F	9647	ENSG00000100034	validated
tarbase	hsa-miR-326	PPM1F	9647	ENSG00000100034	validated
tarbase	hsa-miR-106b-5p	ESS2	8220	ENSG00000100056	validated
tarbase	hsa-miR-181a-5p	CARD10	29775	ENSG00000100065	validated
tarbase	hsa-miR-212-3p	LRP5L	91355	ENSG00000100068	validated
tarbase	hsa-miR-15b-5p	SLC25A1	6576	ENSG00000100075	validated
tarbase	hsa-miR-212-3p	HIRA	7290	ENSG00000100084	validated
tarbase	hsa-miR-181a-5p	PATZ1	23598	ENSG00000100105	validated
tarbase	hsa-miR-18a-5p	TRIOBP	11078	ENSG00000100106	validated
tarbase	hsa-miR-181a-5p	EIF3L	51386	ENSG00000100129	validated
tarbase	hsa-miR-21-5p	EIF3L	51386	ENSG00000100129	validated
tarbase	hsa-miR-301a-3p	EIF3L	51386	ENSG00000100129	validated
tarbase	hsa-miR-21-5p	SNU13	4809	ENSG00000100138	validated
tarbase	hsa-miR-106b-5p	POLR2F	5435	ENSG00000100142	validated
tarbase	hsa-miR-106b-5p	SEPTIN3	55964	ENSG00000100167	validated

tarbase	hsa-miR-15b-5p	SEPTIN3	55964	ENSG00000100167	validated
tarbase	hsa-miR-181a-5p	DDX17	10521	ENSG00000100201	validated
tarbase	hsa-miR-21-5p	DDX17	10521	ENSG00000100201	validated
tarbase	hsa-miR-301a-3p	DDX17	10521	ENSG00000100201	validated
tarbase	hsa-miR-505-3p	DDX17	10521	ENSG00000100201	validated
tarbase	hsa-miR-15b-5p	DDX17	10521	ENSG00000100201	validated
tarbase	hsa-miR-15b-5p	TCF20	6942	ENSG00000100207	validated
tarbase	hsa-miR-106b-5p	CBY1	25776	ENSG00000100211	validated
tarbase	hsa-miR-326	CBY1	25776	ENSG00000100211	validated
tarbase	hsa-miR-192-5p	TOMM22	56993	ENSG00000100216	validated
tarbase	hsa-miR-222-3p	XBP1	7494	ENSG00000100219	validated
tarbase	hsa-miR-326	XBP1	7494	ENSG00000100219	validated
tarbase	hsa-miR-15b-5p	RTCB	51493	ENSG00000100220	validated
tarbase	hsa-miR-106b-5p	JOSD1	9929	ENSG00000100221	validated
tarbase	hsa-miR-192-5p	GTPBP1	9567	ENSG00000100226	validated
tarbase	hsa-miR-192-5p	TIMP3	7078	ENSG00000100234	validated
tarbase	hsa-miR-15b-5p	TIMP3	7078	ENSG00000100234	validated
tarbase	hsa-miR-181a-5p	TIMP3	7078	ENSG00000100234	validated
tarbase	hsa-miR-296-5p	SBF1	6305	ENSG00000100241	validated
tarbase	hsa-miR-212-3p	DNAL4	10126	ENSG00000100246	validated
tarbase	hsa-miR-15b-5p	LMF2	91289	ENSG00000100258	validated
tarbase	hsa-miR-15b-5p	RHBDD3	25807	ENSG00000100263	validated
tarbase	hsa-miR-106b-5p	HMGXB4	10042	ENSG00000100281	validated
tarbase	hsa-miR-212-3p	HMGXB4	10042	ENSG00000100281	validated
tarbase	hsa-miR-582-5p	HMGXB4	10042	ENSG00000100281	validated
tarbase	hsa-miR-15b-5p	THOC5	8563	ENSG00000100296	validated
tarbase	hsa-miR-21-5p	TTLL12	23170	ENSG00000100304	validated
tarbase	hsa-miR-106b-5p	CBX7	23492	ENSG00000100307	validated
tarbase	hsa-miR-181a-5p	CBX7	23492	ENSG00000100307	validated
tarbase	hsa-miR-15b-5p	RBFOX2	23543	ENSG00000100320	validated
tarbase	hsa-miR-222-3p	RBFOX2	23543	ENSG00000100320	validated
tarbase	hsa-miR-106b-5p	MIEF1	54471	ENSG00000100335	validated
tarbase	hsa-miR-212-3p	PNPLA5	150379	ENSG00000100341	validated
tarbase	hsa-miR-21-5p	PNPLA3	80339	ENSG00000100344	validated
tarbase	hsa-miR-21-5p	MYH9	4627	ENSG00000100345	validated
tarbase	hsa-miR-212-3p	MYH9	4627	ENSG00000100345	validated
tarbase	hsa-miR-192-5p	TXN2	25828	ENSG00000100348	validated
tarbase	hsa-miR-144-3p	TNRC6B	23112	ENSG00000100354	validated
tarbase	hsa-miR-192-5p	TNRC6B	23112	ENSG00000100354	validated
tarbase	hsa-miR-296-5p	TNRC6B	23112	ENSG00000100354	validated
tarbase	hsa-miR-505-3p	TNRC6B	23112	ENSG00000100354	validated
tarbase	hsa-miR-582-5p	TNRC6B	23112	ENSG00000100354	validated
tarbase	hsa-miR-296-5p	SGSM3	27352	ENSG00000100359	validated
tarbase	hsa-miR-212-3p	CSF2RB	1439	ENSG00000100368	validated
tarbase	hsa-miR-106b-5p	FAM118A	55007	ENSG00000100376	validated
tarbase	hsa-miR-15b-5p	FAM118A	55007	ENSG00000100376	validated
tarbase	hsa-miR-582-5p	FAM118A	55007	ENSG00000100376	validated
tarbase	hsa-miR-181a-5p	KCTD17	79734	ENSG00000100379	validated
tarbase	hsa-miR-181a-5p	IL2RB	3560	ENSG00000100385	validated
tarbase	hsa-miR-15b-5p	RBX1	9978	ENSG00000100387	validated
tarbase	hsa-miR-106b-5p	EP300	2033	ENSG00000100393	validated
tarbase	hsa-miR-144-3p	EP300	2033	ENSG00000100393	validated
tarbase	hsa-miR-15b-5p	EP300	2033	ENSG00000100393	validated
tarbase	hsa-miR-181a-5p	EP300	2033	ENSG00000100393	validated
tarbase	hsa-miR-192-5p	EP300	2033	ENSG00000100393	validated
tarbase	hsa-miR-212-3p	EP300	2033	ENSG00000100393	validated
tarbase	hsa-miR-15b-5p	RANGAP1	5905	ENSG00000100401	validated
tarbase	hsa-miR-15b-5p	POLR3H	171568	ENSG00000100413	validated
tarbase	hsa-miR-15b-5p	DESI1	27351	ENSG00000100418	validated
tarbase	hsa-miR-18a-5p	CERK	64781	ENSG00000100422	validated
tarbase	hsa-miR-21-5p	CERK	64781	ENSG00000100422	validated
tarbase	hsa-miR-181a-5p	BRD1	23774	ENSG00000100425	validated
tarbase	hsa-miR-21-5p	BRD1	23774	ENSG00000100425	validated
tarbase	hsa-miR-326	BRD1	23774	ENSG00000100425	validated
tarbase	hsa-miR-582-5p	BRD1	23774	ENSG00000100425	validated
tarbase	hsa-miR-301a-3p	ZBED4	9889	ENSG00000100426	validated
tarbase	hsa-miR-582-3p	ABHD4	63874	ENSG00000100439	validated
tarbase	hsa-miR-15b-5p	KHNYN	23351	ENSG00000100441	validated
tarbase	hsa-miR-296-5p	KHNYN	23351	ENSG00000100441	validated
tarbase	hsa-miR-106b-5p	FKBP3	2287	ENSG00000100442	validated
tarbase	hsa-miR-18a-5p	RBM23	55147	ENSG00000100461	validated

tarbase	hsa-miR-106b-5p	PRMT5	10419	ENSG00000100462	validated
tarbase	hsa-miR-18a-5p	PRMT5	10419	ENSG00000100462	validated
tarbase	hsa-miR-212-3p	SOS2	6655	ENSG00000100485	validated
tarbase	hsa-miR-21-5p	SOS2	6655	ENSG00000100485	validated
tarbase	hsa-miR-15b-5p	NIN	51199	ENSG00000100503	validated
tarbase	hsa-miR-212-3p	PYGL	5836	ENSG00000100504	validated
tarbase	hsa-miR-181a-5p	GNPNAT1	64841	ENSG00000100522	validated
tarbase	hsa-miR-301a-3p	GNPNAT1	64841	ENSG00000100522	validated
tarbase	hsa-miR-582-5p	GNPNAT1	64841	ENSG00000100522	validated
tarbase	hsa-miR-181a-5p	DDHD1	80821	ENSG00000100523	validated
tarbase	hsa-miR-181a-5p	CDKN3	1033	ENSG00000100526	validated
tarbase	hsa-miR-212-3p	CDKN3	1033	ENSG00000100526	validated
tarbase	hsa-miR-15b-5p	CNIH1	10175	ENSG00000100528	validated
tarbase	hsa-miR-181a-5p	CNIH1	10175	ENSG00000100528	validated
tarbase	hsa-miR-301a-3p	CNIH1	10175	ENSG00000100528	validated
tarbase	hsa-miR-505-3p	CNIH1	10175	ENSG00000100528	validated
tarbase	hsa-miR-582-3p	CNIH1	10175	ENSG00000100528	validated
tarbase	hsa-miR-21-5p	ATP6V1D	51382	ENSG00000100554	validated
tarbase	hsa-miR-15b-5p	VTI1B	10490	ENSG00000100568	validated
tarbase	hsa-miR-301a-3p	VTI1B	10490	ENSG00000100568	validated
tarbase	hsa-miR-582-3p	VTI1B	10490	ENSG00000100568	validated
tarbase	hsa-miR-212-3p	TIMM9	26520	ENSG00000100575	validated
tarbase	hsa-miR-106b-5p	TMED8	283578	ENSG00000100580	validated
tarbase	hsa-miR-181a-5p	TMED8	283578	ENSG00000100580	validated
tarbase	hsa-miR-212-3p	TMED8	283578	ENSG00000100580	validated
tarbase	hsa-miR-15b-5p	TMED8	283578	ENSG00000100580	validated
tarbase	hsa-miR-212-3p	AHSA1	10598	ENSG00000100591	validated
tarbase	hsa-miR-106b-5p	DAAM1	23002	ENSG00000100592	validated
tarbase	hsa-miR-192-5p	DAAM1	23002	ENSG00000100592	validated
tarbase	hsa-miR-212-3p	DAAM1	23002	ENSG00000100592	validated
tarbase	hsa-miR-15b-5p	SPTLC2	9517	ENSG00000100596	validated
tarbase	hsa-miR-296-5p	SPTLC2	9517	ENSG00000100596	validated
tarbase	hsa-miR-15b-5p	CHGA	1113	ENSG00000100604	validated
tarbase	hsa-miR-582-5p	CHGA	1113	ENSG00000100604	validated
tarbase	hsa-miR-301a-3p	ITPK1	3705	ENSG00000100605	validated
tarbase	hsa-miR-181a-5p	DHRS7	51635	ENSG00000100612	validated
tarbase	hsa-miR-106b-5p	PPM1A	5494	ENSG00000100614	validated
tarbase	hsa-miR-181a-5p	PPM1A	5494	ENSG00000100614	validated
tarbase	hsa-miR-301a-3p	SIX4	51804	ENSG00000100625	validated
tarbase	hsa-miR-212-3p	ERH	2079	ENSG00000100632	validated
tarbase	hsa-miR-21-5p	SUSD6	9766	ENSG00000100647	validated
tarbase	hsa-miR-301a-3p	SUSD6	9766	ENSG00000100647	validated
tarbase	hsa-miR-326	SUSD6	9766	ENSG00000100647	validated
tarbase	hsa-miR-15b-5p	SUSD6	9766	ENSG00000100647	validated
tarbase	hsa-miR-505-3p	SRSF5	6430	ENSG00000100650	validated
tarbase	hsa-miR-181a-5p	SRSF5	6430	ENSG00000100650	validated
tarbase	hsa-miR-181a-5p	EIF5	1983	ENSG00000100664	validated
tarbase	hsa-miR-212-3p	EIF5	1983	ENSG00000100664	validated
tarbase	hsa-miR-301a-3p	EIF5	1983	ENSG00000100664	validated
tarbase	hsa-miR-212-3p	SERPINA4	5267	ENSG00000100665	validated
tarbase	hsa-miR-106b-5p	DICER1	23405	ENSG00000100697	validated
tarbase	hsa-miR-144-3p	DICER1	23405	ENSG00000100697	validated
tarbase	hsa-miR-181a-5p	DICER1	23405	ENSG00000100697	validated
tarbase	hsa-miR-212-3p	DICER1	23405	ENSG00000100697	validated
tarbase	hsa-miR-15b-5p	ZFYVE21	79038	ENSG00000100711	validated
tarbase	hsa-miR-181a-5p	TCL1A	8115	ENSG00000100721	validated
tarbase	hsa-miR-15b-5p	ZC3H14	79882	ENSG00000100722	validated
tarbase	hsa-miR-18a-5p	ZC3H14	79882	ENSG00000100722	validated
tarbase	hsa-miR-21-5p	ZC3H14	79882	ENSG00000100722	validated
tarbase	hsa-miR-106b-5p	PCNX1	22990	ENSG00000100731	validated
tarbase	hsa-miR-181a-5p	PCNX1	22990	ENSG00000100731	validated
tarbase	hsa-miR-301a-3p	PCNX1	22990	ENSG00000100731	validated
tarbase	hsa-miR-582-5p	PCNX1	22990	ENSG00000100731	validated
tarbase	hsa-miR-15b-5p	RPS6KA5	9252	ENSG00000100784	validated
tarbase	hsa-miR-15b-5p	PPP4R3A	55671	ENSG00000100796	validated
tarbase	hsa-miR-21-5p	PPP4R3A	55671	ENSG00000100796	validated
tarbase	hsa-miR-301a-3p	PPP4R3A	55671	ENSG00000100796	validated
tarbase	hsa-miR-21-5p	PSMB5	5693	ENSG00000100804	validated
tarbase	hsa-miR-106b-5p	YY1	7528	ENSG00000100811	validated
tarbase	hsa-miR-181a-5p	YY1	7528	ENSG00000100811	validated
tarbase	hsa-miR-18a-5p	YY1	7528	ENSG00000100811	validated

tarbase	hsa-miR-192-5p	YY1	7528	ENSG00000100811	validated
tarbase	hsa-miR-21-5p	YY1	7528	ENSG00000100811	validated
tarbase	hsa-miR-582-5p	YY1	7528	ENSG00000100811	validated
tarbase	hsa-miR-296-5p	ACIN1	22985	ENSG00000100813	validated
tarbase	hsa-miR-505-3p	TRIP11	9321	ENSG00000100815	validated
tarbase	hsa-miR-212-3p	TRIP11	9321	ENSG00000100815	validated
tarbase	hsa-miR-18a-5p	APEX1	328	ENSG00000100823	validated
tarbase	hsa-miR-192-5p	APEX1	328	ENSG00000100823	validated
tarbase	hsa-miR-15b-5p	PABPN1	8106	ENSG00000100836	validated
tarbase	hsa-miR-296-5p	PABPN1	8106	ENSG00000100836	validated
tarbase	hsa-miR-326	PABPN1	8106	ENSG00000100836	validated
tarbase	hsa-miR-582-3p	PABPN1	8106	ENSG00000100836	validated
tarbase	hsa-miR-106b-5p	ARHGAP5	394	ENSG00000100852	validated
tarbase	hsa-miR-18a-5p	ARHGAP5	394	ENSG00000100852	validated
tarbase	hsa-miR-212-3p	ARHGAP5	394	ENSG00000100852	validated
tarbase	hsa-miR-582-5p	ARHGAP5	394	ENSG00000100852	validated
tarbase	hsa-miR-15b-5p	ARHGAP5	394	ENSG00000100852	validated
tarbase	hsa-miR-106b-5p	CHD8	57680	ENSG00000100888	validated
tarbase	hsa-miR-296-5p	CHD8	57680	ENSG00000100888	validated
tarbase	hsa-miR-212-3p	PCK2	5106	ENSG00000100889	validated
tarbase	hsa-miR-15b-5p	PRORP	9692	ENSG00000100890	validated
tarbase	hsa-miR-212-3p	NFKBIA	4792	ENSG00000100906	validated
tarbase	hsa-miR-18a-5p	NFKBIA	4792	ENSG00000100906	validated
tarbase	hsa-miR-212-3p	EMC9	51016	ENSG00000100908	validated
tarbase	hsa-miR-21-5p	BRMS1L	84312	ENSG00000100916	validated
tarbase	hsa-miR-15b-5p	SEC23A	10484	ENSG00000100934	validated
tarbase	hsa-miR-181a-5p	SEC23A	10484	ENSG00000100934	validated
tarbase	hsa-miR-18a-5p	SEC23A	10484	ENSG00000100934	validated
tarbase	hsa-miR-222-3p	SEC23A	10484	ENSG00000100934	validated
tarbase	hsa-miR-15b-5p	PNN	5411	ENSG00000100941	validated
tarbase	hsa-miR-181a-5p	TRPC4AP	26133	ENSG00000100991	validated
tarbase	hsa-miR-212-3p	TRPC4AP	26133	ENSG00000100991	validated
tarbase	hsa-miR-18a-5p	GINS1	9837	ENSG00000101003	validated
tarbase	hsa-miR-106b-5p	GINS1	9837	ENSG00000101003	validated
tarbase	hsa-miR-106b-5p	ZMYND8	23613	ENSG00000101040	validated
tarbase	hsa-miR-181a-5p	ZMYND8	23613	ENSG00000101040	validated
tarbase	hsa-miR-15b-5p	IFT52	51098	ENSG00000101052	validated
tarbase	hsa-miR-181a-5p	IFT52	51098	ENSG00000101052	validated
tarbase	hsa-miR-582-5p	MYBL2	4605	ENSG00000101057	validated
tarbase	hsa-miR-192-5p	NFATC2	4773	ENSG00000101096	validated
tarbase	hsa-miR-326	RIMS4	140730	ENSG00000101098	validated
tarbase	hsa-miR-21-5p	PABPC1L	80336	ENSG00000101104	validated
tarbase	hsa-miR-192-5p	STK4	6789	ENSG00000101109	validated
tarbase	hsa-miR-212-3p	STK4	6789	ENSG00000101109	validated
tarbase	hsa-miR-301a-3p	STK4	6789	ENSG00000101109	validated
tarbase	hsa-miR-106b-5p	ADNP	23394	ENSG00000101126	validated
tarbase	hsa-miR-18a-5p	ADNP	23394	ENSG00000101126	validated
tarbase	hsa-miR-192-5p	ADNP	23394	ENSG00000101126	validated
tarbase	hsa-miR-301a-3p	ADNP	23394	ENSG00000101126	validated
tarbase	hsa-miR-582-3p	ADNP	23394	ENSG00000101126	validated
tarbase	hsa-miR-582-5p	ADNP	23394	ENSG00000101126	validated
tarbase	hsa-miR-192-5p	CSTF1	1477	ENSG00000101138	validated
tarbase	hsa-miR-582-3p	CSTF1	1477	ENSG00000101138	validated
tarbase	hsa-miR-326	DNAJC5	80331	ENSG00000101152	validated
tarbase	hsa-miR-106b-5p	CTSZ	1522	ENSG00000101160	validated
tarbase	hsa-miR-18a-5p	PRELID3B	51012	ENSG00000101166	validated
tarbase	hsa-miR-582-5p	MRGBP	55257	ENSG00000101189	validated
tarbase	hsa-miR-15b-5p	TCFL5	10732	ENSG00000101190	validated
tarbase	hsa-miR-106b-5p	DIDO1	11083	ENSG00000101191	validated
tarbase	hsa-miR-212-3p	DIDO1	11083	ENSG00000101191	validated
tarbase	hsa-miR-301a-3p	DIDO1	11083	ENSG00000101191	validated
tarbase	hsa-miR-21-5p	GID8	54994	ENSG00000101193	validated
tarbase	hsa-miR-212-3p	SPEF1	25876	ENSG00000101222	validated
tarbase	hsa-miR-212-3p	CDC25B	994	ENSG00000101224	validated
tarbase	hsa-miR-106b-5p	RNF24	11237	ENSG00000101236	validated
tarbase	hsa-miR-181a-5p	RNF24	11237	ENSG00000101236	validated
tarbase	hsa-miR-21-5p	RNF24	11237	ENSG00000101236	validated
tarbase	hsa-miR-222-3p	RNF24	11237	ENSG00000101236	validated
tarbase	hsa-miR-301a-3p	RNF24	11237	ENSG00000101236	validated
tarbase	hsa-miR-296-5p	ARFRP1	10139	ENSG00000101246	validated
tarbase	hsa-miR-326	ARFRP1	10139	ENSG00000101246	validated

tarbase	hsa-miR-106b-5p	TRIB3	57761	ENSG00000101255	validated
tarbase	hsa-miR-106b-5p	CSNK2A1	1457	ENSG00000101266	validated
tarbase	hsa-miR-15b-5p	CSNK2A1	1457	ENSG00000101266	validated
tarbase	hsa-miR-18a-5p	CSNK2A1	1457	ENSG00000101266	validated
tarbase	hsa-miR-21-5p	CSNK2A1	1457	ENSG00000101266	validated
tarbase	hsa-miR-222-3p	CSNK2A1	1457	ENSG00000101266	validated
tarbase	hsa-miR-326	CSNK2A1	1457	ENSG00000101266	validated
tarbase	hsa-miR-21-5p	CDS2	8760	ENSG00000101290	validated
tarbase	hsa-miR-222-3p	CDS2	8760	ENSG00000101290	validated
tarbase	hsa-miR-18a-5p	HM13	81502	ENSG00000101294	validated
tarbase	hsa-miR-296-5p	SNPH	9751	ENSG00000101298	validated
tarbase	hsa-miR-181a-5p	SEC23B	10483	ENSG00000101310	validated
tarbase	hsa-miR-18a-5p	SEC23B	10483	ENSG00000101310	validated
tarbase	hsa-miR-181a-5p	FERMT1	55612	ENSG00000101311	validated
tarbase	hsa-miR-212-3p	PLCB4	5332	ENSG00000101333	validated
tarbase	hsa-miR-18a-5p	MYL9	10398	ENSG00000101335	validated
tarbase	hsa-miR-106b-5p	TM9SF4	9777	ENSG00000101337	validated
tarbase	hsa-miR-212-3p	TM9SF4	9777	ENSG00000101337	validated
tarbase	hsa-miR-15b-5p	POFUT1	23509	ENSG00000101346	validated
tarbase	hsa-miR-18a-5p	POFUT1	23509	ENSG00000101346	validated
tarbase	hsa-miR-21-5p	POFUT1	23509	ENSG00000101346	validated
tarbase	hsa-miR-222-3p	POFUT1	23509	ENSG00000101346	validated
tarbase	hsa-miR-212-3p	SAMHD1	25939	ENSG00000101347	validated
tarbase	hsa-miR-181a-5p	PAK5	57144	ENSG00000101349	validated
tarbase	hsa-miR-192-5p	NOP56	10528	ENSG00000101361	validated
tarbase	hsa-miR-296-5p	NOP56	10528	ENSG00000101361	validated
tarbase	hsa-miR-582-3p	NOP56	10528	ENSG00000101361	validated
tarbase	hsa-miR-192-5p	IDH3B	3420	ENSG00000101365	validated
tarbase	hsa-miR-212-3p	MAPRE1	22919	ENSG00000101367	validated
tarbase	hsa-miR-21-5p	MAPRE1	22919	ENSG00000101367	validated
tarbase	hsa-miR-296-5p	SNTA1	6640	ENSG00000101400	validated
tarbase	hsa-miR-181a-5p	TTI1	9675	ENSG00000101407	validated
tarbase	hsa-miR-301a-3p	TTI1	9675	ENSG00000101407	validated
tarbase	hsa-miR-18a-5p	E2F1	1869	ENSG00000101412	validated
tarbase	hsa-miR-296-5p	E2F1	1869	ENSG00000101412	validated
tarbase	hsa-miR-301a-3p	E2F1	1869	ENSG00000101412	validated
tarbase	hsa-miR-326	E2F1	1869	ENSG00000101412	validated
tarbase	hsa-miR-301a-3p	RPRD1B	58490	ENSG00000101413	validated
tarbase	hsa-miR-296-5p	PXMP4	11264	ENSG00000101417	validated
tarbase	hsa-miR-222-3p	FAM83D	81610	ENSG00000101447	validated
tarbase	hsa-miR-301a-3p	ACOT8	10005	ENSG00000101473	validated
tarbase	hsa-miR-505-3p	APMAP	57136	ENSG00000101474	validated
tarbase	hsa-miR-181a-5p	ZNF516	9658	ENSG00000101493	validated
tarbase	hsa-miR-15b-5p	ADNP2	22850	ENSG00000101544	validated
tarbase	hsa-miR-181a-5p	ADNP2	22850	ENSG00000101544	validated
tarbase	hsa-miR-301a-3p	ADNP2	22850	ENSG00000101544	validated
tarbase	hsa-miR-582-5p	ADNP2	22850	ENSG00000101544	validated
tarbase	hsa-miR-15b-5p	USP14	9097	ENSG00000101557	validated
tarbase	hsa-miR-181a-5p	USP14	9097	ENSG00000101557	validated
tarbase	hsa-miR-21-5p	VAPA	9218	ENSG00000101558	validated
tarbase	hsa-miR-15b-5p	VAPA	9218	ENSG00000101558	validated
tarbase	hsa-miR-106b-5p	METTL4	64863	ENSG00000101574	validated
tarbase	hsa-miR-15b-5p	METTL4	64863	ENSG00000101574	validated
tarbase	hsa-miR-21-5p	SMCHD1	23347	ENSG00000101596	validated
tarbase	hsa-miR-582-5p	SMCHD1	23347	ENSG00000101596	validated
tarbase	hsa-miR-15b-5p	MYL12A	10627	ENSG00000101608	validated
tarbase	hsa-miR-21-5p	CEP192	55125	ENSG00000101639	validated
tarbase	hsa-miR-181a-5p	SMAD7	4092	ENSG00000101665	validated
tarbase	hsa-miR-18a-5p	SMAD7	4092	ENSG00000101665	validated
tarbase	hsa-miR-505-3p	LAMA1	284217	ENSG00000101680	validated
tarbase	hsa-miR-15b-5p	ANKRD12	23253	ENSG00000101745	validated
tarbase	hsa-miR-222-3p	ANKRD12	23253	ENSG00000101745	validated
tarbase	hsa-miR-326	ANKRD12	23253	ENSG00000101745	validated
tarbase	hsa-miR-582-3p	ANKRD12	23253	ENSG00000101745	validated
tarbase	hsa-miR-582-5p	ANKRD12	23253	ENSG00000101745	validated
tarbase	hsa-miR-18a-5p	POLI	11201	ENSG00000101751	validated
tarbase	hsa-miR-181a-5p	MIB1	57534	ENSG00000101752	validated
tarbase	hsa-miR-301a-3p	RBBP8	5932	ENSG00000101773	validated
tarbase	hsa-miR-18a-5p	CSTF2	1478	ENSG00000101811	validated
tarbase	hsa-miR-301a-3p	CSTF2	1478	ENSG00000101811	validated
tarbase	hsa-miR-15b-5p	PGRMC1	10857	ENSG00000101856	validated

tarbase	hsa-miR-192-5p	PGRMC1	10857	ENSG00000101856	validated
tarbase	hsa-miR-21-5p	PGRMC1	10857	ENSG00000101856	validated
tarbase	hsa-miR-582-3p	PGRMC1	10857	ENSG00000101856	validated
tarbase	hsa-miR-106b-5p	MID1	4281	ENSG00000101871	validated
tarbase	hsa-miR-296-5p	MID1	4281	ENSG00000101871	validated
tarbase	hsa-miR-15b-5p	NKAP	79576	ENSG00000101882	validated
tarbase	hsa-miR-106b-5p	ALG13	79868	ENSG00000101901	validated
tarbase	hsa-miR-301a-3p	AMMECR1	9949	ENSG00000101935	validated
tarbase	hsa-miR-582-3p	AMMECR1	9949	ENSG00000101935	validated
tarbase	hsa-miR-15b-5p	XIAP	331	ENSG00000101966	validated
tarbase	hsa-miR-212-3p	STAG2	10735	ENSG00000101972	validated
tarbase	hsa-miR-582-3p	STAG2	10735	ENSG00000101972	validated
tarbase	hsa-miR-18a-5p	STAG2	10735	ENSG00000101972	validated
tarbase	hsa-miR-106b-5p	PLS3	5358	ENSG00000102024	validated
tarbase	hsa-miR-181a-5p	PLS3	5358	ENSG00000102024	validated
tarbase	hsa-miR-21-5p	PLS3	5358	ENSG00000102024	validated
tarbase	hsa-miR-582-5p	PLS3	5358	ENSG00000102024	validated
tarbase	hsa-miR-326	NAA10	8260	ENSG00000102030	validated
tarbase	hsa-miR-15b-5p	SMARCA1	6594	ENSG00000102038	validated
tarbase	hsa-miR-181a-5p	ZC3H12B	340554	ENSG00000102053	validated
tarbase	hsa-miR-144-3p	RBBP7	5931	ENSG00000102054	validated
tarbase	hsa-miR-181a-5p	RBBP7	5931	ENSG00000102054	validated
tarbase	hsa-miR-222-3p	RBBP7	5931	ENSG00000102054	validated
tarbase	hsa-miR-15b-5p	PIM2	11040	ENSG00000102096	validated
tarbase	hsa-miR-106b-5p	SCML2	10389	ENSG00000102098	validated
tarbase	hsa-miR-15b-5p	SCML2	10389	ENSG00000102098	validated
tarbase	hsa-miR-212-3p	SCML2	10389	ENSG00000102098	validated
tarbase	hsa-miR-15b-5p	EMD	2010	ENSG00000102119	validated
tarbase	hsa-miR-296-5p	EMD	2010	ENSG00000102119	validated
tarbase	hsa-miR-181a-5p	PGK1	5230	ENSG00000102144	validated
tarbase	hsa-miR-212-3p	PGK1	5230	ENSG00000102144	validated
tarbase	hsa-miR-21-5p	PGK1	5230	ENSG00000102144	validated
tarbase	hsa-miR-106b-5p	MAGT1	84061	ENSG00000102158	validated
tarbase	hsa-miR-181a-5p	MAGT1	84061	ENSG00000102158	validated
tarbase	hsa-miR-181a-5p	SMS	6611	ENSG00000102172	validated
tarbase	hsa-miR-106b-5p	RP2	6102	ENSG00000102218	validated
tarbase	hsa-miR-212-3p	RP2	6102	ENSG00000102218	validated
tarbase	hsa-miR-296-5p	CDK16	5127	ENSG00000102225	validated
tarbase	hsa-miR-15b-5p	USP11	8237	ENSG00000102226	validated
tarbase	hsa-miR-212-3p	TIMP1	7076	ENSG00000102265	validated
tarbase	hsa-miR-222-3p	PIN4	5303	ENSG00000102309	validated
tarbase	hsa-miR-106b-5p	MAGED2	10916	ENSG00000102316	validated
tarbase	hsa-miR-326	MAGED2	10916	ENSG00000102316	validated
tarbase	hsa-miR-18a-5p	RBM3	5935	ENSG00000102317	validated
tarbase	hsa-miR-106b-5p	SYTL4	94121	ENSG00000102362	validated
tarbase	hsa-miR-106b-5p	CENPI	2491	ENSG00000102384	validated
tarbase	hsa-miR-181a-5p	CENPI	2491	ENSG00000102384	validated
tarbase	hsa-miR-212-3p	ARMCX3	51566	ENSG00000102401	validated
tarbase	hsa-miR-181a-5p	ARMCX3	51566	ENSG00000102401	validated
tarbase	hsa-miR-181a-5p	BEX4	56271	ENSG00000102409	validated
tarbase	hsa-miR-181a-5p	HTR2A	3356	ENSG00000102468	validated
tarbase	hsa-miR-181a-5p	NDFIP2	54602	ENSG00000102471	validated
tarbase	hsa-miR-15b-5p	FNDC3A	22862	ENSG00000102531	validated
tarbase	hsa-miR-181a-5p	FNDC3A	22862	ENSG00000102531	validated
tarbase	hsa-miR-18a-5p	FNDC3A	22862	ENSG00000102531	validated
tarbase	hsa-miR-222-3p	FNDC3A	22862	ENSG00000102531	validated
tarbase	hsa-miR-326	FNDC3A	22862	ENSG00000102531	validated
tarbase	hsa-miR-582-3p	FNDC3A	22862	ENSG00000102531	validated
tarbase	hsa-miR-181a-5p	CDADC1	81602	ENSG00000102543	validated
tarbase	hsa-miR-15b-5p	KLF5	688	ENSG00000102554	validated
tarbase	hsa-miR-582-3p	KLF5	688	ENSG00000102554	validated
tarbase	hsa-miR-21-5p	ARHGEF7	8874	ENSG00000102606	validated
tarbase	hsa-miR-181a-5p	ARHGEF7	8874	ENSG00000102606	validated
tarbase	hsa-miR-15b-5p	FGF9	2254	ENSG00000102678	validated
tarbase	hsa-miR-212-3p	FGF9	2254	ENSG00000102678	validated
tarbase	hsa-miR-106b-5p	KPNA3	3839	ENSG00000102753	validated
tarbase	hsa-miR-18a-5p	KPNA3	3839	ENSG00000102753	validated
tarbase	hsa-miR-192-5p	KPNA3	3839	ENSG00000102753	validated
tarbase	hsa-miR-181a-5p	FLT1	2321	ENSG00000102755	validated
tarbase	hsa-miR-222-3p	FLT1	2321	ENSG00000102755	validated
tarbase	hsa-miR-106b-5p	DGKH	160851	ENSG00000102780	validated

tarbase	hsa-miR-301a-3p	DGKH	160851	ENSG00000102780	validated
tarbase	hsa-miR-582-3p	KATNAL1	84056	ENSG00000102781	validated
tarbase	hsa-miR-181a-5p	KATNAL1	84056	ENSG00000102781	validated
tarbase	hsa-miR-181a-5p	INTS6	26512	ENSG00000102786	validated
tarbase	hsa-miR-582-5p	INTS6	26512	ENSG00000102786	validated
tarbase	hsa-miR-326	ACOD1	730249	ENSG00000102794	validated
tarbase	hsa-miR-15b-5p	TSC22D1	8848	ENSG00000102804	validated
tarbase	hsa-miR-181a-5p	TSC22D1	8848	ENSG00000102804	validated
tarbase	hsa-miR-301a-3p	TSC22D1	8848	ENSG00000102804	validated
tarbase	hsa-miR-212-3p	MSLN	10232	ENSG00000102854	validated
tarbase	hsa-miR-192-5p	MGRN1	23295	ENSG00000102858	validated
tarbase	hsa-miR-181a-5p	ZNF629	23361	ENSG00000102870	validated
tarbase	hsa-miR-18a-5p	ZNF629	23361	ENSG00000102870	validated
tarbase	hsa-miR-222-3p	ZNF629	23361	ENSG00000102870	validated
tarbase	hsa-miR-326	ZNF629	23361	ENSG00000102870	validated
tarbase	hsa-miR-582-5p	ZNF629	23361	ENSG00000102870	validated
tarbase	hsa-miR-181a-5p	MAPK3	5595	ENSG00000102882	validated
tarbase	hsa-miR-212-3p	GDPD3	79153	ENSG00000102886	validated
tarbase	hsa-miR-181a-5p	LYRM1	57149	ENSG00000102897	validated
tarbase	hsa-miR-18a-5p	NUP93	9688	ENSG00000102900	validated
tarbase	hsa-miR-21-5p	NUP93	9688	ENSG00000102900	validated
tarbase	hsa-miR-222-3p	NUP93	9688	ENSG00000102900	validated
tarbase	hsa-miR-212-3p	TSNAXIP1	55815	ENSG00000102904	validated
tarbase	hsa-miR-212-3p	NFAT5	10725	ENSG00000102908	validated
tarbase	hsa-miR-582-3p	NFAT5	10725	ENSG00000102908	validated
tarbase	hsa-miR-181a-5p	NFAT5	10725	ENSG00000102908	validated
tarbase	hsa-miR-326	LONP2	83752	ENSG00000102910	validated
tarbase	hsa-miR-212-3p	N4BP1	9683	ENSG00000102921	validated
tarbase	hsa-miR-301a-3p	N4BP1	9683	ENSG00000102921	validated
tarbase	hsa-miR-212-3p	CBLN1	869	ENSG00000102924	validated
tarbase	hsa-miR-181a-5p	ARL2BP	23568	ENSG00000102931	validated
tarbase	hsa-miR-21-5p	ARL2BP	23568	ENSG00000102931	validated
tarbase	hsa-miR-212-3p	ZNF423	23090	ENSG00000102935	validated
tarbase	hsa-miR-181a-5p	CTCF	10664	ENSG00000102974	validated
tarbase	hsa-miR-222-3p	CTCF	10664	ENSG00000102974	validated
tarbase	hsa-miR-582-3p	CTCF	10664	ENSG00000102974	validated
tarbase	hsa-miR-106b-5p	ACD	65057	ENSG00000102977	validated
tarbase	hsa-miR-582-3p	CYB5B	80777	ENSG00000103018	validated
tarbase	hsa-miR-212-3p	CCDC113	29070	ENSG00000103021	validated
tarbase	hsa-miR-15b-5p	NME3	4832	ENSG00000103024	validated
tarbase	hsa-miR-144-3p	PSMD7	5713	ENSG00000103035	validated
tarbase	hsa-miR-15b-5p	SLC7A6	9057	ENSG00000103064	validated
tarbase	hsa-miR-21-5p	SLC7A6	9057	ENSG00000103064	validated
tarbase	hsa-miR-15b-5p	PLA2G15	23659	ENSG00000103066	validated
tarbase	hsa-miR-181a-5p	ESRP2	80004	ENSG00000103067	validated
tarbase	hsa-miR-301a-3p	ESRP2	80004	ENSG00000103067	validated
tarbase	hsa-miR-15b-5p	MON1B	22879	ENSG00000103111	validated
tarbase	hsa-miR-181a-5p	CMC2	56942	ENSG00000103121	validated
tarbase	hsa-miR-192-5p	AXIN1	8312	ENSG00000103126	validated
tarbase	hsa-miR-21-5p	AXIN1	8312	ENSG00000103126	validated
tarbase	hsa-miR-181a-5p	HSDL1	83693	ENSG00000103160	validated
tarbase	hsa-miR-15b-5p	NME4	4833	ENSG00000103202	validated
tarbase	hsa-miR-296-5p	NME4	4833	ENSG00000103202	validated
tarbase	hsa-miR-326	NME4	4833	ENSG00000103202	validated
tarbase	hsa-miR-582-3p	NME4	4833	ENSG00000103202	validated
tarbase	hsa-miR-582-5p	FOXF1	2294	ENSG00000103241	validated
tarbase	hsa-miR-326	CLCN7	1186	ENSG00000103249	validated
tarbase	hsa-miR-582-3p	SLC7A5	8140	ENSG00000103257	validated
tarbase	hsa-miR-106b-5p	SLC7A5	8140	ENSG00000103257	validated
tarbase	hsa-miR-212-3p	SLC7A5	8140	ENSG00000103257	validated
tarbase	hsa-miR-212-3p	METRN	79006	ENSG00000103260	validated
tarbase	hsa-miR-296-5p	STUB1	10273	ENSG00000103266	validated
tarbase	hsa-miR-192-5p	RHBDL1	9028	ENSG00000103269	validated
tarbase	hsa-miR-15b-5p	UBE2I	7329	ENSG00000103275	validated
tarbase	hsa-miR-181a-5p	UBE2I	7329	ENSG00000103275	validated
tarbase	hsa-miR-18a-5p	CAPN15	6650	ENSG00000103326	validated
tarbase	hsa-miR-296-5p	CAPN15	6650	ENSG00000103326	validated
tarbase	hsa-miR-582-5p	CAPN15	6650	ENSG00000103326	validated
tarbase	hsa-miR-301a-3p	PIEZO1	9780	ENSG00000103335	validated
tarbase	hsa-miR-582-3p	PIEZO1	9780	ENSG00000103335	validated
tarbase	hsa-miR-106b-5p	GSPT1	2935	ENSG00000103342	validated

tarbase	hsa-miR-181a-5p	GSPT1	2935	ENSG00000103342	validated
tarbase	hsa-miR-18a-5p	GSPT1	2935	ENSG00000103342	validated
tarbase	hsa-miR-21-5p	GSPT1	2935	ENSG00000103342	validated
tarbase	hsa-miR-15b-5p	UBFD1	56061	ENSG00000103353	validated
tarbase	hsa-miR-301a-3p	UBFD1	56061	ENSG00000103353	validated
tarbase	hsa-miR-106b-5p	GGA2	23062	ENSG00000103365	validated
tarbase	hsa-miR-15b-5p	GGA2	23062	ENSG00000103365	validated
tarbase	hsa-miR-301a-3p	GGA2	23062	ENSG00000103365	validated
tarbase	hsa-miR-106b-5p	USP31	57478	ENSG00000103404	validated
tarbase	hsa-miR-301a-3p	USP31	57478	ENSG00000103404	validated
tarbase	hsa-miR-582-5p	USP31	57478	ENSG00000103404	validated
tarbase	hsa-miR-15b-5p	DNAJA3	9093	ENSG00000103423	validated
tarbase	hsa-miR-15b-5p	BFAR	51283	ENSG00000103429	validated
tarbase	hsa-miR-106b-5p	SALL1	6299	ENSG00000103449	validated
tarbase	hsa-miR-15b-5p	RBL2	5934	ENSG00000103479	validated
tarbase	hsa-miR-181a-5p	RBL2	5934	ENSG00000103479	validated
tarbase	hsa-miR-582-5p	RBL2	5934	ENSG00000103479	validated
tarbase	hsa-miR-212-3p	RPGRIP1L	23322	ENSG00000103494	validated
tarbase	hsa-miR-15b-5p	MAZ	4150	ENSG00000103495	validated
tarbase	hsa-miR-296-5p	MAZ	4150	ENSG00000103495	validated
tarbase	hsa-miR-15b-5p	CDIPT	10423	ENSG00000103502	validated
tarbase	hsa-miR-181a-5p	CDIPT	10423	ENSG00000103502	validated
tarbase	hsa-miR-181a-5p	TMC5	79838	ENSG00000103534	validated
tarbase	hsa-miR-181a-5p	CCP110	9738	ENSG00000103540	validated
tarbase	hsa-miR-326	RNF40	9810	ENSG00000103549	validated
tarbase	hsa-miR-212-3p	IQCH	64799	ENSG00000103599	validated
tarbase	hsa-miR-301a-3p	CSK	1445	ENSG00000103653	validated
tarbase	hsa-miR-15b-5p	HERC1	8925	ENSG00000103657	validated
tarbase	hsa-miR-15b-5p	RAB11A	8766	ENSG00000103769	validated
tarbase	hsa-miR-21-5p	RAB11A	8766	ENSG00000103769	validated
tarbase	hsa-miR-326	CD276	80381	ENSG00000103855	validated
tarbase	hsa-miR-181a-5p	CEMIP	57214	ENSG00000103888	validated
tarbase	hsa-miR-15b-5p	TMEM87A	25963	ENSG00000103978	validated
tarbase	hsa-miR-212-3p	TMEM87A	25963	ENSG00000103978	validated
tarbase	hsa-miR-106b-5p	ZNF106	64397	ENSG00000103994	validated
tarbase	hsa-miR-181a-5p	ZNF106	64397	ENSG00000103994	validated
tarbase	hsa-miR-212-3p	ZNF106	64397	ENSG00000103994	validated
tarbase	hsa-miR-582-5p	FAM189A1	23359	ENSG00000104059	validated
tarbase	hsa-miR-181a-5p	TJP1	7082	ENSG00000104067	validated
tarbase	hsa-miR-106b-5p	TJP1	7082	ENSG00000104067	validated
tarbase	hsa-miR-296-5p	BMF	90427	ENSG00000104081	validated
tarbase	hsa-miR-582-3p	BMF	90427	ENSG00000104081	validated
tarbase	hsa-miR-15b-5p	DMXL2	23312	ENSG00000104093	validated
tarbase	hsa-miR-181a-5p	DMXL2	23312	ENSG00000104093	validated
tarbase	hsa-miR-212-3p	SCG3	29106	ENSG00000104112	validated
tarbase	hsa-miR-21-5p	EIF3J	8669	ENSG00000104131	validated
tarbase	hsa-miR-212-3p	RHOV	171177	ENSG00000104140	validated
tarbase	hsa-miR-582-5p	OIP5	11339	ENSG00000104147	validated
tarbase	hsa-miR-181a-5p	BLOC1S6	26258	ENSG00000104164	validated
tarbase	hsa-miR-21-5p	BLOC1S6	26258	ENSG00000104164	validated
tarbase	hsa-miR-18a-5p	BLOC1S6	26258	ENSG00000104164	validated
tarbase	hsa-miR-222-3p	TRIM35	23087	ENSG00000104228	validated
tarbase	hsa-miR-181a-5p	ZFAND1	79752	ENSG00000104231	validated
tarbase	hsa-miR-106b-5p			ENSG00000104237	validated
tarbase	hsa-miR-505-3p	CA2	760	ENSG00000104267	validated
tarbase	hsa-miR-582-5p	CA2	760	ENSG00000104267	validated
tarbase	hsa-miR-106b-5p	FZD3	7976	ENSG00000104290	validated
tarbase	hsa-miR-301a-3p	FZD3	7976	ENSG00000104290	validated
tarbase	hsa-miR-582-5p	FZD3	7976	ENSG00000104290	validated
tarbase	hsa-miR-212-3p	EYA1	2138	ENSG00000104313	validated
tarbase	hsa-miR-181a-5p	NBN	4683	ENSG00000104320	validated
tarbase	hsa-miR-181a-5p	IMPAD1	54928	ENSG00000104331	validated
tarbase	hsa-miR-212-3p	LAPTM4B	55353	ENSG00000104341	validated
tarbase	hsa-miR-106b-5p	UBE2W	55284	ENSG00000104343	validated
tarbase	hsa-miR-181a-5p	UBE2W	55284	ENSG00000104343	validated
tarbase	hsa-miR-21-5p	UBE2W	55284	ENSG00000104343	validated
tarbase	hsa-miR-301a-3p	UBE2W	55284	ENSG00000104343	validated
tarbase	hsa-miR-212-3p	NIPAL2	79815	ENSG00000104361	validated
tarbase	hsa-miR-15b-5p	IKBKB	3551	ENSG00000104365	validated
tarbase	hsa-miR-106b-5p	JPH1	56704	ENSG00000104369	validated
tarbase	hsa-miR-106b-5p	EIF3E	3646	ENSG00000104408	validated

tarbase	hsa-miR-222-3p	EIF3E	3646	ENSG00000104408	validated
tarbase	hsa-miR-15b-5p	EMC2	9694	ENSG00000104412	validated
tarbase	hsa-miR-212-3p	EMC2	9694	ENSG00000104412	validated
tarbase	hsa-miR-181a-5p	EMC2	9694	ENSG00000104412	validated
tarbase	hsa-miR-106b-5p	ESRP1	54845	ENSG00000104413	validated
tarbase	hsa-miR-18a-5p	ESRP1	54845	ENSG00000104413	validated
tarbase	hsa-miR-582-3p	ESRP1	54845	ENSG00000104413	validated
tarbase	hsa-miR-106b-5p	NDRG1	10397	ENSG00000104419	validated
tarbase	hsa-miR-212-3p	NDRG1	10397	ENSG00000104419	validated
tarbase	hsa-miR-212-3p	IL7	3574	ENSG00000104432	validated
tarbase	hsa-miR-106b-5p	ARMC1	55156	ENSG00000104442	validated
tarbase	hsa-miR-15b-5p	ARMC1	55156	ENSG00000104442	validated
tarbase	hsa-miR-106b-5p	TRPS1	7227	ENSG00000104447	validated
tarbase	hsa-miR-15b-5p	TRPS1	7227	ENSG00000104447	validated
tarbase	hsa-miR-18a-5p	TRPS1	7227	ENSG00000104447	validated
tarbase	hsa-miR-212-3p	TRPS1	7227	ENSG00000104447	validated
tarbase	hsa-miR-106b-5p	CHRAC1	54108	ENSG00000104472	validated
tarbase	hsa-miR-181a-5p	NCALD	83988	ENSG00000104490	validated
tarbase	hsa-miR-106b-5p	SNX16	64089	ENSG00000104497	validated
tarbase	hsa-miR-212-3p	SNX16	64089	ENSG00000104497	validated
tarbase	hsa-miR-181a-5p	UBR5	51366	ENSG00000104517	validated
tarbase	hsa-miR-301a-3p	UBR5	51366	ENSG00000104517	validated
tarbase	hsa-miR-181a-5p	GSDMD	79792	ENSG00000104518	validated
tarbase	hsa-miR-15b-5p	EEF1D	1936	ENSG00000104529	validated
tarbase	hsa-miR-212-3p	EEF1D	1936	ENSG00000104529	validated
tarbase	hsa-miR-222-3p	EEF1D	1936	ENSG00000104529	validated
tarbase	hsa-miR-296-5p	EEF1D	1936	ENSG00000104529	validated
tarbase	hsa-miR-301a-3p	EEF1D	1936	ENSG00000104529	validated
tarbase	hsa-miR-326	EEF1D	1936	ENSG00000104529	validated
tarbase	hsa-miR-582-3p	EEF1D	1936	ENSG00000104529	validated
tarbase	hsa-miR-15b-5p	SQLE	6713	ENSG00000104549	validated
tarbase	hsa-miR-18a-5p	ERI1	90459	ENSG00000104626	validated
tarbase	hsa-miR-106b-5p	SLC39A14	23516	ENSG00000104635	validated
tarbase	hsa-miR-18a-5p	SLC39A14	23516	ENSG00000104635	validated
tarbase	hsa-miR-21-5p	SLC39A14	23516	ENSG00000104635	validated
tarbase	hsa-miR-181a-5p	MTMR9	66036	ENSG00000104643	validated
tarbase	hsa-miR-301a-3p	MTMR9	66036	ENSG00000104643	validated
tarbase	hsa-miR-21-5p	LEPROTL1	23484	ENSG00000104660	validated
tarbase	hsa-miR-15b-5p	PPP2CB	5516	ENSG00000104695	validated
tarbase	hsa-miR-222-3p	PPP2CB	5516	ENSG00000104695	validated
tarbase	hsa-miR-15b-5p	ERICH1	157697	ENSG00000104714	validated
tarbase	hsa-miR-326	ERICH1	157697	ENSG00000104714	validated
tarbase	hsa-miR-15b-5p	TUSC3	7991	ENSG00000104723	validated
tarbase	hsa-miR-106b-5p	ARHGEF10	9639	ENSG00000104728	validated
tarbase	hsa-miR-106b-5p	MCM4	4173	ENSG00000104738	validated
tarbase	hsa-miR-21-5p	MCM4	4173	ENSG00000104738	validated
tarbase	hsa-miR-212-3p	FGL1	2267	ENSG00000104760	validated
tarbase	hsa-miR-181a-5p	BNIP3L	665	ENSG00000104765	validated
tarbase	hsa-miR-15b-5p	KCNN4	3783	ENSG00000104783	validated
tarbase	hsa-miR-15b-5p	NUCB1	4924	ENSG00000104805	validated
tarbase	hsa-miR-21-5p	NUCB1	4924	ENSG00000104805	validated
tarbase	hsa-miR-18a-5p	HNRNPL	3191	ENSG00000104824	validated
tarbase	hsa-miR-21-5p	SNRNP70	6625	ENSG00000104852	validated
tarbase	hsa-miR-106b-5p	CLPTM1	1209	ENSG00000104853	validated
tarbase	hsa-miR-296-5p	CLPTM1	1209	ENSG00000104853	validated
tarbase	hsa-miR-301a-3p	CLPTM1	1209	ENSG00000104853	validated
tarbase	hsa-miR-212-3p	RELB	5971	ENSG00000104856	validated
tarbase	hsa-miR-212-3p	LIN7B	64130	ENSG00000104863	validated
tarbase	hsa-miR-21-5p	PPP1R37	284352	ENSG00000104866	validated
tarbase	hsa-miR-15b-5p	ARHGEF18	23370	ENSG00000104880	validated
tarbase	hsa-miR-15b-5p	DOT1L	84444	ENSG00000104885	validated
tarbase	hsa-miR-181a-5p	PLEKHJ1	55111	ENSG00000104886	validated
tarbase	hsa-miR-18a-5p	PLEKHJ1	55111	ENSG00000104886	validated
tarbase	hsa-miR-15b-5p	SF3A2	8175	ENSG00000104897	validated
tarbase	hsa-miR-301a-3p	SF3A2	8175	ENSG00000104897	validated
tarbase	hsa-miR-296-5p	AMH	268	ENSG00000104899	validated
tarbase	hsa-miR-181a-5p	OAZ1	4946	ENSG00000104904	validated
tarbase	hsa-miR-296-5p	OAZ1	4946	ENSG00000104904	validated
tarbase	hsa-miR-181a-5p	TRMT1	55621	ENSG00000104907	validated
tarbase	hsa-miR-18a-5p	DMPK	1760	ENSG00000104936	validated
tarbase	hsa-miR-15b-5p	TBC1D17	79735	ENSG00000104946	validated

tarbase	hsa-miR-296-5p	PTOV1	53635	ENSG00000104960	validated
tarbase	hsa-miR-192-5p	TLE5	166	ENSG00000104964	validated
tarbase	hsa-miR-181a-5p	TLE5	166	ENSG00000104964	validated
tarbase	hsa-miR-15b-5p	ASF1B	55723	ENSG00000105011	validated
tarbase	hsa-miR-301a-3p	ASF1B	55723	ENSG00000105011	validated
tarbase	hsa-miR-106b-5p	ASF1B	55723	ENSG00000105011	validated
tarbase	hsa-miR-212-3p	TNNT1	7138	ENSG00000105048	validated
tarbase	hsa-miR-21-5p	VRK3	51231	ENSG00000105053	validated
tarbase	hsa-miR-181a-5p	PPP6R1	22870	ENSG00000105063	validated
tarbase	hsa-miR-181a-5p	MED26	9441	ENSG00000105085	validated
tarbase	hsa-miR-181a-5p	ZNF419	79744	ENSG00000105136	validated
tarbase	hsa-miR-144-3p	CASP14	23581	ENSG00000105141	validated
tarbase	hsa-miR-212-3p	AURKC	6795	ENSG00000105146	validated
tarbase	hsa-miR-181a-5p	CCNE1	898	ENSG00000105173	validated
tarbase	hsa-miR-106b-5p	URI1	8725	ENSG00000105176	validated
tarbase	hsa-miR-181a-5p	URI1	8725	ENSG00000105176	validated
tarbase	hsa-miR-18a-5p	URI1	8725	ENSG00000105176	validated
tarbase	hsa-miR-301a-3p	URI1	8725	ENSG00000105176	validated
tarbase	hsa-miR-582-5p	URI1	8725	ENSG00000105176	validated
tarbase	hsa-miR-192-5p	ANKRD27	84079	ENSG00000105186	validated
tarbase	hsa-miR-21-5p	ANKRD27	84079	ENSG00000105186	validated
tarbase	hsa-miR-181a-5p	FBL	2091	ENSG00000105202	validated
tarbase	hsa-miR-18a-5p	FBL	2091	ENSG00000105202	validated
tarbase	hsa-miR-15b-5p	DYRK1B	9149	ENSG00000105204	validated
tarbase	hsa-miR-296-5p	DYRK1B	9149	ENSG00000105204	validated
tarbase	hsa-miR-326	GPI	2821	ENSG00000105220	validated
tarbase	hsa-miR-326	PIAS4	51588	ENSG00000105229	validated
tarbase	hsa-miR-582-5p	FSD1	79187	ENSG00000105255	validated
tarbase	hsa-miR-18a-5p	CLIP3	25999	ENSG00000105270	validated
tarbase	hsa-miR-301a-3p	ZFR2	23217	ENSG00000105278	validated
tarbase	hsa-miR-192-5p	SLC1A5	6510	ENSG00000105281	validated
tarbase	hsa-miR-296-5p	SLC1A5	6510	ENSG00000105281	validated
tarbase	hsa-miR-15b-5p	APLP1	333	ENSG00000105290	validated
tarbase	hsa-miR-106b-5p	HNRNPUL1	11100	ENSG00000105323	validated
tarbase	hsa-miR-301a-3p	HNRNPUL1	11100	ENSG00000105323	validated
tarbase	hsa-miR-301a-3p	BBC3	27113	ENSG00000105327	validated
tarbase	hsa-miR-106b-5p	DMAC2	55101	ENSG00000105341	validated
tarbase	hsa-miR-296-5p	MYH14	79784	ENSG00000105357	validated
tarbase	hsa-miR-212-3p	CD33	945	ENSG00000105383	validated
tarbase	hsa-miR-18a-5p	BABAM1	29086	ENSG00000105393	validated
tarbase	hsa-miR-582-3p	BABAM1	29086	ENSG00000105393	validated
tarbase	hsa-miR-192-5p	NAPA	8775	ENSG00000105402	validated
tarbase	hsa-miR-212-3p	RABAC1	10567	ENSG00000105404	validated
tarbase	hsa-miR-106b-5p	GRWD1	83743	ENSG00000105447	validated
tarbase	hsa-miR-106b-5p	CARD8	22900	ENSG00000105483	validated
tarbase	hsa-miR-181a-5p	ZNF175	7728	ENSG00000105497	validated
tarbase	hsa-miR-212-3p	PLA2G4C	8605	ENSG00000105499	validated
tarbase	hsa-miR-192-5p	RAB3D	9545	ENSG00000105514	validated
tarbase	hsa-miR-18a-5p	TMEM205	374882	ENSG00000105518	validated
tarbase	hsa-miR-106b-5p	PPP2R1A	5518	ENSG00000105568	validated
tarbase	hsa-miR-15b-5p	PPP2R1A	5518	ENSG00000105568	validated
tarbase	hsa-miR-15b-5p	TNPO2	30000	ENSG00000105576	validated
tarbase	hsa-miR-18a-5p	TNPO2	30000	ENSG00000105576	validated
tarbase	hsa-miR-222-3p	TNPO2	30000	ENSG00000105576	validated
tarbase	hsa-miR-296-5p	TNPO2	30000	ENSG00000105576	validated
tarbase	hsa-miR-222-3p	JAK3	3718	ENSG00000105639	validated
tarbase	hsa-miR-296-5p	PIK3R2	5296	ENSG00000105647	validated
tarbase	hsa-miR-301a-3p	PIK3R2	5296	ENSG00000105647	validated
tarbase	hsa-miR-15b-5p	ISYNA1	51477	ENSG00000105655	validated
tarbase	hsa-miR-296-5p	ISYNA1	51477	ENSG00000105655	validated
tarbase	hsa-miR-15b-5p	ELL	8178	ENSG00000105656	validated
tarbase	hsa-miR-326	COPE	11316	ENSG00000105669	validated
tarbase	hsa-miR-212-3p	COPE	11316	ENSG00000105669	validated
tarbase	hsa-miR-212-3p	ETV2	2116	ENSG00000105672	validated
tarbase	hsa-miR-106b-5p	USF2	7392	ENSG00000105698	validated
tarbase	hsa-miR-15b-5p	LSR	51599	ENSG00000105699	validated
tarbase	hsa-miR-144-3p	FKBP8	23770	ENSG00000105701	validated
tarbase	hsa-miR-181a-5p	ZNF14	7561	ENSG00000105708	validated
tarbase	hsa-miR-21-5p	ERF	2077	ENSG00000105722	validated
tarbase	hsa-miR-192-5p	GSK3A	2931	ENSG00000105723	validated
tarbase	hsa-miR-15b-5p	SIPA1L3	23094	ENSG00000105738	validated

tarbase	hsa-miR-18a-5p	ZNF85	7639	ENSG00000105750	validated
tarbase	hsa-miR-21-5p	SMG9	56006	ENSG00000105771	validated
tarbase	hsa-miR-181a-5p	AVL9	23080	ENSG00000105778	validated
tarbase	hsa-miR-212-3p	AVL9	23080	ENSG00000105778	validated
tarbase	hsa-miR-21-5p	AVL9	23080	ENSG00000105778	validated
tarbase	hsa-miR-212-3p	RUNDC3B	154661	ENSG00000105784	validated
tarbase	hsa-miR-582-3p	GTPBP10	85865	ENSG00000105793	validated
tarbase	hsa-miR-212-3p	CDK6	1021	ENSG00000105810	validated
tarbase	hsa-miR-582-3p	CDK6	1021	ENSG00000105810	validated
tarbase	hsa-miR-582-5p	CDK6	1021	ENSG00000105810	validated
tarbase	hsa-miR-181a-5p	PMPCB	9512	ENSG00000105819	validated
tarbase	hsa-miR-582-5p	DNAJC2	27000	ENSG00000105821	validated
tarbase	hsa-miR-212-3p	TFPI2	7980	ENSG00000105825	validated
tarbase	hsa-miR-15b-5p	TWISTNB	221830	ENSG00000105849	validated
tarbase	hsa-miR-181a-5p	TWISTNB	221830	ENSG00000105849	validated
tarbase	hsa-miR-326	TWISTNB	221830	ENSG00000105849	validated
tarbase	hsa-miR-106b-5p	ITGB8	3696	ENSG00000105855	validated
tarbase	hsa-miR-301a-3p	ITGB8	3696	ENSG00000105855	validated
tarbase	hsa-miR-181a-5p	ITGB8	3696	ENSG00000105855	validated
tarbase	hsa-miR-15b-5p	HBP1	26959	ENSG00000105856	validated
tarbase	hsa-miR-21-5p	HBP1	26959	ENSG00000105856	validated
tarbase	hsa-miR-582-3p	SP4	6671	ENSG00000105866	validated
tarbase	hsa-miR-21-5p	CBLL1	79872	ENSG00000105879	validated
tarbase	hsa-miR-582-3p	CBLL1	79872	ENSG00000105879	validated
tarbase	hsa-miR-106b-5p	MTPN	136319	ENSG00000105887	validated
tarbase	hsa-miR-181a-5p	MTPN	136319	ENSG00000105887	validated
tarbase	hsa-miR-18a-5p	MTPN	136319	ENSG00000105887	validated
tarbase	hsa-miR-582-5p	MTPN	136319	ENSG00000105887	validated
tarbase	hsa-miR-212-3p	MTPN	136319	ENSG00000105887	validated
tarbase	hsa-miR-18a-5p	MPP6	51678	ENSG00000105926	validated
tarbase	hsa-miR-18a-5p	ZC3HAV1	56829	ENSG00000105939	validated
tarbase	hsa-miR-212-3p	ZC3HAV1	56829	ENSG00000105939	validated
tarbase	hsa-miR-212-3p	TTC26	79989	ENSG00000105948	validated
tarbase	hsa-miR-181a-5p	OGDH	4967	ENSG00000105953	validated
tarbase	hsa-miR-15b-5p	H2AZ2	94239	ENSG00000105968	validated
tarbase	hsa-miR-181a-5p	H2AZ2	94239	ENSG00000105968	validated
tarbase	hsa-miR-222-3p	CAV2	858	ENSG00000105971	validated
tarbase	hsa-miR-301a-3p	CAV2	858	ENSG00000105971	validated
tarbase	hsa-miR-582-3p	CAV1	857	ENSG00000105974	validated
tarbase	hsa-miR-181a-5p	MET	4233	ENSG00000105976	validated
tarbase	hsa-miR-301a-3p	MET	4233	ENSG00000105976	validated
tarbase	hsa-miR-222-3p	MET	4233	ENSG00000105976	validated
tarbase	hsa-miR-106b-5p	LMBR1	64327	ENSG00000105983	validated
tarbase	hsa-miR-212-3p	LMBR1	64327	ENSG00000105983	validated
tarbase	hsa-miR-301a-3p	LMBR1	64327	ENSG00000105983	validated
tarbase	hsa-miR-582-3p	HOXA1	3198	ENSG00000105991	validated
tarbase	hsa-miR-212-3p	HOXA1	3198	ENSG00000105991	validated
tarbase	hsa-miR-301a-3p	HOXA3	3200	ENSG00000105997	validated
tarbase	hsa-miR-212-3p	HOXA3	3200	ENSG00000105997	validated
tarbase	hsa-miR-15b-5p	LFNG	3955	ENSG00000106003	validated
tarbase	hsa-miR-582-5p	HOXA5	3202	ENSG00000106004	validated
tarbase	hsa-miR-222-3p	HOXA5	3202	ENSG00000106004	validated
tarbase	hsa-miR-106b-5p	HOXA6	3203	ENSG00000106006	validated
tarbase	hsa-miR-212-3p	IQCE	23288	ENSG00000106012	validated
tarbase	hsa-miR-106b-5p	TSPAN12	23554	ENSG00000106025	validated
tarbase	hsa-miR-18a-5p	TAX1BP1	8887	ENSG00000106052	validated
tarbase	hsa-miR-181a-5p	TAX1BP1	8887	ENSG00000106052	validated
tarbase	hsa-miR-212-3p	GRB10	2887	ENSG00000106070	validated
tarbase	hsa-miR-181a-5p	PLEKHA8	84725	ENSG00000106086	validated
tarbase	hsa-miR-212-3p	PLEKHA8	84725	ENSG00000106086	validated
tarbase	hsa-miR-296-5p	PLEKHA8	84725	ENSG00000106086	validated
tarbase	hsa-miR-106b-5p	GARS1	2617	ENSG00000106105	validated
tarbase	hsa-miR-15b-5p	GARS1	2617	ENSG00000106105	validated
tarbase	hsa-miR-181a-5p	GARS1	2617	ENSG00000106105	validated
tarbase	hsa-miR-212-3p	GARS1	2617	ENSG00000106105	validated
tarbase	hsa-miR-18a-5p	CASP2	835	ENSG00000106144	validated
tarbase	hsa-miR-21-5p	CASP2	835	ENSG00000106144	validated
tarbase	hsa-miR-212-3p	HSPB1	3315	ENSG00000106211	validated
tarbase	hsa-miR-15b-5p	NPTX2	4885	ENSG00000106236	validated
tarbase	hsa-miR-181a-5p	PTCD1	26024	ENSG00000106246	validated
tarbase	hsa-miR-106b-5p	ZKSCAN1	7586	ENSG00000106261	validated

tarbase	hsa-miR-144-3p	ZKSCAN1	7586	ENSG00000106261	validated
tarbase	hsa-miR-181a-5p	ZKSCAN1	7586	ENSG00000106261	validated
tarbase	hsa-miR-192-5p	ZKSCAN1	7586	ENSG00000106261	validated
tarbase	hsa-miR-21-5p	ZKSCAN1	7586	ENSG00000106261	validated
tarbase	hsa-miR-301a-3p	ZKSCAN1	7586	ENSG00000106261	validated
tarbase	hsa-miR-21-5p	EIF3B	8662	ENSG00000106263	validated
tarbase	hsa-miR-192-5p	TAF6	6878	ENSG00000106290	validated
tarbase	hsa-miR-181a-5p	WASL	8976	ENSG00000106299	validated
tarbase	hsa-miR-18a-5p	WASL	8976	ENSG00000106299	validated
tarbase	hsa-miR-582-5p	WASL	8976	ENSG00000106299	validated
tarbase	hsa-miR-106b-5p	AIMP2	7965	ENSG00000106305	validated
tarbase	hsa-miR-106b-5p	RBM28	55131	ENSG00000106344	validated
tarbase	hsa-miR-582-5p	USP42	84132	ENSG00000106346	validated
tarbase	hsa-miR-181a-5p	USP42	84132	ENSG00000106346	validated
tarbase	hsa-miR-222-3p	AGFG2	3268	ENSG00000106351	validated
tarbase	hsa-miR-181a-5p	SERPINE1	5054	ENSG00000106366	validated
tarbase	hsa-miR-326	AP1S1	1174	ENSG00000106367	validated
tarbase	hsa-miR-301a-3p	ZNHIT1	10467	ENSG00000106400	validated
tarbase	hsa-miR-296-5p	CLDN15	24146	ENSG00000106404	validated
tarbase	hsa-miR-144-3p	GLCCI1	113263	ENSG00000106415	validated
tarbase	hsa-miR-181a-5p	GLCCI1	113263	ENSG00000106415	validated
tarbase	hsa-miR-582-5p	GLCCI1	113263	ENSG00000106415	validated
tarbase	hsa-miR-582-3p	TMEM106B	54664	ENSG00000106460	validated
tarbase	hsa-miR-181a-5p	TMEM106B	54664	ENSG00000106460	validated
tarbase	hsa-miR-181a-5p	CEP41	95681	ENSG00000106477	validated
tarbase	hsa-miR-181a-5p	MEST	4232	ENSG00000106484	validated
tarbase	hsa-miR-106b-5p	ANKMY2	57037	ENSG00000106524	validated
tarbase	hsa-miR-301a-3p	TSPAN13	27075	ENSG00000106537	validated
tarbase	hsa-miR-106b-5p	AHR	196	ENSG00000106546	validated
tarbase	hsa-miR-18a-5p	AHR	196	ENSG00000106546	validated
tarbase	hsa-miR-21-5p	CHCHD3	54927	ENSG00000106554	validated
tarbase	hsa-miR-301a-3p	CHCHD3	54927	ENSG00000106554	validated
tarbase	hsa-miR-21-5p	URGCP	55665	ENSG00000106608	validated
tarbase	hsa-miR-301a-3p	URGCP	55665	ENSG00000106608	validated
tarbase	hsa-miR-505-3p	URGCP	55665	ENSG00000106608	validated
tarbase	hsa-miR-18a-5p	TMEM248	55069	ENSG00000106609	validated
tarbase	hsa-miR-222-3p	TMEM248	55069	ENSG00000106609	validated
tarbase	hsa-miR-106b-5p	TMEM248	55069	ENSG00000106609	validated
tarbase	hsa-miR-192-5p	CLIP2	7461	ENSG00000106665	validated
tarbase	hsa-miR-18a-5p	LIMK1	3984	ENSG00000106683	validated
tarbase	hsa-miR-582-5p	LIMK1	3984	ENSG00000106683	validated
tarbase	hsa-miR-15b-5p	FKTN	2218	ENSG00000106692	validated
tarbase	hsa-miR-21-5p	FKTN	2218	ENSG00000106692	validated
tarbase	hsa-miR-212-3p	FSD1L	83856	ENSG00000106701	validated
tarbase	hsa-miR-181a-5p	SPIN1	10927	ENSG00000106723	validated
tarbase	hsa-miR-212-3p	SPIN1	10927	ENSG00000106723	validated
tarbase	hsa-miR-144-3p	TMEM245	23731	ENSG00000106771	validated
tarbase	hsa-miR-181a-5p	TMEM245	23731	ENSG00000106771	validated
tarbase	hsa-miR-301a-3p	TMEM245	23731	ENSG00000106771	validated
tarbase	hsa-miR-15b-5p	MEGF9	1955	ENSG00000106780	validated
tarbase	hsa-miR-181a-5p	MEGF9	1955	ENSG00000106780	validated
tarbase	hsa-miR-212-3p	TRIM14	9830	ENSG00000106785	validated
tarbase	hsa-miR-106b-5p	TGFBR1	7046	ENSG00000106799	validated
tarbase	hsa-miR-21-5p	TGFBR1	7046	ENSG00000106799	validated
tarbase	hsa-miR-181a-5p	C5	727	ENSG00000106804	validated
tarbase	hsa-miR-505-3p	TLE4	7091	ENSG00000106829	validated
tarbase	hsa-miR-106b-5p	SUSD1	64420	ENSG00000106868	validated
tarbase	hsa-miR-181a-5p	AKNA	80709	ENSG00000106948	validated
tarbase	hsa-miR-582-5p	CDC37L1	55664	ENSG00000106993	validated
tarbase	hsa-miR-212-3p	CDC37L1	55664	ENSG00000106993	validated
tarbase	hsa-miR-15b-5p	TBC1D13	54662	ENSG00000107021	validated
tarbase	hsa-miR-301a-3p	TBC1D13	54662	ENSG00000107021	validated
tarbase	hsa-miR-582-3p	TBC1D13	54662	ENSG00000107021	validated
tarbase	hsa-miR-106b-5p	RIC1	57589	ENSG00000107036	validated
tarbase	hsa-miR-144-3p	RIC1	57589	ENSG00000107036	validated
tarbase	hsa-miR-15b-5p	RIC1	57589	ENSG00000107036	validated
tarbase	hsa-miR-21-5p	RIC1	57589	ENSG00000107036	validated
tarbase	hsa-miR-582-5p	RIC1	57589	ENSG00000107036	validated
tarbase	hsa-miR-21-5p	DOCK8	81704	ENSG00000107099	validated
tarbase	hsa-miR-181a-5p	KANK1	23189	ENSG00000107104	validated
tarbase	hsa-miR-15b-5p	ELAVL2	1993	ENSG00000107105	validated

tarbase	hsa-miR-181a-5p	ELAVL2	1993	ENSG00000107105	validated
tarbase	hsa-miR-181a-5p	NCS1	23413	ENSG00000107130	validated
tarbase	hsa-miR-15b-5p	CA9	768	ENSG00000107159	validated
tarbase	hsa-miR-21-5p	MPDZ	8777	ENSG00000107186	validated
tarbase	hsa-miR-301a-3p	MPDZ	8777	ENSG00000107186	validated
tarbase	hsa-miR-326	LHX3	8022	ENSG00000107187	validated
tarbase	hsa-miR-212-3p	DDX58	23586	ENSG00000107201	validated
tarbase	hsa-miR-106b-5p	RAPGEF1	2889	ENSG00000107263	validated
tarbase	hsa-miR-15b-5p	RAPGEF1	2889	ENSG00000107263	validated
tarbase	hsa-miR-21-5p	RAPGEF1	2889	ENSG00000107263	validated
tarbase	hsa-miR-296-5p	NPDC1	56654	ENSG00000107281	validated
tarbase	hsa-miR-301a-3p	NPDC1	56654	ENSG00000107281	validated
tarbase	hsa-miR-326	NPDC1	56654	ENSG00000107281	validated
tarbase	hsa-miR-106b-5p	SETX	23064	ENSG00000107290	validated
tarbase	hsa-miR-15b-5p	SETX	23064	ENSG00000107290	validated
tarbase	hsa-miR-21-5p	SETX	23064	ENSG00000107290	validated
tarbase	hsa-miR-181a-5p	SETX	23064	ENSG00000107290	validated
tarbase	hsa-miR-15b-5p	UBE2R2	54926	ENSG00000107341	validated
tarbase	hsa-miR-18a-5p	UBE2R2	54926	ENSG00000107341	validated
tarbase	hsa-miR-212-3p	UBE2R2	54926	ENSG00000107341	validated
tarbase	hsa-miR-106b-5p	ABHD17B	51104	ENSG00000107362	validated
tarbase	hsa-miR-15b-5p	ABHD17B	51104	ENSG00000107362	validated
tarbase	hsa-miR-181a-5p	EXOSC3	51010	ENSG00000107371	validated
tarbase	hsa-miR-181a-5p	ZFAND5	7763	ENSG00000107372	validated
tarbase	hsa-miR-15b-5p	CCNJ	54619	ENSG00000107443	validated
tarbase	hsa-miR-181a-5p	CCNJ	54619	ENSG00000107443	validated
tarbase	hsa-miR-222-3p	CCNJ	54619	ENSG00000107443	validated
tarbase	hsa-miR-582-5p	CCNJ	54619	ENSG00000107443	validated
tarbase	hsa-miR-15b-5p	GATA3	2625	ENSG00000107485	validated
tarbase	hsa-miR-21-5p	GATA3	2625	ENSG00000107485	validated
tarbase	hsa-miR-18a-5p	ATRNL1	26033	ENSG00000107518	validated
tarbase	hsa-miR-181a-5p	ATRNL1	26033	ENSG00000107518	validated
tarbase	hsa-miR-222-3p	ATRNL1	26033	ENSG00000107518	validated
tarbase	hsa-miR-326	HPS1	3257	ENSG00000107521	validated
tarbase	hsa-miR-181a-5p	PHYH	5264	ENSG00000107537	validated
tarbase	hsa-miR-222-3p	RAB11FIP2	22841	ENSG00000107560	validated
tarbase	hsa-miR-582-3p	RAB11FIP2	22841	ENSG00000107560	validated
tarbase	hsa-miR-144-3p	CXCL12	6387	ENSG00000107562	validated
tarbase	hsa-miR-15b-5p	ERLIN1	10613	ENSG00000107566	validated
tarbase	hsa-miR-18a-5p	ERLIN1	10613	ENSG00000107566	validated
tarbase	hsa-miR-222-3p	ERLIN1	10613	ENSG00000107566	validated
tarbase	hsa-miR-15b-5p	EIF3A	8661	ENSG00000107581	validated
tarbase	hsa-miR-296-5p	EIF3A	8661	ENSG00000107581	validated
tarbase	hsa-miR-21-5p	DDX50	79009	ENSG00000107625	validated
tarbase	hsa-miR-106b-5p	MAPK8	5599	ENSG00000107643	validated
tarbase	hsa-miR-15b-5p	MAPK8	5599	ENSG00000107643	validated
tarbase	hsa-miR-18a-5p	MAPK8	5599	ENSG00000107643	validated
tarbase	hsa-miR-106b-5p	SEC23IP	11196	ENSG00000107651	validated
tarbase	hsa-miR-15b-5p	SEC23IP	11196	ENSG00000107651	validated
tarbase	hsa-miR-106b-5p	ATE1	11101	ENSG00000107669	validated
tarbase	hsa-miR-181a-5p	PLEKHA1	59338	ENSG00000107679	validated
tarbase	hsa-miR-582-3p	PLEKHA1	59338	ENSG00000107679	validated
tarbase	hsa-miR-582-5p	PLEKHA1	59338	ENSG00000107679	validated
tarbase	hsa-miR-15b-5p	UNC5B	219699	ENSG00000107731	validated
tarbase	hsa-miR-582-3p	PPP3CB	5532	ENSG00000107758	validated
tarbase	hsa-miR-181a-5p	PPP3CB	5532	ENSG00000107758	validated
tarbase	hsa-miR-21-5p	CCSER2	54462	ENSG00000107771	validated
tarbase	hsa-miR-181a-5p	CCSER2	54462	ENSG00000107771	validated
tarbase	hsa-miR-106b-5p	LIPA	3988	ENSG00000107798	validated
tarbase	hsa-miR-222-3p	TWNK	56652	ENSG00000107815	validated
tarbase	hsa-miR-582-5p	LZTS2	84445	ENSG00000107816	validated
tarbase	hsa-miR-181a-5p	SFXN3	81855	ENSG00000107819	validated
tarbase	hsa-miR-582-3p	FBXW4	6468	ENSG00000107829	validated
tarbase	hsa-miR-144-3p	TNKS2	80351	ENSG00000107854	validated
tarbase	hsa-miR-15b-5p	TNKS2	80351	ENSG00000107854	validated
tarbase	hsa-miR-18a-5p	TNKS2	80351	ENSG00000107854	validated
tarbase	hsa-miR-222-3p	TNKS2	80351	ENSG00000107854	validated
tarbase	hsa-miR-21-5p	TNKS2	80351	ENSG00000107854	validated
tarbase	hsa-miR-15b-5p	GBF1	8729	ENSG00000107862	validated
tarbase	hsa-miR-326	GBF1	8729	ENSG00000107862	validated
tarbase	hsa-miR-212-3p	FBXL15	79176	ENSG00000107872	validated

tarbase	hsa-miR-326	CUEDC2	79004	ENSG00000107874	validated
tarbase	hsa-miR-106b-5p	SUFU	51684	ENSG00000107882	validated
tarbase	hsa-miR-21-5p	ANKRD26	22852	ENSG00000107890	validated
tarbase	hsa-miR-15b-5p	ANKRD26	22852	ENSG00000107890	validated
tarbase	hsa-miR-212-3p	ANKRD26	22852	ENSG00000107890	validated
tarbase	hsa-miR-15b-5p	ACBD5	91452	ENSG00000107897	validated
tarbase	hsa-miR-212-3p	LHPP	64077	ENSG00000107902	validated
tarbase	hsa-miR-15b-5p	LARP4B	23185	ENSG00000107929	validated
tarbase	hsa-miR-181a-5p	LARP4B	23185	ENSG00000107929	validated
tarbase	hsa-miR-18a-5p	LARP4B	23185	ENSG00000107929	validated
tarbase	hsa-miR-222-3p	LARP4B	23185	ENSG00000107929	validated
tarbase	hsa-miR-301a-3p	LARP4B	23185	ENSG00000107929	validated
tarbase	hsa-miR-582-5p	LARP4B	23185	ENSG00000107929	validated
tarbase	hsa-miR-505-3p	GTPBP4	23560	ENSG00000107937	validated
tarbase	hsa-miR-21-5p	BCCIP	56647	ENSG00000107949	validated
tarbase	hsa-miR-18a-5p	MTPAP	55149	ENSG00000107951	validated
tarbase	hsa-miR-106b-5p	SH3PXD2A	9644	ENSG00000107957	validated
tarbase	hsa-miR-326	SH3PXD2A	9644	ENSG00000107957	validated
tarbase	hsa-miR-106b-5p	MAP3K8	1326	ENSG00000107968	validated
tarbase	hsa-miR-181a-5p	MAP3K8	1326	ENSG00000107968	validated
tarbase	hsa-miR-212-3p	DKK1	22943	ENSG00000107984	validated
tarbase	hsa-miR-582-3p	DKK1	22943	ENSG00000107984	validated
tarbase	hsa-miR-15b-5p	TASOR2	54906	ENSG00000108021	validated
tarbase	hsa-miR-181a-5p	TASOR2	54906	ENSG00000108021	validated
tarbase	hsa-miR-18a-5p	TASOR2	54906	ENSG00000108021	validated
tarbase	hsa-miR-21-5p	TASOR2	54906	ENSG00000108021	validated
tarbase	hsa-miR-582-3p	TASOR2	54906	ENSG00000108021	validated
tarbase	hsa-miR-144-3p	SMC3	9126	ENSG00000108055	validated
tarbase	hsa-miR-15b-5p	SMC3	9126	ENSG00000108055	validated
tarbase	hsa-miR-296-5p	SMC3	9126	ENSG00000108055	validated
tarbase	hsa-miR-582-5p	SMC3	9126	ENSG00000108055	validated
tarbase	hsa-miR-301a-3p	SHOC2	8036	ENSG00000108061	validated
tarbase	hsa-miR-15b-5p	TFAM	7019	ENSG00000108064	validated
tarbase	hsa-miR-21-5p	CCDC6	8030	ENSG00000108091	validated
tarbase	hsa-miR-301a-3p	CCDC6	8030	ENSG00000108091	validated
tarbase	hsa-miR-21-5p	CUL2	8453	ENSG00000108094	validated
tarbase	hsa-miR-181a-5p	CCNY	219771	ENSG00000108100	validated
tarbase	hsa-miR-582-5p	CCNY	219771	ENSG00000108100	validated
tarbase	hsa-miR-296-5p	RPL28	6158	ENSG00000108107	validated
tarbase	hsa-miR-326	RPL28	6158	ENSG00000108107	validated
tarbase	hsa-miR-582-3p	RPL28	6158	ENSG00000108107	validated
tarbase	hsa-miR-181a-5p	ZMIZ1	57178	ENSG00000108175	validated
tarbase	hsa-miR-15b-5p	PPIF	10105	ENSG00000108179	validated
tarbase	hsa-miR-18a-5p	PPIF	10105	ENSG00000108179	validated
tarbase	hsa-miR-301a-3p	PPIF	10105	ENSG00000108179	validated
tarbase	hsa-miR-106b-5p	PPIF	10105	ENSG00000108179	validated
tarbase	hsa-miR-15b-5p	TSPAN14	81619	ENSG00000108219	validated
tarbase	hsa-miR-212-3p	KRT23	25984	ENSG00000108244	validated
tarbase	hsa-miR-144-3p	NUFIP2	57532	ENSG00000108256	validated
tarbase	hsa-miR-18a-5p	NUFIP2	57532	ENSG00000108256	validated
tarbase	hsa-miR-212-3p	NUFIP2	57532	ENSG00000108256	validated
tarbase	hsa-miR-301a-3p	NUFIP2	57532	ENSG00000108256	validated
tarbase	hsa-miR-326	NUFIP2	57532	ENSG00000108256	validated
tarbase	hsa-miR-181a-5p	NUFIP2	57532	ENSG00000108256	validated
tarbase	hsa-miR-222-3p	NUFIP2	57532	ENSG00000108256	validated
tarbase	hsa-miR-15b-5p	RPL19	6143	ENSG00000108298	validated
tarbase	hsa-miR-21-5p	RPL19	6143	ENSG00000108298	validated
tarbase	hsa-miR-18a-5p	UBTF	7343	ENSG00000108312	validated
tarbase	hsa-miR-301a-3p	UBTF	7343	ENSG00000108312	validated
tarbase	hsa-miR-181a-5p	PSMD3	5709	ENSG00000108344	validated
tarbase	hsa-miR-106b-5p	RAPGEFL1	51195	ENSG00000108352	validated
tarbase	hsa-miR-15b-5p	RNF43	54894	ENSG00000108375	validated
tarbase	hsa-miR-15b-5p	RAD51C	5889	ENSG00000108384	validated
tarbase	hsa-miR-18a-5p	RAD51C	5889	ENSG00000108384	validated
tarbase	hsa-miR-301a-3p	RAD51C	5889	ENSG00000108384	validated
tarbase	hsa-miR-18a-5p	MTMR4	9110	ENSG00000108389	validated
tarbase	hsa-miR-301a-3p	MTMR4	9110	ENSG00000108389	validated
tarbase	hsa-miR-15b-5p	TRIM37	4591	ENSG00000108395	validated
tarbase	hsa-miR-106b-5p	TUBD1	51174	ENSG00000108423	validated
tarbase	hsa-miR-15b-5p	KPNB1	3837	ENSG00000108424	validated
tarbase	hsa-miR-181a-5p	KPNB1	3837	ENSG00000108424	validated

tarbase	hsa-miR-18a-5p	KPNB1	3837	ENSG00000108424	validated
tarbase	hsa-miR-326	KPNB1	3837	ENSG00000108424	validated
tarbase	hsa-miR-212-3p	PNPO	55163	ENSG00000108439	validated
tarbase	hsa-miR-301a-3p	RPS6KB1	6198	ENSG00000108443	validated
tarbase	hsa-miR-212-3p	CBX1	10951	ENSG00000108468	validated
tarbase	hsa-miR-326	RECQL5	9400	ENSG00000108469	validated
tarbase	hsa-miR-15b-5p	CAMTA2	23125	ENSG00000108509	validated
tarbase	hsa-miR-181a-5p	CAMTA2	23125	ENSG00000108509	validated
tarbase	hsa-miR-106b-5p	MED13	9969	ENSG00000108510	validated
tarbase	hsa-miR-15b-5p	MED13	9969	ENSG00000108510	validated
tarbase	hsa-miR-181a-5p	MED13	9969	ENSG00000108510	validated
tarbase	hsa-miR-192-5p	MED13	9969	ENSG00000108510	validated
tarbase	hsa-miR-212-3p	MED13	9969	ENSG00000108510	validated
tarbase	hsa-miR-21-5p	MED13	9969	ENSG00000108510	validated
tarbase	hsa-miR-301a-3p	MED13	9969	ENSG00000108510	validated
tarbase	hsa-miR-505-3p	MED13	9969	ENSG00000108510	validated
tarbase	hsa-miR-212-3p	HOXB6	3216	ENSG00000108511	validated
tarbase	hsa-miR-106b-5p	PFN1	5216	ENSG00000108518	validated
tarbase	hsa-miR-106b-5p	RASD1	51655	ENSG00000108551	validated
tarbase	hsa-miR-15b-5p	RAI1	10743	ENSG00000108557	validated
tarbase	hsa-miR-222-3p	NUP88	4927	ENSG00000108559	validated
tarbase	hsa-miR-106b-5p	C1QBP	708	ENSG00000108561	validated
tarbase	hsa-miR-15b-5p	C1QBP	708	ENSG00000108561	validated
tarbase	hsa-miR-181a-5p	BLMH	642	ENSG00000108578	validated
tarbase	hsa-miR-181a-5p	CPD	1362	ENSG00000108582	validated
tarbase	hsa-miR-18a-5p	CPD	1362	ENSG00000108582	validated
tarbase	hsa-miR-505-3p	CPD	1362	ENSG00000108582	validated
tarbase	hsa-miR-15b-5p	CPD	1362	ENSG00000108582	validated
tarbase	hsa-miR-15b-5p	CCDC47	57003	ENSG00000108588	validated
tarbase	hsa-miR-106b-5p	AKAP10	11216	ENSG00000108599	validated
tarbase	hsa-miR-15b-5p	AKAP10	11216	ENSG00000108599	validated
tarbase	hsa-miR-181a-5p	SMARCD2	6603	ENSG00000108604	validated
tarbase	hsa-miR-296-5p	SMARCD2	6603	ENSG00000108604	validated
tarbase	hsa-miR-301a-3p	SMARCD2	6603	ENSG00000108604	validated
tarbase	hsa-miR-181a-5p	DDX5	1655	ENSG00000108654	validated
tarbase	hsa-miR-301a-3p	DDX5	1655	ENSG00000108654	validated
tarbase	hsa-miR-212-3p	C17orf75	64149	ENSG00000108666	validated
tarbase	hsa-miR-296-5p	LGALS3BP	3959	ENSG00000108679	validated
tarbase	hsa-miR-212-3p	LGALS3BP	3959	ENSG00000108679	validated
tarbase	hsa-miR-212-3p	CCL8	6355	ENSG00000108700	validated
tarbase	hsa-miR-582-3p	KAT2A	2648	ENSG00000108773	validated
tarbase	hsa-miR-301a-3p	RAB5C	5878	ENSG00000108774	validated
tarbase	hsa-miR-15b-5p	CNTNAP1	8506	ENSG00000108797	validated
tarbase	hsa-miR-326	EZH1	2145	ENSG00000108799	validated
tarbase	hsa-miR-582-5p	PPP1R9B	84687	ENSG00000108819	validated
tarbase	hsa-miR-181a-5p	COL1A1	1277	ENSG00000108821	validated
tarbase	hsa-miR-15b-5p	LRRC59	55379	ENSG00000108829	validated
tarbase	hsa-miR-18a-5p	LRRC59	55379	ENSG00000108829	validated
tarbase	hsa-miR-21-5p	LRRC59	55379	ENSG00000108829	validated
tarbase	hsa-miR-212-3p	RND2	8153	ENSG00000108830	validated
tarbase	hsa-miR-18a-5p	HDAC5	10014	ENSG00000108840	validated
tarbase	hsa-miR-15b-5p	ABCC3	8714	ENSG00000108846	validated
tarbase	hsa-miR-212-3p	LUC7L3	51747	ENSG00000108848	validated
tarbase	hsa-miR-15b-5p	MPP2	4355	ENSG00000108852	validated
tarbase	hsa-miR-192-5p	MPP2	4355	ENSG00000108852	validated
tarbase	hsa-miR-15b-5p	SMURF2	64750	ENSG00000108854	validated
tarbase	hsa-miR-18a-5p	DUSP3	1845	ENSG00000108861	validated
tarbase	hsa-miR-18a-5p	EFTUD2	9343	ENSG00000108883	validated
tarbase	hsa-miR-326	EFTUD2	9343	ENSG00000108883	validated
tarbase	hsa-miR-181a-5p	HLF	3131	ENSG00000108924	validated
tarbase	hsa-miR-212-3p	HLF	3131	ENSG00000108924	validated
tarbase	hsa-miR-18a-5p	SLC16A6	9120	ENSG00000108932	validated
tarbase	hsa-miR-222-3p	SLC16A6	9120	ENSG00000108932	validated
tarbase	hsa-miR-15b-5p	PRKAR1A	5573	ENSG00000108946	validated
tarbase	hsa-miR-212-3p	PRKAR1A	5573	ENSG00000108946	validated
tarbase	hsa-miR-301a-3p	FAM20A	54757	ENSG00000108950	validated
tarbase	hsa-miR-15b-5p	YWHAE	7531	ENSG00000108953	validated
tarbase	hsa-miR-15b-5p	MMD	23531	ENSG00000108960	validated
tarbase	hsa-miR-301a-3p	MMD	23531	ENSG00000108960	validated
tarbase	hsa-miR-21-5p	MMD	23531	ENSG00000108960	validated
tarbase	hsa-miR-15b-5p	WSB1	26118	ENSG00000109046	validated

tarbase	hsa-miR-181a-5p	WSB1	26118	ENSG00000109046	validated
tarbase	hsa-miR-181a-5p	SLC9A3R1	9368	ENSG00000109062	validated
tarbase	hsa-miR-15b-5p	TNFAIP1	7126	ENSG00000109079	validated
tarbase	hsa-miR-18a-5p	TMEM97	27346	ENSG00000109084	validated
tarbase	hsa-miR-222-3p	PMP22	5376	ENSG00000109099	validated
tarbase	hsa-miR-15b-5p	PMP22	5376	ENSG00000109099	validated
tarbase	hsa-miR-212-3p	FOXN1	8456	ENSG00000109101	validated
tarbase	hsa-miR-301a-3p	ALDOC	230	ENSG00000109107	validated
tarbase	hsa-miR-296-5p	RAB34	83871	ENSG00000109113	validated
tarbase	hsa-miR-106b-5p	PHF12	57649	ENSG00000109118	validated
tarbase	hsa-miR-144-3p	PHF12	57649	ENSG00000109118	validated
tarbase	hsa-miR-15b-5p	PHF12	57649	ENSG00000109118	validated
tarbase	hsa-miR-18a-5p	PHF12	57649	ENSG00000109118	validated
tarbase	hsa-miR-106b-5p	TMEM33	55161	ENSG00000109133	validated
tarbase	hsa-miR-326	TMEM33	55161	ENSG00000109133	validated
tarbase	hsa-miR-181a-5p	TMEM33	55161	ENSG00000109133	validated
tarbase	hsa-miR-181a-5p	GABRA4	2557	ENSG00000109158	validated
tarbase	hsa-miR-181a-5p	SLAIN2	57606	ENSG00000109171	validated
tarbase	hsa-miR-181a-5p	OCIAD1	54940	ENSG00000109180	validated
tarbase	hsa-miR-15b-5p	LAMTOR3	8649	ENSG00000109270	validated
tarbase	hsa-miR-301a-3p	NFKB1	4790	ENSG00000109320	validated
tarbase	hsa-miR-106b-5p	MANBA	4126	ENSG00000109323	validated
tarbase	hsa-miR-106b-5p	UBE2D3	7323	ENSG00000109332	validated
tarbase	hsa-miR-15b-5p	UBE2D3	7323	ENSG00000109332	validated
tarbase	hsa-miR-181a-5p	UBE2D3	7323	ENSG00000109332	validated
tarbase	hsa-miR-18a-5p	UBE2D3	7323	ENSG00000109332	validated
tarbase	hsa-miR-212-3p	UBE2D3	7323	ENSG00000109332	validated
tarbase	hsa-miR-582-5p	UBE2D3	7323	ENSG00000109332	validated
tarbase	hsa-miR-181a-5p	ELF2	1998	ENSG00000109381	validated
tarbase	hsa-miR-15b-5p	TBC1D9	23158	ENSG00000109436	validated
tarbase	hsa-miR-18a-5p	TBC1D9	23158	ENSG00000109436	validated
tarbase	hsa-miR-212-3p	TBC1D9	23158	ENSG00000109436	validated
tarbase	hsa-miR-301a-3p	TBC1D9	23158	ENSG00000109436	validated
tarbase	hsa-miR-326	TBC1D9	23158	ENSG00000109436	validated
tarbase	hsa-miR-181a-5p	TBC1D9	23158	ENSG00000109436	validated
tarbase	hsa-miR-222-3p	ZNF330	27309	ENSG00000109445	validated
tarbase	hsa-miR-21-5p	GAB1	2549	ENSG00000109458	validated
tarbase	hsa-miR-106b-5p	KLHL2	11275	ENSG00000109466	validated
tarbase	hsa-miR-15b-5p	KLHL2	11275	ENSG00000109466	validated
tarbase	hsa-miR-181a-5p	KLHL2	11275	ENSG00000109466	validated
tarbase	hsa-miR-106b-5p	WFS1	7466	ENSG00000109501	validated
tarbase	hsa-miR-106b-5p	GRPEL1	80273	ENSG00000109519	validated
tarbase	hsa-miR-181a-5p	GRPEL1	80273	ENSG00000109519	validated
tarbase	hsa-miR-18a-5p	GRPEL1	80273	ENSG00000109519	validated
tarbase	hsa-miR-21-5p	GRPEL1	80273	ENSG00000109519	validated
tarbase	hsa-miR-15b-5p	CLCN3	1182	ENSG00000109572	validated
tarbase	hsa-miR-222-3p	CLCN3	1182	ENSG00000109572	validated
tarbase	hsa-miR-15b-5p	GALNT7	51809	ENSG00000109586	validated
tarbase	hsa-miR-21-5p	GALNT7	51809	ENSG00000109586	validated
tarbase	hsa-miR-106b-5p	GALNT7	51809	ENSG00000109586	validated
tarbase	hsa-miR-15b-5p	DHX15	1665	ENSG00000109606	validated
tarbase	hsa-miR-181a-5p	DHX15	1665	ENSG00000109606	validated
tarbase	hsa-miR-192-5p	DHX15	1665	ENSG00000109606	validated
tarbase	hsa-miR-192-5p	TRIM2	23321	ENSG00000109654	validated
tarbase	hsa-miR-582-5p	TRIM2	23321	ENSG00000109654	validated
tarbase	hsa-miR-15b-5p	TRIM2	23321	ENSG00000109654	validated
tarbase	hsa-miR-15b-5p	FBXW7	55294	ENSG00000109670	validated
tarbase	hsa-miR-505-3p	FBXW7	55294	ENSG00000109670	validated
tarbase	hsa-miR-212-3p	TBC1D19	55296	ENSG00000109680	validated
tarbase	hsa-miR-212-3p	NSD2	7468	ENSG00000109685	validated
tarbase	hsa-miR-15b-5p	SH3D19	152503	ENSG00000109686	validated
tarbase	hsa-miR-181a-5p	SH3D19	152503	ENSG00000109686	validated
tarbase	hsa-miR-582-5p	STIM2	57620	ENSG00000109689	validated
tarbase	hsa-miR-21-5p	MFSD10	10227	ENSG00000109736	validated
tarbase	hsa-miR-212-3p	GLRB	2743	ENSG00000109738	validated
tarbase	hsa-miR-301a-3p	RAPGEF2	9693	ENSG00000109756	validated
tarbase	hsa-miR-15b-5p	RAPGEF2	9693	ENSG00000109756	validated
tarbase	hsa-miR-106b-5p	SNX25	83891	ENSG00000109762	validated
tarbase	hsa-miR-144-3p	KLF3	51274	ENSG00000109787	validated
tarbase	hsa-miR-181a-5p	KLF3	51274	ENSG00000109787	validated
tarbase	hsa-miR-192-5p	KLF3	51274	ENSG00000109787	validated

tarbase	hsa-miR-212-3p	KLF3	51274	ENSG00000109787	validated
tarbase	hsa-miR-21-5p	KLF3	51274	ENSG00000109787	validated
tarbase	hsa-miR-301a-3p	KLF3	51274	ENSG00000109787	validated
tarbase	hsa-miR-181a-5p	KLHL5	51088	ENSG00000109790	validated
tarbase	hsa-miR-106b-5p	KLHL5	51088	ENSG00000109790	validated
tarbase	hsa-miR-212-3p	NCAPG	64151	ENSG00000109805	validated
tarbase	hsa-miR-106b-5p	UGDH	7358	ENSG00000109814	validated
tarbase	hsa-miR-301a-3p	UGDH	7358	ENSG00000109814	validated
tarbase	hsa-miR-181a-5p	CTSC	1075	ENSG00000109861	validated
tarbase	hsa-miR-21-5p	CTSC	1075	ENSG00000109861	validated
tarbase	hsa-miR-21-5p	ZPR1	8882	ENSG00000109917	validated
tarbase	hsa-miR-181a-5p	MTCH2	23788	ENSG00000109919	validated
tarbase	hsa-miR-15b-5p	FNBP4	23360	ENSG00000109920	validated
tarbase	hsa-miR-15b-5p	TECTA	7007	ENSG00000109927	validated
tarbase	hsa-miR-212-3p	SC5D	6309	ENSG00000109929	validated
tarbase	hsa-miR-106b-5p	B3GAT1	27087	ENSG00000109956	validated
tarbase	hsa-miR-181a-5p	HSPA8	3312	ENSG00000109971	validated
tarbase	hsa-miR-18a-5p	HSPA8	3312	ENSG00000109971	validated
tarbase	hsa-miR-21-5p	HSPA8	3312	ENSG00000109971	validated
tarbase	hsa-miR-222-3p	HSPA8	3312	ENSG00000109971	validated
tarbase	hsa-miR-106b-5p	LPXN	9404	ENSG00000110031	validated
tarbase	hsa-miR-582-3p	DTX4	23220	ENSG00000110042	validated
tarbase	hsa-miR-21-5p	EHD1	10938	ENSG00000110047	validated
tarbase	hsa-miR-301a-3p	DCPS	28960	ENSG00000110063	validated
tarbase	hsa-miR-21-5p	KMT5B	51111	ENSG00000110066	validated
tarbase	hsa-miR-222-3p	KMT5B	51111	ENSG00000110066	validated
tarbase	hsa-miR-212-3p	KMT5B	51111	ENSG00000110066	validated
tarbase	hsa-miR-18a-5p	PPP6R3	55291	ENSG00000110075	validated
tarbase	hsa-miR-15b-5p	NRXN2	9379	ENSG00000110076	validated
tarbase	hsa-miR-15b-5p	CPT1A	1374	ENSG00000110090	validated
tarbase	hsa-miR-181a-5p	CPT1A	1374	ENSG00000110090	validated
tarbase	hsa-miR-181a-5p	CCND1	595	ENSG00000110092	validated
tarbase	hsa-miR-21-5p	CCND1	595	ENSG00000110092	validated
tarbase	hsa-miR-192-5p	CCDC86	79080	ENSG00000110104	validated
tarbase	hsa-miR-106b-5p	PRPF19	27339	ENSG00000110107	validated
tarbase	hsa-miR-296-5p	TMEM109	79073	ENSG00000110108	validated
tarbase	hsa-miR-106b-5p	TMEM109	79073	ENSG00000110108	validated
tarbase	hsa-miR-301a-3p	HPX	3263	ENSG00000110169	validated
tarbase	hsa-miR-106b-5p	TRIM3	10612	ENSG00000110171	validated
tarbase	hsa-miR-21-5p	CHORDC1	26973	ENSG00000110172	validated
tarbase	hsa-miR-296-5p	CHORDC1	26973	ENSG00000110172	validated
tarbase	hsa-miR-15b-5p	CHORDC1	26973	ENSG00000110172	validated
tarbase	hsa-miR-212-3p	CEP126	57562	ENSG00000110318	validated
tarbase	hsa-miR-144-3p	EIF4G2	1982	ENSG00000110321	validated
tarbase	hsa-miR-15b-5p	EIF4G2	1982	ENSG00000110321	validated
tarbase	hsa-miR-21-5p	EIF4G2	1982	ENSG00000110321	validated
tarbase	hsa-miR-326	EIF4G2	1982	ENSG00000110321	validated
tarbase	hsa-miR-505-3p	EIF4G2	1982	ENSG00000110321	validated
tarbase	hsa-miR-326	IL10RA	3587	ENSG00000110324	validated
tarbase	hsa-miR-18a-5p	BIRC2	329	ENSG00000110330	validated
tarbase	hsa-miR-181a-5p	UBE4A	9354	ENSG00000110344	validated
tarbase	hsa-miR-222-3p	UBE4A	9354	ENSG00000110344	validated
tarbase	hsa-miR-301a-3p	UBE4A	9354	ENSG00000110344	validated
tarbase	hsa-miR-106b-5p	DDX6	1656	ENSG00000110367	validated
tarbase	hsa-miR-15b-5p	DDX6	1656	ENSG00000110367	validated
tarbase	hsa-miR-181a-5p	DDX6	1656	ENSG00000110367	validated
tarbase	hsa-miR-18a-5p	DDX6	1656	ENSG00000110367	validated
tarbase	hsa-miR-212-3p	DDX6	1656	ENSG00000110367	validated
tarbase	hsa-miR-15b-5p	NECTIN1	5818	ENSG00000110400	validated
tarbase	hsa-miR-296-5p	NECTIN1	5818	ENSG00000110400	validated
tarbase	hsa-miR-106b-5p	HIPK3	10114	ENSG00000110422	validated
tarbase	hsa-miR-144-3p	HIPK3	10114	ENSG00000110422	validated
tarbase	hsa-miR-15b-5p	HIPK3	10114	ENSG00000110422	validated
tarbase	hsa-miR-181a-5p	HIPK3	10114	ENSG00000110422	validated
tarbase	hsa-miR-192-5p	HIPK3	10114	ENSG00000110422	validated
tarbase	hsa-miR-222-3p	HIPK3	10114	ENSG00000110422	validated
tarbase	hsa-miR-301a-3p	HIPK3	10114	ENSG00000110422	validated
tarbase	hsa-miR-15b-5p	KIAA1549L	25758	ENSG00000110427	validated
tarbase	hsa-miR-106b-5p	FBXO3	26273	ENSG00000110429	validated
tarbase	hsa-miR-15b-5p	FBXO3	26273	ENSG00000110429	validated
tarbase	hsa-miR-222-3p	FBXO3	26273	ENSG00000110429	validated

tarbase	hsa-miR-15b-5p	PDHX	8050	ENSG00000110435	validated
tarbase	hsa-miR-582-5p	PDHX	8050	ENSG00000110435	validated
tarbase	hsa-miR-181a-5p	SLC1A2	6506	ENSG00000110436	validated
tarbase	hsa-miR-212-3p	SLC1A2	6506	ENSG00000110436	validated
tarbase	hsa-miR-296-5p	MADD	8567	ENSG00000110514	validated
tarbase	hsa-miR-181a-5p	CARS1	833	ENSG00000110619	validated
tarbase	hsa-miR-106b-5p	CD81	975	ENSG00000110651	validated
tarbase	hsa-miR-15b-5p	CD81	975	ENSG00000110651	validated
tarbase	hsa-miR-222-3p	CD81	975	ENSG00000110651	validated
tarbase	hsa-miR-15b-5p	C11orf58	10944	ENSG00000110696	validated
tarbase	hsa-miR-18a-5p	C11orf58	10944	ENSG00000110696	validated
tarbase	hsa-miR-15b-5p	AIP	9049	ENSG00000110711	validated
tarbase	hsa-miR-15b-5p	NUP98	4928	ENSG00000110713	validated
tarbase	hsa-miR-296-5p	NDUFS8	4728	ENSG00000110717	validated
tarbase	hsa-miR-15b-5p	GTF2H1	2965	ENSG00000110768	validated
tarbase	hsa-miR-222-3p	GTF2H1	2965	ENSG00000110768	validated
tarbase	hsa-miR-106b-5p	PRDM4	11108	ENSG00000110851	validated
tarbase	hsa-miR-212-3p	CLEC2B	9976	ENSG00000110852	validated
tarbase	hsa-miR-15b-5p	COQ5	84274	ENSG00000110871	validated
tarbase	hsa-miR-106b-5p	CORO1C	23603	ENSG00000110880	validated
tarbase	hsa-miR-212-3p	CORO1C	23603	ENSG00000110880	validated
tarbase	hsa-miR-222-3p	CORO1C	23603	ENSG00000110880	validated
tarbase	hsa-miR-181a-5p	CORO1C	23603	ENSG00000110880	validated
tarbase	hsa-miR-144-3p	KCTD10	83892	ENSG00000110906	validated
tarbase	hsa-miR-15b-5p	KCTD10	83892	ENSG00000110906	validated
tarbase	hsa-miR-21-5p	KCTD10	83892	ENSG00000110906	validated
tarbase	hsa-miR-106b-5p	KCTD10	83892	ENSG00000110906	validated
tarbase	hsa-miR-15b-5p	SLC11A2	4891	ENSG00000110911	validated
tarbase	hsa-miR-181a-5p	SLC11A2	4891	ENSG00000110911	validated
tarbase	hsa-miR-15b-5p	MLEC	9761	ENSG00000110917	validated
tarbase	hsa-miR-181a-5p	MLEC	9761	ENSG00000110917	validated
tarbase	hsa-miR-212-3p	MLEC	9761	ENSG00000110917	validated
tarbase	hsa-miR-222-3p	MLEC	9761	ENSG00000110917	validated
tarbase	hsa-miR-582-3p	MLEC	9761	ENSG00000110917	validated
tarbase	hsa-miR-106b-5p	MVK	4598	ENSG00000110921	validated
tarbase	hsa-miR-212-3p	BIN2	51411	ENSG00000110934	validated
tarbase	hsa-miR-144-3p	ATP5F1B	506	ENSG00000110955	validated
tarbase	hsa-miR-15b-5p	ATP5F1B	506	ENSG00000110955	validated
tarbase	hsa-miR-181a-5p	ATP5F1B	506	ENSG00000110955	validated
tarbase	hsa-miR-301a-3p	ATP5F1B	506	ENSG00000110955	validated
tarbase	hsa-miR-301a-3p	PTGES3	10728	ENSG00000110958	validated
tarbase	hsa-miR-181a-5p	BCL7A	605	ENSG00000110987	validated
tarbase	hsa-miR-18a-5p	BCL7A	605	ENSG00000110987	validated
tarbase	hsa-miR-212-3p	BCL7A	605	ENSG00000110987	validated
tarbase	hsa-miR-212-3p	CYP27B1	1594	ENSG00000111012	validated
tarbase	hsa-miR-15b-5p	KRT18	3875	ENSG00000111057	validated
tarbase	hsa-miR-15b-5p	TNS2	23371	ENSG00000111077	validated
tarbase	hsa-miR-582-3p	PPM1H	57460	ENSG00000111110	validated
tarbase	hsa-miR-106b-5p	METAP2	10988	ENSG00000111142	validated
tarbase	hsa-miR-222-3p	METAP2	10988	ENSG00000111142	validated
tarbase	hsa-miR-21-5p	LTA4H	4048	ENSG00000111144	validated
tarbase	hsa-miR-15b-5p	ELK3	2004	ENSG00000111145	validated
tarbase	hsa-miR-296-5p	TRPV4	59341	ENSG00000111199	validated
tarbase	hsa-miR-212-3p	TRPV4	59341	ENSG00000111199	validated
tarbase	hsa-miR-21-5p	FOXM1	2305	ENSG00000111206	validated
tarbase	hsa-miR-296-5p	FOXM1	2305	ENSG00000111206	validated
tarbase	hsa-miR-18a-5p	VPS29	51699	ENSG00000111237	validated
tarbase	hsa-miR-181a-5p	SH2B3	10019	ENSG00000111252	validated
tarbase	hsa-miR-212-3p	MANSC1	54682	ENSG00000111261	validated
tarbase	hsa-miR-212-3p	DUSP16	80824	ENSG00000111266	validated
tarbase	hsa-miR-21-5p	DUSP16	80824	ENSG00000111266	validated
tarbase	hsa-miR-106b-5p	CREBL2	1389	ENSG00000111269	validated
tarbase	hsa-miR-222-3p	CREBL2	1389	ENSG00000111269	validated
tarbase	hsa-miR-15b-5p	ALDH2	217	ENSG00000111275	validated
tarbase	hsa-miR-18a-5p	CDKN1B	1027	ENSG00000111276	validated
tarbase	hsa-miR-582-5p	CDKN1B	1027	ENSG00000111276	validated
tarbase	hsa-miR-212-3p	CDKN1B	1027	ENSG00000111276	validated
tarbase	hsa-miR-181a-5p	NAA25	80018	ENSG00000111300	validated
tarbase	hsa-miR-21-5p	NAA25	80018	ENSG00000111300	validated
tarbase	hsa-miR-301a-3p	NAA25	80018	ENSG00000111300	validated
tarbase	hsa-miR-106b-5p	CDK2AP1	8099	ENSG00000111328	validated

tarbase	hsa-miR-15b-5p	CDK2AP1	8099	ENSG00000111328	validated
tarbase	hsa-miR-212-3p	CDK2AP1	8099	ENSG00000111328	validated
tarbase	hsa-miR-301a-3p	OAS3	4940	ENSG00000111331	validated
tarbase	hsa-miR-106b-5p	OAS3	4940	ENSG00000111331	validated
tarbase	hsa-miR-212-3p	OAS3	4940	ENSG00000111331	validated
tarbase	hsa-miR-106b-5p	OAS2	4939	ENSG00000111335	validated
tarbase	hsa-miR-212-3p	OAS2	4939	ENSG00000111335	validated
tarbase	hsa-miR-15b-5p	MGP	4256	ENSG00000111341	validated
tarbase	hsa-miR-21-5p	ARHGDIB	397	ENSG00000111348	validated
tarbase	hsa-miR-505-3p	GTF2H3	2967	ENSG00000111358	validated
tarbase	hsa-miR-505-3p	DDX55	57696	ENSG00000111364	validated
tarbase	hsa-miR-106b-5p	SLC38A1	81539	ENSG00000111371	validated
tarbase	hsa-miR-15b-5p	SLC38A1	81539	ENSG00000111371	validated
tarbase	hsa-miR-181a-5p	SLC38A1	81539	ENSG00000111371	validated
tarbase	hsa-miR-212-3p	SLC38A1	81539	ENSG00000111371	validated
tarbase	hsa-miR-181a-5p	C12orf49	79794	ENSG00000111412	validated
tarbase	hsa-miR-582-3p	C12orf49	79794	ENSG00000111412	validated
tarbase	hsa-miR-212-3p	FZD10	11211	ENSG00000111432	validated
tarbase	hsa-miR-222-3p	COPZ1	22818	ENSG00000111481	validated
tarbase	hsa-miR-582-3p	COPZ1	22818	ENSG00000111481	validated
tarbase	hsa-miR-106b-5p	TBC1D30	23329	ENSG00000111490	validated
tarbase	hsa-miR-144-3p	CAND1	55832	ENSG00000111530	validated
tarbase	hsa-miR-15b-5p	CAND1	55832	ENSG00000111530	validated
tarbase	hsa-miR-181a-5p	CAND1	55832	ENSG00000111530	validated
tarbase	hsa-miR-21-5p	CAND1	55832	ENSG00000111530	validated
tarbase	hsa-miR-222-3p	CAND1	55832	ENSG00000111530	validated
tarbase	hsa-miR-301a-3p	CAND1	55832	ENSG00000111530	validated
tarbase	hsa-miR-192-5p	RAB5B	5869	ENSG00000111540	validated
tarbase	hsa-miR-222-3p	RAB5B	5869	ENSG00000111540	validated
tarbase	hsa-miR-326	RAB5B	5869	ENSG00000111540	validated
tarbase	hsa-miR-181a-5p	RAB5B	5869	ENSG00000111540	validated
tarbase	hsa-miR-212-3p	MDM1	56890	ENSG00000111554	validated
tarbase	hsa-miR-18a-5p	NUP107	57122	ENSG00000111581	validated
tarbase	hsa-miR-326	NUP107	57122	ENSG00000111581	validated
tarbase	hsa-miR-301a-3p	CNOT2	4848	ENSG00000111596	validated
tarbase	hsa-miR-144-3p	CPSF6	11052	ENSG00000111605	validated
tarbase	hsa-miR-15b-5p	CPSF6	11052	ENSG00000111605	validated
tarbase	hsa-miR-181a-5p	CPSF6	11052	ENSG00000111605	validated
tarbase	hsa-miR-212-3p	CPSF6	11052	ENSG00000111605	validated
tarbase	hsa-miR-301a-3p	CPSF6	11052	ENSG00000111605	validated
tarbase	hsa-miR-106b-5p	MRPL51	51258	ENSG00000111639	validated
tarbase	hsa-miR-18a-5p	GAPDH	2597	ENSG00000111640	validated
tarbase	hsa-miR-192-5p	GAPDH	2597	ENSG00000111640	validated
tarbase	hsa-miR-21-5p	GAPDH	2597	ENSG00000111640	validated
tarbase	hsa-miR-106b-5p	CHD4	1108	ENSG00000111642	validated
tarbase	hsa-miR-15b-5p	CHD4	1108	ENSG00000111642	validated
tarbase	hsa-miR-301a-3p	CHD4	1108	ENSG00000111642	validated
tarbase	hsa-miR-21-5p	UHRF1BP1L	23074	ENSG00000111647	validated
tarbase	hsa-miR-15b-5p	COPS7A	50813	ENSG00000111652	validated
tarbase	hsa-miR-106b-5p	CDCA3	83461	ENSG00000111665	validated
tarbase	hsa-miR-212-3p	CDCA3	83461	ENSG00000111665	validated
tarbase	hsa-miR-15b-5p	CHPT1	56994	ENSG00000111666	validated
tarbase	hsa-miR-296-5p	ENO2	2026	ENSG00000111674	validated
tarbase	hsa-miR-106b-5p	ENO2	2026	ENSG00000111674	validated
tarbase	hsa-miR-192-5p	ATN1	1822	ENSG00000111676	validated
tarbase	hsa-miR-296-5p	ATN1	1822	ENSG00000111676	validated
tarbase	hsa-miR-326	ATN1	1822	ENSG00000111676	validated
tarbase	hsa-miR-181a-5p	SUDS3	64426	ENSG00000111707	validated
tarbase	hsa-miR-301a-3p	SUDS3	64426	ENSG00000111707	validated
tarbase	hsa-miR-15b-5p	GOLT1B	51026	ENSG00000111711	validated
tarbase	hsa-miR-301a-3p	GOLT1B	51026	ENSG00000111711	validated
tarbase	hsa-miR-181a-5p	GOLT1B	51026	ENSG00000111711	validated
tarbase	hsa-miR-106b-5p	HCFC2	29915	ENSG00000111727	validated
tarbase	hsa-miR-18a-5p	HCFC2	29915	ENSG00000111727	validated
tarbase	hsa-miR-301a-3p	HCFC2	29915	ENSG00000111727	validated
tarbase	hsa-miR-212-3p	HCFC2	29915	ENSG00000111727	validated
tarbase	hsa-miR-212-3p	CLEC4A	50856	ENSG00000111729	validated
tarbase	hsa-miR-181a-5p	C2CD5	9847	ENSG00000111731	validated
tarbase	hsa-miR-18a-5p	C2CD5	9847	ENSG00000111731	validated
tarbase	hsa-miR-301a-3p	C2CD5	9847	ENSG00000111731	validated
tarbase	hsa-miR-15b-5p	RAB35	11021	ENSG00000111737	validated

tarbase	hsa-miR-106b-5p	COL12A1	1303	ENSG00000111799	validated
tarbase	hsa-miR-15b-5p	COL12A1	1303	ENSG00000111799	validated
tarbase	hsa-miR-181a-5p	COL12A1	1303	ENSG00000111799	validated
tarbase	hsa-miR-18a-5p	COL12A1	1303	ENSG00000111799	validated
tarbase	hsa-miR-212-3p	COL12A1	1303	ENSG00000111799	validated
tarbase	hsa-miR-21-5p	COL12A1	1303	ENSG00000111799	validated
tarbase	hsa-miR-212-3p	BTN3A3	10384	ENSG00000111801	validated
tarbase	hsa-miR-15b-5p	TDP2	51567	ENSG00000111802	validated
tarbase	hsa-miR-106b-5p	FRK	2444	ENSG00000111816	validated
tarbase	hsa-miR-15b-5p	FRK	2444	ENSG00000111816	validated
tarbase	hsa-miR-181a-5p	FRK	2444	ENSG00000111816	validated
tarbase	hsa-miR-192-5p	FRK	2444	ENSG00000111816	validated
tarbase	hsa-miR-212-3p	FRK	2444	ENSG00000111816	validated
tarbase	hsa-miR-222-3p	FRK	2444	ENSG00000111816	validated
tarbase	hsa-miR-15b-5p	DSE	29940	ENSG00000111817	validated
tarbase	hsa-miR-212-3p	RSPH4A	345895	ENSG00000111834	validated
tarbase	hsa-miR-181a-5p	TMEM14C	51522	ENSG00000111843	validated
tarbase	hsa-miR-106b-5p	PAK1IP1	55003	ENSG00000111845	validated
tarbase	hsa-miR-181a-5p	GCNT2	2651	ENSG00000111846	validated
tarbase	hsa-miR-21-5p	GCNT2	2651	ENSG00000111846	validated
tarbase	hsa-miR-582-5p	GCNT2	2651	ENSG00000111846	validated
tarbase	hsa-miR-106b-5p	ASF1A	25842	ENSG00000111875	validated
tarbase	hsa-miR-505-3p	ASF1A	25842	ENSG00000111875	validated
tarbase	hsa-miR-18a-5p	MCM9	254394	ENSG00000111877	validated
tarbase	hsa-miR-21-5p	RNGTT	8732	ENSG00000111880	validated
tarbase	hsa-miR-15b-5p	MAN1A1	4121	ENSG00000111885	validated
tarbase	hsa-miR-222-3p	SERINC1	57515	ENSG00000111897	validated
tarbase	hsa-miR-301a-3p	HDDC2	51020	ENSG00000111906	validated
tarbase	hsa-miR-222-3p	HINT3	135114	ENSG00000111911	validated
tarbase	hsa-miR-106b-5p	NCOA7	135112	ENSG00000111912	validated
tarbase	hsa-miR-106b-5p	SASH1	23328	ENSG00000111961	validated
tarbase	hsa-miR-18a-5p	SASH1	23328	ENSG00000111961	validated
tarbase	hsa-miR-301a-3p	SASH1	23328	ENSG00000111961	validated
tarbase	hsa-miR-15b-5p	UST	10090	ENSG00000111962	validated
tarbase	hsa-miR-106b-5p	PPARD	5467	ENSG00000112033	validated
tarbase	hsa-miR-18a-5p	MAPK14	1432	ENSG00000112062	validated
tarbase	hsa-miR-505-3p	MAPK14	1432	ENSG00000112062	validated
tarbase	hsa-miR-15b-5p	KCTD20	222658	ENSG00000112078	validated
tarbase	hsa-miR-181a-5p	KCTD20	222658	ENSG00000112078	validated
tarbase	hsa-miR-301a-3p	KCTD20	222658	ENSG00000112078	validated
tarbase	hsa-miR-18a-5p	KCTD20	222658	ENSG00000112078	validated
tarbase	hsa-miR-106b-5p	STK38	11329	ENSG00000112079	validated
tarbase	hsa-miR-301a-3p	STK38	11329	ENSG00000112079	validated
tarbase	hsa-miR-21-5p	SOD2	6648	ENSG00000112096	validated
tarbase	hsa-miR-301a-3p	SOD2	6648	ENSG00000112096	validated
tarbase	hsa-miR-181a-5p	SOD2	6648	ENSG00000112096	validated
tarbase	hsa-miR-106b-5p	MCM3	4172	ENSG00000112118	validated
tarbase	hsa-miR-21-5p	MCM3	4172	ENSG00000112118	validated
tarbase	hsa-miR-301a-3p	PHACTR1	221692	ENSG00000112137	validated
tarbase	hsa-miR-222-3p	ICK	22858	ENSG00000112144	validated
tarbase	hsa-miR-21-5p	CD83	9308	ENSG00000112149	validated
tarbase	hsa-miR-15b-5p	MDN1	23195	ENSG00000112159	validated
tarbase	hsa-miR-21-5p	MDN1	23195	ENSG00000112159	validated
tarbase	hsa-miR-296-5p	MDN1	23195	ENSG00000112159	validated
tarbase	hsa-miR-181a-5p	BACH2	60468	ENSG00000112182	validated
tarbase	hsa-miR-18a-5p	BACH2	60468	ENSG00000112182	validated
tarbase	hsa-miR-181a-5p	ZNF451	26036	ENSG00000112200	validated
tarbase	hsa-miR-18a-5p	ZNF451	26036	ENSG00000112200	validated
tarbase	hsa-miR-192-5p	ZNF451	26036	ENSG00000112200	validated
tarbase	hsa-miR-222-3p	ZNF451	26036	ENSG00000112200	validated
tarbase	hsa-miR-301a-3p	ZNF451	26036	ENSG00000112200	validated
tarbase	hsa-miR-212-3p	ZNF451	26036	ENSG00000112200	validated
tarbase	hsa-miR-106b-5p	BAG2	9532	ENSG00000112208	validated
tarbase	hsa-miR-181a-5p	RAB23	51715	ENSG00000112210	validated
tarbase	hsa-miR-106b-5p	GPR63	81491	ENSG00000112218	validated
tarbase	hsa-miR-15b-5p	GPR63	81491	ENSG00000112218	validated
tarbase	hsa-miR-301a-3p	GPR63	81491	ENSG00000112218	validated
tarbase	hsa-miR-192-5p	GPR63	81491	ENSG00000112218	validated
tarbase	hsa-miR-144-3p	CCNC	892	ENSG00000112237	validated
tarbase	hsa-miR-15b-5p	CCNC	892	ENSG00000112237	validated
tarbase	hsa-miR-181a-5p	CCNC	892	ENSG00000112237	validated

tarbase	hsa-miR-212-3p	CCNC	892	ENSG00000112237	validated
tarbase	hsa-miR-222-3p	E2F3	1871	ENSG00000112242	validated
tarbase	hsa-miR-296-5p	E2F3	1871	ENSG00000112242	validated
tarbase	hsa-miR-582-3p	E2F3	1871	ENSG00000112242	validated
tarbase	hsa-miR-181a-5p	PTP4A1	7803	ENSG00000112245	validated
tarbase	hsa-miR-18a-5p	PTP4A1	7803	ENSG00000112245	validated
tarbase	hsa-miR-192-5p	PTP4A1	7803	ENSG00000112245	validated
tarbase	hsa-miR-301a-3p	PTP4A1	7803	ENSG00000112245	validated
tarbase	hsa-miR-212-3p	SIM1	6492	ENSG00000112246	validated
tarbase	hsa-miR-106b-5p	ASCC3	10973	ENSG00000112249	validated
tarbase	hsa-miR-15b-5p	ASCC3	10973	ENSG00000112249	validated
tarbase	hsa-miR-18a-5p	ASCC3	10973	ENSG00000112249	validated
tarbase	hsa-miR-106b-5p	BVES	11149	ENSG00000112276	validated
tarbase	hsa-miR-15b-5p	BVES	11149	ENSG00000112276	validated
tarbase	hsa-miR-181a-5p	BVES	11149	ENSG00000112276	validated
tarbase	hsa-miR-212-3p	BVES	11149	ENSG00000112276	validated
tarbase	hsa-miR-18a-5p	MED23	9439	ENSG00000112282	validated
tarbase	hsa-miR-301a-3p	MED23	9439	ENSG00000112282	validated
tarbase	hsa-miR-181a-5p	WASF1	8936	ENSG00000112290	validated
tarbase	hsa-miR-582-5p	ALDH5A1	7915	ENSG00000112294	validated
tarbase	hsa-miR-15b-5p	CRYBG1	202	ENSG00000112297	validated
tarbase	hsa-miR-106b-5p	CRYBG1	202	ENSG00000112297	validated
tarbase	hsa-miR-181a-5p	SMAP1	60682	ENSG00000112305	validated
tarbase	hsa-miR-106b-5p	C6orf62	81688	ENSG00000112308	validated
tarbase	hsa-miR-181a-5p	C6orf62	81688	ENSG00000112308	validated
tarbase	hsa-miR-301a-3p	C6orf62	81688	ENSG00000112308	validated
tarbase	hsa-miR-582-5p	C6orf62	81688	ENSG00000112308	validated
tarbase	hsa-miR-222-3p	GMNN	51053	ENSG00000112312	validated
tarbase	hsa-miR-106b-5p	SOBP	55084	ENSG00000112320	validated
tarbase	hsa-miR-15b-5p	SOBP	55084	ENSG00000112320	validated
tarbase	hsa-miR-181a-5p	SOBP	55084	ENSG00000112320	validated
tarbase	hsa-miR-18a-5p	SOBP	55084	ENSG00000112320	validated
tarbase	hsa-miR-301a-3p	SOBP	55084	ENSG00000112320	validated
tarbase	hsa-miR-212-3p	SLC17A2	10246	ENSG00000112337	validated
tarbase	hsa-miR-301a-3p	TRIM38	10475	ENSG00000112343	validated
tarbase	hsa-miR-181a-5p	PERP	64065	ENSG00000112378	validated
tarbase	hsa-miR-505-3p	PERP	64065	ENSG00000112378	validated
tarbase	hsa-miR-582-5p	PERP	64065	ENSG00000112378	validated
tarbase	hsa-miR-181a-5p	ARFGEF3	57221	ENSG00000112379	validated
tarbase	hsa-miR-106b-5p	SLC16A10	117247	ENSG00000112394	validated
tarbase	hsa-miR-181a-5p	HECA	51696	ENSG00000112406	validated
tarbase	hsa-miR-301a-3p	HECA	51696	ENSG00000112406	validated
tarbase	hsa-miR-106b-5p	PHACTR2	9749	ENSG00000112419	validated
tarbase	hsa-miR-144-3p	PHACTR2	9749	ENSG00000112419	validated
tarbase	hsa-miR-15b-5p	PHACTR2	9749	ENSG00000112419	validated
tarbase	hsa-miR-18a-5p	EPM2A	7957	ENSG00000112425	validated
tarbase	hsa-miR-222-3p	EPM2A	7957	ENSG00000112425	validated
tarbase	hsa-miR-212-3p	EPM2A	7957	ENSG00000112425	validated
tarbase	hsa-miR-181a-5p	CCR6	1235	ENSG00000112486	validated
tarbase	hsa-miR-21-5p	CCR6	1235	ENSG00000112486	validated
tarbase	hsa-miR-106b-5p	PHF1	5252	ENSG00000112511	validated
tarbase	hsa-miR-222-3p	PHF1	5252	ENSG00000112511	validated
tarbase	hsa-miR-15b-5p	QKI	9444	ENSG00000112531	validated
tarbase	hsa-miR-181a-5p	QKI	9444	ENSG00000112531	validated
tarbase	hsa-miR-505-3p	QKI	9444	ENSG00000112531	validated
tarbase	hsa-miR-582-5p	CCND3	896	ENSG00000112576	validated
tarbase	hsa-miR-106b-5p	BYSL	705	ENSG00000112578	validated
tarbase	hsa-miR-15b-5p	BYSL	705	ENSG00000112578	validated
tarbase	hsa-miR-15b-5p	TBP	6908	ENSG00000112592	validated
tarbase	hsa-miR-301a-3p	TBP	6908	ENSG00000112592	validated
tarbase	hsa-miR-181a-5p	BICRAL	23506	ENSG00000112624	validated
tarbase	hsa-miR-212-3p	BICRAL	23506	ENSG00000112624	validated
tarbase	hsa-miR-296-5p	SRF	6722	ENSG00000112658	validated
tarbase	hsa-miR-106b-5p	DNPH1	10591	ENSG00000112667	validated
tarbase	hsa-miR-106b-5p	EXOC2	55770	ENSG00000112685	validated
tarbase	hsa-miR-301a-3p	COX7A2	1347	ENSG00000112695	validated
tarbase	hsa-miR-106b-5p	TMEM30A	55754	ENSG00000112697	validated
tarbase	hsa-miR-144-3p	TMEM30A	55754	ENSG00000112697	validated
tarbase	hsa-miR-15b-5p	TMEM30A	55754	ENSG00000112697	validated
tarbase	hsa-miR-21-5p	TMEM30A	55754	ENSG00000112697	validated
tarbase	hsa-miR-326	VEGFA	7422	ENSG00000112715	validated

tarbase	hsa-miR-106b-5p	PRPF4B	8899	ENSG00000112739	validated
tarbase	hsa-miR-15b-5p	TTK	7272	ENSG00000112742	validated
tarbase	hsa-miR-582-5p	TTK	7272	ENSG00000112742	validated
tarbase	hsa-miR-212-3p	TTK	7272	ENSG00000112742	validated
tarbase	hsa-miR-296-5p	SLC29A1	2030	ENSG00000112759	validated
tarbase	hsa-miR-301a-3p	BTN2A1	11120	ENSG00000112763	validated
tarbase	hsa-miR-106b-5p	TENT5A	55603	ENSG00000112773	validated
tarbase	hsa-miR-15b-5p	TENT5A	55603	ENSG00000112773	validated
tarbase	hsa-miR-222-3p	TENT5A	55603	ENSG00000112773	validated
tarbase	hsa-miR-301a-3p	TENT5A	55603	ENSG00000112773	validated
tarbase	hsa-miR-296-5p	FBRSL1	57666	ENSG00000112787	validated
tarbase	hsa-miR-212-3p	TBX18	9096	ENSG00000112837	validated
tarbase	hsa-miR-212-3p	NUDT12	83594	ENSG00000112874	validated
tarbase	hsa-miR-106b-5p	MAN2A1	4124	ENSG00000112893	validated
tarbase	hsa-miR-21-5p	TENT4A	11044	ENSG00000112941	validated
tarbase	hsa-miR-212-3p	GHR	2690	ENSG00000112964	validated
tarbase	hsa-miR-106b-5p	HMGCS1	3157	ENSG00000112972	validated
tarbase	hsa-miR-15b-5p	HMGCS1	3157	ENSG00000112972	validated
tarbase	hsa-miR-181a-5p	HMGCS1	3157	ENSG00000112972	validated
tarbase	hsa-miR-222-3p	HMGCS1	3157	ENSG00000112972	validated
tarbase	hsa-miR-326	HMGCS1	3157	ENSG00000112972	validated
tarbase	hsa-miR-21-5p	BRD8	10902	ENSG00000112983	validated
tarbase	hsa-miR-18a-5p	KIF20A	10112	ENSG00000112984	validated
tarbase	hsa-miR-212-3p	KIF20A	10112	ENSG00000112984	validated
tarbase	hsa-miR-15b-5p	HSPA9	3313	ENSG00000113013	validated
tarbase	hsa-miR-18a-5p	HSPA9	3313	ENSG00000113013	validated
tarbase	hsa-miR-582-5p	HBEGF	1839	ENSG00000113070	validated
tarbase	hsa-miR-15b-5p	SPARC	6678	ENSG00000113140	validated
tarbase	hsa-miR-222-3p	SPARC	6678	ENSG00000113140	validated
tarbase	hsa-miR-212-3p	SPARC	6678	ENSG00000113140	validated
tarbase	hsa-miR-15b-5p	HMGCR	3156	ENSG00000113161	validated
tarbase	hsa-miR-181a-5p	HMGCR	3156	ENSG00000113161	validated
tarbase	hsa-miR-212-3p	HMGCR	3156	ENSG00000113161	validated
tarbase	hsa-miR-582-5p	HMGCR	3156	ENSG00000113161	validated
tarbase	hsa-miR-582-5p	CERT1	10087	ENSG00000113163	validated
tarbase	hsa-miR-222-3p	FAF2	23197	ENSG00000113194	validated
tarbase	hsa-miR-301a-3p	HAND1	9421	ENSG00000113196	validated
tarbase	hsa-miR-181a-5p	PDE8B	8622	ENSG00000113231	validated
tarbase	hsa-miR-582-5p	PDE8B	8622	ENSG00000113231	validated
tarbase	hsa-miR-181a-5p	CLINT1	9685	ENSG00000113282	validated
tarbase	hsa-miR-582-3p	CLINT1	9685	ENSG00000113282	validated
tarbase	hsa-miR-15b-5p	CNOT6	57472	ENSG00000113300	validated
tarbase	hsa-miR-18a-5p	CNOT6	57472	ENSG00000113300	validated
tarbase	hsa-miR-21-5p	CNOT6	57472	ENSG00000113300	validated
tarbase	hsa-miR-301a-3p	CNOT6	57472	ENSG00000113300	validated
tarbase	hsa-miR-21-5p	TTC1	7265	ENSG00000113312	validated
tarbase	hsa-miR-18a-5p	CCNG1	900	ENSG00000113328	validated
tarbase	hsa-miR-582-5p	CCNG1	900	ENSG00000113328	validated
tarbase	hsa-miR-18a-5p	POLR3G	10622	ENSG00000113356	validated
tarbase	hsa-miR-181a-5p	DROSHA	29102	ENSG00000113360	validated
tarbase	hsa-miR-15b-5p	LMNB1	4001	ENSG00000113368	validated
tarbase	hsa-miR-18a-5p	LMNB1	4001	ENSG00000113368	validated
tarbase	hsa-miR-212-3p	LMNB1	4001	ENSG00000113368	validated
tarbase	hsa-miR-21-5p	LMNB1	4001	ENSG00000113368	validated
tarbase	hsa-miR-181a-5p	LMNB1	4001	ENSG00000113368	validated
tarbase	hsa-miR-106b-5p	ARRDC3	57561	ENSG00000113369	validated
tarbase	hsa-miR-21-5p	ARRDC3	57561	ENSG00000113369	validated
tarbase	hsa-miR-301a-3p	ARRDC3	57561	ENSG00000113369	validated
tarbase	hsa-miR-106b-5p	GOLPH3	64083	ENSG00000113384	validated
tarbase	hsa-miR-181a-5p	GOLPH3	64083	ENSG00000113384	validated
tarbase	hsa-miR-582-3p	GOLPH3	64083	ENSG00000113384	validated
tarbase	hsa-miR-21-5p	SUB1	10923	ENSG00000113387	validated
tarbase	hsa-miR-181a-5p	SUB1	10923	ENSG00000113387	validated
tarbase	hsa-miR-106b-5p	LNPEP	4012	ENSG00000113441	validated
tarbase	hsa-miR-181a-5p	LNPEP	4012	ENSG00000113441	validated
tarbase	hsa-miR-222-3p	LNPEP	4012	ENSG00000113441	validated
tarbase	hsa-miR-181a-5p	RAD1	5810	ENSG00000113456	validated
tarbase	hsa-miR-106b-5p	BRIX1	55299	ENSG00000113460	validated
tarbase	hsa-miR-181a-5p	BRIX1	55299	ENSG00000113460	validated
tarbase	hsa-miR-222-3p	BRIX1	55299	ENSG00000113460	validated
tarbase	hsa-miR-192-5p	PRLR	5618	ENSG00000113494	validated

tarbase	hsa-miR-21-5p	PRLR	5618	ENSG00000113494	validated
tarbase	hsa-miR-212-3p	RAD50	10111	ENSG00000113522	validated
tarbase	hsa-miR-212-3p	IL5	3567	ENSG00000113525	validated
tarbase	hsa-miR-181a-5p	ST8SIA4	7903	ENSG00000113532	validated
tarbase	hsa-miR-192-5p	GNPDA1	10007	ENSG00000113552	validated
tarbase	hsa-miR-181a-5p	SKP1	6500	ENSG00000113558	validated
tarbase	hsa-miR-582-5p	NUP155	9631	ENSG00000113569	validated
tarbase	hsa-miR-181a-5p	NUP155	9631	ENSG00000113569	validated
tarbase	hsa-miR-106b-5p	PPP2CA	5515	ENSG00000113575	validated
tarbase	hsa-miR-15b-5p	PPP2CA	5515	ENSG00000113575	validated
tarbase	hsa-miR-181a-5p	PPP2CA	5515	ENSG00000113575	validated
tarbase	hsa-miR-301a-3p	PPP2CA	5515	ENSG00000113575	validated
tarbase	hsa-miR-144-3p	FGF1	2246	ENSG00000113578	validated
tarbase	hsa-miR-181a-5p	NR3C1	2908	ENSG00000113580	validated
tarbase	hsa-miR-301a-3p	NR3C1	2908	ENSG00000113580	validated
tarbase	hsa-miR-21-5p	NR3C1	2908	ENSG00000113580	validated
tarbase	hsa-miR-181a-5p	LIFR	3977	ENSG00000113594	validated
tarbase	hsa-miR-222-3p	LIFR	3977	ENSG00000113594	validated
tarbase	hsa-miR-181a-5p	TRIM23	373	ENSG00000113595	validated
tarbase	hsa-miR-181a-5p	SEC24A	10802	ENSG00000113615	validated
tarbase	hsa-miR-212-3p	SEC24A	10802	ENSG00000113615	validated
tarbase	hsa-miR-106b-5p	SEC24A	10802	ENSG00000113615	validated
tarbase	hsa-miR-181a-5p	TXNDC15	79770	ENSG00000113621	validated
tarbase	hsa-miR-15b-5p	RARS1	5917	ENSG00000113643	validated
tarbase	hsa-miR-181a-5p	WWC1	23286	ENSG00000113645	validated
tarbase	hsa-miR-192-5p	MACROH2A1	9555	ENSG00000113648	validated
tarbase	hsa-miR-21-5p	MACROH2A1	9555	ENSG00000113648	validated
tarbase	hsa-miR-106b-5p	TCERG1	10915	ENSG00000113649	validated
tarbase	hsa-miR-181a-5p	TCERG1	10915	ENSG00000113649	validated
tarbase	hsa-miR-192-5p	TCERG1	10915	ENSG00000113649	validated
tarbase	hsa-miR-181a-5p	SMAD5	4090	ENSG00000113658	validated
tarbase	hsa-miR-212-3p	SMAD5	4090	ENSG00000113658	validated
tarbase	hsa-miR-301a-3p	SMAD5	4090	ENSG00000113658	validated
tarbase	hsa-miR-15b-5p	CSNK1A1	1452	ENSG00000113712	validated
tarbase	hsa-miR-18a-5p	CSNK1A1	1452	ENSG00000113712	validated
tarbase	hsa-miR-301a-3p	CSNK1A1	1452	ENSG00000113712	validated
tarbase	hsa-miR-18a-5p	ERGIC1	57222	ENSG00000113719	validated
tarbase	hsa-miR-326	ERGIC1	57222	ENSG00000113719	validated
tarbase	hsa-miR-18a-5p	ATP6V0E1	8992	ENSG00000113732	validated
tarbase	hsa-miR-192-5p	ATP6V0E1	8992	ENSG00000113732	validated
tarbase	hsa-miR-181a-5p	STC2	8614	ENSG00000113739	validated
tarbase	hsa-miR-301a-3p	STC2	8614	ENSG00000113739	validated
tarbase	hsa-miR-106b-5p	STC2	8614	ENSG00000113739	validated
tarbase	hsa-miR-15b-5p	CPEB4	80315	ENSG00000113742	validated
tarbase	hsa-miR-301a-3p	CPEB4	80315	ENSG00000113742	validated
tarbase	hsa-miR-212-3p	DBN1	1627	ENSG00000113758	validated
tarbase	hsa-miR-15b-5p	ZNF346	23567	ENSG00000113761	validated
tarbase	hsa-miR-106b-5p	SMC4	10051	ENSG00000113810	validated
tarbase	hsa-miR-181a-5p	SMC4	10051	ENSG00000113810	validated
tarbase	hsa-miR-21-5p	SMC4	10051	ENSG00000113810	validated
tarbase	hsa-miR-301a-3p	SMC4	10051	ENSG00000113810	validated
tarbase	hsa-miR-18a-5p	SMC4	10051	ENSG00000113810	validated
tarbase	hsa-miR-181a-5p	SELENOK	58515	ENSG00000113811	validated
tarbase	hsa-miR-301a-3p	SELENOK	58515	ENSG00000113811	validated
tarbase	hsa-miR-106b-5p	TIMMDC1	51300	ENSG00000113845	validated
tarbase	hsa-miR-582-5p	CRBN	51185	ENSG00000113851	validated
tarbase	hsa-miR-106b-5p	CRBN	51185	ENSG00000113851	validated
tarbase	hsa-miR-181a-5p	AMOTL2	51421	ENSG00000114019	validated
tarbase	hsa-miR-301a-3p	AMOTL2	51421	ENSG00000114019	validated
tarbase	hsa-miR-106b-5p	KPNA1	3836	ENSG00000114030	validated
tarbase	hsa-miR-18a-5p	KPNA1	3836	ENSG00000114030	validated
tarbase	hsa-miR-301a-3p	KPNA1	3836	ENSG00000114030	validated
tarbase	hsa-miR-106b-5p	PCCB	5096	ENSG00000114054	validated
tarbase	hsa-miR-15b-5p	UBE3A	7337	ENSG00000114062	validated
tarbase	hsa-miR-181a-5p	UBE3A	7337	ENSG00000114062	validated
tarbase	hsa-miR-106b-5p	ARMC8	25852	ENSG00000114098	validated
tarbase	hsa-miR-18a-5p	ARMC8	25852	ENSG00000114098	validated
tarbase	hsa-miR-21-5p	ARMC8	25852	ENSG00000114098	validated
tarbase	hsa-miR-582-5p	ARMC8	25852	ENSG00000114098	validated
tarbase	hsa-miR-106b-5p	RBP1	5947	ENSG00000114115	validated
tarbase	hsa-miR-106b-5p	SLC25A36	55186	ENSG00000114120	validated

tarbase	hsa-miR-181a-5p	SLC25A36	55186	ENSG00000114120	validated
tarbase	hsa-miR-212-3p	SLC25A36	55186	ENSG00000114120	validated
tarbase	hsa-miR-582-5p	SLC25A36	55186	ENSG00000114120	validated
tarbase	hsa-miR-181a-5p	RNF7	9616	ENSG00000114125	validated
tarbase	hsa-miR-15b-5p	XRN1	54464	ENSG00000114127	validated
tarbase	hsa-miR-181a-5p	XRN1	54464	ENSG00000114127	validated
tarbase	hsa-miR-212-3p	XRN1	54464	ENSG00000114127	validated
tarbase	hsa-miR-222-3p	XRN1	54464	ENSG00000114127	validated
tarbase	hsa-miR-212-3p	KAT2B	8850	ENSG00000114166	validated
tarbase	hsa-miR-212-3p	WNT5A	7474	ENSG00000114251	validated
tarbase	hsa-miR-301a-3p	WNT5A	7474	ENSG00000114251	validated
tarbase	hsa-miR-181a-5p	FGF12	2257	ENSG00000114279	validated
tarbase	hsa-miR-181a-5p	ACAP2	23527	ENSG00000114331	validated
tarbase	hsa-miR-106b-5p	ECT2	1894	ENSG00000114346	validated
tarbase	hsa-miR-21-5p	ECT2	1894	ENSG00000114346	validated
tarbase	hsa-miR-326	ECT2	1894	ENSG00000114346	validated
tarbase	hsa-miR-181a-5p	GNAI2	2771	ENSG00000114353	validated
tarbase	hsa-miR-301a-3p	GNAI2	2771	ENSG00000114353	validated
tarbase	hsa-miR-15b-5p	TFG	10342	ENSG00000114354	validated
tarbase	hsa-miR-222-3p	TFG	10342	ENSG00000114354	validated
tarbase	hsa-miR-181a-5p	USP9Y	8287	ENSG00000114374	validated
tarbase	hsa-miR-106b-5p	TUSC2	11334	ENSG00000114383	validated
tarbase	hsa-miR-15b-5p	TUSC2	11334	ENSG00000114383	validated
tarbase	hsa-miR-212-3p	TUSC2	11334	ENSG00000114383	validated
tarbase	hsa-miR-326	CYB561D2	11068	ENSG00000114395	validated
tarbase	hsa-miR-181a-5p	C3orf14	57415	ENSG00000114405	validated
tarbase	hsa-miR-106b-5p	FXR1	8087	ENSG00000114416	validated
tarbase	hsa-miR-21-5p	FXR1	8087	ENSG00000114416	validated
tarbase	hsa-miR-212-3p	CBLB	868	ENSG00000114423	validated
tarbase	hsa-miR-18a-5p	BBX	56987	ENSG00000114439	validated
tarbase	hsa-miR-181a-5p	GNB4	59345	ENSG00000114450	validated
tarbase	hsa-miR-222-3p	GNB4	59345	ENSG00000114450	validated
tarbase	hsa-miR-181a-5p	SNX4	8723	ENSG00000114520	validated
tarbase	hsa-miR-15b-5p	FRMD4B	23150	ENSG00000114541	validated
tarbase	hsa-miR-301a-3p	SLC41A3	54946	ENSG00000114544	validated
tarbase	hsa-miR-15b-5p	PLXNA1	5361	ENSG00000114554	validated
tarbase	hsa-miR-18a-5p	PLXNA1	5361	ENSG00000114554	validated
tarbase	hsa-miR-144-3p	ATP6V1A	523	ENSG00000114573	validated
tarbase	hsa-miR-21-5p	ATP6V1A	523	ENSG00000114573	validated
tarbase	hsa-miR-582-3p	ATP6V1A	523	ENSG00000114573	validated
tarbase	hsa-miR-582-5p	ATP6V1A	523	ENSG00000114573	validated
tarbase	hsa-miR-15b-5p	ATP6V1A	523	ENSG00000114573	validated
tarbase	hsa-miR-181a-5p	ATP6V1A	523	ENSG00000114573	validated
tarbase	hsa-miR-106b-5p	MRPL3	11222	ENSG00000114686	validated
tarbase	hsa-miR-192-5p	MRPL3	11222	ENSG00000114686	validated
tarbase	hsa-miR-106b-5p	PLSCR4	57088	ENSG00000114698	validated
tarbase	hsa-miR-181a-5p	CISH	1154	ENSG00000114737	validated
tarbase	hsa-miR-181a-5p	ACVR2B	93	ENSG00000114739	validated
tarbase	hsa-miR-18a-5p	ACVR2B	93	ENSG00000114739	validated
tarbase	hsa-miR-212-3p	ACVR2B	93	ENSG00000114739	validated
tarbase	hsa-miR-222-3p	ACVR2B	93	ENSG00000114739	validated
tarbase	hsa-miR-301a-3p	ACVR2B	93	ENSG00000114739	validated
tarbase	hsa-miR-326	ACVR2B	93	ENSG00000114739	validated
tarbase	hsa-miR-582-5p	ACVR2B	93	ENSG00000114739	validated
tarbase	hsa-miR-106b-5p	WDR48	57599	ENSG00000114742	validated
tarbase	hsa-miR-181a-5p	WDR48	57599	ENSG00000114742	validated
tarbase	hsa-miR-106b-5p	PEX5L	51555	ENSG00000114757	validated
tarbase	hsa-miR-21-5p	PEX5L	51555	ENSG00000114757	validated
tarbase	hsa-miR-15b-5p	RRP9	9136	ENSG00000114767	validated
tarbase	hsa-miR-15b-5p	ABCC5	10057	ENSG00000114770	validated
tarbase	hsa-miR-21-5p	ABCC5	10057	ENSG00000114770	validated
tarbase	hsa-miR-326	ABCC5	10057	ENSG00000114770	validated
tarbase	hsa-miR-18a-5p	ARHGEF26	26084	ENSG00000114790	validated
tarbase	hsa-miR-106b-5p	SSR3	6747	ENSG00000114850	validated
tarbase	hsa-miR-181a-5p	SSR3	6747	ENSG00000114850	validated
tarbase	hsa-miR-222-3p	SSR3	6747	ENSG00000114850	validated
tarbase	hsa-miR-15b-5p	ZBTB47	92999	ENSG00000114853	validated
tarbase	hsa-miR-301a-3p	ZBTB47	92999	ENSG00000114853	validated
tarbase	hsa-miR-212-3p	TNNC1	7134	ENSG00000114854	validated
tarbase	hsa-miR-18a-5p	NKTR	4820	ENSG00000114857	validated
tarbase	hsa-miR-222-3p	NKTR	4820	ENSG00000114857	validated

tarbase	hsa-miR-181a-5p	FOXP1	27086	ENSG00000114861	validated
tarbase	hsa-miR-15b-5p	FOXP1	27086	ENSG00000114861	validated
tarbase	hsa-miR-15b-5p	EIF4G1	1981	ENSG00000114867	validated
tarbase	hsa-miR-21-5p	EIF4G1	1981	ENSG00000114867	validated
tarbase	hsa-miR-296-5p	EIF4G1	1981	ENSG00000114867	validated
tarbase	hsa-miR-326	EIF4G1	1981	ENSG00000114867	validated
tarbase	hsa-miR-582-3p	EIF4G1	1981	ENSG00000114867	validated
tarbase	hsa-miR-18a-5p	INO80D	54891	ENSG00000114933	validated
tarbase	hsa-miR-301a-3p	INO80D	54891	ENSG00000114933	validated
tarbase	hsa-miR-15b-5p	ADAM23	8745	ENSG00000114948	validated
tarbase	hsa-miR-181a-5p	DGUOK	1716	ENSG00000114956	validated
tarbase	hsa-miR-106b-5p	MOB1A	55233	ENSG00000114978	validated
tarbase	hsa-miR-18a-5p	MOB1A	55233	ENSG00000114978	validated
tarbase	hsa-miR-222-3p	MOB1A	55233	ENSG00000114978	validated
tarbase	hsa-miR-296-5p	MOB1A	55233	ENSG00000114978	validated
tarbase	hsa-miR-15b-5p	LMAN2L	81562	ENSG00000114988	validated
tarbase	hsa-miR-301a-3p	RTKN	6242	ENSG00000114993	validated
tarbase	hsa-miR-18a-5p	TTL	150465	ENSG00000114999	validated
tarbase	hsa-miR-21-5p	TTL	150465	ENSG00000114999	validated
tarbase	hsa-miR-301a-3p	TTL	150465	ENSG00000114999	validated
tarbase	hsa-miR-301a-3p	PIKFYVE	200576	ENSG00000115020	validated
tarbase	hsa-miR-106b-5p	NCL	4691	ENSG00000115053	validated
tarbase	hsa-miR-144-3p	NCL	4691	ENSG00000115053	validated
tarbase	hsa-miR-21-5p	NCL	4691	ENSG00000115053	validated
tarbase	hsa-miR-326	NCL	4691	ENSG00000115053	validated
tarbase	hsa-miR-212-3p	ACTR1B	10120	ENSG00000115073	validated
tarbase	hsa-miR-296-5p	ACTR1B	10120	ENSG00000115073	validated
tarbase	hsa-miR-326	ACTR1B	10120	ENSG00000115073	validated
tarbase	hsa-miR-15b-5p	SLC35F5	80255	ENSG00000115084	validated
tarbase	hsa-miR-18a-5p	SLC35F5	80255	ENSG00000115084	validated
tarbase	hsa-miR-212-3p	SLC35F5	80255	ENSG00000115084	validated
tarbase	hsa-miR-181a-5p	SLC35F5	80255	ENSG00000115084	validated
tarbase	hsa-miR-15b-5p	ACTR3	10096	ENSG00000115091	validated
tarbase	hsa-miR-181a-5p	ACTR3	10096	ENSG00000115091	validated
tarbase	hsa-miR-18a-5p	STEAP3	55240	ENSG00000115107	validated
tarbase	hsa-miR-222-3p	SF3B6	51639	ENSG00000115128	validated
tarbase	hsa-miR-18a-5p	DNAJC27	51277	ENSG00000115137	validated
tarbase	hsa-miR-296-5p	DNAJC27	51277	ENSG00000115137	validated
tarbase	hsa-miR-18a-5p	STAM2	10254	ENSG00000115145	validated
tarbase	hsa-miR-106b-5p	GPD2	2820	ENSG00000115159	validated
tarbase	hsa-miR-212-3p	CENPA	1058	ENSG00000115163	validated
tarbase	hsa-miR-18a-5p	TANC1	85461	ENSG00000115183	validated
tarbase	hsa-miR-21-5p	TANC1	85461	ENSG00000115183	validated
tarbase	hsa-miR-301a-3p	TANC1	85461	ENSG00000115183	validated
tarbase	hsa-miR-326	TANC1	85461	ENSG00000115183	validated
tarbase	hsa-miR-15b-5p	GTF3C2	2976	ENSG00000115207	validated
tarbase	hsa-miR-301a-3p	GTF3C2	2976	ENSG00000115207	validated
tarbase	hsa-miR-15b-5p	NRBP1	29959	ENSG00000115216	validated
tarbase	hsa-miR-301a-3p	NRBP1	29959	ENSG00000115216	validated
tarbase	hsa-miR-326	SNX17	9784	ENSG00000115234	validated
tarbase	hsa-miR-15b-5p			ENSG00000115239	validated
tarbase	hsa-miR-106b-5p	PPM1G	5496	ENSG00000115241	validated
tarbase	hsa-miR-212-3p	PPM1G	5496	ENSG00000115241	validated
tarbase	hsa-miR-326	PPM1G	5496	ENSG00000115241	validated
tarbase	hsa-miR-212-3p	IFIH1	64135	ENSG00000115267	validated
tarbase	hsa-miR-18a-5p	RPS15	6209	ENSG00000115268	validated
tarbase	hsa-miR-18a-5p	CLIP4	79745	ENSG00000115295	validated
tarbase	hsa-miR-181a-5p	SPTBN1	6711	ENSG00000115306	validated
tarbase	hsa-miR-21-5p	SPTBN1	6711	ENSG00000115306	validated
tarbase	hsa-miR-301a-3p	SPTBN1	6711	ENSG00000115306	validated
tarbase	hsa-miR-326	SPTBN1	6711	ENSG00000115306	validated
tarbase	hsa-miR-582-5p	SPTBN1	6711	ENSG00000115306	validated
tarbase	hsa-miR-15b-5p	AUP1	550	ENSG00000115307	validated
tarbase	hsa-miR-18a-5p	AUP1	550	ENSG00000115307	validated
tarbase	hsa-miR-192-5p	AUP1	550	ENSG00000115307	validated
tarbase	hsa-miR-106b-5p	RTN4	57142	ENSG00000115310	validated
tarbase	hsa-miR-181a-5p	RTN4	57142	ENSG00000115310	validated
tarbase	hsa-miR-212-3p	RTN4	57142	ENSG00000115310	validated
tarbase	hsa-miR-301a-3p	RTN4	57142	ENSG00000115310	validated
tarbase	hsa-miR-582-5p	RTN4	57142	ENSG00000115310	validated
tarbase	hsa-miR-222-3p	HTRA2	27429	ENSG00000115317	validated

tarbase	hsa-miR-296-5p	HTRA2	27429	ENSG00000115317	validated
tarbase	hsa-miR-15b-5p	GALNT3	2591	ENSG00000115339	validated
tarbase	hsa-miR-301a-3p	POLE4	56655	ENSG00000115350	validated
tarbase	hsa-miR-21-5p	CCDC88A	55704	ENSG00000115355	validated
tarbase	hsa-miR-15b-5p	CCDC88A	55704	ENSG00000115355	validated
tarbase	hsa-miR-222-3p	MRPL19	9801	ENSG00000115364	validated
tarbase	hsa-miR-212-3p	WDR75	84128	ENSG00000115368	validated
tarbase	hsa-miR-222-3p	WDR75	84128	ENSG00000115368	validated
tarbase	hsa-miR-15b-5p	EFEMP1	2202	ENSG00000115380	validated
tarbase	hsa-miR-301a-3p	FN1	2335	ENSG00000115414	validated
tarbase	hsa-miR-181a-5p	STAT1	6772	ENSG00000115415	validated
tarbase	hsa-miR-212-3p	STAT1	6772	ENSG00000115415	validated
tarbase	hsa-miR-181a-5p	GLS	2744	ENSG00000115419	validated
tarbase	hsa-miR-21-5p	GLS	2744	ENSG00000115419	validated
tarbase	hsa-miR-505-3p	GLS	2744	ENSG00000115419	validated
tarbase	hsa-miR-15b-5p	GLS	2744	ENSG00000115419	validated
tarbase	hsa-miR-106b-5p	IGFBP5	3488	ENSG00000115461	validated
tarbase	hsa-miR-15b-5p	IGFBP5	3488	ENSG00000115461	validated
tarbase	hsa-miR-212-3p	IGFBP5	3488	ENSG00000115461	validated
tarbase	hsa-miR-21-5p	IGFBP5	3488	ENSG00000115461	validated
tarbase	hsa-miR-181a-5p	USP34	9736	ENSG00000115464	validated
tarbase	hsa-miR-15b-5p	CCT4	10575	ENSG00000115484	validated
tarbase	hsa-miR-192-5p	CCT4	10575	ENSG00000115484	validated
tarbase	hsa-miR-106b-5p	CCT4	10575	ENSG00000115484	validated
tarbase	hsa-miR-106b-5p	EHBP1	23301	ENSG00000115504	validated
tarbase	hsa-miR-192-5p	EHBP1	23301	ENSG00000115504	validated
tarbase	hsa-miR-106b-5p	COQ10B	80219	ENSG00000115520	validated
tarbase	hsa-miR-18a-5p	COQ10B	80219	ENSG00000115520	validated
tarbase	hsa-miR-106b-5p	SF3B1	23451	ENSG00000115524	validated
tarbase	hsa-miR-15b-5p	SF3B1	23451	ENSG00000115524	validated
tarbase	hsa-miR-18a-5p	SF3B1	23451	ENSG00000115524	validated
tarbase	hsa-miR-15b-5p	ST3GAL5	8869	ENSG00000115525	validated
tarbase	hsa-miR-181a-5p	ST3GAL5	8869	ENSG00000115525	validated
tarbase	hsa-miR-301a-3p	ST3GAL5	8869	ENSG00000115525	validated
tarbase	hsa-miR-301a-3p	HSPE1	3336	ENSG00000115541	validated
tarbase	hsa-miR-582-5p	HSPE1	3336	ENSG00000115541	validated
tarbase	hsa-miR-15b-5p	HSPE1	3336	ENSG00000115541	validated
tarbase	hsa-miR-18a-5p	KDM3A	55818	ENSG00000115548	validated
tarbase	hsa-miR-106b-5p	CHMP3	51652	ENSG00000115561	validated
tarbase	hsa-miR-212-3p	IL1R1	3554	ENSG00000115594	validated
tarbase	hsa-miR-15b-5p	SLC9A2	6549	ENSG00000115616	validated
tarbase	hsa-miR-301a-3p	SLC9A2	6549	ENSG00000115616	validated
tarbase	hsa-miR-181a-5p	FHL2	2274	ENSG00000115641	validated
tarbase	hsa-miR-181a-5p	CNPPD1	27013	ENSG00000115649	validated
tarbase	hsa-miR-192-5p	HDLBP	3069	ENSG00000115677	validated
tarbase	hsa-miR-21-5p	HDLBP	3069	ENSG00000115677	validated
tarbase	hsa-miR-296-5p	HDLBP	3069	ENSG00000115677	validated
tarbase	hsa-miR-582-3p	HDLBP	3069	ENSG00000115677	validated
tarbase	hsa-miR-212-3p	STK25	10494	ENSG00000115694	validated
tarbase	hsa-miR-15b-5p	ID2	3398	ENSG00000115738	validated
tarbase	hsa-miR-212-3p	ID2	3398	ENSG00000115738	validated
tarbase	hsa-miR-15b-5p	ODC1	4953	ENSG00000115758	validated
tarbase	hsa-miR-106b-5p	BIRC6	57448	ENSG00000115760	validated
tarbase	hsa-miR-181a-5p	BIRC6	57448	ENSG00000115760	validated
tarbase	hsa-miR-222-3p	BIRC6	57448	ENSG00000115760	validated
tarbase	hsa-miR-301a-3p	BIRC6	57448	ENSG00000115760	validated
tarbase	hsa-miR-505-3p	BIRC6	57448	ENSG00000115760	validated
tarbase	hsa-miR-18a-5p	BIRC6	57448	ENSG00000115760	validated
tarbase	hsa-miR-106b-5p	PLEKHB2	55041	ENSG00000115762	validated
tarbase	hsa-miR-181a-5p	PLEKHB2	55041	ENSG00000115762	validated
tarbase	hsa-miR-301a-3p	PLEKHB2	55041	ENSG00000115762	validated
tarbase	hsa-miR-18a-5p	GORASP2	26003	ENSG00000115806	validated
tarbase	hsa-miR-301a-3p	GORASP2	26003	ENSG00000115806	validated
tarbase	hsa-miR-582-5p	GORASP2	26003	ENSG00000115806	validated
tarbase	hsa-miR-106b-5p	STRN	6801	ENSG00000115808	validated
tarbase	hsa-miR-181a-5p	STRN	6801	ENSG00000115808	validated
tarbase	hsa-miR-21-5p	STRN	6801	ENSG00000115808	validated
tarbase	hsa-miR-222-3p	STRN	6801	ENSG00000115808	validated
tarbase	hsa-miR-21-5p	CEBPZ	10153	ENSG00000115816	validated
tarbase	hsa-miR-301a-3p	PRKD3	23683	ENSG00000115825	validated
tarbase	hsa-miR-181a-5p	DCAF17	80067	ENSG00000115827	validated

tarbase	hsa-miR-18a-5p	DCAF17	80067	ENSG00000115827	validated
tarbase	hsa-miR-15b-5p	QPCT	25797	ENSG00000115828	validated
tarbase	hsa-miR-181a-5p	RAB3GAP1	22930	ENSG00000115839	validated
tarbase	hsa-miR-296-5p	SLC25A12	8604	ENSG00000115840	validated
tarbase	hsa-miR-301a-3p	SLC25A12	8604	ENSG00000115840	validated
tarbase	hsa-miR-21-5p	DLX2	1746	ENSG00000115844	validated
tarbase	hsa-miR-15b-5p	DARS1	1615	ENSG00000115866	validated
tarbase	hsa-miR-181a-5p	DARS1	1615	ENSG00000115866	validated
tarbase	hsa-miR-301a-3p	SRSF7	6432	ENSG00000115875	validated
tarbase	hsa-miR-144-3p	SDC1	6382	ENSG00000115884	validated
tarbase	hsa-miR-222-3p	SDC1	6382	ENSG00000115884	validated
tarbase	hsa-miR-326	SDC1	6382	ENSG00000115884	validated
tarbase	hsa-miR-181a-5p	PLCL1	5334	ENSG00000115896	validated
tarbase	hsa-miR-212-3p	PLCL1	5334	ENSG00000115896	validated
tarbase	hsa-miR-15b-5p	SLC1A4	6509	ENSG00000115902	validated
tarbase	hsa-miR-106b-5p	SOS1	6654	ENSG00000115904	validated
tarbase	hsa-miR-301a-3p	SOS1	6654	ENSG00000115904	validated
tarbase	hsa-miR-181a-5p	SOS1	6654	ENSG00000115904	validated
tarbase	hsa-miR-21-5p	KYNU	8942	ENSG00000115919	validated
tarbase	hsa-miR-18a-5p	ORC2	4999	ENSG00000115942	validated
tarbase	hsa-miR-15b-5p	ORC2	4999	ENSG00000115942	validated
tarbase	hsa-miR-106b-5p	COX7A2L	9167	ENSG00000115944	validated
tarbase	hsa-miR-181a-5p	COX7A2L	9167	ENSG00000115944	validated
tarbase	hsa-miR-106b-5p	RND3	390	ENSG00000115963	validated
tarbase	hsa-miR-21-5p	RND3	390	ENSG00000115963	validated
tarbase	hsa-miR-212-3p	RND3	390	ENSG00000115963	validated
tarbase	hsa-miR-15b-5p	ATF2	1386	ENSG00000115966	validated
tarbase	hsa-miR-222-3p	ATF2	1386	ENSG00000115966	validated
tarbase	hsa-miR-582-5p	ATF2	1386	ENSG00000115966	validated
tarbase	hsa-miR-181a-5p	ATF2	1386	ENSG00000115966	validated
tarbase	hsa-miR-18a-5p	AAK1	22848	ENSG00000115977	validated
tarbase	hsa-miR-301a-3p	AAK1	22848	ENSG00000115977	validated
tarbase	hsa-miR-181a-5p	AAK1	22848	ENSG00000115977	validated
tarbase	hsa-miR-192-5p	TRAK2	66008	ENSG00000115993	validated
tarbase	hsa-miR-181a-5p	TRAK2	66008	ENSG00000115993	validated
tarbase	hsa-miR-106b-5p	TIA1	7072	ENSG00000116001	validated
tarbase	hsa-miR-181a-5p	PCYOX1	51449	ENSG00000116005	validated
tarbase	hsa-miR-106b-5p	EPAS1	2034	ENSG00000116016	validated
tarbase	hsa-miR-326	EPAS1	2034	ENSG00000116016	validated
tarbase	hsa-miR-106b-5p	ARID3A	1820	ENSG00000116017	validated
tarbase	hsa-miR-296-5p	ARID3A	1820	ENSG00000116017	validated
tarbase	hsa-miR-582-5p	ARID3A	1820	ENSG00000116017	validated
tarbase	hsa-miR-18a-5p	SUMO1	7341	ENSG00000116030	validated
tarbase	hsa-miR-192-5p	SUMO1	7341	ENSG00000116030	validated
tarbase	hsa-miR-296-5p	SUMO1	7341	ENSG00000116030	validated
tarbase	hsa-miR-301a-3p	SUMO1	7341	ENSG00000116030	validated
tarbase	hsa-miR-106b-5p	NFE2L2	4780	ENSG00000116044	validated
tarbase	hsa-miR-18a-5p	NFE2L2	4780	ENSG00000116044	validated
tarbase	hsa-miR-505-3p	NFE2L2	4780	ENSG00000116044	validated
tarbase	hsa-miR-212-3p	MSH6	2956	ENSG00000116062	validated
tarbase	hsa-miR-296-5p	MSH6	2956	ENSG00000116062	validated
tarbase	hsa-miR-15b-5p	PLEKHA3	65977	ENSG00000116095	validated
tarbase	hsa-miR-222-3p	PLEKHA3	65977	ENSG00000116095	validated
tarbase	hsa-miR-18a-5p	EPHA4	2043	ENSG00000116106	validated
tarbase	hsa-miR-301a-3p	EPHA4	2043	ENSG00000116106	validated
tarbase	hsa-miR-192-5p	ALMS1	7840	ENSG00000116127	validated
tarbase	hsa-miR-15b-5p	BCL9	607	ENSG00000116128	validated
tarbase	hsa-miR-18a-5p	PRRX1	5396	ENSG00000116132	validated
tarbase	hsa-miR-21-5p	PRRX1	5396	ENSG00000116132	validated
tarbase	hsa-miR-106b-5p	DHCR24	1718	ENSG00000116133	validated
tarbase	hsa-miR-18a-5p	DHCR24	1718	ENSG00000116133	validated
tarbase	hsa-miR-15b-5p	DNAJC16	23341	ENSG00000116138	validated
tarbase	hsa-miR-18a-5p	DNAJC16	23341	ENSG00000116138	validated
tarbase	hsa-miR-106b-5p	MARK1	4139	ENSG00000116141	validated
tarbase	hsa-miR-15b-5p	MARK1	4139	ENSG00000116141	validated
tarbase	hsa-miR-181a-5p	MARK1	4139	ENSG00000116141	validated
tarbase	hsa-miR-222-3p	MARK1	4139	ENSG00000116141	validated
tarbase	hsa-miR-181a-5p	CACYBP	27101	ENSG00000116161	validated
tarbase	hsa-miR-192-5p	RALGPS2	55103	ENSG00000116191	validated
tarbase	hsa-miR-21-5p	CEP104	9731	ENSG00000116198	validated
tarbase	hsa-miR-15b-5p	TMEM59	9528	ENSG00000116209	validated

tarbase	hsa-miR-222-3p	TMEM59	9528	ENSG00000116209	validated
tarbase	hsa-miR-296-5p	LRRC42	115353	ENSG00000116212	validated
tarbase	hsa-miR-15b-5p	ICMT	23463	ENSG00000116237	validated
tarbase	hsa-miR-181a-5p	ICMT	23463	ENSG00000116237	validated
tarbase	hsa-miR-18a-5p	ICMT	23463	ENSG00000116237	validated
tarbase	hsa-miR-192-5p	ICMT	23463	ENSG00000116237	validated
tarbase	hsa-miR-296-5p	RPL22	6146	ENSG00000116251	validated
tarbase	hsa-miR-15b-5p	QSOX1	5768	ENSG00000116260	validated
tarbase	hsa-miR-326	QSOX1	5768	ENSG00000116260	validated
tarbase	hsa-miR-144-3p	PHF13	148479	ENSG00000116273	validated
tarbase	hsa-miR-582-3p	PHF13	148479	ENSG00000116273	validated
tarbase	hsa-miR-582-5p	PHF13	148479	ENSG00000116273	validated
tarbase	hsa-miR-144-3p	ERRFI1	54206	ENSG00000116285	validated
tarbase	hsa-miR-192-5p	KCNC4	3749	ENSG00000116396	validated
tarbase	hsa-miR-296-5p	KCNC4	3749	ENSG00000116396	validated
tarbase	hsa-miR-326	KCNC4	3749	ENSG00000116396	validated
tarbase	hsa-miR-181a-5p	EDEM3	80267	ENSG00000116406	validated
tarbase	hsa-miR-212-3p	EDEM3	80267	ENSG00000116406	validated
tarbase	hsa-miR-301a-3p	RAP1A	5906	ENSG00000116473	validated
tarbase	hsa-miR-21-5p	HDAC1	3065	ENSG00000116478	validated
tarbase	hsa-miR-15b-5p	CAPZA1	829	ENSG00000116489	validated
tarbase	hsa-miR-212-3p	CAPZA1	829	ENSG00000116489	validated
tarbase	hsa-miR-21-5p	CAPZA1	829	ENSG00000116489	validated
tarbase	hsa-miR-582-3p	CAPZA1	829	ENSG00000116489	validated
tarbase	hsa-miR-296-5p	S100PBP	64766	ENSG00000116497	validated
tarbase	hsa-miR-181a-5p	ASH1L	55870	ENSG00000116539	validated
tarbase	hsa-miR-18a-5p	ASH1L	55870	ENSG00000116539	validated
tarbase	hsa-miR-192-5p	ASH1L	55870	ENSG00000116539	validated
tarbase	hsa-miR-21-5p	ASH1L	55870	ENSG00000116539	validated
tarbase	hsa-miR-301a-3p	ASH1L	55870	ENSG00000116539	validated
tarbase	hsa-miR-505-3p	ASH1L	55870	ENSG00000116539	validated
tarbase	hsa-miR-582-3p	ASH1L	55870	ENSG00000116539	validated
tarbase	hsa-miR-15b-5p	SFPQ	6421	ENSG00000116560	validated
tarbase	hsa-miR-18a-5p	SFPQ	6421	ENSG00000116560	validated
tarbase	hsa-miR-192-5p	SFPQ	6421	ENSG00000116560	validated
tarbase	hsa-miR-212-3p	SFPQ	6421	ENSG00000116560	validated
tarbase	hsa-miR-296-5p	SFPQ	6421	ENSG00000116560	validated
tarbase	hsa-miR-505-3p	MEF2D	4209	ENSG00000116604	validated
tarbase	hsa-miR-582-5p	MEF2D	4209	ENSG00000116604	validated
tarbase	hsa-miR-106b-5p	DOCK7	85440	ENSG00000116641	validated
tarbase	hsa-miR-296-5p	C1orf21	81563	ENSG00000116667	validated
tarbase	hsa-miR-106b-5p	C1orf21	81563	ENSG00000116667	validated
tarbase	hsa-miR-296-5p	MAD2L2	10459	ENSG00000116670	validated
tarbase	hsa-miR-181a-5p	DNAJC6	9829	ENSG00000116675	validated
tarbase	hsa-miR-582-3p	IVNS1ABP	10625	ENSG00000116679	validated
tarbase	hsa-miR-582-5p	IVNS1ABP	10625	ENSG00000116679	validated
tarbase	hsa-miR-181a-5p	IVNS1ABP	10625	ENSG00000116679	validated
tarbase	hsa-miR-181a-5p	SMG7	9887	ENSG00000116698	validated
tarbase	hsa-miR-301a-3p	SMG7	9887	ENSG00000116698	validated
tarbase	hsa-miR-326	SMG7	9887	ENSG00000116698	validated
tarbase	hsa-miR-582-3p	SMG7	9887	ENSG00000116698	validated
tarbase	hsa-miR-18a-5p	SMG7	9887	ENSG00000116698	validated
tarbase	hsa-miR-181a-5p	NCF2	4688	ENSG00000116701	validated
tarbase	hsa-miR-222-3p	SLC35D1	23169	ENSG00000116704	validated
tarbase	hsa-miR-301a-3p	SLC35D1	23169	ENSG00000116704	validated
tarbase	hsa-miR-582-3p	GADD45A	1647	ENSG00000116717	validated
tarbase	hsa-miR-212-3p	GADD45A	1647	ENSG00000116717	validated
tarbase	hsa-miR-326	PRDM2	7799	ENSG00000116731	validated
tarbase	hsa-miR-582-5p	PRDM2	7799	ENSG00000116731	validated
tarbase	hsa-miR-106b-5p	RO60	6738	ENSG00000116747	validated
tarbase	hsa-miR-181a-5p	RO60	6738	ENSG00000116747	validated
tarbase	hsa-miR-192-5p	RO60	6738	ENSG00000116747	validated
tarbase	hsa-miR-222-3p	RO60	6738	ENSG00000116747	validated
tarbase	hsa-miR-582-3p	RO60	6738	ENSG00000116747	validated
tarbase	hsa-miR-18a-5p	RO60	6738	ENSG00000116747	validated
tarbase	hsa-miR-106b-5p	UCHL5	51377	ENSG00000116750	validated
tarbase	hsa-miR-21-5p	BCAS2	10286	ENSG00000116752	validated
tarbase	hsa-miR-106b-5p	CTH	1491	ENSG00000116761	validated
tarbase	hsa-miR-15b-5p	PLEKHM2	23207	ENSG00000116786	validated
tarbase	hsa-miR-106b-5p	TTF2	8458	ENSG00000116830	validated
tarbase	hsa-miR-18a-5p	TTF2	8458	ENSG00000116830	validated

tarbase	hsa-miR-212-3p	TTF2	8458	ENSG00000116830	validated
tarbase	hsa-miR-301a-3p	TMEM9	252839	ENSG00000116857	validated
tarbase	hsa-miR-582-5p	MAP7D1	55700	ENSG00000116871	validated
tarbase	hsa-miR-212-3p	OSCP1	127700	ENSG00000116885	validated
tarbase	hsa-miR-181a-5p	EXOC8	149371	ENSG00000116903	validated
tarbase	hsa-miR-106b-5p	TSNAX	7257	ENSG00000116918	validated
tarbase	hsa-miR-505-3p	C1orf109	54955	ENSG00000116922	validated
tarbase	hsa-miR-212-3p	RRAGC	64121	ENSG00000116954	validated
tarbase	hsa-miR-296-5p	NID1	4811	ENSG00000116962	validated
tarbase	hsa-miR-582-5p	NID1	4811	ENSG00000116962	validated
tarbase	hsa-miR-106b-5p	LGALS8	3964	ENSG00000116977	validated
tarbase	hsa-miR-301a-3p	LGALS8	3964	ENSG00000116977	validated
tarbase	hsa-miR-21-5p	LGALS8	3964	ENSG00000116977	validated
tarbase	hsa-miR-181a-5p	MTR	4548	ENSG00000116984	validated
tarbase	hsa-miR-301a-3p	MTR	4548	ENSG00000116984	validated
tarbase	hsa-miR-582-3p	MTR	4548	ENSG00000116984	validated
tarbase	hsa-miR-106b-5p	MYCL	4610	ENSG00000116990	validated
tarbase	hsa-miR-106b-5p	SIPA1L2	57568	ENSG00000116991	validated
tarbase	hsa-miR-15b-5p	SIPA1L2	57568	ENSG00000116991	validated
tarbase	hsa-miR-181a-5p	AKT3	10000	ENSG00000117020	validated
tarbase	hsa-miR-326	AKT3	10000	ENSG00000117020	validated
tarbase	hsa-miR-505-3p	AKT3	10000	ENSG00000117020	validated
tarbase	hsa-miR-18a-5p	ETV3	2117	ENSG00000117036	validated
tarbase	hsa-miR-222-3p	ETV3	2117	ENSG00000117036	validated
tarbase	hsa-miR-326	ADGRL2	23266	ENSG00000117114	validated
tarbase	hsa-miR-582-3p	ADGRL2	23266	ENSG00000117114	validated
tarbase	hsa-miR-15b-5p	MFAP2	4237	ENSG00000117122	validated
tarbase	hsa-miR-106b-5p	KDM5B	10765	ENSG00000117139	validated
tarbase	hsa-miR-18a-5p	KDM5B	10765	ENSG00000117139	validated
tarbase	hsa-miR-212-3p	KDM5B	10765	ENSG00000117139	validated
tarbase	hsa-miR-212-3p	RGS4	5999	ENSG00000117152	validated
tarbase	hsa-miR-181a-5p	RGS4	5999	ENSG00000117152	validated
tarbase	hsa-miR-181a-5p	KLHL12	59349	ENSG00000117153	validated
tarbase	hsa-miR-18a-5p	SSX2IP	117178	ENSG00000117155	validated
tarbase	hsa-miR-582-5p	SSX2IP	117178	ENSG00000117155	validated
tarbase	hsa-miR-181a-5p	GBP3	2635	ENSG00000117226	validated
tarbase	hsa-miR-212-3p	GBP1	2633	ENSG00000117228	validated
tarbase	hsa-miR-15b-5p	RAB29	8934	ENSG00000117280	validated
tarbase	hsa-miR-15b-5p	ID3	3399	ENSG00000117318	validated
tarbase	hsa-miR-296-5p	ID3	3399	ENSG00000117318	validated
tarbase	hsa-miR-212-3p	ID3	3399	ENSG00000117318	validated
tarbase	hsa-miR-582-5p	CR2	1380	ENSG00000117322	validated
tarbase	hsa-miR-106b-5p	CD46	4179	ENSG00000117335	validated
tarbase	hsa-miR-15b-5p	CD46	4179	ENSG00000117335	validated
tarbase	hsa-miR-106b-5p	APH1A	51107	ENSG00000117362	validated
tarbase	hsa-miR-15b-5p	APH1A	51107	ENSG00000117362	validated
tarbase	hsa-miR-18a-5p	APH1A	51107	ENSG00000117362	validated
tarbase	hsa-miR-21-5p	APH1A	51107	ENSG00000117362	validated
tarbase	hsa-miR-301a-3p	APH1A	51107	ENSG00000117362	validated
tarbase	hsa-miR-15b-5p	IPO13	9670	ENSG00000117408	validated
tarbase	hsa-miR-181a-5p	IPO13	9670	ENSG00000117408	validated
tarbase	hsa-miR-15b-5p	ATP6V0B	533	ENSG00000117410	validated
tarbase	hsa-miR-15b-5p	AKR1A1	10327	ENSG00000117448	validated
tarbase	hsa-miR-21-5p	AKR1A1	10327	ENSG00000117448	validated
tarbase	hsa-miR-18a-5p	PRDX1	5052	ENSG00000117450	validated
tarbase	hsa-miR-181a-5p	PIK3R3	8503	ENSG00000117461	validated
tarbase	hsa-miR-582-5p	PIK3R3	8503	ENSG00000117461	validated
tarbase	hsa-miR-212-3p	PIK3R3	8503	ENSG00000117461	validated
tarbase	hsa-miR-15b-5p	SLC19A2	10560	ENSG00000117479	validated
tarbase	hsa-miR-212-3p	TMED5	50999	ENSG00000117500	validated
tarbase	hsa-miR-15b-5p	DR1	1810	ENSG00000117505	validated
tarbase	hsa-miR-18a-5p	DR1	1810	ENSG00000117505	validated
tarbase	hsa-miR-181a-5p	DR1	1810	ENSG00000117505	validated
tarbase	hsa-miR-106b-5p	PRRC2C	23215	ENSG00000117523	validated
tarbase	hsa-miR-192-5p	PRRC2C	23215	ENSG00000117523	validated
tarbase	hsa-miR-21-5p	PRRC2C	23215	ENSG00000117523	validated
tarbase	hsa-miR-181a-5p	F3	2152	ENSG00000117525	validated
tarbase	hsa-miR-106b-5p	DPH5	51611	ENSG00000117543	validated
tarbase	hsa-miR-301a-3p	DPH5	51611	ENSG00000117543	validated
tarbase	hsa-miR-181a-5p	PTBP2	58155	ENSG00000117569	validated
tarbase	hsa-miR-212-3p	PTBP2	58155	ENSG00000117569	validated

tarbase	hsa-miR-212-3p	TNFSF4	7292	ENSG00000117586	validated
tarbase	hsa-miR-181a-5p	PRDX6	9588	ENSG00000117592	validated
tarbase	hsa-miR-301a-3p	IRF6	3664	ENSG00000117595	validated
tarbase	hsa-miR-15b-5p	UTP25	27042	ENSG00000117597	validated
tarbase	hsa-miR-181a-5p	UTP25	27042	ENSG00000117597	validated
tarbase	hsa-miR-192-5p	UTP25	27042	ENSG00000117597	validated
tarbase	hsa-miR-181a-5p	PLPPR4	9890	ENSG00000117600	validated
tarbase	hsa-miR-181a-5p	SLC35A3	23443	ENSG00000117620	validated
tarbase	hsa-miR-181a-5p	RCOR3	55758	ENSG00000117625	validated
tarbase	hsa-miR-212-3p	RCOR3	55758	ENSG00000117625	validated
tarbase	hsa-miR-181a-5p	STMN1	3925	ENSG00000117632	validated
tarbase	hsa-miR-18a-5p	STMN1	3925	ENSG00000117632	validated
tarbase	hsa-miR-21-5p	STMN1	3925	ENSG00000117632	validated
tarbase	hsa-miR-106b-5p	MAN1C1	57134	ENSG00000117643	validated
tarbase	hsa-miR-106b-5p	DHDDS	79947	ENSG00000117682	validated
tarbase	hsa-miR-18a-5p	DHDDS	79947	ENSG00000117682	validated
tarbase	hsa-miR-582-3p	DHDDS	79947	ENSG00000117682	validated
tarbase	hsa-miR-212-3p	ARID1A	8289	ENSG00000117713	validated
tarbase	hsa-miR-582-5p	ARID1A	8289	ENSG00000117713	validated
tarbase	hsa-miR-181a-5p	ARID1A	8289	ENSG00000117713	validated
tarbase	hsa-miR-106b-5p	CENPF	1063	ENSG00000117724	validated
tarbase	hsa-miR-181a-5p	CENPF	1063	ENSG00000117724	validated
tarbase	hsa-miR-301a-3p	CENPF	1063	ENSG00000117724	validated
tarbase	hsa-miR-212-3p	CENPF	1063	ENSG00000117724	validated
tarbase	hsa-miR-15b-5p	RPA2	6118	ENSG00000117748	validated
tarbase	hsa-miR-301a-3p	RPA2	6118	ENSG00000117748	validated
tarbase	hsa-miR-326	PPP1R8	5511	ENSG00000117751	validated
tarbase	hsa-miR-181a-5p	STX12	23673	ENSG00000117758	validated
tarbase	hsa-miR-212-3p	STX12	23673	ENSG00000117758	validated
tarbase	hsa-miR-582-5p	STX12	23673	ENSG00000117758	validated
tarbase	hsa-miR-106b-5p	OSBPL9	114883	ENSG00000117859	validated
tarbase	hsa-miR-181a-5p	TXNDC12	51060	ENSG00000117862	validated
tarbase	hsa-miR-18a-5p	TXNDC12	51060	ENSG00000117862	validated
tarbase	hsa-miR-582-5p	TXNDC12	51060	ENSG00000117862	validated
tarbase	hsa-miR-326	MESD	23184	ENSG00000117899	validated
tarbase	hsa-miR-181a-5p	CTSD	1509	ENSG00000117984	validated
tarbase	hsa-miR-296-5p	CTSD	1509	ENSG00000117984	validated
tarbase	hsa-miR-106b-5p	STK11	6794	ENSG00000118046	validated
tarbase	hsa-miR-15b-5p	STK11	6794	ENSG00000118046	validated
tarbase	hsa-miR-15b-5p	KMT2A	4297	ENSG00000118058	validated
tarbase	hsa-miR-181a-5p	KMT2A	4297	ENSG00000118058	validated
tarbase	hsa-miR-18a-5p	KMT2A	4297	ENSG00000118058	validated
tarbase	hsa-miR-21-5p	KMT2A	4297	ENSG00000118058	validated
tarbase	hsa-miR-301a-3p	KMT2A	4297	ENSG00000118058	validated
tarbase	hsa-miR-212-3p	TREH	11181	ENSG00000118094	validated
tarbase	hsa-miR-15b-5p	RPS25	6230	ENSG00000118181	validated
tarbase	hsa-miR-582-5p	RPS25	6230	ENSG00000118181	validated
tarbase	hsa-miR-106b-5p	KIF14	9928	ENSG00000118193	validated
tarbase	hsa-miR-15b-5p	KIF14	9928	ENSG00000118193	validated
tarbase	hsa-miR-18a-5p	KIF14	9928	ENSG00000118193	validated
tarbase	hsa-miR-301a-3p	KIF14	9928	ENSG00000118193	validated
tarbase	hsa-miR-212-3p	KIF14	9928	ENSG00000118193	validated
tarbase	hsa-miR-106b-5p	CAMSAP2	23271	ENSG00000118200	validated
tarbase	hsa-miR-144-3p	CAMSAP2	23271	ENSG00000118200	validated
tarbase	hsa-miR-15b-5p	CAMSAP2	23271	ENSG00000118200	validated
tarbase	hsa-miR-181a-5p	CAMSAP2	23271	ENSG00000118200	validated
tarbase	hsa-miR-15b-5p	ATF6	22926	ENSG00000118217	validated
tarbase	hsa-miR-106b-5p	FASTKD2	22868	ENSG00000118246	validated
tarbase	hsa-miR-15b-5p	CREB1	1385	ENSG00000118260	validated
tarbase	hsa-miR-181a-5p	CREB1	1385	ENSG00000118260	validated
tarbase	hsa-miR-15b-5p	KLF7	8609	ENSG00000118263	validated
tarbase	hsa-miR-212-3p	KLF7	8609	ENSG00000118263	validated
tarbase	hsa-miR-21-5p	KLF7	8609	ENSG00000118263	validated
tarbase	hsa-miR-181a-5p	B4GALT6	9331	ENSG00000118276	validated
tarbase	hsa-miR-582-5p	C1orf54	79630	ENSG00000118292	validated
tarbase	hsa-miR-212-3p	CASC1	55259	ENSG00000118307	validated
tarbase	hsa-miR-212-3p	FILIP1	27145	ENSG00000118407	validated
tarbase	hsa-miR-212-3p	CASP8AP2	9994	ENSG00000118412	validated
tarbase	hsa-miR-21-5p	HMGN3	9324	ENSG00000118418	validated
tarbase	hsa-miR-18a-5p	ANKRD13C	81573	ENSG00000118454	validated
tarbase	hsa-miR-301a-3p	ANKRD13C	81573	ENSG00000118454	validated

tarbase	hsa-miR-15b-5p	PHF3	23469	ENSG00000118482	validated
tarbase	hsa-miR-181a-5p	PHF3	23469	ENSG00000118482	validated
tarbase	hsa-miR-212-3p	PHF3	23469	ENSG00000118482	validated
tarbase	hsa-miR-106b-5p	FBXO30	84085	ENSG00000118496	validated
tarbase	hsa-miR-144-3p	FBXO30	84085	ENSG00000118496	validated
tarbase	hsa-miR-181a-5p	FBXO30	84085	ENSG00000118496	validated
tarbase	hsa-miR-18a-5p	FBXO30	84085	ENSG00000118496	validated
tarbase	hsa-miR-301a-3p	FBXO30	84085	ENSG00000118496	validated
tarbase	hsa-miR-106b-5p	TNFAIP3	7128	ENSG00000118503	validated
tarbase	hsa-miR-212-3p	TNFAIP3	7128	ENSG00000118503	validated
tarbase	hsa-miR-15b-5p	MYB	4602	ENSG00000118513	validated
tarbase	hsa-miR-15b-5p	SGK1	6446	ENSG00000118515	validated
tarbase	hsa-miR-505-3p	SGK1	6446	ENSG00000118515	validated
tarbase	hsa-miR-181a-5p	RNF146	81847	ENSG00000118518	validated
tarbase	hsa-miR-181a-5p	CCN2	1490	ENSG00000118523	validated
tarbase	hsa-miR-192-5p	CCN2	1490	ENSG00000118523	validated
tarbase	hsa-miR-212-3p	CCN2	1490	ENSG00000118523	validated
tarbase	hsa-miR-21-5p	FBXL5	26234	ENSG00000118564	validated
tarbase	hsa-miR-301a-3p	FBXL5	26234	ENSG00000118564	validated
tarbase	hsa-miR-106b-5p	MED28	80306	ENSG00000118579	validated
tarbase	hsa-miR-18a-5p	MED28	80306	ENSG00000118579	validated
tarbase	hsa-miR-21-5p	MED28	80306	ENSG00000118579	validated
tarbase	hsa-miR-301a-3p	SLC16A7	9194	ENSG00000118596	validated
tarbase	hsa-miR-18a-5p	ZNF430	80264	ENSG00000118620	validated
tarbase	hsa-miR-15b-5p	VAMP8	8673	ENSG00000118640	validated
tarbase	hsa-miR-18a-5p	DCLRE1B	64858	ENSG00000118655	validated
tarbase	hsa-miR-212-3p	ARMC2	84071	ENSG00000118690	validated
tarbase	hsa-miR-181a-5p	RPN2	6185	ENSG00000118705	validated
tarbase	hsa-miR-15b-5p	TGIF2	60436	ENSG00000118707	validated
tarbase	hsa-miR-18a-5p	TGIF2	60436	ENSG00000118707	validated
tarbase	hsa-miR-582-5p	TGIF2	60436	ENSG00000118707	validated
tarbase	hsa-miR-181a-5p	PKD2	5311	ENSG00000118762	validated
tarbase	hsa-miR-18a-5p	PKD2	5311	ENSG00000118762	validated
tarbase	hsa-miR-181a-5p	SPP1	6696	ENSG00000118785	validated
tarbase	hsa-miR-181a-5p	STBD1	8987	ENSG00000118804	validated
tarbase	hsa-miR-106b-5p	CCNI	10983	ENSG00000118816	validated
tarbase	hsa-miR-181a-5p	CCNI	10983	ENSG00000118816	validated
tarbase	hsa-miR-18a-5p	CCNI	10983	ENSG00000118816	validated
tarbase	hsa-miR-21-5p	CCNI	10983	ENSG00000118816	validated
tarbase	hsa-miR-21-5p	RAB3GAP2	25782	ENSG00000118873	validated
tarbase	hsa-miR-296-5p	RAB3GAP2	25782	ENSG00000118873	validated
tarbase	hsa-miR-301a-3p	RAB3GAP2	25782	ENSG00000118873	validated
tarbase	hsa-miR-582-3p	PPL	5493	ENSG00000118898	validated
tarbase	hsa-miR-21-5p	UBN1	29855	ENSG00000118900	validated
tarbase	hsa-miR-106b-5p	KLF12	11278	ENSG00000118922	validated
tarbase	hsa-miR-582-5p	KLF12	11278	ENSG00000118922	validated
tarbase	hsa-miR-212-3p	KLF12	11278	ENSG00000118922	validated
tarbase	hsa-miR-192-5p	UCHL3	7347	ENSG00000118939	validated
tarbase	hsa-miR-21-5p	UCHL3	7347	ENSG00000118939	validated
tarbase	hsa-miR-21-5p	PCDH17	27253	ENSG00000118946	validated
tarbase	hsa-miR-181a-5p	PCDH17	27253	ENSG00000118946	validated
tarbase	hsa-miR-296-5p	LDAH	60526	ENSG00000118961	validated
tarbase	hsa-miR-144-3p	CCND2	894	ENSG00000118971	validated
tarbase	hsa-miR-18a-5p	CCND2	894	ENSG00000118971	validated
tarbase	hsa-miR-21-5p	CCND2	894	ENSG00000118971	validated
tarbase	hsa-miR-326	CCND2	894	ENSG00000118971	validated
tarbase	hsa-miR-582-3p	CCND2	894	ENSG00000118971	validated
tarbase	hsa-miR-582-5p	CCND2	894	ENSG00000118971	validated
tarbase	hsa-miR-15b-5p	ELL2	22936	ENSG00000118985	validated
tarbase	hsa-miR-212-3p	DNAH7	56171	ENSG00000118997	validated
tarbase	hsa-miR-106b-5p	CYP20A1	57404	ENSG00000119004	validated
tarbase	hsa-miR-181a-5p	SATB2	23314	ENSG00000119042	validated
tarbase	hsa-miR-18a-5p	SATB2	23314	ENSG00000119042	validated
tarbase	hsa-miR-106b-5p	UBE2B	7320	ENSG00000119048	validated
tarbase	hsa-miR-181a-5p	UBE2B	7320	ENSG00000119048	validated
tarbase	hsa-miR-18a-5p	UBE2B	7320	ENSG00000119048	validated
tarbase	hsa-miR-181a-5p	GDA	9615	ENSG00000119125	validated
tarbase	hsa-miR-505-3p	KLF9	687	ENSG00000119138	validated
tarbase	hsa-miR-15b-5p	SENP5	205564	ENSG00000119231	validated
tarbase	hsa-miR-192-5p	SENP5	205564	ENSG00000119231	validated
tarbase	hsa-miR-212-3p	SENP5	205564	ENSG00000119231	validated

tarbase	hsa-miR-106b-5p	C1orf198	84886	ENSG00000119280	validated
tarbase	hsa-miR-296-5p	C1orf198	84886	ENSG00000119280	validated
tarbase	hsa-miR-301a-3p	HEATR1	55127	ENSG00000119285	validated
tarbase	hsa-miR-18a-5p	PTBP3	9991	ENSG00000119314	validated
tarbase	hsa-miR-301a-3p	PTBP3	9991	ENSG00000119314	validated
tarbase	hsa-miR-582-3p	PTBP3	9991	ENSG00000119314	validated
tarbase	hsa-miR-181a-5p	RAD23B	5887	ENSG00000119318	validated
tarbase	hsa-miR-582-3p	RAD23B	5887	ENSG00000119318	validated
tarbase	hsa-miR-212-3p	CTNNAL1	8727	ENSG00000119326	validated
tarbase	hsa-miR-212-3p	ABITRAM	54942	ENSG00000119328	validated
tarbase	hsa-miR-106b-5p	PTPA	5524	ENSG00000119383	validated
tarbase	hsa-miR-15b-5p	PTPA	5524	ENSG00000119383	validated
tarbase	hsa-miR-192-5p	PTPA	5524	ENSG00000119383	validated
tarbase	hsa-miR-15b-5p	RAB14	51552	ENSG00000119396	validated
tarbase	hsa-miR-18a-5p	RAB14	51552	ENSG00000119396	validated
tarbase	hsa-miR-192-5p	RAB14	51552	ENSG00000119396	validated
tarbase	hsa-miR-222-3p	TRIM32	22954	ENSG00000119401	validated
tarbase	hsa-miR-15b-5p	FBXW2	26190	ENSG00000119402	validated
tarbase	hsa-miR-18a-5p	PHF19	26147	ENSG00000119403	validated
tarbase	hsa-miR-181a-5p	PPP6C	5537	ENSG00000119414	validated
tarbase	hsa-miR-21-5p	PPP6C	5537	ENSG00000119414	validated
tarbase	hsa-miR-582-3p	NDUFA8	4702	ENSG00000119421	validated
tarbase	hsa-miR-144-3p	HSDL2	84263	ENSG00000119471	validated
tarbase	hsa-miR-15b-5p	HSDL2	84263	ENSG00000119471	validated
tarbase	hsa-miR-15b-5p	MAPKAP1	79109	ENSG00000119487	validated
tarbase	hsa-miR-301a-3p	MAPKAP1	79109	ENSG00000119487	validated
tarbase	hsa-miR-106b-5p	ALG2	85365	ENSG00000119523	validated
tarbase	hsa-miR-15b-5p	KDSR	2531	ENSG00000119537	validated
tarbase	hsa-miR-18a-5p	KDSR	2531	ENSG00000119537	validated
tarbase	hsa-miR-582-5p	KDSR	2531	ENSG00000119537	validated
tarbase	hsa-miR-15b-5p	ONECUT2	9480	ENSG00000119547	validated
tarbase	hsa-miR-181a-5p	ONECUT2	9480	ENSG00000119547	validated
tarbase	hsa-miR-192-5p	C19orf25	148223	ENSG00000119559	validated
tarbase	hsa-miR-15b-5p	ZBTB45	84878	ENSG00000119574	validated
tarbase	hsa-miR-18a-5p	YLPM1	56252	ENSG00000119596	validated
tarbase	hsa-miR-212-3p	BBOF1	80127	ENSG00000119636	validated
tarbase	hsa-miR-212-3p	ACYP1	97	ENSG00000119640	validated
tarbase	hsa-miR-18a-5p	DNAL1	83544	ENSG00000119661	validated
tarbase	hsa-miR-15b-5p	IRF2BPL	64207	ENSG00000119669	validated
tarbase	hsa-miR-21-5p	IRF2BPL	64207	ENSG00000119669	validated
tarbase	hsa-miR-582-5p	IRF2BPL	64207	ENSG00000119669	validated
tarbase	hsa-miR-212-3p	IRF2BPL	64207	ENSG00000119669	validated
tarbase	hsa-miR-106b-5p	AREL1	9870	ENSG00000119682	validated
tarbase	hsa-miR-15b-5p	AREL1	9870	ENSG00000119682	validated
tarbase	hsa-miR-181a-5p	AREL1	9870	ENSG00000119682	validated
tarbase	hsa-miR-181a-5p	MLH3	27030	ENSG00000119684	validated
tarbase	hsa-miR-15b-5p	FLVCR2	55640	ENSG00000119686	validated
tarbase	hsa-miR-181a-5p	DLST	1743	ENSG00000119689	validated
tarbase	hsa-miR-144-3p	RBM25	58517	ENSG00000119707	validated
tarbase	hsa-miR-15b-5p	RBM25	58517	ENSG00000119707	validated
tarbase	hsa-miR-301a-3p	RBM25	58517	ENSG00000119707	validated
tarbase	hsa-miR-326	RBM25	58517	ENSG00000119707	validated
tarbase	hsa-miR-326	KLHL29	114818	ENSG00000119771	validated
tarbase	hsa-miR-181a-5p	DNMT3A	1788	ENSG00000119772	validated
tarbase	hsa-miR-106b-5p	TMEM214	54867	ENSG00000119777	validated
tarbase	hsa-miR-15b-5p	TMEM214	54867	ENSG00000119777	validated
tarbase	hsa-miR-181a-5p	ATAD2B	54454	ENSG00000119778	validated
tarbase	hsa-miR-301a-3p	ATAD2B	54454	ENSG00000119778	validated
tarbase	hsa-miR-212-3p	ATAD2B	54454	ENSG00000119778	validated
tarbase	hsa-miR-144-3p	ATL2	64225	ENSG00000119787	validated
tarbase	hsa-miR-181a-5p	ATL2	64225	ENSG00000119787	validated
tarbase	hsa-miR-18a-5p	ATL2	64225	ENSG00000119787	validated
tarbase	hsa-miR-582-5p	ATL2	64225	ENSG00000119787	validated
tarbase	hsa-miR-212-3p	ATL2	64225	ENSG00000119787	validated
tarbase	hsa-miR-18a-5p	YPEL5	51646	ENSG00000119801	validated
tarbase	hsa-miR-15b-5p	FAM98A	25940	ENSG00000119812	validated
tarbase	hsa-miR-181a-5p	FAM98A	25940	ENSG00000119812	validated
tarbase	hsa-miR-181a-5p	YIPF4	84272	ENSG00000119820	validated
tarbase	hsa-miR-326	AFTPH	54812	ENSG00000119844	validated
tarbase	hsa-miR-106b-5p	LGALSL	29094	ENSG00000119862	validated
tarbase	hsa-miR-301a-3p	LGALSL	29094	ENSG00000119862	validated

tarbase	hsa-miR-21-5p	BCL11A	53335	ENSG00000119866	validated
tarbase	hsa-miR-301a-3p	BCL11A	53335	ENSG00000119866	validated
tarbase	hsa-miR-106b-5p	CRIPT	9419	ENSG00000119878	validated
tarbase	hsa-miR-106b-5p	SLC17A5	26503	ENSG00000119899	validated
tarbase	hsa-miR-15b-5p	SLC17A5	26503	ENSG00000119899	validated
tarbase	hsa-miR-106b-5p	OGFRL1	79627	ENSG00000119900	validated
tarbase	hsa-miR-15b-5p	OGFRL1	79627	ENSG00000119900	validated
tarbase	hsa-miR-181a-5p	OGFRL1	79627	ENSG00000119900	validated
tarbase	hsa-miR-222-3p	SLF2	55719	ENSG00000119906	validated
tarbase	hsa-miR-301a-3p	SLF2	55719	ENSG00000119906	validated
tarbase	hsa-miR-582-5p	IDE	3416	ENSG00000119912	validated
tarbase	hsa-miR-212-3p	ELOVL3	83401	ENSG00000119915	validated
tarbase	hsa-miR-15b-5p	IFIT3	3437	ENSG00000119917	validated
tarbase	hsa-miR-212-3p	IFIT3	3437	ENSG00000119917	validated
tarbase	hsa-miR-222-3p	IFIT2	3433	ENSG00000119922	validated
tarbase	hsa-miR-212-3p	IFIT2	3433	ENSG00000119922	validated
tarbase	hsa-miR-15b-5p	GPAM	57678	ENSG00000119927	validated
tarbase	hsa-miR-212-3p	PYROXD2	84795	ENSG00000119943	validated
tarbase	hsa-miR-181a-5p	SMNDC1	10285	ENSG00000119953	validated
tarbase	hsa-miR-18a-5p	C10orf88	80007	ENSG00000119965	validated
tarbase	hsa-miR-106b-5p	C10orf88	80007	ENSG00000119965	validated
tarbase	hsa-miR-15b-5p	HELLS	3070	ENSG00000119969	validated
tarbase	hsa-miR-181a-5p	HELLS	3070	ENSG00000119969	validated
tarbase	hsa-miR-582-5p	HELLS	3070	ENSG00000119969	validated
tarbase	hsa-miR-181a-5p	TCTN3	26123	ENSG00000119977	validated
tarbase	hsa-miR-326	WDR11	55717	ENSG00000120008	validated
tarbase	hsa-miR-106b-5p	ARMH3	79591	ENSG00000120029	validated
tarbase	hsa-miR-106b-5p	GNA13	10672	ENSG00000120063	validated
tarbase	hsa-miR-15b-5p	GNA13	10672	ENSG00000120063	validated
tarbase	hsa-miR-181a-5p	GNA13	10672	ENSG00000120063	validated
tarbase	hsa-miR-212-3p	GNA13	10672	ENSG00000120063	validated
tarbase	hsa-miR-18a-5p	KANSL1	284058	ENSG00000120071	validated
tarbase	hsa-miR-301a-3p	KANSL1	284058	ENSG00000120071	validated
tarbase	hsa-miR-192-5p	HOXB5	3215	ENSG00000120075	validated
tarbase	hsa-miR-106b-5p	HOXB3	3213	ENSG00000120093	validated
tarbase	hsa-miR-582-3p	HOXB3	3213	ENSG00000120093	validated
tarbase	hsa-miR-18a-5p	DUSP1	1843	ENSG00000120129	validated
tarbase	hsa-miR-144-3p	PANK3	79646	ENSG00000120137	validated
tarbase	hsa-miR-181a-5p	PANK3	79646	ENSG00000120137	validated
tarbase	hsa-miR-18a-5p	PANK3	79646	ENSG00000120137	validated
tarbase	hsa-miR-212-3p	PANK3	79646	ENSG00000120137	validated
tarbase	hsa-miR-21-5p	PANK3	79646	ENSG00000120137	validated
tarbase	hsa-miR-301a-3p	PANK3	79646	ENSG00000120137	validated
tarbase	hsa-miR-582-5p	PANK3	79646	ENSG00000120137	validated
tarbase	hsa-miR-21-5p	TEK	7010	ENSG00000120156	validated
tarbase	hsa-miR-181a-5p	TEK	7010	ENSG00000120156	validated
tarbase	hsa-miR-15b-5p	CAAP1	79886	ENSG00000120159	validated
tarbase	hsa-miR-15b-5p	MOB3B	79817	ENSG00000120162	validated
tarbase	hsa-miR-106b-5p	MOB3B	79817	ENSG00000120162	validated
tarbase	hsa-miR-106b-5p	CD274	29126	ENSG00000120217	validated
tarbase	hsa-miR-301a-3p	CD274	29126	ENSG00000120217	validated
tarbase	hsa-miR-181a-5p	GRIA2	2891	ENSG00000120251	validated
tarbase	hsa-miR-181a-5p	NUP43	348995	ENSG00000120253	validated
tarbase	hsa-miR-222-3p	NUP43	348995	ENSG00000120253	validated
tarbase	hsa-miR-582-3p	NUP43	348995	ENSG00000120253	validated
tarbase	hsa-miR-582-5p	NUP43	348995	ENSG00000120253	validated
tarbase	hsa-miR-181a-5p	MTHFD1L	25902	ENSG00000120254	validated
tarbase	hsa-miR-181a-5p	LRP11	84918	ENSG00000120256	validated
tarbase	hsa-miR-106b-5p	PCMT1	5110	ENSG00000120265	validated
tarbase	hsa-miR-21-5p	PCMT1	5110	ENSG00000120265	validated
tarbase	hsa-miR-18a-5p	PLEKHG1	57480	ENSG00000120278	validated
tarbase	hsa-miR-21-5p	CXorf21	80231	ENSG00000120280	validated
tarbase	hsa-miR-296-5p	MRPS14	63931	ENSG00000120333	validated
tarbase	hsa-miR-326	CENPL	91687	ENSG00000120334	validated
tarbase	hsa-miR-212-3p	CENPL	91687	ENSG00000120334	validated
tarbase	hsa-miR-212-3p	GORAB	92344	ENSG00000120370	validated
tarbase	hsa-miR-181a-5p	TCP1	6950	ENSG00000120438	validated
tarbase	hsa-miR-15b-5p	SLC10A7	84068	ENSG00000120519	validated
tarbase	hsa-miR-15b-5p	NUDCD1	84955	ENSG00000120526	validated
tarbase	hsa-miR-181a-5p	ENY2	56943	ENSG00000120533	validated
tarbase	hsa-miR-582-5p	MASTL	84930	ENSG00000120539	validated

tarbase	hsa-miR-106b-5p	PLXDC2	84898	ENSG00000120594	validated
tarbase	hsa-miR-15b-5p	EPC1	80314	ENSG00000120616	validated
tarbase	hsa-miR-181a-5p	EPC1	80314	ENSG00000120616	validated
tarbase	hsa-miR-301a-3p	EPC1	80314	ENSG00000120616	validated
tarbase	hsa-miR-106b-5p	PROSER1	80209	ENSG00000120685	validated
tarbase	hsa-miR-18a-5p	PROSER1	80209	ENSG00000120685	validated
tarbase	hsa-miR-212-3p	PROSER1	80209	ENSG00000120685	validated
tarbase	hsa-miR-181a-5p	PROSER1	80209	ENSG00000120685	validated
tarbase	hsa-miR-212-3p	ELF1	1997	ENSG00000120690	validated
tarbase	hsa-miR-222-3p	ELF1	1997	ENSG00000120690	validated
tarbase	hsa-miR-144-3p	HSPH1	10808	ENSG00000120694	validated
tarbase	hsa-miR-15b-5p	HSPH1	10808	ENSG00000120694	validated
tarbase	hsa-miR-18a-5p	HSPH1	10808	ENSG00000120694	validated
tarbase	hsa-miR-212-3p	HSPH1	10808	ENSG00000120694	validated
tarbase	hsa-miR-505-3p	HSPH1	10808	ENSG00000120694	validated
tarbase	hsa-miR-181a-5p	KBTBD7	84078	ENSG00000120696	validated
tarbase	hsa-miR-181a-5p	ETF1	2107	ENSG00000120705	validated
tarbase	hsa-miR-326	ETF1	2107	ENSG00000120705	validated
tarbase	hsa-miR-582-3p	ETF1	2107	ENSG00000120705	validated
tarbase	hsa-miR-212-3p	TGFBI	7045	ENSG00000120708	validated
tarbase	hsa-miR-21-5p	FAM53C	51307	ENSG00000120709	validated
tarbase	hsa-miR-181a-5p	SIL1	64374	ENSG00000120725	validated
tarbase	hsa-miR-212-3p	PAIP2	51247	ENSG00000120727	validated
tarbase	hsa-miR-21-5p	PAIP2	51247	ENSG00000120727	validated
tarbase	hsa-miR-106b-5p	EGR1	1958	ENSG00000120738	validated
tarbase	hsa-miR-21-5p	EGR1	1958	ENSG00000120738	validated
tarbase	hsa-miR-106b-5p	SERP1	27230	ENSG00000120742	validated
tarbase	hsa-miR-212-3p	SERP1	27230	ENSG00000120742	validated
tarbase	hsa-miR-181a-5p	SERP1	27230	ENSG00000120742	validated
tarbase	hsa-miR-582-5p	PLS1	5357	ENSG00000120756	validated
tarbase	hsa-miR-181a-5p	ZFP30	22835	ENSG00000120784	validated
tarbase	hsa-miR-106b-5p	NR2C1	7181	ENSG00000120798	validated
tarbase	hsa-miR-301a-3p	NR2C1	7181	ENSG00000120798	validated
tarbase	hsa-miR-181a-5p	UTP20	27340	ENSG00000120800	validated
tarbase	hsa-miR-181a-5p	TMPO	7112	ENSG00000120802	validated
tarbase	hsa-miR-18a-5p	TMPO	7112	ENSG00000120802	validated
tarbase	hsa-miR-21-5p	TMPO	7112	ENSG00000120802	validated
tarbase	hsa-miR-212-3p	ARL1	400	ENSG00000120805	validated
tarbase	hsa-miR-21-5p	ARL1	400	ENSG00000120805	validated
tarbase	hsa-miR-181a-5p	ARL1	400	ENSG00000120805	validated
tarbase	hsa-miR-222-3p	NFYB	4801	ENSG00000120837	validated
tarbase	hsa-miR-106b-5p	APAF1	317	ENSG00000120868	validated
tarbase	hsa-miR-181a-5p	APAF1	317	ENSG00000120868	validated
tarbase	hsa-miR-181a-5p	DUSP4	1846	ENSG00000120875	validated
tarbase	hsa-miR-15b-5p	TNFRSF10B	8795	ENSG00000120889	validated
tarbase	hsa-miR-212-3p	TNFRSF10B	8795	ENSG00000120889	validated
tarbase	hsa-miR-15b-5p	SORBS3	10174	ENSG00000120896	validated
tarbase	hsa-miR-181a-5p	SORBS3	10174	ENSG00000120896	validated
tarbase	hsa-miR-18a-5p	SORBS3	10174	ENSG00000120896	validated
tarbase	hsa-miR-106b-5p	PPP3CC	5533	ENSG00000120910	validated
tarbase	hsa-miR-212-3p	NPPB	4879	ENSG00000120937	validated
tarbase	hsa-miR-106b-5p	UBIAD1	29914	ENSG00000120942	validated
tarbase	hsa-miR-181a-5p	TARDBP	23435	ENSG00000120948	validated
tarbase	hsa-miR-212-3p	TARDBP	23435	ENSG00000120948	validated
tarbase	hsa-miR-301a-3p	TARDBP	23435	ENSG00000120948	validated
tarbase	hsa-miR-296-5p	TNFRSF8	943	ENSG00000120949	validated
tarbase	hsa-miR-181a-5p	LYPLA1	10434	ENSG00000120992	validated
tarbase	hsa-miR-18a-5p	CRISPLD1	83690	ENSG00000121005	validated
tarbase	hsa-miR-21-5p	COPS5	10987	ENSG00000121022	validated
tarbase	hsa-miR-582-3p	COPS5	10987	ENSG00000121022	validated
tarbase	hsa-miR-212-3p	EPX	8288	ENSG00000121053	validated
tarbase	hsa-miR-296-5p	AKAP1	8165	ENSG00000121057	validated
tarbase	hsa-miR-301a-3p	AKAP1	8165	ENSG00000121057	validated
tarbase	hsa-miR-582-5p	AKAP1	8165	ENSG00000121057	validated
tarbase	hsa-miR-301a-3p	COIL	8161	ENSG00000121058	validated
tarbase	hsa-miR-301a-3p	TRIM25	7706	ENSG00000121060	validated
tarbase	hsa-miR-212-3p	TRIM25	7706	ENSG00000121060	validated
tarbase	hsa-miR-106b-5p	SPOP	8405	ENSG00000121067	validated
tarbase	hsa-miR-192-5p	SPOP	8405	ENSG00000121067	validated
tarbase	hsa-miR-106b-5p	FAM117A	81558	ENSG00000121104	validated
tarbase	hsa-miR-106b-5p	NCAPH	23397	ENSG00000121152	validated

tarbase	hsa-miR-582-5p	TENT4B	64282	ENSG00000121274	validated
tarbase	hsa-miR-192-5p	ADCY7	113	ENSG00000121281	validated
tarbase	hsa-miR-301a-3p	ADCY7	113	ENSG00000121281	validated
tarbase	hsa-miR-106b-5p	PRB2	653247	ENSG00000121335	validated
tarbase	hsa-miR-582-5p	IAPP	3375	ENSG00000121351	validated
tarbase	hsa-miR-212-3p	KCNJ8	3764	ENSG00000121361	validated
tarbase	hsa-miR-18a-5p	ZNF549	256051	ENSG00000121406	validated
tarbase	hsa-miR-106b-5p	LHX4	89884	ENSG00000121454	validated
tarbase	hsa-miR-106b-5p	RNF2	6045	ENSG00000121481	validated
tarbase	hsa-miR-181a-5p	SEC22A	26984	ENSG00000121542	validated
tarbase	hsa-miR-212-3p	POPDC2	64091	ENSG00000121577	validated
tarbase	hsa-miR-106b-5p	B4GALT4	8702	ENSG00000121578	validated
tarbase	hsa-miR-18a-5p	NAA50	80218	ENSG00000121579	validated
tarbase	hsa-miR-326	NAA50	80218	ENSG00000121579	validated
tarbase	hsa-miR-212-3p	KIF18A	81930	ENSG00000121621	validated
tarbase	hsa-miR-106b-5p	DESI2	51029	ENSG00000121644	validated
tarbase	hsa-miR-18a-5p	DESI2	51029	ENSG00000121644	validated
tarbase	hsa-miR-15b-5p	PILRB	29990	ENSG00000121716	validated
tarbase	hsa-miR-296-5p	PILRB	29990	ENSG00000121716	validated
tarbase	hsa-miR-326	PILRB	29990	ENSG00000121716	validated
tarbase	hsa-miR-106b-5p	ZMYM2	7750	ENSG00000121741	validated
tarbase	hsa-miR-181a-5p	ZMYM2	7750	ENSG00000121741	validated
tarbase	hsa-miR-582-3p	ZMYM2	7750	ENSG00000121741	validated
tarbase	hsa-miR-15b-5p	GJB6	10804	ENSG00000121742	validated
tarbase	hsa-miR-15b-5p	ADGRB2	576	ENSG00000121753	validated
tarbase	hsa-miR-144-3p	KHDRBS1	10657	ENSG00000121774	validated
tarbase	hsa-miR-181a-5p	KHDRBS1	10657	ENSG00000121774	validated
tarbase	hsa-miR-212-3p	GHSR	2693	ENSG00000121853	validated
tarbase	hsa-miR-18a-5p	ZNF639	51193	ENSG00000121864	validated
tarbase	hsa-miR-106b-5p	SLITRK3	22865	ENSG00000121871	validated
tarbase	hsa-miR-15b-5p	PIK3CA	5290	ENSG00000121879	validated
tarbase	hsa-miR-15b-5p	PDS5A	23244	ENSG00000121892	validated
tarbase	hsa-miR-18a-5p	LRIF1	55791	ENSG00000121931	validated
tarbase	hsa-miR-582-5p	CLCC1	23155	ENSG00000121940	validated
tarbase	hsa-miR-21-5p	GPSM2	29899	ENSG00000121957	validated
tarbase	hsa-miR-106b-5p	GPSM2	29899	ENSG00000121957	validated
tarbase	hsa-miR-212-3p	GPSM2	29899	ENSG00000121957	validated
tarbase	hsa-miR-18a-5p	ACVR2A	92	ENSG00000121989	validated
tarbase	hsa-miR-212-3p	ACVR2A	92	ENSG00000121989	validated
tarbase	hsa-miR-212-3p	FLT3	2322	ENSG00000122025	validated
tarbase	hsa-miR-212-3p	UBL3	5412	ENSG00000122042	validated
tarbase	hsa-miR-582-5p	UBL3	5412	ENSG00000122042	validated
tarbase	hsa-miR-301a-3p	UBL3	5412	ENSG00000122042	validated
tarbase	hsa-miR-106b-5p	MTERF4	130916	ENSG00000122085	validated
tarbase	hsa-miR-222-3p	MTERF4	130916	ENSG00000122085	validated
tarbase	hsa-miR-212-3p	SASH3	54440	ENSG00000122122	validated
tarbase	hsa-miR-301a-3p	OCRL	4952	ENSG00000122126	validated
tarbase	hsa-miR-15b-5p	MRPS2	51116	ENSG00000122140	validated
tarbase	hsa-miR-144-3p	COPA	1314	ENSG00000122218	validated
tarbase	hsa-miR-181a-5p	COPA	1314	ENSG00000122218	validated
tarbase	hsa-miR-18a-5p	COPA	1314	ENSG00000122218	validated
tarbase	hsa-miR-212-3p	COPA	1314	ENSG00000122218	validated
tarbase	hsa-miR-222-3p	COPA	1314	ENSG00000122218	validated
tarbase	hsa-miR-15b-5p	LY9	4063	ENSG00000122224	validated
tarbase	hsa-miR-582-5p	LY9	4063	ENSG00000122224	validated
tarbase	hsa-miR-106b-5p	RBBP6	5930	ENSG00000122257	validated
tarbase	hsa-miR-18a-5p	RBBP6	5930	ENSG00000122257	validated
tarbase	hsa-miR-301a-3p	RBBP6	5930	ENSG00000122257	validated
tarbase	hsa-miR-582-5p	ZC3H7A	29066	ENSG00000122299	validated
tarbase	hsa-miR-15b-5p	ANXA11	311	ENSG00000122359	validated
tarbase	hsa-miR-21-5p	ANXA11	311	ENSG00000122359	validated
tarbase	hsa-miR-296-5p	ANXA11	311	ENSG00000122359	validated
tarbase	hsa-miR-106b-5p	SHLD2	54537	ENSG00000122376	validated
tarbase	hsa-miR-212-3p	PRXL2A	84293	ENSG00000122378	validated
tarbase	hsa-miR-21-5p	PRXL2A	84293	ENSG00000122378	validated
tarbase	hsa-miR-212-3p	ZNF205	7755	ENSG00000122386	validated
tarbase	hsa-miR-181a-5p	ODF2L	57489	ENSG00000122417	validated
tarbase	hsa-miR-106b-5p	ZNF644	84146	ENSG00000122482	validated
tarbase	hsa-miR-181a-5p	ZNF644	84146	ENSG00000122482	validated
tarbase	hsa-miR-18a-5p	ZNF644	84146	ENSG00000122482	validated
tarbase	hsa-miR-212-3p	ZNF644	84146	ENSG00000122482	validated

tarbase	hsa-miR-181a-5p	SLC66A2	80148	ENSG00000122490	validated
tarbase	hsa-miR-212-3p	BBS9	27241	ENSG00000122507	validated
tarbase	hsa-miR-301a-3p	ZMIZ2	83637	ENSG00000122515	validated
tarbase	hsa-miR-106b-5p	KLHL7	55975	ENSG00000122550	validated
tarbase	hsa-miR-106b-5p	CBX3	11335	ENSG00000122565	validated
tarbase	hsa-miR-15b-5p	CBX3	11335	ENSG00000122565	validated
tarbase	hsa-miR-181a-5p	CBX3	11335	ENSG00000122565	validated
tarbase	hsa-miR-192-5p	CBX3	11335	ENSG00000122565	validated
tarbase	hsa-miR-106b-5p	HNRNPA2B1	3181	ENSG00000122566	validated
tarbase	hsa-miR-181a-5p	HNRNPA2B1	3181	ENSG00000122566	validated
tarbase	hsa-miR-18a-5p	HNRNPA2B1	3181	ENSG00000122566	validated
tarbase	hsa-miR-21-5p	HNRNPA2B1	3181	ENSG00000122566	validated
tarbase	hsa-miR-222-3p	HNRNPA2B1	3181	ENSG00000122566	validated
tarbase	hsa-miR-326	HNRNPA2B1	3181	ENSG00000122566	validated
tarbase	hsa-miR-106b-5p	WIPF3	644150	ENSG00000122574	validated
tarbase	hsa-miR-301a-3p	INHBA	3624	ENSG00000122641	validated
tarbase	hsa-miR-212-3p	INHBA	3624	ENSG00000122641	validated
tarbase	hsa-miR-106b-5p	ARL4A	10124	ENSG00000122644	validated
tarbase	hsa-miR-181a-5p	ARL4A	10124	ENSG00000122644	validated
tarbase	hsa-miR-106b-5p	SMU1	55234	ENSG00000122692	validated
tarbase	hsa-miR-181a-5p	RECK	8434	ENSG00000122707	validated
tarbase	hsa-miR-212-3p	TAF1L	138474	ENSG00000122728	validated
tarbase	hsa-miR-15b-5p	ACO1	48	ENSG00000122729	validated
tarbase	hsa-miR-296-5p	ACO1	48	ENSG00000122729	validated
tarbase	hsa-miR-106b-5p	DCAF10	79269	ENSG00000122741	validated
tarbase	hsa-miR-15b-5p	DCAF10	79269	ENSG00000122741	validated
tarbase	hsa-miR-326	DCAF10	79269	ENSG00000122741	validated
tarbase	hsa-miR-21-5p	KIAA1549	57670	ENSG00000122778	validated
tarbase	hsa-miR-301a-3p	KIAA1549	57670	ENSG00000122778	validated
tarbase	hsa-miR-106b-5p	CALD1	800	ENSG00000122786	validated
tarbase	hsa-miR-181a-5p	CALD1	800	ENSG00000122786	validated
tarbase	hsa-miR-181a-5p	PLAU	5328	ENSG00000122861	validated
tarbase	hsa-miR-18a-5p	SRGN	5552	ENSG00000122862	validated
tarbase	hsa-miR-326	CHST3	9469	ENSG00000122863	validated
tarbase	hsa-miR-212-3p	EGR2	1959	ENSG00000122877	validated
tarbase	hsa-miR-15b-5p	RBM19	9904	ENSG00000122965	validated
tarbase	hsa-miR-106b-5p	HVCN1	84329	ENSG00000122986	validated
tarbase	hsa-miR-106b-5p	MED13L	23389	ENSG00000123066	validated
tarbase	hsa-miR-192-5p	MED13L	23389	ENSG00000123066	validated
tarbase	hsa-miR-296-5p	MED13L	23389	ENSG00000123066	validated
tarbase	hsa-miR-301a-3p	MED13L	23389	ENSG00000123066	validated
tarbase	hsa-miR-106b-5p	CDKN2C	1031	ENSG00000123080	validated
tarbase	hsa-miR-21-5p	RASSF8	11228	ENSG00000123094	validated
tarbase	hsa-miR-582-5p	RASSF8	11228	ENSG00000123094	validated
tarbase	hsa-miR-15b-5p	ITPR2	3709	ENSG00000123104	validated
tarbase	hsa-miR-15b-5p	NECAB1	64168	ENSG00000123119	validated
tarbase	hsa-miR-18a-5p	PRDX4	10549	ENSG00000123131	validated
tarbase	hsa-miR-21-5p	DDX39A	10212	ENSG00000123136	validated
tarbase	hsa-miR-21-5p	ADGRE5	976	ENSG00000123146	validated
tarbase	hsa-miR-106b-5p	SPRYD7	57213	ENSG00000123178	validated
tarbase	hsa-miR-181a-5p	SPRYD7	57213	ENSG00000123178	validated
tarbase	hsa-miR-181a-5p	NLN	57486	ENSG00000123213	validated
tarbase	hsa-miR-18a-5p	ATF1	466	ENSG00000123268	validated
tarbase	hsa-miR-181a-5p	ORMDL2	29095	ENSG00000123353	validated
tarbase	hsa-miR-15b-5p	NR4A1	3164	ENSG00000123358	validated
tarbase	hsa-miR-18a-5p	HOXC13	3229	ENSG00000123364	validated
tarbase	hsa-miR-326	HOXC11	3227	ENSG00000123388	validated
tarbase	hsa-miR-301a-3p	ATG101	60673	ENSG00000123395	validated
tarbase	hsa-miR-192-5p	SMUG1	23583	ENSG00000123415	validated
tarbase	hsa-miR-212-3p	TUBA1B	10376	ENSG00000123416	validated
tarbase	hsa-miR-212-3p	KBTBD4	55709	ENSG00000123444	validated
tarbase	hsa-miR-301a-3p	ATPAF1	64756	ENSG00000123472	validated
tarbase	hsa-miR-212-3p	HJURP	55355	ENSG00000123485	validated
tarbase	hsa-miR-181a-5p	AMD1	262	ENSG00000123505	validated
tarbase	hsa-miR-106b-5p	MORF4L2	9643	ENSG00000123562	validated
tarbase	hsa-miR-181a-5p	MORF4L2	9643	ENSG00000123562	validated
tarbase	hsa-miR-15b-5p	FAM199X	139231	ENSG00000123575	validated
tarbase	hsa-miR-222-3p	FAM199X	139231	ENSG00000123575	validated
tarbase	hsa-miR-181a-5p	FAM199X	139231	ENSG00000123575	validated
tarbase	hsa-miR-106b-5p	RAB9A	9367	ENSG00000123595	validated
tarbase	hsa-miR-181a-5p	RAB9A	9367	ENSG00000123595	validated

tarbase	hsa-miR-18a-5p	RAB9A	9367	ENSG00000123595	validated
tarbase	hsa-miR-582-5p	NMI	9111	ENSG00000123609	validated
tarbase	hsa-miR-212-3p	NMI	9111	ENSG00000123609	validated
tarbase	hsa-miR-106b-5p	NMI	9111	ENSG00000123609	validated
tarbase	hsa-miR-181a-5p	ACVR1C	130399	ENSG00000123612	validated
tarbase	hsa-miR-212-3p	BAZ2B	29994	ENSG00000123636	validated
tarbase	hsa-miR-21-5p	BAZ2B	29994	ENSG00000123636	validated
tarbase	hsa-miR-106b-5p	SLC36A1	206358	ENSG00000123643	validated
tarbase	hsa-miR-21-5p	SLC36A1	206358	ENSG00000123643	validated
tarbase	hsa-miR-144-3p	LPGAT1	9926	ENSG00000123684	validated
tarbase	hsa-miR-212-3p	LPGAT1	9926	ENSG00000123684	validated
tarbase	hsa-miR-301a-3p	LPGAT1	9926	ENSG00000123684	validated
tarbase	hsa-miR-301a-3p	G0S2	50486	ENSG00000123689	validated
tarbase	hsa-miR-18a-5p	KCNJ2	3759	ENSG00000123700	validated
tarbase	hsa-miR-296-5p	RAP2C	57826	ENSG00000123728	validated
tarbase	hsa-miR-301a-3p	RAP2C	57826	ENSG00000123728	validated
tarbase	hsa-miR-15b-5p	PLA2G12A	81579	ENSG00000123739	validated
tarbase	hsa-miR-106b-5p	AGO2	27161	ENSG00000123908	validated
tarbase	hsa-miR-18a-5p	AGO2	27161	ENSG00000123908	validated
tarbase	hsa-miR-192-5p	MXD4	10608	ENSG00000123933	validated
tarbase	hsa-miR-181a-5p	CKS2	1164	ENSG00000123975	validated
tarbase	hsa-miR-15b-5p	ACSL3	2181	ENSG00000123983	validated
tarbase	hsa-miR-106b-5p	CHPF	79586	ENSG00000123989	validated
tarbase	hsa-miR-15b-5p	OBSL1	23363	ENSG00000124006	validated
tarbase	hsa-miR-18a-5p	FAM210B	116151	ENSG00000124098	validated
tarbase	hsa-miR-106b-5p	PI3	5266	ENSG00000124102	validated
tarbase	hsa-miR-15b-5p	TTPAL	79183	ENSG00000124120	validated
tarbase	hsa-miR-582-5p	TTPAL	79183	ENSG00000124120	validated
tarbase	hsa-miR-106b-5p	PREX1	57580	ENSG00000124126	validated
tarbase	hsa-miR-15b-5p	SLC12A5	57468	ENSG00000124140	validated
tarbase	hsa-miR-15b-5p	SDC4	6385	ENSG00000124145	validated
tarbase	hsa-miR-212-3p	SDC4	6385	ENSG00000124145	validated
tarbase	hsa-miR-301a-3p	SDC4	6385	ENSG00000124145	validated
tarbase	hsa-miR-212-3p	NCOA3	8202	ENSG00000124151	validated
tarbase	hsa-miR-296-5p	NCOA5	57727	ENSG00000124160	validated
tarbase	hsa-miR-582-3p	NCOA5	57727	ENSG00000124160	validated
tarbase	hsa-miR-106b-5p	VAPB	9217	ENSG00000124164	validated
tarbase	hsa-miR-15b-5p	VAPB	9217	ENSG00000124164	validated
tarbase	hsa-miR-212-3p	VAPB	9217	ENSG00000124164	validated
tarbase	hsa-miR-222-3p	VAPB	9217	ENSG00000124164	validated
tarbase	hsa-miR-15b-5p	PARD6B	84612	ENSG00000124171	validated
tarbase	hsa-miR-181a-5p	PARD6B	84612	ENSG00000124171	validated
tarbase	hsa-miR-505-3p	PARD6B	84612	ENSG00000124171	validated
tarbase	hsa-miR-582-3p	PARD6B	84612	ENSG00000124171	validated
tarbase	hsa-miR-582-5p	PARD6B	84612	ENSG00000124171	validated
tarbase	hsa-miR-15b-5p	CHD6	84181	ENSG00000124177	validated
tarbase	hsa-miR-212-3p	CHD6	84181	ENSG00000124177	validated
tarbase	hsa-miR-301a-3p	PLCG1	5335	ENSG00000124181	validated
tarbase	hsa-miR-106b-5p	SRSF6	6431	ENSG00000124193	validated
tarbase	hsa-miR-181a-5p	SRSF6	6431	ENSG00000124193	validated
tarbase	hsa-miR-582-3p	SRSF6	6431	ENSG00000124193	validated
tarbase	hsa-miR-106b-5p	ARFGEF2	10564	ENSG00000124198	validated
tarbase	hsa-miR-15b-5p	ARFGEF2	10564	ENSG00000124198	validated
tarbase	hsa-miR-181a-5p	ARFGEF2	10564	ENSG00000124198	validated
tarbase	hsa-miR-15b-5p	ZNFX1	57169	ENSG00000124201	validated
tarbase	hsa-miR-18a-5p	ZNFX1	57169	ENSG00000124201	validated
tarbase	hsa-miR-21-5p	ZNFX1	57169	ENSG00000124201	validated
tarbase	hsa-miR-296-5p	ZNFX1	57169	ENSG00000124201	validated
tarbase	hsa-miR-301a-3p	ZNFX1	57169	ENSG00000124201	validated
tarbase	hsa-miR-582-5p	ZNFX1	57169	ENSG00000124201	validated
tarbase	hsa-miR-212-3p	ZNFX1	57169	ENSG00000124201	validated
tarbase	hsa-miR-181a-5p	CSE1L	1434	ENSG00000124207	validated
tarbase	hsa-miR-21-5p	CSE1L	1434	ENSG00000124207	validated
tarbase	hsa-miR-326	CSE1L	1434	ENSG00000124207	validated
tarbase	hsa-miR-15b-5p	RAB22A	57403	ENSG00000124209	validated
tarbase	hsa-miR-301a-3p	RAB22A	57403	ENSG00000124209	validated
tarbase	hsa-miR-106b-5p	STAU1	6780	ENSG00000124214	validated
tarbase	hsa-miR-15b-5p	STAU1	6780	ENSG00000124214	validated
tarbase	hsa-miR-212-3p	STAU1	6780	ENSG00000124214	validated
tarbase	hsa-miR-296-5p	SNAI1	6615	ENSG00000124216	validated
tarbase	hsa-miR-326	SNAI1	6615	ENSG00000124216	validated

tarbase	hsa-miR-15b-5p	STX16	8675	ENSG00000124222	validated
tarbase	hsa-miR-296-5p	STX16	8675	ENSG00000124222	validated
tarbase	hsa-miR-106b-5p	PMEPA1	56937	ENSG00000124225	validated
tarbase	hsa-miR-212-3p	PMEPA1	56937	ENSG00000124225	validated
tarbase	hsa-miR-301a-3p	PMEPA1	56937	ENSG00000124225	validated
tarbase	hsa-miR-301a-3p	RNF114	55905	ENSG00000124226	validated
tarbase	hsa-miR-212-3p	C20orf85	128602	ENSG00000124237	validated
tarbase	hsa-miR-212-3p	PCK1	5105	ENSG00000124253	validated
tarbase	hsa-miR-181a-5p	MTRR	4552	ENSG00000124275	validated
tarbase	hsa-miR-15b-5p	PEPD	5184	ENSG00000124299	validated
tarbase	hsa-miR-106b-5p	IQSEC2	23096	ENSG00000124313	validated
tarbase	hsa-miR-15b-5p	VAMP7	6845	ENSG00000124333	validated
tarbase	hsa-miR-222-3p	STAMBP	10617	ENSG00000124356	validated
tarbase	hsa-miR-106b-5p	MPHOSPH10	10199	ENSG00000124383	validated
tarbase	hsa-miR-296-5p	USP22	23326	ENSG00000124422	validated
tarbase	hsa-miR-106b-5p	USP22	23326	ENSG00000124422	validated
tarbase	hsa-miR-181a-5p	USP22	23326	ENSG00000124422	validated
tarbase	hsa-miR-582-5p	ZNF576	79177	ENSG00000124444	validated
tarbase	hsa-miR-18a-5p	LYPD3	27076	ENSG00000124466	validated
tarbase	hsa-miR-106b-5p	USP9X	8239	ENSG00000124486	validated
tarbase	hsa-miR-181a-5p	USP9X	8239	ENSG00000124486	validated
tarbase	hsa-miR-18a-5p	USP9X	8239	ENSG00000124486	validated
tarbase	hsa-miR-212-3p	USP9X	8239	ENSG00000124486	validated
tarbase	hsa-miR-21-5p	USP9X	8239	ENSG00000124486	validated
tarbase	hsa-miR-106b-5p	F13A1	2162	ENSG00000124491	validated
tarbase	hsa-miR-106b-5p	SIRT5	23408	ENSG00000124523	validated
tarbase	hsa-miR-192-5p	WRNIP1	56897	ENSG00000124535	validated
tarbase	hsa-miR-181a-5p	RRP36	88745	ENSG00000124541	validated
tarbase	hsa-miR-106b-5p	SNRPC	6631	ENSG00000124562	validated
tarbase	hsa-miR-15b-5p	ABCC10	89845	ENSG00000124574	validated
tarbase	hsa-miR-301a-3p	AARS2	57505	ENSG00000124608	validated
tarbase	hsa-miR-106b-5p	TBCC	6903	ENSG00000124659	validated
tarbase	hsa-miR-106b-5p	KLHDC3	116138	ENSG00000124702	validated
tarbase	hsa-miR-15b-5p	KLHDC3	116138	ENSG00000124702	validated
tarbase	hsa-miR-326	KLHDC3	116138	ENSG00000124702	validated
tarbase	hsa-miR-582-3p	TREM1	54210	ENSG00000124731	validated
tarbase	hsa-miR-106b-5p	MEA1	4201	ENSG00000124733	validated
tarbase	hsa-miR-296-5p	MEA1	4201	ENSG00000124733	validated
tarbase	hsa-miR-18a-5p	KLHL31	401265	ENSG00000124743	validated
tarbase	hsa-miR-212-3p	COL21A1	81578	ENSG00000124749	validated
tarbase	hsa-miR-18a-5p	CDKN1A	1026	ENSG00000124762	validated
tarbase	hsa-miR-301a-3p	CDKN1A	1026	ENSG00000124762	validated
tarbase	hsa-miR-181a-5p	SOX4	6659	ENSG00000124766	validated
tarbase	hsa-miR-192-5p	SOX4	6659	ENSG00000124766	validated
tarbase	hsa-miR-18a-5p	GLO1	2739	ENSG00000124767	validated
tarbase	hsa-miR-301a-3p	GLO1	2739	ENSG00000124767	validated
tarbase	hsa-miR-326	GLO1	2739	ENSG00000124767	validated
tarbase	hsa-miR-15b-5p	RREB1	6239	ENSG00000124782	validated
tarbase	hsa-miR-222-3p	RREB1	6239	ENSG00000124782	validated
tarbase	hsa-miR-106b-5p	SSR1	6745	ENSG00000124783	validated
tarbase	hsa-miR-301a-3p	SSR1	6745	ENSG00000124783	validated
tarbase	hsa-miR-505-3p	SSR1	6745	ENSG00000124783	validated
tarbase	hsa-miR-582-3p	SSR1	6745	ENSG00000124783	validated
tarbase	hsa-miR-582-5p	SSR1	6745	ENSG00000124783	validated
tarbase	hsa-miR-181a-5p	ATXN1	6310	ENSG00000124788	validated
tarbase	hsa-miR-222-3p	ATXN1	6310	ENSG00000124788	validated
tarbase	hsa-miR-296-5p	ATXN1	6310	ENSG00000124788	validated
tarbase	hsa-miR-301a-3p	ATXN1	6310	ENSG00000124788	validated
tarbase	hsa-miR-15b-5p	NUP153	9972	ENSG00000124789	validated
tarbase	hsa-miR-181a-5p	NUP153	9972	ENSG00000124789	validated
tarbase	hsa-miR-301a-3p	NUP153	9972	ENSG00000124789	validated
tarbase	hsa-miR-181a-5p	DEK	7913	ENSG00000124795	validated
tarbase	hsa-miR-21-5p	DEK	7913	ENSG00000124795	validated
tarbase	hsa-miR-222-3p	DEK	7913	ENSG00000124795	validated
tarbase	hsa-miR-505-3p	EEF1E1	9521	ENSG00000124802	validated
tarbase	hsa-miR-181a-5p	EEF1E1	9521	ENSG00000124802	validated
tarbase	hsa-miR-505-3p	RUNX2	860	ENSG00000124813	validated
tarbase	hsa-miR-212-3p	GCM2	9247	ENSG00000124827	validated
tarbase	hsa-miR-212-3p	LRRFIP1	9208	ENSG00000124831	validated
tarbase	hsa-miR-181a-5p	LRRFIP1	9208	ENSG00000124831	validated
tarbase	hsa-miR-301a-3p	CXCL6	6372	ENSG00000124875	validated

tarbase	hsa-miR-106b-5p	AHNAK	79026	ENSG00000124942	validated
tarbase	hsa-miR-181a-5p	AHNAK	79026	ENSG00000124942	validated
tarbase	hsa-miR-18a-5p	AHNAK	79026	ENSG00000124942	validated
tarbase	hsa-miR-21-5p	AHNAK	79026	ENSG00000124942	validated
tarbase	hsa-miR-296-5p	AHNAK	79026	ENSG00000124942	validated
tarbase	hsa-miR-505-3p	AHNAK	79026	ENSG00000124942	validated
tarbase	hsa-miR-301a-3p	EMC3	55831	ENSG00000125037	validated
tarbase	hsa-miR-106b-5p	CNOT1	23019	ENSG00000125107	validated
tarbase	hsa-miR-144-3p	CNOT1	23019	ENSG00000125107	validated
tarbase	hsa-miR-15b-5p	CNOT1	23019	ENSG00000125107	validated
tarbase	hsa-miR-181a-5p	CNOT1	23019	ENSG00000125107	validated
tarbase	hsa-miR-18a-5p	CNOT1	23019	ENSG00000125107	validated
tarbase	hsa-miR-301a-3p	CNOT1	23019	ENSG00000125107	validated
tarbase	hsa-miR-582-3p	CNOT1	23019	ENSG00000125107	validated
tarbase	hsa-miR-296-5p	GOT2	2806	ENSG00000125166	validated
tarbase	hsa-miR-181a-5p	GOT2	2806	ENSG00000125166	validated
tarbase	hsa-miR-15b-5p	TMTC4	84899	ENSG00000125247	validated
tarbase	hsa-miR-15b-5p	RAP2A	5911	ENSG00000125249	validated
tarbase	hsa-miR-181a-5p	RAP2A	5911	ENSG00000125249	validated
tarbase	hsa-miR-106b-5p	EFNB2	1948	ENSG00000125266	validated
tarbase	hsa-miR-301a-3p	EFNB2	1948	ENSG00000125266	validated
tarbase	hsa-miR-212-3p	SOX21	11166	ENSG00000125285	validated
tarbase	hsa-miR-18a-5p	TM9SF2	9375	ENSG00000125304	validated
tarbase	hsa-miR-106b-5p	IRF1	3659	ENSG00000125347	validated
tarbase	hsa-miR-15b-5p	IRF1	3659	ENSG00000125347	validated
tarbase	hsa-miR-212-3p	IRF1	3659	ENSG00000125347	validated
tarbase	hsa-miR-582-5p	TMEM255A	55026	ENSG00000125355	validated
tarbase	hsa-miR-212-3p	TMEM255A	55026	ENSG00000125355	validated
tarbase	hsa-miR-18a-5p	BMP4	652	ENSG00000125378	validated
tarbase	hsa-miR-21-5p	SOX9	6662	ENSG00000125398	validated
tarbase	hsa-miR-18a-5p	HS3ST3B1	9953	ENSG00000125430	validated
tarbase	hsa-miR-326	MRPS7	51081	ENSG00000125445	validated
tarbase	hsa-miR-21-5p	SLC25A19	60386	ENSG00000125454	validated
tarbase	hsa-miR-326	SLC25A19	60386	ENSG00000125454	validated
tarbase	hsa-miR-106b-5p	GTF3C4	9329	ENSG00000125484	validated
tarbase	hsa-miR-15b-5p	GTF3C4	9329	ENSG00000125484	validated
tarbase	hsa-miR-192-5p	GTF3C4	9329	ENSG00000125484	validated
tarbase	hsa-miR-21-5p	GTF3C4	9329	ENSG00000125484	validated
tarbase	hsa-miR-582-5p	GTF3C4	9329	ENSG00000125484	validated
tarbase	hsa-miR-192-5p	BARHL1	56751	ENSG00000125492	validated
tarbase	hsa-miR-15b-5p	MBOAT7	79143	ENSG00000125505	validated
tarbase	hsa-miR-21-5p	SLC2A4RG	56731	ENSG00000125520	validated
tarbase	hsa-miR-301a-3p	SLC2A4RG	56731	ENSG00000125520	validated
tarbase	hsa-miR-326	PPDPF	79144	ENSG00000125534	validated
tarbase	hsa-miR-212-3p	PLGLB2	5342	ENSG00000125551	validated
tarbase	hsa-miR-212-3p	INSIG2	51141	ENSG00000125629	validated
tarbase	hsa-miR-15b-5p	INSIG2	51141	ENSG00000125629	validated
tarbase	hsa-miR-21-5p	CCDC93	54520	ENSG00000125633	validated
tarbase	hsa-miR-181a-5p	THOC2	57187	ENSG00000125676	validated
tarbase	hsa-miR-106b-5p	MED1	5469	ENSG00000125686	validated
tarbase	hsa-miR-15b-5p	MED1	5469	ENSG00000125686	validated
tarbase	hsa-miR-505-3p	MED1	5469	ENSG00000125686	validated
tarbase	hsa-miR-18a-5p	MED1	5469	ENSG00000125686	validated
tarbase	hsa-miR-106b-5p	OPA3	80207	ENSG00000125741	validated
tarbase	hsa-miR-18a-5p	EML2	24139	ENSG00000125746	validated
tarbase	hsa-miR-106b-5p	VASP	7408	ENSG00000125753	validated
tarbase	hsa-miR-18a-5p	VASP	7408	ENSG00000125753	validated
tarbase	hsa-miR-15b-5p	SYMPK	8189	ENSG00000125755	validated
tarbase	hsa-miR-144-3p	GPCPD1	56261	ENSG00000125772	validated
tarbase	hsa-miR-181a-5p	GPCPD1	56261	ENSG00000125772	validated
tarbase	hsa-miR-212-3p	GPCPD1	56261	ENSG00000125772	validated
tarbase	hsa-miR-326	GPCPD1	56261	ENSG00000125772	validated
tarbase	hsa-miR-106b-5p	PANK2	80025	ENSG00000125779	validated
tarbase	hsa-miR-505-3p	PANK2	80025	ENSG00000125779	validated
tarbase	hsa-miR-181a-5p	CD93	22918	ENSG00000125810	validated
tarbase	hsa-miR-106b-5p	NAPB	63908	ENSG00000125814	validated
tarbase	hsa-miR-181a-5p	NAPB	63908	ENSG00000125814	validated
tarbase	hsa-miR-296-5p	CENPB	1059	ENSG00000125817	validated
tarbase	hsa-miR-326	CENPB	1059	ENSG00000125817	validated
tarbase	hsa-miR-212-3p	CENPB	1059	ENSG00000125817	validated
tarbase	hsa-miR-192-5p	TMX4	56255	ENSG00000125827	validated

tarbase	hsa-miR-106b-5p	STK35	140901	ENSG00000125834	validated
tarbase	hsa-miR-15b-5p	STK35	140901	ENSG00000125834	validated
tarbase	hsa-miR-212-3p	STK35	140901	ENSG00000125834	validated
tarbase	hsa-miR-15b-5p	SNRPB	6628	ENSG00000125835	validated
tarbase	hsa-miR-18a-5p	SNRPB	6628	ENSG00000125835	validated
tarbase	hsa-miR-222-3p	SNRPB	6628	ENSG00000125835	validated
tarbase	hsa-miR-192-5p	BMP2	650	ENSG00000125845	validated
tarbase	hsa-miR-106b-5p	MKKS	8195	ENSG00000125863	validated
tarbase	hsa-miR-212-3p	LRRN4	164312	ENSG00000125872	validated
tarbase	hsa-miR-106b-5p	TBC1D20	128637	ENSG00000125875	validated
tarbase	hsa-miR-18a-5p	TBC1D20	128637	ENSG00000125875	validated
tarbase	hsa-miR-296-5p	TBC1D20	128637	ENSG00000125875	validated
tarbase	hsa-miR-582-5p	TBC1D20	128637	ENSG00000125875	validated
tarbase	hsa-miR-21-5p	ITPA	3704	ENSG00000125877	validated
tarbase	hsa-miR-301a-3p	ITPA	3704	ENSG00000125877	validated
tarbase	hsa-miR-212-3p	FAM110A	83541	ENSG00000125898	validated
tarbase	hsa-miR-15b-5p	NCLN	56926	ENSG00000125912	validated
tarbase	hsa-miR-326	NCLN	56926	ENSG00000125912	validated
tarbase	hsa-miR-181a-5p	HNRNPR	10236	ENSG00000125944	validated
tarbase	hsa-miR-212-3p	HNRNPR	10236	ENSG00000125944	validated
tarbase	hsa-miR-15b-5p	MAX	4149	ENSG00000125952	validated
tarbase	hsa-miR-15b-5p	ID1	3397	ENSG00000125968	validated
tarbase	hsa-miR-181a-5p	DYNLRB1	83658	ENSG00000125971	validated
tarbase	hsa-miR-15b-5p	ROMO1	140823	ENSG00000125995	validated
tarbase	hsa-miR-222-3p	BPIFB1	92747	ENSG00000125999	validated
tarbase	hsa-miR-15b-5p	PLAGL2	5326	ENSG00000126003	validated
tarbase	hsa-miR-21-5p	PLAGL2	5326	ENSG00000126003	validated
tarbase	hsa-miR-181a-5p	PLAGL2	5326	ENSG00000126003	validated
tarbase	hsa-miR-181a-5p	AMOT	154796	ENSG00000126016	validated
tarbase	hsa-miR-15b-5p	TMEM115	11070	ENSG00000126062	validated
tarbase	hsa-miR-106b-5p	PSMB2	5690	ENSG00000126067	validated
tarbase	hsa-miR-144-3p	PSMB2	5690	ENSG00000126067	validated
tarbase	hsa-miR-144-3p	AGO3	192669	ENSG00000126070	validated
tarbase	hsa-miR-15b-5p	AGO3	192669	ENSG00000126070	validated
tarbase	hsa-miR-181a-5p	AGO3	192669	ENSG00000126070	validated
tarbase	hsa-miR-18a-5p	AGO3	192669	ENSG00000126070	validated
tarbase	hsa-miR-222-3p	AGO3	192669	ENSG00000126070	validated
tarbase	hsa-miR-296-5p	HECTD3	79654	ENSG00000126107	validated
tarbase	hsa-miR-582-5p	HECTD3	79654	ENSG00000126107	validated
tarbase	hsa-miR-106b-5p	KLC1	3831	ENSG00000126214	validated
tarbase	hsa-miR-15b-5p	KLC1	3831	ENSG00000126214	validated
tarbase	hsa-miR-18a-5p	KLC1	3831	ENSG00000126214	validated
tarbase	hsa-miR-505-3p	KLC1	3831	ENSG00000126214	validated
tarbase	hsa-miR-212-3p	TUBGCP3	10426	ENSG00000126216	validated
tarbase	hsa-miR-106b-5p	TUBGCP3	10426	ENSG00000126216	validated
tarbase	hsa-miR-106b-5p	MCF2L	23263	ENSG00000126217	validated
tarbase	hsa-miR-582-3p	MCF2L	23263	ENSG00000126217	validated
tarbase	hsa-miR-181a-5p	PCID2	55795	ENSG00000126226	validated
tarbase	hsa-miR-192-5p	CAPNS1	826	ENSG00000126247	validated
tarbase	hsa-miR-296-5p	CAPNS1	826	ENSG00000126247	validated
tarbase	hsa-miR-326	CCR7	1236	ENSG00000126353	validated
tarbase	hsa-miR-15b-5p	PRDX5	25824	ENSG00000126432	validated
tarbase	hsa-miR-296-5p	PRDX5	25824	ENSG00000126432	validated
tarbase	hsa-miR-296-5p	IRF3	3661	ENSG00000126456	validated
tarbase	hsa-miR-18a-5p	PRR12	57479	ENSG00000126464	validated
tarbase	hsa-miR-212-3p	PRR12	57479	ENSG00000126464	validated
tarbase	hsa-miR-296-5p	PRR12	57479	ENSG00000126464	validated
tarbase	hsa-miR-15b-5p	BECN1	8678	ENSG00000126581	validated
tarbase	hsa-miR-301a-3p	BECN1	8678	ENSG00000126581	validated
tarbase	hsa-miR-15b-5p	TRAP1	10131	ENSG00000126602	validated
tarbase	hsa-miR-15b-5p	NSRP1	84081	ENSG00000126653	validated
tarbase	hsa-miR-301a-3p	AHDC1	27245	ENSG00000126705	validated
tarbase	hsa-miR-212-3p	IFI6	2537	ENSG00000126709	validated
tarbase	hsa-miR-192-5p	ZNF384	171017	ENSG00000126746	validated
tarbase	hsa-miR-181a-5p	ZNF384	171017	ENSG00000126746	validated
tarbase	hsa-miR-296-5p	ELK1	2002	ENSG00000126767	validated
tarbase	hsa-miR-106b-5p	PCNX4	64430	ENSG00000126773	validated
tarbase	hsa-miR-181a-5p	PCNX4	64430	ENSG00000126773	validated
tarbase	hsa-miR-212-3p	ATG14	22863	ENSG00000126775	validated
tarbase	hsa-miR-15b-5p	KTN1	3895	ENSG00000126777	validated
tarbase	hsa-miR-181a-5p	KTN1	3895	ENSG00000126777	validated

tarbase	hsa-miR-222-3p	SIX1	6495	ENSG00000126778	validated
tarbase	hsa-miR-212-3p	DLGAP5	9787	ENSG00000126787	validated
tarbase	hsa-miR-144-3p	ZBTB1	22890	ENSG00000126804	validated
tarbase	hsa-miR-181a-5p	ZBTB1	22890	ENSG00000126804	validated
tarbase	hsa-miR-21-5p	ZBTB1	22890	ENSG00000126804	validated
tarbase	hsa-miR-212-3p	ZBTB1	22890	ENSG00000126804	validated
tarbase	hsa-miR-106b-5p	SGPP1	81537	ENSG00000126821	validated
tarbase	hsa-miR-181a-5p	EVI2A	2123	ENSG00000126860	validated
tarbase	hsa-miR-18a-5p	NUP214	8021	ENSG00000126883	validated
tarbase	hsa-miR-21-5p	NUP214	8021	ENSG00000126883	validated
tarbase	hsa-miR-326	NUP214	8021	ENSG00000126883	validated
tarbase	hsa-miR-296-5p	MAP2K2	5605	ENSG00000126934	validated
tarbase	hsa-miR-106b-5p	HNRNPH2	3188	ENSG00000126945	validated
tarbase	hsa-miR-15b-5p	ARMCX1	51309	ENSG00000126947	validated
tarbase	hsa-miR-192-5p	CANX	821	ENSG00000127022	validated
tarbase	hsa-miR-301a-3p	CANX	821	ENSG00000127022	validated
tarbase	hsa-miR-106b-5p	ZNF484	83744	ENSG00000127081	validated
tarbase	hsa-miR-181a-5p	ZNF484	83744	ENSG00000127081	validated
tarbase	hsa-miR-106b-5p	HIVEP3	59269	ENSG00000127124	validated
tarbase	hsa-miR-106b-5p	BCL11B	64919	ENSG00000127152	validated
tarbase	hsa-miR-106b-5p	HELB	92797	ENSG00000127311	validated
tarbase	hsa-miR-301a-3p	HELB	92797	ENSG00000127311	validated
tarbase	hsa-miR-106b-5p	RAP1B	5908	ENSG00000127314	validated
tarbase	hsa-miR-326	RAB3IP	117177	ENSG00000127328	validated
tarbase	hsa-miR-582-3p	RAB3IP	117177	ENSG00000127328	validated
tarbase	hsa-miR-181a-5p	RAB3IP	117177	ENSG00000127328	validated
tarbase	hsa-miR-15b-5p	DYRK2	8445	ENSG00000127334	validated
tarbase	hsa-miR-181a-5p	DYRK2	8445	ENSG00000127334	validated
tarbase	hsa-miR-18a-5p	DYRK2	8445	ENSG00000127334	validated
tarbase	hsa-miR-582-3p	DYRK2	8445	ENSG00000127334	validated
tarbase	hsa-miR-21-5p	YEATS4	8089	ENSG00000127337	validated
tarbase	hsa-miR-296-5p	YEATS4	8089	ENSG00000127337	validated
tarbase	hsa-miR-296-5p	FGFRL1	53834	ENSG00000127418	validated
tarbase	hsa-miR-212-3p	EMC1	23065	ENSG00000127463	validated
tarbase	hsa-miR-582-3p	EMC1	23065	ENSG00000127463	validated
tarbase	hsa-miR-106b-5p	UBR4	23352	ENSG00000127481	validated
tarbase	hsa-miR-15b-5p	UBR4	23352	ENSG00000127481	validated
tarbase	hsa-miR-181a-5p	UBR4	23352	ENSG00000127481	validated
tarbase	hsa-miR-222-3p	UBR4	23352	ENSG00000127481	validated
tarbase	hsa-miR-296-5p	UBR4	23352	ENSG00000127481	validated
tarbase	hsa-miR-301a-3p	UBR4	23352	ENSG00000127481	validated
tarbase	hsa-miR-582-5p	UBR4	23352	ENSG00000127481	validated
tarbase	hsa-miR-106b-5p	HP1BP3	50809	ENSG00000127483	validated
tarbase	hsa-miR-181a-5p	SIN3B	23309	ENSG00000127511	validated
tarbase	hsa-miR-18a-5p	SIN3B	23309	ENSG00000127511	validated
tarbase	hsa-miR-181a-5p	SLC35E1	79939	ENSG00000127526	validated
tarbase	hsa-miR-301a-3p	SLC35E1	79939	ENSG00000127526	validated
tarbase	hsa-miR-582-5p	SLC35E1	79939	ENSG00000127526	validated
tarbase	hsa-miR-212-3p	KLF2	10365	ENSG00000127528	validated
tarbase	hsa-miR-212-3p	SYNGR3	9143	ENSG00000127561	validated
tarbase	hsa-miR-222-3p			ENSG00000127603	validated
tarbase	hsa-miR-296-5p			ENSG00000127603	validated
tarbase	hsa-miR-582-5p			ENSG00000127603	validated
tarbase	hsa-miR-15b-5p	SMARCA4	6597	ENSG00000127616	validated
tarbase	hsa-miR-21-5p	KDM4B	23030	ENSG00000127663	validated
tarbase	hsa-miR-582-3p	AAMP	14	ENSG00000127837	validated
tarbase	hsa-miR-301a-3p	RNF6	6049	ENSG00000127870	validated
tarbase	hsa-miR-18a-5p	ECHS1	1892	ENSG00000127884	validated
tarbase	hsa-miR-15b-5p	AKAP9	10142	ENSG00000127914	validated
tarbase	hsa-miR-181a-5p	AKAP9	10142	ENSG00000127914	validated
tarbase	hsa-miR-21-5p	HIP1	3092	ENSG00000127946	validated
tarbase	hsa-miR-106b-5p	PTPN12	5782	ENSG00000127947	validated
tarbase	hsa-miR-15b-5p	PTPN12	5782	ENSG00000127947	validated
tarbase	hsa-miR-18a-5p	PTPN12	5782	ENSG00000127947	validated
tarbase	hsa-miR-212-3p	PTPN12	5782	ENSG00000127947	validated
tarbase	hsa-miR-106b-5p	FGL2	10875	ENSG00000127951	validated
tarbase	hsa-miR-301a-3p	STEAP4	79689	ENSG00000127954	validated
tarbase	hsa-miR-301a-3p	GNAI1	2770	ENSG00000127955	validated
tarbase	hsa-miR-192-5p	PEX1	5189	ENSG00000127980	validated
tarbase	hsa-miR-505-3p	PEX1	5189	ENSG00000127980	validated
tarbase	hsa-miR-21-5p	MTERF1	7978	ENSG00000127989	validated

tarbase	hsa-miR-21-5p	ZNF780B	163131	ENSG00000128000	validated
tarbase	hsa-miR-296-5p	ZFP36	7538	ENSG00000128016	validated
tarbase	hsa-miR-301a-3p	ZFP36	7538	ENSG00000128016	validated
tarbase	hsa-miR-106b-5p	RASL11B	65997	ENSG00000128045	validated
tarbase	hsa-miR-192-5p	RASL11B	65997	ENSG00000128045	validated
tarbase	hsa-miR-582-5p	PAICS	10606	ENSG00000128050	validated
tarbase	hsa-miR-15b-5p	PAICS	10606	ENSG00000128050	validated
tarbase	hsa-miR-106b-5p	KDR	3791	ENSG00000128052	validated
tarbase	hsa-miR-326	KDR	3791	ENSG00000128052	validated
tarbase	hsa-miR-18a-5p	DGCR8	54487	ENSG00000128191	validated
tarbase	hsa-miR-212-3p	DGCR8	54487	ENSG00000128191	validated
tarbase	hsa-miR-181a-5p	YWHAH	7533	ENSG00000128245	validated
tarbase	hsa-miR-212-3p	YWHAH	7533	ENSG00000128245	validated
tarbase	hsa-miR-222-3p	YWHAH	7533	ENSG00000128245	validated
tarbase	hsa-miR-192-5p	ATF4	468	ENSG00000128272	validated
tarbase	hsa-miR-222-3p	ATF4	468	ENSG00000128272	validated
tarbase	hsa-miR-212-3p	BAIAP2L2	80115	ENSG00000128298	validated
tarbase	hsa-miR-181a-5p	LIF	3976	ENSG00000128342	validated
tarbase	hsa-miR-18a-5p	LIF	3976	ENSG00000128342	validated
tarbase	hsa-miR-212-3p	C22orf23	84645	ENSG00000128346	validated
tarbase	hsa-miR-15b-5p	EMC4	51234	ENSG00000128463	validated
tarbase	hsa-miR-106b-5p	SPECC1	92521	ENSG00000128487	validated
tarbase	hsa-miR-181a-5p	DOCK4	9732	ENSG00000128512	validated
tarbase	hsa-miR-21-5p	FOXP2	93986	ENSG00000128573	validated
tarbase	hsa-miR-212-3p	FOXP2	93986	ENSG00000128573	validated
tarbase	hsa-miR-106b-5p	IFT22	64792	ENSG00000128581	validated
tarbase	hsa-miR-106b-5p	MKLN1	4289	ENSG00000128585	validated
tarbase	hsa-miR-181a-5p	MKLN1	4289	ENSG00000128585	validated
tarbase	hsa-miR-582-5p	MKLN1	4289	ENSG00000128585	validated
tarbase	hsa-miR-301a-3p	DNAJB9	4189	ENSG00000128590	validated
tarbase	hsa-miR-106b-5p	CALU	813	ENSG00000128595	validated
tarbase	hsa-miR-212-3p	CALU	813	ENSG00000128595	validated
tarbase	hsa-miR-582-3p	CALU	813	ENSG00000128595	validated
tarbase	hsa-miR-106b-5p	KLHDC10	23008	ENSG00000128607	validated
tarbase	hsa-miR-18a-5p	KLHDC10	23008	ENSG00000128607	validated
tarbase	hsa-miR-326	KLHDC10	23008	ENSG00000128607	validated
tarbase	hsa-miR-301a-3p	NDUFA5	4698	ENSG00000128609	validated
tarbase	hsa-miR-181a-5p	MTX2	10651	ENSG00000128654	validated
tarbase	hsa-miR-301a-3p	ORMDL1	94101	ENSG00000128699	validated
tarbase	hsa-miR-582-3p	ORMDL1	94101	ENSG00000128699	validated
tarbase	hsa-miR-21-5p	HAT1	8520	ENSG00000128708	validated
tarbase	hsa-miR-212-3p	SNRPN	6638	ENSG00000128739	validated
tarbase	hsa-miR-106b-5p	PSMG2	56984	ENSG00000128789	validated
tarbase	hsa-miR-582-5p	PSMG2	56984	ENSG00000128789	validated
tarbase	hsa-miR-296-5p	TWSG1	57045	ENSG00000128791	validated
tarbase	hsa-miR-15b-5p	MYO5C	55930	ENSG00000128833	validated
tarbase	hsa-miR-15b-5p	TTBK2	146057	ENSG00000128881	validated
tarbase	hsa-miR-181a-5p	CCDC32	90416	ENSG00000128891	validated
tarbase	hsa-miR-301a-3p	CCDC32	90416	ENSG00000128891	validated
tarbase	hsa-miR-301a-3p	INO80	54617	ENSG00000128908	validated
tarbase	hsa-miR-181a-5p	ICE2	79664	ENSG00000128915	validated
tarbase	hsa-miR-15b-5p	ICE2	79664	ENSG00000128915	validated
tarbase	hsa-miR-181a-5p	MINDY2	54629	ENSG00000128923	validated
tarbase	hsa-miR-18a-5p	MINDY2	54629	ENSG00000128923	validated
tarbase	hsa-miR-21-5p	MINDY2	54629	ENSG00000128923	validated
tarbase	hsa-miR-181a-5p	IVD	3712	ENSG00000128928	validated
tarbase	hsa-miR-301a-3p	DUT	1854	ENSG00000128951	validated
tarbase	hsa-miR-296-5p	CHAC1	79094	ENSG00000128965	validated
tarbase	hsa-miR-582-3p	CHAC1	79094	ENSG00000128965	validated
tarbase	hsa-miR-106b-5p	CHAC1	79094	ENSG00000128965	validated
tarbase	hsa-miR-106b-5p	ARPP19	10776	ENSG00000128989	validated
tarbase	hsa-miR-15b-5p	ARPP19	10776	ENSG00000128989	validated
tarbase	hsa-miR-181a-5p	ARPP19	10776	ENSG00000128989	validated
tarbase	hsa-miR-21-5p	VPS13C	54832	ENSG00000129003	validated
tarbase	hsa-miR-181a-5p	VPS13C	54832	ENSG00000129003	validated
tarbase	hsa-miR-212-3p	THAP10	56906	ENSG00000129028	validated
tarbase	hsa-miR-181a-5p	MBD4	8930	ENSG00000129071	validated
tarbase	hsa-miR-15b-5p	COPB1	1315	ENSG00000129083	validated
tarbase	hsa-miR-181a-5p	COPB1	1315	ENSG00000129083	validated
tarbase	hsa-miR-18a-5p	COPB1	1315	ENSG00000129083	validated
tarbase	hsa-miR-212-3p	COPB1	1315	ENSG00000129083	validated

tarbase	hsa-miR-212-3p	PSMA1	5682	ENSG00000129084	validated
tarbase	hsa-miR-505-3p	PSMA1	5682	ENSG00000129084	validated
tarbase	hsa-miR-582-3p	SUMF2	25870	ENSG00000129103	validated
tarbase	hsa-miR-106b-5p	PALLD	23022	ENSG00000129116	validated
tarbase	hsa-miR-106b-5p	SPCS3	60559	ENSG00000129128	validated
tarbase	hsa-miR-15b-5p	SPCS3	60559	ENSG00000129128	validated
tarbase	hsa-miR-21-5p	SPCS3	60559	ENSG00000129128	validated
tarbase	hsa-miR-582-5p	SPCS3	60559	ENSG00000129128	validated
tarbase	hsa-miR-181a-5p	SPCS3	60559	ENSG00000129128	validated
tarbase	hsa-miR-106b-5p	KCNC1	3746	ENSG00000129159	validated
tarbase	hsa-miR-212-3p	CSRP3	8048	ENSG00000129170	validated
tarbase	hsa-miR-212-3p	E2F8	79733	ENSG00000129173	validated
tarbase	hsa-miR-15b-5p	KIF1C	10749	ENSG00000129250	validated
tarbase	hsa-miR-18a-5p	KIF1C	10749	ENSG00000129250	validated
tarbase	hsa-miR-296-5p	KIF1C	10749	ENSG00000129250	validated
tarbase	hsa-miR-326	KIF1C	10749	ENSG00000129250	validated
tarbase	hsa-miR-18a-5p	PHF20L1	51105	ENSG00000129292	validated
tarbase	hsa-miR-106b-5p	CCNT1	904	ENSG00000129315	validated
tarbase	hsa-miR-582-3p	CCNT1	904	ENSG00000129315	validated
tarbase	hsa-miR-582-5p	PUS7L	83448	ENSG00000129317	validated
tarbase	hsa-miR-15b-5p	ILF3	3609	ENSG00000129351	validated
tarbase	hsa-miR-181a-5p	ILF3	3609	ENSG00000129351	validated
tarbase	hsa-miR-301a-3p	ILF3	3609	ENSG00000129351	validated
tarbase	hsa-miR-505-3p	ILF3	3609	ENSG00000129351	validated
tarbase	hsa-miR-106b-5p	MTUS1	57509	ENSG00000129422	validated
tarbase	hsa-miR-212-3p	MTUS1	57509	ENSG00000129422	validated
tarbase	hsa-miR-212-3p	RIPK3	11035	ENSG00000129465	validated
tarbase	hsa-miR-15b-5p	BCL2L2	599	ENSG00000129473	validated
tarbase	hsa-miR-181a-5p	BCL2L2	599	ENSG00000129473	validated
tarbase	hsa-miR-212-3p	BCL2L2	599	ENSG00000129473	validated
tarbase	hsa-miR-296-5p	BCL2L2	599	ENSG00000129473	validated
tarbase	hsa-miR-301a-3p	BCL2L2	599	ENSG00000129473	validated
tarbase	hsa-miR-326	BCL2L2	599	ENSG00000129473	validated
tarbase	hsa-miR-106b-5p	AJUBA	84962	ENSG00000129474	validated
tarbase	hsa-miR-181a-5p	AJUBA	84962	ENSG00000129474	validated
tarbase	hsa-miR-18a-5p	AJUBA	84962	ENSG00000129474	validated
tarbase	hsa-miR-15b-5p	DTD2	112487	ENSG00000129480	validated
tarbase	hsa-miR-181a-5p	DTD2	112487	ENSG00000129480	validated
tarbase	hsa-miR-15b-5p	HEATR5A	25938	ENSG00000129493	validated
tarbase	hsa-miR-192-5p	HEATR5A	25938	ENSG00000129493	validated
tarbase	hsa-miR-106b-5p	FOXA1	3169	ENSG00000129514	validated
tarbase	hsa-miR-212-3p	FOXA1	3169	ENSG00000129514	validated
tarbase	hsa-miR-301a-3p	FOXA1	3169	ENSG00000129514	validated
tarbase	hsa-miR-106b-5p	EAPP	55837	ENSG00000129518	validated
tarbase	hsa-miR-181a-5p	EAPP	55837	ENSG00000129518	validated
tarbase	hsa-miR-505-3p	NEDD8	4738	ENSG00000129559	validated
tarbase	hsa-miR-18a-5p	DAD1	1603	ENSG00000129562	validated
tarbase	hsa-miR-326	DAD1	1603	ENSG00000129562	validated
tarbase	hsa-miR-15b-5p	EPB41L4A	64097	ENSG00000129595	validated
tarbase	hsa-miR-582-3p	EPB41L4A	64097	ENSG00000129595	validated
tarbase	hsa-miR-144-3p	REEP5	7905	ENSG00000129625	validated
tarbase	hsa-miR-15b-5p	REEP5	7905	ENSG00000129625	validated
tarbase	hsa-miR-18a-5p	REEP5	7905	ENSG00000129625	validated
tarbase	hsa-miR-181a-5p	REEP5	7905	ENSG00000129625	validated
tarbase	hsa-miR-21-5p	ITFG1	81533	ENSG00000129636	validated
tarbase	hsa-miR-106b-5p	SEC14L1	6397	ENSG00000129657	validated
tarbase	hsa-miR-106b-5p	ARHGEF6	9459	ENSG00000129675	validated
tarbase	hsa-miR-15b-5p	CHRNA10	57053	ENSG00000129749	validated
tarbase	hsa-miR-296-5p	KLF16	83855	ENSG00000129911	validated
tarbase	hsa-miR-301a-3p	KLF16	83855	ENSG00000129911	validated
tarbase	hsa-miR-15b-5p	PGAP6	58986	ENSG00000129925	validated
tarbase	hsa-miR-181a-5p	PUDP	8226	ENSG00000130021	validated
tarbase	hsa-miR-106b-5p	PUDP	8226	ENSG00000130021	validated
tarbase	hsa-miR-106b-5p	PHF10	55274	ENSG00000130024	validated
tarbase	hsa-miR-181a-5p	PHF10	55274	ENSG00000130024	validated
tarbase	hsa-miR-222-3p	PHF10	55274	ENSG00000130024	validated
tarbase	hsa-miR-301a-3p	PHF10	55274	ENSG00000130024	validated
tarbase	hsa-miR-326	PHF10	55274	ENSG00000130024	validated
tarbase	hsa-miR-212-3p	KCNA5	3741	ENSG00000130037	validated
tarbase	hsa-miR-144-3p	SAT1	6303	ENSG00000130066	validated
tarbase	hsa-miR-15b-5p	SAT1	6303	ENSG00000130066	validated

tarbase	hsa-miR-18a-5p	SH3BP4	23677	ENSG00000130147	validated
tarbase	hsa-miR-21-5p	SH3BP4	23677	ENSG00000130147	validated
tarbase	hsa-miR-106b-5p	MOSPD2	158747	ENSG00000130150	validated
tarbase	hsa-miR-15b-5p	MOSPD2	158747	ENSG00000130150	validated
tarbase	hsa-miR-181a-5p	MOSPD2	158747	ENSG00000130150	validated
tarbase	hsa-miR-18a-5p	MOSPD2	158747	ENSG00000130150	validated
tarbase	hsa-miR-15b-5p	DOCK6	57572	ENSG00000130158	validated
tarbase	hsa-miR-21-5p	DOCK6	57572	ENSG00000130158	validated
tarbase	hsa-miR-18a-5p	LDLR	3949	ENSG00000130164	validated
tarbase	hsa-miR-192-5p	LDLR	3949	ENSG00000130164	validated
tarbase	hsa-miR-192-5p	PRKCSH	5589	ENSG00000130175	validated
tarbase	hsa-miR-296-5p	PRKCSH	5589	ENSG00000130175	validated
tarbase	hsa-miR-212-3p	CNN1	1264	ENSG00000130176	validated
tarbase	hsa-miR-192-5p	TOMM40	10452	ENSG00000130204	validated
tarbase	hsa-miR-181a-5p	LRCH2	57631	ENSG00000130224	validated
tarbase	hsa-miR-15b-5p	XPO7	23039	ENSG00000130227	validated
tarbase	hsa-miR-21-5p	XPO7	23039	ENSG00000130227	validated
tarbase	hsa-miR-301a-3p	XPO7	23039	ENSG00000130227	validated
tarbase	hsa-miR-582-3p	XPO7	23039	ENSG00000130227	validated
tarbase	hsa-miR-21-5p	SAFB2	9667	ENSG00000130254	validated
tarbase	hsa-miR-326	RPL36	25873	ENSG00000130255	validated
tarbase	hsa-miR-301a-3p	KIF1A	547	ENSG00000130294	validated
tarbase	hsa-miR-326	KIF1A	547	ENSG00000130294	validated
tarbase	hsa-miR-18a-5p	GTPBP3	84705	ENSG00000130299	validated
tarbase	hsa-miR-15b-5p	COLGALT1	79709	ENSG00000130309	validated
tarbase	hsa-miR-181a-5p	COLGALT1	79709	ENSG00000130309	validated
tarbase	hsa-miR-21-5p	COLGALT1	79709	ENSG00000130309	validated
tarbase	hsa-miR-21-5p	PGLS	25796	ENSG00000130313	validated
tarbase	hsa-miR-106b-5p	TULP4	56995	ENSG00000130338	validated
tarbase	hsa-miR-212-3p	TULP4	56995	ENSG00000130338	validated
tarbase	hsa-miR-296-5p	TULP4	56995	ENSG00000130338	validated
tarbase	hsa-miR-301a-3p	TULP4	56995	ENSG00000130338	validated
tarbase	hsa-miR-181a-5p	TULP4	56995	ENSG00000130338	validated
tarbase	hsa-miR-106b-5p	SNX9	51429	ENSG00000130340	validated
tarbase	hsa-miR-181a-5p	SNX9	51429	ENSG00000130340	validated
tarbase	hsa-miR-582-5p	SNX9	51429	ENSG00000130340	validated
tarbase	hsa-miR-212-3p	MTRES1	51250	ENSG00000130349	validated
tarbase	hsa-miR-212-3p	RSPH3	83861	ENSG00000130363	validated
tarbase	hsa-miR-21-5p	AFDN	4301	ENSG00000130396	validated
tarbase	hsa-miR-15b-5p	STK33	65975	ENSG00000130413	validated
tarbase	hsa-miR-15b-5p	ARPC1B	10095	ENSG00000130429	validated
tarbase	hsa-miR-181a-5p	ARPC1B	10095	ENSG00000130429	validated
tarbase	hsa-miR-15b-5p	ZSWIM6	57688	ENSG00000130449	validated
tarbase	hsa-miR-181a-5p	ZSWIM6	57688	ENSG00000130449	validated
tarbase	hsa-miR-212-3p	ZSWIM6	57688	ENSG00000130449	validated
tarbase	hsa-miR-21-5p	ZSWIM6	57688	ENSG00000130449	validated
tarbase	hsa-miR-222-3p	ZSWIM6	57688	ENSG00000130449	validated
tarbase	hsa-miR-301a-3p	ZSWIM6	57688	ENSG00000130449	validated
tarbase	hsa-miR-106b-5p	ZSWIM6	57688	ENSG00000130449	validated
tarbase	hsa-miR-15b-5p	UNC13A	23025	ENSG00000130477	validated
tarbase	hsa-miR-21-5p	MAP1S	55201	ENSG00000130479	validated
tarbase	hsa-miR-301a-3p	PXDN	7837	ENSG00000130508	validated
tarbase	hsa-miR-505-3p	PXDN	7837	ENSG00000130508	validated
tarbase	hsa-miR-106b-5p	GDF15	9518	ENSG00000130513	validated
tarbase	hsa-miR-181a-5p	GDF15	9518	ENSG00000130513	validated
tarbase	hsa-miR-296-5p	IQCN	80726	ENSG00000130518	validated
tarbase	hsa-miR-212-3p	HRC	3270	ENSG00000130528	validated
tarbase	hsa-miR-212-3p	TRPM4	54795	ENSG00000130529	validated
tarbase	hsa-miR-582-3p	CAMSAP1	157922	ENSG00000130559	validated
tarbase	hsa-miR-181a-5p	CAMSAP1	157922	ENSG00000130559	validated
tarbase	hsa-miR-15b-5p	ZBTB46	140685	ENSG00000130584	validated
tarbase	hsa-miR-212-3p	HELZ2	85441	ENSG00000130589	validated
tarbase	hsa-miR-106b-5p	SAMD10	140700	ENSG00000130590	validated
tarbase	hsa-miR-15b-5p	COL5A1	1289	ENSG00000130635	validated
tarbase	hsa-miR-181a-5p	COL5A1	1289	ENSG00000130635	validated
tarbase	hsa-miR-18a-5p	ATXN10	25814	ENSG00000130638	validated
tarbase	hsa-miR-301a-3p	ATXN10	25814	ENSG00000130638	validated
tarbase	hsa-miR-106b-5p	ZNF337	26152	ENSG00000130684	validated
tarbase	hsa-miR-301a-3p	ZNF337	26152	ENSG00000130684	validated
tarbase	hsa-miR-301a-3p	TAF4	6874	ENSG00000130699	validated
tarbase	hsa-miR-582-3p	ASS1	445	ENSG00000130707	validated

tarbase	hsa-miR-144-3p	POMT1	10585	ENSG00000130714	validated
tarbase	hsa-miR-212-3p	POMT1	10585	ENSG00000130714	validated
tarbase	hsa-miR-21-5p	POMT1	10585	ENSG00000130714	validated
tarbase	hsa-miR-15b-5p	PRRC2B	84726	ENSG00000130723	validated
tarbase	hsa-miR-18a-5p	PRRC2B	84726	ENSG00000130723	validated
tarbase	hsa-miR-296-5p	PRRC2B	84726	ENSG00000130723	validated
tarbase	hsa-miR-296-5p	TRIM28	10155	ENSG00000130726	validated
tarbase	hsa-miR-301a-3p	TRIM28	10155	ENSG00000130726	validated
tarbase	hsa-miR-582-3p	TRIM28	10155	ENSG00000130726	validated
tarbase	hsa-miR-15b-5p	YIPF2	78992	ENSG00000130733	validated
tarbase	hsa-miR-296-5p	YIPF2	78992	ENSG00000130733	validated
tarbase	hsa-miR-15b-5p	EIF2S3	1968	ENSG00000130741	validated
tarbase	hsa-miR-106b-5p	ZC3H4	23211	ENSG00000130749	validated
tarbase	hsa-miR-21-5p	ZC3H4	23211	ENSG00000130749	validated
tarbase	hsa-miR-15b-5p	LRRC47	57470	ENSG00000130764	validated
tarbase	hsa-miR-296-5p	LRRC47	57470	ENSG00000130764	validated
tarbase	hsa-miR-222-3p	SESN2	83667	ENSG00000130766	validated
tarbase	hsa-miR-301a-3p	SESN2	83667	ENSG00000130766	validated
tarbase	hsa-miR-301a-3p	ATP5IF1	93974	ENSG00000130770	validated
tarbase	hsa-miR-106b-5p	CLIP1	6249	ENSG00000130779	validated
tarbase	hsa-miR-181a-5p	CLIP1	6249	ENSG00000130779	validated
tarbase	hsa-miR-18a-5p	CLIP1	6249	ENSG00000130779	validated
tarbase	hsa-miR-181a-5p	HIP1R	9026	ENSG00000130787	validated
tarbase	hsa-miR-181a-5p	ZNF317	57693	ENSG00000130803	validated
tarbase	hsa-miR-301a-3p	DNMT1	1786	ENSG00000130816	validated
tarbase	hsa-miR-15b-5p	SLC6A8	6535	ENSG00000130821	validated
tarbase	hsa-miR-15b-5p	DKC1	1736	ENSG00000130826	validated
tarbase	hsa-miR-181a-5p	DKC1	1736	ENSG00000130826	validated
tarbase	hsa-miR-222-3p	DKC1	1736	ENSG00000130826	validated
tarbase	hsa-miR-106b-5p	ZNF236	7776	ENSG00000130856	validated
tarbase	hsa-miR-15b-5p	ZNF236	7776	ENSG00000130856	validated
tarbase	hsa-miR-181a-5p	ZNF236	7776	ENSG00000130856	validated
tarbase	hsa-miR-301a-3p	NOL11	25926	ENSG00000130935	validated
tarbase	hsa-miR-15b-5p	UBE4B	10277	ENSG00000130939	validated
tarbase	hsa-miR-301a-3p	UBE4B	10277	ENSG00000130939	validated
tarbase	hsa-miR-326	CASZ1	54897	ENSG00000130940	validated
tarbase	hsa-miR-222-3p	PRRG1	5638	ENSG00000130962	validated
tarbase	hsa-miR-21-5p	UBA1	7317	ENSG00000130985	validated
tarbase	hsa-miR-212-3p	PPIL4	85313	ENSG00000131013	validated
tarbase	hsa-miR-106b-5p	PPIL4	85313	ENSG00000131013	validated
tarbase	hsa-miR-192-5p	AKAP12	9590	ENSG00000131016	validated
tarbase	hsa-miR-21-5p	SYNE1	23345	ENSG00000131018	validated
tarbase	hsa-miR-181a-5p	SYNE1	23345	ENSG00000131018	validated
tarbase	hsa-miR-15b-5p	LATS1	9113	ENSG00000131023	validated
tarbase	hsa-miR-181a-5p	LATS1	9113	ENSG00000131023	validated
tarbase	hsa-miR-181a-5p	AAR2	25980	ENSG00000131043	validated
tarbase	hsa-miR-181a-5p	RBM39	9584	ENSG00000131051	validated
tarbase	hsa-miR-181a-5p	ACSS2	55902	ENSG00000131069	validated
tarbase	hsa-miR-212-3p	EDA2R	60401	ENSG00000131080	validated
tarbase	hsa-miR-15b-5p	GFAP	2670	ENSG00000131095	validated
tarbase	hsa-miR-212-3p	ATP6V1E1	529	ENSG00000131100	validated
tarbase	hsa-miR-181a-5p	ZNF227	7770	ENSG00000131115	validated
tarbase	hsa-miR-192-5p	ZNF227	7770	ENSG00000131115	validated
tarbase	hsa-miR-222-3p	ZNF227	7770	ENSG00000131115	validated
tarbase	hsa-miR-15b-5p	ZNF428	126299	ENSG00000131116	validated
tarbase	hsa-miR-582-3p	COX4I1	1327	ENSG00000131143	validated
tarbase	hsa-miR-181a-5p	GSE1	23199	ENSG00000131149	validated
tarbase	hsa-miR-192-5p	GSE1	23199	ENSG00000131149	validated
tarbase	hsa-miR-222-3p	GSE1	23199	ENSG00000131149	validated
tarbase	hsa-miR-326	GSE1	23199	ENSG00000131149	validated
tarbase	hsa-miR-296-5p	CHMP1A	5119	ENSG00000131165	validated
tarbase	hsa-miR-181a-5p	CAP1	10487	ENSG00000131236	validated
tarbase	hsa-miR-212-3p	CAP1	10487	ENSG00000131236	validated
tarbase	hsa-miR-21-5p	CAP1	10487	ENSG00000131236	validated
tarbase	hsa-miR-296-5p	CAP1	10487	ENSG00000131236	validated
tarbase	hsa-miR-192-5p	RLIM	51132	ENSG00000131263	validated
tarbase	hsa-miR-301a-3p	ABCB7	22	ENSG00000131269	validated
tarbase	hsa-miR-15b-5p	TRAF3	7187	ENSG00000131323	validated
tarbase	hsa-miR-301a-3p	HAUS8	93323	ENSG00000131351	validated
tarbase	hsa-miR-15b-5p	MRPS25	64432	ENSG00000131368	validated
tarbase	hsa-miR-301a-3p	SH3BP5	9467	ENSG00000131370	validated

tarbase	hsa-miR-106b-5p	CAPN7	23473	ENSG00000131375	validated
tarbase	hsa-miR-18a-5p	CAPN7	23473	ENSG00000131375	validated
tarbase	hsa-miR-106b-5p	RBSN	64145	ENSG00000131381	validated
tarbase	hsa-miR-15b-5p	RBSN	64145	ENSG00000131381	validated
tarbase	hsa-miR-21-5p	RBSN	64145	ENSG00000131381	validated
tarbase	hsa-miR-582-5p	RBSN	64145	ENSG00000131381	validated
tarbase	hsa-miR-301a-3p	SLC6A6	6533	ENSG00000131389	validated
tarbase	hsa-miR-18a-5p	NR1H2	7376	ENSG00000131408	validated
tarbase	hsa-miR-326	NR1H2	7376	ENSG00000131408	validated
tarbase	hsa-miR-181a-5p	KIF3A	11127	ENSG00000131437	validated
tarbase	hsa-miR-582-5p	KIF3A	11127	ENSG00000131437	validated
tarbase	hsa-miR-18a-5p	KIF3A	11127	ENSG00000131437	validated
tarbase	hsa-miR-144-3p	GFPT2	9945	ENSG00000131459	validated
tarbase	hsa-miR-582-5p	GFPT2	9945	ENSG00000131459	validated
tarbase	hsa-miR-301a-3p	TUBG1	7283	ENSG00000131462	validated
tarbase	hsa-miR-212-3p	PSME3	10197	ENSG00000131467	validated
tarbase	hsa-miR-222-3p	PSME3	10197	ENSG00000131467	validated
tarbase	hsa-miR-582-3p	PSME3	10197	ENSG00000131467	validated
tarbase	hsa-miR-192-5p	RPL27	6155	ENSG00000131469	validated
tarbase	hsa-miR-18a-5p	RPL27	6155	ENSG00000131469	validated
tarbase	hsa-miR-582-3p	PSMC3IP	29893	ENSG00000131470	validated
tarbase	hsa-miR-21-5p	ACLY	47	ENSG00000131473	validated
tarbase	hsa-miR-326	ACLY	47	ENSG00000131473	validated
tarbase	hsa-miR-582-5p	ACLY	47	ENSG00000131473	validated
tarbase	hsa-miR-106b-5p	VPS25	84313	ENSG00000131475	validated
tarbase	hsa-miR-18a-5p	VPS25	84313	ENSG00000131475	validated
tarbase	hsa-miR-21-5p	DIAPH1	1729	ENSG00000131504	validated
tarbase	hsa-miR-18a-5p	NDFIP1	80762	ENSG00000131507	validated
tarbase	hsa-miR-192-5p	EXOC4	60412	ENSG00000131558	validated
tarbase	hsa-miR-21-5p	EXOC4	60412	ENSG00000131558	validated
tarbase	hsa-miR-106b-5p	MAP1B	4131	ENSG00000131711	validated
tarbase	hsa-miR-15b-5p	MAP1B	4131	ENSG00000131711	validated
tarbase	hsa-miR-181a-5p	MAP1B	4131	ENSG00000131711	validated
tarbase	hsa-miR-18a-5p	MAP1B	4131	ENSG00000131711	validated
tarbase	hsa-miR-301a-3p	MAP1B	4131	ENSG00000131711	validated
tarbase	hsa-miR-505-3p	IL13RA1	3597	ENSG00000131724	validated
tarbase	hsa-miR-222-3p	ZCCHC9	84240	ENSG00000131732	validated
tarbase	hsa-miR-181a-5p	ZCCHC9	84240	ENSG00000131732	validated
tarbase	hsa-miR-301a-3p	TOP2A	7153	ENSG00000131747	validated
tarbase	hsa-miR-106b-5p	TOP2A	7153	ENSG00000131747	validated
tarbase	hsa-miR-212-3p	TOP2A	7153	ENSG00000131747	validated
tarbase	hsa-miR-18a-5p	STARD3	10948	ENSG00000131748	validated
tarbase	hsa-miR-106b-5p	PRKAB2	5565	ENSG00000131791	validated
tarbase	hsa-miR-15b-5p	PRKAB2	5565	ENSG00000131791	validated
tarbase	hsa-miR-222-3p	PRKAB2	5565	ENSG00000131791	validated
tarbase	hsa-miR-296-5p	PRKAB2	5565	ENSG00000131791	validated
tarbase	hsa-miR-326	PRKAB2	5565	ENSG00000131791	validated
tarbase	hsa-miR-106b-5p	PDHA1	5160	ENSG00000131828	validated
tarbase	hsa-miR-192-5p	PDHA1	5160	ENSG00000131828	validated
tarbase	hsa-miR-181a-5p	MCCC2	64087	ENSG00000131844	validated
tarbase	hsa-miR-181a-5p	ZNF132	7691	ENSG00000131849	validated
tarbase	hsa-miR-212-3p	ZNF132	7691	ENSG00000131849	validated
tarbase	hsa-miR-15b-5p	SELENOS	55829	ENSG00000131871	validated
tarbase	hsa-miR-212-3p	SELENOS	55829	ENSG00000131871	validated
tarbase	hsa-miR-212-3p	CHSY1	22856	ENSG00000131873	validated
tarbase	hsa-miR-222-3p	CHSY1	22856	ENSG00000131873	validated
tarbase	hsa-miR-106b-5p	THAP1	55145	ENSG00000131931	validated
tarbase	hsa-miR-212-3p	THAP1	55145	ENSG00000131931	validated
tarbase	hsa-miR-18a-5p	RHPN2	85415	ENSG00000131941	validated
tarbase	hsa-miR-181a-5p	C19orf12	83636	ENSG00000131943	validated
tarbase	hsa-miR-106b-5p	LGALS3	3958	ENSG00000131981	validated
tarbase	hsa-miR-212-3p	DNAJB1	3337	ENSG00000132002	validated
tarbase	hsa-miR-326	C19orf57	79173	ENSG00000132016	validated
tarbase	hsa-miR-181a-5p	TRIM21	6737	ENSG00000132109	validated
tarbase	hsa-miR-212-3p	TRIM21	6737	ENSG00000132109	validated
tarbase	hsa-miR-296-5p	LRRC41	10489	ENSG00000132128	validated
tarbase	hsa-miR-21-5p	DHX30	22907	ENSG00000132153	validated
tarbase	hsa-miR-296-5p	DHX30	22907	ENSG00000132153	validated
tarbase	hsa-miR-301a-3p	DHX30	22907	ENSG00000132153	validated
tarbase	hsa-miR-326	RAF1	5894	ENSG00000132155	validated
tarbase	hsa-miR-301a-3p	PPARG	5468	ENSG00000132170	validated

tarbase	hsa-miR-582-5p	ENOSF1	55556	ENSG00000132199	validated
tarbase	hsa-miR-301a-3p	ARFIP2	23647	ENSG00000132254	validated
tarbase	hsa-miR-212-3p	TRIM5	85363	ENSG00000132256	validated
tarbase	hsa-miR-18a-5p	TRIM22	10346	ENSG00000132274	validated
tarbase	hsa-miR-212-3p	TRIM22	10346	ENSG00000132274	validated
tarbase	hsa-miR-15b-5p	TIMM10B	26515	ENSG00000132286	validated
tarbase	hsa-miR-181a-5p	EFR3A	23167	ENSG00000132294	validated
tarbase	hsa-miR-18a-5p	EFR3A	23167	ENSG00000132294	validated
tarbase	hsa-miR-21-5p	EFR3A	23167	ENSG00000132294	validated
tarbase	hsa-miR-505-3p	EFR3A	23167	ENSG00000132294	validated
tarbase	hsa-miR-18a-5p	IMMT	10989	ENSG00000132305	validated
tarbase	hsa-miR-106b-5p	PER2	8864	ENSG00000132326	validated
tarbase	hsa-miR-15b-5p	PER2	8864	ENSG00000132326	validated
tarbase	hsa-miR-222-3p	PER2	8864	ENSG00000132326	validated
tarbase	hsa-miR-18a-5p	SCLY	51540	ENSG00000132330	validated
tarbase	hsa-miR-15b-5p	RAN	5901	ENSG00000132341	validated
tarbase	hsa-miR-106b-5p	PRKAA1	5562	ENSG00000132356	validated
tarbase	hsa-miR-505-3p	PRKAA1	5562	ENSG00000132356	validated
tarbase	hsa-miR-106b-5p	CLUH	23277	ENSG00000132361	validated
tarbase	hsa-miR-212-3p	INPP5K	51763	ENSG00000132376	validated
tarbase	hsa-miR-21-5p	INPP5K	51763	ENSG00000132376	validated
tarbase	hsa-miR-21-5p	MYBBP1A	10514	ENSG00000132382	validated
tarbase	hsa-miR-192-5p	RPA1	6117	ENSG00000132383	validated
tarbase	hsa-miR-106b-5p	UBE2G1	7326	ENSG00000132388	validated
tarbase	hsa-miR-181a-5p	UBE2G1	7326	ENSG00000132388	validated
tarbase	hsa-miR-582-5p	UBE2G1	7326	ENSG00000132388	validated
tarbase	hsa-miR-212-3p	TMEM128	85013	ENSG00000132406	validated
tarbase	hsa-miR-582-5p	COQ3	51805	ENSG00000132423	validated
tarbase	hsa-miR-106b-5p	PNISR	25957	ENSG00000132424	validated
tarbase	hsa-miR-181a-5p	PNISR	25957	ENSG00000132424	validated
tarbase	hsa-miR-582-5p	PNISR	25957	ENSG00000132424	validated
tarbase	hsa-miR-106b-5p	FIGNL1	63979	ENSG00000132436	validated
tarbase	hsa-miR-106b-5p	GRSF1	2926	ENSG00000132463	validated
tarbase	hsa-miR-15b-5p	GRSF1	2926	ENSG00000132463	validated
tarbase	hsa-miR-21-5p	GRSF1	2926	ENSG00000132463	validated
tarbase	hsa-miR-15b-5p	ANKRD17	26057	ENSG00000132466	validated
tarbase	hsa-miR-21-5p	ANKRD17	26057	ENSG00000132466	validated
tarbase	hsa-miR-222-3p	ITGB4	3691	ENSG00000132470	validated
tarbase	hsa-miR-181a-5p	WBP2	23558	ENSG00000132471	validated
tarbase	hsa-miR-212-3p	H3-3B	3021	ENSG00000132475	validated
tarbase	hsa-miR-181a-5p	ZRANB2	9406	ENSG00000132485	validated
tarbase	hsa-miR-15b-5p	EIF5A	1984	ENSG00000132507	validated
tarbase	hsa-miR-21-5p	EIF5A	1984	ENSG00000132507	validated
tarbase	hsa-miR-212-3p	KDM6B	23135	ENSG00000132510	validated
tarbase	hsa-miR-296-5p	KDM6B	23135	ENSG00000132510	validated
tarbase	hsa-miR-181a-5p	KDM6B	23135	ENSG00000132510	validated
tarbase	hsa-miR-18a-5p	GUCY2D	3000	ENSG00000132518	validated
tarbase	hsa-miR-212-3p	XAF1	54739	ENSG00000132530	validated
tarbase	hsa-miR-192-5p	DLG4	1742	ENSG00000132535	validated
tarbase	hsa-miR-212-3p	DLG4	1742	ENSG00000132535	validated
tarbase	hsa-miR-15b-5p	VPS13B	157680	ENSG00000132549	validated
tarbase	hsa-miR-21-5p	VPS13B	157680	ENSG00000132549	validated
tarbase	hsa-miR-296-5p	REEP2	51308	ENSG00000132563	validated
tarbase	hsa-miR-301a-3p	REEP2	51308	ENSG00000132563	validated
tarbase	hsa-miR-296-5p	FLOT2	2319	ENSG00000132589	validated
tarbase	hsa-miR-21-5p	ERAL1	26284	ENSG00000132591	validated
tarbase	hsa-miR-18a-5p	ERAL1	26284	ENSG00000132591	validated
tarbase	hsa-miR-18a-5p	NIP7	51388	ENSG00000132603	validated
tarbase	hsa-miR-181a-5p	NIP7	51388	ENSG00000132603	validated
tarbase	hsa-miR-15b-5p	MTSS2	92154	ENSG00000132613	validated
tarbase	hsa-miR-15b-5p	ANKEF1	63926	ENSG00000132623	validated
tarbase	hsa-miR-326	PCED1A	64773	ENSG00000132635	validated
tarbase	hsa-miR-582-3p	PCED1A	64773	ENSG00000132635	validated
tarbase	hsa-miR-301a-3p	SNAP25	6616	ENSG00000132639	validated
tarbase	hsa-miR-106b-5p	BTBD3	22903	ENSG00000132640	validated
tarbase	hsa-miR-144-3p	BTBD3	22903	ENSG00000132640	validated
tarbase	hsa-miR-15b-5p	BTBD3	22903	ENSG00000132640	validated
tarbase	hsa-miR-106b-5p	PCNA	5111	ENSG00000132646	validated
tarbase	hsa-miR-15b-5p	POLR3F	10621	ENSG00000132664	validated
tarbase	hsa-miR-144-3p	RIN2	54453	ENSG00000132669	validated
tarbase	hsa-miR-505-3p	RIN2	54453	ENSG00000132669	validated

tarbase	hsa-miR-106b-5p	PTPRA	5786	ENSG00000132670	validated
tarbase	hsa-miR-15b-5p	KHDC4	22889	ENSG00000132680	validated
tarbase	hsa-miR-192-5p	KHDC4	22889	ENSG00000132680	validated
tarbase	hsa-miR-212-3p	KHDC4	22889	ENSG00000132680	validated
tarbase	hsa-miR-15b-5p	ARHGEF11	9826	ENSG00000132694	validated
tarbase	hsa-miR-15b-5p	DCAF8	50717	ENSG00000132716	validated
tarbase	hsa-miR-192-5p	DCAF8	50717	ENSG00000132716	validated
tarbase	hsa-miR-181a-5p	MMACHC	25974	ENSG00000132763	validated
tarbase	hsa-miR-18a-5p	TOE1	114034	ENSG00000132773	validated
tarbase	hsa-miR-192-5p	NASP	4678	ENSG00000132780	validated
tarbase	hsa-miR-212-3p	NASP	4678	ENSG00000132780	validated
tarbase	hsa-miR-212-3p	LPIN3	64900	ENSG00000132793	validated
tarbase	hsa-miR-181a-5p	RBM38	55544	ENSG00000132819	validated
tarbase	hsa-miR-21-5p	OSER1	51526	ENSG00000132823	validated
tarbase	hsa-miR-15b-5p	SERINC3	10955	ENSG00000132824	validated
tarbase	hsa-miR-222-3p	SERINC3	10955	ENSG00000132824	validated
tarbase	hsa-miR-181a-5p	PPP1R3D	5509	ENSG00000132825	validated
tarbase	hsa-miR-181a-5p	AP3B1	8546	ENSG00000132842	validated
tarbase	hsa-miR-21-5p	AP3B1	8546	ENSG00000132842	validated
tarbase	hsa-miR-301a-3p	ZBED3	84327	ENSG00000132846	validated
tarbase	hsa-miR-18a-5p	PATJ	10207	ENSG00000132849	validated
tarbase	hsa-miR-15b-5p	SYT4	6860	ENSG00000132872	validated
tarbase	hsa-miR-181a-5p	SYT4	6860	ENSG00000132872	validated
tarbase	hsa-miR-106b-5p	DCTN4	51164	ENSG00000132912	validated
tarbase	hsa-miR-15b-5p	DCTN4	51164	ENSG00000132912	validated
tarbase	hsa-miR-212-3p	DCTN4	51164	ENSG00000132912	validated
tarbase	hsa-miR-21-5p	DCTN4	51164	ENSG00000132912	validated
tarbase	hsa-miR-582-5p	DCTN4	51164	ENSG00000132912	validated
tarbase	hsa-miR-181a-5p	DCTN4	51164	ENSG00000132912	validated
tarbase	hsa-miR-212-3p	ZMYM5	9205	ENSG00000132950	validated
tarbase	hsa-miR-106b-5p	USPL1	10208	ENSG00000132952	validated
tarbase	hsa-miR-181a-5p	USPL1	10208	ENSG00000132952	validated
tarbase	hsa-miR-212-3p	XPO4	64328	ENSG00000132953	validated
tarbase	hsa-miR-18a-5p	XPO4	64328	ENSG00000132953	validated
tarbase	hsa-miR-106b-5p	WASF3	10810	ENSG00000132970	validated
tarbase	hsa-miR-21-5p	RNF17	56163	ENSG00000132972	validated
tarbase	hsa-miR-181a-5p	CHRM3	1131	ENSG00000133019	validated
tarbase	hsa-miR-15b-5p	CHRM3	1131	ENSG00000133019	validated
tarbase	hsa-miR-181a-5p	MYH10	4628	ENSG00000133026	validated
tarbase	hsa-miR-21-5p	MYH10	4628	ENSG00000133026	validated
tarbase	hsa-miR-106b-5p	SCO1	6341	ENSG00000133028	validated
tarbase	hsa-miR-21-5p	SCO1	6341	ENSG00000133028	validated
tarbase	hsa-miR-296-5p	SCO1	6341	ENSG00000133028	validated
tarbase	hsa-miR-301a-3p	SCO1	6341	ENSG00000133028	validated
tarbase	hsa-miR-106b-5p	MPRIP	23164	ENSG00000133030	validated
tarbase	hsa-miR-106b-5p	PIK3C2B	5287	ENSG00000133056	validated
tarbase	hsa-miR-144-3p	DSTYK	25778	ENSG00000133059	validated
tarbase	hsa-miR-212-3p	CHIT1	1118	ENSG00000133063	validated
tarbase	hsa-miR-106b-5p	SLC41A1	254428	ENSG00000133065	validated
tarbase	hsa-miR-18a-5p	SLC41A1	254428	ENSG00000133065	validated
tarbase	hsa-miR-301a-3p	SLC41A1	254428	ENSG00000133065	validated
tarbase	hsa-miR-15b-5p	DCLK1	9201	ENSG00000133083	validated
tarbase	hsa-miR-106b-5p	SPART	23111	ENSG00000133104	validated
tarbase	hsa-miR-192-5p	SPART	23111	ENSG00000133104	validated
tarbase	hsa-miR-21-5p	SPART	23111	ENSG00000133104	validated
tarbase	hsa-miR-212-3p	EPSTI1	94240	ENSG00000133106	validated
tarbase	hsa-miR-181a-5p	POSTN	10631	ENSG00000133110	validated
tarbase	hsa-miR-21-5p	TPT1	7178	ENSG00000133112	validated
tarbase	hsa-miR-582-5p	GPALPP1	55425	ENSG00000133114	validated
tarbase	hsa-miR-106b-5p	RFC3	5983	ENSG00000133119	validated
tarbase	hsa-miR-181a-5p	RFC3	5983	ENSG00000133119	validated
tarbase	hsa-miR-15b-5p	STARD13	90627	ENSG00000133121	validated
tarbase	hsa-miR-15b-5p	IRS4	8471	ENSG00000133124	validated
tarbase	hsa-miR-192-5p	IRS4	8471	ENSG00000133124	validated
tarbase	hsa-miR-326	IRS4	8471	ENSG00000133124	validated
tarbase	hsa-miR-106b-5p	RNF128	79589	ENSG00000133135	validated
tarbase	hsa-miR-18a-5p	BEX1	55859	ENSG00000133169	validated
tarbase	hsa-miR-301a-3p	FAM104A	84923	ENSG00000133193	validated
tarbase	hsa-miR-15b-5p	SRRM1	10250	ENSG00000133226	validated
tarbase	hsa-miR-301a-3p	SLF1	84250	ENSG00000133302	validated
tarbase	hsa-miR-21-5p	CNDP2	55748	ENSG00000133313	validated

tarbase	hsa-miR-301a-3p	WDR74	54663	ENSG00000133316	validated
tarbase	hsa-miR-18a-5p	WDR74	54663	ENSG00000133316	validated
tarbase	hsa-miR-15b-5p	RTN3	10313	ENSG00000133318	validated
tarbase	hsa-miR-212-3p	PLAAT4	5920	ENSG00000133321	validated
tarbase	hsa-miR-21-5p	MYH11	4629	ENSG00000133392	validated
tarbase	hsa-miR-181a-5p	MED10	84246	ENSG00000133398	validated
tarbase	hsa-miR-15b-5p	PDZD2	23037	ENSG00000133401	validated
tarbase	hsa-miR-181a-5p	LARGE1	9215	ENSG00000133424	validated
tarbase	hsa-miR-21-5p	FAM83F	113828	ENSG00000133477	validated
tarbase	hsa-miR-212-3p	FAM83F	113828	ENSG00000133477	validated
tarbase	hsa-miR-18a-5p	MKRN1	23608	ENSG00000133606	validated
tarbase	hsa-miR-21-5p	MKRN1	23608	ENSG00000133606	validated
tarbase	hsa-miR-296-5p	AGAP3	116988	ENSG00000133612	validated
tarbase	hsa-miR-15b-5p	AGAP3	116988	ENSG00000133612	validated
tarbase	hsa-miR-582-5p	ACTR3B	57180	ENSG00000133627	validated
tarbase	hsa-miR-212-3p	ACTR3B	57180	ENSG00000133627	validated
tarbase	hsa-miR-106b-5p	BTG1	694	ENSG00000133639	validated
tarbase	hsa-miR-181a-5p	BTG1	694	ENSG00000133639	validated
tarbase	hsa-miR-106b-5p	ATP13A3	79572	ENSG00000133657	validated
tarbase	hsa-miR-18a-5p	ATP13A3	79572	ENSG00000133657	validated
tarbase	hsa-miR-212-3p	ATP13A3	79572	ENSG00000133657	validated
tarbase	hsa-miR-296-5p	ATP13A3	79572	ENSG00000133657	validated
tarbase	hsa-miR-301a-3p	ATP13A3	79572	ENSG00000133657	validated
tarbase	hsa-miR-106b-5p	IPO8	10526	ENSG00000133704	validated
tarbase	hsa-miR-15b-5p	IPO8	10526	ENSG00000133704	validated
tarbase	hsa-miR-301a-3p	IPO8	10526	ENSG00000133704	validated
tarbase	hsa-miR-582-3p	IPO8	10526	ENSG00000133704	validated
tarbase	hsa-miR-181a-5p	LARS1	51520	ENSG00000133706	validated
tarbase	hsa-miR-106b-5p	IMPA1	3612	ENSG00000133731	validated
tarbase	hsa-miR-15b-5p	E2F5	1875	ENSG00000133740	validated
tarbase	hsa-miR-212-3p	E2F5	1875	ENSG00000133740	validated
tarbase	hsa-miR-505-3p	E2F5	1875	ENSG00000133740	validated
tarbase	hsa-miR-15b-5p	CCDC59	29080	ENSG00000133773	validated
tarbase	hsa-miR-181a-5p	SWAP70	23075	ENSG00000133789	validated
tarbase	hsa-miR-212-3p	ARNTL	406	ENSG00000133794	validated
tarbase	hsa-miR-15b-5p	SBF2	81846	ENSG00000133812	validated
tarbase	hsa-miR-301a-3p	SBF2	81846	ENSG00000133812	validated
tarbase	hsa-miR-326	SBF2	81846	ENSG00000133812	validated
tarbase	hsa-miR-212-3p	SBF2	81846	ENSG00000133812	validated
tarbase	hsa-miR-15b-5p	RRAS2	22800	ENSG00000133818	validated
tarbase	hsa-miR-15b-5p	HSD17B4	3295	ENSG00000133835	validated
tarbase	hsa-miR-21-5p	ZFC3H1	196441	ENSG00000133858	validated
tarbase	hsa-miR-301a-3p	ZFC3H1	196441	ENSG00000133858	validated
tarbase	hsa-miR-21-5p	SARAF	51669	ENSG00000133872	validated
tarbase	hsa-miR-296-5p	SARAF	51669	ENSG00000133872	validated
tarbase	hsa-miR-192-5p	ERG28	11161	ENSG00000133935	validated
tarbase	hsa-miR-15b-5p	NUMB	8650	ENSG00000133961	validated
tarbase	hsa-miR-181a-5p	NUMB	8650	ENSG00000133961	validated
tarbase	hsa-miR-15b-5p	VRTN	55237	ENSG00000133980	validated
tarbase	hsa-miR-192-5p	COX16	51241	ENSG00000133983	validated
tarbase	hsa-miR-18a-5p	MED6	10001	ENSG00000133997	validated
tarbase	hsa-miR-582-5p	MED6	10001	ENSG00000133997	validated
tarbase	hsa-miR-181a-5p	EIF2S1	1965	ENSG00000134001	validated
tarbase	hsa-miR-212-3p	PEBP4	157310	ENSG00000134020	validated
tarbase	hsa-miR-106b-5p	MBD2	8932	ENSG00000134046	validated
tarbase	hsa-miR-18a-5p	MBD2	8932	ENSG00000134046	validated
tarbase	hsa-miR-301a-3p	MBD2	8932	ENSG00000134046	validated
tarbase	hsa-miR-106b-5p	CCNB1	891	ENSG00000134057	validated
tarbase	hsa-miR-181a-5p	CCNB1	891	ENSG00000134057	validated
tarbase	hsa-miR-18a-5p	CCNB1	891	ENSG00000134057	validated
tarbase	hsa-miR-301a-3p	CCNB1	891	ENSG00000134057	validated
tarbase	hsa-miR-582-3p	CCNB1	891	ENSG00000134057	validated
tarbase	hsa-miR-192-5p	THUMPD3	25917	ENSG00000134077	validated
tarbase	hsa-miR-582-5p	THUMPD3	25917	ENSG00000134077	validated
tarbase	hsa-miR-106b-5p	VHL	7428	ENSG00000134086	validated
tarbase	hsa-miR-15b-5p	VHL	7428	ENSG00000134086	validated
tarbase	hsa-miR-301a-3p	VHL	7428	ENSG00000134086	validated
tarbase	hsa-miR-181a-5p	BHLHE40	8553	ENSG00000134107	validated
tarbase	hsa-miR-144-3p	ARL8B	55207	ENSG00000134108	validated
tarbase	hsa-miR-15b-5p	ARL8B	55207	ENSG00000134108	validated
tarbase	hsa-miR-181a-5p	ARL8B	55207	ENSG00000134108	validated

tarbase	hsa-miR-301a-3p	ARL8B	55207	ENSG00000134108	validated
tarbase	hsa-miR-106b-5p	ARL8B	55207	ENSG00000134108	validated
tarbase	hsa-miR-144-3p	EDEM1	9695	ENSG00000134109	validated
tarbase	hsa-miR-15b-5p	CHL1	10752	ENSG00000134121	validated
tarbase	hsa-miR-15b-5p	KATNBL1	79768	ENSG00000134152	validated
tarbase	hsa-miR-181a-5p	KATNBL1	79768	ENSG00000134152	validated
tarbase	hsa-miR-15b-5p	EMC7	56851	ENSG00000134153	validated
tarbase	hsa-miR-21-5p	TSPAN2	10100	ENSG00000134198	validated
tarbase	hsa-miR-212-3p	VAV3	10451	ENSG00000134215	validated
tarbase	hsa-miR-21-5p	PSRC1	84722	ENSG00000134222	validated
tarbase	hsa-miR-181a-5p	PTGFRN	5738	ENSG00000134247	validated
tarbase	hsa-miR-18a-5p	PTGFRN	5738	ENSG00000134247	validated
tarbase	hsa-miR-301a-3p	PTGFRN	5738	ENSG00000134247	validated
tarbase	hsa-miR-106b-5p	LAMTOR5	10542	ENSG00000134248	validated
tarbase	hsa-miR-21-5p	LAMTOR5	10542	ENSG00000134248	validated
tarbase	hsa-miR-106b-5p	NOTCH2	4853	ENSG00000134250	validated
tarbase	hsa-miR-18a-5p	NOTCH2	4853	ENSG00000134250	validated
tarbase	hsa-miR-192-5p	NOTCH2	4853	ENSG00000134250	validated
tarbase	hsa-miR-222-3p	NOTCH2	4853	ENSG00000134250	validated
tarbase	hsa-miR-505-3p	NOTCH2	4853	ENSG00000134250	validated
tarbase	hsa-miR-15b-5p	VTCN1	79679	ENSG00000134258	validated
tarbase	hsa-miR-212-3p	NAPG	8774	ENSG00000134265	validated
tarbase	hsa-miR-106b-5p	NAPG	8774	ENSG00000134265	validated
tarbase	hsa-miR-301a-3p	PPHLN1	51535	ENSG00000134283	validated
tarbase	hsa-miR-144-3p	ARF3	377	ENSG00000134287	validated
tarbase	hsa-miR-15b-5p	ARF3	377	ENSG00000134287	validated
tarbase	hsa-miR-181a-5p	ARF3	377	ENSG00000134287	validated
tarbase	hsa-miR-192-5p	ARF3	377	ENSG00000134287	validated
tarbase	hsa-miR-296-5p	ARF3	377	ENSG00000134287	validated
tarbase	hsa-miR-106b-5p	SLC38A2	54407	ENSG00000134294	validated
tarbase	hsa-miR-144-3p	SLC38A2	54407	ENSG00000134294	validated
tarbase	hsa-miR-21-5p	SLC38A2	54407	ENSG00000134294	validated
tarbase	hsa-miR-582-3p	SLC38A2	54407	ENSG00000134294	validated
tarbase	hsa-miR-212-3p	KIDINS220	57498	ENSG00000134313	validated
tarbase	hsa-miR-106b-5p	ROCK2	9475	ENSG00000134318	validated
tarbase	hsa-miR-21-5p	ROCK2	9475	ENSG00000134318	validated
tarbase	hsa-miR-301a-3p	ROCK2	9475	ENSG00000134318	validated
tarbase	hsa-miR-212-3p	RSAD2	91543	ENSG00000134321	validated
tarbase	hsa-miR-21-5p	LPIN1	23175	ENSG00000134324	validated
tarbase	hsa-miR-301a-3p	LPIN1	23175	ENSG00000134324	validated
tarbase	hsa-miR-106b-5p	LPIN1	23175	ENSG00000134324	validated
tarbase	hsa-miR-212-3p	CMPK2	129607	ENSG00000134326	validated
tarbase	hsa-miR-21-5p	LDHA	3939	ENSG00000134333	validated
tarbase	hsa-miR-222-3p	LDHA	3939	ENSG00000134333	validated
tarbase	hsa-miR-301a-3p	LDHA	3939	ENSG00000134333	validated
tarbase	hsa-miR-106b-5p	IL6ST	3572	ENSG00000134352	validated
tarbase	hsa-miR-15b-5p	IL6ST	3572	ENSG00000134352	validated
tarbase	hsa-miR-21-5p	IL6ST	3572	ENSG00000134352	validated
tarbase	hsa-miR-301a-3p	IL6ST	3572	ENSG00000134352	validated
tarbase	hsa-miR-181a-5p	IL6ST	3572	ENSG00000134352	validated
tarbase	hsa-miR-505-3p	IL6ST	3572	ENSG00000134352	validated
tarbase	hsa-miR-15b-5p	NAV1	89796	ENSG00000134369	validated
tarbase	hsa-miR-296-5p	NAV1	89796	ENSG00000134369	validated
tarbase	hsa-miR-106b-5p	CDC73	79577	ENSG00000134371	validated
tarbase	hsa-miR-15b-5p	CDC73	79577	ENSG00000134371	validated
tarbase	hsa-miR-18a-5p	CDC73	79577	ENSG00000134371	validated
tarbase	hsa-miR-21-5p	CDC73	79577	ENSG00000134371	validated
tarbase	hsa-miR-181a-5p	CDC73	79577	ENSG00000134371	validated
tarbase	hsa-miR-18a-5p	TIMM17A	10440	ENSG00000134375	validated
tarbase	hsa-miR-15b-5p	NARS1	4677	ENSG00000134440	validated
tarbase	hsa-miR-15b-5p	RELCH	57614	ENSG00000134444	validated
tarbase	hsa-miR-301a-3p	RELCH	57614	ENSG00000134444	validated
tarbase	hsa-miR-222-3p	RBM17	84991	ENSG00000134453	validated
tarbase	hsa-miR-106b-5p	ANKRD16	54522	ENSG00000134461	validated
tarbase	hsa-miR-18a-5p	CCNH	902	ENSG00000134480	validated
tarbase	hsa-miR-21-5p	CCNH	902	ENSG00000134480	validated
tarbase	hsa-miR-181a-5p	TMEM241	85019	ENSG00000134490	validated
tarbase	hsa-miR-18a-5p	CABLES1	91768	ENSG00000134508	validated
tarbase	hsa-miR-21-5p	DOCK2	1794	ENSG00000134516	validated
tarbase	hsa-miR-296-5p	EMP1	2012	ENSG00000134531	validated
tarbase	hsa-miR-106b-5p	SOX5	6660	ENSG00000134532	validated

tarbase	hsa-miR-15b-5p	SOX5	6660	ENSG00000134532	validated
tarbase	hsa-miR-15b-5p	ACP2	53	ENSG00000134575	validated
tarbase	hsa-miR-212-3p	RAB33A	9363	ENSG00000134594	validated
tarbase	hsa-miR-192-5p	STK26	51765	ENSG00000134602	validated
tarbase	hsa-miR-212-3p	PIWIL4	143689	ENSG00000134627	validated
tarbase	hsa-miR-144-3p	PUM1	9698	ENSG00000134644	validated
tarbase	hsa-miR-15b-5p	PUM1	9698	ENSG00000134644	validated
tarbase	hsa-miR-18a-5p	PUM1	9698	ENSG00000134644	validated
tarbase	hsa-miR-212-3p	PUM1	9698	ENSG00000134644	validated
tarbase	hsa-miR-582-5p	PUM1	9698	ENSG00000134644	validated
tarbase	hsa-miR-181a-5p	YARS1	8565	ENSG00000134684	validated
tarbase	hsa-miR-18a-5p	YARS1	8565	ENSG00000134684	validated
tarbase	hsa-miR-326	YARS1	8565	ENSG00000134684	validated
tarbase	hsa-miR-18a-5p	PHC2	1912	ENSG00000134686	validated
tarbase	hsa-miR-222-3p	PHC2	1912	ENSG00000134686	validated
tarbase	hsa-miR-181a-5p	AGO4	192670	ENSG00000134698	validated
tarbase	hsa-miR-181a-5p	HOOK1	51361	ENSG00000134709	validated
tarbase	hsa-miR-18a-5p	BTF3L4	91408	ENSG00000134717	validated
tarbase	hsa-miR-21-5p	BTF3L4	91408	ENSG00000134717	validated
tarbase	hsa-miR-15b-5p	TUT4	23318	ENSG00000134744	validated
tarbase	hsa-miR-212-3p	DSC2	1824	ENSG00000134755	validated
tarbase	hsa-miR-21-5p	DSC2	1824	ENSG00000134755	validated
tarbase	hsa-miR-301a-3p	DSC2	1824	ENSG00000134755	validated
tarbase	hsa-miR-106b-5p	RNF138	51444	ENSG00000134758	validated
tarbase	hsa-miR-181a-5p	DSC3	1825	ENSG00000134762	validated
tarbase	hsa-miR-15b-5p	DTNA	1837	ENSG00000134769	validated
tarbase	hsa-miR-18a-5p	TPGS2	25941	ENSG00000134779	validated
tarbase	hsa-miR-15b-5p	FADS2	9415	ENSG00000134824	validated
tarbase	hsa-miR-15b-5p	TMEM258	746	ENSG00000134825	validated
tarbase	hsa-miR-505-3p	CLOCK	9575	ENSG00000134852	validated
tarbase	hsa-miR-106b-5p	COL4A2	1284	ENSG00000134871	validated
tarbase	hsa-miR-15b-5p	DZIP1	22873	ENSG00000134874	validated
tarbase	hsa-miR-21-5p	ARHGAP32	9743	ENSG00000134909	validated
tarbase	hsa-miR-15b-5p	STT3A	3703	ENSG00000134910	validated
tarbase	hsa-miR-181a-5p	STT3A	3703	ENSG00000134910	validated
tarbase	hsa-miR-212-3p	ETS1	2113	ENSG00000134954	validated
tarbase	hsa-miR-15b-5p	TMED7	51014	ENSG00000134970	validated
tarbase	hsa-miR-18a-5p	TMED7	51014	ENSG00000134970	validated
tarbase	hsa-miR-192-5p	TMED7	51014	ENSG00000134970	validated
tarbase	hsa-miR-301a-3p	TMED7	51014	ENSG00000134970	validated
tarbase	hsa-miR-181a-5p	TMED7	51014	ENSG00000134970	validated
tarbase	hsa-miR-181a-5p	APC	324	ENSG00000134982	validated
tarbase	hsa-miR-15b-5p	APC	324	ENSG00000134982	validated
tarbase	hsa-miR-301a-3p	NREP	9315	ENSG00000134986	validated
tarbase	hsa-miR-326	NREP	9315	ENSG00000134986	validated
tarbase	hsa-miR-106b-5p	WDR36	134430	ENSG00000134987	validated
tarbase	hsa-miR-301a-3p	WDR36	134430	ENSG00000134987	validated
tarbase	hsa-miR-181a-5p	RFK	55312	ENSG00000135002	validated
tarbase	hsa-miR-106b-5p	UBQLN1	29979	ENSG00000135018	validated
tarbase	hsa-miR-212-3p	UBQLN1	29979	ENSG00000135018	validated
tarbase	hsa-miR-582-5p	UBQLN1	29979	ENSG00000135018	validated
tarbase	hsa-miR-21-5p	ANXA1	301	ENSG00000135046	validated
tarbase	hsa-miR-222-3p	ANXA1	301	ENSG00000135046	validated
tarbase	hsa-miR-15b-5p	CTSL	1514	ENSG00000135047	validated
tarbase	hsa-miR-15b-5p	CEMIP2	23670	ENSG00000135048	validated
tarbase	hsa-miR-106b-5p	PSAT1	29968	ENSG00000135069	validated
tarbase	hsa-miR-181a-5p	ISCA1	81689	ENSG00000135070	validated
tarbase	hsa-miR-106b-5p	TAOK3	51347	ENSG00000135090	validated
tarbase	hsa-miR-106b-5p	USP30	84749	ENSG00000135093	validated
tarbase	hsa-miR-15b-5p	FBXO21	23014	ENSG00000135108	validated
tarbase	hsa-miR-181a-5p	FBXO21	23014	ENSG00000135108	validated
tarbase	hsa-miR-212-3p	FBXO21	23014	ENSG00000135108	validated
tarbase	hsa-miR-301a-3p	FBXO21	23014	ENSG00000135108	validated
tarbase	hsa-miR-582-3p	FBXO21	23014	ENSG00000135108	validated
tarbase	hsa-miR-212-3p	TBX3	6926	ENSG00000135111	validated
tarbase	hsa-miR-212-3p	OASL	8638	ENSG00000135114	validated
tarbase	hsa-miR-21-5p	HRK	8739	ENSG00000135116	validated
tarbase	hsa-miR-301a-3p	HRK	8739	ENSG00000135116	validated
tarbase	hsa-miR-106b-5p	P2RX4	5025	ENSG00000135124	validated
tarbase	hsa-miR-222-3p	P2RX4	5025	ENSG00000135124	validated
tarbase	hsa-miR-582-3p	TRAFD1	10906	ENSG00000135148	validated

tarbase	hsa-miR-106b-5p	DMTF1	9988	ENSG00000135164	validated
tarbase	hsa-miR-21-5p	TMEM243	79161	ENSG00000135185	validated
tarbase	hsa-miR-212-3p	CCDC146	57639	ENSG00000135205	validated
tarbase	hsa-miR-181a-5p	HILPDA	29923	ENSG00000135245	validated
tarbase	hsa-miR-106b-5p	TES	26136	ENSG00000135269	validated
tarbase	hsa-miR-181a-5p	TES	26136	ENSG00000135269	validated
tarbase	hsa-miR-301a-3p	TES	26136	ENSG00000135269	validated
tarbase	hsa-miR-18a-5p	TES	26136	ENSG00000135269	validated
tarbase	hsa-miR-301a-3p	MDFIC	29969	ENSG00000135272	validated
tarbase	hsa-miR-15b-5p	KHDC1	80759	ENSG00000135314	validated
tarbase	hsa-miR-192-5p	CEP162	22832	ENSG00000135315	validated
tarbase	hsa-miR-106b-5p	SYNCRIP	10492	ENSG00000135316	validated
tarbase	hsa-miR-212-3p	SYNCRIP	10492	ENSG00000135316	validated
tarbase	hsa-miR-301a-3p	SYNCRIP	10492	ENSG00000135316	validated
tarbase	hsa-miR-582-5p	SYNCRIP	10492	ENSG00000135316	validated
tarbase	hsa-miR-181a-5p	SYNCRIP	10492	ENSG00000135316	validated
tarbase	hsa-miR-181a-5p	SNX14	57231	ENSG00000135317	validated
tarbase	hsa-miR-21-5p	NT5E	4907	ENSG00000135318	validated
tarbase	hsa-miR-106b-5p	EPHA7	2045	ENSG00000135333	validated
tarbase	hsa-miR-18a-5p	EPHA7	2045	ENSG00000135333	validated
tarbase	hsa-miR-212-3p	EPHA7	2045	ENSG00000135333	validated
tarbase	hsa-miR-301a-3p	EPHA7	2045	ENSG00000135333	validated
tarbase	hsa-miR-181a-5p	AKIRIN2	55122	ENSG00000135334	validated
tarbase	hsa-miR-582-3p	AKIRIN2	55122	ENSG00000135334	validated
tarbase	hsa-miR-582-5p	AKIRIN2	55122	ENSG00000135334	validated
tarbase	hsa-miR-505-3p	LCA5	167691	ENSG00000135338	validated
tarbase	hsa-miR-212-3p	LCA5	167691	ENSG00000135338	validated
tarbase	hsa-miR-181a-5p	MAP3K7	6885	ENSG00000135341	validated
tarbase	hsa-miR-212-3p	CGA	1081	ENSG00000135346	validated
tarbase	hsa-miR-181a-5p	PRR5L	79899	ENSG00000135362	validated
tarbase	hsa-miR-181a-5p	LMO2	4005	ENSG00000135363	validated
tarbase	hsa-miR-212-3p	LMO2	4005	ENSG00000135363	validated
tarbase	hsa-miR-181a-5p	PRRG4	79056	ENSG00000135378	validated
tarbase	hsa-miR-15b-5p	CAPRIN1	4076	ENSG00000135387	validated
tarbase	hsa-miR-212-3p	CAPRIN1	4076	ENSG00000135387	validated
tarbase	hsa-miR-301a-3p	CAPRIN1	4076	ENSG00000135387	validated
tarbase	hsa-miR-181a-5p	CAPRIN1	4076	ENSG00000135387	validated
tarbase	hsa-miR-18a-5p	GDF11	10220	ENSG00000135414	validated
tarbase	hsa-miR-212-3p	RDH5	5959	ENSG00000135437	validated
tarbase	hsa-miR-18a-5p	CDK4	1019	ENSG00000135446	validated
tarbase	hsa-miR-296-5p	CDK4	1019	ENSG00000135446	validated
tarbase	hsa-miR-301a-3p	TROAP	10024	ENSG00000135451	validated
tarbase	hsa-miR-21-5p	TSPAN31	6302	ENSG00000135452	validated
tarbase	hsa-miR-15b-5p	TFCP2	7024	ENSG00000135457	validated
tarbase	hsa-miR-505-3p	HNRNPA1	3178	ENSG00000135486	validated
tarbase	hsa-miR-15b-5p	ACVR1B	91	ENSG00000135503	validated
tarbase	hsa-miR-296-5p	ACVR1B	91	ENSG00000135503	validated
tarbase	hsa-miR-326	ACVR1B	91	ENSG00000135503	validated
tarbase	hsa-miR-582-5p	ACVR1B	91	ENSG00000135503	validated
tarbase	hsa-miR-106b-5p	LTV1	84946	ENSG00000135521	validated
tarbase	hsa-miR-21-5p	LTV1	84946	ENSG00000135521	validated
tarbase	hsa-miR-15b-5p	MAP7	9053	ENSG00000135525	validated
tarbase	hsa-miR-181a-5p	MAP7	9053	ENSG00000135525	validated
tarbase	hsa-miR-21-5p	MAP7	9053	ENSG00000135525	validated
tarbase	hsa-miR-106b-5p	CD164	8763	ENSG00000135535	validated
tarbase	hsa-miR-15b-5p	CD164	8763	ENSG00000135535	validated
tarbase	hsa-miR-181a-5p	CD164	8763	ENSG00000135535	validated
tarbase	hsa-miR-18a-5p	CD164	8763	ENSG00000135535	validated
tarbase	hsa-miR-212-3p	CD164	8763	ENSG00000135535	validated
tarbase	hsa-miR-21-5p	CD164	8763	ENSG00000135535	validated
tarbase	hsa-miR-222-3p	CD164	8763	ENSG00000135535	validated
tarbase	hsa-miR-18a-5p	NHSL1	57224	ENSG00000135540	validated
tarbase	hsa-miR-15b-5p	REPS1	85021	ENSG00000135597	validated
tarbase	hsa-miR-296-5p	PRADC1	84279	ENSG00000135617	validated
tarbase	hsa-miR-181a-5p	CCT7	10574	ENSG00000135624	validated
tarbase	hsa-miR-18a-5p	CCT7	10574	ENSG00000135624	validated
tarbase	hsa-miR-15b-5p	RAB11FIP5	26056	ENSG00000135631	validated
tarbase	hsa-miR-106b-5p	SMYD5	10322	ENSG00000135632	validated
tarbase	hsa-miR-15b-5p	SMYD5	10322	ENSG00000135632	validated
tarbase	hsa-miR-106b-5p	USP15	9958	ENSG00000135655	validated
tarbase	hsa-miR-21-5p	USP15	9958	ENSG00000135655	validated

tarbase	hsa-miR-582-3p	USP15	9958	ENSG00000135655	validated
tarbase	hsa-miR-15b-5p	GNS	2799	ENSG00000135677	validated
tarbase	hsa-miR-222-3p	GNS	2799	ENSG00000135677	validated
tarbase	hsa-miR-301a-3p	GNS	2799	ENSG00000135677	validated
tarbase	hsa-miR-15b-5p	MDM2	4193	ENSG00000135679	validated
tarbase	hsa-miR-18a-5p	MDM2	4193	ENSG00000135679	validated
tarbase	hsa-miR-192-5p	MDM2	4193	ENSG00000135679	validated
tarbase	hsa-miR-212-3p	MDM2	4193	ENSG00000135679	validated
tarbase	hsa-miR-21-5p	MDM2	4193	ENSG00000135679	validated
tarbase	hsa-miR-505-3p	MDM2	4193	ENSG00000135679	validated
tarbase	hsa-miR-582-5p	MDM2	4193	ENSG00000135679	validated
tarbase	hsa-miR-181a-5p	MPHOSPH6	10200	ENSG00000135698	validated
tarbase	hsa-miR-296-5p	DYNC1LI2	1783	ENSG00000135720	validated
tarbase	hsa-miR-15b-5p	DYNC1LI2	1783	ENSG00000135720	validated
tarbase	hsa-miR-15b-5p	AGT	183	ENSG00000135744	validated
tarbase	hsa-miR-192-5p	PCNX2	80003	ENSG00000135749	validated
tarbase	hsa-miR-106b-5p	KCNK1	3775	ENSG00000135750	validated
tarbase	hsa-miR-144-3p	URB2	9816	ENSG00000135763	validated
tarbase	hsa-miR-222-3p	URB2	9816	ENSG00000135763	validated
tarbase	hsa-miR-15b-5p	EGLN1	54583	ENSG00000135766	validated
tarbase	hsa-miR-181a-5p	EGLN1	54583	ENSG00000135766	validated
tarbase	hsa-miR-106b-5p	CAPN9	10753	ENSG00000135773	validated
tarbase	hsa-miR-18a-5p	TAF5L	27097	ENSG00000135801	validated
tarbase	hsa-miR-301a-3p	TAF5L	27097	ENSG00000135801	validated
tarbase	hsa-miR-582-3p	GLUL	2752	ENSG00000135821	validated
tarbase	hsa-miR-18a-5p	STX6	10228	ENSG00000135823	validated
tarbase	hsa-miR-106b-5p	RNASEL	6041	ENSG00000135828	validated
tarbase	hsa-miR-21-5p	RNASEL	6041	ENSG00000135828	validated
tarbase	hsa-miR-15b-5p	DHX9	1660	ENSG00000135829	validated
tarbase	hsa-miR-326	DHX9	1660	ENSG00000135829	validated
tarbase	hsa-miR-144-3p	CEP350	9857	ENSG00000135837	validated
tarbase	hsa-miR-181a-5p	CEP350	9857	ENSG00000135837	validated
tarbase	hsa-miR-582-5p	CEP350	9857	ENSG00000135837	validated
tarbase	hsa-miR-301a-3p	NIBAN1	116496	ENSG00000135842	validated
tarbase	hsa-miR-212-3p	NIBAN1	116496	ENSG00000135842	validated
tarbase	hsa-miR-181a-5p	LAMC1	3915	ENSG00000135862	validated
tarbase	hsa-miR-21-5p	LAMC1	3915	ENSG00000135862	validated
tarbase	hsa-miR-106b-5p	RC3H1	149041	ENSG00000135870	validated
tarbase	hsa-miR-181a-5p	RC3H1	149041	ENSG00000135870	validated
tarbase	hsa-miR-18a-5p	RC3H1	149041	ENSG00000135870	validated
tarbase	hsa-miR-212-3p	SP110	3431	ENSG00000135899	validated
tarbase	hsa-miR-15b-5p	MRPL44	65080	ENSG00000135900	validated
tarbase	hsa-miR-181a-5p	DOCK10	55619	ENSG00000135905	validated
tarbase	hsa-miR-106b-5p	USP37	57695	ENSG00000135913	validated
tarbase	hsa-miR-18a-5p	USP37	57695	ENSG00000135913	validated
tarbase	hsa-miR-106b-5p	ITM2C	81618	ENSG00000135916	validated
tarbase	hsa-miR-15b-5p	ITM2C	81618	ENSG00000135916	validated
tarbase	hsa-miR-296-5p	ITM2C	81618	ENSG00000135916	validated
tarbase	hsa-miR-181a-5p	ITM2C	81618	ENSG00000135916	validated
tarbase	hsa-miR-106b-5p	SERPINE2	5270	ENSG00000135919	validated
tarbase	hsa-miR-18a-5p	TMBIM1	64114	ENSG00000135926	validated
tarbase	hsa-miR-301a-3p	EIF4E2	9470	ENSG00000135930	validated
tarbase	hsa-miR-212-3p	ARMC9	80210	ENSG00000135931	validated
tarbase	hsa-miR-15b-5p	CAB39	51719	ENSG00000135932	validated
tarbase	hsa-miR-181a-5p	CAB39	51719	ENSG00000135932	validated
tarbase	hsa-miR-301a-3p	COX5B	1329	ENSG00000135940	validated
tarbase	hsa-miR-106b-5p	MFSD9	84804	ENSG00000135953	validated
tarbase	hsa-miR-15b-5p	TMEM127	55654	ENSG00000135956	validated
tarbase	hsa-miR-181a-5p	TMEM127	55654	ENSG00000135956	validated
tarbase	hsa-miR-222-3p	TMEM127	55654	ENSG00000135956	validated
tarbase	hsa-miR-582-3p	TMEM127	55654	ENSG00000135956	validated
tarbase	hsa-miR-21-5p	TGFBRAP1	9392	ENSG00000135966	validated
tarbase	hsa-miR-301a-3p	TGFBRAP1	9392	ENSG00000135966	validated
tarbase	hsa-miR-326	MRPS9	64965	ENSG00000135972	validated
tarbase	hsa-miR-106b-5p	C2orf49	79074	ENSG00000135974	validated
tarbase	hsa-miR-181a-5p	EPC2	26122	ENSG00000135999	validated
tarbase	hsa-miR-301a-3p	EPC2	26122	ENSG00000135999	validated
tarbase	hsa-miR-15b-5p	USP44	84101	ENSG00000136014	validated
tarbase	hsa-miR-212-3p	USP44	84101	ENSG00000136014	validated
tarbase	hsa-miR-212-3p	SCYL2	55681	ENSG00000136021	validated
tarbase	hsa-miR-106b-5p	CKAP4	10970	ENSG00000136026	validated

tarbase	hsa-miR-21-5p	PWP1	11137	ENSG00000136045	validated
tarbase	hsa-miR-15b-5p	SLC41A2	84102	ENSG00000136052	validated
tarbase	hsa-miR-181a-5p	FLNB	2317	ENSG00000136068	validated
tarbase	hsa-miR-296-5p	FLNB	2317	ENSG00000136068	validated
tarbase	hsa-miR-582-3p	FLNB	2317	ENSG00000136068	validated
tarbase	hsa-miR-582-5p	FLNB	2317	ENSG00000136068	validated
tarbase	hsa-miR-296-5p	NEK3	4752	ENSG00000136098	validated
tarbase	hsa-miR-181a-5p	PCDH8	5100	ENSG00000136099	validated
tarbase	hsa-miR-15b-5p	VPS36	51028	ENSG00000136100	validated
tarbase	hsa-miR-106b-5p	RNASEH2B	79621	ENSG00000136104	validated
tarbase	hsa-miR-18a-5p	CKAP2	26586	ENSG00000136108	validated
tarbase	hsa-miR-301a-3p	BORA	79866	ENSG00000136122	validated
tarbase	hsa-miR-106b-5p	LRCH1	23143	ENSG00000136141	validated
tarbase	hsa-miR-15b-5p	SUCLA2	8803	ENSG00000136143	validated
tarbase	hsa-miR-15b-5p	COG3	83548	ENSG00000136152	validated
tarbase	hsa-miR-181a-5p	COG3	83548	ENSG00000136152	validated
tarbase	hsa-miR-18a-5p	COG3	83548	ENSG00000136152	validated
tarbase	hsa-miR-15b-5p	LMO7	4008	ENSG00000136153	validated
tarbase	hsa-miR-301a-3p	ITM2B	9445	ENSG00000136156	validated
tarbase	hsa-miR-326	ITM2B	9445	ENSG00000136156	validated
tarbase	hsa-miR-106b-5p	EDNRB	1910	ENSG00000136160	validated
tarbase	hsa-miR-181a-5p	LCP1	3936	ENSG00000136167	validated
tarbase	hsa-miR-15b-5p	SCRN1	9805	ENSG00000136193	validated
tarbase	hsa-miR-212-3p	SCRN1	9805	ENSG00000136193	validated
tarbase	hsa-miR-15b-5p	TNS3	64759	ENSG00000136205	validated
tarbase	hsa-miR-181a-5p	IGF2BP3	10643	ENSG00000136231	validated
tarbase	hsa-miR-15b-5p	RAC1	5879	ENSG00000136238	validated
tarbase	hsa-miR-505-3p	RAC1	5879	ENSG00000136238	validated
tarbase	hsa-miR-15b-5p	KDELR2	11014	ENSG00000136240	validated
tarbase	hsa-miR-181a-5p	KDELR2	11014	ENSG00000136240	validated
tarbase	hsa-miR-222-3p	KDELR2	11014	ENSG00000136240	validated
tarbase	hsa-miR-106b-5p	NUP42	11097	ENSG00000136243	validated
tarbase	hsa-miR-192-5p	IL6	3569	ENSG00000136244	validated
tarbase	hsa-miR-21-5p	IL6	3569	ENSG00000136244	validated
tarbase	hsa-miR-144-3p	ZDHHC4	55146	ENSG00000136247	validated
tarbase	hsa-miR-15b-5p	ZDHHC4	55146	ENSG00000136247	validated
tarbase	hsa-miR-15b-5p	BZW2	28969	ENSG00000136261	validated
tarbase	hsa-miR-326	DGKB	1607	ENSG00000136267	validated
tarbase	hsa-miR-181a-5p	DGKB	1607	ENSG00000136267	validated
tarbase	hsa-miR-582-3p	DDX56	54606	ENSG00000136271	validated
tarbase	hsa-miR-106b-5p	HUS1	3364	ENSG00000136273	validated
tarbase	hsa-miR-181a-5p	DBNL	28988	ENSG00000136279	validated
tarbase	hsa-miR-212-3p	DBNL	28988	ENSG00000136279	validated
tarbase	hsa-miR-15b-5p	MYO1G	64005	ENSG00000136286	validated
tarbase	hsa-miR-15b-5p	TTYH3	80727	ENSG00000136295	validated
tarbase	hsa-miR-15b-5p	CIDEB	27141	ENSG00000136305	validated
tarbase	hsa-miR-181a-5p	ABHD17C	58489	ENSG00000136379	validated
tarbase	hsa-miR-144-3p	IREB2	3658	ENSG00000136381	validated
tarbase	hsa-miR-15b-5p	IREB2	3658	ENSG00000136381	validated
tarbase	hsa-miR-505-3p	IREB2	3658	ENSG00000136381	validated
tarbase	hsa-miR-222-3p	ALPK3	57538	ENSG00000136383	validated
tarbase	hsa-miR-106b-5p	CALCOCO2	10241	ENSG00000136436	validated
tarbase	hsa-miR-181a-5p	CALCOCO2	10241	ENSG00000136436	validated
tarbase	hsa-miR-21-5p	NMT1	4836	ENSG00000136448	validated
tarbase	hsa-miR-106b-5p	NMT1	4836	ENSG00000136448	validated
tarbase	hsa-miR-106b-5p	SRSF1	6426	ENSG00000136450	validated
tarbase	hsa-miR-212-3p	SRSF1	6426	ENSG00000136450	validated
tarbase	hsa-miR-301a-3p	SRSF1	6426	ENSG00000136450	validated
tarbase	hsa-miR-181a-5p	SRSF1	6426	ENSG00000136450	validated
tarbase	hsa-miR-106b-5p	VEZF1	7716	ENSG00000136451	validated
tarbase	hsa-miR-212-3p	VEZF1	7716	ENSG00000136451	validated
tarbase	hsa-miR-222-3p	VEZF1	7716	ENSG00000136451	validated
tarbase	hsa-miR-301a-3p	VEZF1	7716	ENSG00000136451	validated
tarbase	hsa-miR-582-5p	TEX2	55852	ENSG00000136478	validated
tarbase	hsa-miR-15b-5p	DCAF7	10238	ENSG00000136485	validated
tarbase	hsa-miR-21-5p	DCAF7	10238	ENSG00000136485	validated
tarbase	hsa-miR-296-5p	DCAF7	10238	ENSG00000136485	validated
tarbase	hsa-miR-301a-3p	DCAF7	10238	ENSG00000136485	validated
tarbase	hsa-miR-181a-5p	DCAF7	10238	ENSG00000136485	validated
tarbase	hsa-miR-296-5p	LIMD2	80774	ENSG00000136490	validated
tarbase	hsa-miR-18a-5p	BRIP1	83990	ENSG00000136492	validated

tarbase	hsa-miR-21-5p	KAT7	11143	ENSG00000136504	validated
tarbase	hsa-miR-212-3p	RTP4	64108	ENSG00000136514	validated
tarbase	hsa-miR-582-5p	NDUFB5	4711	ENSG00000136521	validated
tarbase	hsa-miR-106b-5p	MARCHF7	64844	ENSG00000136536	validated
tarbase	hsa-miR-15b-5p	MARCHF7	64844	ENSG00000136536	validated
tarbase	hsa-miR-582-5p	MARCHF7	64844	ENSG00000136536	validated
tarbase	hsa-miR-144-3p	SKIL	6498	ENSG00000136603	validated
tarbase	hsa-miR-15b-5p	SKIL	6498	ENSG00000136603	validated
tarbase	hsa-miR-21-5p	SKIL	6498	ENSG00000136603	validated
tarbase	hsa-miR-582-5p	SKIL	6498	ENSG00000136603	validated
tarbase	hsa-miR-106b-5p	EPRS1	2058	ENSG00000136628	validated
tarbase	hsa-miR-21-5p	EPRS1	2058	ENSG00000136628	validated
tarbase	hsa-miR-21-5p	IL10	3586	ENSG00000136634	validated
tarbase	hsa-miR-212-3p	KCTD3	51133	ENSG00000136636	validated
tarbase	hsa-miR-106b-5p	RPS6KC1	26750	ENSG00000136643	validated
tarbase	hsa-miR-181a-5p	WDR33	55339	ENSG00000136709	validated
tarbase	hsa-miR-18a-5p	WDR33	55339	ENSG00000136709	validated
tarbase	hsa-miR-301a-3p	WDR33	55339	ENSG00000136709	validated
tarbase	hsa-miR-582-5p	WDR33	55339	ENSG00000136709	validated
tarbase	hsa-miR-326	SAP130	79595	ENSG00000136715	validated
tarbase	hsa-miR-192-5p	UGGT1	56886	ENSG00000136731	validated
tarbase	hsa-miR-181a-5p	STAM	8027	ENSG00000136738	validated
tarbase	hsa-miR-181a-5p	GAD2	2572	ENSG00000136750	validated
tarbase	hsa-miR-106b-5p	ABI1	10006	ENSG00000136754	validated
tarbase	hsa-miR-106b-5p	TXN	7295	ENSG00000136810	validated
tarbase	hsa-miR-15b-5p	ECPAS	23392	ENSG00000136813	validated
tarbase	hsa-miR-222-3p	ECPAS	23392	ENSG00000136813	validated
tarbase	hsa-miR-18a-5p	TOR1B	27348	ENSG00000136816	validated
tarbase	hsa-miR-181a-5p	SMC2	10592	ENSG00000136824	validated
tarbase	hsa-miR-18a-5p	TOR1A	1861	ENSG00000136827	validated
tarbase	hsa-miR-106b-5p	TOR1A	1861	ENSG00000136827	validated
tarbase	hsa-miR-212-3p	NIBAN2	64855	ENSG00000136830	validated
tarbase	hsa-miR-212-3p	TMOD1	7111	ENSG00000136842	validated
tarbase	hsa-miR-18a-5p	SLC2A8	29988	ENSG00000136856	validated
tarbase	hsa-miR-192-5p	CDK5RAP2	55755	ENSG00000136861	validated
tarbase	hsa-miR-582-3p	ZFP37	7539	ENSG00000136866	validated
tarbase	hsa-miR-212-3p	ZFP37	7539	ENSG00000136866	validated
tarbase	hsa-miR-15b-5p	SLC31A1	1317	ENSG00000136868	validated
tarbase	hsa-miR-181a-5p	SLC31A1	1317	ENSG00000136868	validated
tarbase	hsa-miR-505-3p	SLC31A1	1317	ENSG00000136868	validated
tarbase	hsa-miR-106b-5p	SLC31A1	1317	ENSG00000136868	validated
tarbase	hsa-miR-181a-5p	ZNF189	7743	ENSG00000136870	validated
tarbase	hsa-miR-106b-5p	ATP6V1G1	9550	ENSG00000136888	validated
tarbase	hsa-miR-15b-5p	ATP6V1G1	9550	ENSG00000136888	validated
tarbase	hsa-miR-21-5p	ATP6V1G1	9550	ENSG00000136888	validated
tarbase	hsa-miR-106b-5p	TEX10	54881	ENSG00000136891	validated
tarbase	hsa-miR-181a-5p	MRPL50	54534	ENSG00000136897	validated
tarbase	hsa-miR-181a-5p	GABBR2	9568	ENSG00000136928	validated
tarbase	hsa-miR-15b-5p	GABBR2	9568	ENSG00000136928	validated
tarbase	hsa-miR-106b-5p	TRMO	51531	ENSG00000136932	validated
tarbase	hsa-miR-222-3p	TRMO	51531	ENSG00000136932	validated
tarbase	hsa-miR-15b-5p	GOLGA1	2800	ENSG00000136935	validated
tarbase	hsa-miR-301a-3p	NCBP1	4686	ENSG00000136937	validated
tarbase	hsa-miR-106b-5p	ANP32B	10541	ENSG00000136938	validated
tarbase	hsa-miR-212-3p	ANP32B	10541	ENSG00000136938	validated
tarbase	hsa-miR-21-5p	ANP32B	10541	ENSG00000136938	validated
tarbase	hsa-miR-222-3p	ANP32B	10541	ENSG00000136938	validated
tarbase	hsa-miR-301a-3p	LMX1B	4010	ENSG00000136944	validated
tarbase	hsa-miR-18a-5p	ENPP2	5168	ENSG00000136960	validated
tarbase	hsa-miR-212-3p	ENPP2	5168	ENSG00000136960	validated
tarbase	hsa-miR-21-5p	ENPP2	5168	ENSG00000136960	validated
tarbase	hsa-miR-192-5p	MYC	4609	ENSG00000136997	validated
tarbase	hsa-miR-301a-3p	MYC	4609	ENSG00000136997	validated
tarbase	hsa-miR-582-3p	MYC	4609	ENSG00000136997	validated
tarbase	hsa-miR-106b-5p	RANBP6	26953	ENSG00000137040	validated
tarbase	hsa-miR-15b-5p	RANBP6	26953	ENSG00000137040	validated
tarbase	hsa-miR-106b-5p	POLR1E	64425	ENSG00000137054	validated
tarbase	hsa-miR-106b-5p	PLAA	9373	ENSG00000137055	validated
tarbase	hsa-miR-15b-5p	PLAA	9373	ENSG00000137055	validated
tarbase	hsa-miR-15b-5p	UBAP2	55833	ENSG00000137073	validated
tarbase	hsa-miR-301a-3p	UBAP2	55833	ENSG00000137073	validated

tarbase	hsa-miR-582-3p	UBAP2	55833	ENSG00000137073	validated
tarbase	hsa-miR-106b-5p	RNF38	152006	ENSG00000137075	validated
tarbase	hsa-miR-18a-5p	RNF38	152006	ENSG00000137075	validated
tarbase	hsa-miR-21-5p	RNF38	152006	ENSG00000137075	validated
tarbase	hsa-miR-301a-3p	RNF38	152006	ENSG00000137075	validated
tarbase	hsa-miR-21-5p	TLN1	7094	ENSG00000137076	validated
tarbase	hsa-miR-296-5p	TLN1	7094	ENSG00000137076	validated
tarbase	hsa-miR-106b-5p	TLN1	7094	ENSG00000137076	validated
tarbase	hsa-miR-505-3p	ALDH1B1	219	ENSG00000137124	validated
tarbase	hsa-miR-212-3p	ARHGEF39	84904	ENSG00000137135	validated
tarbase	hsa-miR-21-5p	DENND4C	55667	ENSG00000137145	validated
tarbase	hsa-miR-301a-3p	RPS6	6194	ENSG00000137154	validated
tarbase	hsa-miR-106b-5p	KIF13A	63971	ENSG00000137177	validated
tarbase	hsa-miR-582-5p	KIF13A	63971	ENSG00000137177	validated
tarbase	hsa-miR-21-5p	ZSCAN9	7746	ENSG00000137185	validated
tarbase	hsa-miR-582-5p	PIM1	5292	ENSG00000137193	validated
tarbase	hsa-miR-18a-5p	TFAP2A	7020	ENSG00000137203	validated
tarbase	hsa-miR-15b-5p	YIPF3	25844	ENSG00000137207	validated
tarbase	hsa-miR-18a-5p	TJAP1	93643	ENSG00000137221	validated
tarbase	hsa-miR-212-3p	IRF4	3662	ENSG00000137265	validated
tarbase	hsa-miR-21-5p	IRF4	3662	ENSG00000137265	validated
tarbase	hsa-miR-296-5p	IRF4	3662	ENSG00000137265	validated
tarbase	hsa-miR-505-3p	IRF4	3662	ENSG00000137265	validated
tarbase	hsa-miR-582-5p	IRF4	3662	ENSG00000137265	validated
tarbase	hsa-miR-144-3p	SLC22A23	63027	ENSG00000137266	validated
tarbase	hsa-miR-181a-5p	SLC22A23	63027	ENSG00000137266	validated
tarbase	hsa-miR-212-3p	SLC22A23	63027	ENSG00000137266	validated
tarbase	hsa-miR-212-3p	TUBB2A	7280	ENSG00000137267	validated
tarbase	hsa-miR-15b-5p	LRRC1	55227	ENSG00000137269	validated
tarbase	hsa-miR-192-5p	LRRC1	55227	ENSG00000137269	validated
tarbase	hsa-miR-106b-5p	FOXF2	2295	ENSG00000137273	validated
tarbase	hsa-miR-301a-3p	FOXF2	2295	ENSG00000137273	validated
tarbase	hsa-miR-326	FOXF2	2295	ENSG00000137273	validated
tarbase	hsa-miR-106b-5p	RIPK1	8737	ENSG00000137275	validated
tarbase	hsa-miR-21-5p	UQCC2	84300	ENSG00000137288	validated
tarbase	hsa-miR-15b-5p	RNF144B	255488	ENSG00000137393	validated
tarbase	hsa-miR-212-3p	RNF144B	255488	ENSG00000137393	validated
tarbase	hsa-miR-582-3p	MTCH1	23787	ENSG00000137409	validated
tarbase	hsa-miR-106b-5p	FAM8A1	51439	ENSG00000137414	validated
tarbase	hsa-miR-212-3p	C6orf52	347744	ENSG00000137434	validated
tarbase	hsa-miR-212-3p	CPEB2	132864	ENSG00000137449	validated
tarbase	hsa-miR-181a-5p	CPEB2	132864	ENSG00000137449	validated
tarbase	hsa-miR-15b-5p	FHDC1	85462	ENSG00000137460	validated
tarbase	hsa-miR-212-3p	TTC29	83894	ENSG00000137473	validated
tarbase	hsa-miR-212-3p	SYTL2	54843	ENSG00000137501	validated
tarbase	hsa-miR-212-3p	RAB30	27314	ENSG00000137502	validated
tarbase	hsa-miR-15b-5p	CREBZF	58487	ENSG00000137504	validated
tarbase	hsa-miR-181a-5p	CREBZF	58487	ENSG00000137504	validated
tarbase	hsa-miR-18a-5p	CREBZF	58487	ENSG00000137504	validated
tarbase	hsa-miR-15b-5p	PRCP	5547	ENSG00000137509	validated
tarbase	hsa-miR-181a-5p	PRCP	5547	ENSG00000137509	validated
tarbase	hsa-miR-301a-3p	MRPL15	29088	ENSG00000137547	validated
tarbase	hsa-miR-18a-5p	GGH	8836	ENSG00000137563	validated
tarbase	hsa-miR-212-3p	SLCO5A1	81796	ENSG00000137571	validated
tarbase	hsa-miR-106b-5p	SDCBP	6386	ENSG00000137575	validated
tarbase	hsa-miR-212-3p	SDCBP	6386	ENSG00000137575	validated
tarbase	hsa-miR-222-3p	SDCBP	6386	ENSG00000137575	validated
tarbase	hsa-miR-15b-5p	SDCBP	6386	ENSG00000137575	validated
tarbase	hsa-miR-212-3p	DDX60	55601	ENSG00000137628	validated
tarbase	hsa-miR-212-3p	TMPRSS4	56649	ENSG00000137648	validated
tarbase	hsa-miR-192-5p	BUD13	84811	ENSG00000137656	validated
tarbase	hsa-miR-212-3p	CFAP300	85016	ENSG00000137691	validated
tarbase	hsa-miR-212-3p	DCUN1D5	84259	ENSG00000137692	validated
tarbase	hsa-miR-15b-5p	YAP1	10413	ENSG00000137693	validated
tarbase	hsa-miR-181a-5p	YAP1	10413	ENSG00000137693	validated
tarbase	hsa-miR-212-3p	YAP1	10413	ENSG00000137693	validated
tarbase	hsa-miR-21-5p	YAP1	10413	ENSG00000137693	validated
tarbase	hsa-miR-301a-3p	RDX	5962	ENSG00000137710	validated
tarbase	hsa-miR-106b-5p	RDX	5962	ENSG00000137710	validated
tarbase	hsa-miR-15b-5p	PPP2R1B	5519	ENSG00000137713	validated
tarbase	hsa-miR-18a-5p	PPP2R1B	5519	ENSG00000137713	validated

tarbase	hsa-miR-212-3p	PPP2R1B	5519	ENSG00000137713	validated
tarbase	hsa-miR-21-5p	PPP2R1B	5519	ENSG00000137713	validated
tarbase	hsa-miR-106b-5p	FDX1	2230	ENSG00000137714	validated
tarbase	hsa-miR-21-5p	C11orf1	64776	ENSG00000137720	validated
tarbase	hsa-miR-326	FXYD2	486	ENSG00000137731	validated
tarbase	hsa-miR-301a-3p	MMP13	4322	ENSG00000137745	validated
tarbase	hsa-miR-15b-5p	UNC13C	440279	ENSG00000137766	validated
tarbase	hsa-miR-212-3p	SQOR	58472	ENSG00000137767	validated
tarbase	hsa-miR-106b-5p	CTDSPL2	51496	ENSG00000137770	validated
tarbase	hsa-miR-181a-5p	CTDSPL2	51496	ENSG00000137770	validated
tarbase	hsa-miR-181a-5p	THBS1	7057	ENSG00000137801	validated
tarbase	hsa-miR-21-5p	THBS1	7057	ENSG00000137801	validated
tarbase	hsa-miR-222-3p	THBS1	7057	ENSG00000137801	validated
tarbase	hsa-miR-505-3p	THBS1	7057	ENSG00000137801	validated
tarbase	hsa-miR-21-5p	NUSAP1	51203	ENSG00000137804	validated
tarbase	hsa-miR-212-3p	RTF1	23168	ENSG00000137815	validated
tarbase	hsa-miR-21-5p	RTF1	23168	ENSG00000137815	validated
tarbase	hsa-miR-582-3p	RTF1	23168	ENSG00000137815	validated
tarbase	hsa-miR-15b-5p	RPLP1	6176	ENSG00000137818	validated
tarbase	hsa-miR-181a-5p	RPLP1	6176	ENSG00000137818	validated
tarbase	hsa-miR-21-5p	RPLP1	6176	ENSG00000137818	validated
tarbase	hsa-miR-106b-5p	RMDN3	55177	ENSG00000137824	validated
tarbase	hsa-miR-15b-5p	ADAM10	102	ENSG00000137845	validated
tarbase	hsa-miR-106b-5p	ZNF280D	54816	ENSG00000137871	validated
tarbase	hsa-miR-21-5p	ZNF280D	54816	ENSG00000137871	validated
tarbase	hsa-miR-106b-5p	SEMA6D	80031	ENSG00000137872	validated
tarbase	hsa-miR-212-3p	SEMA6D	80031	ENSG00000137872	validated
tarbase	hsa-miR-222-3p	SEMA6D	80031	ENSG00000137872	validated
tarbase	hsa-miR-582-5p	SEMA6D	80031	ENSG00000137872	validated
tarbase	hsa-miR-192-5p	RSL24D1	51187	ENSG00000137876	validated
tarbase	hsa-miR-106b-5p	RSL24D1	51187	ENSG00000137876	validated
tarbase	hsa-miR-296-5p	BCAR3	8412	ENSG00000137936	validated
tarbase	hsa-miR-181a-5p	TTLL7	79739	ENSG00000137941	validated
tarbase	hsa-miR-222-3p	GTF2B	2959	ENSG00000137947	validated
tarbase	hsa-miR-212-3p	RABGGTB	5876	ENSG00000137955	validated
tarbase	hsa-miR-106b-5p	IFI44L	10964	ENSG00000137959	validated
tarbase	hsa-miR-212-3p	IFI44L	10964	ENSG00000137959	validated
tarbase	hsa-miR-21-5p	IFI44L	10964	ENSG00000137959	validated
tarbase	hsa-miR-106b-5p	GIPC2	54810	ENSG00000137960	validated
tarbase	hsa-miR-181a-5p	ARHGAP29	9411	ENSG00000137962	validated
tarbase	hsa-miR-212-3p	IFI44	10561	ENSG00000137965	validated
tarbase	hsa-miR-106b-5p	DBT	1629	ENSG00000137992	validated
tarbase	hsa-miR-582-5p	DBT	1629	ENSG00000137992	validated
tarbase	hsa-miR-301a-3p	RTCA	8634	ENSG00000137996	validated
tarbase	hsa-miR-181a-5p	SELENOI	85465	ENSG00000138018	validated
tarbase	hsa-miR-212-3p	SELENOI	85465	ENSG00000138018	validated
tarbase	hsa-miR-582-5p	SELENOI	85465	ENSG00000138018	validated
tarbase	hsa-miR-212-3p	CGREF1	10669	ENSG00000138028	validated
tarbase	hsa-miR-15b-5p	PPM1B	5495	ENSG00000138032	validated
tarbase	hsa-miR-181a-5p	PPM1B	5495	ENSG00000138032	validated
tarbase	hsa-miR-181a-5p	PNPT1	87178	ENSG00000138035	validated
tarbase	hsa-miR-212-3p	THUMPD2	80745	ENSG00000138050	validated
tarbase	hsa-miR-181a-5p	CYP1B1	1545	ENSG00000138061	validated
tarbase	hsa-miR-222-3p	CYP1B1	1545	ENSG00000138061	validated
tarbase	hsa-miR-144-3p	RAB1A	5861	ENSG00000138069	validated
tarbase	hsa-miR-181a-5p	RAB1A	5861	ENSG00000138069	validated
tarbase	hsa-miR-212-3p	RAB1A	5861	ENSG00000138069	validated
tarbase	hsa-miR-21-5p	RAB1A	5861	ENSG00000138069	validated
tarbase	hsa-miR-222-3p	RAB1A	5861	ENSG00000138069	validated
tarbase	hsa-miR-301a-3p	RAB1A	5861	ENSG00000138069	validated
tarbase	hsa-miR-212-3p	ACTR2	10097	ENSG00000138071	validated
tarbase	hsa-miR-181a-5p	ACTR2	10097	ENSG00000138071	validated
tarbase	hsa-miR-582-3p	SLC5A6	8884	ENSG00000138074	validated
tarbase	hsa-miR-106b-5p	PREPL	9581	ENSG00000138078	validated
tarbase	hsa-miR-144-3p	PREPL	9581	ENSG00000138078	validated
tarbase	hsa-miR-582-5p	PREPL	9581	ENSG00000138078	validated
tarbase	hsa-miR-181a-5p	PREPL	9581	ENSG00000138078	validated
tarbase	hsa-miR-106b-5p	FBXO11	80204	ENSG00000138081	validated
tarbase	hsa-miR-582-5p	FBXO11	80204	ENSG00000138081	validated
tarbase	hsa-miR-301a-3p	ATRAID	51374	ENSG00000138085	validated
tarbase	hsa-miR-212-3p	CENPO	79172	ENSG00000138092	validated

tarbase	hsa-miR-106b-5p	ACTR1A	10121	ENSG00000138107	validated
tarbase	hsa-miR-296-5p	ACTR1A	10121	ENSG00000138107	validated
tarbase	hsa-miR-181a-5p	ATAD1	84896	ENSG00000138138	validated
tarbase	hsa-miR-21-5p	DUSP5	1847	ENSG00000138166	validated
tarbase	hsa-miR-144-3p	CEP55	55165	ENSG00000138180	validated
tarbase	hsa-miR-181a-5p	CEP55	55165	ENSG00000138180	validated
tarbase	hsa-miR-222-3p	ENTPD1	953	ENSG00000138185	validated
tarbase	hsa-miR-181a-5p	DNAJC13	23317	ENSG00000138246	validated
tarbase	hsa-miR-106b-5p	ANXA7	310	ENSG00000138279	validated
tarbase	hsa-miR-192-5p	ANXA7	310	ENSG00000138279	validated
tarbase	hsa-miR-106b-5p	TET1	80312	ENSG00000138336	validated
tarbase	hsa-miR-192-5p	MYPN	84665	ENSG00000138347	validated
tarbase	hsa-miR-15b-5p	BARD1	580	ENSG00000138376	validated
tarbase	hsa-miR-181a-5p	BARD1	580	ENSG00000138376	validated
tarbase	hsa-miR-15b-5p	ASNSD1	54529	ENSG00000138381	validated
tarbase	hsa-miR-144-3p	SSB	6741	ENSG00000138385	validated
tarbase	hsa-miR-181a-5p	SSB	6741	ENSG00000138385	validated
tarbase	hsa-miR-181a-5p	NAB1	4664	ENSG00000138386	validated
tarbase	hsa-miR-18a-5p	NAB1	4664	ENSG00000138386	validated
tarbase	hsa-miR-212-3p	MDH1B	130752	ENSG00000138400	validated
tarbase	hsa-miR-21-5p	OLA1	29789	ENSG00000138430	validated
tarbase	hsa-miR-15b-5p	ABI2	10152	ENSG00000138443	validated
tarbase	hsa-miR-21-5p	ABI2	10152	ENSG00000138443	validated
tarbase	hsa-miR-18a-5p	ABI2	10152	ENSG00000138443	validated
tarbase	hsa-miR-582-3p	ITGAV	3685	ENSG00000138448	validated
tarbase	hsa-miR-106b-5p	SLC40A1	30061	ENSG00000138449	validated
tarbase	hsa-miR-222-3p	SLC40A1	30061	ENSG00000138449	validated
tarbase	hsa-miR-181a-5p	SLC35A5	55032	ENSG00000138459	validated
tarbase	hsa-miR-212-3p	SENP7	57337	ENSG00000138468	validated
tarbase	hsa-miR-212-3p	PARP9	83666	ENSG00000138496	validated
tarbase	hsa-miR-222-3p	USP8	9101	ENSG00000138592	validated
tarbase	hsa-miR-15b-5p	SECISBP2L	9728	ENSG00000138593	validated
tarbase	hsa-miR-18a-5p	SECISBP2L	9728	ENSG00000138593	validated
tarbase	hsa-miR-222-3p	SECISBP2L	9728	ENSG00000138593	validated
tarbase	hsa-miR-505-3p	SECISBP2L	9728	ENSG00000138593	validated
tarbase	hsa-miR-144-3p	SPPL2A	84888	ENSG00000138600	validated
tarbase	hsa-miR-21-5p	SPPL2A	84888	ENSG00000138600	validated
tarbase	hsa-miR-301a-3p	SPPL2A	84888	ENSG00000138600	validated
tarbase	hsa-miR-582-5p	SPPL2A	84888	ENSG00000138600	validated
tarbase	hsa-miR-15b-5p	GLCE	26035	ENSG00000138604	validated
tarbase	hsa-miR-301a-3p	GLCE	26035	ENSG00000138604	validated
tarbase	hsa-miR-582-3p	GLCE	26035	ENSG00000138604	validated
tarbase	hsa-miR-18a-5p	APH1B	83464	ENSG00000138613	validated
tarbase	hsa-miR-18a-5p	INTS14	81556	ENSG00000138614	validated
tarbase	hsa-miR-21-5p	INTS14	81556	ENSG00000138614	validated
tarbase	hsa-miR-301a-3p	INTS14	81556	ENSG00000138614	validated
tarbase	hsa-miR-212-3p	INTS14	81556	ENSG00000138614	validated
tarbase	hsa-miR-15b-5p	PARP16	54956	ENSG00000138617	validated
tarbase	hsa-miR-181a-5p	SEMA7A	8482	ENSG00000138623	validated
tarbase	hsa-miR-18a-5p	SEMA7A	8482	ENSG00000138623	validated
tarbase	hsa-miR-301a-3p	UBL7	84993	ENSG00000138629	validated
tarbase	hsa-miR-21-5p	ARHGAP24	83478	ENSG00000138639	validated
tarbase	hsa-miR-212-3p	HERC3	8916	ENSG00000138641	validated
tarbase	hsa-miR-301a-3p	HERC6	55008	ENSG00000138642	validated
tarbase	hsa-miR-212-3p	HERC6	55008	ENSG00000138642	validated
tarbase	hsa-miR-212-3p	HERC5	51191	ENSG00000138646	validated
tarbase	hsa-miR-106b-5p	NDST4	64579	ENSG00000138653	validated
tarbase	hsa-miR-18a-5p	NDST4	64579	ENSG00000138653	validated
tarbase	hsa-miR-212-3p	NDST4	64579	ENSG00000138653	validated
tarbase	hsa-miR-21-5p	ZGRF1	55345	ENSG00000138658	validated
tarbase	hsa-miR-212-3p	AP1AR	55435	ENSG00000138660	validated
tarbase	hsa-miR-181a-5p	AP1AR	55435	ENSG00000138660	validated
tarbase	hsa-miR-21-5p	COPS4	51138	ENSG00000138663	validated
tarbase	hsa-miR-18a-5p	HNRNPD	3184	ENSG00000138668	validated
tarbase	hsa-miR-181a-5p	HNRNPD	3184	ENSG00000138668	validated
tarbase	hsa-miR-15b-5p	RASGEF1B	153020	ENSG00000138670	validated
tarbase	hsa-miR-181a-5p	SEC31A	22872	ENSG00000138674	validated
tarbase	hsa-miR-106b-5p	FGF5	2250	ENSG00000138675	validated
tarbase	hsa-miR-181a-5p	GPAT3	84803	ENSG00000138678	validated
tarbase	hsa-miR-106b-5p	FGF2	2247	ENSG00000138685	validated
tarbase	hsa-miR-181a-5p	BBS7	55212	ENSG00000138686	validated

tarbase	hsa-miR-15b-5p	BBS7	55212	ENSG00000138686	validated
tarbase	hsa-miR-181a-5p	KIAA1109	84162	ENSG00000138688	validated
tarbase	hsa-miR-18a-5p	KIAA1109	84162	ENSG00000138688	validated
tarbase	hsa-miR-301a-3p	KIAA1109	84162	ENSG00000138688	validated
tarbase	hsa-miR-212-3p	LARP1B	55132	ENSG00000138709	validated
tarbase	hsa-miR-181a-5p	MMRN1	22915	ENSG00000138722	validated
tarbase	hsa-miR-212-3p	PRDM5	11107	ENSG00000138738	validated
tarbase	hsa-miR-18a-5p	NAAA	27163	ENSG00000138744	validated
tarbase	hsa-miR-18a-5p	NUP54	53371	ENSG00000138750	validated
tarbase	hsa-miR-106b-5p	BMP2K	55589	ENSG00000138756	validated
tarbase	hsa-miR-212-3p	G3BP2	9908	ENSG00000138757	validated
tarbase	hsa-miR-21-5p	G3BP2	9908	ENSG00000138757	validated
tarbase	hsa-miR-301a-3p	G3BP2	9908	ENSG00000138757	validated
tarbase	hsa-miR-181a-5p	SEPTIN11	55752	ENSG00000138758	validated
tarbase	hsa-miR-18a-5p	SEPTIN11	55752	ENSG00000138758	validated
tarbase	hsa-miR-21-5p	SEPTIN11	55752	ENSG00000138758	validated
tarbase	hsa-miR-15b-5p	SCARB2	950	ENSG00000138760	validated
tarbase	hsa-miR-106b-5p	CCNG2	901	ENSG00000138764	validated
tarbase	hsa-miR-15b-5p	CCNG2	901	ENSG00000138764	validated
tarbase	hsa-miR-181a-5p	CCNG2	901	ENSG00000138764	validated
tarbase	hsa-miR-21-5p	CCNG2	901	ENSG00000138764	validated
tarbase	hsa-miR-301a-3p	CCNG2	901	ENSG00000138764	validated
tarbase	hsa-miR-212-3p	CCNG2	901	ENSG00000138764	validated
tarbase	hsa-miR-15b-5p	CNOT6L	246175	ENSG00000138767	validated
tarbase	hsa-miR-181a-5p	CNOT6L	246175	ENSG00000138767	validated
tarbase	hsa-miR-21-5p	CNOT6L	246175	ENSG00000138767	validated
tarbase	hsa-miR-15b-5p	USO1	8615	ENSG00000138768	validated
tarbase	hsa-miR-18a-5p	USO1	8615	ENSG00000138768	validated
tarbase	hsa-miR-106b-5p	SHROOM3	57619	ENSG00000138771	validated
tarbase	hsa-miR-181a-5p	SHROOM3	57619	ENSG00000138771	validated
tarbase	hsa-miR-212-3p	CENPE	1062	ENSG00000138778	validated
tarbase	hsa-miR-15b-5p	GSTCD	79807	ENSG00000138780	validated
tarbase	hsa-miR-181a-5p	GSTCD	79807	ENSG00000138780	validated
tarbase	hsa-miR-212-3p	ENPEP	2028	ENSG00000138792	validated
tarbase	hsa-miR-21-5p	HADH	3033	ENSG00000138796	validated
tarbase	hsa-miR-222-3p	SEC24B	10427	ENSG00000138802	validated
tarbase	hsa-miR-296-5p	SLC39A8	64116	ENSG00000138821	validated
tarbase	hsa-miR-301a-3p	SLC39A8	64116	ENSG00000138821	validated
tarbase	hsa-miR-212-3p	MTTP	4547	ENSG00000138823	validated
tarbase	hsa-miR-15b-5p	FBN2	2201	ENSG00000138829	validated
tarbase	hsa-miR-18a-5p	FBN2	2201	ENSG00000138829	validated
tarbase	hsa-miR-582-5p	FBN2	2201	ENSG00000138829	validated
tarbase	hsa-miR-15b-5p	GUCD1	83606	ENSG00000138867	validated
tarbase	hsa-miR-301a-3p	GUCD1	83606	ENSG00000138867	validated
tarbase	hsa-miR-15b-5p	B4GALNT3	283358	ENSG00000139044	validated
tarbase	hsa-miR-181a-5p	ETV6	2120	ENSG00000139083	validated
tarbase	hsa-miR-15b-5p	KIF21A	55605	ENSG00000139116	validated
tarbase	hsa-miR-18a-5p	KIF21A	55605	ENSG00000139116	validated
tarbase	hsa-miR-21-5p	KIF21A	55605	ENSG00000139116	validated
tarbase	hsa-miR-296-5p	CPNE8	144402	ENSG00000139117	validated
tarbase	hsa-miR-106b-5p	FGD4	121512	ENSG00000139132	validated
tarbase	hsa-miR-15b-5p	FGD4	121512	ENSG00000139132	validated
tarbase	hsa-miR-181a-5p	FGD4	121512	ENSG00000139132	validated
tarbase	hsa-miR-15b-5p	AEBP2	121536	ENSG00000139154	validated
tarbase	hsa-miR-18a-5p	AEBP2	121536	ENSG00000139154	validated
tarbase	hsa-miR-326	AEBP2	121536	ENSG00000139154	validated
tarbase	hsa-miR-106b-5p	ETNK1	55500	ENSG00000139163	validated
tarbase	hsa-miR-181a-5p	ETNK1	55500	ENSG00000139163	validated
tarbase	hsa-miR-212-3p	ETNK1	55500	ENSG00000139163	validated
tarbase	hsa-miR-222-3p	ETNK1	55500	ENSG00000139163	validated
tarbase	hsa-miR-582-5p	ETNK1	55500	ENSG00000139163	validated
tarbase	hsa-miR-15b-5p	ZCRB1	85437	ENSG00000139168	validated
tarbase	hsa-miR-212-3p	RBP5	83758	ENSG00000139194	validated
tarbase	hsa-miR-301a-3p	AMIGO2	347902	ENSG00000139211	validated
tarbase	hsa-miR-181a-5p	SCAF11	9169	ENSG00000139218	validated
tarbase	hsa-miR-301a-3p	COL2A1	1280	ENSG00000139219	validated
tarbase	hsa-miR-181a-5p	ANP32D	23519	ENSG00000139223	validated
tarbase	hsa-miR-18a-5p	LLPH	84298	ENSG00000139233	validated
tarbase	hsa-miR-212-3p	LRIG3	121227	ENSG00000139263	validated
tarbase	hsa-miR-15b-5p	MARCHF9	92979	ENSG00000139266	validated
tarbase	hsa-miR-15b-5p	TPH2	121278	ENSG00000139287	validated

tarbase	hsa-miR-106b-5p	PHLDA1	22822	ENSG00000139289	validated
tarbase	hsa-miR-106b-5p	TMEM19	55266	ENSG00000139291	validated
tarbase	hsa-miR-192-5p	TMEM19	55266	ENSG00000139291	validated
tarbase	hsa-miR-222-3p	TMEM19	55266	ENSG00000139291	validated
tarbase	hsa-miR-301a-3p	TMEM19	55266	ENSG00000139291	validated
tarbase	hsa-miR-181a-5p	TMEM19	55266	ENSG00000139291	validated
tarbase	hsa-miR-15b-5p	DUSP6	1848	ENSG00000139318	validated
tarbase	hsa-miR-15b-5p	TMTC3	160418	ENSG00000139324	validated
tarbase	hsa-miR-15b-5p	SLC15A4	121260	ENSG00000139370	validated
tarbase	hsa-miR-106b-5p	GIT2	9815	ENSG00000139436	validated
tarbase	hsa-miR-18a-5p	GIT2	9815	ENSG00000139436	validated
tarbase	hsa-miR-582-5p	GIT2	9815	ENSG00000139436	validated
tarbase	hsa-miR-106b-5p	NUP58	9818	ENSG00000139496	validated
tarbase	hsa-miR-15b-5p	NUP58	9818	ENSG00000139496	validated
tarbase	hsa-miR-212-3p	NUP58	9818	ENSG00000139496	validated
tarbase	hsa-miR-21-5p	NUP58	9818	ENSG00000139496	validated
tarbase	hsa-miR-222-3p	NUP58	9818	ENSG00000139496	validated
tarbase	hsa-miR-301a-3p	NUP58	9818	ENSG00000139496	validated
tarbase	hsa-miR-301a-3p	MTMR6	9107	ENSG00000139505	validated
tarbase	hsa-miR-106b-5p	SLC7A1	6541	ENSG00000139514	validated
tarbase	hsa-miR-212-3p	SLC7A1	6541	ENSG00000139514	validated
tarbase	hsa-miR-301a-3p	SLC7A1	6541	ENSG00000139514	validated
tarbase	hsa-miR-212-3p	CCDC65	85478	ENSG00000139537	validated
tarbase	hsa-miR-192-5p	TARBP2	6895	ENSG00000139546	validated
tarbase	hsa-miR-296-5p	TARBP2	6895	ENSG00000139546	validated
tarbase	hsa-miR-15b-5p	KANSL2	54934	ENSG00000139620	validated
tarbase	hsa-miR-18a-5p	C12orf10	60314	ENSG00000139637	validated
tarbase	hsa-miR-301a-3p	C12orf10	60314	ENSG00000139637	validated
tarbase	hsa-miR-15b-5p	TMBIM6	7009	ENSG00000139644	validated
tarbase	hsa-miR-18a-5p	TMBIM6	7009	ENSG00000139644	validated
tarbase	hsa-miR-222-3p	TMBIM6	7009	ENSG00000139644	validated
tarbase	hsa-miR-301a-3p	TMBIM6	7009	ENSG00000139644	validated
tarbase	hsa-miR-15b-5p	ANKRD52	283373	ENSG00000139645	validated
tarbase	hsa-miR-181a-5p	ANKRD52	283373	ENSG00000139645	validated
tarbase	hsa-miR-18a-5p	ANKRD52	283373	ENSG00000139645	validated
tarbase	hsa-miR-296-5p	ANKRD52	283373	ENSG00000139645	validated
tarbase	hsa-miR-301a-3p	ANKRD52	283373	ENSG00000139645	validated
tarbase	hsa-miR-326	ANKRD52	283373	ENSG00000139645	validated
tarbase	hsa-miR-582-5p	ANKRD52	283373	ENSG00000139645	validated
tarbase	hsa-miR-582-5p	ZNF740	283337	ENSG00000139651	validated
tarbase	hsa-miR-18a-5p	ZNF740	283337	ENSG00000139651	validated
tarbase	hsa-miR-18a-5p	ESD	2098	ENSG00000139684	validated
tarbase	hsa-miR-181a-5p	RB1	5925	ENSG00000139687	validated
tarbase	hsa-miR-18a-5p	RB1	5925	ENSG00000139687	validated
tarbase	hsa-miR-301a-3p	RB1	5925	ENSG00000139687	validated
tarbase	hsa-miR-106b-5p	SBNO1	55206	ENSG00000139697	validated
tarbase	hsa-miR-181a-5p	SBNO1	55206	ENSG00000139697	validated
tarbase	hsa-miR-21-5p	SBNO1	55206	ENSG00000139697	validated
tarbase	hsa-miR-18a-5p	SETD1B	23067	ENSG00000139718	validated
tarbase	hsa-miR-212-3p	SETD1B	23067	ENSG00000139718	validated
tarbase	hsa-miR-505-3p	SETD1B	23067	ENSG00000139718	validated
tarbase	hsa-miR-181a-5p	DENR	8562	ENSG00000139726	validated
tarbase	hsa-miR-106b-5p	SLAIN1	122060	ENSG00000139737	validated
tarbase	hsa-miR-301a-3p	SLAIN1	122060	ENSG00000139737	validated
tarbase	hsa-miR-106b-5p	RBM26	64062	ENSG00000139746	validated
tarbase	hsa-miR-15b-5p	RBM26	64062	ENSG00000139746	validated
tarbase	hsa-miR-181a-5p	RBM26	64062	ENSG00000139746	validated
tarbase	hsa-miR-18a-5p	RBM26	64062	ENSG00000139746	validated
tarbase	hsa-miR-326	RBM26	64062	ENSG00000139746	validated
tarbase	hsa-miR-582-3p	RBM26	64062	ENSG00000139746	validated
tarbase	hsa-miR-15b-5p	MBNL2	10150	ENSG00000139793	validated
tarbase	hsa-miR-18a-5p	MBNL2	10150	ENSG00000139793	validated
tarbase	hsa-miR-582-5p	MBNL2	10150	ENSG00000139793	validated
tarbase	hsa-miR-181a-5p	MBNL2	10150	ENSG00000139793	validated
tarbase	hsa-miR-106b-5p	ZIC5	85416	ENSG00000139800	validated
tarbase	hsa-miR-15b-5p	ZIC5	85416	ENSG00000139800	validated
tarbase	hsa-miR-181a-5p	ZIC5	85416	ENSG00000139800	validated
tarbase	hsa-miR-106b-5p	ABHD13	84945	ENSG00000139826	validated
tarbase	hsa-miR-181a-5p	ABHD13	84945	ENSG00000139826	validated
tarbase	hsa-miR-18a-5p	ABHD13	84945	ENSG00000139826	validated
tarbase	hsa-miR-15b-5p	ABHD13	84945	ENSG00000139826	validated

tarbase	hsa-miR-181a-5p	NOVA1	4857	ENSG00000139910	validated
tarbase	hsa-miR-15b-5p	FITM1	161247	ENSG00000139914	validated
tarbase	hsa-miR-181a-5p	TMX1	81542	ENSG00000139921	validated
tarbase	hsa-miR-21-5p	TMX1	81542	ENSG00000139921	validated
tarbase	hsa-miR-505-3p	TMX1	81542	ENSG00000139921	validated
tarbase	hsa-miR-181a-5p	FRMD6	122786	ENSG00000139926	validated
tarbase	hsa-miR-301a-3p	FRMD6	122786	ENSG00000139926	validated
tarbase	hsa-miR-212-3p	FRMD6	122786	ENSG00000139926	validated
tarbase	hsa-miR-15b-5p	PELI2	57161	ENSG00000139946	validated
tarbase	hsa-miR-181a-5p	PELI2	57161	ENSG00000139946	validated
tarbase	hsa-miR-18a-5p	SYT16	83851	ENSG00000139973	validated
tarbase	hsa-miR-181a-5p	SYT16	83851	ENSG00000139973	validated
tarbase	hsa-miR-212-3p	SLC38A6	145389	ENSG00000139974	validated
tarbase	hsa-miR-106b-5p	NAA30	122830	ENSG00000139977	validated
tarbase	hsa-miR-181a-5p	NAA30	122830	ENSG00000139977	validated
tarbase	hsa-miR-301a-3p	NAA30	122830	ENSG00000139977	validated
tarbase	hsa-miR-15b-5p	WDR89	112840	ENSG00000140006	validated
tarbase	hsa-miR-301a-3p	STON2	85439	ENSG00000140022	validated
tarbase	hsa-miR-326	FBLN5	10516	ENSG00000140092	validated
tarbase	hsa-miR-21-5p	WARS1	7453	ENSG00000140105	validated
tarbase	hsa-miR-144-3p	WDR20	91833	ENSG00000140153	validated
tarbase	hsa-miR-181a-5p	WDR20	91833	ENSG00000140153	validated
tarbase	hsa-miR-301a-3p	WDR20	91833	ENSG00000140153	validated
tarbase	hsa-miR-582-5p	WDR20	91833	ENSG00000140153	validated
tarbase	hsa-miR-181a-5p	NIPA2	81614	ENSG00000140157	validated
tarbase	hsa-miR-18a-5p	NIPA2	81614	ENSG00000140157	validated
tarbase	hsa-miR-15b-5p	MFAP1	4236	ENSG00000140259	validated
tarbase	hsa-miR-18a-5p	MFAP1	4236	ENSG00000140259	validated
tarbase	hsa-miR-15b-5p	TCF12	6938	ENSG00000140262	validated
tarbase	hsa-miR-222-3p	TCF12	6938	ENSG00000140262	validated
tarbase	hsa-miR-582-5p	ZSCAN29	146050	ENSG00000140265	validated
tarbase	hsa-miR-181a-5p	ZSCAN29	146050	ENSG00000140265	validated
tarbase	hsa-miR-181a-5p	BNIP2	663	ENSG00000140299	validated
tarbase	hsa-miR-192-5p	BNIP2	663	ENSG00000140299	validated
tarbase	hsa-miR-18a-5p	BNIP2	663	ENSG00000140299	validated
tarbase	hsa-miR-18a-5p	SRP14	6727	ENSG00000140319	validated
tarbase	hsa-miR-296-5p	BAHD1	22893	ENSG00000140320	validated
tarbase	hsa-miR-296-5p	TLE3	7090	ENSG00000140332	validated
tarbase	hsa-miR-181a-5p	ANP32A	8125	ENSG00000140350	validated
tarbase	hsa-miR-582-5p	ANP32A	8125	ENSG00000140350	validated
tarbase	hsa-miR-18a-5p	UBE2Q2	92912	ENSG00000140367	validated
tarbase	hsa-miR-212-3p	PSTPIP1	9051	ENSG00000140368	validated
tarbase	hsa-miR-181a-5p	NCOA2	10499	ENSG00000140396	validated
tarbase	hsa-miR-212-3p	NCOA2	10499	ENSG00000140396	validated
tarbase	hsa-miR-21-5p	NCOA2	10499	ENSG00000140396	validated
tarbase	hsa-miR-582-3p	NCOA2	10499	ENSG00000140396	validated
tarbase	hsa-miR-181a-5p	DNAJA4	55466	ENSG00000140403	validated
tarbase	hsa-miR-296-5p	TLNRD1	59274	ENSG00000140406	validated
tarbase	hsa-miR-106b-5p	IGF1R	3480	ENSG00000140443	validated
tarbase	hsa-miR-15b-5p	IGF1R	3480	ENSG00000140443	validated
tarbase	hsa-miR-181a-5p	IGF1R	3480	ENSG00000140443	validated
tarbase	hsa-miR-505-3p	IGF1R	3480	ENSG00000140443	validated
tarbase	hsa-miR-144-3p	ARRDC4	91947	ENSG00000140450	validated
tarbase	hsa-miR-15b-5p	ARRDC4	91947	ENSG00000140450	validated
tarbase	hsa-miR-15b-5p	PIF1	80119	ENSG00000140451	validated
tarbase	hsa-miR-212-3p	PIF1	80119	ENSG00000140451	validated
tarbase	hsa-miR-582-3p	USP3	9960	ENSG00000140455	validated
tarbase	hsa-miR-582-5p	USP3	9960	ENSG00000140455	validated
tarbase	hsa-miR-212-3p	BBS4	585	ENSG00000140463	validated
tarbase	hsa-miR-15b-5p	PML	5371	ENSG00000140464	validated
tarbase	hsa-miR-212-3p	CYP1A1	1543	ENSG00000140465	validated
tarbase	hsa-miR-106b-5p	LINS1	55180	ENSG00000140471	validated
tarbase	hsa-miR-222-3p	AC091230.1	25989	ENSG00000140474	validated
tarbase	hsa-miR-21-5p	PCSK6	5046	ENSG00000140479	validated
tarbase	hsa-miR-15b-5p	SCAMP2	10066	ENSG00000140497	validated
tarbase	hsa-miR-301a-3p	SCAMP2	10066	ENSG00000140497	validated
tarbase	hsa-miR-106b-5p	FANCI	55215	ENSG00000140525	validated
tarbase	hsa-miR-21-5p	ABHD2	11057	ENSG00000140526	validated
tarbase	hsa-miR-212-3p	WDR93	56964	ENSG00000140527	validated
tarbase	hsa-miR-181a-5p	TICRR	90381	ENSG00000140534	validated
tarbase	hsa-miR-106b-5p	FURIN	5045	ENSG00000140564	validated

tarbase	hsa-miR-181a-5p	FURIN	5045	ENSG00000140564	validated
tarbase	hsa-miR-106b-5p	IQGAP1	8826	ENSG00000140575	validated
tarbase	hsa-miR-21-5p	IQGAP1	8826	ENSG00000140575	validated
tarbase	hsa-miR-181a-5p	GLYR1	84656	ENSG00000140632	validated
tarbase	hsa-miR-18a-5p	GLYR1	84656	ENSG00000140632	validated
tarbase	hsa-miR-296-5p	GLYR1	84656	ENSG00000140632	validated
tarbase	hsa-miR-301a-3p	GLYR1	84656	ENSG00000140632	validated
tarbase	hsa-miR-505-3p	GLYR1	84656	ENSG00000140632	validated
tarbase	hsa-miR-106b-5p	PMM2	5373	ENSG00000140650	validated
tarbase	hsa-miR-18a-5p	TGFB1I1	7041	ENSG00000140682	validated
tarbase	hsa-miR-106b-5p	CDR2	1039	ENSG00000140743	validated
tarbase	hsa-miR-181a-5p	ARHGAP17	55114	ENSG00000140750	validated
tarbase	hsa-miR-21-5p	DHX38	9785	ENSG00000140829	validated
tarbase	hsa-miR-106b-5p	ZFHX3	463	ENSG00000140836	validated
tarbase	hsa-miR-15b-5p	ZFHX3	463	ENSG00000140836	validated
tarbase	hsa-miR-21-5p	ZFHX3	463	ENSG00000140836	validated
tarbase	hsa-miR-222-3p	ZFHX3	463	ENSG00000140836	validated
tarbase	hsa-miR-582-3p	ZFHX3	463	ENSG00000140836	validated
tarbase	hsa-miR-181a-5p	CPNE2	221184	ENSG00000140848	validated
tarbase	hsa-miR-212-3p	NLRC5	84166	ENSG00000140853	validated
tarbase	hsa-miR-15b-5p	CMTM3	123920	ENSG00000140931	validated
tarbase	hsa-miR-106b-5p	MAP1LC3B	81631	ENSG00000140941	validated
tarbase	hsa-miR-106b-5p	MBTPS1	8720	ENSG00000140943	validated
tarbase	hsa-miR-181a-5p	ZCCHC14	23174	ENSG00000140948	validated
tarbase	hsa-miR-18a-5p	ZCCHC14	23174	ENSG00000140948	validated
tarbase	hsa-miR-222-3p	ZCCHC14	23174	ENSG00000140948	validated
tarbase	hsa-miR-301a-3p	ZCCHC14	23174	ENSG00000140948	validated
tarbase	hsa-miR-582-5p	ZCCHC14	23174	ENSG00000140948	validated
tarbase	hsa-miR-21-5p	MEAK7	57707	ENSG00000140950	validated
tarbase	hsa-miR-181a-5p	IRF8	3394	ENSG00000140968	validated
tarbase	hsa-miR-582-3p	RHOT2	89941	ENSG00000140983	validated
tarbase	hsa-miR-212-3p	NDUFB10	4716	ENSG00000140990	validated
tarbase	hsa-miR-181a-5p	PDPK1	5170	ENSG00000140992	validated
tarbase	hsa-miR-106b-5p	DEF8	54849	ENSG00000140995	validated
tarbase	hsa-miR-181a-5p	NCOR1	9611	ENSG00000141027	validated
tarbase	hsa-miR-21-5p	NCOR1	9611	ENSG00000141027	validated
tarbase	hsa-miR-301a-3p	NCOR1	9611	ENSG00000141027	validated
tarbase	hsa-miR-582-3p	NCOR1	9611	ENSG00000141027	validated
tarbase	hsa-miR-106b-5p	COPS3	8533	ENSG00000141030	validated
tarbase	hsa-miR-192-5p	COPS3	8533	ENSG00000141030	validated
tarbase	hsa-miR-18a-5p	ZNF287	57336	ENSG00000141040	validated
tarbase	hsa-miR-296-5p	CTRL	1506	ENSG00000141086	validated
tarbase	hsa-miR-15b-5p	NOB1	28987	ENSG00000141101	validated
tarbase	hsa-miR-15b-5p	UNC45B	146862	ENSG00000141161	validated
tarbase	hsa-miR-106b-5p	PCTP	58488	ENSG00000141179	validated
tarbase	hsa-miR-18a-5p	PCTP	58488	ENSG00000141179	validated
tarbase	hsa-miR-212-3p	PCTP	58488	ENSG00000141179	validated
tarbase	hsa-miR-181a-5p	TOM1L1	10040	ENSG00000141198	validated
tarbase	hsa-miR-106b-5p	TOB1	10140	ENSG00000141232	validated
tarbase	hsa-miR-181a-5p	TOB1	10140	ENSG00000141232	validated
tarbase	hsa-miR-301a-3p	TOB1	10140	ENSG00000141232	validated
tarbase	hsa-miR-106b-5p	NPEPPS	9520	ENSG00000141279	validated
tarbase	hsa-miR-15b-5p	NPEPPS	9520	ENSG00000141279	validated
tarbase	hsa-miR-212-3p	LRRC46	90506	ENSG00000141294	validated
tarbase	hsa-miR-212-3p	SSH2	85464	ENSG00000141298	validated
tarbase	hsa-miR-181a-5p	SSH2	85464	ENSG00000141298	validated
tarbase	hsa-miR-15b-5p	CLTC	1213	ENSG00000141367	validated
tarbase	hsa-miR-181a-5p	CLTC	1213	ENSG00000141367	validated
tarbase	hsa-miR-18a-5p	CLTC	1213	ENSG00000141367	validated
tarbase	hsa-miR-192-5p	CLTC	1213	ENSG00000141367	validated
tarbase	hsa-miR-21-5p	CLTC	1213	ENSG00000141367	validated
tarbase	hsa-miR-301a-3p	CLTC	1213	ENSG00000141367	validated
tarbase	hsa-miR-212-3p	C17orf64	124773	ENSG00000141371	validated
tarbase	hsa-miR-301a-3p	SS18	6760	ENSG00000141380	validated
tarbase	hsa-miR-326	AFG3L2	10939	ENSG00000141385	validated
tarbase	hsa-miR-181a-5p	AFG3L2	10939	ENSG00000141385	validated
tarbase	hsa-miR-106b-5p	SLC39A6	25800	ENSG00000141424	validated
tarbase	hsa-miR-181a-5p	SLC39A6	25800	ENSG00000141424	validated
tarbase	hsa-miR-222-3p	SLC39A6	25800	ENSG00000141424	validated
tarbase	hsa-miR-181a-5p	RPRD1A	55197	ENSG00000141425	validated
tarbase	hsa-miR-181a-5p	C18orf21	83608	ENSG00000141428	validated

tarbase	hsa-miR-18a-5p	GALNT1	2589	ENSG00000141429	validated
tarbase	hsa-miR-582-5p	GALNT1	2589	ENSG00000141429	validated
tarbase	hsa-miR-222-3p	ASXL3	80816	ENSG00000141431	validated
tarbase	hsa-miR-296-5p	ASXL3	80816	ENSG00000141431	validated
tarbase	hsa-miR-212-3p	ASXL3	80816	ENSG00000141431	validated
tarbase	hsa-miR-15b-5p	GAREM1	64762	ENSG00000141441	validated
tarbase	hsa-miR-301a-3p	GAREM1	64762	ENSG00000141441	validated
tarbase	hsa-miR-181a-5p	OSBPL1A	114876	ENSG00000141447	validated
tarbase	hsa-miR-144-3p	GREB1L	80000	ENSG00000141449	validated
tarbase	hsa-miR-181a-5p	GREB1L	80000	ENSG00000141449	validated
tarbase	hsa-miR-212-3p	GREB1L	80000	ENSG00000141449	validated
tarbase	hsa-miR-505-3p	GREB1L	80000	ENSG00000141449	validated
tarbase	hsa-miR-582-5p	GREB1L	80000	ENSG00000141449	validated
tarbase	hsa-miR-181a-5p	PELP1	27043	ENSG00000141456	validated
tarbase	hsa-miR-296-5p	SLC13A5	284111	ENSG00000141485	validated
tarbase	hsa-miR-212-3p	SLC13A5	284111	ENSG00000141485	validated
tarbase	hsa-miR-181a-5p	MINK1	50488	ENSG00000141503	validated
tarbase	hsa-miR-296-5p	MINK1	50488	ENSG00000141503	validated
tarbase	hsa-miR-301a-3p	MINK1	50488	ENSG00000141503	validated
tarbase	hsa-miR-15b-5p	ASGR1	432	ENSG00000141505	validated
tarbase	hsa-miR-181a-5p	TP53	7157	ENSG00000141510	validated
tarbase	hsa-miR-212-3p	TP53	7157	ENSG00000141510	validated
tarbase	hsa-miR-21-5p	ARHGDIA	396	ENSG00000141522	validated
tarbase	hsa-miR-296-5p	ARHGDIA	396	ENSG00000141522	validated
tarbase	hsa-miR-326	ARHGDIA	396	ENSG00000141522	validated
tarbase	hsa-miR-181a-5p	SLC16A3	9123	ENSG00000141526	validated
tarbase	hsa-miR-296-5p	CARD14	79092	ENSG00000141527	validated
tarbase	hsa-miR-181a-5p	RAB40B	10966	ENSG00000141542	validated
tarbase	hsa-miR-106b-5p	CSNK1D	1453	ENSG00000141551	validated
tarbase	hsa-miR-301a-3p	CSNK1D	1453	ENSG00000141551	validated
tarbase	hsa-miR-326	CSNK1D	1453	ENSG00000141551	validated
tarbase	hsa-miR-106b-5p	ANAPC11	51529	ENSG00000141552	validated
tarbase	hsa-miR-15b-5p	NARF	26502	ENSG00000141562	validated
tarbase	hsa-miR-106b-5p	NARF	26502	ENSG00000141562	validated
tarbase	hsa-miR-181a-5p	FOXK2	3607	ENSG00000141568	validated
tarbase	hsa-miR-18a-5p	FOXK2	3607	ENSG00000141568	validated
tarbase	hsa-miR-212-3p	TRIM65	201292	ENSG00000141569	validated
tarbase	hsa-miR-296-5p	TRIM65	201292	ENSG00000141569	validated
tarbase	hsa-miR-106b-5p	ZCCHC2	54877	ENSG00000141664	validated
tarbase	hsa-miR-15b-5p	ZCCHC2	54877	ENSG00000141664	validated
tarbase	hsa-miR-181a-5p	ZCCHC2	54877	ENSG00000141664	validated
tarbase	hsa-miR-15b-5p	CBLN2	147381	ENSG00000141668	validated
tarbase	hsa-miR-212-3p	PMAIP1	5366	ENSG00000141682	validated
tarbase	hsa-miR-222-3p	PMAIP1	5366	ENSG00000141682	validated
tarbase	hsa-miR-18a-5p	PMAIP1	5366	ENSG00000141682	validated
tarbase	hsa-miR-106b-5p	RETREG3	162427	ENSG00000141699	validated
tarbase	hsa-miR-222-3p	RETREG3	162427	ENSG00000141699	validated
tarbase	hsa-miR-181a-5p	IGFBP4	3487	ENSG00000141753	validated
tarbase	hsa-miR-18a-5p	IGFBP4	3487	ENSG00000141753	validated
tarbase	hsa-miR-222-3p	IGFBP4	3487	ENSG00000141753	validated
tarbase	hsa-miR-21-5p	TXNL4A	10907	ENSG00000141759	validated
tarbase	hsa-miR-181a-5p	MISP3	113230	ENSG00000141854	validated
tarbase	hsa-miR-301a-3p	BRD4	23476	ENSG00000141867	validated
tarbase	hsa-miR-505-3p	BRD4	23476	ENSG00000141867	validated
tarbase	hsa-miR-15b-5p	SLC39A3	29985	ENSG00000141873	validated
tarbase	hsa-miR-106b-5p	NFIC	4782	ENSG00000141905	validated
tarbase	hsa-miR-18a-5p	NFIC	4782	ENSG00000141905	validated
tarbase	hsa-miR-222-3p	NFIC	4782	ENSG00000141905	validated
tarbase	hsa-miR-296-5p	PFKL	5211	ENSG00000141959	validated
tarbase	hsa-miR-301a-3p	PFKL	5211	ENSG00000141959	validated
tarbase	hsa-miR-296-5p	FEM1A	55527	ENSG00000141965	validated
tarbase	hsa-miR-192-5p	DPP9	91039	ENSG00000142002	validated
tarbase	hsa-miR-181a-5p	ZFP14	57677	ENSG00000142065	validated
tarbase	hsa-miR-106b-5p	IFITM3	10410	ENSG00000142089	validated
tarbase	hsa-miR-212-3p	IFITM3	10410	ENSG00000142089	validated
tarbase	hsa-miR-296-5p	PGGHG	80162	ENSG00000142102	validated
tarbase	hsa-miR-21-5p	SOD1	6647	ENSG00000142168	validated
tarbase	hsa-miR-582-5p	SOD1	6647	ENSG00000142168	validated
tarbase	hsa-miR-296-5p	SIK1	150094	ENSG00000142178	validated
tarbase	hsa-miR-582-3p	SIK1	150094	ENSG00000142178	validated
tarbase	hsa-miR-18a-5p	SIK1	150094	ENSG00000142178	validated

tarbase	hsa-miR-212-3p	DNMT3L	29947	ENSG00000142182	validated
tarbase	hsa-miR-15b-5p	SCYL1	57410	ENSG00000142186	validated
tarbase	hsa-miR-301a-3p	TMEM50B	757	ENSG00000142188	validated
tarbase	hsa-miR-181a-5p	APP	351	ENSG00000142192	validated
tarbase	hsa-miR-15b-5p	DOP1B	9980	ENSG00000142197	validated
tarbase	hsa-miR-181a-5p	URB1	9875	ENSG00000142207	validated
tarbase	hsa-miR-106b-5p	AKT1	207	ENSG00000142208	validated
tarbase	hsa-miR-18a-5p	SAE1	10055	ENSG00000142230	validated
tarbase	hsa-miR-582-3p	GEMIN7	79760	ENSG00000142252	validated
tarbase	hsa-miR-18a-5p	CARM1	10498	ENSG00000142453	validated
tarbase	hsa-miR-222-3p	CARM1	10498	ENSG00000142453	validated
tarbase	hsa-miR-106b-5p	PSMB6	5694	ENSG00000142507	validated
tarbase	hsa-miR-106b-5p	ZNF473	25888	ENSG00000142528	validated
tarbase	hsa-miR-582-5p	ZNF473	25888	ENSG00000142528	validated
tarbase	hsa-miR-106b-5p	RPS11	6205	ENSG00000142534	validated
tarbase	hsa-miR-301a-3p	RPS11	6205	ENSG00000142534	validated
tarbase	hsa-miR-301a-3p	RPL13A	23521	ENSG00000142541	validated
tarbase	hsa-miR-582-5p	CTU1	90353	ENSG00000142544	validated
tarbase	hsa-miR-106b-5p	ZNF614	80110	ENSG00000142556	validated
tarbase	hsa-miR-301a-3p	ZNF614	80110	ENSG00000142556	validated
tarbase	hsa-miR-505-3p	ZNF614	80110	ENSG00000142556	validated
tarbase	hsa-miR-21-5p	RERE	473	ENSG00000142599	validated
tarbase	hsa-miR-21-5p	EPHA2	1969	ENSG00000142627	validated
tarbase	hsa-miR-21-5p	EFHD2	79180	ENSG00000142634	validated
tarbase	hsa-miR-212-3p	RPL11	6135	ENSG00000142676	validated
tarbase	hsa-miR-212-3p	C1orf216	127703	ENSG00000142686	validated
tarbase	hsa-miR-15b-5p	HSPG2	3339	ENSG00000142798	validated
tarbase	hsa-miR-106b-5p	SERBP1	26135	ENSG00000142864	validated
tarbase	hsa-miR-222-3p	SERBP1	26135	ENSG00000142864	validated
tarbase	hsa-miR-301a-3p	SERBP1	26135	ENSG00000142864	validated
tarbase	hsa-miR-106b-5p	BCL10	8915	ENSG00000142867	validated
tarbase	hsa-miR-212-3p	BCL10	8915	ENSG00000142867	validated
tarbase	hsa-miR-181a-5p	CCN1	3491	ENSG00000142871	validated
tarbase	hsa-miR-222-3p	CCN1	3491	ENSG00000142871	validated
tarbase	hsa-miR-106b-5p	KIF2C	11004	ENSG00000142945	validated
tarbase	hsa-miR-15b-5p	KIF2C	11004	ENSG00000142945	validated
tarbase	hsa-miR-301a-3p	KIF2C	11004	ENSG00000142945	validated
tarbase	hsa-miR-15b-5p	PTPRF	5792	ENSG00000142949	validated
tarbase	hsa-miR-212-3p	PTPRF	5792	ENSG00000142949	validated
tarbase	hsa-miR-326	PTPRF	5792	ENSG00000142949	validated
tarbase	hsa-miR-326	MOB3C	148932	ENSG00000142961	validated
tarbase	hsa-miR-15b-5p	LMO4	8543	ENSG00000143013	validated
tarbase	hsa-miR-18a-5p	LMO4	8543	ENSG00000143013	validated
tarbase	hsa-miR-21-5p	LMO4	8543	ENSG00000143013	validated
tarbase	hsa-miR-326	LMO4	8543	ENSG00000143013	validated
tarbase	hsa-miR-144-3p	MTF2	22823	ENSG00000143033	validated
tarbase	hsa-miR-181a-5p	MTF2	22823	ENSG00000143033	validated
tarbase	hsa-miR-212-3p	MTF2	22823	ENSG00000143033	validated
tarbase	hsa-miR-582-5p	MTF2	22823	ENSG00000143033	validated
tarbase	hsa-miR-181a-5p	IGSF3	3321	ENSG00000143061	validated
tarbase	hsa-miR-15b-5p	ZNF697	90874	ENSG00000143067	validated
tarbase	hsa-miR-181a-5p	ZNF697	90874	ENSG00000143067	validated
tarbase	hsa-miR-106b-5p	STRIP1	85369	ENSG00000143093	validated
tarbase	hsa-miR-15b-5p	STRIP1	85369	ENSG00000143093	validated
tarbase	hsa-miR-212-3p	C1orf162	128346	ENSG00000143110	validated
tarbase	hsa-miR-181a-5p	CELSR2	1952	ENSG00000143126	validated
tarbase	hsa-miR-18a-5p	CELSR2	1952	ENSG00000143126	validated
tarbase	hsa-miR-222-3p	CELSR2	1952	ENSG00000143126	validated
tarbase	hsa-miR-326	CELSR2	1952	ENSG00000143126	validated
tarbase	hsa-miR-15b-5p	CELSR2	1952	ENSG00000143126	validated
tarbase	hsa-miR-144-3p	ATP1B1	481	ENSG00000143153	validated
tarbase	hsa-miR-15b-5p	ATP1B1	481	ENSG00000143153	validated
tarbase	hsa-miR-181a-5p	ATP1B1	481	ENSG00000143153	validated
tarbase	hsa-miR-21-5p	ATP1B1	481	ENSG00000143153	validated
tarbase	hsa-miR-582-5p	ATP1B1	481	ENSG00000143153	validated
tarbase	hsa-miR-106b-5p	POGK	57645	ENSG00000143157	validated
tarbase	hsa-miR-21-5p	POGK	57645	ENSG00000143157	validated
tarbase	hsa-miR-212-3p	DCAF6	55827	ENSG00000143164	validated
tarbase	hsa-miR-212-3p	RXRG	6258	ENSG00000143171	validated
tarbase	hsa-miR-192-5p	UCK2	7371	ENSG00000143179	validated
tarbase	hsa-miR-106b-5p	POU2F1	5451	ENSG00000143190	validated

tarbase	hsa-miR-18a-5p	POU2F1	5451	ENSG00000143190	validated
tarbase	hsa-miR-582-5p	POU2F1	5451	ENSG00000143190	validated
tarbase	hsa-miR-181a-5p	POU2F1	5451	ENSG00000143190	validated
tarbase	hsa-miR-15b-5p	UFC1	51506	ENSG00000143222	validated
tarbase	hsa-miR-144-3p	NUF2	83540	ENSG00000143228	validated
tarbase	hsa-miR-222-3p	NUF2	83540	ENSG00000143228	validated
tarbase	hsa-miR-15b-5p	RGS5	8490	ENSG00000143248	validated
tarbase	hsa-miR-582-3p	SDHC	6391	ENSG00000143252	validated
tarbase	hsa-miR-15b-5p	PFDN2	5202	ENSG00000143256	validated
tarbase	hsa-miR-181a-5p	PRCC	5546	ENSG00000143294	validated
tarbase	hsa-miR-301a-3p	RRNAD1	51093	ENSG00000143303	validated
tarbase	hsa-miR-106b-5p	MRPL24	79590	ENSG00000143314	validated
tarbase	hsa-miR-15b-5p	ISG20L2	81875	ENSG00000143319	validated
tarbase	hsa-miR-301a-3p	ISG20L2	81875	ENSG00000143319	validated
tarbase	hsa-miR-326	ISG20L2	81875	ENSG00000143319	validated
tarbase	hsa-miR-301a-3p	HDGF	3068	ENSG00000143321	validated
tarbase	hsa-miR-106b-5p	ABL2	27	ENSG00000143322	validated
tarbase	hsa-miR-181a-5p	ABL2	27	ENSG00000143322	validated
tarbase	hsa-miR-582-3p	ABL2	27	ENSG00000143322	validated
tarbase	hsa-miR-582-3p	XPR1	9213	ENSG00000143324	validated
tarbase	hsa-miR-181a-5p	TOR1AIP1	26092	ENSG00000143337	validated
tarbase	hsa-miR-212-3p	HMCN1	83872	ENSG00000143341	validated
tarbase	hsa-miR-18a-5p	LYPLAL1	127018	ENSG00000143353	validated
tarbase	hsa-miR-106b-5p	RORC	6097	ENSG00000143365	validated
tarbase	hsa-miR-212-3p	TUFT1	7286	ENSG00000143367	validated
tarbase	hsa-miR-192-5p	SNX27	81609	ENSG00000143376	validated
tarbase	hsa-miR-21-5p	SNX27	81609	ENSG00000143376	validated
tarbase	hsa-miR-301a-3p	SNX27	81609	ENSG00000143376	validated
tarbase	hsa-miR-301a-3p	SETDB1	9869	ENSG00000143379	validated
tarbase	hsa-miR-15b-5p	MCL1	4170	ENSG00000143384	validated
tarbase	hsa-miR-18a-5p	MCL1	4170	ENSG00000143384	validated
tarbase	hsa-miR-505-3p	MCL1	4170	ENSG00000143384	validated
tarbase	hsa-miR-582-3p	MCL1	4170	ENSG00000143384	validated
tarbase	hsa-miR-212-3p	RFX5	5993	ENSG00000143390	validated
tarbase	hsa-miR-582-3p	RFX5	5993	ENSG00000143390	validated
tarbase	hsa-miR-296-5p	PI4KB	5298	ENSG00000143393	validated
tarbase	hsa-miR-181a-5p	ANP32E	81611	ENSG00000143401	validated
tarbase	hsa-miR-21-5p	CERS2	29956	ENSG00000143418	validated
tarbase	hsa-miR-106b-5p	CERS2	29956	ENSG00000143418	validated
tarbase	hsa-miR-15b-5p	ENSA	2029	ENSG00000143420	validated
tarbase	hsa-miR-15b-5p	SEMA6C	10500	ENSG00000143434	validated
tarbase	hsa-miR-181a-5p	ARNT	405	ENSG00000143437	validated
tarbase	hsa-miR-222-3p	ARNT	405	ENSG00000143437	validated
tarbase	hsa-miR-181a-5p	POGZ	23126	ENSG00000143442	validated
tarbase	hsa-miR-18a-5p	POGZ	23126	ENSG00000143442	validated
tarbase	hsa-miR-582-5p	POGZ	23126	ENSG00000143442	validated
tarbase	hsa-miR-21-5p	SYT14	255928	ENSG00000143469	validated
tarbase	hsa-miR-192-5p	KCNH1	3756	ENSG00000143473	validated
tarbase	hsa-miR-106b-5p	DYRK3	8444	ENSG00000143479	validated
tarbase	hsa-miR-21-5p	DYRK3	8444	ENSG00000143479	validated
tarbase	hsa-miR-106b-5p	VASH2	79805	ENSG00000143494	validated
tarbase	hsa-miR-181a-5p	TAF1A	9015	ENSG00000143498	validated
tarbase	hsa-miR-212-3p	TAF1A	9015	ENSG00000143498	validated
tarbase	hsa-miR-301a-3p	SMYD2	56950	ENSG00000143499	validated
tarbase	hsa-miR-15b-5p	SUSD4	55061	ENSG00000143502	validated
tarbase	hsa-miR-15b-5p	ATP8B2	57198	ENSG00000143515	validated
tarbase	hsa-miR-181a-5p	ATP8B2	57198	ENSG00000143515	validated
tarbase	hsa-miR-212-3p	RAB13	5872	ENSG00000143545	validated
tarbase	hsa-miR-21-5p	SNAPIN	23557	ENSG00000143553	validated
tarbase	hsa-miR-15b-5p	SLC27A3	11000	ENSG00000143554	validated
tarbase	hsa-miR-222-3p	S100A7	6278	ENSG00000143556	validated
tarbase	hsa-miR-106b-5p	UBAP2L	9898	ENSG00000143569	validated
tarbase	hsa-miR-181a-5p	UBAP2L	9898	ENSG00000143569	validated
tarbase	hsa-miR-192-5p	UBAP2L	9898	ENSG00000143569	validated
tarbase	hsa-miR-21-5p	UBAP2L	9898	ENSG00000143569	validated
tarbase	hsa-miR-301a-3p	UBAP2L	9898	ENSG00000143569	validated
tarbase	hsa-miR-326	SLC39A1	27173	ENSG00000143570	validated
tarbase	hsa-miR-582-3p	SLC39A1	27173	ENSG00000143570	validated
tarbase	hsa-miR-21-5p	HAX1	10456	ENSG00000143575	validated
tarbase	hsa-miR-106b-5p	EFNA3	1944	ENSG00000143590	validated
tarbase	hsa-miR-301a-3p	EFNA3	1944	ENSG00000143590	validated

tarbase	hsa-miR-582-3p	EFNA3	1944	ENSG00000143590	validated
tarbase	hsa-miR-181a-5p	C1orf43	25912	ENSG00000143612	validated
tarbase	hsa-miR-222-3p	C1orf43	25912	ENSG00000143612	validated
tarbase	hsa-miR-15b-5p	GATAD2B	57459	ENSG00000143614	validated
tarbase	hsa-miR-212-3p	GATAD2B	57459	ENSG00000143614	validated
tarbase	hsa-miR-21-5p	GATAD2B	57459	ENSG00000143614	validated
tarbase	hsa-miR-301a-3p	GATAD2B	57459	ENSG00000143614	validated
tarbase	hsa-miR-106b-5p	RIT1	6016	ENSG00000143622	validated
tarbase	hsa-miR-181a-5p	RIT1	6016	ENSG00000143622	validated
tarbase	hsa-miR-18a-5p	RIT1	6016	ENSG00000143622	validated
tarbase	hsa-miR-181a-5p	INTS3	65123	ENSG00000143624	validated
tarbase	hsa-miR-181a-5p	C1orf131	128061	ENSG00000143633	validated
tarbase	hsa-miR-15b-5p	GALNT2	2590	ENSG00000143641	validated
tarbase	hsa-miR-21-5p	GALNT2	2590	ENSG00000143641	validated
tarbase	hsa-miR-106b-5p	LYST	1130	ENSG00000143669	validated
tarbase	hsa-miR-15b-5p	MAP3K21	84451	ENSG00000143674	validated
tarbase	hsa-miR-181a-5p	MAP3K21	84451	ENSG00000143674	validated
tarbase	hsa-miR-18a-5p	MAP3K21	84451	ENSG00000143674	validated
tarbase	hsa-miR-192-5p	MAP3K21	84451	ENSG00000143674	validated
tarbase	hsa-miR-296-5p	MAP3K21	84451	ENSG00000143674	validated
tarbase	hsa-miR-301a-3p	MAP3K21	84451	ENSG00000143674	validated
tarbase	hsa-miR-15b-5p	CEP170	9859	ENSG00000143702	validated
tarbase	hsa-miR-21-5p	CEP170	9859	ENSG00000143702	validated
tarbase	hsa-miR-222-3p	CEP170	9859	ENSG00000143702	validated
tarbase	hsa-miR-15b-5p	ACP1	52	ENSG00000143727	validated
tarbase	hsa-miR-212-3p	SRP9	6726	ENSG00000143742	validated
tarbase	hsa-miR-106b-5p	SDE2	163859	ENSG00000143751	validated
tarbase	hsa-miR-582-3p	SDE2	163859	ENSG00000143751	validated
tarbase	hsa-miR-181a-5p	SDE2	163859	ENSG00000143751	validated
tarbase	hsa-miR-181a-5p	DEGS1	8560	ENSG00000143753	validated
tarbase	hsa-miR-21-5p	DEGS1	8560	ENSG00000143753	validated
tarbase	hsa-miR-222-3p	DEGS1	8560	ENSG00000143753	validated
tarbase	hsa-miR-582-3p	DEGS1	8560	ENSG00000143753	validated
tarbase	hsa-miR-106b-5p	FBXO28	23219	ENSG00000143756	validated
tarbase	hsa-miR-18a-5p	FBXO28	23219	ENSG00000143756	validated
tarbase	hsa-miR-212-3p	FBXO28	23219	ENSG00000143756	validated
tarbase	hsa-miR-15b-5p	ARF1	375	ENSG00000143761	validated
tarbase	hsa-miR-212-3p	ARF1	375	ENSG00000143761	validated
tarbase	hsa-miR-222-3p	ARF1	375	ENSG00000143761	validated
tarbase	hsa-miR-301a-3p	ARF1	375	ENSG00000143761	validated
tarbase	hsa-miR-582-5p	ARF1	375	ENSG00000143761	validated
tarbase	hsa-miR-106b-5p	CNIH4	29097	ENSG00000143771	validated
tarbase	hsa-miR-181a-5p	CDC42BPA	8476	ENSG00000143776	validated
tarbase	hsa-miR-222-3p	C1orf35	79169	ENSG00000143793	validated
tarbase	hsa-miR-106b-5p	MBOAT2	129642	ENSG00000143797	validated
tarbase	hsa-miR-181a-5p	MBOAT2	129642	ENSG00000143797	validated
tarbase	hsa-miR-301a-3p	PYCR2	29920	ENSG00000143811	validated
tarbase	hsa-miR-15b-5p	LBR	3930	ENSG00000143815	validated
tarbase	hsa-miR-212-3p	LBR	3930	ENSG00000143815	validated
tarbase	hsa-miR-582-5p	LBR	3930	ENSG00000143815	validated
tarbase	hsa-miR-15b-5p	WNT9A	7483	ENSG00000143816	validated
tarbase	hsa-miR-301a-3p	SOX13	9580	ENSG00000143842	validated
tarbase	hsa-miR-18a-5p	PLEKHA6	22874	ENSG00000143850	validated
tarbase	hsa-miR-15b-5p	ARL8A	127829	ENSG00000143862	validated
tarbase	hsa-miR-181a-5p	RHOB	388	ENSG00000143878	validated
tarbase	hsa-miR-582-5p	RHOB	388	ENSG00000143878	validated
tarbase	hsa-miR-15b-5p	HNRNPLL	92906	ENSG00000143889	validated
tarbase	hsa-miR-15b-5p	EML4	27436	ENSG00000143924	validated
tarbase	hsa-miR-181a-5p	EML4	27436	ENSG00000143924	validated
tarbase	hsa-miR-181a-5p	CALM2	805	ENSG00000143933	validated
tarbase	hsa-miR-18a-5p	CALM2	805	ENSG00000143933	validated
tarbase	hsa-miR-18a-5p	CHAC2	494143	ENSG00000143942	validated
tarbase	hsa-miR-21-5p	RPS27A	6233	ENSG00000143947	validated
tarbase	hsa-miR-181a-5p	RPS27A	6233	ENSG00000143947	validated
tarbase	hsa-miR-212-3p	WDPCP	51057	ENSG00000143951	validated
tarbase	hsa-miR-212-3p	REG3G	130120	ENSG00000143954	validated
tarbase	hsa-miR-106b-5p	ASXL2	55252	ENSG00000143970	validated
tarbase	hsa-miR-15b-5p	ASXL2	55252	ENSG00000143970	validated
tarbase	hsa-miR-181a-5p	ASXL2	55252	ENSG00000143970	validated
tarbase	hsa-miR-212-3p	ETAA1	54465	ENSG00000143971	validated
tarbase	hsa-miR-15b-5p	MEIS1	4211	ENSG00000143995	validated

tarbase	hsa-miR-212-3p	MEIS1	4211	ENSG00000143995	validated
tarbase	hsa-miR-296-5p	ZNF514	84874	ENSG00000144026	validated
tarbase	hsa-miR-15b-5p	SNRNP200	23020	ENSG00000144028	validated
tarbase	hsa-miR-181a-5p	SNRNP200	23020	ENSG00000144028	validated
tarbase	hsa-miR-181a-5p	MRPS5	64969	ENSG00000144029	validated
tarbase	hsa-miR-15b-5p	TEX261	113419	ENSG00000144043	validated
tarbase	hsa-miR-21-5p	TEX261	113419	ENSG00000144043	validated
tarbase	hsa-miR-181a-5p	DUSP11	8446	ENSG00000144048	validated
tarbase	hsa-miR-582-5p	TMEM177	80775	ENSG00000144120	validated
tarbase	hsa-miR-15b-5p	SLC20A1	6574	ENSG00000144136	validated
tarbase	hsa-miR-181a-5p	SLC20A1	6574	ENSG00000144136	validated
tarbase	hsa-miR-212-3p	SLC20A1	6574	ENSG00000144136	validated
tarbase	hsa-miR-222-3p	SLC20A1	6574	ENSG00000144136	validated
tarbase	hsa-miR-301a-3p	SLC20A1	6574	ENSG00000144136	validated
tarbase	hsa-miR-106b-5p	LIPT1	51601	ENSG00000144182	validated
tarbase	hsa-miR-15b-5p	LIPT1	51601	ENSG00000144182	validated
tarbase	hsa-miR-18a-5p	LIPT1	51601	ENSG00000144182	validated
tarbase	hsa-miR-21-5p	UBXN4	23190	ENSG00000144224	validated
tarbase	hsa-miR-326	UBXN4	23190	ENSG00000144224	validated
tarbase	hsa-miR-582-5p	UBXN4	23190	ENSG00000144224	validated
tarbase	hsa-miR-301a-3p	SPOPL	339745	ENSG00000144228	validated
tarbase	hsa-miR-15b-5p	POLR2D	5433	ENSG00000144231	validated
tarbase	hsa-miR-106b-5p	AMMECR1L	83607	ENSG00000144233	validated
tarbase	hsa-miR-106b-5p	PKP4	8502	ENSG00000144283	validated
tarbase	hsa-miR-106b-5p	SCN1A	6323	ENSG00000144285	validated
tarbase	hsa-miR-181a-5p	SLC4A10	57282	ENSG00000144290	validated
tarbase	hsa-miR-18a-5p	SCRN3	79634	ENSG00000144306	validated
tarbase	hsa-miR-212-3p	TMEFF2	23671	ENSG00000144339	validated
tarbase	hsa-miR-106b-5p	CDCA7	83879	ENSG00000144354	validated
tarbase	hsa-miR-106b-5p	UBR3	130507	ENSG00000144357	validated
tarbase	hsa-miR-181a-5p	UBR3	130507	ENSG00000144357	validated
tarbase	hsa-miR-21-5p	PHOSPHO2	493911	ENSG00000144362	validated
tarbase	hsa-miR-15b-5p	GULP1	51454	ENSG00000144366	validated
tarbase	hsa-miR-15b-5p	FAM171B	165215	ENSG00000144369	validated
tarbase	hsa-miR-181a-5p	FAM171B	165215	ENSG00000144369	validated
tarbase	hsa-miR-144-3p	HSPD1	3329	ENSG00000144381	validated
tarbase	hsa-miR-181a-5p	METTL21A	151194	ENSG00000144401	validated
tarbase	hsa-miR-15b-5p	UNC80	285175	ENSG00000144406	validated
tarbase	hsa-miR-181a-5p	UNC80	285175	ENSG00000144406	validated
tarbase	hsa-miR-212-3p	CPO	130749	ENSG00000144410	validated
tarbase	hsa-miR-106b-5p	NBEAL1	65065	ENSG00000144426	validated
tarbase	hsa-miR-106b-5p	KANSL1L	151050	ENSG00000144445	validated
tarbase	hsa-miR-21-5p	KANSL1L	151050	ENSG00000144445	validated
tarbase	hsa-miR-181a-5p	SUMF1	285362	ENSG00000144455	validated
tarbase	hsa-miR-21-5p	SUMF1	285362	ENSG00000144455	validated
tarbase	hsa-miR-326	NYAP2	57624	ENSG00000144460	validated
tarbase	hsa-miR-15b-5p	RHBDD1	84236	ENSG00000144468	validated
tarbase	hsa-miR-181a-5p	RHBDD1	84236	ENSG00000144468	validated
tarbase	hsa-miR-15b-5p	COPS7B	64708	ENSG00000144524	validated
tarbase	hsa-miR-181a-5p	COPS7B	64708	ENSG00000144524	validated
tarbase	hsa-miR-301a-3p	COPS7B	64708	ENSG00000144524	validated
tarbase	hsa-miR-326	COPS7B	64708	ENSG00000144524	validated
tarbase	hsa-miR-15b-5p	VGLL4	9686	ENSG00000144560	validated
tarbase	hsa-miR-18a-5p	VGLL4	9686	ENSG00000144560	validated
tarbase	hsa-miR-301a-3p	VGLL4	9686	ENSG00000144560	validated
tarbase	hsa-miR-181a-5p	RAB5A	5868	ENSG00000144566	validated
tarbase	hsa-miR-192-5p	RAB5A	5868	ENSG00000144566	validated
tarbase	hsa-miR-15b-5p	RETREG2	79137	ENSG00000144567	validated
tarbase	hsa-miR-505-3p	RETREG2	79137	ENSG00000144567	validated
tarbase	hsa-miR-106b-5p	RETREG2	79137	ENSG00000144567	validated
tarbase	hsa-miR-15b-5p	CTDSP1	58190	ENSG00000144579	validated
tarbase	hsa-miR-18a-5p	CTDSP1	58190	ENSG00000144579	validated
tarbase	hsa-miR-18a-5p	CNOT9	9125	ENSG00000144580	validated
tarbase	hsa-miR-222-3p	CNOT9	9125	ENSG00000144580	validated
tarbase	hsa-miR-582-5p	CNOT9	9125	ENSG00000144580	validated
tarbase	hsa-miR-212-3p	STK11IP	114790	ENSG00000144589	validated
tarbase	hsa-miR-181a-5p	EAF1	85403	ENSG00000144597	validated
tarbase	hsa-miR-222-3p	EAF1	85403	ENSG00000144597	validated
tarbase	hsa-miR-212-3p	GASK1A	729085	ENSG00000144649	validated
tarbase	hsa-miR-181a-5p	CSRNP1	64651	ENSG00000144655	validated
tarbase	hsa-miR-301a-3p	CSRNP1	64651	ENSG00000144655	validated

tarbase	hsa-miR-181a-5p	GOLGA4	2803	ENSG00000144674	validated
tarbase	hsa-miR-18a-5p	GOLGA4	2803	ENSG00000144674	validated
tarbase	hsa-miR-326	GOLGA4	2803	ENSG00000144674	validated
tarbase	hsa-miR-505-3p	GOLGA4	2803	ENSG00000144674	validated
tarbase	hsa-miR-212-3p	CTDSPL	10217	ENSG00000144677	validated
tarbase	hsa-miR-106b-5p	IL17RD	54756	ENSG00000144730	validated
tarbase	hsa-miR-582-5p	IL17RD	54756	ENSG00000144730	validated
tarbase	hsa-miR-222-3p	UBA3	9039	ENSG00000144744	validated
tarbase	hsa-miR-15b-5p	ARL6IP5	10550	ENSG00000144746	validated
tarbase	hsa-miR-106b-5p	LRIG1	26018	ENSG00000144749	validated
tarbase	hsa-miR-15b-5p	LRIG1	26018	ENSG00000144749	validated
tarbase	hsa-miR-301a-3p	LRIG1	26018	ENSG00000144749	validated
tarbase	hsa-miR-192-5p	LIMD1	8994	ENSG00000144791	validated
tarbase	hsa-miR-21-5p	NFKBIZ	64332	ENSG00000144802	validated
tarbase	hsa-miR-106b-5p	ABHD10	55347	ENSG00000144827	validated
tarbase	hsa-miR-222-3p	ABHD10	55347	ENSG00000144827	validated
tarbase	hsa-miR-106b-5p	ATG3	64422	ENSG00000144848	validated
tarbase	hsa-miR-301a-3p	SRPRB	58477	ENSG00000144867	validated
tarbase	hsa-miR-106b-5p	MED12L	116931	ENSG00000144893	validated
tarbase	hsa-miR-18a-5p	OSBPL11	114885	ENSG00000144909	validated
tarbase	hsa-miR-21-5p	OSBPL11	114885	ENSG00000144909	validated
tarbase	hsa-miR-212-3p	TRPC1	7220	ENSG00000144935	validated
tarbase	hsa-miR-21-5p	NCEH1	57552	ENSG00000144959	validated
tarbase	hsa-miR-301a-3p	NCEH1	57552	ENSG00000144959	validated
tarbase	hsa-miR-212-3p	NCEH1	57552	ENSG00000144959	validated
tarbase	hsa-miR-181a-5p	LPP	4026	ENSG00000145012	validated
tarbase	hsa-miR-21-5p	LPP	4026	ENSG00000145012	validated
tarbase	hsa-miR-106b-5p	RUBCN	9711	ENSG00000145016	validated
tarbase	hsa-miR-21-5p	CCDC39		ENSG00000145075	validated
tarbase	hsa-miR-15b-5p	SLIT2	9353	ENSG00000145147	validated
tarbase	hsa-miR-21-5p	SLIT2	9353	ENSG00000145147	validated
tarbase	hsa-miR-15b-5p	EIF2B5	8893	ENSG00000145191	validated
tarbase	hsa-miR-106b-5p	SCD5	79966	ENSG00000145284	validated
tarbase	hsa-miR-181a-5p	SCD5	79966	ENSG00000145284	validated
tarbase	hsa-miR-212-3p	TRMT10A	93587	ENSG00000145331	validated
tarbase	hsa-miR-106b-5p	KLHL8	57563	ENSG00000145332	validated
tarbase	hsa-miR-21-5p	KLHL8	57563	ENSG00000145332	validated
tarbase	hsa-miR-18a-5p	SNCA	6622	ENSG00000145335	validated
tarbase	hsa-miR-106b-5p	PYURF	100996939	ENSG00000145337	validated
tarbase	hsa-miR-301a-3p	CAMK2D	817	ENSG00000145349	validated
tarbase	hsa-miR-181a-5p	CAMK2D	817	ENSG00000145349	validated
tarbase	hsa-miR-15b-5p	ANK2	287	ENSG00000145362	validated
tarbase	hsa-miR-106b-5p	CCNA2	890	ENSG00000145386	validated
tarbase	hsa-miR-15b-5p	CCNA2	890	ENSG00000145386	validated
tarbase	hsa-miR-18a-5p	CCNA2	890	ENSG00000145386	validated
tarbase	hsa-miR-106b-5p	METTL14	57721	ENSG00000145388	validated
tarbase	hsa-miR-181a-5p	USP53	54532	ENSG00000145390	validated
tarbase	hsa-miR-181a-5p	SETD7	80854	ENSG00000145391	validated
tarbase	hsa-miR-15b-5p	PDGFC	56034	ENSG00000145431	validated
tarbase	hsa-miR-15b-5p	MARCHF6	10299	ENSG00000145495	validated
tarbase	hsa-miR-181a-5p	MARCHF6	10299	ENSG00000145495	validated
tarbase	hsa-miR-18a-5p	MARCHF6	10299	ENSG00000145495	validated
tarbase	hsa-miR-21-5p	MARCHF6	10299	ENSG00000145495	validated
tarbase	hsa-miR-582-5p	MARCHF6	10299	ENSG00000145495	validated
tarbase	hsa-miR-106b-5p	MYO10	4651	ENSG00000145555	validated
tarbase	hsa-miR-181a-5p	MYO10	4651	ENSG00000145555	validated
tarbase	hsa-miR-296-5p	MYO10	4651	ENSG00000145555	validated
tarbase	hsa-miR-505-3p	MYO10	4651	ENSG00000145555	validated
tarbase	hsa-miR-106b-5p	OTULINL	54491	ENSG00000145569	validated
tarbase	hsa-miR-582-5p	OTULINL	54491	ENSG00000145569	validated
tarbase	hsa-miR-301a-3p	SKP2	6502	ENSG00000145604	validated
tarbase	hsa-miR-582-5p	SKP2	6502	ENSG00000145604	validated
tarbase	hsa-miR-301a-3p	OSMR	9180	ENSG00000145623	validated
tarbase	hsa-miR-106b-5p	PIK3R1	5295	ENSG00000145675	validated
tarbase	hsa-miR-181a-5p	PIK3R1	5295	ENSG00000145675	validated
tarbase	hsa-miR-212-3p	PIK3R1	5295	ENSG00000145675	validated
tarbase	hsa-miR-181a-5p	LHFPL2	10184	ENSG00000145685	validated
tarbase	hsa-miR-212-3p	LHFPL2	10184	ENSG00000145685	validated
tarbase	hsa-miR-222-3p	LHFPL2	10184	ENSG00000145685	validated
tarbase	hsa-miR-181a-5p	SSBP2	23635	ENSG00000145687	validated
tarbase	hsa-miR-15b-5p	RASA1	5921	ENSG00000145715	validated

tarbase	hsa-miR-181a-5p	RASA1	5921	ENSG00000145715	validated
tarbase	hsa-miR-301a-3p	RASA1	5921	ENSG00000145715	validated
tarbase	hsa-miR-212-3p	GIN1	54826	ENSG00000145723	validated
tarbase	hsa-miR-181a-5p	PPIP5K2	23262	ENSG00000145725	validated
tarbase	hsa-miR-212-3p	PPIP5K2	23262	ENSG00000145725	validated
tarbase	hsa-miR-106b-5p	PAM	5066	ENSG00000145730	validated
tarbase	hsa-miR-15b-5p	PAM	5066	ENSG00000145730	validated
tarbase	hsa-miR-181a-5p	PAM	5066	ENSG00000145730	validated
tarbase	hsa-miR-301a-3p	PAM	5066	ENSG00000145730	validated
tarbase	hsa-miR-106b-5p	BDP1	55814	ENSG00000145734	validated
tarbase	hsa-miR-15b-5p	BDP1	55814	ENSG00000145734	validated
tarbase	hsa-miR-181a-5p	TNFAIP8	25816	ENSG00000145779	validated
tarbase	hsa-miR-15b-5p	FEM1C	56929	ENSG00000145780	validated
tarbase	hsa-miR-192-5p	FEM1C	56929	ENSG00000145780	validated
tarbase	hsa-miR-212-3p	FEM1C	56929	ENSG00000145780	validated
tarbase	hsa-miR-582-3p	FEM1C	56929	ENSG00000145780	validated
tarbase	hsa-miR-106b-5p	COMMD10	51397	ENSG00000145781	validated
tarbase	hsa-miR-222-3p	ADAMTS19	171019	ENSG00000145808	validated
tarbase	hsa-miR-15b-5p	YIPF5	81555	ENSG00000145817	validated
tarbase	hsa-miR-181a-5p	YIPF5	81555	ENSG00000145817	validated
tarbase	hsa-miR-15b-5p	DDX46	9879	ENSG00000145833	validated
tarbase	hsa-miR-18a-5p	RNF145	153830	ENSG00000145860	validated
tarbase	hsa-miR-181a-5p	RNF145	153830	ENSG00000145860	validated
tarbase	hsa-miR-106b-5p	G3BP1	10146	ENSG00000145907	validated
tarbase	hsa-miR-144-3p	G3BP1	10146	ENSG00000145907	validated
tarbase	hsa-miR-15b-5p	G3BP1	10146	ENSG00000145907	validated
tarbase	hsa-miR-181a-5p	G3BP1	10146	ENSG00000145907	validated
tarbase	hsa-miR-192-5p	G3BP1	10146	ENSG00000145907	validated
tarbase	hsa-miR-212-3p	G3BP1	10146	ENSG00000145907	validated
tarbase	hsa-miR-21-5p	G3BP1	10146	ENSG00000145907	validated
tarbase	hsa-miR-222-3p	G3BP1	10146	ENSG00000145907	validated
tarbase	hsa-miR-301a-3p	G3BP1	10146	ENSG00000145907	validated
tarbase	hsa-miR-582-3p	G3BP1	10146	ENSG00000145907	validated
tarbase	hsa-miR-21-5p	RMND5B	64777	ENSG00000145916	validated
tarbase	hsa-miR-582-5p	RMND5B	64777	ENSG00000145916	validated
tarbase	hsa-miR-21-5p	BOD1	91272	ENSG00000145919	validated
tarbase	hsa-miR-296-5p	BOD1	91272	ENSG00000145919	validated
tarbase	hsa-miR-301a-3p	TENM2	57451	ENSG00000145934	validated
tarbase	hsa-miR-301a-3p	FAM50B	26240	ENSG00000145945	validated
tarbase	hsa-miR-212-3p	MYLK4	340156	ENSG00000145949	validated
tarbase	hsa-miR-192-5p	GFOD1	54438	ENSG00000145990	validated
tarbase	hsa-miR-15b-5p	LRRTM2	26045	ENSG00000146006	validated
tarbase	hsa-miR-582-3p	ZMAT2	153527	ENSG00000146007	validated
tarbase	hsa-miR-21-5p	KLHL3	26249	ENSG00000146021	validated
tarbase	hsa-miR-296-5p	KLHL3	26249	ENSG00000146021	validated
tarbase	hsa-miR-301a-3p	KLHL3	26249	ENSG00000146021	validated
tarbase	hsa-miR-301a-3p	DCDC2	51473	ENSG00000146038	validated
tarbase	hsa-miR-212-3p	H2BC1	255626	ENSG00000146047	validated
tarbase	hsa-miR-21-5p	TRIM7	81786	ENSG00000146054	validated
tarbase	hsa-miR-21-5p	TRIM41	90933	ENSG00000146063	validated
tarbase	hsa-miR-296-5p	FAM193B	54540	ENSG00000146067	validated
tarbase	hsa-miR-222-3p	RNF44	22838	ENSG00000146083	validated
tarbase	hsa-miR-15b-5p	MMUT	4594	ENSG00000146085	validated
tarbase	hsa-miR-144-3p	ABT1	29777	ENSG00000146109	validated
tarbase	hsa-miR-18a-5p	RPL7L1	285855	ENSG00000146223	validated
tarbase	hsa-miR-18a-5p	CYP39A1	51302	ENSG00000146233	validated
tarbase	hsa-miR-212-3p	CYP39A1	51302	ENSG00000146233	validated
tarbase	hsa-miR-106b-5p	TPBG	7162	ENSG00000146242	validated
tarbase	hsa-miR-106b-5p	IRAK1BP1	134728	ENSG00000146243	validated
tarbase	hsa-miR-106b-5p	PHIP	55023	ENSG00000146247	validated
tarbase	hsa-miR-192-5p	PHIP	55023	ENSG00000146247	validated
tarbase	hsa-miR-212-3p	PHIP	55023	ENSG00000146247	validated
tarbase	hsa-miR-301a-3p	PHIP	55023	ENSG00000146247	validated
tarbase	hsa-miR-582-3p	PHIP	55023	ENSG00000146247	validated
tarbase	hsa-miR-106b-5p	MMS22L	253714	ENSG00000146263	validated
tarbase	hsa-miR-505-3p	MMS22L	253714	ENSG00000146263	validated
tarbase	hsa-miR-18a-5p	PNRC1	10957	ENSG00000146278	validated
tarbase	hsa-miR-106b-5p	PM20D2	135293	ENSG00000146281	validated
tarbase	hsa-miR-212-3p	PM20D2	135293	ENSG00000146281	validated
tarbase	hsa-miR-222-3p	RARS2	57038	ENSG00000146282	validated
tarbase	hsa-miR-181a-5p	CLVS2	134829	ENSG00000146352	validated

tarbase	hsa-miR-106b-5p	GPR6	2830	ENSG00000146360	validated
tarbase	hsa-miR-106b-5p	RNF217	154214	ENSG00000146373	validated
tarbase	hsa-miR-15b-5p	RNF217	154214	ENSG00000146373	validated
tarbase	hsa-miR-181a-5p	RNF217	154214	ENSG00000146373	validated
tarbase	hsa-miR-15b-5p	ARHGAP18	93663	ENSG00000146376	validated
tarbase	hsa-miR-181a-5p	ARHGAP18	93663	ENSG00000146376	validated
tarbase	hsa-miR-106b-5p	MTFR2	113115	ENSG00000146410	validated
tarbase	hsa-miR-192-5p	DYNLT1	6993	ENSG00000146425	validated
tarbase	hsa-miR-222-3p	TIAM2	26230	ENSG00000146426	validated
tarbase	hsa-miR-212-3p	TIAM2	26230	ENSG00000146426	validated
tarbase	hsa-miR-106b-5p	TMEM181	57583	ENSG00000146433	validated
tarbase	hsa-miR-15b-5p	TMEM181	57583	ENSG00000146433	validated
tarbase	hsa-miR-18a-5p	TMEM181	57583	ENSG00000146433	validated
tarbase	hsa-miR-212-3p	TMEM181	57583	ENSG00000146433	validated
tarbase	hsa-miR-582-5p	TMEM181	57583	ENSG00000146433	validated
tarbase	hsa-miR-181a-5p	TMEM181	57583	ENSG00000146433	validated
tarbase	hsa-miR-106b-5p	WTAP	9589	ENSG00000146457	validated
tarbase	hsa-miR-15b-5p	WTAP	9589	ENSG00000146457	validated
tarbase	hsa-miR-212-3p	WTAP	9589	ENSG00000146457	validated
tarbase	hsa-miR-181a-5p	WTAP	9589	ENSG00000146457	validated
tarbase	hsa-miR-181a-5p	VIP	7432	ENSG00000146469	validated
tarbase	hsa-miR-181a-5p	ARMT1	79624	ENSG00000146476	validated
tarbase	hsa-miR-301a-3p	ARMT1	79624	ENSG00000146476	validated
tarbase	hsa-miR-106b-5p	SLC22A3	6581	ENSG00000146477	validated
tarbase	hsa-miR-212-3p	SLC22A3	6581	ENSG00000146477	validated
tarbase	hsa-miR-15b-5p	GNA12	2768	ENSG00000146535	validated
tarbase	hsa-miR-181a-5p	GNA12	2768	ENSG00000146535	validated
tarbase	hsa-miR-326	C7orf50	84310	ENSG00000146540	validated
tarbase	hsa-miR-181a-5p	RBAK	57786	ENSG00000146587	validated
tarbase	hsa-miR-296-5p	RBAK	57786	ENSG00000146587	validated
tarbase	hsa-miR-106b-5p	CREB5	9586	ENSG00000146592	validated
tarbase	hsa-miR-212-3p	CREB5	9586	ENSG00000146592	validated
tarbase	hsa-miR-301a-3p	CREB5	9586	ENSG00000146592	validated
tarbase	hsa-miR-181a-5p	EGFR	1956	ENSG00000146648	validated
tarbase	hsa-miR-15b-5p	CDCA5	113130	ENSG00000146670	validated
tarbase	hsa-miR-15b-5p	IGFBP3	3486	ENSG00000146674	validated
tarbase	hsa-miR-21-5p	IGFBP3	3486	ENSG00000146674	validated
tarbase	hsa-miR-18a-5p	PURB	5814	ENSG00000146676	validated
tarbase	hsa-miR-192-5p	PURB	5814	ENSG00000146676	validated
tarbase	hsa-miR-301a-3p	PURB	5814	ENSG00000146676	validated
tarbase	hsa-miR-15b-5p	MDH2	4191	ENSG00000146701	validated
tarbase	hsa-miR-181a-5p	MDH2	4191	ENSG00000146701	validated
tarbase	hsa-miR-212-3p	POMZP3	22932	ENSG00000146707	validated
tarbase	hsa-miR-181a-5p	CCT6A	908	ENSG00000146731	validated
tarbase	hsa-miR-106b-5p	PSPH	5723	ENSG00000146733	validated
tarbase	hsa-miR-181a-5p	ZNF92	168374	ENSG00000146757	validated
tarbase	hsa-miR-106b-5p	TMEM168	64418	ENSG00000146802	validated
tarbase	hsa-miR-301a-3p	TMEM168	64418	ENSG00000146802	validated
tarbase	hsa-miR-582-5p	TMEM168	64418	ENSG00000146802	validated
tarbase	hsa-miR-18a-5p	MAP11	55262	ENSG00000146826	validated
tarbase	hsa-miR-296-5p	MAP11	55262	ENSG00000146826	validated
tarbase	hsa-miR-15b-5p	GIGYF1	64599	ENSG00000146830	validated
tarbase	hsa-miR-192-5p	GIGYF1	64599	ENSG00000146830	validated
tarbase	hsa-miR-326	GIGYF1	64599	ENSG00000146830	validated
tarbase	hsa-miR-21-5p	GIGYF1	64599	ENSG00000146830	validated
tarbase	hsa-miR-505-3p	MEPCE	56257	ENSG00000146834	validated
tarbase	hsa-miR-582-3p	MEPCE	56257	ENSG00000146834	validated
tarbase	hsa-miR-106b-5p	TMEM209	84928	ENSG00000146842	validated
tarbase	hsa-miR-18a-5p	TMEM209	84928	ENSG00000146842	validated
tarbase	hsa-miR-21-5p	TLK2	11011	ENSG00000146872	validated
tarbase	hsa-miR-212-3p	CNPY1	285888	ENSG00000146910	validated
tarbase	hsa-miR-106b-5p	NCAPG2	54892	ENSG00000146918	validated
tarbase	hsa-miR-181a-5p	LUC7L2	51631	ENSG00000146963	validated
tarbase	hsa-miR-18a-5p	LUC7L2	51631	ENSG00000146963	validated
tarbase	hsa-miR-21-5p	LUC7L2	51631	ENSG00000146963	validated
tarbase	hsa-miR-582-5p	LUC7L2	51631	ENSG00000146963	validated
tarbase	hsa-miR-212-3p	CLTRN	57393	ENSG00000147003	validated
tarbase	hsa-miR-15b-5p	TMEM47	83604	ENSG00000147027	validated
tarbase	hsa-miR-15b-5p	KDM6A	7403	ENSG00000147050	validated
tarbase	hsa-miR-212-3p	KDM6A	7403	ENSG00000147050	validated
tarbase	hsa-miR-15b-5p	MSN	4478	ENSG00000147065	validated

tarbase	hsa-miR-18a-5p	ZNF182	7569	ENSG00000147118	validated
tarbase	hsa-miR-15b-5p	ZMYM3	9203	ENSG00000147130	validated
tarbase	hsa-miR-301a-3p	ZMYM3	9203	ENSG00000147130	validated
tarbase	hsa-miR-296-5p	TAF1	6872	ENSG00000147133	validated
tarbase	hsa-miR-15b-5p	NONO	4841	ENSG00000147140	validated
tarbase	hsa-miR-192-5p	NONO	4841	ENSG00000147140	validated
tarbase	hsa-miR-181a-5p	OGT	8473	ENSG00000147162	validated
tarbase	hsa-miR-582-5p	OGT	8473	ENSG00000147162	validated
tarbase	hsa-miR-212-3p	SNX12	29934	ENSG00000147164	validated
tarbase	hsa-miR-301a-3p	SNX12	29934	ENSG00000147164	validated
tarbase	hsa-miR-21-5p	GCNA	93953	ENSG00000147174	validated
tarbase	hsa-miR-106b-5p	ZNF711	7552	ENSG00000147180	validated
tarbase	hsa-miR-144-3p	ZNF711	7552	ENSG00000147180	validated
tarbase	hsa-miR-181a-5p	ZNF711	7552	ENSG00000147180	validated
tarbase	hsa-miR-222-3p	ZNF711	7552	ENSG00000147180	validated
tarbase	hsa-miR-326	ZNF711	7552	ENSG00000147180	validated
tarbase	hsa-miR-582-3p	ZNF711	7552	ENSG00000147180	validated
tarbase	hsa-miR-582-5p	ZNF711	7552	ENSG00000147180	validated
tarbase	hsa-miR-222-3p	PRPS1	5631	ENSG00000147224	validated
tarbase	hsa-miR-212-3p	PRPS1	5631	ENSG00000147224	validated
tarbase	hsa-miR-15b-5p	FRMPD3	84443	ENSG00000147234	validated
tarbase	hsa-miR-21-5p	DOCK11	139818	ENSG00000147251	validated
tarbase	hsa-miR-15b-5p	GPC3	2719	ENSG00000147257	validated
tarbase	hsa-miR-301a-3p	MCPH1	79648	ENSG00000147316	validated
tarbase	hsa-miR-181a-5p	MFHAS1	9258	ENSG00000147324	validated
tarbase	hsa-miR-296-5p	MFHAS1	9258	ENSG00000147324	validated
tarbase	hsa-miR-18a-5p	MFHAS1	9258	ENSG00000147324	validated
tarbase	hsa-miR-21-5p	ZNF185	7739	ENSG00000147394	validated
tarbase	hsa-miR-106b-5p	CSGALNACT1	55790	ENSG00000147408	validated
tarbase	hsa-miR-582-5p	CCDC25	55246	ENSG00000147419	validated
tarbase	hsa-miR-15b-5p	ERLIN2	11160	ENSG00000147475	validated
tarbase	hsa-miR-296-5p	ERLIN2	11160	ENSG00000147475	validated
tarbase	hsa-miR-582-3p	ERLIN2	11160	ENSG00000147475	validated
tarbase	hsa-miR-106b-5p	ERLIN2	11160	ENSG00000147475	validated
tarbase	hsa-miR-15b-5p	TACC1	6867	ENSG00000147526	validated
tarbase	hsa-miR-21-5p	TACC1	6867	ENSG00000147526	validated
tarbase	hsa-miR-222-3p	TACC1	6867	ENSG00000147526	validated
tarbase	hsa-miR-301a-3p	TACC1	6867	ENSG00000147526	validated
tarbase	hsa-miR-326	GOLGA7	51125	ENSG00000147533	validated
tarbase	hsa-miR-181a-5p	GOLGA7	51125	ENSG00000147533	validated
tarbase	hsa-miR-212-3p	GINS4	84296	ENSG00000147536	validated
tarbase	hsa-miR-106b-5p	NSD3	54904	ENSG00000147548	validated
tarbase	hsa-miR-505-3p	NSD3	54904	ENSG00000147548	validated
tarbase	hsa-miR-15b-5p	NSD3	54904	ENSG00000147548	validated
tarbase	hsa-miR-181a-5p	NSD3	54904	ENSG00000147548	validated
tarbase	hsa-miR-15b-5p	PMP2	5375	ENSG00000147588	validated
tarbase	hsa-miR-144-3p	LACTB2	51110	ENSG00000147592	validated
tarbase	hsa-miR-18a-5p	LACTB2	51110	ENSG00000147592	validated
tarbase	hsa-miR-106b-5p	SYBU	55638	ENSG00000147642	validated
tarbase	hsa-miR-21-5p	SYBU	55638	ENSG00000147642	validated
tarbase	hsa-miR-301a-3p	SYBU	55638	ENSG00000147642	validated
tarbase	hsa-miR-582-3p	SYBU	55638	ENSG00000147642	validated
tarbase	hsa-miR-212-3p	DPYS	1807	ENSG00000147647	validated
tarbase	hsa-miR-106b-5p	MTDH	92140	ENSG00000147649	validated
tarbase	hsa-miR-582-5p	MTDH	92140	ENSG00000147649	validated
tarbase	hsa-miR-181a-5p	POLR2K	5440	ENSG00000147669	validated
tarbase	hsa-miR-301a-3p	MAL2	114569	ENSG00000147676	validated
tarbase	hsa-miR-106b-5p	UTP23	84294	ENSG00000147679	validated
tarbase	hsa-miR-106b-5p	VLDLR	7436	ENSG00000147852	validated
tarbase	hsa-miR-212-3p	VLDLR	7436	ENSG00000147852	validated
tarbase	hsa-miR-21-5p	VLDLR	7436	ENSG00000147852	validated
tarbase	hsa-miR-192-5p	VLDLR	7436	ENSG00000147852	validated
tarbase	hsa-miR-106b-5p	AK3	50808	ENSG00000147853	validated
tarbase	hsa-miR-181a-5p	AK3	50808	ENSG00000147853	validated
tarbase	hsa-miR-15b-5p	NFIB	4781	ENSG00000147862	validated
tarbase	hsa-miR-181a-5p	NFIB	4781	ENSG00000147862	validated
tarbase	hsa-miR-212-3p	NFIB	4781	ENSG00000147862	validated
tarbase	hsa-miR-15b-5p	FBXO10	26267	ENSG00000147912	validated
tarbase	hsa-miR-301a-3p	FBXO10	26267	ENSG00000147912	validated
tarbase	hsa-miR-18a-5p	SIGMAR1	10280	ENSG00000147955	validated
tarbase	hsa-miR-212-3p	CEP78	84131	ENSG00000148019	validated

tarbase	hsa-miR-15b-5p	NTRK2	4915	ENSG00000148053	validated
tarbase	hsa-miR-181a-5p	NTRK2	4915	ENSG00000148053	validated
tarbase	hsa-miR-15b-5p	MFSD14B	84641	ENSG00000148110	validated
tarbase	hsa-miR-21-5p	ZNF462	58499	ENSG00000148143	validated
tarbase	hsa-miR-106b-5p	INIP	58493	ENSG00000148153	validated
tarbase	hsa-miR-181a-5p	INIP	58493	ENSG00000148153	validated
tarbase	hsa-miR-144-3p	UGCG	7357	ENSG00000148154	validated
tarbase	hsa-miR-181a-5p	UGCG	7357	ENSG00000148154	validated
tarbase	hsa-miR-21-5p	UGCG	7357	ENSG00000148154	validated
tarbase	hsa-miR-222-3p	UGCG	7357	ENSG00000148154	validated
tarbase	hsa-miR-106b-5p	SNX30	401548	ENSG00000148158	validated
tarbase	hsa-miR-15b-5p	SNX30	401548	ENSG00000148158	validated
tarbase	hsa-miR-582-3p	STOM	2040	ENSG00000148175	validated
tarbase	hsa-miR-15b-5p	STOM	2040	ENSG00000148175	validated
tarbase	hsa-miR-18a-5p	NR6A1	2649	ENSG00000148200	validated
tarbase	hsa-miR-301a-3p	NR6A1	2649	ENSG00000148200	validated
tarbase	hsa-miR-106b-5p	POLE3	54107	ENSG00000148229	validated
tarbase	hsa-miR-18a-5p	POLE3	54107	ENSG00000148229	validated
tarbase	hsa-miR-15b-5p	SURF4	6836	ENSG00000148248	validated
tarbase	hsa-miR-18a-5p	SURF4	6836	ENSG00000148248	validated
tarbase	hsa-miR-212-3p	SURF4	6836	ENSG00000148248	validated
tarbase	hsa-miR-212-3p	SURF2	6835	ENSG00000148291	validated
tarbase	hsa-miR-212-3p	ASB6	140459	ENSG00000148331	validated
tarbase	hsa-miR-21-5p	ASB6	140459	ENSG00000148331	validated
tarbase	hsa-miR-21-5p	PTGES2	80142	ENSG00000148334	validated
tarbase	hsa-miR-15b-5p	NTMT1	28989	ENSG00000148335	validated
tarbase	hsa-miR-301a-3p	SLC25A25	114789	ENSG00000148339	validated
tarbase	hsa-miR-222-3p	LRSAM1	90678	ENSG00000148356	validated
tarbase	hsa-miR-15b-5p	INPP5E	56623	ENSG00000148384	validated
tarbase	hsa-miR-296-5p	INPP5E	56623	ENSG00000148384	validated
tarbase	hsa-miR-181a-5p	SEC16A	9919	ENSG00000148396	validated
tarbase	hsa-miR-212-3p	SEC16A	9919	ENSG00000148396	validated
tarbase	hsa-miR-296-5p	SEC16A	9919	ENSG00000148396	validated
tarbase	hsa-miR-15b-5p	NACC2	138151	ENSG00000148411	validated
tarbase	hsa-miR-181a-5p	NACC2	138151	ENSG00000148411	validated
tarbase	hsa-miR-212-3p	NACC2	138151	ENSG00000148411	validated
tarbase	hsa-miR-222-3p	NACC2	138151	ENSG00000148411	validated
tarbase	hsa-miR-181a-5p	USP6NL	9712	ENSG00000148429	validated
tarbase	hsa-miR-106b-5p	COMMD3	23412	ENSG00000148444	validated
tarbase	hsa-miR-212-3p	MSRB2	22921	ENSG00000148450	validated
tarbase	hsa-miR-15b-5p	FAM171A1	221061	ENSG00000148468	validated
tarbase	hsa-miR-181a-5p	FAM171A1	221061	ENSG00000148468	validated
tarbase	hsa-miR-326	PARD3	56288	ENSG00000148498	validated
tarbase	hsa-miR-212-3p	FAM13C	220965	ENSG00000148541	validated
tarbase	hsa-miR-106b-5p	NRBF2	29982	ENSG00000148572	validated
tarbase	hsa-miR-212-3p	CDHR1	92211	ENSG00000148600	validated
tarbase	hsa-miR-15b-5p	POLR3A	11128	ENSG00000148606	validated
tarbase	hsa-miR-181a-5p	HERC4	26091	ENSG00000148634	validated
tarbase	hsa-miR-212-3p	HERC4	26091	ENSG00000148634	validated
tarbase	hsa-miR-212-3p	ADIRF	10974	ENSG00000148671	validated
tarbase	hsa-miR-212-3p	ANKRD1	27063	ENSG00000148677	validated
tarbase	hsa-miR-181a-5p	ADD3	120	ENSG00000148700	validated
tarbase	hsa-miR-18a-5p	ADD3	120	ENSG00000148700	validated
tarbase	hsa-miR-21-5p	ADD3	120	ENSG00000148700	validated
tarbase	hsa-miR-181a-5p	EIF4EBP2	1979	ENSG00000148730	validated
tarbase	hsa-miR-222-3p	EIF4EBP2	1979	ENSG00000148730	validated
tarbase	hsa-miR-326	EIF4EBP2	1979	ENSG00000148730	validated
tarbase	hsa-miR-144-3p	TCF7L2	6934	ENSG00000148737	validated
tarbase	hsa-miR-181a-5p	TCF7L2	6934	ENSG00000148737	validated
tarbase	hsa-miR-106b-5p	MKI67	4288	ENSG00000148773	validated
tarbase	hsa-miR-222-3p	MKI67	4288	ENSG00000148773	validated
tarbase	hsa-miR-212-3p	INA	9118	ENSG00000148798	validated
tarbase	hsa-miR-296-5p	GSTO1	9446	ENSG00000148834	validated
tarbase	hsa-miR-582-5p	TAF5	6877	ENSG00000148835	validated
tarbase	hsa-miR-15b-5p	PPRC1	23082	ENSG00000148840	validated
tarbase	hsa-miR-212-3p	PPRC1	23082	ENSG00000148840	validated
tarbase	hsa-miR-301a-3p	PPRC1	23082	ENSG00000148840	validated
tarbase	hsa-miR-582-3p	PPRC1	23082	ENSG00000148840	validated
tarbase	hsa-miR-582-3p	ITPRIP	85450	ENSG00000148841	validated
tarbase	hsa-miR-582-5p	CNNM2	54805	ENSG00000148842	validated
tarbase	hsa-miR-582-3p	PDCD11	22984	ENSG00000148843	validated

tarbase	hsa-miR-181a-5p	ADAM12	8038	ENSG00000148848	validated
tarbase	hsa-miR-106b-5p	BTBD10	84280	ENSG00000148925	validated
tarbase	hsa-miR-181a-5p	ADM	133	ENSG00000148926	validated
tarbase	hsa-miR-181a-5p	LIN7C	55327	ENSG00000148943	validated
tarbase	hsa-miR-222-3p	LIN7C	55327	ENSG00000148943	validated
tarbase	hsa-miR-192-5p	LIN7C	55327	ENSG00000148943	validated
tarbase	hsa-miR-212-3p	IMMP1L	196294	ENSG00000148950	validated
tarbase	hsa-miR-15b-5p	TUT1	64852	ENSG00000149016	validated
tarbase	hsa-miR-582-3p	EIF3M	10480	ENSG00000149100	validated
tarbase	hsa-miR-106b-5p	TNKS1BP1	85456	ENSG00000149115	validated
tarbase	hsa-miR-15b-5p	TNKS1BP1	85456	ENSG00000149115	validated
tarbase	hsa-miR-21-5p	TNKS1BP1	85456	ENSG00000149115	validated
tarbase	hsa-miR-212-3p	PTPRJ	5795	ENSG00000149177	validated
tarbase	hsa-miR-222-3p	PTPRJ	5795	ENSG00000149177	validated
tarbase	hsa-miR-301a-3p	PTPRJ	5795	ENSG00000149177	validated
tarbase	hsa-miR-582-5p	PTPRJ	5795	ENSG00000149177	validated
tarbase	hsa-miR-15b-5p	C11orf49	79096	ENSG00000149179	validated
tarbase	hsa-miR-106b-5p	CELF1	10658	ENSG00000149187	validated
tarbase	hsa-miR-15b-5p	CELF1	10658	ENSG00000149187	validated
tarbase	hsa-miR-181a-5p	CELF1	10658	ENSG00000149187	validated
tarbase	hsa-miR-18a-5p	CELF1	10658	ENSG00000149187	validated
tarbase	hsa-miR-192-5p	CELF1	10658	ENSG00000149187	validated
tarbase	hsa-miR-21-5p	CELF1	10658	ENSG00000149187	validated
tarbase	hsa-miR-296-5p	CELF1	10658	ENSG00000149187	validated
tarbase	hsa-miR-301a-3p	CELF1	10658	ENSG00000149187	validated
tarbase	hsa-miR-326	CELF1	10658	ENSG00000149187	validated
tarbase	hsa-miR-505-3p	CELF1	10658	ENSG00000149187	validated
tarbase	hsa-miR-15b-5p	SESN3	143686	ENSG00000149212	validated
tarbase	hsa-miR-181a-5p	SESN3	143686	ENSG00000149212	validated
tarbase	hsa-miR-222-3p	SESN3	143686	ENSG00000149212	validated
tarbase	hsa-miR-301a-3p	SESN3	143686	ENSG00000149212	validated
tarbase	hsa-miR-181a-5p	ENDOD1	23052	ENSG00000149218	validated
tarbase	hsa-miR-15b-5p	CCDC82	79780	ENSG00000149231	validated
tarbase	hsa-miR-181a-5p	CCDC82	79780	ENSG00000149231	validated
tarbase	hsa-miR-212-3p	SERPINH1	871	ENSG00000149257	validated
tarbase	hsa-miR-505-3p	RPS3	6188	ENSG00000149273	validated
tarbase	hsa-miR-582-5p	RPS3	6188	ENSG00000149273	validated
tarbase	hsa-miR-181a-5p	ZC3H12C	85463	ENSG00000149289	validated
tarbase	hsa-miR-18a-5p	ZC3H12C	85463	ENSG00000149289	validated
tarbase	hsa-miR-212-3p	ZC3H12C	85463	ENSG00000149289	validated
tarbase	hsa-miR-301a-3p	ZC3H12C	85463	ENSG00000149289	validated
tarbase	hsa-miR-212-3p	ATM	472	ENSG00000149311	validated
tarbase	hsa-miR-15b-5p	LAMTOR1	55004	ENSG00000149357	validated
tarbase	hsa-miR-18a-5p	LAMTOR1	55004	ENSG00000149357	validated
tarbase	hsa-miR-21-5p	LAMTOR1	55004	ENSG00000149357	validated
tarbase	hsa-miR-106b-5p	HYOU1	10525	ENSG00000149428	validated
tarbase	hsa-miR-18a-5p	HYOU1	10525	ENSG00000149428	validated
tarbase	hsa-miR-222-3p	HYOU1	10525	ENSG00000149428	validated
tarbase	hsa-miR-296-5p	HYOU1	10525	ENSG00000149428	validated
tarbase	hsa-miR-15b-5p	KAT14	57325	ENSG00000149474	validated
tarbase	hsa-miR-181a-5p	TMEM138	51524	ENSG00000149483	validated
tarbase	hsa-miR-326	FADS1	3992	ENSG00000149485	validated
tarbase	hsa-miR-106b-5p	EML3	256364	ENSG00000149499	validated
tarbase	hsa-miR-21-5p	EML3	256364	ENSG00000149499	validated
tarbase	hsa-miR-192-5p	EI24	9538	ENSG00000149547	validated
tarbase	hsa-miR-106b-5p	CHEK1	1111	ENSG00000149554	validated
tarbase	hsa-miR-301a-3p	CHEK1	1111	ENSG00000149554	validated
tarbase	hsa-miR-106b-5p	MPZL2	10205	ENSG00000149573	validated
tarbase	hsa-miR-212-3p	SCN2B	6327	ENSG00000149575	validated
tarbase	hsa-miR-106b-5p	TMEM25	84866	ENSG00000149582	validated
tarbase	hsa-miR-192-5p	COMMD7	149951	ENSG00000149600	validated
tarbase	hsa-miR-212-3p	DSN1	79980	ENSG00000149636	validated
tarbase	hsa-miR-106b-5p	SOGA1	140710	ENSG00000149639	validated
tarbase	hsa-miR-15b-5p	SOGA1	140710	ENSG00000149639	validated
tarbase	hsa-miR-181a-5p	SOGA1	140710	ENSG00000149639	validated
tarbase	hsa-miR-296-5p	SOGA1	140710	ENSG00000149639	validated
tarbase	hsa-miR-15b-5p	LSM14B	149986	ENSG00000149657	validated
tarbase	hsa-miR-18a-5p	LSM14B	149986	ENSG00000149657	validated
tarbase	hsa-miR-296-5p	LSM14B	149986	ENSG00000149657	validated
tarbase	hsa-miR-301a-3p	LSM14B	149986	ENSG00000149657	validated
tarbase	hsa-miR-106b-5p	YTHDF1	54915	ENSG00000149658	validated

tarbase	hsa-miR-15b-5p	YTHDF1	54915	ENSG00000149658	validated
tarbase	hsa-miR-18a-5p	YTHDF1	54915	ENSG00000149658	validated
tarbase	hsa-miR-212-3p	CABLES2	81928	ENSG00000149679	validated
tarbase	hsa-miR-21-5p	MRPL49	740	ENSG00000149792	validated
tarbase	hsa-miR-15b-5p	TM7SF2	7108	ENSG00000149809	validated
tarbase	hsa-miR-296-5p	TM7SF2	7108	ENSG00000149809	validated
tarbase	hsa-miR-296-5p	PPP4C	5531	ENSG00000149923	validated
tarbase	hsa-miR-15b-5p	ALDOA	226	ENSG00000149925	validated
tarbase	hsa-miR-18a-5p	ALDOA	226	ENSG00000149925	validated
tarbase	hsa-miR-21-5p	ALDOA	226	ENSG00000149925	validated
tarbase	hsa-miR-296-5p	ALDOA	226	ENSG00000149925	validated
tarbase	hsa-miR-301a-3p	ALDOA	226	ENSG00000149925	validated
tarbase	hsa-miR-15b-5p	DOC2A	8448	ENSG00000149927	validated
tarbase	hsa-miR-106b-5p	TAOK2	9344	ENSG00000149930	validated
tarbase	hsa-miR-15b-5p	TAOK2	9344	ENSG00000149930	validated
tarbase	hsa-miR-18a-5p	TAOK2	9344	ENSG00000149930	validated
tarbase	hsa-miR-296-5p	TAOK2	9344	ENSG00000149930	validated
tarbase	hsa-miR-326	TAOK2	9344	ENSG00000149930	validated
tarbase	hsa-miR-106b-5p	HMGA2	8091	ENSG00000149948	validated
tarbase	hsa-miR-15b-5p	HMGA2	8091	ENSG00000149948	validated
tarbase	hsa-miR-301a-3p	HMGA2	8091	ENSG00000149948	validated
tarbase	hsa-miR-212-3p	MKX	283078	ENSG00000150051	validated
tarbase	hsa-miR-212-3p	MPP7	143098	ENSG00000150054	validated
tarbase	hsa-miR-181a-5p	ITGB1	3688	ENSG00000150093	validated
tarbase	hsa-miR-582-3p	ITGB1	3688	ENSG00000150093	validated
tarbase	hsa-miR-18a-5p	CWC15	51503	ENSG00000150316	validated
tarbase	hsa-miR-15b-5p	ARID5B	84159	ENSG00000150347	validated
tarbase	hsa-miR-212-3p	ARID5B	84159	ENSG00000150347	validated
tarbase	hsa-miR-21-5p	ARID5B	84159	ENSG00000150347	validated
tarbase	hsa-miR-301a-3p	ARID5B	84159	ENSG00000150347	validated
tarbase	hsa-miR-181a-5p	TMCO3	55002	ENSG00000150403	validated
tarbase	hsa-miR-106b-5p	LATS2	26524	ENSG00000150457	validated
tarbase	hsa-miR-15b-5p	LATS2	26524	ENSG00000150457	validated
tarbase	hsa-miR-301a-3p	LATS2	26524	ENSG00000150457	validated
tarbase	hsa-miR-15b-5p	KIAA1328	57536	ENSG00000150477	validated
tarbase	hsa-miR-301a-3p	LYPD1	116372	ENSG00000150551	validated
tarbase	hsa-miR-144-3p	PDCD4	27250	ENSG00000150593	validated
tarbase	hsa-miR-181a-5p	PDCD4	27250	ENSG00000150593	validated
tarbase	hsa-miR-181a-5p	GPM6A	2823	ENSG00000150625	validated
tarbase	hsa-miR-212-3p	SPATA4	132851	ENSG00000150628	validated
tarbase	hsa-miR-21-5p	VEGFC	7424	ENSG00000150630	validated
tarbase	hsa-miR-15b-5p	CCDC102B	79839	ENSG00000150636	validated
tarbase	hsa-miR-212-3p	FSIP1	161835	ENSG00000150667	validated
tarbase	hsa-miR-582-5p	PRSS23	11098	ENSG00000150687	validated
tarbase	hsa-miR-21-5p	CCT5	22948	ENSG00000150753	validated
tarbase	hsa-miR-222-3p	CCT5	22948	ENSG00000150753	validated
tarbase	hsa-miR-582-5p	CCT5	22948	ENSG00000150753	validated
tarbase	hsa-miR-181a-5p	DOCK1	1793	ENSG00000150760	validated
tarbase	hsa-miR-21-5p	DOCK1	1793	ENSG00000150760	validated
tarbase	hsa-miR-15b-5p	DIXDC1	85458	ENSG00000150764	validated
tarbase	hsa-miR-192-5p	DLAT	1737	ENSG00000150768	validated
tarbase	hsa-miR-21-5p	DLAT	1737	ENSG00000150768	validated
tarbase	hsa-miR-301a-3p	IL18	3606	ENSG00000150782	validated
tarbase	hsa-miR-181a-5p	PIP4K2A	5305	ENSG00000150867	validated
tarbase	hsa-miR-15b-5p	FREM2	341640	ENSG00000150893	validated
tarbase	hsa-miR-326	FREM2	341640	ENSG00000150893	validated
tarbase	hsa-miR-212-3p	FREM2	341640	ENSG00000150893	validated
tarbase	hsa-miR-582-3p	FOXO1	2308	ENSG00000150907	validated
tarbase	hsa-miR-582-5p	FOXO1	2308	ENSG00000150907	validated
tarbase	hsa-miR-106b-5p	CRIM1	51232	ENSG00000150938	validated
tarbase	hsa-miR-181a-5p	CRIM1	51232	ENSG00000150938	validated
tarbase	hsa-miR-296-5p	ABCB9	23457	ENSG00000150967	validated
tarbase	hsa-miR-326	UBC	7316	ENSG00000150991	validated
tarbase	hsa-miR-301a-3p	SLC7A11	23657	ENSG00000151012	validated
tarbase	hsa-miR-192-5p	NOCT	25819	ENSG00000151014	validated
tarbase	hsa-miR-21-5p	NOCT	25819	ENSG00000151014	validated
tarbase	hsa-miR-21-5p	THRB	7068	ENSG00000151090	validated
tarbase	hsa-miR-106b-5p	UEVLD	55293	ENSG00000151116	validated
tarbase	hsa-miR-301a-3p	UBE3B	89910	ENSG00000151148	validated
tarbase	hsa-miR-15b-5p	ANK3	288	ENSG00000151150	validated
tarbase	hsa-miR-106b-5p	IPMK	253430	ENSG00000151151	validated

tarbase	hsa-miR-582-5p	IPMK	253430	ENSG00000151151	validated
tarbase	hsa-miR-192-5p	RAD9B	144715	ENSG00000151164	validated
tarbase	hsa-miR-212-3p	PLBD2	196463	ENSG00000151176	validated
tarbase	hsa-miR-301a-3p	PLBD2	196463	ENSG00000151176	validated
tarbase	hsa-miR-326	DLG5	9231	ENSG00000151208	validated
tarbase	hsa-miR-106b-5p	GXYLT1	283464	ENSG00000151233	validated
tarbase	hsa-miR-181a-5p	GXYLT1	283464	ENSG00000151233	validated
tarbase	hsa-miR-192-5p	GXYLT1	283464	ENSG00000151233	validated
tarbase	hsa-miR-181a-5p	TWF1	5756	ENSG00000151239	validated
tarbase	hsa-miR-301a-3p	TWF1	5756	ENSG00000151239	validated
tarbase	hsa-miR-106b-5p	DIP2C	22982	ENSG00000151240	validated
tarbase	hsa-miR-181a-5p	DIP2C	22982	ENSG00000151240	validated
tarbase	hsa-miR-106b-5p	EIF4E	1977	ENSG00000151247	validated
tarbase	hsa-miR-106b-5p	TEX30	93081	ENSG00000151287	validated
tarbase	hsa-miR-181a-5p	FAM177A1	283635	ENSG00000151327	validated
tarbase	hsa-miR-15b-5p	MBIP	51562	ENSG00000151332	validated
tarbase	hsa-miR-212-3p	MBIP	51562	ENSG00000151332	validated
tarbase	hsa-miR-15b-5p	MIPOL1	145282	ENSG00000151338	validated
tarbase	hsa-miR-15b-5p	EXT2	2132	ENSG00000151348	validated
tarbase	hsa-miR-582-3p	EXT2	2132	ENSG00000151348	validated
tarbase	hsa-miR-144-3p	NDUFC2	4718	ENSG00000151366	validated
tarbase	hsa-miR-222-3p	NDUFC2	4718	ENSG00000151366	validated
tarbase	hsa-miR-181a-5p	NEK7	140609	ENSG00000151414	validated
tarbase	hsa-miR-18a-5p	NEK7	140609	ENSG00000151414	validated
tarbase	hsa-miR-21-5p	NEK7	140609	ENSG00000151414	validated
tarbase	hsa-miR-222-3p	NEK7	140609	ENSG00000151414	validated
tarbase	hsa-miR-582-3p	NEK7	140609	ENSG00000151414	validated
tarbase	hsa-miR-181a-5p	ANKRD50	57182	ENSG00000151458	validated
tarbase	hsa-miR-21-5p	ANKRD50	57182	ENSG00000151458	validated
tarbase	hsa-miR-18a-5p	ANKRD50	57182	ENSG00000151458	validated
tarbase	hsa-miR-21-5p	CDC123	8872	ENSG00000151465	validated
tarbase	hsa-miR-582-3p	SCLT1	132320	ENSG00000151466	validated
tarbase	hsa-miR-212-3p	SCLT1	132320	ENSG00000151466	validated
tarbase	hsa-miR-106b-5p	FRMD4A	55691	ENSG00000151474	validated
tarbase	hsa-miR-106b-5p	EPS8	2059	ENSG00000151491	validated
tarbase	hsa-miR-181a-5p	QDPR	5860	ENSG00000151552	validated
tarbase	hsa-miR-15b-5p	FAM160B1	57700	ENSG00000151553	validated
tarbase	hsa-miR-181a-5p	FAM160B1	57700	ENSG00000151553	validated
tarbase	hsa-miR-18a-5p	FAM160B1	57700	ENSG00000151553	validated
tarbase	hsa-miR-21-5p	FAM160B1	57700	ENSG00000151553	validated
tarbase	hsa-miR-582-5p	FAM160B1	57700	ENSG00000151553	validated
tarbase	hsa-miR-15b-5p	QTRT2	79691	ENSG00000151576	validated
tarbase	hsa-miR-106b-5p	ZNF827	152485	ENSG00000151612	validated
tarbase	hsa-miR-15b-5p	ZNF827	152485	ENSG00000151612	validated
tarbase	hsa-miR-212-3p	NR3C2	4306	ENSG00000151623	validated
tarbase	hsa-miR-222-3p	AKR1C2	1646	ENSG00000151632	validated
tarbase	hsa-miR-181a-5p	INPP1	3628	ENSG00000151689	validated
tarbase	hsa-miR-181a-5p	MFSD6	54842	ENSG00000151690	validated
tarbase	hsa-miR-106b-5p	ASAP2	8853	ENSG00000151693	validated
tarbase	hsa-miR-301a-3p	ASAP2	8853	ENSG00000151693	validated
tarbase	hsa-miR-222-3p	ADAM17	6868	ENSG00000151694	validated
tarbase	hsa-miR-21-5p	FLI1	2313	ENSG00000151702	validated
tarbase	hsa-miR-582-5p	FLI1	2313	ENSG00000151702	validated
tarbase	hsa-miR-106b-5p	WWC2	80014	ENSG00000151718	validated
tarbase	hsa-miR-181a-5p	WWC2	80014	ENSG00000151718	validated
tarbase	hsa-miR-15b-5p	CENPU	79682	ENSG00000151725	validated
tarbase	hsa-miR-181a-5p	SLC25A4	291	ENSG00000151729	validated
tarbase	hsa-miR-15b-5p	AMN1	196394	ENSG00000151743	validated
tarbase	hsa-miR-212-3p	BICD1	636	ENSG00000151746	validated
tarbase	hsa-miR-181a-5p	SAV1	60485	ENSG00000151748	validated
tarbase	hsa-miR-21-5p	SAV1	60485	ENSG00000151748	validated
tarbase	hsa-miR-212-3p	CCDC122	160857	ENSG00000151773	validated
tarbase	hsa-miR-15b-5p	SACS	26278	ENSG00000151835	validated
tarbase	hsa-miR-181a-5p	SACS	26278	ENSG00000151835	validated
tarbase	hsa-miR-21-5p	PABPC3	5042	ENSG00000151846	validated
tarbase	hsa-miR-222-3p	PABPC3	5042	ENSG00000151846	validated
tarbase	hsa-miR-18a-5p	TMEM267	64417	ENSG00000151881	validated
tarbase	hsa-miR-15b-5p	GFRA1	2674	ENSG00000151892	validated
tarbase	hsa-miR-106b-5p	DST	667	ENSG00000151914	validated
tarbase	hsa-miR-181a-5p	DST	667	ENSG00000151914	validated
tarbase	hsa-miR-222-3p	DST	667	ENSG00000151914	validated

tarbase	hsa-miR-301a-3p	DST	667	ENSG00000151914	validated
tarbase	hsa-miR-505-3p	DST	667	ENSG00000151914	validated
tarbase	hsa-miR-582-3p	DST	667	ENSG00000151914	validated
tarbase	hsa-miR-222-3p	BAG3	9531	ENSG00000151929	validated
tarbase	hsa-miR-181a-5p	AP1S3	130340	ENSG00000152056	validated
tarbase	hsa-miR-144-3p	RABGAP1L	9910	ENSG00000152061	validated
tarbase	hsa-miR-212-3p	RABGAP1L	9910	ENSG00000152061	validated
tarbase	hsa-miR-106b-5p	TLCD4	148534	ENSG00000152078	validated
tarbase	hsa-miR-181a-5p	TLCD4	148534	ENSG00000152078	validated
tarbase	hsa-miR-181a-5p	FAM168B	130074	ENSG00000152102	validated
tarbase	hsa-miR-222-3p	FAM168B	130074	ENSG00000152102	validated
tarbase	hsa-miR-582-3p	PTPN14	5784	ENSG00000152104	validated
tarbase	hsa-miR-326	GEMIN6	79833	ENSG00000152147	validated
tarbase	hsa-miR-106b-5p	OBI1	79596	ENSG00000152193	validated
tarbase	hsa-miR-222-3p	OBI1	79596	ENSG00000152193	validated
tarbase	hsa-miR-301a-3p	OBI1	79596	ENSG00000152193	validated
tarbase	hsa-miR-212-3p	SETBP1	26040	ENSG00000152217	validated
tarbase	hsa-miR-21-5p	ARL14EP	120534	ENSG00000152219	validated
tarbase	hsa-miR-15b-5p	EPG5	57724	ENSG00000152223	validated
tarbase	hsa-miR-301a-3p	EPG5	57724	ENSG00000152223	validated
tarbase	hsa-miR-181a-5p	ATP5F1A	498	ENSG00000152234	validated
tarbase	hsa-miR-301a-3p	C18orf25	147339	ENSG00000152242	validated
tarbase	hsa-miR-106b-5p	SPC25	57405	ENSG00000152253	validated
tarbase	hsa-miR-106b-5p	TCF7L1	83439	ENSG00000152284	validated
tarbase	hsa-miR-181a-5p	TGOLN2	10618	ENSG00000152291	validated
tarbase	hsa-miR-21-5p	TGOLN2	10618	ENSG00000152291	validated
tarbase	hsa-miR-301a-3p	TGOLN2	10618	ENSG00000152291	validated
tarbase	hsa-miR-212-3p	SH2D6	284948	ENSG00000152292	validated
tarbase	hsa-miR-106b-5p	UHMK1	127933	ENSG00000152332	validated
tarbase	hsa-miR-144-3p	UHMK1	127933	ENSG00000152332	validated
tarbase	hsa-miR-15b-5p	UHMK1	127933	ENSG00000152332	validated
tarbase	hsa-miR-181a-5p	UHMK1	127933	ENSG00000152332	validated
tarbase	hsa-miR-18a-5p	UHMK1	127933	ENSG00000152332	validated
tarbase	hsa-miR-21-5p	UHMK1	127933	ENSG00000152332	validated
tarbase	hsa-miR-301a-3p	UHMK1	127933	ENSG00000152332	validated
tarbase	hsa-miR-582-5p	UHMK1	127933	ENSG00000152332	validated
tarbase	hsa-miR-181a-5p	SPOCK1	6695	ENSG00000152377	validated
tarbase	hsa-miR-582-3p	TADA1	117143	ENSG00000152382	validated
tarbase	hsa-miR-15b-5p	GUCY1A2	2977	ENSG00000152402	validated
tarbase	hsa-miR-181a-5p	GUCY1A2	2977	ENSG00000152402	validated
tarbase	hsa-miR-106b-5p	JMY	133746	ENSG00000152409	validated
tarbase	hsa-miR-212-3p	ZNF547	284306	ENSG00000152433	validated
tarbase	hsa-miR-181a-5p	ZNF776	284309	ENSG00000152443	validated
tarbase	hsa-miR-18a-5p	ZNF776	284309	ENSG00000152443	validated
tarbase	hsa-miR-212-3p	ZSCAN1	284312	ENSG00000152467	validated
tarbase	hsa-miR-106b-5p	CCDC50	152137	ENSG00000152492	validated
tarbase	hsa-miR-181a-5p	CCDC50	152137	ENSG00000152492	validated
tarbase	hsa-miR-181a-5p	CAMK4	814	ENSG00000152495	validated
tarbase	hsa-miR-582-3p	CAMK4	814	ENSG00000152495	validated
tarbase	hsa-miR-15b-5p	TRIM36	55521	ENSG00000152503	validated
tarbase	hsa-miR-222-3p	ZFP36L2	678	ENSG00000152518	validated
tarbase	hsa-miR-212-3p	PAN3	255967	ENSG00000152520	validated
tarbase	hsa-miR-301a-3p	PAN3	255967	ENSG00000152520	validated
tarbase	hsa-miR-15b-5p	TMEM123	114908	ENSG00000152558	validated
tarbase	hsa-miR-181a-5p	TMEM123	114908	ENSG00000152558	validated
tarbase	hsa-miR-106b-5p	MBNL1	4154	ENSG00000152601	validated
tarbase	hsa-miR-181a-5p	MBNL1	4154	ENSG00000152601	validated
tarbase	hsa-miR-18a-5p	MBNL1	4154	ENSG00000152601	validated
tarbase	hsa-miR-212-3p	MBNL1	4154	ENSG00000152601	validated
tarbase	hsa-miR-222-3p	MBNL1	4154	ENSG00000152601	validated
tarbase	hsa-miR-15b-5p	MBNL1	4154	ENSG00000152601	validated
tarbase	hsa-miR-21-5p	NADK2	133686	ENSG00000152620	validated
tarbase	hsa-miR-181a-5p	SLC30A6	55676	ENSG00000152683	validated
tarbase	hsa-miR-582-3p	SLC30A6	55676	ENSG00000152683	validated
tarbase	hsa-miR-106b-5p	PELO	53918	ENSG00000152684	validated
tarbase	hsa-miR-106b-5p	SAR1B	51128	ENSG00000152700	validated
tarbase	hsa-miR-15b-5p	SAR1B	51128	ENSG00000152700	validated
tarbase	hsa-miR-301a-3p	SAR1B	51128	ENSG00000152700	validated
tarbase	hsa-miR-212-3p	CATSPER3	347732	ENSG00000152705	validated
tarbase	hsa-miR-212-3p	WDR78	79819	ENSG00000152763	validated
tarbase	hsa-miR-212-3p	IFIT5	24138	ENSG00000152778	validated

tarbase	hsa-miR-181a-5p	PANK1	53354	ENSG00000152782	validated
tarbase	hsa-miR-212-3p	PANK1	53354	ENSG00000152782	validated
tarbase	hsa-miR-181a-5p	HNRNPDL	9987	ENSG00000152795	validated
tarbase	hsa-miR-582-5p	HNRNPDL	9987	ENSG00000152795	validated
tarbase	hsa-miR-212-3p	GRM1	2911	ENSG00000152822	validated
tarbase	hsa-miR-181a-5p	GGPS1	9453	ENSG00000152904	validated
tarbase	hsa-miR-15b-5p	CNTNAP4	85445	ENSG00000152910	validated
tarbase	hsa-miR-181a-5p	RAB3C	115827	ENSG00000152932	validated
tarbase	hsa-miR-21-5p	MED21	9412	ENSG00000152944	validated
tarbase	hsa-miR-301a-3p	MED21	9412	ENSG00000152944	validated
tarbase	hsa-miR-582-3p	MED21	9412	ENSG00000152944	validated
tarbase	hsa-miR-301a-3p	PLOD2	5352	ENSG00000152952	validated
tarbase	hsa-miR-505-3p	PLOD2	5352	ENSG00000152952	validated
tarbase	hsa-miR-21-5p	SREK1IP1	285672	ENSG00000153006	validated
tarbase	hsa-miR-212-3p	BCL2L11	10018	ENSG00000153094	validated
tarbase	hsa-miR-582-5p	BCL2L11	10018	ENSG00000153094	validated
tarbase	hsa-miR-106b-5p	CETN3	1070	ENSG00000153140	validated
tarbase	hsa-miR-15b-5p	SMARCA5	8467	ENSG00000153147	validated
tarbase	hsa-miR-18a-5p	SMARCA5	8467	ENSG00000153147	validated
tarbase	hsa-miR-222-3p	SMARCA5	8467	ENSG00000153147	validated
tarbase	hsa-miR-296-5p	SMARCA5	8467	ENSG00000153147	validated
tarbase	hsa-miR-106b-5p	SMARCA5	8467	ENSG00000153147	validated
tarbase	hsa-miR-192-5p	BMP6	654	ENSG00000153162	validated
tarbase	hsa-miR-301a-3p	BMP6	654	ENSG00000153162	validated
tarbase	hsa-miR-106b-5p	RASSF3	283349	ENSG00000153179	validated
tarbase	hsa-miR-222-3p	RASSF3	283349	ENSG00000153179	validated
tarbase	hsa-miR-582-5p	RASSF3	283349	ENSG00000153179	validated
tarbase	hsa-miR-106b-5p	HNRNPU	3192	ENSG00000153187	validated
tarbase	hsa-miR-15b-5p	HNRNPU	3192	ENSG00000153187	validated
tarbase	hsa-miR-181a-5p	HNRNPU	3192	ENSG00000153187	validated
tarbase	hsa-miR-21-5p	HNRNPU	3192	ENSG00000153187	validated
tarbase	hsa-miR-222-3p	HNRNPU	3192	ENSG00000153187	validated
tarbase	hsa-miR-296-5p	HNRNPU	3192	ENSG00000153187	validated
tarbase	hsa-miR-326	HNRNPU	3192	ENSG00000153187	validated
tarbase	hsa-miR-106b-5p	RANBP2	5903	ENSG00000153201	validated
tarbase	hsa-miR-15b-5p	RANBP2	5903	ENSG00000153201	validated
tarbase	hsa-miR-181a-5p	RANBP2	5903	ENSG00000153201	validated
tarbase	hsa-miR-582-5p	RANBP2	5903	ENSG00000153201	validated
tarbase	hsa-miR-106b-5p	AHCTF1	25909	ENSG00000153207	validated
tarbase	hsa-miR-15b-5p	AHCTF1	25909	ENSG00000153207	validated
tarbase	hsa-miR-181a-5p	AHCTF1	25909	ENSG00000153207	validated
tarbase	hsa-miR-181a-5p	MERTK	10461	ENSG00000153208	validated
tarbase	hsa-miR-15b-5p	TMEM87B	84910	ENSG00000153214	validated
tarbase	hsa-miR-181a-5p	TMEM87B	84910	ENSG00000153214	validated
tarbase	hsa-miR-18a-5p	TMEM87B	84910	ENSG00000153214	validated
tarbase	hsa-miR-212-3p	TMEM87B	84910	ENSG00000153214	validated
tarbase	hsa-miR-181a-5p	NR4A2	4929	ENSG00000153234	validated
tarbase	hsa-miR-212-3p	NR4A2	4929	ENSG00000153234	validated
tarbase	hsa-miR-212-3p	CCDC148	130940	ENSG00000153237	validated
tarbase	hsa-miR-144-3p	PLA2R1	22925	ENSG00000153246	validated
tarbase	hsa-miR-106b-5p	RBMS1	5937	ENSG00000153250	validated
tarbase	hsa-miR-15b-5p	RBMS1	5937	ENSG00000153250	validated
tarbase	hsa-miR-181a-5p	RBMS1	5937	ENSG00000153250	validated
tarbase	hsa-miR-21-5p	RBMS1	5937	ENSG00000153250	validated
tarbase	hsa-miR-222-3p	RBMS1	5937	ENSG00000153250	validated
tarbase	hsa-miR-181a-5p	CD96	10225	ENSG00000153283	validated
tarbase	hsa-miR-181a-5p	SLC25A27	9481	ENSG00000153291	validated
tarbase	hsa-miR-181a-5p	FAM49B	51571	ENSG00000153310	validated
tarbase	hsa-miR-192-5p	FAM49B	51571	ENSG00000153310	validated
tarbase	hsa-miR-15b-5p	ASAP1	50807	ENSG00000153317	validated
tarbase	hsa-miR-301a-3p	ASAP1	50807	ENSG00000153317	validated
tarbase	hsa-miR-222-3p	LPCAT1	79888	ENSG00000153395	validated
tarbase	hsa-miR-582-5p	LPCAT1	79888	ENSG00000153395	validated
tarbase	hsa-miR-15b-5p	PLEKHG4B	153478	ENSG00000153404	validated
tarbase	hsa-miR-212-3p	CMTM7	112616	ENSG00000153551	validated
tarbase	hsa-miR-181a-5p	UBP1	7342	ENSG00000153560	validated
tarbase	hsa-miR-582-3p	UBP1	7342	ENSG00000153560	validated
tarbase	hsa-miR-15b-5p	RMND5A	64795	ENSG00000153561	validated
tarbase	hsa-miR-181a-5p	RMND5A	64795	ENSG00000153561	validated
tarbase	hsa-miR-18a-5p	RMND5A	64795	ENSG00000153561	validated
tarbase	hsa-miR-326	RMND5A	64795	ENSG00000153561	validated

tarbase	hsa-miR-505-3p	RMND5A	64795	ENSG00000153561	validated
tarbase	hsa-miR-582-3p	RMND5A	64795	ENSG00000153561	validated
tarbase	hsa-miR-106b-5p	RPIA	22934	ENSG00000153574	validated
tarbase	hsa-miR-505-3p	RPIA	22934	ENSG00000153574	validated
tarbase	hsa-miR-106b-5p	PTPRD	5789	ENSG00000153707	validated
tarbase	hsa-miR-301a-3p	PTPRD	5789	ENSG00000153707	validated
tarbase	hsa-miR-21-5p	LURAP1L	286343	ENSG00000153714	validated
tarbase	hsa-miR-212-3p	CNKSR3	154043	ENSG00000153721	validated
tarbase	hsa-miR-301a-3p	CNKSR3	154043	ENSG00000153721	validated
tarbase	hsa-miR-181a-5p	ZDHHC7	55625	ENSG00000153786	validated
tarbase	hsa-miR-212-3p	ZDHHC7	55625	ENSG00000153786	validated
tarbase	hsa-miR-21-5p	ZDHHC7	55625	ENSG00000153786	validated
tarbase	hsa-miR-582-5p	C7orf31	136895	ENSG00000153790	validated
tarbase	hsa-miR-106b-5p	JAZF1	221895	ENSG00000153814	validated
tarbase	hsa-miR-181a-5p	JAZF1	221895	ENSG00000153814	validated
tarbase	hsa-miR-505-3p	CMIP	80790	ENSG00000153815	validated
tarbase	hsa-miR-582-3p	CMIP	80790	ENSG00000153815	validated
tarbase	hsa-miR-582-5p	CMIP	80790	ENSG00000153815	validated
tarbase	hsa-miR-18a-5p	CMIP	80790	ENSG00000153815	validated
tarbase	hsa-miR-181a-5p	TRIP12	9320	ENSG00000153827	validated
tarbase	hsa-miR-21-5p	TRIP12	9320	ENSG00000153827	validated
tarbase	hsa-miR-181a-5p	CEBPG	1054	ENSG00000153879	validated
tarbase	hsa-miR-301a-3p	CEBPG	1054	ENSG00000153879	validated
tarbase	hsa-miR-505-3p	CEBPG	1054	ENSG00000153879	validated
tarbase	hsa-miR-181a-5p	DDAH1	23576	ENSG00000153904	validated
tarbase	hsa-miR-106b-5p	SREK1	140890	ENSG00000153914	validated
tarbase	hsa-miR-181a-5p	CHD1	1105	ENSG00000153922	validated
tarbase	hsa-miR-582-5p	CHD1	1105	ENSG00000153922	validated
tarbase	hsa-miR-326	DGKE	8526	ENSG00000153933	validated
tarbase	hsa-miR-582-5p	HS2ST1	9653	ENSG00000153936	validated
tarbase	hsa-miR-106b-5p	MSI2	124540	ENSG00000153944	validated
tarbase	hsa-miR-15b-5p	MSI2	124540	ENSG00000153944	validated
tarbase	hsa-miR-181a-5p	MSI2	124540	ENSG00000153944	validated
tarbase	hsa-miR-21-5p	MSI2	124540	ENSG00000153944	validated
tarbase	hsa-miR-15b-5p	CACNA2D1	781	ENSG00000153956	validated
tarbase	hsa-miR-106b-5p	ANKRD29	147463	ENSG00000154065	validated
tarbase	hsa-miR-582-5p	ANKRD29	147463	ENSG00000154065	validated
tarbase	hsa-miR-296-5p	THY1	7070	ENSG00000154096	validated
tarbase	hsa-miR-106b-5p	TBCEL	219899	ENSG00000154114	validated
tarbase	hsa-miR-21-5p	TBCEL	219899	ENSG00000154114	validated
tarbase	hsa-miR-15b-5p	TBCEL	219899	ENSG00000154114	validated
tarbase	hsa-miR-181a-5p	TBCEL	219899	ENSG00000154114	validated
tarbase	hsa-miR-15b-5p	ANKH	56172	ENSG00000154122	validated
tarbase	hsa-miR-106b-5p	OTULIN	90268	ENSG00000154124	validated
tarbase	hsa-miR-582-5p	OTULIN	90268	ENSG00000154124	validated
tarbase	hsa-miR-296-5p	UBASH3B	84959	ENSG00000154127	validated
tarbase	hsa-miR-582-3p	RETREG1	54463	ENSG00000154153	validated
tarbase	hsa-miR-212-3p	CDH12	1010	ENSG00000154162	validated
tarbase	hsa-miR-181a-5p	TOMM70	9868	ENSG00000154174	validated
tarbase	hsa-miR-18a-5p	TOMM70	9868	ENSG00000154174	validated
tarbase	hsa-miR-21-5p	TOMM70	9868	ENSG00000154174	validated
tarbase	hsa-miR-181a-5p	ABI3BP	25890	ENSG00000154175	validated
tarbase	hsa-miR-212-3p	ANGPT1	284	ENSG00000154188	validated
tarbase	hsa-miR-15b-5p	CC2D1B	200014	ENSG00000154222	validated
tarbase	hsa-miR-212-3p	CC2D1B	200014	ENSG00000154222	validated
tarbase	hsa-miR-301a-3p	CC2D1B	200014	ENSG00000154222	validated
tarbase	hsa-miR-326	CC2D1B	200014	ENSG00000154222	validated
tarbase	hsa-miR-582-3p	PRKCA	5578	ENSG00000154229	validated
tarbase	hsa-miR-15b-5p	LRRK1	79705	ENSG00000154237	validated
tarbase	hsa-miR-144-3p	ABCA5	23461	ENSG00000154265	validated
tarbase	hsa-miR-15b-5p	MIA3	375056	ENSG00000154305	validated
tarbase	hsa-miR-18a-5p	MIA3	375056	ENSG00000154305	validated
tarbase	hsa-miR-21-5p	MIA3	375056	ENSG00000154305	validated
tarbase	hsa-miR-222-3p	MIA3	375056	ENSG00000154305	validated
tarbase	hsa-miR-582-3p	DISP1	84976	ENSG00000154309	validated
tarbase	hsa-miR-106b-5p	FAM167A	83648	ENSG00000154319	validated
tarbase	hsa-miR-212-3p	FAM167A	83648	ENSG00000154319	validated
tarbase	hsa-miR-296-5p	FAM167A	83648	ENSG00000154319	validated
tarbase	hsa-miR-15b-5p	WNT3A	89780	ENSG00000154342	validated
tarbase	hsa-miR-192-5p	OBSCN	84033	ENSG00000154358	validated
tarbase	hsa-miR-301a-3p	TRIM11	81559	ENSG00000154370	validated

tarbase	hsa-miR-326	TRIM11	81559	ENSG00000154370	validated
tarbase	hsa-miR-15b-5p	ENAH	55740	ENSG00000154380	validated
tarbase	hsa-miR-106b-5p	CCSAP	126731	ENSG00000154429	validated
tarbase	hsa-miR-181a-5p	CCSAP	126731	ENSG00000154429	validated
tarbase	hsa-miR-106b-5p	SH3RF1	57630	ENSG00000154447	validated
tarbase	hsa-miR-15b-5p	BUB3	9184	ENSG00000154473	validated
tarbase	hsa-miR-181a-5p	BUB3	9184	ENSG00000154473	validated
tarbase	hsa-miR-582-3p	BUB3	9184	ENSG00000154473	validated
tarbase	hsa-miR-222-3p	BUB3	9184	ENSG00000154473	validated
tarbase	hsa-miR-212-3p	CCDC173	129881	ENSG00000154479	validated
tarbase	hsa-miR-18a-5p	DIPK1A	388650	ENSG00000154511	validated
tarbase	hsa-miR-582-5p	DIPK1A	388650	ENSG00000154511	validated
tarbase	hsa-miR-21-5p	ATP5MC3	518	ENSG00000154518	validated
tarbase	hsa-miR-582-5p	ATP5MC3	518	ENSG00000154518	validated
tarbase	hsa-miR-181a-5p	ELOC	6921	ENSG00000154582	validated
tarbase	hsa-miR-301a-3p	ELOC	6921	ENSG00000154582	validated
tarbase	hsa-miR-181a-5p	BTG3	10950	ENSG00000154640	validated
tarbase	hsa-miR-18a-5p	BTG3	10950	ENSG00000154640	validated
tarbase	hsa-miR-18a-5p	ATP5PF	522	ENSG00000154723	validated
tarbase	hsa-miR-21-5p	GABPA	2551	ENSG00000154727	validated
tarbase	hsa-miR-106b-5p	ADAMTS1	9510	ENSG00000154734	validated
tarbase	hsa-miR-181a-5p	ADAMTS1	9510	ENSG00000154734	validated
tarbase	hsa-miR-212-3p	ADAMTS1	9510	ENSG00000154734	validated
tarbase	hsa-miR-582-3p	ADAMTS1	9510	ENSG00000154734	validated
tarbase	hsa-miR-106b-5p	ADAMTS5	11096	ENSG00000154736	validated
tarbase	hsa-miR-15b-5p	ADAMTS5	11096	ENSG00000154736	validated
tarbase	hsa-miR-181a-5p	ADAMTS5	11096	ENSG00000154736	validated
tarbase	hsa-miR-21-5p	TSEN2	80746	ENSG00000154743	validated
tarbase	hsa-miR-18a-5p	OXNAD1	92106	ENSG00000154814	validated
tarbase	hsa-miR-301a-3p	PLCL2	23228	ENSG00000154822	validated
tarbase	hsa-miR-181a-5p	RAB6B	51560	ENSG00000154917	validated
tarbase	hsa-miR-15b-5p	ANKRD40	91369	ENSG00000154945	validated
tarbase	hsa-miR-192-5p	ANKRD40	91369	ENSG00000154945	validated
tarbase	hsa-miR-212-3p	ANKRD40	91369	ENSG00000154945	validated
tarbase	hsa-miR-106b-5p	ZNF18	7566	ENSG00000154957	validated
tarbase	hsa-miR-106b-5p	APOOL	139322	ENSG00000155008	validated
tarbase	hsa-miR-582-3p	APOOL	139322	ENSG00000155008	validated
tarbase	hsa-miR-106b-5p	CYP2U1	113612	ENSG00000155016	validated
tarbase	hsa-miR-212-3p	AK9	221264	ENSG00000155085	validated
tarbase	hsa-miR-144-3p	KLF10	7071	ENSG00000155090	validated
tarbase	hsa-miR-18a-5p	KLF10	7071	ENSG00000155090	validated
tarbase	hsa-miR-296-5p	KLF10	7071	ENSG00000155090	validated
tarbase	hsa-miR-582-5p	KLF10	7071	ENSG00000155090	validated
tarbase	hsa-miR-15b-5p	PTPRN2	5799	ENSG00000155093	validated
tarbase	hsa-miR-15b-5p	AZIN1	51582	ENSG00000155096	validated
tarbase	hsa-miR-181a-5p	AZIN1	51582	ENSG00000155096	validated
tarbase	hsa-miR-212-3p	AZIN1	51582	ENSG00000155096	validated
tarbase	hsa-miR-21-5p	AZIN1	51582	ENSG00000155096	validated
tarbase	hsa-miR-181a-5p	ATP6V1C1	528	ENSG00000155097	validated
tarbase	hsa-miR-18a-5p	ATP6V1C1	528	ENSG00000155097	validated
tarbase	hsa-miR-144-3p	PIP4P2	55529	ENSG00000155099	validated
tarbase	hsa-miR-15b-5p	PIP4P2	55529	ENSG00000155099	validated
tarbase	hsa-miR-301a-3p	PIP4P2	55529	ENSG00000155099	validated
tarbase	hsa-miR-582-5p	PIP4P2	55529	ENSG00000155099	validated
tarbase	hsa-miR-181a-5p	OTUD6B	51633	ENSG00000155100	validated
tarbase	hsa-miR-18a-5p	OTUD6B	51633	ENSG00000155100	validated
tarbase	hsa-miR-21-5p	OTUD6B	51633	ENSG00000155100	validated
tarbase	hsa-miR-15b-5p	TTC39B	158219	ENSG00000155158	validated
tarbase	hsa-miR-144-3p	AGPAT5	55326	ENSG00000155189	validated
tarbase	hsa-miR-21-5p	AGPAT5	55326	ENSG00000155189	validated
tarbase	hsa-miR-15b-5p	MMS19	64210	ENSG00000155229	validated
tarbase	hsa-miR-18a-5p	PI4K2A	55361	ENSG00000155252	validated
tarbase	hsa-miR-582-3p	ZFYVE27	118813	ENSG00000155256	validated
tarbase	hsa-miR-106b-5p	SLC25A28	81894	ENSG00000155287	validated
tarbase	hsa-miR-326	SLC25A28	81894	ENSG00000155287	validated
tarbase	hsa-miR-18a-5p	HSPA13	6782	ENSG00000155304	validated
tarbase	hsa-miR-106b-5p	HSPA13	6782	ENSG00000155304	validated
tarbase	hsa-miR-15b-5p	USP25	29761	ENSG00000155313	validated
tarbase	hsa-miR-106b-5p	USP25	29761	ENSG00000155313	validated
tarbase	hsa-miR-505-3p	GRAMD2B	65983	ENSG00000155324	validated
tarbase	hsa-miR-222-3p	ZCCHC10	54819	ENSG00000155329	validated

tarbase	hsa-miR-106b-5p	C16orf87	388272	ENSG00000155330	validated
tarbase	hsa-miR-181a-5p	C16orf87	388272	ENSG00000155330	validated
tarbase	hsa-miR-18a-5p	MOV10	4343	ENSG00000155363	validated
tarbase	hsa-miR-212-3p	MOV10	4343	ENSG00000155363	validated
tarbase	hsa-miR-296-5p	RHOC	389	ENSG00000155366	validated
tarbase	hsa-miR-18a-5p	SLC16A1	6566	ENSG00000155380	validated
tarbase	hsa-miR-21-5p	SLC16A1	6566	ENSG00000155380	validated
tarbase	hsa-miR-505-3p	SLC16A1	6566	ENSG00000155380	validated
tarbase	hsa-miR-582-3p	SLC16A1	6566	ENSG00000155380	validated
tarbase	hsa-miR-582-5p	SLC16A1	6566	ENSG00000155380	validated
tarbase	hsa-miR-106b-5p	SLC16A1	6566	ENSG00000155380	validated
tarbase	hsa-miR-15b-5p	SLC16A1	6566	ENSG00000155380	validated
tarbase	hsa-miR-505-3p	NIFK	84365	ENSG00000155438	validated
tarbase	hsa-miR-106b-5p	OXA1L	5018	ENSG00000155463	validated
tarbase	hsa-miR-301a-3p	OXA1L	5018	ENSG00000155463	validated
tarbase	hsa-miR-106b-5p	SLC7A7	9056	ENSG00000155465	validated
tarbase	hsa-miR-106b-5p	LARP1	23367	ENSG00000155506	validated
tarbase	hsa-miR-15b-5p	LARP1	23367	ENSG00000155506	validated
tarbase	hsa-miR-18a-5p	LARP1	23367	ENSG00000155506	validated
tarbase	hsa-miR-21-5p	LARP1	23367	ENSG00000155506	validated
tarbase	hsa-miR-296-5p	LARP1	23367	ENSG00000155506	validated
tarbase	hsa-miR-326	LARP1	23367	ENSG00000155506	validated
tarbase	hsa-miR-15b-5p	CNOT8	9337	ENSG00000155508	validated
tarbase	hsa-miR-301a-3p	CNOT8	9337	ENSG00000155508	validated
tarbase	hsa-miR-106b-5p	MIER3	166968	ENSG00000155545	validated
tarbase	hsa-miR-181a-5p	MIER3	166968	ENSG00000155545	validated
tarbase	hsa-miR-222-3p	MIER3	166968	ENSG00000155545	validated
tarbase	hsa-miR-301a-3p	MIER3	166968	ENSG00000155545	validated
tarbase	hsa-miR-582-3p	MIER3	166968	ENSG00000155545	validated
tarbase	hsa-miR-106b-5p	NUP205	23165	ENSG00000155561	validated
tarbase	hsa-miR-21-5p	NUP205	23165	ENSG00000155561	validated
tarbase	hsa-miR-582-5p	NUP205	23165	ENSG00000155561	validated
tarbase	hsa-miR-15b-5p	ZKSCAN2	342357	ENSG00000155592	validated
tarbase	hsa-miR-181a-5p	TTN	7273	ENSG00000155657	validated
tarbase	hsa-miR-582-5p	TTN	7273	ENSG00000155657	validated
tarbase	hsa-miR-212-3p	VSIG4	11326	ENSG00000155659	validated
tarbase	hsa-miR-15b-5p	PDIA4	9601	ENSG00000155660	validated
tarbase	hsa-miR-15b-5p	FAM126B	285172	ENSG00000155744	validated
tarbase	hsa-miR-181a-5p	FAM126B	285172	ENSG00000155744	validated
tarbase	hsa-miR-212-3p	FLACC1	130540	ENSG00000155749	validated
tarbase	hsa-miR-106b-5p	FMN2	56776	ENSG00000155816	validated
tarbase	hsa-miR-144-3p	FMN2	56776	ENSG00000155816	validated
tarbase	hsa-miR-582-5p	FMN2	56776	ENSG00000155816	validated
tarbase	hsa-miR-212-3p	FMN2	56776	ENSG00000155816	validated
tarbase	hsa-miR-181a-5p	RNF20	56254	ENSG00000155827	validated
tarbase	hsa-miR-144-3p	PPARGC1B	133522	ENSG00000155846	validated
tarbase	hsa-miR-301a-3p	PPARGC1B	133522	ENSG00000155846	validated
tarbase	hsa-miR-106b-5p	MED7	9443	ENSG00000155868	validated
tarbase	hsa-miR-15b-5p	RRAGA	10670	ENSG00000155876	validated
tarbase	hsa-miR-106b-5p	SLC24A2	25769	ENSG00000155886	validated
tarbase	hsa-miR-15b-5p	SLC24A2	25769	ENSG00000155886	validated
tarbase	hsa-miR-106b-5p	PXYLP1	92370	ENSG00000155893	validated
tarbase	hsa-miR-15b-5p	PXYLP1	92370	ENSG00000155893	validated
tarbase	hsa-miR-106b-5p	RASA2	5922	ENSG00000155903	validated
tarbase	hsa-miR-18a-5p	RASA2	5922	ENSG00000155903	validated
tarbase	hsa-miR-181a-5p	VBP1	7411	ENSG00000155959	validated
tarbase	hsa-miR-21-5p	CLIC2	1193	ENSG00000155962	validated
tarbase	hsa-miR-181a-5p	MICU3	286097	ENSG00000155970	validated
tarbase	hsa-miR-212-3p	MICU3	286097	ENSG00000155970	validated
tarbase	hsa-miR-106b-5p	KIF5A	3798	ENSG00000155980	validated
tarbase	hsa-miR-15b-5p	KIF5A	3798	ENSG00000155980	validated
tarbase	hsa-miR-18a-5p	KIF5A	3798	ENSG00000155980	validated
tarbase	hsa-miR-326	KIF5A	3798	ENSG00000155980	validated
tarbase	hsa-miR-106b-5p	PSD3	23362	ENSG00000156011	validated
tarbase	hsa-miR-181a-5p	PSD3	23362	ENSG00000156011	validated
tarbase	hsa-miR-15b-5p	PSD3	23362	ENSG00000156011	validated
tarbase	hsa-miR-212-3p	CARNMT1	138199	ENSG00000156017	validated
tarbase	hsa-miR-15b-5p	ELMSAN1	91748	ENSG00000156030	validated
tarbase	hsa-miR-21-5p	ELMSAN1	91748	ENSG00000156030	validated
tarbase	hsa-miR-296-5p	ELMSAN1	91748	ENSG00000156030	validated
tarbase	hsa-miR-15b-5p	GNAQ	2776	ENSG00000156052	validated

tarbase	hsa-miR-181a-5p	GNAQ	2776	ENSG00000156052	validated
tarbase	hsa-miR-181a-5p	ADK	132	ENSG00000156110	validated
tarbase	hsa-miR-21-5p	DCK	1633	ENSG00000156136	validated
tarbase	hsa-miR-18a-5p	NDUFAF6	137682	ENSG00000156170	validated
tarbase	hsa-miR-212-3p	C8orf37	157657	ENSG00000156172	validated
tarbase	hsa-miR-212-3p	N6AMT1	29104	ENSG00000156239	validated
tarbase	hsa-miR-21-5p	CCT8	10694	ENSG00000156261	validated
tarbase	hsa-miR-18a-5p	NAA11	84779	ENSG00000156269	validated
tarbase	hsa-miR-106b-5p	BACH1	571	ENSG00000156273	validated
tarbase	hsa-miR-15b-5p	BACH1	571	ENSG00000156273	validated
tarbase	hsa-miR-181a-5p	BACH1	571	ENSG00000156273	validated
tarbase	hsa-miR-18a-5p	BACH1	571	ENSG00000156273	validated
tarbase	hsa-miR-21-5p	BACH1	571	ENSG00000156273	validated
tarbase	hsa-miR-582-3p	BACH1	571	ENSG00000156273	validated
tarbase	hsa-miR-582-5p	TIAM1	7074	ENSG00000156299	validated
tarbase	hsa-miR-15b-5p	SCAF4	57466	ENSG00000156304	validated
tarbase	hsa-miR-106b-5p	ANKRD9	122416	ENSG00000156381	validated
tarbase	hsa-miR-106b-5p	SFXN2	118980	ENSG00000156398	validated
tarbase	hsa-miR-15b-5p	ATP5MPL	9556	ENSG00000156411	validated
tarbase	hsa-miR-192-5p	PCDH1	5097	ENSG00000156453	validated
tarbase	hsa-miR-212-3p	PCDH1	5097	ENSG00000156453	validated
tarbase	hsa-miR-106b-5p	PTDSS1	9791	ENSG00000156471	validated
tarbase	hsa-miR-18a-5p	RPL30	6156	ENSG00000156482	validated
tarbase	hsa-miR-106b-5p	FAM122B	159090	ENSG00000156504	validated
tarbase	hsa-miR-181a-5p	FAM122B	159090	ENSG00000156504	validated
tarbase	hsa-miR-21-5p	EEF1A1	1915	ENSG00000156508	validated
tarbase	hsa-miR-326	FBXO43	286151	ENSG00000156509	validated
tarbase	hsa-miR-212-3p	FBXO43	286151	ENSG00000156509	validated
tarbase	hsa-miR-15b-5p	HK1	3098	ENSG00000156515	validated
tarbase	hsa-miR-181a-5p	HK1	3098	ENSG00000156515	validated
tarbase	hsa-miR-21-5p	PHF6	84295	ENSG00000156531	validated
tarbase	hsa-miR-301a-3p	PHF6	84295	ENSG00000156531	validated
tarbase	hsa-miR-106b-5p	CD109	135228	ENSG00000156535	validated
tarbase	hsa-miR-212-3p	UBE2L6	9246	ENSG00000156587	validated
tarbase	hsa-miR-212-3p	ZDHHC5	25921	ENSG00000156599	validated
tarbase	hsa-miR-181a-5p	ZFAND3	60685	ENSG00000156639	validated
tarbase	hsa-miR-15b-5p	NPTN	27020	ENSG00000156642	validated
tarbase	hsa-miR-181a-5p	NPTN	27020	ENSG00000156642	validated
tarbase	hsa-miR-18a-5p	NPTN	27020	ENSG00000156642	validated
tarbase	hsa-miR-21-5p	NPTN	27020	ENSG00000156642	validated
tarbase	hsa-miR-296-5p	NPTN	27020	ENSG00000156642	validated
tarbase	hsa-miR-301a-3p	NPTN	27020	ENSG00000156642	validated
tarbase	hsa-miR-106b-5p	NPTN	27020	ENSG00000156642	validated
tarbase	hsa-miR-15b-5p	SAMD8	142891	ENSG00000156671	validated
tarbase	hsa-miR-181a-5p	SAMD8	142891	ENSG00000156671	validated
tarbase	hsa-miR-21-5p	SAMD8	142891	ENSG00000156671	validated
tarbase	hsa-miR-15b-5p	RAB11FIP1	80223	ENSG00000156675	validated
tarbase	hsa-miR-582-3p	RAB11FIP1	80223	ENSG00000156675	validated
tarbase	hsa-miR-181a-5p	UNC5D	137970	ENSG00000156687	validated
tarbase	hsa-miR-181a-5p	UTP14A	10813	ENSG00000156697	validated
tarbase	hsa-miR-21-5p	BAG4	9530	ENSG00000156735	validated
tarbase	hsa-miR-212-3p	FBRS	64319	ENSG00000156860	validated
tarbase	hsa-miR-582-3p	FBRS	64319	ENSG00000156860	validated
tarbase	hsa-miR-106b-5p	MFSD14A	64645	ENSG00000156875	validated
tarbase	hsa-miR-15b-5p	MFSD14A	64645	ENSG00000156875	validated
tarbase	hsa-miR-21-5p	MFSD14A	64645	ENSG00000156875	validated
tarbase	hsa-miR-301a-3p	MFSD14A	64645	ENSG00000156875	validated
tarbase	hsa-miR-582-3p	MFSD14A	64645	ENSG00000156875	validated
tarbase	hsa-miR-18a-5p	MFSD14A	64645	ENSG00000156875	validated
tarbase	hsa-miR-222-3p	BUB1B	701	ENSG00000156970	validated
tarbase	hsa-miR-15b-5p	EIF4A2	1974	ENSG00000156976	validated
tarbase	hsa-miR-181a-5p	EIF4A2	1974	ENSG00000156976	validated
tarbase	hsa-miR-212-3p	EIF4A2	1974	ENSG00000156976	validated
tarbase	hsa-miR-582-5p	SST	6750	ENSG00000157005	validated
tarbase	hsa-miR-15b-5p	TATDN2	9797	ENSG00000157014	validated
tarbase	hsa-miR-18a-5p	TATDN2	9797	ENSG00000157014	validated
tarbase	hsa-miR-106b-5p	ATP2B2	491	ENSG00000157087	validated
tarbase	hsa-miR-21-5p	SMG1	23049	ENSG00000157106	validated
tarbase	hsa-miR-301a-3p	SMG1	23049	ENSG00000157106	validated
tarbase	hsa-miR-582-3p	SMG1	23049	ENSG00000157106	validated
tarbase	hsa-miR-212-3p	TIMP4	7079	ENSG00000157150	validated

tarbase	hsa-miR-106b-5p	ODR4	54953	ENSG00000157181	validated
tarbase	hsa-miR-18a-5p	CPT2	1376	ENSG00000157184	validated
tarbase	hsa-miR-301a-3p	LRP8	7804	ENSG00000157193	validated
tarbase	hsa-miR-144-3p	STEAP2	261729	ENSG00000157214	validated
tarbase	hsa-miR-212-3p	ARSL	415	ENSG00000157399	validated
tarbase	hsa-miR-18a-5p	KIT	3815	ENSG00000157404	validated
tarbase	hsa-miR-181a-5p	KIT	3815	ENSG00000157404	validated
tarbase	hsa-miR-106b-5p	ZNF19	7567	ENSG00000157429	validated
tarbase	hsa-miR-15b-5p	CACNA2D3	55799	ENSG00000157445	validated
tarbase	hsa-miR-212-3p	CACNA2D3	55799	ENSG00000157445	validated
tarbase	hsa-miR-106b-5p	APPL1	26060	ENSG00000157500	validated
tarbase	hsa-miR-15b-5p	APPL1	26060	ENSG00000157500	validated
tarbase	hsa-miR-21-5p	TSC22D3	1831	ENSG00000157514	validated
tarbase	hsa-miR-222-3p	TSC22D3	1831	ENSG00000157514	validated
tarbase	hsa-miR-106b-5p	TSC22D3	1831	ENSG00000157514	validated
tarbase	hsa-miR-296-5p	VPS26C	10311	ENSG00000157538	validated
tarbase	hsa-miR-15b-5p	DYRK1A	1859	ENSG00000157540	validated
tarbase	hsa-miR-222-3p	DYRK1A	1859	ENSG00000157540	validated
tarbase	hsa-miR-505-3p	DYRK1A	1859	ENSG00000157540	validated
tarbase	hsa-miR-582-5p	DYRK1A	1859	ENSG00000157540	validated
tarbase	hsa-miR-181a-5p	ERG	2078	ENSG00000157554	validated
tarbase	hsa-miR-21-5p	ERG	2078	ENSG00000157554	validated
tarbase	hsa-miR-222-3p	ETS2	2114	ENSG00000157557	validated
tarbase	hsa-miR-181a-5p	TMEM164	84187	ENSG00000157600	validated
tarbase	hsa-miR-21-5p	TMEM164	84187	ENSG00000157600	validated
tarbase	hsa-miR-106b-5p	C2CD2	25966	ENSG00000157617	validated
tarbase	hsa-miR-181a-5p	C2CD2	25966	ENSG00000157617	validated
tarbase	hsa-miR-18a-5p	C2CD2	25966	ENSG00000157617	validated
tarbase	hsa-miR-212-3p	C2CD2	25966	ENSG00000157617	validated
tarbase	hsa-miR-106b-5p	TAB3	257397	ENSG00000157625	validated
tarbase	hsa-miR-15b-5p	TAB3	257397	ENSG00000157625	validated
tarbase	hsa-miR-222-3p	TAB3	257397	ENSG00000157625	validated
tarbase	hsa-miR-296-5p	SLC38A10	124565	ENSG00000157637	validated
tarbase	hsa-miR-212-3p	C9orf43	257169	ENSG00000157653	validated
tarbase	hsa-miR-144-3p	PALM2AKAP2	445815	ENSG00000157654	validated
tarbase	hsa-miR-15b-5p	PALM2AKAP2	445815	ENSG00000157654	validated
tarbase	hsa-miR-181a-5p	PALM2AKAP2	445815	ENSG00000157654	validated
tarbase	hsa-miR-181a-5p	ZNF618	114991	ENSG00000157657	validated
tarbase	hsa-miR-181a-5p	TMEM268	203197	ENSG00000157693	validated
tarbase	hsa-miR-212-3p	TMEM268	203197	ENSG00000157693	validated
tarbase	hsa-miR-582-5p	TMEM268	203197	ENSG00000157693	validated
tarbase	hsa-miR-106b-5p	UBN2	254048	ENSG00000157741	validated
tarbase	hsa-miR-181a-5p	UBN2	254048	ENSG00000157741	validated
tarbase	hsa-miR-505-3p	UBN2	254048	ENSG00000157741	validated
tarbase	hsa-miR-18a-5p	UBN2	254048	ENSG00000157741	validated
tarbase	hsa-miR-106b-5p	BRAF	673	ENSG00000157764	validated
tarbase	hsa-miR-18a-5p	BRAF	673	ENSG00000157764	validated
tarbase	hsa-miR-212-3p	WDR19	57728	ENSG00000157796	validated
tarbase	hsa-miR-192-5p	SLC37A3	84255	ENSG00000157800	validated
tarbase	hsa-miR-582-3p	SLC37A3	84255	ENSG00000157800	validated
tarbase	hsa-miR-144-3p	FMNL2	114793	ENSG00000157827	validated
tarbase	hsa-miR-181a-5p	FMNL2	114793	ENSG00000157827	validated
tarbase	hsa-miR-212-3p	GAREM2	150946	ENSG00000157833	validated
tarbase	hsa-miR-296-5p	SPPL3	121665	ENSG00000157837	validated
tarbase	hsa-miR-21-5p	SKI	6497	ENSG00000157933	validated
tarbase	hsa-miR-296-5p	SKI	6497	ENSG00000157933	validated
tarbase	hsa-miR-106b-5p	LDLRAP1	26119	ENSG00000157978	validated
tarbase	hsa-miR-181a-5p	LDLRAP1	26119	ENSG00000157978	validated
tarbase	hsa-miR-181a-5p	AGAP1	116987	ENSG00000157985	validated
tarbase	hsa-miR-15b-5p	PAFAH2	5051	ENSG00000158006	validated
tarbase	hsa-miR-212-3p	EXTL1	2134	ENSG00000158008	validated
tarbase	hsa-miR-106b-5p	MRPL17	63875	ENSG00000158042	validated
tarbase	hsa-miR-15b-5p	DUSP2	1844	ENSG00000158050	validated
tarbase	hsa-miR-296-5p	DUSP2	1844	ENSG00000158050	validated
tarbase	hsa-miR-212-3p	GRHL3	57822	ENSG00000158055	validated
tarbase	hsa-miR-15b-5p	TPRG1L	127262	ENSG00000158109	validated
tarbase	hsa-miR-106b-5p	PRXL2C	195827	ENSG00000158122	validated
tarbase	hsa-miR-222-3p	CNNM4	26504	ENSG00000158158	validated
tarbase	hsa-miR-181a-5p	EYA3	2140	ENSG00000158161	validated
tarbase	hsa-miR-21-5p	WASF2	10163	ENSG00000158195	validated
tarbase	hsa-miR-222-3p	WASF2	10163	ENSG00000158195	validated

tarbase	hsa-miR-296-5p	WASF2	10163	ENSG00000158195	validated
tarbase	hsa-miR-222-3p	ABHD3	171586	ENSG00000158201	validated
tarbase	hsa-miR-15b-5p	CUL4B	8450	ENSG00000158290	validated
tarbase	hsa-miR-181a-5p	CUL4B	8450	ENSG00000158290	validated
tarbase	hsa-miR-212-3p	CUL4B	8450	ENSG00000158290	validated
tarbase	hsa-miR-505-3p			ENSG00000158301	validated
tarbase	hsa-miR-106b-5p	AUTS2	26053	ENSG00000158321	validated
tarbase	hsa-miR-15b-5p	H2BC5	3017	ENSG00000158373	validated
tarbase	hsa-miR-212-3p	CDC25C	995	ENSG00000158402	validated
tarbase	hsa-miR-212-3p	H4C8	8365	ENSG00000158406	validated
tarbase	hsa-miR-106b-5p	MITD1	129531	ENSG00000158411	validated
tarbase	hsa-miR-181a-5p	EIF5B	9669	ENSG00000158417	validated
tarbase	hsa-miR-21-5p	EIF5B	9669	ENSG00000158417	validated
tarbase	hsa-miR-301a-3p	EIF5B	9669	ENSG00000158417	validated
tarbase	hsa-miR-582-5p	EIF5B	9669	ENSG00000158417	validated
tarbase	hsa-miR-212-3p	RIBC1	158787	ENSG00000158423	validated
tarbase	hsa-miR-18a-5p	CNOT11	55571	ENSG00000158435	validated
tarbase	hsa-miR-212-3p	NRG2	9542	ENSG00000158458	validated
tarbase	hsa-miR-222-3p	AHCYL2	23382	ENSG00000158467	validated
tarbase	hsa-miR-106b-5p	B4GALT5	9334	ENSG00000158470	validated
tarbase	hsa-miR-181a-5p	B4GALT5	9334	ENSG00000158470	validated
tarbase	hsa-miR-21-5p	B4GALT5	9334	ENSG00000158470	validated
tarbase	hsa-miR-301a-3p	B4GALT5	9334	ENSG00000158470	validated
tarbase	hsa-miR-15b-5p	SPATA2	9825	ENSG00000158480	validated
tarbase	hsa-miR-181a-5p	SPATA2	9825	ENSG00000158480	validated
tarbase	hsa-miR-296-5p	SPATA2	9825	ENSG00000158480	validated
tarbase	hsa-miR-301a-3p	SPATA2	9825	ENSG00000158480	validated
tarbase	hsa-miR-582-3p	SPATA2	9825	ENSG00000158480	validated
tarbase	hsa-miR-212-3p	ZFAND2B	130617	ENSG00000158552	validated
tarbase	hsa-miR-15b-5p	GDPD5	81544	ENSG00000158555	validated
tarbase	hsa-miR-21-5p	TMED4	222068	ENSG00000158604	validated
tarbase	hsa-miR-301a-3p	TMED4	222068	ENSG00000158604	validated
tarbase	hsa-miR-181a-5p	PPP1R15B	84919	ENSG00000158615	validated
tarbase	hsa-miR-18a-5p	PPP1R15B	84919	ENSG00000158615	validated
tarbase	hsa-miR-192-5p	PPP1R15B	84919	ENSG00000158615	validated
tarbase	hsa-miR-212-3p	PPP1R15B	84919	ENSG00000158615	validated
tarbase	hsa-miR-582-3p	PPP1R15B	84919	ENSG00000158615	validated
tarbase	hsa-miR-21-5p	EMSY	56946	ENSG00000158636	validated
tarbase	hsa-miR-301a-3p	EMSY	56946	ENSG00000158636	validated
tarbase	hsa-miR-106b-5p	TAGLN2	8407	ENSG00000158710	validated
tarbase	hsa-miR-212-3p	TAGLN2	8407	ENSG00000158710	validated
tarbase	hsa-miR-18a-5p	ELK4	2005	ENSG00000158711	validated
tarbase	hsa-miR-192-5p	ELK4	2005	ENSG00000158711	validated
tarbase	hsa-miR-212-3p	SLAMF8	56833	ENSG00000158714	validated
tarbase	hsa-miR-301a-3p	RNF166	115992	ENSG00000158717	validated
tarbase	hsa-miR-106b-5p	F11R	50848	ENSG00000158769	validated
tarbase	hsa-miR-296-5p	NIT1	4817	ENSG00000158793	validated
tarbase	hsa-miR-106b-5p	DEDD	9191	ENSG00000158796	validated
tarbase	hsa-miR-212-3p	DEDD	9191	ENSG00000158796	validated
tarbase	hsa-miR-296-5p	DEDD	9191	ENSG00000158796	validated
tarbase	hsa-miR-296-5p	ZNF276	92822	ENSG00000158805	validated
tarbase	hsa-miR-15b-5p	EDA	1896	ENSG00000158813	validated
tarbase	hsa-miR-15b-5p	PINK1	65018	ENSG00000158828	validated
tarbase	hsa-miR-106b-5p	NDUFS2	4720	ENSG00000158864	validated
tarbase	hsa-miR-21-5p	CCAR2	57805	ENSG00000158941	validated
tarbase	hsa-miR-106b-5p	CACHD1	57685	ENSG00000158966	validated
tarbase	hsa-miR-106b-5p	CDC42SE2	56990	ENSG00000158985	validated
tarbase	hsa-miR-144-3p	CDC42SE2	56990	ENSG00000158985	validated
tarbase	hsa-miR-181a-5p	CDC42SE2	56990	ENSG00000158985	validated
tarbase	hsa-miR-212-3p	CDC42SE2	56990	ENSG00000158985	validated
tarbase	hsa-miR-582-5p	CDC42SE2	56990	ENSG00000158985	validated
tarbase	hsa-miR-18a-5p	RAPGEF6	51735	ENSG00000158987	validated
tarbase	hsa-miR-181a-5p	RAPGEF6	51735	ENSG00000158987	validated
tarbase	hsa-miR-181a-5p	EPB41	2035	ENSG00000159023	validated
tarbase	hsa-miR-212-3p	FBXW5	54461	ENSG00000159069	validated
tarbase	hsa-miR-301a-3p	IFNAR2	3455	ENSG00000159110	validated
tarbase	hsa-miR-106b-5p	MRPL10	124995	ENSG00000159111	validated
tarbase	hsa-miR-15b-5p	GART	2618	ENSG00000159131	validated
tarbase	hsa-miR-15b-5p	SON	6651	ENSG00000159140	validated
tarbase	hsa-miR-181a-5p	SON	6651	ENSG00000159140	validated
tarbase	hsa-miR-212-3p	SON	6651	ENSG00000159140	validated

tarbase	hsa-miR-296-5p	SON	6651	ENSG00000159140	validated
tarbase	hsa-miR-301a-3p	SON	6651	ENSG00000159140	validated
tarbase	hsa-miR-326	SON	6651	ENSG00000159140	validated
tarbase	hsa-miR-582-5p	SON	6651	ENSG00000159140	validated
tarbase	hsa-miR-582-3p	DONSON	29980	ENSG00000159147	validated
tarbase	hsa-miR-18a-5p	SV2A	9900	ENSG00000159164	validated
tarbase	hsa-miR-106b-5p	STC1	6781	ENSG00000159167	validated
tarbase	hsa-miR-222-3p	STC1	6781	ENSG00000159167	validated
tarbase	hsa-miR-301a-3p	STC1	6781	ENSG00000159167	validated
tarbase	hsa-miR-15b-5p	C1QC	714	ENSG00000159189	validated
tarbase	hsa-miR-106b-5p	RCAN1	1827	ENSG00000159200	validated
tarbase	hsa-miR-212-3p	RCAN1	1827	ENSG00000159200	validated
tarbase	hsa-miR-144-3p	UBE2Z	65264	ENSG00000159202	validated
tarbase	hsa-miR-181a-5p	UBE2Z	65264	ENSG00000159202	validated
tarbase	hsa-miR-18a-5p	UBE2Z	65264	ENSG00000159202	validated
tarbase	hsa-miR-326	UBE2Z	65264	ENSG00000159202	validated
tarbase	hsa-miR-582-5p	UBE2Z	65264	ENSG00000159202	validated
tarbase	hsa-miR-106b-5p	RUNX1	861	ENSG00000159216	validated
tarbase	hsa-miR-212-3p	RUNX1	861	ENSG00000159216	validated
tarbase	hsa-miR-106b-5p	IGF2BP1	10642	ENSG00000159217	validated
tarbase	hsa-miR-181a-5p	IGF2BP1	10642	ENSG00000159217	validated
tarbase	hsa-miR-21-5p	IGF2BP1	10642	ENSG00000159217	validated
tarbase	hsa-miR-301a-3p	IGF2BP1	10642	ENSG00000159217	validated
tarbase	hsa-miR-582-3p	IGF2BP1	10642	ENSG00000159217	validated
tarbase	hsa-miR-181a-5p	MORC3	23515	ENSG00000159256	validated
tarbase	hsa-miR-212-3p	MORC3	23515	ENSG00000159256	validated
tarbase	hsa-miR-18a-5p	SIM2	6493	ENSG00000159263	validated
tarbase	hsa-miR-181a-5p	HLCS	3141	ENSG00000159267	validated
tarbase	hsa-miR-106b-5p	ADIPOR1	51094	ENSG00000159346	validated
tarbase	hsa-miR-15b-5p	ADIPOR1	51094	ENSG00000159346	validated
tarbase	hsa-miR-222-3p	ADIPOR1	51094	ENSG00000159346	validated
tarbase	hsa-miR-18a-5p	CYB5R1	51706	ENSG00000159348	validated
tarbase	hsa-miR-106b-5p	PSMB4	5692	ENSG00000159377	validated
tarbase	hsa-miR-18a-5p	BTG2	7832	ENSG00000159388	validated
tarbase	hsa-miR-212-3p	BTG2	7832	ENSG00000159388	validated
tarbase	hsa-miR-222-3p	BTG2	7832	ENSG00000159388	validated
tarbase	hsa-miR-296-5p	BTG2	7832	ENSG00000159388	validated
tarbase	hsa-miR-326	BTG2	7832	ENSG00000159388	validated
tarbase	hsa-miR-582-5p	BTG2	7832	ENSG00000159388	validated
tarbase	hsa-miR-181a-5p	HK2	3099	ENSG00000159399	validated
tarbase	hsa-miR-21-5p	HK2	3099	ENSG00000159399	validated
tarbase	hsa-miR-106b-5p	UBR1	197131	ENSG00000159459	validated
tarbase	hsa-miR-106b-5p	AMFR	267	ENSG00000159461	validated
tarbase	hsa-miR-21-5p	AMFR	267	ENSG00000159461	validated
tarbase	hsa-miR-15b-5p	AMFR	267	ENSG00000159461	validated
tarbase	hsa-miR-181a-5p	MED8	112950	ENSG00000159479	validated
tarbase	hsa-miR-15b-5p	RGL4	266747	ENSG00000159496	validated
tarbase	hsa-miR-212-3p	ISL2	64843	ENSG00000159556	validated
tarbase	hsa-miR-181a-5p	RSPRY1	89970	ENSG00000159579	validated
tarbase	hsa-miR-301a-3p	RSPRY1	89970	ENSG00000159579	validated
tarbase	hsa-miR-505-3p	RSPRY1	89970	ENSG00000159579	validated
tarbase	hsa-miR-106b-5p	GPBP1L1	60313	ENSG00000159592	validated
tarbase	hsa-miR-15b-5p	GPBP1L1	60313	ENSG00000159592	validated
tarbase	hsa-miR-181a-5p	GPBP1L1	60313	ENSG00000159592	validated
tarbase	hsa-miR-15b-5p	NAE1	8883	ENSG00000159593	validated
tarbase	hsa-miR-18a-5p	NAE1	8883	ENSG00000159593	validated
tarbase	hsa-miR-582-5p	NAE1	8883	ENSG00000159593	validated
tarbase	hsa-miR-301a-3p	EFCAB14	9813	ENSG00000159658	validated
tarbase	hsa-miR-106b-5p	CTBP1	1487	ENSG00000159692	validated
tarbase	hsa-miR-18a-5p	TPPP3	51673	ENSG00000159713	validated
tarbase	hsa-miR-18a-5p	ZDHHC1	29800	ENSG00000159714	validated
tarbase	hsa-miR-212-3p	ATP6V0D1	9114	ENSG00000159720	validated
tarbase	hsa-miR-582-3p	ATP6V0D1	9114	ENSG00000159720	validated
tarbase	hsa-miR-181a-5p	PSKH1	5681	ENSG00000159792	validated
tarbase	hsa-miR-582-3p	PSKH1	5681	ENSG00000159792	validated
tarbase	hsa-miR-15b-5p	ZYX	7791	ENSG00000159840	validated
tarbase	hsa-miR-192-5p	ABR	29	ENSG00000159842	validated
tarbase	hsa-miR-212-3p	ZNF230	7773	ENSG00000159882	validated
tarbase	hsa-miR-212-3p	ZNF222	7673	ENSG00000159885	validated
tarbase	hsa-miR-212-3p	ZNF233	353355	ENSG00000159915	validated
tarbase	hsa-miR-212-3p	ZNF235	9310	ENSG00000159917	validated

tarbase	hsa-miR-18a-5p	GNE	10020	ENSG00000159921	validated
tarbase	hsa-miR-15b-5p	ARHGAP35	2909	ENSG00000160007	validated
tarbase	hsa-miR-181a-5p	ARHGAP35	2909	ENSG00000160007	validated
tarbase	hsa-miR-21-5p	ARHGAP35	2909	ENSG00000160007	validated
tarbase	hsa-miR-181a-5p	CALM3	808	ENSG00000160014	validated
tarbase	hsa-miR-296-5p	CALM3	808	ENSG00000160014	validated
tarbase	hsa-miR-18a-5p	DFFA	1676	ENSG00000160049	validated
tarbase	hsa-miR-212-3p	DFFA	1676	ENSG00000160049	validated
tarbase	hsa-miR-15b-5p	BSDC1	55108	ENSG00000160058	validated
tarbase	hsa-miR-296-5p	BSDC1	55108	ENSG00000160058	validated
tarbase	hsa-miR-326	BSDC1	55108	ENSG00000160058	validated
tarbase	hsa-miR-106b-5p	ZBTB8A	653121	ENSG00000160062	validated
tarbase	hsa-miR-18a-5p	ZBTB8A	653121	ENSG00000160062	validated
tarbase	hsa-miR-582-3p	ZBTB8A	653121	ENSG00000160062	validated
tarbase	hsa-miR-222-3p	UBE2J2	118424	ENSG00000160087	validated
tarbase	hsa-miR-106b-5p	ZNF362	149076	ENSG00000160094	validated
tarbase	hsa-miR-212-3p	ZNF362	149076	ENSG00000160094	validated
tarbase	hsa-miR-106b-5p	VMA21	203547	ENSG00000160131	validated
tarbase	hsa-miR-144-3p	VMA21	203547	ENSG00000160131	validated
tarbase	hsa-miR-15b-5p	VMA21	203547	ENSG00000160131	validated
tarbase	hsa-miR-181a-5p	VMA21	203547	ENSG00000160131	validated
tarbase	hsa-miR-212-3p	RSPH1	89765	ENSG00000160188	validated
tarbase	hsa-miR-106b-5p	WDR4	10785	ENSG00000160193	validated
tarbase	hsa-miR-181a-5p	WDR4	10785	ENSG00000160193	validated
tarbase	hsa-miR-18a-5p	NDUFV3	4731	ENSG00000160194	validated
tarbase	hsa-miR-181a-5p	PKNOX1	5316	ENSG00000160199	validated
tarbase	hsa-miR-296-5p	CBS	875	ENSG00000160200	validated
tarbase	hsa-miR-18a-5p	U2AF1	7307	ENSG00000160201	validated
tarbase	hsa-miR-181a-5p	RRP1B	23076	ENSG00000160208	validated
tarbase	hsa-miR-192-5p	RRP1B	23076	ENSG00000160208	validated
tarbase	hsa-miR-301a-3p	G6PD	2539	ENSG00000160211	validated
tarbase	hsa-miR-21-5p	CSTB	1476	ENSG00000160213	validated
tarbase	hsa-miR-106b-5p	AGPAT3	56894	ENSG00000160216	validated
tarbase	hsa-miR-15b-5p	AGPAT3	56894	ENSG00000160216	validated
tarbase	hsa-miR-181a-5p	AGPAT3	56894	ENSG00000160216	validated
tarbase	hsa-miR-18a-5p	AGPAT3	56894	ENSG00000160216	validated
tarbase	hsa-miR-18a-5p	TRAPPC10	7109	ENSG00000160218	validated
tarbase	hsa-miR-21-5p	TRAPPC10	7109	ENSG00000160218	validated
tarbase	hsa-miR-181a-5p	ZNF66	7617	ENSG00000160229	validated
tarbase	hsa-miR-15b-5p	FTCD	10841	ENSG00000160282	validated
tarbase	hsa-miR-181a-5p	LSS	4047	ENSG00000160285	validated
tarbase	hsa-miR-21-5p	LSS	4047	ENSG00000160285	validated
tarbase	hsa-miR-181a-5p	MCM3AP	8888	ENSG00000160294	validated
tarbase	hsa-miR-296-5p	C21orf58	54058	ENSG00000160298	validated
tarbase	hsa-miR-326	DIP2A	23181	ENSG00000160305	validated
tarbase	hsa-miR-505-3p	DIP2A	23181	ENSG00000160305	validated
tarbase	hsa-miR-212-3p	CLDND2	125875	ENSG00000160318	validated
tarbase	hsa-miR-106b-5p	ZNF714	148206	ENSG00000160352	validated
tarbase	hsa-miR-18a-5p	ZNF714	148206	ENSG00000160352	validated
tarbase	hsa-miR-18a-5p	GPSM1	26086	ENSG00000160360	validated
tarbase	hsa-miR-192-5p	GPSM1	26086	ENSG00000160360	validated
tarbase	hsa-miR-296-5p	GPSM1	26086	ENSG00000160360	validated
tarbase	hsa-miR-326	GPSM1	26086	ENSG00000160360	validated
tarbase	hsa-miR-181a-5p	ST6GALNAC6	30815	ENSG00000160408	validated
tarbase	hsa-miR-15b-5p	ZER1	10444	ENSG00000160445	validated
tarbase	hsa-miR-15b-5p	ZDHHC12	84885	ENSG00000160446	validated
tarbase	hsa-miR-181a-5p	ZDHHC12	84885	ENSG00000160446	validated
tarbase	hsa-miR-296-5p	PKN3	29941	ENSG00000160447	validated
tarbase	hsa-miR-212-3p	PLPP7	84814	ENSG00000160539	validated
tarbase	hsa-miR-106b-5p	TAOK1	57551	ENSG00000160551	validated
tarbase	hsa-miR-181a-5p	TAOK1	57551	ENSG00000160551	validated
tarbase	hsa-miR-212-3p	TAOK1	57551	ENSG00000160551	validated
tarbase	hsa-miR-21-5p	TAOK1	57551	ENSG00000160551	validated
tarbase	hsa-miR-222-3p	TAOK1	57551	ENSG00000160551	validated
tarbase	hsa-miR-296-5p	TAOK1	57551	ENSG00000160551	validated
tarbase	hsa-miR-326	TAOK1	57551	ENSG00000160551	validated
tarbase	hsa-miR-15b-5p	SIK3	23387	ENSG00000160584	validated
tarbase	hsa-miR-181a-5p	SIK3	23387	ENSG00000160584	validated
tarbase	hsa-miR-326	SIK3	23387	ENSG00000160584	validated
tarbase	hsa-miR-15b-5p	MPZL3	196264	ENSG00000160588	validated
tarbase	hsa-miR-301a-3p	CHTOP	26097	ENSG00000160679	validated

tarbase	hsa-miR-106b-5p	FLAD1	80308	ENSG00000160688	validated
tarbase	hsa-miR-21-5p	FLAD1	80308	ENSG00000160688	validated
tarbase	hsa-miR-18a-5p	FLAD1	80308	ENSG00000160688	validated
tarbase	hsa-miR-15b-5p	NLRX1	79671	ENSG00000160703	validated
tarbase	hsa-miR-301a-3p	ADAR	103	ENSG00000160710	validated
tarbase	hsa-miR-582-5p	ADAR	103	ENSG00000160710	validated
tarbase	hsa-miR-212-3p	ADAR	103	ENSG00000160710	validated
tarbase	hsa-miR-212-3p	IL6R	3570	ENSG00000160712	validated
tarbase	hsa-miR-181a-5p	UBE2Q1	55585	ENSG00000160714	validated
tarbase	hsa-miR-21-5p	UBE2Q1	55585	ENSG00000160714	validated
tarbase	hsa-miR-106b-5p	CRTC2	200186	ENSG00000160741	validated
tarbase	hsa-miR-192-5p	CRTC2	200186	ENSG00000160741	validated
tarbase	hsa-miR-301a-3p	CRTC2	200186	ENSG00000160741	validated
tarbase	hsa-miR-106b-5p	RUSC1	23623	ENSG00000160753	validated
tarbase	hsa-miR-15b-5p	RUSC1	23623	ENSG00000160753	validated
tarbase	hsa-miR-15b-5p	FAM189B	10712	ENSG00000160767	validated
tarbase	hsa-miR-296-5p	FAM189B	10712	ENSG00000160767	validated
tarbase	hsa-miR-15b-5p	PAQR6	79957	ENSG00000160781	validated
tarbase	hsa-miR-301a-3p	SLC25A44	9673	ENSG00000160785	validated
tarbase	hsa-miR-18a-5p	LMNA	4000	ENSG00000160789	validated
tarbase	hsa-miR-296-5p	LMNA	4000	ENSG00000160789	validated
tarbase	hsa-miR-301a-3p	LMNA	4000	ENSG00000160789	validated
tarbase	hsa-miR-106b-5p	NACC1	112939	ENSG00000160877	validated
tarbase	hsa-miR-181a-5p	NACC1	112939	ENSG00000160877	validated
tarbase	hsa-miR-296-5p	NACC1	112939	ENSG00000160877	validated
tarbase	hsa-miR-106b-5p	ZNF394	84124	ENSG00000160908	validated
tarbase	hsa-miR-212-3p	CPSF4	10898	ENSG00000160917	validated
tarbase	hsa-miR-15b-5p	LY6E	4061	ENSG00000160932	validated
tarbase	hsa-miR-212-3p	LY6E	4061	ENSG00000160932	validated
tarbase	hsa-miR-301a-3p	PWWP3A	84939	ENSG00000160953	validated
tarbase	hsa-miR-212-3p	LRRC14	9684	ENSG00000160959	validated
tarbase	hsa-miR-301a-3p	PPP1R16A	84988	ENSG00000160972	validated
tarbase	hsa-miR-18a-5p	ALKBH4	54784	ENSG00000160993	validated
tarbase	hsa-miR-15b-5p	SQSTM1	8878	ENSG00000161011	validated
tarbase	hsa-miR-222-3p	SQSTM1	8878	ENSG00000161011	validated
tarbase	hsa-miR-15b-5p	MGAT4B	11282	ENSG00000161013	validated
tarbase	hsa-miR-18a-5p	MGAT4B	11282	ENSG00000161013	validated
tarbase	hsa-miR-301a-3p	MGAT4B	11282	ENSG00000161013	validated
tarbase	hsa-miR-212-3p	MAML1	9794	ENSG00000161021	validated
tarbase	hsa-miR-222-3p	MAML1	9794	ENSG00000161021	validated
tarbase	hsa-miR-301a-3p	MAML1	9794	ENSG00000161021	validated
tarbase	hsa-miR-106b-5p	NAPEPLD	222236	ENSG00000161048	validated
tarbase	hsa-miR-222-3p	NAPEPLD	222236	ENSG00000161048	validated
tarbase	hsa-miR-15b-5p	PSMC2	5701	ENSG00000161057	validated
tarbase	hsa-miR-18a-5p	PSMC2	5701	ENSG00000161057	validated
tarbase	hsa-miR-582-5p	PSMC2	5701	ENSG00000161057	validated
tarbase	hsa-miR-106b-5p	DVL3	1857	ENSG00000161202	validated
tarbase	hsa-miR-15b-5p	DVL3	1857	ENSG00000161202	validated
tarbase	hsa-miR-15b-5p	ABCF3	55324	ENSG00000161204	validated
tarbase	hsa-miR-106b-5p	PCYT1A	5130	ENSG00000161217	validated
tarbase	hsa-miR-212-3p	U2AF1L4	199746	ENSG00000161265	validated
tarbase	hsa-miR-212-3p	ZNF382	84911	ENSG00000161298	validated
tarbase	hsa-miR-181a-5p	SRSF2	6427	ENSG00000161547	validated
tarbase	hsa-miR-21-5p	SRSF2	6427	ENSG00000161547	validated
tarbase	hsa-miR-15b-5p	SRSF2	6427	ENSG00000161547	validated
tarbase	hsa-miR-296-5p	ALDH16A1	126133	ENSG00000161618	validated
tarbase	hsa-miR-326	ITGA5	3678	ENSG00000161638	validated
tarbase	hsa-miR-18a-5p	EMC10	284361	ENSG00000161671	validated
tarbase	hsa-miR-326	EMC10	284361	ENSG00000161671	validated
tarbase	hsa-miR-582-3p	EMC10	284361	ENSG00000161671	validated
tarbase	hsa-miR-15b-5p	JOSD2	126119	ENSG00000161677	validated
tarbase	hsa-miR-15b-5p	LARP4	113251	ENSG00000161813	validated
tarbase	hsa-miR-181a-5p	LARP4	113251	ENSG00000161813	validated
tarbase	hsa-miR-21-5p	LARP4	113251	ENSG00000161813	validated
tarbase	hsa-miR-301a-3p	LARP4	113251	ENSG00000161813	validated
tarbase	hsa-miR-301a-3p	RAVER1	125950	ENSG00000161847	validated
tarbase	hsa-miR-21-5p	SPC24	147841	ENSG00000161888	validated
tarbase	hsa-miR-181a-5p	SENP3	26168	ENSG00000161956	validated
tarbase	hsa-miR-15b-5p	POLR3K	51728	ENSG00000161980	validated
tarbase	hsa-miR-505-3p	POLR3K	51728	ENSG00000161980	validated
tarbase	hsa-miR-106b-5p	CCNF	899	ENSG00000162063	validated

tarbase	hsa-miR-106b-5p	TBC1D24	57465	ENSG00000162065	validated
tarbase	hsa-miR-296-5p	AMDHD2	51005	ENSG00000162066	validated
tarbase	hsa-miR-301a-3p	PAQR4	124222	ENSG00000162073	validated
tarbase	hsa-miR-212-3p	ZG16B	124220	ENSG00000162078	validated
tarbase	hsa-miR-106b-5p	SHANK2	22941	ENSG00000162105	validated
tarbase	hsa-miR-144-3p	SHANK2	22941	ENSG00000162105	validated
tarbase	hsa-miR-15b-5p	SHANK2	22941	ENSG00000162105	validated
tarbase	hsa-miR-18a-5p	SHANK2	22941	ENSG00000162105	validated
tarbase	hsa-miR-301a-3p	SHANK2	22941	ENSG00000162105	validated
tarbase	hsa-miR-222-3p	TAF6L	10629	ENSG00000162227	validated
tarbase	hsa-miR-212-3p	SYVN1	84447	ENSG00000162298	validated
tarbase	hsa-miR-582-3p	SYVN1	84447	ENSG00000162298	validated
tarbase	hsa-miR-106b-5p	RPS6KA4	8986	ENSG00000162302	validated
tarbase	hsa-miR-296-5p	LRP5	4041	ENSG00000162337	validated
tarbase	hsa-miR-21-5p	TPCN2	219931	ENSG00000162341	validated
tarbase	hsa-miR-181a-5p	ELAVL4	1996	ENSG00000162374	validated
tarbase	hsa-miR-106b-5p	ZYG11B	79699	ENSG00000162378	validated
tarbase	hsa-miR-181a-5p	ZYG11B	79699	ENSG00000162378	validated
tarbase	hsa-miR-212-3p	ZYG11B	79699	ENSG00000162378	validated
tarbase	hsa-miR-296-5p	PARS2	25973	ENSG00000162396	validated
tarbase	hsa-miR-181a-5p	USP24	23358	ENSG00000162402	validated
tarbase	hsa-miR-301a-3p	USP24	23358	ENSG00000162402	validated
tarbase	hsa-miR-582-5p	USP24	23358	ENSG00000162402	validated
tarbase	hsa-miR-21-5p	PLPP3	8613	ENSG00000162407	validated
tarbase	hsa-miR-505-3p	PLPP3	8613	ENSG00000162407	validated
tarbase	hsa-miR-18a-5p	NOL9	79707	ENSG00000162408	validated
tarbase	hsa-miR-21-5p	PRKAA2	5563	ENSG00000162409	validated
tarbase	hsa-miR-222-3p	PRKAA2	5563	ENSG00000162409	validated
tarbase	hsa-miR-296-5p	PRKAA2	5563	ENSG00000162409	validated
tarbase	hsa-miR-181a-5p	PRKAA2	5563	ENSG00000162409	validated
tarbase	hsa-miR-582-3p	KLHL21	9903	ENSG00000162413	validated
tarbase	hsa-miR-18a-5p	ZSWIM5	57643	ENSG00000162415	validated
tarbase	hsa-miR-212-3p	ZSWIM5	57643	ENSG00000162415	validated
tarbase	hsa-miR-296-5p	SELENON	57190	ENSG00000162430	validated
tarbase	hsa-miR-212-3p	SELENON	57190	ENSG00000162430	validated
tarbase	hsa-miR-212-3p	AK4	205	ENSG00000162433	validated
tarbase	hsa-miR-21-5p	RAVER2	55225	ENSG00000162437	validated
tarbase	hsa-miR-106b-5p	DHRS3	9249	ENSG00000162496	validated
tarbase	hsa-miR-15b-5p	SDC3	9672	ENSG00000162512	validated
tarbase	hsa-miR-296-5p	PEF1	553115	ENSG00000162517	validated
tarbase	hsa-miR-106b-5p	KIAA1522	57648	ENSG00000162522	validated
tarbase	hsa-miR-15b-5p	KIAA1522	57648	ENSG00000162522	validated
tarbase	hsa-miR-582-3p	KIAA1522	57648	ENSG00000162522	validated
tarbase	hsa-miR-106b-5p	CAMK2N1	55450	ENSG00000162545	validated
tarbase	hsa-miR-181a-5p	CAMK2N1	55450	ENSG00000162545	validated
tarbase	hsa-miR-18a-5p	CAMK2N1	55450	ENSG00000162545	validated
tarbase	hsa-miR-212-3p	CAMK2N1	55450	ENSG00000162545	validated
tarbase	hsa-miR-106b-5p	NFIA	4774	ENSG00000162599	validated
tarbase	hsa-miR-181a-5p	NFIA	4774	ENSG00000162599	validated
tarbase	hsa-miR-301a-3p	NFIA	4774	ENSG00000162599	validated
tarbase	hsa-miR-212-3p	NFIA	4774	ENSG00000162599	validated
tarbase	hsa-miR-15b-5p	OMA1	115209	ENSG00000162600	validated
tarbase	hsa-miR-15b-5p	MYSM1	114803	ENSG00000162601	validated
tarbase	hsa-miR-181a-5p	MYSM1	114803	ENSG00000162601	validated
tarbase	hsa-miR-181a-5p	USP1	7398	ENSG00000162607	validated
tarbase	hsa-miR-21-5p	USP1	7398	ENSG00000162607	validated
tarbase	hsa-miR-301a-3p	USP1	7398	ENSG00000162607	validated
tarbase	hsa-miR-18a-5p	FUBP1	8880	ENSG00000162613	validated
tarbase	hsa-miR-106b-5p	FUBP1	8880	ENSG00000162613	validated
tarbase	hsa-miR-15b-5p	DNAJB4	11080	ENSG00000162616	validated
tarbase	hsa-miR-192-5p	TYW3	127253	ENSG00000162623	validated
tarbase	hsa-miR-212-3p	LHX8	431707	ENSG00000162624	validated
tarbase	hsa-miR-301a-3p	NTNG1	22854	ENSG00000162631	validated
tarbase	hsa-miR-21-5p	FAM102B	284611	ENSG00000162636	validated
tarbase	hsa-miR-181a-5p	C1orf52	148423	ENSG00000162642	validated
tarbase	hsa-miR-212-3p	WDR63	126820	ENSG00000162643	validated
tarbase	hsa-miR-582-5p	GBP4	115361	ENSG00000162654	validated
tarbase	hsa-miR-106b-5p	ZNF326	284695	ENSG00000162664	validated
tarbase	hsa-miR-15b-5p	ZNF326	284695	ENSG00000162664	validated
tarbase	hsa-miR-181a-5p	ZNF326	284695	ENSG00000162664	validated
tarbase	hsa-miR-212-3p	BRINP3	339479	ENSG00000162670	validated

tarbase	hsa-miR-106b-5p	AGL	178	ENSG00000162688	validated
tarbase	hsa-miR-212-3p	EXTL2	2135	ENSG00000162694	validated
tarbase	hsa-miR-582-3p	EXTL2	2135	ENSG00000162694	validated
tarbase	hsa-miR-106b-5p	EXTL2	2135	ENSG00000162694	validated
tarbase	hsa-miR-15b-5p	SLC30A7	148867	ENSG00000162695	validated
tarbase	hsa-miR-18a-5p	SLC30A7	148867	ENSG00000162695	validated
tarbase	hsa-miR-582-5p	SLC30A7	148867	ENSG00000162695	validated
tarbase	hsa-miR-15b-5p	ZNF281	23528	ENSG00000162702	validated
tarbase	hsa-miR-181a-5p	ZNF281	23528	ENSG00000162702	validated
tarbase	hsa-miR-18a-5p	ZNF281	23528	ENSG00000162702	validated
tarbase	hsa-miR-21-5p	ZNF281	23528	ENSG00000162702	validated
tarbase	hsa-miR-582-5p	ZNF281	23528	ENSG00000162702	validated
tarbase	hsa-miR-212-3p	IGSF8	93185	ENSG00000162729	validated
tarbase	hsa-miR-18a-5p	DDR2	4921	ENSG00000162733	validated
tarbase	hsa-miR-21-5p	PEA15	8682	ENSG00000162734	validated
tarbase	hsa-miR-15b-5p	PEX19	5824	ENSG00000162735	validated
tarbase	hsa-miR-222-3p	SLAMF6	114836	ENSG00000162739	validated
tarbase	hsa-miR-296-5p	SLAMF6	114836	ENSG00000162739	validated
tarbase	hsa-miR-212-3p	FCRLB	127943	ENSG00000162746	validated
tarbase	hsa-miR-106b-5p	KLHDC9	126823	ENSG00000162755	validated
tarbase	hsa-miR-181a-5p	RBM15	64783	ENSG00000162775	validated
tarbase	hsa-miR-18a-5p	IER5	51278	ENSG00000162783	validated
tarbase	hsa-miR-15b-5p	BPNT1	10380	ENSG00000162813	validated
tarbase	hsa-miR-212-3p	SPATA17	128153	ENSG00000162814	validated
tarbase	hsa-miR-18a-5p	BROX	148362	ENSG00000162819	validated
tarbase	hsa-miR-301a-3p	BROX	148362	ENSG00000162819	validated
tarbase	hsa-miR-106b-5p	CNST	163882	ENSG00000162852	validated
tarbase	hsa-miR-15b-5p	CNST	163882	ENSG00000162852	validated
tarbase	hsa-miR-181a-5p	CNST	163882	ENSG00000162852	validated
tarbase	hsa-miR-15b-5p	PPP1R21	129285	ENSG00000162869	validated
tarbase	hsa-miR-212-3p	CAPN2	824	ENSG00000162909	validated
tarbase	hsa-miR-21-5p	CAPN2	824	ENSG00000162909	validated
tarbase	hsa-miR-106b-5p	WDR26	80232	ENSG00000162923	validated
tarbase	hsa-miR-15b-5p	WDR26	80232	ENSG00000162923	validated
tarbase	hsa-miR-181a-5p	WDR26	80232	ENSG00000162923	validated
tarbase	hsa-miR-18a-5p	WDR26	80232	ENSG00000162923	validated
tarbase	hsa-miR-212-3p	WDR26	80232	ENSG00000162923	validated
tarbase	hsa-miR-21-5p	REL	5966	ENSG00000162924	validated
tarbase	hsa-miR-106b-5p	PEX13	5194	ENSG00000162928	validated
tarbase	hsa-miR-505-3p	KIAA1841	84542	ENSG00000162929	validated
tarbase	hsa-miR-181a-5p	DPY30	84661	ENSG00000162961	validated
tarbase	hsa-miR-106b-5p	TYW5	129450	ENSG00000162971	validated
tarbase	hsa-miR-15b-5p	MAIP1	79568	ENSG00000162972	validated
tarbase	hsa-miR-301a-3p	SLC66A3	130814	ENSG00000162976	validated
tarbase	hsa-miR-181a-5p	ARL5A	26225	ENSG00000162980	validated
tarbase	hsa-miR-212-3p	CLHC1	130162	ENSG00000162994	validated
tarbase	hsa-miR-18a-5p	CFAP36	112942	ENSG00000163001	validated
tarbase	hsa-miR-15b-5p	FBXO41	150726	ENSG00000163013	validated
tarbase	hsa-miR-18a-5p	SMC6	79677	ENSG00000163029	validated
tarbase	hsa-miR-212-3p	VSNL1	7447	ENSG00000163032	validated
tarbase	hsa-miR-181a-5p	COQ8A	56997	ENSG00000163050	validated
tarbase	hsa-miR-106b-5p	SLC16A14	151473	ENSG00000163053	validated
tarbase	hsa-miR-15b-5p	SLC16A14	151473	ENSG00000163053	validated
tarbase	hsa-miR-212-3p	SPATA18	132671	ENSG00000163071	validated
tarbase	hsa-miR-15b-5p	SGPP2	130367	ENSG00000163082	validated
tarbase	hsa-miR-212-3p	INHBB	3625	ENSG00000163083	validated
tarbase	hsa-miR-301a-3p	INHBB	3625	ENSG00000163083	validated
tarbase	hsa-miR-106b-5p	PDLIM5	10611	ENSG00000163110	validated
tarbase	hsa-miR-18a-5p	PDLIM5	10611	ENSG00000163110	validated
tarbase	hsa-miR-181a-5p	PDLIM5	10611	ENSG00000163110	validated
tarbase	hsa-miR-212-3p	NEURL3	93082	ENSG00000163121	validated
tarbase	hsa-miR-222-3p	RPRD2	23248	ENSG00000163125	validated
tarbase	hsa-miR-15b-5p	ANKRD23	200539	ENSG00000163126	validated
tarbase	hsa-miR-505-3p	PACRGL	133015	ENSG00000163138	validated
tarbase	hsa-miR-222-3p	LYSMD1	388695	ENSG00000163155	validated
tarbase	hsa-miR-505-3p	LYSMD1	388695	ENSG00000163155	validated
tarbase	hsa-miR-582-3p	LYSMD1	388695	ENSG00000163155	validated
tarbase	hsa-miR-212-3p	LYSMD1	388695	ENSG00000163155	validated
tarbase	hsa-miR-106b-5p	VPS72	6944	ENSG00000163159	validated
tarbase	hsa-miR-15b-5p	VPS72	6944	ENSG00000163159	validated
tarbase	hsa-miR-106b-5p	RNF149	284996	ENSG00000163162	validated

tarbase	hsa-miR-21-5p	RNF149	284996	ENSG00000163162	validated
tarbase	hsa-miR-582-5p	RNF149	284996	ENSG00000163162	validated
tarbase	hsa-miR-181a-5p	IWS1	55677	ENSG00000163166	validated
tarbase	hsa-miR-212-3p	IVL	3713	ENSG00000163207	validated
tarbase	hsa-miR-212-3p	CCNYL1	151195	ENSG00000163249	validated
tarbase	hsa-miR-192-5p	FZD5	7855	ENSG00000163251	validated
tarbase	hsa-miR-106b-5p	FZD5	7855	ENSG00000163251	validated
tarbase	hsa-miR-181a-5p	DCAF16	54876	ENSG00000163257	validated
tarbase	hsa-miR-505-3p	DCAF16	54876	ENSG00000163257	validated
tarbase	hsa-miR-15b-5p	NPPC	4880	ENSG00000163273	validated
tarbase	hsa-miR-301a-3p	NPPC	4880	ENSG00000163273	validated
tarbase	hsa-miR-21-5p	ALPP	250	ENSG00000163283	validated
tarbase	hsa-miR-21-5p	PAQR3	152559	ENSG00000163291	validated
tarbase	hsa-miR-582-3p	PAQR3	152559	ENSG00000163291	validated
tarbase	hsa-miR-15b-5p	PAQR3	152559	ENSG00000163291	validated
tarbase	hsa-miR-181a-5p	PAQR3	152559	ENSG00000163291	validated
tarbase	hsa-miR-106b-5p	NIPAL1	152519	ENSG00000163293	validated
tarbase	hsa-miR-21-5p	NIPAL1	152519	ENSG00000163293	validated
tarbase	hsa-miR-192-5p	ALPI	248	ENSG00000163295	validated
tarbase	hsa-miR-106b-5p	ANTXR2	118429	ENSG00000163297	validated
tarbase	hsa-miR-582-5p	MRPS18C	51023	ENSG00000163319	validated
tarbase	hsa-miR-15b-5p	CGGBP1	8545	ENSG00000163320	validated
tarbase	hsa-miR-212-3p	CGGBP1	8545	ENSG00000163320	validated
tarbase	hsa-miR-582-5p	CGGBP1	8545	ENSG00000163320	validated
tarbase	hsa-miR-106b-5p	ABRAXAS1	84142	ENSG00000163322	validated
tarbase	hsa-miR-582-5p	ABRAXAS1	84142	ENSG00000163322	validated
tarbase	hsa-miR-212-3p	ABRAXAS1	84142	ENSG00000163322	validated
tarbase	hsa-miR-21-5p	GPR155	151556	ENSG00000163328	validated
tarbase	hsa-miR-15b-5p	GPR155	151556	ENSG00000163328	validated
tarbase	hsa-miR-106b-5p	PBXIP1	57326	ENSG00000163346	validated
tarbase	hsa-miR-15b-5p	PBXIP1	57326	ENSG00000163346	validated
tarbase	hsa-miR-296-5p	PYGO2	90780	ENSG00000163348	validated
tarbase	hsa-miR-106b-5p	HIPK1	204851	ENSG00000163349	validated
tarbase	hsa-miR-15b-5p	HIPK1	204851	ENSG00000163349	validated
tarbase	hsa-miR-181a-5p	HIPK1	204851	ENSG00000163349	validated
tarbase	hsa-miR-222-3p	HIPK1	204851	ENSG00000163349	validated
tarbase	hsa-miR-505-3p	HIPK1	204851	ENSG00000163349	validated
tarbase	hsa-miR-21-5p	COL6A3	1293	ENSG00000163359	validated
tarbase	hsa-miR-301a-3p	COL6A3	1293	ENSG00000163359	validated
tarbase	hsa-miR-212-3p	YY1AP1	55249	ENSG00000163374	validated
tarbase	hsa-miR-106b-5p	KBTBD8	84541	ENSG00000163376	validated
tarbase	hsa-miR-212-3p	KBTBD8	84541	ENSG00000163376	validated
tarbase	hsa-miR-301a-3p	KBTBD8	84541	ENSG00000163376	validated
tarbase	hsa-miR-106b-5p	EOGT	285203	ENSG00000163378	validated
tarbase	hsa-miR-326	EOGT	285203	ENSG00000163378	validated
tarbase	hsa-miR-181a-5p	SLC22A15	55356	ENSG00000163393	validated
tarbase	hsa-miR-192-5p	IGFN1	91156	ENSG00000163395	validated
tarbase	hsa-miR-192-5p	ATP1A1	476	ENSG00000163399	validated
tarbase	hsa-miR-301a-3p	ATP1A1	476	ENSG00000163399	validated
tarbase	hsa-miR-15b-5p	LRRC58	116064	ENSG00000163428	validated
tarbase	hsa-miR-181a-5p	LRRC58	116064	ENSG00000163428	validated
tarbase	hsa-miR-582-5p	LRRC58	116064	ENSG00000163428	validated
tarbase	hsa-miR-106b-5p	FSTL1	11167	ENSG00000163430	validated
tarbase	hsa-miR-15b-5p	FSTL1	11167	ENSG00000163430	validated
tarbase	hsa-miR-181a-5p	FSTL1	11167	ENSG00000163430	validated
tarbase	hsa-miR-21-5p	FSTL1	11167	ENSG00000163430	validated
tarbase	hsa-miR-326	FSTL1	11167	ENSG00000163430	validated
tarbase	hsa-miR-582-5p	FSTL1	11167	ENSG00000163430	validated
tarbase	hsa-miR-582-5p	ELF3	1999	ENSG00000163435	validated
tarbase	hsa-miR-15b-5p	TMEM183A	92703	ENSG00000163444	validated
tarbase	hsa-miR-212-3p	TMEM169	92691	ENSG00000163449	validated
tarbase	hsa-miR-181a-5p	IGFBP7	3490	ENSG00000163453	validated
tarbase	hsa-miR-326	ARPC2	10109	ENSG00000163466	validated
tarbase	hsa-miR-181a-5p	CCT3	7203	ENSG00000163468	validated
tarbase	hsa-miR-296-5p	SSR2	6746	ENSG00000163479	validated
tarbase	hsa-miR-212-3p	IHH	3549	ENSG00000163501	validated
tarbase	hsa-miR-212-3p	CIP2A	57650	ENSG00000163507	validated
tarbase	hsa-miR-181a-5p	TGFBR2	7048	ENSG00000163513	validated
tarbase	hsa-miR-192-5p	HDAC11	79885	ENSG00000163517	validated
tarbase	hsa-miR-106b-5p	STT3B	201595	ENSG00000163527	validated
tarbase	hsa-miR-181a-5p	STT3B	201595	ENSG00000163527	validated

tarbase	hsa-miR-301a-3p	STT3B	201595	ENSG00000163527	validated
tarbase	hsa-miR-212-3p	SGO2	151246	ENSG00000163535	validated
tarbase	hsa-miR-15b-5p	CLASP2	23122	ENSG00000163539	validated
tarbase	hsa-miR-181a-5p	CLASP2	23122	ENSG00000163539	validated
tarbase	hsa-miR-15b-5p	SUCLG1	8802	ENSG00000163541	validated
tarbase	hsa-miR-181a-5p	NUAK2	81788	ENSG00000163545	validated
tarbase	hsa-miR-181a-5p	MNDA	4332	ENSG00000163563	validated
tarbase	hsa-miR-212-3p	IFI16	3428	ENSG00000163565	validated
tarbase	hsa-miR-222-3p	EIF5A2	56648	ENSG00000163577	validated
tarbase	hsa-miR-106b-5p	RYBP	23429	ENSG00000163602	validated
tarbase	hsa-miR-192-5p	RYBP	23429	ENSG00000163602	validated
tarbase	hsa-miR-212-3p	RYBP	23429	ENSG00000163602	validated
tarbase	hsa-miR-21-5p	RYBP	23429	ENSG00000163602	validated
tarbase	hsa-miR-106b-5p	PPP4R2	151987	ENSG00000163605	validated
tarbase	hsa-miR-15b-5p	PPP4R2	151987	ENSG00000163605	validated
tarbase	hsa-miR-181a-5p	PPP4R2	151987	ENSG00000163605	validated
tarbase	hsa-miR-222-3p	PPP4R2	151987	ENSG00000163605	validated
tarbase	hsa-miR-106b-5p	CD200R1	131450	ENSG00000163606	validated
tarbase	hsa-miR-181a-5p	CD200R1	131450	ENSG00000163606	validated
tarbase	hsa-miR-582-5p	CD200R1	131450	ENSG00000163606	validated
tarbase	hsa-miR-15b-5p	CDS1	1040	ENSG00000163624	validated
tarbase	hsa-miR-21-5p	CDS1	1040	ENSG00000163624	validated
tarbase	hsa-miR-301a-3p	CDS1	1040	ENSG00000163624	validated
tarbase	hsa-miR-106b-5p	WDFY3	23001	ENSG00000163625	validated
tarbase	hsa-miR-144-3p	WDFY3	23001	ENSG00000163625	validated
tarbase	hsa-miR-15b-5p	WDFY3	23001	ENSG00000163625	validated
tarbase	hsa-miR-582-3p	WDFY3	23001	ENSG00000163625	validated
tarbase	hsa-miR-181a-5p	PTPN13	5783	ENSG00000163629	validated
tarbase	hsa-miR-18a-5p	PTPN13	5783	ENSG00000163629	validated
tarbase	hsa-miR-21-5p	PTPN13	5783	ENSG00000163629	validated
tarbase	hsa-miR-582-5p	PTPN13	5783	ENSG00000163629	validated
tarbase	hsa-miR-106b-5p	THOC7	80145	ENSG00000163634	validated
tarbase	hsa-miR-18a-5p	ATXN7	6314	ENSG00000163635	validated
tarbase	hsa-miR-144-3p	PSMD6	9861	ENSG00000163636	validated
tarbase	hsa-miR-181a-5p	PRICKLE2	166336	ENSG00000163637	validated
tarbase	hsa-miR-181a-5p	ADAMTS9	56999	ENSG00000163638	validated
tarbase	hsa-miR-582-5p	ADAMTS9	56999	ENSG00000163638	validated
tarbase	hsa-miR-15b-5p	PPM1K	152926	ENSG00000163644	validated
tarbase	hsa-miR-582-5p	PPM1K	152926	ENSG00000163644	validated
tarbase	hsa-miR-18a-5p	GMPS	8833	ENSG00000163655	validated
tarbase	hsa-miR-181a-5p	GMPS	8833	ENSG00000163655	validated
tarbase	hsa-miR-106b-5p	TIPARP	25976	ENSG00000163659	validated
tarbase	hsa-miR-18a-5p	TIPARP	25976	ENSG00000163659	validated
tarbase	hsa-miR-21-5p	TIPARP	25976	ENSG00000163659	validated
tarbase	hsa-miR-212-3p	TIPARP	25976	ENSG00000163659	validated
tarbase	hsa-miR-181a-5p	SLMAP	7871	ENSG00000163681	validated
tarbase	hsa-miR-301a-3p	SLMAP	7871	ENSG00000163681	validated
tarbase	hsa-miR-181a-5p	SMIM14	201895	ENSG00000163683	validated
tarbase	hsa-miR-106b-5p	RPP14	11102	ENSG00000163684	validated
tarbase	hsa-miR-15b-5p	C3orf67	200844	ENSG00000163689	validated
tarbase	hsa-miR-106b-5p	RBM47	54502	ENSG00000163694	validated
tarbase	hsa-miR-212-3p	CRELD1	78987	ENSG00000163703	validated
tarbase	hsa-miR-21-5p	U2SURP	23350	ENSG00000163714	validated
tarbase	hsa-miR-15b-5p	TTC14	151613	ENSG00000163728	validated
tarbase	hsa-miR-21-5p	CXCL3	2921	ENSG00000163734	validated
tarbase	hsa-miR-15b-5p	CXCL5	6374	ENSG00000163735	validated
tarbase	hsa-miR-301a-3p	CXCL5	6374	ENSG00000163735	validated
tarbase	hsa-miR-18a-5p	MTHFD2L	441024	ENSG00000163738	validated
tarbase	hsa-miR-106b-5p	MTHFD2L	441024	ENSG00000163738	validated
tarbase	hsa-miR-212-3p	CXCL1	2919	ENSG00000163739	validated
tarbase	hsa-miR-212-3p	RCHY1	25898	ENSG00000163743	validated
tarbase	hsa-miR-222-3p	RCHY1	25898	ENSG00000163743	validated
tarbase	hsa-miR-212-3p	PLSCR2	57047	ENSG00000163746	validated
tarbase	hsa-miR-106b-5p	HPS3	84343	ENSG00000163755	validated
tarbase	hsa-miR-15b-5p	HPS3	84343	ENSG00000163755	validated
tarbase	hsa-miR-21-5p	TM4SF18	116441	ENSG00000163762	validated
tarbase	hsa-miR-15b-5p	SNRK	54861	ENSG00000163788	validated
tarbase	hsa-miR-18a-5p	SNRK	54861	ENSG00000163788	validated
tarbase	hsa-miR-106b-5p	SLC4A1AP	22950	ENSG00000163798	validated
tarbase	hsa-miR-18a-5p	SLC4A1AP	22950	ENSG00000163798	validated
tarbase	hsa-miR-15b-5p	WDR43	23160	ENSG00000163811	validated

tarbase	hsa-miR-181a-5p	ZDHHC3	51304	ENSG00000163812	validated
tarbase	hsa-miR-106b-5p	CDCP1	64866	ENSG00000163814	validated
tarbase	hsa-miR-15b-5p	FYCO1	79443	ENSG00000163820	validated
tarbase	hsa-miR-296-5p	FYCO1	79443	ENSG00000163820	validated
tarbase	hsa-miR-301a-3p	FYCO1	79443	ENSG00000163820	validated
tarbase	hsa-miR-582-5p	FYCO1	79443	ENSG00000163820	validated
tarbase	hsa-miR-212-3p	DTX3L	151636	ENSG00000163840	validated
tarbase	hsa-miR-106b-5p	ZNF148	7707	ENSG00000163848	validated
tarbase	hsa-miR-18a-5p	ZNF148	7707	ENSG00000163848	validated
tarbase	hsa-miR-21-5p	ZNF148	7707	ENSG00000163848	validated
tarbase	hsa-miR-181a-5p	ZMYM6	9204	ENSG00000163867	validated
tarbase	hsa-miR-212-3p	ZMYM6	9204	ENSG00000163867	validated
tarbase	hsa-miR-296-5p	YEATS2	55689	ENSG00000163872	validated
tarbase	hsa-miR-181a-5p	SNIP1	79753	ENSG00000163877	validated
tarbase	hsa-miR-18a-5p	SNIP1	79753	ENSG00000163877	validated
tarbase	hsa-miR-21-5p	SNIP1	79753	ENSG00000163877	validated
tarbase	hsa-miR-106b-5p	POLR2H	5437	ENSG00000163882	validated
tarbase	hsa-miR-15b-5p	POLR2H	5437	ENSG00000163882	validated
tarbase	hsa-miR-181a-5p	KLF15	28999	ENSG00000163884	validated
tarbase	hsa-miR-181a-5p	TMEM41A	90407	ENSG00000163900	validated
tarbase	hsa-miR-212-3p	TMEM41A	90407	ENSG00000163900	validated
tarbase	hsa-miR-21-5p	RPN1	6184	ENSG00000163902	validated
tarbase	hsa-miR-301a-3p	RPN1	6184	ENSG00000163902	validated
tarbase	hsa-miR-18a-5p	BAP1	8314	ENSG00000163930	validated
tarbase	hsa-miR-505-3p	BAP1	8314	ENSG00000163930	validated
tarbase	hsa-miR-18a-5p	PRKCD	5580	ENSG00000163932	validated
tarbase	hsa-miR-15b-5p	RFT1	91869	ENSG00000163933	validated
tarbase	hsa-miR-106b-5p	SFMBT1	51460	ENSG00000163935	validated
tarbase	hsa-miR-326	SFMBT1	51460	ENSG00000163935	validated
tarbase	hsa-miR-582-5p	SFMBT1	51460	ENSG00000163935	validated
tarbase	hsa-miR-18a-5p	PBRM1	55193	ENSG00000163939	validated
tarbase	hsa-miR-18a-5p	UVSSA	57654	ENSG00000163945	validated
tarbase	hsa-miR-15b-5p	TASOR	23272	ENSG00000163946	validated
tarbase	hsa-miR-18a-5p	TASOR	23272	ENSG00000163946	validated
tarbase	hsa-miR-301a-3p	TASOR	23272	ENSG00000163946	validated
tarbase	hsa-miR-106b-5p	ARHGEF3	50650	ENSG00000163947	validated
tarbase	hsa-miR-181a-5p	ARHGEF3	50650	ENSG00000163947	validated
tarbase	hsa-miR-212-3p	SLBP	7884	ENSG00000163950	validated
tarbase	hsa-miR-582-3p	SLBP	7884	ENSG00000163950	validated
tarbase	hsa-miR-181a-5p	SLBP	7884	ENSG00000163950	validated
tarbase	hsa-miR-505-3p	LRPAP1	4043	ENSG00000163956	validated
tarbase	hsa-miR-106b-5p	UBXN7	26043	ENSG00000163960	validated
tarbase	hsa-miR-181a-5p	UBXN7	26043	ENSG00000163960	validated
tarbase	hsa-miR-21-5p	RNF168	165918	ENSG00000163961	validated
tarbase	hsa-miR-106b-5p	S100P	6286	ENSG00000163993	validated
tarbase	hsa-miR-181a-5p	SGMS2	166929	ENSG00000164023	validated
tarbase	hsa-miR-15b-5p	DNAJB14	79982	ENSG00000164031	validated
tarbase	hsa-miR-181a-5p	DNAJB14	79982	ENSG00000164031	validated
tarbase	hsa-miR-582-5p	H2AZ1	3015	ENSG00000164032	validated
tarbase	hsa-miR-212-3p	BDH2	56898	ENSG00000164039	validated
tarbase	hsa-miR-144-3p	PGRMC2	10424	ENSG00000164040	validated
tarbase	hsa-miR-106b-5p	CDC25A	993	ENSG00000164045	validated
tarbase	hsa-miR-15b-5p	SHISA5	51246	ENSG00000164054	validated
tarbase	hsa-miR-18a-5p	SHISA5	51246	ENSG00000164054	validated
tarbase	hsa-miR-15b-5p	SPRY1	10252	ENSG00000164056	validated
tarbase	hsa-miR-18a-5p	BSN	8927	ENSG00000164061	validated
tarbase	hsa-miR-212-3p	BSN	8927	ENSG00000164061	validated
tarbase	hsa-miR-212-3p	INTU	27152	ENSG00000164066	validated
tarbase	hsa-miR-582-5p	HSPA4L	22824	ENSG00000164070	validated
tarbase	hsa-miR-181a-5p	HSPA4L	22824	ENSG00000164070	validated
tarbase	hsa-miR-181a-5p	MFSD8	256471	ENSG00000164073	validated
tarbase	hsa-miR-21-5p	RAD54L2	23132	ENSG00000164080	validated
tarbase	hsa-miR-301a-3p	RAD54L2	23132	ENSG00000164080	validated
tarbase	hsa-miR-326	DUSP7	1849	ENSG00000164086	validated
tarbase	hsa-miR-106b-5p	WDR82	80335	ENSG00000164091	validated
tarbase	hsa-miR-15b-5p	WDR82	80335	ENSG00000164091	validated
tarbase	hsa-miR-181a-5p	WDR82	80335	ENSG00000164091	validated
tarbase	hsa-miR-192-5p	WDR82	80335	ENSG00000164091	validated
tarbase	hsa-miR-222-3p	WDR82	80335	ENSG00000164091	validated
tarbase	hsa-miR-301a-3p	WDR82	80335	ENSG00000164091	validated
tarbase	hsa-miR-21-5p	PITX2	5308	ENSG00000164093	validated

tarbase	hsa-miR-212-3p	PITX2	5308	ENSG00000164093	validated
tarbase	hsa-miR-18a-5p	PRSS12	8492	ENSG00000164099	validated
tarbase	hsa-miR-21-5p	HMGB2	3148	ENSG00000164104	validated
tarbase	hsa-miR-222-3p	HMGB2	3148	ENSG00000164104	validated
tarbase	hsa-miR-15b-5p	ANXA5	308	ENSG00000164111	validated
tarbase	hsa-miR-212-3p	TMEM155	132332	ENSG00000164112	validated
tarbase	hsa-miR-181a-5p	MAP9	79884	ENSG00000164114	validated
tarbase	hsa-miR-18a-5p	GUCY1A1	2982	ENSG00000164116	validated
tarbase	hsa-miR-301a-3p	GUCY1A1	2982	ENSG00000164116	validated
tarbase	hsa-miR-106b-5p	GUCY1A1	2982	ENSG00000164116	validated
tarbase	hsa-miR-18a-5p	CEP44	80817	ENSG00000164118	validated
tarbase	hsa-miR-15b-5p	TMEM144	55314	ENSG00000164124	validated
tarbase	hsa-miR-181a-5p	GASK1B	51313	ENSG00000164125	validated
tarbase	hsa-miR-301a-3p	NPY1R	4886	ENSG00000164128	validated
tarbase	hsa-miR-106b-5p	NAA15	80155	ENSG00000164134	validated
tarbase	hsa-miR-212-3p	IL15	3600	ENSG00000164136	validated
tarbase	hsa-miR-15b-5p	FAM160A1	729830	ENSG00000164142	validated
tarbase	hsa-miR-18a-5p	FAM160A1	729830	ENSG00000164142	validated
tarbase	hsa-miR-181a-5p	ARFIP1	27236	ENSG00000164144	validated
tarbase	hsa-miR-212-3p	ANAPC10	10393	ENSG00000164162	validated
tarbase	hsa-miR-301a-3p	ABCE1	6059	ENSG00000164163	validated
tarbase	hsa-miR-144-3p	OTUD4	54726	ENSG00000164164	validated
tarbase	hsa-miR-15b-5p	OTUD4	54726	ENSG00000164164	validated
tarbase	hsa-miR-181a-5p	OTUD4	54726	ENSG00000164164	validated
tarbase	hsa-miR-18a-5p	OTUD4	54726	ENSG00000164164	validated
tarbase	hsa-miR-301a-3p	OTUD4	54726	ENSG00000164164	validated
tarbase	hsa-miR-181a-5p	TMEM184C	55751	ENSG00000164168	validated
tarbase	hsa-miR-181a-5p	ITGA2	3673	ENSG00000164171	validated
tarbase	hsa-miR-326	ITGA2	3673	ENSG00000164171	validated
tarbase	hsa-miR-582-5p	ITGA2	3673	ENSG00000164171	validated
tarbase	hsa-miR-582-3p	EDIL3	10085	ENSG00000164176	validated
tarbase	hsa-miR-15b-5p	ELOVL7	79993	ENSG00000164181	validated
tarbase	hsa-miR-181a-5p	LMBRD2	92255	ENSG00000164187	validated
tarbase	hsa-miR-106b-5p	NIPBL	25836	ENSG00000164190	validated
tarbase	hsa-miR-15b-5p	NIPBL	25836	ENSG00000164190	validated
tarbase	hsa-miR-181a-5p	NIPBL	25836	ENSG00000164190	validated
tarbase	hsa-miR-18a-5p	NIPBL	25836	ENSG00000164190	validated
tarbase	hsa-miR-192-5p	NIPBL	25836	ENSG00000164190	validated
tarbase	hsa-miR-582-5p	STARD4	134429	ENSG00000164211	validated
tarbase	hsa-miR-212-3p	ANKRD33B	651746	ENSG00000164236	validated
tarbase	hsa-miR-21-5p	CMBL	134147	ENSG00000164237	validated
tarbase	hsa-miR-222-3p	CMBL	134147	ENSG00000164237	validated
tarbase	hsa-miR-106b-5p	PRRC1	133619	ENSG00000164244	validated
tarbase	hsa-miR-15b-5p	PRRC1	133619	ENSG00000164244	validated
tarbase	hsa-miR-18a-5p	PRRC1	133619	ENSG00000164244	validated
tarbase	hsa-miR-301a-3p	PRRC1	133619	ENSG00000164244	validated
tarbase	hsa-miR-181a-5p	PRRC1	133619	ENSG00000164244	validated
tarbase	hsa-miR-181a-5p	AGGF1	55109	ENSG00000164252	validated
tarbase	hsa-miR-212-3p	WDR41	55255	ENSG00000164253	validated
tarbase	hsa-miR-21-5p	RHOBTB3	22836	ENSG00000164292	validated
tarbase	hsa-miR-15b-5p	RHOBTB3	22836	ENSG00000164292	validated
tarbase	hsa-miR-212-3p	TIGD6	81789	ENSG00000164296	validated
tarbase	hsa-miR-15b-5p	SERINC5	256987	ENSG00000164300	validated
tarbase	hsa-miR-144-3p	CASP3	836	ENSG00000164305	validated
tarbase	hsa-miR-222-3p	CASP3	836	ENSG00000164305	validated
tarbase	hsa-miR-212-3p	ERAP2	64167	ENSG00000164308	validated
tarbase	hsa-miR-21-5p	CFAP97	57587	ENSG00000164323	validated
tarbase	hsa-miR-18a-5p	CFAP97	57587	ENSG00000164323	validated
tarbase	hsa-miR-144-3p	RICTOR	253260	ENSG00000164327	validated
tarbase	hsa-miR-15b-5p	RICTOR	253260	ENSG00000164327	validated
tarbase	hsa-miR-181a-5p	RICTOR	253260	ENSG00000164327	validated
tarbase	hsa-miR-192-5p	RICTOR	253260	ENSG00000164327	validated
tarbase	hsa-miR-212-3p	RICTOR	253260	ENSG00000164327	validated
tarbase	hsa-miR-582-3p	RICTOR	253260	ENSG00000164327	validated
tarbase	hsa-miR-106b-5p	TENT2	167153	ENSG00000164329	validated
tarbase	hsa-miR-18a-5p	TENT2	167153	ENSG00000164329	validated
tarbase	hsa-miR-21-5p	TENT2	167153	ENSG00000164329	validated
tarbase	hsa-miR-106b-5p	ANKRA2	57763	ENSG00000164331	validated
tarbase	hsa-miR-15b-5p	UBLCP1	134510	ENSG00000164332	validated
tarbase	hsa-miR-106b-5p	UTP15	84135	ENSG00000164338	validated
tarbase	hsa-miR-15b-5p	UTP15	84135	ENSG00000164338	validated

tarbase	hsa-miR-212-3p	UTP15	84135	ENSG00000164338	validated
tarbase	hsa-miR-212-3p	TLR3	7098	ENSG00000164342	validated
tarbase	hsa-miR-15b-5p	CCDC127	133957	ENSG00000164366	validated
tarbase	hsa-miR-21-5p	CCDC127	133957	ENSG00000164366	validated
tarbase	hsa-miR-21-5p	ACSL6	23305	ENSG00000164398	validated
tarbase	hsa-miR-15b-5p	ACSL6	23305	ENSG00000164398	validated
tarbase	hsa-miR-181a-5p	ACSL6	23305	ENSG00000164398	validated
tarbase	hsa-miR-181a-5p	SEPTIN8	23176	ENSG00000164402	validated
tarbase	hsa-miR-212-3p	GDF9	2661	ENSG00000164404	validated
tarbase	hsa-miR-181a-5p	UQCRQ	27089	ENSG00000164405	validated
tarbase	hsa-miR-301a-3p	UQCRQ	27089	ENSG00000164405	validated
tarbase	hsa-miR-212-3p	LEAP2	116842	ENSG00000164406	validated
tarbase	hsa-miR-106b-5p	SLC35A1	10559	ENSG00000164414	validated
tarbase	hsa-miR-212-3p	SLC35A1	10559	ENSG00000164414	validated
tarbase	hsa-miR-106b-5p	CITED2	10370	ENSG00000164442	validated
tarbase	hsa-miR-181a-5p	CITED2	10370	ENSG00000164442	validated
tarbase	hsa-miR-106b-5p	CREBRF	153222	ENSG00000164463	validated
tarbase	hsa-miR-144-3p	CREBRF	153222	ENSG00000164463	validated
tarbase	hsa-miR-181a-5p	CREBRF	153222	ENSG00000164463	validated
tarbase	hsa-miR-192-5p	CREBRF	153222	ENSG00000164463	validated
tarbase	hsa-miR-21-5p	CREBRF	153222	ENSG00000164463	validated
tarbase	hsa-miR-15b-5p	DCBLD1	285761	ENSG00000164465	validated
tarbase	hsa-miR-15b-5p	SFXN1	94081	ENSG00000164466	validated
tarbase	hsa-miR-181a-5p	SFXN1	94081	ENSG00000164466	validated
tarbase	hsa-miR-222-3p	SFXN1	94081	ENSG00000164466	validated
tarbase	hsa-miR-301a-3p	SFXN1	94081	ENSG00000164466	validated
tarbase	hsa-miR-505-3p	SFXN1	94081	ENSG00000164466	validated
tarbase	hsa-miR-212-3p	DACT2	168002	ENSG00000164488	validated
tarbase	hsa-miR-18a-5p	PDSS2	57107	ENSG00000164494	validated
tarbase	hsa-miR-212-3p	TBX20	57057	ENSG00000164532	validated
tarbase	hsa-miR-106b-5p	KIAA0895	23366	ENSG00000164542	validated
tarbase	hsa-miR-144-3p	KIAA0895	23366	ENSG00000164542	validated
tarbase	hsa-miR-18a-5p	KIAA0895	23366	ENSG00000164542	validated
tarbase	hsa-miR-326	KIAA0895	23366	ENSG00000164542	validated
tarbase	hsa-miR-582-3p	KIAA0895	23366	ENSG00000164542	validated
tarbase	hsa-miR-212-3p	KIAA0895	23366	ENSG00000164542	validated
tarbase	hsa-miR-18a-5p	STK17A	9263	ENSG00000164543	validated
tarbase	hsa-miR-106b-5p	GALNT10	55568	ENSG00000164574	validated
tarbase	hsa-miR-15b-5p	RPS14	6208	ENSG00000164587	validated
tarbase	hsa-miR-181a-5p	RPS14	6208	ENSG00000164587	validated
tarbase	hsa-miR-222-3p	RPS14	6208	ENSG00000164587	validated
tarbase	hsa-miR-106b-5p	COG5	10466	ENSG00000164597	validated
tarbase	hsa-miR-21-5p	COG5	10466	ENSG00000164597	validated
tarbase	hsa-miR-212-3p	COG5	10466	ENSG00000164597	validated
tarbase	hsa-miR-18a-5p	BMT2	154743	ENSG00000164603	validated
tarbase	hsa-miR-301a-3p	BMT2	154743	ENSG00000164603	validated
tarbase	hsa-miR-15b-5p	KCNK5	8645	ENSG00000164626	validated
tarbase	hsa-miR-212-3p	KIF6	221458	ENSG00000164627	validated
tarbase	hsa-miR-18a-5p	ZNF12	7559	ENSG00000164631	validated
tarbase	hsa-miR-212-3p	ZNF12	7559	ENSG00000164631	validated
tarbase	hsa-miR-21-5p	ZNF12	7559	ENSG00000164631	validated
tarbase	hsa-miR-582-5p	ZNF12	7559	ENSG00000164631	validated
tarbase	hsa-miR-106b-5p	MIOS	54468	ENSG00000164654	validated
tarbase	hsa-miR-181a-5p	MIOS	54468	ENSG00000164654	validated
tarbase	hsa-miR-301a-3p	MIOS	54468	ENSG00000164654	validated
tarbase	hsa-miR-212-3p	IQUB	154865	ENSG00000164675	validated
tarbase	hsa-miR-106b-5p	ZNF704	619279	ENSG00000164684	validated
tarbase	hsa-miR-181a-5p	ZNF704	619279	ENSG00000164684	validated
tarbase	hsa-miR-106b-5p	TAGAP	117289	ENSG00000164691	validated
tarbase	hsa-miR-192-5p	COL1A2	1278	ENSG00000164692	validated
tarbase	hsa-miR-212-3p	FNDC1	84624	ENSG00000164694	validated
tarbase	hsa-miR-106b-5p	LMTK2	22853	ENSG00000164715	validated
tarbase	hsa-miR-301a-3p	LMTK2	22853	ENSG00000164715	validated
tarbase	hsa-miR-181a-5p	LMTK2	22853	ENSG00000164715	validated
tarbase	hsa-miR-15b-5p	DLC1	10395	ENSG00000164741	validated
tarbase	hsa-miR-192-5p	DLC1	10395	ENSG00000164741	validated
tarbase	hsa-miR-296-5p	ADCY1	107	ENSG00000164742	validated
tarbase	hsa-miR-181a-5p	ADCY1	107	ENSG00000164742	validated
tarbase	hsa-miR-106b-5p	RAD21	5885	ENSG00000164754	validated
tarbase	hsa-miR-212-3p	RAD21	5885	ENSG00000164754	validated
tarbase	hsa-miR-301a-3p	RAD21	5885	ENSG00000164754	validated

tarbase	hsa-miR-582-5p	RAD21	5885	ENSG00000164754	validated
tarbase	hsa-miR-181a-5p	RAD21	5885	ENSG00000164754	validated
tarbase	hsa-miR-15b-5p	SLC30A8	169026	ENSG00000164756	validated
tarbase	hsa-miR-296-5p	SBSPON	157869	ENSG00000164764	validated
tarbase	hsa-miR-296-5p	EN2	2020	ENSG00000164778	validated
tarbase	hsa-miR-18a-5p	ORC5	5001	ENSG00000164815	validated
tarbase	hsa-miR-212-3p	ORC5	5001	ENSG00000164815	validated
tarbase	hsa-miR-15b-5p	DNAAF5	54919	ENSG00000164818	validated
tarbase	hsa-miR-181a-5p	DNAAF5	54919	ENSG00000164818	validated
tarbase	hsa-miR-181a-5p	OSGIN2	734	ENSG00000164823	validated
tarbase	hsa-miR-21-5p	SUN1	23353	ENSG00000164828	validated
tarbase	hsa-miR-18a-5p	OXR1	55074	ENSG00000164830	validated
tarbase	hsa-miR-222-3p	OXR1	55074	ENSG00000164830	validated
tarbase	hsa-miR-296-5p	INTS1	26173	ENSG00000164880	validated
tarbase	hsa-miR-15b-5p	SLC4A2	6522	ENSG00000164889	validated
tarbase	hsa-miR-15b-5p	FASTK	10922	ENSG00000164896	validated
tarbase	hsa-miR-301a-3p	FASTK	10922	ENSG00000164896	validated
tarbase	hsa-miR-106b-5p	FASTK	10922	ENSG00000164896	validated
tarbase	hsa-miR-15b-5p	TMUB1	83590	ENSG00000164897	validated
tarbase	hsa-miR-15b-5p	PHAX	51808	ENSG00000164902	validated
tarbase	hsa-miR-181a-5p	FOXK1	221937	ENSG00000164916	validated
tarbase	hsa-miR-21-5p	FOXK1	221937	ENSG00000164916	validated
tarbase	hsa-miR-582-5p	FOXK1	221937	ENSG00000164916	validated
tarbase	hsa-miR-106b-5p	FZD6	8323	ENSG00000164930	validated
tarbase	hsa-miR-582-3p	FZD6	8323	ENSG00000164930	validated
tarbase	hsa-miR-181a-5p	FZD6	8323	ENSG00000164930	validated
tarbase	hsa-miR-181a-5p	SLC25A32	81034	ENSG00000164933	validated
tarbase	hsa-miR-301a-3p	DCAF13	25879	ENSG00000164934	validated
tarbase	hsa-miR-144-3p	TP53INP1	94241	ENSG00000164938	validated
tarbase	hsa-miR-15b-5p	TP53INP1	94241	ENSG00000164938	validated
tarbase	hsa-miR-192-5p	TP53INP1	94241	ENSG00000164938	validated
tarbase	hsa-miR-222-3p	TP53INP1	94241	ENSG00000164938	validated
tarbase	hsa-miR-301a-3p	TP53INP1	94241	ENSG00000164938	validated
tarbase	hsa-miR-582-5p	TP53INP1	94241	ENSG00000164938	validated
tarbase	hsa-miR-21-5p	GEM	2669	ENSG00000164949	validated
tarbase	hsa-miR-15b-5p	PDP1	54704	ENSG00000164951	validated
tarbase	hsa-miR-192-5p	TMEM67	91147	ENSG00000164953	validated
tarbase	hsa-miR-15b-5p	MYORG	57462	ENSG00000164976	validated
tarbase	hsa-miR-222-3p	PSIP1	11168	ENSG00000164985	validated
tarbase	hsa-miR-181a-5p	PSIP1	11168	ENSG00000164985	validated
tarbase	hsa-miR-212-3p	CCDC171	203238	ENSG00000164989	validated
tarbase	hsa-miR-106b-5p	UBAP1	51271	ENSG00000165006	validated
tarbase	hsa-miR-15b-5p	UBAP1	51271	ENSG00000165006	validated
tarbase	hsa-miR-18a-5p	UBAP1	51271	ENSG00000165006	validated
tarbase	hsa-miR-192-5p	UBAP1	51271	ENSG00000165006	validated
tarbase	hsa-miR-296-5p	UBAP1	51271	ENSG00000165006	validated
tarbase	hsa-miR-181a-5p	UBAP1	51271	ENSG00000165006	validated
tarbase	hsa-miR-15b-5p	DIRAS2	54769	ENSG00000165023	validated
tarbase	hsa-miR-106b-5p	NFIL3	4783	ENSG00000165030	validated
tarbase	hsa-miR-222-3p	METTL2B	55798	ENSG00000165055	validated
tarbase	hsa-miR-21-5p	ALDH1A1	216	ENSG00000165092	validated
tarbase	hsa-miR-222-3p	ALDH1A1	216	ENSG00000165092	validated
tarbase	hsa-miR-15b-5p	HGSNAT	138050	ENSG00000165102	validated
tarbase	hsa-miR-21-5p	HGSNAT	138050	ENSG00000165102	validated
tarbase	hsa-miR-222-3p	RASEF	158158	ENSG00000165105	validated
tarbase	hsa-miR-106b-5p	GKAP1	80318	ENSG00000165113	validated
tarbase	hsa-miR-301a-3p	HNRNPK	3190	ENSG00000165119	validated
tarbase	hsa-miR-15b-5p	ANKS6	203286	ENSG00000165138	validated
tarbase	hsa-miR-21-5p	PGAP4	84302	ENSG00000165152	validated
tarbase	hsa-miR-15b-5p	ZHX1	11244	ENSG00000165156	validated
tarbase	hsa-miR-181a-5p	ZHX1	11244	ENSG00000165156	validated
tarbase	hsa-miR-582-5p	DYNLT3	6990	ENSG00000165169	validated
tarbase	hsa-miR-15b-5p	DYNLT3	6990	ENSG00000165169	validated
tarbase	hsa-miR-181a-5p	DYNLT3	6990	ENSG00000165169	validated
tarbase	hsa-miR-505-3p	KIAA1958	158405	ENSG00000165185	validated
tarbase	hsa-miR-212-3p	RNF183	138065	ENSG00000165188	validated
tarbase	hsa-miR-181a-5p	STRBP	55342	ENSG00000165209	validated
tarbase	hsa-miR-582-3p	STRBP	55342	ENSG00000165209	validated
tarbase	hsa-miR-106b-5p	GAPVD1	26130	ENSG00000165219	validated
tarbase	hsa-miR-181a-5p	GAPVD1	26130	ENSG00000165219	validated
tarbase	hsa-miR-222-3p	ATP7A	538	ENSG00000165240	validated

tarbase	hsa-miR-106b-5p	ZNF367	195828	ENSG00000165244	validated
tarbase	hsa-miR-144-3p	ZNF367	195828	ENSG00000165244	validated
tarbase	hsa-miR-181a-5p	ZNF367	195828	ENSG00000165244	validated
tarbase	hsa-miR-222-3p	ZNF367	195828	ENSG00000165244	validated
tarbase	hsa-miR-582-5p	ZNF367	195828	ENSG00000165244	validated
tarbase	hsa-miR-15b-5p	VCP	7415	ENSG00000165280	validated
tarbase	hsa-miR-301a-3p	VCP	7415	ENSG00000165280	validated
tarbase	hsa-miR-15b-5p	PIGO	84720	ENSG00000165282	validated
tarbase	hsa-miR-21-5p	PIGO	84720	ENSG00000165282	validated
tarbase	hsa-miR-212-3p	SLITRK5	26050	ENSG00000165300	validated
tarbase	hsa-miR-106b-5p	OTUD1	220213	ENSG00000165312	validated
tarbase	hsa-miR-18a-5p	OTUD1	220213	ENSG00000165312	validated
tarbase	hsa-miR-18a-5p	ARHGAP12	94134	ENSG00000165322	validated
tarbase	hsa-miR-582-5p	ARHGAP12	94134	ENSG00000165322	validated
tarbase	hsa-miR-212-3p	ARHGAP12	94134	ENSG00000165322	validated
tarbase	hsa-miR-21-5p	FAT3	120114	ENSG00000165323	validated
tarbase	hsa-miR-301a-3p	FAT3	120114	ENSG00000165323	validated
tarbase	hsa-miR-15b-5p	FAT3	120114	ENSG00000165323	validated
tarbase	hsa-miR-106b-5p	HECTD2	143279	ENSG00000165338	validated
tarbase	hsa-miR-181a-5p	HECTD2	143279	ENSG00000165338	validated
tarbase	hsa-miR-222-3p	HECTD2	143279	ENSG00000165338	validated
tarbase	hsa-miR-301a-3p	SPTSSA	171546	ENSG00000165389	validated
tarbase	hsa-miR-301a-3p	MARCHF8	220972	ENSG00000165406	validated
tarbase	hsa-miR-106b-5p	MARCHF8	220972	ENSG00000165406	validated
tarbase	hsa-miR-18a-5p	CFL2	1073	ENSG00000165410	validated
tarbase	hsa-miR-15b-5p	SUGT1	10910	ENSG00000165416	validated
tarbase	hsa-miR-181a-5p	SUGT1	10910	ENSG00000165416	validated
tarbase	hsa-miR-21-5p	SUGT1	10910	ENSG00000165416	validated
tarbase	hsa-miR-582-5p	SUGT1	10910	ENSG00000165416	validated
tarbase	hsa-miR-106b-5p	GTF2A1	2957	ENSG00000165417	validated
tarbase	hsa-miR-144-3p	GTF2A1	2957	ENSG00000165417	validated
tarbase	hsa-miR-181a-5p	GTF2A1	2957	ENSG00000165417	validated
tarbase	hsa-miR-18a-5p	GTF2A1	2957	ENSG00000165417	validated
tarbase	hsa-miR-212-3p	GTF2A1	2957	ENSG00000165417	validated
tarbase	hsa-miR-582-3p	GTF2A1	2957	ENSG00000165417	validated
tarbase	hsa-miR-222-3p	ZCCHC24	219654	ENSG00000165424	validated
tarbase	hsa-miR-106b-5p	ZCCHC24	219654	ENSG00000165424	validated
tarbase	hsa-miR-301a-3p	PGM2L1	283209	ENSG00000165434	validated
tarbase	hsa-miR-15b-5p	PGM2L1	283209	ENSG00000165434	validated
tarbase	hsa-miR-181a-5p	PGM2L1	283209	ENSG00000165434	validated
tarbase	hsa-miR-15b-5p	SLC16A9	220963	ENSG00000165449	validated
tarbase	hsa-miR-212-3p	SLC16A9	220963	ENSG00000165449	validated
tarbase	hsa-miR-296-5p	CRYL1	51084	ENSG00000165475	validated
tarbase	hsa-miR-212-3p	REEP3	221035	ENSG00000165476	validated
tarbase	hsa-miR-181a-5p	SKA3	221150	ENSG00000165480	validated
tarbase	hsa-miR-301a-3p	SKA3	221150	ENSG00000165480	validated
tarbase	hsa-miR-106b-5p	DDIAS	220042	ENSG00000165490	validated
tarbase	hsa-miR-301a-3p	DDIAS	220042	ENSG00000165490	validated
tarbase	hsa-miR-18a-5p	PCF11	51585	ENSG00000165494	validated
tarbase	hsa-miR-106b-5p	LRR1	122769	ENSG00000165501	validated
tarbase	hsa-miR-296-5p	DEPP1	11067	ENSG00000165507	validated
tarbase	hsa-miR-181a-5p	ZNF22	7570	ENSG00000165512	validated
tarbase	hsa-miR-582-5p	KLHDC2	23588	ENSG00000165516	validated
tarbase	hsa-miR-212-3p	EML5	161436	ENSG00000165521	validated
tarbase	hsa-miR-15b-5p	NEMF	9147	ENSG00000165525	validated
tarbase	hsa-miR-15b-5p	RPUSD4	84881	ENSG00000165526	validated
tarbase	hsa-miR-106b-5p	ARF6	382	ENSG00000165527	validated
tarbase	hsa-miR-181a-5p	TTC8	123016	ENSG00000165533	validated
tarbase	hsa-miR-181a-5p	AMER2	219287	ENSG00000165566	validated
tarbase	hsa-miR-582-5p	KBTBD6	89890	ENSG00000165572	validated
tarbase	hsa-miR-212-3p	OXGR1	27199	ENSG00000165621	validated
tarbase	hsa-miR-222-3p	TAF3	83860	ENSG00000165632	validated
tarbase	hsa-miR-582-5p	TAF3	83860	ENSG00000165632	validated
tarbase	hsa-miR-181a-5p	TAF3	83860	ENSG00000165632	validated
tarbase	hsa-miR-212-3p	COMTD1	118881	ENSG00000165644	validated
tarbase	hsa-miR-15b-5p	SLC18A2	6571	ENSG00000165646	validated
tarbase	hsa-miR-212-3p	SLC18A2	6571	ENSG00000165646	validated
tarbase	hsa-miR-15b-5p	PDZD8	118987	ENSG00000165650	validated
tarbase	hsa-miR-21-5p	PDZD8	118987	ENSG00000165650	validated
tarbase	hsa-miR-296-5p	PDZD8	118987	ENSG00000165650	validated
tarbase	hsa-miR-301a-3p	PDZD8	118987	ENSG00000165650	validated

tarbase	hsa-miR-582-3p	PDZD8	118987	ENSG00000165650	validated
tarbase	hsa-miR-181a-5p	ABRAXAS2	23172	ENSG00000165660	validated
tarbase	hsa-miR-301a-3p	ABRAXAS2	23172	ENSG00000165660	validated
tarbase	hsa-miR-18a-5p	QSOX2	169714	ENSG00000165661	validated
tarbase	hsa-miR-21-5p	QSOX2	169714	ENSG00000165661	validated
tarbase	hsa-miR-106b-5p	NSD1	64324	ENSG00000165671	validated
tarbase	hsa-miR-144-3p	NSD1	64324	ENSG00000165671	validated
tarbase	hsa-miR-15b-5p	NSD1	64324	ENSG00000165671	validated
tarbase	hsa-miR-181a-5p	NSD1	64324	ENSG00000165671	validated
tarbase	hsa-miR-192-5p	NSD1	64324	ENSG00000165671	validated
tarbase	hsa-miR-222-3p	NSD1	64324	ENSG00000165671	validated
tarbase	hsa-miR-296-5p	NSD1	64324	ENSG00000165671	validated
tarbase	hsa-miR-326	NSD1	64324	ENSG00000165671	validated
tarbase	hsa-miR-106b-5p	PRDX3	10935	ENSG00000165672	validated
tarbase	hsa-miR-18a-5p	PRDX3	10935	ENSG00000165672	validated
tarbase	hsa-miR-21-5p	PRDX3	10935	ENSG00000165672	validated
tarbase	hsa-miR-181a-5p	GHITM	27069	ENSG00000165678	validated
tarbase	hsa-miR-15b-5p	PMPCA	23203	ENSG00000165688	validated
tarbase	hsa-miR-21-5p	TSC1	7248	ENSG00000165699	validated
tarbase	hsa-miR-301a-3p	TSC1	7248	ENSG00000165699	validated
tarbase	hsa-miR-144-3p	HPRT1	3251	ENSG00000165704	validated
tarbase	hsa-miR-144-3p	DIPK1B	138311	ENSG00000165716	validated
tarbase	hsa-miR-15b-5p	DIPK1B	138311	ENSG00000165716	validated
tarbase	hsa-miR-296-5p	DIPK1B	138311	ENSG00000165716	validated
tarbase	hsa-miR-301a-3p	DIPK1B	138311	ENSG00000165716	validated
tarbase	hsa-miR-106b-5p	DDX21	9188	ENSG00000165732	validated
tarbase	hsa-miR-15b-5p	DDX21	9188	ENSG00000165732	validated
tarbase	hsa-miR-181a-5p	DDX21	9188	ENSG00000165732	validated
tarbase	hsa-miR-21-5p	DDX21	9188	ENSG00000165732	validated
tarbase	hsa-miR-301a-3p	DDX21	9188	ENSG00000165732	validated
tarbase	hsa-miR-106b-5p	JCAD	57608	ENSG00000165757	validated
tarbase	hsa-miR-296-5p	JCAD	57608	ENSG00000165757	validated
tarbase	hsa-miR-301a-3p	JCAD	57608	ENSG00000165757	validated
tarbase	hsa-miR-212-3p	JCAD	57608	ENSG00000165757	validated
tarbase	hsa-miR-192-5p	PIP4P1	90809	ENSG00000165782	validated
tarbase	hsa-miR-301a-3p	PIP4P1	90809	ENSG00000165782	validated
tarbase	hsa-miR-296-5p	ZNF219	51222	ENSG00000165804	validated
tarbase	hsa-miR-301a-3p	ZNF219	51222	ENSG00000165804	validated
tarbase	hsa-miR-18a-5p	CASP7	840	ENSG00000165806	validated
tarbase	hsa-miR-212-3p	CASP7	840	ENSG00000165806	validated
tarbase	hsa-miR-582-5p	CCDC186	55088	ENSG00000165813	validated
tarbase	hsa-miR-106b-5p	TRUB1	142940	ENSG00000165832	validated
tarbase	hsa-miR-181a-5p	TRUB1	142940	ENSG00000165832	validated
tarbase	hsa-miR-15b-5p	TRUB1	142940	ENSG00000165832	validated
tarbase	hsa-miR-106b-5p	ZFYVE1	53349	ENSG00000165861	validated
tarbase	hsa-miR-15b-5p	ZFYVE1	53349	ENSG00000165861	validated
tarbase	hsa-miR-181a-5p	HSPA12A	259217	ENSG00000165868	validated
tarbase	hsa-miR-212-3p	ANKRD2	26287	ENSG00000165887	validated
tarbase	hsa-miR-106b-5p	E2F7	144455	ENSG00000165891	validated
tarbase	hsa-miR-181a-5p	E2F7	144455	ENSG00000165891	validated
tarbase	hsa-miR-18a-5p	E2F7	144455	ENSG00000165891	validated
tarbase	hsa-miR-212-3p	E2F7	144455	ENSG00000165891	validated
tarbase	hsa-miR-301a-3p	E2F7	144455	ENSG00000165891	validated
tarbase	hsa-miR-582-5p	OTOGL	283310	ENSG00000165899	validated
tarbase	hsa-miR-15b-5p	LARGE2	120071	ENSG00000165905	validated
tarbase	hsa-miR-21-5p	PSMC3	5702	ENSG00000165916	validated
tarbase	hsa-miR-21-5p	TC2N	123036	ENSG00000165929	validated
tarbase	hsa-miR-212-3p	IFI27	3429	ENSG00000165949	validated
tarbase	hsa-miR-15b-5p	CLMN	79789	ENSG00000165959	validated
tarbase	hsa-miR-582-3p	CLMN	79789	ENSG00000165959	validated
tarbase	hsa-miR-301a-3p	PTER	9317	ENSG00000165983	validated
tarbase	hsa-miR-212-3p	PTER	9317	ENSG00000165983	validated
tarbase	hsa-miR-15b-5p	C1QL3	389941	ENSG00000165985	validated
tarbase	hsa-miR-144-3p	CACNB2	783	ENSG00000165995	validated
tarbase	hsa-miR-106b-5p	ARL5B	221079	ENSG00000165997	validated
tarbase	hsa-miR-15b-5p	ARL5B	221079	ENSG00000165997	validated
tarbase	hsa-miR-181a-5p	ARL5B	221079	ENSG00000165997	validated
tarbase	hsa-miR-18a-5p	ARL5B	221079	ENSG00000165997	validated
tarbase	hsa-miR-192-5p	ARL5B	221079	ENSG00000165997	validated
tarbase	hsa-miR-212-3p	ARL5B	221079	ENSG00000165997	validated
tarbase	hsa-miR-582-5p	SMCO4	56935	ENSG00000166002	validated

tarbase	hsa-miR-212-3p	SMCO4	56935	ENSG00000166002	validated
tarbase	hsa-miR-18a-5p	SMCO4	56935	ENSG00000166002	validated
tarbase	hsa-miR-15b-5p	KCNC2	3747	ENSG00000166006	validated
tarbase	hsa-miR-181a-5p	KCNC2	3747	ENSG00000166006	validated
tarbase	hsa-miR-18a-5p	R3HCC1L	27291	ENSG00000166024	validated
tarbase	hsa-miR-301a-3p	AMOTL1	154810	ENSG00000166025	validated
tarbase	hsa-miR-181a-5p	AMOTL1	154810	ENSG00000166025	validated
tarbase	hsa-miR-15b-5p	HTRA1	5654	ENSG00000166033	validated
tarbase	hsa-miR-181a-5p	CEP57	9702	ENSG00000166037	validated
tarbase	hsa-miR-181a-5p	SPRED1	161742	ENSG00000166068	validated
tarbase	hsa-miR-106b-5p	GPR176	11245	ENSG00000166073	validated
tarbase	hsa-miR-18a-5p	GPR176	11245	ENSG00000166073	validated
tarbase	hsa-miR-582-5p	GLB1L3	112937	ENSG00000166105	validated
tarbase	hsa-miR-301a-3p	GPT2	84706	ENSG00000166123	validated
tarbase	hsa-miR-106b-5p	RAB8B	51762	ENSG00000166128	validated
tarbase	hsa-miR-181a-5p	RAB8B	51762	ENSG00000166128	validated
tarbase	hsa-miR-181a-5p	IKBIP	121457	ENSG00000166130	validated
tarbase	hsa-miR-18a-5p	HIF1AN	55662	ENSG00000166135	validated
tarbase	hsa-miR-301a-3p	HIF1AN	55662	ENSG00000166135	validated
tarbase	hsa-miR-212-3p	PPP1R14D	54866	ENSG00000166143	validated
tarbase	hsa-miR-106b-5p	FBN1	2200	ENSG00000166147	validated
tarbase	hsa-miR-181a-5p	FBN1	2200	ENSG00000166147	validated
tarbase	hsa-miR-21-5p	FBN1	2200	ENSG00000166147	validated
tarbase	hsa-miR-582-3p	FBN1	2200	ENSG00000166147	validated
tarbase	hsa-miR-582-5p	FBN1	2200	ENSG00000166147	validated
tarbase	hsa-miR-15b-5p	LRTM2	654429	ENSG00000166159	validated
tarbase	hsa-miR-212-3p	LRTM2	654429	ENSG00000166159	validated
tarbase	hsa-miR-21-5p	BRD7	29117	ENSG00000166164	validated
tarbase	hsa-miR-296-5p	BRD7	29117	ENSG00000166164	validated
tarbase	hsa-miR-15b-5p	CKB	1152	ENSG00000166165	validated
tarbase	hsa-miR-21-5p	CKB	1152	ENSG00000166165	validated
tarbase	hsa-miR-106b-5p	BTRC	8945	ENSG00000166167	validated
tarbase	hsa-miR-21-5p	BTRC	8945	ENSG00000166167	validated
tarbase	hsa-miR-192-5p	POLL	27343	ENSG00000166169	validated
tarbase	hsa-miR-296-5p	POLL	27343	ENSG00000166169	validated
tarbase	hsa-miR-15b-5p	BAG5	9529	ENSG00000166170	validated
tarbase	hsa-miR-18a-5p	BAG5	9529	ENSG00000166170	validated
tarbase	hsa-miR-301a-3p	BAG5	9529	ENSG00000166170	validated
tarbase	hsa-miR-192-5p	NOLC1	9221	ENSG00000166197	validated
tarbase	hsa-miR-212-3p	NOLC1	9221	ENSG00000166197	validated
tarbase	hsa-miR-106b-5p	COPS2	9318	ENSG00000166200	validated
tarbase	hsa-miR-582-3p	GABRB3	2562	ENSG00000166206	validated
tarbase	hsa-miR-222-3p	SGPL1	8879	ENSG00000166224	validated
tarbase	hsa-miR-326	SGPL1	8879	ENSG00000166224	validated
tarbase	hsa-miR-15b-5p	FRS2	10818	ENSG00000166225	validated
tarbase	hsa-miR-181a-5p	FRS2	10818	ENSG00000166225	validated
tarbase	hsa-miR-212-3p	FRS2	10818	ENSG00000166225	validated
tarbase	hsa-miR-222-3p	FRS2	10818	ENSG00000166225	validated
tarbase	hsa-miR-582-3p	FRS2	10818	ENSG00000166225	validated
tarbase	hsa-miR-505-3p	FRS2	10818	ENSG00000166225	validated
tarbase	hsa-miR-106b-5p	CCT2	10576	ENSG00000166226	validated
tarbase	hsa-miR-505-3p	CCT2	10576	ENSG00000166226	validated
tarbase	hsa-miR-582-3p	CCT2	10576	ENSG00000166226	validated
tarbase	hsa-miR-582-5p	CCT2	10576	ENSG00000166226	validated
tarbase	hsa-miR-106b-5p	ARIH1	25820	ENSG00000166233	validated
tarbase	hsa-miR-181a-5p	ARIH1	25820	ENSG00000166233	validated
tarbase	hsa-miR-212-3p	SCN3B	55800	ENSG00000166257	validated
tarbase	hsa-miR-181a-5p	COX11	1353	ENSG00000166260	validated
tarbase	hsa-miR-15b-5p	ZNF202	7753	ENSG00000166261	validated
tarbase	hsa-miR-212-3p	STXBP4	252983	ENSG00000166263	validated
tarbase	hsa-miR-326			ENSG00000166272	validated
tarbase	hsa-miR-582-5p	BORCS7	119032	ENSG00000166275	validated
tarbase	hsa-miR-212-3p	TMEM100	55273	ENSG00000166292	validated
tarbase	hsa-miR-222-3p	ANAPC16	119504	ENSG00000166295	validated
tarbase	hsa-miR-326	SMPD1	6609	ENSG00000166311	validated
tarbase	hsa-miR-15b-5p	APBB1	322	ENSG00000166313	validated
tarbase	hsa-miR-212-3p	C11orf65	160140	ENSG00000166323	validated
tarbase	hsa-miR-15b-5p	TRIM44	54765	ENSG00000166326	validated
tarbase	hsa-miR-15b-5p	PPFIBP2	8495	ENSG00000166387	validated
tarbase	hsa-miR-212-3p	CYB5R2	51700	ENSG00000166394	validated
tarbase	hsa-miR-222-3p	TUB	7275	ENSG00000166402	validated

tarbase	hsa-miR-296-5p	TUB	7275	ENSG00000166402	validated
tarbase	hsa-miR-326	TUB	7275	ENSG00000166402	validated
tarbase	hsa-miR-326	RPL27A	6157	ENSG00000166441	validated
tarbase	hsa-miR-21-5p	DENND2B	6764	ENSG00000166444	validated
tarbase	hsa-miR-144-3p	CDYL2	124359	ENSG00000166446	validated
tarbase	hsa-miR-15b-5p	TMEM130	222865	ENSG00000166448	validated
tarbase	hsa-miR-181a-5p	PRTG	283659	ENSG00000166450	validated
tarbase	hsa-miR-15b-5p	CENPN	55839	ENSG00000166451	validated
tarbase	hsa-miR-212-3p	TMEM41B	440026	ENSG00000166471	validated
tarbase	hsa-miR-106b-5p	ZNF143	7702	ENSG00000166478	validated
tarbase	hsa-miR-582-3p	ZNF143	7702	ENSG00000166478	validated
tarbase	hsa-miR-181a-5p	TMX3	54495	ENSG00000166479	validated
tarbase	hsa-miR-144-3p	WEE1	7465	ENSG00000166483	validated
tarbase	hsa-miR-181a-5p	WEE1	7465	ENSG00000166483	validated
tarbase	hsa-miR-212-3p	WEE1	7465	ENSG00000166483	validated
tarbase	hsa-miR-21-5p	WEE1	7465	ENSG00000166483	validated
tarbase	hsa-miR-222-3p	WEE1	7465	ENSG00000166483	validated
tarbase	hsa-miR-106b-5p	MAPK7	5598	ENSG00000166484	validated
tarbase	hsa-miR-15b-5p	HDGFL3	50810	ENSG00000166503	validated
tarbase	hsa-miR-15b-5p	MCM7	4176	ENSG00000166508	validated
tarbase	hsa-miR-301a-3p	MCM7	4176	ENSG00000166508	validated
tarbase	hsa-miR-106b-5p	MCM7	4176	ENSG00000166508	validated
tarbase	hsa-miR-301a-3p	ZNF3	7551	ENSG00000166526	validated
tarbase	hsa-miR-326	ZNF3	7551	ENSG00000166526	validated
tarbase	hsa-miR-181a-5p	RIMKLB	57494	ENSG00000166532	validated
tarbase	hsa-miR-326	RIMKLB	57494	ENSG00000166532	validated
tarbase	hsa-miR-15b-5p	RIMKLB	57494	ENSG00000166532	validated
tarbase	hsa-miR-15b-5p	TMED3	23423	ENSG00000166557	validated
tarbase	hsa-miR-106b-5p	TMEM135	65084	ENSG00000166575	validated
tarbase	hsa-miR-181a-5p	TMEM135	65084	ENSG00000166575	validated
tarbase	hsa-miR-212-3p	IQCD	115811	ENSG00000166578	validated
tarbase	hsa-miR-18a-5p	NDEL1	81565	ENSG00000166579	validated
tarbase	hsa-miR-301a-3p	NDEL1	81565	ENSG00000166579	validated
tarbase	hsa-miR-326	NDEL1	81565	ENSG00000166579	validated
tarbase	hsa-miR-21-5p	HSP90B1	7184	ENSG00000166598	validated
tarbase	hsa-miR-106b-5p	HSP90B1	7184	ENSG00000166598	validated
tarbase	hsa-miR-15b-5p	BLCAP	10904	ENSG00000166619	validated
tarbase	hsa-miR-181a-5p	BLCAP	10904	ENSG00000166619	validated
tarbase	hsa-miR-21-5p	BEX3	27018	ENSG00000166681	validated
tarbase	hsa-miR-301a-3p	BEX3	27018	ENSG00000166681	validated
tarbase	hsa-miR-21-5p	COG1	9382	ENSG00000166685	validated
tarbase	hsa-miR-326	ZNF606	80095	ENSG00000166704	validated
tarbase	hsa-miR-181a-5p	ZNF606	80095	ENSG00000166704	validated
tarbase	hsa-miR-192-5p	B2M	567	ENSG00000166710	validated
tarbase	hsa-miR-212-3p	B2M	567	ENSG00000166710	validated
tarbase	hsa-miR-21-5p	B2M	567	ENSG00000166710	validated
tarbase	hsa-miR-106b-5p	ZNF592	9640	ENSG00000166716	validated
tarbase	hsa-miR-15b-5p	ZNF592	9640	ENSG00000166716	validated
tarbase	hsa-miR-582-3p	ZNF592	9640	ENSG00000166716	validated
tarbase	hsa-miR-212-3p	AP1G1	164	ENSG00000166747	validated
tarbase	hsa-miR-222-3p	AP1G1	164	ENSG00000166747	validated
tarbase	hsa-miR-301a-3p	AP1G1	164	ENSG00000166747	validated
tarbase	hsa-miR-582-3p	SLFN5	162394	ENSG00000166750	validated
tarbase	hsa-miR-212-3p	SLFN5	162394	ENSG00000166750	validated
tarbase	hsa-miR-18a-5p	MARF1	9665	ENSG00000166783	validated
tarbase	hsa-miR-582-5p	CIAO2A	84191	ENSG00000166797	validated
tarbase	hsa-miR-15b-5p	FAM111A	63901	ENSG00000166801	validated
tarbase	hsa-miR-212-3p	LDHD	197257	ENSG00000166816	validated
tarbase	hsa-miR-212-3p	PLIN1	5346	ENSG00000166819	validated
tarbase	hsa-miR-18a-5p	MESP1	55897	ENSG00000166823	validated
tarbase	hsa-miR-326	NAV2	89797	ENSG00000166833	validated
tarbase	hsa-miR-212-3p	DCTN5	84516	ENSG00000166847	validated
tarbase	hsa-miR-505-3p	DCTN5	84516	ENSG00000166847	validated
tarbase	hsa-miR-21-5p	PLK1	5347	ENSG00000166851	validated
tarbase	hsa-miR-212-3p	PLK1	5347	ENSG00000166851	validated
tarbase	hsa-miR-106b-5p	PLK1	5347	ENSG00000166851	validated
tarbase	hsa-miR-15b-5p	CLPX	10845	ENSG00000166855	validated
tarbase	hsa-miR-15b-5p	ZBTB39	9880	ENSG00000166860	validated
tarbase	hsa-miR-212-3p	ZBTB39	9880	ENSG00000166860	validated
tarbase	hsa-miR-212-3p	CHP2	63928	ENSG00000166869	validated
tarbase	hsa-miR-181a-5p	NEMP1	23306	ENSG00000166881	validated

tarbase	hsa-miR-212-3p	NEMP1	23306	ENSG00000166881	validated
tarbase	hsa-miR-181a-5p	PATL1	219988	ENSG00000166889	validated
tarbase	hsa-miR-106b-5p	STX3	6809	ENSG00000166900	validated
tarbase	hsa-miR-222-3p	STX3	6809	ENSG00000166900	validated
tarbase	hsa-miR-301a-3p	STX3	6809	ENSG00000166900	validated
tarbase	hsa-miR-21-5p	MRPL16	54948	ENSG00000166902	validated
tarbase	hsa-miR-181a-5p	PIP4K2C	79837	ENSG00000166908	validated
tarbase	hsa-miR-15b-5p	MTMR10	54893	ENSG00000166912	validated
tarbase	hsa-miR-144-3p	YWHAB	7529	ENSG00000166913	validated
tarbase	hsa-miR-15b-5p	YWHAB	7529	ENSG00000166913	validated
tarbase	hsa-miR-181a-5p	YWHAB	7529	ENSG00000166913	validated
tarbase	hsa-miR-212-3p	YWHAB	7529	ENSG00000166913	validated
tarbase	hsa-miR-301a-3p	YWHAB	7529	ENSG00000166913	validated
tarbase	hsa-miR-212-3p	C15orf48	84419	ENSG00000166920	validated
tarbase	hsa-miR-212-3p	SCG5	6447	ENSG00000166922	validated
tarbase	hsa-miR-301a-3p	CCNDBP1	23582	ENSG00000166946	validated
tarbase	hsa-miR-144-3p	MAP1A	4130	ENSG00000166963	validated
tarbase	hsa-miR-15b-5p	MAP1A	4130	ENSG00000166963	validated
tarbase	hsa-miR-15b-5p	AKTIP	64400	ENSG00000166971	validated
tarbase	hsa-miR-15b-5p	MAPRE2	10982	ENSG00000166974	validated
tarbase	hsa-miR-106b-5p	MARS1	4141	ENSG00000166986	validated
tarbase	hsa-miR-15b-5p	MARS1	4141	ENSG00000166986	validated
tarbase	hsa-miR-181a-5p	MBD6	114785	ENSG00000166987	validated
tarbase	hsa-miR-296-5p	MBD6	114785	ENSG00000166987	validated
tarbase	hsa-miR-181a-5p	NUDT21	11051	ENSG00000167005	validated
tarbase	hsa-miR-21-5p	NUDT21	11051	ENSG00000167005	validated
tarbase	hsa-miR-144-3p	NKX3-1	4824	ENSG00000167034	validated
tarbase	hsa-miR-15b-5p	NKX3-1	4824	ENSG00000167034	validated
tarbase	hsa-miR-106b-5p	PBX3	5090	ENSG00000167081	validated
tarbase	hsa-miR-15b-5p	PBX3	5090	ENSG00000167081	validated
tarbase	hsa-miR-212-3p	PHB	5245	ENSG00000167085	validated
tarbase	hsa-miR-21-5p	SNRPD1	6632	ENSG00000167088	validated
tarbase	hsa-miR-222-3p	SNRPD1	6632	ENSG00000167088	validated
tarbase	hsa-miR-301a-3p	SNRPD1	6632	ENSG00000167088	validated
tarbase	hsa-miR-181a-5p	FAM102A	399665	ENSG00000167106	validated
tarbase	hsa-miR-301a-3p	FAM102A	399665	ENSG00000167106	validated
tarbase	hsa-miR-582-3p	FAM102A	399665	ENSG00000167106	validated
tarbase	hsa-miR-181a-5p	URM1	81605	ENSG00000167118	validated
tarbase	hsa-miR-15b-5p	CERCAM	51148	ENSG00000167123	validated
tarbase	hsa-miR-301a-3p	C15orf39	56905	ENSG00000167173	validated
tarbase	hsa-miR-222-3p	SP2	6668	ENSG00000167182	validated
tarbase	hsa-miR-15b-5p	PRR15L	79170	ENSG00000167183	validated
tarbase	hsa-miR-212-3p	CRK	1398	ENSG00000167193	validated
tarbase	hsa-miR-505-3p	CRK	1398	ENSG00000167193	validated
tarbase	hsa-miR-106b-5p	TBC1D2B	23102	ENSG00000167202	validated
tarbase	hsa-miR-296-5p	TBC1D2B	23102	ENSG00000167202	validated
tarbase	hsa-miR-212-3p	LOXHD1	125336	ENSG00000167210	validated
tarbase	hsa-miR-15b-5p	HDHD2	84064	ENSG00000167220	validated
tarbase	hsa-miR-181a-5p	ZNF91	7644	ENSG00000167232	validated
tarbase	hsa-miR-18a-5p	ZNF91	7644	ENSG00000167232	validated
tarbase	hsa-miR-222-3p	ZNF91	7644	ENSG00000167232	validated
tarbase	hsa-miR-144-3p	IGF2	3481	ENSG00000167244	validated
tarbase	hsa-miR-15b-5p	CDK12	51755	ENSG00000167258	validated
tarbase	hsa-miR-18a-5p	CDK12	51755	ENSG00000167258	validated
tarbase	hsa-miR-192-5p	CDK12	51755	ENSG00000167258	validated
tarbase	hsa-miR-212-3p	CDK12	51755	ENSG00000167258	validated
tarbase	hsa-miR-582-3p	CDK12	51755	ENSG00000167258	validated
tarbase	hsa-miR-21-5p	DUS2	54920	ENSG00000167264	validated
tarbase	hsa-miR-106b-5p	MYO5B	4645	ENSG00000167306	validated
tarbase	hsa-miR-15b-5p	MYO5B	4645	ENSG00000167306	validated
tarbase	hsa-miR-18a-5p	RRM1	6240	ENSG00000167325	validated
tarbase	hsa-miR-212-3p	MMP26	56547	ENSG00000167346	validated
tarbase	hsa-miR-15b-5p	FN3K	64122	ENSG00000167363	validated
tarbase	hsa-miR-212-3p	ZNF23	7571	ENSG00000167377	validated
tarbase	hsa-miR-15b-5p	IRGQ	126298	ENSG00000167378	validated
tarbase	hsa-miR-192-5p	IRGQ	126298	ENSG00000167378	validated
tarbase	hsa-miR-222-3p	IRGQ	126298	ENSG00000167378	validated
tarbase	hsa-miR-222-3p	ZNF226	7769	ENSG00000167380	validated
tarbase	hsa-miR-181a-5p	ZNF180	7733	ENSG00000167384	validated
tarbase	hsa-miR-18a-5p	ZNF180	7733	ENSG00000167384	validated
tarbase	hsa-miR-301a-3p	ZNF180	7733	ENSG00000167384	validated

tarbase	hsa-miR-296-5p	ZNF646	9726	ENSG00000167395	validated
tarbase	hsa-miR-212-3p	GNG8	94235	ENSG00000167414	validated
tarbase	hsa-miR-21-5p	SMG8	55181	ENSG00000167447	validated
tarbase	hsa-miR-192-5p	RAB8A	4218	ENSG00000167461	validated
tarbase	hsa-miR-15b-5p	MIDN	90007	ENSG00000167470	validated
tarbase	hsa-miR-181a-5p	MIDN	90007	ENSG00000167470	validated
tarbase	hsa-miR-296-5p	MIDN	90007	ENSG00000167470	validated
tarbase	hsa-miR-301a-3p	MIDN	90007	ENSG00000167470	validated
tarbase	hsa-miR-15b-5p	KLHL26	55295	ENSG00000167487	validated
tarbase	hsa-miR-296-5p	GATAD2A	54815	ENSG00000167491	validated
tarbase	hsa-miR-296-5p	CDT1	81620	ENSG00000167513	validated
tarbase	hsa-miR-582-3p	CDT1	81620	ENSG00000167513	validated
tarbase	hsa-miR-222-3p	CDT1	81620	ENSG00000167513	validated
tarbase	hsa-miR-106b-5p	ANKRD11	29123	ENSG00000167522	validated
tarbase	hsa-miR-296-5p	ANKRD11	29123	ENSG00000167522	validated
tarbase	hsa-miR-582-3p	ANKRD11	29123	ENSG00000167522	validated
tarbase	hsa-miR-296-5p	SPATA33	124045	ENSG00000167523	validated
tarbase	hsa-miR-212-3p	RSKR	124923	ENSG00000167524	validated
tarbase	hsa-miR-15b-5p	DHRS13	147015	ENSG00000167536	validated
tarbase	hsa-miR-181a-5p	KMT2D	8085	ENSG00000167548	validated
tarbase	hsa-miR-21-5p	KMT2D	8085	ENSG00000167548	validated
tarbase	hsa-miR-296-5p	KMT2D	8085	ENSG00000167548	validated
tarbase	hsa-miR-301a-3p	KMT2D	8085	ENSG00000167548	validated
tarbase	hsa-miR-326	KMT2D	8085	ENSG00000167548	validated
tarbase	hsa-miR-15b-5p	TUBA1A	7846	ENSG00000167552	validated
tarbase	hsa-miR-15b-5p	SERTAD3	29946	ENSG00000167565	validated
tarbase	hsa-miR-192-5p	PROSER3	148137	ENSG00000167595	validated
tarbase	hsa-miR-18a-5p	PROSER3	148137	ENSG00000167595	validated
tarbase	hsa-miR-192-5p	AXL	558	ENSG00000167601	validated
tarbase	hsa-miR-21-5p	AXL	558	ENSG00000167601	validated
tarbase	hsa-miR-301a-3p	AXL	558	ENSG00000167601	validated
tarbase	hsa-miR-15b-5p	LENG8	114823	ENSG00000167615	validated
tarbase	hsa-miR-296-5p	LENG8	114823	ENSG00000167615	validated
tarbase	hsa-miR-212-3p	LAIR2	3904	ENSG00000167618	validated
tarbase	hsa-miR-301a-3p	ZNF526	116115	ENSG00000167625	validated
tarbase	hsa-miR-106b-5p	ZNF146	7705	ENSG00000167635	validated
tarbase	hsa-miR-15b-5p	ZNF146	7705	ENSG00000167635	validated
tarbase	hsa-miR-181a-5p	ZNF146	7705	ENSG00000167635	validated
tarbase	hsa-miR-18a-5p	ZNF146	7705	ENSG00000167635	validated
tarbase	hsa-miR-582-5p	ZNF146	7705	ENSG00000167635	validated
tarbase	hsa-miR-301a-3p	DAPK3	1613	ENSG00000167657	validated
tarbase	hsa-miR-296-5p	EEF2	1938	ENSG00000167658	validated
tarbase	hsa-miR-326	EEF2	1938	ENSG00000167658	validated
tarbase	hsa-miR-582-3p	EEF2	1938	ENSG00000167658	validated
tarbase	hsa-miR-212-3p	PLIN4	729359	ENSG00000167676	validated
tarbase	hsa-miR-505-3p	NXN	64359	ENSG00000167693	validated
tarbase	hsa-miR-296-5p	KIFC2	90990	ENSG00000167702	validated
tarbase	hsa-miR-212-3p	KIFC2	90990	ENSG00000167702	validated
tarbase	hsa-miR-301a-3p	WDR81	124997	ENSG00000167716	validated
tarbase	hsa-miR-181a-5p	TSR1	55720	ENSG00000167721	validated
tarbase	hsa-miR-326	TSR1	55720	ENSG00000167721	validated
tarbase	hsa-miR-15b-5p	C19orf48	84798	ENSG00000167747	validated
tarbase	hsa-miR-296-5p	C19orf48	84798	ENSG00000167747	validated
tarbase	hsa-miR-301a-3p	C19orf48	84798	ENSG00000167747	validated
tarbase	hsa-miR-296-5p	KRT80	144501	ENSG00000167767	validated
tarbase	hsa-miR-582-5p	KRT80	144501	ENSG00000167767	validated
tarbase	hsa-miR-15b-5p	SPRYD3	84926	ENSG00000167778	validated
tarbase	hsa-miR-582-3p	SPRYD3	84926	ENSG00000167778	validated
tarbase	hsa-miR-212-3p	IGFBP6	3489	ENSG00000167779	validated
tarbase	hsa-miR-181a-5p	ZNF232	7775	ENSG00000167840	validated
tarbase	hsa-miR-15b-5p	CD300A	11314	ENSG00000167851	validated
tarbase	hsa-miR-15b-5p	TMC8	147138	ENSG00000167895	validated
tarbase	hsa-miR-106b-5p	ABCA3	21	ENSG00000167972	validated
tarbase	hsa-miR-15b-5p	SRRM2	23524	ENSG00000167978	validated
tarbase	hsa-miR-181a-5p	SRRM2	23524	ENSG00000167978	validated
tarbase	hsa-miR-192-5p	SRRM2	23524	ENSG00000167978	validated
tarbase	hsa-miR-21-5p	SRRM2	23524	ENSG00000167978	validated
tarbase	hsa-miR-296-5p	SRRM2	23524	ENSG00000167978	validated
tarbase	hsa-miR-301a-3p	SRRM2	23524	ENSG00000167978	validated
tarbase	hsa-miR-326	SRRM2	23524	ENSG00000167978	validated
tarbase	hsa-miR-582-5p	SRRM2	23524	ENSG00000167978	validated

tarbase	hsa-miR-106b-5p	SDHAF2	54949	ENSG00000167985	validated
tarbase	hsa-miR-301a-3p	SDHAF2	54949	ENSG00000167985	validated
tarbase	hsa-miR-15b-5p	DDB1	1642	ENSG00000167986	validated
tarbase	hsa-miR-18a-5p	DDB1	1642	ENSG00000167986	validated
tarbase	hsa-miR-582-5p	DDB1	1642	ENSG00000167986	validated
tarbase	hsa-miR-15b-5p	VPS37C	55048	ENSG00000167987	validated
tarbase	hsa-miR-21-5p	VWCE	220001	ENSG00000167992	validated
tarbase	hsa-miR-144-3p	BEST1	7439	ENSG00000167995	validated
tarbase	hsa-miR-505-3p	SLC3A2	6520	ENSG00000168003	validated
tarbase	hsa-miR-21-5p	RPSA	3921	ENSG00000168028	validated
tarbase	hsa-miR-15b-5p	CTNNB1	1499	ENSG00000168036	validated
tarbase	hsa-miR-18a-5p	CTNNB1	1499	ENSG00000168036	validated
tarbase	hsa-miR-21-5p	CTNNB1	1499	ENSG00000168036	validated
tarbase	hsa-miR-222-3p	CTNNB1	1499	ENSG00000168036	validated
tarbase	hsa-miR-582-5p	CTNNB1	1499	ENSG00000168036	validated
tarbase	hsa-miR-106b-5p	CTNNB1	1499	ENSG00000168036	validated
tarbase	hsa-miR-181a-5p	ULK4	54986	ENSG00000168038	validated
tarbase	hsa-miR-212-3p	ULK4	54986	ENSG00000168038	validated
tarbase	hsa-miR-192-5p	LTBP3	4054	ENSG00000168056	validated
tarbase	hsa-miR-212-3p	LTBP3	4054	ENSG00000168056	validated
tarbase	hsa-miR-296-5p	LTBP3	4054	ENSG00000168056	validated
tarbase	hsa-miR-326	LTBP3	4054	ENSG00000168056	validated
tarbase	hsa-miR-505-3p	SF1	7536	ENSG00000168066	validated
tarbase	hsa-miR-21-5p	MAP4K2	5871	ENSG00000168067	validated
tarbase	hsa-miR-301a-3p	SCARA3	51435	ENSG00000168077	validated
tarbase	hsa-miR-21-5p	PBK	55872	ENSG00000168078	validated
tarbase	hsa-miR-106b-5p	PBK	55872	ENSG00000168078	validated
tarbase	hsa-miR-21-5p	COPS6	10980	ENSG00000168090	validated
tarbase	hsa-miR-181a-5p	PAFAH1B2	5049	ENSG00000168092	validated
tarbase	hsa-miR-212-3p	NUDT16L1	84309	ENSG00000168101	validated
tarbase	hsa-miR-212-3p	KIAA1586	57691	ENSG00000168116	validated
tarbase	hsa-miR-15b-5p	SETD5	55209	ENSG00000168137	validated
tarbase	hsa-miR-181a-5p	SETD5	55209	ENSG00000168137	validated
tarbase	hsa-miR-18a-5p	SETD5	55209	ENSG00000168137	validated
tarbase	hsa-miR-296-5p	SETD5	55209	ENSG00000168137	validated
tarbase	hsa-miR-505-3p	SETD5	55209	ENSG00000168137	validated
tarbase	hsa-miR-212-3p	THAP9	79725	ENSG00000168152	validated
tarbase	hsa-miR-15b-5p	RNF187	149603	ENSG00000168159	validated
tarbase	hsa-miR-18a-5p	RNF187	149603	ENSG00000168159	validated
tarbase	hsa-miR-222-3p	HOOK3	84376	ENSG00000168172	validated
tarbase	hsa-miR-106b-5p	MAPK1IP1L	93487	ENSG00000168175	validated
tarbase	hsa-miR-18a-5p	MAPK1IP1L	93487	ENSG00000168175	validated
tarbase	hsa-miR-222-3p	DDIT4	54541	ENSG00000168209	validated
tarbase	hsa-miR-106b-5p	DDIT4	54541	ENSG00000168209	validated
tarbase	hsa-miR-106b-5p	RBPJ	3516	ENSG00000168214	validated
tarbase	hsa-miR-181a-5p	RBPJ	3516	ENSG00000168214	validated
tarbase	hsa-miR-21-5p	RBPJ	3516	ENSG00000168214	validated
tarbase	hsa-miR-582-5p	RBPJ	3516	ENSG00000168214	validated
tarbase	hsa-miR-106b-5p	ZCCHC4	29063	ENSG00000168228	validated
tarbase	hsa-miR-15b-5p	PTGDR	5729	ENSG00000168229	validated
tarbase	hsa-miR-181a-5p	GLYCTK	132158	ENSG00000168237	validated
tarbase	hsa-miR-222-3p	UBTD2	92181	ENSG00000168246	validated
tarbase	hsa-miR-582-5p	UBTD2	92181	ENSG00000168246	validated
tarbase	hsa-miR-106b-5p	UBTD2	92181	ENSG00000168246	validated
tarbase	hsa-miR-301a-3p	NKIRAS2	28511	ENSG00000168256	validated
tarbase	hsa-miR-582-5p	NKIRAS2	28511	ENSG00000168256	validated
tarbase	hsa-miR-15b-5p	IRF2BP2	359948	ENSG00000168264	validated
tarbase	hsa-miR-301a-3p	IRF2BP2	359948	ENSG00000168264	validated
tarbase	hsa-miR-326	IRF2BP2	359948	ENSG00000168264	validated
tarbase	hsa-miR-582-3p	IRF2BP2	359948	ENSG00000168264	validated
tarbase	hsa-miR-18a-5p	NT5DC2	64943	ENSG00000168268	validated
tarbase	hsa-miR-15b-5p	KIF5C	3800	ENSG00000168280	validated
tarbase	hsa-miR-21-5p	KIF5C	3800	ENSG00000168280	validated
tarbase	hsa-miR-582-5p	KIF5C	3800	ENSG00000168280	validated
tarbase	hsa-miR-181a-5p	KIF5C	3800	ENSG00000168280	validated
tarbase	hsa-miR-181a-5p	MGAT2	4247	ENSG00000168282	validated
tarbase	hsa-miR-181a-5p	BMI1	648	ENSG00000168283	validated
tarbase	hsa-miR-222-3p	BMI1	648	ENSG00000168283	validated
tarbase	hsa-miR-15b-5p	H1-4	3008	ENSG00000168298	validated
tarbase	hsa-miR-21-5p	H1-4	3008	ENSG00000168298	validated
tarbase	hsa-miR-212-3p	H1-4	3008	ENSG00000168298	validated

tarbase	hsa-miR-326	PCMTD1	115294	ENSG00000168300	validated
tarbase	hsa-miR-106b-5p	MPLKIP	136647	ENSG00000168303	validated
tarbase	hsa-miR-326	FAM107A	11170	ENSG00000168309	validated
tarbase	hsa-miR-181a-5p	FAM107A	11170	ENSG00000168309	validated
tarbase	hsa-miR-15b-5p	IRF2	3660	ENSG00000168310	validated
tarbase	hsa-miR-326	IRF2	3660	ENSG00000168310	validated
tarbase	hsa-miR-21-5p	ARF4	378	ENSG00000168374	validated
tarbase	hsa-miR-222-3p	ARF4	378	ENSG00000168374	validated
tarbase	hsa-miR-301a-3p	ARF4	378	ENSG00000168374	validated
tarbase	hsa-miR-144-3p	SEPTIN2	4735	ENSG00000168385	validated
tarbase	hsa-miR-192-5p	SEPTIN2	4735	ENSG00000168385	validated
tarbase	hsa-miR-301a-3p	SEPTIN2	4735	ENSG00000168385	validated
tarbase	hsa-miR-18a-5p	SEPTIN2	4735	ENSG00000168385	validated
tarbase	hsa-miR-212-3p	TAP1	6890	ENSG00000168394	validated
tarbase	hsa-miR-301a-3p	ATG4B	23192	ENSG00000168397	validated
tarbase	hsa-miR-15b-5p	BDKRB2	624	ENSG00000168398	validated
tarbase	hsa-miR-15b-5p	RFWD3	55159	ENSG00000168411	validated
tarbase	hsa-miR-106b-5p	CDC40	51362	ENSG00000168438	validated
tarbase	hsa-miR-15b-5p	STIP1	10963	ENSG00000168439	validated
tarbase	hsa-miR-212-3p	STIP1	10963	ENSG00000168439	validated
tarbase	hsa-miR-192-5p	REEP4	80346	ENSG00000168476	validated
tarbase	hsa-miR-18a-5p	ATXN2L	11273	ENSG00000168488	validated
tarbase	hsa-miR-21-5p	ATXN2L	11273	ENSG00000168488	validated
tarbase	hsa-miR-296-5p	ATXN2L	11273	ENSG00000168488	validated
tarbase	hsa-miR-301a-3p	MTCL1	23255	ENSG00000168502	validated
tarbase	hsa-miR-181a-5p	CDKN2AIP	55602	ENSG00000168564	validated
tarbase	hsa-miR-222-3p	CDKN2AIP	55602	ENSG00000168564	validated
tarbase	hsa-miR-212-3p	CDKN2AIP	55602	ENSG00000168564	validated
tarbase	hsa-miR-15b-5p	SNRNP48	154007	ENSG00000168566	validated
tarbase	hsa-miR-15b-5p	SLC20A2	6575	ENSG00000168575	validated
tarbase	hsa-miR-181a-5p	SLC20A2	6575	ENSG00000168575	validated
tarbase	hsa-miR-18a-5p	SLC20A2	6575	ENSG00000168575	validated
tarbase	hsa-miR-212-3p	DYNLRB2	83657	ENSG00000168589	validated
tarbase	hsa-miR-15b-5p	STAT3	6774	ENSG00000168610	validated
tarbase	hsa-miR-18a-5p	STAT3	6774	ENSG00000168610	validated
tarbase	hsa-miR-301a-3p	STAT3	6774	ENSG00000168610	validated
tarbase	hsa-miR-15b-5p	ZSWIM1	90204	ENSG00000168612	validated
tarbase	hsa-miR-106b-5p	ADAM9	8754	ENSG00000168615	validated
tarbase	hsa-miR-18a-5p	ADAM9	8754	ENSG00000168615	validated
tarbase	hsa-miR-21-5p	ADAM9	8754	ENSG00000168615	validated
tarbase	hsa-miR-18a-5p	AXIN2	8313	ENSG00000168646	validated
tarbase	hsa-miR-181a-5p	NDUFS5	4725	ENSG00000168653	validated
tarbase	hsa-miR-21-5p	NDUFS5	4725	ENSG00000168653	validated
tarbase	hsa-miR-181a-5p	ZNF30	90075	ENSG00000168661	validated
tarbase	hsa-miR-212-3p	LRATD2	157638	ENSG00000168672	validated
tarbase	hsa-miR-301a-3p	LRATD2	157638	ENSG00000168672	validated
tarbase	hsa-miR-505-3p	LRATD2	157638	ENSG00000168672	validated
tarbase	hsa-miR-15b-5p	SEMA4C	54910	ENSG00000168758	validated
tarbase	hsa-miR-106b-5p	TET2	54790	ENSG00000168769	validated
tarbase	hsa-miR-222-3p	TET2	54790	ENSG00000168769	validated
tarbase	hsa-miR-301a-3p	TET2	54790	ENSG00000168769	validated
tarbase	hsa-miR-212-3p	TET2	54790	ENSG00000168769	validated
tarbase	hsa-miR-212-3p	CXXC4	80319	ENSG00000168772	validated
tarbase	hsa-miR-181a-5p	TSPAN5	10098	ENSG00000168785	validated
tarbase	hsa-miR-296-5p	ABHD15	116236	ENSG00000168792	validated
tarbase	hsa-miR-326	ZBTB5	9925	ENSG00000168795	validated
tarbase	hsa-miR-144-3p	SNTB2	6645	ENSG00000168807	validated
tarbase	hsa-miR-21-5p	SNTB2	6645	ENSG00000168807	validated
tarbase	hsa-miR-582-5p	SNTB2	6645	ENSG00000168807	validated
tarbase	hsa-miR-144-3p	ZNF507	22847	ENSG00000168813	validated
tarbase	hsa-miR-582-5p	ZNF507	22847	ENSG00000168813	validated
tarbase	hsa-miR-106b-5p	NSG1	27065	ENSG00000168824	validated
tarbase	hsa-miR-181a-5p	NSG1	27065	ENSG00000168824	validated
tarbase	hsa-miR-181a-5p	GFM1	85476	ENSG00000168827	validated
tarbase	hsa-miR-301a-3p	FSTL5	56884	ENSG00000168843	validated
tarbase	hsa-miR-21-5p	ANKRD49	54851	ENSG00000168876	validated
tarbase	hsa-miR-106b-5p	TNIP2	79155	ENSG00000168884	validated
tarbase	hsa-miR-18a-5p	TNIP2	79155	ENSG00000168884	validated
tarbase	hsa-miR-15b-5p	MAT2A	4144	ENSG00000168906	validated
tarbase	hsa-miR-181a-5p	MAT2A	4144	ENSG00000168906	validated
tarbase	hsa-miR-505-3p	MAT2A	4144	ENSG00000168906	validated

tarbase	hsa-miR-582-3p	MAT2A	4144	ENSG00000168906	validated
tarbase	hsa-miR-181a-5p	LETM1	3954	ENSG00000168924	validated
tarbase	hsa-miR-326	TMEM129	92305	ENSG00000168936	validated
tarbase	hsa-miR-106b-5p	CEP120	153241	ENSG00000168944	validated
tarbase	hsa-miR-181a-5p	CEP120	153241	ENSG00000168944	validated
tarbase	hsa-miR-301a-3p	CEP120	153241	ENSG00000168944	validated
tarbase	hsa-miR-296-5p	JMJD7-PLA2G4B	8681	ENSG00000168970	validated
tarbase	hsa-miR-212-3p	CPLX1	10815	ENSG00000168993	validated
tarbase	hsa-miR-15b-5p	FEM1B	10116	ENSG00000169018	validated
tarbase	hsa-miR-181a-5p	FEM1B	10116	ENSG00000169018	validated
tarbase	hsa-miR-301a-3p	FEM1B	10116	ENSG00000169018	validated
tarbase	hsa-miR-582-3p	FEM1B	10116	ENSG00000169018	validated
tarbase	hsa-miR-212-3p	FEM1B	10116	ENSG00000169018	validated
tarbase	hsa-miR-15b-5p	MAP2K1	5604	ENSG00000169032	validated
tarbase	hsa-miR-106b-5p	IRS1	3667	ENSG00000169047	validated
tarbase	hsa-miR-301a-3p	MECP2	4204	ENSG00000169057	validated
tarbase	hsa-miR-212-3p	VXN	254778	ENSG00000169085	validated
tarbase	hsa-miR-15b-5p	HSPBAP1	79663	ENSG00000169087	validated
tarbase	hsa-miR-212-3p	HSPBAP1	79663	ENSG00000169087	validated
tarbase	hsa-miR-296-5p	SLC25A6	293	ENSG00000169100	validated
tarbase	hsa-miR-301a-3p	CSNK1G1	53944	ENSG00000169118	validated
tarbase	hsa-miR-505-3p	CSNK1G1	53944	ENSG00000169118	validated
tarbase	hsa-miR-181a-5p	CSNK1G1	53944	ENSG00000169118	validated
tarbase	hsa-miR-15b-5p	FAM110B	90362	ENSG00000169122	validated
tarbase	hsa-miR-212-3p	UBE2V2	7336	ENSG00000169139	validated
tarbase	hsa-miR-106b-5p	ZBTB43	23099	ENSG00000169155	validated
tarbase	hsa-miR-21-5p	ZBTB43	23099	ENSG00000169155	validated
tarbase	hsa-miR-15b-5p	PCSK9	255738	ENSG00000169174	validated
tarbase	hsa-miR-15b-5p	XPO6	23214	ENSG00000169180	validated
tarbase	hsa-miR-181a-5p	XPO6	23214	ENSG00000169180	validated
tarbase	hsa-miR-181a-5p	APEX2	27301	ENSG00000169188	validated
tarbase	hsa-miR-212-3p	APEX2	27301	ENSG00000169188	validated
tarbase	hsa-miR-301a-3p	CCDC126	90693	ENSG00000169193	validated
tarbase	hsa-miR-301a-3p	TBC1D10B	26000	ENSG00000169221	validated
tarbase	hsa-miR-181a-5p	PRELID1	27166	ENSG00000169230	validated
tarbase	hsa-miR-222-3p	PRELID1	27166	ENSG00000169230	validated
tarbase	hsa-miR-212-3p	THBS3	7059	ENSG00000169231	validated
tarbase	hsa-miR-21-5p	CA5B	11238	ENSG00000169239	validated
tarbase	hsa-miR-15b-5p	EFNA1	1942	ENSG00000169242	validated
tarbase	hsa-miR-212-3p	CXCL10	3627	ENSG00000169245	validated
tarbase	hsa-miR-212-3p	CXCL11	6373	ENSG00000169248	validated
tarbase	hsa-miR-106b-5p	NMD3	51068	ENSG00000169251	validated
tarbase	hsa-miR-212-3p	GPRIN1	114787	ENSG00000169258	validated
tarbase	hsa-miR-212-3p	HSPB3	8988	ENSG00000169271	validated
tarbase	hsa-miR-15b-5p	MRPL1	65008	ENSG00000169288	validated
tarbase	hsa-miR-181a-5p	SIN3A	25942	ENSG00000169375	validated
tarbase	hsa-miR-212-3p	ARL13B	200894	ENSG00000169379	validated
tarbase	hsa-miR-106b-5p	PTK2	5747	ENSG00000169398	validated
tarbase	hsa-miR-181a-5p	PTK2	5747	ENSG00000169398	validated
tarbase	hsa-miR-106b-5p	CXCL8	3576	ENSG00000169429	validated
tarbase	hsa-miR-21-5p	CXCL8	3576	ENSG00000169429	validated
tarbase	hsa-miR-212-3p	CXCL8	3576	ENSG00000169429	validated
tarbase	hsa-miR-21-5p	SDC2	6383	ENSG00000169439	validated
tarbase	hsa-miR-222-3p	SDC2	6383	ENSG00000169439	validated
tarbase	hsa-miR-15b-5p	SDC2	6383	ENSG00000169439	validated
tarbase	hsa-miR-181a-5p	SDC2	6383	ENSG00000169439	validated
tarbase	hsa-miR-222-3p	MMGT1	93380	ENSG00000169446	validated
tarbase	hsa-miR-582-5p	MMGT1	93380	ENSG00000169446	validated
tarbase	hsa-miR-106b-5p	MMGT1	93380	ENSG00000169446	validated
tarbase	hsa-miR-181a-5p	MMGT1	93380	ENSG00000169446	validated
tarbase	hsa-miR-222-3p	SPRR1B	6699	ENSG00000169469	validated
tarbase	hsa-miR-18a-5p	CLIC4	25932	ENSG00000169504	validated
tarbase	hsa-miR-222-3p	CLIC4	25932	ENSG00000169504	validated
tarbase	hsa-miR-181a-5p	GPR183	1880	ENSG00000169508	validated
tarbase	hsa-miR-192-5p	ZEB2	9839	ENSG00000169554	validated
tarbase	hsa-miR-582-3p	ZEB2	9839	ENSG00000169554	validated
tarbase	hsa-miR-212-3p	GJB1	2705	ENSG00000169562	validated
tarbase	hsa-miR-181a-5p	PCBP1	5093	ENSG00000169564	validated
tarbase	hsa-miR-15b-5p	HINT1	3094	ENSG00000169567	validated
tarbase	hsa-miR-296-5p	INO80E	283899	ENSG00000169592	validated
tarbase	hsa-miR-18a-5p	BNC1	646	ENSG00000169594	validated

tarbase	hsa-miR-106b-5p	DFFB	1677	ENSG00000169598	validated
tarbase	hsa-miR-21-5p	ANTXR1	84168	ENSG00000169604	validated
tarbase	hsa-miR-582-5p	CKAP2L	150468	ENSG00000169607	validated
tarbase	hsa-miR-212-3p	CKAP2L	150468	ENSG00000169607	validated
tarbase	hsa-miR-222-3p	CKAP2L	150468	ENSG00000169607	validated
tarbase	hsa-miR-192-5p	C15orf40	123207	ENSG00000169609	validated
tarbase	hsa-miR-326	RGPD8	727851	ENSG00000169629	validated
tarbase	hsa-miR-192-5p	HIC2	23119	ENSG00000169635	validated
tarbase	hsa-miR-296-5p	HIC2	23119	ENSG00000169635	validated
tarbase	hsa-miR-301a-3p	HIC2	23119	ENSG00000169635	validated
tarbase	hsa-miR-106b-5p	LUZP1	7798	ENSG00000169641	validated
tarbase	hsa-miR-21-5p	BUB1	699	ENSG00000169679	validated
tarbase	hsa-miR-582-5p	BUB1	699	ENSG00000169679	validated
tarbase	hsa-miR-582-3p	LRRC45	201255	ENSG00000169683	validated
tarbase	hsa-miR-15b-5p	CHRNA5	1138	ENSG00000169684	validated
tarbase	hsa-miR-212-3p	CHRNA5	1138	ENSG00000169684	validated
tarbase	hsa-miR-15b-5p	AGPAT2	10555	ENSG00000169692	validated
tarbase	hsa-miR-18a-5p	ASPSCR1	79058	ENSG00000169696	validated
tarbase	hsa-miR-301a-3p	FASN	2194	ENSG00000169710	validated
tarbase	hsa-miR-15b-5p	CNBP	7555	ENSG00000169714	validated
tarbase	hsa-miR-212-3p	CNBP	7555	ENSG00000169714	validated
tarbase	hsa-miR-15b-5p	DUS1L	64118	ENSG00000169718	validated
tarbase	hsa-miR-301a-3p	DUS1L	64118	ENSG00000169718	validated
tarbase	hsa-miR-326	DUS1L	64118	ENSG00000169718	validated
tarbase	hsa-miR-192-5p	DCXR	51181	ENSG00000169738	validated
tarbase	hsa-miR-181a-5p	ZNF32	7580	ENSG00000169740	validated
tarbase	hsa-miR-582-3p	RAC3	5881	ENSG00000169750	validated
tarbase	hsa-miR-21-5p	LIMS1	3987	ENSG00000169756	validated
tarbase	hsa-miR-15b-5p	TAPT1	202018	ENSG00000169762	validated
tarbase	hsa-miR-212-3p	UGP2	7360	ENSG00000169764	validated
tarbase	hsa-miR-181a-5p	HNRNPF	3185	ENSG00000169813	validated
tarbase	hsa-miR-192-5p	HNRNPF	3185	ENSG00000169813	validated
tarbase	hsa-miR-222-3p	HNRNPF	3185	ENSG00000169813	validated
tarbase	hsa-miR-144-3p	PCDH7	5099	ENSG00000169851	validated
tarbase	hsa-miR-582-5p	PCDH7	5099	ENSG00000169851	validated
tarbase	hsa-miR-106b-5p	ROBO1	6091	ENSG00000169855	validated
tarbase	hsa-miR-181a-5p	AVEN	57099	ENSG00000169857	validated
tarbase	hsa-miR-181a-5p	P2RY1	5028	ENSG00000169860	validated
tarbase	hsa-miR-212-3p	TRIM56	81844	ENSG00000169871	validated
tarbase	hsa-miR-181a-5p	REPS2	9185	ENSG00000169891	validated
tarbase	hsa-miR-106b-5p	SYAP1	94056	ENSG00000169895	validated
tarbase	hsa-miR-15b-5p	SYAP1	94056	ENSG00000169895	validated
tarbase	hsa-miR-181a-5p	SYAP1	94056	ENSG00000169895	validated
tarbase	hsa-miR-18a-5p	TOR1AIP2	163590	ENSG00000169905	validated
tarbase	hsa-miR-212-3p	TOR1AIP2	163590	ENSG00000169905	validated
tarbase	hsa-miR-301a-3p	TOR1AIP2	163590	ENSG00000169905	validated
tarbase	hsa-miR-181a-5p	TM4SF1	4071	ENSG00000169908	validated
tarbase	hsa-miR-106b-5p	OTUD3	23252	ENSG00000169914	validated
tarbase	hsa-miR-15b-5p	OTUD3	23252	ENSG00000169914	validated
tarbase	hsa-miR-582-3p	OTUD3	23252	ENSG00000169914	validated
tarbase	hsa-miR-15b-5p	GUSB	2990	ENSG00000169919	validated
tarbase	hsa-miR-212-3p	GUSB	2990	ENSG00000169919	validated
tarbase	hsa-miR-15b-5p	BRD3	8019	ENSG00000169925	validated
tarbase	hsa-miR-301a-3p	KLF13	51621	ENSG00000169926	validated
tarbase	hsa-miR-106b-5p	FRMPD4	9758	ENSG00000169933	validated
tarbase	hsa-miR-296-5p	ZNF768	79724	ENSG00000169957	validated
tarbase	hsa-miR-181a-5p	MAP3K2	10746	ENSG00000169967	validated
tarbase	hsa-miR-18a-5p	MAP3K2	10746	ENSG00000169967	validated
tarbase	hsa-miR-212-3p	MAP3K2	10746	ENSG00000169967	validated
tarbase	hsa-miR-192-5p	ZNF35	7584	ENSG00000169981	validated
tarbase	hsa-miR-326	ZNF35	7584	ENSG00000169981	validated
tarbase	hsa-miR-212-3p	TIGD4	201798	ENSG00000169989	validated
tarbase	hsa-miR-181a-5p	IFFO2	126917	ENSG00000169991	validated
tarbase	hsa-miR-582-5p	IFFO2	126917	ENSG00000169991	validated
tarbase	hsa-miR-212-3p	IFFO2	126917	ENSG00000169991	validated
tarbase	hsa-miR-15b-5p	NLGN2	57555	ENSG00000169992	validated
tarbase	hsa-miR-296-5p	NLGN2	57555	ENSG00000169992	validated
tarbase	hsa-miR-15b-5p	CHD3	1107	ENSG00000170004	validated
tarbase	hsa-miR-212-3p	TMEM154	201799	ENSG00000170006	validated
tarbase	hsa-miR-15b-5p	ALCAM	214	ENSG00000170017	validated
tarbase	hsa-miR-18a-5p	ALCAM	214	ENSG00000170017	validated

tarbase	hsa-miR-505-3p	ALCAM	214	ENSG00000170017	validated
tarbase	hsa-miR-106b-5p	ALCAM	214	ENSG00000170017	validated
tarbase	hsa-miR-15b-5p	YWHAG	7532	ENSG00000170027	validated
tarbase	hsa-miR-181a-5p	YWHAG	7532	ENSG00000170027	validated
tarbase	hsa-miR-212-3p	YWHAG	7532	ENSG00000170027	validated
tarbase	hsa-miR-582-3p	YWHAG	7532	ENSG00000170027	validated
tarbase	hsa-miR-296-5p	TRAPPC1	58485	ENSG00000170043	validated
tarbase	hsa-miR-222-3p	KCNAB3	9196	ENSG00000170049	validated
tarbase	hsa-miR-212-3p	SERPINA9	327657	ENSG00000170054	validated
tarbase	hsa-miR-106b-5p	TMEM192	201931	ENSG00000170088	validated
tarbase	hsa-miR-18a-5p	ZNF778	197320	ENSG00000170100	validated
tarbase	hsa-miR-181a-5p	NIPA1	123606	ENSG00000170113	validated
tarbase	hsa-miR-15b-5p	GPR25	2848	ENSG00000170128	validated
tarbase	hsa-miR-106b-5p	UBE2E1	7324	ENSG00000170142	validated
tarbase	hsa-miR-582-5p	UBE2E1	7324	ENSG00000170142	validated
tarbase	hsa-miR-106b-5p	HNRNPA3	220988	ENSG00000170144	validated
tarbase	hsa-miR-21-5p	HNRNPA3	220988	ENSG00000170144	validated
tarbase	hsa-miR-106b-5p	RNF150	57484	ENSG00000170153	validated
tarbase	hsa-miR-181a-5p	RNF150	57484	ENSG00000170153	validated
tarbase	hsa-miR-15b-5p	USP38	84640	ENSG00000170185	validated
tarbase	hsa-miR-181a-5p	USP38	84640	ENSG00000170185	validated
tarbase	hsa-miR-212-3p	USP38	84640	ENSG00000170185	validated
tarbase	hsa-miR-192-5p	PWWP2A	114825	ENSG00000170234	validated
tarbase	hsa-miR-144-3p	USP47	55031	ENSG00000170242	validated
tarbase	hsa-miR-582-5p	USP47	55031	ENSG00000170242	validated
tarbase	hsa-miR-301a-3p	FAM161A	84140	ENSG00000170264	validated
tarbase	hsa-miR-15b-5p	ELP5	23587	ENSG00000170291	validated
tarbase	hsa-miR-192-5p	CDK1	983	ENSG00000170312	validated
tarbase	hsa-miR-222-3p	CDK1	983	ENSG00000170312	validated
tarbase	hsa-miR-582-5p	CDK1	983	ENSG00000170312	validated
tarbase	hsa-miR-212-3p	UBB	7314	ENSG00000170315	validated
tarbase	hsa-miR-18a-5p	UBB	7314	ENSG00000170315	validated
tarbase	hsa-miR-192-5p	UBB	7314	ENSG00000170315	validated
tarbase	hsa-miR-181a-5p	B3GNT2	10678	ENSG00000170340	validated
tarbase	hsa-miR-212-3p	FOS	2353	ENSG00000170345	validated
tarbase	hsa-miR-15b-5p	TMED10	10972	ENSG00000170348	validated
tarbase	hsa-miR-181a-5p	TMED10	10972	ENSG00000170348	validated
tarbase	hsa-miR-21-5p	TMED10	10972	ENSG00000170348	validated
tarbase	hsa-miR-296-5p	TMED10	10972	ENSG00000170348	validated
tarbase	hsa-miR-582-3p	TMED10	10972	ENSG00000170348	validated
tarbase	hsa-miR-181a-5p	SMAD1	4086	ENSG00000170365	validated
tarbase	hsa-miR-212-3p	EMX2	2018	ENSG00000170370	validated
tarbase	hsa-miR-144-3p	SLC30A1	7779	ENSG00000170385	validated
tarbase	hsa-miR-18a-5p	SLC30A1	7779	ENSG00000170385	validated
tarbase	hsa-miR-222-3p	SLC30A1	7779	ENSG00000170385	validated
tarbase	hsa-miR-505-3p	SLC30A1	7779	ENSG00000170385	validated
tarbase	hsa-miR-212-3p	ZNF804A	91752	ENSG00000170396	validated
tarbase	hsa-miR-212-3p	KRT8	3856	ENSG00000170421	validated
tarbase	hsa-miR-15b-5p	ADORA2B	136	ENSG00000170425	validated
tarbase	hsa-miR-21-5p	METTL7B	196410	ENSG00000170439	validated
tarbase	hsa-miR-181a-5p	DENND5B	160518	ENSG00000170456	validated
tarbase	hsa-miR-212-3p	DENND5B	160518	ENSG00000170456	validated
tarbase	hsa-miR-181a-5p	RIOX1	79697	ENSG00000170468	validated
tarbase	hsa-miR-181a-5p	RALGAPB	57148	ENSG00000170471	validated
tarbase	hsa-miR-212-3p	RALGAPB	57148	ENSG00000170471	validated
tarbase	hsa-miR-301a-3p	RALGAPB	57148	ENSG00000170471	validated
tarbase	hsa-miR-15b-5p	PYM1	84305	ENSG00000170473	validated
tarbase	hsa-miR-212-3p	MZB1	51237	ENSG00000170476	validated
tarbase	hsa-miR-181a-5p	LONRF2	164832	ENSG00000170500	validated
tarbase	hsa-miR-296-5p	LONRF2	164832	ENSG00000170500	validated
tarbase	hsa-miR-326	LONRF2	164832	ENSG00000170500	validated
tarbase	hsa-miR-582-5p	LONRF2	164832	ENSG00000170500	validated
tarbase	hsa-miR-181a-5p	ELOVL6	79071	ENSG00000170522	validated
tarbase	hsa-miR-106b-5p	PFKFB3	5209	ENSG00000170525	validated
tarbase	hsa-miR-301a-3p	PFKFB3	5209	ENSG00000170525	validated
tarbase	hsa-miR-15b-5p	ARL6IP1	23204	ENSG00000170540	validated
tarbase	hsa-miR-181a-5p	ARL6IP1	23204	ENSG00000170540	validated
tarbase	hsa-miR-18a-5p	ARL6IP1	23204	ENSG00000170540	validated
tarbase	hsa-miR-582-5p	ARL6IP1	23204	ENSG00000170540	validated
tarbase	hsa-miR-15b-5p	SERPINB9	5272	ENSG00000170542	validated
tarbase	hsa-miR-222-3p	CDH2	1000	ENSG00000170558	validated

tarbase	hsa-miR-582-3p	CDH2	1000	ENSG00000170558	validated
tarbase	hsa-miR-582-5p	CDH2	1000	ENSG00000170558	validated
tarbase	hsa-miR-15b-5p	EMB	133418	ENSG00000170571	validated
tarbase	hsa-miR-181a-5p	DLGAP1	9229	ENSG00000170579	validated
tarbase	hsa-miR-15b-5p	IRF2BP1	26145	ENSG00000170604	validated
tarbase	hsa-miR-21-5p	HSPA4	3308	ENSG00000170606	validated
tarbase	hsa-miR-212-3p	FAM71B	153745	ENSG00000170613	validated
tarbase	hsa-miR-21-5p	GTSF1	121355	ENSG00000170627	validated
tarbase	hsa-miR-181a-5p	ARMC10	83787	ENSG00000170632	validated
tarbase	hsa-miR-301a-3p	RNF34	80196	ENSG00000170633	validated
tarbase	hsa-miR-505-3p	RNF34	80196	ENSG00000170633	validated
tarbase	hsa-miR-15b-5p	ATF7	11016	ENSG00000170653	validated
tarbase	hsa-miR-301a-3p	ATF7	11016	ENSG00000170653	validated
tarbase	hsa-miR-15b-5p	SOCS6	9306	ENSG00000170677	validated
tarbase	hsa-miR-18a-5p	SOCS6	9306	ENSG00000170677	validated
tarbase	hsa-miR-192-5p	SOCS6	9306	ENSG00000170677	validated
tarbase	hsa-miR-301a-3p	SOCS6	9306	ENSG00000170677	validated
tarbase	hsa-miR-582-5p	SOCS6	9306	ENSG00000170677	validated
tarbase	hsa-miR-18a-5p	HOXB9	3219	ENSG00000170689	validated
tarbase	hsa-miR-582-5p	POLH	5429	ENSG00000170734	validated
tarbase	hsa-miR-106b-5p	KIF5B	3799	ENSG00000170759	validated
tarbase	hsa-miR-144-3p	KIF5B	3799	ENSG00000170759	validated
tarbase	hsa-miR-21-5p	KIF5B	3799	ENSG00000170759	validated
tarbase	hsa-miR-582-3p	KIF5B	3799	ENSG00000170759	validated
tarbase	hsa-miR-18a-5p	KIF5B	3799	ENSG00000170759	validated
tarbase	hsa-miR-106b-5p	CDCA4	55038	ENSG00000170779	validated
tarbase	hsa-miR-181a-5p	CDCA4	55038	ENSG00000170779	validated
tarbase	hsa-miR-18a-5p	CDCA4	55038	ENSG00000170779	validated
tarbase	hsa-miR-505-3p	CDCA4	55038	ENSG00000170779	validated
tarbase	hsa-miR-18a-5p	FOXN2	3344	ENSG00000170802	validated
tarbase	hsa-miR-222-3p	FOXN2	3344	ENSG00000170802	validated
tarbase	hsa-miR-301a-3p	FOXN2	3344	ENSG00000170802	validated
tarbase	hsa-miR-582-5p	FOXN2	3344	ENSG00000170802	validated
tarbase	hsa-miR-212-3p	CEL	1056	ENSG00000170835	validated
tarbase	hsa-miR-18a-5p	PPM1D	8493	ENSG00000170836	validated
tarbase	hsa-miR-21-5p	PPM1D	8493	ENSG00000170836	validated
tarbase	hsa-miR-15b-5p	KBTBD2	25948	ENSG00000170852	validated
tarbase	hsa-miR-181a-5p	KBTBD2	25948	ENSG00000170852	validated
tarbase	hsa-miR-106b-5p	KBTBD2	25948	ENSG00000170852	validated
tarbase	hsa-miR-21-5p	TRIAP1	51499	ENSG00000170855	validated
tarbase	hsa-miR-582-5p	TRIAP1	51499	ENSG00000170855	validated
tarbase	hsa-miR-15b-5p	TRIAP1	51499	ENSG00000170855	validated
tarbase	hsa-miR-21-5p	LSM3	27258	ENSG00000170860	validated
tarbase	hsa-miR-15b-5p	KIAA0232	9778	ENSG00000170871	validated
tarbase	hsa-miR-326	RNF139	11236	ENSG00000170881	validated
tarbase	hsa-miR-582-5p	RNF139	11236	ENSG00000170881	validated
tarbase	hsa-miR-18a-5p	RPS9	6203	ENSG00000170889	validated
tarbase	hsa-miR-181a-5p	MSANTD4	84437	ENSG00000170903	validated
tarbase	hsa-miR-15b-5p	TANC2	26115	ENSG00000170921	validated
tarbase	hsa-miR-15b-5p	DNAJC24	120526	ENSG00000170946	validated
tarbase	hsa-miR-18a-5p	ZNF160	90338	ENSG00000170949	validated
tarbase	hsa-miR-192-5p	ZNF160	90338	ENSG00000170949	validated
tarbase	hsa-miR-181a-5p	ZNF160	90338	ENSG00000170949	validated
tarbase	hsa-miR-181a-5p	HAS2	3037	ENSG00000170961	validated
tarbase	hsa-miR-222-3p	PDGFD	80310	ENSG00000170962	validated
tarbase	hsa-miR-106b-5p	S1PR1	1901	ENSG00000170989	validated
tarbase	hsa-miR-15b-5p	S1PR1	1901	ENSG00000170989	validated
tarbase	hsa-miR-181a-5p	S1PR1	1901	ENSG00000170989	validated
tarbase	hsa-miR-301a-3p	S1PR1	1901	ENSG00000170989	validated
tarbase	hsa-miR-582-5p	HS6ST2	90161	ENSG00000171004	validated
tarbase	hsa-miR-18a-5p	PYGO1	26108	ENSG00000171016	validated
tarbase	hsa-miR-106b-5p	PKIA	5569	ENSG00000171033	validated
tarbase	hsa-miR-181a-5p	PKIA	5569	ENSG00000171033	validated
tarbase	hsa-miR-212-3p	PKIA	5569	ENSG00000171033	validated
tarbase	hsa-miR-301a-3p	FEZ2	9637	ENSG00000171055	validated
tarbase	hsa-miR-18a-5p	KCNG3	170850	ENSG00000171126	validated
tarbase	hsa-miR-181a-5p	ATP6V0E2	155066	ENSG00000171130	validated
tarbase	hsa-miR-181a-5p	PRKCE	5581	ENSG00000171132	validated
tarbase	hsa-miR-192-5p	JAGN1	84522	ENSG00000171135	validated
tarbase	hsa-miR-18a-5p	SOCS5	9655	ENSG00000171150	validated
tarbase	hsa-miR-212-3p	SOCS5	9655	ENSG00000171150	validated

tarbase	hsa-miR-181a-5p	C9orf16	79095	ENSG00000171159	validated
tarbase	hsa-miR-301a-3p	ZNF692	55657	ENSG00000171163	validated
tarbase	hsa-miR-181a-5p	NETO2	81831	ENSG00000171208	validated
tarbase	hsa-miR-181a-5p	JUNB	3726	ENSG00000171223	validated
tarbase	hsa-miR-212-3p	LRG1	116844	ENSG00000171236	validated
tarbase	hsa-miR-582-3p	SHCBP1	79801	ENSG00000171241	validated
tarbase	hsa-miR-106b-5p	SHCBP1	79801	ENSG00000171241	validated
tarbase	hsa-miR-181a-5p	FAM98B	283742	ENSG00000171262	validated
tarbase	hsa-miR-15b-5p	CHD7	55636	ENSG00000171316	validated
tarbase	hsa-miR-181a-5p	CHD7	55636	ENSG00000171316	validated
tarbase	hsa-miR-326	CHD7	55636	ENSG00000171316	validated
tarbase	hsa-miR-582-5p	CHD7	55636	ENSG00000171316	validated
tarbase	hsa-miR-301a-3p	ESCO2	157570	ENSG00000171320	validated
tarbase	hsa-miR-15b-5p	CLCN5	1184	ENSG00000171365	validated
tarbase	hsa-miR-301a-3p	CLCN5	1184	ENSG00000171365	validated
tarbase	hsa-miR-582-5p	PDE7B	27115	ENSG00000171408	validated
tarbase	hsa-miR-212-3p	KRT20	54474	ENSG00000171431	validated
tarbase	hsa-miR-181a-5p	KSR2	283455	ENSG00000171435	validated
tarbase	hsa-miR-301a-3p	DSEL	92126	ENSG00000171451	validated
tarbase	hsa-miR-15b-5p	POLR1C	9533	ENSG00000171453	validated
tarbase	hsa-miR-192-5p	POLR1C	9533	ENSG00000171453	validated
tarbase	hsa-miR-21-5p	POLR1C	9533	ENSG00000171453	validated
tarbase	hsa-miR-181a-5p	ASXL1	171023	ENSG00000171456	validated
tarbase	hsa-miR-326	ASXL1	171023	ENSG00000171456	validated
tarbase	hsa-miR-181a-5p	ZNF561	93134	ENSG00000171469	validated
tarbase	hsa-miR-582-3p	WIPF2	147179	ENSG00000171475	validated
tarbase	hsa-miR-181a-5p	LRRC8C	84230	ENSG00000171488	validated
tarbase	hsa-miR-181a-5p	RSL1D1	26156	ENSG00000171490	validated
tarbase	hsa-miR-326	RSL1D1	26156	ENSG00000171490	validated
tarbase	hsa-miR-106b-5p	LRRC8D	55144	ENSG00000171492	validated
tarbase	hsa-miR-18a-5p	LRRC8D	55144	ENSG00000171492	validated
tarbase	hsa-miR-192-5p	LRRC8D	55144	ENSG00000171492	validated
tarbase	hsa-miR-212-3p	ETFDH	2110	ENSG00000171503	validated
tarbase	hsa-miR-15b-5p	LPAR3	23566	ENSG00000171517	validated
tarbase	hsa-miR-296-5p	TBCA	6902	ENSG00000171530	validated
tarbase	hsa-miR-106b-5p	OTP	23440	ENSG00000171540	validated
tarbase	hsa-miR-21-5p	FGB	2244	ENSG00000171564	validated
tarbase	hsa-miR-106b-5p	CLSTN1	22883	ENSG00000171603	validated
tarbase	hsa-miR-181a-5p	CLSTN1	22883	ENSG00000171603	validated
tarbase	hsa-miR-21-5p	CLSTN1	22883	ENSG00000171603	validated
tarbase	hsa-miR-18a-5p	ENC1	8507	ENSG00000171617	validated
tarbase	hsa-miR-21-5p	ENC1	8507	ENSG00000171617	validated
tarbase	hsa-miR-582-5p	ENC1	8507	ENSG00000171617	validated
tarbase	hsa-miR-15b-5p	ENC1	8507	ENSG00000171617	validated
tarbase	hsa-miR-212-3p	ENC1	8507	ENSG00000171617	validated
tarbase	hsa-miR-106b-5p	BPTF	2186	ENSG00000171634	validated
tarbase	hsa-miR-181a-5p	BPTF	2186	ENSG00000171634	validated
tarbase	hsa-miR-18a-5p	ATF7IP	55729	ENSG00000171681	validated
tarbase	hsa-miR-192-5p	ATF7IP	55729	ENSG00000171681	validated
tarbase	hsa-miR-212-3p	ATF7IP	55729	ENSG00000171681	validated
tarbase	hsa-miR-192-5p	TCEA2	6919	ENSG00000171703	validated
tarbase	hsa-miR-21-5p	ANO5	203859	ENSG00000171714	validated
tarbase	hsa-miR-181a-5p	ANO5	203859	ENSG00000171714	validated
tarbase	hsa-miR-326	HDAC3	8841	ENSG00000171720	validated
tarbase	hsa-miR-144-3p	VAT1L	57687	ENSG00000171724	validated
tarbase	hsa-miR-212-3p	TMEM51	55092	ENSG00000171729	validated
tarbase	hsa-miR-18a-5p	CAMTA1	23261	ENSG00000171735	validated
tarbase	hsa-miR-296-5p	CAMTA1	23261	ENSG00000171735	validated
tarbase	hsa-miR-301a-3p	CAMTA1	23261	ENSG00000171735	validated
tarbase	hsa-miR-181a-5p	GATM	2628	ENSG00000171766	validated
tarbase	hsa-miR-582-5p	BCL2	596	ENSG00000171791	validated
tarbase	hsa-miR-21-5p	CTPS1	1503	ENSG00000171793	validated
tarbase	hsa-miR-296-5p	KNDC1	85442	ENSG00000171798	validated
tarbase	hsa-miR-106b-5p	PWWP2B	170394	ENSG00000171813	validated
tarbase	hsa-miR-15b-5p	PWWP2B	170394	ENSG00000171813	validated
tarbase	hsa-miR-106b-5p	EXOSC10	5394	ENSG00000171824	validated
tarbase	hsa-miR-181a-5p	ZNF570	148268	ENSG00000171827	validated
tarbase	hsa-miR-212-3p	FAM90A1	55138	ENSG00000171847	validated
tarbase	hsa-miR-18a-5p	RRM2	6241	ENSG00000171848	validated
tarbase	hsa-miR-212-3p	RRM2	6241	ENSG00000171848	validated
tarbase	hsa-miR-212-3p	IFNB1	3456	ENSG00000171855	validated

tarbase	hsa-miR-212-3p	C3AR1	719	ENSG00000171860	validated
tarbase	hsa-miR-21-5p	MRM3	55178	ENSG00000171861	validated
tarbase	hsa-miR-582-3p	PTEN	5728	ENSG00000171862	validated
tarbase	hsa-miR-181a-5p	PRNP	5621	ENSG00000171867	validated
tarbase	hsa-miR-106b-5p	AQP4	361	ENSG00000171885	validated
tarbase	hsa-miR-181a-5p	AQP4	361	ENSG00000171885	validated
tarbase	hsa-miR-301a-3p	TVP23B	51030	ENSG00000171928	validated
tarbase	hsa-miR-106b-5p	ZNF217	7764	ENSG00000171940	validated
tarbase	hsa-miR-144-3p	ZNF217	7764	ENSG00000171940	validated
tarbase	hsa-miR-181a-5p	ZNF217	7764	ENSG00000171940	validated
tarbase	hsa-miR-18a-5p	ZNF217	7764	ENSG00000171940	validated
tarbase	hsa-miR-212-3p	ZNF217	7764	ENSG00000171940	validated
tarbase	hsa-miR-301a-3p	ZNF217	7764	ENSG00000171940	validated
tarbase	hsa-miR-582-3p	ZNF217	7764	ENSG00000171940	validated
tarbase	hsa-miR-582-5p	ZNF217	7764	ENSG00000171940	validated
tarbase	hsa-miR-181a-5p	SCG2	7857	ENSG00000171951	validated
tarbase	hsa-miR-106b-5p	FOXB1	27023	ENSG00000171956	validated
tarbase	hsa-miR-181a-5p	FOXB1	27023	ENSG00000171956	validated
tarbase	hsa-miR-212-3p	DRC3	83450	ENSG00000171962	validated
tarbase	hsa-miR-144-3p	JMJD1C	221037	ENSG00000171988	validated
tarbase	hsa-miR-15b-5p	JMJD1C	221037	ENSG00000171988	validated
tarbase	hsa-miR-181a-5p	JMJD1C	221037	ENSG00000171988	validated
tarbase	hsa-miR-21-5p	JMJD1C	221037	ENSG00000171988	validated
tarbase	hsa-miR-582-5p	JMJD1C	221037	ENSG00000171988	validated
tarbase	hsa-miR-212-3p	LDHAL6B	92483	ENSG00000171989	validated
tarbase	hsa-miR-15b-5p	THOP1	7064	ENSG00000172009	validated
tarbase	hsa-miR-301a-3p	THOP1	7064	ENSG00000172009	validated
tarbase	hsa-miR-212-3p	REG1B	5968	ENSG00000172023	validated
tarbase	hsa-miR-222-3p	QARS1	5859	ENSG00000172053	validated
tarbase	hsa-miR-15b-5p	ORMDL3	94103	ENSG00000172057	validated
tarbase	hsa-miR-222-3p	ORMDL3	94103	ENSG00000172057	validated
tarbase	hsa-miR-181a-5p	ORMDL3	94103	ENSG00000172057	validated
tarbase	hsa-miR-106b-5p	KLF11	8462	ENSG00000172059	validated
tarbase	hsa-miR-181a-5p	EIF2AK3	9451	ENSG00000172071	validated
tarbase	hsa-miR-21-5p	EIF2AK3	9451	ENSG00000172071	validated
tarbase	hsa-miR-212-3p	TEX37	200523	ENSG00000172073	validated
tarbase	hsa-miR-212-3p	KRCC1	51315	ENSG00000172086	validated
tarbase	hsa-miR-181a-5p	NME6	10201	ENSG00000172113	validated
tarbase	hsa-miR-326	NME6	10201	ENSG00000172113	validated
tarbase	hsa-miR-582-5p	CYCS	54205	ENSG00000172115	validated
tarbase	hsa-miR-181a-5p	CYCS	54205	ENSG00000172115	validated
tarbase	hsa-miR-212-3p	OR1A2	26189	ENSG00000172150	validated
tarbase	hsa-miR-181a-5p	MRPL13	28998	ENSG00000172172	validated
tarbase	hsa-miR-212-3p	ISG20	3669	ENSG00000172183	validated
tarbase	hsa-miR-181a-5p	NEGR1	257194	ENSG00000172260	validated
tarbase	hsa-miR-301a-3p	ZNF131	7690	ENSG00000172262	validated
tarbase	hsa-miR-181a-5p	ZNF131	7690	ENSG00000172262	validated
tarbase	hsa-miR-212-3p	MACROD2	140733	ENSG00000172264	validated
tarbase	hsa-miR-106b-5p	CERS6	253782	ENSG00000172292	validated
tarbase	hsa-miR-21-5p	POP7	10248	ENSG00000172336	validated
tarbase	hsa-miR-582-3p	POP7	10248	ENSG00000172336	validated
tarbase	hsa-miR-212-3p	ALG14	199857	ENSG00000172339	validated
tarbase	hsa-miR-15b-5p	GNB2	2783	ENSG00000172354	validated
tarbase	hsa-miR-181a-5p	ARNT2	9915	ENSG00000172379	validated
tarbase	hsa-miR-212-3p	ARNT2	9915	ENSG00000172379	validated
tarbase	hsa-miR-106b-5p	GNG12	55970	ENSG00000172380	validated
tarbase	hsa-miR-18a-5p	GNG12	55970	ENSG00000172380	validated
tarbase	hsa-miR-21-5p	GNG12	55970	ENSG00000172380	validated
tarbase	hsa-miR-222-3p	GNG12	55970	ENSG00000172380	validated
tarbase	hsa-miR-301a-3p	GNG12	55970	ENSG00000172380	validated
tarbase	hsa-miR-582-5p	GNG12	55970	ENSG00000172380	validated
tarbase	hsa-miR-212-3p	PRSS27	83886	ENSG00000172382	validated
tarbase	hsa-miR-181a-5p	GTPBP2	54676	ENSG00000172432	validated
tarbase	hsa-miR-212-3p	GTPBP2	54676	ENSG00000172432	validated
tarbase	hsa-miR-181a-5p	FUT9	10690	ENSG00000172461	validated
tarbase	hsa-miR-106b-5p	ZNF24	7572	ENSG00000172466	validated
tarbase	hsa-miR-18a-5p	ZNF24	7572	ENSG00000172466	validated
tarbase	hsa-miR-212-3p	ZNF24	7572	ENSG00000172466	validated
tarbase	hsa-miR-582-5p	ZNF24	7572	ENSG00000172466	validated
tarbase	hsa-miR-181a-5p	ZNF24	7572	ENSG00000172466	validated
tarbase	hsa-miR-212-3p	RAB40A	142684	ENSG00000172476	validated

tarbase	hsa-miR-212-3p	BANP	54971	ENSG00000172530	validated
tarbase	hsa-miR-18a-5p	HCFC1	3054	ENSG00000172534	validated
tarbase	hsa-miR-326	HCFC1	3054	ENSG00000172534	validated
tarbase	hsa-miR-505-3p	PDE3A	5139	ENSG00000172572	validated
tarbase	hsa-miR-582-3p	PDE3A	5139	ENSG00000172572	validated
tarbase	hsa-miR-21-5p	KLHL6	89857	ENSG00000172578	validated
tarbase	hsa-miR-181a-5p	CHCHD1	118487	ENSG00000172586	validated
tarbase	hsa-miR-15b-5p	TMEM134	80194	ENSG00000172663	validated
tarbase	hsa-miR-18a-5p	ZMAT3	64393	ENSG00000172667	validated
tarbase	hsa-miR-21-5p	ZMAT3	64393	ENSG00000172667	validated
tarbase	hsa-miR-106b-5p	ZNF738	148203	ENSG00000172687	validated
tarbase	hsa-miR-15b-5p	SLFN11	91607	ENSG00000172716	validated
tarbase	hsa-miR-181a-5p	SLFN11	91607	ENSG00000172716	validated
tarbase	hsa-miR-21-5p	SLFN11	91607	ENSG00000172716	validated
tarbase	hsa-miR-18a-5p	SLFN11	91607	ENSG00000172716	validated
tarbase	hsa-miR-222-3p	FUT10	84750	ENSG00000172728	validated
tarbase	hsa-miR-212-3p	ZNF596	169270	ENSG00000172748	validated
tarbase	hsa-miR-181a-5p	CFL1	1072	ENSG00000172757	validated
tarbase	hsa-miR-21-5p	CFL1	1072	ENSG00000172757	validated
tarbase	hsa-miR-505-3p	CFL1	1072	ENSG00000172757	validated
tarbase	hsa-miR-106b-5p	TMCC1	23023	ENSG00000172765	validated
tarbase	hsa-miR-15b-5p	TMCC1	23023	ENSG00000172765	validated
tarbase	hsa-miR-18a-5p	PSME3IP1	80011	ENSG00000172775	validated
tarbase	hsa-miR-144-3p	DCP2	167227	ENSG00000172795	validated
tarbase	hsa-miR-15b-5p	DCP2	167227	ENSG00000172795	validated
tarbase	hsa-miR-181a-5p	DCP2	167227	ENSG00000172795	validated
tarbase	hsa-miR-212-3p	DCP2	167227	ENSG00000172795	validated
tarbase	hsa-miR-301a-3p	DCP2	167227	ENSG00000172795	validated
tarbase	hsa-miR-15b-5p	PDP2	57546	ENSG00000172840	validated
tarbase	hsa-miR-15b-5p	SP3	6670	ENSG00000172845	validated
tarbase	hsa-miR-181a-5p	SP3	6670	ENSG00000172845	validated
tarbase	hsa-miR-192-5p	SP3	6670	ENSG00000172845	validated
tarbase	hsa-miR-212-3p	SP3	6670	ENSG00000172845	validated
tarbase	hsa-miR-21-5p	SP3	6670	ENSG00000172845	validated
tarbase	hsa-miR-582-5p	SP3	6670	ENSG00000172845	validated
tarbase	hsa-miR-18a-5p	DMXL1	1657	ENSG00000172869	validated
tarbase	hsa-miR-21-5p	DMXL1	1657	ENSG00000172869	validated
tarbase	hsa-miR-181a-5p	DMXL1	1657	ENSG00000172869	validated
tarbase	hsa-miR-212-3p	METAP1D	254042	ENSG00000172878	validated
tarbase	hsa-miR-181a-5p	ZNF621	285268	ENSG00000172888	validated
tarbase	hsa-miR-582-3p	ZNF621	285268	ENSG00000172888	validated
tarbase	hsa-miR-15b-5p	NBEA	26960	ENSG00000172915	validated
tarbase	hsa-miR-15b-5p	MYD88	4615	ENSG00000172936	validated
tarbase	hsa-miR-21-5p	XKR3	150165	ENSG00000172967	validated
tarbase	hsa-miR-18a-5p	DCAKD	79877	ENSG00000172992	validated
tarbase	hsa-miR-181a-5p	TADA2B	93624	ENSG00000173011	validated
tarbase	hsa-miR-301a-3p	GRK2	156	ENSG00000173020	validated
tarbase	hsa-miR-106b-5p	ZNF680	340252	ENSG00000173041	validated
tarbase	hsa-miR-582-3p	ZNF680	340252	ENSG00000173041	validated
tarbase	hsa-miR-15b-5p	HECTD4	283450	ENSG00000173064	validated
tarbase	hsa-miR-582-3p	HECTD4	283450	ENSG00000173064	validated
tarbase	hsa-miR-222-3p	FAM222B	55731	ENSG00000173065	validated
tarbase	hsa-miR-106b-5p	BNC2	54796	ENSG00000173068	validated
tarbase	hsa-miR-15b-5p	BNC2	54796	ENSG00000173068	validated
tarbase	hsa-miR-18a-5p	RXFP4	339403	ENSG00000173080	validated
tarbase	hsa-miR-18a-5p	HPSE	10855	ENSG00000173083	validated
tarbase	hsa-miR-106b-5p	COQ2	27235	ENSG00000173085	validated
tarbase	hsa-miR-18a-5p	LRRN3	54674	ENSG00000173114	validated
tarbase	hsa-miR-106b-5p	KDM2A	22992	ENSG00000173120	validated
tarbase	hsa-miR-301a-3p	KDM2A	22992	ENSG00000173120	validated
tarbase	hsa-miR-326	KDM2A	22992	ENSG00000173120	validated
tarbase	hsa-miR-582-5p	KDM2A	22992	ENSG00000173120	validated
tarbase	hsa-miR-212-3p	ADCK5	203054	ENSG00000173137	validated
tarbase	hsa-miR-106b-5p	RAPH1	65059	ENSG00000173166	validated
tarbase	hsa-miR-222-3p	RAPH1	65059	ENSG00000173166	validated
tarbase	hsa-miR-296-5p	RAPH1	65059	ENSG00000173166	validated
tarbase	hsa-miR-212-3p	PARP14	54625	ENSG00000173193	validated
tarbase	hsa-miR-106b-5p	ABCD2	225	ENSG00000173208	validated
tarbase	hsa-miR-192-5p	AHSA2P		ENSG00000173209	validated
tarbase	hsa-miR-21-5p	AHSA2P		ENSG00000173209	validated
tarbase	hsa-miR-106b-5p	VANGL1	81839	ENSG00000173218	validated

tarbase	hsa-miR-301a-3p	VANGL1	81839	ENSG00000173218	validated
tarbase	hsa-miR-582-5p	VANGL1	81839	ENSG00000173218	validated
tarbase	hsa-miR-21-5p	IQCB1	9657	ENSG00000173226	validated
tarbase	hsa-miR-106b-5p	GOLGB1	2804	ENSG00000173230	validated
tarbase	hsa-miR-15b-5p	GOLGB1	2804	ENSG00000173230	validated
tarbase	hsa-miR-21-5p	GOLGB1	2804	ENSG00000173230	validated
tarbase	hsa-miR-181a-5p	ZNF483	158399	ENSG00000173258	validated
tarbase	hsa-miR-15b-5p	TNKS	8658	ENSG00000173273	validated
tarbase	hsa-miR-212-3p	TNKS	8658	ENSG00000173273	validated
tarbase	hsa-miR-106b-5p	ZBTB21	49854	ENSG00000173276	validated
tarbase	hsa-miR-15b-5p	ZBTB21	49854	ENSG00000173276	validated
tarbase	hsa-miR-181a-5p	ZBTB21	49854	ENSG00000173276	validated
tarbase	hsa-miR-18a-5p	ZBTB21	49854	ENSG00000173276	validated
tarbase	hsa-miR-301a-3p	ZBTB21	49854	ENSG00000173276	validated
tarbase	hsa-miR-181a-5p	PPP1R3B	79660	ENSG00000173281	validated
tarbase	hsa-miR-21-5p	PPP1R3B	79660	ENSG00000173281	validated
tarbase	hsa-miR-106b-5p	TRIB1	10221	ENSG00000173334	validated
tarbase	hsa-miR-212-3p	TRIB1	10221	ENSG00000173334	validated
tarbase	hsa-miR-301a-3p	TRIB1	10221	ENSG00000173334	validated
tarbase	hsa-miR-582-3p	OLR1	4973	ENSG00000173391	validated
tarbase	hsa-miR-582-5p	DAG1	1605	ENSG00000173402	validated
tarbase	hsa-miR-21-5p	DAG1	1605	ENSG00000173402	validated
tarbase	hsa-miR-181a-5p	ARV1	64801	ENSG00000173409	validated
tarbase	hsa-miR-15b-5p	NAA20	51126	ENSG00000173418	validated
tarbase	hsa-miR-181a-5p	NAA20	51126	ENSG00000173418	validated
tarbase	hsa-miR-582-5p	CCDC36	339834	ENSG00000173421	validated
tarbase	hsa-miR-582-5p	THAP2	83591	ENSG00000173451	validated
tarbase	hsa-miR-212-3p	THAP2	83591	ENSG00000173451	validated
tarbase	hsa-miR-18a-5p	SMARCC1	6599	ENSG00000173473	validated
tarbase	hsa-miR-18a-5p	ZNF417	147687	ENSG00000173480	validated
tarbase	hsa-miR-222-3p	PTPRM	5797	ENSG00000173482	validated
tarbase	hsa-miR-181a-5p	PEAK1	79834	ENSG00000173517	validated
tarbase	hsa-miR-582-3p	PEAK1	79834	ENSG00000173517	validated
tarbase	hsa-miR-582-5p	PEAK1	79834	ENSG00000173517	validated
tarbase	hsa-miR-582-5p	TNFRSF10D	8793	ENSG00000173530	validated
tarbase	hsa-miR-212-3p	TNFRSF10D	8793	ENSG00000173530	validated
tarbase	hsa-miR-106b-5p	MOB1B	92597	ENSG00000173542	validated
tarbase	hsa-miR-18a-5p	MOB1B	92597	ENSG00000173542	validated
tarbase	hsa-miR-106b-5p	ZNF622	90441	ENSG00000173545	validated
tarbase	hsa-miR-181a-5p	ZNF622	90441	ENSG00000173545	validated
tarbase	hsa-miR-106b-5p	CHD2	1106	ENSG00000173575	validated
tarbase	hsa-miR-15b-5p	CHD2	1106	ENSG00000173575	validated
tarbase	hsa-miR-181a-5p	CHD2	1106	ENSG00000173575	validated
tarbase	hsa-miR-326	CHD2	1106	ENSG00000173575	validated
tarbase	hsa-miR-15b-5p	CEP83	51134	ENSG00000173588	validated
tarbase	hsa-miR-582-5p	CEP83	51134	ENSG00000173588	validated
tarbase	hsa-miR-15b-5p	SCAI	286205	ENSG00000173611	validated
tarbase	hsa-miR-505-3p	SCAI	286205	ENSG00000173611	validated
tarbase	hsa-miR-15b-5p	RCE1	9986	ENSG00000173653	validated
tarbase	hsa-miR-15b-5p	UQCRH	7388	ENSG00000173660	validated
tarbase	hsa-miR-181a-5p	EIF1AX	1964	ENSG00000173674	validated
tarbase	hsa-miR-326	EIF1AX	1964	ENSG00000173674	validated
tarbase	hsa-miR-582-5p	EIF1AX	1964	ENSG00000173674	validated
tarbase	hsa-miR-181a-5p	BCLAF3	256643	ENSG00000173681	validated
tarbase	hsa-miR-212-3p	BCLAF3	256643	ENSG00000173681	validated
tarbase	hsa-miR-301a-3p	PSMD1	5707	ENSG00000173692	validated
tarbase	hsa-miR-582-3p	PSMD1	5707	ENSG00000173692	validated
tarbase	hsa-miR-106b-5p	HEG1	57493	ENSG00000173706	validated
tarbase	hsa-miR-181a-5p	HEG1	57493	ENSG00000173706	validated
tarbase	hsa-miR-222-3p	HEG1	57493	ENSG00000173706	validated
tarbase	hsa-miR-326	C11orf80	79703	ENSG00000173715	validated
tarbase	hsa-miR-15b-5p	TOMM20	9804	ENSG00000173726	validated
tarbase	hsa-miR-181a-5p	TOMM20	9804	ENSG00000173726	validated
tarbase	hsa-miR-582-5p	TOMM20	9804	ENSG00000173726	validated
tarbase	hsa-miR-106b-5p	AGFG1	3267	ENSG00000173744	validated
tarbase	hsa-miR-192-5p	AGFG1	3267	ENSG00000173744	validated
tarbase	hsa-miR-301a-3p	AGFG1	3267	ENSG00000173744	validated
tarbase	hsa-miR-15b-5p	JUP	3728	ENSG00000173801	validated
tarbase	hsa-miR-181a-5p	EIF1	10209	ENSG00000173812	validated
tarbase	hsa-miR-18a-5p	EIF1	10209	ENSG00000173812	validated
tarbase	hsa-miR-222-3p	EIF1	10209	ENSG00000173812	validated

tarbase	hsa-miR-582-5p	EIF1	10209	ENSG00000173812	validated
tarbase	hsa-miR-15b-5p	ENDOV	284131	ENSG00000173818	validated
tarbase	hsa-miR-15b-5p	RNF213	57674	ENSG00000173821	validated
tarbase	hsa-miR-18a-5p	RNF213	57674	ENSG00000173821	validated
tarbase	hsa-miR-21-5p	RNF213	57674	ENSG00000173821	validated
tarbase	hsa-miR-505-3p	RNF213	57674	ENSG00000173821	validated
tarbase	hsa-miR-15b-5p	NET1	10276	ENSG00000173848	validated
tarbase	hsa-miR-212-3p	NET1	10276	ENSG00000173848	validated
tarbase	hsa-miR-301a-3p	DPY19L1	23333	ENSG00000173852	validated
tarbase	hsa-miR-181a-5p	DPY19L1	23333	ENSG00000173852	validated
tarbase	hsa-miR-21-5p	ZNF791	163049	ENSG00000173875	validated
tarbase	hsa-miR-106b-5p	PHC3	80012	ENSG00000173889	validated
tarbase	hsa-miR-18a-5p	PHC3	80012	ENSG00000173889	validated
tarbase	hsa-miR-212-3p	PHC3	80012	ENSG00000173889	validated
tarbase	hsa-miR-21-5p	PHC3	80012	ENSG00000173889	validated
tarbase	hsa-miR-582-3p	PHC3	80012	ENSG00000173889	validated
tarbase	hsa-miR-15b-5p	SPTBN2	6712	ENSG00000173898	validated
tarbase	hsa-miR-15b-5p	GOLIM4	27333	ENSG00000173905	validated
tarbase	hsa-miR-181a-5p	GOLIM4	27333	ENSG00000173905	validated
tarbase	hsa-miR-15b-5p	RBM4B	83759	ENSG00000173914	validated
tarbase	hsa-miR-212-3p	RBM4B	83759	ENSG00000173914	validated
tarbase	hsa-miR-106b-5p	HOXB2	3212	ENSG00000173917	validated
tarbase	hsa-miR-181a-5p	MARCHF3	115123	ENSG00000173926	validated
tarbase	hsa-miR-15b-5p	RBM4	5936	ENSG00000173933	validated
tarbase	hsa-miR-181a-5p	RBM4	5936	ENSG00000173933	validated
tarbase	hsa-miR-222-3p	RBM4	5936	ENSG00000173933	validated
tarbase	hsa-miR-301a-3p	RBM4	5936	ENSG00000173933	validated
tarbase	hsa-miR-301a-3p	UBXN2A	165324	ENSG00000173960	validated
tarbase	hsa-miR-18a-5p	KLHL15	80311	ENSG00000174010	validated
tarbase	hsa-miR-301a-3p	KLHL15	80311	ENSG00000174010	validated
tarbase	hsa-miR-582-3p	KLHL15	80311	ENSG00000174010	validated
tarbase	hsa-miR-505-3p	KLHL15	80311	ENSG00000174010	validated
tarbase	hsa-miR-144-3p	FBXO45	200933	ENSG00000174013	validated
tarbase	hsa-miR-181a-5p	FBXO45	200933	ENSG00000174013	validated
tarbase	hsa-miR-181a-5p	SLC25A30	253512	ENSG00000174032	validated
tarbase	hsa-miR-15b-5p	LEMD3	23592	ENSG00000174106	validated
tarbase	hsa-miR-181a-5p	LEMD3	23592	ENSG00000174106	validated
tarbase	hsa-miR-18a-5p	FAM174A	345757	ENSG00000174132	validated
tarbase	hsa-miR-582-5p	FAM174A	345757	ENSG00000174132	validated
tarbase	hsa-miR-144-3p	RGMB	285704	ENSG00000174136	validated
tarbase	hsa-miR-18a-5p	RGMB	285704	ENSG00000174136	validated
tarbase	hsa-miR-222-3p	RGMB	285704	ENSG00000174136	validated
tarbase	hsa-miR-582-3p	RGMB	285704	ENSG00000174136	validated
tarbase	hsa-miR-181a-5p	RGMB	285704	ENSG00000174136	validated
tarbase	hsa-miR-212-3p	NWD2	57495	ENSG00000174145	validated
tarbase	hsa-miR-106b-5p	CYB561D1	284613	ENSG00000174151	validated
tarbase	hsa-miR-21-5p	CYB561D1	284613	ENSG00000174151	validated
tarbase	hsa-miR-582-3p	CYB561D1	284613	ENSG00000174151	validated
tarbase	hsa-miR-212-3p	CYB561D1	284613	ENSG00000174151	validated
tarbase	hsa-miR-15b-5p	MGA	23269	ENSG00000174197	validated
tarbase	hsa-miR-181a-5p	MGA	23269	ENSG00000174197	validated
tarbase	hsa-miR-326	MGA	23269	ENSG00000174197	validated
tarbase	hsa-miR-582-5p	MGA	23269	ENSG00000174197	validated
tarbase	hsa-miR-15b-5p	C12orf66	144577	ENSG00000174206	validated
tarbase	hsa-miR-21-5p	C12orf66	144577	ENSG00000174206	validated
tarbase	hsa-miR-326	C12orf66	144577	ENSG00000174206	validated
tarbase	hsa-miR-212-3p	C12orf66	144577	ENSG00000174206	validated
tarbase	hsa-miR-582-5p	SNX31	169166	ENSG00000174226	validated
tarbase	hsa-miR-15b-5p	PRPF8	10594	ENSG00000174231	validated
tarbase	hsa-miR-181a-5p	PRPF8	10594	ENSG00000174231	validated
tarbase	hsa-miR-18a-5p	PRPF8	10594	ENSG00000174231	validated
tarbase	hsa-miR-296-5p	PRPF8	10594	ENSG00000174231	validated
tarbase	hsa-miR-301a-3p	PRPF8	10594	ENSG00000174231	validated
tarbase	hsa-miR-15b-5p	ADCY6	112	ENSG00000174233	validated
tarbase	hsa-miR-296-5p	PITPNA	5306	ENSG00000174238	validated
tarbase	hsa-miR-301a-3p	DDX23	9416	ENSG00000174243	validated
tarbase	hsa-miR-212-3p	ZNHIT2	741	ENSG00000174276	validated
tarbase	hsa-miR-18a-5p	ZBTB4	57659	ENSG00000174282	validated
tarbase	hsa-miR-192-5p	ZBTB4	57659	ENSG00000174282	validated
tarbase	hsa-miR-326	ZBTB4	57659	ENSG00000174282	validated
tarbase	hsa-miR-582-3p	ZBTB4	57659	ENSG00000174282	validated

tarbase	hsa-miR-106b-5p	ZHX3	23051	ENSG00000174306	validated
tarbase	hsa-miR-18a-5p	ZHX3	23051	ENSG00000174306	validated
tarbase	hsa-miR-15b-5p	PHLDA3	23612	ENSG00000174307	validated
tarbase	hsa-miR-212-3p	C11orf45	219833	ENSG00000174370	validated
tarbase	hsa-miR-18a-5p	EXO1	9156	ENSG00000174371	validated
tarbase	hsa-miR-192-5p	EXO1	9156	ENSG00000174371	validated
tarbase	hsa-miR-106b-5p	EXO1	9156	ENSG00000174371	validated
tarbase	hsa-miR-144-3p	LIG4	3981	ENSG00000174405	validated
tarbase	hsa-miR-15b-5p	LIG4	3981	ENSG00000174405	validated
tarbase	hsa-miR-582-5p	LIG4	3981	ENSG00000174405	validated
tarbase	hsa-miR-222-3p	MIR1-1HG	128826	ENSG00000174407	validated
tarbase	hsa-miR-106b-5p	ATP2A2	488	ENSG00000174437	validated
tarbase	hsa-miR-181a-5p	ATP2A2	488	ENSG00000174437	validated
tarbase	hsa-miR-18a-5p	ATP2A2	488	ENSG00000174437	validated
tarbase	hsa-miR-212-3p	ATP2A2	488	ENSG00000174437	validated
tarbase	hsa-miR-301a-3p	ATP2A2	488	ENSG00000174437	validated
tarbase	hsa-miR-15b-5p	C12orf76	400073	ENSG00000174456	validated
tarbase	hsa-miR-18a-5p	C12orf76	400073	ENSG00000174456	validated
tarbase	hsa-miR-15b-5p	ZCCHC12	170261	ENSG00000174460	validated
tarbase	hsa-miR-106b-5p	CNTNAP2	26047	ENSG00000174469	validated
tarbase	hsa-miR-192-5p	GALNTL6	442117	ENSG00000174473	validated
tarbase	hsa-miR-18a-5p	DENND4A	10260	ENSG00000174485	validated
tarbase	hsa-miR-212-3p	DENND4A	10260	ENSG00000174485	validated
tarbase	hsa-miR-212-3p	PELI3	246330	ENSG00000174516	validated
tarbase	hsa-miR-181a-5p	AKIRIN1	79647	ENSG00000174574	validated
tarbase	hsa-miR-212-3p	MSL2	55167	ENSG00000174579	validated
tarbase	hsa-miR-21-5p	MSL2	55167	ENSG00000174579	validated
tarbase	hsa-miR-301a-3p	MSL2	55167	ENSG00000174579	validated
tarbase	hsa-miR-106b-5p	CMKLR1	1240	ENSG00000174600	validated
tarbase	hsa-miR-181a-5p	ANGEL2	90806	ENSG00000174606	validated
tarbase	hsa-miR-301a-3p	ANGEL2	90806	ENSG00000174606	validated
tarbase	hsa-miR-15b-5p	UGT8	7368	ENSG00000174607	validated
tarbase	hsa-miR-301a-3p	UGT8	7368	ENSG00000174607	validated
tarbase	hsa-miR-181a-5p	UGT8	7368	ENSG00000174607	validated
tarbase	hsa-miR-21-5p	UGT8	7368	ENSG00000174607	validated
tarbase	hsa-miR-106b-5p	SLC29A2	3177	ENSG00000174669	validated
tarbase	hsa-miR-15b-5p	SLC29A2	3177	ENSG00000174669	validated
tarbase	hsa-miR-296-5p	BRSK2	9024	ENSG00000174672	validated
tarbase	hsa-miR-106b-5p	B4GAT1	11041	ENSG00000174684	validated
tarbase	hsa-miR-15b-5p	B4GAT1	11041	ENSG00000174684	validated
tarbase	hsa-miR-582-3p	TMEM167A	153339	ENSG00000174695	validated
tarbase	hsa-miR-15b-5p	RESF1	55196	ENSG00000174718	validated
tarbase	hsa-miR-582-5p	RESF1	55196	ENSG00000174718	validated
tarbase	hsa-miR-144-3p	NR1D2	9975	ENSG00000174738	validated
tarbase	hsa-miR-21-5p	NR1D2	9975	ENSG00000174738	validated
tarbase	hsa-miR-582-5p	NR1D2	9975	ENSG00000174738	validated
tarbase	hsa-miR-181a-5p	RPL15	6138	ENSG00000174748	validated
tarbase	hsa-miR-181a-5p	SRP72	6731	ENSG00000174780	validated
tarbase	hsa-miR-212-3p	SRP72	6731	ENSG00000174780	validated
tarbase	hsa-miR-21-5p	SRP72	6731	ENSG00000174780	validated
tarbase	hsa-miR-181a-5p	CEP135	9662	ENSG00000174799	validated
tarbase	hsa-miR-144-3p	DENND6A	201627	ENSG00000174839	validated
tarbase	hsa-miR-301a-3p	DENND6A	201627	ENSG00000174839	validated
tarbase	hsa-miR-582-5p	DENND6A	201627	ENSG00000174839	validated
tarbase	hsa-miR-106b-5p	PDE12	201626	ENSG00000174840	validated
tarbase	hsa-miR-15b-5p	PDE12	201626	ENSG00000174840	validated
tarbase	hsa-miR-181a-5p	PDE12	201626	ENSG00000174840	validated
tarbase	hsa-miR-18a-5p	NDUFA11	126328	ENSG00000174886	validated
tarbase	hsa-miR-212-3p	CATSPERD	257062	ENSG00000174898	validated
tarbase	hsa-miR-301a-3p	RAB1B	81876	ENSG00000174903	validated
tarbase	hsa-miR-212-3p	KCTD13	253980	ENSG00000174943	validated
tarbase	hsa-miR-212-3p	FUT1	2523	ENSG00000174951	validated
tarbase	hsa-miR-144-3p	DHX36	170506	ENSG00000174953	validated
tarbase	hsa-miR-15b-5p	DHX36	170506	ENSG00000174953	validated
tarbase	hsa-miR-582-5p	ATR	545	ENSG00000175054	validated
tarbase	hsa-miR-106b-5p	UBE2C	11065	ENSG00000175063	validated
tarbase	hsa-miR-106b-5p	VCPIP1	80124	ENSG00000175073	validated
tarbase	hsa-miR-15b-5p	VCPIP1	80124	ENSG00000175073	validated
tarbase	hsa-miR-181a-5p	VCPIP1	80124	ENSG00000175073	validated
tarbase	hsa-miR-301a-3p	VCPIP1	80124	ENSG00000175073	validated
tarbase	hsa-miR-582-5p	VCPIP1	80124	ENSG00000175073	validated

tarbase	hsa-miR-301a-3p	PDIK1L	149420	ENSG00000175087	validated
tarbase	hsa-miR-15b-5p	SPSB4	92369	ENSG00000175093	validated
tarbase	hsa-miR-18a-5p	ZNF654	55279	ENSG00000175105	validated
tarbase	hsa-miR-212-3p	ZNF654	55279	ENSG00000175105	validated
tarbase	hsa-miR-582-5p	MRPS22	56945	ENSG00000175110	validated
tarbase	hsa-miR-106b-5p	PACS1	55690	ENSG00000175115	validated
tarbase	hsa-miR-144-3p	MARCKSL1	65108	ENSG00000175130	validated
tarbase	hsa-miR-181a-5p	MARCKSL1	65108	ENSG00000175130	validated
tarbase	hsa-miR-21-5p	MARCKSL1	65108	ENSG00000175130	validated
tarbase	hsa-miR-296-5p	MARCKSL1	65108	ENSG00000175130	validated
tarbase	hsa-miR-582-5p	MARCKSL1	65108	ENSG00000175130	validated
tarbase	hsa-miR-15b-5p	SH3BP5L	80851	ENSG00000175137	validated
tarbase	hsa-miR-15b-5p	CADM2	253559	ENSG00000175161	validated
tarbase	hsa-miR-181a-5p	CADM2	253559	ENSG00000175161	validated
tarbase	hsa-miR-21-5p	PSMD2	5708	ENSG00000175166	validated
tarbase	hsa-miR-15b-5p	CTDSP2	10106	ENSG00000175215	validated
tarbase	hsa-miR-181a-5p	CTDSP2	10106	ENSG00000175215	validated
tarbase	hsa-miR-21-5p	CTDSP2	10106	ENSG00000175215	validated
tarbase	hsa-miR-296-5p	CTDSP2	10106	ENSG00000175215	validated
tarbase	hsa-miR-192-5p	CTDSP2	10106	ENSG00000175215	validated
tarbase	hsa-miR-181a-5p	CKAP5	9793	ENSG00000175216	validated
tarbase	hsa-miR-18a-5p	ARHGAP1	392	ENSG00000175220	validated
tarbase	hsa-miR-296-5p	MED16	10025	ENSG00000175221	validated
tarbase	hsa-miR-15b-5p	ATG13	9776	ENSG00000175224	validated
tarbase	hsa-miR-301a-3p	ATG13	9776	ENSG00000175224	validated
tarbase	hsa-miR-212-3p	GOLGA8A	23015	ENSG00000175265	validated
tarbase	hsa-miR-21-5p	TP53I11	9537	ENSG00000175274	validated
tarbase	hsa-miR-212-3p	DOLK	22845	ENSG00000175283	validated
tarbase	hsa-miR-212-3p	CCNE2	9134	ENSG00000175305	validated
tarbase	hsa-miR-326	PHYKPL	85007	ENSG00000175309	validated
tarbase	hsa-miR-15b-5p	GRAMD2A	196996	ENSG00000175318	validated
tarbase	hsa-miR-181a-5p	ZNF519	162655	ENSG00000175322	validated
tarbase	hsa-miR-18a-5p	ZNF519	162655	ENSG00000175322	validated
tarbase	hsa-miR-181a-5p	TMEM9B	56674	ENSG00000175348	validated
tarbase	hsa-miR-18a-5p	TMEM9B	56674	ENSG00000175348	validated
tarbase	hsa-miR-301a-3p	TMEM9B	56674	ENSG00000175348	validated
tarbase	hsa-miR-582-5p	PTPN2	5771	ENSG00000175354	validated
tarbase	hsa-miR-222-3p	EIF1AD	84285	ENSG00000175376	validated
tarbase	hsa-miR-181a-5p	EIF3F	8665	ENSG00000175390	validated
tarbase	hsa-miR-301a-3p	EIF3F	8665	ENSG00000175390	validated
tarbase	hsa-miR-326	PCSK1	5122	ENSG00000175426	validated
tarbase	hsa-miR-181a-5p	CCDC14	64770	ENSG00000175455	validated
tarbase	hsa-miR-181a-5p	MCTP1	79772	ENSG00000175471	validated
tarbase	hsa-miR-181a-5p	ALG10B	144245	ENSG00000175548	validated
tarbase	hsa-miR-301a-3p	LONRF3	79836	ENSG00000175556	validated
tarbase	hsa-miR-21-5p	UCP2	7351	ENSG00000175567	validated
tarbase	hsa-miR-15b-5p	C11orf68	83638	ENSG00000175573	validated
tarbase	hsa-miR-212-3p	RAB6A	5870	ENSG00000175582	validated
tarbase	hsa-miR-106b-5p	FOSL1	8061	ENSG00000175592	validated
tarbase	hsa-miR-18a-5p	FOSL1	8061	ENSG00000175592	validated
tarbase	hsa-miR-222-3p	ERCC4	2072	ENSG00000175595	validated
tarbase	hsa-miR-15b-5p	ERCC4	2072	ENSG00000175595	validated
tarbase	hsa-miR-212-3p	TMEM70	54968	ENSG00000175606	validated
tarbase	hsa-miR-301a-3p	TOM1L2	146691	ENSG00000175662	validated
tarbase	hsa-miR-181a-5p	ZNF77	58492	ENSG00000175691	validated
tarbase	hsa-miR-18a-5p	MLXIP	22877	ENSG00000175727	validated
tarbase	hsa-miR-21-5p	MLXIP	22877	ENSG00000175727	validated
tarbase	hsa-miR-15b-5p	NR2F1	7025	ENSG00000175745	validated
tarbase	hsa-miR-212-3p	SFN	2810	ENSG00000175793	validated
tarbase	hsa-miR-212-3p	CALCB	797	ENSG00000175868	validated
tarbase	hsa-miR-181a-5p	CREG2	200407	ENSG00000175874	validated
tarbase	hsa-miR-15b-5p	ZDHHC21	340481	ENSG00000175893	validated
tarbase	hsa-miR-181a-5p	ZDHHC21	340481	ENSG00000175893	validated
tarbase	hsa-miR-181a-5p	ARL4D	379	ENSG00000175906	validated
tarbase	hsa-miR-21-5p	UBE2O	63893	ENSG00000175931	validated
tarbase	hsa-miR-326	UBE2O	63893	ENSG00000175931	validated
tarbase	hsa-miR-106b-5p	UBE2O	63893	ENSG00000175931	validated
tarbase	hsa-miR-106b-5p	UNC119B	84747	ENSG00000175970	validated
tarbase	hsa-miR-15b-5p	B3GALT6	126792	ENSG00000176022	validated
tarbase	hsa-miR-212-3p	NUPR1	26471	ENSG00000176046	validated
tarbase	hsa-miR-181a-5p	MBLAC2	153364	ENSG00000176055	validated

tarbase	hsa-miR-106b-5p	SLC35A4	113829	ENSG00000176087	validated
tarbase	hsa-miR-15b-5p	SLC35A4	113829	ENSG00000176087	validated
tarbase	hsa-miR-15b-5p	IP6K1	9807	ENSG00000176095	validated
tarbase	hsa-miR-21-5p	IP6K1	9807	ENSG00000176095	validated
tarbase	hsa-miR-106b-5p	CSTF3	1479	ENSG00000176102	validated
tarbase	hsa-miR-15b-5p	YES1	7525	ENSG00000176105	validated
tarbase	hsa-miR-181a-5p	YES1	7525	ENSG00000176105	validated
tarbase	hsa-miR-222-3p	YES1	7525	ENSG00000176105	validated
tarbase	hsa-miR-181a-5p	TMEM39A	55254	ENSG00000176142	validated
tarbase	hsa-miR-301a-3p	LRRTM4	80059	ENSG00000176204	validated
tarbase	hsa-miR-212-3p	SMIM19	114926	ENSG00000176209	validated
tarbase	hsa-miR-18a-5p	ZNF404	342908	ENSG00000176222	validated
tarbase	hsa-miR-15b-5p	SLC35G1	159371	ENSG00000176273	validated
tarbase	hsa-miR-106b-5p	COX8A	1351	ENSG00000176340	validated
tarbase	hsa-miR-15b-5p	CRLF3	51379	ENSG00000176390	validated
tarbase	hsa-miR-106b-5p	CRLF3	51379	ENSG00000176390	validated
tarbase	hsa-miR-301a-3p	RNPEP	6051	ENSG00000176393	validated
tarbase	hsa-miR-106b-5p	EID2B	126272	ENSG00000176401	validated
tarbase	hsa-miR-106b-5p	KCMF1	56888	ENSG00000176407	validated
tarbase	hsa-miR-15b-5p	KCMF1	56888	ENSG00000176407	validated
tarbase	hsa-miR-21-5p	SPRYD4	283377	ENSG00000176422	validated
tarbase	hsa-miR-222-3p	DIRAS1	148252	ENSG00000176490	validated
tarbase	hsa-miR-106b-5p	PRR15	222171	ENSG00000176532	validated
tarbase	hsa-miR-181a-5p	KBTBD11	9920	ENSG00000176595	validated
tarbase	hsa-miR-296-5p	LMNB2	84823	ENSG00000176619	validated
tarbase	hsa-miR-212-3p	LMNB2	84823	ENSG00000176619	validated
tarbase	hsa-miR-18a-5p	RMDN1	51115	ENSG00000176623	validated
tarbase	hsa-miR-181a-5p	MEX3C	51320	ENSG00000176624	validated
tarbase	hsa-miR-212-3p	MEX3C	51320	ENSG00000176624	validated
tarbase	hsa-miR-212-3p	RNF152	220441	ENSG00000176641	validated
tarbase	hsa-miR-192-5p	MYO1D	4642	ENSG00000176658	validated
tarbase	hsa-miR-212-3p	MYO1D	4642	ENSG00000176658	validated
tarbase	hsa-miR-301a-3p	MYO1D	4642	ENSG00000176658	validated
tarbase	hsa-miR-192-5p	FOXL1	2300	ENSG00000176678	validated
tarbase	hsa-miR-15b-5p	CDK5R1	8851	ENSG00000176749	validated
tarbase	hsa-miR-222-3p	CDK5R1	8851	ENSG00000176749	validated
tarbase	hsa-miR-301a-3p	CDK5R1	8851	ENSG00000176749	validated
tarbase	hsa-miR-326	CDK5R1	8851	ENSG00000176749	validated
tarbase	hsa-miR-18a-5p	CDK5R1	8851	ENSG00000176749	validated
tarbase	hsa-miR-212-3p	NCKAP5	344148	ENSG00000176771	validated
tarbase	hsa-miR-192-5p	BASP1	10409	ENSG00000176788	validated
tarbase	hsa-miR-181a-5p	FAM91A1	157769	ENSG00000176853	validated
tarbase	hsa-miR-301a-3p	FAM91A1	157769	ENSG00000176853	validated
tarbase	hsa-miR-582-5p	FAM91A1	157769	ENSG00000176853	validated
tarbase	hsa-miR-222-3p	WSB2	55884	ENSG00000176871	validated
tarbase	hsa-miR-106b-5p	SOX11	6664	ENSG00000176887	validated
tarbase	hsa-miR-144-3p	SOX11	6664	ENSG00000176887	validated
tarbase	hsa-miR-296-5p	SOX11	6664	ENSG00000176887	validated
tarbase	hsa-miR-326	SOX11	6664	ENSG00000176887	validated
tarbase	hsa-miR-106b-5p	PNMA1	9240	ENSG00000176903	validated
tarbase	hsa-miR-181a-5p	PNMA1	9240	ENSG00000176903	validated
tarbase	hsa-miR-582-3p	PNMA1	9240	ENSG00000176903	validated
tarbase	hsa-miR-181a-5p	ANKLE2	23141	ENSG00000176915	validated
tarbase	hsa-miR-212-3p	EFCAB5	374786	ENSG00000176927	validated
tarbase	hsa-miR-106b-5p	GCNT4	51301	ENSG00000176928	validated
tarbase	hsa-miR-15b-5p	SEC24C	9632	ENSG00000176986	validated
tarbase	hsa-miR-181a-5p	SEC24C	9632	ENSG00000176986	validated
tarbase	hsa-miR-582-5p	SEC24C	9632	ENSG00000176986	validated
tarbase	hsa-miR-15b-5p	SMCR8	140775	ENSG00000176994	validated
tarbase	hsa-miR-106b-5p	SMCR8	140775	ENSG00000176994	validated
tarbase	hsa-miR-144-3p	MTX3	345778	ENSG00000177034	validated
tarbase	hsa-miR-106b-5p	FBXO46	23403	ENSG00000177051	validated
tarbase	hsa-miR-21-5p	FBXO46	23403	ENSG00000177051	validated
tarbase	hsa-miR-21-5p	ZDHHC13	54503	ENSG00000177054	validated
tarbase	hsa-miR-15b-5p	SLC38A9	153129	ENSG00000177058	validated
tarbase	hsa-miR-212-3p	POLE	5426	ENSG00000177084	validated
tarbase	hsa-miR-106b-5p	ANO6	196527	ENSG00000177119	validated
tarbase	hsa-miR-181a-5p	ANO6	196527	ENSG00000177119	validated
tarbase	hsa-miR-18a-5p	ANO6	196527	ENSG00000177119	validated
tarbase	hsa-miR-212-3p	ZBTB34	403341	ENSG00000177125	validated
tarbase	hsa-miR-21-5p	ZBTB34	403341	ENSG00000177125	validated

tarbase	hsa-miR-18a-5p	ULK1	8408	ENSG00000177169	validated
tarbase	hsa-miR-296-5p	ULK1	8408	ENSG00000177169	validated
tarbase	hsa-miR-106b-5p	RIMKLA	284716	ENSG00000177181	validated
tarbase	hsa-miR-15b-5p	RIMKLA	284716	ENSG00000177181	validated
tarbase	hsa-miR-106b-5p	RPS6KA3	6197	ENSG00000177189	validated
tarbase	hsa-miR-582-5p	RPS6KA3	6197	ENSG00000177189	validated
tarbase	hsa-miR-212-3p	B3GNT8	374907	ENSG00000177191	validated
tarbase	hsa-miR-192-5p	PUS1	80324	ENSG00000177192	validated
tarbase	hsa-miR-15b-5p	CHD9	80205	ENSG00000177200	validated
tarbase	hsa-miR-18a-5p	CHD9	80205	ENSG00000177200	validated
tarbase	hsa-miR-21-5p	CHD9	80205	ENSG00000177200	validated
tarbase	hsa-miR-301a-3p	CHD9	80205	ENSG00000177200	validated
tarbase	hsa-miR-192-5p	ZBTB38	253461	ENSG00000177311	validated
tarbase	hsa-miR-301a-3p	ZBTB38	253461	ENSG00000177311	validated
tarbase	hsa-miR-212-3p	C10orf71	118461	ENSG00000177354	validated
tarbase	hsa-miR-106b-5p	MAGEF1	64110	ENSG00000177383	validated
tarbase	hsa-miR-212-3p	SAMD9L	219285	ENSG00000177409	validated
tarbase	hsa-miR-181a-5p	PAWR	5074	ENSG00000177425	validated
tarbase	hsa-miR-582-3p	PAWR	5074	ENSG00000177425	validated
tarbase	hsa-miR-15b-5p	TGIF1	7050	ENSG00000177426	validated
tarbase	hsa-miR-212-3p	ERICH5	203111	ENSG00000177459	validated
tarbase	hsa-miR-181a-5p	NR2C2	7182	ENSG00000177463	validated
tarbase	hsa-miR-212-3p	NR2C2	7182	ENSG00000177463	validated
tarbase	hsa-miR-21-5p	ARIH2	10425	ENSG00000177479	validated
tarbase	hsa-miR-222-3p	ZBTB33	10009	ENSG00000177485	validated
tarbase	hsa-miR-326	ZBTB33	10009	ENSG00000177485	validated
tarbase	hsa-miR-582-5p	ZBTB33	10009	ENSG00000177485	validated
tarbase	hsa-miR-15b-5p	ST8SIA3	51046	ENSG00000177511	validated
tarbase	hsa-miR-181a-5p	RPRM	56475	ENSG00000177519	validated
tarbase	hsa-miR-21-5p	SLC25A22	79751	ENSG00000177542	validated
tarbase	hsa-miR-106b-5p	NHLH2	4808	ENSG00000177551	validated
tarbase	hsa-miR-222-3p	NHLH2	4808	ENSG00000177551	validated
tarbase	hsa-miR-15b-5p	ATOX1	475	ENSG00000177556	validated
tarbase	hsa-miR-301a-3p	TBL1XR1	79718	ENSG00000177565	validated
tarbase	hsa-miR-582-3p	TBL1XR1	79718	ENSG00000177565	validated
tarbase	hsa-miR-212-3p	TBL1XR1	79718	ENSG00000177565	validated
tarbase	hsa-miR-18a-5p	SAMD12	401474	ENSG00000177570	validated
tarbase	hsa-miR-222-3p	C18orf32	497661	ENSG00000177576	validated
tarbase	hsa-miR-15b-5p	PIDD1	55367	ENSG00000177595	validated
tarbase	hsa-miR-296-5p	PIDD1	55367	ENSG00000177595	validated
tarbase	hsa-miR-181a-5p	HASPIN	83903	ENSG00000177602	validated
tarbase	hsa-miR-144-3p	JUN	3725	ENSG00000177606	validated
tarbase	hsa-miR-181a-5p	JUN	3725	ENSG00000177606	validated
tarbase	hsa-miR-18a-5p	JUN	3725	ENSG00000177606	validated
tarbase	hsa-miR-212-3p	JUN	3725	ENSG00000177606	validated
tarbase	hsa-miR-106b-5p	CSTF2T	23283	ENSG00000177613	validated
tarbase	hsa-miR-582-3p	CSTF2T	23283	ENSG00000177613	validated
tarbase	hsa-miR-301a-3p	DNAJC28	54943	ENSG00000177692	validated
tarbase	hsa-miR-15b-5p	CD151	977	ENSG00000177697	validated
tarbase	hsa-miR-15b-5p	POLR2L	5441	ENSG00000177700	validated
tarbase	hsa-miR-15b-5p	NECTIN3	25945	ENSG00000177707	validated
tarbase	hsa-miR-212-3p	ANXA2R	389289	ENSG00000177721	validated
tarbase	hsa-miR-326	FLII	2314	ENSG00000177731	validated
tarbase	hsa-miR-301a-3p	SOX12	6666	ENSG00000177732	validated
tarbase	hsa-miR-222-3p	SOX12	6666	ENSG00000177732	validated
tarbase	hsa-miR-222-3p	HNRNPA0	10949	ENSG00000177733	validated
tarbase	hsa-miR-181a-5p	KCNJ10	3766	ENSG00000177807	validated
tarbase	hsa-miR-106b-5p	ZNF518A	9849	ENSG00000177853	validated
tarbase	hsa-miR-21-5p	ZNF518A	9849	ENSG00000177853	validated
tarbase	hsa-miR-212-3p	SVBP	374969	ENSG00000177868	validated
tarbase	hsa-miR-181a-5p	AP3S1	1176	ENSG00000177879	validated
tarbase	hsa-miR-21-5p	AP3S1	1176	ENSG00000177879	validated
tarbase	hsa-miR-181a-5p	GRB2	2885	ENSG00000177885	validated
tarbase	hsa-miR-301a-3p	GRB2	2885	ENSG00000177885	validated
tarbase	hsa-miR-106b-5p	ZBTB41	360023	ENSG00000177888	validated
tarbase	hsa-miR-181a-5p	ZBTB41	360023	ENSG00000177888	validated
tarbase	hsa-miR-192-5p	ZBTB41	360023	ENSG00000177888	validated
tarbase	hsa-miR-212-3p	ZBTB41	360023	ENSG00000177888	validated
tarbase	hsa-miR-181a-5p	UBE2N	7334	ENSG00000177889	validated
tarbase	hsa-miR-21-5p	ZNF354C	30832	ENSG00000177932	validated
tarbase	hsa-miR-15b-5p	BET1L	51272	ENSG00000177951	validated

tarbase	hsa-miR-15b-5p	RIC8A	60626	ENSG00000177963	validated
tarbase	hsa-miR-301a-3p	RIC8A	60626	ENSG00000177963	validated
tarbase	hsa-miR-21-5p	IMP3	55272	ENSG00000177971	validated
tarbase	hsa-miR-212-3p	DPY19L2	283417	ENSG00000177990	validated
tarbase	hsa-miR-326	LRRC75B	388886	ENSG00000178026	validated
tarbase	hsa-miR-106b-5p	CALHM5	254228	ENSG00000178033	validated
tarbase	hsa-miR-21-5p	IMPDH2	3615	ENSG00000178035	validated
tarbase	hsa-miR-192-5p	MLF1	4291	ENSG00000178053	validated
tarbase	hsa-miR-15b-5p	NDUFAF3	25915	ENSG00000178057	validated
tarbase	hsa-miR-15b-5p	C2orf69	205327	ENSG00000178074	validated
tarbase	hsa-miR-18a-5p	C2orf69	205327	ENSG00000178074	validated
tarbase	hsa-miR-212-3p	GRAMD1C	54762	ENSG00000178075	validated
tarbase	hsa-miR-15b-5p	PDE4DIP	9659	ENSG00000178104	validated
tarbase	hsa-miR-181a-5p	PDE4DIP	9659	ENSG00000178104	validated
tarbase	hsa-miR-301a-3p	PDE4DIP	9659	ENSG00000178104	validated
tarbase	hsa-miR-582-3p	ZNF518B	85460	ENSG00000178163	validated
tarbase	hsa-miR-106b-5p	LCORL	254251	ENSG00000178177	validated
tarbase	hsa-miR-212-3p	LCORL	254251	ENSG00000178177	validated
tarbase	hsa-miR-106b-5p	PARD6G	84552	ENSG00000178184	validated
tarbase	hsa-miR-192-5p	SH2B1	25970	ENSG00000178188	validated
tarbase	hsa-miR-296-5p	SH2B1	25970	ENSG00000178188	validated
tarbase	hsa-miR-106b-5p	POGLUT3	143888	ENSG00000178202	validated
tarbase	hsa-miR-15b-5p	POGLUT3	143888	ENSG00000178202	validated
tarbase	hsa-miR-301a-3p	POGLUT3	143888	ENSG00000178202	validated
tarbase	hsa-miR-181a-5p	PLEC	5339	ENSG00000178209	validated
tarbase	hsa-miR-21-5p	PLEC	5339	ENSG00000178209	validated
tarbase	hsa-miR-181a-5p	ZNF543	125919	ENSG00000178229	validated
tarbase	hsa-miR-15b-5p	WDR6	11180	ENSG00000178252	validated
tarbase	hsa-miR-296-5p	WDR6	11180	ENSG00000178252	validated
tarbase	hsa-miR-301a-3p	WDR6	11180	ENSG00000178252	validated
tarbase	hsa-miR-212-3p	ZNF354B	117608	ENSG00000178338	validated
tarbase	hsa-miR-212-3p	ZNF223	7766	ENSG00000178386	validated
tarbase	hsa-miR-181a-5p	BEND3	57673	ENSG00000178409	validated
tarbase	hsa-miR-301a-3p	BEND3	57673	ENSG00000178409	validated
tarbase	hsa-miR-181a-5p	KLHL11	55175	ENSG00000178502	validated
tarbase	hsa-miR-212-3p	SLC25A20	788	ENSG00000178537	validated
tarbase	hsa-miR-106b-5p	EPM2AIP1	9852	ENSG00000178567	validated
tarbase	hsa-miR-212-3p	MAF	4094	ENSG00000178573	validated
tarbase	hsa-miR-15b-5p	CTNNBIP1	56998	ENSG00000178585	validated
tarbase	hsa-miR-301a-3p	ERN1	2081	ENSG00000178607	validated
tarbase	hsa-miR-106b-5p	CSRNP3	80034	ENSG00000178662	validated
tarbase	hsa-miR-301a-3p	CSRNP3	80034	ENSG00000178662	validated
tarbase	hsa-miR-212-3p	CSRNP3	80034	ENSG00000178662	validated
tarbase	hsa-miR-301a-3p	SUZ12	23512	ENSG00000178691	validated
tarbase	hsa-miR-18a-5p	KCTD12	115207	ENSG00000178695	validated
tarbase	hsa-miR-222-3p	KCTD12	115207	ENSG00000178695	validated
tarbase	hsa-miR-582-5p	KCTD12	115207	ENSG00000178695	validated
tarbase	hsa-miR-301a-3p	GRINA	2907	ENSG00000178719	validated
tarbase	hsa-miR-192-5p	COX5A	9377	ENSG00000178741	validated
tarbase	hsa-miR-106b-5p	FAM219B	57184	ENSG00000178761	validated
tarbase	hsa-miR-212-3p	ZHX2	22882	ENSG00000178764	validated
tarbase	hsa-miR-212-3p	APOLD1	81575	ENSG00000178878	validated
tarbase	hsa-miR-106b-5p	DPY19L3	147991	ENSG00000178904	validated
tarbase	hsa-miR-106b-5p	TAF7	6879	ENSG00000178913	validated
tarbase	hsa-miR-15b-5p	TAF7	6879	ENSG00000178913	validated
tarbase	hsa-miR-181a-5p	TAF7	6879	ENSG00000178913	validated
tarbase	hsa-miR-222-3p	PFAS	5198	ENSG00000178921	validated
tarbase	hsa-miR-18a-5p	ZBTB7A	51341	ENSG00000178951	validated
tarbase	hsa-miR-326	ZBTB7A	51341	ENSG00000178951	validated
tarbase	hsa-miR-192-5p	TUFM	7284	ENSG00000178952	validated
tarbase	hsa-miR-301a-3p	TUFM	7284	ENSG00000178952	validated
tarbase	hsa-miR-181a-5p	CTC1	80169	ENSG00000178971	validated
tarbase	hsa-miR-301a-3p	FBXO34	55030	ENSG00000178974	validated
tarbase	hsa-miR-15b-5p	FBXO34	55030	ENSG00000178974	validated
tarbase	hsa-miR-15b-5p	EIF3K	27335	ENSG00000178982	validated
tarbase	hsa-miR-296-5p	EIF3K	27335	ENSG00000178982	validated
tarbase	hsa-miR-301a-3p	AURKB	9212	ENSG00000178999	validated
tarbase	hsa-miR-106b-5p	AURKB	9212	ENSG00000178999	validated
tarbase	hsa-miR-15b-5p	MRFAP1	93621	ENSG00000179010	validated
tarbase	hsa-miR-21-5p	MRFAP1	93621	ENSG00000179010	validated
tarbase	hsa-miR-296-5p	C3orf38	285237	ENSG00000179021	validated

tarbase	hsa-miR-21-5p	RCC2	55920	ENSG00000179051	validated
tarbase	hsa-miR-301a-3p	RCC2	55920	ENSG00000179051	validated
tarbase	hsa-miR-212-3p	RCC2	55920	ENSG00000179051	validated
tarbase	hsa-miR-192-5p	CYC1	1537	ENSG00000179091	validated
tarbase	hsa-miR-301a-3p	PER1	5187	ENSG00000179094	validated
tarbase	hsa-miR-326	PER1	5187	ENSG00000179094	validated
tarbase	hsa-miR-15b-5p	SPTY2D1	144108	ENSG00000179119	validated
tarbase	hsa-miR-181a-5p	SPTY2D1	144108	ENSG00000179119	validated
tarbase	hsa-miR-212-3p	SPTY2D1	144108	ENSG00000179119	validated
tarbase	hsa-miR-21-5p	SPTY2D1	144108	ENSG00000179119	validated
tarbase	hsa-miR-301a-3p	SPTY2D1	144108	ENSG00000179119	validated
tarbase	hsa-miR-582-5p	SAMD4B	55095	ENSG00000179134	validated
tarbase	hsa-miR-212-3p	CYP11B2	1585	ENSG00000179142	validated
tarbase	hsa-miR-296-5p	EDC3	80153	ENSG00000179151	validated
tarbase	hsa-miR-212-3p	PXT1	222659	ENSG00000179165	validated
tarbase	hsa-miR-106b-5p	ZNF664	144348	ENSG00000179195	validated
tarbase	hsa-miR-15b-5p	ZNF664	144348	ENSG00000179195	validated
tarbase	hsa-miR-21-5p	ZNF664	144348	ENSG00000179195	validated
tarbase	hsa-miR-15b-5p	CALR	811	ENSG00000179218	validated
tarbase	hsa-miR-181a-5p	CALR	811	ENSG00000179218	validated
tarbase	hsa-miR-301a-3p	CALR	811	ENSG00000179218	validated
tarbase	hsa-miR-326	CALR	811	ENSG00000179218	validated
tarbase	hsa-miR-15b-5p	MAGED1	9500	ENSG00000179222	validated
tarbase	hsa-miR-181a-5p	MAGED1	9500	ENSG00000179222	validated
tarbase	hsa-miR-222-3p	MAGED1	9500	ENSG00000179222	validated
tarbase	hsa-miR-21-5p	RAD23A	5886	ENSG00000179262	validated
tarbase	hsa-miR-18a-5p	NSUN7	79730	ENSG00000179299	validated
tarbase	hsa-miR-212-3p	RAB39A	54734	ENSG00000179331	validated
tarbase	hsa-miR-212-3p	GATA2	2624	ENSG00000179348	validated
tarbase	hsa-miR-181a-5p	EGR3	1960	ENSG00000179388	validated
tarbase	hsa-miR-212-3p	EGR3	1960	ENSG00000179388	validated
tarbase	hsa-miR-15b-5p	GEMIN4	50628	ENSG00000179409	validated
tarbase	hsa-miR-301a-3p	FJX1	24147	ENSG00000179431	validated
tarbase	hsa-miR-15b-5p	KLHL28	54813	ENSG00000179454	validated
tarbase	hsa-miR-21-5p	KLHL28	54813	ENSG00000179454	validated
tarbase	hsa-miR-181a-5p	KLHL28	54813	ENSG00000179454	validated
tarbase	hsa-miR-181a-5p	ZBTB18	10472	ENSG00000179456	validated
tarbase	hsa-miR-212-3p	ZBTB18	10472	ENSG00000179456	validated
tarbase	hsa-miR-582-3p	ZBTB18	10472	ENSG00000179456	validated
tarbase	hsa-miR-181a-5p	C14orf28	122525	ENSG00000179476	validated
tarbase	hsa-miR-301a-3p	C14orf28	122525	ENSG00000179476	validated
tarbase	hsa-miR-212-3p	HTR1D	3352	ENSG00000179546	validated
tarbase	hsa-miR-296-5p	GCC1	79571	ENSG00000179562	validated
tarbase	hsa-miR-15b-5p	GCC1	79571	ENSG00000179562	validated
tarbase	hsa-miR-212-3p	ZBTB42	100128927	ENSG00000179627	validated
tarbase	hsa-miR-212-3p	LACC1	144811	ENSG00000179630	validated
tarbase	hsa-miR-212-3p	ARL14	80117	ENSG00000179674	validated
tarbase	hsa-miR-18a-5p	PIPOX	51268	ENSG00000179761	validated
tarbase	hsa-miR-15b-5p	MYADM	91663	ENSG00000179820	validated
tarbase	hsa-miR-18a-5p	MYADM	91663	ENSG00000179820	validated
tarbase	hsa-miR-21-5p	MROH1	727957	ENSG00000179832	validated
tarbase	hsa-miR-106b-5p	SERTAD2	9792	ENSG00000179833	validated
tarbase	hsa-miR-181a-5p	SERTAD2	9792	ENSG00000179833	validated
tarbase	hsa-miR-222-3p	SERTAD2	9792	ENSG00000179833	validated
tarbase	hsa-miR-301a-3p	SERTAD2	9792	ENSG00000179833	validated
tarbase	hsa-miR-582-5p	SERTAD2	9792	ENSG00000179833	validated
tarbase	hsa-miR-106b-5p	AKAP5	9495	ENSG00000179841	validated
tarbase	hsa-miR-15b-5p	AKAP5	9495	ENSG00000179841	validated
tarbase	hsa-miR-15b-5p	ABCA13	154664	ENSG00000179869	validated
tarbase	hsa-miR-181a-5p	NRXN1	9378	ENSG00000179915	validated
tarbase	hsa-miR-15b-5p	C14orf119	55017	ENSG00000179933	validated
tarbase	hsa-miR-326	C14orf119	55017	ENSG00000179933	validated
tarbase	hsa-miR-301a-3p	DCTPP1	79077	ENSG00000179958	validated
tarbase	hsa-miR-18a-5p	TSHZ1	10194	ENSG00000179981	validated
tarbase	hsa-miR-301a-3p	TSHZ1	10194	ENSG00000179981	validated
tarbase	hsa-miR-181a-5p	SOCS4	122809	ENSG00000180008	validated
tarbase	hsa-miR-106b-5p	ZADH2	284273	ENSG00000180011	validated
tarbase	hsa-miR-301a-3p	MED14	9282	ENSG00000180182	validated
tarbase	hsa-miR-212-3p	F2	2147	ENSG00000180210	validated
tarbase	hsa-miR-15b-5p	PRKRA	8575	ENSG00000180228	validated
tarbase	hsa-miR-181a-5p	ZNRF2	223082	ENSG00000180233	validated

tarbase	hsa-miR-582-5p	ZNRF2	223082	ENSG00000180233	validated
tarbase	hsa-miR-212-3p	RRH	10692	ENSG00000180245	validated
tarbase	hsa-miR-192-5p	ZNF816	125893	ENSG00000180257	validated
tarbase	hsa-miR-582-3p	ALX1	8092	ENSG00000180318	validated
tarbase	hsa-miR-181a-5p	TIGD2	166815	ENSG00000180346	validated
tarbase	hsa-miR-296-5p	ITPRID1	223075	ENSG00000180347	validated
tarbase	hsa-miR-106b-5p	MTURN	222166	ENSG00000180354	validated
tarbase	hsa-miR-505-3p	MTURN	222166	ENSG00000180354	validated
tarbase	hsa-miR-15b-5p	MTURN	222166	ENSG00000180354	validated
tarbase	hsa-miR-582-5p	ZNF609	23060	ENSG00000180357	validated
tarbase	hsa-miR-106b-5p	PAK2	5062	ENSG00000180370	validated
tarbase	hsa-miR-18a-5p	PAK2	5062	ENSG00000180370	validated
tarbase	hsa-miR-21-5p	PAK2	5062	ENSG00000180370	validated
tarbase	hsa-miR-106b-5p	MCFD2	90411	ENSG00000180398	validated
tarbase	hsa-miR-181a-5p	MCFD2	90411	ENSG00000180398	validated
tarbase	hsa-miR-192-5p	MCFD2	90411	ENSG00000180398	validated
tarbase	hsa-miR-181a-5p	SERTM1	400120	ENSG00000180440	validated
tarbase	hsa-miR-301a-3p	GAS1	2619	ENSG00000180447	validated
tarbase	hsa-miR-181a-5p	ZNF571	51276	ENSG00000180479	validated
tarbase	hsa-miR-106b-5p	MIGA1	374986	ENSG00000180488	validated
tarbase	hsa-miR-181a-5p	MIGA1	374986	ENSG00000180488	validated
tarbase	hsa-miR-18a-5p	MIGA1	374986	ENSG00000180488	validated
tarbase	hsa-miR-222-3p	MIGA1	374986	ENSG00000180488	validated
tarbase	hsa-miR-301a-3p	MIGA1	374986	ENSG00000180488	validated
tarbase	hsa-miR-582-5p	MIGA1	374986	ENSG00000180488	validated
tarbase	hsa-miR-192-5p	NRIP1	8204	ENSG00000180530	validated
tarbase	hsa-miR-21-5p	NRIP1	8204	ENSG00000180530	validated
tarbase	hsa-miR-301a-3p	NRIP1	8204	ENSG00000180530	validated
tarbase	hsa-miR-212-3p	H2AC6	8334	ENSG00000180573	validated
tarbase	hsa-miR-505-3p	EIF2S3B	255308	ENSG00000180574	validated
tarbase	hsa-miR-106b-5p	SKIDA1	387640	ENSG00000180592	validated
tarbase	hsa-miR-21-5p	SKIDA1	387640	ENSG00000180592	validated
tarbase	hsa-miR-301a-3p	SKIDA1	387640	ENSG00000180592	validated
tarbase	hsa-miR-212-3p	H2BC4	8347	ENSG00000180596	validated
tarbase	hsa-miR-181a-5p	PCGF5	84333	ENSG00000180628	validated
tarbase	hsa-miR-582-5p	PCGF5	84333	ENSG00000180628	validated
tarbase	hsa-miR-15b-5p	PCGF5	84333	ENSG00000180628	validated
tarbase	hsa-miR-18a-5p	YOD1	55432	ENSG00000180667	validated
tarbase	hsa-miR-222-3p	YOD1	55432	ENSG00000180667	validated
tarbase	hsa-miR-296-5p	YOD1	55432	ENSG00000180667	validated
tarbase	hsa-miR-18a-5p	TMEM64	169200	ENSG00000180694	validated
tarbase	hsa-miR-212-3p	TMEM64	169200	ENSG00000180694	validated
tarbase	hsa-miR-21-5p	TMEM64	169200	ENSG00000180694	validated
tarbase	hsa-miR-222-3p	TMEM64	169200	ENSG00000180694	validated
tarbase	hsa-miR-582-5p	CLRN3	119467	ENSG00000180745	validated
tarbase	hsa-miR-15b-5p	GPR157	80045	ENSG00000180758	validated
tarbase	hsa-miR-181a-5p	GPR157	80045	ENSG00000180758	validated
tarbase	hsa-miR-106b-5p	SLC36A4	120103	ENSG00000180773	validated
tarbase	hsa-miR-18a-5p	ZDHHC20	253832	ENSG00000180776	validated
tarbase	hsa-miR-301a-3p	ZDHHC20	253832	ENSG00000180776	validated
tarbase	hsa-miR-301a-3p	ARSJ	79642	ENSG00000180801	validated
tarbase	hsa-miR-181a-5p	PPA1	5464	ENSG00000180817	validated
tarbase	hsa-miR-18a-5p	HOXC10	3226	ENSG00000180818	validated
tarbase	hsa-miR-181a-5p	ZNF443	10224	ENSG00000180855	validated
tarbase	hsa-miR-212-3p	C11orf42	160298	ENSG00000180878	validated
tarbase	hsa-miR-181a-5p	CAPS2	84698	ENSG00000180881	validated
tarbase	hsa-miR-106b-5p	ZNF792	126375	ENSG00000180884	validated
tarbase	hsa-miR-582-5p	SCRIB	23513	ENSG00000180900	validated
tarbase	hsa-miR-296-5p	D2HGDH	728294	ENSG00000180902	validated
tarbase	hsa-miR-301a-3p	CMTR2	55783	ENSG00000180917	validated
tarbase	hsa-miR-212-3p	FAM83H	286077	ENSG00000180921	validated
tarbase	hsa-miR-21-5p	PITPNB	23760	ENSG00000180957	validated
tarbase	hsa-miR-505-3p	PITPNB	23760	ENSG00000180957	validated
tarbase	hsa-miR-106b-5p	GPR137C	283554	ENSG00000180998	validated
tarbase	hsa-miR-15b-5p	GPR137C	283554	ENSG00000180998	validated
tarbase	hsa-miR-181a-5p	GPR137C	283554	ENSG00000180998	validated
tarbase	hsa-miR-212-3p	GPR137C	283554	ENSG00000180998	validated
tarbase	hsa-miR-18a-5p	ZFP82	284406	ENSG00000181007	validated
tarbase	hsa-miR-106b-5p	AEN	64782	ENSG00000181026	validated
tarbase	hsa-miR-181a-5p	AEN	64782	ENSG00000181026	validated
tarbase	hsa-miR-296-5p	AEN	64782	ENSG00000181026	validated

tarbase	hsa-miR-18a-5p	FKRP	79147	ENSG00000181027	validated
tarbase	hsa-miR-106b-5p	HIGD1A	25994	ENSG00000181061	validated
tarbase	hsa-miR-15b-5p	EHMT1	79813	ENSG00000181090	validated
tarbase	hsa-miR-18a-5p	EHMT1	79813	ENSG00000181090	validated
tarbase	hsa-miR-326	EHMT1	79813	ENSG00000181090	validated
tarbase	hsa-miR-212-3p	ADIPOQ	9370	ENSG00000181092	validated
tarbase	hsa-miR-181a-5p	F2R	2149	ENSG00000181104	validated
tarbase	hsa-miR-21-5p	F2R	2149	ENSG00000181104	validated
tarbase	hsa-miR-106b-5p	NPM1	4869	ENSG00000181163	validated
tarbase	hsa-miR-326	NPM1	4869	ENSG00000181163	validated
tarbase	hsa-miR-15b-5p	PJA1	64219	ENSG00000181191	validated
tarbase	hsa-miR-15b-5p	H2AW	92815	ENSG00000181218	validated
tarbase	hsa-miR-15b-5p	POLR2A	5430	ENSG00000181222	validated
tarbase	hsa-miR-181a-5p	POLR2A	5430	ENSG00000181222	validated
tarbase	hsa-miR-192-5p	POLR2A	5430	ENSG00000181222	validated
tarbase	hsa-miR-21-5p	POLR2A	5430	ENSG00000181222	validated
tarbase	hsa-miR-222-3p	POLR2A	5430	ENSG00000181222	validated
tarbase	hsa-miR-106b-5p	ZNF322	79692	ENSG00000181315	validated
tarbase	hsa-miR-212-3p	DDX60L	91351	ENSG00000181381	validated
tarbase	hsa-miR-181a-5p	ZNF678	339500	ENSG00000181450	validated
tarbase	hsa-miR-505-3p	ZNF678	339500	ENSG00000181450	validated
tarbase	hsa-miR-144-3p	RAP2B	5912	ENSG00000181467	validated
tarbase	hsa-miR-212-3p	RAP2B	5912	ENSG00000181467	validated
tarbase	hsa-miR-582-3p	RAP2B	5912	ENSG00000181467	validated
tarbase	hsa-miR-212-3p	ZBTB2	57621	ENSG00000181472	validated
tarbase	hsa-miR-582-5p	RNF135	84282	ENSG00000181481	validated
tarbase	hsa-miR-18a-5p	SGSH	6448	ENSG00000181523	validated
tarbase	hsa-miR-192-5p	SETD2	29072	ENSG00000181555	validated
tarbase	hsa-miR-106b-5p	MEX3D	399664	ENSG00000181588	validated
tarbase	hsa-miR-15b-5p	MRPS23	51649	ENSG00000181610	validated
tarbase	hsa-miR-181a-5p	MRPS23	51649	ENSG00000181610	validated
tarbase	hsa-miR-301a-3p	ZFP41	286128	ENSG00000181638	validated
tarbase	hsa-miR-18a-5p	ZFP41	286128	ENSG00000181638	validated
tarbase	hsa-miR-582-5p	ATG9B	285973	ENSG00000181652	validated
tarbase	hsa-miR-15b-5p	GPR88	54112	ENSG00000181656	validated
tarbase	hsa-miR-106b-5p	ZNF875	284459	ENSG00000181666	validated
tarbase	hsa-miR-106b-5p	PLAG1	5324	ENSG00000181690	validated
tarbase	hsa-miR-18a-5p	PLAG1	5324	ENSG00000181690	validated
tarbase	hsa-miR-21-5p	PLAG1	5324	ENSG00000181690	validated
tarbase	hsa-miR-106b-5p	YIPF6	286451	ENSG00000181704	validated
tarbase	hsa-miR-18a-5p	YIPF6	286451	ENSG00000181704	validated
tarbase	hsa-miR-15b-5p	DIPK2A	205428	ENSG00000181744	validated
tarbase	hsa-miR-301a-3p	DIPK2A	205428	ENSG00000181744	validated
tarbase	hsa-miR-106b-5p	C5orf30	90355	ENSG00000181751	validated
tarbase	hsa-miR-15b-5p	C5orf30	90355	ENSG00000181751	validated
tarbase	hsa-miR-181a-5p	C5orf30	90355	ENSG00000181751	validated
tarbase	hsa-miR-301a-3p	C5orf30	90355	ENSG00000181751	validated
tarbase	hsa-miR-15b-5p	AMIGO1	57463	ENSG00000181754	validated
tarbase	hsa-miR-144-3p	SIAH2	6478	ENSG00000181788	validated
tarbase	hsa-miR-106b-5p	SIAH2	6478	ENSG00000181788	validated
tarbase	hsa-miR-296-5p	COPG1	22820	ENSG00000181789	validated
tarbase	hsa-miR-192-5p	RFX7	64864	ENSG00000181827	validated
tarbase	hsa-miR-21-5p	RFX7	64864	ENSG00000181827	validated
tarbase	hsa-miR-222-3p	RFX7	64864	ENSG00000181827	validated
tarbase	hsa-miR-326	RFX7	64864	ENSG00000181827	validated
tarbase	hsa-miR-212-3p	RFX7	64864	ENSG00000181827	validated
tarbase	hsa-miR-181a-5p	RFX7	64864	ENSG00000181827	validated
tarbase	hsa-miR-301a-3p	RNF41	10193	ENSG00000181852	validated
tarbase	hsa-miR-326	IBA57	200205	ENSG00000181873	validated
tarbase	hsa-miR-21-5p	CLDN7	1366	ENSG00000181885	validated
tarbase	hsa-miR-181a-5p	ZNF101	94039	ENSG00000181896	validated
tarbase	hsa-miR-106b-5p	C5orf24	134553	ENSG00000181904	validated
tarbase	hsa-miR-15b-5p	C5orf24	134553	ENSG00000181904	validated
tarbase	hsa-miR-181a-5p	C5orf24	134553	ENSG00000181904	validated
tarbase	hsa-miR-582-5p	C5orf24	134553	ENSG00000181904	validated
tarbase	hsa-miR-21-5p	C5orf24	134553	ENSG00000181904	validated
tarbase	hsa-miR-212-3p	LINC02724		ENSG00000181908	validated
tarbase	hsa-miR-106b-5p	ADO	84890	ENSG00000181915	validated
tarbase	hsa-miR-181a-5p	ADO	84890	ENSG00000181915	validated
tarbase	hsa-miR-15b-5p	PRKAG1	5571	ENSG00000181929	validated
tarbase	hsa-miR-212-3p	RTKN2	219790	ENSG00000182010	validated

tarbase	hsa-miR-296-5p	TMEM259	91304	ENSG00000182087	validated
tarbase	hsa-miR-222-3p	TNRC18	84629	ENSG00000182095	validated
tarbase	hsa-miR-296-5p	TNRC18	84629	ENSG00000182095	validated
tarbase	hsa-miR-301a-3p	TNRC18	84629	ENSG00000182095	validated
tarbase	hsa-miR-15b-5p	FAM181B	220382	ENSG00000182103	validated
tarbase	hsa-miR-18a-5p	ZNF716	441234	ENSG00000182111	validated
tarbase	hsa-miR-21-5p	FAM89A	375061	ENSG00000182118	validated
tarbase	hsa-miR-192-5p	ZNF708	7562	ENSG00000182141	validated
tarbase	hsa-miR-582-5p	ZNF708	7562	ENSG00000182141	validated
tarbase	hsa-miR-15b-5p	IST1	9798	ENSG00000182149	validated
tarbase	hsa-miR-582-5p	ERCC6L2	375748	ENSG00000182150	validated
tarbase	hsa-miR-181a-5p	ERCC6L2	375748	ENSG00000182150	validated
tarbase	hsa-miR-192-5p	CREB3L2	64764	ENSG00000182158	validated
tarbase	hsa-miR-582-3p	CREB3L2	64764	ENSG00000182158	validated
tarbase	hsa-miR-106b-5p	CREB3L2	64764	ENSG00000182158	validated
tarbase	hsa-miR-222-3p	UNC5C	8633	ENSG00000182168	validated
tarbase	hsa-miR-106b-5p	RGMA	56963	ENSG00000182175	validated
tarbase	hsa-miR-296-5p	SHMT2	6472	ENSG00000182199	validated
tarbase	hsa-miR-144-3p	ATP6AP2	10159	ENSG00000182220	validated
tarbase	hsa-miR-181a-5p	ATP6AP2	10159	ENSG00000182220	validated
tarbase	hsa-miR-582-5p	ATP6AP2	10159	ENSG00000182220	validated
tarbase	hsa-miR-181a-5p	BACE2	25825	ENSG00000182240	validated
tarbase	hsa-miR-222-3p	BACE2	25825	ENSG00000182240	validated
tarbase	hsa-miR-212-3p	UBE2E2	7325	ENSG00000182247	validated
tarbase	hsa-miR-106b-5p	SYNM	23336	ENSG00000182253	validated
tarbase	hsa-miR-181a-5p	FIGN	55137	ENSG00000182263	validated
tarbase	hsa-miR-582-3p	FIGN	55137	ENSG00000182263	validated
tarbase	hsa-miR-212-3p	FIGN	55137	ENSG00000182263	validated
tarbase	hsa-miR-296-5p	B4GALNT4	338707	ENSG00000182272	validated
tarbase	hsa-miR-301a-3p	AP1S2	8905	ENSG00000182287	validated
tarbase	hsa-miR-181a-5p	C8orf33	65265	ENSG00000182307	validated
tarbase	hsa-miR-296-5p	ZSCAN22	342945	ENSG00000182318	validated
tarbase	hsa-miR-296-5p	FBXL6	26233	ENSG00000182325	validated
tarbase	hsa-miR-582-5p	KBTBD3	143879	ENSG00000182359	validated
tarbase	hsa-miR-212-3p	KBTBD3	143879	ENSG00000182359	validated
tarbase	hsa-miR-181a-5p	CACNB4	785	ENSG00000182389	validated
tarbase	hsa-miR-18a-5p	CACNB4	785	ENSG00000182389	validated
tarbase	hsa-miR-212-3p	IFNL1	282618	ENSG00000182393	validated
tarbase	hsa-miR-222-3p	PGBD4	161779	ENSG00000182405	validated
tarbase	hsa-miR-15b-5p	NPLOC4	55666	ENSG00000182446	validated
tarbase	hsa-miR-582-3p	TEX19	400629	ENSG00000182459	validated
tarbase	hsa-miR-144-3p	TSHZ2	128553	ENSG00000182463	validated
tarbase	hsa-miR-296-5p	CAPN12	147968	ENSG00000182472	validated
tarbase	hsa-miR-296-5p	EXOC7	23265	ENSG00000182473	validated
tarbase	hsa-miR-21-5p	KPNA2	3838	ENSG00000182481	validated
tarbase	hsa-miR-301a-3p	KPNA2	3838	ENSG00000182481	validated
tarbase	hsa-miR-181a-5p	CEP97	79598	ENSG00000182504	validated
tarbase	hsa-miR-301a-3p	CEP97	79598	ENSG00000182504	validated
tarbase	hsa-miR-326	MXRA7	439921	ENSG00000182534	validated
tarbase	hsa-miR-15b-5p	MFSD5	84975	ENSG00000182544	validated
tarbase	hsa-miR-212-3p	MFSD5	84975	ENSG00000182544	validated
tarbase	hsa-miR-18a-5p	SATB1	6304	ENSG00000182568	validated
tarbase	hsa-miR-222-3p	SATB1	6304	ENSG00000182568	validated
tarbase	hsa-miR-212-3p	SATB1	6304	ENSG00000182568	validated
tarbase	hsa-miR-181a-5p	TRAK1	22906	ENSG00000182606	validated
tarbase	hsa-miR-15b-5p	SKA2	348235	ENSG00000182628	validated
tarbase	hsa-miR-181a-5p	SKA2	348235	ENSG00000182628	validated
tarbase	hsa-miR-21-5p	SKA2	348235	ENSG00000182628	validated
tarbase	hsa-miR-106b-5p	NDN	4692	ENSG00000182636	validated
tarbase	hsa-miR-15b-5p	TTC3	7267	ENSG00000182670	validated
tarbase	hsa-miR-301a-3p	TTC3	7267	ENSG00000182670	validated
tarbase	hsa-miR-582-5p	IGIP	492311	ENSG00000182700	validated
tarbase	hsa-miR-301a-3p	TSKU	25987	ENSG00000182704	validated
tarbase	hsa-miR-15b-5p	CMC4	100272147	ENSG00000182712	validated
tarbase	hsa-miR-212-3p	ANXA2	302	ENSG00000182718	validated
tarbase	hsa-miR-296-5p	HOXB4	3214	ENSG00000182742	validated
tarbase	hsa-miR-582-3p	SLC35D3	340146	ENSG00000182747	validated
tarbase	hsa-miR-15b-5p	PAPPA	5069	ENSG00000182752	validated
tarbase	hsa-miR-21-5p	PAPPA	5069	ENSG00000182752	validated
tarbase	hsa-miR-192-5p	NGRN	51335	ENSG00000182768	validated
tarbase	hsa-miR-301a-3p	ACBD3	64746	ENSG00000182827	validated

tarbase	hsa-miR-106b-5p	C16orf72	29035	ENSG00000182831	validated
tarbase	hsa-miR-181a-5p	C16orf72	29035	ENSG00000182831	validated
tarbase	hsa-miR-212-3p	C16orf72	29035	ENSG00000182831	validated
tarbase	hsa-miR-222-3p	C16orf72	29035	ENSG00000182831	validated
tarbase	hsa-miR-301a-3p	C16orf72	29035	ENSG00000182831	validated
tarbase	hsa-miR-212-3p	SLC25A18	83733	ENSG00000182902	validated
tarbase	hsa-miR-212-3p	C11orf54	28970	ENSG00000182919	validated
tarbase	hsa-miR-296-5p	CEP63	80254	ENSG00000182923	validated
tarbase	hsa-miR-212-3p	CEP63	80254	ENSG00000182923	validated
tarbase	hsa-miR-326	SRPRA	6734	ENSG00000182934	validated
tarbase	hsa-miR-181a-5p	EWSR1	2130	ENSG00000182944	validated
tarbase	hsa-miR-582-3p	EWSR1	2130	ENSG00000182944	validated
tarbase	hsa-miR-18a-5p	SPATA13	221178	ENSG00000182957	validated
tarbase	hsa-miR-296-5p	SPATA13	221178	ENSG00000182957	validated
tarbase	hsa-miR-181a-5p	MTA1	9112	ENSG00000182979	validated
tarbase	hsa-miR-181a-5p	CADM1	23705	ENSG00000182985	validated
tarbase	hsa-miR-18a-5p	ZNF320	162967	ENSG00000182986	validated
tarbase	hsa-miR-15b-5p	NAA38	84316	ENSG00000183011	validated
tarbase	hsa-miR-181a-5p	SLC8A1	6546	ENSG00000183023	validated
tarbase	hsa-miR-212-3p	SLC25A21	89874	ENSG00000183032	validated
tarbase	hsa-miR-106b-5p	ABAT	18	ENSG00000183044	validated
tarbase	hsa-miR-21-5p	ABAT	18	ENSG00000183044	validated
tarbase	hsa-miR-301a-3p	FANCF	2188	ENSG00000183161	validated
tarbase	hsa-miR-212-3p	FANCF	2188	ENSG00000183161	validated
tarbase	hsa-miR-181a-5p	GDPGP1	390637	ENSG00000183208	validated
tarbase	hsa-miR-15b-5p	PTTG1IP	754	ENSG00000183255	validated
tarbase	hsa-miR-582-5p	PTTG1IP	754	ENSG00000183255	validated
tarbase	hsa-miR-106b-5p	DAZAP2	9802	ENSG00000183283	validated
tarbase	hsa-miR-18a-5p	DAZAP2	9802	ENSG00000183283	validated
tarbase	hsa-miR-222-3p	SELENOF	9403	ENSG00000183291	validated
tarbase	hsa-miR-144-3p	ZNF623	9831	ENSG00000183309	validated
tarbase	hsa-miR-212-3p	ZNF623	9831	ENSG00000183309	validated
tarbase	hsa-miR-181a-5p	ZNF623	9831	ENSG00000183309	validated
tarbase	hsa-miR-212-3p	BOLA2	552900	ENSG00000183336	validated
tarbase	hsa-miR-106b-5p	BCOR	54880	ENSG00000183337	validated
tarbase	hsa-miR-222-3p	BCOR	54880	ENSG00000183337	validated
tarbase	hsa-miR-582-5p	BCOR	54880	ENSG00000183337	validated
tarbase	hsa-miR-106b-5p	JRKL	8690	ENSG00000183340	validated
tarbase	hsa-miR-181a-5p	KIAA2026	158358	ENSG00000183354	validated
tarbase	hsa-miR-144-3p	RIPK4	54101	ENSG00000183421	validated
tarbase	hsa-miR-301a-3p	RIPK4	54101	ENSG00000183421	validated
tarbase	hsa-miR-212-3p	SF3A3	10946	ENSG00000183431	validated
tarbase	hsa-miR-326	SF3A3	10946	ENSG00000183431	validated
tarbase	hsa-miR-18a-5p	GRIN2A	2903	ENSG00000183454	validated
tarbase	hsa-miR-181a-5p	GRIN2A	2903	ENSG00000183454	validated
tarbase	hsa-miR-18a-5p	MEX3B	84206	ENSG00000183496	validated
tarbase	hsa-miR-21-5p	TENT5C	54855	ENSG00000183508	validated
tarbase	hsa-miR-106b-5p	COA5	493753	ENSG00000183513	validated
tarbase	hsa-miR-582-5p	UTP11	51118	ENSG00000183520	validated
tarbase	hsa-miR-15b-5p	PRR14L	253143	ENSG00000183530	validated
tarbase	hsa-miR-181a-5p	PRR14L	253143	ENSG00000183530	validated
tarbase	hsa-miR-181a-5p	FBXL7	23194	ENSG00000183580	validated
tarbase	hsa-miR-181a-5p	SFXN4	119559	ENSG00000183605	validated
tarbase	hsa-miR-212-3p	FAM167B	84734	ENSG00000183615	validated
tarbase	hsa-miR-181a-5p	ZNF530	348327	ENSG00000183647	validated
tarbase	hsa-miR-212-3p	TRMT12	55039	ENSG00000183665	validated
tarbase	hsa-miR-18a-5p	ALYREF	10189	ENSG00000183684	validated
tarbase	hsa-miR-326	ALYREF	10189	ENSG00000183684	validated
tarbase	hsa-miR-212-3p	UPP1	7378	ENSG00000183696	validated
tarbase	hsa-miR-212-3p	IFNL2	282616	ENSG00000183709	validated
tarbase	hsa-miR-181a-5p	TBK1	29110	ENSG00000183735	validated
tarbase	hsa-miR-21-5p	TBK1	29110	ENSG00000183735	validated
tarbase	hsa-miR-106b-5p	CBX6	23466	ENSG00000183741	validated
tarbase	hsa-miR-192-5p	CBX6	23466	ENSG00000183741	validated
tarbase	hsa-miR-296-5p	CBX6	23466	ENSG00000183741	validated
tarbase	hsa-miR-301a-3p	CBX6	23466	ENSG00000183741	validated
tarbase	hsa-miR-505-3p	CBX6	23466	ENSG00000183741	validated
tarbase	hsa-miR-582-3p	CBX6	23466	ENSG00000183741	validated
tarbase	hsa-miR-106b-5p	MACC1	346389	ENSG00000183742	validated
tarbase	hsa-miR-15b-5p	TBL3	10607	ENSG00000183751	validated
tarbase	hsa-miR-212-3p	CHEK2	11200	ENSG00000183765	validated

tarbase	hsa-miR-15b-5p	KCTD16	57528	ENSG00000183775	validated
tarbase	hsa-miR-181a-5p	KCTD16	57528	ENSG00000183775	validated
tarbase	hsa-miR-181a-5p	ZNF703	80139	ENSG00000183779	validated
tarbase	hsa-miR-212-3p	ZNF703	80139	ENSG00000183779	validated
tarbase	hsa-miR-296-5p	ZNF703	80139	ENSG00000183779	validated
tarbase	hsa-miR-106b-5p	ZNF703	80139	ENSG00000183779	validated
tarbase	hsa-miR-301a-3p	TUBA8	51807	ENSG00000183785	validated
tarbase	hsa-miR-212-3p	RBM12B	389677	ENSG00000183808	validated
tarbase	hsa-miR-21-5p	RBM12B	389677	ENSG00000183808	validated
tarbase	hsa-miR-301a-3p	RBM12B	389677	ENSG00000183808	validated
tarbase	hsa-miR-212-3p	CCR4	1233	ENSG00000183813	validated
tarbase	hsa-miR-15b-5p	BTBD9	114781	ENSG00000183826	validated
tarbase	hsa-miR-181a-5p	ZNF730	100129543	ENSG00000183850	validated
tarbase	hsa-miR-106b-5p	KIRREL1	55243	ENSG00000183853	validated
tarbase	hsa-miR-18a-5p	IQGAP3	128239	ENSG00000183856	validated
tarbase	hsa-miR-301a-3p	TOB2	10766	ENSG00000183864	validated
tarbase	hsa-miR-106b-5p	TOB2	10766	ENSG00000183864	validated
tarbase	hsa-miR-212-3p	SCN5A	6331	ENSG00000183873	validated
tarbase	hsa-miR-212-3p	PRKX	5613	ENSG00000183943	validated
tarbase	hsa-miR-181a-5p	KMT5A	387893	ENSG00000183955	validated
tarbase	hsa-miR-106b-5p	KCNH8	131096	ENSG00000183960	validated
tarbase	hsa-miR-15b-5p	SMTN	6525	ENSG00000183963	validated
tarbase	hsa-miR-15b-5p	PTP4A2	8073	ENSG00000184007	validated
tarbase	hsa-miR-21-5p	PTP4A2	8073	ENSG00000184007	validated
tarbase	hsa-miR-15b-5p	ACTG1	71	ENSG00000184009	validated
tarbase	hsa-miR-212-3p	ACTG1	71	ENSG00000184009	validated
tarbase	hsa-miR-582-3p	ACTG1	71	ENSG00000184009	validated
tarbase	hsa-miR-326	DENND5A	23258	ENSG00000184014	validated
tarbase	hsa-miR-21-5p	DENND5A	23258	ENSG00000184014	validated
tarbase	hsa-miR-144-3p	TBX1	6899	ENSG00000184058	validated
tarbase	hsa-miR-301a-3p	UQCR10	29796	ENSG00000184076	validated
tarbase	hsa-miR-212-3p	EIF3C	8663	ENSG00000184110	validated
tarbase	hsa-miR-181a-5p	KCNQ3	3786	ENSG00000184156	validated
tarbase	hsa-miR-106b-5p	NR2C2AP	126382	ENSG00000184162	validated
tarbase	hsa-miR-222-3p	NR2C2AP	126382	ENSG00000184162	validated
tarbase	hsa-miR-301a-3p	NR2C2AP	126382	ENSG00000184162	validated
tarbase	hsa-miR-15b-5p	PPP1R2	5504	ENSG00000184203	validated
tarbase	hsa-miR-222-3p	PPP1R2	5504	ENSG00000184203	validated
tarbase	hsa-miR-582-5p	PPP1R2	5504	ENSG00000184203	validated
tarbase	hsa-miR-15b-5p	TSPYL2	64061	ENSG00000184205	validated
tarbase	hsa-miR-181a-5p	GOLGA6L4	643707	ENSG00000184206	validated
tarbase	hsa-miR-296-5p	IRAK1	3654	ENSG00000184216	validated
tarbase	hsa-miR-212-3p	IRAK1	3654	ENSG00000184216	validated
tarbase	hsa-miR-15b-5p	IRAK1	3654	ENSG00000184216	validated
tarbase	hsa-miR-212-3p	OLIG1	116448	ENSG00000184221	validated
tarbase	hsa-miR-15b-5p	PCDH9	5101	ENSG00000184226	validated
tarbase	hsa-miR-181a-5p	PCDH9	5101	ENSG00000184226	validated
tarbase	hsa-miR-296-5p	OAF	220323	ENSG00000184232	validated
tarbase	hsa-miR-15b-5p	ALDH1A3	220	ENSG00000184254	validated
tarbase	hsa-miR-106b-5p	POU6F1	5463	ENSG00000184271	validated
tarbase	hsa-miR-15b-5p	TACSTD2	4070	ENSG00000184292	validated
tarbase	hsa-miR-181a-5p	TACSTD2	4070	ENSG00000184292	validated
tarbase	hsa-miR-212-3p	PRKD1	5587	ENSG00000184304	validated
tarbase	hsa-miR-15b-5p	CCSER1	401145	ENSG00000184305	validated
tarbase	hsa-miR-222-3p	CCSER1	401145	ENSG00000184305	validated
tarbase	hsa-miR-15b-5p	ZDHHC23	254887	ENSG00000184307	validated
tarbase	hsa-miR-301a-3p	ZDHHC23	254887	ENSG00000184307	validated
tarbase	hsa-miR-21-5p	H1-5	3009	ENSG00000184357	validated
tarbase	hsa-miR-212-3p	MAP7D2	256714	ENSG00000184368	validated
tarbase	hsa-miR-181a-5p	SS18L1	26039	ENSG00000184402	validated
tarbase	hsa-miR-21-5p	COPB2	9276	ENSG00000184432	validated
tarbase	hsa-miR-181a-5p	KNTC1	9735	ENSG00000184445	validated
tarbase	hsa-miR-301a-3p	KNTC1	9735	ENSG00000184445	validated
tarbase	hsa-miR-15b-5p	CCR10	2826	ENSG00000184451	validated
tarbase	hsa-miR-212-3p	POU3F2	5454	ENSG00000184486	validated
tarbase	hsa-miR-21-5p	HDDC3	374659	ENSG00000184508	validated
tarbase	hsa-miR-15b-5p	ZFP1	162239	ENSG00000184517	validated
tarbase	hsa-miR-301a-3p	CEND1	51286	ENSG00000184524	validated
tarbase	hsa-miR-106b-5p	DUSP8	1850	ENSG00000184545	validated
tarbase	hsa-miR-296-5p	SOCS3	9021	ENSG00000184557	validated
tarbase	hsa-miR-326	SOCS3	9021	ENSG00000184557	validated

tarbase	hsa-miR-212-3p	SPEM2	201243	ENSG00000184560	validated
tarbase	hsa-miR-192-5p	PIWIL3	440822	ENSG00000184571	validated
tarbase	hsa-miR-296-5p	PIWIL3	440822	ENSG00000184571	validated
tarbase	hsa-miR-106b-5p	XPOT	11260	ENSG00000184575	validated
tarbase	hsa-miR-192-5p	XPOT	11260	ENSG00000184575	validated
tarbase	hsa-miR-144-3p	SNN	8303	ENSG00000184602	validated
tarbase	hsa-miR-21-5p	SNN	8303	ENSG00000184602	validated
tarbase	hsa-miR-582-3p	SNN	8303	ENSG00000184602	validated
tarbase	hsa-miR-181a-5p	SNN	8303	ENSG00000184602	validated
tarbase	hsa-miR-192-5p	SEPTIN9	10801	ENSG00000184640	validated
tarbase	hsa-miR-106b-5p	CDCA2	157313	ENSG00000184661	validated
tarbase	hsa-miR-18a-5p	CDCA2	157313	ENSG00000184661	validated
tarbase	hsa-miR-212-3p	CDCA2	157313	ENSG00000184661	validated
tarbase	hsa-miR-296-5p	AMER1	139285	ENSG00000184675	validated
tarbase	hsa-miR-144-3p	H2BC21	8349	ENSG00000184678	validated
tarbase	hsa-miR-15b-5p	SEPTIN5	5413	ENSG00000184702	validated
tarbase	hsa-miR-181a-5p	EIF4ENIF1	56478	ENSG00000184708	validated
tarbase	hsa-miR-15b-5p	FAM110C	642273	ENSG00000184731	validated
tarbase	hsa-miR-301a-3p	FAM110C	642273	ENSG00000184731	validated
tarbase	hsa-miR-326	DDX53	168400	ENSG00000184735	validated
tarbase	hsa-miR-15b-5p	ATL3	25923	ENSG00000184743	validated
tarbase	hsa-miR-181a-5p	ATL3	25923	ENSG00000184743	validated
tarbase	hsa-miR-212-3p	ATL3	25923	ENSG00000184743	validated
tarbase	hsa-miR-21-5p	ATL3	25923	ENSG00000184743	validated
tarbase	hsa-miR-222-3p	ATL3	25923	ENSG00000184743	validated
tarbase	hsa-miR-582-3p	NDUFA12	55967	ENSG00000184752	validated
tarbase	hsa-miR-212-3p	TRARG1	286753	ENSG00000184811	validated
tarbase	hsa-miR-106b-5p	RBM33	155435	ENSG00000184863	validated
tarbase	hsa-miR-301a-3p	RBM33	155435	ENSG00000184863	validated
tarbase	hsa-miR-15b-5p	ARMCX2	9823	ENSG00000184867	validated
tarbase	hsa-miR-15b-5p	BTBD6	90135	ENSG00000184887	validated
tarbase	hsa-miR-222-3p	H1-10	8971	ENSG00000184897	validated
tarbase	hsa-miR-15b-5p	SUMO3	6612	ENSG00000184900	validated
tarbase	hsa-miR-181a-5p	IMMP2L	83943	ENSG00000184903	validated
tarbase	hsa-miR-181a-5p	ZFP90	146198	ENSG00000184939	validated
tarbase	hsa-miR-181a-5p	USP18	11274	ENSG00000184979	validated
tarbase	hsa-miR-212-3p	USP18	11274	ENSG00000184979	validated
tarbase	hsa-miR-15b-5p	TMEM121	80757	ENSG00000184986	validated
tarbase	hsa-miR-18a-5p	TMEM121	80757	ENSG00000184986	validated
tarbase	hsa-miR-181a-5p	SIVA1	10572	ENSG00000184990	validated
tarbase	hsa-miR-15b-5p	BRI3BP	140707	ENSG00000184992	validated
tarbase	hsa-miR-505-3p	BRI3BP	140707	ENSG00000184992	validated
tarbase	hsa-miR-18a-5p	DGAT1	8694	ENSG00000185000	validated
tarbase	hsa-miR-212-3p	RFX6	222546	ENSG00000185002	validated
tarbase	hsa-miR-192-5p	RFX6	222546	ENSG00000185002	validated
tarbase	hsa-miR-212-3p	ROBO2	6092	ENSG00000185008	validated
tarbase	hsa-miR-222-3p	AP3M1	26985	ENSG00000185009	validated
tarbase	hsa-miR-301a-3p	AP3M1	26985	ENSG00000185009	validated
tarbase	hsa-miR-582-3p	AP3M1	26985	ENSG00000185009	validated
tarbase	hsa-miR-181a-5p	ANKS1B	56899	ENSG00000185046	validated
tarbase	hsa-miR-301a-3p	SLC24A3	57419	ENSG00000185052	validated
tarbase	hsa-miR-18a-5p	MANEAL	149175	ENSG00000185090	validated
tarbase	hsa-miR-212-3p	FAM43A	131583	ENSG00000185112	validated
tarbase	hsa-miR-15b-5p	NSMCE3	56160	ENSG00000185115	validated
tarbase	hsa-miR-192-5p	C6orf120	387263	ENSG00000185127	validated
tarbase	hsa-miR-301a-3p	PURA	5813	ENSG00000185129	validated
tarbase	hsa-miR-582-5p	PURA	5813	ENSG00000185129	validated
tarbase	hsa-miR-181a-5p	PURA	5813	ENSG00000185129	validated
tarbase	hsa-miR-18a-5p	DDX51	317781	ENSG00000185163	validated
tarbase	hsa-miR-106b-5p	IFITM2	10581	ENSG00000185201	validated
tarbase	hsa-miR-212-3p	IFITM2	10581	ENSG00000185201	validated
tarbase	hsa-miR-192-5p	TNFAIP2	7127	ENSG00000185215	validated
tarbase	hsa-miR-106b-5p	ZNF445	353274	ENSG00000185219	validated
tarbase	hsa-miR-181a-5p	PRPF39	55015	ENSG00000185246	validated
tarbase	hsa-miR-212-3p	UBALD2	283991	ENSG00000185262	validated
tarbase	hsa-miR-212-3p	CDNF	441549	ENSG00000185267	validated
tarbase	hsa-miR-181a-5p	ZBTB37	84614	ENSG00000185278	validated
tarbase	hsa-miR-18a-5p	CCDC137	339230	ENSG00000185298	validated
tarbase	hsa-miR-106b-5p	SOCS1	8651	ENSG00000185338	validated
tarbase	hsa-miR-296-5p	GAS2L1	10634	ENSG00000185340	validated
tarbase	hsa-miR-212-3p	PRKN	5071	ENSG00000185345	validated

tarbase	hsa-miR-296-5p	MAPK11	5600	ENSG00000185386	validated
tarbase	hsa-miR-18a-5p	GPRIN3	285513	ENSG00000185477	validated
tarbase	hsa-miR-18a-5p	PARPBP	55010	ENSG00000185480	validated
tarbase	hsa-miR-18a-5p	ROR1	4919	ENSG00000185483	validated
tarbase	hsa-miR-296-5p	FAAP100	80233	ENSG00000185504	validated
tarbase	hsa-miR-212-3p	IRF7	3665	ENSG00000185507	validated
tarbase	hsa-miR-505-3p	SV2B	9899	ENSG00000185518	validated
tarbase	hsa-miR-106b-5p	NR2F2	7026	ENSG00000185551	validated
tarbase	hsa-miR-192-5p	NR2F2	7026	ENSG00000185551	validated
tarbase	hsa-miR-505-3p	NR2F2	7026	ENSG00000185551	validated
tarbase	hsa-miR-181a-5p	LSAMP	4045	ENSG00000185565	validated
tarbase	hsa-miR-15b-5p	SP1	6667	ENSG00000185591	validated
tarbase	hsa-miR-212-3p	SP1	6667	ENSG00000185591	validated
tarbase	hsa-miR-301a-3p	SP1	6667	ENSG00000185591	validated
tarbase	hsa-miR-18a-5p	PCGF3	10336	ENSG00000185619	validated
tarbase	hsa-miR-212-3p	LMLN	89782	ENSG00000185621	validated
tarbase	hsa-miR-18a-5p	P4HB	5034	ENSG00000185624	validated
tarbase	hsa-miR-326	P4HB	5034	ENSG00000185624	validated
tarbase	hsa-miR-181a-5p	PBX1	5087	ENSG00000185630	validated
tarbase	hsa-miR-18a-5p	ZFP36L1	677	ENSG00000185650	validated
tarbase	hsa-miR-21-5p	ZFP36L1	677	ENSG00000185650	validated
tarbase	hsa-miR-222-3p	ZFP36L1	677	ENSG00000185650	validated
tarbase	hsa-miR-15b-5p	BRWD1	54014	ENSG00000185658	validated
tarbase	hsa-miR-181a-5p	BRWD1	54014	ENSG00000185658	validated
tarbase	hsa-miR-18a-5p	BRWD1	54014	ENSG00000185658	validated
tarbase	hsa-miR-21-5p	BRWD1	54014	ENSG00000185658	validated
tarbase	hsa-miR-582-3p	BRWD1	54014	ENSG00000185658	validated
tarbase	hsa-miR-106b-5p	MYBL1	4603	ENSG00000185697	validated
tarbase	hsa-miR-15b-5p	MYBL1	4603	ENSG00000185697	validated
tarbase	hsa-miR-181a-5p	MYBL1	4603	ENSG00000185697	validated
tarbase	hsa-miR-222-3p	MYBL1	4603	ENSG00000185697	validated
tarbase	hsa-miR-301a-3p	MYBL1	4603	ENSG00000185697	validated
tarbase	hsa-miR-212-3p	MOSMO	730094	ENSG00000185716	validated
tarbase	hsa-miR-326	MOSMO	730094	ENSG00000185716	validated
tarbase	hsa-miR-181a-5p	ANKFY1	51479	ENSG00000185722	validated
tarbase	hsa-miR-582-3p	ANKFY1	51479	ENSG00000185722	validated
tarbase	hsa-miR-106b-5p	YTHDF3	253943	ENSG00000185728	validated
tarbase	hsa-miR-181a-5p	YTHDF3	253943	ENSG00000185728	validated
tarbase	hsa-miR-18a-5p	YTHDF3	253943	ENSG00000185728	validated
tarbase	hsa-miR-212-3p	YTHDF3	253943	ENSG00000185728	validated
tarbase	hsa-miR-222-3p	YTHDF3	253943	ENSG00000185728	validated
tarbase	hsa-miR-212-3p	ZNF696	79943	ENSG00000185730	validated
tarbase	hsa-miR-21-5p	IFIT1	3434	ENSG00000185745	validated
tarbase	hsa-miR-212-3p	IFIT1	3434	ENSG00000185745	validated
tarbase	hsa-miR-181a-5p	KCNQ5	56479	ENSG00000185760	validated
tarbase	hsa-miR-301a-3p	KCNQ5	56479	ENSG00000185760	validated
tarbase	hsa-miR-212-3p	KCNQ5	56479	ENSG00000185760	validated
tarbase	hsa-miR-181a-5p	DMWD	1762	ENSG00000185800	validated
tarbase	hsa-miR-296-5p	DMWD	1762	ENSG00000185800	validated
tarbase	hsa-miR-582-3p	DMWD	1762	ENSG00000185800	validated
tarbase	hsa-miR-15b-5p	NAT8L	339983	ENSG00000185818	validated
tarbase	hsa-miR-181a-5p	NAT8L	339983	ENSG00000185818	validated
tarbase	hsa-miR-582-5p	NAT8L	339983	ENSG00000185818	validated
tarbase	hsa-miR-15b-5p	DNAH14	127602	ENSG00000185842	validated
tarbase	hsa-miR-212-3p	DNAH14	127602	ENSG00000185842	validated
tarbase	hsa-miR-301a-3p	DNAH14	127602	ENSG00000185842	validated
tarbase	hsa-miR-212-3p	TRIM69	140691	ENSG00000185880	validated
tarbase	hsa-miR-212-3p	IFITM1	8519	ENSG00000185885	validated
tarbase	hsa-miR-15b-5p	POMK	84197	ENSG00000185900	validated
tarbase	hsa-miR-301a-3p	SETD4	54093	ENSG00000185917	validated
tarbase	hsa-miR-181a-5p	ZNF267	10308	ENSG00000185947	validated
tarbase	hsa-miR-582-5p	ZNF267	10308	ENSG00000185947	validated
tarbase	hsa-miR-106b-5p	IRS2	8660	ENSG00000185950	validated
tarbase	hsa-miR-181a-5p	IRS2	8660	ENSG00000185950	validated
tarbase	hsa-miR-18a-5p	BICD2	23299	ENSG00000185963	validated
tarbase	hsa-miR-222-3p	BICD2	23299	ENSG00000185963	validated
tarbase	hsa-miR-301a-3p	BICD2	23299	ENSG00000185963	validated
tarbase	hsa-miR-582-5p	BICD2	23299	ENSG00000185963	validated
tarbase	hsa-miR-18a-5p	ZNF566	84924	ENSG00000186017	validated
tarbase	hsa-miR-181a-5p	ZNF566	84924	ENSG00000186017	validated
tarbase	hsa-miR-212-3p	ZNF566	84924	ENSG00000186017	validated

tarbase	hsa-miR-181a-5p	ZNF529	57711	ENSG00000186020	validated
tarbase	hsa-miR-212-3p	ZPBP2	124626	ENSG00000186075	validated
tarbase	hsa-miR-21-5p	KRT5	3852	ENSG00000186081	validated
tarbase	hsa-miR-212-3p	GSAP	54103	ENSG00000186088	validated
tarbase	hsa-miR-326	ANKRD46	157567	ENSG00000186106	validated
tarbase	hsa-miR-15b-5p	ANKRD46	157567	ENSG00000186106	validated
tarbase	hsa-miR-106b-5p	ANKRD46	157567	ENSG00000186106	validated
tarbase	hsa-miR-144-3p	PIP5K1C	23396	ENSG00000186111	validated
tarbase	hsa-miR-582-5p	PIP5K1C	23396	ENSG00000186111	validated
tarbase	hsa-miR-212-3p	ZBTB6	10773	ENSG00000186130	validated
tarbase	hsa-miR-181a-5p	BCL9L	283149	ENSG00000186174	validated
tarbase	hsa-miR-181a-5p	POLR1D	51082	ENSG00000186184	validated
tarbase	hsa-miR-15b-5p	KIF18B	146909	ENSG00000186185	validated
tarbase	hsa-miR-212-3p	KIF18B	146909	ENSG00000186185	validated
tarbase	hsa-miR-301a-3p	BPIFB4	149954	ENSG00000186191	validated
tarbase	hsa-miR-15b-5p	EDARADD	128178	ENSG00000186197	validated
tarbase	hsa-miR-212-3p	EDARADD	128178	ENSG00000186197	validated
tarbase	hsa-miR-21-5p	MARC1	64757	ENSG00000186205	validated
tarbase	hsa-miR-212-3p	BLOC1S4	55330	ENSG00000186222	validated
tarbase	hsa-miR-301a-3p	BLOC1S4	55330	ENSG00000186222	validated
tarbase	hsa-miR-222-3p	ZNF749	388567	ENSG00000186230	validated
tarbase	hsa-miR-212-3p	KLHL32	114792	ENSG00000186231	validated
tarbase	hsa-miR-106b-5p	MRTFB	57496	ENSG00000186260	validated
tarbase	hsa-miR-15b-5p	MRTFB	57496	ENSG00000186260	validated
tarbase	hsa-miR-582-5p	MRTFB	57496	ENSG00000186260	validated
tarbase	hsa-miR-505-3p	BTLA	151888	ENSG00000186265	validated
tarbase	hsa-miR-301a-3p	ZNF17	7565	ENSG00000186272	validated
tarbase	hsa-miR-212-3p	ZNF17	7565	ENSG00000186272	validated
tarbase	hsa-miR-181a-5p	PPP1CC	5501	ENSG00000186298	validated
tarbase	hsa-miR-192-5p	PPP1CC	5501	ENSG00000186298	validated
tarbase	hsa-miR-212-3p	PPP1CC	5501	ENSG00000186298	validated
tarbase	hsa-miR-301a-3p	PPP1CC	5501	ENSG00000186298	validated
tarbase	hsa-miR-326	PPP1CC	5501	ENSG00000186298	validated
tarbase	hsa-miR-582-3p	PPP1CC	5501	ENSG00000186298	validated
tarbase	hsa-miR-212-3p	ZNF555	148254	ENSG00000186300	validated
tarbase	hsa-miR-181a-5p	NAP1L3	4675	ENSG00000186310	validated
tarbase	hsa-miR-296-5p	THBS2	7058	ENSG00000186340	validated
tarbase	hsa-miR-21-5p	RXRA	6256	ENSG00000186350	validated
tarbase	hsa-miR-21-5p	ZNF75D	7626	ENSG00000186376	validated
tarbase	hsa-miR-181a-5p	NKRF	55922	ENSG00000186416	validated
tarbase	hsa-miR-582-3p	NKRF	55922	ENSG00000186416	validated
tarbase	hsa-miR-106b-5p	KPNA4	3840	ENSG00000186432	validated
tarbase	hsa-miR-181a-5p	KPNA4	3840	ENSG00000186432	validated
tarbase	hsa-miR-21-5p	KPNA4	3840	ENSG00000186432	validated
tarbase	hsa-miR-222-3p	KPNA4	3840	ENSG00000186432	validated
tarbase	hsa-miR-212-3p	FAM228A	653140	ENSG00000186453	validated
tarbase	hsa-miR-181a-5p	NAP1L2	4674	ENSG00000186462	validated
tarbase	hsa-miR-505-3p	NAP1L2	4674	ENSG00000186462	validated
tarbase	hsa-miR-15b-5p	RGS7BP	401190	ENSG00000186479	validated
tarbase	hsa-miR-181a-5p	INSIG1	3638	ENSG00000186480	validated
tarbase	hsa-miR-301a-3p	INSIG1	3638	ENSG00000186480	validated
tarbase	hsa-miR-326	INSIG1	3638	ENSG00000186480	validated
tarbase	hsa-miR-21-5p	SEPTIN10	151011	ENSG00000186522	validated
tarbase	hsa-miR-192-5p	GPATCH8	23131	ENSG00000186566	validated
tarbase	hsa-miR-296-5p	GPATCH8	23131	ENSG00000186566	validated
tarbase	hsa-miR-582-3p	GPATCH8	23131	ENSG00000186566	validated
tarbase	hsa-miR-212-3p	CEACAM19	56971	ENSG00000186567	validated
tarbase	hsa-miR-21-5p	NF2	4771	ENSG00000186575	validated
tarbase	hsa-miR-296-5p	SMIM29	221491	ENSG00000186577	validated
tarbase	hsa-miR-582-5p	UBE2H	7328	ENSG00000186591	validated
tarbase	hsa-miR-106b-5p	ZFP91	80829	ENSG00000186660	validated
tarbase	hsa-miR-18a-5p	ZFP91	80829	ENSG00000186660	validated
tarbase	hsa-miR-326	ZFP91	80829	ENSG00000186660	validated
tarbase	hsa-miR-582-3p	ZFP91	80829	ENSG00000186660	validated
tarbase	hsa-miR-106b-5p	SPIN4	139886	ENSG00000186767	validated
tarbase	hsa-miR-301a-3p	SPIN4	139886	ENSG00000186767	validated
tarbase	hsa-miR-212-3p	VSIG10L	147645	ENSG00000186806	validated
tarbase	hsa-miR-181a-5p	ZNF397	84307	ENSG00000186812	validated
tarbase	hsa-miR-21-5p	ZSCAN30	100101467	ENSG00000186814	validated
tarbase	hsa-miR-222-3p	TPCN1	53373	ENSG00000186815	validated
tarbase	hsa-miR-15b-5p	TPCN1	53373	ENSG00000186815	validated

tarbase	hsa-miR-106b-5p	HEXIM1	10614	ENSG00000186834	validated
tarbase	hsa-miR-15b-5p	HEXIM1	10614	ENSG00000186834	validated
tarbase	hsa-miR-181a-5p	HEXIM1	10614	ENSG00000186834	validated
tarbase	hsa-miR-212-3p	HEXIM1	10614	ENSG00000186834	validated
tarbase	hsa-miR-326	HEXIM1	10614	ENSG00000186834	validated
tarbase	hsa-miR-212-3p	TMEM17	200728	ENSG00000186889	validated
tarbase	hsa-miR-106b-5p	ZDHHC17	23390	ENSG00000186908	validated
tarbase	hsa-miR-18a-5p	ZDHHC17	23390	ENSG00000186908	validated
tarbase	hsa-miR-582-3p	ZDHHC17	23390	ENSG00000186908	validated
tarbase	hsa-miR-15b-5p	ZDHHC17	23390	ENSG00000186908	validated
tarbase	hsa-miR-296-5p	ZNF395	55893	ENSG00000186918	validated
tarbase	hsa-miR-106b-5p	PPARA	5465	ENSG00000186951	validated
tarbase	hsa-miR-181a-5p	PPARA	5465	ENSG00000186951	validated
tarbase	hsa-miR-106b-5p	C3orf70	285382	ENSG00000187068	validated
tarbase	hsa-miR-505-3p	C3orf70	285382	ENSG00000187068	validated
tarbase	hsa-miR-505-3p	TEAD1	7003	ENSG00000187079	validated
tarbase	hsa-miR-15b-5p	ENTPD5	957	ENSG00000187097	validated
tarbase	hsa-miR-192-5p	ENTPD5	957	ENSG00000187097	validated
tarbase	hsa-miR-106b-5p	NAP1L1	4673	ENSG00000187109	validated
tarbase	hsa-miR-181a-5p	NAP1L1	4673	ENSG00000187109	validated
tarbase	hsa-miR-21-5p	NAP1L1	4673	ENSG00000187109	validated
tarbase	hsa-miR-212-3p	FOXD3	27022	ENSG00000187140	validated
tarbase	hsa-miR-181a-5p	RNF220	55182	ENSG00000187147	validated
tarbase	hsa-miR-21-5p	SHTN1	57698	ENSG00000187164	validated
tarbase	hsa-miR-181a-5p	TSPYL4	23270	ENSG00000187189	validated
tarbase	hsa-miR-106b-5p	TSPYL4	23270	ENSG00000187189	validated
tarbase	hsa-miR-212-3p	GCNT1	2650	ENSG00000187210	validated
tarbase	hsa-miR-301a-3p	SESTD1	91404	ENSG00000187231	validated
tarbase	hsa-miR-212-3p	FNBP1	23048	ENSG00000187239	validated
tarbase	hsa-miR-21-5p	DYNC2H1	79659	ENSG00000187240	validated
tarbase	hsa-miR-212-3p	FAM9C	171484	ENSG00000187268	validated
tarbase	hsa-miR-192-5p	CHP1	11261	ENSG00000187446	validated
tarbase	hsa-miR-181a-5p	HSPA14	51182	ENSG00000187522	validated
tarbase	hsa-miR-301a-3p	SIRT7	51547	ENSG00000187531	validated
tarbase	hsa-miR-212-3p	PRAMEF10	343071	ENSG00000187545	validated
tarbase	hsa-miR-144-3p	USP7	7874	ENSG00000187555	validated
tarbase	hsa-miR-181a-5p	USP7	7874	ENSG00000187555	validated
tarbase	hsa-miR-582-5p	USP7	7874	ENSG00000187555	validated
tarbase	hsa-miR-212-3p	COX8C	341947	ENSG00000187581	validated
tarbase	hsa-miR-212-3p	MAGEH1	28986	ENSG00000187601	validated
tarbase	hsa-miR-144-3p	TET3	200424	ENSG00000187605	validated
tarbase	hsa-miR-181a-5p	TET3	200424	ENSG00000187605	validated
tarbase	hsa-miR-301a-3p	TET3	200424	ENSG00000187605	validated
tarbase	hsa-miR-582-5p	TET3	200424	ENSG00000187605	validated
tarbase	hsa-miR-21-5p	TET3	200424	ENSG00000187605	validated
tarbase	hsa-miR-21-5p	ZNF286A	57335	ENSG00000187607	validated
tarbase	hsa-miR-212-3p	ISG15	9636	ENSG00000187608	validated
tarbase	hsa-miR-15b-5p	EXD3	54932	ENSG00000187609	validated
tarbase	hsa-miR-582-5p	EXD3	54932	ENSG00000187609	validated
tarbase	hsa-miR-212-3p	C17orf97	400566	ENSG00000187624	validated
tarbase	hsa-miR-106b-5p	SPRY4	81848	ENSG00000187678	validated
tarbase	hsa-miR-15b-5p	SPRY4	81848	ENSG00000187678	validated
tarbase	hsa-miR-15b-5p	TMEM203	94107	ENSG00000187713	validated
tarbase	hsa-miR-582-5p	TCEA1	6917	ENSG00000187735	validated
tarbase	hsa-miR-15b-5p	FANCA	2175	ENSG00000187741	validated
tarbase	hsa-miR-212-3p	ADH1A	124	ENSG00000187758	validated
tarbase	hsa-miR-181a-5p	LIN28B	389421	ENSG00000187772	validated
tarbase	hsa-miR-21-5p	ZNF70	7621	ENSG00000187792	validated
tarbase	hsa-miR-181a-5p	PEAR1	375033	ENSG00000187800	validated
tarbase	hsa-miR-18a-5p	ZFP69B	65243	ENSG00000187801	validated
tarbase	hsa-miR-18a-5p	ZFP69	339559	ENSG00000187815	validated
tarbase	hsa-miR-181a-5p	ZFP69	339559	ENSG00000187815	validated
tarbase	hsa-miR-15b-5p	H1-2	3006	ENSG00000187837	validated
tarbase	hsa-miR-181a-5p	AC091057.1		ENSG00000187951	validated
tarbase	hsa-miR-15b-5p	DNER	92737	ENSG00000187957	validated
tarbase	hsa-miR-296-5p	KLHL17	339451	ENSG00000187961	validated
tarbase	hsa-miR-181a-5p	UBQLN2	29978	ENSG00000188021	validated
tarbase	hsa-miR-582-5p	UBQLN2	29978	ENSG00000188021	validated
tarbase	hsa-miR-106b-5p	ARL4C	10123	ENSG00000188042	validated
tarbase	hsa-miR-144-3p	ARL4C	10123	ENSG00000188042	validated
tarbase	hsa-miR-582-3p	ARL4C	10123	ENSG00000188042	validated

tarbase	hsa-miR-106b-5p	C11orf95	65998	ENSG00000188070	validated
tarbase	hsa-miR-21-5p	C11orf95	65998	ENSG00000188070	validated
tarbase	hsa-miR-296-5p	C11orf95	65998	ENSG00000188070	validated
tarbase	hsa-miR-212-3p	GPR89B	51463	ENSG00000188092	validated
tarbase	hsa-miR-582-3p	MAPK12	6300	ENSG00000188130	validated
tarbase	hsa-miR-15b-5p	COL4A5	1287	ENSG00000188153	validated
tarbase	hsa-miR-15b-5p	AGRN	375790	ENSG00000188157	validated
tarbase	hsa-miR-181a-5p	AGRN	375790	ENSG00000188157	validated
tarbase	hsa-miR-301a-3p	AGRN	375790	ENSG00000188157	validated
tarbase	hsa-miR-222-3p	OTOG	340990	ENSG00000188162	validated
tarbase	hsa-miR-212-3p	TMPPE	643853	ENSG00000188167	validated
tarbase	hsa-miR-326	TMPPE	643853	ENSG00000188167	validated
tarbase	hsa-miR-181a-5p	ZNF626	199777	ENSG00000188171	validated
tarbase	hsa-miR-18a-5p	ZNF626	199777	ENSG00000188171	validated
tarbase	hsa-miR-212-3p	HEPACAM2	253012	ENSG00000188175	validated
tarbase	hsa-miR-15b-5p	ZC3H6	376940	ENSG00000188177	validated
tarbase	hsa-miR-582-5p	ZC3H6	376940	ENSG00000188177	validated
tarbase	hsa-miR-212-3p	ZC3H6	376940	ENSG00000188177	validated
tarbase	hsa-miR-212-3p	DCUN1D3	123879	ENSG00000188215	validated
tarbase	hsa-miR-21-5p	DCUN1D3	123879	ENSG00000188215	validated
tarbase	hsa-miR-18a-5p	TUBB4B	10383	ENSG00000188229	validated
tarbase	hsa-miR-181a-5p	AC010442.1		ENSG00000188242	validated
tarbase	hsa-miR-144-3p	HES4	57801	ENSG00000188290	validated
tarbase	hsa-miR-222-3p	HES4	57801	ENSG00000188290	validated
tarbase	hsa-miR-326	HES4	57801	ENSG00000188290	validated
tarbase	hsa-miR-582-5p	HES4	57801	ENSG00000188290	validated
tarbase	hsa-miR-212-3p	PLSCR1	5359	ENSG00000188313	validated
tarbase	hsa-miR-15b-5p	C3orf62	375341	ENSG00000188315	validated
tarbase	hsa-miR-505-3p	ZNF559	84527	ENSG00000188321	validated
tarbase	hsa-miR-192-5p	FOCAD	54914	ENSG00000188352	validated
tarbase	hsa-miR-21-5p	CHM	1121	ENSG00000188419	validated
tarbase	hsa-miR-582-3p	CHM	1121	ENSG00000188419	validated
tarbase	hsa-miR-18a-5p	H2AX	3014	ENSG00000188486	validated
tarbase	hsa-miR-222-3p	KRTDAP	388533	ENSG00000188508	validated
tarbase	hsa-miR-21-5p	NBR1	4077	ENSG00000188554	validated
tarbase	hsa-miR-18a-5p	RALGAPA2	57186	ENSG00000188559	validated
tarbase	hsa-miR-15b-5p	RTL6	84247	ENSG00000188636	validated
tarbase	hsa-miR-181a-5p	RTL6	84247	ENSG00000188636	validated
tarbase	hsa-miR-192-5p	S100A16	140576	ENSG00000188643	validated
tarbase	hsa-miR-21-5p	S100A16	140576	ENSG00000188643	validated
tarbase	hsa-miR-18a-5p	PTAR1	375743	ENSG00000188647	validated
tarbase	hsa-miR-212-3p	SAXO2	283726	ENSG00000188659	validated
tarbase	hsa-miR-192-5p	RHCE	6006	ENSG00000188672	validated
tarbase	hsa-miR-505-3p	PARVB	29780	ENSG00000188677	validated
tarbase	hsa-miR-326	SMIM15	643155	ENSG00000188725	validated
tarbase	hsa-miR-106b-5p	SMIM15	643155	ENSG00000188725	validated
tarbase	hsa-miR-106b-5p	RBM34	23029	ENSG00000188739	validated
tarbase	hsa-miR-144-3p	SKOR1	390598	ENSG00000188779	validated
tarbase	hsa-miR-15b-5p	MTF1	4520	ENSG00000188786	validated
tarbase	hsa-miR-222-3p	MTF1	4520	ENSG00000188786	validated
tarbase	hsa-miR-301a-3p	MTF1	4520	ENSG00000188786	validated
tarbase	hsa-miR-192-5p	TMEM201	199953	ENSG00000188807	validated
tarbase	hsa-miR-222-3p	NHLRC3	387921	ENSG00000188811	validated
tarbase	hsa-miR-212-3p	CNR2	1269	ENSG00000188822	validated
tarbase	hsa-miR-106b-5p	BEND4	389206	ENSG00000188848	validated
tarbase	hsa-miR-181a-5p	BEND4	389206	ENSG00000188848	validated
tarbase	hsa-miR-15b-5p	BEND4	389206	ENSG00000188848	validated
tarbase	hsa-miR-106b-5p	MSL1	339287	ENSG00000188895	validated
tarbase	hsa-miR-212-3p	MSL1	339287	ENSG00000188895	validated
tarbase	hsa-miR-582-3p	MSL1	339287	ENSG00000188895	validated
tarbase	hsa-miR-181a-5p	TRMT2B	79979	ENSG00000188917	validated
tarbase	hsa-miR-15b-5p	NOC2L	26155	ENSG00000188976	validated
tarbase	hsa-miR-301a-3p	NOC2L	26155	ENSG00000188976	validated
tarbase	hsa-miR-212-3p	LRRC66	339977	ENSG00000188993	validated
tarbase	hsa-miR-106b-5p	ZNF292	23036	ENSG00000188994	validated
tarbase	hsa-miR-18a-5p	ZNF292	23036	ENSG00000188994	validated
tarbase	hsa-miR-582-3p	ADAT2	134637	ENSG00000189007	validated
tarbase	hsa-miR-181a-5p	ZNF567	163081	ENSG00000189042	validated
tarbase	hsa-miR-21-5p	ZNF567	163081	ENSG00000189042	validated
tarbase	hsa-miR-212-3p	APOD	347	ENSG00000189058	validated
tarbase	hsa-miR-106b-5p	H1-0	3005	ENSG00000189060	validated

tarbase	hsa-miR-18a-5p	H1-0	3005	ENSG00000189060	validated
tarbase	hsa-miR-582-3p	H1-0	3005	ENSG00000189060	validated
tarbase	hsa-miR-106b-5p	LITAF	9516	ENSG00000189067	validated
tarbase	hsa-miR-181a-5p	LITAF	9516	ENSG00000189067	validated
tarbase	hsa-miR-181a-5p	SF3B3	23450	ENSG00000189091	validated
tarbase	hsa-miR-192-5p	SF3B3	23450	ENSG00000189091	validated
tarbase	hsa-miR-212-3p	SF3B3	23450	ENSG00000189091	validated
tarbase	hsa-miR-21-5p	SF3B3	23450	ENSG00000189091	validated
tarbase	hsa-miR-15b-5p	CLDN4	1364	ENSG00000189143	validated
tarbase	hsa-miR-192-5p	ZNF573	126231	ENSG00000189144	validated
tarbase	hsa-miR-181a-5p	ZNF573	126231	ENSG00000189144	validated
tarbase	hsa-miR-212-3p	JPT1	51155	ENSG00000189159	validated
tarbase	hsa-miR-181a-5p	S100A13	6284	ENSG00000189171	validated
tarbase	hsa-miR-106b-5p	TSPYL1	7259	ENSG00000189241	validated
tarbase	hsa-miR-18a-5p	TSPYL1	7259	ENSG00000189241	validated
tarbase	hsa-miR-212-3p	TSPYL1	7259	ENSG00000189241	validated
tarbase	hsa-miR-296-5p	TSPYL1	7259	ENSG00000189241	validated
tarbase	hsa-miR-301a-3p	TSPYL1	7259	ENSG00000189241	validated
tarbase	hsa-miR-181a-5p	TSPYL1	7259	ENSG00000189241	validated
tarbase	hsa-miR-212-3p	GJB5	2709	ENSG00000189280	validated
tarbase	hsa-miR-582-3p	FHIT	2272	ENSG00000189283	validated
tarbase	hsa-miR-106b-5p	LIN54	132660	ENSG00000189308	validated
tarbase	hsa-miR-181a-5p	LIN54	132660	ENSG00000189308	validated
tarbase	hsa-miR-222-3p	FAM53B	9679	ENSG00000189319	validated
tarbase	hsa-miR-301a-3p	KAZN	23254	ENSG00000189337	validated
tarbase	hsa-miR-296-5p	SLC35E2B	728661	ENSG00000189339	validated
tarbase	hsa-miR-15b-5p	TOGARAM2	165186	ENSG00000189350	validated
tarbase	hsa-miR-106b-5p	GSPT2	23708	ENSG00000189369	validated
tarbase	hsa-miR-106b-5p	ZNF724	440519	ENSG00000196081	validated
tarbase	hsa-miR-18a-5p	ZNF724	440519	ENSG00000196081	validated
tarbase	hsa-miR-144-3p	IL1RAP	3556	ENSG00000196083	validated
tarbase	hsa-miR-301a-3p	IL1RAP	3556	ENSG00000196083	validated
tarbase	hsa-miR-15b-5p	PTPRT	11122	ENSG00000196090	validated
tarbase	hsa-miR-181a-5p	PTPRT	11122	ENSG00000196090	validated
tarbase	hsa-miR-296-5p	SERPINA3	12	ENSG00000196136	validated
tarbase	hsa-miR-212-3p	AKR1C3	8644	ENSG00000196139	validated
tarbase	hsa-miR-222-3p	AKR1C3	8644	ENSG00000196139	validated
tarbase	hsa-miR-222-3p	SPATS2L	26010	ENSG00000196141	validated
tarbase	hsa-miR-301a-3p	ZNF250	58500	ENSG00000196150	validated
tarbase	hsa-miR-15b-5p	WDSUB1	151525	ENSG00000196151	validated
tarbase	hsa-miR-18a-5p	ZNF681	148213	ENSG00000196172	validated
tarbase	hsa-miR-15b-5p	ACADSB	36	ENSG00000196177	validated
tarbase	hsa-miR-296-5p	STK40	83931	ENSG00000196182	validated
tarbase	hsa-miR-301a-3p	TMEM63A	9725	ENSG00000196187	validated
tarbase	hsa-miR-326	TMEM63A	9725	ENSG00000196187	validated
tarbase	hsa-miR-301a-3p	MPHOSPH8	54737	ENSG00000196199	validated
tarbase	hsa-miR-582-5p	MPHOSPH8	54737	ENSG00000196199	validated
tarbase	hsa-miR-106b-5p	GREB1	9687	ENSG00000196208	validated
tarbase	hsa-miR-15b-5p	GREB1	9687	ENSG00000196208	validated
tarbase	hsa-miR-181a-5p	ZNF766	90321	ENSG00000196214	validated
tarbase	hsa-miR-15b-5p	SRGAP3	9901	ENSG00000196220	validated
tarbase	hsa-miR-15b-5p	FAM217B	63939	ENSG00000196227	validated
tarbase	hsa-miR-18a-5p	TUBB	203068	ENSG00000196230	validated
tarbase	hsa-miR-192-5p	TUBB	203068	ENSG00000196230	validated
tarbase	hsa-miR-21-5p	TUBB	203068	ENSG00000196230	validated
tarbase	hsa-miR-301a-3p	TUBB	203068	ENSG00000196230	validated
tarbase	hsa-miR-582-3p	TUBB	203068	ENSG00000196230	validated
tarbase	hsa-miR-106b-5p	LCOR	84458	ENSG00000196233	validated
tarbase	hsa-miR-18a-5p	LCOR	84458	ENSG00000196233	validated
tarbase	hsa-miR-212-3p	LCOR	84458	ENSG00000196233	validated
tarbase	hsa-miR-21-5p	LCOR	84458	ENSG00000196233	validated
tarbase	hsa-miR-301a-3p	LCOR	84458	ENSG00000196233	validated
tarbase	hsa-miR-582-5p	LCOR	84458	ENSG00000196233	validated
tarbase	hsa-miR-15b-5p	XPNPEP3	63929	ENSG00000196236	validated
tarbase	hsa-miR-326	XPNPEP3	63929	ENSG00000196236	validated
tarbase	hsa-miR-18a-5p	ZNF107	51427	ENSG00000196247	validated
tarbase	hsa-miR-212-3p	ZNF107	51427	ENSG00000196247	validated
tarbase	hsa-miR-582-5p	ZNF107	51427	ENSG00000196247	validated
tarbase	hsa-miR-21-5p	ZNF107	51427	ENSG00000196247	validated
tarbase	hsa-miR-21-5p	PPIA	5478	ENSG00000196262	validated
tarbase	hsa-miR-582-5p	PPIA	5478	ENSG00000196262	validated

tarbase	hsa-miR-212-3p	ZNF471	57573	ENSG00000196263	validated
tarbase	hsa-miR-18a-5p	ZNF493	284443	ENSG00000196268	validated
tarbase	hsa-miR-21-5p	ZNF493	284443	ENSG00000196268	validated
tarbase	hsa-miR-181a-5p	NIF3L1	60491	ENSG00000196290	validated
tarbase	hsa-miR-15b-5p	IARS1	3376	ENSG00000196305	validated
tarbase	hsa-miR-18a-5p	IARS1	3376	ENSG00000196305	validated
tarbase	hsa-miR-144-3p	POM121	9883	ENSG00000196313	validated
tarbase	hsa-miR-212-3p	POM121	9883	ENSG00000196313	validated
tarbase	hsa-miR-21-5p	ZBTB44	29068	ENSG00000196323	validated
tarbase	hsa-miR-181a-5p	ZBTB44	29068	ENSG00000196323	validated
tarbase	hsa-miR-212-3p	STK31	56164	ENSG00000196335	validated
tarbase	hsa-miR-192-5p	CD55	1604	ENSG00000196352	validated
tarbase	hsa-miR-192-5p	CPNE4	131034	ENSG00000196353	validated
tarbase	hsa-miR-181a-5p	ZNF565	147929	ENSG00000196357	validated
tarbase	hsa-miR-15b-5p	TRRAP	8295	ENSG00000196367	validated
tarbase	hsa-miR-181a-5p	TRRAP	8295	ENSG00000196367	validated
tarbase	hsa-miR-212-3p	TRRAP	8295	ENSG00000196367	validated
tarbase	hsa-miR-15b-5p	FUT4	2526	ENSG00000196371	validated
tarbase	hsa-miR-582-5p	ZNF140	7699	ENSG00000196387	validated
tarbase	hsa-miR-192-5p	PTPN1	5770	ENSG00000196396	validated
tarbase	hsa-miR-301a-3p	PTPN1	5770	ENSG00000196396	validated
tarbase	hsa-miR-301a-3p	EPHB4	2050	ENSG00000196411	validated
tarbase	hsa-miR-181a-5p	ZNF124	7678	ENSG00000196418	validated
tarbase	hsa-miR-192-5p	XRCC6	2547	ENSG00000196419	validated
tarbase	hsa-miR-21-5p	XRCC6	2547	ENSG00000196419	validated
tarbase	hsa-miR-106b-5p	TSC22D2	9819	ENSG00000196428	validated
tarbase	hsa-miR-301a-3p	TSC22D2	9819	ENSG00000196428	validated
tarbase	hsa-miR-181a-5p	TSC22D2	9819	ENSG00000196428	validated
tarbase	hsa-miR-181a-5p	ZNF569	148266	ENSG00000196437	validated
tarbase	hsa-miR-192-5p	ARMCX4	100131755	ENSG00000196440	validated
tarbase	hsa-miR-18a-5p	YRDC	79693	ENSG00000196449	validated
tarbase	hsa-miR-106b-5p	YRDC	79693	ENSG00000196449	validated
tarbase	hsa-miR-181a-5p	YRDC	79693	ENSG00000196449	validated
tarbase	hsa-miR-582-5p	SIAH1	6477	ENSG00000196470	validated
tarbase	hsa-miR-15b-5p	SIAH1	6477	ENSG00000196470	validated
tarbase	hsa-miR-222-3p	SIAH1	6477	ENSG00000196470	validated
tarbase	hsa-miR-212-3p	ESRRG	2104	ENSG00000196482	validated
tarbase	hsa-miR-192-5p	NCOR2	9612	ENSG00000196498	validated
tarbase	hsa-miR-15b-5p	PRPF40A	55660	ENSG00000196504	validated
tarbase	hsa-miR-582-5p	PRPF40A	55660	ENSG00000196504	validated
tarbase	hsa-miR-21-5p	TCEAL3	85012	ENSG00000196507	validated
tarbase	hsa-miR-181a-5p	ANAPC7	51434	ENSG00000196510	validated
tarbase	hsa-miR-222-3p	SLC6A9	6536	ENSG00000196517	validated
tarbase	hsa-miR-15b-5p	AFAP1	60312	ENSG00000196526	validated
tarbase	hsa-miR-181a-5p	AFAP1	60312	ENSG00000196526	validated
tarbase	hsa-miR-212-3p	AFAP1	60312	ENSG00000196526	validated
tarbase	hsa-miR-582-5p	AFAP1	60312	ENSG00000196526	validated
tarbase	hsa-miR-18a-5p	NACA	4666	ENSG00000196531	validated
tarbase	hsa-miR-15b-5p	MME	4311	ENSG00000196549	validated
tarbase	hsa-miR-222-3p	MME	4311	ENSG00000196549	validated
tarbase	hsa-miR-296-5p	MME	4311	ENSG00000196549	validated
tarbase	hsa-miR-212-3p	MME	4311	ENSG00000196549	validated
tarbase	hsa-miR-106b-5p	SULF2	55959	ENSG00000196562	validated
tarbase	hsa-miR-15b-5p	PLXNB2	23654	ENSG00000196576	validated
tarbase	hsa-miR-106b-5p	XRCC2	7516	ENSG00000196584	validated
tarbase	hsa-miR-21-5p	HDAC2	3066	ENSG00000196591	validated
tarbase	hsa-miR-106b-5p	UGT2B15	7366	ENSG00000196620	validated
tarbase	hsa-miR-21-5p	TCF4	6925	ENSG00000196628	validated
tarbase	hsa-miR-301a-3p	TCF4	6925	ENSG00000196628	validated
tarbase	hsa-miR-106b-5p	RABL6	55684	ENSG00000196642	validated
tarbase	hsa-miR-15b-5p	RABL6	55684	ENSG00000196642	validated
tarbase	hsa-miR-296-5p	RABL6	55684	ENSG00000196642	validated
tarbase	hsa-miR-326	RABL6	55684	ENSG00000196642	validated
tarbase	hsa-miR-106b-5p	ZNF502	91392	ENSG00000196653	validated
tarbase	hsa-miR-15b-5p	TTC30B	150737	ENSG00000196659	validated
tarbase	hsa-miR-212-3p	TTC30B	150737	ENSG00000196659	validated
tarbase	hsa-miR-21-5p	TECPR2	9895	ENSG00000196663	validated
tarbase	hsa-miR-181a-5p	ZFP62	643836	ENSG00000196670	validated
tarbase	hsa-miR-21-5p	HSH2D	84941	ENSG00000196684	validated
tarbase	hsa-miR-15b-5p	ZNF33B	7582	ENSG00000196693	validated
tarbase	hsa-miR-181a-5p	ZNF33B	7582	ENSG00000196693	validated

tarbase	hsa-miR-296-5p	ZNF512B	57473	ENSG00000196700	validated
tarbase	hsa-miR-326	ZNF512B	57473	ENSG00000196700	validated
tarbase	hsa-miR-181a-5p	ZNF431	170959	ENSG00000196705	validated
tarbase	hsa-miR-15b-5p	NF1	4763	ENSG00000196712	validated
tarbase	hsa-miR-181a-5p	NF1	4763	ENSG00000196712	validated
tarbase	hsa-miR-212-3p	NF1	4763	ENSG00000196712	validated
tarbase	hsa-miR-21-5p	NF1	4763	ENSG00000196712	validated
tarbase	hsa-miR-582-5p	NF1	4763	ENSG00000196712	validated
tarbase	hsa-miR-106b-5p	VKORC1L1	154807	ENSG00000196715	validated
tarbase	hsa-miR-181a-5p	VKORC1L1	154807	ENSG00000196715	validated
tarbase	hsa-miR-296-5p	VKORC1L1	154807	ENSG00000196715	validated
tarbase	hsa-miR-15b-5p	VKORC1L1	154807	ENSG00000196715	validated
tarbase	hsa-miR-181a-5p	DAPK1	1612	ENSG00000196730	validated
tarbase	hsa-miR-18a-5p	DAPK1	1612	ENSG00000196730	validated
tarbase	hsa-miR-505-3p	DAPK1	1612	ENSG00000196730	validated
tarbase	hsa-miR-15b-5p	HLA-DQA1	3117	ENSG00000196735	validated
tarbase	hsa-miR-18a-5p	HLA-DQA1	3117	ENSG00000196735	validated
tarbase	hsa-miR-212-3p	HLA-DQA1	3117	ENSG00000196735	validated
tarbase	hsa-miR-181a-5p	ZNF700	90592	ENSG00000196757	validated
tarbase	hsa-miR-301a-3p	STRN3	29966	ENSG00000196792	validated
tarbase	hsa-miR-212-3p	STRN3	29966	ENSG00000196792	validated
tarbase	hsa-miR-181a-5p	ZNF239	8187	ENSG00000196793	validated
tarbase	hsa-miR-181a-5p	ILRUN	64771	ENSG00000196821	validated
tarbase	hsa-miR-212-3p	PATE2	399967	ENSG00000196844	validated
tarbase	hsa-miR-106b-5p	PPTC7	160760	ENSG00000196850	validated
tarbase	hsa-miR-181a-5p	PPTC7	160760	ENSG00000196850	validated
tarbase	hsa-miR-582-3p	PPTC7	160760	ENSG00000196850	validated
tarbase	hsa-miR-582-5p	PPTC7	160760	ENSG00000196850	validated
tarbase	hsa-miR-15b-5p	NHLRC2	374354	ENSG00000196865	validated
tarbase	hsa-miR-192-5p	NHLRC2	374354	ENSG00000196865	validated
tarbase	hsa-miR-582-5p	NHLRC2	374354	ENSG00000196865	validated
tarbase	hsa-miR-212-3p	NHLRC2	374354	ENSG00000196865	validated
tarbase	hsa-miR-15b-5p	KPNA5	3841	ENSG00000196911	validated
tarbase	hsa-miR-192-5p	ARHGEF12	23365	ENSG00000196914	validated
tarbase	hsa-miR-212-3p	ARHGEF12	23365	ENSG00000196914	validated
tarbase	hsa-miR-296-5p	ARHGEF12	23365	ENSG00000196914	validated
tarbase	hsa-miR-326	ARHGEF12	23365	ENSG00000196914	validated
tarbase	hsa-miR-15b-5p	ARHGEF12	23365	ENSG00000196914	validated
tarbase	hsa-miR-212-3p	FLNA	2316	ENSG00000196924	validated
tarbase	hsa-miR-326	FLNA	2316	ENSG00000196924	validated
tarbase	hsa-miR-18a-5p	SRGAP1	57522	ENSG00000196935	validated
tarbase	hsa-miR-212-3p	SRGAP1	57522	ENSG00000196935	validated
tarbase	hsa-miR-21-5p	SRGAP1	57522	ENSG00000196935	validated
tarbase	hsa-miR-15b-5p	NOP9	161424	ENSG00000196943	validated
tarbase	hsa-miR-15b-5p	SLC39A10	57181	ENSG00000196950	validated
tarbase	hsa-miR-181a-5p	SLC39A10	57181	ENSG00000196950	validated
tarbase	hsa-miR-21-5p	AP2A1	160	ENSG00000196961	validated
tarbase	hsa-miR-296-5p	AP2A1	160	ENSG00000196961	validated
tarbase	hsa-miR-15b-5p	ZNF585A	199704	ENSG00000196967	validated
tarbase	hsa-miR-15b-5p	ANXA4	307	ENSG00000196975	validated
tarbase	hsa-miR-15b-5p	METTL9	51108	ENSG00000197006	validated
tarbase	hsa-miR-181a-5p	METTL9	51108	ENSG00000197006	validated
tarbase	hsa-miR-106b-5p	ZNF138	7697	ENSG00000197008	validated
tarbase	hsa-miR-18a-5p	ZNF470	388566	ENSG00000197016	validated
tarbase	hsa-miR-18a-5p	ZNF100	163227	ENSG00000197020	validated
tarbase	hsa-miR-106b-5p	ZNF398	57541	ENSG00000197024	validated
tarbase	hsa-miR-301a-3p	ZSCAN25	221785	ENSG00000197037	validated
tarbase	hsa-miR-212-3p	ZNF441	126068	ENSG00000197044	validated
tarbase	hsa-miR-106b-5p	GMFB	2764	ENSG00000197045	validated
tarbase	hsa-miR-181a-5p	GMFB	2764	ENSG00000197045	validated
tarbase	hsa-miR-181a-5p	ZMYM1	79830	ENSG00000197056	validated
tarbase	hsa-miR-582-3p	ZMYM1	79830	ENSG00000197056	validated
tarbase	hsa-miR-296-5p	H4C3	8364	ENSG00000197061	validated
tarbase	hsa-miR-18a-5p	H4C3	8364	ENSG00000197061	validated
tarbase	hsa-miR-106b-5p	ZSCAN26	7741	ENSG00000197062	validated
tarbase	hsa-miR-106b-5p	MAFG	4097	ENSG00000197063	validated
tarbase	hsa-miR-15b-5p	MAFG	4097	ENSG00000197063	validated
tarbase	hsa-miR-582-5p	IGF2R	3482	ENSG00000197081	validated
tarbase	hsa-miR-106b-5p	DYNC1H1	1778	ENSG00000197102	validated
tarbase	hsa-miR-15b-5p	DYNC1H1	1778	ENSG00000197102	validated
tarbase	hsa-miR-181a-5p	DYNC1H1	1778	ENSG00000197102	validated

tarbase	hsa-miR-582-5p	DYNC1H1	1778	ENSG00000197102	validated
tarbase	hsa-miR-181a-5p	PCBP2	5094	ENSG00000197111	validated
tarbase	hsa-miR-21-5p	PCBP2	5094	ENSG00000197111	validated
tarbase	hsa-miR-106b-5p	PGAP1	80055	ENSG00000197121	validated
tarbase	hsa-miR-181a-5p	ZNF772	400720	ENSG00000197128	validated
tarbase	hsa-miR-21-5p	LRRC8B	23507	ENSG00000197147	validated
tarbase	hsa-miR-106b-5p	PSMD12	5718	ENSG00000197170	validated
tarbase	hsa-miR-222-3p	PSMD12	5718	ENSG00000197170	validated
tarbase	hsa-miR-15b-5p	MIRLET7BHG		ENSG00000197182	validated
tarbase	hsa-miR-21-5p	NOL4L	140688	ENSG00000197183	validated
tarbase	hsa-miR-15b-5p	ENTPD4	9583	ENSG00000197217	validated
tarbase	hsa-miR-18a-5p	TBC1D9B	23061	ENSG00000197226	validated
tarbase	hsa-miR-192-5p	TBC1D9B	23061	ENSG00000197226	validated
tarbase	hsa-miR-15b-5p	SERPINA1	5265	ENSG00000197249	validated
tarbase	hsa-miR-212-3p	SERPINA1	5265	ENSG00000197249	validated
tarbase	hsa-miR-296-5p	SERPINA1	5265	ENSG00000197249	validated
tarbase	hsa-miR-301a-3p	KANK2	25959	ENSG00000197256	validated
tarbase	hsa-miR-15b-5p	SYNGAP1	8831	ENSG00000197283	validated
tarbase	hsa-miR-106b-5p	FITM2	128486	ENSG00000197296	validated
tarbase	hsa-miR-15b-5p	FITM2	128486	ENSG00000197296	validated
tarbase	hsa-miR-15b-5p	DDI2	84301	ENSG00000197312	validated
tarbase	hsa-miR-181a-5p	DDI2	84301	ENSG00000197312	validated
tarbase	hsa-miR-21-5p	DDI2	84301	ENSG00000197312	validated
tarbase	hsa-miR-15b-5p	SVIL	6840	ENSG00000197321	validated
tarbase	hsa-miR-15b-5p	C17orf102		ENSG00000197322	validated
tarbase	hsa-miR-15b-5p	TRIM33	51592	ENSG00000197323	validated
tarbase	hsa-miR-181a-5p	TRIM33	51592	ENSG00000197323	validated
tarbase	hsa-miR-222-3p	TRIM33	51592	ENSG00000197323	validated
tarbase	hsa-miR-106b-5p	PELI1	57162	ENSG00000197329	validated
tarbase	hsa-miR-181a-5p	PELI1	57162	ENSG00000197329	validated
tarbase	hsa-miR-18a-5p	PELI1	57162	ENSG00000197329	validated
tarbase	hsa-miR-181a-5p	ZNF655	79027	ENSG00000197343	validated
tarbase	hsa-miR-181a-5p	ZNF675	171392	ENSG00000197372	validated
tarbase	hsa-miR-212-3p	ZNF675	171392	ENSG00000197372	validated
tarbase	hsa-miR-582-3p	ZNF675	171392	ENSG00000197372	validated
tarbase	hsa-miR-106b-5p	ADARB1	104	ENSG00000197381	validated
tarbase	hsa-miR-181a-5p	ADARB1	104	ENSG00000197381	validated
tarbase	hsa-miR-301a-3p	ADARB1	104	ENSG00000197381	validated
tarbase	hsa-miR-15b-5p	HTT	3064	ENSG00000197386	validated
tarbase	hsa-miR-192-5p	HTT	3064	ENSG00000197386	validated
tarbase	hsa-miR-18a-5p	HTT	3064	ENSG00000197386	validated
tarbase	hsa-miR-106b-5p	MAP3K5	4217	ENSG00000197442	validated
tarbase	hsa-miR-181a-5p	MAP3K5	4217	ENSG00000197442	validated
tarbase	hsa-miR-15b-5p	PDGFA	5154	ENSG00000197461	validated
tarbase	hsa-miR-212-3p	GALP	85569	ENSG00000197487	validated
tarbase	hsa-miR-21-5p	MYO5A	4644	ENSG00000197535	validated
tarbase	hsa-miR-301a-3p	MYO5A	4644	ENSG00000197535	validated
tarbase	hsa-miR-181a-5p	MYO5A	4644	ENSG00000197535	validated
tarbase	hsa-miR-106b-5p	SIPA1L1	26037	ENSG00000197555	validated
tarbase	hsa-miR-212-3p	TTC30A	92104	ENSG00000197557	validated
tarbase	hsa-miR-106b-5p	PIGN	23556	ENSG00000197563	validated
tarbase	hsa-miR-212-3p	ZNF624	57547	ENSG00000197566	validated
tarbase	hsa-miR-181a-5p	ENTPD6	955	ENSG00000197586	validated
tarbase	hsa-miR-18a-5p	ENPP1	5167	ENSG00000197594	validated
tarbase	hsa-miR-181a-5p	FAR1	84188	ENSG00000197601	validated
tarbase	hsa-miR-212-3p	CPLANE1	65250	ENSG00000197603	validated
tarbase	hsa-miR-301a-3p	MYH6	4624	ENSG00000197616	validated
tarbase	hsa-miR-15b-5p	CDC42SE1	56882	ENSG00000197622	validated
tarbase	hsa-miR-18a-5p	CDC42SE1	56882	ENSG00000197622	validated
tarbase	hsa-miR-21-5p	CDC42SE1	56882	ENSG00000197622	validated
tarbase	hsa-miR-296-5p	CDC42SE1	56882	ENSG00000197622	validated
tarbase	hsa-miR-21-5p	SERPINB2	5055	ENSG00000197632	validated
tarbase	hsa-miR-21-5p	SPTAN1	6709	ENSG00000197694	validated
tarbase	hsa-miR-18a-5p	KLHL14	57565	ENSG00000197705	validated
tarbase	hsa-miR-18a-5p	FAM114A1	92689	ENSG00000197712	validated
tarbase	hsa-miR-181a-5p	ZNF460	10794	ENSG00000197714	validated
tarbase	hsa-miR-192-5p	ZNF460	10794	ENSG00000197714	validated
tarbase	hsa-miR-301a-3p	ZNF460	10794	ENSG00000197714	validated
tarbase	hsa-miR-15b-5p	PSAP	5660	ENSG00000197746	validated
tarbase	hsa-miR-181a-5p	PSAP	5660	ENSG00000197746	validated
tarbase	hsa-miR-301a-3p	PSAP	5660	ENSG00000197746	validated

tarbase	hsa-miR-582-5p	PSAP	5660	ENSG00000197746	validated
tarbase	hsa-miR-212-3p	CFAP43	80217	ENSG00000197748	validated
tarbase	hsa-miR-212-3p	HOXC6	3223	ENSG00000197757	validated
tarbase	hsa-miR-301a-3p	TXNRD3	114112	ENSG00000197763	validated
tarbase	hsa-miR-18a-5p	MCMBP	79892	ENSG00000197771	validated
tarbase	hsa-miR-106b-5p	ZNF81	347344	ENSG00000197779	validated
tarbase	hsa-miR-181a-5p	ZNF780A	284323	ENSG00000197782	validated
tarbase	hsa-miR-106b-5p	ZNF461	92283	ENSG00000197808	validated
tarbase	hsa-miR-18a-5p	OCLN	100506658	ENSG00000197822	validated
tarbase	hsa-miR-296-5p	H4-16	121504	ENSG00000197837	validated
tarbase	hsa-miR-15b-5p	ZNF181	339318	ENSG00000197841	validated
tarbase	hsa-miR-18a-5p	ZNF181	339318	ENSG00000197841	validated
tarbase	hsa-miR-15b-5p	GPAA1	8733	ENSG00000197858	validated
tarbase	hsa-miR-181a-5p	GPAA1	8733	ENSG00000197858	validated
tarbase	hsa-miR-222-3p	SGTB	54557	ENSG00000197860	validated
tarbase	hsa-miR-582-5p	NKIRAS1	28512	ENSG00000197885	validated
tarbase	hsa-miR-212-3p	MEIG1	644890	ENSG00000197889	validated
tarbase	hsa-miR-181a-5p	ADH5	128	ENSG00000197894	validated
tarbase	hsa-miR-326	SPG7	6687	ENSG00000197912	validated
tarbase	hsa-miR-18a-5p	ERO1A	30001	ENSG00000197930	validated
tarbase	hsa-miR-181a-5p	ERO1A	30001	ENSG00000197930	validated
tarbase	hsa-miR-181a-5p	ZNF823	55552	ENSG00000197933	validated
tarbase	hsa-miR-106b-5p	DNM3	26052	ENSG00000197959	validated
tarbase	hsa-miR-18a-5p	DNM3	26052	ENSG00000197959	validated
tarbase	hsa-miR-181a-5p	DNM3	26052	ENSG00000197959	validated
tarbase	hsa-miR-212-3p	DNM3	26052	ENSG00000197959	validated
tarbase	hsa-miR-106b-5p	ZNF121	7675	ENSG00000197961	validated
tarbase	hsa-miR-222-3p	ZNF121	7675	ENSG00000197961	validated
tarbase	hsa-miR-582-5p	ZNF121	7675	ENSG00000197961	validated
tarbase	hsa-miR-181a-5p	VPS13A	23230	ENSG00000197969	validated
tarbase	hsa-miR-15b-5p	MBP	4155	ENSG00000197971	validated
tarbase	hsa-miR-15b-5p	NOL8	55035	ENSG00000198000	validated
tarbase	hsa-miR-192-5p	AC093012.1	51135	ENSG00000198001	validated
tarbase	hsa-miR-21-5p	AC093012.1	51135	ENSG00000198001	validated
tarbase	hsa-miR-212-3p	CCDC151	115948	ENSG00000198003	validated
tarbase	hsa-miR-181a-5p	DLGAP2	9228	ENSG00000198010	validated
tarbase	hsa-miR-18a-5p	MRPL42	28977	ENSG00000198015	validated
tarbase	hsa-miR-18a-5p	ENTPD7	57089	ENSG00000198018	validated
tarbase	hsa-miR-582-5p	ENTPD7	57089	ENSG00000198018	validated
tarbase	hsa-miR-181a-5p	ZNF273	10793	ENSG00000198039	validated
tarbase	hsa-miR-582-5p	MAK16	84549	ENSG00000198042	validated
tarbase	hsa-miR-326	SIRPA	140885	ENSG00000198053	validated
tarbase	hsa-miR-582-3p	SIRPA	140885	ENSG00000198053	validated
tarbase	hsa-miR-15b-5p	MARCHF5	54708	ENSG00000198060	validated
tarbase	hsa-miR-212-3p	AKR1B10	57016	ENSG00000198074	validated
tarbase	hsa-miR-15b-5p	ZBTB14	7541	ENSG00000198081	validated
tarbase	hsa-miR-106b-5p	CD2AP	23607	ENSG00000198087	validated
tarbase	hsa-miR-181a-5p	CD2AP	23607	ENSG00000198087	validated
tarbase	hsa-miR-21-5p	CD2AP	23607	ENSG00000198087	validated
tarbase	hsa-miR-301a-3p	CD2AP	23607	ENSG00000198087	validated
tarbase	hsa-miR-301a-3p	ZNF649	65251	ENSG00000198093	validated
tarbase	hsa-miR-181a-5p	LPAR1	1902	ENSG00000198121	validated
tarbase	hsa-miR-15b-5p	HIBCH	26275	ENSG00000198130	validated
tarbase	hsa-miR-181a-5p	ZNF544	27300	ENSG00000198131	validated
tarbase	hsa-miR-106b-5p	SOWAHC	65124	ENSG00000198142	validated
tarbase	hsa-miR-582-5p	SOWAHC	65124	ENSG00000198142	validated
tarbase	hsa-miR-301a-3p	MIER1	57708	ENSG00000198160	validated
tarbase	hsa-miR-106b-5p	MAN1A2	10905	ENSG00000198162	validated
tarbase	hsa-miR-15b-5p	MAN1A2	10905	ENSG00000198162	validated
tarbase	hsa-miR-106b-5p	SVIP	258010	ENSG00000198168	validated
tarbase	hsa-miR-15b-5p	SVIP	258010	ENSG00000198168	validated
tarbase	hsa-miR-296-5p	SVIP	258010	ENSG00000198168	validated
tarbase	hsa-miR-181a-5p	ZNF607	84775	ENSG00000198182	validated
tarbase	hsa-miR-222-3p	ZXDA	7789	ENSG00000198205	validated
tarbase	hsa-miR-18a-5p	RPS6KL1	83694	ENSG00000198208	validated
tarbase	hsa-miR-18a-5p	AC092143.1		ENSG00000198211	validated
tarbase	hsa-miR-212-3p	SLC29A3	55315	ENSG00000198246	validated
tarbase	hsa-miR-106b-5p	STYX	6815	ENSG00000198252	validated
tarbase	hsa-miR-15b-5p	STYX	6815	ENSG00000198252	validated
tarbase	hsa-miR-106b-5p	HELZ	9931	ENSG00000198265	validated
tarbase	hsa-miR-181a-5p	HELZ	9931	ENSG00000198265	validated

tarbase	hsa-miR-192-5p	HELZ	9931	ENSG00000198265	validated
tarbase	hsa-miR-15b-5p	UCKL1	54963	ENSG00000198276	validated
tarbase	hsa-miR-212-3p	ZNF485	220992	ENSG00000198298	validated
tarbase	hsa-miR-181a-5p	PEG3	5178	ENSG00000198300	validated
tarbase	hsa-miR-106b-5p	SDAD1	55153	ENSG00000198301	validated
tarbase	hsa-miR-181a-5p	SDAD1	55153	ENSG00000198301	validated
tarbase	hsa-miR-181a-5p	ZKSCAN8	7745	ENSG00000198315	validated
tarbase	hsa-miR-18a-5p	ZNF813	126017	ENSG00000198346	validated
tarbase	hsa-miR-181a-5p	PIM3	415116	ENSG00000198355	validated
tarbase	hsa-miR-21-5p	GET3	439	ENSG00000198356	validated
tarbase	hsa-miR-212-3p	ASPH	444	ENSG00000198363	validated
tarbase	hsa-miR-222-3p	ASPH	444	ENSG00000198363	validated
tarbase	hsa-miR-181a-5p	ASPH	444	ENSG00000198363	validated
tarbase	hsa-miR-106b-5p	ASPH	444	ENSG00000198363	validated
tarbase	hsa-miR-106b-5p	GFPT1	2673	ENSG00000198380	validated
tarbase	hsa-miR-15b-5p	GFPT1	2673	ENSG00000198380	validated
tarbase	hsa-miR-181a-5p	GFPT1	2673	ENSG00000198380	validated
tarbase	hsa-miR-18a-5p	GFPT1	2673	ENSG00000198380	validated
tarbase	hsa-miR-301a-3p	GFPT1	2673	ENSG00000198380	validated
tarbase	hsa-miR-181a-5p	ZNF26	7574	ENSG00000198393	validated
tarbase	hsa-miR-212-3p	TMEM207	131920	ENSG00000198398	validated
tarbase	hsa-miR-106b-5p	OGA	10724	ENSG00000198408	validated
tarbase	hsa-miR-18a-5p	OGA	10724	ENSG00000198408	validated
tarbase	hsa-miR-212-3p	OGA	10724	ENSG00000198408	validated
tarbase	hsa-miR-21-5p	OGA	10724	ENSG00000198408	validated
tarbase	hsa-miR-582-3p	OGA	10724	ENSG00000198408	validated
tarbase	hsa-miR-582-5p	OGA	10724	ENSG00000198408	validated
tarbase	hsa-miR-15b-5p	OGA	10724	ENSG00000198408	validated
tarbase	hsa-miR-301a-3p	NRARP	441478	ENSG00000198435	validated
tarbase	hsa-miR-181a-5p	ZNF568	374900	ENSG00000198453	validated
tarbase	hsa-miR-326	ZNF587	84914	ENSG00000198466	validated
tarbase	hsa-miR-222-3p	TPM2	7169	ENSG00000198467	validated
tarbase	hsa-miR-15b-5p	SH3BGRL2	83699	ENSG00000198478	validated
tarbase	hsa-miR-582-3p	TMA16	55319	ENSG00000198498	validated
tarbase	hsa-miR-106b-5p	TMA16	55319	ENSG00000198498	validated
tarbase	hsa-miR-326	HLA-DRB5	3127	ENSG00000198502	validated
tarbase	hsa-miR-15b-5p	ATL1	51062	ENSG00000198513	validated
tarbase	hsa-miR-18a-5p	MAFK	7975	ENSG00000198517	validated
tarbase	hsa-miR-582-5p	MAFK	7975	ENSG00000198517	validated
tarbase	hsa-miR-106b-5p	ZNF43	7594	ENSG00000198521	validated
tarbase	hsa-miR-15b-5p	ZNF43	7594	ENSG00000198521	validated
tarbase	hsa-miR-18a-5p	ZNF43	7594	ENSG00000198521	validated
tarbase	hsa-miR-106b-5p	C2CD4A	145741	ENSG00000198535	validated
tarbase	hsa-miR-106b-5p	ZNF28	7576	ENSG00000198538	validated
tarbase	hsa-miR-21-5p	ZNF28	7576	ENSG00000198538	validated
tarbase	hsa-miR-181a-5p	ZNF511	118472	ENSG00000198546	validated
tarbase	hsa-miR-15b-5p	ZNF627	199692	ENSG00000198551	validated
tarbase	hsa-miR-181a-5p	ZNF627	199692	ENSG00000198551	validated
tarbase	hsa-miR-15b-5p	WDHD1	11169	ENSG00000198554	validated
tarbase	hsa-miR-212-3p	WDHD1	11169	ENSG00000198554	validated
tarbase	hsa-miR-106b-5p	CTNND1	1500	ENSG00000198561	validated
tarbase	hsa-miR-301a-3p	CTNND1	1500	ENSG00000198561	validated
tarbase	hsa-miR-582-5p	CTNND1	1500	ENSG00000198561	validated
tarbase	hsa-miR-106b-5p	DDX39B	7919	ENSG00000198563	validated
tarbase	hsa-miR-181a-5p	DDX39B	7919	ENSG00000198563	validated
tarbase	hsa-miR-582-3p	DDX39B	7919	ENSG00000198563	validated
tarbase	hsa-miR-18a-5p	ARC	23237	ENSG00000198576	validated
tarbase	hsa-miR-222-3p	ARC	23237	ENSG00000198576	validated
tarbase	hsa-miR-18a-5p	TLK1	9874	ENSG00000198586	validated
tarbase	hsa-miR-222-3p	TLK1	9874	ENSG00000198586	validated
tarbase	hsa-miR-582-5p	TLK1	9874	ENSG00000198586	validated
tarbase	hsa-miR-15b-5p	LRBA	987	ENSG00000198589	validated
tarbase	hsa-miR-212-3p	ZNF536	9745	ENSG00000198597	validated
tarbase	hsa-miR-326	BAZ1A	11177	ENSG00000198604	validated
tarbase	hsa-miR-106b-5p	MDM4	4194	ENSG00000198625	validated
tarbase	hsa-miR-181a-5p	MDM4	4194	ENSG00000198625	validated
tarbase	hsa-miR-301a-3p	MDM4	4194	ENSG00000198625	validated
tarbase	hsa-miR-106b-5p	KLHL9	55958	ENSG00000198642	validated
tarbase	hsa-miR-15b-5p	KLHL9	55958	ENSG00000198642	validated
tarbase	hsa-miR-21-5p	KLHL9	55958	ENSG00000198642	validated
tarbase	hsa-miR-181a-5p	NCOA6	23054	ENSG00000198646	validated

tarbase	hsa-miR-192-5p	NCOA6	23054	ENSG00000198646	validated
tarbase	hsa-miR-222-3p	STK39	27347	ENSG00000198648	validated
tarbase	hsa-miR-181a-5p	C6orf89	221477	ENSG00000198663	validated
tarbase	hsa-miR-222-3p	C6orf89	221477	ENSG00000198663	validated
tarbase	hsa-miR-582-5p	C6orf89	221477	ENSG00000198663	validated
tarbase	hsa-miR-106b-5p	CALM1	801	ENSG00000198668	validated
tarbase	hsa-miR-15b-5p	CALM1	801	ENSG00000198668	validated
tarbase	hsa-miR-181a-5p	CALM1	801	ENSG00000198668	validated
tarbase	hsa-miR-505-3p	CALM1	801	ENSG00000198668	validated
tarbase	hsa-miR-582-3p	CALM1	801	ENSG00000198668	validated
tarbase	hsa-miR-181a-5p	TTC37	9652	ENSG00000198677	validated
tarbase	hsa-miR-106b-5p	SLC9A6	10479	ENSG00000198689	validated
tarbase	hsa-miR-181a-5p	SLC9A6	10479	ENSG00000198689	validated
tarbase	hsa-miR-15b-5p	IPO9	55705	ENSG00000198700	validated
tarbase	hsa-miR-212-3p	IPO9	55705	ENSG00000198700	validated
tarbase	hsa-miR-296-5p	IPO9	55705	ENSG00000198700	validated
tarbase	hsa-miR-106b-5p	CEP290	80184	ENSG00000198707	validated
tarbase	hsa-miR-106b-5p	MT-CO2	4513	ENSG00000198712	validated
tarbase	hsa-miR-192-5p	MT-CO2	4513	ENSG00000198712	validated
tarbase	hsa-miR-15b-5p	GLMP	112770	ENSG00000198715	validated
tarbase	hsa-miR-212-3p	TOGARAM1	23116	ENSG00000198718	validated
tarbase	hsa-miR-301a-3p	TOGARAM1	23116	ENSG00000198718	validated
tarbase	hsa-miR-15b-5p	DLL1	28514	ENSG00000198719	validated
tarbase	hsa-miR-296-5p	DLL1	28514	ENSG00000198719	validated
tarbase	hsa-miR-301a-3p	DLL1	28514	ENSG00000198719	validated
tarbase	hsa-miR-21-5p	UNC13B	10497	ENSG00000198722	validated
tarbase	hsa-miR-582-5p	UNC13B	10497	ENSG00000198722	validated
tarbase	hsa-miR-21-5p	MT-CYB	4519	ENSG00000198727	validated
tarbase	hsa-miR-15b-5p	PPP1R14C	81706	ENSG00000198729	validated
tarbase	hsa-miR-212-3p	ZNF652	22834	ENSG00000198740	validated
tarbase	hsa-miR-106b-5p	SMURF1	57154	ENSG00000198742	validated
tarbase	hsa-miR-212-3p	SMURF1	57154	ENSG00000198742	validated
tarbase	hsa-miR-326	SMURF1	57154	ENSG00000198742	validated
tarbase	hsa-miR-582-3p	SMURF1	57154	ENSG00000198742	validated
tarbase	hsa-miR-15b-5p	SLC5A3	6526	ENSG00000198743	validated
tarbase	hsa-miR-18a-5p	SLC5A3	6526	ENSG00000198743	validated
tarbase	hsa-miR-192-5p	SLC5A3	6526	ENSG00000198743	validated
tarbase	hsa-miR-582-5p	SLC5A3	6526	ENSG00000198743	validated
tarbase	hsa-miR-212-3p	EGFL6	25975	ENSG00000198759	validated
tarbase	hsa-miR-106b-5p	MT-ND2	4536	ENSG00000198763	validated
tarbase	hsa-miR-18a-5p	MT-ND2	4536	ENSG00000198763	validated
tarbase	hsa-miR-21-5p	MT-ND2	4536	ENSG00000198763	validated
tarbase	hsa-miR-222-3p	RCSD1	92241	ENSG00000198771	validated
tarbase	hsa-miR-582-3p	RCSD1	92241	ENSG00000198771	validated
tarbase	hsa-miR-212-3p	RASSF9	9182	ENSG00000198774	validated
tarbase	hsa-miR-106b-5p	FAM169A	26049	ENSG00000198780	validated
tarbase	hsa-miR-181a-5p	FAM169A	26049	ENSG00000198780	validated
tarbase	hsa-miR-15b-5p	MT-ND5	4540	ENSG00000198786	validated
tarbase	hsa-miR-181a-5p	MT-ND5	4540	ENSG00000198786	validated
tarbase	hsa-miR-18a-5p	MT-ND5	4540	ENSG00000198786	validated
tarbase	hsa-miR-21-5p	MT-ND5	4540	ENSG00000198786	validated
tarbase	hsa-miR-222-3p	MT-ND5	4540	ENSG00000198786	validated
tarbase	hsa-miR-301a-3p	CNOT7	29883	ENSG00000198791	validated
tarbase	hsa-miR-301a-3p	TMEM184B	25829	ENSG00000198792	validated
tarbase	hsa-miR-212-3p	ZNF521	25925	ENSG00000198795	validated
tarbase	hsa-miR-181a-5p	LRIG2	9860	ENSG00000198799	validated
tarbase	hsa-miR-106b-5p	MT-CO1	4512	ENSG00000198804	validated
tarbase	hsa-miR-21-5p	MT-CO1	4512	ENSG00000198804	validated
tarbase	hsa-miR-15b-5p	PNP	4860	ENSG00000198805	validated
tarbase	hsa-miR-192-5p	PNP	4860	ENSG00000198805	validated
tarbase	hsa-miR-212-3p	GK	2710	ENSG00000198814	validated
tarbase	hsa-miR-181a-5p	FOXJ3	22887	ENSG00000198815	validated
tarbase	hsa-miR-181a-5p	SFT2D1	113402	ENSG00000198818	validated
tarbase	hsa-miR-15b-5p	CHAMP1	283489	ENSG00000198824	validated
tarbase	hsa-miR-301a-3p	INPP5F	22876	ENSG00000198825	validated
tarbase	hsa-miR-106b-5p	ARHGAP11A	9824	ENSG00000198826	validated
tarbase	hsa-miR-15b-5p	ARHGAP11A	9824	ENSG00000198826	validated
tarbase	hsa-miR-181a-5p	ARHGAP11A	9824	ENSG00000198826	validated
tarbase	hsa-miR-212-3p	ARHGAP11A	9824	ENSG00000198826	validated
tarbase	hsa-miR-15b-5p	SELENOM	140606	ENSG00000198832	validated
tarbase	hsa-miR-106b-5p	UBE2J1	51465	ENSG00000198833	validated

tarbase	hsa-miR-181a-5p	UBE2J1	51465	ENSG00000198833	validated
tarbase	hsa-miR-192-5p	UBE2J1	51465	ENSG00000198833	validated
tarbase	hsa-miR-222-3p	UBE2J1	51465	ENSG00000198833	validated
tarbase	hsa-miR-582-3p	UBE2J1	51465	ENSG00000198833	validated
tarbase	hsa-miR-18a-5p	OPA1	4976	ENSG00000198836	validated
tarbase	hsa-miR-505-3p	OPA1	4976	ENSG00000198836	validated
tarbase	hsa-miR-15b-5p	DENND4B	9909	ENSG00000198837	validated
tarbase	hsa-miR-301a-3p	DENND4B	9909	ENSG00000198837	validated
tarbase	hsa-miR-15b-5p	ZNF277	11179	ENSG00000198839	validated
tarbase	hsa-miR-582-3p	SELENOT	51714	ENSG00000198843	validated
tarbase	hsa-miR-181a-5p	ARHGEF15	22899	ENSG00000198844	validated
tarbase	hsa-miR-296-5p	RUSC2	9853	ENSG00000198853	validated
tarbase	hsa-miR-21-5p	FICD	11153	ENSG00000198855	validated
tarbase	hsa-miR-301a-3p	FICD	11153	ENSG00000198855	validated
tarbase	hsa-miR-21-5p	OSTC	58505	ENSG00000198856	validated
tarbase	hsa-miR-21-5p	LTN1	26046	ENSG00000198862	validated
tarbase	hsa-miR-301a-3p	DCAF12	25853	ENSG00000198876	validated
tarbase	hsa-miR-181a-5p	MT-ND4	4538	ENSG00000198886	validated
tarbase	hsa-miR-18a-5p	MT-ND4	4538	ENSG00000198886	validated
tarbase	hsa-miR-21-5p	MT-ND4	4538	ENSG00000198886	validated
tarbase	hsa-miR-222-3p	MT-ND4	4538	ENSG00000198886	validated
tarbase	hsa-miR-21-5p	SMC5	23137	ENSG00000198887	validated
tarbase	hsa-miR-181a-5p	MT-ND1	4535	ENSG00000198888	validated
tarbase	hsa-miR-212-3p	CAPZA2	830	ENSG00000198898	validated
tarbase	hsa-miR-18a-5p	MT-ATP6	4508	ENSG00000198899	validated
tarbase	hsa-miR-192-5p	MT-ATP6	4508	ENSG00000198899	validated
tarbase	hsa-miR-21-5p	MT-ATP6	4508	ENSG00000198899	validated
tarbase	hsa-miR-15b-5p	TOP1	7150	ENSG00000198900	validated
tarbase	hsa-miR-222-3p	TOP1	7150	ENSG00000198900	validated
tarbase	hsa-miR-582-5p	TOP1	7150	ENSG00000198900	validated
tarbase	hsa-miR-15b-5p	PRC1	9055	ENSG00000198901	validated
tarbase	hsa-miR-21-5p	PRC1	9055	ENSG00000198901	validated
tarbase	hsa-miR-212-3p	PRC1	9055	ENSG00000198901	validated
tarbase	hsa-miR-181a-5p	BHLHB9	80823	ENSG00000198908	validated
tarbase	hsa-miR-582-5p	BHLHB9	80823	ENSG00000198908	validated
tarbase	hsa-miR-212-3p	MAP3K3	4215	ENSG00000198909	validated
tarbase	hsa-miR-181a-5p	L1CAM	3897	ENSG00000198910	validated
tarbase	hsa-miR-181a-5p	SREBF2	6721	ENSG00000198911	validated
tarbase	hsa-miR-21-5p	SREBF2	6721	ENSG00000198911	validated
tarbase	hsa-miR-326	ATG9A	79065	ENSG00000198925	validated
tarbase	hsa-miR-192-5p	TBKBP1	9755	ENSG00000198933	validated
tarbase	hsa-miR-326	TBKBP1	9755	ENSG00000198933	validated
tarbase	hsa-miR-15b-5p	MT-CO3	4514	ENSG00000198938	validated
tarbase	hsa-miR-18a-5p	MT-CO3	4514	ENSG00000198938	validated
tarbase	hsa-miR-21-5p	MT-CO3	4514	ENSG00000198938	validated
tarbase	hsa-miR-212-3p	ZFP2	80108	ENSG00000198939	validated
tarbase	hsa-miR-181a-5p	SOWAHA	134548	ENSG00000198944	validated
tarbase	hsa-miR-212-3p	SOWAHA	134548	ENSG00000198944	validated
tarbase	hsa-miR-192-5p	DMD	1756	ENSG00000198947	validated
tarbase	hsa-miR-106b-5p	MFAP3L	9848	ENSG00000198948	validated
tarbase	hsa-miR-18a-5p	MFAP3L	9848	ENSG00000198948	validated
tarbase	hsa-miR-21-5p	PJA2	9867	ENSG00000198961	validated
tarbase	hsa-miR-181a-5p	RORB	6096	ENSG00000198963	validated
tarbase	hsa-miR-301a-3p	RORB	6096	ENSG00000198963	validated
tarbase	hsa-miR-582-5p	RORB	6096	ENSG00000198963	validated
tarbase	hsa-miR-212-3p	SGMS1	259230	ENSG00000198964	validated
tarbase	hsa-miR-18a-5p	CHML	1122	ENSG00000203668	validated
tarbase	hsa-miR-181a-5p	CHML	1122	ENSG00000203668	validated
tarbase	hsa-miR-212-3p	CAPN8	388743	ENSG00000203697	validated
tarbase	hsa-miR-212-3p	PRR9	574414	ENSG00000203783	validated
tarbase	hsa-miR-212-3p	RIPPLY2	134701	ENSG00000203877	validated
tarbase	hsa-miR-181a-5p	GDI1	2664	ENSG00000203879	validated
tarbase	hsa-miR-18a-5p	PCMTD2	55251	ENSG00000203880	validated
tarbase	hsa-miR-15b-5p	OOEP	441161	ENSG00000203907	validated
tarbase	hsa-miR-21-5p	EFCAB7	84455	ENSG00000203965	validated
tarbase	hsa-miR-212-3p	EFCAB7	84455	ENSG00000203965	validated
tarbase	hsa-miR-15b-5p	SYS1	90196	ENSG00000204070	validated
tarbase	hsa-miR-106b-5p	GIGYF2	26058	ENSG00000204120	validated
tarbase	hsa-miR-21-5p	GIGYF2	26058	ENSG00000204120	validated
tarbase	hsa-miR-505-3p	C2orf72	257407	ENSG00000204128	validated
tarbase	hsa-miR-181a-5p	RUFY2	55680	ENSG00000204130	validated

tarbase	hsa-miR-296-5p	ZDHHC18	84243	ENSG00000204160	validated
tarbase	hsa-miR-222-3p	BMPR2	659	ENSG00000204217	validated
tarbase	hsa-miR-301a-3p	BMPR2	659	ENSG00000204217	validated
tarbase	hsa-miR-222-3p	PFDN6	10471	ENSG00000204220	validated
tarbase	hsa-miR-181a-5p	PFDN6	10471	ENSG00000204220	validated
tarbase	hsa-miR-15b-5p	BRD2	6046	ENSG00000204256	validated
tarbase	hsa-miR-222-3p	BRD2	6046	ENSG00000204256	validated
tarbase	hsa-miR-301a-3p	BRD2	6046	ENSG00000204256	validated
tarbase	hsa-miR-212-3p	COL5A2	1290	ENSG00000204262	validated
tarbase	hsa-miR-212-3p	PSMB8	5696	ENSG00000204264	validated
tarbase	hsa-miR-212-3p	TAP2	6891	ENSG00000204267	validated
tarbase	hsa-miR-144-3p	PBX2	5089	ENSG00000204304	validated
tarbase	hsa-miR-15b-5p	AGPAT1	10554	ENSG00000204310	validated
tarbase	hsa-miR-15b-5p	STK19	8859	ENSG00000204344	validated
tarbase	hsa-miR-21-5p	DXO	1797	ENSG00000204348	validated
tarbase	hsa-miR-582-3p	SKIV2L	6499	ENSG00000204351	validated
tarbase	hsa-miR-106b-5p	ZBTB12	221527	ENSG00000204366	validated
tarbase	hsa-miR-181a-5p	SNHG32		ENSG00000204387	validated
tarbase	hsa-miR-18a-5p	SNHG32		ENSG00000204387	validated
tarbase	hsa-miR-212-3p	SNHG32		ENSG00000204387	validated
tarbase	hsa-miR-144-3p	HSPA1B	3304	ENSG00000204388	validated
tarbase	hsa-miR-192-5p	HSPA1A	3303	ENSG00000204389	validated
tarbase	hsa-miR-21-5p	VARS1	7407	ENSG00000204394	validated
tarbase	hsa-miR-15b-5p	BAG6	7917	ENSG00000204463	validated
tarbase	hsa-miR-21-5p	BAG6	7917	ENSG00000204463	validated
tarbase	hsa-miR-192-5p	PRRC2A	7916	ENSG00000204469	validated
tarbase	hsa-miR-212-3p	PRRC2A	7916	ENSG00000204469	validated
tarbase	hsa-miR-296-5p	PRRC2A	7916	ENSG00000204469	validated
tarbase	hsa-miR-301a-3p	PRRC2A	7916	ENSG00000204469	validated
tarbase	hsa-miR-505-3p	PRRC2A	7916	ENSG00000204469	validated
tarbase	hsa-miR-582-3p	PRRC2A	7916	ENSG00000204469	validated
tarbase	hsa-miR-15b-5p	NFKBIL1	4795	ENSG00000204498	validated
tarbase	hsa-miR-192-5p	NFKBIL1	4795	ENSG00000204498	validated
tarbase	hsa-miR-181a-5p	MICB	4277	ENSG00000204516	validated
tarbase	hsa-miR-106b-5p	MICA	100507436	ENSG00000204520	validated
tarbase	hsa-miR-15b-5p	MICA	100507436	ENSG00000204520	validated
tarbase	hsa-miR-144-3p	ZNF805	390980	ENSG00000204524	validated
tarbase	hsa-miR-296-5p	HLA-C	3107	ENSG00000204525	validated
tarbase	hsa-miR-181a-5p	POU5F1	5460	ENSG00000204531	validated
tarbase	hsa-miR-212-3p	DEFB121	245934	ENSG00000204548	validated
tarbase	hsa-miR-181a-5p	MRPS18B	28973	ENSG00000204568	validated
tarbase	hsa-miR-296-5p	PPP1R10	5514	ENSG00000204569	validated
tarbase	hsa-miR-301a-3p	PPP1R10	5514	ENSG00000204569	validated
tarbase	hsa-miR-15b-5p	ABCF1	23	ENSG00000204574	validated
tarbase	hsa-miR-15b-5p	PRR3	80742	ENSG00000204576	validated
tarbase	hsa-miR-181a-5p	PRR3	80742	ENSG00000204576	validated
tarbase	hsa-miR-15b-5p	DDR1	780	ENSG00000204580	validated
tarbase	hsa-miR-296-5p	DDR1	780	ENSG00000204580	validated
tarbase	hsa-miR-296-5p	HLA-E	3133	ENSG00000204592	validated
tarbase	hsa-miR-222-3p	ZNF616	90317	ENSG00000204611	validated
tarbase	hsa-miR-212-3p	PPP1R11	6992	ENSG00000204619	validated
tarbase	hsa-miR-296-5p	PPP1R11	6992	ENSG00000204619	validated
tarbase	hsa-miR-296-5p	DISP3	57540	ENSG00000204624	validated
tarbase	hsa-miR-15b-5p	RACK1	10399	ENSG00000204628	validated
tarbase	hsa-miR-212-3p	RACK1	10399	ENSG00000204628	validated
tarbase	hsa-miR-21-5p	RACK1	10399	ENSG00000204628	validated
tarbase	hsa-miR-326	RACK1	10399	ENSG00000204628	validated
tarbase	hsa-miR-212-3p	HLA-F	3134	ENSG00000204642	validated
tarbase	hsa-miR-296-5p	GABBR1	2550	ENSG00000204681	validated
tarbase	hsa-miR-18a-5p	TRIM27	5987	ENSG00000204713	validated
tarbase	hsa-miR-212-3p	TTC25	83538	ENSG00000204815	validated
tarbase	hsa-miR-301a-3p	ATXN2	6311	ENSG00000204842	validated
tarbase	hsa-miR-181a-5p	ZBTB48	3104	ENSG00000204859	validated
tarbase	hsa-miR-106b-5p	MZT1	440145	ENSG00000204899	validated
tarbase	hsa-miR-301a-3p	MZT1	440145	ENSG00000204899	validated
tarbase	hsa-miR-326	MZT1	440145	ENSG00000204899	validated
tarbase	hsa-miR-505-3p	MZT1	440145	ENSG00000204899	validated
tarbase	hsa-miR-181a-5p	MZT1	440145	ENSG00000204899	validated
tarbase	hsa-miR-212-3p	LINC01545		ENSG00000204904	validated
tarbase	hsa-miR-212-3p	FAM221B	392307	ENSG00000204930	validated
tarbase	hsa-miR-582-3p	PCDHGA1	56114	ENSG00000204956	validated

tarbase	hsa-miR-15b-5p	PCDHA9	9752	ENSG00000204961	validated
tarbase	hsa-miR-18a-5p	PCDHA9	9752	ENSG00000204961	validated
tarbase	hsa-miR-21-5p	PCDHA9	9752	ENSG00000204961	validated
tarbase	hsa-miR-181a-5p	PCDHA9	9752	ENSG00000204961	validated
tarbase	hsa-miR-326	PRSS1	5644	ENSG00000204983	validated
tarbase	hsa-miR-15b-5p	SPIRE2	84501	ENSG00000204991	validated
tarbase	hsa-miR-144-3p	SLC35B4	84912	ENSG00000205060	validated
tarbase	hsa-miR-18a-5p	SLC35B4	84912	ENSG00000205060	validated
tarbase	hsa-miR-18a-5p	TRIQK	286144	ENSG00000205133	validated
tarbase	hsa-miR-106b-5p	ZBTB10	65986	ENSG00000205189	validated
tarbase	hsa-miR-212-3p	ZBTB10	65986	ENSG00000205189	validated
tarbase	hsa-miR-222-3p	ZBTB10	65986	ENSG00000205189	validated
tarbase	hsa-miR-326	ZBTB10	65986	ENSG00000205189	validated
tarbase	hsa-miR-106b-5p	LGR4	55366	ENSG00000205213	validated
tarbase	hsa-miR-144-3p	LGR4	55366	ENSG00000205213	validated
tarbase	hsa-miR-18a-5p	LGR4	55366	ENSG00000205213	validated
tarbase	hsa-miR-212-3p	LGR4	55366	ENSG00000205213	validated
tarbase	hsa-miR-106b-5p	PDE7A	5150	ENSG00000205268	validated
tarbase	hsa-miR-15b-5p	PDE7A	5150	ENSG00000205268	validated
tarbase	hsa-miR-212-3p	PDE7A	5150	ENSG00000205268	validated
tarbase	hsa-miR-582-5p	PDE7A	5150	ENSG00000205268	validated
tarbase	hsa-miR-18a-5p	TMEM170B	100113407	ENSG00000205269	validated
tarbase	hsa-miR-296-5p	TMEM170B	100113407	ENSG00000205269	validated
tarbase	hsa-miR-212-3p	TMEM170B	100113407	ENSG00000205269	validated
tarbase	hsa-miR-181a-5p	TMEM170B	100113407	ENSG00000205269	validated
tarbase	hsa-miR-301a-3p	SNX2	6643	ENSG00000205302	validated
tarbase	hsa-miR-582-3p	SNX2	6643	ENSG00000205302	validated
tarbase	hsa-miR-15b-5p	IPO7	10527	ENSG00000205339	validated
tarbase	hsa-miR-181a-5p	IPO7	10527	ENSG00000205339	validated
tarbase	hsa-miR-18a-5p	IPO7	10527	ENSG00000205339	validated
tarbase	hsa-miR-212-3p	IPO7	10527	ENSG00000205339	validated
tarbase	hsa-miR-21-5p	IPO7	10527	ENSG00000205339	validated
tarbase	hsa-miR-582-3p	IPO7	10527	ENSG00000205339	validated
tarbase	hsa-miR-181a-5p	SAMD9	54809	ENSG00000205413	validated
tarbase	hsa-miR-212-3p	SAMD9	54809	ENSG00000205413	validated
tarbase	hsa-miR-106b-5p	CNEP1R1	255919	ENSG00000205423	validated
tarbase	hsa-miR-181a-5p	CNEP1R1	255919	ENSG00000205423	validated
tarbase	hsa-miR-212-3p	KRT81	3887	ENSG00000205426	validated
tarbase	hsa-miR-15b-5p	CCDC85C	317762	ENSG00000205476	validated
tarbase	hsa-miR-192-5p	NAP1L4	4676	ENSG00000205531	validated
tarbase	hsa-miR-181a-5p	TMSB4X	7114	ENSG00000205542	validated
tarbase	hsa-miR-192-5p	HMGN1	3150	ENSG00000205581	validated
tarbase	hsa-miR-181a-5p	LCMT1	51451	ENSG00000205629	validated
tarbase	hsa-miR-582-5p	LIN52	91750	ENSG00000205659	validated
tarbase	hsa-miR-181a-5p	ETFRF1	144363	ENSG00000205707	validated
tarbase	hsa-miR-582-3p	ITSN1	6453	ENSG00000205726	validated
tarbase	hsa-miR-15b-5p	ITSN1	6453	ENSG00000205726	validated
tarbase	hsa-miR-21-5p	ITPRIPL2	162073	ENSG00000205730	validated
tarbase	hsa-miR-15b-5p	C5orf51	285636	ENSG00000205765	validated
tarbase	hsa-miR-181a-5p	C5orf51	285636	ENSG00000205765	validated
tarbase	hsa-miR-192-5p	C5orf51	285636	ENSG00000205765	validated
tarbase	hsa-miR-212-3p	CYS1	192668	ENSG00000205795	validated
tarbase	hsa-miR-15b-5p	PLPP6	403313	ENSG00000205808	validated
tarbase	hsa-miR-296-5p	ZNF316	100131017	ENSG00000205903	validated
tarbase	hsa-miR-181a-5p	JPT2	90861	ENSG00000206053	validated
tarbase	hsa-miR-21-5p	JPT2	90861	ENSG00000206053	validated
tarbase	hsa-miR-582-3p	JPT2	90861	ENSG00000206053	validated
tarbase	hsa-miR-212-3p	RAB12	201475	ENSG00000206418	validated
tarbase	hsa-miR-301a-3p	RAB12	201475	ENSG00000206418	validated
tarbase	hsa-miR-21-5p	HACD2	201562	ENSG00000206527	validated
tarbase	hsa-miR-212-3p	CFAP44	55779	ENSG00000206530	validated
tarbase	hsa-miR-106b-5p	VGLL3	389136	ENSG00000206538	validated
tarbase	hsa-miR-505-3p	VGLL3	389136	ENSG00000206538	validated
tarbase	hsa-miR-15b-5p	TRIM71	131405	ENSG00000206557	validated
tarbase	hsa-miR-181a-5p	TRIM71	131405	ENSG00000206557	validated
tarbase	hsa-miR-21-5p	TRIM71	131405	ENSG00000206557	validated
tarbase	hsa-miR-222-3p	TRIM71	131405	ENSG00000206557	validated
tarbase	hsa-miR-212-3p	TRIM71	131405	ENSG00000206557	validated
tarbase	hsa-miR-15b-5p	ANKRD28	23243	ENSG00000206560	validated
tarbase	hsa-miR-296-5p	COLQ	8292	ENSG00000206561	validated
tarbase	hsa-miR-21-5p	STK38L	23012	ENSG00000211455	validated

tarbase	hsa-miR-15b-5p	SACM1L	22908	ENSG00000211456	validated
tarbase	hsa-miR-582-5p	SACM1L	22908	ENSG00000211456	validated
tarbase	hsa-miR-181a-5p	SACM1L	22908	ENSG00000211456	validated
tarbase	hsa-miR-106b-5p	TSN	7247	ENSG00000211460	validated
tarbase	hsa-miR-15b-5p	TSN	7247	ENSG00000211460	validated
tarbase	hsa-miR-18a-5p	TSN	7247	ENSG00000211460	validated
tarbase	hsa-miR-21-5p	TSN	7247	ENSG00000211460	validated
tarbase	hsa-miR-582-3p	TSN	7247	ENSG00000211460	validated
tarbase	hsa-miR-106b-5p	SLC48A1	55652	ENSG00000211584	validated
tarbase	hsa-miR-181a-5p	LINC02693		ENSG00000212719	validated
tarbase	hsa-miR-18a-5p	LINC02693		ENSG00000212719	validated
tarbase	hsa-miR-301a-3p	LINC02693		ENSG00000212719	validated
tarbase	hsa-miR-106b-5p	MT-ND4L	4539	ENSG00000212907	validated
tarbase	hsa-miR-15b-5p	ZNF580	51157	ENSG00000213015	validated
tarbase	hsa-miR-326	ZNF580	51157	ENSG00000213015	validated
tarbase	hsa-miR-106b-5p	NUP62	23636	ENSG00000213024	validated
tarbase	hsa-miR-212-3p	DENND1B	163486	ENSG00000213047	validated
tarbase	hsa-miR-21-5p	DENND1B	163486	ENSG00000213047	validated
tarbase	hsa-miR-106b-5p	SFT2D2	375035	ENSG00000213064	validated
tarbase	hsa-miR-181a-5p	SFT2D2	375035	ENSG00000213064	validated
tarbase	hsa-miR-21-5p	SFT2D2	375035	ENSG00000213064	validated
tarbase	hsa-miR-222-3p	SFT2D2	375035	ENSG00000213064	validated
tarbase	hsa-miR-301a-3p	SFT2D2	375035	ENSG00000213064	validated
tarbase	hsa-miR-582-3p	SFT2D2	375035	ENSG00000213064	validated
tarbase	hsa-miR-15b-5p	SCAF8	22828	ENSG00000213079	validated
tarbase	hsa-miR-222-3p	SCAF8	22828	ENSG00000213079	validated
tarbase	hsa-miR-212-3p	ACKR1	2532	ENSG00000213088	validated
tarbase	hsa-miR-212-3p	TCTEX1D2	255758	ENSG00000213123	validated
tarbase	hsa-miR-18a-5p			ENSG00000213160	validated
tarbase	hsa-miR-106b-5p	TRIM59	286827	ENSG00000213186	validated
tarbase	hsa-miR-18a-5p	SUPT4H1	6827	ENSG00000213246	validated
tarbase	hsa-miR-192-5p	SUPT4H1	6827	ENSG00000213246	validated
tarbase	hsa-miR-106b-5p	NRAS	4893	ENSG00000213281	validated
tarbase	hsa-miR-21-5p	NRAS	4893	ENSG00000213281	validated
tarbase	hsa-miR-15b-5p	CHUK	1147	ENSG00000213341	validated
tarbase	hsa-miR-181a-5p	CHUK	1147	ENSG00000213341	validated
tarbase	hsa-miR-301a-3p	CHUK	1147	ENSG00000213341	validated
tarbase	hsa-miR-21-5p	CHUK	1147	ENSG00000213341	validated
tarbase	hsa-miR-582-5p	COG8	84342	ENSG00000213380	validated
tarbase	hsa-miR-301a-3p	LCAT	3931	ENSG00000213398	validated
tarbase	hsa-miR-582-5p	MAGEA12	4111	ENSG00000213401	validated
tarbase	hsa-miR-106b-5p	MAGEA12	4111	ENSG00000213401	validated
tarbase	hsa-miR-15b-5p	ARL2	402	ENSG00000213465	validated
tarbase	hsa-miR-582-3p	DNAJC9	23234	ENSG00000213551	validated
tarbase	hsa-miR-21-5p	VDAC1	7416	ENSG00000213585	validated
tarbase	hsa-miR-582-5p	VDAC1	7416	ENSG00000213585	validated
tarbase	hsa-miR-18a-5p	ZBTB9	221504	ENSG00000213588	validated
tarbase	hsa-miR-222-3p	LEPROT	54741	ENSG00000213625	validated
tarbase	hsa-miR-181a-5p	LEPROT	54741	ENSG00000213625	validated
tarbase	hsa-miR-181a-5p	PPP1CB	5500	ENSG00000213639	validated
tarbase	hsa-miR-582-3p	PPP1CB	5500	ENSG00000213639	validated
tarbase	hsa-miR-326	NCKIPSD	51517	ENSG00000213672	validated
tarbase	hsa-miR-222-3p	SLC35F6	54978	ENSG00000213699	validated
tarbase	hsa-miR-212-3p	CLIC1	1192	ENSG00000213719	validated
tarbase	hsa-miR-15b-5p	DDAH2	23564	ENSG00000213722	validated
tarbase	hsa-miR-296-5p	DDAH2	23564	ENSG00000213722	validated
tarbase	hsa-miR-192-5p	RPS29	6235	ENSG00000213741	validated
tarbase	hsa-miR-301a-3p	RPS29	6235	ENSG00000213741	validated
tarbase	hsa-miR-106b-5p	ZNF134	7693	ENSG00000213762	validated
tarbase	hsa-miR-181a-5p	ZNF134	7693	ENSG00000213762	validated
tarbase	hsa-miR-18a-5p	ZNF134	7693	ENSG00000213762	validated
tarbase	hsa-miR-301a-3p	ZNF134	7693	ENSG00000213762	validated
tarbase	hsa-miR-18a-5p	EMP2	2013	ENSG00000213853	validated
tarbase	hsa-miR-301a-3p	EMP2	2013	ENSG00000213853	validated
tarbase	hsa-miR-15b-5p	LTB4R2	56413	ENSG00000213906	validated
tarbase	hsa-miR-18a-5p	LTB4R2	56413	ENSG00000213906	validated
tarbase	hsa-miR-326	CSNK1E	1454	ENSG00000213923	validated
tarbase	hsa-miR-212-3p	IRF9	10379	ENSG00000213928	validated
tarbase	hsa-miR-106b-5p	NUDT19	390916	ENSG00000213965	validated
tarbase	hsa-miR-181a-5p	NUDT19	390916	ENSG00000213965	validated
tarbase	hsa-miR-21-5p	ZNF726	730087	ENSG00000213967	validated

tarbase	hsa-miR-505-3p	NAXD	55739	ENSG00000213995	validated
tarbase	hsa-miR-15b-5p	REPIN1	29803	ENSG00000214022	validated
tarbase	hsa-miR-582-5p	ZNF891	101060200	ENSG00000214029	validated
tarbase	hsa-miR-212-3p	ZNF891	101060200	ENSG00000214029	validated
tarbase	hsa-miR-106b-5p	CPNE1	8904	ENSG00000214078	validated
tarbase	hsa-miR-21-5p	CPNE1	8904	ENSG00000214078	validated
tarbase	hsa-miR-212-3p	MYCBP	26292	ENSG00000214114	validated
tarbase	hsa-miR-181a-5p	ZNF788P		ENSG00000214189	validated
tarbase	hsa-miR-21-5p	FIS1	51024	ENSG00000214253	validated
tarbase	hsa-miR-21-5p	HAUS3	79441	ENSG00000214367	validated
tarbase	hsa-miR-106b-5p	BBIP1	92482	ENSG00000214413	validated
tarbase	hsa-miR-15b-5p	PPME1	51400	ENSG00000214517	validated
tarbase	hsa-miR-296-5p	ZSWIM8	23053	ENSG00000214655	validated
tarbase	hsa-miR-18a-5p	ZSWIM8	23053	ENSG00000214655	validated
tarbase	hsa-miR-21-5p	ZSWIM8	23053	ENSG00000214655	validated
tarbase	hsa-miR-106b-5p	IFRD2	7866	ENSG00000214706	validated
tarbase	hsa-miR-192-5p	TOMM6	100188893	ENSG00000214736	validated
tarbase	hsa-miR-21-5p	TOMM6	100188893	ENSG00000214736	validated
tarbase	hsa-miR-15b-5p	HNRNPUL2	221092	ENSG00000214753	validated
tarbase	hsa-miR-181a-5p	ARHGEF28	64283	ENSG00000214944	validated
tarbase	hsa-miR-21-5p	BRD2	6046	ENSG00000215077	validated
tarbase	hsa-miR-222-3p	UBXN2B	137886	ENSG00000215114	validated
tarbase	hsa-miR-15b-5p	UBE2QL1	134111	ENSG00000215218	validated
tarbase	hsa-miR-181a-5p	UBE2QL1	134111	ENSG00000215218	validated
tarbase	hsa-miR-181a-5p	FASTKD5	60493	ENSG00000215251	validated
tarbase	hsa-miR-144-3p	DDX3X	1654	ENSG00000215301	validated
tarbase	hsa-miR-222-3p	DDX3X	1654	ENSG00000215301	validated
tarbase	hsa-miR-582-3p	DDX3X	1654	ENSG00000215301	validated
tarbase	hsa-miR-582-5p	DDX3X	1654	ENSG00000215301	validated
tarbase	hsa-miR-18a-5p	DDX3X	1654	ENSG00000215301	validated
tarbase	hsa-miR-222-3p	VPS16	64601	ENSG00000215305	validated
tarbase	hsa-miR-212-3p	MYL5	4636	ENSG00000215375	validated
tarbase	hsa-miR-301a-3p	NPEPL1	79716	ENSG00000215440	validated
tarbase	hsa-miR-15b-5p	TMEM167B	56900	ENSG00000215717	validated
tarbase	hsa-miR-21-5p	TMEM167B	56900	ENSG00000215717	validated
tarbase	hsa-miR-15b-5p	IFI30	10437	ENSG00000216490	validated
tarbase	hsa-miR-301a-3p	IFI30	10437	ENSG00000216490	validated
tarbase	hsa-miR-192-5p	CCDC7	79741	ENSG00000216937	validated
tarbase	hsa-miR-15b-5p	FNIP1	96459	ENSG00000217128	validated
tarbase	hsa-miR-212-3p	FNIP1	96459	ENSG00000217128	validated
tarbase	hsa-miR-192-5p	SP9	100131390	ENSG00000217236	validated
tarbase	hsa-miR-301a-3p	TENM3	55714	ENSG00000218336	validated
tarbase	hsa-miR-212-3p	AC110619.1		ENSG00000218416	validated
tarbase	hsa-miR-15b-5p	NBPF1	55672	ENSG00000219481	validated
tarbase	hsa-miR-21-5p	NBPF1	55672	ENSG00000219481	validated
tarbase	hsa-miR-106b-5p	FAM228B	375190	ENSG00000219626	validated
tarbase	hsa-miR-212-3p	FAM228B	375190	ENSG00000219626	validated
tarbase	hsa-miR-21-5p	ZGLP1	100125288	ENSG00000220201	validated
tarbase	hsa-miR-326	VAMP2	6844	ENSG00000220205	validated
tarbase	hsa-miR-18a-5p	VAMP2	6844	ENSG00000220205	validated
tarbase	hsa-miR-15b-5p	PPP3R1	5534	ENSG00000221823	validated
tarbase	hsa-miR-181a-5p	PPP3R1	5534	ENSG00000221823	validated
tarbase	hsa-miR-301a-3p	PPP3R1	5534	ENSG00000221823	validated
tarbase	hsa-miR-106b-5p	FANCG	2189	ENSG00000221829	validated
tarbase	hsa-miR-222-3p	CEBPD	1052	ENSG00000221869	validated
tarbase	hsa-miR-212-3p	OR1C1	26188	ENSG00000221888	validated
tarbase	hsa-miR-106b-5p	PPP2R2A	5520	ENSG00000221914	validated
tarbase	hsa-miR-15b-5p	PPP2R2A	5520	ENSG00000221914	validated
tarbase	hsa-miR-106b-5p	ZNF880	400713	ENSG00000221923	validated
tarbase	hsa-miR-15b-5p	ZNF880	400713	ENSG00000221923	validated
tarbase	hsa-miR-212-3p	FXYD7	53822	ENSG00000221946	validated
tarbase	hsa-miR-181a-5p	CCNL2	81669	ENSG00000221978	validated
tarbase	hsa-miR-18a-5p	CCNL2	81669	ENSG00000221978	validated
tarbase	hsa-miR-192-5p	UBA52	7311	ENSG00000221983	validated
tarbase	hsa-miR-15b-5p	PPT2	9374	ENSG00000221988	validated
tarbase	hsa-miR-326	EXOC3-AS1		ENSG00000221990	validated
tarbase	hsa-miR-212-3p	EXOC3-AS1		ENSG00000221990	validated
tarbase	hsa-miR-106b-5p	EXOSC6	118460	ENSG00000223496	validated
tarbase	hsa-miR-18a-5p	EXOSC6	118460	ENSG00000223496	validated
tarbase	hsa-miR-296-5p	EXOSC6	118460	ENSG00000223496	validated
tarbase	hsa-miR-301a-3p	EXOSC6	118460	ENSG00000223496	validated

tarbase	hsa-miR-212-3p	ADM5	199800	ENSG00000224420	validated
tarbase	hsa-miR-15b-5p	ATXN1L	342371	ENSG00000224470	validated
tarbase	hsa-miR-192-5p	ATXN1L	342371	ENSG00000224470	validated
tarbase	hsa-miR-301a-3p	ATXN1L	342371	ENSG00000224470	validated
tarbase	hsa-miR-106b-5p	ATXN1L	342371	ENSG00000224470	validated
tarbase	hsa-miR-15b-5p	SMIM13	221710	ENSG00000224531	validated
tarbase	hsa-miR-181a-5p	SMIM13	221710	ENSG00000224531	validated
tarbase	hsa-miR-212-3p	SMIM13	221710	ENSG00000224531	validated
tarbase	hsa-miR-21-5p	SMIM13	221710	ENSG00000224531	validated
tarbase	hsa-miR-18a-5p	SMIM13	221710	ENSG00000224531	validated
tarbase	hsa-miR-181a-5p	ZNF812P		ENSG00000224689	validated
tarbase	hsa-miR-181a-5p	MRPL20-AS1		ENSG00000224870	validated
tarbase	hsa-miR-326	SLC26A6	65010	ENSG00000225697	validated
tarbase	hsa-miR-582-3p			ENSG00000225830	validated
tarbase	hsa-miR-106b-5p	ZNF717	100131827	ENSG00000227124	validated
tarbase	hsa-miR-181a-5p	ZNF717	100131827	ENSG00000227124	validated
tarbase	hsa-miR-301a-3p	KLLN	100144748	ENSG00000227268	validated
tarbase	hsa-miR-18a-5p	PARG	8505	ENSG00000227345	validated
tarbase	hsa-miR-301a-3p	SCAMP4	113178	ENSG00000227500	validated
tarbase	hsa-miR-212-3p	TEX46	729059	ENSG00000227868	validated
tarbase	hsa-miR-222-3p	RPL41	6171	ENSG00000229117	validated
tarbase	hsa-miR-296-5p	NAMPTP1		ENSG00000229644	validated
tarbase	hsa-miR-326	PET100	100131801	ENSG00000229833	validated
tarbase	hsa-miR-212-3p	PPP5D1	100506012	ENSG00000230510	validated
tarbase	hsa-miR-106b-5p	PRB4	5545	ENSG00000230657	validated
tarbase	hsa-miR-21-5p	HSBP1	3281	ENSG00000230989	validated
tarbase	hsa-miR-18a-5p	TAPBP	6892	ENSG00000231925	validated
tarbase	hsa-miR-212-3p	TAPBP	6892	ENSG00000231925	validated
tarbase	hsa-miR-212-3p	ZBED9	114821	ENSG00000232040	validated
tarbase	hsa-miR-212-3p	ZNF687-AS1		ENSG00000232671	validated
tarbase	hsa-miR-212-3p	ZNF879	345462	ENSG00000234284	validated
tarbase	hsa-miR-181a-5p	ZNF736	728927	ENSG00000234444	validated
tarbase	hsa-miR-582-5p	ZNF736	728927	ENSG00000234444	validated
tarbase	hsa-miR-106b-5p	ZNF736	728927	ENSG00000234444	validated
tarbase	hsa-miR-181a-5p	JRK	8629	ENSG00000234616	validated
tarbase	hsa-miR-212-3p	HLA-B	3106	ENSG00000234745	validated
tarbase	hsa-miR-212-3p	RNF148	378925	ENSG00000235631	validated
tarbase	hsa-miR-192-5p	ZBTB22	9278	ENSG00000236104	validated
tarbase	hsa-miR-15b-5p	ZBED5	58486	ENSG00000236287	validated
tarbase	hsa-miR-181a-5p	ZBED5	58486	ENSG00000236287	validated
tarbase	hsa-miR-301a-3p	ZNF853	54753	ENSG00000236609	validated
tarbase	hsa-miR-212-3p	IFNA4	3441	ENSG00000236637	validated
tarbase	hsa-miR-15b-5p	TMEM250	90120	ENSG00000238227	validated
tarbase	hsa-miR-18a-5p	TMEM250	90120	ENSG00000238227	validated
tarbase	hsa-miR-296-5p	TMEM250	90120	ENSG00000238227	validated
tarbase	hsa-miR-301a-3p	TMEM250	90120	ENSG00000238227	validated
tarbase	hsa-miR-106b-5p	RNF103	7844	ENSG00000239305	validated
tarbase	hsa-miR-181a-5p	RNF103	7844	ENSG00000239305	validated
tarbase	hsa-miR-18a-5p	RNF103	7844	ENSG00000239305	validated
tarbase	hsa-miR-192-5p	RNF103	7844	ENSG00000239305	validated
tarbase	hsa-miR-15b-5p	RBM14	10432	ENSG00000239306	validated
tarbase	hsa-miR-18a-5p	RBM14	10432	ENSG00000239306	validated
tarbase	hsa-miR-296-5p	RBM14	10432	ENSG00000239306	validated
tarbase	hsa-miR-505-3p	RBM14	10432	ENSG00000239306	validated
tarbase	hsa-miR-301a-3p	WBP1	23559	ENSG00000239779	validated
tarbase	hsa-miR-15b-5p	GET4	51608	ENSG00000239857	validated
tarbase	hsa-miR-212-3p	C1orf226	400793	ENSG00000239887	validated
tarbase	hsa-miR-212-3p	LILRA4	23547	ENSG00000239961	validated
tarbase	hsa-miR-15b-5p	LY6G5B	58496	ENSG00000240053	validated
tarbase	hsa-miR-181a-5p	LCE1F	353137	ENSG00000240386	validated
tarbase	hsa-miR-181a-5p	PNMA2	10687	ENSG00000240694	validated
tarbase	hsa-miR-15b-5p	PCDHGC5	56097	ENSG00000240764	validated
tarbase	hsa-miR-181a-5p	MIF	4282	ENSG00000240972	validated
tarbase	hsa-miR-21-5p	YAE1	57002	ENSG00000241127	validated
tarbase	hsa-miR-505-3p	RPL36A	6173	ENSG00000241343	validated
tarbase	hsa-miR-106b-5p	SSX2	6757	ENSG00000241476	validated
tarbase	hsa-miR-192-5p	ARPC4	10093	ENSG00000241553	validated
tarbase	hsa-miR-296-5p	ARPC4	10093	ENSG00000241553	validated
tarbase	hsa-miR-301a-3p	ARPC4	10093	ENSG00000241553	validated
tarbase	hsa-miR-106b-5p	ARPC4	10093	ENSG00000241553	validated
tarbase	hsa-miR-301a-3p	ARPC1A	10552	ENSG00000241685	validated

tarbase	hsa-miR-21-5p	ATP5PO	539	ENSG00000241837	validated
tarbase	hsa-miR-192-5p	PISD	23761	ENSG00000241878	validated
tarbase	hsa-miR-326	PISD	23761	ENSG00000241878	validated
tarbase	hsa-miR-212-3p	HOGA1	112817	ENSG00000241935	validated
tarbase	hsa-miR-15b-5p	PI4KA	5297	ENSG00000241973	validated
tarbase	hsa-miR-21-5p	PI4KA	5297	ENSG00000241973	validated
tarbase	hsa-miR-15b-5p	ARFGAP3	26286	ENSG00000242247	validated
tarbase	hsa-miR-326	ARFGAP3	26286	ENSG00000242247	validated
tarbase	hsa-miR-144-3p	PEG10	23089	ENSG00000242265	validated
tarbase	hsa-miR-181a-5p	PEG10	23089	ENSG00000242265	validated
tarbase	hsa-miR-18a-5p	PEG10	23089	ENSG00000242265	validated
tarbase	hsa-miR-212-3p	PEG10	23089	ENSG00000242265	validated
tarbase	hsa-miR-582-3p	PEG10	23089	ENSG00000242265	validated
tarbase	hsa-miR-15b-5p	PEG10	23089	ENSG00000242265	validated
tarbase	hsa-miR-18a-5p	EIF6	3692	ENSG00000242372	validated
tarbase	hsa-miR-296-5p	PCDHGC4	56098	ENSG00000242419	validated
tarbase	hsa-miR-15b-5p	MRPL20	55052	ENSG00000242485	validated
tarbase	hsa-miR-15b-5p	GNG10	2790	ENSG00000242616	validated
tarbase	hsa-miR-212-3p	PSMB9	5698	ENSG00000242711	validated
tarbase	hsa-miR-18a-5p	PSG4	5672	ENSG00000243137	validated
tarbase	hsa-miR-181a-5p	MICAL3	57553	ENSG00000243156	validated
tarbase	hsa-miR-296-5p	PPAN-P2RY11	692312	ENSG00000243207	validated
tarbase	hsa-miR-106b-5p	STON1	11037	ENSG00000243244	validated
tarbase	hsa-miR-212-3p	STON1	11037	ENSG00000243244	validated
tarbase	hsa-miR-181a-5p	STMP1	647087	ENSG00000243317	validated
tarbase	hsa-miR-582-5p	KCTD7	154881	ENSG00000243335	validated
tarbase	hsa-miR-15b-5p	C4orf48	401115	ENSG00000243449	validated
tarbase	hsa-miR-181a-5p	TNFRSF6B	8771	ENSG00000243509	validated
tarbase	hsa-miR-18a-5p	UPK3B	105375355	ENSG00000243566	validated
tarbase	hsa-miR-15b-5p	DDOST	1650	ENSG00000244038	validated
tarbase	hsa-miR-15b-5p	TMEM199	147007	ENSG00000244045	validated
tarbase	hsa-miR-181a-5p	TMEM199	147007	ENSG00000244045	validated
tarbase	hsa-miR-15b-5p	DBNDD2	55861	ENSG00000244274	validated
tarbase	hsa-miR-106b-5p	RBM12	10137	ENSG00000244462	validated
tarbase	hsa-miR-144-3p	RBM12	10137	ENSG00000244462	validated
tarbase	hsa-miR-15b-5p	RBM12	10137	ENSG00000244462	validated
tarbase	hsa-miR-192-5p	RBM12	10137	ENSG00000244462	validated
tarbase	hsa-miR-301a-3p	RBM12	10137	ENSG00000244462	validated
tarbase	hsa-miR-21-5p	RBM12	10137	ENSG00000244462	validated
tarbase	hsa-miR-15b-5p	SCARF2	91179	ENSG00000244486	validated
tarbase	hsa-miR-181a-5p	N4BP2L2	10443	ENSG00000244754	validated
tarbase	hsa-miR-212-3p	AC008393.1		ENSG00000245317	validated
tarbase	hsa-miR-181a-5p	H2AJ	55766	ENSG00000246705	validated
tarbase	hsa-miR-106b-5p	PGAM5	192111	ENSG00000247077	validated
tarbase	hsa-miR-296-5p	PGAM5	192111	ENSG00000247077	validated
tarbase	hsa-miR-181a-5p	ZCCHC3	85364	ENSG00000247315	validated
tarbase	hsa-miR-18a-5p	ZCCHC3	85364	ENSG00000247315	validated
tarbase	hsa-miR-212-3p	ZCCHC3	85364	ENSG00000247315	validated
tarbase	hsa-miR-301a-3p	MARS2	92935	ENSG00000247626	validated
tarbase	hsa-miR-106b-5p	ADH1C	126	ENSG00000248144	validated
tarbase	hsa-miR-212-3p	C4orf54	285556	ENSG00000248713	validated
tarbase	hsa-miR-21-5p	AC004997.1		ENSG00000248751	validated
tarbase	hsa-miR-181a-5p	ZNF564	163050	ENSG00000249709	validated
tarbase	hsa-miR-18a-5p	ZNF564	163050	ENSG00000249709	validated
tarbase	hsa-miR-181a-5p	PDCD6	10016	ENSG00000249915	validated
tarbase	hsa-miR-21-5p	PDCD6	10016	ENSG00000249915	validated
tarbase	hsa-miR-582-5p	PDCD6	10016	ENSG00000249915	validated
tarbase	hsa-miR-301a-3p	AC093791.1		ENSG00000250038	validated
tarbase	hsa-miR-582-5p	ZNF718	255403	ENSG00000250312	validated
tarbase	hsa-miR-296-5p	CHCHD10	400916	ENSG00000250479	validated
tarbase	hsa-miR-192-5p	GLI4	2738	ENSG00000250571	validated
tarbase	hsa-miR-106b-5p	TMED7-TICAM2	100302736	ENSG00000251201	validated
tarbase	hsa-miR-222-3p	TMED7-TICAM2	100302736	ENSG00000251201	validated
tarbase	hsa-miR-301a-3p	TMED7-TICAM2	100302736	ENSG00000251201	validated
tarbase	hsa-miR-181a-5p	AP000350.4		ENSG00000251357	validated
tarbase	hsa-miR-106b-5p	FOXD1	2297	ENSG00000251493	validated
tarbase	hsa-miR-106b-5p	PRB1	5542	ENSG00000251655	validated
tarbase	hsa-miR-582-5p	C8orf88	100127983	ENSG00000253250	validated
tarbase	hsa-miR-106b-5p	SHLD3	112441434	ENSG00000253251	validated
tarbase	hsa-miR-181a-5p	HOXA10	3206	ENSG00000253293	validated
tarbase	hsa-miR-21-5p	ALG11	440138	ENSG00000253710	validated

tarbase	hsa-miR-212-3p	ATXN7L3B	552889	ENSG00000253719	validated
tarbase	hsa-miR-21-5p	ATXN7L3B	552889	ENSG00000253719	validated
tarbase	hsa-miR-106b-5p	PRKDC	5591	ENSG00000253729	validated
tarbase	hsa-miR-15b-5p	PRKDC	5591	ENSG00000253729	validated
tarbase	hsa-miR-181a-5p	PRKDC	5591	ENSG00000253729	validated
tarbase	hsa-miR-18a-5p	PRKDC	5591	ENSG00000253729	validated
tarbase	hsa-miR-212-3p	PRKDC	5591	ENSG00000253729	validated
tarbase	hsa-miR-21-5p	PRKDC	5591	ENSG00000253729	validated
tarbase	hsa-miR-106b-5p	UTP14C	9724	ENSG00000253797	validated
tarbase	hsa-miR-15b-5p	UTP14C	9724	ENSG00000253797	validated
tarbase	hsa-miR-18a-5p	PCDHGB2	56103	ENSG00000253910	validated
tarbase	hsa-miR-181a-5p	ZNF260	339324	ENSG00000254004	validated
tarbase	hsa-miR-106b-5p	LYN	4067	ENSG00000254087	validated
tarbase	hsa-miR-18a-5p	LYN	4067	ENSG00000254087	validated
tarbase	hsa-miR-106b-5p	PABPC4L	132430	ENSG00000254535	validated
tarbase	hsa-miR-326	PABPC4L	132430	ENSG00000254535	validated
tarbase	hsa-miR-212-3p	FPGT	8790	ENSG00000254685	validated
tarbase	hsa-miR-15b-5p	AL136295.1		ENSG00000254692	validated
tarbase	hsa-miR-212-3p	MEX3A	92312	ENSG00000254726	validated
tarbase	hsa-miR-222-3p	MEX3A	92312	ENSG00000254726	validated
tarbase	hsa-miR-181a-5p	MEX3A	92312	ENSG00000254726	validated
tarbase	hsa-miR-106b-5p	EEF1G	1937	ENSG00000254772	validated
tarbase	hsa-miR-301a-3p	EEF1G	1937	ENSG00000254772	validated
tarbase	hsa-miR-15b-5p	CKLF-CMTM1	100529251	ENSG00000254788	validated
tarbase	hsa-miR-15b-5p	ANKHD1-EIF4EBP3	404734	ENSG00000254996	validated
tarbase	hsa-miR-192-5p	ANKHD1-EIF4EBP3	404734	ENSG00000254996	validated
tarbase	hsa-miR-212-3p	ANKHD1-EIF4EBP3	404734	ENSG00000254996	validated
tarbase	hsa-miR-301a-3p	ANKHD1-EIF4EBP3	404734	ENSG00000254996	validated
tarbase	hsa-miR-212-3p	KRTAP5-9	3846	ENSG00000254997	validated
tarbase	hsa-miR-222-3p	BRK1	55845	ENSG00000254999	validated
tarbase	hsa-miR-18a-5p	AP001318.2		ENSG00000255062	validated
tarbase	hsa-miR-106b-5p	ZFP91-CNTF		ENSG00000255073	validated
tarbase	hsa-miR-301a-3p	ZFP91-CNTF		ENSG00000255073	validated
tarbase	hsa-miR-15b-5p	EID1	23741	ENSG00000255302	validated
tarbase	hsa-miR-212-3p	AL096711.2		ENSG00000255330	validated
tarbase	hsa-miR-181a-5p	AL096711.2		ENSG00000255330	validated
tarbase	hsa-miR-181a-5p	POLR2M	81488	ENSG00000255529	validated
tarbase	hsa-miR-582-3p	MTRNR2L9		ENSG00000255633	validated
tarbase	hsa-miR-212-3p	TRIL	9865	ENSG00000255690	validated
tarbase	hsa-miR-582-3p	MTRNR2L8	100463486	ENSG00000255823	validated
tarbase	hsa-miR-106b-5p	COA8	84334	ENSG00000256053	validated
tarbase	hsa-miR-181a-5p	ZNF432	9668	ENSG00000256087	validated
tarbase	hsa-miR-15b-5p	AP000721.1		ENSG00000256100	validated
tarbase	hsa-miR-181a-5p	ZNF10	7556	ENSG00000256223	validated
tarbase	hsa-miR-21-5p	ZNF10	7556	ENSG00000256223	validated
tarbase	hsa-miR-181a-5p	HMBS	3145	ENSG00000256269	validated
tarbase	hsa-miR-212-3p	AC021092.2	7768	ENSG00000256294	validated
tarbase	hsa-miR-21-5p	SALL3	27164	ENSG00000256463	validated
tarbase	hsa-miR-582-3p	MTRNR2L1	100462977	ENSG00000256618	validated
tarbase	hsa-miR-18a-5p	ZNF253	56242	ENSG00000256771	validated
tarbase	hsa-miR-582-5p	LSM14A	26065	ENSG00000257103	validated
tarbase	hsa-miR-15b-5p	GATC	283459	ENSG00000257218	validated
tarbase	hsa-miR-181a-5p	GATC	283459	ENSG00000257218	validated
tarbase	hsa-miR-301a-3p	GATC	283459	ENSG00000257218	validated
tarbase	hsa-miR-212-3p	LINC01619		ENSG00000257242	validated
tarbase	hsa-miR-181a-5p	ZBED6	100381270	ENSG00000257315	validated
tarbase	hsa-miR-212-3p	ZBED6	100381270	ENSG00000257315	validated
tarbase	hsa-miR-212-3p	MGAM	8972	ENSG00000257335	validated
tarbase	hsa-miR-181a-5p	ZNF625	90589	ENSG00000257591	validated
tarbase	hsa-miR-15b-5p	CUX1	1523	ENSG00000257923	validated
tarbase	hsa-miR-21-5p	CUX1	1523	ENSG00000257923	validated
tarbase	hsa-miR-301a-3p	CUX1	1523	ENSG00000257923	validated
tarbase	hsa-miR-582-3p	CUX1	1523	ENSG00000257923	validated
tarbase	hsa-miR-181a-5p	CHURC1	91612	ENSG00000258289	validated
tarbase	hsa-miR-21-5p	AC009779.4		ENSG00000258311	validated
tarbase	hsa-miR-212-3p	C17orf49	124944	ENSG00000258315	validated
tarbase	hsa-miR-181a-5p	RTEL1	51750	ENSG00000258366	validated
tarbase	hsa-miR-582-3p	AC011448.1		ENSG00000258674	validated
tarbase	hsa-miR-21-5p	CEP95	90799	ENSG00000258890	validated
tarbase	hsa-miR-106b-5p	BLOC1S5-TXNDC5		ENSG00000259040	validated
tarbase	hsa-miR-181a-5p	BLOC1S5-TXNDC5		ENSG00000259040	validated

tarbase	hsa-miR-505-3p	BLOC1S5-TXNDC5		ENSG00000259040	validated
tarbase	hsa-miR-106b-5p	POC1B-GALNT4	100528030	ENSG00000259075	validated
tarbase	hsa-miR-181a-5p	POC1B-GALNT4	100528030	ENSG00000259075	validated
tarbase	hsa-miR-15b-5p	THTPA	79178	ENSG00000259431	validated
tarbase	hsa-miR-106b-5p	RBM15B	29890	ENSG00000259956	validated
tarbase	hsa-miR-222-3p	HOXB7	3217	ENSG00000260027	validated
tarbase	hsa-miR-18a-5p	FRRS1L	23732	ENSG00000260230	validated
tarbase	hsa-miR-296-5p	AC093525.2		ENSG00000260272	validated
tarbase	hsa-miR-212-3p	MRC1	4360	ENSG00000260314	validated
tarbase	hsa-miR-15b-5p	AL035425.3	8471	ENSG00000260548	validated
tarbase	hsa-miR-181a-5p	AL035425.3	8471	ENSG00000260548	validated
tarbase	hsa-miR-106b-5p	CCPG1	9236	ENSG00000260916	validated
tarbase	hsa-miR-15b-5p	TMEM178B	100507421	ENSG00000261115	validated
tarbase	hsa-miR-212-3p	EPPK1	83481	ENSG00000261150	validated
tarbase	hsa-miR-106b-5p	GAN	8139	ENSG00000261609	validated
tarbase	hsa-miR-144-3p	GAN	8139	ENSG00000261609	validated
tarbase	hsa-miR-15b-5p	GAN	8139	ENSG00000261609	validated
tarbase	hsa-miR-181a-5p	GAN	8139	ENSG00000261609	validated
tarbase	hsa-miR-18a-5p	GAN	8139	ENSG00000261609	validated
tarbase	hsa-miR-21-5p	GAN	8139	ENSG00000261609	validated
tarbase	hsa-miR-301a-3p	GAN	8139	ENSG00000261609	validated
tarbase	hsa-miR-212-3p	C15orf65	145788	ENSG00000261652	validated
tarbase	hsa-miR-326	LY6L	101928108	ENSG00000261667	validated
tarbase	hsa-miR-212-3p	TCF24	100129654	ENSG00000261787	validated
tarbase	hsa-miR-212-3p	MMP12	4321	ENSG00000262406	validated
tarbase	hsa-miR-144-3p	GTF2I	2969	ENSG00000263001	validated
tarbase	hsa-miR-106b-5p	NOTCH2NLA	388677	ENSG00000264343	validated
tarbase	hsa-miR-144-3p	DYNLL2	140735	ENSG00000264364	validated
tarbase	hsa-miR-192-5p	DYNLL2	140735	ENSG00000264364	validated
tarbase	hsa-miR-301a-3p	DYNLL2	140735	ENSG00000264364	validated
tarbase	hsa-miR-15b-5p	OTUD7B	56957	ENSG00000264522	validated
tarbase	hsa-miR-301a-3p	AC138696.1		ENSG00000264668	validated
tarbase	hsa-miR-106b-5p	RBM8A	9939	ENSG00000265241	validated
tarbase	hsa-miR-181a-5p	RBM8A	9939	ENSG00000265241	validated
tarbase	hsa-miR-301a-3p	TIMM23	100287932	ENSG00000265354	validated
tarbase	hsa-miR-181a-5p	RNF115	27246	ENSG00000265491	validated
tarbase	hsa-miR-18a-5p	RPL17	6139	ENSG00000265681	validated
tarbase	hsa-miR-144-3p	TXNIP	10628	ENSG00000265972	validated
tarbase	hsa-miR-181a-5p	TXNIP	10628	ENSG00000265972	validated
tarbase	hsa-miR-192-5p	TXNIP	10628	ENSG00000265972	validated
tarbase	hsa-miR-21-5p	TXNIP	10628	ENSG00000265972	validated
tarbase	hsa-miR-301a-3p	BAHCC1	57597	ENSG00000266074	validated
tarbase	hsa-miR-106b-5p	RASSF5	83593	ENSG00000266094	validated
tarbase	hsa-miR-21-5p	NCOA4	8031	ENSG00000266412	validated
tarbase	hsa-miR-106b-5p	ZNF850	342892	ENSG00000267041	validated
tarbase	hsa-miR-181a-5p	ZNF850	342892	ENSG00000267041	validated
tarbase	hsa-miR-212-3p	ZNF850	342892	ENSG00000267041	validated
tarbase	hsa-miR-181a-5p	FDX2	112812	ENSG00000267673	validated
tarbase	hsa-miR-106b-5p	ZNF224	7767	ENSG00000267680	validated
tarbase	hsa-miR-15b-5p	ZNF224	7767	ENSG00000267680	validated
tarbase	hsa-miR-18a-5p	AC024592.3		ENSG00000267740	validated
tarbase	hsa-miR-301a-3p	NDUFA7	4701	ENSG00000267855	validated
tarbase	hsa-miR-21-5p	SLC6A14	11254	ENSG00000268104	validated
tarbase	hsa-miR-18a-5p	MTRNR2L12	100462981	ENSG00000269028	validated
tarbase	hsa-miR-18a-5p	ZNF728	388523	ENSG00000269067	validated
tarbase	hsa-miR-18a-5p	ZNF587B	100293516	ENSG00000269343	validated
tarbase	hsa-miR-192-5p	ZNF587B	100293516	ENSG00000269343	validated
tarbase	hsa-miR-192-5p	SPIB	6689	ENSG00000269404	validated
tarbase	hsa-miR-15b-5p	TMEM185A	84548	ENSG00000269556	validated
tarbase	hsa-miR-192-5p	H4C15	554313	ENSG00000270276	validated
tarbase	hsa-miR-192-5p	AL121758.1		ENSG00000270299	validated
tarbase	hsa-miR-192-5p	H4C14	8370	ENSG00000270882	validated
tarbase	hsa-miR-181a-5p	RASL10B	91608	ENSG00000270885	validated
tarbase	hsa-miR-212-3p	CCL5	6352	ENSG00000271503	validated
tarbase	hsa-miR-582-3p	LIX1L	128077	ENSG00000271601	validated
tarbase	hsa-miR-212-3p	ANKRD34A	284615	ENSG00000272031	validated
tarbase	hsa-miR-15b-5p	KMT2B	9757	ENSG00000272333	validated
tarbase	hsa-miR-296-5p	KMT2B	9757	ENSG00000272333	validated
tarbase	hsa-miR-21-5p	ZNF595	152687	ENSG00000272602	validated
tarbase	hsa-miR-212-3p	ZNF595	152687	ENSG00000272602	validated
tarbase	hsa-miR-15b-5p	DCP1A	55802	ENSG00000272886	validated

tarbase	hsa-miR-181a-5p	DCP1A	55802	ENSG00000272886	validated
tarbase	hsa-miR-21-5p	Z94721.2		ENSG00000272980	validated
tarbase	hsa-miR-212-3p	C2orf15	150590	ENSG00000273045	validated
tarbase	hsa-miR-15b-5p	GRIN2B	2904	ENSG00000273079	validated
tarbase	hsa-miR-181a-5p	GRIN2B	2904	ENSG00000273079	validated
tarbase	hsa-miR-192-5p	C1QTNF3-AMACR		ENSG00000273294	validated
tarbase	hsa-miR-15b-5p	ZNHIT3	9326	ENSG00000273611	validated
tarbase	hsa-miR-582-3p	CYFIP1	23191	ENSG00000273749	validated
tarbase	hsa-miR-15b-5p	H2BC8	8339	ENSG00000273802	validated
tarbase	hsa-miR-181a-5p	TAF9	6880	ENSG00000273841	validated
tarbase	hsa-miR-192-5p	NOL12	79159	ENSG00000273899	validated
tarbase	hsa-miR-15b-5p	NATD1	256302	ENSG00000274180	validated
tarbase	hsa-miR-144-3p	SOCS7	30837	ENSG00000274211	validated
tarbase	hsa-miR-582-5p	SOCS7	30837	ENSG00000274211	validated
tarbase	hsa-miR-212-3p	ZNF658	26149	ENSG00000274349	validated
tarbase	hsa-miR-212-3p	H2BC17	8348	ENSG00000274641	validated
tarbase	hsa-miR-212-3p	H3C6	8353	ENSG00000274750	validated
tarbase	hsa-miR-192-5p	NPHP3-ACAD11		ENSG00000274810	validated
tarbase	hsa-miR-15b-5p	H2AC12	85235	ENSG00000274997	validated
tarbase	hsa-miR-181a-5p	H2AC12	85235	ENSG00000274997	validated
tarbase	hsa-miR-18a-5p	ZNF280B	140883	ENSG00000275004	validated
tarbase	hsa-miR-192-5p	MLLT6	4302	ENSG00000275023	validated
tarbase	hsa-miR-212-3p	MLLT6	4302	ENSG00000275023	validated
tarbase	hsa-miR-296-5p	MLLT6	4302	ENSG00000275023	validated
tarbase	hsa-miR-301a-3p	MLLT6	4302	ENSG00000275023	validated
tarbase	hsa-miR-15b-5p	PPP4R3B	57223	ENSG00000275052	validated
tarbase	hsa-miR-181a-5p	SYNRG	11276	ENSG00000275066	validated
tarbase	hsa-miR-301a-3p	SYNRG	11276	ENSG00000275066	validated
tarbase	hsa-miR-212-3p	ZNF2	7549	ENSG00000275111	validated
tarbase	hsa-miR-106b-5p	ZNF2	7549	ENSG00000275111	validated
tarbase	hsa-miR-212-3p	CCL4	6351	ENSG00000275302	validated
tarbase	hsa-miR-106b-5p	PRAG1	157285	ENSG00000275342	validated
tarbase	hsa-miR-296-5p	PRAG1	157285	ENSG00000275342	validated
tarbase	hsa-miR-212-3p	H3C11	8354	ENSG00000275379	validated
tarbase	hsa-miR-212-3p	H2BC9	8345	ENSG00000275713	validated
tarbase	hsa-miR-326	H3C1	8350	ENSG00000275714	validated
tarbase	hsa-miR-192-5p	DUSP14	11072	ENSG00000276023	validated
tarbase	hsa-miR-181a-5p	ORAI1	84876	ENSG00000276045	validated
tarbase	hsa-miR-296-5p	ORAI1	84876	ENSG00000276045	validated
tarbase	hsa-miR-296-5p	H4C9	8294	ENSG00000276180	validated
tarbase	hsa-miR-21-5p	TADA2A	6871	ENSG00000276234	validated
tarbase	hsa-miR-181a-5p	PIP4K2B	8396	ENSG00000276293	validated
tarbase	hsa-miR-212-3p	PIP4K2B	8396	ENSG00000276293	validated
tarbase	hsa-miR-15b-5p	H2AC14	8331	ENSG00000276368	validated
tarbase	hsa-miR-212-3p	DACH1	1602	ENSG00000276644	validated
tarbase	hsa-miR-301a-3p	H4C5	8367	ENSG00000276966	validated
tarbase	hsa-miR-21-5p	H2AC8	3012	ENSG00000277075	validated
tarbase	hsa-miR-106b-5p	PCGF2	7703	ENSG00000277258	validated
tarbase	hsa-miR-181a-5p	PCGF2	7703	ENSG00000277258	validated
tarbase	hsa-miR-301a-3p	SRCIN1	80725	ENSG00000277363	validated
tarbase	hsa-miR-212-3p	MARCKS	4082	ENSG00000277443	validated
tarbase	hsa-miR-212-3p	ZNF670	93474	ENSG00000277462	validated
tarbase	hsa-miR-582-3p	CCL3	6348	ENSG00000277632	validated
tarbase	hsa-miR-15b-5p	ZNF8	7554	ENSG00000278129	validated
tarbase	hsa-miR-18a-5p	ZNF8	7554	ENSG00000278129	validated
tarbase	hsa-miR-106b-5p	MYO19	80179	ENSG00000278259	validated
tarbase	hsa-miR-15b-5p	MYO19	80179	ENSG00000278259	validated
tarbase	hsa-miR-106b-5p	GGNBP2	79893	ENSG00000278311	validated
tarbase	hsa-miR-181a-5p	GGNBP2	79893	ENSG00000278311	validated
tarbase	hsa-miR-181a-5p	ZNF229	7772	ENSG00000278318	validated
tarbase	hsa-miR-15b-5p	H2AC4	8335	ENSG00000278463	validated
tarbase	hsa-miR-15b-5p	ACACA	31	ENSG00000278540	validated
tarbase	hsa-miR-192-5p	H4C2	8366	ENSG00000278705	validated
tarbase	hsa-miR-21-5p	HIF-1???€			validated
tarbase	hsa-miR-21-5p	HNRPH1			validated
tarbase	hsa-miR-192-5p	HSS00009331			validated
tarbase	hsa-miR-192-5p	HSS00018228			validated
tarbase	hsa-miR-192-5p	HSS00060369			validated
tarbase	hsa-miR-192-5p	HSS00061034			validated
tarbase	hsa-miR-192-5p	HSS00062828			validated
tarbase	hsa-miR-192-5p	HSS00075325			validated

tarbase	hsa-miR-192-5p	HSS00087671			validated
tarbase	hsa-miR-192-5p	HSS00090480			validated
tarbase	hsa-miR-192-5p	HSS00102527			validated
tarbase	hsa-miR-192-5p	HSS00104143			validated
tarbase	hsa-miR-192-5p	HSS00138557			validated
tarbase	hsa-miR-192-5p	HSS00142930			validated
tarbase	hsa-miR-192-5p	HSS00209806			validated
tarbase	hsa-miR-192-5p	HSS00250837			validated
tarbase	hsa-miR-192-5p	HSS00264804			validated
tarbase	hsa-miR-192-5p	HSS00300718			validated
tarbase	hsa-miR-192-5p	HSS00303498			validated
tarbase	hsa-miR-192-5p	HSS00303609			validated
tarbase	hsa-miR-192-5p	HSS00305022			validated
tarbase	hsa-miR-192-5p	HSS00309444			validated
tarbase	hsa-miR-192-5p	HSS00321855			validated
tarbase	hsa-miR-192-5p	HSS00330832			validated
tarbase	hsa-miR-192-5p	HSS00368570			validated
tarbase	hsa-miR-192-5p	HSS00386249			validated
tarbase	hsa-miR-192-5p	HSS00388495			validated
tarbase	hsa-miR-21-5p	MMP-2			validated
tarbase	hsa-miR-192-5p	NM_006333			validated
tarbase	hsa-miR-192-5p	NM_015393			validated
tarbase	hsa-miR-192-5p	NM_015595			validated
tarbase	hsa-miR-192-5p	NM_016125			validated
tarbase	hsa-miR-192-5p	NM_017842			validated
tarbase	hsa-miR-192-5p	NM_018059			validated
tarbase	hsa-miR-192-5p	NM_018071			validated
tarbase	hsa-miR-192-5p	NM_018590			validated
tarbase	hsa-miR-192-5p	NM_020467			validated
tarbase	hsa-miR-192-5p	NM_020962			validated
tarbase	hsa-miR-192-5p	NM_024084			validated
tarbase	hsa-miR-192-5p	NM_030941			validated
tarbase	hsa-miR-192-5p	NM_032706			validated
tarbase	hsa-miR-192-5p	NM_052864			validated
tarbase	hsa-miR-192-5p	NM_053048			validated
tarbase	hsa-miR-192-5p	NM_144710			validated
tarbase	hsa-miR-192-5p	NM_173466			validated
tarbase	hsa-miR-21-5p	RASSF9	9182	ENSG00000198774	validated
tarbase	hsa-miR-21-5p	PPAR???€			validated
tarbase	hsa-miR-21-5p	PRS6			validated
tarbase	hsa-miR-192-5p	MZT1	440145	ENSG00000204899	validated
tarbase	hsa-miR-192-5p	RP11-679B17.1			validated
tarbase	hsa-miR-192-5p	RSE_00000212796			validated
tarbase	hsa-miR-192-5p	RSE_00000676604			validated
tarbase	hsa-miR-106b-5p	Smad2/3			validated
tarbase	hsa-miR-21-5p	TGF-2			validated
tarbase	hsa-miR-21-5p	ANO3	63982	ENSG00000134343	validated
tarbase	hsa-miR-106b-5p	T2RII			validated
tarbase	hsa-miR-192-5p	U29115			validated
tarbase	hsa-miR-192-5p	U79257			validated
tarbase	hsa-miR-192-5p	X97444			validated
tarbase	hsa-miR-192-5p	XM_070846			validated
tarbase	hsa-miR-192-5p	XM_170418			validated
tarbase	hsa-miR-192-5p	XM_172878			validated
tarbase	hsa-miR-192-5p	XM_208797			validated
tarbase	hsa-miR-192-5p	XM_210054			validated
tarbase	hsa-miR-192-5p	XM_210341			validated
tarbase	hsa-miR-192-5p	XM_210560			validated
tarbase	hsa-miR-192-5p	XM_211811			validated
tarbase	hsa-miR-192-5p	XM_212432			validated
tarbase	hsa-miR-192-5p	Y16700			validated
tarbase	hsa-miR-21-5p	cyclinD1			validated
tarbase	hsa-miR-192-5p	hCG_1790474			validated
tarbase	hsa-miR-192-5p	hCT10607.2			validated
tarbase	hsa-miR-192-5p	hCT14363.3			validated
tarbase	hsa-miR-192-5p	hCT1640064.3			validated
tarbase	hsa-miR-192-5p	hCT1640801.2			validated
tarbase	hsa-miR-192-5p	hCT1641762.3			validated
tarbase	hsa-miR-192-5p	hCT1642490.1			validated
tarbase	hsa-miR-192-5p	hCT1645074.2			validated
tarbase	hsa-miR-192-5p	hCT1648208.3			validated

tarbase	hsa-miR-192-5p	hCT1770636.1		validated
tarbase	hsa-miR-192-5p	hCT1776373.2		validated
tarbase	hsa-miR-192-5p	hCT1788782.2		validated
tarbase	hsa-miR-192-5p	hCT1812405.2		validated
tarbase	hsa-miR-192-5p	hCT1819827.1		validated
tarbase	hsa-miR-192-5p	hCT1831218		validated
tarbase	hsa-miR-192-5p	hCT1831253.2		validated
tarbase	hsa-miR-192-5p	hCT1835226.2		validated
tarbase	hsa-miR-192-5p	hCT1957917.1		validated
tarbase	hsa-miR-192-5p	hCT1971266.1		validated
tarbase	hsa-miR-192-5p	hCT1971392.1		validated
tarbase	hsa-miR-192-5p	hCT1971393.1		validated
tarbase	hsa-miR-192-5p	hCT2250864		validated
tarbase	hsa-miR-192-5p	hCT2255806		validated
tarbase	hsa-miR-192-5p	hCT2256635		validated
tarbase	hsa-miR-192-5p	hCT2259022		validated
tarbase	hsa-miR-192-5p	hCT2261744		validated
tarbase	hsa-miR-192-5p	hCT2283962		validated
tarbase	hsa-miR-192-5p	hCT2284819		validated
tarbase	hsa-miR-192-5p	hCT2285841		validated
tarbase	hsa-miR-192-5p	hCT2297806		validated
tarbase	hsa-miR-192-5p	hCT2306120		validated
tarbase	hsa-miR-192-5p	hCT2306937		validated
tarbase	hsa-miR-192-5p	hCT2309798		validated
tarbase	hsa-miR-192-5p	hCT2318908		validated
tarbase	hsa-miR-192-5p	hCT2318966		validated
tarbase	hsa-miR-192-5p	hCT2328509		validated
tarbase	hsa-miR-192-5p	hCT2328745		validated
tarbase	hsa-miR-192-5p	hCT2336241		validated
tarbase	hsa-miR-192-5p	hCT32256.3		validated

Table S3. Gene Ontology terms enriched among the miRNA targeted genes.

ONTOLOGY	ID	Description	Gene_Ratio	BgRatio	*P*-value	*P*-adjust	Q-value
BP	GO:0010498	proteasomal protein catabolic process	325/7778	477/18670	2.96E-32	1.91E-28	1.16E-28
BP	GO:0043161	proteasome-mediated ubiquitin-dependent protein catabolic process	290/7778	419/18670	1.03E-30	3.31E-27	2.02E-27
BP	GO:0006914	autophagy	324/7778	496/18670	4.67E-27	7.53E-24	4.58E-24
BP	GO:0061919	process utilizing autophagic mechanism	324/7778	496/18670	4.67E-27	7.53E-24	4.58E-24
BP	GO:2001233	regulation of apoptotic signaling pathway	272/7778	406/18670	2.10E-25	2.51E-22	1.53E-22
BP	GO:0000209	protein polyubiquitination	219/7778	310/18670	2.34E-25	2.51E-22	1.53E-22
BP	GO:0016569	covalent chromatin modification	304/7778	474/18670	1.59E-23	1.46E-20	8.90E-21
BP	GO:0016570	histone modification	293/7778	454/18670	2.44E-23	1.97E-20	1.20E-20
BP	GO:0034504	protein localization to nucleus	188/7778	262/18670	3.14E-23	2.25E-20	1.37E-20
BP	GO:0051169	nuclear transport	233/7778	346/18670	2.16E-22	1.39E-19	8.48E-20
BP	GO:0006913	nucleocytoplasmic transport	231/7778	343/18670	3.22E-22	1.89E-19	1.15E-19
BP	GO:0097193	intrinsic apoptotic signaling pathway	201/7778	289/18670	4.61E-22	2.48E-19	1.51E-19
BP	GO:2001234	negative regulation of apoptotic signaling pathway	167/7778	230/18670	1.13E-21	5.61E-19	3.42E-19
BP	GO:0033044	regulation of chromosome organization	229/7778	342/18670	1.55E-21	7.12E-19	4.33E-19
BP	GO:0072331	signal transduction by p53 class mediator	185/7778	267/18670	3.97E-20	1.70E-17	1.04E-17
BP	GO:0006260	DNA replication	188/7778	273/18670	5.76E-20	2.32E-17	1.41E-17
BP	GO:0009896	positive regulation of catabolic process	269/7778	425/18670	8.87E-20	3.36E-17	2.05E-17
BP	GO:0018205	peptidyl-lysine modification	254/7778	397/18670	1.19E-19	4.27E-17	2.60E-17
BP	GO:0010506	regulation of autophagy	217/7778	328/18670	1.63E-19	5.54E-17	3.37E-17
BP	GO:0016236	macroautophagy	199/7778	295/18670	1.90E-19	6.12E-17	3.73E-17
BP	GO:0006402	mRNA catabolic process	236/7778	364/18670	2.05E-19	6.29E-17	3.83E-17
BP	GO:0031331	positive regulation of cellular catabolic process	234/7778	362/18670	5.10E-19	1.49E-16	9.08E-17
BP	GO:0042176	regulation of protein catabolic process	243/7778	381/18670	1.34E-18	3.74E-16	2.28E-16
BP	GO:0045787	positive regulation of cell cycle	247/7778	389/18670	1.60E-18	4.29E-16	2.61E-16
BP	GO:0140014	mitotic nuclear division	180/7778	264/18670	1.83E-18	4.68E-16	2.85E-16
BP	GO:0006401	RNA catabolic process	251/7778	397/18670	1.89E-18	4.68E-16	2.85E-16
BP	GO:0031647	regulation of protein stability	190/7778	284/18670	4.91E-18	1.17E-15	7.13E-16
BP	GO:0051168	nuclear export	140/7778	194/18670	5.28E-18	1.22E-15	7.40E-16
BP	GO:1901990	regulation of mitotic cell cycle phase transition	274/7778	444/18670	6.12E-18	1.36E-15	8.28E-16
BP	GO:2001020	regulation of response to DNA damage stimulus	151/7778	214/18670	7.96E-18	1.71E-15	1.04E-15
BP	GO:1901987	regulation of cell cycle phase transition	292/7778	480/18670	8.72E-18	1.81E-15	1.10E-15
BP	GO:0001701	in utero embryonic development	236/7778	373/18670	1.75E-17	3.53E-15	2.15E-15
BP	GO:0001655	urogenital system development	212/7778	330/18670	6.54E-17	1.26E-14	7.66E-15
BP	GO:0061458	reproductive system development	266/7778	434/18670	6.64E-17	1.26E-14	7.66E-15
BP	GO:0033157	regulation of intracellular protein transport	169/7778	250/18670	7.36E-17	1.36E-14	8.25E-15
BP	GO:0045930	negative regulation of mitotic cell cycle	216/7778	338/18670	8.00E-17	1.43E-14	8.71E-15
BP	GO:0048608	reproductive structure development	264/7778	431/18670	9.72E-17	1.69E-14	1.03E-14
BP	GO:0010256	endomembrane system organization	271/7778	445/18670	1.10E-16	1.81E-14	1.10E-14
BP	GO:0018105	peptidyl-serine phosphorylation	195/7778	299/18670	1.13E-16	1.81E-14	1.10E-14
BP	GO:0070646	protein modification by small protein removal	195/7778	299/18670	1.13E-16	1.81E-14	1.10E-14
BP	GO:0048193	Golgi vesicle transport	231/7778	368/18670	1.49E-16	2.34E-14	1.43E-14
BP	GO:0034976	response to endoplasmic reticulum stress	187/7778	285/18670	1.90E-16	2.91E-14	1.77E-14
BP	GO:0043543	protein acylation	167/7778	248/18670	1.98E-16	2.97E-14	1.81E-14
BP	GO:0016050	vesicle organization	208/7778	325/18670	2.36E-16	3.45E-14	2.10E-14
BP	GO:0000075	cell cycle checkpoint	149/7778	216/18670	3.26E-16	4.66E-14	2.84E-14
BP	GO:1903311	regulation of mRNA metabolic process	207/7778	324/18670	3.67E-16	4.95E-14	3.01E-14
BP	GO:1903829	positive regulation of cellular protein localization	207/7778	324/18670	3.67E-16	4.95E-14	3.01E-14
BP	GO:0051656	establishment of organelle localization	247/7778	401/18670	3.69E-16	4.95E-14	3.01E-14
BP	GO:0044839	cell cycle G2/M phase transition	176/7778	266/18670	4.31E-16	5.67E-14	3.45E-14
BP	GO:0097191	extrinsic apoptotic signaling pathway	153/7778	224/18670	5.20E-16	6.70E-14	4.08E-14
BP	GO:0007050	cell cycle arrest	160/7778	237/18670	5.91E-16	7.46E-14	4.54E-14
BP	GO:0032386	regulation of intracellular transport	230/7778	370/18670	8.77E-16	1.07E-13	6.54E-14
BP	GO:0018209	peptidyl-serine modification	205/7778	322/18670	8.84E-16	1.07E-13	6.54E-14
BP	GO:0000086	G2/M transition of mitotic cell cycle	165/7778	247/18670	9.39E-16	1.12E-13	6.82E-14
BP	GO:0010948	negative regulation of cell cycle process	225/7778	361/18670	1.18E-15	1.38E-13	8.41E-14
BP	GO:0006611	protein export from nucleus	127/7778	179/18670	1.63E-15	1.87E-13	1.14E-13
BP	GO:2001252	positive regulation of chromosome organization	124/7778	174/18670	2.03E-15	2.29E-13	1.40E-13
BP	GO:0016579	protein deubiquitination	183/7778	283/18670	3.41E-15	3.79E-13	2.30E-13
BP	GO:0051052	regulation of DNA metabolic process	218/7778	351/18670	5.68E-15	6.20E-13	3.77E-13
BP	GO:0043434	response to peptide hormone	261/7778	436/18670	7.79E-15	8.36E-13	5.09E-13
BP	GO:0006352	DNA-templated transcription, initiation	164/7778	249/18670	8.07E-15	8.53E-13	5.19E-13
BP	GO:1901796	regulation of signal transduction by p53 class mediator	126/7778	180/18670	1.10E-14	1.14E-12	6.96E-13
BP	GO:0019058	viral life cycle	205/7778	328/18670	1.46E-14	1.49E-12	9.07E-13
BP	GO:0022604	regulation of cell morphogenesis	284/7778	484/18670	1.74E-14	1.75E-12	1.07E-12
BP	GO:0006403	RNA localization	153/7778	230/18670	1.79E-14	1.78E-12	1.08E-12
BP	GO:0016573	histone acetylation	112/7778	156/18670	1.99E-14	1.94E-12	1.18E-12
BP	GO:0061013	regulation of mRNA catabolic process	136/7778	199/18670	2.03E-14	1.95E-12	1.19E-12
BP	GO:0018394	peptidyl-lysine acetylation	119/7778	169/18670	3.05E-14	2.89E-12	1.76E-12
BP	GO:0030111	regulation of Wnt signaling pathway	222/7778	363/18670	3.47E-14	3.24E-12	1.97E-12
BP	GO:0016311	dephosphorylation	280/7778	478/18670	3.56E-14	3.28E-12	2.00E-12
BP	GO:0006475	internal protein amino acid acetylation	117/7778	166/18670	4.49E-14	4.07E-12	2.48E-12
BP	GO:0018393	internal peptidyl-lysine acetylation	114/7778	161/18670	5.66E-14	5.06E-12	3.08E-12
BP	GO:1900180	regulation of protein localization to nucleus	88/7778	116/18670	6.23E-14	5.50E-12	3.35E-12
BP	GO:0030099	myeloid cell differentiation	248/7778	416/18670	7.05E-14	6.06E-12	3.69E-12
BP	GO:0072001	renal system development	185/7778	293/18670	7.06E-14	6.06E-12	3.69E-12
BP	GO:0048732	gland development	257/7778	434/18670	7.31E-14	6.20E-12	3.77E-12
BP	GO:0007093	mitotic cell cycle checkpoint	116/7778	165/18670	7.66E-14	6.41E-12	3.90E-12
BP	GO:1902275	regulation of chromatin organization	127/7778	185/18670	8.77E-14	7.24E-12	4.41E-12
BP	GO:0006986	response to unfolded protein	122/7778	176/18670	8.99E-14	7.33E-12	4.46E-12
BP	GO:0010639	negative regulation of organelle organization	236/7778	393/18670	9.45E-14	7.61E-12	4.63E-12
BP	GO:0001933	negative regulation of protein phosphorylation	254/7778	429/18670	1.05E-13	8.34E-12	5.08E-12
BP	GO:0060968	regulation of gene silencing	101/7778	139/18670	1.08E-13	8.46E-12	5.15E-12
BP	GO:0015931	nucleobase-containing compound transport	157/7778	241/18670	1.23E-13	9.51E-12	5.79E-12
BP	GO:0006473	protein acetylation	137/7778	204/18670	1.36E-13	1.04E-11	6.35E-12
BP	GO:0070482	response to oxygen levels	236/7778	394/18670	1.39E-13	1.05E-11	6.42E-12
BP	GO:0042326	negative regulation of phosphorylation	273/7778	468/18670	1.48E-13	1.11E-11	6.74E-12
BP	GO:0009314	response to radiation	263/7778	448/18670	1.52E-13	1.12E-11	6.83E-12
BP	GO:0043488	regulation of mRNA stability	122/7778	177/18670	1.71E-13	1.25E-11	7.62E-12
BP	GO:1902850	microtubule cytoskeleton organization involved in mitosis	96/7778	131/18670	1.84E-13	1.33E-11	8.11E-12

BP	GO:0030522	intracellular receptor signaling pathway	176/7778	278/18670	2.01E-13	1.44E-11	8.75E-12
BP	GO:0046822	regulation of nucleocytoplasmic transport	80/7778	104/18670	2.31E-13	1.64E-11	9.96E-12
BP	GO:0043487	regulation of RNA stability	125/7778	183/18670	2.42E-13	1.70E-11	1.03E-11
BP	GO:2001242	regulation of intrinsic apoptotic signaling pathway	115/7778	165/18670	2.55E-13	1.76E-11	1.07E-11
BP	GO:0051236	establishment of RNA localization	132/7778	196/18670	2.73E-13	1.87E-11	1.14E-11
BP	GO:0060964	regulation of gene silencing by miRNA	85/7778	113/18670	3.82E-13	2.59E-11	1.58E-11
BP	GO:0031396	regulation of protein ubiquitination	136/7778	204/18670	3.93E-13	2.64E-11	1.60E-11
BP	GO:0050657	nucleic acid transport	130/7778	193/18670	4.06E-13	2.67E-11	1.63E-11
BP	GO:0050658	RNA transport	130/7778	193/18670	4.06E-13	2.67E-11	1.63E-11
BP	GO:2001236	regulation of extrinsic apoptotic signaling pathway	109/7778	155/18670	4.18E-13	2.72E-11	1.66E-11
BP	GO:0048285	organelle fission	262/7778	449/18670	4.37E-13	2.79E-11	1.70E-11
BP	GO:0032868	response to insulin	172/7778	272/18670	4.38E-13	2.79E-11	1.70E-11
BP	GO:0001822	kidney development	175/7778	278/18670	4.92E-13	3.11E-11	1.89E-11
BP	GO:0035966	response to topologically incorrect protein	133/7778	199/18670	5.35E-13	3.35E-11	2.04E-11
BP	GO:0060147	regulation of posttranscriptional gene silencing	87/7778	117/18670	6.24E-13	3.83E-11	2.33E-11
BP	GO:0060966	regulation of gene silencing by RNA	87/7778	117/18670	6.24E-13	3.83E-11	2.33E-11
BP	GO:1903320	regulation of protein modification by small protein conjugation or rem	150/7778	231/18670	6.33E-13	3.85E-11	2.34E-11
BP	GO:0044843	cell cycle G1/S phase transition	185/7778	298/18670	6.84E-13	4.08E-11	2.48E-11
BP	GO:0090068	positive regulation of cell cycle process	185/7778	298/18670	6.84E-13	4.08E-11	2.48E-11
BP	GO:0006338	chromatin remodeling	144/7778	220/18670	7.25E-13	4.28E-11	2.61E-11
BP	GO:0000082	G1/S transition of mitotic cell cycle	175/7778	279/18670	7.80E-13	4.57E-11	2.78E-11
BP	GO:0001666	response to hypoxia	216/7778	359/18670	8.06E-13	4.68E-11	2.85E-11
BP	GO:0042770	signal transduction in response to DNA damage	96/7778	133/18670	8.48E-13	4.88E-11	2.97E-11
BP	GO:0051098	regulation of binding	223/7778	373/18670	8.57E-13	4.89E-11	2.98E-11
BP	GO:0050821	protein stabilization	121/7778	178/18670	9.84E-13	5.52E-11	3.36E-11
BP	GO:1903362	regulation of cellular protein catabolic process	158/7778	247/18670	9.85E-13	5.52E-11	3.36E-11
BP	GO:0007265	Ras protein signal transduction	260/7778	448/18670	1.24E-12	6.82E-11	4.15E-11
BP	GO:0036293	response to decreased oxygen levels	221/7778	370/18670	1.24E-12	6.82E-11	4.15E-11
BP	GO:0006470	protein dephosphorylation	196/7778	321/18670	1.35E-12	7.39E-11	4.50E-11
BP	GO:0007163	establishment or maintenance of cell polarity	138/7778	210/18670	1.39E-12	7.52E-11	4.58E-11
BP	GO:0048872	homeostasis of number of cells	157/7778	246/18670	1.53E-12	8.21E-11	5.00E-11
BP	GO:0006979	response to oxidative stress	261/7778	451/18670	1.73E-12	9.18E-11	5.59E-11
BP	GO:0062197	cellular response to chemical stress	210/7778	350/18670	2.44E-12	1.29E-10	7.86E-11
BP	GO:0010212	response to ionizing radiation	103/7778	147/18670	2.64E-12	1.38E-10	8.40E-11
BP	GO:1903706	regulation of hemopoiesis	272/7778	475/18670	3.03E-12	1.57E-10	9.57E-11
BP	GO:0071496	cellular response to external stimulus	204/7778	339/18670	3.40E-12	1.75E-10	1.07E-10
BP	GO:0016241	regulation of macroautophagy	116/7778	171/18670	3.58E-12	1.83E-10	1.11E-10
BP	GO:0000280	nuclear division	238/7778	407/18670	3.82E-12	1.94E-10	1.18E-10
BP	GO:0032388	positive regulation of intracellular transport	147/7778	229/18670	4.06E-12	2.04E-10	1.24E-10
BP	GO:0031503	protein-containing complex localization	174/7778	281/18670	4.51E-12	2.25E-10	1.37E-10
BP	GO:0006367	transcription initiation from RNA polymerase II promoter	125/7778	188/18670	4.61E-12	2.27E-10	1.38E-10
BP	GO:2001237	negative regulation of extrinsic apoptotic signaling pathway	78/7778	104/18670	4.61E-12	2.27E-10	1.38E-10
BP	GO:1900034	regulation of cellular response to heat	63/7778	79/18670	4.71E-12	2.30E-10	1.40E-10
BP	GO:0010975	regulation of neuron projection development	283/7778	499/18670	4.98E-12	2.41E-10	1.47E-10
BP	GO:0010821	regulation of mitochondrion organization	120/7778	179/18670	5.28E-12	2.54E-10	1.54E-10
BP	GO:0070585	protein localization to mitochondrion	99/7778	141/18670	5.73E-12	2.73E-10	1.66E-10
BP	GO:0031056	regulation of histone modification	100/7778	143/18670	6.61E-12	3.13E-10	1.90E-10
BP	GO:1902749	regulation of cell cycle G2/M phase transition	138/7778	213/18670	6.74E-12	3.17E-10	1.93E-10
BP	GO:0006605	protein targeting	251/7778	435/18670	7.06E-12	3.30E-10	2.01E-10
BP	GO:0090316	positive regulation of intracellular protein transport	118/7778	176/18670	7.84E-12	3.63E-10	2.21E-10
BP	GO:0019080	viral gene expression	126/7778	191/18670	8.68E-12	3.99E-10	2.43E-10
BP	GO:1901991	negative regulation of mitotic cell cycle phase transition	156/7778	248/18670	9.76E-12	4.46E-10	2.72E-10
BP	GO:0051650	establishment of vesicle localization	122/7778	184/18670	1.10E-11	5.01E-10	3.05E-10
BP	GO:1903050	regulation of proteolysis involved in cellular protein catabolic process	138/7778	214/18670	1.12E-11	5.01E-10	3.05E-10
BP	GO:0070936	protein K48-linked ubiquitination	48/7778	56/18670	1.12E-11	5.01E-10	3.05E-10
BP	GO:0071103	DNA conformation change	215/7778	364/18670	1.13E-11	5.01E-10	3.05E-10
BP	GO:0032869	cellular response to insulin stimulus	139/7778	216/18670	1.18E-11	5.23E-10	3.18E-10
BP	GO:0062012	regulation of small molecule metabolic process	247/7778	429/18670	1.41E-11	6.19E-10	3.77E-10
BP	GO:1990778	protein localization to cell periphery	192/7778	319/18670	1.43E-11	6.22E-10	3.79E-10
BP	GO:0072655	establishment of protein localization to mitochondrion	96/7778	137/18670	1.44E-11	6.22E-10	3.79E-10
BP	GO:0071214	cellular response to abiotic stimulus	198/7778	331/18670	1.51E-11	6.46E-10	3.93E-10
BP	GO:0104004	cellular response to environmental stimulus	198/7778	331/18670	1.51E-11	6.46E-10	3.93E-10
BP	GO:0031570	DNA integrity checkpoint	107/7778	157/18670	1.56E-11	6.63E-10	4.04E-10
BP	GO:0043687	post-translational protein modification	213/7778	361/18670	1.62E-11	6.81E-10	4.15E-10
BP	GO:1901988	negative regulation of cell cycle phase transition	165/7778	267/18670	2.02E-11	8.46E-10	5.15E-10
BP	GO:0045931	positive regulation of mitotic cell cycle	110/7778	163/18670	2.13E-11	8.85E-10	5.39E-10
BP	GO:0009408	response to heat	117/7778	176/18670	2.25E-11	9.28E-10	5.65E-10
BP	GO:0051348	negative regulation of transferase activity	174/7778	285/18670	2.47E-11	1.01E-09	6.16E-10
BP	GO:0006839	mitochondrial transport	160/7778	258/18670	2.73E-11	1.11E-09	6.79E-10
BP	GO:0060070	canonical Wnt signaling pathway	199/7778	335/18670	3.24E-11	1.31E-09	8.00E-10
BP	GO:0070507	regulation of microtubule cytoskeleton organization	122/7778	186/18670	3.29E-11	1.32E-09	8.07E-10
BP	GO:0000070	mitotic sister chromatid segregation	103/7778	151/18670	3.45E-11	1.38E-09	8.39E-10
BP	GO:0060828	regulation of canonical Wnt signaling pathway	174/7778	286/18670	3.72E-11	1.48E-09	9.00E-10
BP	GO:0007088	regulation of mitotic nuclear division	110/7778	164/18670	3.83E-11	1.51E-09	9.20E-10
BP	GO:0071456	cellular response to hypoxia	133/7778	207/18670	3.85E-11	1.51E-09	9.20E-10
BP	GO:2001243	negative regulation of intrinsic apoptotic signaling pathway	73/7778	98/18670	3.90E-11	1.52E-09	9.26E-10
BP	GO:0019083	viral transcription	117/7778	177/18670	3.92E-11	1.52E-09	9.26E-10
BP	GO:0006405	RNA export from nucleus	94/7778	135/18670	4.13E-11	1.59E-09	9.71E-10
BP	GO:0034599	cellular response to oxidative stress	182/7778	302/18670	4.16E-11	1.60E-09	9.71E-10
BP	GO:0010389	regulation of G2/M transition of mitotic cell cycle	127/7778	196/18670	4.55E-11	1.73E-09	1.06E-09
BP	GO:0034605	cellular response to heat	95/7778	137/18670	4.71E-11	1.79E-09	1.09E-09
BP	GO:0061136	regulation of proteasomal protein catabolic process	120/7778	183/18670	4.89E-11	1.84E-09	1.12E-09
BP	GO:0016049	cell growth	272/7778	484/18670	4.91E-11	1.84E-09	1.12E-09
BP	GO:0098727	maintenance of cell number	107/7778	159/18670	5.18E-11	1.92E-09	1.17E-09
BP	GO:0030330	DNA damage response, signal transduction by p53 class mediator	78/7778	107/18670	5.20E-11	1.92E-09	1.17E-09
BP	GO:0051170	import into nucleus	109/7778	163/18670	6.22E-11	2.29E-09	1.39E-09
BP	GO:0031098	stress-activated protein kinase signaling cascade	188/7778	315/18670	6.47E-11	2.37E-09	1.44E-09
BP	GO:0071375	cellular response to peptide hormone stimulus	191/7778	321/18670	6.59E-11	2.40E-09	1.46E-09
BP	GO:0008630	intrinsic apoptotic signaling pathway in response to DNA damage	76/7778	104/18670	7.52E-11	2.72E-09	1.66E-09
BP	GO:0000077	DNA damage checkpoint	99/7778	145/18670	7.59E-11	2.73E-09	1.66E-09
BP	GO:0001558	regulation of cell growth	238/7778	416/18670	7.82E-11	2.80E-09	1.70E-09
BP	GO:0007052	mitotic spindle organization	77/7778	106/18670	9.17E-11	3.26E-09	1.99E-09

BP	GO:0090150	establishment of protein localization to membrane	196/7778	332/18670	9.69E-11	3.43E-09	2.09E-09
BP	GO:0071453	cellular response to oxygen levels	146/7778	234/18670	1.06E-10	3.73E-09	2.27E-09
BP	GO:0030177	positive regulation of Wnt signaling pathway	117/7778	179/18670	1.15E-10	4.04E-09	2.46E-09
BP	GO:0044766	multi-organism transport	50/7778	61/18670	1.19E-10	4.12E-09	2.51E-09
BP	GO:1902579	multi-organism localization	50/7778	61/18670	1.19E-10	4.12E-09	2.51E-09
BP	GO:0036294	cellular response to decreased oxygen levels	137/7778	217/18670	1.21E-10	4.18E-09	2.55E-09
BP	GO:0048511	rhythmic process	177/7778	295/18670	1.27E-10	4.37E-09	2.66E-09
BP	GO:0010769	regulation of cell morphogenesis involved in differentiation	180/7778	301/18670	1.31E-10	4.46E-09	2.71E-09
BP	GO:0019827	stem cell population maintenance	105/7778	157/18670	1.37E-10	4.66E-09	2.84E-09
BP	GO:0006513	protein monoubiquitination	53/7778	66/18670	1.46E-10	4.91E-09	2.99E-09
BP	GO:0044786	cell cycle DNA replication	56/7778	71/18670	1.62E-10	5.45E-09	3.32E-09
BP	GO:0045732	positive regulation of protein catabolic process	135/7778	214/18670	1.79E-10	5.98E-09	3.64E-09
BP	GO:0071902	positive regulation of protein serine/threonine kinase activity	196/7778	334/18670	1.99E-10	6.60E-09	4.02E-09
BP	GO:0016197	endosomal transport	141/7778	226/18670	2.21E-10	7.32E-09	4.45E-09
BP	GO:0072332	intrinsic apoptotic signaling pathway by p53 class mediator	60/7778	78/18670	2.31E-10	7.58E-09	4.62E-09
BP	GO:0046794	transport of virus	49/7778	60/18670	2.36E-10	7.71E-09	4.69E-09
BP	GO:0098732	macromolecule deacylation	75/7778	104/18670	2.83E-10	9.20E-09	5.60E-09
BP	GO:0007059	chromosome segregation	189/7778	321/18670	2.85E-10	9.21E-09	5.61E-09
BP	GO:0006302	double-strand break repair	152/7778	248/18670	2.95E-10	9.46E-09	5.76E-09
BP	GO:0071383	cellular response to steroid hormone stimulus	152/7778	248/18670	2.95E-10	9.46E-09	5.76E-09
BP	GO:0034620	cellular response to unfolded protein	95/7778	140/18670	3.08E-10	9.82E-09	5.98E-09
BP	GO:0001503	ossification	227/7778	398/18670	3.16E-10	1.00E-08	6.10E-09
BP	GO:0051648	vesicle localization	128/7778	202/18670	3.44E-10	1.09E-08	6.62E-09
BP	GO:0051403	stress-activated MAPK cascade	171/7778	286/18670	3.84E-10	1.20E-08	7.32E-09
BP	GO:0006469	negative regulation of protein kinase activity	145/7778	235/18670	3.85E-10	1.20E-08	7.32E-09
BP	GO:0070997	neuron death	202/7778	348/18670	3.91E-10	1.22E-08	7.41E-09
BP	GO:0030010	establishment of cell polarity	92/7778	135/18670	4.11E-10	1.27E-08	7.74E-09
BP	GO:2001251	negative regulation of chromosome organization	98/7778	146/18670	4.13E-10	1.27E-08	7.74E-09
BP	GO:0051054	positive regulation of DNA metabolic process	122/7778	191/18670	4.24E-10	1.30E-08	7.93E-09
BP	GO:0032479	regulation of type I interferon production	87/7778	126/18670	4.32E-10	1.32E-08	8.02E-09
BP	GO:2000756	regulation of peptidyl-lysine acetylation	48/7778	59/18670	4.65E-10	1.41E-08	8.61E-09
BP	GO:0032606	type I interferon production	88/7778	128/18670	4.87E-10	1.47E-08	8.97E-09
BP	GO:0043903	regulation of interspecies interactions between organisms	138/7778	222/18670	4.92E-10	1.47E-08	8.97E-09
BP	GO:0032392	DNA geometric change	83/7778	119/18670	4.94E-10	1.47E-08	8.97E-09
BP	GO:0035601	protein deacylation	74/7778	103/18670	4.94E-10	1.47E-08	8.97E-09
BP	GO:0030705	cytoskeleton-dependent intracellular transport	116/7778	180/18670	5.11E-10	1.52E-08	9.23E-09
BP	GO:1905897	regulation of response to endoplasmic reticulum stress	63/7778	84/18670	5.23E-10	1.55E-08	9.42E-09
BP	GO:0051028	mRNA transport	101/7778	152/18670	5.35E-10	1.58E-08	9.59E-09
BP	GO:1903578	regulation of ATP metabolic process	84/7778	121/18670	5.63E-10	1.65E-08	1.00E-08
BP	GO:0009615	response to virus	189/7778	323/18670	5.79E-10	1.69E-08	1.03E-08
BP	GO:1900182	positive regulation of protein localization to nucleus	57/7778	74/18670	5.94E-10	1.72E-08	1.05E-08
BP	GO:0006606	protein import into nucleus	96/7778	143/18670	6.13E-10	1.77E-08	1.08E-08
BP	GO:0051783	regulation of nuclear division	120/7778	188/18670	6.28E-10	1.80E-08	1.10E-08
BP	GO:0016482	cytosolic transport	104/7778	158/18670	6.76E-10	1.93E-08	1.18E-08
BP	GO:0017038	protein import	122/7778	192/18670	6.88E-10	1.96E-08	1.19E-08
BP	GO:0045862	positive regulation of proteolysis	206/7778	358/18670	7.42E-10	2.11E-08	1.28E-08
BP	GO:2000058	regulation of ubiquitin-dependent protein catabolic process	99/7778	149/18670	7.96E-10	2.25E-08	1.37E-08
BP	GO:0071426	ribonucleoprotein complex export from nucleus	87/7778	127/18670	8.11E-10	2.28E-08	1.39E-08
BP	GO:0071479	cellular response to ionizing radiation	55/7778	71/18670	8.16E-10	2.29E-08	1.39E-08
BP	GO:0060562	epithelial tube morphogenesis	188/7778	322/18670	8.23E-10	2.30E-08	1.40E-08
BP	GO:0016358	dendrite development	143/7778	233/18670	8.61E-10	2.39E-08	1.45E-08
BP	GO:0006261	DNA-dependent DNA replication	100/7778	151/18670	8.63E-10	2.39E-08	1.45E-08
BP	GO:0046777	protein autophosphorylation	144/7778	235/18670	8.79E-10	2.42E-08	1.47E-08
BP	GO:1901653	cellular response to peptide	219/7778	385/18670	8.92E-10	2.45E-08	1.49E-08
BP	GO:0007051	spindle organization	110/7778	170/18670	1.00E-09	2.73E-08	1.66E-08
BP	GO:0000819	sister chromatid segregation	120/7778	189/18670	1.02E-09	2.76E-08	1.68E-08
BP	GO:2000027	regulation of animal organ morphogenesis	153/7778	253/18670	1.02E-09	2.77E-08	1.69E-08
BP	GO:0033673	negative regulation of kinase activity	155/7778	257/18670	1.05E-09	2.82E-08	1.72E-08
BP	GO:0035329	hippo signaling	34/7778	38/18670	1.06E-09	2.82E-08	1.72E-08
BP	GO:0097064	ncRNA export from nucleus	34/7778	38/18670	1.06E-09	2.82E-08	1.72E-08
BP	GO:0019079	viral genome replication	84/7778	122/18670	1.07E-09	2.84E-08	1.73E-08
BP	GO:0032434	regulation of proteasomal ubiquitin-dependent protein catabolic proc	84/7778	122/18670	1.07E-09	2.84E-08	1.73E-08
BP	GO:0035967	cellular response to topologically incorrect protein	105/7778	161/18670	1.23E-09	3.26E-08	1.98E-08
BP	GO:1904951	positive regulation of establishment of protein localization	253/7778	456/18670	1.25E-09	3.28E-08	2.00E-08
BP	GO:0051817	modulation of process of other organism involved in symbiotic intera	71/7778	99/18670	1.26E-09	3.29E-08	2.01E-08
BP	GO:0043401	steroid hormone mediated signaling pathway	116/7778	182/18670	1.37E-09	3.58E-08	2.18E-08
BP	GO:0000956	nuclear-transcribed mRNA catabolic process	129/7778	207/18670	1.42E-09	3.69E-08	2.25E-08
BP	GO:0071166	ribonucleoprotein complex localization	87/7778	128/18670	1.50E-09	3.88E-08	2.36E-08
BP	GO:0072659	protein localization to plasma membrane	156/7778	260/18670	1.57E-09	4.05E-08	2.46E-08
BP	GO:0010770	positive regulation of cell morphogenesis involved in differentiation	101/7778	154/18670	1.60E-09	4.11E-08	2.50E-08
BP	GO:0009411	response to UV	94/7778	141/18670	1.62E-09	4.14E-08	2.52E-08
BP	GO:0000910	cytokinesis	110/7778	171/18670	1.66E-09	4.23E-08	2.57E-08
BP	GO:0051983	regulation of chromosome segregation	73/7778	103/18670	1.75E-09	4.42E-08	2.69E-08
BP	GO:1905269	positive regulation of chromatin organization	73/7778	103/18670	1.75E-09	4.42E-08	2.69E-08
BP	GO:0030968	endoplasmic reticulum unfolded protein response	83/7778	121/18670	1.79E-09	4.48E-08	2.73E-08
BP	GO:0075733	intracellular transport of virus	46/7778	57/18670	1.79E-09	4.48E-08	2.73E-08
BP	GO:0001649	osteoblast differentiation	138/7778	225/18670	1.79E-09	4.48E-08	2.73E-08
BP	GO:0009266	response to temperature stimulus	147/7778	243/18670	2.10E-09	5.22E-08	3.18E-08
BP	GO:0050792	regulation of viral process	129/7778	208/18670	2.21E-09	5.48E-08	3.33E-08
BP	GO:0032147	activation of protein kinase activity	192/7778	333/18670	2.23E-09	5.51E-08	3.35E-08
BP	GO:1903364	positive regulation of cellular protein catabolic process	93/7778	140/18670	2.62E-09	6.43E-08	3.92E-08
BP	GO:0006476	protein deacetylation	67/7778	93/18670	2.69E-09	6.59E-08	4.01E-08
BP	GO:0043467	regulation of generation of precursor metabolites and energy	102/7778	157/18670	2.90E-09	7.07E-08	4.31E-08
BP	GO:0000079	regulation of cyclin-dependent protein serine/threonine kinase activity	72/7778	102/18670	3.00E-09	7.29E-08	4.44E-08
BP	GO:2001021	negative regulation of response to DNA damage stimulus	60/7778	81/18670	3.09E-09	7.49E-08	4.56E-08
BP	GO:0006282	regulation of DNA repair	83/7778	122/18670	3.31E-09	7.99E-08	4.86E-08
BP	GO:0009895	negative regulation of catabolic process	179/7778	308/18670	3.38E-09	8.13E-08	4.95E-08
BP	GO:1903322	positive regulation of protein modification by small protein conjugatio	90/7778	135/18670	3.58E-09	8.57E-08	5.22E-08
BP	GO:0008637	apoptotic mitochondrial changes	84/7778	124/18670	3.67E-09	8.76E-08	5.34E-08
BP	GO:0034502	protein localization to chromosome	61/7778	83/18670	3.85E-09	9.14E-08	5.57E-08
BP	GO:1904029	regulation of cyclin-dependent protein kinase activity	74/7778	106/18670	4.00E-09	9.47E-08	5.76E-08
BP	GO:0043405	regulation of MAP kinase activity	193/7778	337/18670	4.25E-09	1.00E-07	6.10E-08

BP	GO:0031398	positive regulation of protein ubiquitination	80/7778	117/18670	4.41E-09	1.04E-07	6.32E-08
BP	GO:0035065	regulation of histone acetylation	43/7778	53/18670	4.49E-09	1.05E-07	6.40E-08
BP	GO:0032200	telomere organization	111/7778	175/18670	4.62E-09	1.08E-07	6.57E-08
BP	GO:0031330	negative regulation of cellular catabolic process	151/7778	253/18670	4.76E-09	1.11E-07	6.74E-08
BP	GO:0006997	nucleus organization	87/7778	130/18670	4.88E-09	1.13E-07	6.88E-08
BP	GO:0048813	dendrite morphogenesis	94/7778	143/18670	4.90E-09	1.13E-07	6.88E-08
BP	GO:0050673	epithelial cell proliferation	240/7778	434/18670	4.93E-09	1.13E-07	6.91E-08
BP	GO:0032508	DNA duplex unwinding	76/7778	110/18670	5.18E-09	1.19E-07	7.23E-08
BP	GO:0043618	regulation of transcription from RNA polymerase II promoter in respo	82/7778	121/18670	5.46E-09	1.25E-07	7.59E-08
BP	GO:0002262	myeloid cell homeostasis	96/7778	147/18670	5.62E-09	1.28E-07	7.79E-08
BP	GO:0031346	positive regulation of cell projection organization	215/7778	383/18670	5.86E-09	1.33E-07	8.10E-08
BP	GO:0071478	cellular response to radiation	119/7778	191/18670	6.20E-09	1.40E-07	8.54E-08
BP	GO:0007548	sex differentiation	159/7778	270/18670	7.10E-09	1.60E-07	9.74E-08
BP	GO:0061418	regulation of transcription from RNA polymerase II promoter in respo	57/7778	77/18670	7.98E-09	1.79E-07	1.09E-07
BP	GO:0032984	protein-containing complex disassembly	185/7778	323/18670	8.75E-09	1.96E-07	1.19E-07
BP	GO:0034401	chromatin organization involved in regulation of transcription	88/7778	133/18670	9.32E-09	2.08E-07	1.26E-07
BP	GO:1902115	regulation of organelle assembly	120/7778	194/18670	9.91E-09	2.20E-07	1.34E-07
BP	GO:0045814	negative regulation of gene expression, epigenetic	71/7778	102/18670	1.00E-08	2.22E-07	1.35E-07
BP	GO:0071559	response to transforming growth factor beta	151/7778	255/18670	1.01E-08	2.22E-07	1.35E-07
BP	GO:0046824	positive regulation of nucleocytoplasmic transport	48/7778	62/18670	1.02E-08	2.22E-07	1.35E-07
BP	GO:0001763	morphogenesis of a branching structure	121/7778	196/18670	1.02E-08	2.22E-07	1.35E-07
BP	GO:0071897	DNA biosynthetic process	121/7778	196/18670	1.02E-08	2.22E-07	1.35E-07
BP	GO:0001890	placenta development	98/7778	152/18670	1.06E-08	2.31E-07	1.41E-07
BP	GO:0006892	post-Golgi vesicle-mediated transport	72/7778	104/18670	1.14E-08	2.47E-07	1.50E-07
BP	GO:2001235	positive regulation of apoptotic signaling pathway	112/7778	179/18670	1.22E-08	2.63E-07	1.60E-07
BP	GO:0032886	regulation of microtubule-based process	132/7778	218/18670	1.26E-08	2.71E-07	1.65E-07
BP	GO:0044774	mitotic DNA integrity checkpoint	73/7778	106/18670	1.29E-08	2.76E-07	1.68E-07
BP	GO:0007044	cell-substrate junction assembly	68/7778	97/18670	1.29E-08	2.76E-07	1.68E-07
BP	GO:0150115	cell-substrate junction organization	68/7778	97/18670	1.29E-08	2.76E-07	1.68E-07
BP	GO:0008380	RNA splicing	255/7778	469/18670	1.34E-08	2.85E-07	1.74E-07
BP	GO:0000723	telomere maintenance	103/7778	162/18670	1.36E-08	2.89E-07	1.76E-07
BP	GO:0044783	G1 DNA damage checkpoint	49/7778	64/18670	1.37E-08	2.90E-07	1.76E-07
BP	GO:1905475	regulation of protein localization to membrane	116/7778	187/18670	1.38E-08	2.90E-07	1.76E-07
BP	GO:0035196	production of miRNAs involved in gene silencing by miRNA	38/7778	46/18670	1.39E-08	2.92E-07	1.78E-07
BP	GO:0031668	cellular response to extracellular stimulus	157/7778	268/18670	1.45E-08	3.03E-07	1.85E-07
BP	GO:0019693	ribose phosphate metabolic process	233/7778	424/18670	1.70E-08	3.55E-07	2.16E-07
BP	GO:0031050	dsRNA processing	41/7778	51/18670	1.72E-08	3.56E-07	2.17E-07
BP	GO:0070918	production of small RNA involved in gene silencing by RNA	41/7778	51/18670	1.72E-08	3.56E-07	2.17E-07
BP	GO:0035303	regulation of dephosphorylation	127/7778	209/18670	1.75E-08	3.61E-07	2.20E-07
BP	GO:1901214	regulation of neuron death	179/7778	313/18670	1.77E-08	3.64E-07	2.21E-07
BP	GO:0061008	hepaticobiliary system development	90/7778	138/18670	1.84E-08	3.77E-07	2.30E-07
BP	GO:0031589	cell-substrate adhesion	199/7778	354/18670	1.84E-08	3.77E-07	2.30E-07
BP	GO:0043393	regulation of protein binding	131/7778	217/18670	1.85E-08	3.77E-07	2.30E-07
BP	GO:0006457	protein folding	136/7778	227/18670	1.95E-08	3.97E-07	2.42E-07
BP	GO:1901983	regulation of protein acetylation	54/7778	73/18670	2.06E-08	4.16E-07	2.53E-07
BP	GO:0043620	regulation of DNA-templated transcription in response to stress	84/7778	127/18670	2.06E-08	4.16E-07	2.53E-07
BP	GO:0071560	cellular response to transforming growth factor beta stimulus	147/7778	249/18670	2.07E-08	4.16E-07	2.53E-07
BP	GO:0043470	regulation of carbohydrate catabolic process	62/7778	87/18670	2.07E-08	4.16E-07	2.53E-07
BP	GO:0010970	transport along microtubule	102/7778	161/18670	2.11E-08	4.21E-07	2.56E-07
BP	GO:0010822	positive regulation of mitochondrion organization	78/7778	116/18670	2.18E-08	4.34E-07	2.64E-07
BP	GO:2000736	regulation of stem cell differentiation	78/7778	116/18670	2.18E-08	4.34E-07	2.64E-07
BP	GO:0006409	tRNA export from nucleus	30/7778	34/18670	2.24E-08	4.43E-07	2.70E-07
BP	GO:0071431	tRNA-containing ribonucleoprotein complex export from nucleus	30/7778	34/18670	2.24E-08	4.43E-07	2.70E-07
BP	GO:0048524	positive regulation of viral process	73/7778	107/18670	2.40E-08	4.73E-07	2.88E-07
BP	GO:0031124	mRNA 3'-end processing	68/7778	98/18670	2.50E-08	4.92E-07	2.99E-07
BP	GO:0031571	mitotic G1 DNA damage checkpoint	48/7778	63/18670	2.54E-08	4.96E-07	3.02E-07
BP	GO:0044819	mitotic G1/S transition checkpoint	48/7778	63/18670	2.54E-08	4.96E-07	3.02E-07
BP	GO:0046034	ATP metabolic process	174/7778	304/18670	2.56E-08	4.98E-07	3.03E-07
BP	GO:0016925	protein sumoylation	59/7778	82/18670	2.57E-08	4.98E-07	3.03E-07
BP	GO:0006090	pyruvate metabolic process	96/7778	150/18670	2.59E-08	5.01E-07	3.05E-07
BP	GO:0001889	liver development	88/7778	135/18670	2.74E-08	5.28E-07	3.21E-07
BP	GO:0061640	cytoskeleton-dependent cytokinesis	69/7778	100/18670	2.84E-08	5.44E-07	3.31E-07
BP	GO:0070498	interleukin-1-mediated signaling pathway	69/7778	100/18670	2.84E-08	5.44E-07	3.31E-07
BP	GO:0007409	axonogenesis	253/7778	468/18670	3.01E-08	5.75E-07	3.50E-07
BP	GO:0009259	ribonucleotide metabolic process	227/7778	414/18670	3.32E-08	6.32E-07	3.85E-07
BP	GO:0007034	vacuolar transport	92/7778	143/18670	3.46E-08	6.58E-07	4.01E-07
BP	GO:0033260	nuclear DNA replication	46/7778	60/18670	3.55E-08	6.73E-07	4.10E-07
BP	GO:1901800	positive regulation of proteasomal protein catabolic process	71/7778	104/18670	3.57E-08	6.75E-07	4.11E-07
BP	GO:0045069	regulation of viral genome replication	66/7778	95/18670	3.71E-08	6.99E-07	4.26E-07
BP	GO:0030518	intracellular steroid hormone receptor signaling pathway	85/7778	130/18670	3.81E-08	7.15E-07	4.35E-07
BP	GO:0090263	positive regulation of canonical Wnt signaling pathway	94/7778	147/18670	3.84E-08	7.18E-07	4.37E-07
BP	GO:0048863	stem cell differentiation	150/7778	257/18670	4.20E-08	7.84E-07	4.77E-07
BP	GO:0044773	mitotic DNA damage checkpoint	67/7778	97/18670	4.21E-08	7.84E-07	4.77E-07
BP	GO:0045637	regulation of myeloid cell differentiation	147/7778	251/18670	4.23E-08	7.85E-07	4.78E-07
BP	GO:0051222	positive regulation of protein transport	239/7778	440/18670	4.24E-08	7.85E-07	4.78E-07
BP	GO:0018210	peptidyl-threonine modification	87/7778	134/18670	4.33E-08	7.97E-07	4.85E-07
BP	GO:0022406	membrane docking	110/7778	178/18670	4.33E-08	7.97E-07	4.85E-07
BP	GO:0006275	regulation of DNA replication	73/7778	108/18670	4.40E-08	8.07E-07	4.91E-07
BP	GO:0051031	tRNA transport	31/7778	36/18670	4.52E-08	8.28E-07	5.04E-07
BP	GO:0061138	morphogenesis of a branching epithelium	112/7778	182/18670	4.55E-08	8.30E-07	5.05E-07
BP	GO:0032922	circadian regulation of gene expression	47/7778	62/18670	4.69E-08	8.50E-07	5.18E-07
BP	GO:0070059	intrinsic apoptotic signaling pathway in response to endoplasmic retic	47/7778	62/18670	4.69E-08	8.50E-07	5.18E-07
BP	GO:0031058	positive regulation of histone modification	63/7778	90/18670	4.81E-08	8.70E-07	5.30E-07
BP	GO:0003279	cardiac septum development	75/7778	112/18670	5.30E-08	9.56E-07	5.82E-07
BP	GO:0007033	vacuole organization	102/7778	163/18670	5.32E-08	9.56E-07	5.82E-07
BP	GO:0071824	protein-DNA complex subunit organization	165/7778	288/18670	5.33E-08	9.56E-07	5.82E-07
BP	GO:0043087	regulation of GTPase activity	257/7778	479/18670	5.67E-08	1.02E-06	6.18E-07
BP	GO:0006888	endoplasmic reticulum to Golgi vesicle-mediated transport	127/7778	212/18670	5.73E-08	1.02E-06	6.23E-07
BP	GO:0035264	multicellular organism growth	93/7778	146/18670	5.95E-08	1.06E-06	6.45E-07
BP	GO:0048545	response to steroid hormone	211/7778	383/18670	6.13E-08	1.09E-06	6.62E-07
BP	GO:1901532	regulation of hematopoietic progenitor cell differentiation	60/7778	85/18670	6.15E-08	1.09E-06	6.63E-07
BP	GO:0035148	tube formation	94/7778	148/18670	6.23E-08	1.10E-06	6.69E-07

BP	GO:1901216	positive regulation of neuron death	65/7778	94/18670	6.25E-08	1.10E-06	6.70E-07
BP	GO:0043254	regulation of protein-containing complex assembly	233/7778	429/18670	6.32E-08	1.11E-06	6.75E-07
BP	GO:0001841	neural tube formation	71/7778	105/18670	6.54E-08	1.15E-06	6.97E-07
BP	GO:0030521	androgen receptor signaling pathway	45/7778	59/18670	6.59E-08	1.15E-06	7.00E-07
BP	GO:0051099	positive regulation of binding	110/7778	179/18670	6.65E-08	1.16E-06	7.04E-07
BP	GO:1903052	positive regulation of proteolysis involved in cellular protein catabolic	79/7778	120/18670	7.32E-08	1.27E-06	7.74E-07
BP	GO:0007179	transforming growth factor beta receptor signaling pathway	120/7778	199/18670	7.87E-08	1.36E-06	8.30E-07
BP	GO:0030278	regulation of ossification	122/7778	203/18670	8.05E-08	1.39E-06	8.45E-07
BP	GO:0007249	I-kappaB kinase/NF-kappaB signaling	155/7778	269/18670	8.06E-08	1.39E-06	8.45E-07
BP	GO:0002244	hematopoietic progenitor cell differentiation	103/7778	166/18670	8.58E-08	1.46E-06	8.91E-07
BP	GO:0045137	development of primary sexual characteristics	132/7778	223/18670	8.60E-08	1.46E-06	8.91E-07
BP	GO:1903902	positive regulation of viral life cycle	46/7778	61/18670	8.60E-08	1.46E-06	8.91E-07
BP	GO:0006406	mRNA export from nucleus	74/7778	111/18670	8.61E-08	1.46E-06	8.91E-07
BP	GO:0071427	mRNA-containing ribonucleoprotein complex export from nucleus	74/7778	111/18670	8.61E-08	1.46E-06	8.91E-07
BP	GO:0018107	peptidyl-threonine phosphorylation	82/7778	126/18670	8.97E-08	1.52E-06	9.26E-07
BP	GO:0043200	response to amino acid	75/7778	113/18670	9.35E-08	1.58E-06	9.63E-07
BP	GO:0071230	cellular response to amino acid stimulus	50/7778	68/18670	9.53E-08	1.61E-06	9.78E-07
BP	GO:0009267	cellular response to starvation	94/7778	149/18670	9.99E-08	1.68E-06	1.02E-06
BP	GO:0019216	regulation of lipid metabolic process	223/7778	410/18670	1.04E-07	1.74E-06	1.06E-06
BP	GO:0006903	vesicle targeting	64/7778	93/18670	1.05E-07	1.75E-06	1.07E-06
BP	GO:0016575	histone deacetylation	55/7778	77/18670	1.14E-07	1.91E-06	1.16E-06
BP	GO:0007623	circadian rhythm	124/7778	208/18670	1.20E-07	2.00E-06	1.22E-06
BP	GO:0006890	retrograde vesicle-mediated transport, Golgi to endoplasmic reticulur	60/7778	86/18670	1.21E-07	2.01E-06	1.22E-06
BP	GO:0045815	positive regulation of gene expression, epigenetic	44/7778	58/18670	1.22E-07	2.01E-06	1.23E-06
BP	GO:0051090	regulation of DNA-binding transcription factor activity	233/7778	432/18670	1.34E-07	2.22E-06	1.35E-06
BP	GO:0000281	mitotic cytokinesis	52/7778	72/18670	1.41E-07	2.30E-06	1.40E-06
BP	GO:0038034	signal transduction in absence of ligand	52/7778	72/18670	1.41E-07	2.30E-06	1.40E-06
BP	GO:0097192	extrinsic apoptotic signaling pathway in absence of ligand	52/7778	72/18670	1.41E-07	2.30E-06	1.40E-06
BP	GO:1903747	regulation of establishment of protein localization to mitochondrion	52/7778	72/18670	1.41E-07	2.30E-06	1.40E-06
BP	GO:0002478	antigen processing and presentation of exogenous peptide antigen	107/7778	175/18670	1.45E-07	2.36E-06	1.44E-06
BP	GO:0034340	response to type I interferon	67/7778	99/18670	1.45E-07	2.36E-06	1.44E-06
BP	GO:0031109	microtubule polymerization or depolymerization	74/7778	112/18670	1.51E-07	2.44E-06	1.49E-06
BP	GO:0006757	ATP generation from ADP	75/7778	114/18670	1.62E-07	2.62E-06	1.60E-06
BP	GO:0072395	signal transduction involved in cell cycle checkpoint	53/7778	74/18670	1.68E-07	2.71E-06	1.65E-06
BP	GO:0006109	regulation of carbohydrate metabolic process	119/7778	199/18670	1.70E-07	2.73E-06	1.66E-06
BP	GO:0002011	morphogenesis of an epithelial sheet	42/7778	55/18670	1.71E-07	2.74E-06	1.67E-06
BP	GO:0044380	protein localization to cytoskeleton	42/7778	55/18670	1.71E-07	2.74E-06	1.67E-06
BP	GO:0007569	cell aging	76/7778	116/18670	1.74E-07	2.78E-06	1.69E-06
BP	GO:0008406	gonad development	128/7778	217/18670	1.77E-07	2.82E-06	1.72E-06
BP	GO:0048145	regulation of fibroblast proliferation	58/7778	83/18670	1.79E-07	2.85E-06	1.74E-06
BP	GO:0021915	neural tube development	99/7778	160/18670	1.84E-07	2.93E-06	1.78E-06
BP	GO:0010508	positive regulation of autophagy	78/7778	120/18670	1.98E-07	3.14E-06	1.91E-06
BP	GO:0008088	axo-dendritic transport	50/7778	69/18670	2.06E-07	3.25E-06	1.98E-06
BP	GO:0097549	chromatin organization involved in negative regulation of transcriptior	79/7778	122/18670	2.10E-07	3.31E-06	2.02E-06
BP	GO:0001838	embryonic epithelial tube formation	80/7778	124/18670	2.23E-07	3.49E-06	2.13E-06
BP	GO:0072431	signal transduction involved in mitotic G1 DNA damage checkpoint	43/7778	57/18670	2.23E-07	3.49E-06	2.13E-06
BP	GO:1902400	intracellular signal transduction involved in G1 DNA damage checkpo	43/7778	57/18670	2.23E-07	3.49E-06	2.13E-06
BP	GO:0098813	nuclear chromosome segregation	150/7778	262/18670	2.24E-07	3.49E-06	2.13E-06
BP	GO:0061614	pri-miRNA transcription by RNA polymerase II	37/7778	47/18670	2.38E-07	3.70E-06	2.25E-06
BP	GO:0006301	postreplication repair	40/7778	52/18670	2.39E-07	3.70E-06	2.25E-06
BP	GO:0045839	negative regulation of mitotic nuclear division	40/7778	52/18670	2.39E-07	3.70E-06	2.25E-06
BP	GO:0051607	defense response to virus	138/7778	238/18670	2.46E-07	3.80E-06	2.31E-06
BP	GO:0036498	IRE1-mediated unfolded protein response	47/7778	64/18670	2.46E-07	3.80E-06	2.31E-06
BP	GO:0001667	ameboidal-type cell migration	246/7778	462/18670	2.49E-07	3.82E-06	2.33E-06
BP	GO:0009612	response to mechanical stimulus	124/7778	210/18670	2.52E-07	3.87E-06	2.36E-06
BP	GO:0006096	glycolytic process	74/7778	113/18670	2.59E-07	3.95E-06	2.40E-06
BP	GO:0035821	modulation of process of other organism	74/7778	113/18670	2.59E-07	3.95E-06	2.40E-06
BP	GO:0033045	regulation of sister chromatid segregation	56/7778	80/18670	2.66E-07	4.05E-06	2.46E-06
BP	GO:0072175	epithelial tube formation	84/7778	132/18670	2.72E-07	4.14E-06	2.52E-06
BP	GO:0042769	DNA damage response, detection of DNA damage	32/7778	39/18670	2.74E-07	4.15E-06	2.53E-06
BP	GO:0010976	positive regulation of neuron projection development	159/7778	281/18670	2.75E-07	4.16E-06	2.53E-06
BP	GO:0009416	response to light stimulus	175/7778	314/18670	2.87E-07	4.32E-06	2.63E-06
BP	GO:0072401	signal transduction involved in DNA integrity checkpoint	52/7778	73/18670	2.91E-07	4.37E-06	2.66E-06
BP	GO:0072422	signal transduction involved in DNA damage checkpoint	52/7778	73/18670	2.91E-07	4.37E-06	2.66E-06
BP	GO:0140056	organelle localization by membrane tethering	103/7778	169/18670	3.07E-07	4.60E-06	2.80E-06
BP	GO:0003205	cardiac chamber development	104/7778	171/18670	3.13E-07	4.67E-06	2.85E-06
BP	GO:0097345	mitochondrial outer membrane permeabilization	41/7778	54/18670	3.16E-07	4.72E-06	2.87E-06
BP	GO:0000045	autophagosome assembly	63/7778	93/18670	3.22E-07	4.79E-06	2.92E-06
BP	GO:0045841	negative regulation of mitotic metaphase/anaphase transition	30/7778	36/18670	3.38E-07	5.02E-06	3.06E-06
BP	GO:0050769	positive regulation of neurogenesis	251/7778	474/18670	3.43E-07	5.07E-06	3.09E-06
BP	GO:0070302	regulation of stress-activated protein kinase signaling cascade	138/7778	239/18670	3.44E-07	5.07E-06	3.09E-06
BP	GO:0048144	fibroblast proliferation	58/7778	84/18670	3.44E-07	5.07E-06	3.09E-06
BP	GO:0031669	cellular response to nutrient levels	137/7778	237/18670	3.46E-07	5.09E-06	3.10E-06
BP	GO:0042594	response to starvation	114/7778	191/18670	3.51E-07	5.14E-06	3.13E-06
BP	GO:0006413	translational initiation	115/7778	193/18670	3.53E-07	5.16E-06	3.14E-06
BP	GO:0031146	SCF-dependent proteasomal ubiquitin-dependent protein catabolic p	64/7778	95/18670	3.55E-07	5.16E-06	3.14E-06
BP	GO:0060337	type I interferon signaling pathway	64/7778	95/18670	3.55E-07	5.16E-06	3.14E-06
BP	GO:0071357	cellular response to type I interferon	64/7778	95/18670	3.55E-07	5.16E-06	3.14E-06
BP	GO:0007568	aging	178/7778	321/18670	3.60E-07	5.22E-06	3.18E-06
BP	GO:0033047	regulation of mitotic sister chromatid segregation	49/7778	68/18670	3.62E-07	5.23E-06	3.18E-06
BP	GO:0070555	response to interleukin-1	122/7778	207/18670	3.62E-07	5.23E-06	3.18E-06
BP	GO:0016331	morphogenesis of embryonic epithelium	93/7778	150/18670	3.76E-07	5.40E-06	3.29E-06
BP	GO:0035567	non-canonical Wnt signaling pathway	93/7778	150/18670	3.76E-07	5.40E-06	3.29E-06
BP	GO:0008625	extrinsic apoptotic signaling pathway via death domain receptors	59/7778	86/18670	3.87E-07	5.56E-06	3.38E-06
BP	GO:0014020	primary neural tube formation	65/7778	97/18670	3.89E-07	5.56E-06	3.38E-06
BP	GO:2001022	positive regulation of response to DNA damage stimulus	65/7778	97/18670	3.89E-07	5.56E-06	3.38E-06
BP	GO:0006110	regulation of glycolytic process	54/7778	77/18670	3.94E-07	5.61E-06	3.42E-06
BP	GO:0043414	macromolecule methylation	172/7778	309/18670	4.03E-07	5.73E-06	3.49E-06
BP	GO:0006977	DNA damage response, signal transduction by p53 class mediator re	42/7778	56/18670	4.08E-07	5.79E-06	3.53E-06
BP	GO:1903828	negative regulation of cellular protein localization	73/7778	112/18670	4.11E-07	5.82E-06	3.54E-06
BP	GO:0009150	purine ribonucleotide metabolic process	215/7778	399/18670	4.43E-07	6.27E-06	3.81E-06
BP	GO:0043484	regulation of RNA splicing	85/7778	135/18670	4.55E-07	6.42E-06	3.91E-06

BP	GO:0065004	protein-DNA complex assembly	142/7778	248/18670	4.61E-07	6.49E-06	3.95E-06
BP	GO:0007178	transmembrane receptor protein serine/threonine kinase signaling pa	191/7778	349/18670	4.73E-07	6.64E-06	4.04E-06
BP	GO:0001843	neural tube closure	61/7778	90/18670	4.80E-07	6.72E-06	4.09E-06
BP	GO:0019884	antigen processing and presentation of exogenous antigen	109/7778	182/18670	4.94E-07	6.91E-06	4.21E-06
BP	GO:0019882	antigen processing and presentation	131/7778	226/18670	5.03E-07	7.01E-06	4.27E-06
BP	GO:0001738	morphogenesis of a polarized epithelium	88/7778	141/18670	5.05E-07	7.03E-06	4.28E-06
BP	GO:0051701	interaction with host	119/7778	202/18670	5.18E-07	7.19E-06	4.38E-06
BP	GO:0034248	regulation of cellular amide metabolic process	251/7778	476/18670	5.36E-07	7.43E-06	4.52E-06
BP	GO:0034101	erythrocyte homeostasis	78/7778	122/18670	5.44E-07	7.50E-06	4.57E-06
BP	GO:0046031	ADP metabolic process	78/7778	122/18670	5.44E-07	7.50E-06	4.57E-06
BP	GO:0050773	regulation of dendrite development	92/7778	149/18670	5.66E-07	7.76E-06	4.73E-06
BP	GO:1903900	regulation of viral life cycle	92/7778	149/18670	5.66E-07	7.76E-06	4.73E-06
BP	GO:0002831	regulation of response to biotic stimulus	215/7778	400/18670	5.66E-07	7.76E-06	4.73E-06
BP	GO:0001736	establishment of planar polarity	79/7778	124/18670	5.70E-07	7.78E-06	4.74E-06
BP	GO:0007164	establishment of tissue polarity	79/7778	124/18670	5.70E-07	7.78E-06	4.74E-06
BP	GO:0045666	positive regulation of neuron differentiation	201/7778	371/18670	6.28E-07	8.55E-06	5.21E-06
BP	GO:1905037	autophagosome organization	64/7778	96/18670	6.33E-07	8.60E-06	5.23E-06
BP	GO:0051784	negative regulation of nuclear division	44/7778	60/18670	6.38E-07	8.65E-06	5.26E-06
BP	GO:0048568	embryonic organ development	228/7778	428/18670	6.46E-07	8.75E-06	5.32E-06
BP	GO:0032872	regulation of stress-activated MAPK cascade	136/7778	237/18670	6.75E-07	9.12E-06	5.55E-06
BP	GO:0045070	positive regulation of viral genome replication	29/7778	35/18670	6.81E-07	9.16E-06	5.58E-06
BP	GO:1901030	positive regulation of mitochondrial outer membrane permeabilizatior	29/7778	35/18670	6.81E-07	9.16E-06	5.58E-06
BP	GO:0006626	protein targeting to mitochondrion	65/7778	98/18670	6.86E-07	9.21E-06	5.61E-06
BP	GO:0071347	cellular response to interleukin-1	107/7778	179/18670	7.14E-07	9.57E-06	5.82E-06
BP	GO:0010810	regulation of cell-substrate adhesion	125/7778	215/18670	7.28E-07	9.73E-06	5.93E-06
BP	GO:0071229	cellular response to acid chemical	122/7778	209/18670	7.36E-07	9.82E-06	5.98E-06
BP	GO:0042306	regulation of protein import into nucleus	41/7778	55/18670	7.41E-07	9.87E-06	6.01E-06
BP	GO:0051865	protein autoubiquitination	49/7778	69/18670	7.43E-07	9.87E-06	6.01E-06
BP	GO:0050678	regulation of epithelial cell proliferation	204/7778	378/18670	7.49E-07	9.93E-06	6.04E-06
BP	GO:0007030	Golgi organization	88/7778	142/18670	7.87E-07	1.04E-05	6.34E-06
BP	GO:0071786	endoplasmic reticulum tubular network organization	16/7778	16/18670	8.16E-07	1.08E-05	6.56E-06
BP	GO:0014065	phosphatidylinositol 3-kinase signaling	91/7778	148/18670	8.48E-07	1.12E-05	6.80E-06
BP	GO:0042771	intrinsic apoptotic signaling pathway in response to DNA damage by	35/7778	45/18670	8.60E-07	1.13E-05	6.88E-06
BP	GO:0060606	tube closure	61/7778	91/18670	8.65E-07	1.13E-05	6.91E-06
BP	GO:0097150	neuronal stem cell population maintenance	21/7778	23/18670	9.36E-07	1.22E-05	7.45E-06
BP	GO:2000637	positive regulation of gene silencing by miRNA	21/7778	23/18670	9.36E-07	1.22E-05	7.45E-06
BP	GO:0046661	male sex differentiation	97/7778	160/18670	9.48E-07	1.24E-05	7.53E-06
BP	GO:0000302	response to reactive oxygen species	133/7778	232/18670	9.60E-07	1.25E-05	7.61E-06
BP	GO:0071156	regulation of cell cycle arrest	70/7778	108/18670	9.67E-07	1.25E-05	7.63E-06
BP	GO:2000278	regulation of DNA biosynthetic process	70/7778	108/18670	9.67E-07	1.25E-05	7.63E-06
BP	GO:0007254	JNK cascade	124/7778	214/18670	1.03E-06	1.33E-05	8.10E-06
BP	GO:1905330	regulation of morphogenesis of an epithelium	107/7778	180/18670	1.04E-06	1.35E-05	8.20E-06
BP	GO:1902100	negative regulation of metaphase/anaphase transition of cell cycle	30/7778	37/18670	1.07E-06	1.38E-05	8.37E-06
BP	GO:1904358	positive regulation of telomere maintenance via telomere lengthening	30/7778	37/18670	1.07E-06	1.38E-05	8.37E-06
BP	GO:2000142	regulation of DNA-templated transcription, initiation	30/7778	37/18670	1.07E-06	1.38E-05	8.37E-06
BP	GO:0006353	DNA-templated transcription, termination	52/7778	75/18670	1.13E-06	1.44E-05	8.76E-06
BP	GO:0033143	regulation of intracellular steroid hormone receptor signaling pathway	52/7778	75/18670	1.13E-06	1.44E-05	8.76E-06
BP	GO:0072413	signal transduction involved in mitotic cell cycle checkpoint	43/7778	59/18670	1.13E-06	1.44E-05	8.76E-06
BP	GO:1902402	signal transduction involved in mitotic DNA damage checkpoint	43/7778	59/18670	1.13E-06	1.44E-05	8.76E-06
BP	GO:1902403	signal transduction involved in mitotic DNA integrity checkpoint	43/7778	59/18670	1.13E-06	1.44E-05	8.76E-06
BP	GO:0030218	erythrocyte differentiation	73/7778	114/18670	1.14E-06	1.44E-05	8.78E-06
BP	GO:0070849	response to epidermal growth factor	36/7778	47/18670	1.16E-06	1.47E-05	8.93E-06
BP	GO:0033048	negative regulation of mitotic sister chromatid segregation	33/7778	42/18670	1.18E-06	1.49E-05	9.05E-06
BP	GO:0008286	insulin receptor signaling pathway	87/7778	141/18670	1.19E-06	1.49E-05	9.10E-06
BP	GO:0007094	mitotic spindle assembly checkpoint	28/7778	34/18670	1.36E-06	1.71E-05	1.04E-05
BP	GO:0031577	spindle checkpoint	28/7778	34/18670	1.36E-06	1.71E-05	1.04E-05
BP	GO:0071173	spindle assembly checkpoint	28/7778	34/18670	1.36E-06	1.71E-05	1.04E-05
BP	GO:0071174	mitotic spindle checkpoint	28/7778	34/18670	1.36E-06	1.71E-05	1.04E-05
BP	GO:0007160	cell-matrix adhesion	129/7778	225/18670	1.38E-06	1.72E-05	1.05E-05
BP	GO:0071260	cellular response to mechanical stimulus	54/7778	79/18670	1.43E-06	1.78E-05	1.09E-05
BP	GO:0034341	response to interferon-gamma	116/7778	199/18670	1.50E-06	1.86E-05	1.13E-05
BP	GO:0048736	appendage development	106/7778	179/18670	1.51E-06	1.86E-05	1.13E-05
BP	GO:0060173	limb development	106/7778	179/18670	1.51E-06	1.86E-05	1.13E-05
BP	GO:0002221	pattern recognition receptor signaling pathway	115/7778	197/18670	1.51E-06	1.86E-05	1.13E-05
BP	GO:0048015	phosphatidylinositol-mediated signaling	107/7778	181/18670	1.51E-06	1.86E-05	1.13E-05
BP	GO:0050770	regulation of axonogenesis	108/7778	183/18670	1.51E-06	1.86E-05	1.13E-05
BP	GO:0061912	selective autophagy	37/7778	49/18670	1.52E-06	1.86E-05	1.13E-05
BP	GO:0048002	antigen processing and presentation of peptide antigen	111/7778	189/18670	1.52E-06	1.86E-05	1.13E-05
BP	GO:1902229	regulation of intrinsic apoptotic signaling pathway in response to DN/	31/7778	39/18670	1.59E-06	1.94E-05	1.18E-05
BP	GO:0032204	regulation of telomere maintenance	55/7778	81/18670	1.59E-06	1.95E-05	1.18E-05
BP	GO:0006165	nucleoside diphosphate phosphorylation	82/7778	132/18670	1.61E-06	1.95E-05	1.19E-05
BP	GO:0042177	negative regulation of protein catabolic process	82/7778	132/18670	1.61E-06	1.95E-05	1.19E-05
BP	GO:0006163	purine nucleotide metabolic process	228/7778	432/18670	1.61E-06	1.95E-05	1.19E-05
BP	GO:0033046	negative regulation of sister chromatid segregation	34/7778	44/18670	1.62E-06	1.96E-05	1.19E-05
BP	GO:1901028	regulation of mitochondrial outer membrane permeabilization involve	34/7778	44/18670	1.62E-06	1.96E-05	1.19E-05
BP	GO:0000724	double-strand break repair via homologous recombination	83/7778	134/18670	1.65E-06	1.99E-05	1.21E-05
BP	GO:0009135	purine nucleoside diphosphate metabolic process	83/7778	134/18670	1.65E-06	1.99E-05	1.21E-05
BP	GO:0009179	purine ribonucleoside diphosphate metabolic process	83/7778	134/18670	1.65E-06	1.99E-05	1.21E-05
BP	GO:0090307	mitotic spindle assembly	41/7778	56/18670	1.65E-06	1.99E-05	1.21E-05
BP	GO:1902230	negative regulation of intrinsic apoptotic signaling pathway in respon:	26/7778	31/18670	1.68E-06	2.01E-05	1.22E-05
BP	GO:1902036	regulation of hematopoietic stem cell differentiation	50/7778	72/18670	1.68E-06	2.02E-05	1.23E-05
BP	GO:0009185	ribonucleoside diphosphate metabolic process	84/7778	136/18670	1.69E-06	2.03E-05	1.23E-05
BP	GO:0060485	mesenchyme development	155/7778	279/18670	1.72E-06	2.05E-05	1.25E-05
BP	GO:0034329	cell junction assembly	217/7778	409/18670	1.75E-06	2.08E-05	1.26E-05
BP	GO:0030217	T cell differentiation	136/7778	240/18670	1.75E-06	2.08E-05	1.26E-05
BP	GO:0060218	hematopoietic stem cell differentiation	56/7778	83/18670	1.76E-06	2.08E-05	1.27E-05
BP	GO:1900542	regulation of purine nucleotide metabolic process	73/7778	115/18670	1.85E-06	2.19E-05	1.33E-05
BP	GO:0002224	toll-like receptor signaling pathway	89/7778	146/18670	1.88E-06	2.23E-05	1.36E-05
BP	GO:0072665	protein localization to vacuole	46/7778	65/18670	1.89E-06	2.24E-05	1.36E-05
BP	GO:0072384	organelle transport along microtubule	57/7778	85/18670	1.93E-06	2.26E-05	1.38E-05
BP	GO:0006342	chromatin silencing	38/7778	51/18670	1.93E-06	2.26E-05	1.38E-05
BP	GO:0030071	regulation of mitotic metaphase/anaphase transition	38/7778	51/18670	1.93E-06	2.26E-05	1.38E-05

BP	GO:0032206	positive regulation of telomere maintenance	38/7778	51/18670	1.93E-06	2.26E-05	1.38E-05
BP	GO:0048754	branching morphogenesis of an epithelial tube	91/7778	150/18670	1.95E-06	2.28E-05	1.39E-05
BP	GO:1904589	regulation of protein import	42/7778	58/18670	2.00E-06	2.34E-05	1.42E-05
BP	GO:0001952	regulation of cell-matrix adhesion	75/7778	119/18670	2.01E-06	2.34E-05	1.42E-05
BP	GO:0003007	heart morphogenesis	145/7778	259/18670	2.06E-06	2.39E-05	1.46E-05
BP	GO:0051056	regulation of small GTPase mediated signal transduction	183/7778	338/18670	2.07E-06	2.41E-05	1.47E-05
BP	GO:1902806	regulation of cell cycle G1/S phase transition	117/7778	202/18670	2.11E-06	2.44E-05	1.49E-05
BP	GO:0016052	carbohydrate catabolic process	115/7778	198/18670	2.13E-06	2.46E-05	1.50E-05
BP	GO:0006900	vesicle budding from membrane	66/7778	102/18670	2.14E-06	2.47E-05	1.50E-05
BP	GO:0006984	ER-nucleus signaling pathway	35/7778	46/18670	2.15E-06	2.48E-05	1.51E-05
BP	GO:0090090	negative regulation of canonical Wnt signaling pathway	105/7778	178/18670	2.17E-06	2.49E-05	1.51E-05
BP	GO:0048017	inositol lipid-mediated signaling	108/7778	184/18670	2.17E-06	2.49E-05	1.51E-05
BP	GO:2000045	regulation of G1/S transition of mitotic cell cycle	108/7778	184/18670	2.17E-06	2.49E-05	1.51E-05
BP	GO:0045926	negative regulation of growth	140/7778	249/18670	2.23E-06	2.55E-05	1.55E-05
BP	GO:1902903	regulation of supramolecular fiber organization	190/7778	353/18670	2.23E-06	2.55E-05	1.55E-05
BP	GO:1902893	regulation of pri-miRNA transcription by RNA polymerase II	32/7778	41/18670	2.24E-06	2.55E-05	1.55E-05
BP	GO:0071887	leukocyte apoptotic process	67/7778	104/18670	2.26E-06	2.57E-05	1.57E-05
BP	GO:0060021	roof of mouth development	59/7778	89/18670	2.28E-06	2.59E-05	1.58E-05
BP	GO:0007041	lysosomal transport	68/7778	106/18670	2.38E-06	2.68E-05	1.63E-05
BP	GO:1901989	positive regulation of cell cycle phase transition	68/7778	106/18670	2.38E-06	2.68E-05	1.63E-05
BP	GO:0043966	histone H3 acetylation	43/7778	60/18670	2.39E-06	2.68E-05	1.63E-05
BP	GO:0051893	regulation of focal adhesion assembly	43/7778	60/18670	2.39E-06	2.68E-05	1.63E-05
BP	GO:0090109	regulation of cell-substrate junction assembly	43/7778	60/18670	2.39E-06	2.68E-05	1.63E-05
BP	GO:0150116	regulation of cell-substrate junction organization	43/7778	60/18670	2.39E-06	2.68E-05	1.63E-05
BP	GO:0022613	ribonucleoprotein complex biogenesis	244/7778	468/18670	2.39E-06	2.68E-05	1.63E-05
BP	GO:0051402	neuron apoptotic process	135/7778	239/18670	2.41E-06	2.69E-05	1.64E-05
BP	GO:0048638	regulation of developmental growth	187/7778	347/18670	2.41E-06	2.70E-05	1.64E-05
BP	GO:0043122	regulation of I-kappaB kinase/NF-kappaB signaling	134/7778	237/18670	2.44E-06	2.72E-05	1.66E-05
BP	GO:0000725	recombinational repair	83/7778	135/18670	2.54E-06	2.83E-05	1.73E-05
BP	GO:0090175	regulation of establishment of planar polarity	70/7778	110/18670	2.62E-06	2.92E-05	1.78E-05
BP	GO:0032092	positive regulation of protein binding	61/7778	93/18670	2.64E-06	2.93E-05	1.78E-05
BP	GO:0046546	development of primary male sexual characteristics	85/7778	139/18670	2.65E-06	2.94E-05	1.79E-05
BP	GO:0001837	epithelial to mesenchymal transition	86/7778	141/18670	2.70E-06	2.99E-05	1.82E-05
BP	GO:1901797	negative regulation of signal transduction by p53 class mediator	27/7778	33/18670	2.72E-06	3.00E-05	1.83E-05
BP	GO:0043406	positive regulation of MAP kinase activity	144/7778	258/18670	2.80E-06	3.09E-05	1.88E-05
BP	GO:1902686	mitochondrial outer membrane permeabilization involved in programm	44/7778	62/18670	2.80E-06	3.09E-05	1.88E-05
BP	GO:0071236	cellular response to antibiotic	89/7778	147/18670	2.83E-06	3.11E-05	1.90E-05
BP	GO:0010833	telomere maintenance via telomere lengthening	55/7778	82/18670	2.88E-06	3.15E-05	1.92E-05
BP	GO:0071158	positive regulation of cell cycle arrest	55/7778	82/18670	2.88E-06	3.15E-05	1.92E-05
BP	GO:0071364	cellular response to epidermal growth factor stimulus	33/7778	43/18670	3.03E-06	3.31E-05	2.02E-05
BP	GO:0046825	regulation of protein export from nucleus	30/7778	38/18670	3.05E-06	3.33E-05	2.03E-05
BP	GO:1905898	positive regulation of response to endoplasmic reticulum stress	30/7778	38/18670	3.05E-06	3.33E-05	2.03E-05
BP	GO:0006479	protein methylation	105/7778	179/18670	3.11E-06	3.37E-05	2.05E-05
BP	GO:0008213	protein alkylation	105/7778	179/18670	3.11E-06	3.37E-05	2.05E-05
BP	GO:0035690	cellular response to drug	227/7778	433/18670	3.19E-06	3.46E-05	2.11E-05
BP	GO:0035794	positive regulation of mitochondrial membrane permeability	45/7778	64/18670	3.25E-06	3.52E-05	2.14E-05
BP	GO:1905477	positive regulation of protein localization to membrane	76/7778	122/18670	3.28E-06	3.54E-05	2.16E-05
BP	GO:0007098	centrosome cycle	77/7778	124/18670	3.37E-06	3.63E-05	2.21E-05
BP	GO:0048284	organelle fusion	77/7778	124/18670	3.37E-06	3.63E-05	2.21E-05
BP	GO:0051101	regulation of DNA binding	77/7778	124/18670	3.37E-06	3.63E-05	2.21E-05
BP	GO:0001844	protein insertion into mitochondrial membrane involved in apoptotic s	25/7778	30/18670	3.40E-06	3.65E-05	2.22E-05
BP	GO:0072698	protein localization to microtubule cytoskeleton	37/7778	50/18670	3.49E-06	3.74E-05	2.28E-05
BP	GO:0060760	positive regulation of response to cytokine stimulus	41/7778	57/18670	3.52E-06	3.76E-05	2.29E-05
BP	GO:0003281	ventricular septum development	51/7778	75/18670	3.55E-06	3.79E-05	2.31E-05
BP	GO:0009791	post-embryonic development	58/7778	88/18670	3.67E-06	3.91E-05	2.38E-05
BP	GO:0045995	regulation of embryonic development	81/7778	132/18670	3.72E-06	3.96E-05	2.41E-05
BP	GO:0048762	mesenchymal cell differentiation	125/7778	220/18670	3.74E-06	3.98E-05	2.42E-05
BP	GO:0007006	mitochondrial membrane organization	82/7778	134/18670	3.80E-06	4.03E-05	2.45E-05
BP	GO:0046939	nucleotide phosphorylation	82/7778	134/18670	3.80E-06	4.03E-05	2.45E-05
BP	GO:0032481	positive regulation of type I interferon production	52/7778	77/18670	3.92E-06	4.14E-05	2.52E-05
BP	GO:0008584	male gonad development	84/7778	138/18670	3.94E-06	4.16E-05	2.53E-05
BP	GO:0030178	negative regulation of Wnt signaling pathway	120/7778	210/18670	3.97E-06	4.17E-05	2.54E-05
BP	GO:0051985	negative regulation of chromosome segregation	34/7778	45/18670	3.97E-06	4.17E-05	2.54E-05
BP	GO:0072073	kidney epithelium development	85/7778	140/18670	4.00E-06	4.21E-05	2.56E-05
BP	GO:0050679	positive regulation of epithelial cell proliferation	118/7778	206/18670	4.05E-06	4.25E-05	2.59E-05
BP	GO:0006006	glucose metabolic process	117/7778	204/18670	4.09E-06	4.28E-05	2.61E-05
BP	GO:0060541	respiratory system development	114/7778	198/18670	4.19E-06	4.39E-05	2.67E-05
BP	GO:0006623	protein targeting to vacuole	31/7778	40/18670	4.23E-06	4.42E-05	2.69E-05
BP	GO:1901888	regulation of cell junction assembly	111/7778	192/18670	4.29E-06	4.46E-05	2.71E-05
BP	GO:0006998	nuclear envelope organization	38/7778	52/18670	4.29E-06	4.46E-05	2.71E-05
BP	GO:0051438	regulation of ubiquitin-protein transferase activity	38/7778	52/18670	4.29E-06	4.46E-05	2.71E-05
BP	GO:0030308	negative regulation of cell growth	108/7778	186/18670	4.36E-06	4.53E-05	2.76E-05
BP	GO:0031667	response to nutrient levels	257/7778	499/18670	4.44E-06	4.59E-05	2.79E-05
BP	GO:0034614	cellular response to reactive oxygen species	99/7778	168/18670	4.45E-06	4.59E-05	2.79E-05
BP	GO:0051302	regulation of cell division	99/7778	168/18670	4.45E-06	4.59E-05	2.79E-05
BP	GO:0033962	cytoplasmic mRNA processing body assembly	19/7778	21/18670	4.52E-06	4.66E-05	2.83E-05
BP	GO:0060148	positive regulation of posttranscriptional gene silencing	21/7778	24/18670	4.52E-06	4.66E-05	2.83E-05
BP	GO:0060249	anatomical structure homeostasis	229/7778	439/18670	4.60E-06	4.73E-05	2.88E-05
BP	GO:0006140	regulation of nucleotide metabolic process	73/7778	117/18670	4.68E-06	4.77E-05	2.91E-05
BP	GO:0031145	anaphase-promoting complex-dependent catabolic process	54/7778	81/18670	4.68E-06	4.77E-05	2.91E-05
BP	GO:0032436	positive regulation of proteasomal ubiquitin-dependent protein catabo	54/7778	81/18670	4.68E-06	4.77E-05	2.91E-05
BP	GO:0048041	focal adhesion assembly	54/7778	81/18670	4.68E-06	4.77E-05	2.91E-05
BP	GO:0110110	positive regulation of animal organ morphogenesis	54/7778	81/18670	4.68E-06	4.77E-05	2.91E-05
BP	GO:0006999	nuclear pore organization	14/7778	14/18670	4.71E-06	4.79E-05	2.92E-05
BP	GO:0002474	antigen processing and presentation of peptide antigen via MHC clas	62/7778	96/18670	4.73E-06	4.81E-05	2.93E-05
BP	GO:0043547	positive regulation of GTPase activity	213/7778	405/18670	4.78E-06	4.85E-05	2.95E-05
BP	GO:0090151	establishment of protein localization to mitochondrial membrane	35/7778	47/18670	5.04E-06	5.11E-05	3.11E-05
BP	GO:0009952	anterior/posterior pattern specification	124/7778	219/18670	5.17E-06	5.22E-05	3.18E-05
BP	GO:0007091	metaphase/anaphase transition of mitotic cell cycle	39/7778	54/18670	5.17E-06	5.22E-05	3.18E-05
BP	GO:0000375	RNA splicing, via transesterification reactions	202/7778	382/18670	5.23E-06	5.28E-05	3.21E-05
BP	GO:0034446	substrate adhesion-dependent cell spreading	64/7778	100/18670	5.25E-06	5.29E-05	3.22E-05
BP	GO:0090114	COPII-coated vesicle budding	49/7778	72/18670	5.30E-06	5.32E-05	3.24E-05

BP	GO:0008608	attachment of spindle microtubules to kinetochore	26/7778	32/18670	5.38E-06	5.39E-05	3.28E-05
BP	GO:1901976	regulation of cell cycle checkpoint	26/7778	32/18670	5.38E-06	5.39E-05	3.28E-05
BP	GO:0050772	positive regulation of axonogenesis	56/7778	85/18670	5.47E-06	5.47E-05	3.33E-05
BP	GO:0031023	microtubule organizing center organization	81/7778	133/18670	5.65E-06	5.64E-05	3.44E-05
BP	GO:0060537	muscle tissue development	214/7778	408/18670	5.77E-06	5.76E-05	3.50E-05
BP	GO:0000289	nuclear-transcribed mRNA poly(A) tail shortening	29/7778	37/18670	5.84E-06	5.81E-05	3.54E-05
BP	GO:0048199	vesicle targeting, to, from or within Golgi	50/7778	74/18670	5.85E-06	5.82E-05	3.54E-05
BP	GO:0001892	embryonic placenta development	57/7778	87/18670	5.87E-06	5.82E-05	3.55E-05
BP	GO:0035304	regulation of protein dephosphorylation	84/7778	139/18670	5.91E-06	5.85E-05	3.56E-05
BP	GO:0072521	purine-containing compound metabolic process	240/7778	464/18670	6.12E-06	6.06E-05	3.69E-05
BP	GO:0010332	response to gamma radiation	40/7778	56/18670	6.13E-06	6.06E-05	3.69E-05
BP	GO:0060071	Wnt signaling pathway, planar cell polarity pathway	68/7778	108/18670	6.23E-06	6.15E-05	3.74E-05
BP	GO:0071349	cellular response to interleukin-12	36/7778	49/18670	6.26E-06	6.16E-05	3.75E-05
BP	GO:0097306	cellular response to alcohol	58/7778	89/18670	6.26E-06	6.16E-05	3.75E-05
BP	GO:0007029	endoplasmic reticulum organization	45/7778	65/18670	6.31E-06	6.18E-05	3.76E-05
BP	GO:0045739	positive regulation of DNA repair	45/7778	65/18670	6.31E-06	6.18E-05	3.76E-05
BP	GO:0010921	regulation of phosphatase activity	102/7778	175/18670	6.31E-06	6.18E-05	3.76E-05
BP	GO:0042752	regulation of circadian rhythm	71/7778	114/18670	6.89E-06	6.74E-05	4.10E-05
BP	GO:0000377	RNA splicing, via transesterification reactions with bulged adenosine	200/7778	379/18670	6.93E-06	6.75E-05	4.11E-05
BP	GO:0000398	mRNA splicing, via spliceosome	200/7778	379/18670	6.93E-06	6.75E-05	4.11E-05
BP	GO:0001101	response to acid chemical	183/7778	343/18670	7.01E-06	6.82E-05	4.15E-05
BP	GO:0098930	axonal transport	41/7778	58/18670	7.16E-06	6.94E-05	4.23E-05
BP	GO:1902041	regulation of extrinsic apoptotic signaling pathway via death domain	41/7778	58/18670	7.16E-06	6.94E-05	4.23E-05
BP	GO:1903749	positive regulation of establishment of protein localization to mitocho	41/7778	58/18670	7.16E-06	6.94E-05	4.23E-05
BP	GO:2000060	positive regulation of ubiquitin-dependent protein catabolic process	61/7778	95/18670	7.43E-06	7.18E-05	4.37E-05
BP	GO:0097061	dendritic spine organization	53/7778	80/18670	7.58E-06	7.32E-05	4.46E-05
BP	GO:0007566	embryo implantation	37/7778	51/18670	7.60E-06	7.32E-05	4.46E-05
BP	GO:0048146	positive regulation of fibroblast proliferation	37/7778	51/18670	7.60E-06	7.32E-05	4.46E-05
BP	GO:0032212	positive regulation of telomere maintenance via telomerase	27/7778	34/18670	7.94E-06	7.61E-05	4.63E-05
BP	GO:0043516	regulation of DNA damage response, signal transduction by p53 clas	27/7778	34/18670	7.94E-06	7.61E-05	4.63E-05
BP	GO:2000816	negative regulation of mitotic sister chromatid separation	30/7778	39/18670	7.94E-06	7.61E-05	4.63E-05
BP	GO:0051851	modulation by host of symbiont process	42/7778	60/18670	8.26E-06	7.89E-05	4.81E-05
BP	GO:1902110	positive regulation of mitochondrial membrane permeability involved	42/7778	60/18670	8.26E-06	7.89E-05	4.81E-05
BP	GO:0042119	neutrophil activation	255/7778	498/18670	8.44E-06	8.04E-05	4.90E-05
BP	GO:0060759	regulation of response to cytokine stimulus	109/7778	190/18670	8.44E-06	8.04E-05	4.90E-05
BP	GO:0009755	hormone-mediated signaling pathway	130/7778	233/18670	8.54E-06	8.13E-05	4.95E-05
BP	GO:0043409	negative regulation of MAPK cascade	106/7778	184/18670	8.66E-06	8.23E-05	5.01E-05
BP	GO:0008654	phospholipid biosynthetic process	143/7778	260/18670	8.77E-06	8.32E-05	5.06E-05
BP	GO:0071346	cellular response to interferon-gamma	104/7778	180/18670	8.79E-06	8.33E-05	5.07E-05
BP	GO:0038127	ERBB signaling pathway	85/7778	142/18670	8.84E-06	8.36E-05	5.09E-05
BP	GO:1902099	regulation of metaphase/anaphase transition of cell cycle	38/7778	53/18670	9.06E-06	8.56E-05	5.21E-05
BP	GO:0009132	nucleoside diphosphate metabolic process	91/7778	154/18670	9.11E-06	8.58E-05	5.22E-05
BP	GO:0032480	negative regulation of type I interferon production	34/7778	46/18670	9.11E-06	8.58E-05	5.22E-05
BP	GO:1904356	regulation of telomere maintenance via telomere lengthening	43/7778	62/18670	9.41E-06	8.85E-05	5.39E-05
BP	GO:0043044	ATP-dependent chromatin remodeling	57/7778	88/18670	9.92E-06	9.29E-05	5.66E-05
BP	GO:0106027	neuron projection organization	57/7778	88/18670	9.92E-06	9.29E-05	5.66E-05
BP	GO:0043281	regulation of cysteine-type endopeptidase activity involved in apopto	121/7778	215/18670	9.92E-06	9.29E-05	5.66E-05
BP	GO:0032535	regulation of cellular component size	195/7778	370/18670	9.97E-06	9.32E-05	5.67E-05
BP	GO:0002446	neutrophil mediated immunity	255/7778	499/18670	1.02E-05	9.51E-05	5.79E-05
BP	GO:0019318	hexose metabolic process	135/7778	244/18670	1.03E-05	9.63E-05	5.86E-05
BP	GO:0019985	translesion synthesis	31/7778	41/18670	1.04E-05	9.68E-05	5.90E-05
BP	GO:0045185	maintenance of protein location	58/7778	90/18670	1.05E-05	9.75E-05	5.93E-05
BP	GO:1902253	regulation of intrinsic apoptotic signaling pathway by p53 class medi	25/7778	31/18670	1.06E-05	9.82E-05	5.98E-05
BP	GO:0006360	transcription by RNA polymerase I	44/7778	64/18670	1.06E-05	9.83E-05	5.98E-05
BP	GO:0046578	regulation of Ras protein signal transduction	132/7778	238/18670	1.10E-05	0.0001014	6.17E-05
BP	GO:0061014	positive regulation of mRNA catabolic process	35/7778	48/18670	1.12E-05	0.0001031	6.28E-05
BP	GO:1905710	positive regulation of membrane permeability	45/7778	66/18670	1.18E-05	0.0001092	6.65E-05
BP	GO:0014706	striated muscle tissue development	204/7778	390/18670	1.19E-05	0.0001093	6.66E-05
BP	GO:0099111	microtubule-based transport	107/7778	187/18670	1.19E-05	0.0001095	6.67E-05
BP	GO:0007369	gastrulation	106/7778	185/18670	1.20E-05	0.0001102	6.71E-05
BP	GO:0046328	regulation of JNK cascade	106/7778	185/18670	1.20E-05	0.0001102	6.71E-05
BP	GO:0006333	chromatin assembly or disassembly	127/7778	228/18670	1.20E-05	0.0001102	6.71E-05
BP	GO:0032970	regulation of actin filament-based process	203/7778	388/18670	1.22E-05	0.0001118	6.81E-05
BP	GO:0008361	regulation of cell size	103/7778	179/18670	1.24E-05	0.0001129	6.87E-05
BP	GO:0072163	mesonephric epithelium development	62/7778	98/18670	1.26E-05	0.0001152	7.01E-05
BP	GO:0072164	mesonephric tubule development	62/7778	98/18670	1.26E-05	0.0001152	7.01E-05
BP	GO:0010634	positive regulation of epithelial cell migration	99/7778	171/18670	1.27E-05	0.0001157	7.04E-05
BP	GO:0042254	ribosome biogenesis	160/7778	297/18670	1.28E-05	0.0001161	7.07E-05
BP	GO:0106106	cold-induced thermogenesis	85/7778	143/18670	1.29E-05	0.0001172	7.13E-05
BP	GO:0120161	regulation of cold-induced thermogenesis	85/7778	143/18670	1.29E-05	0.0001172	7.13E-05
BP	GO:0071901	negative regulation of protein serine/threonine kinase activity	86/7778	145/18670	1.30E-05	0.0001175	7.15E-05
BP	GO:0016239	positive regulation of macroautophagy	46/7778	68/18670	1.31E-05	0.0001183	7.20E-05
BP	GO:0036503	ERAD pathway	63/7778	100/18670	1.31E-05	0.0001185	7.22E-05
BP	GO:0008631	intrinsic apoptotic signaling pathway in response to oxidative stress	32/7778	43/18670	1.32E-05	0.000119	7.25E-05
BP	GO:0006323	DNA packaging	136/7778	247/18670	1.33E-05	0.0001199	7.30E-05
BP	GO:0010718	positive regulation of epithelial to mesenchymal transition	36/7778	50/18670	1.34E-05	0.0001199	7.30E-05
BP	GO:0046854	phosphatidylinositol phosphorylation	36/7778	50/18670	1.34E-05	0.0001199	7.30E-05
BP	GO:0070671	response to interleukin-12	36/7778	50/18670	1.34E-05	0.0001199	7.30E-05
BP	GO:0001836	release of cytochrome c from mitochondria	41/7778	59/18670	1.40E-05	0.0001256	7.65E-05
BP	GO:0042110	T cell activation	238/7778	464/18670	1.43E-05	0.0001275	7.76E-05
BP	GO:0007004	telomere maintenance via telomerase	47/7778	70/18670	1.44E-05	0.0001282	7.81E-05
BP	GO:2000116	regulation of cysteine-type endopeptidase activity	132/7778	239/18670	1.45E-05	0.0001291	7.86E-05
BP	GO:0002573	myeloid leukocyte differentiation	115/7778	204/18670	1.47E-05	0.0001307	7.96E-05
BP	GO:0002756	MyD88-independent toll-like receptor signaling pathway	26/7778	33/18670	1.53E-05	0.0001352	8.23E-05
BP	GO:0033028	myeloid cell apoptotic process	26/7778	33/18670	1.53E-05	0.0001352	8.23E-05
BP	GO:2000758	positive regulation of peptidyl-lysine acetylation	26/7778	33/18670	1.53E-05	0.0001352	8.23E-05
BP	GO:0022407	regulation of cell-cell adhesion	209/7778	402/18670	1.56E-05	0.0001377	8.39E-05
BP	GO:0061180	mammary gland epithelium development	48/7778	72/18670	1.57E-05	0.0001384	8.43E-05
BP	GO:0099518	vesicle cytoskeletal trafficking	37/7778	52/18670	1.58E-05	0.0001392	8.47E-05
BP	GO:0003170	heart valve development	42/7778	61/18670	1.58E-05	0.0001396	8.50E-05
BP	GO:0046390	ribose phosphate biosynthetic process	109/7778	192/18670	1.60E-05	0.0001405	8.55E-05
BP	GO:0016574	histone ubiquitination	33/7778	45/18670	1.63E-05	0.0001433	8.73E-05

BP	GO:0051204	protein insertion into mitochondrial membrane	33/7778	45/18670	1.63E-05	0.0001433	8.73E-05
BP	GO:0006661	phosphatidylinositol biosynthetic process	71/7778	116/18670	1.64E-05	0.0001437	8.75E-05
BP	GO:0032259	methylation	192/7778	366/18670	1.68E-05	0.0001473	8.97E-05
BP	GO:0017015	regulation of transforming growth factor beta receptor signaling pathw	73/7778	120/18670	1.70E-05	0.0001485	9.04E-05
BP	GO:1900101	regulation of endoplasmic reticulum unfolded protein response	21/7778	25/18670	1.71E-05	0.0001492	9.09E-05
BP	GO:0048814	regulation of dendrite morphogenesis	58/7778	91/18670	1.72E-05	0.0001497	9.11E-05
BP	GO:0060333	interferon-gamma-mediated signaling pathway	58/7778	91/18670	1.72E-05	0.0001497	9.11E-05
BP	GO:1901992	positive regulation of mitotic cell cycle phase transition	58/7778	91/18670	1.72E-05	0.0001497	9.11E-05
BP	GO:1903844	regulation of cellular response to transforming growth factor beta stin	74/7778	122/18670	1.73E-05	0.0001498	9.12E-05
BP	GO:0006417	regulation of translation	219/7778	424/18670	1.73E-05	0.0001498	9.12E-05
BP	GO:0006310	DNA recombination	157/7778	292/18670	1.76E-05	0.0001527	9.30E-05
BP	GO:0003231	cardiac ventricle development	78/7778	130/18670	1.81E-05	0.0001563	9.52E-05
BP	GO:0000422	autophagy of mitochondrion	50/7778	76/18670	1.82E-05	0.0001572	9.57E-05
BP	GO:0046902	regulation of mitochondrial membrane permeability	50/7778	76/18670	1.82E-05	0.0001572	9.57E-05
BP	GO:0061726	mitochondrion disassembly	50/7778	76/18670	1.82E-05	0.0001572	9.57E-05
BP	GO:0032210	regulation of telomere maintenance via telomerase	38/7778	54/18670	1.83E-05	0.0001574	9.58E-05
BP	GO:0032956	regulation of actin cytoskeleton organization	181/7778	343/18670	1.85E-05	0.0001591	9.68E-05
BP	GO:0072006	nephron development	84/7778	142/18670	1.87E-05	0.0001604	9.76E-05
BP	GO:0035107	appendage morphogenesis	87/7778	148/18670	1.88E-05	0.0001605	9.77E-05
BP	GO:0035108	limb morphogenesis	87/7778	148/18670	1.88E-05	0.0001605	9.77E-05
BP	GO:1905819	negative regulation of chromosome separation	30/7778	40/18670	1.91E-05	0.000163	9.92E-05
BP	GO:0045785	positive regulation of cell adhesion	209/7778	403/18670	1.91E-05	0.000163	9.93E-05
BP	GO:0001657	ureteric bud development	61/7778	97/18670	1.94E-05	0.0001654	0.000101
BP	GO:0060996	dendritic spine development	61/7778	97/18670	1.94E-05	0.0001654	0.000101
BP	GO:0035722	interleukin-12-mediated signaling pathway	34/7778	47/18670	1.97E-05	0.0001675	0.000102
BP	GO:2001239	regulation of extrinsic apoptotic signaling pathway in absence of ligar	34/7778	47/18670	1.97E-05	0.0001675	0.000102
BP	GO:0051091	positive regulation of DNA-binding transcription factor activity	142/7778	261/18670	1.98E-05	0.0001682	0.000102
BP	GO:0006925	inflammatory cell apoptotic process	19/7778	22/18670	2.01E-05	0.0001698	0.000103
BP	GO:0045943	positive regulation of transcription by RNA polymerase I	19/7778	22/18670	2.01E-05	0.0001698	0.000103
BP	GO:0010631	epithelial cell migration	185/7778	352/18670	2.06E-05	0.0001739	0.000106
BP	GO:1902751	positive regulation of cell cycle G2/M phase transition	24/7778	30/18670	2.07E-05	0.0001741	0.000106
BP	GO:0048194	Golgi vesicle budding	52/7778	80/18670	2.08E-05	0.0001751	0.000107
BP	GO:0006369	termination of RNA polymerase II transcription	27/7778	35/18670	2.09E-05	0.0001757	0.000107
BP	GO:0044784	metaphase/anaphase transition of cell cycle	39/7778	56/18670	2.10E-05	0.000176	0.000107
BP	GO:0046112	nucleobase biosynthetic process	17/7778	19/18670	2.14E-05	0.0001797	0.000109
BP	GO:1902750	negative regulation of cell cycle G2/M phase transition	65/7778	105/18670	2.20E-05	0.0001842	0.000112
BP	GO:0034644	cellular response to UV	53/7778	82/18670	2.20E-05	0.0001843	0.000112
BP	GO:0006289	nucleotide-excision repair	67/7778	109/18670	2.31E-05	0.0001927	0.000117
BP	GO:0010507	negative regulation of autophagy	54/7778	84/18670	2.32E-05	0.0001938	0.000118
BP	GO:0043123	positive regulation of I-kappaB kinase/NF-kappaB signaling	104/7778	183/18670	2.33E-05	0.0001938	0.000118
BP	GO:0050807	regulation of synapse organization	121/7778	218/18670	2.38E-05	0.0001981	0.000121
BP	GO:0001959	regulation of cytokine-mediated signaling pathway	101/7778	177/18670	2.41E-05	0.0002001	0.000122
BP	GO:0060491	regulation of cell projection assembly	101/7778	177/18670	2.41E-05	0.0002001	0.000122
BP	GO:0120032	regulation of plasma membrane bounded cell projection assembly	100/7778	175/18670	2.44E-05	0.0002015	0.000123
BP	GO:0046660	female sex differentiation	70/7778	115/18670	2.44E-05	0.0002015	0.000123
BP	GO:1904375	regulation of protein localization to cell periphery	70/7778	115/18670	2.44E-05	0.0002015	0.000123
BP	GO:0048013	ephrin receptor signaling pathway	55/7778	86/18670	2.44E-05	0.0002015	0.000123
BP	GO:0090559	regulation of membrane permeability	55/7778	86/18670	2.44E-05	0.0002015	0.000123
BP	GO:0051100	negative regulation of binding	97/7778	169/18670	2.51E-05	0.0002069	0.000126
BP	GO:0051053	negative regulation of DNA metabolic process	74/7778	123/18670	2.57E-05	0.0002117	0.000129
BP	GO:0099173	postsynapse organization	94/7778	163/18670	2.58E-05	0.0002117	0.000129
BP	GO:2000134	negative regulation of G1/S transition of mitotic cell cycle	75/7778	125/18670	2.60E-05	0.0002132	0.00013
BP	GO:0030901	midbrain development	57/7778	90/18670	2.67E-05	0.0002187	0.000133
BP	GO:0050684	regulation of mRNA processing	81/7778	137/18670	2.68E-05	0.0002194	0.000134
BP	GO:0016571	histone methylation	82/7778	139/18670	2.69E-05	0.0002194	0.000134
BP	GO:0033135	regulation of peptidyl-serine phosphorylation	82/7778	139/18670	2.69E-05	0.0002194	0.000134
BP	GO:0030879	mammary gland development	84/7778	143/18670	2.69E-05	0.0002194	0.000134
BP	GO:1902916	positive regulation of protein polyubiquitination	12/7778	12/18670	2.72E-05	0.0002215	0.000135
BP	GO:0000731	DNA synthesis involved in DNA repair	36/7778	51/18670	2.73E-05	0.0002217	0.000135
BP	GO:0006283	transcription-coupled nucleotide-excision repair	48/7778	73/18670	2.73E-05	0.0002217	0.000135
BP	GO:0050803	regulation of synapse structure or activity	125/7778	227/18670	2.90E-05	0.0002354	0.000143
BP	GO:0006356	regulation of transcription by RNA polymerase I	25/7778	32/18670	2.91E-05	0.0002361	0.000144
BP	GO:1902117	positive regulation of organelle assembly	49/7778	75/18670	2.92E-05	0.0002362	0.000144
BP	GO:0002753	cytoplasmic pattern recognition receptor signaling pathway	42/7778	62/18670	2.94E-05	0.0002367	0.000144
BP	GO:0048857	neural nucleus development	42/7778	62/18670	2.94E-05	0.0002367	0.000144
BP	GO:1902305	regulation of sodium ion transmembrane transport	42/7778	62/18670	2.94E-05	0.0002367	0.000144
BP	GO:0045665	negative regulation of neuron differentiation	124/7778	225/18670	2.96E-05	0.0002385	0.000145
BP	GO:0098876	vesicle-mediated transport to the plasma membrane	60/7778	96/18670	2.97E-05	0.0002385	0.000145
BP	GO:1990823	response to leukemia inhibitory factor	60/7778	96/18670	2.97E-05	0.0002385	0.000145
BP	GO:1990830	cellular response to leukemia inhibitory factor	60/7778	96/18670	2.97E-05	0.0002385	0.000145
BP	GO:0043112	receptor metabolic process	108/7778	192/18670	2.99E-05	0.0002394	0.000146
BP	GO:0045444	fat cell differentiation	123/7778	223/18670	3.03E-05	0.0002421	0.000147
BP	GO:0000288	nuclear-transcribed mRNA catabolic process, deadenylation-depend	50/7778	77/18670	3.10E-05	0.0002475	0.000151
BP	GO:0051702	interaction with symbiont	50/7778	77/18670	3.10E-05	0.0002475	0.000151
BP	GO:2000779	regulation of double-strand break repair	50/7778	77/18670	3.10E-05	0.0002475	0.000151
BP	GO:0010823	negative regulation of mitochondrion organization	37/7778	53/18670	3.13E-05	0.0002491	0.000152
BP	GO:0045088	regulation of innate immune response	162/7778	305/18670	3.15E-05	0.0002503	0.000152
BP	GO:0090174	organelle membrane fusion	62/7778	100/18670	3.15E-05	0.0002504	0.000152
BP	GO:0031334	positive regulation of protein-containing complex assembly	133/7778	244/18670	3.18E-05	0.0002519	0.000153
BP	GO:0046834	lipid phosphorylation	43/7778	64/18670	3.22E-05	0.0002555	0.000156
BP	GO:0001823	mesonephros development	63/7778	102/18670	3.23E-05	0.0002559	0.000156
BP	GO:0007032	endosome organization	51/7778	79/18670	3.29E-05	0.0002595	0.000158
BP	GO:0031110	regulation of microtubule polymerization or depolymerization	51/7778	79/18670	3.29E-05	0.0002595	0.000158
BP	GO:0090100	positive regulation of transmembrane receptor protein serine/threonir	64/7778	104/18670	3.31E-05	0.000261	0.000159
BP	GO:0051961	negative regulation of nervous system development	167/7778	316/18670	3.44E-05	0.0002709	0.000165
BP	GO:1903533	regulation of protein targeting	52/7778	81/18670	3.46E-05	0.0002724	0.000166
BP	GO:0010761	fibroblast migration	29/7778	39/18670	3.47E-05	0.0002724	0.000166
BP	GO:0072595	maintenance of protein localization in organelle	29/7778	39/18670	3.47E-05	0.0002724	0.000166
BP	GO:0048207	vesicle targeting, rough ER to cis-Golgi	44/7778	66/18670	3.51E-05	0.000275	0.000167
BP	GO:0048208	COPII vesicle coating	44/7778	66/18670	3.51E-05	0.000275	0.000167
BP	GO:0034724	DNA replication-independent nucleosome organization	38/7778	55/18670	3.55E-05	0.0002772	0.000169
BP	GO:0034968	histone lysine methylation	69/7778	114/18670	3.61E-05	0.0002823	0.000172

BP	GO:0009743	response to carbohydrate	126/7778	230/18670	3.72E-05	0.0002902	0.000177
BP	GO:1990845	adaptive thermogenesis	89/7778	154/18670	3.73E-05	0.0002907	0.000177
BP	GO:0022612	gland morphogenesis	72/7778	120/18670	3.74E-05	0.0002907	0.000177
BP	GO:0014066	regulation of phosphatidylinositol 3-kinase signaling	74/7778	124/18670	3.79E-05	0.0002948	0.000179
BP	GO:0016601	Rac protein signal transduction	26/7778	34/18670	3.91E-05	0.0003033	0.000185
BP	GO:0090132	epithelium migration	185/7778	355/18670	3.91E-05	0.0003034	0.000185
BP	GO:0010965	regulation of mitotic sister chromatid separation	39/7778	57/18670	3.97E-05	0.0003075	0.000187
BP	GO:0051642	centrosome localization	23/7778	29/18670	4.00E-05	0.0003096	0.000188
BP	GO:0030098	lymphocyte differentiation	184/7778	353/18670	4.03E-05	0.0003114	0.00019
BP	GO:0008089	anterograde axonal transport	34/7778	48/18670	4.06E-05	0.0003123	0.00019
BP	GO:0051653	spindle localization	34/7778	48/18670	4.06E-05	0.0003123	0.00019
BP	GO:0061647	histone H3-K9 modification	34/7778	48/18670	4.06E-05	0.0003123	0.00019
BP	GO:0006901	vesicle coating	46/7778	70/18670	4.08E-05	0.000314	0.000191
BP	GO:0032465	regulation of cytokinesis	56/7778	89/18670	4.11E-05	0.0003156	0.000192
BP	GO:0051604	protein maturation	152/7778	285/18670	4.13E-05	0.0003169	0.000193
BP	GO:0003002	regionalization	183/7778	351/18670	4.15E-05	0.000318	0.000194
BP	GO:0043312	neutrophil degranulation	245/7778	485/18670	4.16E-05	0.000318	0.000194
BP	GO:0090305	nucleic acid phosphodiester bond hydrolysis	160/7778	302/18670	4.18E-05	0.0003197	0.000195
BP	GO:0043153	entrainment of circadian clock by photoperiod	18/7778	21/18670	4.19E-05	0.0003197	0.000195
BP	GO:0071459	protein localization to chromosome, centromeric region	18/7778	21/18670	4.19E-05	0.0003197	0.000195
BP	GO:0098927	vesicle-mediated transport between endosomal compartments	30/7778	41/18670	4.27E-05	0.0003253	0.000198
BP	GO:0009260	ribonucleotide biosynthetic process	104/7778	185/18670	4.30E-05	0.0003267	0.000199
BP	GO:0036473	cell death in response to oxidative stress	58/7778	93/18670	4.39E-05	0.0003337	0.000203
BP	GO:0097711	ciliary basal body-plasma membrane docking	59/7778	95/18670	4.52E-05	0.0003431	0.000209
BP	GO:0060560	developmental growth involved in morphogenesis	128/7778	235/18670	4.63E-05	0.0003509	0.000214
BP	GO:1900006	positive regulation of dendrite development	48/7778	74/18670	4.64E-05	0.0003511	0.000214
BP	GO:0001961	positive regulation of cytokine-mediated signaling pathway	35/7778	50/18670	4.67E-05	0.0003534	0.000215
BP	GO:0001659	temperature homeostasis	98/7778	173/18670	4.69E-05	0.0003544	0.000216
BP	GO:0002283	neutrophil activation involved in immune response	246/7778	488/18670	4.80E-05	0.000362	0.00022
BP	GO:0051188	cofactor biosynthetic process	126/7778	231/18670	4.85E-05	0.0003649	0.000222
BP	GO:0061448	connective tissue development	146/7778	273/18670	4.85E-05	0.0003649	0.000222
BP	GO:0046545	development of primary female sexual characteristics	62/7778	101/18670	4.86E-05	0.0003649	0.000222
BP	GO:1903008	organelle disassembly	62/7778	101/18670	4.86E-05	0.0003649	0.000222
BP	GO:0046785	microtubule polymerization	49/7778	76/18670	4.90E-05	0.0003677	0.000224
BP	GO:0048871	multicellular organismal homeostasis	245/7778	486/18670	4.95E-05	0.000371	0.000226
BP	GO:0006612	protein targeting to membrane	112/7778	202/18670	4.99E-05	0.000373	0.000227
BP	GO:0045740	positive regulation of DNA replication	27/7778	36/18670	5.03E-05	0.000376	0.000229
BP	GO:0030433	ubiquitin-dependent ERAD pathway	50/7778	78/18670	5.16E-05	0.0003853	0.000235
BP	GO:0003179	heart valve morphogenesis	36/7778	52/18670	5.31E-05	0.0003953	0.000241
BP	GO:2000649	regulation of sodium ion transmembrane transporter activity	36/7778	52/18670	5.31E-05	0.0003953	0.000241
BP	GO:0032897	negative regulation of viral transcription	21/7778	26/18670	5.38E-05	0.0003984	0.000243
BP	GO:0060765	regulation of androgen receptor signaling pathway	21/7778	26/18670	5.38E-05	0.0003984	0.000243
BP	GO:1900739	regulation of protein insertion into mitochondrial membrane involved	21/7778	26/18670	5.38E-05	0.0003984	0.000243
BP	GO:1900740	positive regulation of protein insertion into mitochondrial membrane i	21/7778	26/18670	5.38E-05	0.0003984	0.000243
BP	GO:1903203	regulation of oxidative stress-induced neuron death	21/7778	26/18670	5.38E-05	0.0003984	0.000243
BP	GO:0007173	epidermal growth factor receptor signaling pathway	71/7778	119/18670	5.46E-05	0.0004036	0.000246
BP	GO:1901293	nucleoside phosphate biosynthetic process	150/7778	282/18670	5.51E-05	0.000406	0.000247
BP	GO:0010165	response to X-ray	24/7778	31/18670	5.52E-05	0.000406	0.000247
BP	GO:0045737	positive regulation of cyclin-dependent protein serine/threonine kinas	24/7778	31/18670	5.52E-05	0.000406	0.000247
BP	GO:1902895	positive regulation of pri-miRNA transcription by RNA polymerase II	24/7778	31/18670	5.52E-05	0.000406	0.000247
BP	GO:0030326	embryonic limb morphogenesis	74/7778	125/18670	5.53E-05	0.000406	0.000247
BP	GO:0035113	embryonic appendage morphogenesis	74/7778	125/18670	5.53E-05	0.000406	0.000247
BP	GO:0090130	tissue migration	187/7778	361/18670	5.57E-05	0.0004086	0.000249
BP	GO:0031345	negative regulation of cell projection organization	105/7778	188/18670	5.68E-05	0.0004161	0.000253
BP	GO:0009165	nucleotide biosynthetic process	148/7778	278/18670	5.82E-05	0.0004262	0.000259
BP	GO:0042692	muscle cell differentiation	198/7778	385/18670	5.93E-05	0.0004337	0.000264
BP	GO:0006336	DNA replication-independent nucleosome assembly	37/7778	54/18670	5.95E-05	0.0004349	0.000265
BP	GO:0048538	thymus development	32/7778	45/18670	6.04E-05	0.0004407	0.000268
BP	GO:2000573	positive regulation of DNA biosynthetic process	44/7778	67/18670	6.12E-05	0.0004458	0.000271
BP	GO:0045022	early endosome to late endosome transport	28/7778	38/18670	6.27E-05	0.0004561	0.000278
BP	GO:0030323	respiratory tube development	99/7778	176/18670	6.28E-05	0.0004563	0.000278
BP	GO:0030900	forebrain development	196/7778	381/18670	6.31E-05	0.000458	0.000279
BP	GO:0002064	epithelial cell development	114/7778	207/18670	6.32E-05	0.0004583	0.000279
BP	GO:0007389	pattern specification process	226/7778	446/18670	6.32E-05	0.0004583	0.000279
BP	GO:0046488	phosphatidylinositol metabolic process	98/7778	174/18670	6.37E-05	0.0004614	0.000281
BP	GO:0045778	positive regulation of ossification	56/7778	90/18670	6.49E-05	0.000469	0.000286
BP	GO:0006906	vesicle fusion	58/7778	94/18670	6.83E-05	0.0004936	0.000301
BP	GO:0047496	vesicle transport along microtubule	33/7778	47/18670	6.98E-05	0.0005031	0.000306
BP	GO:0008585	female gonad development	59/7778	96/18670	6.99E-05	0.0005031	0.000306
BP	GO:0048010	vascular endothelial growth factor receptor signaling pathway	59/7778	96/18670	6.99E-05	0.0005031	0.000306
BP	GO:1902254	negative regulation of intrinsic apoptotic signaling pathway by p53 cla	19/7778	23/18670	7.01E-05	0.0005038	0.000307
BP	GO:0051235	maintenance of location	165/7778	315/18670	7.05E-05	0.0005065	0.000308
BP	GO:0010464	regulation of mesenchymal cell proliferation	25/7778	33/18670	7.25E-05	0.000519	0.000316
BP	GO:0016242	negative regulation of macroautophagy	25/7778	33/18670	7.25E-05	0.000519	0.000316
BP	GO:0035306	positive regulation of dephosphorylation	39/7778	58/18670	7.26E-05	0.000519	0.000316
BP	GO:0051058	negative regulation of small GTPase mediated signal transduction	39/7778	58/18670	7.26E-05	0.000519	0.000316
BP	GO:0007219	Notch signaling pathway	107/7778	193/18670	7.29E-05	0.0005206	0.000317
BP	GO:0090092	regulation of transmembrane receptor protein serine/threonine kinase	130/7778	241/18670	7.34E-05	0.0005237	0.000319
BP	GO:0007517	muscle organ development	209/7778	410/18670	7.47E-05	0.0005325	0.000324
BP	GO:0048705	skeletal system morphogenesis	129/7778	239/18670	7.53E-05	0.0005358	0.000326
BP	GO:0042542	response to hydrogen peroxide	84/7778	146/18670	7.57E-05	0.0005384	0.000328
BP	GO:0044003	modulation by symbiont of host process	29/7778	40/18670	7.59E-05	0.0005393	0.000328
BP	GO:0051225	spindle assembly	65/7778	108/18670	7.67E-05	0.0005436	0.000331
BP	GO:0033137	negative regulation of peptidyl-serine phosphorylation	22/7778	28/18670	7.70E-05	0.0005436	0.000331
BP	GO:0033598	mammary gland epithelial cell proliferation	22/7778	28/18670	7.70E-05	0.0005436	0.000331
BP	GO:0051984	positive regulation of chromosome segregation	22/7778	28/18670	7.70E-05	0.0005436	0.000331
BP	GO:0060260	regulation of transcription initiation from RNA polymerase II promoter	22/7778	28/18670	7.70E-05	0.0005436	0.000331
BP	GO:0050808	synapse organization	208/7778	408/18670	7.71E-05	0.0005439	0.000331
BP	GO:0032507	maintenance of protein location in cell	40/7778	60/18670	7.91E-05	0.0005566	0.000339
BP	GO:0051306	mitotic sister chromatid separation	40/7778	60/18670	7.91E-05	0.0005566	0.000339
BP	GO:0000184	nuclear-transcribed mRNA catabolic process, nonsense-mediated de	71/7778	120/18670	7.95E-05	0.000559	0.00034
BP	GO:0043523	regulation of neuron apoptotic process	115/7778	210/18670	8.11E-05	0.0005699	0.000347

BP	GO:0006644	phospholipid metabolic process	218/7778	430/18670	8.13E-05	0.0005706	0.000347
BP	GO:0051205	protein insertion into membrane	41/7778	62/18670	8.54E-05	0.0005976	0.000364
BP	GO:1905268	negative regulation of chromatin organization	41/7778	62/18670	8.54E-05	0.0005976	0.000364
BP	GO:1905818	regulation of chromosome separation	41/7778	62/18670	8.54E-05	0.0005976	0.000364
BP	GO:0070987	error-free translesion synthesis	17/7778	20/18670	8.69E-05	0.0006073	0.00037
BP	GO:0072666	establishment of protein localization to vacuole	35/7778	51/18670	8.94E-05	0.0006237	0.00038
BP	GO:0031057	negative regulation of histone modification	30/7778	42/18670	8.99E-05	0.0006256	0.000381
BP	GO:0042149	cellular response to glucose starvation	30/7778	42/18670	8.99E-05	0.0006256	0.000381
BP	GO:1903146	regulation of autophagy of mitochondrion	30/7778	42/18670	8.99E-05	0.0006256	0.000381
BP	GO:0032435	negative regulation of proteasomal ubiquitin-dependent protein catat	26/7778	35/18670	9.16E-05	0.0006345	0.000386
BP	GO:0033144	negative regulation of intracellular steroid hormone receptor signaling	26/7778	35/18670	9.16E-05	0.0006345	0.000386
BP	GO:0060338	regulation of type I interferon-mediated signaling pathway	26/7778	35/18670	9.16E-05	0.0006345	0.000386
BP	GO:1904031	positive regulation of cyclin-dependent protein kinase activity	26/7778	35/18670	9.16E-05	0.0006345	0.000386
BP	GO:0046782	regulation of viral transcription	42/7778	64/18670	9.17E-05	0.0006345	0.000386
BP	GO:0046677	response to antibiotic	170/7778	327/18670	9.28E-05	0.0006415	0.000391
BP	GO:0002028	regulation of sodium ion transport	53/7778	85/18670	9.31E-05	0.0006428	0.000391
BP	GO:0045333	cellular respiration	107/7778	194/18670	9.64E-05	0.0006649	0.000405
BP	GO:0006268	DNA unwinding involved in DNA replication	13/7778	14/18670	9.72E-05	0.0006678	0.000407
BP	GO:0043922	negative regulation by host of viral transcription	13/7778	14/18670	9.72E-05	0.0006678	0.000407
BP	GO:0060211	regulation of nuclear-transcribed mRNA poly(A) tail shortening	13/7778	14/18670	9.72E-05	0.0006678	0.000407
BP	GO:0090110	COPII-coated vesicle cargo loading	13/7778	14/18670	9.72E-05	0.0006678	0.000407
BP	GO:1902108	regulation of mitochondrial membrane permeability involved in apopt	43/7778	66/18670	9.78E-05	0.0006708	0.000408
BP	GO:0007565	female pregnancy	106/7778	192/18670	9.84E-05	0.0006745	0.000411
BP	GO:1901185	negative regulation of ERBB signaling pathway	36/7778	53/18670	9.92E-05	0.0006793	0.000414
BP	GO:0006895	Golgi to endosome transport	15/7778	17/18670	9.93E-05	0.0006794	0.000414
BP	GO:0010972	negative regulation of G2/M transition of mitotic cell cycle	57/7778	93/18670	0.0001027	0.0007015	0.000427
BP	GO:0007162	negative regulation of cell adhesion	152/7778	289/18670	0.0001028	0.0007015	0.000427
BP	GO:1902105	regulation of leukocyte differentiation	144/7778	272/18670	0.0001031	0.0007031	0.000428
BP	GO:0035066	positive regulation of histone acetylation	23/7778	30/18670	0.0001037	0.0007046	0.000429
BP	GO:0061842	microtubule organizing center localization	23/7778	30/18670	0.0001037	0.0007046	0.000429
BP	GO:0071480	cellular response to gamma radiation	23/7778	30/18670	0.0001037	0.0007046	0.000429
BP	GO:0007019	microtubule depolymerization	31/7778	44/18670	0.0001044	0.0007074	0.000431
BP	GO:0007080	mitotic metaphase plate congression	31/7778	44/18670	0.0001044	0.0007074	0.000431
BP	GO:1903573	negative regulation of response to endoplasmic reticulum stress	31/7778	44/18670	0.0001044	0.0007074	0.000431
BP	GO:0006650	glycerophospholipid metabolic process	166/7778	319/18670	0.0001046	0.0007078	0.000431
BP	GO:0002053	positive regulation of mesenchymal cell proliferation	20/7778	25/18670	0.0001053	0.0007112	0.000433
BP	GO:0009648	photoperiodism	20/7778	25/18670	0.0001053	0.0007112	0.000433
BP	GO:0045927	positive regulation of growth	143/7778	270/18670	0.0001061	0.0007155	0.000436
BP	GO:0045017	glycerolipid biosynthetic process	134/7778	251/18670	0.0001076	0.0007249	0.000441
BP	GO:0007018	microtubule-based movement	150/7778	285/18670	0.0001089	0.0007329	0.000446
BP	GO:0048525	negative regulation of viral process	61/7778	101/18670	0.0001093	0.0007348	0.000447
BP	GO:0010565	regulation of cellular ketone metabolic process	100/7778	180/18670	0.0001109	0.0007447	0.000453
BP	GO:0051258	protein polymerization	149/7778	283/18670	0.000112	0.0007519	0.000458
BP	GO:0042307	positive regulation of protein import into nucleus	27/7778	37/18670	0.0001122	0.0007519	0.000458
BP	GO:1902807	negative regulation of cell cycle G1/S phase transition	76/7778	131/18670	0.0001124	0.0007526	0.000458
BP	GO:0048675	axon extension	71/7778	121/18670	0.0001144	0.0007656	0.000466
BP	GO:0030324	lung development	96/7778	172/18670	0.0001194	0.0007978	0.000486
BP	GO:2000377	regulation of reactive oxygen species metabolic process	107/7778	195/18670	0.0001267	0.0008459	0.000515
BP	GO:0061077	chaperone-mediated protein folding	39/7778	59/18670	0.0001283	0.0008555	0.000521
BP	GO:0043407	negative regulation of MAP kinase activity	49/7778	78/18670	0.0001294	0.0008609	0.000524
BP	GO:1903313	positive regulation of mRNA metabolic process	49/7778	78/18670	0.0001294	0.0008609	0.000524
BP	GO:0060317	cardiac epithelial to mesenchymal transition	24/7778	32/18670	0.0001334	0.0008858	0.000539
BP	GO:1902235	regulation of endoplasmic reticulum stress-induced intrinsic apoptotic	24/7778	32/18670	0.0001334	0.0008858	0.000539
BP	GO:2000826	regulation of heart morphogenesis	28/7778	39/18670	0.0001338	0.0008878	0.00054
BP	GO:0030865	cortical cytoskeleton organization	33/7778	48/18670	0.0001342	0.0008879	0.000541
BP	GO:0042059	negative regulation of epidermal growth factor receptor signaling pat	33/7778	48/18670	0.0001342	0.0008879	0.000541
BP	GO:2000059	negative regulation of ubiquitin-dependent protein catabolic process	33/7778	48/18670	0.0001342	0.0008879	0.000541
BP	GO:0015980	energy derivation by oxidation of organic compounds	150/7778	286/18670	0.0001356	0.0008957	0.000545
BP	GO:2001244	positive regulation of intrinsic apoptotic signaling pathway	40/7778	61/18670	0.0001375	0.0009078	0.000553
BP	GO:0007063	regulation of sister chromatid cohesion	18/7778	22/18670	0.0001402	0.0009206	0.00056
BP	GO:0032069	regulation of nuclease activity	18/7778	22/18670	0.0001402	0.0009206	0.00056
BP	GO:0035024	negative regulation of Rho protein signal transduction	18/7778	22/18670	0.0001402	0.0009206	0.00056
BP	GO:0051457	maintenance of protein location in nucleus	18/7778	22/18670	0.0001402	0.0009206	0.00056
BP	GO:0000187	activation of MAPK activity	86/7778	152/18670	0.0001403	0.0009206	0.00056
BP	GO:0034250	positive regulation of cellular amide metabolic process	86/7778	152/18670	0.0001403	0.0009206	0.00056
BP	GO:1903708	positive regulation of hemopoiesis	102/7778	185/18670	0.0001411	0.0009244	0.000563
BP	GO:0008360	regulation of cell shape	85/7778	150/18670	0.0001423	0.0009307	0.000567
BP	GO:0031123	RNA 3'-end processing	85/7778	150/18670	0.0001423	0.0009307	0.000567
BP	GO:0001776	leukocyte homeostasis	53/7778	86/18670	0.0001448	0.0009461	0.000576
BP	GO:0009649	entrainment of circadian clock	21/7778	27/18670	0.0001467	0.0009526	0.00058
BP	GO:0010971	positive regulation of G2/M transition of mitotic cell cycle	21/7778	27/18670	0.0001467	0.0009526	0.00058
BP	GO:0036475	neuron death in response to oxidative stress	21/7778	27/18670	0.0001467	0.0009526	0.00058
BP	GO:0061437	renal system vasculature development	21/7778	27/18670	0.0001467	0.0009526	0.00058
BP	GO:0061440	kidney vasculature development	21/7778	27/18670	0.0001467	0.0009526	0.00058
BP	GO:1902175	regulation of oxidative stress-induced intrinsic apoptotic signaling pat	21/7778	27/18670	0.0001467	0.0009526	0.00058
BP	GO:0060425	lung morphogenesis	34/7778	50/18670	0.0001492	0.0009671	0.000589
BP	GO:0070534	protein K63-linked ubiquitination	34/7778	50/18670	0.0001492	0.0009671	0.000589
BP	GO:0031497	chromatin assembly	110/7778	202/18670	0.0001544	0.0009995	0.000609
BP	GO:0031060	regulation of histone methylation	42/7778	65/18670	0.0001551	0.0010032	0.000611
BP	GO:0006458	'de novo' protein folding	29/7778	41/18670	0.0001562	0.0010092	0.000614
BP	GO:0030219	megakaryocyte differentiation	58/7778	96/18670	0.0001576	0.0010162	0.000619
BP	GO:0097327	response to antineoplastic agent	58/7778	96/18670	0.0001576	0.0010162	0.000619
BP	GO:0019886	antigen processing and presentation of exogenous peptide antigen v	59/7778	98/18670	0.0001593	0.0010253	0.000624
BP	GO:0099175	regulation of postsynapse organization	59/7778	98/18670	0.0001593	0.0010253	0.000624
BP	GO:0045667	regulation of osteoblast differentiation	73/7778	126/18670	0.0001611	0.001036	0.000631
BP	GO:0046580	negative regulation of Ras protein signal transduction	35/7778	52/18670	0.0001641	0.0010526	0.000641
BP	GO:1990089	response to nerve growth factor	35/7778	52/18670	0.0001641	0.0010526	0.000641
BP	GO:0010922	positive regulation of phosphatase activity	25/7778	34/18670	0.0001654	0.0010585	0.000644
BP	GO:0048048	embryonic eye morphogenesis	25/7778	34/18670	0.0001654	0.0010585	0.000644
BP	GO:0030307	positive regulation of cell growth	94/7778	169/18670	0.0001655	0.0010585	0.000644
BP	GO:0046474	glycerophospholipid biosynthetic process	117/7778	217/18670	0.0001678	0.0010727	0.000653
BP	GO:0005996	monosaccharide metabolic process	150/7778	287/18670	0.0001681	0.0010736	0.000654

BP	GO:0051293	establishment of spindle localization	30/7778	43/18670	0.000179	0.0011405	0.000694
BP	GO:1901985	positive regulation of protein acetylation	30/7778	43/18670	0.000179	0.0011405	0.000694
BP	GO:0010977	negative regulation of neuron projection development	87/7778	155/18670	0.0001873	0.0011926	0.000726
BP	GO:0034728	nucleosome organization	101/7778	184/18670	0.0001895	0.0012051	0.000734
BP	GO:0034284	response to monosaccharide	112/7778	207/18670	0.0001903	0.0012088	0.000736
BP	GO:0034508	centromere complex assembly	37/7778	56/18670	0.0001928	0.0012203	0.000743
BP	GO:0010390	histone monoubiquitination	22/7778	29/18670	0.000193	0.0012203	0.000743
BP	GO:0033032	regulation of myeloid cell apoptotic process	22/7778	29/18670	0.000193	0.0012203	0.000743
BP	GO:0035666	TRIF-dependent toll-like receptor signaling pathway	22/7778	29/18670	0.000193	0.0012203	0.000743
BP	GO:0070198	protein localization to chromosome, telomeric region	22/7778	29/18670	0.000193	0.0012203	0.000743
BP	GO:0006278	RNA-dependent DNA biosynthetic process	48/7778	77/18670	0.000198	0.0012493	0.000761
BP	GO:0031397	negative regulation of protein ubiquitination	48/7778	77/18670	0.000198	0.0012493	0.000761
BP	GO:0035872	nucleotide-binding domain, leucine rich repeat containing receptor si	26/7778	36/18670	0.000199	0.0012544	0.000764
BP	GO:0006446	regulation of translational initiation	49/7778	79/18670	0.0002036	0.0012806	0.00078
BP	GO:0042590	antigen processing and presentation of exogenous peptide antigen v	49/7778	79/18670	0.0002036	0.0012806	0.00078
BP	GO:0007097	nuclear migration	19/7778	24/18670	0.0002044	0.0012831	0.000781
BP	GO:1902914	regulation of protein polyubiquitination	19/7778	24/18670	0.0002044	0.0012831	0.000781
BP	GO:0030033	microvillus assembly	14/7778	16/18670	0.0002118	0.0013275	0.000808
BP	GO:0035970	peptidyl-threonine dephosphorylation	14/7778	16/18670	0.0002118	0.0013275	0.000808
BP	GO:0009749	response to glucose	107/7778	197/18670	0.000215	0.001346	0.00082
BP	GO:0050863	regulation of T cell activation	162/7778	314/18670	0.0002177	0.0013578	0.000827
BP	GO:0042532	negative regulation of tyrosine phosphorylation of STAT protein	12/7778	13/18670	0.0002177	0.0013578	0.000827
BP	GO:0043970	histone H3-K9 acetylation	12/7778	13/18670	0.0002177	0.0013578	0.000827
BP	GO:0090399	replicative senescence	12/7778	13/18670	0.0002177	0.0013578	0.000827
BP	GO:0034612	response to tumor necrosis factor	161/7778	312/18670	0.0002244	0.0013981	0.000851
BP	GO:1901654	response to ketone	105/7778	193/18670	0.0002256	0.0014039	0.000855
BP	GO:1901655	cellular response to ketone	56/7778	93/18670	0.0002299	0.0014299	0.000871
BP	GO:1902882	regulation of response to oxidative stress	58/7778	97/18670	0.0002338	0.0014523	0.000884
BP	GO:0001654	eye development	184/7778	362/18670	0.0002363	0.0014664	0.000893
BP	GO:0019048	modulation by virus of host process	23/7778	31/18670	0.0002431	0.0015044	0.000916
BP	GO:0044319	wound healing, spreading of cells	23/7778	31/18670	0.0002431	0.0015044	0.000916
BP	GO:0090505	epiboly involved in wound healing	23/7778	31/18670	0.0002431	0.0015044	0.000916
BP	GO:1990090	cellular response to nerve growth factor stimulus	33/7778	49/18670	0.0002471	0.0015276	0.00093
BP	GO:0071356	cellular response to tumor necrosis factor	151/7778	291/18670	0.0002483	0.0015334	0.000934
BP	GO:0021953	central nervous system neuron differentiation	100/7778	183/18670	0.0002535	0.0015644	0.000952
BP	GO:0030902	hindbrain development	85/7778	152/18670	0.0002609	0.0016084	0.000979
BP	GO:0009746	response to hexose	109/7778	202/18670	0.000264	0.0016257	0.00099
BP	GO:0033866	nucleoside bisphosphate biosynthetic process	43/7778	68/18670	0.000266	0.0016335	0.000995
BP	GO:0034030	ribonucleoside bisphosphate biosynthetic process	43/7778	68/18670	0.000266	0.0016335	0.000995
BP	GO:0034033	purine nucleoside bisphosphate biosynthetic process	43/7778	68/18670	0.000266	0.0016335	0.000995
BP	GO:0033077	T cell differentiation in thymus	44/7778	70/18670	0.0002758	0.0016922	0.00103
BP	GO:0007020	microtubule nucleation	20/7778	26/18670	0.000277	0.0016949	0.001032
BP	GO:0031954	positive regulation of protein autophosphorylation	20/7778	26/18670	0.000277	0.0016949	0.001032
BP	GO:0048596	embryonic camera-type eye morphogenesis	20/7778	26/18670	0.000277	0.0016949	0.001032
BP	GO:0046827	positive regulation of protein export from nucleus	17/7778	21/18670	0.0002781	0.0016983	0.001034
BP	GO:2000114	regulation of establishment of cell polarity	17/7778	21/18670	0.0002781	0.0016983	0.001034
BP	GO:0007015	actin filament organization	201/7778	400/18670	0.0002811	0.001715	0.001044
BP	GO:0000186	activation of MAPKK activity	35/7778	53/18670	0.0002901	0.001768	0.001076
BP	GO:0060998	regulation of dendritic spine development	46/7778	74/18670	0.0002933	0.0017857	0.001087
BP	GO:0006891	intra-Golgi vesicle-mediated transport	24/7778	33/18670	0.0002957	0.0017954	0.001093
BP	GO:0040001	establishment of mitotic spindle localization	24/7778	33/18670	0.0002957	0.0017954	0.001093
BP	GO:0070536	protein K63-linked deubiquitination	24/7778	33/18670	0.0002957	0.0017954	0.001093
BP	GO:0007272	ensheathment of neurons	76/7778	134/18670	0.0003007	0.0018184	0.001107
BP	GO:0008366	axon ensheathment	76/7778	134/18670	0.0003007	0.0018184	0.001107
BP	GO:0000380	alternative mRNA splicing, via spliceosome	47/7778	76/18670	0.0003009	0.0018184	0.001107
BP	GO:0050000	chromosome localization	47/7778	76/18670	0.0003009	0.0018184	0.001107
BP	GO:0060411	cardiac septum morphogenesis	47/7778	76/18670	0.0003009	0.0018184	0.001107
BP	GO:0090287	regulation of cellular response to growth factor stimulus	151/7778	292/18670	0.0003045	0.0018345	0.001117
BP	GO:0042476	odontogenesis	75/7778	132/18670	0.0003047	0.0018345	0.001117
BP	GO:0042552	myelination	75/7778	132/18670	0.0003047	0.0018345	0.001117
BP	GO:0045598	regulation of fat cell differentiation	75/7778	132/18670	0.0003047	0.0018345	0.001117
BP	GO:0010721	negative regulation of cell development	175/7778	344/18670	0.0003133	0.0018848	0.001148
BP	GO:0043462	regulation of ATPase activity	49/7778	80/18670	0.000314	0.0018872	0.001149
BP	GO:0048706	embryonic skeletal system development	72/7778	126/18670	0.0003161	0.0018978	0.001155
BP	GO:0070828	heterochromatin organization	51/7778	84/18670	0.0003244	0.001946	0.001185
BP	GO:0051310	metaphase plate congression	37/7778	57/18670	0.0003298	0.0019761	0.001203
BP	GO:0009060	aerobic respiration	53/7778	88/18670	0.0003322	0.0019891	0.001211
BP	GO:1903214	regulation of protein targeting to mitochondrion	30/7778	44/18670	0.0003387	0.0020262	0.001234
BP	GO:0060993	kidney morphogenesis	56/7778	94/18670	0.0003395	0.002029	0.001235
BP	GO:0048024	regulation of mRNA splicing, via spliceosome	58/7778	98/18670	0.0003418	0.0020372	0.00124
BP	GO:0002504	antigen processing and presentation of peptide or polysaccharide an	60/7778	102/18670	0.0003421	0.0020372	0.00124
BP	GO:0035335	peptidyl-tyrosine dephosphorylation	60/7778	102/18670	0.0003421	0.0020372	0.00124
BP	GO:0002181	cytoplasmic translation	59/7778	100/18670	0.0003422	0.0020372	0.00124
BP	GO:0021700	developmental maturation	147/7778	284/18670	0.0003439	0.0020457	0.001245
BP	GO:0032801	receptor catabolic process	25/7778	35/18670	0.0003497	0.0020765	0.001264
BP	GO:0070423	nucleotide-binding oligomerization domain containing signaling pathw	25/7778	35/18670	0.0003497	0.0020765	0.001264
BP	GO:0010038	response to metal ion	183/7778	362/18670	0.0003501	0.002077	0.001265
BP	GO:0003180	aortic valve morphogenesis	21/7778	28/18670	0.0003559	0.0021093	0.001284
BP	GO:0031032	actomyosin structure organization	107/7778	199/18670	0.0003564	0.0021102	0.001285
BP	GO:0050768	negative regulation of neurogenesis	152/7778	295/18670	0.0003609	0.0021347	0.0013
BP	GO:0070200	establishment of protein localization to telomere	15/7778	18/18670	0.0003647	0.0021543	0.001312
BP	GO:0046879	hormone secretion	158/7778	308/18670	0.0003648	0.0021543	0.001312
BP	GO:0010952	positive regulation of peptidase activity	106/7778	197/18670	0.0003657	0.0021576	0.001314
BP	GO:0070972	protein localization to endoplasmic reticulum	82/7778	147/18670	0.0003693	0.0021768	0.001325
BP	GO:0009152	purine ribonucleotide biosynthetic process	94/7778	172/18670	0.0003798	0.0022367	0.001362
BP	GO:0006893	Golgi to plasma membrane transport	40/7778	63/18670	0.0003814	0.002242	0.001365
BP	GO:0007062	sister chromatid cohesion	40/7778	63/18670	0.0003814	0.002242	0.001365
BP	GO:0110053	regulation of actin filament organization	136/7778	261/18670	0.0003896	0.0022877	0.001393
BP	GO:0000715	nucleotide-excision repair, DNA damage recognition	18/7778	23/18670	0.0003927	0.0023021	0.001402
BP	GO:0007064	mitotic sister chromatid cohesion	18/7778	23/18670	0.0003927	0.0023021	0.001402
BP	GO:0045580	regulation of T cell differentiation	78/7778	139/18670	0.0003963	0.0023207	0.001413
BP	GO:1990138	neuron projection extension	92/7778	168/18670	0.0003976	0.0023263	0.001416

BP	GO:0007599	hemostasis	173/7778	341/18670	0.0004009	0.0023433	0.001427
BP	GO:0003156	regulation of animal organ formation	26/7778	37/18670	0.0004043	0.0023525	0.001432
BP	GO:0006270	DNA replication initiation	26/7778	37/18670	0.0004043	0.0023525	0.001432
BP	GO:0032885	regulation of polysaccharide biosynthetic process	26/7778	37/18670	0.0004043	0.0023525	0.001432
BP	GO:0051084	'de novo' posttranslational protein folding	26/7778	37/18670	0.0004043	0.0023525	0.001432
BP	GO:0060236	regulation of mitotic spindle organization	26/7778	37/18670	0.0004043	0.0023525	0.001432
BP	GO:0032648	regulation of interferon-beta production	32/7778	48/18670	0.0004055	0.0023535	0.001433
BP	GO:0060324	face development	32/7778	48/18670	0.0004055	0.0023535	0.001433
BP	GO:0090199	regulation of release of cytochrome c from mitochondria	32/7778	48/18670	0.0004055	0.0023535	0.001433
BP	GO:0033500	carbohydrate homeostasis	127/7778	242/18670	0.00041	0.0023764	0.001447
BP	GO:0043967	histone H4 acetylation	42/7778	67/18670	0.0004102	0.0023764	0.001447
BP	GO:0045216	cell-cell junction organization	100/7778	185/18670	0.0004262	0.0024669	0.001502
BP	GO:0003206	cardiac chamber morphogenesis	73/7778	129/18670	0.0004286	0.002476	0.001507
BP	GO:0018022	peptidyl-lysine methylation	73/7778	129/18670	0.0004286	0.002476	0.001507
BP	GO:0032608	interferon-beta production	33/7778	50/18670	0.0004371	0.0025206	0.001535
BP	GO:0043489	RNA stabilization	33/7778	50/18670	0.0004371	0.0025206	0.001535
BP	GO:0010586	miRNA metabolic process	22/7778	30/18670	0.0004387	0.0025257	0.001538
BP	GO:0050690	regulation of defense response to virus by virus	22/7778	30/18670	0.0004387	0.0025257	0.001538
BP	GO:0006491	N-glycan processing	13/7778	15/18670	0.0004483	0.0025695	0.001564
BP	GO:0042921	glucocorticoid receptor signaling pathway	13/7778	15/18670	0.0004483	0.0025695	0.001564
BP	GO:0043518	negative regulation of DNA damage response, signal transduction by	13/7778	15/18670	0.0004483	0.0025695	0.001564
BP	GO:0060766	negative regulation of androgen receptor signaling pathway	13/7778	15/18670	0.0004483	0.0025695	0.001564
BP	GO:1902969	mitotic DNA replication	13/7778	15/18670	0.0004483	0.0025695	0.001564
BP	GO:0002479	antigen processing and presentation of exogenous peptide antigen v	46/7778	75/18670	0.000454	0.0025925	0.001578
BP	GO:0032482	Rab protein signal transduction	46/7778	75/18670	0.000454	0.0025925	0.001578
BP	GO:0051303	establishment of chromosome localization	46/7778	75/18670	0.000454	0.0025925	0.001578
BP	GO:1903201	regulation of oxidative stress-induced cell death	46/7778	75/18670	0.000454	0.0025925	0.001578
BP	GO:0050775	positive regulation of dendrite morphogenesis	27/7778	39/18670	0.0004584	0.0026111	0.00159
BP	GO:1902042	negative regulation of extrinsic apoptotic signaling pathway via death	27/7778	39/18670	0.0004584	0.0026111	0.00159
BP	GO:1904591	positive regulation of protein import	27/7778	39/18670	0.0004584	0.0026111	0.00159
BP	GO:0048588	developmental cell growth	123/7778	234/18670	0.0004607	0.0026217	0.001596
BP	GO:0043666	regulation of phosphoprotein phosphatase activity	67/7778	117/18670	0.0004625	0.0026296	0.001601
BP	GO:0031529	ruffle organization	34/7778	52/18670	0.0004671	0.0026511	0.001614
BP	GO:1900024	regulation of substrate adhesion-dependent cell spreading	34/7778	52/18670	0.0004671	0.0026511	0.001614
BP	GO:0016126	sterol biosynthetic process	49/7778	81/18670	0.0004754	0.0026959	0.001641
BP	GO:0097237	cellular response to toxic substance	129/7778	247/18670	0.0004799	0.0027193	0.001656
BP	GO:2000106	regulation of leukocyte apoptotic process	50/7778	83/18670	0.0004806	0.0027205	0.001656
BP	GO:0150063	visual system development	184/7778	366/18670	0.0004818	0.0027233	0.001658
BP	GO:0061351	neural precursor cell proliferation	83/7778	150/18670	0.0004828	0.0027233	0.001658
BP	GO:0014031	mesenchymal cell development	51/7778	85/18670	0.0004848	0.0027233	0.001658
BP	GO:0006983	ER overload response	11/7778	12/18670	0.0004849	0.0027233	0.001658
BP	GO:0010826	negative regulation of centrosome duplication	11/7778	12/18670	0.0004849	0.0027233	0.001658
BP	GO:0035330	regulation of hippo signaling	11/7778	12/18670	0.0004849	0.0027233	0.001658
BP	GO:0060213	positive regulation of nuclear-transcribed mRNA poly(A) tail shortenir	11/7778	12/18670	0.0004849	0.0027233	0.001658
BP	GO:0071888	macrophage apoptotic process	11/7778	12/18670	0.0004849	0.0027233	0.001658
BP	GO:1903894	regulation of IRE1-mediated unfolded protein response	11/7778	12/18670	0.0004849	0.0027233	0.001658
BP	GO:0014068	positive regulation of phosphatidylinositol 3-kinase signaling	52/7778	87/18670	0.0004882	0.0027397	0.001668
BP	GO:0006497	protein lipidation	53/7778	89/18670	0.0004908	0.0027507	0.001675
BP	GO:0002495	antigen processing and presentation of peptide antigen via MHC clas	59/7778	101/18670	0.000491	0.0027507	0.001675
BP	GO:0070301	cellular response to hydrogen peroxide	58/7778	99/18670	0.0004926	0.0027515	0.001675
BP	GO:0062207	regulation of pattern recognition receptor signaling pathway	55/7778	93/18670	0.0004936	0.0027515	0.001675
BP	GO:0072080	nephron tubule development	55/7778	93/18670	0.0004936	0.0027515	0.001675
BP	GO:0050810	regulation of steroid biosynthetic process	56/7778	95/18670	0.0004939	0.0027515	0.001675
BP	GO:0061326	renal tubule development	56/7778	95/18670	0.0004939	0.0027515	0.001675
BP	GO:1903076	regulation of protein localization to plasma membrane	56/7778	95/18670	0.0004939	0.0027515	0.001675
BP	GO:0035384	thioester biosynthetic process	35/7778	54/18670	0.0004955	0.0027515	0.001675
BP	GO:0043392	negative regulation of DNA binding	35/7778	54/18670	0.0004955	0.0027515	0.001675
BP	GO:0071616	acyl-CoA biosynthetic process	35/7778	54/18670	0.0004955	0.0027515	0.001675
BP	GO:0090329	regulation of DNA-dependent DNA replication	35/7778	54/18670	0.0004955	0.0027515	0.001675
BP	GO:0048562	embryonic organ morphogenesis	148/7778	288/18670	0.0004973	0.0027594	0.00168
BP	GO:0009108	coenzyme biosynthetic process	81/7778	146/18670	0.0005026	0.0027864	0.001696
BP	GO:0097178	ruffle assembly	28/7778	41/18670	0.0005115	0.0028334	0.001725
BP	GO:0033522	histone H2A ubiquitination	19/7778	25/18670	0.000518	0.0028568	0.001739
BP	GO:0044068	modulation by symbiont of host cellular process	19/7778	25/18670	0.000518	0.0028568	0.001739
BP	GO:0060330	regulation of response to interferon-gamma	19/7778	25/18670	0.000518	0.0028568	0.001739
BP	GO:0060334	regulation of interferon-gamma-mediated signaling pathway	19/7778	25/18670	0.000518	0.0028568	0.001739
BP	GO:0072012	glomerulus vasculature development	19/7778	25/18670	0.000518	0.0028568	0.001739
BP	GO:0060538	skeletal muscle organ development	92/7778	169/18670	0.0005186	0.002858	0.00174
BP	GO:0090183	regulation of kidney development	36/7778	56/18670	0.0005221	0.0028746	0.00175
BP	GO:0003176	aortic valve development	23/7778	32/18670	0.0005235	0.0028774	0.001752
BP	GO:0090504	epiboly	23/7778	32/18670	0.0005235	0.0028774	0.001752
BP	GO:0042593	glucose homeostasis	126/7778	241/18670	0.0005246	0.0028813	0.001754
BP	GO:0033209	tumor necrosis factor-mediated signaling pathway	91/7778	167/18670	0.0005311	0.0029143	0.001774
BP	GO:0048880	sensory system development	186/7778	371/18670	0.0005367	0.0029425	0.001791
BP	GO:0042276	error-prone translesion synthesis	16/7778	20/18670	0.0005463	0.0029781	0.001813
BP	GO:0046823	negative regulation of nucleocytoplasmic transport	16/7778	20/18670	0.0005463	0.0029781	0.001813
BP	GO:0046931	pore complex assembly	16/7778	20/18670	0.0005463	0.0029781	0.001813
BP	GO:1900151	regulation of nuclear-transcribed mRNA catabolic process, deadenyl	16/7778	20/18670	0.0005463	0.0029781	0.001813
BP	GO:1902236	negative regulation of endoplasmic reticulum stress-induced intrinsic	16/7778	20/18670	0.0005463	0.0029781	0.001813
BP	GO:1903204	negative regulation of oxidative stress-induced neuron death	16/7778	20/18670	0.0005463	0.0029781	0.001813
BP	GO:0030100	regulation of endocytosis	109/7778	205/18670	0.0005465	0.0029781	0.001813
BP	GO:0060997	dendritic spine morphogenesis	37/7778	58/18670	0.0005469	0.0029781	0.001813
BP	GO:0007266	Rho protein signal transduction	108/7778	203/18670	0.0005619	0.0030555	0.00186
BP	GO:0021762	substantia nigra development	29/7778	43/18670	0.000563	0.0030555	0.00186
BP	GO:0030866	cortical actin cytoskeleton organization	29/7778	43/18670	0.000563	0.0030555	0.00186
BP	GO:0045773	positive regulation of axon extension	29/7778	43/18670	0.000563	0.0030555	0.00186
BP	GO:0051146	striated muscle cell differentiation	150/7778	293/18670	0.0005674	0.003077	0.001873
BP	GO:0032271	regulation of protein polymerization	115/7778	218/18670	0.0005805	0.0031453	0.001915
BP	GO:0034660	ncRNA metabolic process	233/7778	475/18670	0.0005878	0.0031819	0.001937
BP	GO:0018108	peptidyl-tyrosine phosphorylation	182/7778	363/18670	0.0006094	0.0032948	0.002006
BP	GO:0045669	positive regulation of osteoblast differentiation	40/7778	64/18670	0.0006102	0.0032948	0.002006
BP	GO:0048645	animal organ formation	40/7778	64/18670	0.0006102	0.0032948	0.002006

BP	GO:0009101	glycoprotein biosynthetic process	175/7778	348/18670	0.0006412	0.0034593	0.002106
BP	GO:2000144	positive regulation of DNA-templated transcription, initiation	20/7778	27/18670	0.0006495	0.0035011	0.002132
BP	GO:0018198	peptidyl-cysteine modification	31/7778	47/18670	0.0006594	0.003546	0.002159
BP	GO:0031952	regulation of protein autophosphorylation	31/7778	47/18670	0.0006594	0.003546	0.002159
BP	GO:0045646	regulation of erythrocyte differentiation	31/7778	47/18670	0.0006594	0.003546	0.002159
BP	GO:0006354	DNA-templated transcription, elongation	64/7778	112/18670	0.0006626	0.0035598	0.002167
BP	GO:1903051	negative regulation of proteolysis involved in cellular protein catabolic	44/7778	72/18670	0.0006691	0.0035922	0.002187
BP	GO:0072088	nephron epithelium morphogenesis	46/7778	76/18670	0.0006886	0.0036906	0.002247
BP	GO:1902653	secondary alcohol biosynthetic process	46/7778	76/18670	0.0006886	0.0036906	0.002247
BP	GO:1901099	negative regulation of signal transduction in absence of ligand	25/7778	36/18670	0.0006923	0.0037043	0.002255
BP	GO:2001240	negative regulation of extrinsic apoptotic signaling pathway in absence	25/7778	36/18670	0.0006923	0.0037043	0.002255
BP	GO:0002367	cytokine production involved in immune response	59/7778	102/18670	0.0006954	0.003712	0.00226
BP	GO:0031016	pancreas development	47/7778	78/18670	0.0006961	0.003712	0.00226
BP	GO:0061333	renal tubule morphogenesis	47/7778	78/18670	0.0006961	0.003712	0.00226
BP	GO:0072028	nephron morphogenesis	47/7778	78/18670	0.0006961	0.003712	0.00226
BP	GO:0018212	peptidyl-tyrosine modification	183/7778	366/18670	0.0006989	0.0037241	0.002267
BP	GO:0034260	negative regulation of GTPase activity	32/7778	49/18670	0.0007038	0.0037416	0.002278
BP	GO:0001824	blastocyst development	57/7778	98/18670	0.0007045	0.0037416	0.002278
BP	GO:0030038	contractile actin filament bundle assembly	57/7778	98/18670	0.0007045	0.0037416	0.002278
BP	GO:0043149	stress fiber assembly	57/7778	98/18670	0.0007045	0.0037416	0.002278
BP	GO:0097581	lamellipodium organization	49/7778	82/18670	0.0007068	0.0037506	0.002283
BP	GO:0006303	double-strand break repair via nonhomologous end joining	51/7778	86/18670	0.0007124	0.003771	0.002296
BP	GO:1903363	negative regulation of cellular protein catabolic process	51/7778	86/18670	0.0007124	0.003771	0.002296
BP	GO:0042147	retrograde transport, endosome to Golgi	52/7778	88/18670	0.0007135	0.003771	0.002296
BP	GO:1900407	regulation of cellular response to oxidative stress	52/7778	88/18670	0.0007135	0.003771	0.002296
BP	GO:0001656	metanephros development	53/7778	90/18670	0.0007136	0.003771	0.002296
BP	GO:0009100	glycoprotein metabolic process	207/7778	419/18670	0.0007293	0.0038509	0.002345
BP	GO:0002833	positive regulation of response to biotic stimulus	129/7778	249/18670	0.0007299	0.0038509	0.002345
BP	GO:0015693	magnesium ion transport	14/7778	17/18670	0.0007369	0.003875	0.002359
BP	GO:0033262	regulation of nuclear cell cycle DNA replication	14/7778	17/18670	0.0007369	0.003875	0.002359
BP	GO:0034501	protein localization to kinetochore	14/7778	17/18670	0.0007369	0.003875	0.002359
BP	GO:0060644	mammary gland epithelial cell differentiation	14/7778	17/18670	0.0007369	0.003875	0.002359
BP	GO:0038061	NIK/NF-kappaB signaling	98/7778	183/18670	0.0007378	0.0038767	0.00236
BP	GO:0000423	mitophagy	17/7778	22/18670	0.000747	0.0039059	0.002378
BP	GO:0001759	organ induction	17/7778	22/18670	0.000747	0.0039059	0.002378
BP	GO:0010869	regulation of receptor biosynthetic process	17/7778	22/18670	0.000747	0.0039059	0.002378
BP	GO:0032469	endoplasmic reticulum calcium ion homeostasis	17/7778	22/18670	0.000747	0.0039059	0.002378
BP	GO:0032727	positive regulation of interferon-alpha production	17/7778	22/18670	0.000747	0.0039059	0.002378
BP	GO:1901522	positive regulation of transcription from RNA polymerase II promoter	17/7778	22/18670	0.000747	0.0039059	0.002378
BP	GO:0097352	autophagosome maturation	26/7778	38/18670	0.0007738	0.004043	0.002461
BP	GO:0007596	blood coagulation	169/7778	336/18670	0.0007759	0.0040503	0.002466
BP	GO:0052126	movement in host environment	86/7778	158/18670	0.0007786	0.0040615	0.002473
BP	GO:0000132	establishment of mitotic spindle orientation	21/7778	29/18670	0.0007835	0.0040654	0.002475
BP	GO:0030511	positive regulation of transforming growth factor beta receptor signali	21/7778	29/18670	0.0007835	0.0040654	0.002475
BP	GO:0031114	regulation of microtubule depolymerization	21/7778	29/18670	0.0007835	0.0040654	0.002475
BP	GO:1903846	positive regulation of cellular response to transforming growth factor	21/7778	29/18670	0.0007835	0.0040654	0.002475
BP	GO:0036297	interstrand cross-link repair	34/7778	53/18670	0.0007838	0.0040654	0.002475
BP	GO:0045840	positive regulation of mitotic nuclear division	34/7778	53/18670	0.0007838	0.0040654	0.002475
BP	GO:0050732	negative regulation of peptidyl-tyrosine phosphorylation	34/7778	53/18670	0.0007838	0.0040654	0.002475
BP	GO:0099003	vesicle-mediated transport in synapse	112/7778	213/18670	0.000792	0.0041047	0.002499
BP	GO:0072593	reactive oxygen species metabolic process	145/7778	284/18670	0.0008031	0.0041586	0.002532
BP	GO:0045727	positive regulation of translation	71/7778	127/18670	0.0008128	0.0042055	0.00256
BP	GO:0031929	TOR signaling	70/7778	125/18670	0.0008274	0.0042778	0.002604
BP	GO:0001678	cellular glucose homeostasis	83/7778	152/18670	0.0008361	0.0043194	0.00263
BP	GO:0006734	NADH metabolic process	27/7778	40/18670	0.0008521	0.004395	0.002676
BP	GO:0061462	protein localization to lysosome	27/7778	40/18670	0.0008521	0.004395	0.002676
BP	GO:0071774	response to fibroblast growth factor	82/7778	150/18670	0.0008559	0.0044095	0.002685
BP	GO:0001704	formation of primary germ layer	68/7778	121/18670	0.0008563	0.0044095	0.002685
BP	GO:0044706	multi-multicellular organism process	116/7778	222/18670	0.0008758	0.0045066	0.002744
BP	GO:1902369	negative regulation of RNA catabolic process	37/7778	59/18670	0.0008813	0.0045308	0.002758
BP	GO:0022904	respiratory electron transport chain	66/7778	117/18670	0.0008845	0.0045438	0.002766
BP	GO:0072522	purine-containing compound biosynthetic process	108/7778	205/18670	0.0008897	0.0045669	0.00278
BP	GO:0035051	cardiocyte differentiation	90/7778	167/18670	0.0009109	0.0046719	0.002844
BP	GO:0046620	regulation of organ growth	64/7778	113/18670	0.0009116	0.0046719	0.002844
BP	GO:0022900	electron transport chain	99/7778	186/18670	0.0009125	0.0046728	0.002845
BP	GO:0010762	regulation of fibroblast migration	22/7778	31/18670	0.0009169	0.004688	0.002854
BP	GO:0071539	protein localization to centrosome	22/7778	31/18670	0.0009169	0.004688	0.002854
BP	GO:0044088	regulation of vacuole organization	28/7778	42/18670	0.0009265	0.0047334	0.002882
BP	GO:0009141	nucleoside triphosphate metabolic process	62/7778	109/18670	0.0009373	0.0047789	0.00291
BP	GO:0072009	nephron epithelium development	62/7778	109/18670	0.0009373	0.0047789	0.00291
BP	GO:0036092	phosphatidylinositol-3-phosphate biosynthetic process	12/7778	14/18670	0.0009407	0.0047789	0.00291
BP	GO:0071428	rRNA-containing ribonucleoprotein complex export from nucleus	12/7778	14/18670	0.0009407	0.0047789	0.00291
BP	GO:0090231	regulation of spindle checkpoint	12/7778	14/18670	0.0009407	0.0047789	0.00291
BP	GO:0090266	regulation of mitotic cell cycle spindle assembly checkpoint	12/7778	14/18670	0.0009407	0.0047789	0.00291
BP	GO:1903504	regulation of mitotic spindle checkpoint	12/7778	14/18670	0.0009407	0.0047789	0.00291
BP	GO:0098781	ncRNA transcription	61/7778	107/18670	0.0009495	0.0048198	0.002934
BP	GO:0000381	regulation of alternative mRNA splicing, via spliceosome	40/7778	65/18670	0.0009522	0.00483	0.002941
BP	GO:0001783	B cell apoptotic process	18/7778	24/18670	0.0009579	0.0048475	0.002951
BP	GO:0032878	regulation of establishment or maintenance of cell polarity	18/7778	24/18670	0.0009579	0.0048475	0.002951
BP	GO:0099563	modification of synaptic structure	18/7778	24/18670	0.0009579	0.0048475	0.002951
BP	GO:0000018	regulation of DNA recombination	58/7778	101/18670	0.0009825	0.0049682	0.003025
BP	GO:0070227	lymphocyte apoptotic process	42/7778	69/18670	0.0009859	0.0049809	0.003033
BP	GO:0045089	positive regulation of innate immune response	112/7778	214/18670	0.0009866	0.0049809	0.003033
BP	GO:0010463	mesenchymal cell proliferation	29/7778	44/18670	0.0009966	0.0050118	0.003051
BP	GO:0018023	peptidyl-lysine trimethylation	29/7778	44/18670	0.0009966	0.0050118	0.003051
BP	GO:0060412	ventricular septum morphogenesis	29/7778	44/18670	0.0009966	0.0050118	0.003051
BP	GO:0061001	regulation of dendritic spine morphogenesis	29/7778	44/18670	0.0009966	0.0050118	0.003051
BP	GO:0090311	regulation of protein deacetylation	29/7778	44/18670	0.0009966	0.0050118	0.003051
BP	GO:0120162	positive regulation of cold-induced thermogenesis	56/7778	97/18670	0.001001	0.0050301	0.003062
BP	GO:0110020	regulation of actomyosin structure organization	55/7778	95/18670	0.001009	0.0050618	0.003082
BP	GO:0031100	animal organ regeneration	44/7778	73/18670	0.0010097	0.0050618	0.003082
BP	GO:0050688	regulation of defense response to virus	44/7778	73/18670	0.0010097	0.0050618	0.003082

BP	GO:1901184	regulation of ERBB signaling pathway	54/7778	93/18670	0.001016	0.0050892	0.003099
BP	GO:0006695	cholesterol biosynthetic process	45/7778	75/18670	0.0010182	0.0050967	0.003103
BP	GO:0045638	negative regulation of myeloid cell differentiation	53/7778	91/18670	0.0010219	0.0051109	0.003112
BP	GO:0022409	positive regulation of cell-cell adhesion	131/7778	255/18670	0.0010258	0.0051265	0.003121
BP	GO:0061053	somite development	52/7778	89/18670	0.0010266	0.0051267	0.003121
BP	GO:1903312	negative regulation of mRNA metabolic process	47/7778	79/18670	0.0010292	0.0051356	0.003127
BP	GO:0048864	stem cell development	50/7778	85/18670	0.0010322	0.0051466	0.003133
BP	GO:0061572	actin filament bundle organization	85/7778	157/18670	0.0010348	0.0051557	0.003139
BP	GO:0051897	positive regulation of protein kinase B signaling	94/7778	176/18670	0.0010471	0.005213	0.003174
BP	GO:1903707	negative regulation of hemopoiesis	84/7778	155/18670	0.0010611	0.0052706	0.003209
BP	GO:0016082	synaptic vesicle priming	15/7778	19/18670	0.0010618	0.0052706	0.003209
BP	GO:1903798	regulation of production of miRNAs involved in gene silencing by miF	15/7778	19/18670	0.0010618	0.0052706	0.003209
BP	GO:0031648	protein destabilization	30/7778	46/18670	0.001062	0.0052706	0.003209
BP	GO:0034470	ncRNA processing	190/7778	384/18670	0.0010703	0.0053054	0.00323
BP	GO:0002328	pro-B cell differentiation	10/7778	11/18670	0.0010731	0.0053054	0.00323
BP	GO:0009113	purine nucleobase biosynthetic process	10/7778	11/18670	0.0010731	0.0053054	0.00323
BP	GO:0042023	DNA endoreduplication	10/7778	11/18670	0.0010731	0.0053054	0.00323
BP	GO:2000615	regulation of histone H3-K9 acetylation	10/7778	11/18670	0.0010731	0.0053054	0.00323
BP	GO:0051017	actin filament bundle assembly	83/7778	153/18670	0.0010878	0.0053739	0.003272
BP	GO:0002683	negative regulation of immune system process	213/7778	435/18670	0.0011035	0.0054474	0.003317
BP	GO:0030850	prostate gland development	31/7778	48/18670	0.0011224	0.0055323	0.003368
BP	GO:0031055	chromatin remodeling at centromere	31/7778	48/18670	0.0011224	0.0055323	0.003368
BP	GO:0021537	telencephalon development	128/7778	249/18670	0.0011262	0.0055466	0.003377
BP	GO:0071772	response to BMP	91/7778	170/18670	0.001136	0.0055864	0.003401
BP	GO:0071773	cellular response to BMP stimulus	91/7778	170/18670	0.001136	0.0055864	0.003401
BP	GO:1902905	positive regulation of supramolecular fiber organization	107/7778	204/18670	0.0011441	0.0056221	0.003423
BP	GO:0022011	myelination in peripheral nervous system	19/7778	26/18670	0.0011719	0.005714	0.003479
BP	GO:0032292	peripheral nervous system axon ensheathment	19/7778	26/18670	0.0011719	0.005714	0.003479
BP	GO:0032800	receptor biosynthetic process	19/7778	26/18670	0.0011719	0.005714	0.003479
BP	GO:0060706	cell differentiation involved in embryonic placenta development	19/7778	26/18670	0.0011719	0.005714	0.003479
BP	GO:0150117	positive regulation of cell-substrate junction organization	19/7778	26/18670	0.0011719	0.005714	0.003479
BP	GO:1903959	regulation of anion transmembrane transport	19/7778	26/18670	0.0011719	0.005714	0.003479
BP	GO:0001893	maternal placenta development	24/7778	35/18670	0.0011731	0.005714	0.003479
BP	GO:0019076	viral release from host cell	24/7778	35/18670	0.0011731	0.005714	0.003479
BP	GO:0035890	exit from host	24/7778	35/18670	0.0011731	0.005714	0.003479
BP	GO:0035891	exit from host cell	24/7778	35/18670	0.0011731	0.005714	0.003479
BP	GO:2000785	regulation of autophagosome assembly	24/7778	35/18670	0.0011731	0.005714	0.003479
BP	GO:0009161	ribonucleoside monophosphate metabolic process	32/7778	50/18670	0.0011779	0.005714	0.003479
BP	GO:0010171	body morphogenesis	32/7778	50/18670	0.0011779	0.005714	0.003479
BP	GO:0032873	negative regulation of stress-activated MAPK cascade	32/7778	50/18670	0.0011779	0.005714	0.003479
BP	GO:0045540	regulation of cholesterol biosynthetic process	32/7778	50/18670	0.0011779	0.005714	0.003479
BP	GO:0070303	negative regulation of stress-activated protein kinase signaling casca	32/7778	50/18670	0.0011779	0.005714	0.003479
BP	GO:0106118	regulation of sterol biosynthetic process	32/7778	50/18670	0.0011779	0.005714	0.003479
BP	GO:0060348	bone development	113/7778	217/18670	0.0011866	0.0057516	0.003502
BP	GO:0007612	learning	79/7778	145/18670	0.0011994	0.0058095	0.003537
BP	GO:0008333	endosome to lysosome transport	33/7778	52/18670	0.0012284	0.0059368	0.003615
BP	GO:0072132	mesenchyme morphogenesis	33/7778	52/18670	0.0012284	0.0059368	0.003615
BP	GO:0044344	cellular response to fibroblast growth factor stimulus	78/7778	143/18670	0.0012285	0.0059368	0.003615
BP	GO:0007611	learning or memory	131/7778	256/18670	0.0012462	0.0060136	0.003661
BP	GO:0090596	sensory organ morphogenesis	131/7778	256/18670	0.0012462	0.0060136	0.003661
BP	GO:0046890	regulation of lipid biosynthetic process	104/7778	198/18670	0.00125	0.0060271	0.00367
BP	GO:0009914	hormone transport	159/7778	317/18670	0.001268	0.0061097	0.00372
BP	GO:0061387	regulation of extent of cell growth	62/7778	110/18670	0.0012825	0.0061746	0.003759
BP	GO:1903037	regulation of leukocyte cell-cell adhesion	153/7778	304/18670	0.0012915	0.0061995	0.003774
BP	GO:0034405	response to fluid shear stress	25/7778	37/18670	0.0012924	0.0061995	0.003774
BP	GO:0060323	head morphogenesis	25/7778	37/18670	0.0012924	0.0061995	0.003774
BP	GO:1900026	positive regulation of substrate adhesion-dependent cell spreading	25/7778	37/18670	0.0012924	0.0061995	0.003774
BP	GO:1905314	semi-lunar valve development	25/7778	37/18670	0.0012924	0.0061995	0.003774
BP	GO:0051495	positive regulation of cytoskeleton organization	117/7778	226/18670	0.0012941	0.0062026	0.003776
BP	GO:0007519	skeletal muscle tissue development	86/7778	160/18670	0.0012984	0.0062189	0.003786
BP	GO:0010632	regulation of epithelial cell migration	147/7778	291/18670	0.0013101	0.0062703	0.003818
BP	GO:0035904	aorta development	36/7778	58/18670	0.0013507	0.006455	0.00393
BP	GO:0061951	establishment of protein localization to plasma membrane	36/7778	58/18670	0.0013507	0.006455	0.00393
BP	GO:0043486	histone exchange	37/7778	60/18670	0.0013822	0.006596	0.004016
BP	GO:0032647	regulation of interferon-alpha production	20/7778	28/18670	0.0013833	0.006596	0.004016
BP	GO:0051647	nucleus localization	20/7778	28/18670	0.0013833	0.006596	0.004016
BP	GO:0006622	protein targeting to lysosome	16/7778	21/18670	0.0014046	0.0066584	0.004054
BP	GO:0035455	response to interferon-alpha	16/7778	21/18670	0.0014046	0.0066584	0.004054
BP	GO:0043984	histone H4-K16 acetylation	16/7778	21/18670	0.0014046	0.0066584	0.004054
BP	GO:0070920	regulation of production of small RNA involved in gene silencing by F	16/7778	21/18670	0.0014046	0.0066584	0.004054
BP	GO:0072111	cell proliferation involved in kidney development	16/7778	21/18670	0.0014046	0.0066584	0.004054
BP	GO:0031062	positive regulation of histone methylation	26/7778	39/18670	0.0014046	0.0066584	0.004054
BP	GO:0032506	cytokinetic process	26/7778	39/18670	0.0014046	0.0066584	0.004054
BP	GO:0043001	Golgi to plasma membrane protein transport	26/7778	39/18670	0.0014046	0.0066584	0.004054
BP	GO:0006220	pyrimidine nucleotide metabolic process	38/7778	62/18670	0.0014094	0.0066711	0.004062
BP	GO:0006521	regulation of cellular amino acid metabolic process	38/7778	62/18670	0.0014094	0.0066711	0.004062
BP	GO:0030516	regulation of axon extension	55/7778	96/18670	0.0014127	0.0066817	0.004068
BP	GO:0042158	lipoprotein biosynthetic process	54/7778	94/18670	0.001428	0.006749	0.004109
BP	GO:0060135	maternal process involved in female pregnancy	39/7778	64/18670	0.0014325	0.0067604	0.004116
BP	GO:1901607	alpha-amino acid biosynthetic process	39/7778	64/18670	0.0014325	0.0067604	0.004116
BP	GO:2000117	negative regulation of cysteine-type endopeptidase activity	53/7778	92/18670	0.0014421	0.0068009	0.004141
BP	GO:0048738	cardiac muscle tissue development	120/7778	233/18670	0.0014445	0.0068071	0.004144
BP	GO:0072171	mesonephric tubule morphogenesis	40/7778	66/18670	0.0014516	0.0068358	0.004162
BP	GO:0014033	neural crest cell differentiation	52/7778	90/18670	0.001455	0.0068415	0.004165
BP	GO:0051304	chromosome separation	52/7778	90/18670	0.001455	0.0068415	0.004165
BP	GO:1903321	negative regulation of protein modification by small protein conjugatio	51/7778	88/18670	0.0014664	0.0068766	0.004187
BP	GO:0035914	skeletal muscle cell differentiation	41/7778	68/18670	0.0014671	0.0068766	0.004187
BP	GO:0046323	glucose import	41/7778	68/18670	0.0014671	0.0068766	0.004187
BP	GO:0050890	cognition	149/7778	296/18670	0.0014674	0.0068766	0.004187
BP	GO:0031958	corticosteroid receptor signaling pathway	13/7778	16/18670	0.0014731	0.0068766	0.004187
BP	GO:0034975	protein folding in endoplasmic reticulum	13/7778	16/18670	0.0014731	0.0068766	0.004187
BP	GO:0035372	protein localization to microtubule	13/7778	16/18670	0.0014731	0.0068766	0.004187

BP	GO:0051444	negative regulation of ubiquitin-protein transferase activity	13/7778	16/18670	0.0014731	0.0068766	0.004187
BP	GO:1902166	negative regulation of intrinsic apoptotic signaling pathway in respons	13/7778	16/18670	0.0014731	0.0068766	0.004187
BP	GO:1903830	magnesium ion transmembrane transport	13/7778	16/18670	0.0014731	0.0068766	0.004187
BP	GO:0035270	endocrine system development	70/7778	127/18670	0.0014748	0.0068797	0.004189
BP	GO:0043154	negative regulation of cysteine-type endopeptidase activity involved i	49/7778	84/18670	0.0014845	0.0069141	0.00421
BP	GO:0051492	regulation of stress fiber assembly	49/7778	84/18670	0.0014845	0.0069141	0.00421
BP	GO:0045619	regulation of lymphocyte differentiation	90/7778	169/18670	0.0014854	0.0069141	0.00421
BP	GO:0035019	somatic stem cell population maintenance	43/7778	72/18670	0.001488	0.0069159	0.004211
BP	GO:0051881	regulation of mitochondrial membrane potential	43/7778	72/18670	0.001488	0.0069159	0.004211
BP	GO:0072078	nephron tubule morphogenesis	44/7778	74/18670	0.0014937	0.0069333	0.004221
BP	GO:0007492	endoderm development	45/7778	76/18670	0.0014967	0.0069333	0.004221
BP	GO:0030500	regulation of bone mineralization	45/7778	76/18670	0.0014967	0.0069333	0.004221
BP	GO:0031507	heterochromatin assembly	45/7778	76/18670	0.0014967	0.0069333	0.004221
BP	GO:0090398	cellular senescence	46/7778	78/18670	0.0014971	0.0069333	0.004221
BP	GO:0061028	establishment of endothelial barrier	27/7778	41/18670	0.001509	0.0069734	0.004246
BP	GO:0090224	regulation of spindle organization	27/7778	41/18670	0.001509	0.0069734	0.004246
BP	GO:0090317	negative regulation of intracellular protein transport	27/7778	41/18670	0.001509	0.0069734	0.004246
BP	GO:0006364	rRNA processing	111/7778	214/18670	0.0015556	0.0071836	0.004374
BP	GO:0032728	positive regulation of interferon-beta production	21/7778	30/18670	0.0015879	0.0073169	0.004455
BP	GO:0048873	homeostasis of number of cells within a tissue	21/7778	30/18670	0.0015879	0.0073169	0.004455
BP	GO:0061157	mRNA destabilization	21/7778	30/18670	0.0015879	0.0073169	0.004455
BP	GO:0034198	cellular response to amino acid starvation	28/7778	43/18670	0.0016051	0.0073912	0.0045
BP	GO:0016072	rRNA metabolic process	116/7778	225/18670	0.0016357	0.0075268	0.004583
BP	GO:0010569	regulation of double-strand break repair via homologous recombinati	29/7778	45/18670	0.0016929	0.0077787	0.004736
BP	GO:0048255	mRNA stabilization	29/7778	45/18670	0.0016929	0.0077787	0.004736
BP	GO:0043244	regulation of protein-containing complex disassembly	63/7778	113/18670	0.0017027	0.007818	0.00476
BP	GO:0035305	negative regulation of dephosphorylation	62/7778	111/18670	0.0017352	0.0079616	0.004847
BP	GO:0006541	glutamine metabolic process	17/7778	23/18670	0.0017506	0.0080211	0.004883
BP	GO:0009067	aspartate family amino acid biosynthetic process	17/7778	23/18670	0.0017506	0.0080211	0.004883
BP	GO:0030509	BMP signaling pathway	84/7778	157/18670	0.0017526	0.0080243	0.004885
BP	GO:0120163	negative regulation of cold-induced thermogenesis	30/7778	47/18670	0.0017723	0.0081088	0.004937
BP	GO:0039694	viral RNA genome replication	22/7778	32/18670	0.0017825	0.0081214	0.004945
BP	GO:0046685	response to arsenic-containing substance	22/7778	32/18670	0.0017825	0.0081214	0.004945
BP	GO:0050779	RNA destabilization	22/7778	32/18670	0.0017825	0.0081214	0.004945
BP	GO:0060674	placenta blood vessel development	22/7778	32/18670	0.0017825	0.0081214	0.004945
BP	GO:1905508	protein localization to microtubule organizing center	22/7778	32/18670	0.0017825	0.0081214	0.004945
BP	GO:0032496	response to lipopolysaccharide	164/7778	330/18670	0.0017826	0.0081214	0.004945
BP	GO:0051651	maintenance of location in cell	113/7778	219/18670	0.0017955	0.0081745	0.004977
BP	GO:0046486	glycerolipid metabolic process	202/7778	414/18670	0.0018043	0.0082085	0.004998
BP	GO:0044262	cellular carbohydrate metabolic process	142/7778	282/18670	0.0018355	0.0083446	0.00508
BP	GO:2001238	positive regulation of extrinsic apoptotic signaling pathway	31/7778	49/18670	0.0018435	0.008375	0.005099
BP	GO:0043280	positive regulation of cysteine-type endopeptidase activity involved ir	72/7778	132/18670	0.0018515	0.0083967	0.005112
BP	GO:0016485	protein processing	112/7778	217/18670	0.0018522	0.0083967	0.005112
BP	GO:0043624	cellular protein complex disassembly	112/7778	217/18670	0.0018522	0.0083967	0.005112
BP	GO:0032091	negative regulation of protein binding	58/7778	103/18670	0.0018623	0.0084366	0.005136
BP	GO:0046632	alpha-beta T cell differentiation	57/7778	101/18670	0.0018928	0.008569	0.005217
BP	GO:0042157	lipoprotein metabolic process	71/7778	130/18670	0.0018963	0.0085787	0.005223
BP	GO:0007159	leukocyte cell-cell adhesion	167/7778	337/18670	0.0019023	0.0085998	0.005236
BP	GO:0072527	pyrimidine-containing compound metabolic process	56/7778	99/18670	0.0019227	0.0086859	0.005288
BP	GO:0002576	platelet degranulation	70/7778	128/18670	0.0019417	0.0087487	0.005326
BP	GO:0048592	eye morphogenesis	80/7778	149/18670	0.0019527	0.0087487	0.005326
BP	GO:2001056	positive regulation of cysteine-type endopeptidase activity	80/7778	149/18670	0.0019527	0.0087487	0.005326
BP	GO:0000054	ribosomal subunit export from nucleus	11/7778	13/18670	0.0019543	0.0087487	0.005326
BP	GO:0007183	SMAD protein complex assembly	11/7778	13/18670	0.0019543	0.0087487	0.005326
BP	GO:0008298	intracellular mRNA localization	11/7778	13/18670	0.0019543	0.0087487	0.005326
BP	GO:0033750	ribosome localization	11/7778	13/18670	0.0019543	0.0087487	0.005326
BP	GO:0034982	mitochondrial protein processing	11/7778	13/18670	0.0019543	0.0087487	0.005326
BP	GO:0051315	attachment of mitotic spindle microtubules to kinetochore	11/7778	13/18670	0.0019543	0.0087487	0.005326
BP	GO:0060340	positive regulation of type I interferon-mediated signaling pathway	11/7778	13/18670	0.0019543	0.0087487	0.005326
BP	GO:0061029	eyelid development in camera-type eye	11/7778	13/18670	0.0019543	0.0087487	0.005326
BP	GO:1900103	positive regulation of endoplasmic reticulum unfolded protein respon	11/7778	13/18670	0.0019543	0.0087487	0.005326
BP	GO:1903960	negative regulation of anion transmembrane transport	11/7778	13/18670	0.0019543	0.0087487	0.005326
BP	GO:0031638	zymogen activation	33/7778	53/18670	0.0019621	0.008761	0.005334
BP	GO:0002183	cytoplasmic translational initiation	23/7778	34/18670	0.0019652	0.008761	0.005334
BP	GO:0018345	protein palmitoylation	23/7778	34/18670	0.0019652	0.008761	0.005334
BP	GO:0031128	developmental induction	23/7778	34/18670	0.0019652	0.008761	0.005334
BP	GO:0043276	anoikis	23/7778	34/18670	0.0019652	0.008761	0.005334
BP	GO:0062208	positive regulation of pattern recognition receptor signaling pathway	23/7778	34/18670	0.0019652	0.008761	0.005334
BP	GO:0060271	cilium assembly	180/7778	366/18670	0.0020042	0.008929	0.005436
BP	GO:0048704	embryonic skeletal system morphogenesis	53/7778	93/18670	0.0020068	0.008934	0.005439
BP	GO:0019218	regulation of steroid metabolic process	68/7778	124/18670	0.0020343	0.0090419	0.005505
BP	GO:0007221	positive regulation of transcription of Notch receptor target	14/7778	18/18670	0.0020394	0.0090419	0.005505
BP	GO:0031468	nuclear envelope reassembly	14/7778	18/18670	0.0020394	0.0090419	0.005505
BP	GO:1900153	positive regulation of nuclear-transcribed mRNA catabolic process, d	14/7778	18/18670	0.0020394	0.0090419	0.005505
BP	GO:1902165	regulation of intrinsic apoptotic signaling pathway in response to DN/	14/7778	18/18670	0.0020394	0.0090419	0.005505
BP	GO:2000757	negative regulation of peptidyl-lysine acetylation	14/7778	18/18670	0.0020394	0.0090419	0.005505
BP	GO:0043525	positive regulation of neuron apoptotic process	35/7778	57/18670	0.0020511	0.0090751	0.005525
BP	GO:0048008	platelet-derived growth factor receptor signaling pathway	35/7778	57/18670	0.0020511	0.0090751	0.005525
BP	GO:1901799	negative regulation of proteasomal protein catabolic process	35/7778	57/18670	0.0020511	0.0090751	0.005525
BP	GO:0072599	establishment of protein localization to endoplasmic reticulum	67/7778	122/18670	0.0020813	0.009202	0.005602
BP	GO:0001658	branching involved in ureteric bud morphogenesis	36/7778	59/18670	0.0020855	0.009202	0.005602
BP	GO:0033619	membrane protein proteolysis	36/7778	59/18670	0.0020855	0.009202	0.005602
BP	GO:0045071	negative regulation of viral genome replication	36/7778	59/18670	0.0020855	0.009202	0.005602
BP	GO:0001945	lymph vessel development	18/7778	25/18670	0.0020892	0.0092042	0.005604
BP	GO:0051894	positive regulation of focal adhesion assembly	18/7778	25/18670	0.0020892	0.0092042	0.005604
BP	GO:0050817	coagulation	169/7778	342/18670	0.0020903	0.0092042	0.005604
BP	GO:0009199	ribonucleoside triphosphate metabolic process	48/7778	83/18670	0.0021188	0.009317	0.005673
BP	GO:1903845	negative regulation of cellular response to transforming growth factor	48/7778	83/18670	0.0021188	0.009317	0.005673
BP	GO:0006007	glucose catabolic process	24/7778	36/18670	0.0021345	0.0093512	0.005693
BP	GO:0051973	positive regulation of telomerase activity	24/7778	36/18670	0.0021345	0.0093512	0.005693
BP	GO:0014032	neural crest cell development	47/7778	81/18670	0.0021353	0.0093512	0.005693
BP	GO:0030512	negative regulation of transforming growth factor beta receptor signa	47/7778	81/18670	0.0021353	0.0093512	0.005693

BP	GO:0033238	regulation of cellular amine metabolic process	47/7778	81/18670	0.0021353	0.0093512	0.005693
BP	GO:0034109	homotypic cell-cell adhesion	47/7778	81/18670	0.0021353	0.0093512	0.005693
BP	GO:0045652	regulation of megakaryocyte differentiation	46/7778	79/18670	0.0021493	0.009406	0.005727
BP	GO:0060675	ureteric bud morphogenesis	39/7778	65/18670	0.0021529	0.0094153	0.005732
BP	GO:0051298	centrosome duplication	40/7778	67/18670	0.0021647	0.0094606	0.00576
BP	GO:0006164	purine nucleotide biosynthetic process	100/7778	192/18670	0.0021769	0.0095077	0.005789
BP	GO:0042180	cellular ketone metabolic process	125/7778	246/18670	0.0022008	0.0096054	0.005848
BP	GO:0016572	histone phosphorylation	25/7778	38/18670	0.0022898	0.0099668	0.006068
BP	GO:0046326	positive regulation of glucose import	25/7778	38/18670	0.0022898	0.0099668	0.006068
BP	GO:0046676	negative regulation of insulin secretion	25/7778	38/18670	0.0022898	0.0099668	0.006068
BP	GO:2000279	negative regulation of DNA biosynthetic process	25/7778	38/18670	0.0022898	0.0099668	0.006068
BP	GO:0070304	positive regulation of stress-activated protein kinase signaling cascad	91/7778	173/18670	0.002292	0.0099696	0.00607
BP	GO:0001938	positive regulation of endothelial cell proliferation	62/7778	112/18670	0.0023224	0.0100951	0.006146
BP	GO:0002934	desmosome organization	9/7778	10/18670	0.0023574	0.010174	0.006194
BP	GO:0014041	regulation of neuron maturation	9/7778	10/18670	0.0023574	0.010174	0.006194
BP	GO:0032875	regulation of DNA endoreduplication	9/7778	10/18670	0.0023574	0.010174	0.006194
BP	GO:0034770	histone H4-K20 methylation	9/7778	10/18670	0.0023574	0.010174	0.006194
BP	GO:0035520	monoubiquitinated protein deubiquitination	9/7778	10/18670	0.0023574	0.010174	0.006194
BP	GO:1903405	protein localization to nuclear body	9/7778	10/18670	0.0023574	0.010174	0.006194
BP	GO:1903800	positive regulation of production of miRNAs involved in gene silencin	9/7778	10/18670	0.0023574	0.010174	0.006194
BP	GO:1904851	positive regulation of establishment of protein localization to telomere	9/7778	10/18670	0.0023574	0.010174	0.006194
BP	GO:1904867	protein localization to Cajal body	9/7778	10/18670	0.0023574	0.010174	0.006194
BP	GO:2000109	regulation of macrophage apoptotic process	9/7778	10/18670	0.0023574	0.010174	0.006194
BP	GO:0090501	RNA phosphodiester bond hydrolysis	82/7778	154/18670	0.0023579	0.010174	0.006194
BP	GO:0000188	inactivation of MAPK activity	19/7778	27/18670	0.0024127	0.0103965	0.00633
BP	GO:0071514	genetic imprinting	19/7778	27/18670	0.0024127	0.0103965	0.00633
BP	GO:0048639	positive regulation of developmental growth	96/7778	184/18670	0.0024585	0.0105865	0.006445
BP	GO:0071482	cellular response to light stimulus	70/7778	129/18670	0.0025336	0.0109027	0.006638
BP	GO:0043010	camera-type eye development	158/7778	319/18670	0.0025377	0.0109131	0.006644
BP	GO:0048489	synaptic vesicle transport	27/7778	42/18670	0.0025574	0.0109905	0.006691
BP	GO:0051321	meiotic cell cycle	126/7778	249/18670	0.0025622	0.011004	0.0067
BP	GO:2000379	positive regulation of reactive oxygen species metabolic process	57/7778	102/18670	0.0025665	0.0110148	0.006706
BP	GO:0002320	lymphoid progenitor cell differentiation	15/7778	20/18670	0.0026082	0.0111792	0.006806
BP	GO:0060602	branch elongation of an epithelium	15/7778	20/18670	0.0026082	0.0111792	0.006806
BP	GO:0060840	artery development	56/7778	100/18670	0.0026146	0.0111914	0.006814
BP	GO:0120034	positive regulation of plasma membrane bounded cell projection ass	56/7778	100/18670	0.0026146	0.0111914	0.006814
BP	GO:1901215	negative regulation of neuron death	107/7778	208/18670	0.0026482	0.0113279	0.006897
BP	GO:0030225	macrophage differentiation	28/7778	44/18670	0.0026701	0.0113836	0.006931
BP	GO:0034080	CENP-A containing nucleosome assembly	28/7778	44/18670	0.0026701	0.0113836	0.006931
BP	GO:0035307	positive regulation of protein dephosphorylation	28/7778	44/18670	0.0026701	0.0113836	0.006931
BP	GO:0060999	positive regulation of dendritic spine development	28/7778	44/18670	0.0026701	0.0113836	0.006931
BP	GO:0061641	CENP-A containing chromatin organization	28/7778	44/18670	0.0026701	0.0113836	0.006931
BP	GO:0099504	synaptic vesicle cycle	100/7778	193/18670	0.0026872	0.0114489	0.00697
BP	GO:0010950	positive regulation of endopeptidase activity	93/7778	178/18670	0.0026926	0.0114644	0.00698
BP	GO:0071322	cellular response to carbohydrate stimulus	77/7778	144/18670	0.0027101	0.011512	0.007009
BP	GO:1902107	positive regulation of leukocyte differentiation	77/7778	144/18670	0.0027101	0.011512	0.007009
BP	GO:0005979	regulation of glycogen biosynthetic process	20/7778	29/18670	0.0027163	0.011512	0.007009
BP	GO:0010962	regulation of glucan biosynthetic process	20/7778	29/18670	0.0027163	0.011512	0.007009
BP	GO:0031116	positive regulation of microtubule polymerization	20/7778	29/18670	0.0027163	0.011512	0.007009
BP	GO:0031365	N-terminal protein amino acid modification	20/7778	29/18670	0.0027163	0.011512	0.007009
BP	GO:1900181	negative regulation of protein localization to nucleus	20/7778	29/18670	0.0027163	0.011512	0.007009
BP	GO:0043491	protein kinase B signaling	135/7778	269/18670	0.0027367	0.0115908	0.007057
BP	GO:0000726	non-recombinational repair	53/7778	94/18670	0.0027549	0.0116452	0.00709
BP	GO:0070167	regulation of biomineral tissue development	53/7778	94/18670	0.0027549	0.0116452	0.00709
BP	GO:0110149	regulation of biomineralization	53/7778	94/18670	0.0027549	0.0116452	0.00709
BP	GO:0043631	RNA polyadenylation	29/7778	46/18670	0.0027692	0.0116902	0.007117
BP	GO:1990928	response to amino acid starvation	29/7778	46/18670	0.0027692	0.0116902	0.007117
BP	GO:0010811	positive regulation of cell-substrate adhesion	66/7778	121/18670	0.0027971	0.0117994	0.007184
BP	GO:0001819	positive regulation of cytokine production	223/7778	464/18670	0.0027987	0.0117994	0.007184
BP	GO:0046330	positive regulation of JNK cascade	75/7778	140/18670	0.0028631	0.012063	0.007344
BP	GO:0045921	positive regulation of exocytosis	50/7778	88/18670	0.0028857	0.0121501	0.007397
BP	GO:0001764	neuron migration	83/7778	157/18670	0.0028981	0.0121806	0.007416
BP	GO:0050870	positive regulation of T cell activation	104/7778	202/18670	0.0029099	0.0121806	0.007416
BP	GO:0002483	antigen processing and presentation of endogenous peptide antigen	12/7778	15/18670	0.0029099	0.0121806	0.007416
BP	GO:0019885	antigen processing and presentation of endogenous peptide antigen	12/7778	15/18670	0.0029099	0.0121806	0.007416
BP	GO:0031054	pre-miRNA processing	12/7778	15/18670	0.0029099	0.0121806	0.007416
BP	GO:0035067	negative regulation of histone acetylation	12/7778	15/18670	0.0029099	0.0121806	0.007416
BP	GO:0035635	entry of bacterium into host cell	12/7778	15/18670	0.0029099	0.0121806	0.007416
BP	GO:0090141	positive regulation of mitochondrial fission	12/7778	15/18670	0.0029099	0.0121806	0.007416
BP	GO:1903358	regulation of Golgi organization	12/7778	15/18670	0.0029099	0.0121806	0.007416
BP	GO:0042058	regulation of epidermal growth factor receptor signaling pathway	49/7778	86/18670	0.0029262	0.012238	0.007451
BP	GO:0031113	regulation of microtubule polymerization	31/7778	50/18670	0.0029294	0.012238	0.007451
BP	GO:0060976	coronary vasculature development	31/7778	50/18670	0.0029294	0.012238	0.007451
BP	GO:0032874	positive regulation of stress-activated MAPK cascade	90/7778	172/18670	0.0029482	0.0123088	0.007494
BP	GO:0002718	regulation of cytokine production involved in immune response	48/7778	84/18670	0.0029646	0.0123692	0.007531
BP	GO:1902373	negative regulation of mRNA catabolic process	32/7778	52/18670	0.0029918	0.0124551	0.007583
BP	GO:0002082	regulation of oxidative phosphorylation	21/7778	31/18670	0.0029968	0.0124551	0.007583
BP	GO:0007143	female meiotic nuclear division	21/7778	31/18670	0.0029968	0.0124551	0.007583
BP	GO:0034694	response to prostaglandin	21/7778	31/18670	0.0029968	0.0124551	0.007583
BP	GO:1902186	regulation of viral release from host cell	21/7778	31/18670	0.0029968	0.0124551	0.007583
BP	GO:2000781	positive regulation of double-strand break repair	21/7778	31/18670	0.0029968	0.0124551	0.007583
BP	GO:0007292	female gamete generation	73/7778	136/18670	0.0030233	0.0125571	0.007645
BP	GO:0140029	exocytic process	46/7778	80/18670	0.0030344	0.0125952	0.007668
BP	GO:0000271	polysaccharide biosynthetic process	45/7778	78/18670	0.0030651	0.0127145	0.007741
BP	GO:0072528	pyrimidine-containing compound biosynthetic process	34/7778	56/18670	0.0030848	0.0127879	0.007786
BP	GO:0046605	regulation of centrosome cycle	35/7778	58/18670	0.003117	0.0129128	0.007862
BP	GO:0006305	DNA alkylation	42/7778	72/18670	0.003136	0.0129666	0.007894
BP	GO:0006306	DNA methylation	42/7778	72/18670	0.003136	0.0129666	0.007894
BP	GO:0009123	nucleoside monophosphate metabolic process	42/7778	72/18670	0.003136	0.0129666	0.007894
BP	GO:0022037	metencephalon development	61/7778	111/18670	0.0031474	0.0129916	0.00791
BP	GO:0001756	somitogenesis	41/7778	70/18670	0.0031511	0.0129916	0.00791
BP	GO:0002260	lymphocyte homeostasis	37/7778	62/18670	0.003156	0.0129916	0.00791

BP	GO:0032835	glomerulus development	37/7778	62/18670	0.003156	0.0129916	0.00791
BP	GO:0045649	regulation of macrophage differentiation	16/7778	22/18670	0.0031582	0.0129916	0.00791
BP	GO:0071379	cellular response to prostaglandin stimulus	16/7778	22/18670	0.0031582	0.0129916	0.00791
BP	GO:0140112	extracellular vesicle biogenesis	16/7778	22/18670	0.0031582	0.0129916	0.00791
BP	GO:1901984	negative regulation of protein acetylation	16/7778	22/18670	0.0031582	0.0129916	0.00791
BP	GO:0030032	lamellipodium assembly	38/7778	64/18670	0.0031642	0.0129916	0.00791
BP	GO:0045600	positive regulation of fat cell differentiation	38/7778	64/18670	0.0031642	0.0129916	0.00791
BP	GO:0048278	vesicle docking	38/7778	64/18670	0.0031642	0.0129916	0.00791
BP	GO:0003158	endothelium development	71/7778	132/18670	0.0031907	0.0130919	0.007971
BP	GO:0006613	cotranslational protein targeting to membrane	60/7778	109/18670	0.0032196	0.0131939	0.008033
BP	GO:0051261	protein depolymerization	60/7778	109/18670	0.0032196	0.0131939	0.008033
BP	GO:0051216	cartilage development	107/7778	209/18670	0.0032264	0.0132133	0.008045
BP	GO:0009156	ribonucleoside monophosphate biosynthetic process	22/7778	33/18670	0.0032527	0.0132788	0.008085
BP	GO:0009303	rRNA transcription	22/7778	33/18670	0.0032527	0.0132788	0.008085
BP	GO:0031112	positive regulation of microtubule polymerization or depolymerization	22/7778	33/18670	0.0032527	0.0132788	0.008085
BP	GO:0051443	positive regulation of ubiquitin-protein transferase activity	22/7778	33/18670	0.0032527	0.0132788	0.008085
BP	GO:0071985	multivesicular body sorting pathway	22/7778	33/18670	0.0032527	0.0132788	0.008085
BP	GO:0097305	response to alcohol	118/7778	233/18670	0.0033123	0.0135134	0.008227
BP	GO:0044272	sulfur compound biosynthetic process	99/7778	192/18670	0.0034047	0.0138817	0.008452
BP	GO:0002237	response to molecule of bacterial origin	168/7778	343/18670	0.0034123	0.013904	0.008465
BP	GO:0006732	coenzyme metabolic process	128/7778	255/18670	0.0034295	0.0139651	0.008502
BP	GO:0006334	nucleosome assembly	77/7778	145/18670	0.0034467	0.0140262	0.00854
BP	GO:0003230	cardiac atrium development	23/7778	35/18670	0.0034834	0.01414	0.008609
BP	GO:0021955	central nervous system neuron axonogenesis	23/7778	35/18670	0.0034834	0.01414	0.008609
BP	GO:0051294	establishment of spindle orientation	23/7778	35/18670	0.0034834	0.01414	0.008609
BP	GO:0071108	protein K48-linked deubiquitination	23/7778	35/18670	0.0034834	0.01414	0.008609
BP	GO:0030316	osteoclast differentiation	54/7778	97/18670	0.0036599	0.0148377	0.009034
BP	GO:0032231	regulation of actin filament bundle assembly	54/7778	97/18670	0.0036599	0.0148377	0.009034
BP	GO:0022616	DNA strand elongation	17/7778	24/18670	0.0036758	0.0148742	0.009056
BP	GO:0034629	cellular protein-containing complex localization	17/7778	24/18670	0.0036758	0.0148742	0.009056
BP	GO:2000209	regulation of anoikis	17/7778	24/18670	0.0036758	0.0148742	0.009056
BP	GO:0006296	nucleotide-excision repair, DNA incision, 5'-to lesion	24/7778	37/18670	0.0036891	0.0148812	0.00906
BP	GO:0031111	negative regulation of microtubule polymerization or depolymerizatio	24/7778	37/18670	0.0036891	0.0148812	0.00906
BP	GO:0051567	histone H3-K9 methylation	24/7778	37/18670	0.0036891	0.0148812	0.00906
BP	GO:1901998	toxin transport	24/7778	37/18670	0.0036891	0.0148812	0.00906
BP	GO:1903432	regulation of TORC1 signaling	24/7778	37/18670	0.0036891	0.0148812	0.00906
BP	GO:0006520	cellular amino acid metabolic process	170/7778	348/18670	0.0037107	0.014959	0.009108
BP	GO:0045047	protein targeting to ER	64/7778	118/18670	0.0038331	0.0154427	0.009402
BP	GO:0071216	cellular response to biotic stimulus	119/7778	236/18670	0.0038444	0.0154682	0.009418
BP	GO:0061025	membrane fusion	81/7778	154/18670	0.003863	0.0154682	0.009418
BP	GO:0007076	mitotic chromosome condensation	13/7778	17/18670	0.0038659	0.0154682	0.009418
BP	GO:0033599	regulation of mammary gland epithelial cell proliferation	13/7778	17/18670	0.0038659	0.0154682	0.009418
BP	GO:0035313	wound healing, spreading of epidermal cells	13/7778	17/18670	0.0038659	0.0154682	0.009418
BP	GO:0035518	histone H2A monoubiquitination	13/7778	17/18670	0.0038659	0.0154682	0.009418
BP	GO:0061298	retina vasculature development in camera-type eye	13/7778	17/18670	0.0038659	0.0154682	0.009418
BP	GO:0062033	positive regulation of mitotic sister chromatid segregation	13/7778	17/18670	0.0038659	0.0154682	0.009418
BP	GO:1901524	regulation of mitophagy	13/7778	17/18670	0.0038659	0.0154682	0.009418
BP	GO:2000001	regulation of DNA damage checkpoint	13/7778	17/18670	0.0038659	0.0154682	0.009418
BP	GO:2000641	regulation of early endosome to late endosome transport	13/7778	17/18670	0.0038659	0.0154682	0.009418
BP	GO:1902116	negative regulation of organelle assembly	25/7778	39/18670	0.0038704	0.015477	0.009423
BP	GO:0071331	cellular response to hexose stimulus	72/7778	135/18670	0.0039733	0.0158783	0.009667
BP	GO:0002371	dendritic cell cytokine production	10/7778	12/18670	0.0040142	0.0159037	0.009683
BP	GO:0031581	hemidesmosome assembly	10/7778	12/18670	0.0040142	0.0159037	0.009683
BP	GO:0051988	regulation of attachment of spindle microtubules to kinetochore	10/7778	12/18670	0.0040142	0.0159037	0.009683
BP	GO:0060707	trophoblast giant cell differentiation	10/7778	12/18670	0.0040142	0.0159037	0.009683
BP	GO:0060900	embryonic camera-type eye formation	10/7778	12/18670	0.0040142	0.0159037	0.009683
BP	GO:0070601	centromeric sister chromatid cohesion	10/7778	12/18670	0.0040142	0.0159037	0.009683
BP	GO:0071481	cellular response to X-ray	10/7778	12/18670	0.0040142	0.0159037	0.009683
BP	GO:0072697	protein localization to cell cortex	10/7778	12/18670	0.0040142	0.0159037	0.009683
BP	GO:0097201	negative regulation of transcription from RNA polymerase II promote	10/7778	12/18670	0.0040142	0.0159037	0.009683
BP	GO:0097284	hepatocyte apoptotic process	10/7778	12/18670	0.0040142	0.0159037	0.009683
BP	GO:0098885	modification of postsynaptic actin cytoskeleton	10/7778	12/18670	0.0040142	0.0159037	0.009683
BP	GO:0098974	postsynaptic actin cytoskeleton organization	10/7778	12/18670	0.0040142	0.0159037	0.009683
BP	GO:1904816	positive regulation of protein localization to chromosome, telomeric r	10/7778	12/18670	0.0040142	0.0159037	0.009683
BP	GO:1990173	protein localization to nucleoplasm	10/7778	12/18670	0.0040142	0.0159037	0.009683
BP	GO:0045005	DNA-dependent DNA replication maintenance of fidelity	26/7778	41/18670	0.0040285	0.0159406	0.009705
BP	GO:0048286	lung alveolus development	26/7778	41/18670	0.0040285	0.0159406	0.009705
BP	GO:1903901	negative regulation of viral life cycle	48/7778	85/18670	0.0040862	0.016151	0.009833
BP	GO:0071333	cellular response to glucose stimulus	71/7778	133/18670	0.0040866	0.016151	0.009833
BP	GO:0010906	regulation of glucose metabolic process	61/7778	112/18670	0.0041341	0.0163287	0.009941
BP	GO:0045992	negative regulation of embryonic development	18/7778	26/18670	0.0041531	0.0163834	0.009975
BP	GO:1904353	regulation of telomere capping	18/7778	26/18670	0.0041531	0.0163834	0.009975
BP	GO:0010824	regulation of centrosome duplication	27/7778	43/18670	0.0041644	0.0163978	0.009984
BP	GO:0033173	calcineurin-NFAT signaling cascade	27/7778	43/18670	0.0041644	0.0163978	0.009984
BP	GO:0043616	keratinocyte proliferation	27/7778	43/18670	0.0041644	0.0163978	0.009984
BP	GO:0055072	iron ion homeostasis	46/7778	81/18670	0.0042161	0.0165915	0.010101
BP	GO:0003151	outflow tract morphogenesis	45/7778	79/18670	0.0042773	0.0168102	0.010235
BP	GO:0032456	endocytic recycling	28/7778	45/18670	0.0042795	0.0168102	0.010235
BP	GO:0035987	endodermal cell differentiation	28/7778	45/18670	0.0042795	0.0168102	0.010235
BP	GO:0050804	modulation of chemical synaptic transmission	209/7778	436/18670	0.0042964	0.0168663	0.010269
BP	GO:0035265	organ growth	104/7778	204/18670	0.0043072	0.0168984	0.010288
BP	GO:0030261	chromosome condensation	29/7778	47/18670	0.0043753	0.0171447	0.010438
BP	GO:1903580	positive regulation of ATP metabolic process	29/7778	47/18670	0.0043753	0.0171447	0.010438
BP	GO:0033002	muscle cell proliferation	120/7778	239/18670	0.0044426	0.0173977	0.010592
BP	GO:0050852	T cell receptor signaling pathway	103/7778	202/18670	0.0044477	0.0174071	0.010598
BP	GO:0097300	programmed necrotic cell death	30/7778	49/18670	0.0044532	0.0174182	0.010605
BP	GO:0031122	cytoplasmic microtubule organization	32/7778	53/18670	0.0045608	0.0178066	0.010841
BP	GO:0038066	p38MAPK cascade	32/7778	53/18670	0.0045608	0.0178066	0.010841
BP	GO:0098586	cellular response to virus	32/7778	53/18670	0.0045608	0.0178066	0.010841
BP	GO:0071168	protein localization to chromatin	19/7778	28/18670	0.0045859	0.0178938	0.010894
BP	GO:0016233	telomere capping	33/7778	55/18670	0.0045932	0.0179004	0.010898
BP	GO:0048016	inositol phosphate-mediated signaling	33/7778	55/18670	0.0045932	0.0179004	0.010898

BP	GO:0042982	amyloid precursor protein metabolic process	37/7778	63/18670	0.0046075	0.0179454	0.010926
BP	GO:0002218	activation of innate immune response	75/7778	142/18670	0.0046162	0.0179659	0.010938
BP	GO:0043388	positive regulation of DNA binding	35/7778	59/18670	0.0046212	0.0179659	0.010938
BP	GO:0071806	protein transmembrane transport	35/7778	59/18670	0.0046212	0.0179659	0.010938
BP	GO:0021549	cerebellum development	56/7778	102/18670	0.0046646	0.018124	0.011035
BP	GO:0035282	segmentation	55/7778	100/18670	0.0047742	0.0184482	0.011232
BP	GO:0006337	nucleosome disassembly	14/7778	19/18670	0.0047767	0.0184482	0.011232
BP	GO:0010888	negative regulation of lipid storage	14/7778	19/18670	0.0047767	0.0184482	0.011232
BP	GO:0034063	stress granule assembly	14/7778	19/18670	0.0047767	0.0184482	0.011232
BP	GO:0036303	lymph vessel morphogenesis	14/7778	19/18670	0.0047767	0.0184482	0.011232
BP	GO:0051546	keratinocyte migration	14/7778	19/18670	0.0047767	0.0184482	0.011232
BP	GO:0072074	kidney mesenchyme development	14/7778	19/18670	0.0047767	0.0184482	0.011232
BP	GO:0090201	negative regulation of release of cytochrome c from mitochondria	14/7778	19/18670	0.0047767	0.0184482	0.011232
BP	GO:1902176	negative regulation of oxidative stress-induced intrinsic apoptotic sig	14/7778	19/18670	0.0047767	0.0184482	0.011232
BP	GO:2000042	negative regulation of double-strand break repair via homologous rec	14/7778	19/18670	0.0047767	0.0184482	0.011232
BP	GO:1901861	regulation of muscle tissue development	81/7778	155/18670	0.0048257	0.0186262	0.01134
BP	GO:0099177	regulation of trans-synaptic signaling	209/7778	437/18670	0.004877	0.0188129	0.011454
BP	GO:0046631	alpha-beta T cell activation	73/7778	138/18670	0.0048962	0.0188757	0.011492
BP	GO:0001782	B cell homeostasis	20/7778	30/18670	0.0049731	0.019115	0.011638
BP	GO:0032607	interferon-alpha production	20/7778	30/18670	0.0049731	0.019115	0.011638
BP	GO:0044788	modulation by host of viral process	20/7778	30/18670	0.0049731	0.019115	0.011638
BP	GO:1901889	negative regulation of cell junction assembly	20/7778	30/18670	0.0049731	0.019115	0.011638
BP	GO:1904837	beta-catenin-TCF complex assembly	20/7778	30/18670	0.0049731	0.019115	0.011638
BP	GO:0071326	cellular response to monosaccharide stimulus	72/7778	136/18670	0.005042	0.0193682	0.011792
BP	GO:0002761	regulation of myeloid leukocyte differentiation	63/7778	117/18670	0.0050951	0.0195606	0.011909
BP	GO:1903039	positive regulation of leukocyte cell-cell adhesion	110/7778	218/18670	0.005145	0.0197405	0.012019
BP	GO:0032355	response to estradiol	71/7778	134/18670	0.0051917	0.0199079	0.012121
BP	GO:0051092	positive regulation of NF-kappaB transcription factor activity	78/7778	149/18670	0.0052893	0.0202698	0.012341
BP	GO:0046949	fatty-acyl-CoA biosynthetic process	21/7778	32/18670	0.005315	0.0203321	0.012379
BP	GO:0051085	chaperone cofactor-dependent protein refolding	21/7778	32/18670	0.005315	0.0203321	0.012379
BP	GO:0060325	face morphogenesis	21/7778	32/18670	0.005315	0.0203321	0.012379
BP	GO:0046883	regulation of hormone secretion	130/7778	262/18670	0.0053273	0.0203647	0.012399
BP	GO:0042475	odontogenesis of dentin-containing tooth	50/7778	90/18670	0.0053299	0.0203647	0.012399
BP	GO:0044782	cilium organization	185/7778	384/18670	0.0053453	0.0204004	0.01242
BP	GO:0055007	cardiac muscle cell differentiation	70/7778	132/18670	0.0053455	0.0204004	0.01242
BP	GO:0021987	cerebral cortex development	61/7778	113/18670	0.0053757	0.0205034	0.012483
BP	GO:0022404	molting cycle process	49/7778	88/18670	0.005441	0.0207157	0.012612
BP	GO:0022405	hair cycle process	49/7778	88/18670	0.005441	0.0207157	0.012612
BP	GO:2000177	regulation of neural precursor cell proliferation	49/7778	88/18670	0.005441	0.0207157	0.012612
BP	GO:1904019	epithelial cell apoptotic process	60/7778	111/18670	0.0055202	0.0210048	0.012788
BP	GO:0006099	tricarboxylic acid cycle	22/7778	34/18670	0.0056132	0.0212615	0.012945
BP	GO:0010765	positive regulation of sodium ion transport	22/7778	34/18670	0.0056132	0.0212615	0.012945
BP	GO:0018196	peptidyl-asparagine modification	22/7778	34/18670	0.0056132	0.0212615	0.012945
BP	GO:0033120	positive regulation of RNA splicing	22/7778	34/18670	0.0056132	0.0212615	0.012945
BP	GO:0045738	negative regulation of DNA repair	22/7778	34/18670	0.0056132	0.0212615	0.012945
BP	GO:0031498	chromatin disassembly	15/7778	21/18670	0.0056174	0.0212615	0.012945
BP	GO:0032986	protein-DNA complex disassembly	15/7778	21/18670	0.0056174	0.0212615	0.012945
BP	GO:0035162	embryonic hemopoiesis	15/7778	21/18670	0.0056174	0.0212615	0.012945
BP	GO:2000737	negative regulation of stem cell differentiation	15/7778	21/18670	0.0056174	0.0212615	0.012945
BP	GO:0006119	oxidative phosphorylation	76/7778	145/18670	0.0056219	0.0212662	0.012948
BP	GO:0071826	ribonucleoprotein complex subunit organization	118/7778	236/18670	0.0056363	0.0213082	0.012973
BP	GO:0006368	transcription elongation from RNA polymerase II promoter	47/7778	84/18670	0.005661	0.0213135	0.012976
BP	GO:0010595	positive regulation of endothelial cell migration	68/7778	128/18670	0.0056655	0.0213135	0.012976
BP	GO:0046606	negative regulation of centrosome cycle	11/7778	14/18670	0.0056708	0.0213135	0.012976
BP	GO:0051547	regulation of keratinocyte migration	11/7778	14/18670	0.0056708	0.0213135	0.012976
BP	GO:0051645	Golgi localization	11/7778	14/18670	0.0056708	0.0213135	0.012976
BP	GO:0099188	postsynaptic cytoskeleton organization	11/7778	14/18670	0.0056708	0.0213135	0.012976
BP	GO:0110154	RNA decapping	11/7778	14/18670	0.0056708	0.0213135	0.012976
BP	GO:0110156	methylguanosine-cap decapping	11/7778	14/18670	0.0056708	0.0213135	0.012976
BP	GO:1901722	regulation of cell proliferation involved in kidney development	11/7778	14/18670	0.0056708	0.0213135	0.012976
BP	GO:1904814	regulation of protein localization to chromosome, telomeric region	11/7778	14/18670	0.0056708	0.0213135	0.012976
BP	GO:0006361	transcription initiation from RNA polymerase I promoter	23/7778	36/18670	0.0058699	0.0220491	0.013424
BP	GO:0008593	regulation of Notch signaling pathway	57/7778	105/18670	0.0059699	0.0223983	0.013637
BP	GO:0032006	regulation of TOR signaling	57/7778	105/18670	0.0059699	0.0223983	0.013637
BP	GO:0010827	regulation of glucose transmembrane transport	44/7778	78/18670	0.0059791	0.0224067	0.013642
BP	GO:0021954	central nervous system neuron development	44/7778	78/18670	0.0059791	0.0224067	0.013642
BP	GO:0071695	anatomical structure maturation	116/7778	232/18670	0.0060158	0.0225314	0.013718
BP	GO:0045454	cell redox homeostasis	43/7778	76/18670	0.00608	0.0227484	0.01385
BP	GO:0006084	acetyl-CoA metabolic process	24/7778	38/18670	0.0060879	0.0227484	0.01385
BP	GO:0007223	Wnt signaling pathway, calcium modulating pathway	24/7778	38/18670	0.0060879	0.0227484	0.01385
BP	GO:0046329	negative regulation of JNK cascade	24/7778	38/18670	0.0060879	0.0227484	0.01385
BP	GO:0021543	pallium development	87/7778	169/18670	0.0060965	0.0227674	0.013862
BP	GO:0007584	response to nutrient	110/7778	219/18670	0.0061497	0.0229527	0.013974
BP	GO:1903532	positive regulation of secretion by cell	193/7778	403/18670	0.0061721	0.023023	0.014017
BP	GO:1900076	regulation of cellular response to insulin stimulus	42/7778	74/18670	0.0061774	0.0230293	0.014021
BP	GO:0008156	negative regulation of DNA replication	25/7778	40/18670	0.0062699	0.0233474	0.014215
BP	GO:0090184	positive regulation of kidney development	25/7778	40/18670	0.0062699	0.0233474	0.014215
BP	GO:0022408	negative regulation of cell-cell adhesion	92/7778	180/18670	0.0063364	0.023581	0.014357
BP	GO:0010675	regulation of cellular carbohydrate metabolic process	72/7778	137/18670	0.0063484	0.0236045	0.014371
BP	GO:0072089	stem cell proliferation	64/7778	120/18670	0.0063568	0.0236045	0.014371
BP	GO:0034121	regulation of toll-like receptor signaling pathway	40/7778	70/18670	0.0063589	0.0236045	0.014371
BP	GO:0016202	regulation of striated muscle tissue development	79/7778	152/18670	0.0063749	0.0236045	0.014371
BP	GO:0006297	nucleotide-excision repair, DNA gap filling	16/7778	23/18670	0.0063756	0.0236045	0.014371
BP	GO:0016578	histone deubiquitination	16/7778	23/18670	0.0063756	0.0236045	0.014371
BP	GO:0090140	regulation of mitochondrial fission	16/7778	23/18670	0.0063756	0.0236045	0.014371
BP	GO:1903429	regulation of cell maturation	16/7778	23/18670	0.0063756	0.0236045	0.014371
BP	GO:2000050	regulation of non-canonical Wnt signaling pathway	16/7778	23/18670	0.0063756	0.0236045	0.014371
BP	GO:0006509	membrane protein ectodomain proteolysis	26/7778	42/18670	0.0064189	0.0237238	0.014444
BP	GO:0010559	regulation of glycoprotein biosynthetic process	26/7778	42/18670	0.0064189	0.0237238	0.014444
BP	GO:0090503	RNA phosphodiester bond hydrolysis, exonucleolytic	26/7778	42/18670	0.0064189	0.0237238	0.014444
BP	GO:0010812	negative regulation of cell-substrate adhesion	39/7778	68/18670	0.0064414	0.0237932	0.014486
BP	GO:0030072	peptide hormone secretion	124/7778	250/18670	0.0064741	0.0239004	0.014551

BP	GO:0051785	positive regulation of nuclear division	38/7778	66/18670	0.0065172	0.0240223	0.014626
BP	GO:0003197	endocardial cushion development	27/7778	44/18670	0.0065376	0.0240223	0.014626
BP	GO:0006378	mRNA polyadenylation	27/7778	44/18670	0.0065376	0.0240223	0.014626
BP	GO:0009124	nucleoside monophosphate biosynthetic process	27/7778	44/18670	0.0065376	0.0240223	0.014626
BP	GO:0010828	positive regulation of glucose transmembrane transport	27/7778	44/18670	0.0065376	0.0240223	0.014626
BP	GO:0045910	negative regulation of DNA recombination	27/7778	44/18670	0.0065376	0.0240223	0.014626
BP	GO:0070266	necroptotic process	27/7778	44/18670	0.0065376	0.0240223	0.014626
BP	GO:0070897	transcription preinitiation complex assembly	27/7778	44/18670	0.0065376	0.0240223	0.014626
BP	GO:0048593	camera-type eye morphogenesis	63/7778	118/18670	0.0065407	0.0240223	0.014626
BP	GO:0043297	apical junction assembly	37/7778	64/18670	0.0065854	0.024159	0.014709
BP	GO:0043550	regulation of lipid kinase activity	37/7778	64/18670	0.0065854	0.024159	0.014709
BP	GO:0042775	mitochondrial ATP synthesis coupled electron transport	53/7778	97/18670	0.0066041	0.0242137	0.014742
BP	GO:0006221	pyrimidine nucleotide biosynthetic process	28/7778	46/18670	0.0066289	0.0242357	0.014755
BP	GO:0032527	protein exit from endoplasmic reticulum	28/7778	46/18670	0.0066289	0.0242357	0.014755
BP	GO:0035088	establishment or maintenance of apical/basal cell polarity	28/7778	46/18670	0.0066289	0.0242357	0.014755
BP	GO:0061245	establishment or maintenance of bipolar cell polarity	28/7778	46/18670	0.0066289	0.0242357	0.014755
BP	GO:0071675	regulation of mononuclear cell migration	28/7778	46/18670	0.0066289	0.0242357	0.014755
BP	GO:0002562	somatic diversification of immune receptors via germline recombinati	36/7778	62/18670	0.0066448	0.0242663	0.014774
BP	GO:0016444	somatic cell DNA recombination	36/7778	62/18670	0.0066448	0.0242663	0.014774
BP	GO:0046324	regulation of glucose import	35/7778	60/18670	0.0066942	0.0244189	0.014867
BP	GO:1902808	positive regulation of cell cycle G1/S phase transition	35/7778	60/18670	0.0066942	0.0244189	0.014867
BP	GO:0030520	intracellular estrogen receptor signaling pathway	34/7778	58/18670	0.0067322	0.0245436	0.014943
BP	GO:0001706	endoderm formation	30/7778	50/18670	0.006739	0.0245548	0.01495
BP	GO:1902017	regulation of cilium assembly	33/7778	56/18670	0.0067574	0.024605	0.01498
BP	GO:2000677	regulation of transcription regulatory region DNA binding	31/7778	52/18670	0.0067626	0.024605	0.01498
BP	GO:0002702	positive regulation of production of molecular mediator of immune re	52/7778	95/18670	0.006768	0.024605	0.01498
BP	GO:0002720	positive regulation of cytokine production involved in immune respon	32/7778	54/18670	0.0067681	0.024605	0.01498
BP	GO:0045446	endothelial cell differentiation	61/7778	114/18670	0.006922	0.0251502	0.015312
BP	GO:0055123	digestive system development	76/7778	146/18670	0.0070017	0.0254255	0.01548
BP	GO:0007026	negative regulation of microtubule depolymerization	17/7778	25/18670	0.0070475	0.0255055	0.015529
BP	GO:0008334	histone mRNA metabolic process	17/7778	25/18670	0.0070475	0.0255055	0.015529
BP	GO:0009299	mRNA transcription	17/7778	25/18670	0.0070475	0.0255055	0.015529
BP	GO:0035459	vesicle cargo loading	17/7778	25/18670	0.0070475	0.0255055	0.015529
BP	GO:0046856	phosphatidylinositol dephosphorylation	17/7778	25/18670	0.0070475	0.0255055	0.015529
BP	GO:2000104	negative regulation of DNA-dependent DNA replication	17/7778	25/18670	0.0070475	0.0255055	0.015529
BP	GO:0045639	positive regulation of myeloid cell differentiation	50/7778	91/18670	0.0071008	0.0256841	0.015637
BP	GO:0007626	locomotory behavior	100/7778	198/18670	0.0071252	0.0257578	0.015682
BP	GO:0007043	cell-cell junction assembly	68/7778	129/18670	0.0071634	0.0258816	0.015758
BP	GO:0006086	acetyl-CoA biosynthetic process from pyruvate	12/7778	16/18670	0.0072205	0.0259568	0.015803
BP	GO:0044794	positive regulation by host of viral process	12/7778	16/18670	0.0072205	0.0259568	0.015803
BP	GO:0044827	modulation by host of viral genome replication	12/7778	16/18670	0.0072205	0.0259568	0.015803
BP	GO:0055119	relaxation of cardiac muscle	12/7778	16/18670	0.0072205	0.0259568	0.015803
BP	GO:0061684	chaperone-mediated autophagy	12/7778	16/18670	0.0072205	0.0259568	0.015803
BP	GO:0072176	nephric duct development	12/7778	16/18670	0.0072205	0.0259568	0.015803
BP	GO:0080182	histone H3-K4 trimethylation	12/7778	16/18670	0.0072205	0.0259568	0.015803
BP	GO:0097067	cellular response to thyroid hormone stimulus	12/7778	16/18670	0.0072205	0.0259568	0.015803
BP	GO:1904294	positive regulation of ERAD pathway	12/7778	16/18670	0.0072205	0.0259568	0.015803
BP	GO:0006024	glycosaminoglycan biosynthetic process	59/7778	110/18670	0.0073215	0.0263053	0.016016
BP	GO:0030073	insulin secretion	104/7778	207/18670	0.0074821	0.0268673	0.016358
BP	GO:0009144	purine nucleoside triphosphate metabolic process	47/7778	85/18670	0.0076088	0.027275	0.016606
BP	GO:0009798	axis specification	47/7778	85/18670	0.0076088	0.027275	0.016606
BP	GO:0006735	NADH regeneration	18/7778	27/18670	0.0076338	0.027275	0.016606
BP	GO:0032201	telomere maintenance via semi-conservative replication	18/7778	27/18670	0.0076338	0.027275	0.016606
BP	GO:0034067	protein localization to Golgi apparatus	18/7778	27/18670	0.0076338	0.027275	0.016606
BP	GO:0039528	cytoplasmic pattern recognition receptor signaling pathway in respon	18/7778	27/18670	0.0076338	0.027275	0.016606
BP	GO:0061621	canonical glycolysis	18/7778	27/18670	0.0076338	0.027275	0.016606
BP	GO:0061718	glucose catabolic process to pyruvate	18/7778	27/18670	0.0076338	0.027275	0.016606
BP	GO:0070199	establishment of protein localization to chromosome	18/7778	27/18670	0.0076338	0.027275	0.016606
BP	GO:0002062	chondrocyte differentiation	65/7778	123/18670	0.0078381	0.0279894	0.017041
BP	GO:0030168	platelet activation	79/7778	153/18670	0.0078686	0.0280829	0.017098
BP	GO:0008064	regulation of actin polymerization or depolymerization	91/7778	179/18670	0.0079353	0.0283054	0.017233
BP	GO:0010923	negative regulation of phosphatase activity	56/7778	104/18670	0.0079552	0.0283605	0.017267
BP	GO:0001570	vasculogenesis	44/7778	79/18670	0.0081184	0.0285861	0.017404
BP	GO:0000727	double-strand break repair via break-induced replication	9/7778	11/18670	0.0081368	0.0285861	0.017404
BP	GO:0002730	regulation of dendritic cell cytokine production	9/7778	11/18670	0.0081368	0.0285861	0.017404
BP	GO:0019042	viral latency	9/7778	11/18670	0.0081368	0.0285861	0.017404
BP	GO:0030422	production of siRNA involved in RNA interference	9/7778	11/18670	0.0081368	0.0285861	0.017404
BP	GO:0032060	bleb assembly	9/7778	11/18670	0.0081368	0.0285861	0.017404
BP	GO:0033148	positive regulation of intracellular estrogen receptor signaling pathwa	9/7778	11/18670	0.0081368	0.0285861	0.017404
BP	GO:0035404	histone-serine phosphorylation	9/7778	11/18670	0.0081368	0.0285861	0.017404
BP	GO:0043619	regulation of transcription from RNA polymerase II promoter in respo	9/7778	11/18670	0.0081368	0.0285861	0.017404
BP	GO:0045793	positive regulation of cell size	9/7778	11/18670	0.0081368	0.0285861	0.017404
BP	GO:0048096	chromatin-mediated maintenance of transcription	9/7778	11/18670	0.0081368	0.0285861	0.017404
BP	GO:0061307	cardiac neural crest cell differentiation involved in heart development	9/7778	11/18670	0.0081368	0.0285861	0.017404
BP	GO:0061308	cardiac neural crest cell development involved in heart development	9/7778	11/18670	0.0081368	0.0285861	0.017404
BP	GO:0061314	Notch signaling involved in heart development	9/7778	11/18670	0.0081368	0.0285861	0.017404
BP	GO:0070203	regulation of establishment of protein localization to telomere	9/7778	11/18670	0.0081368	0.0285861	0.017404
BP	GO:1900102	negative regulation of endoplasmic reticulum unfolded protein respor	9/7778	11/18670	0.0081368	0.0285861	0.017404
BP	GO:1901096	regulation of autophagosome maturation	9/7778	11/18670	0.0081368	0.0285861	0.017404
BP	GO:2000786	positive regulation of autophagosome assembly	9/7778	11/18670	0.0081368	0.0285861	0.017404
BP	GO:2001241	positive regulation of extrinsic apoptotic signaling pathway in absenc	9/7778	11/18670	0.0081368	0.0285861	0.017404
BP	GO:0010575	positive regulation of vascular endothelial growth factor production	19/7778	29/18670	0.0081383	0.0285861	0.017404
BP	GO:0014044	Schwann cell development	19/7778	29/18670	0.0081383	0.0285861	0.017404
BP	GO:0030970	retrograde protein transport, ER to cytosol	19/7778	29/18670	0.0081383	0.0285861	0.017404
BP	GO:0031063	regulation of histone deacetylation	19/7778	29/18670	0.0081383	0.0285861	0.017404
BP	GO:0060441	epithelial tube branching involved in lung morphogenesis	19/7778	29/18670	0.0081383	0.0285861	0.017404
BP	GO:0061099	negative regulation of protein tyrosine kinase activity	19/7778	29/18670	0.0081383	0.0285861	0.017404
BP	GO:0090162	establishment of epithelial cell polarity	19/7778	29/18670	0.0081383	0.0285861	0.017404
BP	GO:1903513	endoplasmic reticulum to cytosol transport	19/7778	29/18670	0.0081383	0.0285861	0.017404
BP	GO:0048332	mesoderm morphogenesis	43/7778	77/18670	0.0082865	0.0290907	0.017711
BP	GO:0051047	positive regulation of secretion	203/7778	428/18670	0.0084355	0.0295978	0.01802
BP	GO:0002200	somatic diversification of immune receptors	42/7778	75/18670	0.0084526	0.0296415	0.018047

BP	GO:0007411	axon guidance	135/7778	276/18670	0.0084578	0.0296438	0.018048
BP	GO:0006482	protein demethylation	20/7778	31/18670	0.0085664	0.0299812	0.018254
BP	GO:0008214	protein dealkylation	20/7778	31/18670	0.0085664	0.0299812	0.018254
BP	GO:0002902	regulation of B cell apoptotic process	13/7778	18/18670	0.0086146	0.0299812	0.018254
BP	GO:0006349	regulation of gene expression by genetic imprinting	13/7778	18/18670	0.0086146	0.0299812	0.018254
BP	GO:0007252	I-kappaB phosphorylation	13/7778	18/18670	0.0086146	0.0299812	0.018254
BP	GO:0009048	dosage compensation by inactivation of X chromosome	13/7778	18/18670	0.0086146	0.0299812	0.018254
BP	GO:0010766	negative regulation of sodium ion transport	13/7778	18/18670	0.0086146	0.0299812	0.018254
BP	GO:0019054	modulation by virus of host cellular process	13/7778	18/18670	0.0086146	0.0299812	0.018254
BP	GO:0021756	striatum development	13/7778	18/18670	0.0086146	0.0299812	0.018254
BP	GO:0031293	membrane protein intracellular domain proteolysis	13/7778	18/18670	0.0086146	0.0299812	0.018254
BP	GO:0035994	response to muscle stretch	13/7778	18/18670	0.0086146	0.0299812	0.018254
BP	GO:0043555	regulation of translation in response to stress	13/7778	18/18670	0.0086146	0.0299812	0.018254
BP	GO:0061323	cell proliferation involved in heart morphogenesis	13/7778	18/18670	0.0086146	0.0299812	0.018254
BP	GO:0042773	ATP synthesis coupled electron transport	53/7778	98/18670	0.0086294	0.0300003	0.018265
BP	GO:0045807	positive regulation of endocytosis	53/7778	98/18670	0.0086294	0.0300003	0.018265
BP	GO:0033692	cellular polysaccharide biosynthetic process	40/7778	71/18670	0.008776	0.0304935	0.018565
BP	GO:0006023	aminoglycan biosynthetic process	61/7778	115/18670	0.0088288	0.0306603	0.018667
BP	GO:0010039	response to iron ion	21/7778	33/18670	0.008924	0.0309077	0.018818
BP	GO:0018279	protein N-linked glycosylation via asparagine	21/7778	33/18670	0.008924	0.0309077	0.018818
BP	GO:0030513	positive regulation of BMP signaling pathway	21/7778	33/18670	0.008924	0.0309077	0.018818
BP	GO:0036314	response to sterol	21/7778	33/18670	0.008924	0.0309077	0.018818
BP	GO:0048536	spleen development	21/7778	33/18670	0.008924	0.0309077	0.018818
BP	GO:1902414	protein localization to cell junction	51/7778	94/18670	0.0091003	0.0314846	0.019169
BP	GO:1990542	mitochondrial transmembrane transport	51/7778	94/18670	0.0091003	0.0314846	0.019169
BP	GO:0030198	extracellular matrix organization	176/7778	368/18670	0.0091586	0.0316691	0.019281
BP	GO:0000266	mitochondrial fission	22/7778	35/18670	0.0092174	0.0318213	0.019374
BP	GO:0003203	endocardial cushion morphogenesis	22/7778	35/18670	0.0092174	0.0318213	0.019374
BP	GO:0034142	toll-like receptor 4 signaling pathway	22/7778	35/18670	0.0092174	0.0318213	0.019374
BP	GO:0060389	pathway-restricted SMAD protein phosphorylation	37/7778	65/18670	0.0092254	0.0318317	0.01938
BP	GO:0048634	regulation of muscle organ development	80/7778	156/18670	0.0093408	0.0322129	0.019612
BP	GO:0046637	regulation of alpha-beta T cell differentiation	36/7778	63/18670	0.0093611	0.0322311	0.019623
BP	GO:0090181	regulation of cholesterol metabolic process	36/7778	63/18670	0.0093611	0.0322311	0.019623
BP	GO:1904377	positive regulation of protein localization to cell periphery	36/7778	63/18670	0.0093611	0.0322311	0.019623
BP	GO:0007431	salivary gland development	23/7778	37/18670	0.0094527	0.0325117	0.019794
BP	GO:0071542	dopaminergic neuron differentiation	23/7778	37/18670	0.0094527	0.0325117	0.019794
BP	GO:0007040	lysosome organization	35/7778	61/18670	0.0094876	0.0325794	0.019835
BP	GO:0042733	embryonic digit morphogenesis	35/7778	61/18670	0.0094876	0.0325794	0.019835
BP	GO:0080171	lytic vacuole organization	35/7778	61/18670	0.0094876	0.0325794	0.019835
BP	GO:0030832	regulation of actin filament length	91/7778	180/18670	0.0095703	0.0328459	0.019998
BP	GO:0010717	regulation of epithelial to mesenchymal transition	49/7778	90/18670	0.0095871	0.0328862	0.020022
BP	GO:0001885	endothelial cell development	34/7778	59/18670	0.0096031	0.0329061	0.020034
BP	GO:0070527	platelet aggregation	34/7778	59/18670	0.0096031	0.0329061	0.020034
BP	GO:0033146	regulation of intracellular estrogen receptor signaling pathway	24/7778	39/18670	0.0096359	0.0329306	0.020049
BP	GO:0033683	nucleotide-excision repair, DNA incision	24/7778	39/18670	0.0096359	0.0329306	0.020049
BP	GO:0038179	neurotrophin signaling pathway	24/7778	39/18670	0.0096359	0.0329306	0.020049
BP	GO:0043114	regulation of vascular permeability	24/7778	39/18670	0.0096359	0.0329306	0.020049
BP	GO:0050856	regulation of T cell receptor signaling pathway	24/7778	39/18670	0.0096359	0.0329306	0.020049
BP	GO:0035337	fatty-acyl-CoA metabolic process	25/7778	41/18670	0.0097723	0.0332634	0.020252
BP	GO:0001541	ovarian follicle development	32/7778	55/18670	0.0097941	0.0332634	0.020252
BP	GO:0006383	transcription by RNA polymerase III	32/7778	55/18670	0.0097941	0.0332634	0.020252
BP	GO:0097479	synaptic vesicle localization	32/7778	55/18670	0.0097941	0.0332634	0.020252
BP	GO:0097485	neuron projection guidance	135/7778	277/18670	0.009805	0.0332634	0.020252
BP	GO:0098773	skin epidermis development	48/7778	88/18670	0.0098359	0.0332634	0.020252
BP	GO:0007549	dosage compensation	14/7778	20/18670	0.0098365	0.0332634	0.020252
BP	GO:0010560	positive regulation of glycoprotein biosynthetic process	14/7778	20/18670	0.0098365	0.0332634	0.020252
BP	GO:0034143	regulation of toll-like receptor 4 signaling pathway	14/7778	20/18670	0.0098365	0.0332634	0.020252
BP	GO:0039529	RIG-I signaling pathway	14/7778	20/18670	0.0098365	0.0332634	0.020252
BP	GO:0043371	negative regulation of CD4-positive, alpha-beta T cell differentiation	14/7778	20/18670	0.0098365	0.0332634	0.020252
BP	GO:0048745	smooth muscle tissue development	14/7778	20/18670	0.0098365	0.0332634	0.020252
BP	GO:0060039	pericardium development	14/7778	20/18670	0.0098365	0.0332634	0.020252
BP	GO:0060216	definitive hemopoiesis	14/7778	20/18670	0.0098365	0.0332634	0.020252
BP	GO:0060261	positive regulation of transcription initiation from RNA polymerase II	14/7778	20/18670	0.0098365	0.0332634	0.020252
BP	GO:0070262	peptidyl-serine dephosphorylation	14/7778	20/18670	0.0098365	0.0332634	0.020252
BP	GO:0070932	histone H3 deacetylation	14/7778	20/18670	0.0098365	0.0332634	0.020252
BP	GO:0097062	dendritic spine maintenance	14/7778	20/18670	0.0098365	0.0332634	0.020252
BP	GO:0097734	extracellular exosome biogenesis	14/7778	20/18670	0.0098365	0.0332634	0.020252
BP	GO:1901673	regulation of mitotic spindle assembly	14/7778	20/18670	0.0098365	0.0332634	0.020252
BP	GO:0032881	regulation of polysaccharide metabolic process	26/7778	43/18670	0.009867	0.0332967	0.020272
BP	GO:0038202	TORC1 signaling	26/7778	43/18670	0.009867	0.0332967	0.020272
BP	GO:0045197	establishment or maintenance of epithelial cell apical/basal polarity	26/7778	43/18670	0.009867	0.0332967	0.020272
BP	GO:0060612	adipose tissue development	26/7778	43/18670	0.009867	0.0332967	0.020272
BP	GO:0001954	positive regulation of cell-matrix adhesion	30/7778	51/18670	0.0099168	0.0333868	0.020327
BP	GO:0032007	negative regulation of TOR signaling	30/7778	51/18670	0.0099168	0.0333868	0.020327
BP	GO:0045599	negative regulation of fat cell differentiation	30/7778	51/18670	0.0099168	0.0333868	0.020327
BP	GO:0031018	endocrine pancreas development	27/7778	45/18670	0.0099248	0.0333868	0.020327
BP	GO:0044275	cellular carbohydrate catabolic process	27/7778	45/18670	0.0099248	0.0333868	0.020327
BP	GO:0048701	embryonic cranial skeleton morphogenesis	27/7778	45/18670	0.0099248	0.0333868	0.020327
BP	GO:0065002	intracellular protein transmembrane transport	29/7778	49/18670	0.009946	0.0334013	0.020336
BP	GO:1903018	regulation of glycoprotein metabolic process	29/7778	49/18670	0.009946	0.0334013	0.020336
BP	GO:0061383	trabecula morphogenesis	28/7778	47/18670	0.0099499	0.0334013	0.020336
BP	GO:0070231	T cell apoptotic process	28/7778	47/18670	0.0099499	0.0334013	0.020336
BP	GO:0001942	hair follicle development	47/7778	86/18670	0.010088	0.0338121	0.020586
BP	GO:0006919	activation of cysteine-type endopeptidase activity involved in apoptot	47/7778	86/18670	0.010088	0.0338121	0.020586
BP	GO:0034637	cellular carbohydrate biosynthetic process	47/7778	86/18670	0.010088	0.0338121	0.020586
BP	GO:0051896	regulation of protein kinase B signaling	120/7778	244/18670	0.0101443	0.0339832	0.02069
BP	GO:0006614	SRP-dependent cotranslational protein targeting to membrane	56/7778	105/18670	0.0102242	0.0342329	0.020842
BP	GO:0043062	extracellular structure organization	176/7778	369/18670	0.0103877	0.0347623	0.021164
BP	GO:0050921	positive regulation of chemotaxis	70/7778	135/18670	0.0104874	0.0350777	0.021356
BP	GO:0008154	actin polymerization or depolymerization	104/7778	209/18670	0.0105654	0.0353202	0.021504
BP	GO:0051494	negative regulation of cytoskeleton organization	76/7778	148/18670	0.0106363	0.0355391	0.021637
BP	GO:0007224	smoothened signaling pathway	69/7778	133/18670	0.0108275	0.0359475	0.021886

BP	GO:0055076	transition metal ion homeostasis	69/7778	133/18670	0.0108275	0.0359475	0.021886
BP	GO:0048259	regulation of receptor-mediated endocytosis	54/7778	101/18670	0.0108336	0.0359475	0.021886
BP	GO:0048661	positive regulation of smooth muscle cell proliferation	54/7778	101/18670	0.0108336	0.0359475	0.021886
BP	GO:0000212	meiotic spindle organization	10/7778	13/18670	0.0108806	0.0359475	0.021886
BP	GO:0001833	inner cell mass cell proliferation	10/7778	13/18670	0.0108806	0.0359475	0.021886
BP	GO:0010745	negative regulation of macrophage derived foam cell differentiation	10/7778	13/18670	0.0108806	0.0359475	0.021886
BP	GO:0010763	positive regulation of fibroblast migration	10/7778	13/18670	0.0108806	0.0359475	0.021886
BP	GO:0033127	regulation of histone phosphorylation	10/7778	13/18670	0.0108806	0.0359475	0.021886
BP	GO:0033145	positive regulation of intracellular steroid hormone receptor signaling	10/7778	13/18670	0.0108806	0.0359475	0.021886
BP	GO:0034214	protein hexamerization	10/7778	13/18670	0.0108806	0.0359475	0.021886
BP	GO:0046831	regulation of RNA export from nucleus	10/7778	13/18670	0.0108806	0.0359475	0.021886
BP	GO:0071696	ectodermal placode development	10/7778	13/18670	0.0108806	0.0359475	0.021886
BP	GO:0090239	regulation of histone H4 acetylation	10/7778	13/18670	0.0108806	0.0359475	0.021886
BP	GO:0098787	mRNA cleavage involved in mRNA processing	10/7778	13/18670	0.0108806	0.0359475	0.021886
BP	GO:0099640	axo-dendritic protein transport	10/7778	13/18670	0.0108806	0.0359475	0.021886
BP	GO:1902894	negative regulation of pri-miRNA transcription by RNA polymerase II	10/7778	13/18670	0.0108806	0.0359475	0.021886
BP	GO:0006085	acetyl-CoA biosynthetic process	15/7778	22/18670	0.0108869	0.0359475	0.021886
BP	GO:0006379	mRNA cleavage	15/7778	22/18670	0.0108869	0.0359475	0.021886
BP	GO:0008053	mitochondrial fusion	15/7778	22/18670	0.0108869	0.0359475	0.021886
BP	GO:0050765	negative regulation of phagocytosis	15/7778	22/18670	0.0108869	0.0359475	0.021886
BP	GO:1903077	negative regulation of protein localization to plasma membrane	15/7778	22/18670	0.0108869	0.0359475	0.021886
BP	GO:1904666	regulation of ubiquitin protein ligase activity	15/7778	22/18670	0.0108869	0.0359475	0.021886
BP	GO:0006487	protein N-linked glycosylation	43/7778	78/18670	0.0111213	0.0367026	0.022346
BP	GO:0030258	lipid modification	117/7778	238/18670	0.0111887	0.036906	0.02247
BP	GO:0006486	protein glycosylation	126/7778	258/18670	0.0113138	0.0372806	0.022698
BP	GO:0043413	macromolecule glycosylation	126/7778	258/18670	0.0113138	0.0372806	0.022698
BP	GO:0051147	regulation of muscle cell differentiation	91/7778	181/18670	0.0114807	0.0378112	0.023021
BP	GO:0060349	bone morphogenesis	60/7778	114/18670	0.0115064	0.0378764	0.02306
BP	GO:0070373	negative regulation of ERK1 and ERK2 cascade	41/7778	74/18670	0.0116459	0.0383159	0.023328
BP	GO:0001919	regulation of receptor recycling	16/7778	24/18670	0.0117752	0.038447	0.023408
BP	GO:0003181	atrioventricular valve morphogenesis	16/7778	24/18670	0.0117752	0.038447	0.023408
BP	GO:0003272	endocardial cushion formation	16/7778	24/18670	0.0117752	0.038447	0.023408
BP	GO:0007530	sex determination	16/7778	24/18670	0.0117752	0.038447	0.023408
BP	GO:0032528	microvillus organization	16/7778	24/18670	0.0117752	0.038447	0.023408
BP	GO:0036010	protein localization to endosome	16/7778	24/18670	0.0117752	0.038447	0.023408
BP	GO:0036315	cellular response to sterol	16/7778	24/18670	0.0117752	0.038447	0.023408
BP	GO:0044346	fibroblast apoptotic process	16/7778	24/18670	0.0117752	0.038447	0.023408
BP	GO:0046426	negative regulation of receptor signaling pathway via JAK-STAT	16/7778	24/18670	0.0117752	0.038447	0.023408
BP	GO:0046639	negative regulation of alpha-beta T cell differentiation	16/7778	24/18670	0.0117752	0.038447	0.023408
BP	GO:0050687	negative regulation of defense response to virus	16/7778	24/18670	0.0117752	0.038447	0.023408
BP	GO:0051220	cytoplasmic sequestering of protein	16/7778	24/18670	0.0117752	0.038447	0.023408
BP	GO:1903020	positive regulation of glycoprotein metabolic process	16/7778	24/18670	0.0117752	0.038447	0.023408
BP	GO:1903649	regulation of cytoplasmic transport	16/7778	24/18670	0.0117752	0.038447	0.023408
BP	GO:1904376	negative regulation of protein localization to cell periphery	16/7778	24/18670	0.0117752	0.038447	0.023408
BP	GO:0002832	negative regulation of response to biotic stimulus	51/7778	95/18670	0.0118038	0.0385207	0.023453
BP	GO:0098693	regulation of synaptic vesicle cycle	59/7778	112/18670	0.0118655	0.0387026	0.023563
BP	GO:0035023	regulation of Rho protein signal transduction	72/7778	140/18670	0.0121148	0.0394958	0.024046
BP	GO:0098840	protein transport along microtubule	38/7778	68/18670	0.0124262	0.04047	0.02464
BP	GO:0099118	microtubule-based protein transport	38/7778	68/18670	0.0124262	0.04047	0.02464
BP	GO:0001556	oocyte maturation	17/7778	26/18670	0.0125146	0.0406552	0.024752
BP	GO:0003171	atrioventricular valve development	17/7778	26/18670	0.0125146	0.0406552	0.024752
BP	GO:0048169	regulation of long-term neuronal synaptic plasticity	17/7778	26/18670	0.0125146	0.0406552	0.024752
BP	GO:0060740	prostate gland epithelium morphogenesis	17/7778	26/18670	0.0125146	0.0406552	0.024752
BP	GO:0070911	global genome nucleotide-excision repair	17/7778	26/18670	0.0125146	0.0406552	0.024752
BP	GO:0040014	regulation of multicellular organism growth	37/7778	66/18670	0.0126806	0.0410962	0.025021
BP	GO:0046626	regulation of insulin receptor signaling pathway	37/7778	66/18670	0.0126806	0.0410962	0.025021
BP	GO:0099072	regulation of postsynaptic membrane neurotransmitter receptor level	37/7778	66/18670	0.0126806	0.0410962	0.025021
BP	GO:1904888	cranial skeletal system development	37/7778	66/18670	0.0126806	0.0410962	0.025021
BP	GO:0046165	alcohol biosynthetic process	88/7778	175/18670	0.0126823	0.0410962	0.025021
BP	GO:0016051	carbohydrate biosynthetic process	103/7778	208/18670	0.0128903	0.0417492	0.025418
BP	GO:0035773	insulin secretion involved in cellular response to glucose stimulus	36/7778	64/18670	0.0129299	0.0418565	0.025484
BP	GO:0006760	folic acid-containing compound metabolic process	18/7778	28/18670	0.0131199	0.0422591	0.025729
BP	GO:0031440	regulation of mRNA 3'-end processing	18/7778	28/18670	0.0131199	0.0422591	0.025729
BP	GO:0035456	response to interferon-beta	18/7778	28/18670	0.0131199	0.0422591	0.025729
BP	GO:0051123	RNA polymerase II preinitiation complex assembly	18/7778	28/18670	0.0131199	0.0422591	0.025729
BP	GO:0060512	prostate gland morphogenesis	18/7778	28/18670	0.0131199	0.0422591	0.025729
BP	GO:0061615	glycolytic process through fructose-6-phosphate	18/7778	28/18670	0.0131199	0.0422591	0.025729
BP	GO:0061620	glycolytic process through glucose-6-phosphate	18/7778	28/18670	0.0131199	0.0422591	0.025729
BP	GO:0070076	histone lysine demethylation	18/7778	28/18670	0.0131199	0.0422591	0.025729
BP	GO:0090344	negative regulation of cell aging	18/7778	28/18670	0.0131199	0.0422591	0.025729
BP	GO:2000108	positive regulation of leukocyte apoptotic process	18/7778	28/18670	0.0131199	0.0422591	0.025729
BP	GO:0032387	negative regulation of intracellular transport	35/7778	62/18670	0.0131727	0.0423188	0.025765
BP	GO:0001711	endodermal cell fate commitment	11/7778	15/18670	0.0132632	0.0423188	0.025765
BP	GO:0015936	coenzyme A metabolic process	11/7778	15/18670	0.0132632	0.0423188	0.025765
BP	GO:0032239	regulation of nucleobase-containing compound transport	11/7778	15/18670	0.0132632	0.0423188	0.025765
BP	GO:0032486	Rap protein signal transduction	11/7778	15/18670	0.0132632	0.0423188	0.025765
BP	GO:0034134	toll-like receptor 2 signaling pathway	11/7778	15/18670	0.0132632	0.0423188	0.025765
BP	GO:0034616	response to laminar fluid shear stress	11/7778	15/18670	0.0132632	0.0423188	0.025765
BP	GO:0042762	regulation of sulfur metabolic process	11/7778	15/18670	0.0132632	0.0423188	0.025765
BP	GO:0043584	nose development	11/7778	15/18670	0.0132632	0.0423188	0.025765
BP	GO:0045898	regulation of RNA polymerase II transcription preinitiation complex a:	11/7778	15/18670	0.0132632	0.0423188	0.025765
BP	GO:0048569	post-embryonic animal organ development	11/7778	15/18670	0.0132632	0.0423188	0.025765
BP	GO:0060841	venous blood vessel development	11/7778	15/18670	0.0132632	0.0423188	0.025765
BP	GO:0070365	hepatocyte differentiation	11/7778	15/18670	0.0132632	0.0423188	0.025765
BP	GO:0072075	metanephric mesenchyme development	11/7778	15/18670	0.0132632	0.0423188	0.025765
BP	GO:1902187	negative regulation of viral release from host cell	11/7778	15/18670	0.0132632	0.0423188	0.025765
BP	GO:1902188	positive regulation of viral release from host cell	11/7778	15/18670	0.0132632	0.0423188	0.025765
BP	GO:1903729	regulation of plasma membrane organization	11/7778	15/18670	0.0132632	0.0423188	0.025765
BP	GO:2001028	positive regulation of endothelial cell chemotaxis	11/7778	15/18670	0.0132632	0.0423188	0.025765
BP	GO:2001185	regulation of CD8-positive, alpha-beta T cell activation	11/7778	15/18670	0.0132632	0.0423188	0.025765
BP	GO:0050920	regulation of chemotaxis	107/7778	217/18670	0.0132745	0.0423339	0.025774
BP	GO:0032273	positive regulation of protein polymerization	69/7778	134/18670	0.0133594	0.0425416	0.025901

BP	GO:0044409	entry into host	69/7778	134/18670	0.0133594	0.0425416	0.025901
BP	GO:0048565	digestive tract development	69/7778	134/18670	0.0133594	0.0425416	0.025901
BP	GO:0043524	negative regulation of neuron apoptotic process	75/7778	147/18670	0.0134134	0.0426923	0.025993
BP	GO:0003209	cardiac atrium morphogenesis	19/7778	30/18670	0.0136055	0.0431119	0.026248
BP	GO:0006517	protein deglycosylation	19/7778	30/18670	0.0136055	0.0431119	0.026248
BP	GO:0021696	cerebellar cortex morphogenesis	19/7778	30/18670	0.0136055	0.0431119	0.026248
BP	GO:0044818	mitotic G2/M transition checkpoint	19/7778	30/18670	0.0136055	0.0431119	0.026248
BP	GO:0045880	positive regulation of smoothened signaling pathway	19/7778	30/18670	0.0136055	0.0431119	0.026248
BP	GO:0048147	negative regulation of fibroblast proliferation	19/7778	30/18670	0.0136055	0.0431119	0.026248
BP	GO:0090200	positive regulation of release of cytochrome c from mitochondria	19/7778	30/18670	0.0136055	0.0431119	0.026248
BP	GO:0097421	liver regeneration	19/7778	30/18670	0.0136055	0.0431119	0.026248
BP	GO:1904292	regulation of ERAD pathway	19/7778	30/18670	0.0136055	0.0431119	0.026248
BP	GO:0031663	lipopolysaccharide-mediated signaling pathway	33/7778	58/18670	0.0136314	0.0431515	0.026272
BP	GO:0071385	cellular response to glucocorticoid stimulus	33/7778	58/18670	0.0136314	0.0431515	0.026272
BP	GO:0030148	sphingolipid biosynthetic process	54/7778	102/18670	0.0138239	0.0437355	0.026628
BP	GO:0051568	histone H3-K4 methylation	32/7778	56/18670	0.013843	0.0437355	0.026628
BP	GO:0060688	regulation of morphogenesis of a branching structure	32/7778	56/18670	0.013843	0.0437355	0.026628
BP	GO:1903202	negative regulation of oxidative stress-induced cell death	32/7778	56/18670	0.013843	0.0437355	0.026628
BP	GO:1903955	positive regulation of protein targeting to mitochondrion	20/7778	32/18670	0.0139853	0.0441419	0.026875
BP	GO:2000780	negative regulation of double-strand break repair	20/7778	32/18670	0.0139853	0.0441419	0.026875
BP	GO:0048660	regulation of smooth muscle cell proliferation	85/7778	169/18670	0.0140141	0.0442109	0.026917
BP	GO:0051353	positive regulation of oxidoreductase activity	31/7778	54/18670	0.0140395	0.0442694	0.026953
BP	GO:0016447	somatic recombination of immunoglobulin gene segments	30/7778	52/18670	0.0142178	0.0448059	0.027279
BP	GO:0030856	regulation of epithelial cell differentiation	79/7778	156/18670	0.0142345	0.0448059	0.027279
BP	GO:0098657	import into cell	95/7778	191/18670	0.0142352	0.0448059	0.027279
BP	GO:0007435	salivary gland morphogenesis	21/7778	34/18670	0.0142722	0.0448059	0.027279
BP	GO:0010092	specification of animal organ identity	21/7778	34/18670	0.0142722	0.0448059	0.027279
BP	GO:0042558	pteridine-containing compound metabolic process	21/7778	34/18670	0.0142722	0.0448059	0.027279
BP	GO:0060969	negative regulation of gene silencing	21/7778	34/18670	0.0142722	0.0448059	0.027279
BP	GO:0097009	energy homeostasis	21/7778	34/18670	0.0142722	0.0448059	0.027279
BP	GO:1904030	negative regulation of cyclin-dependent protein kinase activity	21/7778	34/18670	0.0142722	0.0448059	0.027279
BP	GO:0008652	cellular amino acid biosynthetic process	44/7778	81/18670	0.0143219	0.0449399	0.027361
BP	GO:0009112	nucleobase metabolic process	22/7778	36/18670	0.0144777	0.0453625	0.027618
BP	GO:0030224	monocyte differentiation	22/7778	36/18670	0.0144777	0.0453625	0.027618
BP	GO:1903131	mononuclear cell differentiation	22/7778	36/18670	0.0144777	0.0453625	0.027618
BP	GO:0035272	exocrine system development	28/7778	48/18670	0.0145065	0.0454305	0.02766
BP	GO:0048599	oocyte development	27/7778	46/18670	0.0146086	0.0456505	0.027794
BP	GO:0051646	mitochondrion localization	27/7778	46/18670	0.0146086	0.0456505	0.027794
BP	GO:0097720	calcineurin-mediated signaling	27/7778	46/18670	0.0146086	0.0456505	0.027794
BP	GO:1904036	negative regulation of epithelial cell apoptotic process	27/7778	46/18670	0.0146086	0.0456505	0.027794
BP	GO:0001825	blastocyst formation	23/7778	38/18670	0.0146121	0.0456505	0.027794
BP	GO:0042987	amyloid precursor protein catabolic process	26/7778	44/18670	0.0146762	0.0457742	0.027869
BP	GO:0000183	rDNA heterochromatin assembly	24/7778	40/18670	0.0146847	0.0457742	0.027869
BP	GO:0002369	T cell cytokine production	24/7778	40/18670	0.0146847	0.0457742	0.027869
BP	GO:0072583	clathrin-dependent endocytosis	24/7778	40/18670	0.0146847	0.0457742	0.027869
BP	GO:0043473	pigmentation	52/7778	98/18670	0.0146872	0.0457742	0.027869
BP	GO:0006284	base-excision repair	25/7778	42/18670	0.0147037	0.0457811	0.027873
BP	GO:0017145	stem cell division	25/7778	42/18670	0.0147037	0.0457811	0.027873
BP	GO:0002223	stimulatory C-type lectin receptor signaling pathway	59/7778	113/18670	0.0148942	0.0463295	0.028207
BP	GO:0048640	negative regulation of developmental growth	59/7778	113/18670	0.0148942	0.0463295	0.028207
BP	GO:0009205	purine ribonucleoside triphosphate metabolic process	42/7778	77/18670	0.0150998	0.0469464	0.028583
BP	GO:0003184	pulmonary valve morphogenesis	12/7778	17/18670	0.0152717	0.0472302	0.028755
BP	GO:0003198	epithelial to mesenchymal transition involved in endocardial cushion	12/7778	17/18670	0.0152717	0.0472302	0.028755
BP	GO:0016246	RNA interference	12/7778	17/18670	0.0152717	0.0472302	0.028755
BP	GO:0035855	megakaryocyte development	12/7778	17/18670	0.0152717	0.0472302	0.028755
BP	GO:0043923	positive regulation by host of viral transcription	12/7778	17/18670	0.0152717	0.0472302	0.028755
BP	GO:0090185	negative regulation of kidney development	12/7778	17/18670	0.0152717	0.0472302	0.028755
BP	GO:0099010	modification of postsynaptic structure	12/7778	17/18670	0.0152717	0.0472302	0.028755
BP	GO:1904355	positive regulation of telomere capping	12/7778	17/18670	0.0152717	0.0472302	0.028755
BP	GO:1904948	midbrain dopaminergic neuron differentiation	12/7778	17/18670	0.0152717	0.0472302	0.028755
BP	GO:2000136	regulation of cell proliferation involved in heart morphogenesis	12/7778	17/18670	0.0152717	0.0472302	0.028755
BP	GO:2000811	negative regulation of anoikis	12/7778	17/18670	0.0152717	0.0472302	0.028755
BP	GO:0022618	ribonucleoprotein complex assembly	112/7778	229/18670	0.0153645	0.0474943	0.028916
BP	GO:0031623	receptor internalization	58/7778	111/18670	0.0153758	0.0475064	0.028923
BP	GO:0001707	mesoderm formation	41/7778	75/18670	0.0154966	0.0478566	0.029137
BP	GO:0044728	DNA methylation or demethylation	50/7778	94/18670	0.0155986	0.0481488	0.029315
BP	GO:0002312	B cell activation involved in immune response	40/7778	73/18670	0.0158977	0.049025	0.029848
BP	GO:0060350	endochondral bone morphogenesis	40/7778	73/18670	0.0158977	0.049025	0.029848
BP	GO:0000338	protein deneddylation	8/7778	10/18670	0.0162368	0.0494551	0.03011
BP	GO:0007084	mitotic nuclear envelope reassembly	8/7778	10/18670	0.0162368	0.0494551	0.03011
BP	GO:0010603	regulation of cytoplasmic mRNA processing body assembly	8/7778	10/18670	0.0162368	0.0494551	0.03011
BP	GO:0010944	negative regulation of transcription by competitive promoter binding	8/7778	10/18670	0.0162368	0.0494551	0.03011
BP	GO:0032070	regulation of deoxyribonuclease activity	8/7778	10/18670	0.0162368	0.0494551	0.03011
BP	GO:0035871	protein K11-linked deubiquitination	8/7778	10/18670	0.0162368	0.0494551	0.03011
BP	GO:0035999	tetrahydrofolate interconversion	8/7778	10/18670	0.0162368	0.0494551	0.03011
BP	GO:0036500	ATF6-mediated unfolded protein response	8/7778	10/18670	0.0162368	0.0494551	0.03011
BP	GO:0043471	regulation of cellular carbohydrate catabolic process	8/7778	10/18670	0.0162368	0.0494551	0.03011
BP	GO:0045876	positive regulation of sister chromatid cohesion	8/7778	10/18670	0.0162368	0.0494551	0.03011
BP	GO:0051409	response to nitrosative stress	8/7778	10/18670	0.0162368	0.0494551	0.03011
BP	GO:0061299	retina vasculature morphogenesis in camera-type eye	8/7778	10/18670	0.0162368	0.0494551	0.03011
BP	GO:0072124	regulation of glomerular mesangial cell proliferation	8/7778	10/18670	0.0162368	0.0494551	0.03011
BP	GO:0072203	cell proliferation involved in metanephros development	8/7778	10/18670	0.0162368	0.0494551	0.03011
BP	GO:0072641	type I interferon secretion	8/7778	10/18670	0.0162368	0.0494551	0.03011
BP	GO:0090309	positive regulation of DNA methylation-dependent heterochromatin a	8/7778	10/18670	0.0162368	0.0494551	0.03011
BP	GO:0090557	establishment of endothelial intestinal barrier	8/7778	10/18670	0.0162368	0.0494551	0.03011
BP	GO:0098734	macromolecule depalmitoylation	8/7778	10/18670	0.0162368	0.0494551	0.03011
BP	GO:0150011	regulation of neuron projection arborization	8/7778	10/18670	0.0162368	0.0494551	0.03011
BP	GO:1900122	positive regulation of receptor binding	8/7778	10/18670	0.0162368	0.0494551	0.03011
BP	GO:1901725	regulation of histone deacetylase activity	8/7778	10/18670	0.0162368	0.0494551	0.03011
BP	GO:1901838	positive regulation of transcription of nucleolar large rRNA by RNA p	8/7778	10/18670	0.0162368	0.0494551	0.03011
BP	GO:1903862	positive regulation of oxidative phosphorylation	8/7778	10/18670	0.0162368	0.0494551	0.03011
BP	GO:2000271	positive regulation of fibroblast apoptotic process	8/7778	10/18670	0.0162368	0.0494551	0.03011

BP	GO:2000628	regulation of miRNA metabolic process	8/7778	10/18670	0.0162368	0.0494551	0.03011
BP	GO:2000644	regulation of receptor catabolic process	8/7778	10/18670	0.0162368	0.0494551	0.03011
BP	GO:0051592	response to calcium ion	75/7778	148/18670	0.0162666	0.0495224	0.030151
BP	GO:0042273	ribosomal large subunit biogenesis	39/7778	71/18670	0.0163025	0.0496082	0.030203
CC	GO:0030055	cell-substrate junction	279/8049	412/19717	7.99E-29	6.09E-26	3.52E-26
CC	GO:0005925	focal adhesion	274/8049	405/19717	3.17E-28	1.21E-25	6.98E-26
CC	GO:0098687	chromosomal region	231/8049	347/19717	1.51E-22	3.84E-20	2.22E-20
CC	GO:0000151	ubiquitin ligase complex	192/8049	282/19717	1.21E-20	2.02E-18	1.17E-18
CC	GO:0016607	nuclear speck	253/8049	397/19717	1.32E-20	2.02E-18	1.17E-18
CC	GO:0035770	ribonucleoprotein granule	156/8049	223/19717	8.98E-19	1.14E-16	6.58E-17
CC	GO:0005667	transcription regulator complex	257/8049	415/19717	1.72E-18	1.87E-16	1.08E-16
CC	GO:0000790	nuclear chromatin	236/8049	376/19717	4.14E-18	3.94E-16	2.28E-16
CC	GO:0005819	spindle	219/8049	347/19717	2.67E-17	2.26E-15	1.31E-15
CC	GO:0036464	cytoplasmic ribonucleoprotein granule	147/8049	212/19717	3.17E-17	2.42E-15	1.39E-15
CC	GO:0061695	transferase complex, transferring phosphorus-containing groups	169/8049	259/19717	1.30E-15	8.97E-14	5.18E-14
CC	GO:0005635	nuclear envelope	273/8049	464/19717	2.00E-15	1.27E-13	7.34E-14
CC	GO:0000775	chromosome, centromeric region	132/8049	193/19717	7.16E-15	4.20E-13	2.42E-13
CC	GO:0016605	PML body	78/8049	99/19717	1.25E-14	6.79E-13	3.92E-13
CC	GO:0010008	endosome membrane	277/8049	479/19717	2.41E-14	1.22E-12	7.06E-13
CC	GO:0005769	early endosome	212/8049	350/19717	4.75E-14	2.26E-12	1.31E-12
CC	GO:0005874	microtubule	243/8049	416/19717	2.12E-13	9.50E-12	5.48E-12
CC	GO:0031252	cell leading edge	236/8049	403/19717	3.25E-13	1.37E-11	7.94E-12
CC	GO:0031965	nuclear membrane	182/8049	296/19717	4.33E-13	1.74E-11	1.00E-11
CC	GO:0031461	cullin-RING ubiquitin ligase complex	108/8049	158/19717	2.11E-12	8.03E-11	4.64E-11
CC	GO:0005774	vacuolar membrane	236/8049	412/19717	7.64E-12	2.77E-10	1.60E-10
CC	GO:0010494	cytoplasmic stress granule	54/8049	66/19717	9.62E-12	3.20E-10	1.85E-10
CC	GO:0031984	organelle subcompartment	220/8049	380/19717	9.67E-12	3.20E-10	1.85E-10
CC	GO:0005741	mitochondrial outer membrane	117/8049	178/19717	1.46E-11	4.46E-10	2.58E-10
CC	GO:0005798	Golgi-associated vesicle	117/8049	178/19717	1.46E-11	4.46E-10	2.58E-10
CC	GO:0030496	midbody	114/8049	173/19717	2.09E-11	6.12E-10	3.54E-10
CC	GO:0018995	host cellular component	57/8049	73/19717	9.00E-11	2.45E-09	1.41E-09
CC	GO:0043657	host cell	57/8049	73/19717	9.00E-11	2.45E-09	1.41E-09
CC	GO:0031968	organelle outer membrane	127/8049	201/19717	1.12E-10	2.94E-09	1.70E-09
CC	GO:0019867	outer membrane	128/8049	203/19717	1.16E-10	2.94E-09	1.70E-09
CC	GO:0017053	transcription repressor complex	59/8049	77/19717	1.66E-10	4.08E-09	2.35E-09
CC	GO:0005765	lysosomal membrane	203/8049	354/19717	1.93E-10	4.60E-09	2.65E-09
CC	GO:0030027	lamellipodium	122/8049	193/19717	2.48E-10	5.73E-09	3.31E-09
CC	GO:0098852	lytic vacuole membrane	203/8049	355/19717	2.70E-10	6.05E-09	3.49E-09
CC	GO:0000792	heterochromatin	58/8049	76/19717	3.14E-10	6.83E-09	3.94E-09
CC	GO:0000776	kinetochore	91/8049	135/19717	3.57E-10	7.56E-09	4.36E-09
CC	GO:0000781	chromosome, telomeric region	103/8049	158/19717	4.84E-10	9.98E-09	5.76E-09
CC	GO:0098791	Golgi apparatus subcompartment	204/8049	359/19717	5.12E-10	1.03E-08	5.93E-09
CC	GO:0001650	fibrillar center	89/8049	132/19717	5.45E-10	1.07E-08	6.15E-09
CC	GO:0000784	nuclear chromosome, telomeric region	84/8049	123/19717	6.22E-10	1.19E-08	6.85E-09
CC	GO:0030427	site of polarized growth	112/8049	176/19717	7.47E-10	1.39E-08	8.02E-09
CC	GO:0072686	mitotic spindle	76/8049	109/19717	9.10E-10	1.65E-08	9.53E-09
CC	GO:0030426	growth cone	109/8049	171/19717	1.09E-09	1.93E-08	1.11E-08
CC	GO:0031248	protein acetyltransferase complex	68/8049	95/19717	1.17E-09	1.98E-08	1.14E-08
CC	GO:1902493	acetyltransferase complex	68/8049	95/19717	1.17E-09	1.98E-08	1.14E-08
CC	GO:0005876	spindle microtubule	47/8049	59/19717	1.21E-09	2.01E-08	1.16E-08
CC	GO:0090575	RNA polymerase II transcription regulator complex	106/8049	166/19717	1.59E-09	2.57E-08	1.49E-08
CC	GO:0030135	coated vesicle	166/8049	289/19717	7.22E-09	1.15E-07	6.62E-08
CC	GO:0031301	integral component of organelle membrane	195/8049	350/19717	1.04E-08	1.61E-07	9.32E-08
CC	GO:0120111	neuron projection cytoplasm	60/8049	84/19717	1.25E-08	1.91E-07	1.10E-07
CC	GO:0031300	intrinsic component of organelle membrane	209/8049	381/19717	1.71E-08	2.56E-07	1.48E-07
CC	GO:0000793	condensed chromosome	132/8049	223/19717	2.07E-08	3.04E-07	1.76E-07
CC	GO:0031519	PcG protein complex	38/8049	47/19717	2.27E-08	3.26E-07	1.88E-07
CC	GO:0000779	condensed chromosome, centromeric region	78/8049	118/19717	2.42E-08	3.41E-07	1.97E-07
CC	GO:0000123	histone acetyltransferase complex	60/8049	85/19717	2.56E-08	3.55E-07	2.05E-07
CC	GO:1902911	protein kinase complex	73/8049	109/19717	2.94E-08	4.00E-07	2.31E-07
CC	GO:0005938	cell cortex	173/8049	308/19717	3.20E-08	4.28E-07	2.47E-07
CC	GO:0005802	trans-Golgi network	137/8049	236/19717	6.02E-08	7.90E-07	4.56E-07
CC	GO:0030117	membrane coat	65/8049	96/19717	8.83E-08	1.12E-06	6.47E-07
CC	GO:0048475	coated membrane	65/8049	96/19717	8.83E-08	1.12E-06	6.47E-07
CC	GO:0005881	cytoplasmic microtubule	50/8049	69/19717	9.67E-08	1.21E-06	6.97E-07
CC	GO:1904115	axon cytoplasm	43/8049	57/19717	1.13E-07	1.39E-06	8.00E-07
CC	GO:1904949	ATPase complex	56/8049	80/19717	1.18E-07	1.43E-06	8.26E-07
CC	GO:0030176	integral component of endoplasmic reticulum membrane	93/8049	150/19717	1.25E-07	1.48E-06	8.57E-07
CC	GO:0001726	ruffle	104/8049	172/19717	1.45E-07	1.70E-06	9.79E-07
CC	GO:0030660	Golgi-associated vesicle membrane	74/8049	114/19717	1.69E-07	1.92E-06	1.11E-06
CC	GO:0035097	histone methyltransferase complex	59/8049	86/19717	1.69E-07	1.92E-06	1.11E-06
CC	GO:0030133	transport vesicle	210/8049	392/19717	1.83E-07	2.05E-06	1.18E-06
CC	GO:1902554	serine/threonine protein kinase complex	60/8049	88/19717	1.87E-07	2.07E-06	1.20E-06
CC	GO:0005657	replication fork	50/8049	70/19717	2.04E-07	2.19E-06	1.27E-06
CC	GO:0005770	late endosome	145/8049	256/19717	2.04E-07	2.19E-06	1.27E-06
CC	GO:0005793	endoplasmic reticulum-Golgi intermediate compartment	80/8049	126/19717	2.21E-07	2.34E-06	1.35E-06
CC	GO:0034708	methyltransferase complex	73/8049	113/19717	2.71E-07	2.83E-06	1.63E-06
CC	GO:0031227	intrinsic component of endoplasmic reticulum membrane	96/8049	158/19717	3.12E-07	3.21E-06	1.85E-06
CC	GO:0042470	melanosome	69/8049	106/19717	3.72E-07	3.73E-06	2.15E-06
CC	GO:0048770	pigment granule	69/8049	106/19717	3.72E-07	3.73E-06	2.15E-06
CC	GO:0034399	nuclear periphery	82/8049	131/19717	3.80E-07	3.76E-06	2.17E-06
CC	GO:0008287	protein serine/threonine phosphatase complex	35/8049	45/19717	4.83E-07	4.61E-06	2.66E-06
CC	GO:1903293	phosphatase complex	35/8049	45/19717	4.83E-07	4.61E-06	2.66E-06
CC	GO:0000932	P-body	57/8049	84/19717	4.84E-07	4.61E-06	2.66E-06
CC	GO:0000407	phagophore assembly site	27/8049	32/19717	5.08E-07	4.78E-06	2.76E-06
CC	GO:0000777	condensed chromosome kinetochore	68/8049	105/19717	6.00E-07	5.57E-06	3.22E-06
CC	GO:0030662	coated vesicle membrane	107/8049	182/19717	6.94E-07	6.34E-06	3.66E-06
CC	GO:0150034	distal axon	157/8049	285/19717	6.99E-07	6.34E-06	3.66E-06
CC	GO:0070603	SWI/SNF superfamily-type complex	52/8049	76/19717	1.03E-06	9.23E-06	5.33E-06
CC	GO:0030120	vesicle coat	42/8049	58/19717	1.06E-06	9.38E-06	5.42E-06
CC	GO:0101002	ficolin-1-rich granule	77/8049	124/19717	1.34E-06	1.16E-05	6.72E-06
CC	GO:1904813	ficolin-1-rich granule lumen	77/8049	124/19717	1.34E-06	1.16E-05	6.72E-06

CC	GO:0016234	inclusion body	55/8049	82/19717	1.36E-06	1.16E-05	6.72E-06
CC	GO:0000922	spindle pole	97/8049	164/19717	1.55E-06	1.31E-05	7.59E-06
CC	GO:0005720	nuclear heterochromatin	27/8049	33/19717	1.70E-06	1.42E-05	8.22E-06
CC	GO:0030175	filopodium	66/8049	104/19717	2.53E-06	2.10E-05	1.21E-05
CC	GO:0005643	nuclear pore	58/8049	89/19717	2.94E-06	2.38E-05	1.38E-05
CC	GO:1905368	peptidase complex	58/8049	89/19717	2.94E-06	2.38E-05	1.38E-05
CC	GO:0055037	recycling endosome	102/8049	176/19717	3.10E-06	2.49E-05	1.44E-05
CC	GO:0031463	Cul3-RING ubiquitin ligase complex	29/8049	37/19717	3.60E-06	2.86E-05	1.65E-05
CC	GO:0005681	spliceosomal complex	106/8049	185/19717	4.14E-06	3.25E-05	1.88E-05
CC	GO:0090734	site of DNA damage	49/8049	73/19717	4.91E-06	3.82E-05	2.21E-05
CC	GO:1905369	endopeptidase complex	44/8049	64/19717	5.68E-06	4.37E-05	2.53E-05
CC	GO:0030014	CCR4-NOT complex	16/8049	17/19717	6.18E-06	4.71E-05	2.72E-05
CC	GO:0000118	histone deacetylase complex	47/8049	70/19717	7.55E-06	5.70E-05	3.29E-05
CC	GO:0000502	proteasome complex	43/8049	63/19717	9.68E-06	7.23E-05	4.18E-05
CC	GO:0031901	early endosome membrane	87/8049	149/19717	1.07E-05	7.91E-05	4.57E-05
CC	GO:0000803	sex chromosome	24/8049	30/19717	1.37E-05	0.0001006	5.81E-05
CC	GO:0043025	neuronal cell body	249/8049	497/19717	1.44E-05	0.0001044	6.03E-05
CC	GO:0005743	mitochondrial inner membrane	238/8049	473/19717	1.51E-05	0.0001087	6.28E-05
CC	GO:0098793	presynapse	246/8049	491/19717	1.61E-05	0.0001148	6.63E-05
CC	GO:0030137	COPI-coated vesicle	22/8049	27/19717	1.86E-05	0.0001314	7.59E-05
CC	GO:0016363	nuclear matrix	66/8049	109/19717	2.44E-05	0.0001709	9.87E-05
CC	GO:0051233	spindle midzone	26/8049	34/19717	2.56E-05	0.0001774	0.000102
CC	GO:1990752	microtubule end	23/8049	29/19717	2.71E-05	0.0001864	0.000108
CC	GO:0030127	COPII vesicle coat	14/8049	15/19717	3.29E-05	0.0002241	0.000129
CC	GO:0032587	ruffle membrane	58/8049	94/19717	3.43E-05	0.0002312	0.000134
CC	GO:0099568	cytoplasmic region	134/8049	251/19717	3.54E-05	0.0002367	0.000137
CC	GO:0030880	RNA polymerase complex	67/8049	112/19717	3.69E-05	0.0002446	0.000141
CC	GO:0035861	site of double-strand break	39/8049	58/19717	4.24E-05	0.0002786	0.000161
CC	GO:0080008	Cul4-RING E3 ubiquitin ligase complex	25/8049	33/19717	4.85E-05	0.0003158	0.000182
CC	GO:0098589	membrane region	169/8049	328/19717	5.09E-05	0.0003286	0.00019
CC	GO:0019005	SCF ubiquitin ligase complex	42/8049	64/19717	5.25E-05	0.0003309	0.000191
CC	GO:0005776	autophagosome	57/8049	93/19717	5.25E-05	0.0003309	0.000191
CC	GO:0030134	COPII-coated ER to Golgi transport vesicle	57/8049	93/19717	5.25E-05	0.0003309	0.000191
CC	GO:0005911	cell-cell junction	209/8049	416/19717	5.47E-05	0.0003404	0.000197
CC	GO:0055029	nuclear DNA-directed RNA polymerase complex	64/8049	107/19717	5.49E-05	0.0003404	0.000197
CC	GO:0000307	cyclin-dependent protein kinase holoenzyme complex	30/8049	42/19717	5.73E-05	0.0003523	0.000203
CC	GO:0032993	protein-DNA complex	110/8049	202/19717	5.84E-05	0.0003561	0.000206
CC	GO:0030663	COPI-coated vesicle membrane	17/8049	20/19717	6.40E-05	0.0003868	0.000223
CC	GO:0000940	condensed chromosome outer kinetochore	13/8049	14/19717	7.56E-05	0.0004537	0.000262
CC	GO:0045121	membrane raft	162/8049	315/19717	8.14E-05	0.0004808	0.000278
CC	GO:0000428	DNA-directed RNA polymerase complex	64/8049	108/19717	8.14E-05	0.0004808	0.000278
CC	GO:0098562	cytoplasmic side of membrane	98/8049	178/19717	8.26E-05	0.0004842	0.00028
CC	GO:0005795	Golgi stack	84/8049	149/19717	8.67E-05	0.0005041	0.000291
CC	GO:0098857	membrane microdomain	162/8049	316/19717	0.0001002	0.0005785	0.000334
CC	GO:0098978	glutamatergic synapse	177/8049	349/19717	0.0001041	0.0005961	0.000344
CC	GO:0055038	recycling endosome membrane	50/8049	81/19717	0.0001148	0.0006525	0.000377
CC	GO:1902562	H4 histone acetyltransferase complex	30/8049	43/19717	0.0001156	0.0006525	0.000377
CC	GO:0071782	endoplasmic reticulum tubular network	16/8049	19/19717	0.0001342	0.0007473	0.000431
CC	GO:0014069	postsynaptic density	165/8049	324/19717	0.0001343	0.0007473	0.000431
CC	GO:0030658	transport vesicle membrane	111/8049	208/19717	0.0001619	0.000888	0.000513
CC	GO:0034045	phagophore assembly site membrane	14/8049	16/19717	0.0001635	0.000888	0.000513
CC	GO:0035098	ESC/E(Z) complex	14/8049	16/19717	0.0001635	0.000888	0.000513
CC	GO:0005759	mitochondrial matrix	230/8049	469/19717	0.0001643	0.000888	0.000513
CC	GO:0098984	neuron to neuron synapse	176/8049	350/19717	0.0001915	0.0010208	0.000589
CC	GO:0032279	asymmetric synapse	166/8049	328/19717	0.0001916	0.0010208	0.000589
CC	GO:0016328	lateral plasma membrane	37/8049	57/19717	0.000203	0.0010745	0.00062
CC	GO:0000242	pericentriolar material	17/8049	21/19717	0.0002073	0.0010893	0.000629
CC	GO:0005875	microtubule associated complex	84/8049	152/19717	0.000217	0.0011324	0.000654
CC	GO:0030667	secretory granule membrane	152/8049	298/19717	0.0002192	0.0011364	0.000656
CC	GO:0070461	SAGA-type complex	21/8049	28/19717	0.0002544	0.00131	0.000756
CC	GO:0071013	catalytic step 2 spliceosome	52/8049	87/19717	0.000272	0.0013912	0.000803
CC	GO:0045335	phagocytic vesicle	74/8049	132/19717	0.00028	0.0014223	0.000821
CC	GO:0098827	endoplasmic reticulum subcompartment	18/8049	23/19717	0.0002906	0.0014663	0.000847
CC	GO:0031256	leading edge membrane	92/8049	170/19717	0.0003006	0.0015061	0.00087
CC	GO:0031253	cell projection membrane	164/8049	326/19717	0.0003024	0.0015061	0.00087
CC	GO:0043596	nuclear replication fork	28/8049	41/19717	0.0003437	0.0017006	0.000982
CC	GO:0030126	COPI vesicle coat	13/8049	15/19717	0.000353	0.0017245	0.000996
CC	GO:0030173	integral component of Golgi membrane	38/8049	60/19717	0.000353	0.0017245	0.000996
CC	GO:0012507	ER to Golgi transport vesicle membrane	39/8049	62/19717	0.0003637	0.001765	0.001019
CC	GO:0000152	nuclear ubiquitin ligase complex	29/8049	43/19717	0.0003757	0.0018118	0.001046
CC	GO:0017101	aminoacyl-tRNA synthetase multienzyme complex	11/8049	12/19717	0.0003926	0.0018816	0.001087
CC	GO:0030670	phagocytic vesicle membrane	46/8049	76/19717	0.0004044	0.001926	0.001112
CC	GO:0035371	microtubule plus-end	16/8049	20/19717	0.0004154	0.0019659	0.001135
CC	GO:0099572	postsynaptic specialization	173/8049	348/19717	0.0004429	0.0020831	0.001203
CC	GO:0032838	plasma membrane bounded cell projection cytoplasm	109/8049	208/19717	0.0004563	0.0021332	0.001232
CC	GO:0032154	cleavage furrow	35/8049	55/19717	0.0005247	0.0024379	0.001408
CC	GO:0005814	centriole	76/8049	139/19717	0.0006467	0.0029865	0.001725
CC	GO:0044309	neuron spine	86/8049	161/19717	0.000802	0.0036622	0.002115
CC	GO:0031983	vesicle lumen	162/8049	327/19717	0.0008026	0.0036622	0.002115
CC	GO:0060205	cytoplasmic vesicle lumen	161/8049	325/19717	0.0008339	0.0037825	0.002184
CC	GO:0030139	endocytic vesicle	151/8049	303/19717	0.0008611	0.0038827	0.002242
CC	GO:0035577	azurophil granule membrane	36/8049	58/19717	0.0008702	0.003884	0.002243
CC	GO:0000780	condensed nuclear chromosome, centromeric region	19/8049	26/19717	0.0008716	0.003884	0.002243
CC	GO:0034774	secretory granule lumen	159/8049	321/19717	0.0009003	0.0039885	0.002303
CC	GO:0016592	mediator complex	25/8049	37/19717	0.0009095	0.0040026	0.002311
CC	GO:0032153	cell division site	41/8049	68/19717	0.000914	0.0040026	0.002311
CC	GO:0044295	axonal growth cone	20/8049	28/19717	0.0010214	0.0044475	0.002568
CC	GO:0042581	specific granule	85/8049	160/19717	0.0010677	0.0046227	0.002669
CC	GO:0044232	organelle membrane contact site	16/8049	21/19717	0.0010813	0.004655	0.002688
CC	GO:0031091	platelet alpha granule	52/8049	91/19717	0.0011924	0.0051045	0.002947
CC	GO:0005791	rough endoplasmic reticulum	49/8049	85/19717	0.0012566	0.0053494	0.003089
CC	GO:0016591	RNA polymerase II, holoenzyme	48/8049	83/19717	0.0012762	0.0054027	0.00312

CC	GO:0005811	lipid droplet	47/8049	81/19717	0.0012947	0.0054323	0.003137
CC	GO:0032839	dendrite cytoplasm	22/8049	32/19717	0.0012975	0.0054323	0.003137
CC	GO:0005758	mitochondrial intermembrane space	46/8049	79/19717	0.0013118	0.0054622	0.003154
CC	GO:0005871	kinesin complex	34/8049	55/19717	0.0013295	0.0055058	0.003179
CC	GO:0033116	endoplasmic reticulum-Golgi intermediate compartment membrane	42/8049	71/19717	0.001363	0.0056098	0.003239
CC	GO:0005905	clathrin-coated pit	41/8049	69/19717	0.0013703	0.0056098	0.003239
CC	GO:0031228	intrinsic component of Golgi membrane	39/8049	65/19717	0.0013767	0.0056098	0.003239
CC	GO:0043197	dendritic spine	84/8049	159/19717	0.0014142	0.0057321	0.00331
CC	GO:0005736	RNA polymerase I complex	11/8049	13/19717	0.0016018	0.0064239	0.003709
CC	GO:0042405	nuclear inclusion body	11/8049	13/19717	0.0016018	0.0064239	0.003709
CC	GO:0005788	endoplasmic reticulum lumen	152/8049	309/19717	0.0016524	0.0065923	0.003807
CC	GO:0031902	late endosome membrane	72/8049	134/19717	0.0016634	0.0066017	0.003812
CC	GO:0031970	organelle envelope lumen	50/8049	88/19717	0.0017374	0.0068596	0.003961
CC	GO:0005942	phosphatidylinositol 3-kinase complex	19/8049	27/19717	0.0018169	0.0071366	0.004121
CC	GO:0098573	intrinsic component of mitochondrial membrane	43/8049	74/19717	0.001965	0.0076609	0.004424
CC	GO:0000164	protein phosphatase type 1 complex	9/8049	10/19717	0.0019873	0.0076609	0.004424
CC	GO:0036513	Derlin-1 retrotranslocation complex	9/8049	10/19717	0.0019873	0.0076609	0.004424
CC	GO:0005844	polysome	42/8049	72/19717	0.0019906	0.0076609	0.004424
CC	GO:0009898	cytoplasmic side of plasma membrane	81/8049	154/19717	0.0020039	0.0076731	0.004431
CC	GO:0000794	condensed nuclear chromosome	55/8049	99/19717	0.0021165	0.0080639	0.004656
CC	GO:0030140	trans-Golgi network transport vesicle	21/8049	31/19717	0.0022245	0.0084169	0.00486
CC	GO:0031985	Golgi cisterna	63/8049	116/19717	0.0022313	0.0084169	0.00486
CC	GO:0000778	condensed nuclear chromosome kinetochore	12/8049	15/19717	0.0023664	0.0087112	0.00503
CC	GO:0005671	Ada2/Gcn5/Ada3 transcription activator complex	12/8049	15/19717	0.0023664	0.0087112	0.00503
CC	GO:0016581	NuRD complex	12/8049	15/19717	0.0023664	0.0087112	0.00503
CC	GO:0090545	CHD-type complex	12/8049	15/19717	0.0023664	0.0087112	0.00503
CC	GO:0098554	cytoplasmic side of endoplasmic reticulum membrane	12/8049	15/19717	0.0023664	0.0087112	0.00503
CC	GO:0101031	chaperone complex	16/8049	22/19717	0.0024614	0.0090173	0.005207
CC	GO:0032592	integral component of mitochondrial membrane	42/8049	73/19717	0.0028542	0.0104063	0.006009
CC	GO:0098858	actin-based cell projection	105/8049	208/19717	0.0029135	0.0105718	0.006104
CC	GO:0009295	nucleoid	27/8049	43/19717	0.0029633	0.0106012	0.006121
CC	GO:0016235	aggresome	27/8049	43/19717	0.0029633	0.0106012	0.006121
CC	GO:0042645	mitochondrial nucleoid	27/8049	43/19717	0.0029633	0.0106012	0.006121
CC	GO:0019898	extrinsic component of membrane	144/8049	295/19717	0.0031109	0.0110564	0.006384
CC	GO:0005666	RNA polymerase III complex	13/8049	17/19717	0.0031196	0.0110564	0.006384
CC	GO:0030867	rough endoplasmic reticulum membrane	18/8049	26/19717	0.0031894	0.0111997	0.006467
CC	GO:0030894	replisome	18/8049	26/19717	0.0031894	0.0111997	0.006467
CC	GO:0005684	U2-type spliceosomal complex	50/8049	90/19717	0.0032914	0.0113672	0.006564
CC	GO:0005845	mRNA cap binding complex	10/8049	12/19717	0.0033565	0.0113672	0.006564
CC	GO:0030877	beta-catenin destruction complex	10/8049	12/19717	0.0033565	0.0113672	0.006564
CC	GO:0031464	Cul4A-RING E3 ubiquitin ligase complex	10/8049	12/19717	0.0033565	0.0113672	0.006564
CC	GO:0042555	MCM complex	10/8049	12/19717	0.0033565	0.0113672	0.006564
CC	GO:0044615	nuclear pore nuclear basket	10/8049	12/19717	0.0033565	0.0113672	0.006564
CC	GO:0097504	Gemini of coiled bodies	10/8049	12/19717	0.0033565	0.0113672	0.006564
CC	GO:1990909	Wnt signalosome	10/8049	12/19717	0.0033565	0.0113672	0.006564
CC	GO:0016514	SWI/SNF complex	14/8049	19/19717	0.0038253	0.012841	0.007415
CC	GO:0043034	costamere	14/8049	19/19717	0.0038253	0.012841	0.007415
CC	GO:0030136	clathrin-coated vesicle	95/8049	188/19717	0.0043011	0.0143749	0.0083
CC	GO:0008180	COP9 signalosome	23/8049	36/19717	0.0043491	0.0144717	0.008356
CC	GO:0035579	specific granule membrane	50/8049	91/19717	0.0044405	0.0147116	0.008495
CC	GO:0042575	DNA polymerase complex	15/8049	21/19717	0.0044648	0.0147279	0.008504
CC	GO:0010369	chromocenter	11/8049	14/19717	0.004704	0.015318	0.008845
CC	GO:0033276	transcription factor TFTC complex	11/8049	14/19717	0.004704	0.015318	0.008845
CC	GO:0034518	RNA cap binding complex	11/8049	14/19717	0.004704	0.015318	0.008845
CC	GO:0030118	clathrin coat	28/8049	46/19717	0.004743	0.0153795	0.008881
CC	GO:0008021	synaptic vesicle	96/8049	191/19717	0.00502	0.0162086	0.009359
CC	GO:0030666	endocytic vesicle membrane	85/8049	167/19717	0.0052034	0.01673	0.00966
CC	GO:0042641	actomyosin	44/8049	79/19717	0.005281	0.0169081	0.009763
CC	GO:0030057	desmosome	17/8049	25/19717	0.0055189	0.0175957	0.01016
CC	GO:0032432	actin filament bundle	42/8049	75/19717	0.0055716	0.0176897	0.010215
CC	GO:0044291	cell-cell contact zone	40/8049	71/19717	0.0058621	0.0184829	0.010673
CC	GO:0097431	mitotic spindle pole	18/8049	27/19717	0.0059344	0.0184829	0.010673
CC	GO:0000153	cytoplasmic ubiquitin ligase complex	12/8049	16/19717	0.0059427	0.0184829	0.010673
CC	GO:0005852	eukaryotic translation initiation factor 3 complex	12/8049	16/19717	0.0059427	0.0184829	0.010673
CC	GO:0070822	Sin3-type complex	12/8049	16/19717	0.0059427	0.0184829	0.010673
CC	GO:0001725	stress fiber	38/8049	67/19717	0.0061477	0.0189658	0.010951
CC	GO:0097517	contractile actin filament bundle	38/8049	67/19717	0.0061477	0.0189658	0.010951
CC	GO:0005766	primary lysosome	79/8049	155/19717	0.0065811	0.0201398	0.011629
CC	GO:0042582	azurophil granule	79/8049	155/19717	0.0065811	0.0201398	0.011629
CC	GO:0016323	basolateral plasma membrane	107/8049	217/19717	0.0067243	0.0204806	0.011826
CC	GO:0030863	cortical cytoskeleton	61/8049	116/19717	0.0067462	0.0204806	0.011826
CC	GO:0098685	Schaffer collateral - CA1 synapse	45/8049	82/19717	0.0069361	0.0205776	0.011882
CC	GO:0000235	astral microtubule	9/8049	11/19717	0.0069402	0.0205776	0.011882
CC	GO:0005652	nuclear lamina	9/8049	11/19717	0.0069402	0.0205776	0.011882
CC	GO:0005818	aster	9/8049	11/19717	0.0069402	0.0205776	0.011882
CC	GO:0034663	endoplasmic reticulum chaperone complex	9/8049	11/19717	0.0069402	0.0205776	0.011882
CC	GO:1990907	beta-catenin-TCF complex	9/8049	11/19717	0.0069402	0.0205776	0.011882
CC	GO:0031201	SNARE complex	28/8049	47/19717	0.0072075	0.0212872	0.012292
CC	GO:0030119	AP-type membrane coat adaptor complex	27/8049	45/19717	0.0072391	0.0212981	0.012298
CC	GO:0005604	basement membrane	51/8049	95/19717	0.0075191	0.0220369	0.012725
CC	GO:0030684	preribosome	42/8049	76/19717	0.0075972	0.0221805	0.012808
CC	GO:0005721	pericentric heterochromatin	14/8049	20/19717	0.0079724	0.023187	0.013389
CC	GO:0001741	XY body	10/8049	13/19717	0.0092054	0.0262716	0.01517
CC	GO:0008250	oligosaccharyltransferase complex	10/8049	13/19717	0.0092054	0.0262716	0.01517
CC	GO:0016580	Sin3 complex	10/8049	13/19717	0.0092054	0.0262716	0.01517
CC	GO:0044233	mitochondria-associated endoplasmic reticulum membrane	10/8049	13/19717	0.0092054	0.0262716	0.01517
CC	GO:1990023	mitotic spindle midzone	10/8049	13/19717	0.0092054	0.0262716	0.01517
CC	GO:0043601	nuclear replisome	16/8049	24/19717	0.0094019	0.0267324	0.015436
CC	GO:0090543	Flemming body	18/8049	28/19717	0.0103229	0.0292419	0.016885
CC	GO:0032580	Golgi cisterna membrane	49/8049	92/19717	0.010502	0.0296389	0.017114
CC	GO:0002102	podosome	19/8049	30/19717	0.0106278	0.0298832	0.017255
CC	GO:0042788	polysomal ribosome	20/8049	32/19717	0.0108462	0.0303853	0.017545

CC	GO:0099738	cell cortex region	25/8049	42/19717	0.0110079	0.0305134	0.017619
CC	GO:0000421	autophagosome membrane	22/8049	36/19717	0.0110693	0.0305134	0.017619
CC	GO:0032040	small-subunit processome	23/8049	38/19717	0.0110936	0.0305134	0.017619
CC	GO:0031462	Cul2-RING ubiquitin ligase complex	11/8049	15/19717	0.0111322	0.0305134	0.017619
CC	GO:0033290	eukaryotic 48S preinitiation complex	11/8049	15/19717	0.0111322	0.0305134	0.017619
CC	GO:0035102	PRC1 complex	11/8049	15/19717	0.0111322	0.0305134	0.017619
CC	GO:0070382	exocytic vesicle	101/8049	207/19717	0.0118978	0.0324952	0.018764
CC	GO:0098798	mitochondrial protein complex	124/8049	259/19717	0.0122598	0.0333641	0.019265
CC	GO:0031092	platelet alpha granule membrane	12/8049	17/19717	0.0127181	0.0344882	0.019914
CC	GO:0005912	adherens junction	41/8049	76/19717	0.0139876	0.0371174	0.021433
CC	GO:0031594	neuromuscular junction	41/8049	76/19717	0.0139876	0.0371174	0.021433
CC	GO:0000159	protein phosphatase type 2A complex	13/8049	19/19717	0.0139908	0.0371174	0.021433
CC	GO:0000805	X chromosome	8/8049	10/19717	0.014126	0.0371174	0.021433
CC	GO:0031010	ISWI-type complex	8/8049	10/19717	0.014126	0.0371174	0.021433
CC	GO:0031080	nuclear pore outer ring	8/8049	10/19717	0.014126	0.0371174	0.021433
CC	GO:0031414	N-terminal protein acetyltransferase complex	8/8049	10/19717	0.014126	0.0371174	0.021433
CC	GO:0070938	contractile ring	8/8049	10/19717	0.014126	0.0371174	0.021433
CC	GO:0072546	ER membrane protein complex	8/8049	10/19717	0.014126	0.0371174	0.021433
CC	GO:0070469	respirasome	52/8049	100/19717	0.0153129	0.0400978	0.023154
CC	GO:0046930	pore complex	15/8049	23/19717	0.0157474	0.0410942	0.023729
CC	GO:0031304	intrinsic component of mitochondrial inner membrane	26/8049	45/19717	0.0159364	0.0413045	0.02385
CC	GO:0031305	integral component of mitochondrial inner membrane	26/8049	45/19717	0.0159364	0.0413045	0.02385
CC	GO:0005801	cis-Golgi network	37/8049	68/19717	0.0160635	0.0414929	0.023959
CC	GO:0044665	MLL1/2 complex	18/8049	29/19717	0.0169373	0.0434552	0.025092
CC	GO:0071339	MLL1 complex	18/8049	29/19717	0.0169373	0.0434552	0.025092
CC	GO:0030914	STAGA complex	9/8049	12/19717	0.0176915	0.0450867	0.026034
CC	GO:0071564	npBAF complex	9/8049	12/19717	0.0176915	0.0450867	0.026034
CC	GO:0022626	cytosolic ribosome	57/8049	112/19717	0.0195022	0.0495356	0.028603
MF	GO:0019787	ubiquitin-like protein transferase activity	277/7791	407/17696	4.07E-23	4.80E-20	3.74E-20
MF	GO:0045296	cadherin binding	231/7791	331/17696	1.13E-21	5.15E-19	4.00E-19
MF	GO:0050839	cell adhesion molecule binding	324/7791	499/17696	1.31E-21	5.15E-19	4.00E-19
MF	GO:0044389	ubiquitin-like protein ligase binding	217/7791	308/17696	2.98E-21	8.81E-19	6.85E-19
MF	GO:0004842	ubiquitin-protein transferase activity	258/7791	382/17696	6.70E-21	1.58E-18	1.23E-18
MF	GO:0031625	ubiquitin protein ligase binding	205/7791	290/17696	2.01E-20	3.96E-18	3.08E-18
MF	GO:0140297	DNA-binding transcription factor binding	225/7791	351/17696	1.73E-14	2.92E-12	2.27E-12
MF	GO:0004674	protein serine/threonine kinase activity	265/7791	439/17696	2.48E-12	3.66E-10	2.85E-10
MF	GO:0003713	transcription coactivator activity	200/7791	319/17696	1.04E-11	1.37E-09	1.06E-09
MF	GO:0061629	RNA polymerase II-specific DNA-binding transcription factor binding	177/7791	277/17696	1.60E-11	1.89E-09	1.47E-09
MF	GO:0003725	double-stranded RNA binding	61/7791	75/17696	3.40E-11	3.65E-09	2.84E-09
MF	GO:0061659	ubiquitin-like protein ligase activity	149/7791	230/17696	1.47E-10	1.44E-08	1.12E-08
MF	GO:0032182	ubiquitin-like protein binding	73/7791	96/17696	1.59E-10	1.44E-08	1.12E-08
MF	GO:0003714	transcription corepressor activity	153/7791	238/17696	1.95E-10	1.65E-08	1.28E-08
MF	GO:0004386	helicase activity	109/7791	163/17696	2.97E-09	2.34E-07	1.82E-07
MF	GO:0061630	ubiquitin protein ligase activity	140/7791	221/17696	4.80E-09	3.54E-07	2.76E-07
MF	GO:0042393	histone binding	127/7791	197/17696	5.14E-09	3.57E-07	2.78E-07
MF	GO:0016887	ATPase activity	250/7791	434/17696	6.19E-09	4.06E-07	3.16E-07
MF	GO:0019903	protein phosphatase binding	95/7791	140/17696	9.75E-09	6.06E-07	4.72E-07
MF	GO:0017016	Ras GTPase binding	246/7791	429/17696	1.43E-08	8.42E-07	6.55E-07
MF	GO:0046332	SMAD binding	60/7791	80/17696	1.69E-08	9.49E-07	7.38E-07
MF	GO:0031267	small GTPase binding	252/7791	443/17696	2.54E-08	1.37E-06	1.06E-06
MF	GO:0002039	p53 binding	51/7791	66/17696	3.66E-08	1.88E-06	1.46E-06
MF	GO:0019902	phosphatase binding	117/7791	185/17696	9.81E-08	4.83E-06	3.75E-06
MF	GO:0032549	ribonucleoside binding	218/7791	381/17696	1.18E-07	5.59E-06	4.35E-06
MF	GO:0043130	ubiquitin binding	56/7791	76/17696	1.43E-07	6.51E-06	5.07E-06
MF	GO:0003727	single-stranded RNA binding	67/7791	95/17696	1.49E-07	6.54E-06	5.09E-06
MF	GO:0019207	kinase regulator activity	128/7791	207/17696	1.67E-07	7.03E-06	5.47E-06
MF	GO:0005525	GTP binding	213/7791	374/17696	2.66E-07	1.05E-05	8.21E-06
MF	GO:0032550	purine ribonucleoside binding	215/7791	378/17696	2.68E-07	1.05E-05	8.21E-06
MF	GO:0001882	nucleoside binding	219/7791	388/17696	4.60E-07	1.75E-05	1.36E-05
MF	GO:0001883	purine nucleoside binding	215/7791	381/17696	5.98E-07	2.21E-05	1.72E-05
MF	GO:0003730	mRNA 3'-UTR binding	63/7791	91/17696	9.97E-07	3.57E-05	2.78E-05
MF	GO:0008022	protein C-terminus binding	115/7791	187/17696	1.03E-06	3.59E-05	2.79E-05
MF	GO:0042826	histone deacetylase binding	74/7791	111/17696	1.18E-06	3.99E-05	3.11E-05
MF	GO:0043021	ribonucleoprotein complex binding	86/7791	133/17696	1.22E-06	3.99E-05	3.11E-05
MF	GO:0015631	tubulin binding	191/7791	336/17696	1.29E-06	4.11E-05	3.20E-05
MF	GO:0019887	protein kinase regulator activity	110/7791	180/17696	2.70E-06	8.38E-05	6.52E-05
MF	GO:0001217	DNA-binding transcription repressor activity	142/7791	242/17696	2.84E-06	8.38E-05	6.52E-05
MF	GO:0001227	DNA-binding transcription repressor activity, RNA polymerase II-spec	142/7791	242/17696	2.84E-06	8.38E-05	6.52E-05
MF	GO:0048156	tau protein binding	35/7791	45/17696	4.03E-06	0.0001162	9.04E-05
MF	GO:0003678	DNA helicase activity	56/7791	81/17696	4.22E-06	0.0001187	9.23E-05
MF	GO:0140030	modification-dependent protein binding	90/7791	144/17696	5.80E-06	0.0001592	0.000124
MF	GO:0070491	repressing transcription factor binding	50/7791	71/17696	6.00E-06	0.0001611	0.000125
MF	GO:0035257	nuclear hormone receptor binding	94/7791	152/17696	6.92E-06	0.0001817	0.000141
MF	GO:0008094	DNA-dependent ATPase activity	73/7791	113/17696	7.99E-06	0.0002035	0.000158
MF	GO:0051087	chaperone binding	67/7791	102/17696	8.10E-06	0.0002035	0.000158
MF	GO:0019001	guanyl nucleotide binding	216/7791	394/17696	8.72E-06	0.0002102	0.000164
MF	GO:0032561	guanyl ribonucleotide binding	216/7791	394/17696	8.72E-06	0.0002102	0.000164
MF	GO:0001228	DNA-binding transcription activator activity, RNA polymerase II-speci	238/7791	439/17696	9.06E-06	0.000214	0.000167
MF	GO:0016791	phosphatase activity	154/7791	270/17696	1.03E-05	0.0002357	0.000183
MF	GO:0003924	GTPase activity	181/7791	324/17696	1.04E-05	0.0002357	0.000183
MF	GO:0001216	DNA-binding transcription activator activity	238/7791	440/17696	1.12E-05	0.0002495	0.000194
MF	GO:0019003	GDP binding	51/7791	74/17696	1.29E-05	0.0002828	0.00022
MF	GO:0019210	kinase inhibitor activity	47/7791	67/17696	1.34E-05	0.0002886	0.000225
MF	GO:0031491	nucleosome binding	57/7791	85/17696	1.49E-05	0.0003148	0.000245
MF	GO:0003697	single-stranded DNA binding	72/7791	113/17696	1.87E-05	0.0003868	0.000301
MF	GO:0004721	phosphoprotein phosphatase activity	108/7791	182/17696	2.15E-05	0.000437	0.00034
MF	GO:0019894	kinesin binding	32/7791	42/17696	2.24E-05	0.0004492	0.00035
MF	GO:0034212	peptide N-acetyltransferase activity	51/7791	75/17696	2.32E-05	0.0004496	0.00035
MF	GO:0034450	ubiquitin-ubiquitin ligase activity	13/7791	13/17696	2.32E-05	0.0004496	0.00035
MF	GO:0016538	cyclin-dependent protein serine/threonine kinase regulator activity	36/7791	49/17696	2.76E-05	0.0005263	0.00041
MF	GO:0017048	Rho GTPase binding	105/7791	177/17696	2.83E-05	0.0005297	0.000412
MF	GO:0004860	protein kinase inhibitor activity	44/7791	63/17696	3.01E-05	0.0005546	0.000432

MF	GO:0032183	SUMO binding	15/7791	16/17696	4.22E-05	0.0007675	0.000597
MF	GO:0097718	disordered domain specific binding	26/7791	33/17696	4.91E-05	0.0008793	0.000684
MF	GO:0008017	microtubule binding	139/7791	246/17696	5.05E-05	0.0008796	0.000684
MF	GO:0061980	regulatory RNA binding	32/7791	43/17696	5.06E-05	0.0008796	0.000684
MF	GO:0061631	ubiquitin conjugating enzyme activity	29/7791	38/17696	5.20E-05	0.0008906	0.000693
MF	GO:0004402	histone acetyltransferase activity	44/7791	64/17696	5.53E-05	0.0009324	0.000725
MF	GO:0061733	peptide-lysine-N-acetyltransferase activity	45/7791	66/17696	6.27E-05	0.0010387	0.000808
MF	GO:0000287	magnesium ion binding	122/7791	213/17696	6.33E-05	0.0010387	0.000808
MF	GO:0061650	ubiquitin-like protein conjugating enzyme activity	30/7791	40/17696	6.82E-05	0.0011026	0.000858
MF	GO:0035258	steroid hormone receptor binding	59/7791	92/17696	7.86E-05	0.0012545	0.000976
MF	GO:0051219	phosphoprotein binding	54/7791	83/17696	8.83E-05	0.0013904	0.001082
MF	GO:0003688	DNA replication origin binding	20/7791	24/17696	9.03E-05	0.0013988	0.001088
MF	GO:0043539	protein serine/threonine kinase activator activity	28/7791	37/17696	9.12E-05	0.0013988	0.001088
MF	GO:0003684	damaged DNA binding	44/7791	65/17696	9.83E-05	0.0014888	0.001158
MF	GO:0031072	heat shock protein binding	73/7791	119/17696	0.0001027	0.001536	0.001195
MF	GO:0140097	catalytic activity, acting on DNA	121/7791	213/17696	0.0001101	0.0016254	0.001265
MF	GO:0070411	I-SMAD binding	11/7791	11/17696	0.00012	0.0017496	0.001361
MF	GO:0016922	nuclear receptor binding	68/7791	110/17696	0.0001254	0.0018062	0.001405
MF	GO:0042578	phosphoric ester hydrolase activity	196/7791	367/17696	0.0001655	0.0023553	0.001833
MF	GO:0004712	protein serine/threonine/tyrosine kinase activity	31/7791	43/17696	0.0001795	0.0025238	0.001964
MF	GO:0048487	beta-tubulin binding	28/7791	38/17696	0.0002009	0.0027909	0.002172
MF	GO:0005123	death receptor binding	15/7791	17/17696	0.0002114	0.0029034	0.002259
MF	GO:0060090	molecular adaptor activity	140/7791	254/17696	0.0002259	0.0030663	0.002386
MF	GO:0051082	unfolded protein binding	78/7791	131/17696	0.0002433	0.0032656	0.002541
MF	GO:0004879	nuclear receptor activity	33/7791	47/17696	0.0002524	0.0033118	0.002577
MF	GO:0098531	ligand-activated transcription factor activity	33/7791	47/17696	0.0002524	0.0033118	0.002577
MF	GO:0004722	protein serine/threonine phosphatase activity	49/7791	76/17696	0.0002562	0.0033253	0.002587
MF	GO:0001103	RNA polymerase II repressing transcription factor binding	26/7791	35/17696	0.0002736	0.0035123	0.002733
MF	GO:0008013	beta-catenin binding	52/7791	82/17696	0.0003093	0.0039275	0.003056
MF	GO:0003779	actin binding	225/7791	431/17696	0.0003368	0.0042318	0.003293
MF	GO:0030145	manganese ion binding	41/7791	62/17696	0.0003647	0.0045216	0.003518
MF	GO:0047485	protein N-terminus binding	66/7791	109/17696	0.0003675	0.0045216	0.003518
MF	GO:0008080	N-acetyltransferase activity	56/7791	90/17696	0.0003781	0.0046033	0.003582
MF	GO:0004535	poly(A)-specific ribonuclease activity	12/7791	13/17696	0.0004076	0.0049122	0.003822
MF	GO:0043022	ribosome binding	38/7791	57/17696	0.0004653	0.0055502	0.004319
MF	GO:0033613	activating transcription factor binding	53/7791	85/17696	0.0004986	0.0058483	0.004551
MF	GO:0035035	histone acetyltransferase binding	22/7791	29/17696	0.0005002	0.0058483	0.004551
MF	GO:0001085	RNA polymerase II transcription factor binding	40/7791	61/17696	0.0005563	0.0064415	0.005012
MF	GO:0019789	SUMO transferase activity	17/7791	21/17696	0.0006136	0.0070357	0.005475
MF	GO:0003724	RNA helicase activity	49/7791	78/17696	0.0006265	0.0071146	0.005536
MF	GO:0035198	miRNA binding	23/7791	31/17696	0.0006407	0.0071386	0.005555
MF	GO:0140142	nucleocytoplasmic carrier activity	23/7791	31/17696	0.0006407	0.0071386	0.005555
MF	GO:0001046	core promoter sequence-specific DNA binding	31/7791	45/17696	0.000655	0.0072297	0.005626
MF	GO:0030374	nuclear receptor transcription coactivator activity	43/7791	67/17696	0.000693	0.007578	0.005896
MF	GO:0035091	phosphatidylinositol binding	133/7791	245/17696	0.0007382	0.0079982	0.006223
MF	GO:0070064	proline-rich region binding	15/7791	18/17696	0.0007484	0.0080355	0.006253
MF	GO:0008187	poly-pyrimidine tract binding	24/7791	33/17696	0.0007927	0.0084341	0.006563
MF	GO:0140098	catalytic activity, acting on RNA	201/7791	386/17696	0.0008	0.0084359	0.006564
MF	GO:0005161	platelet-derived growth factor receptor binding	13/7791	15/17696	0.0008553	0.0089393	0.006956
MF	GO:0043425	bHLH transcription factor binding	21/7791	28/17696	0.0008737	0.0090171	0.007016
MF	GO:0019208	phosphatase regulator activity	58/7791	96/17696	0.000878	0.0090171	0.007016
MF	GO:0051427	hormone receptor binding	103/7791	185/17696	0.0009019	0.0091826	0.007145
MF	GO:0031593	polyubiquitin modification-dependent protein binding	34/7791	51/17696	0.0009163	0.0092495	0.007197
MF	GO:0016407	acetyltransferase activity	65/7791	110/17696	0.0010221	0.0102299	0.00796
MF	GO:0050681	androgen receptor binding	30/7791	44/17696	0.0010416	0.0103369	0.008043
MF	GO:0005085	guanyl-nucleotide exchange factor activity	117/7791	214/17696	0.0010551	0.0103753	0.008073
MF	GO:0008135	translation factor activity, RNA binding	52/7791	85/17696	0.001063	0.0103753	0.008073
MF	GO:0070717	poly-purine tract binding	22/7791	30/17696	0.0010956	0.0106061	0.008253
MF	GO:0042162	telomeric DNA binding	26/7791	37/17696	0.0011209	0.010762	0.008374
MF	GO:0017124	SH3 domain binding	75/7791	130/17696	0.0011489	0.010942	0.008514
MF	GO:0048027	mRNA 5'-UTR binding	19/7791	25/17696	0.0011839	0.0111855	0.008704
MF	GO:0097110	scaffold protein binding	38/7791	59/17696	0.001265	0.0118571	0.009226
MF	GO:0031489	myosin V binding	14/7791	17/17696	0.0014322	0.0131117	0.010202
MF	GO:0051010	microtubule plus-end binding	14/7791	17/17696	0.0014322	0.0131117	0.010202
MF	GO:1990381	ubiquitin-specific protease binding	14/7791	17/17696	0.0014322	0.0131117	0.010202
MF	GO:0031490	chromatin DNA binding	69/7791	119/17696	0.0014782	0.0134292	0.010449
MF	GO:0045182	translation regulator activity	80/7791	141/17696	0.0015635	0.0140671	0.010946
MF	GO:0000979	RNA polymerase II core promoter sequence-specific DNA binding	24/7791	34/17696	0.0015723	0.0140671	0.010946
MF	GO:0017166	vinculin binding	10/7791	11/17696	0.0018003	0.0159864	0.012439
MF	GO:0033558	protein deacetylase activity	21/7791	29/17696	0.0018541	0.0163413	0.012715
MF	GO:0005178	integrin binding	75/7791	132/17696	0.0020426	0.0178691	0.013904
MF	GO:0070577	lysine-acetylated histone binding	15/7791	19/17696	0.002102	0.0181205	0.0141
MF	GO:0140033	acetylation-dependent protein binding	15/7791	19/17696	0.002102	0.0181205	0.0141
MF	GO:0060589	nucleoside-triphosphatase regulator activity	178/7791	344/17696	0.0022083	0.0188985	0.014705
MF	GO:0060590	ATPase regulator activity	27/7791	40/17696	0.0023156	0.0195658	0.015224
MF	GO:0005160	transforming growth factor beta receptor binding	33/7791	51/17696	0.0023194	0.0195658	0.015224
MF	GO:0001102	RNA polymerase II activating transcription factor binding	34/7791	53/17696	0.0024793	0.0207663	0.016158
MF	GO:0030544	Hsp70 protein binding	28/7791	42/17696	0.0025601	0.0208951	0.016259
MF	GO:0004709	MAP kinase kinase kinase activity	19/7791	26/17696	0.0025831	0.0208951	0.016259
MF	GO:0017075	syntaxin-1 binding	19/7791	26/17696	0.0025831	0.0208951	0.016259
MF	GO:0051019	mitogen-activated protein kinase binding	19/7791	26/17696	0.0025831	0.0208951	0.016259
MF	GO:0051787	misfolded protein binding	19/7791	26/17696	0.0025831	0.0208951	0.016259
MF	GO:0005521	lamin binding	13/7791	16/17696	0.0027121	0.0214963	0.016726
MF	GO:0015095	magnesium ion transmembrane transporter activity	13/7791	16/17696	0.0027121	0.0214963	0.016726
MF	GO:0035259	glucocorticoid receptor binding	13/7791	16/17696	0.0027121	0.0214963	0.016726
MF	GO:0050750	low-density lipoprotein particle receptor binding	16/7791	21/17696	0.0028316	0.0222939	0.017347
MF	GO:0004407	histone deacetylase activity	20/7791	28/17696	0.0031029	0.024268	0.018883
MF	GO:0017017	MAP kinase tyrosine/serine/threonine phosphatase activity	11/7791	13/17696	0.0033436	0.0259095	0.02016
MF	GO:0004725	protein tyrosine phosphatase activity	58/7791	100/17696	0.0033566	0.0259095	0.02016
MF	GO:0016747	transferase activity, transferring acyl groups other than amino-acyl gr	123/7791	232/17696	0.0034677	0.0265933	0.020693
MF	GO:0048365	Rac GTPase binding	42/7791	69/17696	0.0035454	0.0270134	0.021019
MF	GO:0008266	poly(U) RNA binding	21/7791	30/17696	0.003624	0.0274353	0.021348

MF	GO:0004576	oligosaccharyl transferase activity	9/7791	10/17696	0.0037444	0.0279885	0.021778
MF	GO:0030957	Tat protein binding	9/7791	10/17696	0.0037444	0.0279885	0.021778
MF	GO:0019905	syntaxin binding	45/7791	75/17696	0.0038397	0.0285203	0.022192
MF	GO:0019199	transmembrane receptor protein kinase activity	47/7791	79/17696	0.0040051	0.0295628	0.023003
MF	GO:0043621	protein self-association	35/7791	56/17696	0.0040737	0.0297365	0.023138
MF	GO:0004527	exonuclease activity	48/7791	81/17696	0.004079	0.0297365	0.023138
MF	GO:0016417	S-acyltransferase activity	22/7791	32/17696	0.0041389	0.0298048	0.023191
MF	GO:0051721	protein phosphatase 2A binding	22/7791	32/17696	0.0041389	0.0298048	0.023191
MF	GO:0016667	oxidoreductase activity, acting on a sulfur group of donors	36/7791	58/17696	0.0042534	0.0304209	0.023671
MF	GO:0001221	transcription cofactor binding	28/7791	43/17696	0.0042759	0.0304209	0.023671
MF	GO:0008139	nuclear localization sequence binding	18/7791	25/17696	0.0043649	0.030868	0.024019
MF	GO:0016779	nucleotidyltransferase activity	76/7791	137/17696	0.0044918	0.031576	0.02457
MF	GO:0043138	3'-5' DNA helicase activity	15/7791	20/17696	0.004983	0.0342525	0.026652
MF	GO:0030674	protein-macromolecule adaptor activity	110/7791	207/17696	0.0049873	0.0342525	0.026652
MF	GO:0001056	RNA polymerase III activity	12/7791	15/17696	0.0050755	0.0342525	0.026652
MF	GO:0003841	1-acylglycerol-3-phosphate O-acyltransferase activity	12/7791	15/17696	0.0050755	0.0342525	0.026652
MF	GO:0005024	transforming growth factor beta-activated receptor activity	12/7791	15/17696	0.0050755	0.0342525	0.026652
MF	GO:0033549	MAP kinase phosphatase activity	12/7791	15/17696	0.0050755	0.0342525	0.026652
MF	GO:0042171	lysophosphatidic acid acyltransferase activity	12/7791	15/17696	0.0050755	0.0342525	0.026652
MF	GO:0003743	translation initiation factor activity	32/7791	51/17696	0.0054247	0.0364007	0.028324
MF	GO:0051117	ATPase binding	47/7791	80/17696	0.0056068	0.0374102	0.029109
MF	GO:0046966	thyroid hormone receptor binding	20/7791	29/17696	0.0058778	0.0389627	0.030317
MF	GO:0002020	protease binding	71/7791	128/17696	0.0059054	0.0389627	0.030317
MF	GO:0000149	SNARE binding	62/7791	110/17696	0.0060679	0.0398121	0.030978
MF	GO:0005158	insulin receptor binding	16/7791	22/17696	0.0061444	0.039871	0.031024
MF	GO:0050321	tau-protein kinase activity	16/7791	22/17696	0.0061444	0.039871	0.031024
MF	GO:0000340	RNA 7-methylguanosine cap binding	10/7791	12/17696	0.0065054	0.0415293	0.032314
MF	GO:0001054	RNA polymerase I activity	10/7791	12/17696	0.0065054	0.0415293	0.032314
MF	GO:0031994	insulin-like growth factor I binding	10/7791	12/17696	0.0065054	0.0415293	0.032314
MF	GO:0042813	Wnt-activated receptor activity	13/7791	17/17696	0.0068716	0.0433979	0.033768
MF	GO:0070628	proteasome binding	13/7791	17/17696	0.0068716	0.0433979	0.033768
MF	GO:0005048	signal sequence binding	29/7791	46/17696	0.0072328	0.0451952	0.035167
MF	GO:0032813	tumor necrosis factor receptor superfamily binding	29/7791	46/17696	0.0072328	0.0451952	0.035167
MF	GO:0008143	poly(A) binding	17/7791	24/17696	0.007281	0.0452572	0.035215
MF	GO:1990841	promoter-specific chromatin binding	30/7791	48/17696	0.0075818	0.0468804	0.036478
MF	GO:0051018	protein kinase A binding	31/7791	50/17696	0.0079069	0.0486355	0.037844

BP, biological process; CC, cellular component; MF, molecular function.

Table S4. KEGG terms enriched among the miRNA targeted genes.

ID	Description	Gene_Ratio	*P*-value	*P*-adjust	qvalue	Gene_ID	Count
hsa04218	Cellular senescence	121/3698	2.82E-16	9.12E-14	5.14E-14	1869/1026/10114/	121
hsa05205	Proteoglycans in cancer	149/3698	2.49E-15	4.02E-13	2.26E-13	27250/1026/7422/	149
hsa04115	p53 signaling pathway	64/3698	6.89E-14	6.66E-12	3.75E-12	1026/5268/355/27	64
hsa04120	Ubiquitin mediated proteolysis	107/3698	9.10E-14	6.66E-12	3.75E-12	83737/331/672/99	107
hsa04110	Cell cycle	97/3698	1.03E-13	6.66E-12	3.75E-12	1869/1026/1021/1	97
hsa05211	Renal cell carcinoma	60/3698	9.53E-13	4.74E-11	2.67E-11	1026/7422/7040/2	60
hsa05223	Non-small cell lung cancer	62/3698	1.03E-12	4.74E-11	2.67E-11	1869/1026/1021/5	62
hsa04140	Autophagy - animal	102/3698	5.93E-12	2.40E-10	1.35E-10	596/5728/207/208	102
hsa05210	Colorectal cancer	70/3698	9.46E-12	3.10E-10	1.75E-10	1026/596/7048/70	70
hsa04068	FoxO signaling pathway	98/3698	9.58E-12	3.10E-10	1.75E-10	1026/23678/1005	98
hsa04668	TNF signaling pathway	86/3698	1.64E-11	4.77E-10	2.69E-10	355/83737/2353/1	86
hsa04390	Hippo signaling pathway	113/3698	1.77E-11	4.77E-10	2.69E-10	659/7048/7159/70	113
hsa05215	Prostate cancer	76/3698	4.36E-11	1.08E-09	6.11E-10	1869/1026/1027/5	76
hsa05161	Hepatitis B	115/3698	5.01E-11	1.16E-09	6.52E-10	1869/1026/355/31	115
hsa05168	Herpes simplex virus 1 infection	298/3698	5.99E-11	1.25E-09	7.05E-10	355/317/596/1616	298
hsa05132	Salmonella infection	162/3698	6.19E-11	1.25E-09	7.05E-10	596/581/2353/60/	162
hsa05167	Kaposi sarcoma-associated herpesvi	132/3698	1.21E-10	2.22E-09	1.25E-09	1869/1026/7422/3	132
hsa05212	Pancreatic cancer	62/3698	1.24E-10	2.22E-09	1.25E-09	1869/1026/7422/1	62
hsa05220	Chronic myeloid leukemia	60/3698	2.93E-09	4.97E-08	2.80E-08	1869/1026/1021/1	60
hsa04141	Protein processing in endoplasmic re	116/3698	3.50E-09	5.66E-08	3.19E-08	596/581/267/821/	116
hsa05131	Shigellosis	155/3698	5.15E-09	7.91E-08	4.46E-08	596/581/60/71/207	155
hsa01522	Endocrine resistance	73/3698	6.04E-09	8.86E-08	5.00E-08	1869/1026/1027/5	73
hsa05213	Endometrial cancer	48/3698	6.43E-09	9.03E-08	5.09E-08	1026/5728/595/58	48
hsa04010	MAPK signaling pathway	182/3698	1.16E-08	1.53E-07	8.62E-08	3815/7422/355/70	182
hsa05225	Hepatocellular carcinoma	113/3698	1.18E-08	1.53E-07	8.62E-08	1869/1026/1021/7	113
hsa04510	Focal adhesion	131/3698	1.69E-08	2.09E-07	1.18E-07	7422/596/5728/59	131
hsa05163	Human cytomegalovirus infection	144/3698	1.95E-08	2.34E-07	1.32E-07	1869/1026/7422/3	144
hsa04933	AGE-RAGE signaling pathway in diat	73/3698	2.61E-08	2.95E-07	1.67E-07	7422/1027/596/70	73
hsa05169	Epstein-Barr virus infection	131/3698	2.65E-08	2.95E-07	1.67E-07	1869/1026/355/31	131
hsa04350	TGF-beta signaling pathway	69/3698	4.35E-08	4.69E-07	2.64E-07	659/7048/7040/70	69
hsa04520	Adherens junction	55/3698	4.54E-08	4.73E-07	2.67E-07	7082/7048/51701/	55
hsa05010	Alzheimer disease	219/3698	6.16E-08	5.98E-07	3.37E-07	355/317/10105/20	219
hsa05160	Hepatitis C	105/3698	6.17E-08	5.98E-07	3.37E-07	1869/1026/355/31	105
hsa05203	Viral carcinogenesis	131/3698	6.39E-08	5.98E-07	3.37E-07	1026/1021/1027/8	131
hsa05165	Human papillomavirus infection	199/3698	6.48E-08	5.98E-07	3.37E-07	1869/1026/7422/3	199
hsa05014	Amyotrophic lateral sclerosis	216/3698	7.74E-08	6.95E-07	3.92E-07	317/596/1616/581	216
hsa04722	Neurotrophin signaling pathway	83/3698	9.00E-08	7.85E-07	4.43E-07	596/581/207/208/	83
hsa04066	HIF-1 signaling pathway	77/3698	1.12E-07	9.46E-07	5.34E-07	1026/7422/3570/1	77
hsa05222	Small cell lung cancer	67/3698	1.14E-07	9.46E-07	5.34E-07	1869/1026/317/10	67
hsa04150	mTOR signaling pathway	103/3698	1.35E-07	1.07E-06	6.02E-07	5728/207/208/526	103
hsa05162	Measles	94/3698	1.36E-07	1.07E-06	6.02E-07	355/317/1021/102	94
hsa01521	EGFR tyrosine kinase inhibitor resista	59/3698	1.50E-07	1.15E-06	6.50E-07	7422/3570/596/57	59
hsa05166	Human T-cell leukemia virus 1 infecti	138/3698	1.55E-07	1.17E-06	6.57E-07	1869/1026/898/70	138
hsa04550	Signaling pathways regulating pluripo	96/3698	1.72E-07	1.23E-06	6.96E-07	659/6774/648/90/	96
hsa05017	Spinocerebellar ataxia	96/3698	1.72E-07	1.23E-06	6.96E-07	10105/207/208/29	96
hsa04210	Apoptosis	92/3698	1.80E-07	1.24E-06	7.01E-07	355/317/596/1616	92
hsa05170	Human immunodeficiency virus 1 infe	134/3698	1.81E-07	1.24E-06	7.01E-07	355/1073/596/581	134
hsa05224	Breast cancer	97/3698	5.17E-07	3.48E-06	1.96E-06	3815/1869/1026/1	97
hsa05418	Fluid shear stress and atherosclerosi	92/3698	8.26E-07	5.44E-06	3.07E-06	7422/659/596/235	92
hsa01524	Platinum drug resistance	54/3698	8.56E-07	5.53E-06	3.12E-06	1026/355/317/596	54
hsa05219	Bladder cancer	34/3698	1.03E-06	6.50E-06	3.66E-06	1869/1026/7422/5	34
hsa05214	Glioma	55/3698	1.09E-06	6.74E-06	3.80E-06	1869/1026/1021/5	55
hsa04370	VEGF signaling pathway	45/3698	1.69E-06	1.03E-05	5.80E-06	7422/207/208/842	45
hsa05202	Transcriptional misregulation in cance	120/3698	1.85E-06	1.10E-05	6.23E-06	3207/1026/1027/7	120
hsa05135	Yersinia infection	86/3698	1.94E-06	1.14E-05	6.42E-06	2353/60/71/207/2	86
hsa05216	Thyroid cancer	31/3698	2.03E-06	1.17E-05	6.60E-06	1026/595/581/149	31
hsa04071	Sphingolipid signaling pathway	79/3698	4.11E-06	2.33E-05	1.31E-05	596/5728/581/552	79
hsa04919	Thyroid hormone signaling pathway	80/3698	4.55E-06	2.49E-05	1.41E-05	117247/2099/595/	80
hsa04932	Non-alcoholic fatty liver disease	96/3698	4.55E-06	2.49E-05	1.41E-05	355/3570/83737/7	96
hsa05130	Pathogenic Escherichia coli infection	119/3698	5.38E-06	2.90E-05	1.63E-05	7082/355/581/235	119
hsa04015	Rap1 signaling pathway	128/3698	5.59E-06	2.96E-05	1.67E-05	3815/7422/7057/6	128
hsa05164	Influenza A	107/3698	6.14E-06	3.16E-05	1.78E-05	355/317/1021/581	107
hsa05142	Chagas disease	69/3698	6.16E-06	3.16E-05	1.78E-05	355/7048/7040/23	69
hsa04012	ErbB signaling pathway	59/3698	8.37E-06	4.22E-05	2.38E-05	1026/1027/6776/2	59
hsa04211	Longevity regulating pathway	61/3698	1.12E-05	5.54E-05	3.13E-05	27244/581/6648/1	61

hsa05218	Melanoma	51/3698	1.39E-05	6.82E-05	3.84E-05	1869/1026/1021/5	51
hsa04360	Axon guidance	111/3698	1.57E-05	7.57E-05	4.27E-05	659/1073/6608/59	111
hsa04144	Endocytosis	146/3698	1.84E-05	8.74E-05	4.93E-05	83737/9146/7048/	146
hsa04810	Regulation of actin cytoskeleton	128/3698	2.10E-05	9.81E-05	5.53E-05	1073/60/71/324/99	128
hsa05012	Parkinson disease	146/3698	2.46E-05	0.000114	6.40E-05	317/1616/10105/5	146
hsa05226	Gastric cancer	93/3698	2.83E-05	0.000129	7.27E-05	1869/1026/1027/5	93
hsa04137	Mitophagy - animal	48/3698	2.88E-05	0.000129	7.30E-05	1869/598/1457/23	48
hsa05230	Central carbon metabolism in cancer	49/3698	3.41E-05	0.00015	8.48E-05	3815/5728/207/20	49
hsa04152	AMPK signaling pathway	77/3698	3.45E-05	0.00015	8.48E-05	595/5520/31/207/2	77
hsa03018	RNA degradation	54/3698	3.98E-05	0.000172	9.67E-05	7832/694/1656/20	54
hsa05221	Acute myeloid leukemia	47/3698	4.51E-05	0.000192	0.000108	3815/595/6776/67	47
hsa04151	PI3K-Akt signaling pathway	198/3698	5.85E-05	0.000245	0.000138	3815/1026/7422/3	198
hsa05235	PD-L1 expression and PD-1 checkpo	59/3698	7.14E-05	0.000295	0.000167	5728/10125/2353/	59
hsa04660	T cell receptor signaling pathway	67/3698	9.14E-05	0.000374	0.000211	10125/2353/207/2	67
hsa04917	Prolactin signaling pathway	48/3698	9.38E-05	0.000379	0.000214	9655/2099/595/23	48
hsa04070	Phosphatidylinositol signaling system	63/3698	0.000103	0.000412	0.000232	5728/805/3612/36	63
hsa04931	Insulin resistance	69/3698	0.000106	0.000418	0.000236	5728/5465/6774/2	69
hsa04213	Longevity regulating pathway - multip	43/3698	0.000144	0.000561	0.000316	6648/107/115/207	43
hsa05100	Bacterial invasion of epithelial cells	49/3698	0.000182	0.000699	0.000394	60/71/391/857/998	49
hsa04215	Apoptosis - multiple species	25/3698	0.000191	0.000726	0.000409	317/596/581/2711	25
hsa04935	Growth hormone synthesis, secretion	74/3698	0.000215	0.000807	0.000455	2353/6776/6774/1	74
hsa04910	Insulin signaling pathway	83/3698	0.00032	0.001188	0.00067	31/207/208/805/1	83
hsa05231	Choline metabolism in cancer	62/3698	0.000348	0.001279	0.000721	2353/207/208/195	62
hsa04620	Toll-like receptor signaling pathway	65/3698	0.000417	0.001512	0.000853	2353/9641/207/20	65
hsa04912	GnRH signaling pathway	59/3698	0.000432	0.001552	0.000875	107/115/805/998/1	59
hsa04926	Relaxin signaling pathway	78/3698	0.000522	0.001852	0.001044	7422/59/7048/704	78
hsa05020	Prion disease	152/3698	0.000546	0.001917	0.001081	317/10105/581/29	152
hsa04310	Wnt signaling pathway	94/3698	0.000604	0.002099	0.001184	595/51701/324/89	94
hsa05016	Huntington disease	168/3698	0.0007	0.002406	0.001356	317/10105/581/66	168
hsa04136	Autophagy - other	24/3698	0.000747	0.002541	0.001433	2475/9140/9474/1	24
hsa03015	mRNA surveillance pathway	60/3698	0.001002	0.003371	0.0019	5520/2107/4686/5	60
hsa04530	Tight junction	94/3698	0.001067	0.003552	0.002003	7082/595/5520/60	94
hsa04611	Platelet activation	74/3698	0.001215	0.004005	0.002258	10125/60/71/107/1	74
hsa04340	Hedgehog signaling pathway	34/3698	0.001232	0.004017	0.002265	596/6608/595/156	34
hsa04710	Circadian rhythm	23/3698	0.001244	0.004017	0.002265	1385/1408/1454/5	23
hsa04380	Osteoclast differentiation	76/3698	0.001286	0.004113	0.002319	7048/7040/2353/2	76
hsa05120	Epithelial cell signaling in Helicobacte	45/3698	0.001351	0.004279	0.002412	7082/526/528/836	45
hsa04014	Ras signaling pathway	129/3698	0.001491	0.004675	0.002636	3815/7422/5921/1	129
hsa04064	NF-kappa B signaling pathway	63/3698	0.001625	0.004998	0.002818	596/142/331/472/5	63
hsa04625	C-type lectin receptor signaling pathw	63/3698	0.001625	0.004998	0.002818	9641/207/208/805	63
hsa04928	Parathyroid hormone synthesis, secre	64/3698	0.001685	0.005134	0.002894	1026/596/2353/10	64
hsa04720	Long-term potentiation	43/3698	0.001801	0.005412	0.003052	107/805/814/2776	43
hsa04666	Fc gamma R-mediated phagocytosis	57/3698	0.00181	0.005412	0.003052	1073/4082/207/20	57
hsa04662	B cell receptor signaling pathway	51/3698	0.001968	0.005833	0.003289	2353/207/208/975	51
hsa04657	IL-17 signaling pathway	57/3698	0.002569	0.007543	0.004252	2353/4312/9641/8	57
hsa04664	Fc epsilon RI signaling pathway	43/3698	0.002748	0.007998	0.004509	207/208/2534/288	43
hsa04114	Oocyte meiosis	75/3698	0.003043	0.008777	0.004948	898/107/115/367/8	75
hsa04392	Hippo signaling pathway - multiple sp	21/3698	0.003342	0.009552	0.005385	1454/4771/6788/7	21
hsa00310	Lysine degradation	40/3698	0.00343	0.00972	0.00548	38/223/1892/2145	40
hsa04540	Gap junction	53/3698	0.004376	0.012292	0.00693	7082/107/115/983	53
hsa00562	Inositol phosphate metabolism	45/3698	0.004523	0.012594	0.007101	5728/3612/3636/3	45
hsa04371	Apelin signaling pathway	78/3698	0.005415	0.014949	0.008428	59/595/1490/182/1	78
hsa04670	Leukocyte transendothelial migration	66/3698	0.005994	0.016408	0.009251	60/71/399/998/149	66
hsa05134	Legionellosis	36/3698	0.006096	0.016546	0.009328	317/836/840/841/8	36
hsa05217	Basal cell carcinoma	39/3698	0.007184	0.019337	0.010902	1026/6608/581/32	39
hsa04914	Progesterone-mediated oocyte matur	58/3698	0.009137	0.02439	0.013751	993/107/115/207/2	58
hsa03030	DNA replication	24/3698	0.009265	0.02453	0.01383	2237/4172/4174/4	24
hsa04621	NOD-like receptor signaling pathway	99/3698	0.009364	0.024591	0.013864	596/9641/331/598	99
hsa04730	Long-term depression	37/3698	0.009515	0.024786	0.013974	2771/2773/2776/2	37
hsa04072	Phospholipase D signaling pathway	82/3698	0.011281	0.02915	0.016435	3815/107/115/207	82
hsa04916	Melanogenesis	58/3698	0.012021	0.030817	0.017374	3815/107/115/805	58
hsa05110	Vibrio cholerae infection	31/3698	0.015264	0.038822	0.021888	7082/60/71/115/52	31
hsa00513	Various types of N-glycan biosynthes	25/3698	0.016205	0.040503	0.022835	1603/1650/2683/4	25
hsa00620	Pyruvate metabolism	25/3698	0.016205	0.040503	0.022835	31/38/97/223/273	25
hsa05145	Toxoplasmosis	63/3698	0.016302	0.040503	0.022835	596/10105/7040/6	63
hsa03013	RNA transport	100/3698	0.016654	0.041063	0.023151	1915/1917/1964/1	100
hsa04728	Dopaminergic synapse	73/3698	0.01706	0.041367	0.023323	2353/5520/207/20	73
hsa00020	Citrate cycle (TCA cycle)	20/3698	0.017102	0.041367	0.023323	4190/4191/5160/5	20

hsa04915	Estrogen signaling pathway	76/3698	0.017162	0.041367	0.023323	596/2099/2353/10	76
hsa03440	Homologous recombination	26/3698	0.017318	0.041435	0.023361	472/580/641/672/6	26
hsa04022	cGMP-PKG signaling pathway	90/3698	0.020672	0.049097	0.02768	10105/107/115/14	90
hsa04934	Cushing syndrome	84/3698	0.020978	0.049459	0.027885	1869/1026/1021/1	84

Table S5. HALLMARK terms enriched among the miRNA targeted genes.

ID	Description	GeneRatio	*P*-value	*P*-adjust	Q-value
HALLMARK_E2F_TARGETS	HALLMARK_E2F_TARGETS	171/2535	6.05E-18	3.02E-16	1.78E-16
HALLMARK_G2M_CHECKPOINT	HALLMARK_G2M_CHECKPOINT	169/2535	1.14E-16	2.86E-15	1.68E-15
HALLMARK_MTORC1_SIGNALING	HALLMARK_MTORC1_SIGNALING	165/2535	2.60E-14	4.33E-13	2.55E-13
HALLMARK_MYC_TARGETS_V1	HALLMARK_MYC_TARGETS_V1	163/2535	3.17E-13	3.96E-12	2.33E-12
HALLMARK_TNFA_SIGNALING_VIA_NFKB	HALLMARK_TNFA_SIGNALING_VIA_NFKB	159/2535	3.17E-11	3.17E-10	1.87E-10
HALLMARK_UV_RESPONSE_DN	HALLMARK_UV_RESPONSE_DN	114/2535	3.50E-08	2.91E-07	1.72E-07
HALLMARK_MITOTIC_SPINDLE	HALLMARK_MITOTIC_SPINDLE	151/2535	4.17E-08	2.98E-07	1.76E-07
HALLMARK_TGF_BETA_SIGNALING	HALLMARK_TGF_BETA_SIGNALING	46/2535	1.39E-05	8.71E-05	5.13E-05
HALLMARK_PROTEIN_SECRETION	HALLMARK_PROTEIN_SECRETION	74/2535	5.25E-05	0.00029	0.00017
HALLMARK_UNFOLDED_PROTEIN_RESPONSE	HALLMARK_UNFOLDED_PROTEIN_RESPONSE	85/2535	7.31E-05	0.00037	0.00022
HALLMARK_APOPTOSIS	HALLMARK_APOPTOSIS	116/2535	0.0001	0.00046	0.00027
HALLMARK_ANDROGEN_RESPONSE	HALLMARK_ANDROGEN_RESPONSE	74/2535	0.00051	0.00212	0.00125
HALLMARK_INTERFERON_ALPHA_RESPONSE	HALLMARK_INTERFERON_ALPHA_RESPONSE	71/2535	0.00111	0.00426	0.00251
HALLMARK_EPITHELIAL_MESENCHYMAL_TRANSITION	HALLMARK_EPITHELIAL_MESENCHYMAL_TRANSITI	135/2535	0.00263	0.0094	0.00554
HALLMARK_INTERFERON_GAMMA_RESPONSE	HALLMARK_INTERFERON_GAMMA_RESPONSE	134/2535	0.00415	0.01382	0.00815
HALLMARK_NOTCH_SIGNALING	HALLMARK_NOTCH_SIGNALING	26/2535	0.00455	0.01421	0.00837
HALLMARK_P53_PATHWAY	HALLMARK_P53_PATHWAY	131/2535	0.01428	0.04199	0.02475
